# Supplementary material for: Marine Science Can Contribute to the Search for Extra-Terrestrial Life
Source: Life (Basel). 2024 May 24;14(6):676. doi: 10.3390/life14060676 (PMC11205085; doi:10.3390/life14060676)
Supplement: Supplementary file 1 [file life-14-00676-s001.zip › life-2979823-supplementary.pdf]

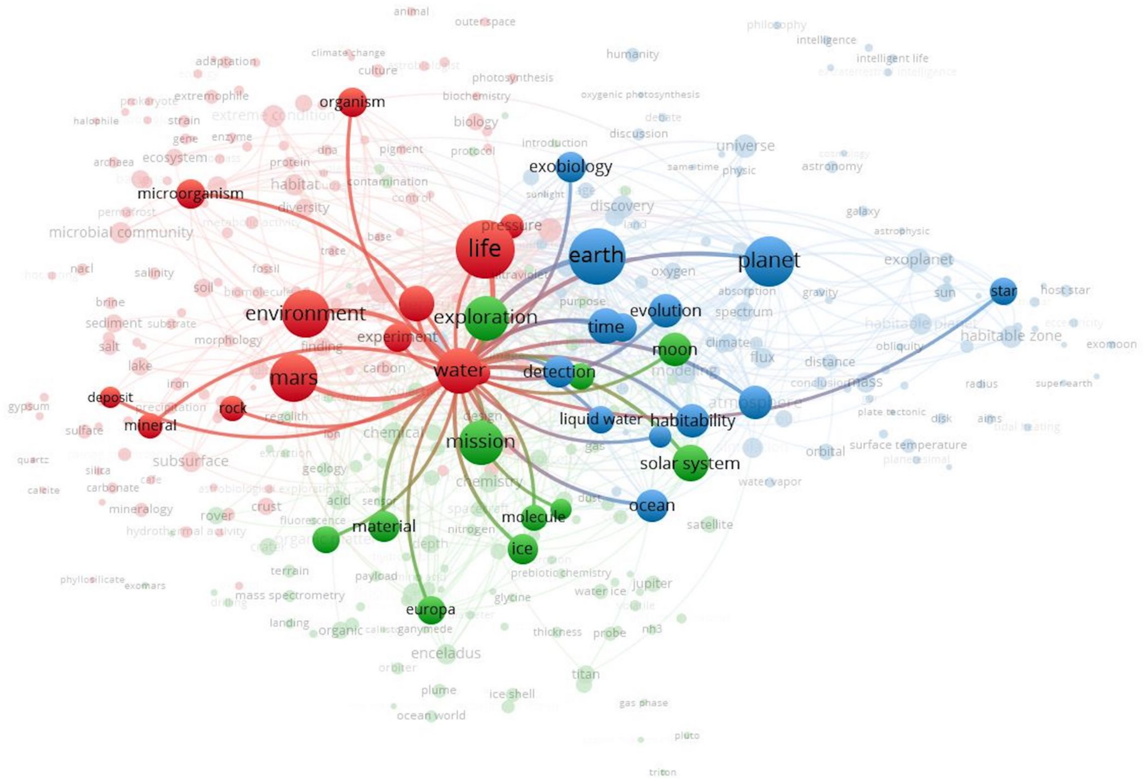

Figure S1. Highlighted term connections radiating from the term "water", from Fig. 2.

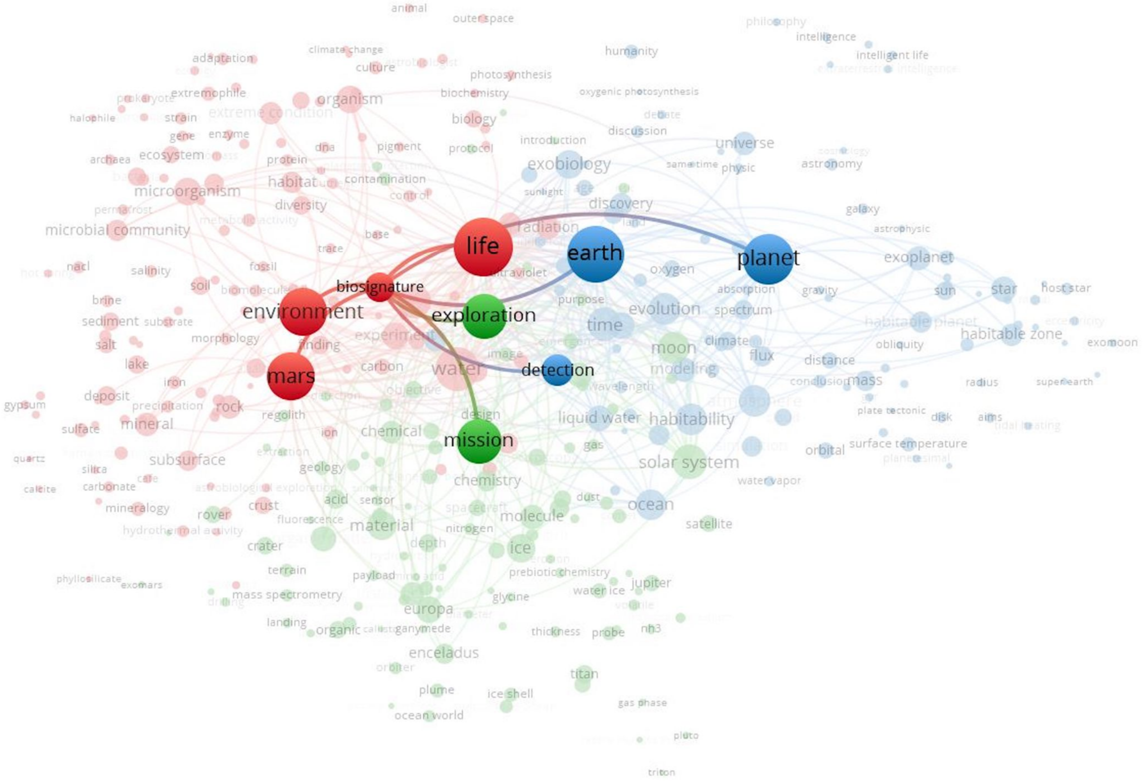

Figure S2. Highlighted term connections radiating from the term "biosignature", from Figure 2.

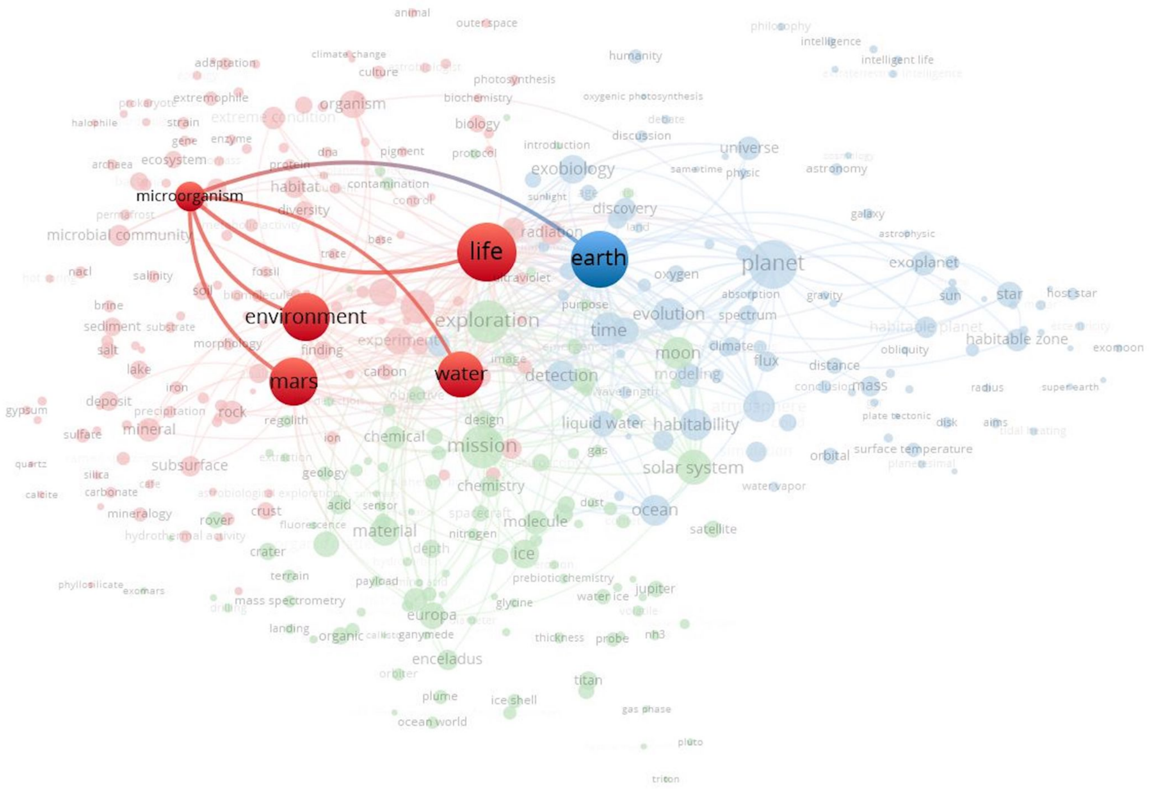

Figure S3. Highlighted term connections radiating from the term "microorganism", from Figure 2.

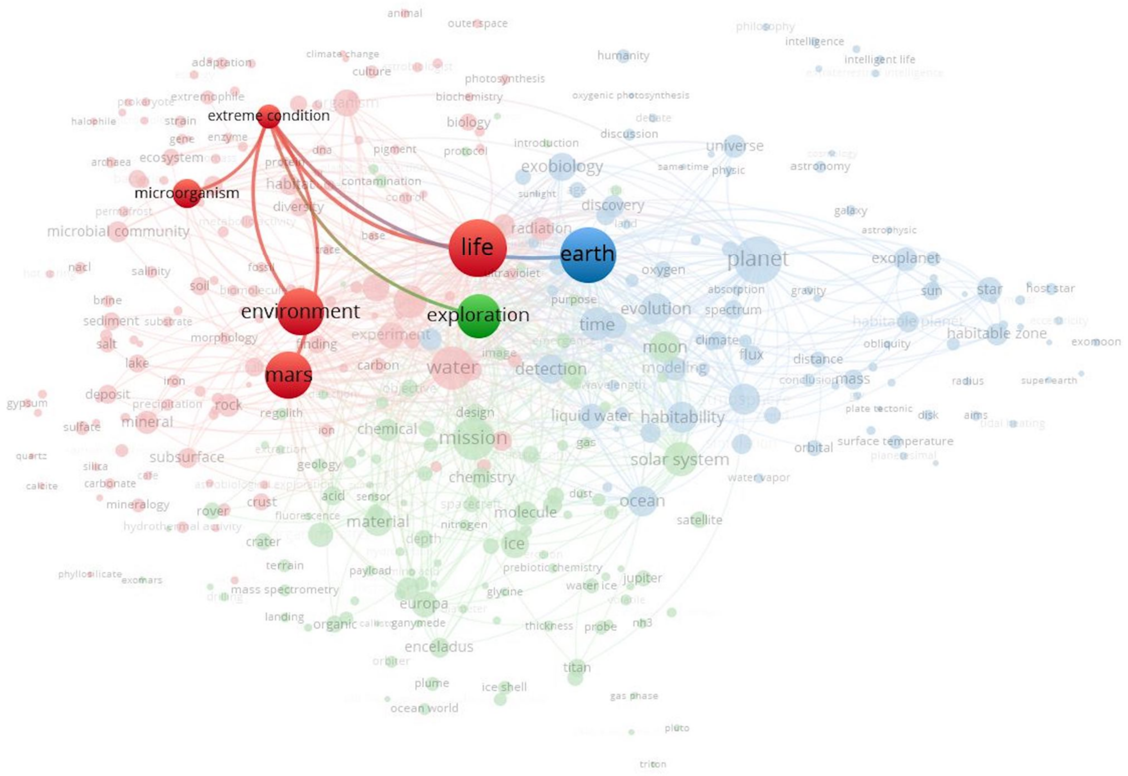

Figure S4. Highlighted term connections radiating from the term "extreme condition", from Figure 2.

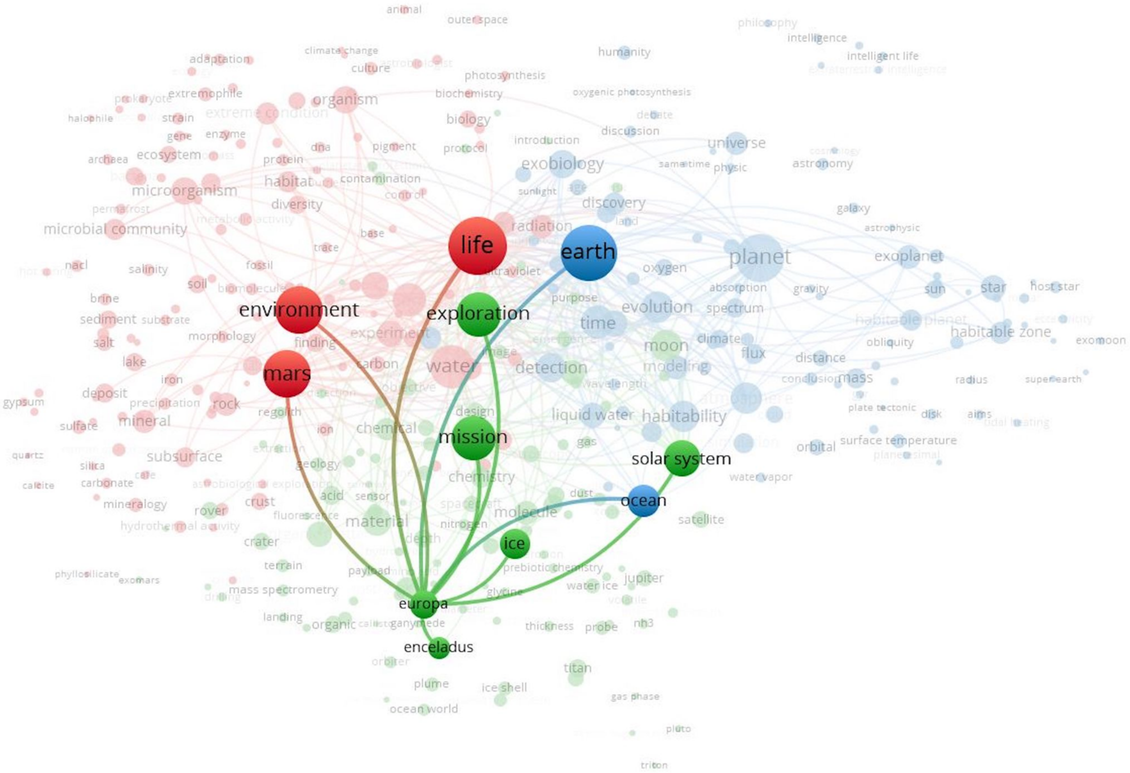

Figure S5. Highlighted term connections radiating from the term “Europa”, from Figure 2.
