# Supplementary material for: Marine Science Can Contribute to the Search for Extra-Terrestrial Life
Source: Life (Basel). 2024 May 24;14(6):676. doi: 10.3390/life14060676 (PMC11205085; doi:10.3390/life14060676)
Supplement: Supplementary file 1 [file Supplementary material S2_RIS.pdf › Supplementary material S2_RIS.pdf]

TY - JOUR
AU - Packer, E.
AU - Scher, S.
AU - Sagan, C.
TI - Biological contamination of Mars II. Cold and aridity as constraints on the survival of terrestial microorganisms in simulated Martian environments
PY - 1963
T2 - Icarus
VL - 2
IS - C
SP - 293
EP - 316
DO - 10.1016/0019-1035(63)90024-1
UR - https://www.scopus.com/inward/record.uri?eid=2-s2.0-0012492970&doi=10.1016%2f0019-1035%2863%2990024-1&partnerID=40&md5=4240be932584985ca9df63a55249f241
AD - Space Sciences Laboratory, University of California, Berkeley, CA, United States
AB - It has been postulated that the accidental introduction of terrestial microorganisms to other planets during the course of space exploration may impede or bias the detection of organic matter and possible indigenous organisms, and thereby obscure subsequent studies of extraterrestial life. To assess the likelihood of biological contamination of Mars, we have applied the principle of natural selection on a laboratory scale. Terrestial microorganisms were collected from a variety of environments, including regions of high alkalinity, low mean daily temperature, and low annual rainfall. The air-dried soils were then subjected in the presence of a dessicant to a stimulated Martian environment involving 12-hour freeze-thaw cycles from about −60°C to about +20°C; atmospheres of 95% nitrogen, 5% carbon dioxide, and low moisture content; ≤0.1 atm pressure; and a total ultraviolet dose at 2537 Å of 109 erg cm-2. Survivors were scored on supplemented agar. Preliminary results indicate a wide variety of survivors including obligate and facultative anaerobic spore-formers and non-spore-forming facultative anaerobic bacteria. Diurnal freezing and thawing were continued for 6 months. There was no significant loss of viability after the first freeze-thaw cycle. An extensive survey of the literature shows that survival of terrestial microorganisms under individual simulated Martian conditions has been known for decades. Survival varies greatly with such parameters as the rate of freezing and thawing, the physics and chemistry of the medium, and the individual species studied. A primary deleterious effect of low temperatures is the unavailability of liquid water. The present investigation shows the absence of pronounced synergistic effects inhibiting survival. The probable existence of organic matter and moisture on Mars, at least in restricted locales and times, makes it possible that terrestial microorganisms can also reproduce on Mars. The demonstration that all samples of terrestial soil tested contain a population of microorganisms which survive in simulated Martian environments strongly underscores the need for scrupulous sterilization of all spacecraft intended for Mars landings. © 1963.
SN - 00191035 (ISSN)

LA - English
J2 - Icarus
M3 - Article
DB - Scopus
N1 - Export Date: 23 February 2023; Cited By: 23; CODEN: ICRSA
ER -

TY - JOUR
AU - Opfell, J.B.
TI - A general review of chemical sterilization in space research.
PY - 1964
T2 - Life sciences and space research
VL - 2
SP - 385
EP - 405
UR - https://www.scopus.com/inward/record.uri?eid=2-s2.0-0013773554&partnerID=40&md5=a0a6163bdc1f4ca578b7313acb83472f
AD - Dynamic Science Corporation, South Pasadena, California, United States
AB - The unsolved problems in the biology and the chemistry of chemical sterilization are legion. This discussion of them emphasizes some interesting areas for useful research. Studies in molecular biology and cancer chemotherapy have produced information which has important implications in chemical sterilization, particularly with the alkylating and the oxidizing agents. Many of these problems must be solved soon if chemical sterilization is to play the role it should in space research by providing means for effectively controlling the rate and extent of mingling of the forms of terrestrial life with those of extraterrestrial life. In space research in the United States, recent work on chemical sterilization has laid heavy emphasis on the identification and exploration of principles of engineering sciences which control sterilization effectiveness. These principles are reviewed and their significance in chemical sterilization is discussed in terms of the engineering factors involved. Among these factors are: consequence of sterilant polymerization or reaction with the object to be sterilized; criteria for chemical sterilization process effectiveness; depletion of the concentration of the sterilant by evaporation or chemical reaction, including polymerization; obstacles to the diffusion or convection of the sterilizing chemical to the immediate vicinity of the microorganisms to be destroyed; protection of the microorganisms from direct contact with the sterilant; phase behavior of the sterilant in the environment of the sterilization process; presence of water or water vapor; the nature, sources and numbers of microorganisms which survive standard chemical sterilization processes; and the length of exposure required to sterilize at each temperature. The significance of chemical sterilization to space science has been well reviewed at the previous COSPAR symposia. Because several thorough reviews of the empirical knowledge concerning the effects of chemical agents on microorganisms have been published recently, this information is not discussed again for chemical sterilants other than the alkylating agents and several of the oxidizing agents. This lack of emphasis indicates only that these other chemicals have not been applied to a large extent in

space research to date and that discussions of their sterilizing properties are readily available in other recent reviews. In the future, interest in many of these other chemical sterilants will increase as the variety of devices to be sterilized and the variety of functions which sterilized devices are expected to perform increase.
KW - Alkylating Agents
KW - Antifungal Agents
KW - Containment of Biohazards
KW - Decontamination
KW - Environmental Microbiology
KW - Exobiology
KW - Spacecraft
KW - Spores, Bacterial
KW - Sterilization
KW - alkylating agent
KW - antifungal agent
KW - bacterial spore
KW - biology
KW - drug effect
KW - hazardous waste
KW - instrument sterilization
KW - methodology
KW - microbiology
KW - physiology
KW - review
KW - space flight
KW - waste management
SN - 00759422 (ISSN)
C2 - 11883446
LA - English
J2 - Life Sci Space Res
M3 - Review
DB - Scopus
N1 - Export Date: 23 February 2023; Cited By: 1
ER -

TY - JOUR
AU - Nagy, B.
TI - The possibility of extraterrestrial life: Ultra-microchemical analyses and electro-microscopic studies of microstructures in carbonaceous meteorites
PY - 1967
T2 - Review of Palaeobotany and Palynology
VL - 3
IS - 1-4
SP - 237
EP - 242
DO - 10.1016/0034-6667(67)90056-5
UR - https://www.scopus.com/inward/record.uri?eid=2-s2.0-49949135585&doi=10.1016%2f0034-6667%2867%2990056-5&partnerID=40&md5=e9b21439fbc787ae60e1ff4818226d40
AD - Department of Chemistry, University of California at San Diego, La Jolla, CA, United States

AB - There are a few stony meteorites which are composed of clay-type minerals, carbonates, oxides of iron, gypsum, magnesium sulfate, elementary sulfur and organic matter. These meteorites are observed falls. They may contain as much as 20% water, part of which has deuterium/hydrogen ratios entirely different from those of terrestrial waters. The mineral assemblage is generally regarded to indicate an aqueous, low temperature, slightly alkaline and somewhat reducing environment on the meteorite parent body. These meteorites also contain microstructures (i.e., organized elements) which morphologically resemble microfossils. Electron probe X-ray microanalysis showed that these microstructures are partially mineralized with iron and contain traces of chlorine or they contain silicates. The acid insoluble residues of the organized elements gave ultraviolet and visible ultramicrospectra characteristic of a variety of organic substances, including the possibility of nucleic acid constituents. Ultra-thin sections of a carbonaceous meteorite showed small, rounded objects occasionally with double wall-like structures. These microstructures were firmly embedded in the mineral matrix of the meteorite. The nature and origin of the organized elements is still unknown. © 1967.
SN - 00346667 (ISSN)
LA - English
J2 - Rev. Palaeobot. Palynol.
M3 - Article
DB - Scopus
N1 - Export Date: 23 February 2023; Cited By: 2; CODEN: RPPYA
ER -

TY - JOUR
AU - Kok, B.
AU - Varner, J.E.
TI - Extraterrestrial life detection based on oxygen isotope exchange reactions
PY - 1967
T2 - Science
VL - 155
IS - 3766
SP - 1110
EP - 1112
DO - 10.1126/science.155.3766.1110
UR - https://www.scopus.com/inward/record.uri?eid=2-s2.0-21344439400&doi=10.1126%2fscience.155.3766.1110&partnerID=40&md5=28e33369e5bedb5568a81e2da212cd57
AD - Research Institute for Advanced Studies, Baltimore, MD 21227, United States
AD - Michigan State University, East Lansing
AB - A method is described for detecting extraterrestrial life, based on catalysis of isotopic oxygen exchange between water and oxygen-containing onions such as phosphate, nitrate, or sulfate. This catalytic activity appears to be unique to living systems. Its applicability requires very few assumptions concerning the chemical nature of "life." Data obtained so far indicate that the experiment is sound and technically feasible.
SN - 00368075 (ISSN)

LA - English
M3 - Article
DB - Scopus
N1 - Export Date: 23 February 2023; Cited By: 7
ER -

TY - JOUR
AU - Libby, W.F.
TI - Life in space
PY - 1968
T2 - Space Life Sciences
VL - 1
IS - 1
SP - 5
EP - 9
DO - 10.1007/BF00924228
UR - https://www.scopus.com/inward/record.uri?eid=2-s2.0-0014254392&doi=10.1007%2fBF00924228&partnerID=40&md5=a516d871a0e13e24f795d0ff5312d80c
AD - Dept. of Chemistry and Inst. of Geophysics, University of California, Los Angeles, Calif., United States
AB - The physical conditions of Space are most inhospitable and the higher forms of life probably could exist extraterrestrially only on Venus, Jupiter, and Saturn in our Solar System, and the chances there are poor in light of present knowledge. Thus intelligent life probably exists only on the Earth. Although indigenous intelligent extraterrestrial life seems to be improbable it is by no means clear that man cannot learn to live reasonably comfortably on most of our planets and planetoids such as our moon, and it seems certain that he will be able to travel great distances in the solar system. Lower forms of life may well occur extraterrestrially. © 1968 D. Reidel Publishing Company.
KW - Animal
KW - Astronomy
KW - Biochemistry
KW - Biology
KW - Evolution
KW - Extraterrestrial Environment
KW - Food
KW - Human
KW - Intelligence
KW - Philosophy
KW - Radiation Protection
KW - Space Flight
KW - Sunlight
KW - Temperature
KW - animal
KW - article
KW - astronomy
KW - biochemistry
KW - biology
KW - evolution
KW - food
KW - human

KW - intelligence
KW - microclimate
KW - philosophy
KW - radiation protection
KW - space flight
KW - sunlight
KW - temperature
PB - Kluwer Academic Publishers
SN - 15730875 (ISSN)
C2 - 5760602
LA - English
J2 - Space Life Sciences
M3 - Article
DB - Scopus
N1 - Export Date: 23 February 2023; Cited By: 0; CODEN: OLEBE
ER -

TY - JOUR
AU - Herzog, L.F.
TI - Mass spectroscopy beyond the moon
PY - 1970
T2 - International Journal of Mass Spectrometry and Ion Physics
VL - 4
IS - 4
SP - 337
EP - 363
DO - 10.1016/0020-7381(70)85049-5
UR - https://www.scopus.com/inward/record.uri?eid=2-s2.0-49849113695&doi=10.1016%2f0020-7381%2870%2985049-5&partnerID=40&md5=245a1195c241cb513fc0686141d2a142
AD - Nuclide Corporation, State College, United States
AB - Mass spectrometers reveal the elemental, isotopic and molecular composition of solids, liquids, and gases, from major to microtrace constituents. Hence, the use of "robot" mass spectrometer probes in the exploration of the solar system has been proposed. This paper surveys our present knowledge of solar system composition and the kinds of data on various solar system components it is hoped to obtain through the space program, and the areas in which mass spectroscopy can be most useful. Specific applications discussed include: determining planetary atmospheric compositions, classifying extraterrestrial rock, "geological" (radioactive parent/daughter) age determination, the detection of extraterrestrial "life", determining the compositions of asteroids, comets and interplanetary dust, and the study of the solar wind and its effects. The mass spectrometric instrumentation available is reviewed and a recommendation is made for consideration of the development of two types of systems for space exploration - a low resolution mass spectrometer without pumping, possibly with "hybrid" tof-coincidence-mass filter capabilities, and a medium resolution system with pumping, probably using a double-focusing ion analyzer with a permanent magnet. © 1970.
SN - 00207381 (ISSN)
LA - English
M3 - Article

DB - Scopus
N1 - Export Date: 23 February 2023; Cited By: 2
ER -

TY - JOUR
AU - Baker, B.L.
TI - Review of organic matter in the Orgueil meteorite
PY - 1971
T2 - Space Life Sciences
VL - 2
IS - 4
SP - 472
EP - 497
DO - 10.1007/BF00941363
UR - https://www.scopus.com/inward/record.uri?eid=2-s2.0-34250450810&doi=10.1007%2fBF00941363&partnerID=40&md5=2ecf410c23af01573ca0485a1d739373
AD - Exobiology Research Group, University of Calgary, Calgary 44, Canada
AB - The Orgueil meteorite is a carbonaceous chondrite containing about 3.1% carbon, 5.5% sulfur and 19.9% water. Virtually all of the carbon is present as organic carbon although only about 20% is soluble in common organic solvents; the remainder is in the form of a highly substituted, irregular and aromatic polymer. Detailed methods of analysis have been improved in the past ten years sufficient for the detection of individual compounds in most of the following classes of organic compounds: hydrocarbons, oxygen-, sulfur- and nitrogen-containing organic compounds, optically active species, isotopes, bacteria and organized elements. Ten series of homologous compounds have been observed in the aliphatic hydrocarbons. In the 1950's, when interest was renewed in the Orgueil meteorite, the analytical capabilities may have given a bias toward biogenic agencies for the formation of the organic matter found in the meteorites. Some of the key biochemical compounds for extraterrestrial life are present. There is doubt, however, that these particular compounds are truly indigenous. The possibility that the indigenous organic compounds in the meteorite are present as a result of abiogenic syntheses in the cosmos is becoming more generally accepted. © 1971 D. Reidel Publishing Company.
PB - Kluwer Academic Publishers
SN - 15730875 (ISSN)
LA - English
J2 - Space Life Sciences
M3 - Review
DB - Scopus
N1 - Export Date: 23 February 2023; Cited By: 7; CODEN: OLEBE
ER -

TY - JOUR
AU - Phillips, G.B.
TI - Back contamination
PY - 1971
T2 - Environmental Biology and Medicine
VL - 1

IS - 2
SP - 121
EP - 160
UR - https://www.scopus.com/inward/record.uri?eid=2-s2.0-0015194086&partnerID=40&md5=f89d34ec2bb4bc27e4669dee108fda4f
AD - United States
AB - Back contamination is defined as contamination of the terrestrial biosphere with organisms or materials returned from outer space that are capable of potentially harmful terrestrial activity. Back contamination control relies heavily on physical barriers to contain extraterrestrial life until it can be examined and evaluated for harmful activity. An understanding of underlying basic concepts of microbial barrier systems is necessary for selecting the most appropriate measures for back contamination control. The present day concept of the prevention of back contamination resulting from samples returned from other planets is to place such samples behind a microbiologic barrier while suitable quarantine testing is being carried out. Recommended fundamental quarantine and sample release plans for space exploration missions are presented, for landings that are made on the Moon or other planets, and where samples, space craft, and/or crew are returned. Obviously, the schemes do not cover all possible technical details regarding quarantine test methods and containment provisions, but are intended to provide a useful framework for more specific planning.
KW - Decontamination
KW - Environmental Exposure
KW - Environmental Pollution
KW - Extraterrestrial Environment
KW - Human
KW - Methods
KW - Microbiology
KW - Quarantine
KW - Space Flight
KW - Sterilization
KW - United States
KW - epidemiology
KW - methodology
KW - space flight
KW - article
KW - environmental exposure
KW - human
KW - infection control
KW - instrument sterilization
KW - microbiology
KW - microclimate
KW - pollution
KW - space flight
KW - technique
KW - United States
KW - waste management
SN - 00462233 (ISSN)
C2 - 5173466
LA - English

J2 - ENVIRON. BIOL. MED.
M3 - Article
DB - Scopus
N1 - Export Date: 23 February 2023; Cited By: 3; CODEN: EVBMA
ER -

TY - JOUR
AU - Ross, L.W.
AU - Pecoraro, J.N.
TI - POLLUTION SOLUTIONS? A LOOK A NASA SPACE R&D COULD SUPPLY ANSWERS.
PY - 1973
T2 - Mechanical Engineering
VL - 95
IS - 1
SP - 8
EP - 13
UR - https://www.scopus.com/inward/record.uri?eid=2-s2.0-0015559128&partnerID=40&md5=2721ce249a9e5b9249ba3d96ae33f0b0
AB - The large scale of the nation's environmental problems, contrasted with the small scale of NASA's life-support mission, are only apparently in conflict. There are several points of overlap. It is even possible that some NASA life-support hardware may find application almost directly. Today's problems of extraterrestrial life support may be tomorrow's problems on earth itself. Our supply of oxygen is sufficient, but only if it remains free of contamination. Our supply of water is much more sensitive, and is already threatened.
KW - AIR POLLUTION - Control
KW - SPACE VEHICLES - Life Support Systems
KW - WATER POLLUTION - Control
KW - Environmental engineering
SN - 00256501 (ISSN)
LA - undefined
J2 - Mech Eng
M3 - Article
DB - Scopus
N1 - Export Date: 23 February 2023; Cited By: 0; CODEN: MEENA
ER -

TY - JOUR
AU - Masursky, H.
AU - Strobell, M.E.
AU - Dial, A.L.
TI - Martian channels and the search for extraterrestrial life
PY - 1979
T2 - Journal of Molecular Evolution
VL - 14
IS - 1-3
SP - 39
EP - 55
DO - 10.1007/BF01732366
UR - https://www.scopus.com/inward/record.uri?eid=2-s2.0-0018556475&doi=10.1007%2fBF01732366&partnerID=40&md5=865c0e3fa0

1d74475da29e053bceab4e
AD - U.S. Geological Survey, Flagstaff, 86001, Arizona, 2255 North Gemini Drive, United States
AB - The origin of the channels on Mars has been a subject of intense interest since they were first recognized on early Mariner 9 images (Driscoll, 1972; Masursky, 1973). Their presence on the planet, and their striking resemblance to terrestrial flood channels related to glacial outbursts or to dendritic river systems has suggested to most investigators (Baker, 1974, 1977; Nummedal, 1978; Carr, 1979; Masursky et al., 1977) that they were formed by running water. Because life as we know it is dependent on water, the discovery by the 'Mariner cameras', of watercut channels and volcanoes as a source for water, and water ice in the residual north polar cap by Viking, has reaffirmed the choice of Mars as the best target for the search for extraterrestrial life. © 1979 Springer-Verlag.
KW - Biogenesis
KW - Extraterrestrial Environment
KW - Geology
KW - Water
KW - water
KW - article
KW - biogenesis
KW - geology
KW - microclimate
PB - Springer-Verlag
SN - 14321432 (ISSN)
C2 - 522157
LA - English
J2 - J Mol Evol
M3 - Article
DB - Scopus
N1 - Export Date: 23 February 2023; Cited By: 7; CODEN: JMEVA
ER -

TY - JOUR
AU - Hubbard, J.S.
TI - Laboratory simulations of the pyrolytic release experiments: An interim report
PY - 1979
T2 - Journal of Molecular Evolution
VL - 14
IS - 1-3
SP - 211
EP - 221
DO - 10.1007/BF01732379
UR - https://www.scopus.com/inward/record.uri?eid=2-s2.0-0018608043&doi=10.1007%2fBF01732379&partnerID=40&md5=2cc4466e619a89eb76f2381e7a7324a0
AD - School of Biology, Georgia Institute of Technology, Atlanta, 30332, Georgia, United States
AB - During its operation on Mars the pyrolytic release experiment (PR) detected the fixation of small amounts of CO2 and/or CO. Laboratory simulations of the experimental conditions were made in

an attempt to substantiate the previous conclusion that these reactions were chemical rather than biological. The selection of model substrata for these tests was based on the known properties of the Martian surface material. After pretreatment and incubation under various conditions, pyrolytic analysis was used to indicate the extent of surface catalyzed conversion of14CO2 or14CO to14C-organic compounds. This abiotic synthesis was detected in experiments with three iron oxides, viz. hematite, magnetite and maghemite. When the incubation atmosphere was supplemented with water vapor, the levels of synthesis were in a range comparable to that detected in the Viking PR tests. An abiotic synthesis was also detected in experiments with a mixture of clays and minerals (Mars analog soil) or with montmorillonite artifically enriched in iron. With either substratum the reaction appeared to be the result of a photocatalytic synthesis of14C-organics from14CO and surface hydroxyl groups. This process was not dependent on the presence of water vapor in the incubation atmosphere. Although a duplication of the Viking data has not been achieved, these findings support the abiotic interpretation of the PR results. © 1979 Springer-Verlag.
KW - Carbon Dioxide
KW - Carbon Monoxide
KW - Chemistry
KW - Chemistry, Organic
KW - Extraterrestrial Environment
KW - Iron
KW - Metals
KW - Models, Theoretical
KW - Support, U.S. Gov't, Non-P.H.S.
KW - Temperature
KW - carbon 14
KW - astronomy
KW - biological model
KW - extraterrestrial life
KW - photochemistry
KW - preliminary communication
KW - pyrolysis
KW - Mars
KW - Photocatalytic synthesis
KW - Pyrolytic release experiment
KW - Viking Mission
PB - Springer-Verlag
SN - 14321432 (ISSN)
C2 - 522153
LA - English
J2 - J Mol Evol
M3 - Article
DB - Scopus
N1 - Export Date: 23 February 2023; Cited By: 12; CODEN: JMEVA
ER -

TY - JOUR
AU - Levin, G.V.
AU - Straat, P.A.
TI - Laboratory simulations of the Viking Labeled Release

experiment: Kinetics following second nutrient injection and the nature of the gaseous end product
PY - 1979
T2 - Journal of Molecular Evolution
VL - 14
IS - 1-3
SP - 185
EP - 197
DO - 10.1007/BF01732377
UR - https://www.scopus.com/inward/record.uri?eid=2-s2.0-0018634981&doi=10.1007%2fBF01732377&partnerID=40&md5=6dc7df71071a43c107f04c01aab78a70
AD - Biospherics Incorporated, Rockville, 20852, Maryland, 4928 Wyaconda Road, United States
AB - Injection of14C-labeled nutrient onto Mars soil produced an evolution of14C gas in the Viking Labeled Release (LR) experiment. However, a second injection of nutrient seven days later was followed by an abrupt diminution of the amount of radioactive gas in the test cell. Simulation experiments performed in the LR Test Standards Module (TSM) have yielded a plausible explanation for this diminution. Radioactive carbon gases were injected into the TSM test cell in the presence and absence of two Mars analog soils. After equilibration, water was injected and its effect observed. The results indicate that the flight data following second nutrient injection can be explained on a physico-chemical basis involving a carbon dioxide/water/soil equilibrium in the test cell. The results also suggest that the gaseous end product of the Labeled Release reaction on Mars is more likely carbon dioxide than carbon monoxide.
© 1979 Springer-Verlag.
KW - Biogenesis
KW - Carbon Radioisotopes
KW - Extraterrestrial Environment
KW - Gases
KW - Isotope Labeling
KW - Soil
KW - Support, U.S. Gov't, Non-P.H.S.
KW - Time Factors
KW - Water
KW - carbon 14
KW - carbon dioxide
KW - carbon monoxide
KW - organic compound
KW - radioisotope
KW - astronomy
KW - biological model
KW - chemical reaction kinetics
KW - extraterrestrial life
KW - preliminary communication
KW - Extraterrrestrial life detection
KW - Labeled release experiment
KW - Viking biology
PB - Springer-Verlag
SN - 14321432 (ISSN)
C2 - 534573

LA - English
J2 - J Mol Evol
M3 - Article
DB - Scopus
N1 - Export Date: 23 February 2023; Cited By: 17; CODEN: JMEVA
ER -

TY - JOUR
AU - Oyama, V.I.
AU - Berdahl, B.J.
TI - A model of Martian surface chemistry
PY - 1979
T2 - Journal of Molecular Evolution
VL - 14
IS - 1-3
SP - 199
EP - 210
DO - 10.1007/BF01732378
UR - https://www.scopus.com/inward/record.uri?eid=2-s2.0-0018575635&doi=10.1007%2fBF01732378&partnerID=40&md5=3b315f3149143a648a5914982b89380f
AD - Extraterrestrial Research Division, NASA-Ames Research Center, Moffett Field, 94035, CA, United States
AB - Alkaline earth and alkali metal superoxides and peroxides, γ-Fe2O3 and carbon suboxide polymer are proposed to be constituents of the Martian surface material. These reactive substances explain the water modified reactions and thermal behaviors of the Martian samples demonstrated by all of the Viking Biology Experiments. It is also proposed that the syntheses of these substances result mainly from electrical discharges between wind-mobilized particles at Martian pressures; plasmas are initiated and maintained by these discharges. Active species in the plasma either combine to form or react with inorganic surfaces to create the reactive constitutents. © 1979 Springer-Verlag.
KW - Carbon Dioxide
KW - Chemistry
KW - Electrochemistry
KW - Extraterrestrial Environment
KW - Models, Theoretical
KW - Oxygen
KW - Peroxides
KW - Soil
KW - Superoxides
KW - Water
KW - alkali metal
KW - peroxide
KW - superoxide
KW - astronomy
KW - biological model
KW - carbon suboxide
KW - extraterrestrial life
KW - preliminary communication
KW - Carbon suboxide
KW - Carbon suboxide polymer

KW - Electrical discharge
KW - Martian surface chemistry
KW - Peroxides
KW - Plasma
KW - Superoxides
KW - Water on Mars
PB - Springer-Verlag
SN - 14321432 (ISSN)
C2 - 230358
LA - English
J2 - J Mol Evol
M3 - Article
DB - Scopus
N1 - Export Date: 23 February 2023; Cited By: 47; CODEN: JMEVA
ER -

TY - CONF
AU - Martin, Anthony R.
AU - Bond, Alan
TI - IS MANKIND UNIQUE IN THE GALAXY?
PY - 1984
UR - https://www.scopus.com/inward/record.uri?eid=2-s2.0-0021652765&partnerID=40&md5=ecf4d0d886b3b7155858ab61a1583933
AD - Culham Lab, Abingdon, Engl, Culham Lab, Abingdon, Engl
AB - If intelligent life is a common occurrence in the Galaxy, then many civilisations should have arisen which by now are far in excess of mankind in both their age and their capabilities. It is to be expected on technical grounds that some of these should have crossed the threshold of their home planetary system and ventured out into interstellar space. In a time that is short when measured against astronomical timescales such civilisations could have colonised the planets orbiting every suitable star in the Galaxy. As the Solar System is relatively young in the Galaxy, there should never have been a time in history when mankind was not aware of the presence of such civilisations. And yet, no signs of intelligent life in the Galaxy other than ourselves are seen. This paradox was first expressed by Enrico Fermi 40 years ago, and today is still as relevant to discussions on the possible existence of intelligent life in the Universe and the methods of searching for signs of such life as it was then. The authors have reviewed the many aspects of the Fermi Paradox for the last decade. This paper is an attempt to contribute to the debate by exploring some of the arguments prompted by the Paradox. It is concluded that all of these arguments have only one self-consistent resolution namely that mankind is unique in the Galaxy.
KW - SPACE RESEARCH
KW - EXTRATERRESTRIAL LIFE
KW - FERMI PARADOX
KW - SEARCH FOR EXTRATERRESTRIAL INTELLIGENCE (SETI)
KW - ASTRONOMY
PB - AIAA
LA - English
M3 - Conference paper
DB - Scopus

N1 - Export Date: 23 February 2023; Cited By: 1
ER -

TY - CONF
AU - Freitas Jr., Robert A.
AU - Valdes, Francisco
TI - SEARCH FOR EXTRATERRESTRIAL ARTIFACTS (SETA).
PY - 1984
UR - https://www.scopus.com/inward/record.uri?eid=2-s2.0-0021652764&partnerID=40&md5=cc3a1da480ec9b78edd7e237b75dc6ee
AD - Xenology Research Inst, Sacramento,, CA, USA, Xenology Research Inst, Sacramento, CA, USA
AB - The rationale for the use of interstellar artifacts by intelligent life in the universe is described. Four classes of artifacts are defined: those seeking contact, those seeking to avoid contact, those intended to provide a passive technological threshold for detection, and those for which detection is irrelevant. The Search for Extraterrestrial Artifacts (SETA) is based on the latter two classes. Under the assumption that an extraterrestrial probe will be interested in life in the solar system, a near-Earth search space is defined.
KW - RADAR - Space Applications
KW - SPACE PROBES
KW - TELESCOPES - Space Applications
KW - EARTH-MOON ORBITS
KW - EARTH-SUN ORBITS
KW - EXTRATERRESTRIAL LIFE
KW - INTERSTELLAR PROBES
KW - SEARCH FOR EXTRATERRESTRIAL ARTIFACTS (SETA)
KW - ASTRONOMY
PB - AIAA
LA - English
M3 - Conference paper
DB - Scopus
N1 - Export Date: 23 February 2023; Cited By: 0
ER -

TY - JOUR
TI - BOOK REVIEWS
PY - 1987
T2 - The Heythrop Journal
VL - 28
IS - 4
SP - 441
EP - 501
DO - 10.1111/j.1468-2265.1987.tb00106.x
UR - https://www.scopus.com/inward/record.uri?eid=2-s2.0-84978585067&doi=10.1111%2fj.1468-2265.1987.tb00106.x&partnerID=40&md5=1dd1ab427995dcba75356d57b069adff
AB - Philosophy in Russia: From Herzen to Lenin and Berdyaev. By Frederick C. Copleston. pp.x, 445, Tunbridge Wells, Search Press, 1986, £25.00. Philosophy's Place in Culture: a Model. By Mark D. Morelli. pp. xv, 154, Lanham MD., University Press of America, 1984, $20.00 (hardback), $10.50 (paperback). Kant's Transcendental

Idealism: An Interpretation and Defense. By Henry E. Allison, pp. ix, 389, New Haven & London, Yale University Press, 1983, £29.00. The Science of the Mind. By Owen J. Flanagan, Jr. pp. xii, 336, Cambridge (Mass.), The MIT Press; London, 1984, £23.75 (hardback) £11.90 (paperback). Freud and the Mind. By Ilham Oilman. pp. vii, 204, Oxford, Basil Blackwell, 1984, £15.00. Free Will and Responsibility. By Jennifer Trusted. pp. x, 195, Oxford University Press, 1984, £12.50 (hardback), £3.95 (paperback). The Personalist Challenge: Intersubjectivity and Ontology. By Maurice Nédoncelle, translated by Francois C. Gérard and Francis F. Burch. pp. xvi.203, Allison Park, Pennsylvania, Pickwick Publications, 1984, no price given. Reading the Old Testament: Method in Biblical Study. By John Barton. pp. xvi,256. London, Darton, Longman and Todd, 1984, £7.95. Hosea: an Israelite Prophet in Judean Perspective. By Grace I. Emmersqn. pp. xi;224 (Journal for the Study of the Old Testament Supplement Series 28), Sheffield, JSOT Press, 1984, £18.50 (hard-back), £7.50 (paperback). Jesus and the Politics of His Day. Edited by Ernst Bammel and C.D.F. Moule. pp. xi,511, Cambridge University Press, 1984, £37.50. Matthew and Paul: A Comparison of Ethical Perspectives. By Roger Mohrlang. pp. xiii, 242, (SNTS Monographs No.48), Cambridge University Press, 1984, £18.00. St Paul's Corinth: Texts and Archaeology. By Jerome Murphy-O'Connor. pp. xxi, 192 (Good News Studies 6), Wilmington (Delaware), Michael Glazier: Dublin, Veritas, 1984, £9.95. Crisis and Catharsis: The Power of the Apocalypse. By Adela Yarbro Collins. pp. 179, Philadelphia, The Westminster Press, 1984, $11.95. The Apocalypse: Biblical Revelation Explained. By Patrick J. Sena. pp. viii, 116, New York, Alba House, 1983, $6.95. The Eucharist in Bible and Liturgy. By G.D. Kilpatrick. pp. vii, 115. Cambridge University Press, 1984, £15.00. Medieval Theory of Authorship: Scholastic Literary Attitudes in the Later Middle Ages. By A.J. Minnis. pp. xviii, 323, London, Scolar Press, 1984, £35.00. Die griechische Gregoriosanaphora. Ein Beitrag zur Geschichte des eucharistischen Hochgebets. By Albert Gerhards. pp. xxiv, 261 (Liturgiewissenschaftliche Quellen und Forschungen 65), Münster, Aschendorff, 1984, DM78. Remembering the Future: Vatican II and Tomorrow's Liturgical Agenda. Edited by Carl A. Last. pp. 113, New York, Paulist Press, 1984, £5.00. On Liturgical Theology. By Aidan Kavanagh. pp. xiii,205, New York, Pueblo Publishing, 1984, No price given. The Promise of Narrative Theology. By George W. Stroup. pp. 288, London, SCM, 1984, £7.95. Conoscenza e Interiorità. By Bernard Lonergan, translated by Natalino Spaccapelo, pp. 262 (Nuovi Saggi Teologici, n.21), Bologna, Edizioni Dehoniane, 1984, 16,000 It. Lire. Justification and Sanctification. By Peter Toon, pp. 162, (Foundations for Faith Series), London, Marshall Morgan and Scott, 1983, £5.95. Justification: An Ecumenical Study. By George Tavard. pp. vi, 137, Ramsey, N.J., Paulist Press, 1983, $7.95. L'Uomo tra Legge e Grazia: Analisi teologica del 'De Spiritu et Littera' di S. Agostino. By Domenico Marafioti, S.J. pp. 249, Brescia, Morcelliana, 1983, It. Lire 22,000. The Lay-Centred Church. Leonard Doohan. pp. 175. Minneapolis, Winston Press, 1984, £6.95. Magisterium: Teaching Authority in the Catholic Church. By Francis A. Sullivan, pp. 234, New York, Paulist Press, 1983, $8.95; Dublin, Gill and Macmillan, 1984, £6.95. The Christians as the Romans saw Them. By Robert L.

Wilken. pp. xix,214, New Haven, Yale University Press, 1984, £12.95. Divine Pedagogy: A Patristic View of Non-Christian Religions. By Chrys Saldanha Pp. 192 (Biblioteca di Scienze Religiose 57), Rome, Libreria Ateneo Salesiano, 1984, L.20,000. The Language and Logic of the Bible: The Earlier Middle Ages. By G.R. Evans. pp. xix, 199, Cambridge University Press, 1984, £18.50. The World of John of Salisbury. Edited by Michael Wilks. pp. xii, 469 (Studies in Church History. Subsidia 3), Oxford, Basil Blackwell, for the Ecclesiastical History Society, 1984, £25.00. Robert Grosseteste De Cessatione Legalium. Edited by Richard C. Dales and Edward B. King. pp. xxx, 215 (Auctores Britannici Medii Aevi VII), London, Oxford University Press for the British Academy, 1986, £25.00. Guilds and Civil Society in European Political Thought from the Twelfth Century to the Present. By Antony Black. pp. xv, 280, London, Methuen, 1984, £20.00. Muslims, Christians and Jews in the Crusader Kingdom of Valencia. By Robert I. Burns. pp. xx, 363, Cambridge University Press, 1984, £29.50. Religion and Society in Early Modern Europe 1500–1800. Edited by Kaspar von Greyerz. pp. xii, 281, London, Allen & Unwin, 1984, £22.50. The Mathematicians' Apprenticeship: Science, Universities and Society in England, 1560–1640. By Mordecai Feingold. pp. viii, 248, Cambridge University Press, 1984, £22.50. Christian England, Vol II: From the Reformation to the 18th Century. By David L. Edwards. pp. 526 (Fount Paperbacks), London, Collins, 1984, £4.95. Christian Hebraists and Dutch Rabbis. Seventeenth Century Apologetics and the Study of Maimonides' ‘Mishneh Torah’. By Aaron L. Katchen. pp. xvii, 391, Cambridge (Mass.), Harvard University Press, 1984, £27.95. The Letters of Armand-Jean de Rancé, Abbot and Reformer of La Trappe. Presented by A.J. Krailsheimer. 2 vols. (Cistercian Studies Series 80 & 81) Kalamazoo, Cistercian Publications, 1984, no price given. Cherokees and Missionaries, 1789–1839. By William G. McLoughlin. pp. xiii, 375, New Haven and London, Yale University Press, 1984, £29.00. George Anthony Denison: The Firebrand By Joyce Coombs. pp. xxii, 244, London, The Church Literature Association, 1984, £12.50. English Catholic Modernism: Maude Petre's Way of Faith. By Clyde F. Crews. pp. xii, 156, Notre Dame (Ind.), University of Notre Dame Press; London, Burns & Oates, 1984, $16.95. Charles Williams: An Exploration of His Life and Work. By Alice Mary Hadfield. Pp. ix, 268, Oxford University Press, 1983, £15.00. Parents and Children: The Ethics of the Family. By Jeffrey Blustein. pp. 274, New York and Oxford, Oxford University Press, 1982, £8.95. Dimensions of Moral Education By Robert E. Carter, pp. xi, 242, Toronto, University of Toronto Press, 1984, no price given. Radical Departures: Desperate Detours to Growing Up. By Saul Levine. pp. xvi. 196, San Diego, Harcourt Brace Jovanovich, 1984, $15.95. Gender Politics. By Ethel Klein, pp. viii, 209, Cambridge (Mass.), Harvard University Press, 1984, £14.50. The Moral Status of Animals. By Stephen R.L Clark. pp. 221, Oxford, Clarendon Press, 1984, £3.95. The Nature of the Beast: Are Animals Moral? By Stephen R.L. Clark. Pp. vii, 127, Oxford University Press, 1982, £7.95 (hardback), 1985 (paperback), £2.95. Rights, Killing and Suffering: Moral Vegetarianism and Applied Ethics. By R.G. Frey. pp. xii, 256, Oxford, Basil Blackwell, 1983, £17.50. In Defence of Animals. Edited by Peter Singer. pp. xi, 224, Oxford, Basil Blackwell, 1985, £15.00 (hardback), £4.95

(paperback). Plurality of Worlds: The Origins of the Extraterrestrial Life Debate from Democritus to Kant. By Steven J. Dick. pp. x, 247, Cambridge University Press, 1984, £7.50. Copyright © 1987, Wiley Blackwell. All rights reserved
SN - 00181196 (ISSN)
LA - English
J2 - Heythrop J. Q. Rev. Philos. Theology
M3 - Article
DB - Scopus
N1 - Export Date: 23 February 2023; Cited By: 0
ER -

TY - JOUR
AU - Papagiannis, M.D.
TI - Toward a multi-path approach for SETI
PY - 1989
T2 - Acta Astronautica
VL - 19
IS - 11
SP - 897
EP - 902
DO - 10.1016/0094-5765(89)90081-7
UR - https://www.scopus.com/inward/record.uri?eid=2-s2.0-45349110397&doi=10.1016%2f0094-5765%2889%2990081-7&partnerID=40&md5=e2de6dd650036cbbe35e3ec3b4d390ca
AD - Department of Astronomy, Boston University, Boston, MA 02215, United States
AB - The concept of "the plurality of worlds" has been with us from the days of Ancient Greece, but only in the 19th century did we begin to consider practical means of communication, none of which, however, was implemented. In the second half of the 20th century, the advent of the space era made possible the actual search for extraterrestrial life in our solar system, and the development of radio astronomy made possible the search for radio signals from other advanced civilizations in our galaxy. It is interesting to note that our ideas about possible modes of communication have evolved hand in hand with technology and are progressively expanding toward broader concepts. Therefore, a multi-path approach for SETI appears to be a wiser strategy towards a complex problem, for which it is very difficult to predict with certainty the means and the time that other stellar civilizations (if they do exist) will select to contact us. © 1989.
SN - 00945765 (ISSN)
LA - English
J2 - Acta Astronaut
M3 - Article
DB - Scopus
N1 - Export Date: 23 February 2023; Cited By: 1; CODEN: AASTC
ER -

TY - JOUR
AU - Ponnamperuma, C.
AU - Honda, Y.
AU - Navarro-González, R.

TI - Chemical studies on the existence of extraterrestrial life.
PY - 1992
T2 - Journal of the British Interplanetary Society
VL - 45
SP - 241
EP - 249
UR - https://www.scopus.com/inward/record.uri?eid=2-s2.0-0027106785&partnerID=40&md5=4b43df1df864aba6573cfd9e151b1737
AD - Department of Chemistry and Biochemistry, University of Maryland, College Park 20742., United States
AB - Although the search for extraterrestrial intelligence has not produced any direct evidence of extraterrestrial life, the emergence of life on Earth, which appears to be controlled by universal laws of physics and chemistry, must have been repeated elsewhere in the universe. The experimental approaches in our laboratory to understand the origin of life on the Earth are summarized in an attempt to obtain a better insight into the chemical basis of extraterrestrial life.
KW - Amino Acids
KW - Biogenesis
KW - Chemistry
KW - Earth (Planet)
KW - Exobiology
KW - Extraterrestrial Environment
KW - Mars
KW - Minerals
KW - Moon
KW - Nucleotides
KW - Planets
KW - Polymers
KW - Research Design
KW - Solar System
KW - Trace Elements
KW - NASA Discipline Exobiology
KW - NASA Discipline Number 52-20
KW - NASA Program Exobiology
KW - Non-NASA Center
KW - amino acid
KW - mineral
KW - nucleotide
KW - polymer
KW - trace element
KW - astronomy
KW - biogenesis
KW - biology
KW - chemistry
KW - methodology
KW - microclimate
KW - moon
KW - NASA Discipline Exobiology
KW - NASA Discipline Number 52-20
KW - NASA Program Exobiology
KW - Non-NASA Center
KW - review

SN - 0007084X (ISSN)
C2 - 11537802
LA - English
J2 - J Br Interplanet Soc
M3 - Review
DB - Scopus
N1 - Export Date: 23 February 2023; Cited By: 5
ER -

TY - JOUR
AU - Kasting, J.F.
AU - Whitmire, D.P.
AU - Reynolds, R.T.
TI - Habitable Zones around Main Sequence Stars
PY - 1993
T2 - Icarus
VL - 101
IS - 1
SP - 108
EP - 128
DO - 10.1006/icar.1993.1010
UR - https://www.scopus.com/inward/record.uri?eid=2-s2.0-0027538885&doi=10.1006%2ficar.1993.1010&partnerID=40&md5=655b57dd7d1e2ca54452cca3e6853aa7
AD - Department of Geosciences, Penn State University, University Park, PA 16802, 211 Deike, United States
AD - Department of Physics, University of Southwestern Louisiana, Lafayette, LA 70504-4210, United States
AD - Space Science Division, NASA Ames Research Center, Moffett Field, CA 94035, MS 245-3, United States
AB - A one-dimensional climate model is used to estimate the width of the habitable zone (HZ) around our Sun and around other main sequence stars. Our basic premise is that we are dealing with Earth-like planets with CO2/H2O/N2 atmospheres and that habitability requires the presence of liquid water on the planet's surface. The inner edge of the HZ is determined in our model by loss of water via photolysis and hydrogen escape. The outer edge of the HZ is determined by the formation of CO2 clouds, which cool a planet's surface by increasing its albedo and by lowering the convective lapse rate. Conservative estimates for these distances in our own Solar System are 0.95 and 1.37 AU, respectively; the actual width of the present HZ could be much greater. Between these two limits, climate stability is ensured by a feedback mechanism in which atmospheric CO2 concentrations vary inversely with planetary surface temperature. The width of the HZ is slightly greater for planets that are larger than Earth and for planets which have higher N2 partial pressures. The HZ evolves outward in time because the Sun increases in luminosity as it ages. A conservative estimate for the width of the 4.6-Gyr continuously habitable zone (CHZ) is 0.95 to 1.15 AU. Stars later than F0 have main sequence lifetimes exceeding 2 Gyr and, so, are also potential candidates for harboring habitable planets. The HZ around an F star is larger and occurs farther out than for our Sun; the HZ around K and M stars is smaller and occurs farther in. Nevertheless, the widths of all of these HZs are

approximately the same if distance is expressed on a logarithmic scale. A log distance scale is probably the appropriate scale for this problem because the planets in our own Solar System are spaced logarithmically and because the distance at which another star would be expected to form planets should be related to the star's mass. The width of the CHZ around other stars depends on the time that a planet is required to remain habitable and on whether a planet that is initially frozen can be thawed by modest increases in stellar luminosity. For a specified period of habitability, CHZs around K and M stars are wider (in log distance) than for our Sun because these stars evolve more slowly. Planets orbiting late K stars and M stars may not be habitable, however, because they can become trapped in synchronous rotation as a consequence of tidal damping. F stars have narrower (log distance) CHZ's than our Sun because they evolve more rapidly. Our results suggest that mid-to-early K stars should be considered along with G stars as optimal candidates in the search for extraterrestrial life. © 1993 by Academic Press, Inc.
KW - Atmosphere
KW - Biogenesis
KW - Carbon Dioxide
KW - Carbonates
KW - Climate
KW - Exobiology
KW - Extraterrestrial Environment
KW - Models, Theoretical
KW - Nitrogen
KW - Planets
KW - Silicates
KW - Solar System
KW - Support, Non-U.S. Gov't
KW - Support, U.S. Gov't, Non-P.H.S.
KW - Temperature
KW - Water
KW - NASA Discipline Exobiology
KW - NASA Discipline Number 52-30
KW - NASA Program Exobiology
KW - Non-NASA Center
KW - carbon dioxide
KW - carbonic acid derivative
KW - nitrogen
KW - silicate
KW - water
KW - article
KW - astronomy
KW - atmosphere
KW - biogenesis
KW - biology
KW - chemistry
KW - climate
KW - microclimate
KW - NASA Discipline Exobiology
KW - NASA Discipline Number 52-30
KW - NASA Program Exobiology
KW - Non-NASA Center

KW - temperature
KW - theoretical model
SN - 00191035 (ISSN)
C2 - 11536936
LA - English
J2 - Icarus
M3 - Article
DB - Scopus
N1 - Export Date: 23 February 2023; Cited By: 1600
ER -

TY - JOUR
AU - Sagan, C.
AU - Thompson, W.R.
AU - Carlson, R.
AU - Gurnett, D.
AU - Hord, C.
TI - A search for life on Earth from the Galileo spacecraft
PY - 1993
T2 - Nature
VL - 365
IS - 6448
SP - 715
EP - 721
DO - 10.1038/365715a0
UR - https://www.scopus.com/inward/record.uri?eid=2-s2.0-0027790330&doi=10.1038%2f365715a0&partnerID=40&md5=917976fabe90819a9256c66196aa240f
AD - Laboratory for Planetary Studies, Cornell University, Ithaca, NY 14853, United States
AD - Atmospheric and Cometary Sciences Section, Jet Propulsion Laboratory, Pasadena, CA 91109, United States
AD - Department of Physics and Astronomy, University of Iowa, Iowa City, IA 52242-1479, United States
AD - Laboratory for Atmospheric and Space Physics, University of Colorado, Boulder, CO 80309, United States
AB - In its December 1990 fly-by of Earth, the Galileo spacecraft found evidence of abundant gaseous oxygen, a widely distributed surface pigment with a sharp absorption edge in the red part of the visible spectrum, and atmospheric methane in extreme thermodynamic disequilibrium; together, these are strongly suggestive of life on Earth. Moreover, the presence of narrow-band, pulsed, amplitude-modulated radio transmission seems uniquely attributable to intelligence. These observations constitute a control experiment for the search for extraterrestrial life by modern interplanetary spacecraft. © 1993 Nature Publishing Group.
KW - Flight Experiment
KW - Galileo Project
KW - long duration
KW - unmanned
KW - Atmosphere
KW - Earth (Planet)
KW - Exobiology
KW - Extraterrestrial Environment

KW - Methane
KW - Oxygen
KW - Radio Waves
KW - Space Flight
KW - Spacecraft
KW - Spectrophotometry, Infrared
KW - Spectrophotometry, Ultraviolet
KW - Water
KW - NASA Discipline Exobiology
KW - Non-NASA Center
KW - methane
KW - oxygen
KW - water
KW - atmospheric methane
KW - extraterrestrial life
KW - Galileo spacecraft
KW - radio transmission
KW - surface pigment
KW - article
KW - astronomy
KW - atmosphere
KW - biology
KW - chemistry
KW - Flight Experiment
KW - Galileo Project
KW - infrared spectrophotometry
KW - long duration
KW - microclimate
KW - NASA Discipline Exobiology
KW - Non-NASA Center
KW - radiofrequency radiation
KW - space flight
KW - ultraviolet spectrophotometry
KW - unmanned
SN - 00280836 (ISSN)
C2 - 11536539
LA - English
M3 - Article
DB - Scopus
N1 - Export Date: 23 February 2023; Cited By: 260
ER -

TY - JOUR
AU - Nullet, D.
TI - Unique earth
PY - 1994
T2 - Geography
VL - 79
IS - 1
SP - 77
EP - 78
UR - https://www.scopus.com/inward/record.uri?eid=2-s2.0-0028193806&partnerID=40&md5=c870d09669bdbfe461068740d69c55bc
AB - Examples of the uniqueness of the Earth's physical features

compared with planetary phenomena are suggested. Impact craters are numerous on other bodies in the solar system but scarce on the Earth. Plate tectonics and associated features seem to be confined to this planet which is contrasted with Venus and Mars. Climatically the Earth is different and the abundance of water on its surface, unique in itself, has some bearing on this. Also, the dynamic character of the earth's ice is not matched elsewhere in the solar system. While there is still speculation about extraterrestrial life, the fact that the Earth displays a continuum of biotic diversity from tropical rain forests to the bleak, almost lifeliess, extremes of heat and dryness invites comparison with how much further down this continuum the surface environments of other planets lie. It is argued that comparisons with other planets have a place in physical geography. -D.G.Price
KW - Mars
KW - Venus
KW - biosphere
KW - biotic diversity
KW - climate
KW - Earth science
KW - impact crater
KW - physical feature
KW - planetary science
KW - plate tectonics
KW - water abundance
SN - 00167487 (ISSN)
LA - English
M3 - Article
DB - Scopus
N1 - Export Date: 23 February 2023; Cited By: 0
ER -

TY - JOUR
AU - Sagan, C.
TI - The search for extraterrestrial life.
PY - 1994
T2 - Scientific American
VL - 271
IS - 4
SP - 92
EP - 99
DO - 10.1038/scientificamerican1094-92
UR - https://www.scopus.com/inward/record.uri?eid=2-s2.0-0028521155&doi=10.1038%2fscientificamerican1094-92&partnerID=40&md5=48e929641e94e8c7a0a525bd3efb1b60
AD - Laboratory for Planetary Studies, Cornell University, United States
AB - The earth remains the only inhabited world known so far, but scientists are finding that the universe abounds with the chemistry of life.
KW - Flight Experiment
KW - Galileo Project
KW - long duration
KW - unmanned

KW - Viking Project
KW - Atmosphere
KW - Earth (Planet)
KW - Evolution, Chemical
KW - Evolution, Planetary
KW - Exobiology
KW - Extraterrestrial Environment
KW - Life
KW - Mars
KW - Meteoroids
KW - Minor Planets
KW - Saturn
KW - Solar System
KW - Space Flight
KW - Spacecraft
KW - NASA Discipline Exobiology
KW - Non-NASA Center
KW - astronomy
KW - atmosphere
KW - biology
KW - chemistry
KW - Flight Experiment
KW - Galileo Project
KW - life
KW - long duration
KW - microclimate
KW - NASA Discipline Exobiology
KW - Non-NASA Center
KW - review
KW - space flight
KW - unmanned
KW - Viking Project
SN - 00368733 (ISSN)
C2 - 11536646
LA - English
J2 - Sci. Am.
M3 - Review
DB - Scopus
N1 - Export Date: 23 February 2023; Cited By: 22
ER -

TY - JOUR
AU - Wharton Jr., R.A.
AU - Crosby, J.M.
AU - McKay, C.P.
AU - Rice Jr., J.W.
TI - Paleolakes on Mars
PY - 1995
T2 - Journal of Paleolimnology
VL - 13
IS - 3
SP - 267
EP - 283
DO - 10.1007/BF00682769

UR - https://www.scopus.com/inward/record.uri?eid=2-s2.0-0029414407&doi=10.1007%2fBF00682769&partnerID=40&md5=115380efba115eb2dd5c2456d60de2ff
AD - Desert Research Institute, Reno, 89506, Nevada, P.O. Box 60220, United States
AD - NASA-Ames Research Center, Moffett Field, 94035, California, United States
AD - Department of Geography, Arizona State University, Tempe, 85287, Arizona, United States
AB - Observational evidence such as outflow channels and valley networks suggest that in the past there was flowing water on Mars. The images of fluvial features on Mars logically suggest that there must exist downstream locations in which the water pooled and the sediment load deposited (i.e. lakes). Sediments and morphological features associated with the martian paleolakes are believed to occur in Valles Marineris, and several large basins including Amazonis, Chryse and Elysium planitia. As Mars became progressively colder over geological time, any lakes on its surface would have become seasonally, and eventually perennially ice-covered. We know from polar lakes on Earth that ice-covered lakes can persist even when the mean annual temperature falls below freezing. Thus, the most recent lacustrine sediments on Mars were probably deposited in ice-covered lakes. While life outside of the Earth's atmosphere has yet to be observed, there is a general consensus among exobiologists that the search for extraterrestrial life should be based upon liquid water. The inference that there was liquid water on Mars during an earlier epoch is the primary motivation for considering the possibility of life during this time. It would be of enormous interest from both an exobiological and paleolimnological perspective to discover lakes or the evidence of former lakes on another planet such as Mars. Limnology would then become an interplanetary science. © 1995 Kluwer Academic Publishers.
KW - Atmosphere
KW - Carbon Dioxide
KW - Climate
KW - Evolution, Planetary
KW - Exobiology
KW - Extraterrestrial Environment
KW - Geologic Sediments
KW - Ice
KW - Mars
KW - Paleontology
KW - Water
KW - Mars
KW - NASA Center ARC
KW - NASA Discipline Exobiology
KW - Non-NASA Center
KW - carbon dioxide
KW - ice
KW - water
KW - fluvial feature
KW - ice covered lake
KW - lacustrine sediment
KW - outflow channel

KW - palaeolake
KW - valley network
KW - article
KW - astronomy
KW - atmosphere
KW - biology
KW - climate
KW - microclimate
KW - NASA Center ARC
KW - NASA Discipline Exobiology
KW - Non-NASA Center
KW - paleontology
KW - sediment
KW - ice-covered lakes
KW - lacustrine sediments
KW - Mars
KW - martian paleolakes
PB - Kluwer Academic Publishers
SN - 15730417 (ISSN)
C2 - 11539841
LA - English
J2 - J Paleolimnol
M3 - Article
DB - Scopus
N1 - Export Date: 23 February 2023; Cited By: 40; CODEN: JOUPE
ER -

TY - JOUR
AU - Crowe, M.J.
TI - A history of the extraterrestrial life debate
PY - 1997
T2 - Zygon
VL - 32
IS - 2
SP - 147
EP - 162
DO - 10.1111/0591-2385.801997079
UR - https://www.scopus.com/inward/record.uri?eid=2-s2.0-53249098774&doi=10.1111%2f0591-2385.801997079&partnerID=40&md5=046e8c9215b529822025fc90be1c9799
AD - Program of Liberal Studies, Graduate Program in History and Philosophy of Science, University of Notre Dame, United States
AD - Program of Liberal Studies, University of Notre Dame, Notre Dame, IN 46556, United States
AB - From antiquity to the present, humans have debated whether intelligent life exists elsewhere in the universe. This presentation will survey this debate, examining the roles played in it by science, religion, philosophy, and other areas of human learning. One thesis that will be developed is that whether or not extraterrestrials exist, ideas about them have strongly influenced Western thought. © 1997 by the Joint Publication Board of Zygon.
KW - Exobiology
KW - Extraterrestrial life ideas
KW - Plurality of worlds

KW - Religion
KW - Theology
PB - Blackwell Publishing Ltd
SN - 05912385 (ISSN)
LA - English
J2 - Zygon
M3 - Article
DB - Scopus
N1 - Export Date: 23 February 2023; Cited By: 25
ER -

TY - CONF
AU - Edwards, B.C.
AU - Chyba, C.F.
AU - Abshire, J.B.
AU - Burns, J.A.
AU - Geissler, P.
AU - Konopliv, A.S.
AU - Malin, M.C.
AU - Ostro, S.J.
AU - Rhodes, C.
AU - Rudiger, C.
AU - Shao, X.-M.
AU - Smith, D.E.
AU - Squyres, S.W.
AU - Thomas, P.C.
AU - Uphoff, C.W.
AU - Walberg, G.D.
AU - Werner, C.L.
AU - Yoder, C.F.
AU - Zuber, M.T.
TI - The Europa Ocean Discovery mission
PY - 1997
T2 - Proceedings of SPIE - The International Society for Optical Engineering
VL - 3111
SP - 249
EP - 261
DO - 10.1117/12.278778
UR - https://www.scopus.com/inward/record.uri?eid=2-s2.0-0031306426&doi=10.1117%2f12.278778&partnerID=40&md5=300605518cf02d46e81faa33ad3624e5
AD - Los Alamos National Laboratory, Los Alamos, NM, United States
AB - Ever since the first proposal that tidal heating of Europa by Jupiter might lead to liquid water oceans below Europa's ice cover1,2,3,4, scientists have speculated over the exobiological implications of such an ocean. Liquid water is thought to be an essential ingredient for life, so the existence of a second water ocean in the Solar System would be of paramount importance in any search for life beyond Earth. We present here a Discovery-class mission concept (Europa Ocean Discovery) to determine the existence of a liquid water ocean on Europa and to characterize Europa's surface structure. The technical goal of the Europa Ocean Discovery mission is to study Europa with an orbiting spacecraft. This goal is

challenging but entirely feasible within the Discovery envelope. There are four key challenges: entering Europan orbit, generating power, surviving long enough in the radiation environment to return valuable science, and completing the mission within the Discovery program's constraints on launch vehicle (Delta II or smaller) and budget (~$250M plus launch). Europa Ocean Discovery will carry four scientific instruments to study Europa: (1) An ice-penetrating radar sounder to probe tens of kilometers below Europa's surface; (2) A laser altimeter, to determine the height and phase of Europa's time-varying tidal bulge; (3) An X-band transponder to determine Europa's gravity field; and (4) A solid-state optical imager. These instruments will provide important information about Europa's surface, subsurface, and will provide definitive evidence about the existence of a Europan ocean.
KW - Aneroid altimeters
KW - Optical radar
KW - Solar system
KW - Spacecraft
KW - Surface structure
KW - Orbiting spacecraft
KW - Space research
KW - Discovery program
KW - Europa
KW - Extraterrestrial life
KW - Jupiter
KW - Lidar
KW - Ocean
KW - Radar
KW - Solar system exploration
KW - Spacecraft
SN - 0277786X (ISSN)
LA - English
J2 - Proc SPIE Int Soc Opt Eng
M3 - Conference paper
DB - Scopus
N1 - Export Date: 23 February 2023; Cited By: 4; CODEN: PSISD
ER -

TY - CONF
AU - Koenig, D.W.
AU - Bell-Robinson, D.M.
AU - Valadez, V.A.
AU - Pierson, D.L.
TI - Microbial colonization of closed life support chambers
PY - 1997
T2 - SAE Technical Papers
DO - 10.4271/972414
UR - https://www.scopus.com/inward/record.uri?eid=2-s2.0-85072430575&doi=10.4271%2f972414&partnerID=40&md5=98545d6cb888adce4bcb3e8322199e1c
AD - KRUG Life Sciences Inc., United States
AD - NASA-Johnson Space Center, United States
AB - The first two phases of the Lunar-Mars Life Support Test Project [LMLSTP] involved housing human volunteers in closed

chambers that mimic future extraterrestrial life support systems. The Phase I test involved one person living for 15 days in a chamber with wheat as the primary means of air revitalization. The Phase II test involved 4 people living for 30 days in a chamber with physical/chemical air revitalization and waste water recycling. The consequences of closure on microbial ecology and the influence that microbes had on these closed environmental life support systems were determined during both tests. The air, water, and surfaces of each chamber were sampled for microbial content before, during, and after each test. The numbers of microbes on the Phase I habitation chamber surfaces increased with length of occupation. However, the numbers of microbes on the surfaces of the Phase II chamber did not increase, a finding that contrasts with past results from other closed chamber studies and from Space Shuttle missions. The types of microbes found in the air and on the surfaces from both tests were similar. The most common bacteria were Bacillus sp. and Staphylococcus sp., with Pencillium sp. and Cladosporium sp. the most common fungi. Neither test had levels or types of microbes that would be considered an occupational health threat. One explanation for the lack of microbial colonization of the chamber surfaces during the Phase II test is that the air-handling system of this chamber was designed for habitation by 8 individuals, but the test was conducted with only 4. Moreover, the relative humidity of the chambers was maintained at 95% during Phase I and at 40% during Phase II. The lower humidity may have contributed to the lesser microbial load during the Phase II test. Results from Phase I and II tests imply that human flora may ultimately dominate the ecology of a closed life support system. Nevertheless, the effect of long-term closure on the microflora cannot be fully appreciated at this time because of the relatively short evaluation periods.
KW - Bacteria
KW - Bacteriology
KW - Ecology
KW - Health risks
KW - Industrial hygiene
KW - Space shuttles
KW - Wastewater reclamation
KW - Air handling systems
KW - Extraterrestrial life
KW - Microbial colonization
KW - Microbial contents
KW - Microbial ecology
KW - Occupational health
KW - Space shuttle missions
KW - Staphylococcus sp
KW - Water recycling
PB - SAE International
SN - 01487191 (ISSN)
LA - English
J2 - SAE Techni. Paper.
M3 - Conference paper
DB - Scopus
N1 - Export Date: 23 February 2023; Cited By: 2
ER -

TY - JOUR
AU - Galimov, E.M.
TI - Concerning the existence of life on mars
PY - 1997
T2 - Solar System Research
VL - 31
IS - 3
SP - 183
EP - 190
UR - https://www.scopus.com/inward/record.uri?eid=2-s2.0-27644450445&partnerID=40&md5=bb42b0de97d937ef50ee6742cce7ad7b
AD - Vernadsky Institute of Geochemistry and Analytical Chemistry, Russian Academy of Sciences, Moscow, 117975, ul. Kosygina 19, Russian Federation
AB - In a paper published in the August issue of Science, the American researchers McKay et al. (1996) reported the results of their investigation of the Martian Antarctic meteorite ALH 84001. They regard these results as evidence of the existence (in the past, at any rate) of life on Mars. However, a study of the geochemical data in the paper suggests that simple inorganic processes offer a more plausible explanation of the results. Thermodynamic analysis reveals that a mineral association, including pyrrhotite, magnetite, and magnesite, is quite stable in a hydrothermal system at a temperature of the order of 150°C and does not require invoking a biological mechanism. The interpretation by McKay et al. (1996), based on the isotopic compositions of oxygen and carbon, led them to conclude, as Romanek et al. (1994) concluded, that the formation of carbonate globules was a low-temperature (from 0 to 80°C) process. This conclusion has been made based on less than convincing assumptions. The very same isotope data suggest that the globules are more likely to have formed as a result of a high-temperature (~700°C) process, with the subsequent superimposition of a hydrothermal alteration process at about 150°C. Polycyclic aromatic hydrocarbons could have arisen abiogenically in the same processes. The intriguing fact remains that the discovered structures have a specific morphology. However, it cannot be ruled out that such structures are trivial in the inorganic world as well on a nanometric scale. Nevertheless, the work of McKay et al. (1996) is important since it shows that a combination of state-of-the-art analytical techniques, in principle, provides an approach to both the problem of the existence of extraterrestrial life and the problem of the origin of life on Earth. © 1997 MAHK Hayka/ Interperiodica Publishing.
SN - 00380946 (ISSN)
LA - English
J2 - Sol. Syst. Res.
M3 - Article
DB - Scopus
N1 - Export Date: 23 February 2023; Cited By: 5
ER -

TY - CONF
AU - Horvath, J.

AU - Carsey, F.
AU - Cutts, J.
AU - Jones, J.
AU - Johnson, E.
AU - Landry, B.
AU - Lane, L.
AU - Lynch, G.
AU - Jezek, K.
AU - Chela-Flores, J.
AU - Jeng, T.-W.
AU - Bradley, A.
TI - Searching for ice and ocean biogenic activity on Europa and Earth
PY - 1997
T2 - Proceedings of SPIE - The International Society for Optical Engineering
VL - 3111
SP - 490
EP - 500
DO - 10.1117/12.278805
UR - https://www.scopus.com/inward/record.uri?eid=2-s2.0-0031310647&doi=10.1117%2f12.278805&partnerID=40&md5=32ffb845dc9b9941d3a9ee0fe35d76f0
AD - Jet Propulsion Laboratory, California Institute of Technology, Pasadena, CA 91109, 4800 Oak Grove Drive, United States
AB - One of the more likely places in the solar system for the existence of extraterrestrial life forms is the Jovian moon Europa. It has been postulated that a volcanically-heated ocean is likely to exist underneath Europa's icy surface. If a detailed remote-sensing reconnaissance of Europa determines that an ocean does exist under the ice, then in-situ measurements will be needed to directly explore the Europan ocean and the ice that lies above it. In order to make quantitative measurements of the Europan environment, a lander spacecraft capable of penetrating the surface ice layer by melting through it is proposed. This vehicle, dubbed a "Cryobot", will be designed to carry a small deployable submersible (a "Hydrobot") equipped with a complement of instruments. The design of an instrument package to search for life across the wide range of thermal and pressure environments expected on Europa, the issues in sample handling, and long-term reliability for a potential multi-year transit through the ice all present difficult design issues. Opportunities for performing investigations of deep, submerged Antarctic lakes on Earth are described which would test the Cryobot/Hydrobot system while collecting intrinsically valuable terrestrial science data.
KW - Extraterrestrial atmospheres
KW - Ice
KW - Oceanography
KW - Remote sensing
KW - Solar system
KW - Spacecraft
KW - Biogenic activity
KW - Planetary surface analysis
KW - Europa

KW - Exobiology
KW - Ice probes
KW - Instrumentation
KW - Submersibles
SN - 0277786X (ISSN)
LA - English
J2 - Proc SPIE Int Soc Opt Eng
M3 - Conference paper
DB - Scopus
N1 - Export Date: 23 February 2023; Cited By: 13; Correspondence Address: J. Horvath; Jet Propulsion Laboratory, California Institute of Technology, Pasadena, CA 91109, 4800 Oak Grove Drive, United States; email: joan.c.horvath@jpl.nasa.gov; CODEN: PSISD
ER -

TY - JOUR
AU - Sweetser, T.H.
AU - Maddock, R.
AU - Johannesen, J.
AU - Bell, J.
AU - Penzo, P.A.
AU - Wolf, A.
AU - Williams, S.N.
AU - Matousek, S.
AU - Weinstein, S.
TI - Trajectory design for a Europa orbiter mission: A plethora of astrodynamic challenges
PY - 1997
T2 - Advances in the Astronautical Sciences
VL - 95 PART 2
SP - 901
EP - 919
UR - https://www.scopus.com/inward/record.uri?eid=2-s2.0-0006139752&partnerID=40&md5=abee62ad9d83212ba61acb92f29a6c9c
AD - Jet Propulsion Laboratory, California Lnstutute of Technology, Pasadena, CA 91109-8099, 4800 Oak Grove Drive, United States
AB - The icy moon of Europa is a hot topic in planetary exploration. This paper discusses the trajectory design of a Europa orbiter mission intended to find out if Europa is a possible habitat for extraterrestrial life. Getting into orbit at Europa is difficult; the simplest trajectory design (a direct Hohmann-type transfer to Jupiter followed by an immediate insertion into orbit at Europa) requires more than 5.5 km/s of velocity change (ΔV) capability by the spacecraft. This paper describes a number of trajectory design techniques which can reduce this ΔV requirement step by step down to around 2.5 km/s. The variety of trajectory "tricks" involved make this mission a showcase of modern trajectory design.
SN - 00653438 (ISSN)
LA - English
J2 - Adv Astronaut Sci
M3 - Article
DB - Scopus
N1 - Export Date: 23 February 2023; Cited By: 34; CODEN: ADASA

ER -

TY - CONF
AU - Chela-Flores, J.
TI - Europa: A potential source of parallel evolution for micro-organisms
PY - 1998
T2 - Proceedings of SPIE - The International Society for Optical Engineering
VL - 3441
SP - 55
EP - 67
DO - 10.1117/12.319857
UR - https://www.scopus.com/inward/record.uri?eid=2-s2.0-0038336055&doi=10.1117%2f12.319857&partnerID=40&md5=f1bdeed1b0e207576b770c390dc0c673
AD - Abdus Salam Intl. Ctr. Theor. Phys., 34136 Trieste, Miramare, P.O.Box 586, Italy
AD - Instituto de Estudios Avanzados, Universidad Simon Bolivar, Caracas 1015A, Apartado 17606 Parque Central, Venezuela
AB - Europa, the ice-covered satellite of Jupiter, is currently the most favourable site for the search of extraterrestrial life. Hydrothermal vents on the Earth's sea floor have been found to sustain life forms that can live without solar energy. Similar possible volcanic activity on Europa, caused by its interaction with Jupiter and the other Galilean satellites, makes this Jovian moon the best target for identifying a separate evolutionary line. This search addresses the main problem remaining in astrobiology, namely, the quest for discrete, or 'parallel' evolutionary lines in the universe. We explore ideas related to Europa's possible biological activity, particularly its likely degree of evolution. We have conjectured that evolution may have occurred in Europa and that the experimental test of such a conjecture is feasible: A lander space craft capable of penetrating the European surface ice-layer does not seem beyond present technological capabilities. Although difficult instrumentation issues are involved, we have initiated the discussion of what would seem to be a reasonable biological experiment. The possibility of detecting biomolecules on the ice surface of Europa has recently been made by others. A possible mechanism for bringing biomolecules to Europa's surface will be critically reviewed.
KW - Astrophysics
KW - Extraterrestrial atmospheres
KW - Lunar surface analysis
KW - Microorganisms
KW - Molecules
KW - Planets
KW - Satellites
KW - Solar energy
KW - Astrobiology
KW - Extraterrestial life
KW - Ice layer
KW - Jupiter
KW - Parallel evolution

KW - Solar system exploration
KW - Microbiology
KW - Astrobiology
KW - Distribution of life in the universe
KW - Eukaryogenesis
KW - Europa
KW - Instrumentation
KW - Solar system exploration
SN - 0277786X (ISSN)
LA - English
J2 - Proc SPIE Int Soc Opt Eng
M3 - Conference paper
DB - Scopus
N1 - Export Date: 23 February 2023; Cited By: 2; CODEN: PSISD
ER -

TY - CONF
TI - Proceedings of SPIE: Instruments, Methods, and Missions for Astrobiology
PY - 1998
T2 - Proceedings of SPIE - The International Society for Optical Engineering
VL - 3441
UR - https://www.scopus.com/inward/record.uri?eid=2-s2.0-0038674391&partnerID=40&md5=0f247d71bb302cf1beedeca79740e3fe
AB - This Volume 3441 of the conference proceedings contains 34 papers. Topics discussed include instruments, method, and missions for astrobiology, remote sensing and spectroscopy, extremophile of relevance to astrobiology, halophiles and lithoautotrophs, nanobacteria, ancient terrestial and marine microorganisms, biomarkers and microfossils in rock and meteorites and viability and transport of microorganisms in the space environment.
KW - Astronomy
KW - Bacteria
KW - Biology
KW - Biomarkers
KW - Cosmic rays
KW - Martian surface analysis
KW - Meteorites
KW - Microorganisms
KW - Remote sensing
KW - Rocks
KW - Space research
KW - Spectroscopy
KW - Astrobiology
KW - Bacterial paleontology
KW - Cyanobacteria
KW - EiRev
KW - Extremophiles
KW - Hydrocarbon crystals
KW - Marine microorganisms
KW - Microfossils
KW - Nanobacteria
KW - Phosphate biomineralization

KW - Optical variables measurement
SN - 0277786X (ISSN)
LA - English
J2 - Proc SPIE Int Soc Opt Eng
M3 - Conference review
DB - Scopus
N1 - Export Date: 23 February 2023; Cited By: 0; CODEN: PSISD
ER -

TY - CONF
AU - Levin, G.V.
AU - Levin, R.L.
TI - Liquid water and life on Mars
PY - 1998
T2 - Proceedings of SPIE - The International Society for Optical Engineering
VL - 3441
SP - 30
EP - 43
DO - 10.1117/12.319849
UR - https://www.scopus.com/inward/record.uri?eid=2-s2.0-0002821482&doi=10.1117%2f12.319849&partnerID=40&md5=a1dc0ecbc93c09f8eb6534ce40a18069
AD - Biospherics Incorporated, Beltsville, MD 20705, 12051 Indian Creek Court, United States
AD - Lincoln Laboratory, Massachusetts Inst. of Technology, Lexington, MA 02173-0073, United States
AB - Many objections have been raised to challenge a biological interpretation of the 1976 Viking Mission Labeled Release (LR) life detection experiment on Mars. Over the years, they have dwindled in the face of the failure of experiments and theories to demonstrate a nonbiological alternative. Recently, NASA's chief scientist, responding to the rapidly accumulating knowledge about life in extreme environments, reduced the remaining obstacles to a single one: the lack of liquid water. A model for the diurnal presence of precipitable micron amounts of liquid water over large areas of Mars is presented. The model is consistent with the thermodynamics of the triple point of water. Viking and Pathfinder meteorological data are congruent with the model, as are Viking Lander images of deposits of water ice-frost and snow on the ground. The amounts of soil moisture predicted by the model are within the moisture content range of terrestrial soils in which the LR detected living microorganisms. The last objection to a biological interpretation of the LR Mars data is thus met. Consequential recommendations for the near-term planetary program are made.
KW - Extraterrestrial atmospheres
KW - Mathematical models
KW - Meteorology
KW - Microorganisms
KW - Moisture
KW - Planets
KW - Soils
KW - Space applications
KW - Thermodynamic properties

KW - Water
KW - Biological interpretations
KW - Mars
KW - Micron amounts
KW - Soil moisture
KW - Terrestial soils
KW - Biology
KW - Extraterrestrial life
KW - Life detection
KW - Life on Mars
KW - Mars
KW - Viking labeled release experiment
KW - Water on Mars
SN - 0277786X (ISSN)
LA - English
J2 - Proc SPIE Int Soc Opt Eng
M3 - Conference paper
DB - Scopus
N1 - Export Date: 23 February 2023; Cited By: 27; CODEN: PSISD
ER -

TY - JOUR
AU - Donnelly, C.
AU - Werthimer, D.
AU - Bowyer, S.
AU - Cobb, J.
AU - Malina, R.F.
TI - The serendip SETI project: New instrumentation for SETI and results of recent observations
PY - 1998
T2 - Acta Astronautica
VL - 42
IS - 10-12
SP - 655
EP - 659
DO - 10.1016/S0094-5765(98)00021-6
UR - https://www.scopus.com/inward/record.uri?eid=2-s2.0-0032064395&doi=10.1016%2fS0094-5765%2898%2900021-6&partnerID=40&md5=137f7cf8a9451e4a6076118996a95884
AD - Space Sciences Laboratory, University of California, Berkeley, CA 94720-7450, United States
AD - Laboratoire d'Astronomie Spatiale, CNRS, Marseille, Cedex 12, BP 8, France
AB - Over the past 30 years research into the existence of extraterrestrial life has focused on attempts to detect stable narrowband radio signals emitted in the microwave portion of the radio frequency (RF) spectrum. The SERENDIP SETI group is currently conducting search operations on the world's largest radio telescope at the Arecibo Observatory in Puerto Rico. The third generation SERENDIP system, SERENDIP III, is a 4 million channel FFT-based spectrum analyzer with 0.6 Hz frequency resolution. In this paper, we will discuss the results of our recent 3.5 year sky survey. SERENDIP looked at 95% of the sky visible from Arecibo in the 424-436 MHz range, analyzed 1014 spectral bins, and logged

information on over 2.5 × 108 signals. The fourth generation SERENDIP system expands on the SERENDIP III design. SERENDIP IV computes 2 × 1011 operations each second, providing spectral analysis on 160 million channels in 1.7 s. We will discuss the design and use of the SERENDIP IV system and future observing plans.
© 1995 International Astronautical Federation. Published by Elsevier Science Ltd. All rights reserved.
KW - Bandwidth
KW - Communication channels (information theory)
KW - Fast Fourier transforms
KW - Microwave spectroscopy
KW - Radio telescopes
KW - Signal detection
KW - Frequency resolution
KW - Narrowband radio signal detection
KW - Search for extraterrestrial intelligence (SETI)
KW - Radio astronomy
PB - Elsevier Ltd
SN - 00945765 (ISSN)
LA - English
J2 - Acta Astronaut
M3 - Article
DB - Scopus
N1 - Export Date: 23 February 2023; Cited By: 0; CODEN: AASTC
ER -

TY - JOUR
AU - Jenniskens, P.
AU - Butow, S.J.
AU - Fonda, M.
TI - The 1999 Leonid multi-instrument aircraft campaign - An early review
PY - 1998
T2 - Earth, Moon and Planets
VL - 82-83
SP - 1
EP - 26
DO - 10.1023/a:1017020106785
UR - https://www.scopus.com/inward/record.uri?eid=2-s2.0-0034548126&doi=10.1023%2fa%3a1017020106785&partnerID=40&md5=57ed2e8804903bd52271a330828549e8
AD - NASA Ames Research Center, Mail Stop 239-4, Moffett Field, CA 94035-1000, United States
AD - SETI Institute, United States
AB - Two B707-type research aircraft of the 452nd Flight Test Squadron at Edwards Air Force Base were deployed to study the Leonid meteor storm of 1999 over the Mediterranean Sea on Nov. 18. The mission was sponsored by various science programs of NASA, and offered an international team of 35 researchers observing conditions free of clouds and low altitude extinction at a prime location for viewing the storm. This 1999 Leonid Multi-Instrument Aircraft Campaign followed a similar effort in 1998, improving upon mission strategy and scope. As before, spectroscopic and imaging experiments targeted meteors and persistent trains, but also airglow, aurora,

elves and sprites. The research aimed to address outstanding questions in astrobiology, planetary science, astronomy, and upper atmospheric research. In addition, USAF co-sponsored the mission to provide near real-time flux measurements for space weather awareness. First results are presented in these issues of Earth, Moon, and Planets in preparation for future missions that will target the exceptional Leonid returns of 2001 and 2002. An early review of the scientific achievements in the context of campaign objectives is given.
KW - meteorite
KW - Airborne astronomy
KW - Astrobiology
KW - Chemistry
KW - Comets
KW - Composition
KW - Elves
KW - Exobiology
KW - Instrumental techniques
KW - Leonid MAC
KW - Leonids 1999
KW - Lower thermosphere
KW - Mesosphere
KW - Meteor storm
KW - Meteoroids
KW - Meteors
KW - Orbital dynamics
KW - Satellite impact hazard
KW - Sprites
PB - Kluwer Academic Publishers
SN - 01679295 (ISSN)
LA - English
J2 - Earth Moon Planets
M3 - Review
DB - Scopus
N1 - Export Date: 23 February 2023; Cited By: 44; Correspondence Address: P. Jenniskens; NASA Ames Research Center, Mail Stop 239-4, Moffett Field, CA 94035-1000, United States; email: pjenniskens@mail.arc.nasa.gov
ER -

TY - CONF
AU - Sims, M.R.
AU - Whitehead, S.
AU - Mills, A.
AU - Grant, W.D.
TI - Low resource techniques for astrobiology on Mars
PY - 1998
T2 - Proceedings of SPIE - The International Society for Optical Engineering
VL - 3441
SP - 10
EP - 14
DO - 10.1117/12.319831
UR - https://www.scopus.com/inward/record.uri?eid=2-

s2.0-0010610822&doi=10.1117%2f12.319831&partnerID=40&md5=6f683e8775f113980ea6150c58e53c1b
AD - Space Research Centre, Dept. of Physics and Astronomy, University of Leicester, Leicester LE1 7RH, University Road, United Kingdom
AD - Department of Geology, University of Leicester, Leicester LE1 7RH, University Road, United Kingdom
AD - Dept. of Microbiology and Immunology, University of Leicester, Leicester LE1 7RH, University Road, United Kingdom
AB - Martian Landers have limited mass, volume, and power resources. A number of potential "low resource" techniques will be described for astrobiology on Mars. These include consideration of alternative power systems for obtaining subsurface rock samples and the adaptation of nano-technology and miniaturised sensors for use in detecting organic molecules on Mars. The techniques (including where known the advantages, and disadvantages) will be described.
KW - Astrophysics
KW - Chemical sensors
KW - Electric power systems
KW - Martian surface analysis
KW - Molecules
KW - Nanotechnology
KW - Organic compounds
KW - Space applications
KW - Astrobiology
KW - Low resource techniques
KW - martian landers
KW - Miniaturised sensors
KW - Organic molecules
KW - Biology
KW - Astrobiology
KW - Instrumentation
KW - Mars
KW - Miniaturisation
KW - Nano-technology
KW - Organic molecules
KW - Polymers
SN - 0277786X (ISSN)
LA - English
J2 - Proc SPIE Int Soc Opt Eng
M3 - Conference paper
DB - Scopus
N1 - Export Date: 23 February 2023; Cited By: 1; Correspondence Address: M.R. Sims; Space Research Centre, Dept. of Physics and Astronomy, University of Leicester, Leicester LE1 7RH, University Road, United Kingdom; email: mrs@star.le.ac.uk; CODEN: PSISD
ER -

TY - JOUR
AU - Pilcher, C.
TI - Conversations: with Carl Pilcher [interview by Johan Benson]
PY - 1998
T2 - Aerospace America
VL - 36

IS - 11
SP - 16
EP - 18
UR - https://www.scopus.com/inward/record.uri?eid=2-s2.0-0032200338&partnerID=40&md5=ae8bb8576a23638960bb3c953e4de1f8
AD - NASA Headquarters, Washington, DC, United States
AB - An interview with Carl Pilcher, science program director for solar system exploration at NASA, examines NASA's past, present, and planned missions to explore the solar system. Specific questions relate to the status of current and planned missions, science results of the Pathfinder mission to Mars, cooperation with the Japanese space agency, the status of the search for extraterrestrial life in solar system meteoroids and asteroids, mission size for more in-depth exploration, reports of water on the moon, and the exploration of near-Earth objects.
KW - Exobiology
KW - Extraterrestrial Environment
KW - Mars
KW - Minor Planets
KW - Moon
KW - Solar System
KW - Space Flight
KW - Spacecraft
KW - United States
KW - United States National Aeronautics and Space Administration
KW - article
KW - astronomy
KW - biology
KW - government
KW - interview
KW - microclimate
KW - moon
KW - space flight
KW - United States
SN - 0740722X (ISSN)
C2 - 11541412
LA - English
J2 - Aerosp Am
M3 - Article
DB - Scopus
N1 - Export Date: 23 February 2023; Cited By: 0
ER -

TY - JOUR
AU - Raulin, F.
TI - From exobiology to cosmobiology at LISA and elsewhere.
PY - 1998
T2 - Biological sciences in space = Uchū seibutsu kagaku
VL - 12
IS - 2
SP - 66
EP - 72
DO - 10.2187/bss.12.66
UR - https://www.scopus.com/inward/record.uri?eid=2-

s2.0-0032082578&doi=10.2187%2fbss.12.66&partnerID=40&md5=f0f90179a06
90eb63e24d3434309a37d
AD - LISA-Laboratoire Interuniversitaire des Systemes,
Atmospheriques, Universite Paris 7, Creteil, France
AB - Since the emergence of Exobiology, back to the l960ties, this field drastically increased and, although differently named, is today a largely recognized scientific domain of wild interdisciplinarity. It includes not only the search for extraterrestrial living Systems, in particular by direct exploration of planetary bodies and studies of extraterrestrial materials, but also the study on the origins of life on Earth and, in connection to this field, the study of extraterrestrial organic chemistry. The exobiology programmes currently developed at LISA are related to this last aspect. They include the study of prebiotic-like chemistry in the gas and solid phases, based on laboratory simulation experiments, theoretical modeling and future in situ measurements in Titan's atmosphere and in cometary nuclei. A national program of exobiology, coordinated by LISA is under development in France, it covers many of the various aspects of Exobiology, including the study of life in extreme environments, as a reference tool for extraterrestrial life, the study of the primitive environment of the Earth, of the organic chemistry in comets and on Titan, of Mars and Europa and even of extrasolar planets as potential niches for extraterrestrial living systems, associated to the determination of the electromagnetic signatures of life. In parallel to this general program, a proposal for a large simulation chamber to be used as a national facility in particular to simulate the organic chemistry in various planetary environments, and in the interstellar medium, is under preparation. International cooperations linked to these programmes, in particular in the frame of the development of an exobiology facility on the International Space Station, would be of crucial interest.
KW - Atmosphere
KW - Evolution, Chemical
KW - Exobiology
KW - Extraterrestrial Environment
KW - France
KW - Laboratories
KW - Meteoroids
KW - Research
KW - Saturn
KW - Universities
KW - article
KW - astronomy
KW - atmosphere
KW - biology
KW - chemistry
KW - France
KW - instrumentation
KW - laboratory
KW - microclimate
KW - research
KW - university
SN - 09149201 (ISSN)

C2 - 11541884
LA - English
J2 - Biol. Sci. Space
M3 - Article
DB - Scopus
N1 - Export Date: 23 February 2023; Cited By: 1; Correspondence Address: F. Raulin; email: raulin@lisa.univ-paris12.fr
ER -

TY - JOUR
AU - Burckle, L.H.
AU - Delaney, J.S.
TI - Terrestrial microfossils in Antarctic ordinary chondrites
PY - 1999
T2 - Meteoritics and Planetary Science
VL - 34
IS - 3
SP - 475
EP - 478
DO - 10.1111/j.1945-5100.1999.tb01354.x
UR - https://www.scopus.com/inward/record.uri?eid=2-s2.0-0033134060&doi=10.1111%2fj.1945-5100.1999.tb01354.x&partnerID=40&md5=271bc7e20c20b57ddbe690c2d2e27be3
AD - Lamont-Doherty Earth Observatory, Columbia University, Palisades, NY 10964, United States
AD - Department of Geological Sciences, Rutgers University, Piscataway, NJ 08854-8066, United States
AB - Microfossils have been separated and identified in four high metamorphic grade chondrites from Allan Hills and Queen Alexandra Range, Antarctica. Diatoms and opal phytoliths representing both marine and terrestrial flora were recognized among the dust removed from cracks in all meteorites studied. It is likely that contamination of Antarctic meteorites with such biogenic material is ubiquitous. Standard clean room procedures to avoid laboratory introduction of microfossils into the meteorites were followed, and the genera and species identified so far are characteristic of marine, freshwater, and continental environments. The most probable mechanism for introduction of the microfossils into the meteorites is eolian transport to and on the polar ice cap. It is likely that wind-driven systems may sample atmospherically transported material from large portions of the southern hemisphere. Entrainment of terrestrial microfossils is probably a typical interaction of meteorites with the Antarctic environment and must be recognized and accounted for in any attempt to use Antarctic meteorites as sources of extraterrestrial life forms. Organic molecules derived from microfossils are likely to be pervasive throughout any crack network present in a meteorite at all scales from millimeter to submicron. Cracks are a ubiquitous consequence of weathering in and on the Antarctic ice and the probability that crack surfaces contain terrestrial organic materials is high.
KW - Allan Hills meteorite
KW - chondrite
KW - exobiology

KW - fossil
KW - meteorite
KW - micropaleontology
PB - University of Arkansas
SN - 10869379 (ISSN)
LA - English
J2 - Meteorit. Planet. Sci.
M3 - Article
DB - Scopus
N1 - Export Date: 23 February 2023; Cited By: 8; Correspondence Address: L.H. Burckle; Lamont-Doherty Earth Observatory, Columbia University, Palisades, NY 10964, United States; email: burckle@ldeo.columbia.edu; CODEN: MPSCF
ER -

TY - CONF
AU - Hoover, Richard B.
AU - Abyzov, Sabit S.
AU - Ivanov, Mikhail V.
TI - Environmental scanning electron microscopy investigations of ancient microorganisms from deep ice above Lake Vostok
PY - 1999
T2 - Proceedings of SPIE - The International Society for Optical Engineering
VL - 3755
SP - 187
EP - 197
UR - https://www.scopus.com/inward/record.uri?eid=2-s2.0-0033326382&partnerID=40&md5=ecec74311dee0739952d6d614ff25c27
AD - NASA/Marshall Space Flight Cent, Huntsville, United States
AB - Extensive microbiological studies carried out at Vostok Station, Antarctica over the past several years have revealed a complex assemblage of well preserved microbial cells. The Antarctic ice sheet provides an ideal model for the investigation of the types of microbial forms and the state of preservation that may be expected after long term preservation in ice. The diversity of forms that are present in the Vostok core material is very great with many different taxonomic groups are represented in the ice sheet horizons above 300000 years. The microorganisms encountered include diatoms and other algae, cyanobacteria, bacteria and bacterial spores, fungi, yeasts, actinomycetes, and plant pollen. The microorganisms of the Earth's polar ice sheets provide analogs for microbiota that might be encountered in the polar ice sheets and glaciers of Mars, and the ice of comets or frozen moons such as Europa, Callisto, Ganymede, and Miranda. Since most bodies of our Solar System are frozen worlds, the microbial life of the terrestrial cryosphere is extremely important to Astrobiology. These microorganisms help establish the thermal and temporal limitations of life on Earth. The ice microbiota help define how and where we should look for signatures of life elsewhere, and they may hold clues to the distribution of life in the Cosmos. Some microorganisms can remain viable for geological periods frozen in ice. We have used the Environmental Scanning Electron Microscope at the NASA Marshall Space Flight Center to investigate the ultra-microstructure,

morphology, and chemical composition of cryopreserved microorganisms from deep ice cores just above Lake Vostok in Antarctica and a database of images has been generated.
KW - Algae
KW - Bacteria
KW - Glaciers
KW - Lakes
KW - Microstructure
KW - Morphology
KW - Scanning electron microscopy
KW - Sea ice
KW - Space research
KW - Yeast
KW - Environmental scanning electron microscopy
KW - Microbiology
PB - Society of Photo-Optical Instrumentation Engineers
SN - 0277786X (ISSN)
LA - English
J2 - Proc SPIE Int Soc Opt Eng
M3 - Conference paper
DB - Scopus
N1 - Export Date: 23 February 2023; Cited By: 10; CODEN: PSISD
ER -

TY - CONF
AU - Rosenberg, S.D.
AU - Makel, D.B.
AU - Carranza, S.
AU - Finn, J.E.
TI - Synthesis of ethylene and other useful products on spacecraft and planetary bases
PY - 1999
T2 - 37th Aerospace Sciences Meeting and Exhibit
C7 - AIAA-99-0853
DO - 10.2514/6.1999-853
UR - https://www.scopus.com/inward/record.uri?eid=2-s2.0-84983167677&doi=10.2514%2f6.1999-853&partnerID=40&md5=c0f751553cf3d30940bae857582a3ade
AD - In-Space Propulsion, Ltd., Sacramento, CA, United States
AD - Makel Engineering Inc., Chico, CA, United States
AD - NASA Ames Research Center, Moffet Field, CA, United States
AB - Advanced life support systems are essential for the success of the future human planetary exploration. Striving for self-sufficiency and autonomous operation, future life support systems will integrate physical and chemical processes with biological processes, resulting in hybrid systems. A program is being conducted to demonstrate the synthesis of ethylene and other useful products, e.g., polyethylene and ethanol, from metabolic wastes, i.e., carbon dioxide and water, as an adjunct to the Life Support Systems required in manned spacecraft, e.g., Space Station Freedom, and planetary bases, e.g., on the Moon and Mars. These products will be synthesized using inorganic processes based on chemical engineering principles, making use of the major components of metabolic waste, carbon, hydrogen and oxygen. This same technology can be used on

Mars by converting carbon dioxide (atmosphere) and water (atmosphere and soils, et.) to ethylene and other useful products for the manufacture of foodstuffs for the life support and plastics for the fabrication of structures and spare parts for Martian bases. In addition, this technology can be used for the production of oxygen and methane for use as propellants in local and space propulsion systems. The program is focused on two synthetic paths to produce ethylene, (1) direct catalytic reduction of carbon dioxide with hydrogen and (2) catalytic reforming of methane produced by the reduction of carbon dioxide with hydrogen, as shown in the following equations. The benefits to be derived from this program are: (1) conversion of metabolic wastes to useful products for use on manned spacecraft and planetary bases, (2) use of indigenous Martian resources for production of useful products for life support, base construction, and propulsion system fueling/refueling, 93) weight savings which result form reduced on-board supply requirements: (4) production of useful products based on efficient engineering principles, i.e., mass, volume, and energy, and (5) reduced resupply form Earth which enables economic exploration and colonization of space. The chemistry and chemical engineering processes which were demonstrated on the program are presented and discussed, e.g., (a) the direct synthesis of ethylene from carbon dioxide and (b) the indirect two-step synthesis of ethylene using water electrolysis and modified Fischer-Tropsch processes. They are directly applicable to the development of closed life support systems for manned spacecraft, lunar and Martian bases, and, ultimately, lunar and Martian colonies, e.g., the conversion of the Martian atmosphere to methane, ethylene, ethanol, and a variety of polymers for construction and other uses. This may be followed by-other interesting syntheses, e.g., polyethylene, a plastic with many varied uses, and ethanol, a potential nutrient for astrobiological research, a foodstuff supplement for astronauts, and a precursor to polyesters, another very useful plastic. © 1999 by Sanders D Rosenberg. Published by the American Institute of Aeronautics, Inc.
KW - Aerospace engineering
KW - Carbon dioxide
KW - Carbon dioxide process
KW - Carbon monoxide
KW - Catalytic reforming
KW - Coal liquefaction
KW - Earth (planet)
KW - Ethanol
KW - Ethylene
KW - Fischer-Tropsch synthesis
KW - Hybrid systems
KW - Hydrogen
KW - Interplanetary spacecraft
KW - Life support systems (spacecraft)
KW - Manned space flight
KW - Metabolic engineering
KW - Metabolism
KW - Methane
KW - Moon
KW - Oxygen

KW - Polyethylenes
KW - Space stations
KW - Advanced life supports
KW - Autonomous operations
KW - Chemical engineering process
KW - Engineering principles
KW - Fischer-Tropsch process
KW - Planetary exploration
KW - Reforming of methane
KW - Space propulsion system
KW - Spacecraft propulsion
PB - American Institute of Aeronautics and Astronautics Inc, AIAA
LA - English
J2 - Aerosp. Sci. meet. exhib.
M3 - Conference paper
DB - Scopus
N1 - Export Date: 23 February 2023; Cited By: 0
ER -

TY - JOUR
AU - Conway Morris, S.
TI - Palaeodiversifications: Mass extinctions, "Clocks", and other worlds
PY - 1999
T2 - Geobios
VL - 32
IS - 2
SP - 165
EP - 174
DO - 10.1016/S0016-6995(99)80028-X
UR - https://www.scopus.com/inward/record.uri?eid=2-s2.0-0032781111&doi=10.1016%2fS0016-6995%2899%2980028-X&partnerID=40&md5=50b15bc1383e63d8fab0d20a2daa075e
AD - Department of Earth Sciences, University of Cambridge, Cambridge CB2 3EQ, Downing Street, United Kingdom
AB - The overall pattern of ancient diversifications was apparent more than a century ago, and since then has shown no basic change. This suggests that the fossil record is both robust and reliable. Yet many questions remain to be answered. The recent surge of interest in mass extinctions, for example, has emphasized the causes of trauma and the victims involved. Relatively less attention has been paid to the survivors, where their refugia were located or whether some so-called Lazarus taxa are in fact homeomorphic mimics. Another question concerns times of divergence. Despite the apparent adequacy of the fossil record, it is now claimed that in many cases there are serious discrepancies between the divergence times as seen in the stratigraphic record as against those estimated on the basis of the molecular "clock". For the most part, the latter point to times of divergence substantially in excess of the fossil ranges. No single explanation need apply to explain all these discrepancies, but in at least some cases a non-constant "clock" appears to be a more likely source of the discrepancy than a massive failure of the fossil record. By necessity the history of the Earth and its palaeodiversifications is unique, and it is not known if distant

Earth-like planets are similar or utterly different. Yet there are constraints on the possible range of forms that could evolve, and even if the history of diversification was different the end-results may be remarkably similar.
KW - chronology
KW - exobiology
KW - fossil record
KW - mass extinction
KW - species diversity
KW - Diversity
KW - Extraterrestrial life
KW - Fossil record
KW - Mass extinctions
KW - Molecular "clocks"
PB - Elsevier Masson SAS
SN - 00166995 (ISSN)
LA - English
J2 - Geobios
M3 - Article
DB - Scopus
N1 - Export Date: 23 February 2023; Cited By: 9
ER -

TY - CONF
AU - Hoover, Richard B.
TI - Astrobiological significance of microorganisms in permafrost and ice
PY - 1999
T2 - Proceedings of SPIE - The International Society for Optical Engineering
VL - 3755
SP - 59
EP - 71
UR - https://www.scopus.com/inward/record.uri?eid=2-s2.0-0033317206&partnerID=40&md5=27fb5e0bd19ac98c44396dbb7e555ef8
AD - NASA/Marshall Space Flight Cent, Huntsville, United States
AB - Microorganisms of the permafrost, glaciers, and polar ice sheets of planet Earth provide analogs for microbial life forms that may be encountered in ice or permafrost of Mars, Europa, Callisto, Ganymede, or even asteroids or comets. Most bodies of our Solar System are frozen worlds. The microbiota of the terrestrial cryosphere help establish the thermal and temporal limitations of life on Earth and provide clues to of where and how we should search for evidence of life elsewhere in the Cosmos. Consequently, these life forms are relevant to Astrobiology. Cryopreserved microorganisms can remain viable (in deep anabiosis) in permafrost and ice for millions of years and may contain intact ancient DNA, lipids, enzymes, proteins and genes. Some microorganisms carry out metabolic processes in water films and brine, acidic, or alkaline channels in permafrost or ice at temperatures far below 0C. Complex microbial ecosystems may inhabit snow, ice-bubbles, and cryoconite holes on glaciers and the polar caps. At the NASA Marshall Space Flight Center we employed the Environmental Scanning Electron Microscope to study the morphology, ultra-microstructure and

chemical composition of microorganisms in-situ in ancient permafrost and ice. In this paper we present images of cryopreserved microorganisms from deep ice cores above Lake Vostok and thermokarst ponds of the Fox Tunnel of Alaska.
KW - Ice
KW - Microbiology
KW - Microorganisms
KW - Microstructure
KW - Morphology
KW - Permafrost
KW - Planets
KW - Scanning electron microscopy
KW - Astrobiology
KW - Environmental scanning electron microscopy
KW - Space research
PB - Society of Photo-Optical Instrumentation Engineers
SN - 0277786X (ISSN)
LA - English
J2 - Proc SPIE Int Soc Opt Eng
M3 - Conference paper
DB - Scopus
N1 - Export Date: 23 February 2023; Cited By: 2; CODEN: PSISD
ER -

TY - JOUR
AU - Soffen, G.A.
TI - Astrobiology
PY - 1999
T2 - Advances in Space Research
VL - 23
IS - 2
SP - 283
EP - 288
DO - 10.1016/S0273-1177(99)00048-4
UR - https://www.scopus.com/inward/record.uri?eid=2-s2.0-0032765975&doi=10.1016%2fS0273-1177%2899%2900048-4&partnerID=40&md5=4cdf326e150f766adcc1f3dbd0f6390a
AD - NASA HQ, Washington, DC, United States
AB - If we believe life to be a cosmic imperative, the understanding of life processes becomes a universal aspect of cosmology. How does life fit into our understanding of the universe. As a compliment to NASA's 'Origins' Program we are developing a new venture to embark on one of the great scientific questions of our time, our origins, evolution and our destiny. NASA already deals with a number of related biological questions. We are introducing a new unifying approach to biology within the NASA... Astrobiology. Astrobiology is the study of the chemistry, physics and adaptations that influence the origin, evolution and destiny of life. We intent to raise the conscious level relating relevant biological questions to the formation and development of the universe through space missions and research programs. By linking certain aspects of exobiology, ecology, gravitational biology, and adding efforts in molecular biology, evolutionary biology, and planetary biology and joining this to Astronomy and planetology, we seek a deeper

understanding of where the living process fits in to our cosmological theories. We do this through laboratory experiments, space observations, computer modeling, missions and discovery of what appear to be extreme conditions for us, but conditions in which life thrives. NASA has formed an international 'virtual' Astrobiology Institute as a nucleus to initiate this consolidating idea. NASA's technology will play a major role in this endeavor.
KW - exobiology
KW - methodology
PB - Elsevier Ltd
SN - 02731177 (ISSN)
LA - English
J2 - Adv. Space Res.
M3 - Article
DB - Scopus
N1 - Export Date: 23 February 2023; Cited By: 8; CODEN: ASRSD
ER -

TY - JOUR
AU - Kawasaki, Y.
TI - Direct detection of Martian microorganisms based on fluorescence microscopy
PY - 1999
T2 - Advances in Space Research
VL - 23
IS - 2
SP - 309
EP - 317
DO - 10.1016/S0273-1177(99)00052-6
UR - https://www.scopus.com/inward/record.uri?eid=2-s2.0-0032812301&doi=10.1016%2fS0273-1177%2899%2900052-6&partnerID=40&md5=28734a2a7808cdde7f561e2b50bad870
AD - Mitsubishi Kasei Inst. of Life Sci., Machida, Tokyo 194-8511, 11 Minamiooya, Japan
AB - The direct detection of microorganisms and their traces using optical microscopes is one of the most promising techniques to obtain the decisive evidences for extraterrestrial life. The most significant points of this technique are high sensitivity and spatial information with a resolution of 0.2mm. Besides, information on local environments and microscopic ecology can also be obtained. Many difficulties, however, must be solved to get reliable results. We have started to develop a noble technique based on the fluorescence microscopy with special interest to the detection of microorganisms in extreme environments including Mars. The principle is to detect molecules/subcellular organs which are responsible for the three basic characteristics of life; genetic information, metabolism, and discrimination of self from non-self. We have screened fluorescence probes and found several are applicable. We could detect almost all the microorganisms already identified. Discrimination of viable from dead cells was possible. The terrestrial microfossils, some of the artificial primitive microorganism-like-objects, dried bacteria and polycyclic aromatic hydrocarbons mixed with simulated Martian sand could be detected. We are now designing a compact detection hardware.

KW - exobiology
KW - extraterrestrial matter
KW - Mars
PB - Elsevier Ltd
SN - 02731177 (ISSN)
LA - English
J2 - Adv. Space Res.
M3 - Article
DB - Scopus
N1 - Export Date: 23 February 2023; Cited By: 10; CODEN: ASRSD
ER -

TY - CONF
AU - Rosenberg, S.D.
AU - Makel, D.B.
AU - Carranza, S.
AU - Finn, J.E.
TI - The manufacture of useful products in manned: Spacecraft and planetary bases
PY - 1999
T2 - 35th Joint Propulsion Conference and Exhibit
C7 - AIAA-99-2411
UR - https://www.scopus.com/inward/record.uri?eid=2-s2.0-84963865230&partnerID=40&md5=3ce0a4adf80c77ba05410841041dd68b
AD - In-Space Propulsion, Ltd, Sacramento, CA, United States
AD - Makel Engineering Inc, Chico, CA, United States
AD - Chemical Engineer, Makel Engineering Inc, Chico, CA, United States
AD - NASA Ames Research Center, Moffet Field, CA, United States
AB - We present results of a program which was performed to demonstrate the feasibility of one or more processes for the direct and/or indirect conversion of carbon dioxide and water to ethylene. Our program addressed the requirements topic “Spacecraft Life Support Infrastructure.” It focused on catalytic reduction of carbon dioxide with hydrogen using modified Fischer-Tropsch processes to demonstrate ethylene synthesis. Benefits of this approach to the development of modified life support systems are (1) major weight savings resulting from reduced on-board requirements for spacecraft and transport requirements for planetary bases, e.g., Earth to Mars, and (2) conversion of two metabolic wastes, carbon dioxide and water, to ethylene, a key building block for a variety of useful materials, including ethanol, a high caloric nutrient source for organisms for the support of Astrobiology research and foodstuff synthesis based on engineering principles rather than life processes. The specific purpose of our research was to investigate the synthesis of ethylene by two chemical processes, one direct, one indirect. The direct process is based on the catalytic reduction of carbon dioxide with hydrogen at ca. 300°C (572°F) to form ethylene and other C, compounds. The indirect process is based on the catalytic reformation of methane in the presence of oxygen at ca. 800°C (1472°F) to form ethylene and other C, compounds. The preparation of ethylene opens the way to the preparation of other useful products on manned spacecraft and planetary bases, e.g., ethanol for use as a nutrient to support Astrobiology research

programs, polyethylene and polyester polymer formulations to support the manufacture of spare parts and planetary habitats, and ethylene for use as a fuel in propulsion systems, e.g., the Mars Ascent Vehicle. The primary approach, the direct process, to the synthesis of ethylene and other C, compounds was demonstrated using two different hydrogenation catalysts: iron dodecacarbonyl, Fe3(CO)12, and iron-zinc, Fe-Zn. The secondary approach, the indirect process, was demonstrated using two different reforming catalysts: manganese-promoted sodium tungstate on a magnesia support, Mn(2wt%)/Na2WO4(5wt%)/MgO, and pure silver, Ag. The presence of ethylene and other C, compounds in the reaction product gas streams was confirmed by detailed vapor phase chromatographic analysis in all of the direct reduction and reforming experiments. Some of the experiments conducted are described in detail. © 1999 by the American Institute of Aeronautics and Astronautics, Inc. © 1999 by the American Institute of Aeronautics and Astronautics. All rights reserved.
KW - Automobile manufacture
KW - Carbon dioxide
KW - Carbon monoxide
KW - Catalysts
KW - Catalytic reforming
KW - Chromatographic analysis
KW - Coal liquefaction
KW - Ethanol
KW - Ethylene
KW - Fischer-Tropsch synthesis
KW - Gas chromatography
KW - Hydrogenation
KW - Iron compounds
KW - Life support systems (spacecraft)
KW - Manganese
KW - Manned space flight
KW - Manufacture
KW - Nutrients
KW - Propulsion
KW - Silver
KW - Space flight
KW - Spacecraft
KW - Spacecraft propulsion
KW - Zinc
KW - American Institute of Aeronautics and Astronautics
KW - Catalytic reduction
KW - Engineering principles
KW - Fischer-Tropsch process
KW - Hydrogenation catalyst
KW - Mars Ascent vehicle
KW - Planetary habitats
KW - Polymer formulation
KW - Carbon dioxide process
PB - American Institute of Aeronautics and Astronautics Inc, AIAA
LA - English
J2 - Jt. Propuls. Conf. exhib.
M3 - Conference paper
DB - Scopus

N1 - Export Date: 23 February 2023; Cited By: 3
ER -

TY - CONF
AU - Flynn, M.
AU - Hines, J.
AU - McKay, C.
TI - Astrobiology hydrothermal vent technology development
PY - 2000
T2 - SAE Technical Papers
DO - 10.4271/2000-01-2342
UR - https://www.scopus.com/inward/record.uri?eid=2-s2.0-85072419320&doi=10.4271%2f2000-01-2342&partnerID=40&md5=0c3bdb5d0389d992ad24cddfe169f5a3
AD - Mail Stop 239-15, NASA, Ames Research Center, Moffett Field, CA 94035-1000, United States
AB - The study of life in extreme environments provides an important basis from which we can undertake the search for extraterrestrial life. This paper provides a description of a program focused on developing technologies which are necessary to evaluate the potential for the existence of a deep sub-seafloor biosphere.
KW - Automotive engineering
KW - Extraterrestrial life
KW - Extreme environment
KW - Hydrothermal vent
KW - Sub-seafloor biosphere
KW - Technology development
KW - Hot springs
PB - SAE International
SN - 01487191 (ISSN)
LA - English
J2 - SAE Techni. Paper.
M3 - Conference paper
DB - Scopus
N1 - Export Date: 23 February 2023; Cited By: 0; Correspondence Address: M. Flynn; Mail Stop 239-15, NASA, Ames Research Center, Moffett Field, CA 94035-1000, United States; email: mflynn@mail.arc.nasa.gov
ER -

TY - JOUR
AU - Naganuma, T.
TI - Microbes on the edge of global biosphere
PY - 2000
T2 - Biological sciences in space = Uchū seibutsu kagaku
VL - 14
IS - 4
SP - 323
EP - 331
DO - 10.2187/bss.14.323
UR - https://www.scopus.com/inward/record.uri?eid=2-s2.0-0034578054&doi=10.2187%2fbss.14.323&partnerID=40&md5=43517fc9ef41a0241aaee01da7258ca7

AD - Faculty of Applied Biological Science, Hiroshima University., Japan
AB - The search for life on the edge of global biosphere is a frontier to bridge conventional bio/ecology and exo/astrobiology. This communication reviews the foci of microbiological studies on the inhabitants of the selected "edges", i.e., deep-sea, deep subsurface and Antarctic habitats. The deep-sea is characterized as the no-light (non-photosynthetic) habitat, and the primary production is mostly due to the chemosynthetic autotrophy at the hydrothermal vents and methane-rich seeps. Formation of the chemosynthesis-dependent animal communities in the deep leads to the idea that such communities may be found in "ocean" of the Jovian satellite, Europa. The oxygen minimal layer (OML) in mid-water provides another field of deep-sea research. Modern OML is a relatively thin layer, found between the water depth of 200 and 1000 m, but was much thicker during the periods of oceanic anoxia events (OAEs) in the past. The history of oceanic biosphere is regarded as the cycle of OAE and non-OAE periods, and the remnants of the past OAEs may be seen in the modem OML. Anoxic (no-O2) condition is also characteristic of deep subsurface biosphere. Microorganisms in deep subsurface biosphere exploit every available oxidant, or terminal electron acceptor (TEA), for anaerobic respiration. Sulfate, nitrate, iron (III) and CO2 are the representative TEAs in the deep subsurface. Subsurface of hydrothermal vents, or sub-vent biosphere, may house brine (high salt) habitats and halophilic microorganisms. Some sub-vent halophiles were phylogenetically closely similar to the ones found in the Antarctic habitats which are extremely dry by the liophilizing climate. Below the 3000-4000 m-thick glacier on Antarctica, there have been >70 lakes with liquid water located. One of such sub-glacial lakes, Lake Vostok, has been a target of "life in extreme environments" and is about to be drill-penetrated for microbiological studies. These 'microbiological platforms' will provide new knowledge about the diversity and potential of the Earth's life and facilitate the capability of astrobiologial exploration.
KW - Antarctic Regions
KW - Bacteria
KW - Cold Climate
KW - Ecosystem
KW - Environmental Microbiology
KW - Evolution
KW - Exobiology
KW - Fresh Water
KW - Japan
KW - Jupiter
KW - Oxygen
KW - Seawater
KW - fresh water
KW - oxygen
KW - sea water
KW - MLCS
KW - MLOWN
KW - Antarctica
KW - article

KW - astronomy
KW - bacterium
KW - biology
KW - chemistry
KW - cold climate
KW - ecosystem
KW - evolution
KW - Japan
KW - metabolism
KW - microbiology
SN - 09149201 (ISSN)
C2 - 11589224
LA - Japanese
J2 - Biol. Sci. Space
M3 - Article
DB - Scopus
N1 - Export Date: 23 February 2023; Cited By: 1; Correspondence Address: T. Naganuma; email: takn@hiroshima-u.ac.jp
ER -

TY - JOUR
AU - Irion, R.
TI - The science of astrobiology takes shape
PY - 2000
T2 - Science
VL - 288
IS - 5466
SP - 603+605
DO - 10.1126/science.288.5466.603
UR - https://www.scopus.com/inward/record.uri?eid=2-s2.0-0034725066&doi=10.1126%2fscience.288.5466.603&partnerID=40&md5=adfb3bd8135330c619a87456e6fdc85b
AB - About 600 researchers from 30 countries came to NASA's Ames Research Center on 3 to 5 April, eager to help mold a new field. At the First Astrobiology Science Conference every talk, it seemed, touched on a new discipline. Topics ranged from prebiotic chemicals to icy life, but the conference was a story unto itself.
KW - Amino Acids
KW - Cosmic Dust
KW - Exobiology
KW - Extraterrestrial Environment
KW - Heat
KW - Ice
KW - Jupiter
KW - Meteoroids
KW - Pressure
KW - United States
KW - United States National Aeronautics and Space Administration
KW - Water Microbiology
KW - amino acid
KW - amino acid
KW - ice
KW - amino acid analysis
KW - Arctic

KW - astronomy
KW - geology
KW - organization
KW - priority journal
KW - research
KW - science
KW - short survey
KW - United States
KW - astronomy
KW - biology
KW - chemistry
KW - conference paper
KW - government
KW - heat
KW - microbiology
KW - microclimate
KW - pressure
PB - American Association for the Advancement of Science
SN - 00368075 (ISSN)
C2 - 10798994
LA - English
J2 - Science
M3 - Short survey
DB - Scopus
N1 - Export Date: 23 February 2023; Cited By: 5; CODEN: SCIEA
ER -

TY - JOUR
AU - Fogg, M.J.
TI - The ethical dimensions of space settlement
PY - 2000
T2 - Space Policy
VL - 16
IS - 3
SP - 205
EP - 211
DO - 10.1016/S0265-9646(00)00024-2
UR - https://www.scopus.com/inward/record.uri?eid=2-s2.0-0034674795&doi=10.1016%2fS0265-9646%2800%2900024-2&partnerID=40&md5=a4be7175c2c2c43e6b33d86e46a84e7c
AD - Probability Research Group, London SE19 1UY, c/o 44 Hogarth Court, Fountain Drive, United Kingdom
AB - While proposals for settling in the space frontier have appeared in the technical literature for over 20 years, it is in the case of Mars that the ethical dimensions of space settlement have been most studied. Mars raises the questions of the rights and wrongs of the enterprise more forcefully because: (a) Mars may possess a primitive biota; and (b) it may be possible to terraform Mars and transform the entire planet into a living world. The moral questions implicit in space settlement are examined below from the standpoints of four theories of environmental ethics: anthropocentrism, zoocentrism, ecocentrism and preservationism. In the absence of extraterrestrial life, only preservationism concludes that space settlement would be immoral if it was seen to be to the

benefit of terrestrial life. Even if Mars is not sterile, protection for Martian life can be argued for either on intrinsic or instrumental grounds from the standpoints of all of these theories. It is argued further that a strict preservationist ethic is untenable as it assumes that human consciousness, creativity, culture and technology stand outside nature, rather than having been a product of natural selection. If Homo sapiens is the first spacefaring species to have evolved on Earth, space settlement would not involve acting 'outside nature', but legitimately 'within our nature'. © 1999 International Astronautical Federation or the International Academy of Astronautics. Published by Elsevier Science Ltd. All rights reserved.
PB - Elsevier BV
SN - 02659646 (ISSN)
LA - English
J2 - Space Policy
M3 - Article
DB - Scopus
N1 - Export Date: 23 February 2023; Cited By: 28; CODEN: SPCPE
ER -

TY - JOUR
AU - Sterns, P.M.
TI - SETI and space law: jurisprudential and philosophical considerations for humankind in relation to extraterrestrial life
PY - 2000
T2 - Acta Astronautica
VL - 46
IS - 10
SP - 759
EP - 763
DO - 10.1016/S0094-5765(00)00044-8
UR - https://www.scopus.com/inward/record.uri?eid=2-s2.0-0342845315&doi=10.1016%2fS0094-5765%2800%2900044-8&partnerID=40&md5=4054e82660b8132343250b1682deb479
AD - Law Offices of Sterns and Tennen, Attorneys and Counselors at Law, Phoenix, AZ, United States
AB - The impact of confirmation of life outside the small ecosphere we call Earth will be profound on the terran population as a whole. The 'Declaration Of Principles Concerning Activities Following The Detection of Extraterrestrial Intelligence' and the IAA Position Paper 'A Decision Process for Examining the Possibility of Sending Communications To Extraterrestrial Civilizations: A Proposal' provide a firm basis for the development of a new body of space law. It is important that space law design and prepare for implementation of a protocol to guide the nations of the world concerning the search for extraterrestrial intelligence (SETI), through the advice and cooperation of scientists, jurisprudential, philosophical, political and sociological scholars. Through the IAA, the IISL, the United Nations and other organizations, formal documentation should be drafted to encode the Declaration of Principles and IAA Position Paper referred to above. In this way, a body of metalaw can be developed to enable human communication with non-terrestrial life. This paper discusses the philosophical and sociological parameters

of terran understanding of our place in the universe which will dramatically impact jurisprudential thought and action in light of the realization of the infinitesimally small niche that humankind occupies. A discussion of these interdisciplinary concerns will be necessary to realize a metalegal approach to interstellar communications and relations.
KW - Economic and social effects
KW - Extraterrestrial atmospheres
KW - Extraterrestrial communication links
KW - Laws and legislation
KW - Philosophical aspects
KW - Social aspects
KW - Societies and institutions
KW - Search for extraterrestrial intelligence
KW - Space law
KW - Space research
PB - Elsevier Science Ltd
SN - 00945765 (ISSN)
LA - English
J2 - Acta Astronaut
M3 - Article
DB - Scopus
N1 - Export Date: 23 February 2023; Cited By: 10; CODEN: AASTC
ER -

TY - CONF
PY - 2000
T2 - Proceedings of SPIE - The International Society for Optical Engineering
VL - 4137
SP - Varpagings
UR - https://www.scopus.com/inward/record.uri?eid=2-s2.0-0033633568&partnerID=40&md5=785fc62d9dd5669e1fa768fc154b33aa
AB - The proceedings contains 11 papers from SPIE-The International Society for Optical Engineering on instruments, methods, and missions for astrobiology III. The topics discussed include: oil and natural gas on Mars; biomineral homologies, organismobiosis, and the problem of biomarkers; detection of extrasolar planets via a network of robotic imaging telescopes; formation of ordered structures of charged microparticles in near-surface cometry gas-dusty atmosphere; potential for prebiotic chemistry at the poles of the Moon; and extremophilic microorganisms as candidates for extraterrestrial life.
KW - Biochemistry
KW - Biomarkers
KW - Earth atmosphere
KW - Extraterrestrial atmospheres
KW - Lunar surface analysis
KW - Meteorites
KW - Microorganisms
KW - Natural gas deposits
KW - Optical telescopes
KW - Petroleum deposits
KW - Planets

KW - Astrobiology
KW - Astromaterials
KW - Biomineral homologies
KW - Cometry atmosphere
KW - EiRev
KW - Extrasolar planets
KW - Halobacteriales
KW - Planetary surface analysis
PB - Society of Photo-Optical Instrumentation Engineers
SN - 0277786X (ISSN)
LA - English
J2 - Proc SPIE Int Soc Opt Eng
M3 - Conference review
DB - Scopus
N1 - Export Date: 23 February 2023; Cited By: 0; CODEN: PSISD
ER -

TY - CONF
AU - Creech, N.
AU - Dick, G.
AU - Gantner, B.
AU - Hoffower, H.
AU - Johnson, S.
AU - Lacey, M.
AU - Lamb, D.
AU - Obland, M.
AU - Papineau, D.
AU - Rick, C.
AU - Smith, B.
AU - Vanyo, C.
AU - Weber, B.
TI - Scouting for lunar resources: Advantages and implications of a lander-rover combo
PY - 2000
T2 - Space 2000
SP - 687
EP - 694
DO - 10.1061/40479(204)83
UR - https://www.scopus.com/inward/record.uri?eid=2-s2.0-0034587154&doi=10.1061%2f40479%28204%2983&partnerID=40&md5=a67a273153b13db8d6a6a65b4edb29a1
AB - With the recent success of Lunar Prospector and the possibility of both water-ice and 3He at the lunar poles, NASA should follow up with further missions and data gathering. Clearly, there exists the need to directly confirm the existence of these important lunar resources. The 1999 Ames Astrobiology Academy feels this can be accomplished most effectively by sending a lander-rover combination to locally analyze the surface material. Our proposal will outline a "Discovery" type mission including information regarding appropriate landing sites, rocketry, instrumentation, navigation, power systems, and communications. In the "faster, better, cheaper" paradigm, we will mainly emphasize existing technology, but we will also suggest reasonable improvements to landers and rovers to achieve these goals.

KW - Helium
KW - Ice
KW - Lunar landing
KW - Water
KW - Lunar resources
KW - Lunar missions
A2 - Johnson S.W.
A2 - Chua K.M.
A2 - Galloway R.G.
A2 - Richter P.J.
PB - ASCE - American Society of Civil Engineers
SN - 0784404798 (ISBN); 978-078440479-9 (ISBN)
LA - English
J2 - Space 2002
M3 - Conference paper
DB - Scopus
N1 - Export Date: 23 February 2023; Cited By: 0
ER -

TY - JOUR
AU - Trent, J.D.
TI - Extremophiles in astrobiology: per Ardua ad Astra.
PY - 2000
T2 - Gravitational and space biology bulletin : publication of the American Society for Gravitational and Space Biology
VL - 13
IS - 2
SP - 5
EP - 11
UR - https://www.scopus.com/inward/record.uri?eid=2-s2.0-0034200806&partnerID=40&md5=4e96dc2eb574136d907b0b64db20e0cd
AD - NASA Ames Research Center, Moffett Field, Astrobiology Technology Branch, CA, United States
AB - As we consider the possibilities of finding life on other planets, it behooves us to evaluate what we know about the limits for life on planet Earth. In our continued exploration of Earth, we are finding microbes in a variety of unexpected habitats. In geothermal hot springs, we have discovered organisms thriving at temperatures near the boiling point of water and at pH values down to 0.5; in the deepest parts of the oceans, those that grow optimally at pressures above 1000 bars and die at pressures below 500 bars; and at the poles, those that grow below the freezing point of water and die at temperatures above 10 degrees C. All of these organisms are living proof that the biochemical "machinery" of life can be adapted to conditions that, from our anthropocentric perspective, appear to be extreme. By studying the molecular adaptations of extremophiles, we begin to identify the critical cellular components that expand the envelope for life. As an example, I will discuss what we have learned about the role of the proteins we call "heat shock proteins" in pushing the upper temperature limit of life and how our studies have provided a new perspective on the function of these proteins.
KW - Adaptation, Physiological
KW - Cell Membrane Permeability

KW - Chaperonin 60
KW - Environmental Microbiology
KW - Exobiology
KW - Heat
KW - Heat-Shock Proteins
KW - Sulfolobus
KW - chaperonin
KW - heat shock protein
KW - adaptation
KW - article
KW - biology
KW - cell membrane permeability
KW - heat
KW - microbiology
KW - physiology
KW - Sulfolobus
SN - 1089988X (ISSN)
C2 - 11543281
LA - English
J2 - Gravit Space Biol Bull
M3 - Article
DB - Scopus
N1 - Export Date: 23 February 2023; Cited By: 12
ER -

TY - CONF
AU - van der Meer, Wieb
AU - Alletto, James
AU - Bryant, Dudley
AU - Carini, Mike
AU - Elliott, Larry
AU - Gelderman, Richard
AU - Mason, Wayne
AU - McDaniel, Kerrie
AU - McGruder III, Charles
AU - Rinehart, Claire
AU - Tyler, Rico
AU - Walker, Linda
TI - Astrobiology as a tool for getting high school students interested in science
PY - 2000
T2 - Proceedings of SPIE - The International Society for Optical Engineering
VL - 4137
SP - 106
EP - 112
DO - 10.1117/12.411615
UR - https://www.scopus.com/inward/record.uri?eid=2-s2.0-17744367608&doi=10.1117%2f12.411615&partnerID=40&md5=46a89dc73938359c21f8640cdaf936f9
AD - Western Kentucky Univ, Bowling Green, United States
AB - A workshop was held (10/99) for high school students and teachers on astrobiology. NASA provided support through an IDEAS grant. Out of 63 qualified applicants, 29 were accepted: 22 students

(11 minorities) and 7 teachers. The workshop was held on 2 successive weekends. Activities included: culturing microbes from human skin, discussing `what is life?', building and using a 2-inch refractive telescope and a van-Leeuwenhoek-type microscope (each participant built and kept them), listening to lectures by Dr. Richard Gelderman on detecting extra solar planets and by Dr. Richard Hoover on life in extreme environments. Other activities included: collecting samples and isolating microorganisms from the lost river cave, studying microbial life from extreme environments in the laboratory, using the internet as a research tool and debating the logistics and feasibility of a lunar colony. Written evaluations of the workshop led to the following conclusions: 48% of the students considered a possible career in the biological and/or astrophysical sciences, and half of these stated they were spurred on by the workshop itself.
KW - Microbiology
KW - Microorganisms
KW - Microscopic examination
KW - Optical telescopes
KW - Planets
KW - Technical presentations
KW - Culturing microbes
KW - Van-Leeuwenhoek-type microscope
KW - Astrophysics
PB - Society of Photo-Optical Instrumentation Engineers
SN - 0277786X (ISSN)
LA - English
J2 - Proc SPIE Int Soc Opt Eng
M3 - Conference paper
DB - Scopus
N1 - Export Date: 23 February 2023; Cited By: 1; CODEN: PSISD
ER -

TY - CONF
AU - Seckbach, Joseph
AU - Oren, Aharon
TI - Extremophilic microorganisms as candidates for extraterrestrial life
PY - 2000
T2 - Proceedings of SPIE - The International Society for Optical Engineering
VL - 4137
SP - 89
EP - 96
DO - 10.1117/12.411613
UR - https://www.scopus.com/inward/record.uri?eid=2-s2.0-0034467982&doi=10.1117%2f12.411613&partnerID=40&md5=20c3dd510ef8e9d5e7610325d0841919
AB - Microbial life is found all over the globe. Diverse communities are even found in such places in which extreme conditions with respect of temperature, salinity, pH, and pressure prevail. Many of these environments were until recently considered too harsh to harbor microbial life. The microorganisms adapted to an existence at the edge of life are termed extremophiles. They include

members of the Prokaryotes (domains Archaea and Bacteria) and the Eukarya, including algae and protozoa. Extremophilic microbes thrive at low and high temperatures - from subzero levels to above the boiling point of water, at both sides of the pH scale - in acidic as well as in alkaline media, in hypersaline environments with salt concentrations of up to saturation, at high pressure, both in the deep sea and in the terrestrial deep subsurface where they are exposed to pressures of hundreds of atmospheres, and in other extreme conditions. In many cases they tolerate combinations of more than one environmental stress factor. Some of these extremophiles may be considered as `living fossils' since their environment resembles the conditions that may have existed during the time life arose on Earth, more than 3.5 billion years ago. In view of these properties the extremophilic microorganisms may be considered as model organisms when exploring the possibilities of the existence of extraterrestrial life. For example, the microbes discovered in ice cores recovered from the depth of the Lake Vostok in Antarctica may serve as a model simulating conditions prevailing in the permafrost subsurface area of Mars or Jupiter's moon Europa. Microbial life in the Dead Sea or in Great Salt Lake may resemble halophilic life forms that may exist elsewhere in the universe, adapted to life at low water activities. Likewise, hyperthermophilic microorganisms present on Earth in hot springs, hydrothermal vents and other sites heated by volcanic activity in terrestrial or marine areas, may resemble life forms that may exist on hot planets such as Venus.
KW - Algae
KW - Alkalinity
KW - Biodiversity
KW - Microorganisms
KW - pH effects
KW - Thermal effects
KW - Extraterrestrial life
KW - Hyperthermophilic microorganisms
KW - Extraterrestrial atmospheres
PB - Society of Photo-Optical Instrumentation Engineers
SN - 0277786X (ISSN)
LA - English
J2 - Proc SPIE Int Soc Opt Eng
M3 - Conference paper
DB - Scopus
N1 - Export Date: 23 February 2023; Cited By: 3; CODEN: PSISD
ER -

TY - JOUR
AU - Benner, S.A.
AU - Devine, K.G.
AU - Matveeva, L.N.
AU - Powell, D.H.
TI - The missing organic molecules on Mars
PY - 2000
T2 - Proceedings of the National Academy of Sciences of the United States of America
VL - 97
IS - 6

SP - 2425
EP - 2430
DO - 10.1073/pnas.040539497
UR - https://www.scopus.com/inward/record.uri?eid=2-s2.0-0034646253&doi=10.1073%2fpnas.040539497&partnerID=40&md5=1b77cff06ce55194f1f4b4142c1a2f56
AD - Depts. Chem., Anat., and Cell Biol., University of Florida, Gainesville, FL 32611, United States
AB - GC-MS on the Viking 1976 Mars missions did not detect organic molecules on the Martian surface, even those expected from meteorite bombardment. This result suggested that the Martian regolith might hold a potent oxidant that converts all organic molecules to carbon dioxide rapidly relative to the rate at which they arrive. This conclusion is influencing the design of Mars missions. We reexamine this conclusion in light of what is known about the oxidation of organic compounds generally and the nature of organics likely to come to Mars via meteorite. We conclude that nonvolatile salts of benzenecarboxylic acids, and perhaps oxalic and acetic acid, should be metastable intermediates of meteoritic organics under oxidizing conditions. Salts of these organic acids would have been largely invisible to GC-MS. Experiments show that one of these, benzenehexacarboxylic acid (mellitic acid), is generated by oxidation of organic matter known to come to Mars, is rather stable to further oxidation, and would not have been easily detected by the Viking experiments. Approximately 2 kg of meteorite-derived mellitic acid may have been generated per m2 of Martian surface over 3 billion years. How much remains depends on decomposition rates under Martian conditions. As available data do not require that the surface of Mars be very strongly oxidizing, some organic molecules might be found near the surface of Mars, perhaps in amounts sufficient to be a resource. Missions should seek these and recognize that these complicate the search for organics from entirely hypothetical Martian life.
KW - Flight Experiment
KW - long duration
KW - unmanned
KW - Viking Project
KW - Alkanes
KW - Amino Acids
KW - Benzene
KW - Biogenesis
KW - Carboxylic Acids
KW - Chemistry, Organic
KW - Mars
KW - Meteoroids
KW - Models, Chemical
KW - Naphthalenes
KW - Oxidation-Reduction
KW - Water
KW - NASA Discipline Exobiology
KW - Non-NASA Center
KW - acetic acid
KW - benzene derivative
KW - carbon dioxide

KW - carboxylic acid derivative
KW - organic compound
KW - oxalic acid
KW - article
KW - astronomy
KW - gas chromatography
KW - mass spectrometry
KW - molecular dynamics
KW - oxidation
KW - priority journal
KW - surface property
KW - Astrobiology
KW - Exobiology
KW - Organic compounds
KW - Viking
SN - 00278424 (ISSN)
C2 - 10706606
LA - English
J2 - Proc. Natl. Acad. Sci. U. S. A.
M3 - Article
DB - Scopus
N1 - Export Date: 23 February 2023; Cited By: 329; Correspondence Address: S.A. Benner; Department of Chemistry, University of Florida, Gainesville, FL 32611, United States; email: benner@chem.ufl.edu; CODEN: PNASA
ER -

TY - JOUR
AU - Horneck, G.
TI - The microbial world and the case for Mars
PY - 2000
T2 - Planetary and Space Science
VL - 48
IS - 11
SP - 1053
EP - 1063
DO - 10.1016/s0032-0633(00)00079-9
UR - https://www.scopus.com/inward/record.uri?eid=2-s2.0-0345792455&doi=10.1016%2fs0032-0633%2800%2900079-9&partnerID=40&md5=0b8cc4eaa36f05571f567027d50c7a48
AD - Deutsches Zentrum Luft- Raumfahrt, Inst. fur Luft- und Raumfahrtmedizin, Strahlenbiologie, D51170 Köln, Germany
AB - Microbial prokaryotes have flourished on Earth for more than 3.5 Ga. They dominated the Earth's biosphere during the first 2 Ga of its history before the first unicellular mitotic eukaryotes appeared. Therefore, in a search for extant life beyond the Earth, microorganisms are the most likely candidates for a putative biota of an extraterrestrial habitat. On Earth, life has developed strategies to cope with the so-called extreme conditions, such as hot vents, permafrost, permanent ice, subsurface regions, high atmosphere, rocks or salt crystals. By analogy with terrestrial extremophile communities, potential protected niches have been postulated for Mars, such as sulfur-rich sub-surface areas for chemoautotrophic communities, rocks for endolithic communities,

permafrost regions, hydrothermal vents, soil, or evaporite crystals. Several methods exist to trace and identify microbial communities on Earth. Some of these biogenic signatures and biomarkers may also be applicable in attempts to search for life on Mars. However, the search for signatures indicative for putative extant life on Mars can only be the final steps in a research strategy in the quest for extraterrestrial life. In particular, prior to any "search for extant life" experiment, more data are required on the geology (paleolakes, volcanism, hydrothermal vents, carbonates), climate (hydrosphere, duration of phases which allow liquid water) and radiation environment, present state and past evolution as well as organic molecules in sediments. The search for possible biological oases will be connected with the detection of areas where liquid water still exists under the current conditions on that planet. © 2000 Elsevier Science Ltd. All rights reserved.
KW - Biomarker
KW - Extremophiles
KW - Mars
KW - Microbial communities
PB - Elsevier Ltd
SN - 00320633 (ISSN)
LA - English
J2 - Planet. Space Sci.
M3 - Article
DB - Scopus
N1 - Export Date: 23 February 2023; Cited By: 86; Correspondence Address: G. Horneck; Deutsches Zentrum Luft- Raumfahrt, Inst. fur Luft- und Raumfahrtmedizin, Strahlenbiologie, D51170 Köln, Germany; email: gerda.horneck@dlr.de; CODEN: PLSSA
ER -

TY - JOUR
AU - Kato, C.
AU - Takai, K.
TI - Microbial diversity of deep-sea extremophiles--Piezophiles, Hyperthermophiles, and subsurface microorganisms
PY - 2000
T2 - Biological sciences in space = Uchū seibutsu kagaku
VL - 14
IS - 4
SP - 341
EP - 352
DO - 10.2187/bss.14.341
UR - https://www.scopus.com/inward/record.uri?eid=2-s2.0-0034578412&doi=10.2187%2fbss.14.341&partnerID=40&md5=0a98a3460c6ed2734968735617ed890b
AD - The DEEPSTAR Group, Japan Marine Science and Technology Center., Japan
AB - Knowledge of our Planet's biosphere has increased tremendously during the last 10 to 20 years. In the field of Microbiology in particular, scientists have discovered novel "extremophiles", microorganisms capable of living in extreme environments such as highly acidic or alkaline conditions, at high salt concentration, with no oxygen, extreme temperatures (as low as -20 degrees C and as

high as 300 degrees C), at high concentrations of heavy metals and in high pressure environments such as the deep-sea. It is apparent that microorganisms can exist in any extreme environment of the Earth, yet already scientists have started to look for life on other planets; the so-called "Exobiology" project. But as yet we have little knowledge of the deep-sea and subsurface biosphere of our own planet. We believe that we should elucidate the Biodiversity of Earth more thoroughly before exploring life on other planets, and these attempts would provide deeper insight into clarifying the existence of extraterrestrial life. We focused on two deep-sea extremophiles in this article; one is "Piezophiles", and another is "Hyperthermophiles". Piezophiles are typical microorganisms adapted to high-pressure and cold temperature environments, and located in deep-sea bottom. Otherwise, hyperthermophiles are living in high temperature environment, and located at around the hydrothermal vent systems in deep-sea. They are not typical deep-sea microorganisms, but they can grow well at high-pressure condition, just like piezophiles. Deming and Baross mentioned that most of the hyperthermophilic archaea isolated from deep-sea hydrothermal vents are able to grow under conditions of high temperature and pressure, and in most cases their optimal pressure for growth was greater than the environmental pressure they were isolated from. It is possible that originally their native environment may have been deeper than the sea floor and that there had to be a deeper biosphere. This implication suggests that the deep-sea hydrothermal vents are the windows to a deep subsurface biosphere. A vast array of chemoautotrophic deep-sea animal communities have been found to exist in cold seep environments, and most of these animals are common with those found in hydrothermal vent environments. Thus, it is possible to consider that the cold seeps are also one of slit windows to a deep subsurface biosphere. We conclude that the deep-sea extremophiles are very closely related into the unseen majority in subsurface biosphere, and the subsurface biosphere probably concerns to consider the "exobiology".

KW - Archaea
KW - Atmospheric Pressure
KW - Bacteria
KW - Cold
KW - Ecosystem
KW - Exobiology
KW - Heat
KW - Seawater
KW - Water Microbiology
KW - sea water
KW - Archaebacterium
KW - article
KW - atmospheric pressure
KW - bacterium
KW - biology
KW - cold
KW - ecosystem
KW - heat
KW - microbiology
SN - 09149201 (ISSN)

C2 - 11589226
LA - Japanese
J2 - Biol. Sci. Space
M3 - Article
DB - Scopus
N1 - Export Date: 23 February 2023; Cited By: 5; Correspondence Address: C. Kato; email: katoc@jamstec.go.jp
ER -

TY - JOUR
AU - Hogan, C.J.
TI - Why the universe is just so
PY - 2000
T2 - Reviews of Modern Physics
VL - 72
IS - 4
SP - 1149
EP - 1161
DO - 10.1103/revmodphys.72.1149
UR - https://www.scopus.com/inward/record.uri?eid=2-s2.0-0034336329&doi=10.1103%2frevmodphys.72.1149&partnerID=40&md5=ac8937344c70d5d1f525afdb8eadabbc
AD - Astronomy and Physics Departments, University of Washington, Seattle, WA 98195, United States
AB - Some properties of the world are fixed by physics derived from mathematical symmetries, while others are selected from an ensemble of possibilities. Several successes and failures of "anthropic" reasoning in this context are reviewed in light of recent developments in astrobiology, cosmology, and unification physics. Specific issues raised include our space-time location (including the reason for the present age of the universe), the time scale of biological evolution, the tuning of global cosmological parameters, and the origin of the Large Numbers of astrophysics and the parameters of the standard model. Out of the 20 parameters of the standard model, the basic behavior and structures of the world (nucleons, nuclei, atoms, molecules, planets, stars, galaxies) depend mainly on five of them: me, mu , md, α, and αG (where mproton and αQCD are taken as defined quantities). Three of these appear to be independent in the context of Grand Unified Theories (that is, not fixed by any known symmetry) and at the same time have values within a very narrow window which provides for stable nucleons and nuclei and abundant carbon. The conjecture is made that the two light quark masses and one coupling constant are ultimately determined even in the "final theory" by a choice from a large or continuous ensemble, and the prediction is offered that the correct unification scheme will not allow calculation of (md - mu)/mproton from first principles alone.
PB - American Physical Society
SN - 00346861 (ISSN)
LA - English
J2 - Rev. Mod. Phys.
M3 - Article
DB - Scopus
N1 - Export Date: 23 February 2023; Cited By: 162

ER -

TY - JOUR
AU - González de Posada, F.
TI - Current considerations around the search for extraterrestrial life
ST - Consideraciones de actualidad en torno a la búsqueda de vida extraterrestre.
PY - 2000
T2 - Anales de la Real Academia Nacional de Medicina
VL - 117
IS - 1
SP - 207
EP - 223
UR - https://www.scopus.com/inward/record.uri?eid=2-s2.0-0034575722&partnerID=40&md5=9fae1a3a19adc910cb9780b5e8b366e0
AB - In this paper, the current cosmological topics are considered: a) The fourth centenary celebration of Giordano Bruno's death at the Roman's inquisition stake. This eminent philosopher, based on the Coppernican Revolution, concibed the Cosmos as a infinite universe with innumerable inhabited worlds. He acted on reason to believe not only in extraterrestrial life but in extraterrestrial intelligent life. Here we write a few words in his memory and honour. b) The active project SETI@home in the framework of today's classic program "Search for Extra-Terrestrial Intelligence", by means of the reception of radioelectrical signals. c) Search for extrasolar planets.
KW - Astronomy
KW - Extraterrestrial Environment
KW - History, 17th Century
KW - Life
KW - Rome
KW - article
KW - astronomy
KW - history
KW - Italy
KW - life
KW - microclimate
SN - 00340634 (ISSN)
C2 - 11209557
LA - Spanish
J2 - An R Acad Nac Med (Madr)
M3 - Article
DB - Scopus
N1 - Export Date: 23 February 2023; Cited By: 0
ER -

TY - JOUR
AU - Blake, R.E.
AU - Alt, J.C.
AU - Martini, A.M.
TI - Oxygen isotope ratios of PO4: An inorganic indicator of enzymatic activity and P metabolism and a new biomarker in the search for life

PY - 2001
T2 - Proceedings of the National Academy of Sciences of the United States of America
VL - 98
IS - 5
SP - 2148
EP - 2153
DO - 10.1073/pnas.051515898
UR - https://www.scopus.com/inward/record.uri?eid=2-s2.0-0035957002&doi=10.1073%2fpnas.051515898&partnerID=40&md5=c2641b3995e379801366676cad233822
AD - Department of Geology and Geophysics, Yale University, New Haven, CT 06520-8109, United States
AD - Department of Geology, University of Michigan, Ann Arbor, MI 48109, United States
AD - Department of Geology, Amherst College, Amherst, MA 01002, United States
AB - The distinctive relations between biological activity and isotopic effect recorded in biomarkers (e.g., carbon and sulfur isotope ratios) have allowed scientists to suggest that life originated on this planet nearly 3.8 billion years ago. The existence of life on other planets may be similarly identified by geochemical biomarkers, including the oxygen isotope ratio of phosphate (δ18Op) presented here. At low near-surface temperatures, the exchange of oxygen isotopes between phosphate and water requires enzymatic catalysis. Because enzymes are indicative of cellular activity, the demonstration of enzyme-catalyzed PO4-H2O exchange is indicative of the presence of life. Results of laboratory experiments are presented that clearly show that δ18Op values of inorganic phosphate can be used to detect enzymatic activity and microbial metabolism of phosphate. Applications of δ18Op as a biomarker are presented for two Earth environments relevant to the search for extraterrestrial life: a shallow groundwater reservoir and a marine hydrothermal vent system. With the development of in situ analytical techniques and future planned sample return strategies, δ18Op may provide an important biosignature of the presence of life in extraterrestrial systems such as that on Mars.
KW - Biological Markers
KW - Enzymes
KW - Life
KW - Oxygen Isotopes
KW - Phosphates
KW - Phosphorus
KW - Bacteria (microorganisms)
KW - biological marker
KW - ground water
KW - oxygen
KW - phosphate
KW - article
KW - astronomy
KW - bacterial metabolism
KW - catalysis
KW - cell activity
KW - enzyme activity

KW - phosphate metabolism
KW - priority journal
SN - 00278424 (ISSN)
C2 - 11226207
LA - English
J2 - Proc. Natl. Acad. Sci. U. S. A.
M3 - Article
DB - Scopus
N1 - Export Date: 23 February 2023; Cited By: 66; Correspondence Address: R.E. Blake; Department of Geology and Geophysics, Yale University, New Haven, CT 06520-8109, United States; email: blake@geology.yale.edu; CODEN: PNASA
ER -

TY - CONF
AU - Seckbach, J.
AU - Chela-Flores, J.
TI - Frontiers of extremophilic microorganisms: From life on the edge to astrobiology
PY - 2001
T2 - European Space Agency, (Special Publication) ESA SP
IS - 496
SP - 255
EP - 260
UR - https://www.scopus.com/inward/record.uri?eid=2-s2.0-0034751756&partnerID=40&md5=6c0086cc077c13f082d26f6ff6781102
AD - Hebrew University of Jerusalem, Efrat 90435, P.O.B. 1132, Israel
AB - Extremophiles thrive on the edge of temperature, pH, pressure, hypersalinity, dryness, and desiccation. Such microorganisms may resemble the first living organisms which evolved on the early Earth. They may also serve as analogues of microbes on other worlds. Some celestial bodies may provide conditions for life, such as liquid water and other essential ingredients for microbial life. Consequently, life may exist elsewhere in the Solar System, even though the environments available may be more extreme than those on Earth. Wherever there is life we expect the presence of microbial life such as prokaryotic microorganisms. Prokaryotes have thrived on Earth at least since the period corresponding to 3.5 to 3.8 billion years before the present (Ga BP). They have dominated our biosphere during its first 2 Ga, even before the first eukaryote appeared, and have been the least affected through major climatic astronomical and geological events in the early Earth. Therefore, prokaryotes are the most likely candidates for a presumed biota on other worlds (although eukaryotes are also a possibility). The most promising worlds, which may harbor living microbes are Mars and the Jovian moon Europa.
SN - 03796566 (ISSN)
LA - English
J2 - Eur Space Agency Spec Publ ESA SP
M3 - Conference paper
DB - Scopus
N1 - Export Date: 23 February 2023; Cited By: 0; Correspondence Address: J. Seckbach; Hebrew University of Jerusalem, Efrat 90435,

P.O.B. 1132, Israel; email: seckbach@huji.ac.il; CODEN: ESPUD
ER -

TY - CONF
AU - Simakov, M.B.
TI - The possible sites for exobiological activity on Titan
PY - 2001
T2 - European Space Agency, (Special Publication) ESA SP
IS - 496
SP - 211
EP - 214
UR - https://www.scopus.com/inward/record.uri?eid=2-s2.0-0034751963&partnerID=40&md5=90bba126885cef77c25cad99629926b3
AD - Group of Exobiology, Institute of Cytology RAS, St. Petersburg, 194064, Tikhoretsky Av. 4, Russian Federation
AB - Titan, the largest satellite of Saturn, is currently the most favorable site for search of extraterrestrial life. This moon is one of the Solar system bodies, which could have a large quantity of liquid water. The putative internal ocean along with a rich dense atmosphere provide some new exobiological niches: (1) the upper layer of the internal water ocean; (2) water pockets and liquid veins inside icy layer; (3) the places of cryogenic volcanism; (4) set of caves in icy layer connecting with cryovolcanic processes; (5) the brine-filled cracks in icy crust caused by tidal forces; (6) liquid water pools on the surface originated from meteoritic strikes; (7) the sites of hydrothermal activity on the bottom of ocean. All conditions needed for exobiology - liquid water, complex organic chemistry and energy sources for support of biological processes - are on Saturnian moon.
SN - 03796566 (ISSN)
LA - English
J2 - Eur Space Agency Spec Publ ESA SP
M3 - Conference paper
DB - Scopus
N1 - Export Date: 23 February 2023; Cited By: 9; Correspondence Address: M.B. Simakov; Group of Exobiology, Institute of Cytology RAS, St. Petersburg, 194064, Tikhoretsky Av. 4, Russian Federation; email: exobio@usa.net; CODEN: ESPUD
ER -

TY - JOUR
AU - Boston, P.J.
AU - Spilde, M.N.
AU - Northup, D.E.
AU - Melim, L.A.
AU - Soroka, D.S.
AU - Kleina, L.G.
AU - Lavoie, K.H.
AU - Hose, L.D.
AU - Mallory, L.M.
AU - Dahm, C.N.
AU - Crossey, L.J.
AU - Schelble, R.T.
TI - Cave biosignature suites: Microbes, minerals, and Mars

PY - 2001
T2 - Astrobiology
VL - 1
IS - 1
SP - 25
EP - 55
DO - 10.1089/153110701750137413
UR - https://www.scopus.com/inward/record.uri?eid=2-s2.0-0035278887&doi=10.1089%2f153110701750137413&partnerID=40&md5=d09dc9341f7349a6626c668b9742bdd1
AD - Biology Department, University of New Mexico, Albuquerque, NM, United States
AD - Complex Systems Research, Inc., Box 11320, Boulder, CO 80301, United States
AD - Institute of Meteoritics, University of New Mexico, Albuquerque, NM, United States
AD - Geology Department, Western Illinois University, Macomb, IL, United States
AD - Caves of Tabasco Project, National Speleological Society, Huntsville, AL, United States
AD - SUNY, Plattsburgh, NY, United States
AD - Department of Environmental and Chemical Science, Chapman University, Orange, CA, United States
AD - Biomes, Inc., Amherst, MA, United States
AD - Department of Earth and Planetary Sciences, University of New Mexico, Albuquerque, NM, United States
AB - Earth's subsurface offers one of the best possible sites to search for microbial life and the characteristic lithologies that life leaves behind. The subterrain may be equally valuable for astrobiology. Where surface conditions are particularly hostile, like on Mars, the subsurface may offer the only habitat for extant lifeforms and access to recognizable biosignatures. We have identified numerous unequivocally biogenic macroscopic, microscopic, and chemical/geochemical cave biosignatures. However, to be especially useful for astrobiology, we are looking for suites of characteristics. Ideally, "biosignature suites" should be both macroscopically and microscopically detectable, independently verifiable by nonmorphological means, and as independent as possible of specific details of life chemistries - demanding (and sometimes conflicting) criteria. Working in fragile, legally protected environments, we developed noninvasive and minimal impact techniques for life and biosignature detection/characterization analogous to Planetary Protection Protocols. Our difficult field conditions have shared limitations common to extraterrestrial robotic and human missions. Thus, the cave/subsurface astrobiology model addresses the most important goals from both scientific and operational points of view. We present details of cave biosignature suites involving manganese and iron oxides, calcite, and sulfur minerals. Suites include morphological fossils, mineral-coated filaments, living microbial mats and preserved biofabrics, 13C and 34S values consistent with microbial metabolism, genetic data, unusual elemental abundances and ratios, and crystallographic mineral forms.
© Mary Ann Liebert, Inc.
KW - Exobiology

KW - Extraterrestrial Environment
KW - Humans
KW - Life
KW - Mars
KW - Minerals
KW - Robotics
KW - Space Flight
KW - mineral
KW - article
KW - astronomy
KW - biology
KW - human
KW - life
KW - methodology
KW - microclimate
KW - robotics
KW - space flight
KW - Biosignature suites
KW - Caves
KW - Life detection
KW - Mars
KW - Microorganism mineral assemblages
SN - 15311074 (ISSN)
C2 - 12448994
LA - English
J2 - Astrobiology
M3 - Article
DB - Scopus
N1 - Export Date: 23 February 2023; Cited By: 161; Correspondence Address: P. J. Boston; Complex Systems Research, Inc., Box 11320, Boulder, CO 80301, United States; email: pboston@complex.org
ER -

TY - CONF
AU - Stan-Lotter, H.
AU - Radax, C.
AU - Gruber, C.
AU - Legat, A.
AU - Pfaffenhuemer, M.
AU - Wieland, H.
TI - Viable halobacteria from permo-triassic salt deposits and the possibility of extraterrestrial microbial life
PY - 2001
T2 - European Space Agency, (Special Publication) ESA SP
IS - 496
SP - 25
EP - 31
UR - https://www.scopus.com/inward/record.uri?eid=2-s2.0-0034748657&partnerID=40&md5=4da667fbbb87711b5d89abe6cf5b951c
AD - Inst. of Genetics and Gen. Biology, A-5020 Salzburg, Hellbrunnerstr. 34, Austria
AB - The range of physico-chemical parameters for the presence of life is ever expanding, making it conceivable to search in seemingly inhospitable environments for extraterrestrial forms of life.

Extremely halophilic archaebacteria (haloarchaea) were isolated from ancient salt deposits, adding another dimension - potentially exorbitant longevity - to the unusual characteristics of prokaryotic life. Distribution of haloarchaea in salt sediments appears to be worldwide; one particular strain, Halococcus salifodinae, was found in Alpine and Zechstein sediments. By using amplification of 16S rRNA genes with DNA extracted from rock salt, we obtained evidence for numerous haloarchaea, which have not been cultivated yet. Phylogenetic analysis revealed novel taxa within known lineages of haloarchaea. The results could be of relevance for astrobiology: together with the recent findings of halite, sylvite and water in some meteorites, it is intriguing to consider a specific search for extreme halophiles in Mars return samples or other materials from celestial bodies.
SN - 03796566 (ISSN)
LA - English
J2 - Eur Space Agency Spec Publ ESA SP
M3 - Conference paper
DB - Scopus
N1 - Export Date: 23 February 2023; Cited By: 8; Correspondence Address: H. Stan-Lotter; Inst. of Genetics and Gen. Biology, A-5020 Salzburg, Hellbrunnerstr. 34, Austria; email: helga.stan-lotter@sbg.ac.at; CODEN: ESPUD
ER -

TY - CONF
AU - Foing, B.H.
TI - Exo-astrobiology with ESA space science missions
PY - 2001
T2 - European Space Agency, (Special Publication) ESA SP
IS - 496
SP - 121
EP - 126
UR - https://www.scopus.com/inward/record.uri?eid=2-s2.0-0034750421&partnerID=40&md5=b9f71063cb1465d2036bb1621e55001b
AD - ESA Research Division, ESTEC/SCI-SR, NL-2200 AG Noordwijk, Postbus 299, Netherlands
AB - Key questions of astrobiology can be addressed by several space missions from the ESA Science Horizons 2000 Programme, such as: - How do solar and stellar systems form ? (with ISO, FIRST, SMART-1, Rosetta, Colombo, Gaia) - Geological evolution of terrestrial planets (with Living planet, Mars-express, SMART-1, Bepi-Colombo to Mercury) - Interstellar Complex organic chemistry (with ISO, ISS/EXPOSE, FIRST, Rosetta) - Co-evolution of Earth-Moon, impacts life frustration (with SMART-1, Bepi-Colombo) - How to detect other solar systems and habitable zones (with space photometry, COROT, Eddington, Gaia, IRSI-Darwin) - Early Earth and alternative environments (Huygens/Cassini and Mars-express) - Signature of biosphere, global biomarkers and photosynthesis evolution (living Planet missions, Darwin) - Water and exobiology on Mars (with orbiter instruments and Beagle-2 lander on Mars Express) - Study of biomarkers and delivery of organics (with Mars-express and future missions) We shall review the Exo-Astrobiology potential from these ESA missions.

KW - Astrobiology
KW - Astrochemistry
KW - Astronomy
KW - Comets
KW - Exobiology
KW - Meteorit es
KW - Origin of life
KW - Planets
SN - 03796566 (ISSN)
LA - English
J2 - Eur Space Agency Spec Publ ESA SP
M3 - Conference paper
DB - Scopus
N1 - Export Date: 23 February 2023; Cited By: 1; CODEN: ESPUD
ER -

TY - CONF
AU - Jones, H.
TI - Extraterrestrial ecology (exoecology)
PY - 2001
T2 - SAE Technical Papers
DO - 10.4271/2001-01-2143
UR - https://www.scopus.com/inward/record.uri?eid=2-s2.0-85072473279&doi=10.4271%2f2001-01-2143&partnerID=40&md5=2ee558c436935fdc9c90b0eb40a6b602
AD - Mail Stop 239-8, NASA, Ames Research Center, Moffett Field, CA 94035-1000, United States
AB - Researchers in astrobiology should develop alternate concepts for the detection of extraterrestrial life. We should search for extraterrestrial ecology, exoecology, as well as for extraterrestrial biology, exobiology. Ecology describes the interactions of living things with their environment. All ecosystems are highly constrained by their environment and conform to well-known and inescapable system design principles. An ecology could exist wherever there is an energy source and living things can employ some method to capture, store, and use the available energy. Terrestrial ecosystems use energy sources including light, organic molecules, and, in thermal vents and elsewhere, simple inorganic molecules. Ecosystem behavior is controlled by matter and energy conservation laws and is described by dynamic systems theory. Typically in an ecosystem different molecules are not in chemical equilibrium and scarce materials are conserved, stored, or recycled. Temporal cycles and spatial variations are often observed. The general concepts of exoecology can help guide the search for extraterrestrial life. The best strategy for the detection of extraterrestrial life is probably a mixed strategy. We should use detailed assumptions based on terrestrial biology to guide some but not all future searches for alien life. The systems principles of exoecology seem much more fundamental and inevitable than the analogies with terrestrial biology that are usually accepted in exobiology. We should search for exoecology based on general principles as well as for exobiology using familiar mechanisms. Copyright © 2001 Society of Automotive Engineers, Inc.
KW - Astrobiology

KW - Biology
KW - Molecules
KW - System theory
KW - Chemical equilibriums
KW - Design Principles
KW - Energy conservation law
KW - Extraterrestrial life
KW - Inorganic molecules
KW - Spatial variations
KW - Systems principles
KW - Terrestrial ecosystems
KW - Ecosystems
PB - SAE International
SN - 01487191 (ISSN)
LA - English
J2 - SAE Techni. Paper.
M3 - Conference paper
DB - Scopus
N1 - Export Date: 23 February 2023; Cited By: 0; Correspondence Address: H. Jones; Mail Stop 239-8, NASA, Ames Research Center, Moffett Field, CA 94035-1000, United States; email: hjones@mail.arc.nasa.gov
ER -

TY - CONF
AU - Westall, F.
AU - Nijman, W.
AU - Brack, A.
AU - Steele, A.
AU - Toporski, J.
TI - The oldest fossil life on Earth, its geological context and life on Mars
PY - 2001
T2 - European Space Agency, (Special Publication) ESA SP
IS - 496
SP - 81
EP - 89
UR - https://www.scopus.com/inward/record.uri?eid=2-s2.0-0034750365&partnerID=40&md5=b09e63de521939d9b0692f7b7c247c1a
AD - Centre de Biophysique Moleculaire, CNRS, 45071 Orleans Cedex 2, Rue Charles Sadron, France
AB - Life on Earth, as the only example that we have, provides the only acailable vehicle of study for potential extraterrestrial life. Given the similarities in the early histories of the terrestrial planets, life could have arisen on Mars or Venus, as well as elsewhere in our solar system. The most suitable candidate in the search for extraterrestrial life is Mars. Since Mars has not been subjected to the destructive plate tectonic forces which have eliminated the early crustal record from the Earth, there is also the possibility that Mars may harbour important evidence relating to the origin of life in which is lacking on Earth. Nevertheless, the remains of the early terrestrial record document a planetary context for the origin of life and its early evolution which was very different to that of the modern day Earth. Submerged

protocontinenal platforms provided shallow water environments in a volcanically and hydrothermally dynamic environment, subject to bolide impacts. Early life was abundant in these environments. Early Mars differed in that it was not water covered. However, this may not have been relevant for the surface-bound processes leading to the origin of life and its distribution.
SN - 03796566 (ISSN)
LA - English
J2 - Eur Space Agency Spec Publ ESA SP
M3 - Conference paper
DB - Scopus
N1 - Export Date: 23 February 2023; Cited By: 5; Correspondence Address: F. Westall; Centre de Biophysique Moleculaire, CNRS, 45071 Orleans Cedex 2, Rue Charles Sadron, France; email: frances.westall@tin.it; CODEN: ESPUD
ER -

TY - JOUR
AU - Rummel, J.D.
TI - Planetary exploration in the time of astrobiology: Protecting against biological contamination
PY - 2001
T2 - Proceedings of the National Academy of Sciences of the United States of America
VL - 98
IS - 5
SP - 2128
EP - 2131
DO - 10.1073/pnas.061021398
UR - https://www.scopus.com/inward/record.uri?eid=2-s2.0-0035956897&doi=10.1073%2fpnas.061021398&partnerID=40&md5=ae0a53f35235413f7398ff59f2023dd7
AD - Natl. Aeronautics/Space Admin. Hq., Washington, DC 20546, United States
AB - These are intriguing times in the exploration of other solar-system bodies. Continuing discoveries about life on Earth and the return of data suggesting the presence of liquid water environments on or under the surfaces of other planets and moons have combined to suggest the significant possibility that extraterrestrial life may exist in this solar system. Similarly, not since the Viking missions of the mid-1970s has there been as great an appreciation for the potential for Earth life to contaminate other worlds. Current plans for the exploration of the solar system include constraints intended to prevent biological contamination from being spread by solar-system exploration missions.
KW - Exobiology
KW - Planets
KW - Bacteria (microorganisms)
KW - bacterium contamination
KW - environment
KW - priority journal
KW - review
KW - space
KW - space flight

SN - 00278424 (ISSN)
C2 - 11226203
LA - English
J2 - Proc. Natl. Acad. Sci. U. S. A.
M3 - Review
DB - Scopus
N1 - Export Date: 23 February 2023; Cited By: 87; Correspondence Address: J.D. Rummel; Natl. Aeronautics/Space Admin. Hq., Washington, DC 20546, United States; email: jrummel@hq.nasa.gov; CODEN: PNASA
ER -

TY - JOUR
AU - Crawford, R.L.
AU - Paszczynski, A.
AU - Lang, Q.
AU - Cheng, I.Francis
AU - Barnes, B.
AU - Anderson, T.J.
AU - Wells, R.
AU - Wai, C.
AU - Corti, G.
AU - Allenbach, L.
AU - Erwin, D.P.
AU - Park, J.
AU - Assefi, T.
AU - Mojarradi, M.
TI - In search of the molecules of life
PY - 2001
T2 - Icarus
VL - 154
IS - 2
SP - 531
EP - 539
DO - 10.1006/icar.2001.6714
UR - https://www.scopus.com/inward/record.uri?eid=2-s2.0-0035736232&doi=10.1006%2ficar.2001.6714&partnerID=40&md5=9782465667f6e2588aba58d9d07014ac
AD - Environmental Biology Institute, University of Idaho, Moscow, Idaho, 83844, United States
AD - Department of Chemistry, University of Idaho, Moscow, Idaho, 83844, United States
AD - Department of Electrical Engineering, And Microelectronics Research and Communication Institute, University of Idaho, Moscow, Idaho, 83844, United States
AD - Department of Mechanical Engineering, And Microelectronics Research and Communication Institute, University of Idaho, Moscow, Idaho, 83844, United States
AD - Microelectronics Research and Communication Institute, University of Idaho, Moscow, Idaho, 83844, United States
AD - Environmental Biology Institute, University of Idaho, Moscow, Idaho, 83844, United States
AD - Jet Propulsion Laboratory, California Institute of Technology, 4800 Oak Grove Drive, Pasadena, California, 91109, United States

AB - The remote detection of a chemical signature of extraterrestrial life ideally requires a broad and nonEarth-centric definition of life. Thus, our proposed approach to detection is based on fundamental thermodynamic assumptions, and some assumptions of how life might obtain energy from its environment. Life, defined as the ability to self-perpetuate, requires a continual input of energy and information. The energy must be tapped in controlled oxidation-reduction reactions between electron donors and acceptors along an electron transport chain. Therefore, the core chemical components of such electron transport chains should be detectable as a signature of life. On Earth, such core structures are principally molecules resembling the porphyrins, quinones, flavins, and nicotinamides (e.g., photosynthetic or respiratory pigments, redox enzymes, and cytochromes). Similar redox-active molecules, perhaps with different structures, might be associated with extraterrestrial life. To validate an approach based on redox signature, signature chemicals were extracted from soil and analyzed by different methods and equipment that may eventually form an integrated "laboratory on a chip" to be used on other planets or their moons. Its components are a supercritical fluid or solvent extraction module, a separation module, and a detection module with an electrochemical detector and electrospray tandem mass-spectrometer. © 2001 Elsevier Science (USA).
KW - Exobiology
KW - Instrumentation
KW - Organic chemistry
PB - Academic Press Inc.
SN - 00191035 (ISSN)
LA - English
J2 - Icarus
M3 - Article
DB - Scopus
N1 - Export Date: 23 February 2023; Cited By: 16; Correspondence Address: R.L. Crawford; Environmental Biology Institute, University of Idaho, Moscow, ID 83844, United States; email: crawford@uidaho.edu; CODEN: ICRSA
ER -

TY - CONF
AU - Kaiser, R.I.
TI - The role of cyano (CN) and ethynyl (C2H) radicals in astrobiology and implications to the origin of life on earth
PY - 2001
T2 - European Space Agency, (Special Publication) ESA SP
IS - 496
SP - 145
EP - 153
UR - https://www.scopus.com/inward/record.uri?eid=2-s2.0-0034749097&partnerID=40&md5=937a46ae6435feda9b6e274f8cadb808
AD - Department of Chemistry, University of York, York YO10 5DD, United Kingdom
AB - Crossed beam experiments of cyano (CN(X2Σ+) and ethynyl C2H(X2Σ+) radicals with unsaturated hydrocarbons acetylene, ethylene, methylacetylene, and benzene have been performed to

investigate synthetic routes to form nitriles, polyynes, and substituted allenes in hydrocarbon-rich atmospheres of planets and their moons. All radical reactions proceed without entrance barrier, have exit barriers well below the energy of the reactant molecules, and are strongly exothermic. The predominant identification of the radical versus atomic hydrogen exchange channel makes these reactions compelling candidates to form complex organic chemicals - precursors to biologically important amino acids - in solar system environments.
SN - 03796566 (ISSN)
LA - English
J2 - Eur Space Agency Spec Publ ESA SP
M3 - Conference paper
DB - Scopus
N1 - Export Date: 23 February 2023; Cited By: 7; Correspondence Address: R.I. Kaiser; Department of Chemistry, University of York, York YO10 5DD, United Kingdom; email: rikl@york.ac.uk; CODEN: ESPUD
ER -

TY - CONF
AU - Rosenberg, S.D.
AU - Makel, D.B.
AU - Carranza, S.
TI - In situ synthesis of nutrients and hydrocarbons for isolated habitat support
PY - 2001
T2 - 39th Aerospace Sciences Meeting and Exhibit
UR - https://www.scopus.com/inward/record.uri?eid=2-s2.0-84897845952&partnerID=40&md5=a654f232ef15116951a598d5c4455604
AD - President, In-Space Propulsion, Ltd, Sacramento, CA, United States
AD - President, Makel Engineering Inc, Chico, CA, United States
AD - Chemical Engineer, Makel Engineering Inc, Chico, CA, United States
AB - Our program is based on these premises: (1) ethylene, ethanol, polyethylene, and other useful products can be synthesized from metabolic wastes to support off-earth ecosystems and (2) these products are best synthesized using inorganic processes based on chemical engineering principles. Our long term objective is to provide the means to obtain closed cycle ecosystems in remote, isolated habitats. We have demonstrated: (1) synthesis of ethylene by catalytic reduction of carbon dioxide with hydrogen, (2) catalytic reformation of methane with oxygen to form ethylene, and (3) feasibility of using ethylene to synthesize ethanol which, in turn, nay become part of an environmental control system for the preparation of other nutrients for use in remote, isolated habitats to support Astrobiological research. Note that these in situ products will be synthesized using inorganic processes based on chemical engineering principles, making use of the major components of metabolic waste, carbon, hydrogen, and oxygen. This technology can be used as an adjunct to life support systems required in manned spacecraft, e.g., Space Station Freedom, and planetary bases, e.g., the Moon and Mars. In addition, it can be used on Mars by converting carbon dioxide (atmosphere) and water (atmosphere and soils, etc.)

to ethylene and other useful products for the manufacture of foodstuffs for life support and plastics for the fabrication of structures and spare parts for Martian bases. In addition, this technology can be used for the production of oxygen and methane for use as propellants in local and space propulsion systems. The program is focused on two synthetic paths: to produce ethylene, (1) direct catalytic reduction of carbon dioxide with hydrogen, as shown in the following equations. © 2001 by Sanders D. Rosenberg.
KW - Aerospace engineering
KW - Carbon dioxide
KW - Chemical engineering
KW - Ethanol
KW - Ethylene
KW - Fabrication
KW - Manned space flight
KW - Metabolism
KW - Methane
KW - Nutrients
KW - Oxygen
KW - Space stations
KW - Spacecraft propulsion
KW - Catalytic reduction
KW - Engineering principles
KW - Environmental control system
KW - In-situ synthesis
KW - Inorganic process
KW - Life supports
KW - Manned spacecraft
KW - Space propulsion system
KW - Ecosystems
LA - English
J2 - 39th Aerosp. Sci. Meet. Exhibit
M3 - Conference paper
DB - Scopus
N1 - Export Date: 23 February 2023; Cited By: 0
ER -

TY - JOUR
AU - Davies, P.
TI - The origin of life. I: When and where did it begin?
PY - 2001
T2 - Science progress
VL - 84
IS - Pt 1
SP - 1
EP - 16
DO - 10.3184/003685001783239069
UR - https://www.scopus.com/inward/record.uri?eid=2-s2.0-0035235019&doi=10.3184%2f003685001783239069&partnerID=40&md5=b7b31409364f5ba87df7f414840f5bf1
AD - Physics Department, Imperial College London.
AB - For decades most scientists assumed that life emerged billions of years ago in a "primordial soup" somewhere on the Earth's surface. Evidence is mounting, however, that life may have begun

deep beneath the surface, perhaps near a volcanic ocean vent or even inside the hot crust itself. Since there are hints that life's history on Earth extends back through the phase of massive cosmic bombardment, it may be that life started on Mars and came here later, perhaps inside rocks ejected from the Red Planet by large impacts. The traffic of intact rocks between Mars and Earth is now an established fact, and experiments confirm that microbes could survive the rigours of the journey through space if cocooned within such material. Unfortunately, this planetary cross-contamination compromises astrobiologists' hope of finding a second genesis in the solar system.
KW - Animals
KW - Bacteria
KW - Biogenesis
KW - Ecosystem
KW - Humans
KW - Mars
KW - Phylogeny
KW - animal
KW - article
KW - astronomy
KW - bacterium
KW - biogenesis
KW - ecosystem
KW - human
KW - phylogeny
SN - 00368504 (ISSN)
C2 - 11382134
LA - English
J2 - Sci Prog
M3 - Article
DB - Scopus
N1 - Export Date: 23 February 2023; Cited By: 5
ER -

TY - CONF
AU - Guzelian, A.A.
AU - Sylvia, J.M.
AU - Janni, J.A.
AU - Clauson, S.L.
AU - Spencer, K.M.
TI - SERS of whole cell bacteria and trace levels of biological molecules
PY - 2001
T2 - Proceedings of SPIE - The International Society for Optical Engineering
VL - 4577
SP - 182
EP - 192
DO - 10.1117/12.455736
UR - https://www.scopus.com/inward/record.uri?eid=2-s2.0-0035776158&doi=10.1117%2f12.455736&partnerID=40&md5=7f12984fa2e977d875a7a9654d24d7c1
AD - EIC Laboratories, Inc., Norwood, MA 02062, 111 Downey Street,

United States
AB - Through its several orders of magnitude signal enhancement over normal Raman, surface-enhanced Raman spectroscopy (SERS) provides an opportunity to extend the benefits of vibrational spectroscopy to trace level detection. SERS in particular holds great potential for biological sensing due to the weak Raman bands of water and the reduction in fluorescence backgrounds from interactions of the analyte with the metal SERS substrate. This work examines the trace level detection (<100 ppm) of biological molecules and oligomers such as amino acids, peptides, and oligonucleotides as well as the detection of whole cell bacteria. The SERS substrates employed are electrochemically roughened gold. The biological molecules show well-resolved and intense bands that are an effective spectral signature; these bands also persist in corresponding oligomeric compounds. Spectra from whole cell bacteria have been obtained for several species, including gram-positive and gram-negative strains. Viable and nonviable cells have also been examined and significant spectral differences are observed. The results show the potential for using SERS as an analytical tool for the identification of biological molecules and microorganisms with applications in biological agent detection, food and water monitoring, and the search for signs of extraterrestrial life.
KW - Amino acids
KW - Bacteria
KW - Cells
KW - Fluorescence
KW - Molecular vibrations
KW - Nucleic acids
KW - Oligomers
KW - Raman scattering
KW - Surface plasmon resonance
KW - Trace analysis
KW - Biological sensing
KW - Oligonucleotides
KW - Peptides
KW - Resonance Raman scattering
KW - Surface-enhanced Raman spectroscopy
KW - Vibrational spectroscopy
KW - Raman spectroscopy
KW - Amino acids
KW - Bacteria
KW - Nucleic acids
KW - Raman
KW - SERS
KW - Trace detection
SN - 0277786X (ISSN)
LA - English
J2 - Proc SPIE Int Soc Opt Eng
M3 - Conference paper
DB - Scopus
N1 - Export Date: 23 February 2023; Cited By: 49; CODEN: PSISD
ER -

TY - JOUR

AU - Bada, J.L.
TI - State-of-the-art instruments for detecting extraterrestrial life
PY - 2001
T2 - Proceedings of the National Academy of Sciences of the United States of America
VL - 98
IS - 3
SP - 797
EP - 800
DO - 10.1073/pnas.98.3.797
UR - https://www.scopus.com/inward/record.uri?eid=2-s2.0-0035969992&doi=10.1073%2fpnas.98.3.797&partnerID=40&md5=b39030dbcf96d75ef24fae53fd53f4ca
AD - Scripps Institution of Oceanography, University of California, San Diego, CA 92093-0212, United States
KW - Amino Acids
KW - Biogenesis
KW - Extraterrestrial Environment
KW - Mars
KW - Solar System
KW - Space Flight
KW - carbon
KW - hydrocarbon
KW - methane
KW - nucleic acid base
KW - ozone
KW - polycyclic aromatic compound
KW - protein
KW - water
KW - astronomy
KW - atmosphere
KW - biogenesis
KW - catalysis
KW - chemical composition
KW - DNA replication
KW - evolution
KW - instrument
KW - life
KW - photosynthesis
KW - priority journal
KW - review
KW - technology
SN - 00278424 (ISSN)
C2 - 11158548
LA - English
J2 - Proc. Natl. Acad. Sci. U. S. A.
M3 - Review
DB - Scopus
N1 - Export Date: 23 February 2023; Cited By: 49; Correspondence Address: J.L. Bada; Scripps Institution of Oceanography, University of California, San Diego, CA 92093-0212, United States; email: jbada@ucsd.edu; CODEN: PNASA
ER -

TY - JOUR
AU - Cockell, C.S.
TI - 'Astrobiology' and the ethics of new science
PY - 2001
T2 - Interdisciplinary Science Reviews
VL - 26
IS - 2
SP - 90
EP - 96
DO - 10.1179/isr.2001.26.2.90
UR - https://www.scopus.com/inward/record.uri?eid=2-s2.0-0005222994&doi=10.1179%2fisr.2001.26.2.90&partnerID=40&md5=ae170a60385ab0e0af5103d86e365b29
AD - Cambridge CBI 2NU, 12 Kingston Street, United Kingdom
AD - Natural Sciences, Fitzwilliam College, Cambridge, United Kingdom
AB - Astrobiology has recently been feted as a science for the twenty-first century. Founded as a science in the Soviet Union in 1953, its recent manifestation has been accompanied by a large media response and a series of new books. The histories of the term 'astrobiology' and its twin sister 'exobiology' raise interesting ethical questions about how the scientific community recognises a field as being a separate entity and about the processes by which the international scientific community engages in debate on the establishment (or in this case rebirth) of a field. Comparison with the history of the emergence of fields such as nanotechnology and neurobiology illustrates the complexities involved. These complexities have become exaggerated in a world where the majority of scientific output is presided over by market economics. In societies where marketing and media interest have become intimately intertwined with scientific funding and political support for science, the declaration of new fields of activity presents novel problems in the process of scientific enquiry.
PB - IOM Communications Ltd.
SN - 03080188 (ISSN)
LA - English
J2 - Interdiscip. Sci. Rev.
M3 - Article
DB - Scopus
N1 - Export Date: 23 February 2023; Cited By: 15; Correspondence Address: C.S. Cockell; Cambridge CBI 2NU, 12 Kingston Street, United Kingdom; email: csco@pcmail.nerc-bas.ac.uk
ER -

TY - JOUR
AU - Bartsev, S.I.
AU - Mezhevikin, V.V.
AU - Okhonin, V.A.
TI - Life as a set of matter transformation cycles: Ecological attributes of life
PY - 2001
T2 - Advances in Space Research
VL - 28

IS - 4
SP - 607
EP - 612
DO - 10.1016/S0273-1177(01)00389-1
UR - https://www.scopus.com/inward/record.uri?eid=2-s2.0-0035413647&doi=10.1016%2fS0273-1177%2801%2900389-1&partnerID=40&md5=ba8a72e51e10a5bed7ea9b056d7b6e19
AD - Institute of Biophysics, Siberian Branch, Russian Academy of Sciences, Krasnoyarsk, 660036, Russian Federation
AB - An approach to searching for extraterrestrial life on the base of "autotroph" concept of the origin of life is presented in the paper. According to this concept the origin of life took place in three stages. The first stage was developed inside the global geochemical cycle in which the turnover of different chemical transformations was implemented by solar radiation and/or heat energy of bowels of the Earth. At the second stage, after the autocatalytic systems have emerged these systems evolved as a result of "natural selection" by autocatalysis parameters up to emergence of special inheritance systems that drastically improved the autocatalysis parameters. The best in terms of autocatalysis parameters were the autocatalysis systems based on phase-separated particles where complex structures can form not only on the basis of covalent interactions. Such autocatalysis systems can emerge only in liquid in a certain range of temperatures and pressures. At this stage the geochemical cycle complicated involving new substances. At the third stage the evolution involved improvement of inheritance systems resulting in formation of the modern type of genetic apparatus. This concept formed the basis to consider approaches to experimental modeling of major aspects of the origin of life and to outlining some general features of life that can extend the sensitive horizon of searching for extraterrestrial life.
KW - Biogenesis
KW - Carbon Dioxide
KW - Catalysis
KW - Earth (Planet)
KW - Evolution
KW - Evolution, Chemical
KW - Exobiology
KW - Hydrogen
KW - Methane
KW - Oxygen
KW - Sunlight
KW - Water
KW - Computer simulation
KW - Extraterrestrial atmospheres
KW - Geochemistry
KW - Pressure effects
KW - Solar radiation
KW - Thermal effects
KW - carbon dioxide
KW - hydrogen
KW - methane
KW - oxygen
KW - water

KW - Autocatalytic systems
KW - autotrophy
KW - exobiology
KW - origin of life
KW - article
KW - astronomy
KW - biogenesis
KW - biology
KW - catalysis
KW - chemistry
KW - evolution
KW - sunlight
KW - Ecology
PB - Elsevier Ltd
SN - 02731177 (ISSN)
C2 - 11803961
LA - English
J2 - Adv. Space Res.
M3 - Article
DB - Scopus
N1 - Export Date: 23 February 2023; Cited By: 7; Correspondence Address: S.I. Bartsev; Institute of Biophysics, Siberian Branch of Russian Academy, Krasnoyarsk, 660036, Russian Federation; email: bartsev@online.ru; CODEN: ASRSD
ER -

TY - JOUR
AU - Irwin, L.N.
AU - Schulze-Makuch, D.
TI - Assessing the plausibility of life on other worlds
PY - 2001
T2 - Astrobiology
VL - 1
IS - 2
SP - 143
EP - 160
DO - 10.1089/153110701753198918
UR - https://www.scopus.com/inward/record.uri?eid=2-s2.0-0035372570&doi=10.1089%2f153110701753198918&partnerID=40&md5=66ce36060ab0e7016b4ffd1617be8577
AD - Department of Biological Sciences, University of Texas at El Paso, El Paso, TX 79968, United States
AD - Department of Geological Sciences, University of Texas at El Paso, El Paso, TX 79968, United States
AB - As the field of astrobiology matures and search strategies for life on other worlds are developed, the need to analyze in a systematic way the plausibility for life on other planetary systems becomes increasingly apparent. We propose the adoption of a simple plausibility of life (POL) rating system based on specific criteria. Category I applies to any body shown to have conditions essentially equivalent to those on Earth. Category II applies to bodies for which there is evidence of liquid water and sources of energy and where organic compounds have been detected or can reasonably be inferred (Mars, Europa). Category III applies to worlds where

conditions are physically extreme but possibly capable of supporting exotic forms of life unknown on Earth (Titan, Triton). Category IV applies to bodies that could have seen the origin of life prior to the development of conditions so harsh as to make its perseverance at present unlikely but conceivable in isolated habitats (Venus, Io). Category V would be reserved for sites where conditions are so unfavorable for life by any reasonable definition that its origin or persistence there cannot be rated a realistic probability (the Sun, gas giant planets). The proposed system is intended to be generic. It assumes that life is based on polymeric chemistry occurring in a liquid medium with uptake and degradation of energy from the environment. Without any additional specific assumptions about the nature of life, the POL system is universally applicable. © Mary Ann Liebert, Inc.
KW - Biogenesis
KW - Earth (Planet)
KW - Evolution
KW - Exobiology
KW - Extraterrestrial Environment
KW - Geology
KW - Jupiter
KW - Mars
KW - Neptune
KW - Planets
KW - Saturn
KW - Solar System
KW - Venus
KW - Water
KW - water
KW - article
KW - astronomy
KW - biogenesis
KW - biology
KW - evolution
KW - geology
KW - microclimate
KW - Origin life
KW - Persistence of life
KW - Plausibility of life
KW - Requirements for life
KW - Solar system
SN - 15311074 (ISSN)
C2 - 12467118
LA - English
J2 - Astrobiology
M3 - Article
DB - Scopus
N1 - Export Date: 23 February 2023; Cited By: 46; Correspondence Address: L. N. Irwin; Department of Biological Sciences, University of Texas at El Paso, El Paso, TX 79968, United States; email: lirwin@utep.edu
ER -

TY - JOUR

AU - Brack, A.
AU - Horneck, G.
AU - Wynn-Williams, D.
TI - Exo/astrobiology in Europe
PY - 2001
T2 - Origins of Life and Evolution of the Biosphere
VL - 31
IS - 4-5
SP - 459
EP - 480
DO - 10.1023/A:1011832902468
UR - https://www.scopus.com/inward/record.uri?eid=2-s2.0-0034836693&doi=10.1023%2fA%3a1011832902468&partnerID=40&md5=deeabcea455e25f805b26ed5035764ca
AD - Centre de Biophysique Moléculaire, CNRS, 45071 Orléans Cedex, rue Charles Sadron, France
AD - DLR, Institute of Aerospace Medicine, Linder Höhe, 51147 Köln, Germany
AD - British Antarctic Survey, Cambridge CB3 0ET, High Cross, Madingley Road, United Kingdom
AB - The question of the chemical origins of life is engraved in the European scientific patrimony as it can be traced back to the pioneer ideas of Charles Darwin, Louis Pasteur, and more recently to Alexander Oparin. During the last decades, the European community of origin of life scientists has organized seven out of the twelve International Conferences on the Origins of Life held since 1957. This community contributed also to enlarge the field of research to the study of life in extreme environments and to the search for extraterrestrial life, i.e. exobiology in its classical definition or astrobiology if one uses a more NASA-inspired terminology. The present paper aims to describe the European science background in exo/astrobiology as well as the project of a European Network of Exo/Astrobiology.
KW - Astronomy
KW - Biogenesis
KW - Europe
KW - Exobiology
KW - Extraterrestrial Environment
KW - International Cooperation
KW - Phylogeny
KW - Research
KW - Solar System
KW - Europe
KW - cosmochemistry
KW - exobiology
KW - history of geology
KW - origin of life
KW - article
KW - astronomy
KW - biogenesis
KW - biology
KW - Europe
KW - international cooperation
KW - microclimate

KW - phylogeny
KW - research
KW - Chemical evolution
KW - Europe
KW - Exoplanets
KW - Extremophiles
KW - Homochirality
KW - Hydrothermal systems
KW - Mars
KW - Microfossils
KW - Micrometeorites
KW - Origins of life
KW - Panspermia
KW - RNA
KW - Titan
SN - 01696149 (ISSN)
C2 - 11599180
LA - English
J2 - Orig. Life Evol. Biosph.
M3 - Article
DB - Scopus
N1 - Export Date: 23 February 2023; Cited By: 13; CODEN: OLEBE
ER -

TY - JOUR
AU - Newsom, H.E.
AU - Hagerty, J.J.
AU - Thorsos, I.E.
TI - Location and sampling of aqueous and hydrothermal deposits in martian impact craters
PY - 2001
T2 - Astrobiology
VL - 1
IS - 1
SP - 71
EP - 88
DO - 10.1089/153110701750137459
UR - https://www.scopus.com/inward/record.uri?eid=2-s2.0-0035279284&doi=10.1089%2f153110701750137459&partnerID=40&md5=276a68197a07255347e487137f79f2d1
AD - Institute of Meteoritics, Department of Earth and Planetary Sciences, University of New Mexico, Albuquerque, NM, United States
AD - Institute of Meteoritics, Department of Earth and Planetary Sciences, University of New Mexico, Albuquerque, NM 8713, United States
AB - Do large craters on Mars represent sites that contain aqueous and hydrothermal deposits that provide clues to astrobiological processes? Are these materials available for sampling in large craters? Several lines of evidence strongly support the exploration of large impact craters to study deposits important for astrobiology. The great depth of impact craters, up to several kilometers relative to the surrounding terrain, can allow the breaching of local aquifers, providing a source of water for lakes and hydrothermal systems. Craters can also be filled with water from

outflow channels and valley networks to form large lakes with accompanying sedimentation. Impact melt and uplifted basement heat sources in craters >50 km in diameter should be sufficient to drive substantial hydrothermal activity and keep crater lakes from freezing for thousands of years, even under cold climatic conditions. Fluid flow in hydrothermal systems is focused at the edges of large planar impact melt sheets, suggesting that the edge of the melt sheets will have experienced substantial hydrothermal alteration and mineral deposition. Hydrothermal deposits, fine-grained lacustrine sediments, and playa evaporite deposits may preserve evidence for biogeochemical processes that occurred in the aquifers and craters. Therefore, large craters may represent giant Petri dishes for culturing preexisting life on Mars and promoting biogeochemical processes. Landing sites must be identified in craters where access to the buried lacustrine sediments and impact melt deposits is provided by processes such as erosion from outflow channels, faulting, aeolian erosion, or excavation by later superimposed cratering events. Very recent gully formation and small impacts within craters may allow surface sampling of organic materials exposed only recently to the harsh oxidizing surface environment. © Mary Ann Liebert, Inc.
KW - Exobiology
KW - Extraterrestrial Environment
KW - Geography
KW - Geologic Sediments
KW - Mars
KW - Thermodynamics
KW - Water
KW - water
KW - article
KW - astronomy
KW - biology
KW - geography
KW - methodology
KW - microclimate
KW - sediment
KW - thermodynamics
KW - Aqueous environments
KW - Central uplift
KW - Crater lakes
KW - Hydrothermal deposits
KW - Impact craters
KW - Impact melt
KW - Landing sites
KW - Mars
SN - 15311074 (ISSN)
C2 - 12448996
LA - English
J2 - Astrobiology
M3 - Article
DB - Scopus
N1 - Export Date: 23 February 2023; Cited By: 66; Correspondence Address: H. E. Newsom; Institute of Meteoritics, Department of Earth and Planetary Sciences, University of New Mexico, Albuquerque, NM

8713, United States; email: newsom@unm.edu
ER -

TY - JOUR
AU - Cooper, J.F.
AU - Johnson, R.E.
AU - Mauk, B.H.
AU - Garrett, H.B.
AU - Gehrels, N.
TI - Energetic Ion and Electron Irradiation of the Icy Galilean Satellites
PY - 2001
T2 - Icarus
VL - 149
IS - 1
SP - 133
EP - 159
DO - 10.1006/icar.2000.6498
UR - https://www.scopus.com/inward/record.uri?eid=2-s2.0-0000444307&doi=10.1006%2ficar.2000.6498&partnerID=40&md5=e8dbdb8ae5543eb67787be7e2cc962d5
AD - Raytheon Info. Technol. Sci. Serv., SSDOO Project, Code 632, NASA Goddard Space Flight Center, Greenbelt, MD 20771, United States
AD - Engineering Physics, Thornton Hall B103, University of Virginia, Charlottesville, VA 22903, United States
AD - NASA/Goddard Inst. for Space Studies, New York, NY 10025, 2880 Broadway, United States
AD - Applied Physics Laboratory, Johns Hopkins University, Laurel, MD 20723-6099, 11100 Johns Hopkins Road, United States
AD - Jet Propulsion Laboratory, M/S: 301-456, Pasadena, CA 91109-8099, 4800 Oak Grove Drive, United States
AD - Lab. for High Energy Astrophysics, Code 661, NASA Goddard Space Flight Center, Greenbelt, MD 20771, United States
AB - Galileo Orbiter measurements of energetic ions (20 keV to 100 MeV) and electrons (20-700 keV) in Jupiter's magnetosphere are used, in conjunction with the JPL electron model (<40 MeV), to compute irradiation effects in the surface layers of Europa, Ganymede, and Callisto. Significant elemental modifications are produced on unshielded surfaces to approximately centimeter depths in times of ≤106 years, whereas micrometer depths on Europa are fully processed in ~10 years. Most observations of surface composition are limited to optical depths of ~1 mm, which are in direct contact with the space environment. Incident flux modeling includes Størmer deflection by the Ganymede dipole magnetic field, likely variable over that satellite's irradiation history. Delivered energy flux of ~8×1010 keV (cm2-s)-1 at Europa is comparable to total internal heat flux in the same units from tidal and radiogenic sources, while exceeding that for solar UV energies (〉 6 eV) relevant to ice chemistry. Particle energy fluxes to Ganymede's equator and Callisto are similar at ~2-3×108 keV (cm2-s)-1 with 5×109 at Ganymede's polar cap, the latter being comparable to radiogenic energy input. Rates of change in optical reflectance and molecular composition on Europa, and on Ganymede's polar cap, are strongly driven by energy from irradiation, even in relatively young regions. Irradiation of

nonice materials can produce SO2 and CO2, detected on Callisto and Europa, and simple to complex hydrocarbons. Iogenic neutral atoms and meteoroids deliver negligible energy ~104-5 keV (cm2-s)-1 but impacts of the latter are important for burial or removal of irradiation products. Downward transport of radiation produced oxidants and hydrocarbons could deliver significant chemical energy into the satellite interiors for astrobiological evolution in putative sub-surface oceans. © 2001 Academic Press.
KW - Callisto
KW - Europa
KW - Ganymede
KW - Radiation chemistry
KW - Satellite
KW - Surfaces
SN - 00191035 (ISSN)
LA - English
J2 - Icarus
M3 - Article
DB - Scopus
N1 - Export Date: 23 February 2023; Cited By: 309; CODEN: ICRSA
ER -

TY - JOUR
AU - Wilson, T.L.
TI - The search for extraterrestrial intelligence
PY - 2001
T2 - Nature
VL - 409
IS - 6823
SP - 1110
EP - 1114
DO - 10.1038/35059235
UR - https://www.scopus.com/inward/record.uri?eid=2-s2.0-0035931875&doi=10.1038%2f35059235&partnerID=40&md5=39cbca968a71a0c47753a34eddca6321
AD - Max-Planck-Inst. fur Radioastronomie, D 53121 Bonn, Auf dem Huegel 69, Germany
AB - As far as we know, humanity is alone in the Universe there is no definite evidence for the existence of extraterrestrial life, let alone extraterrestrial civilizations (ETCs) capable of communicating or travelling over interstellar distances. Yet popular speculation about the existence of ETCs abounds, including reports of alien visitations either now or in the past. But there is a middle way. It is now possible to put limits on the existence of ETCs of varying capabilities, within arbitrary distances from the Solar System, and conceive of real-world strategies whereby we might communicate with ETCs, or they with us.
KW - Exobiology
KW - Extraterrestrial Environment
KW - Radio Waves
KW - astronomy
KW - cosmos
KW - intelligence
KW - life

KW - microwave radiation
KW - priority journal
KW - radiofrequency radiation
KW - review
KW - telecommunication
SN - 00280836 (ISSN)
C2 - 11234025
LA - English
J2 - Nature
M3 - Review
DB - Scopus
N1 - Export Date: 23 February 2023; Cited By: 14; Correspondence Address: T.L. Wilson; Max-Planck-Inst. fur Radioastronomie, D 53121 Bonn, Auf dem Huegel 69, Germany; email: twilson@as.arizona.edu; CODEN: NATUA
ER -

TY - JOUR
AU - Cabrol, N.A.
AU - Wynn-Williams, D.D.
AU - Crawford, D.A.
AU - Grin, E.A.
TI - Recent aqueous environments in martian impact craters: An astrological perspective
PY - 2001
T2 - Icarus
VL - 154
IS - 1
SP - 98
EP - 112
DO - 10.1006/icar.2001.6661
UR - https://www.scopus.com/inward/record.uri?eid=2-s2.0-0035722245&doi=10.1006%2ficar.2001.6661&partnerID=40&md5=4ed33d275a70b1b1b0e4ed9c58204b1b
AD - SETI Institute, Mountain View, CA 94043, 2035 Landing Drive, United States
AD - Space Science Division, NASA Ames Research Center, MS 245-3, Moffett Field, CA 94035-1000, United States
AD - British Antarctic Survey, Natural Environment Research Council, Cambridge CB3 OET, High Cross, Madingley Road, United Kingdom
AD - Sandia National Laboratories, Albuquerque, NM 87185-0820, P.O. Box 5800, United States
AB - The discovery of gullies and debris aprons raises the question of the existence of aqueous environments on Mars in recent geological times and its astrobiological implications. Three cases of such environments are surveyed at MOC high resolution in the E-Gorgonum chaos and Newton and Hale craters. The regional setting of these craters suggests that the mechanisms of aquifer destabilization, flow discharge, and gully formation in these three cases result from local geological triggers that can include impact cratering, and tectonic processes, rather than climate or insolation factor. We take as a working hypothesis that microbial life appeared on Mars in ancient geological times, probably in a geothermal

environment but potentially evolving via infrared detection systems to give photosynthetic communities under the selective pressure of energetic solar radiation. We hypothesize that some microbial communities could have survived underground in either dormant or active state, or that their biomolecules could be preserved either frozen or desiccated in the subsurface beneath the upper oxidized zone. We assess the known environmental constraints for life and what type of potential habitats are provided in these three craters by aquifer discharges using comparison with terrestrial analogues and their associated microbial communities. These environments include: (1) the release of water on a dry crater floor in E-Gorgonum and the possibility for microorganisms and preserved biomolecules to be flushed out and mixed in with the sediment exposed at the surface; (2) the evidence of a recent lacustrine episode in the Newton crater with analogy to Antarctic Dry Valley lakes; (3) the exposure on the floor of Hale crater of material from a regional subsurface that is likely to have retained traces of one of the oldest martian bodies of water recognized to date (Parker et al. 2000) in the Argyre basin. We show how the water in Argyre (~3.8 billion years ago (Gya) was likely to have been alterated by hydrothermal processes and how the subsequent formation of the 150-km Hale crater on the northern ring of Argyre generated hydrothermal pumping. This accounts for the anomalously high location of the springs on the crater crests today with respect to the rest of the regional subsurface distribution. Finally, we envision current impact cratering as a factor for destabilizing aquifers on Mars today, thus creating new environments. We analyze the implications of impacts for two geological types of rock units that could harbor traces of life. As a result, we compare the potential of astrobiological exploration of crater floors, rims, and ejecta on future missions to Mars. © 2001 Elsevier Science.
KW - Climate
KW - Exobiology
KW - Mars
KW - Surface
PB - Academic Press Inc.
SN - 00191035 (ISSN)
LA - English
J2 - Icarus
M3 - Article
DB - Scopus
N1 - Export Date: 23 February 2023; Cited By: 65; Correspondence Address: N.A. Cabrol; SETI Institute, Mountain View, CA 94043, 2035 Landing Drive, United States; email: ncabrol@mail.arc.nasa.gov; CODEN: ICRSA
ER -

TY - CONF
AU - Tarter, J.
TI - What is SETI?
PY - 2001
T2 - Annals of the New York Academy of Sciences
VL - 950
SP - 269

EP - 275
DO - 10.1111/j.1749-6632.2001.tb02144.x
UR - https://www.scopus.com/inward/record.uri?eid=2-s2.0-0035687393&doi=10.1111%2fj.1749-6632.2001.tb02144.x&partnerID=40&md5=680a946871d990874f2ab340ed70d2d7
AD - SETI Institute, Mountain View, CA 94043, United States
AD - Project Phoenix, SETI Institute, Mountain View, CA 94043, 2035 Landings Drive, United States
AB - This paper describes the efforts of SETI that are going on around the world. It presents a brief rationale for the choice of methodology and the limits that can be set on the basis of such results to date.
KW - artificial intelligence
KW - astronomy
KW - biology
KW - conference paper
KW - cosmos
KW - life
KW - methodology
KW - research
KW - technology
KW - Astrobiology
KW - Exobiology
KW - Search for intelligent life
PB - New York Academy of Sciences
SN - 00778923 (ISSN)
C2 - 11797755
LA - English
J2 - Ann. New York Acad. Sci.
M3 - Conference paper
DB - Scopus
N1 - Export Date: 23 February 2023; Cited By: 3; CODEN: ANYAA
ER -

TY - CONF
AU - Hode, T.
AU - Wallinder, F.
TI - The Swedish Astrobiology Network (SWAN)
PY - 2002
T2 - European Space Agency, (Special Publication) ESA SP
IS - 518
SP - 515
EP - 516
UR - https://www.scopus.com/inward/record.uri?eid=2-s2.0-0036766636&partnerID=40&md5=22d9ed85273b5d9709b91f7678720fd1
AD - Swedish Museum of Natural History, Department of Paleozoology, Box 50007, SE-104 05 Stockholm, Sweden
AD - Örebro University, Department of Natural Sciences, SE-701 82 Örebro, Sweden
AB - The Swedish Astrobiology Network (SWAN) was founded in 2001. The members are Swedish scientists with an interest in astrobiology, and come from disciplines such as astronomy, molecular biology, geology and theoretical philosophy. The members investigate subjects such as the deep biosphere, extremophiles, prebiotic chemistry and

origin of life, planet and star formation in connection with astrochemistry. The network is supported by various organizations, but has no funding opportunities at present. The aim of the network is to promote astrobiology through interdisciplinary meetings and conferences, cooperative projects, interaction with sponsor agencies and spreading information to the general public. The network web address is www.astrobiologi.nu.
KW - Education
KW - Geology
KW - Molecular biology
KW - World Wide Web
KW - Astrobiology
KW - Astronomy
SN - 03796566 (ISSN)
LA - English
J2 - Eur Space Agency Spec Publ ESA SP
M3 - Conference paper
DB - Scopus
N1 - Export Date: 23 February 2023; Cited By: 0; Correspondence Address: T. Hode; Swedish Museum of Natural History, Department of Paleozoology, Box 50007, SE-104 05 Stockholm, Sweden; email: tomas.hode@nrm.se; CODEN: ESPUD
ER -

TY - CONF
AU - Gorshkov, A.I.
AU - Sivtsov, A.V.
AU - Sheglova, I.K.
AU - Balashova, V.V.
TI - Biogenic marine bacteria of siderococcus genus from paleontological and astrobiological point of view
PY - 2002
T2 - Proceedings of SPIE - The International Society for Optical Engineering
VL - 4939
SP - 224
EP - 226
DO - 10.1117/12.486684
UR - https://www.scopus.com/inward/record.uri?eid=2-s2.0-0038175173&doi=10.1117%2f12.486684&partnerID=40&md5=2cdc33d67b44f74442090732584bee6c
AD - IGMOD, RAS, Moscow, Russian Federation
AD - Institute of Chemistry, RAS, Far-Eastern Division, Moscow, Russian Federation
AD - Institute of Microbiology, RAS, Moscow, Russian Federation
AB - Biogenic mineral formation in the culture of marine psychrophilic ferrioxidizing bacteria of Siderococcus genus is studied in this work. During this process, hematite was formed through ferrihydride. The method of microdiffraction of mineral's electrons on ultrafine cuts showed hematite sedimentation on the surface of the cells deprived of cell walls. Fine homogeneity of this mineral is vindicated. The laboratory model of evolutionary ancient marine bacteria Siderococcus was used to show the formation of hypergene oxides: protoferrihydride --- ferrihydride ---

hematite. Microdiffractional analysis of biogenic material, which was taken from technogene zone, gave additional characteristic of the ecosystem through the detection of hematite mineral modification, which was formed by ingress of seawater Pb ions into the structure of the mineral.
KW - Astrophysics
KW - Cell culture
KW - Ecosystems
KW - Electron microscopy
KW - Marine biology
KW - Mineralogy
KW - Oxides
KW - Sedimentation
KW - Biogenic marine bacteria
KW - Ferribacteria
KW - Hematite
KW - Microdiffraction
KW - Paleontology
KW - Siderococcus genus
KW - Bacteria
KW - Biogenic mineral formation
KW - Ferribacteria
KW - Hematite
KW - Microdiffraction
KW - Siderococcus
SN - 0277786X (ISSN)
LA - English
J2 - Proc SPIE Int Soc Opt Eng
M3 - Conference paper
DB - Scopus
N1 - Export Date: 23 February 2023; Cited By: 0; CODEN: PSISD
ER -

TY - JOUR
AU - Trevors, J.T.
TI - The subsurface origin of microbial life on the Earth
PY - 2002
T2 - Research in Microbiology
VL - 153
IS - 8
SP - 487
EP - 491
DO - 10.1016/S0923-2508(02)01360-8
UR - https://www.scopus.com/inward/record.uri?eid=2-s2.0-0036031963&doi=10.1016%2fS0923-2508%2802%2901360-8&partnerID=40&md5=2d472329fa54fe0abab88aba166918ff
AD - Laboratory of Microbial Technology, Department of Environmental Biology, University of Guelph, Guelph, Ont. N1G 2W1, Canada
AB - Life on Earth can be divided into life on the surface made possible by photosynthesis and subsurface life with chemical energy as the driving force. An understanding of both environments is central to our understanding of the origin of life, the search for novel microbial species in the subsurface and for extraterrestrial

life or life signatures. In this manuscript, the surface and subsurface worlds are examined with a focus on the origin or assembly of bacterial life. © 2002 Éditions scientifiques et médicales Elsevier SAS. All rights reserved.
KW - Bacteria
KW - Biogenesis
KW - Earth (Planet)
KW - Energy Metabolism
KW - Evolution, Chemical
KW - Heat
KW - Thermodynamics
KW - Bacteria (microorganisms)
KW - astronomy
KW - bacterium
KW - biogenesis
KW - energy
KW - microorganism
KW - photosynthesis
KW - priority journal
KW - short survey
KW - surface property
KW - Assembly
KW - Bacteria
KW - Biosphere
KW - Earth
KW - Evolution
KW - Heat
KW - Life
KW - Origin
KW - Subsurface
KW - Surface
SN - 09232508 (ISSN)
C2 - 12437209
LA - English
J2 - Res. Microbiol.
M3 - Short survey
DB - Scopus
N1 - Export Date: 23 February 2023; Cited By: 22; Correspondence Address: J.T. Trevors; Laboratory of Microbial Technology, Department of Environmental Biology, University of Guelph, Guelph, Ont. N1G 2W1, Canada; email: jtrevors@uoguelph.ca; CODEN: RMCRE
ER -

TY - JOUR
AU - Woolf, N.J.
AU - Smith, P.S.
AU - Traub, W.A.
AU - Jucks, K.W.
TI - The spectrum of earthshine: A pale blue dot observed from the ground
PY - 2002
T2 - Astrophysical Journal
VL - 574
IS - 1 I

SP - 430
EP - 433
DO - 10.1086/340929
UR - https://www.scopus.com/inward/record.uri?eid=2-s2.0-0000549853&doi=10.1086%2f340929&partnerID=40&md5=fd90d064be282b484bfd9a1a76a0444c
AD - Steward Observatory, University of Arizona, Tucson, AZ 85721, United States
AD - Harvard-Smithsonian Ctr. Astrophys., Cambridge, MA 02138, 60 Garden Street, United States
AB - We report the visible reflection spectrum of the integrated Earth, illuminated as it would be seen as a spatially unresolved extrasolar planet. The spectrum was derived from observation of lunar earthshine in the range 4800-9200 Å at a spectral resolution of about 600. We observe absorption features of ozone, molecular oxygen, and water. We see enhanced reflectivity at short wavelengths from Rayleigh scattering and apparently negligible contributions from aerosol and ocean water scattering. We also see enhanced reflectivity at long wavelengths starting at about 7300 Å, corresponding to the well-known red reflectivity edge of vegetation because of its chlorophyll content; however, this signal is not conclusive because of the breakdown of our simple model at wavelengths beyond 7900 Å.
KW - Astrobiology
KW - Earth
KW - Moon
KW - Planetary systems
PB - Institute of Physics Publishing
SN - 0004637X (ISSN)
LA - English
J2 - Astrophys. J.
M3 - Article
DB - Scopus
N1 - Export Date: 23 February 2023; Cited By: 153; Correspondence Address: N.J. Woolf; Steward Observatory, University of Arizona, Tucson, AZ 85721, United States; email: nwoolf@as.arizona.edu
ER -

TY - JOUR
AU - Kounaves, S.P.
AU - Noll, R.A.
AU - Buehler, M.G.
AU - Hecht, M.H.
AU - Lankford, K.
AU - West, S.J.
TI - Microbial life detection with minimal assumptions
PY - 2002
T2 - Proceedings of SPIE - The International Society for Optical Engineering
VL - 4495
SP - 137
EP - 144
DO - 10.1117/12.454772
UR - https://www.scopus.com/inward/record.uri?eid=2-

s2.0-0036396815&doi=10.1117%2f12.454772&partnerID=40&md5=8f3b55a88e04d47683507b0b141db65d
AD - Department of Chemistry, Tufts University, Medford, MA, 02155, United States
AD - Jet Propulsion Laboratory, Pasadena, CA, 91109, 4800 Oak Drive, United States
AD - Starsys Research, Boulder, CO, 80301, Tufts University, United States
AD - Thermo Orion, Beverly, MA, 01915, 500 Cummings Center, United States
AB - To produce definitive and unambiguous results, any life detection experiment must make minimal assumptions about the nature of extraterrestrial life. The only criteria that fits this definition is the ability to reproduce and in the process create a disequilibrium in the chemical and redox environment. The Life Detection Array (LIDA), an instrument proposed for the 2007 NASA Mars Scout Mission, and in the future for the Jovian moons, enables such an experiment. LIDA responds to minute biogenic chemical and physical changes in two identical "growth" chambers. The sensitivity is provided by two differentially monitored electrochemical sensor arrays. Growth in one of the chambers alters the chemistry and ionic properties and results in a signal. This life detection system makes minimal assumptions; that after addition of water the microorganism replicates and in the process will produce small changes in its immediate surroundings by consuming, metabolizing, and excreting a number of molecules and/or ionic species. The experiment begins by placing an homogenized split-sample of soil or water into each chamber, adding water if soil, sterilizing via high temperature, and equilibrating. In the absence of any microorganism in either chamber, no signal will be detected. The "inoculation" of one chamber with even a few microorganisms which reproduce, will create a sufficient disequilibrium in the system (compared to the control) to be detectable. Replication of the experiment and positive results would lead to a definitive conclusion of biologically induced changes. The split sample and the "nanogram" inoculation eliminates chemistry as a causal agent.
KW - Atmospheric temperature
KW - Biology
KW - Microorganisms
KW - Planets
KW - Redox reactions
KW - Space research
KW - Astrobiology
KW - Microbial life detection
KW - Astrophysics
KW - Astrobiology
KW - Life detection
KW - Mars
KW - Microbial-growth
SN - 0277786X (ISSN)
LA - English
J2 - Proc SPIE Int Soc Opt Eng
M3 - Article
DB - Scopus

N1 - Export Date: 23 February 2023; Cited By: 9
ER -

TY - JOUR
AU - Fike, D.A.
AU - Cockell, C.
AU - Pearce, D.
AU - Lee, P.
TI - Heterotrophic microbial colonization of the interior of impact-shocked rocks from Haughton impact structure, Devon Island, Nunavut, Canadian High Arctic
PY - 2002
T2 - International Journal of Astrobiology
VL - 1
IS - 4
SP - 311
EP - 323
DO - 10.1017/S1473550403001290
UR - https://www.scopus.com/inward/record.uri?eid=2-s2.0-85023152373&doi=10.1017%2fS1473550403001290&partnerID=40&md5=605001981c702d65a811f4716678adbe
AD - Department of Earth Atmospheric, Department of Planetary Sciences, Massachusetts Institute of Technology, 54-812, Cambridge, MA 01239, 77 Massachusetts Ave., United States
AD - British Antarctic Survey, Cambridge CB3 0ET, High Cross, Madingley Road, United Kingdom
AD - SETI Institute, NASA Ames Research Center, Moffett Field, CA 94035-1000, United States
AB - The polar desert is one of the most extreme environments on Earth. Endolithic organisms can escape or mitigate the hazards of the polar desert by using the resources available in the interior of rocks. We examined endolithic communities within crystalline rocks that have undergone shock metamorphism as a result of an asteroid or comet impact. Specifically, we present a characterization of the heterotrophic endolithic community and its environment in the interior of impact-shocked gneisses and their host polymict breccia from the Haughton impact structure on Devon Island, Nunavut, Canadian High Arctic. Microbiological colonization of impact-shocked rocks is facilitated by impact-induced fissures and cavities, which occur throughout the samples, the walls of which are lined with high abundances of biologically important elements owing to the partial volatilization of minerals within the rock during the impact. 27 heterotrophic bacteria were isolated from these shocked rocks and were identified by 16S rDNA sequencing. The isolates from the shocked gneiss and the host breccia are similar to each other, and to other heterotrophic communities isolated from polar environments, suggesting that the interiors of the rocks are colonized by microorganisms from the surrounding country rocks and soils. Inductively coupled plasma–atomic emission spectroscopy (ICP-AES), scanning electron microscopy (SEM) and energy dispersive X-ray (EDX) analysis were used to identify the chemical composition of the shocked materials and to document the in situ growth of microbes in their interiors. The identification of these heterotrophic communities within impact-shocked crystalline rocks extends our

knowledge of the habitable biosphere on Earth. The colonization of the interiors of these samples has astrobiological applications both for considering terrestrial, microbiological contamination of meteorites from the Antarctic ice sheet and for investigating possible habitats for microbial organisms on the early Earth, and more speculatively, on Mars and other planetary bodies. © 2002, Cambridge University Press. All rights reserved.
KW - endolith
KW - Haughton impact structure
KW - microbial colonization
KW - shock-metamorphism
KW - shocked gneiss
SN - 14735504 (ISSN)
LA - English
J2 - Int. J. Astrobiology
M3 - Article
DB - Scopus
N1 - Export Date: 23 February 2023; Cited By: 19
ER -

TY - CONF
AU - Pikuta, E.V.
AU - Hoover, R.B.
TI - Psychrophiles and astrobiology: Microbial life of frozen worlds
PY - 2002
T2 - Proceedings of SPIE - The International Society for Optical Engineering
VL - 4939
SP - 103
EP - 116
DO - 10.1117/12.501866
UR - https://www.scopus.com/inward/record.uri?eid=2-s2.0-0037837530&doi=10.1117%2f12.501866&partnerID=40&md5=a2bfa1daeff972129548182af87631ef
AD - Astrobiology Group, Ctr. of Space Plasma Aero. Res./UAH, Huntsville, AL 35899, United States
AD - Space Sciences Directorate, NASA - Marshall Space Flight Center, Huntsville, AL 35812, United States
AB - The Astrobiological significance of the Fox Tunnel with its rich assemblage of frozen microbes was considered as proxy for developing techniques that may help optimize the search for evidence of life in the permafrost of Mars. Images of a novel anaerobic, heterotrophic, psychotrophic bacterium (str.FTR1) isolated in pure culture from the Fox Tunnel were provided. Novel psychotrophs isolated from guano of the Magellanic penguin (Spheniscus magellanicus) from the southern tip of Patagonia were considered as these strains (PmaG1 and PPP2) represent new species and genera of anaerobic microbes that grow at very low temperatures. The lowest limit for growth without morphological changes of str. PmaG1 is -4°C.
KW - Atmospheric temperature
KW - Bacteria
KW - Glaciers

KW - Interfaces (materials)
KW - Microstructure
KW - Permafrost
KW - Sea ice
KW - Sediments
KW - Transmission electron microscopy
KW - Astrobiology
KW - Frozen World
KW - Polar ice sheet
KW - Psychrophiles
KW - Astrophysics
KW - Anaerobes
KW - Astrobiology
KW - Fox permafrost tunnel
KW - Mars
KW - Microbial extremephiles
KW - Psychrophiles
KW - Psychrotrophs
SN - 0277786X (ISSN)
LA - English
J2 - Proc SPIE Int Soc Opt Eng
M3 - Conference paper
DB - Scopus
N1 - Export Date: 23 February 2023; Cited By: 14; CODEN: PSISD
ER -

TY - CONF
AU - Beauchamp, P.
AU - Lunine, J.I.
AU - Welch, C.
TI - The potential for chemical evolution on Titan
PY - 2002
T2 - IEEE Aerospace Conference Proceedings
VL - 1
C7 - 1036853
SP - 337
EP - 347
DO - 10.1109/AERO.2002.1036853
UR - https://www.scopus.com/inward/record.uri?eid=2-s2.0-84879328185&doi=10.1109%2fAERO.2002.1036853&partnerID=40&md5=e956341c8bffa5e29e503b8551b2acc5
AD - Jet Propulsion Lab., California Inst. of Technol., Pasadena, CA 91109-8099, 4800 Oak Grove Drive, United States
AD - Lunar and Planetary Laboratory, University of Arizona, Tucson, AZ 85721-0092, 1629 E. University Blvd., United States
AD - Merck and Company, Inc., Whitehouse Station, NJ 08889-0100, One Merck Drive, United States
AB - Saturn's moon, Titan, is a Mercury-sized body that is rich in organics and water ice. The Cassini-Huygens mission will arrive at Saturn in July 2004 and deploy the European Space Agency (ESA) Huygens probe, which will descend to the surface, providing in situ chemical analysis, images, spectra and other data that will characterize in detail the atmosphere and, if it survives, a region of the surface. The Saturn Orbiter instruments will map Titan's

surface at a range of wavelengths over 4 years and approximately 45 flybys. Radar and near-IR mapping will distinguish between areas of water ice, ammonia-water, and organics, as well as provide perhaps sufficient data to distinguish liquid organics from solid organics. We then need to be prepared to examine what scientific questions would follow from these results and what subsequent mission will enable a more complete picture of Titan to be developed. We propose that Titan is an exciting astrobiological target. If the Cassini/ Huygen's data show that there is no variation in the composition of surface organics, then atmospheric photochemistry has dominated the production of organics with little further evolution. However, if there is variation from region to region, this might suggest that additional organic chemistry has acted on the products of stratospheric photochemistry. Should evidence for variability in the organic phases on Titan be found, subsequent exploration of the surface ought to be undertaken, with an eye toward understanding the extent to which organic chemistry has proceeded. Sampling of organics to determine oxygen content, extent of acetylene polymerization, existence of chiral molecules and enantiomeric excesses, and searches for specific polymer products, would be of interest in assessing how organic chemistry evolves toward biochemistry. Such efforts would require fairly sophisticated chemical analyses from landed missions. This paper examines this chemistry and the potential instruments that could distinguish chemical evolution. © 2002 IEEE.
KW - Chemical analysis
KW - Interplanetary flight
KW - Photochemical reactions
KW - Atmospheric photochemistry
KW - Cassini-Huygens mission
KW - Chemical evolution
KW - Enantiomeric excess
KW - European Space Agency
KW - Organic Chemistry
KW - Situ chemical analysis
KW - Stratospheric photochemistry
KW - Stereochemistry
SN - 1095323X (ISSN); 078037231X (ISBN); 978-078037231-3 (ISBN)
LA - English
J2 - IEEE Aerosp. Conf. Proc.
M3 - Conference paper
DB - Scopus
N1 - Export Date: 23 February 2023; Cited By: 0
ER -

TY - JOUR
AU - Schulze-Makuch, D.
AU - Irwin, L.N.
TI - Reassessing the possibility of life on venus: Proposal for an astrobiology mission
PY - 2002
T2 - Astrobiology
VL - 2
IS - 2

SP - 197
EP - 202
DO - 10.1089/15311070260192264
UR - https://www.scopus.com/inward/record.uri?eid=2-s2.0-0036616686&doi=10.1089%2f15311070260192264&partnerID=40&md5=48777163f65c2a16c27d6da2f45f12e6
AD - Department of Geological Sciences, University of Texas at El Paso, El Paso, TX 79968-0555, 500 West University Avenue, United States
AD - Department of Biological Sciences, University of Texas at El Paso, El Paso, TX, United States
AB - With their similar size, chemical composition, and distance from the Sun, Venus and Earth may have shared a similar early history. Though surface conditions on Venus are now too extreme for life as we know it, it likely had abundant water and favorable conditions for life when the Sun was fainter early in the Solar System. Given the persistence of life under stabilizing selection in static environments, it is possible that life could exist in restricted environmental niches, where it may have retreated after conditions on the surface became untenable. High-pressure subsurface habitats with water in the supercritical liquid state could be a potential refugium, as could be the zone of dense cloud cover where thermoacidophilic life might have retreated. Technology based on the Stardust Mission to collect comet particles could readily be adapted for a pass through the appropriate cloud layer for sample collection and return to Earth. © Mary Ann Liebert, Inc.
KW - Biogenesis
KW - Earth (Planet)
KW - Exobiology
KW - Solar System
KW - Space Flight
KW - Venus
KW - Water
KW - water
KW - article
KW - astronomy
KW - biogenesis
KW - biology
KW - space flight
KW - Cloud particle
KW - Extreme environment
KW - Life
KW - Mission
KW - Supercritical water
KW - Venus
SN - 15311074 (ISSN)
C2 - 12469368
LA - English
J2 - Astrobiology
M3 - Article
DB - Scopus
N1 - Export Date: 23 February 2023; Cited By: 66; Correspondence Address: D. Schulze-Makuch; Department of Geological Sciences, University of Texas at El Paso, El Paso, TX 79968-0555, 500 West

University Avenue, United States; email: dirksm@geo.utep.edu
ER -

TY - CONF
AU - Sadeh, E.
TI - Space and the extraterrestrial environment
PY - 2002
T2 - Space 2002 and Robotics 2002
SP - 571
EP - 579
DO - 10.1061/40625(203)71
UR - https://www.scopus.com/inward/record.uri?eid=2-s2.0-0036991891&doi=10.1061%2f40625%28203%2971&partnerID=40&md5=f4c6c6bef5bb14ff8cb755deba31bdea
AD - Department of Space Studies, Odegard School of Aerospace Sciences, University of North Dakota, Grand Forks, ND 58202-9008, United States
AB - As humankind proceeds with space exploration, colonization, and development of space activities in the 21st Century, the relationship between space and the extraterrestrial environment emerges as a crucial area. A number of questions in this regard need to be considered and addressed: do humans have a right to extract resources from space and planetary bodies, to create human supported biospheres in space and on planetary surfaces, and to terraform celestial bodes inhospitable to "human" life; should space be protected to safeguard human ends such as scientific inquiry; does humanity have a direct obligation to the welfare of the space environment; do the needs of extraterrestrial life forms (even if only microorganisms) take precedence over (or require protection from) life from Earth; do humans need to protect indigenous life forms from "Earth" life forms that may cause harmful effects; what is the human responsibility to preserve the worlds of the Solar System in their wilderness states for present and future generations; and what are the guidelines for space exploration activities on planetary bodies to keep potential environmental impacts to a minimum. In addressing the issues raised by these questions, this paper identifies and discusses three environmental ethical framework: (1) anthropocentrism; (2) biocentrism; and (3) cosmocentrism. These frameworks serve as a roadmap for the range of space exploration polices that can be directed to spread life, and search for life, in the Solar System in an environmentally responsible fashion.
KW - Earth sciences
KW - Environmental engineering
KW - Environmental impact
KW - Extraterrestrial communication links
KW - Research and development management
KW - Space applications
KW - Extraterrestrial environment
KW - Extraterrestrial life forms
KW - Space colonization
KW - Space exploration
KW - Civil engineering
A2 - Laubscher B.E.

A2 - Johnson S.W.
A2 - Moskowitz S.E.
A2 - Richter P.
A2 - Klingler D.
PB - ASCE - American Society of Civil Engineers
SN - 0784406251 (ISBN); 978-078440625-0 (ISBN)
LA - English
J2 - Space 2002 and Robotics 2002
M3 - Conference paper
DB - Scopus
N1 - Export Date: 23 February 2023; Cited By: 0; Correspondence Address: E. Sadeh; Department of Space Studies, Odegard School of Aerospace Sciences, University of North Dakota, Grand Forks, ND 58202-9008, United States; email: sadeh@space.edu
ER -

TY - JOUR
AU - Chapelle, F.H.
AU - O'Neill, K.
AU - Bradley, P.M.
AU - Methá, B.A.
AU - Ciufo, S.A.
AU - Knobel, L.L.
AU - Lovley, D.R.
TI - A hydrogen-based subsurface microbial community dominated by methanogens
PY - 2002
T2 - Nature
VL - 415
IS - 6869
SP - 312
EP - 315
DO - 10.1038/415312a
UR - https://www.scopus.com/inward/record.uri?eid=2-s2.0-0037122793&doi=10.1038%2f415312a&partnerID=40&md5=4683cc7e80d21bb53dde8035866366d1
AD - US Geological Survey, Columbia, SC 29210, United States
AD - Department of Microbiology, Uiversity of Massachusetts, Amherst, MA 01003, United States
AD - US Geological Survey, Idaho Falls, Idaho 83402, United States
AB - The search for extraterrestrial life may be facilitated if ecosystems can be found on Earth that exist under conditions analogous to those present on other planets or moons. It has been proposed, on the basis of geochemical and thermodynamic considerations, that geologically derived hydrogen might support subsurface microbial communities on Mars and Europa in which methanogens form the base of the ecosystem1-5. Here we describe a unique subsurface microbial community in which hydrogen-consuming, methane-producing Archaea far outnumber the Bacteria. More than 90% of the 16s ribosomal DNA sequences recovered from hydrothermal waters circulating through deeply buried igneous rocks in Idaho are related to hydrogen-using methanogenic microorganisms. Geochemical characterization indicates that geothermal hydrogen, not organic carbon, is the primary energy source for this methanogen-dominated

microbial community. These results demonstrate that hydrogen-based methanogenic communities do occur in Earth's subsurface, providing an analogue for possible subsurface microbial ecosystems on other planets.
KW - Bacteria
KW - DNA, Archaeal
KW - DNA, Bacterial
KW - Ecosystem
KW - Euryarchaeota
KW - Exobiology
KW - Hydrogen
KW - Molecular Sequence Data
KW - Phylogeny
KW - RNA, Ribosomal, 16S
KW - Water Microbiology
KW - United States
KW - Archaea
KW - Bacteria (microorganisms)
KW - Methanobacterium
KW - DNA sequences
KW - Ecosystems
KW - Geology
KW - Hydrogen
KW - Igneous rocks
KW - Solar system
KW - DNA 16S
KW - hydrogen
KW - ribosome DNA
KW - early Earth
KW - extraterrestrial matter
KW - geothermal system
KW - hydrogen
KW - hydrothermal fluid
KW - methanogenic bacterium
KW - microbial community
KW - Archaebacterium
KW - article
KW - astronomy
KW - ecosystem
KW - energy resource
KW - geochemistry
KW - geology
KW - Methanobacterium
KW - microorganism
KW - nonhuman
KW - priority journal
KW - thermodynamics
KW - Hydrothermal waters
KW - Subsurface microbial communities
KW - Methanogens
SN - 00280836 (ISSN)
C2 - 11797006
LA - English
J2 - Nature

M3 - Article
DB - Scopus
N1 - Export Date: 23 February 2023; Cited By: 356
ER -

TY - JOUR
AU - Ellery, A.A.
AU - Cockell, C.S.
AU - Edwards, H.G.M.
AU - Dickensheets, D.L.
AU - Welch, C.S.
TI - Vanguard – a proposed European astrobiology experiment on Mars
PY - 2002
T2 - International Journal of Astrobiology
VL - 1
IS - 3
SP - 191
EP - 199
DO - 10.1017/S1473550402001155
UR - https://www.scopus.com/inward/record.uri?eid=2-s2.0-0038443561&doi=10.1017%2fS1473550402001155&partnerID=40&md5=defc7cbe35917c7f4806c1a60053fb8c
AD - School of Engineering, Kingston University, London, United Kingdom
AD - British Antarctic Survey, High Cross, Cambridge, United Kingdom
AD - Department of Chemical & Forensic Sciences, University of Bradford, Bradford, United Kingdom
AD - Department of Electrical and Computer Engineering, Montana State University, Bozeman, Montana, United States
AB - We propose a new type of robotic mission for the exploration of Mars. This mission is called Vanguard and represents the fruits of a collaboration that is both international and multi-disciplinary. Vanguard is designed for sub-surface penetration and investigation using remote instruments and unlike previous robotic architectures it offers the opportunity for multiple subsurface site analysis using three moles. The moles increase the probability that a subsurface signature of life can be found and by accomplishing subsurface analysis across a transect, the statistical rigour of Martian scientific exploration would be improved. There is no provision for returning samples to the surface for analysis by a gas-chromatograph/mass-spectrometer (GCMS) – this minimizes the complexity invoked by sophisticated robotic overheads. The primary scientific instruments to be deployed are the Raman spectrometer, infrared spectrometer and laser-induced breakdown spectroscope – the Raman spectrometer in particular is discussed. We concentrate primarily on the scientific rationale for the Vanguard mission proposal. The Vanguard mission proposal represents a logical opportunity for extending European robotic missions to Mars. © 2002, Cambridge University Press. All rights reserved.
KW - astrobiology
KW - experimental astrobiology
KW - Mars
KW - robotic Mars mission

SN - 14735504 (ISSN)
LA - English
J2 - Int. J. Astrobiology
M3 - Article
DB - Scopus
N1 - Export Date: 23 February 2023; Cited By: 10
ER -

TY - CONF
AU - Hjalmarson, A.
TI - Odin observations of H2O and O2 in comets and interstellar clouds
PY - 2002
T2 - European Space Agency, (Special Publication) ESA SP
IS - 518
SP - 41
EP - 43
UR - https://www.scopus.com/inward/record.uri?eid=2-s2.0-0036766395&partnerID=40&md5=c4a14f8f471d413d586d25e7e219ac4c
AD - Ctr. for Astrophys. and Space Sci., Chalmers University of Technology, Onsala Space Observatory, SE-439 92 Onsala, Sweden
AB - We here report on results from single-position observations, and in some cases also mapping, of the 557 GHz ortho-H2O line in several comets and in many interstellar molecular clouds by the Odin sub-millimetre wave spectroscopy satellite. The H2O production rates have been accurately determined in four comets, C/2001 A2 (LINEAR), 19P/Borrelly, C/2000 WM, (LINEAR), and 153P/ 2002 Cl (Ikeya-Zhang). In comet Ikeya-Zhang our detection at a low level of the corresponding H218O emission line verifies the H2O production rate (which depends upon the assumed radiative and collisional excitation and also upon radiative transfer modelling) and is consistent with a nearly terrestrial 16O/18O-isotope ratio. In an astro-biological context, the cometary H2O production rates are especially important as reference levels for comparison with abundances of other molecules simultaneously observed with ground-based telescopes. In interstellar clouds the observed gas-phase H2O abundances (vs H2) range from 5 × 10-4 in the Orion KL outflow/shock region (where essentially all oxygen is locked up in H2O) to circa 10-8 in quiescent cloud regions (where H2O is just one of many trace molecules). From an astrobiological point of view, the molecular abundances in star forming clouds are important in terms of initial conditions for the chemistry in proto-planetary disks ("proto-solar nebulae"), the formation sites of new planetary systems. In simultaneous observations, Odin has also detected the 572 GHz ortho-NH3 line in cold and warm clouds as well as in the Orion outflow and Bar/PDR regions (an area of increased ionization caused by the intense UV flux from newly born massive stars). In other simultaneous observations, we have performed sensitive searches for O2 at 119 GHz. Although no detection can be reported as yet, the resulting very low abundance limits < 10-7) are very intriguing when they are compared with current "standard" model expectations, which fall in the range 10-5-10-4.
KW - Ammonia
KW - Clouds

KW - Frequencies
KW - Oxygen
KW - Water
KW - Interstellar clouds
KW - Space research
SN - 03796566 (ISSN)
LA - English
J2 - Eur Space Agency Spec Publ ESA SP
M3 - Conference paper
DB - Scopus
N1 - Export Date: 23 February 2023; Cited By: 0; Correspondence Address: A. Hjalmarson; Ctr. for Astrophys. and Space Sci., Chalmers University of Technology, Onsala Space Observatory, SE-439 92 Onsala, Sweden; email: hjalmar@oso.chalmers.se; CODEN: ESPUD
ER -

TY - CONF
AU - Biele, J.
AU - Ulamec, S.
AU - Garry, J.
AU - Sheridan, S.
AU - Morse, A.D.
AU - Barber, S.
AU - Wright, I.
AU - Tüg, H.
AU - Mock, T.
TI - Melting probes at Lake Vostok and Europa
PY - 2002
T2 - European Space Agency, (Special Publication) ESA SP
IS - 518
SP - 253
EP - 260
UR - https://www.scopus.com/inward/record.uri?eid=2-s2.0-0036766396&partnerID=40&md5=75c0b468bbd9aa4740d46ccc4f3056e7
AD - DLR, RS/MUSC, D-51147 Cologne, Linder Höhe, Germany
AD - Open University, Milton Keynes MK7 6AA, United Kingdom
AD - Alfred-Wegener Inst. Polar/Mar. Res., D-27515 Bremerhaven, P.O. box 120161, Germany
AB - Signatures indicative of a subglacial ocean on the Jovian satellite Europa have been discovered by the Galileo spaceprobe. The structure and chemistry of this postulated ocean, have received considerable attention, and its exploration is seen as an important goal for the fields of comparative planetology and astrobiology. Terrestrial subglacial lakes (e.g., Lake Vostok) have been considered as analogues for several aspects of a Europan ocean and the means of exploring such an environment will be discussed. We review the concept of melting probes, i.e. small probes with a heated tip ("hot point"), that melt through the ice. Such devices can carry instruments for in-situ measurements, or act as sample-return probes by the new concept of melting "upwards" once the sampling in the liquid ocean has been performed. Experience with melting probes in Antarctica exists; a demonstration mission to, e.g., Lake Vostok is proposed, and a possible design for a mission to Europa is outlined. Although, for a Europan mission, a probe

capable of in-situ analysis might differ substantially from the terrestrial 'sample-return' version.
KW - Ice
KW - Melting
KW - Orbits
KW - Probes
KW - Melting probes
KW - Astrophysics
SN - 03796566 (ISSN)
LA - English
J2 - Eur Space Agency Spec Publ ESA SP
M3 - Conference paper
DB - Scopus
N1 - Export Date: 23 February 2023; Cited By: 9; Correspondence Address: J. Biele; DLR, RS/MUSC, D-51147 Cologne, Linder Höhe, Germany; email: jens.biele@dlr.de; CODEN: ESPUD
ER -

TY - CONF
AU - DiGregorio, B.E.
TI - Dissolution cavities in Upper Ordovician sandstones from Lake Ontario: Analogs to vesiculated rocks on Mars?
PY - 2002
T2 - Proceedings of SPIE - The International Society for Optical Engineering
VL - 4859
SP - 57
EP - 71
DO - 10.1117/12.457566
UR - https://www.scopus.com/inward/record.uri?eid=2-s2.0-0037515802&doi=10.1117%2f12.457566&partnerID=40&md5=126db46aa90e5d0b3246acb60d49bc84
AD - Cardiff Centre for Astrobiology, Cardiff University, Cardiff, United Kingdom
AD - Middleport, NY 14105, 16 North Hartland Street, United States
AB - Rocks imaged by NASA's Viking 2 lander in 1976 revealed ubiquitou "vesicles" that to this day remain enigmatic because the mineralogy of Martian rocks is not adequately analyzed to date. Neither a sedimentary nor a volcanic origin for the rock is firmly established. Furthermore, proposed theories on the evolution of the Utopia Basin near the Viking 2 landing site include an ancient shallow ocean and glacial scouring. This paper draws attention to the morphological similarities of biogenic disolution cavities in terrestrial sandstones and in the near-field rocks at the Viking 2 landing site on Mars.
KW - Astronomy
KW - Biology
KW - Glaciers
KW - Martian surface analysis
KW - Mineralogy
KW - Space research
KW - Astrobiology
KW - Carbonic acid
KW - Fossiliferous sandstones

KW - Fossilization
KW - Humic acids
KW - Karst
KW - Martian glaciers
KW - Martian rocks
KW - Sandstone
KW - Beagle 2
KW - Carbonic acid
KW - Dissolution cavities in caves
KW - Fossiliferous sandstone
KW - Fossilization
KW - Karst
KW - Lake Ontario
KW - Life on Mars
KW - Martian glaciers
KW - Martian ocean
KW - Ordovician
KW - Vesicles
KW - Viking lander 2 rock images
SN - 0277786X (ISSN)
LA - English
J2 - Proc SPIE Int Soc Opt Eng
M3 - Conference paper
DB - Scopus
N1 - Export Date: 23 February 2023; Cited By: 1; Correspondence Address: B.E. DiGregorio; Cardiff Centre for Astrobiology, Cardiff University, Cardiff, United Kingdom; email: Icamsr@buffnet.net; CODEN: PSISD
ER -

TY - JOUR
AU - Hoffman, N.
TI - Active polar gullies on Mars and the role of carbon dioxide.
PY - 2002
T2 - Astrobiology
VL - 2
IS - 3
SP - 313
EP - 323
DO - 10.1089/153110702762027899
UR - https://www.scopus.com/inward/record.uri?eid=2-s2.0-0038345093&doi=10.1089%2f153110702762027899&partnerID=40&md5=1be6b46694d24a27aedb35a7eaf083af
AD - School of Earth Sciences, University of Melbourne, Parkville, Australia
AB - The detection of geologically recent channels and gullies on Mars has been interpreted as evidence for recent water activity on this arid and cold planet. The presence of active water would have considerable implications for the history of Mars and for the potential for an active near-surface biota; however, water is not the only substance that can flow and may actually be one of the least likely candidates when the modern permafrost environment is considered. Here, a key site of recent gully and channel development on Mars is reported at 71 degrees S in Sisyphi Cavi, within the

annual zone of polar CO2 ice and snow accumulation. Superposition of channel features over and/or through the defrosting CO2 snowpack shows that the channels are active at the present day and probably have fluid flows every spring during the annual defrosting. In itself, this is a significant observation as active fluid flows of any nature have not yet been proven on Mars. However, the ambient temperature at the time of gully activity appears to require a role for CO2 in the formation of the channels, rather than water. A model is proposed for gas-lubricated flow in the channels based on avalanching of CO2 snowpack and clastic debris. If similar mechanisms are responsible for all the recent gullies on Mars, then perhaps no near-surface astrobiological targets are available.
KW - Carbon Dioxide
KW - Mars
KW - Models, Theoretical
KW - Water
KW - carbon dioxide
KW - water
KW - article
KW - astronomy
KW - theoretical model
SN - 15311074 (ISSN)
C2 - 12530241
LA - English
J2 - Astrobiology
M3 - Article
DB - Scopus
N1 - Export Date: 23 February 2023; Cited By: 60; Correspondence Address: N. Hoffman; email: nhoffman@unimelb.edu.au
ER -

TY - CONF
TI - Proceedings of the second European workshop on EXO - Astrobiology
PY - 2002
T2 - European Space Agency, (Special Publication) ESA SP
IS - 518
UR - https://www.scopus.com/inward/record.uri?eid=2-s2.0-0036766393&partnerID=40&md5=80d312c3312b3b234c45e6406a8590b4
AB - The proceedings contains 157 paper from the Conference on Proceedings of the Second European Workshop on Exo - Astrobiology. Topics discussed include: astrophysical and astrochemical insights into the origin of life; organic chemistry in meteorites; origin of planetary atmospheres and their role in the evolution of life; the prebiotic synthesis of amino acids and survivability and protection of bacterial spores.
KW - Amino acids
KW - Ammonia
KW - Astrophysics
KW - Carbon
KW - Clouds
KW - Earth (planet)
KW - Extraterrestrial atmospheres
KW - Galaxies

KW - Orbits
KW - Oxygen
KW - Phase equilibria
KW - Quantum theory
KW - Solar system
KW - Sugar (sucrose)
KW - Sulfur
KW - Water
KW - Allotropy
KW - Atmospheric mechanisms
KW - Carbonaceous chondrites
KW - EiRev
KW - Interstellar clouds
KW - Interstellar medium (ISM)
KW - Molecular clouds
KW - Planetary atmospheres
KW - Quantum fluids
KW - Space research
SN - 03796566 (ISSN)
LA - English
J2 - Eur Space Agency Spec Publ ESA SP
M3 - Conference review
DB - Scopus
N1 - Export Date: 23 February 2023; Cited By: 0; CODEN: ESPUD
ER -

TY - CONF
AU - Vishnivetskaya, T.A.
AU - Vorobyova, E.A.
AU - Gilichinsky, D.A.
TI - Viable green algae and cyanobacteria within terrestrial permafrost
PY - 2002
T2 - European Space Agency, (Special Publication) ESA SP
IS - 518
SP - 295
EP - 298
UR - https://www.scopus.com/inward/record.uri?eid=2-s2.0-0036766592&partnerID=40&md5=6dc00f533b8c47207bc5ac066879b49c
AD - Inst. Physicochem./Biol. P.S.S., Russian Academy of Sciences, 142290, Pushchino, Moscow Region, Russian Federation
AD - Moscow State University, Vorobyovy Gory 119899, Russian Federation
AB - Viable permafrost algae have preserved their morphological characteristics and photosynthetic apparatus for the longest documented period of time on Earth. Unicellular green algae of the order Chlorococcales and filamentous cyanobacteria of the orders Oscillatoriales and Nostocales were isolated from deep subsurface permafrost sediments. Both were discovered within Siberian permafrost from the Holocene up to the Early Pleistocene age. However, green algae were found to be predominant and also able to survive inside colder Antarctic sediments. These findings will have an important impact on astrobiology and the study of organisms that exist in extreme environments.

KW - Bacteria
KW - Biology
KW - Environmental impact
KW - Permafrost
KW - Photosynthesis
KW - Sediments
KW - Astrobiology
KW - Algae
SN - 03796566 (ISSN)
LA - English
J2 - Eur Space Agency Spec Publ ESA SP
M3 - Conference paper
DB - Scopus
N1 - Export Date: 23 February 2023; Cited By: 4; Correspondence Address: T.A. Vishnivetskaya; Inst. Physicochem./Biol. P.S.S., Russian Academy of Sciences, 142290, Pushchino, Moscow Region, Russian Federation; email: Tatyana.Vishnivetskaya@mcmail.vanderbilt.edu; CODEN: ESPUD
ER -

TY - JOUR
AU - Schulze-Makuch, D.
AU - Irwin, L.N.
TI - Energy cycling and hypothetical organisms in Europa's ocean
PY - 2002
T2 - Astrobiology
VL - 2
IS - 1
SP - 105
EP - 121
DO - 10.1089/153110702753621385
UR - https://www.scopus.com/inward/record.uri?eid=2-s2.0-0036490356&doi=10.1089%2f153110702753621385&partnerID=40&md5=2f408b0435fd437988bed0d09c5b0f48
AD - Department of Geological Sciences, University of Texas at El Paso, El Paso, TX 79968-0555, 500 West University Avenue, United States
AD - Department of Biological Sciences, University of Texas at El Paso, El Paso, TX, United States
AB - While Europa has emerged as a leading candidate for harboring extraterrestrial life, the apparent lack of a source of free energy for sustaining living systems has been argued. In this theoretical analysis, we have quantified the amount of energy that could in principle be obtained from chemical cycling, heat, osmotic gradients, kinetic motion, magnetic fields, and gravity in Europa's subsurface ocean. Using reasonable assumptions based on known organisms on Earth, our calculations suggest that chemical oxidation-reduction cycles in Europa's subsurface ocean could support life. Osmotic and thermal gradients, as well as the kinetic energy of convection currents, also represent plausible alternative sources of energy for living systems at Europa. Organisms thriving on these gradients could interact with each other to form the complex energy cycling necessary for establishing a stable ecosystem. © Mary Ann Liebert, Inc.

KW - Energy-Generating Resources
KW - Exobiology
KW - Gravitation
KW - Jupiter
KW - Kinetics
KW - Magnetics
KW - Oceans and Seas
KW - Osmosis
KW - article
KW - astronomy
KW - biology
KW - energy resource
KW - gravity
KW - kinetics
KW - magnetism
KW - osmosis
KW - sea
KW - Ecosystem
KW - Energy sources
KW - Europa
KW - Extraterrestrial life
KW - Planetary ocean
SN - 15311074 (ISSN)
C2 - 12449859
LA - English
J2 - Astrobiology
M3 - Article
DB - Scopus
N1 - Export Date: 23 February 2023; Cited By: 50; Correspondence Address: D. Schulze-Makuch; Department of Geological Sciences, University of Texas at El Paso, El Paso, TX 79968-0555, 500 West University Avenue, United States; email: dirksm@geo.utep.edu
ER -

TY - CONF
AU - Kazeminejad, B.
AU - Lebreton, J.-P.
AU - Matson, D.L.
AU - Spilker, L.
AU - Raulin, F.
TI - The Cassini/Huygens mission to Saturn and Titan and its relevance to EXO/astrobiology
PY - 2002
T2 - European Space Agency, (Special Publication) ESA SP
IS - 518
SP - 261
EP - 266
UR - https://www.scopus.com/inward/record.uri?eid=2-s2.0-0036766663&partnerID=40&md5=49a77bbc5db33f711a5a3a5141ae69ad
AD - ESA Res./Sci. Support Department, ESTEC/SCI-SB, 2200 AG Noordwijk, Netherlands
AD - Jet Propulsion Laboratory, California Institute of Technology, Pasadena, CA 91109, 4800 Oak Grove Drive, United States
AD - Lab. Interuniv. Syst. Atmospheriques, Univ. Paris 12 and 7,

94010 Créteil Cedex, France
AB - The Cassini/Huygens spacecraft was launched in October, 1997. Since that time it has been on an interplanetary trajectory toward Saturn. The spacecraft comprises a Saturn orbiter and a Titan entry probe. It arrives at Saturn and goes into orbit about the planet on July 1, 2004. Cassini will perform 75 orbits around Saturn during the nominal 4-year mission. Among them, 44 include close flybys of Titan. Huygens is an entry probe designed to descend by parachute through the atmosphere, down to the surface, of Titan, Saturn's largest moon. The Probe mission is now planned for the third orbit, on 14 January 2005, after two flybys of Titan (in October and December 2004). Huygens will be released about 3 weeks before it reaches Titan. This paper provides an overview of the mission with emphasis on Titan's exploration and the relevance of Titan and this mission to exo/astrobiology. Part of this work was carried out at the Jet Propulsion Laboratory, California Institute of Technology, under contract to NASA.
KW - Planets
KW - Probes
KW - Spacecraft
KW - Trajectories
KW - Astrobiology
KW - Astrophysics
KW - Cassini/Huygens mission
KW - Exobiology
KW - Titan
SN - 03796566 (ISSN)
LA - English
J2 - Eur Space Agency Spec Publ ESA SP
M3 - Conference paper
DB - Scopus
N1 - Export Date: 23 February 2023; Cited By: 1; CODEN: ESPUD
ER -

TY - CONF
AU - Kereszturi, A.
TI - Astrobiological potentials of brown dwarfs
PY - 2002
T2 - European Space Agency, (Special Publication) ESA SP
IS - 518
SP - 525
EP - 526
UR - https://www.scopus.com/inward/record.uri?eid=2-s2.0-0036766105&partnerID=40&md5=0d25013d524aa97f609cc1f2f3c57ca0
AB - Astrobiological potentials of brown dwarfs (BD) were discussed. The analysis mostly covers the processes in their atmospheres. Results showed that at certain mass and age intervals water can appear in the atmosphere of BDs in clouds, during the evolution of BDs the optimal atmospheric layer sinks downwards into higher pressure environment and that the organic matter formation could be problematic even in the late cold phase of BDs.
KW - Atmospheric radiation
KW - Biology
KW - Cooling

KW - Planets
KW - Astrobiology
KW - Astronomy
PB - European Space Agency
SN - 03796566 (ISSN)
LA - English
J2 - Eur Space Agency Spec Publ ESA SP
M3 - Conference paper
DB - Scopus
N1 - Export Date: 23 February 2023; Cited By: 0; CODEN: ESPUD
ER -

TY - JOUR
AU - Andersen, D.T.
AU - Pollard, W.H.
AU - McKay, C.P.
AU - Heldmann, J.
TI - Cold springs in permafrost on Earth and Mars
PY - 2002
T2 - Journal of Geophysical Research: Planets
VL - 107
IS - 3
SP - 4
EP - 1
DO - 10.1029/2000je001436
UR - https://www.scopus.com/inward/record.uri?eid=2-s2.0-0036077811&doi=10.1029%2f2000je001436&partnerID=40&md5=1e87ed8bc5ba4e9384b1f9f0612b7917
AD - Department of Geography, McGill University, Montreal, Que., Canada
AD - NASA, Ames Research Center, Moffett Field, CA, United States
AB - Perennial springs located on west central Axel Heiberg Island in the Canadian High Arctic occur in a region with a mean annual air temperature of − 15°C and flow through continuous permafrost 600 m thick. The spring water is a low-temperature (up to 6°C) brine that maintains constant discharge temperatures and flow rates throughout the year. Here we report on observations of temperature and discharge rate of these springs and develop a combined flow and thermal model of the subsurface flow using the measured geothermal gradient. We also consider the implications these springs have for the search for similar environments, past or present, on Mars.
KW - Axel Heiberg
KW - Canada
KW - Nunavut
KW - arctic environment
KW - geothermal gradient
KW - hydrology
KW - Mars
KW - permafrost
KW - spring water
KW - subsurface flow
KW - Arctic
KW - Astrobiology
KW - Hydrology

KW - Mars
KW - Permafrost
KW - Springs
PB - American Geophysical Union
SN - 01480227 (ISSN)
LA - English
J2 - J. Geophys. Res. E Planets
M3 - Article
DB - Scopus
N1 - Export Date: 23 February 2023; Cited By: 71; Correspondence Address: D.T. Andersen; Department of Geography, McGill University, Montreal, Que. H3A 2K6, 805 Sherbrooke Street West, Canada; email: andersen@gal.arc.nasa.gov
ER -

TY - CONF
AU - Deutsch, L.J.
TI - Resolving the Cassini/Huygens relay radio anomaly
PY - 2002
T2 - IEEE Aerospace Conference Proceedings
VL - 3
C7 - 1035262
SP - 1295
EP - 1302
DO - 10.1109/AERO.2002.1035262
UR - https://www.scopus.com/inward/record.uri?eid=2-s2.0-79751522132&doi=10.1109%2fAERO.2002.1035262&partnerID=40&md5=a56be8ee3769cd8532293b9cbde45524
AD - Jet Propulsion Lab., California Inst. of Technol., Pasadena, CA, United States
AB - NASA's Cassini mission to Saturn carries the European Space Agency's (ESA's) Huygens probe, which it will release shortly before an encounter with Saturn's moon, Titan, a possible location for extraterrestrial life within our Solar System. As it parachutes towards Titan's surface, Huygens will acquire scientific information which will be relayed to Earth through Cassini. Comprehensive testing of this relay radio link was not performed prior to Cassini launch and cannot be done during cruise. A test using NASA's Deep Space Network (DSN) to mimic the probe's signal was performed in 2000 and uncovered an anomaly that, unchecked, would result in nearly complete loss of the Huygens mission. An international team of experts from NASA and ESA was assembled to solve this problem: the Huygens Recovery Task Force (HRTF.) This team, co-chaired by the author, performed extensive testing, modeling, and simulation to understand the failure mechanism. Each Huygens science team determined mission impacts for various scenarios based on these results. This led to a suggested modification to the Cassini trajectory that will result in nearly complete data return for Huygens with minimal impact on Cassini. © 2002 IEEE.
KW - Antenna arrays
KW - Moon
KW - NASA
KW - Radio links
KW - Comprehensive testing

KW - Deep space networks
KW - European Space Agency
KW - Extensive testing
KW - Extraterrestrial life
KW - Failure mechanism
KW - International team
KW - Scientific information
KW - Space flight
SN - 1095323X (ISSN); 078037231X (ISBN); 978-078037231-3 (ISBN)
LA - English
J2 - IEEE Aerosp. Conf. Proc.
M3 - Conference paper
DB - Scopus
N1 - Export Date: 23 February 2023; Cited By: 9; Correspondence Address: L.J. Deutsch; Jet Propulsion Lab., California Inst. of Technol., Pasadena, CA, United States; email: leslie.j.deutsch@jpl.nasa.gov
ER -

TY - CHAP
AU - Frey, H.U.
AU - Lummerzheim, D.
TI - Can conditions for life be inferred from optical emissions of extra-solar-system planets?
PY - 2002
T2 - Geophysical Monograph Series
VL - 130
SP - 381
EP - 388
DO - 10.1029/130GM26
UR - https://www.scopus.com/inward/record.uri?eid=2-s2.0-52149103918&doi=10.1029%2f130GM26&partnerID=40&md5=4877a042192a7399feaa513d410ccb71
AD - Space Sciences Laboratory, University of California, Berkeley, 94720-7450, CA, United States
AD - Geophysical Institute, University of Alaska, Fairbanks, 99775, AK, United States
AB - The recent detection of several planets in other solar systems has revived the discussion about possibilities of extraterrestrial life and of ways of a remote detection. According to our present understanding of the development of life on Earth and the basics of a water-carbon based system, there are several environmental requirements for the development of life. These conditions, however, are necessary but not sufficient indicators for the presence of life on other planets. On Earth, early life forms had a great impact on compositional changes of the terrestrial atmosphere by the production and continued supply of free oxygen. This paper discusses spectral features of oxygen emission and absorption and the possibilities of their use for a remote characterization of the chemical composition of extraterrestrial atmospheres. Observations of planets and moons in the solar system indicate, that oxygen emissions can be produced by many processes and that only a complete characterization of an atmosphere and the detection of significant amounts of molecular oxygen can be used as an indicator for the

possible existence of higher life forms. © 2002 by the American Geophysical Union.
A2 - Nagy A.
A2 - Waite J.H.
A2 - Mendillo M.
PB - Blackwell Publishing Ltd
SN - 00658448 (ISSN); 978-111866957-0 (ISBN); 978-087590989-9 (ISBN)
LA - English
J2 - Geophys. Monogr. Ser.
M3 - Book chapter
DB - Scopus
N1 - Export Date: 23 February 2023; Cited By: 4
ER -

TY - JOUR
AU - Cavicchioli, R.
TI - Extremophiles and the search for extraterrestrial life.
PY - 2002
T2 - Astrobiology
VL - 2
IS - 3
SP - 281
EP - 292
DO - 10.1089/153110702762027862
UR - https://www.scopus.com/inward/record.uri?eid=2-s2.0-0037669304&doi=10.1089%2f153110702762027862&partnerID=40&md5=b16e862a7c4b4d9524d19bba830c303e
AD - School of Biotechnology and Biomolecular Sciences, University of New South Wales, Sydney, New South Wales, Australia
AB - Extremophiles thrive in ice, boiling water, acid, the water core of nuclear reactors, salt crystals, and toxic waste and in a range of other extreme habitats that were previously thought to be inhospitable for life. Extremophiles include representatives of all three domains (Bacteria, Archaea, and Eucarya); however, the majority are microorganisms, and a high proportion of these are Archaea. Knowledge of extremophile habitats is expanding the number and types of extraterrestrial locations that may be targeted for exploration. In addition, contemporary biological studies are being fueled by the increasing availability of genome sequences and associated functional studies of extremophiles. This is leading to the identification of new biomarkers, an accurate assessment of cellular evolution, insight into the ability of microorganisms to survive in meteorites and during periods of global extinction, and knowledge of how to process and examine environmental samples to detect viable life forms. This paper evaluates extremophiles and extreme environments in the context of astrobiology and the search for extraterrestrial life.
KW - Archaea
KW - Bacteria
KW - Cold
KW - Eukaryotic Cells
KW - Exobiology
KW - Genomics

KW - Space Flight
KW - Archaebacterium
KW - article
KW - bacterium
KW - biology
KW - cold
KW - eukaryotic cell
KW - genomics
KW - space flight
SN - 15311074 (ISSN)
C2 - 12530238
LA - English
J2 - Astrobiology
M3 - Article
DB - Scopus
N1 - Export Date: 23 February 2023; Cited By: 150; Correspondence Address: R. Cavicchioli; email: r.cavicchioli@unsw.edu.au
ER -

TY - JOUR
AU - Ellery, A.
AU - Kolb, C.
AU - Lammer, H.
AU - Parnell, J.
AU - Edwards, H.
AU - Richter, L.
AU - Patel, M.
AU - Romstedt, J.
AU - Dickensheets, D.
AU - Steele, A.
AU - Cockell, C.
TI - Astrobiological instrumentation for Mars – the only way is down
PY - 2002
T2 - International Journal of Astrobiology
VL - 1
IS - 4
SP - 365
EP - 380
DO - 10.1017/S1473550403001204
UR - https://www.scopus.com/inward/record.uri?eid=2-s2.0-85023153380&doi=10.1017%2fS1473550403001204&partnerID=40&md5=f3f55e41fd10bbf92fcb9da5b781f514
AD - School of Engineering, Kingston University, London, United Kingdom
AD - Space Research Institute, Austrian Academy of Sciences, Graz, Austria
AD - Department of Geology & Petroleum Geology, University of Aberdeen, Aberdeen, United Kingdom
AD - Department of Chemical & Forensic Sciences, University of Bradford, Bradford, United Kingdom
AD - DLR, Cologne, Germany
AD - Planetary & Space Sciences Research Institute, Open University, Milton Keynes, United Kingdom

AD - ESA-ESTEC, Noordwijk, Netherlands
AD - Department of Electrical and Computer Engineering, Montana State University, Bozeman, Montana, United States
AD - Carnegie Institute of Washington, Geophysical Laboratory, Washington, DC, United States
AD - British Antarctic Survey, Cambridge, United Kingdom
AB - In this paper, in this edition of the Journal commemorating the life and work of David Wynn-Williams, we consider approaches to the astrobiological investigation of Mars. We provide a brief account of the scientific rationale behind the approach presented here. In particular, we outline the capabilities of the Raman spectrometer for the detection of biomarkers. David Wynn-Williams was an active champion of this instrument who was keen to field-qualify a version in Antarctica with a view to flying a Raman instrument onboard a Mars-bound space mission. We examine a scenario for the deployment of such an instrument in conjunction with other instrumentation and argue that subsurface deployment of scientific instruments is essential if we are to succeed in detecting any evidence that may exist for former life on Mars. We outline a mission scenario – Vanguard – which represents a novel but low-risk, low-cost approach to Mars exploration that was conceived and developed jointly by one of the authors (Ellery) and the late David Wynn-Williams. © 2002, Cambridge University Press. All rights reserved.
KW - astrobiology
KW - Mars
KW - mole
KW - oxidant
KW - Raman
KW - rover
SN - 14735504 (ISSN)
LA - English
J2 - Int. J. Astrobiology
M3 - Article
DB - Scopus
N1 - Export Date: 23 February 2023; Cited By: 30
ER -

TY - CONF
AU - Hoover, R.B.
AU - Jerman, G.
AU - Rozanov, A.Y.
AU - Davies, P.C.W.
TI - Biomarkers and microfossils in the Murchison, Rainbow, and Tagish Lake meteorites
PY - 2002
T2 - Proceedings of SPIE - The International Society for Optical Engineering
VL - 4859
SP - 15
EP - 31
DO - 10.1117/12.463321
UR - https://www.scopus.com/inward/record.uri?eid=2-s2.0-0038104843&doi=10.1117%2f12.463321&partnerID=40&md5=333ac22cfc6

28d064b60b28fae2bed68
AD - Astrobiology Group Leader, Space Science Department/SD-50, NASA-George C. Marshall SFC, Huntsville, AL 35812, United States
AD - Space Science Department/ED-21, NASA-George C. Marshall SFC, Huntsville, AL 35812, United States
AD - Paleontological Institute, Russian Academy of Sciences, 117 Moscow, 123 Profsoyuznaya Str., Russian Federation
AD - Astrobiology Institute, Sydney, NSW, Australia
AB - During the past six years, we have conducted extensive scanning electron and optical microscopy investigations and x-ray analysis to determine the morphology, life cycle processes, and elemental distributions in living and fossil cyanobacteria, bacteria, archaea, fungi, and algae sampled from terrestrial environments relevant to Astrobiology. Biominerals, pseudomorphs and microfossils have been studied for diverse microbial groups in various states of preservation in many types of rocks (e.g., oil shales, graphites, shungites, bauxites, limestones, pyrites, phosphorites, and hydrothermal vent chimneys). Results of these studies have been applied to the search for biosignatures in carbonaceous chondrites, stony, and nickel iron meteorites. We review important biomarkers found in terrestrial rocks and meteorites and present additional evidence for the existence of indigenous bacterial microfossils in-situ in freshly fractured surfaces of the Murchison, Rainbow and Tagish Lake carbonaceous meteorites. We provide secondary and backscatter electron images and spectral data obtained with Field Emission and Environmental Scanning Electron Microscopes of biominerals and microfossils. We discuss techniques for discriminating indigenous microfossils from recent terrestrial contaminants. Images are provided of framboidal magnetites in oil shales and meteorites and images and 2D x-ray maps are shown of bacterial microfossils embedded in the mineral matrix of the Murchison, Rainbow and Tagish Lake Carbonaceous Meteorites. These microfossils exhibit characteristics that preclude their interpretation as post-arrival contaminants and we interpret them as indigenous biogenic remains.
KW - Astronomy
KW - Bacteria
KW - Biomarkers
KW - Fungi
KW - Microbiology
KW - Morphology
KW - Optical microscopy
KW - Rocks
KW - Scanning electron microscopy
KW - X ray analysis
KW - Archaea
KW - Astrobiology
KW - Biominerals
KW - Biosignatures
KW - Carbonaceous chondrites
KW - Cyanobacteria
KW - Microfossils
KW - Pseudomorphs
KW - Meteorites

KW - Astrobiology
KW - Biomarkers
KW - Meteorites
KW - Microfossils
KW - Murchison
KW - Rainbow
KW - Tagish Lake
SN - 0277786X (ISSN)
LA - English
J2 - Proc SPIE Int Soc Opt Eng
M3 - Conference paper
DB - Scopus
N1 - Export Date: 23 February 2023; Cited By: 19; Correspondence Address: R.B. Hoover; Astrobiology Group Leader, Space Science Department/SD-50, NASA-George C. Marshall SFC, Huntsville, AL 35812, United States; email: Richard.Hoover@msfc.nasa.gov; CODEN: PSISD
ER -

TY - CONF
AU - Irion, R.
TI - Astrobiology science conference: Astrobiologists try to 'follow the water to life'
PY - 2002
T2 - Science
VL - 296
IS - 5568
SP - 647
EP - 648
DO - 10.1126/science.296.5568.647
UR - https://www.scopus.com/inward/record.uri?eid=2-s2.0-0037177572&doi=10.1126%2fscience.296.5568.647&partnerID=40&md5=6b023a74325ed18cd83cb9de546a6568
AB - That slogan, expressed by a speaker at the biannual Astrobiology Science Conference held 8 to 11 April here at NASA's Ames Research Center, unified most of the talks heard by more than 700 astronomers, biologists, chemists, geologists, planetary scientists, and even virologists. Among the topics raised within the cavernous 60-meter-high Hangar One - which once housed a pre-World War II Navy dirigible - were the liquid milieu in which the first cells may have formed and the effects of water and ice on an Arctic impact crater.
KW - Arctic Regions
KW - Biogenesis
KW - Evolution, Chemical
KW - Exobiology
KW - Extraterrestrial Environment
KW - Geologic Sediments
KW - Ice
KW - Mars
KW - Meteoroids
KW - Seawater
KW - Water
KW - Arctic
KW - Astronomy

KW - Cells
KW - Ice
KW - Research
KW - Water
KW - sea water
KW - astronomy
KW - biology
KW - crater
KW - impact
KW - Arctic
KW - astronomy
KW - conference paper
KW - cosmos
KW - desert
KW - priority journal
KW - salinity
KW - space
KW - Crater
KW - Biology
SN - 00368075 (ISSN)
C2 - 11976427
LA - English
J2 - Science
M3 - Conference paper
DB - Scopus
N1 - Export Date: 23 February 2023; Cited By: 3; CODEN: SCIEA
ER -

TY - JOUR
AU - Rothschild, L.J.
AU - Giver, L.J.
TI - Photosynthesis below the surface in a cryptic microbial mat
PY - 2002
T2 - International Journal of Astrobiology
VL - 1
IS - 4
SP - 295
EP - 304
DO - 10.1017/S1473550403001320
UR - https://www.scopus.com/inward/record.uri?eid=2-s2.0-33746415233&doi=10.1017%2fS1473550403001320&partnerID=40&md5=5d473476d6124b09dde19aa0ba354ade
AD - Ecosystem Science and Technology Branch, NASA/Ames Research Center, Moffett Field, CA 94035-1000, United States
AD - Codexis Inc., Redwood City, CA 94063, 515 Galveston Drive, United States
AB - The discovery of subsurface communities has encouraged speculation that such communities might be present on planetary bodies exposed to harsh surface conditions, including the early Earth. While the astrobiology community has focused on the deep subsurface, near-subsurface environments are unique in that they provide some protection while allowing partial access to photosynthetically active radiation. Previously we identified near-surface microbial communities based on photosynthesis. Here we

assess the productivity of such an ecosystem by measuring in situ carbon fixation rates in an intertidal marine beach through a diurnal cycle, and find them surprisingly productive. Gross fixation along a transect (99×1 m) perpendicular to the shore was highly variable and depended on factors such as moisture and mat type, with a mean of ~41 mg C fixed m-2 day-1. In contrast, an adjacent well-established cyanobacterial mat dominated by Lyngbya aestuarii was ~12 times as productive (~500 mg C fixed m-2 day-1). Measurements made of the Lyngbya mat at several times per year revealed a correlation between total hours of daylight and gross daily production. From these data, annual gross fixation was estimated for the Lyngbya mat and yielded a value of ~1.3×105 g m-2 yr-1. An analysis of pulse-chase data obtained in the study in conjunction with published literature on similar ecosystems suggests that subsurface interstitial mats may be an overlooked endogenous source of organic carbon, mostly in the form of excreted fixed carbon. © 2002, Cambridge University Press. All rights reserved.
KW - carbon fixation
KW - cyanobacteria
KW - intertidal
KW - Lyngbya aestuarii
KW - microbial mat
KW - photosynthesis
KW - subsurface
SN - 14735504 (ISSN)
LA - English
J2 - Int. J. Astrobiology
M3 - Article
DB - Scopus
N1 - Export Date: 23 February 2023; Cited By: 7
ER -

TY - JOUR
AU - Blumberg, B.S.
TI - Astrobiology: An introduction
PY - 2002
T2 - Anatomical Record
VL - 268
IS - 3
SP - 169
EP - 170
DO - 10.1002/ar.10150
UR - https://www.scopus.com/inward/record.uri?eid=2-s2.0-0036829768&doi=10.1002%2far.10150&partnerID=40&md5=9ef15abb5c35a7735d18de55b6f69adf
AD - NASA Astrobiology Institute, Ames Research Center, Moffett Field, CA, United States
AD - NASA Astrobiology Institute, MS 240-1, Ames Research Center, Moffett Field, CA 94035, United States
KW - Biogenesis
KW - Exobiology
KW - United States
KW - United States National Aeronautics and Space Administration
KW - astronomy

KW - biogenesis
KW - biology
KW - cell type
KW - continuing education
KW - editorial
KW - environment
KW - evolution
KW - geology
KW - human
KW - nonhuman
KW - oceanography
KW - priority journal
KW - space
KW - species differentiation
SN - 0003276X (ISSN)
C2 - 12382314
LA - English
J2 - Anat. Rec.
M3 - Editorial
DB - Scopus
N1 - Export Date: 23 February 2023; Cited By: 1; Correspondence Address: B.S. Blumberg; NASA Astrobiology Institute, MS 240-1, Ames Research Center, Moffett Field, CA 94035, United States; email: bblumberg@mail.arc.nasa.gov; CODEN: ANREA
ER -

TY - CONF
AU - Lorenz, R.D.
TI - Navigation of aerial platforms on Titan
PY - 2002
T2 - Advances in the Astronautical Sciences
VL - 112 I
SP - 563
EP - 573
UR - https://www.scopus.com/inward/record.uri?eid=2-s2.0-0036919068&partnerID=40&md5=4203ddbc00641195772f0dcd126ce144
AD - Lunar and Planetary Lab, University of Arizona, Tucson, AZ 85721-0092, United States
AB - Future missions to Saturn's giant atmosphere-shrouded moon Titan may involve aerial platforms such as airships or helicopters. A significant challenge is the navigation of such vehicles, both in terms of global position determination and path planning in the zonal wind field, and for local stationkeeping to acquire organic surface samples of astrobiological interest. This paper overviews the Titan environment and the likely science goals and mission scenarios for post-Cassini missions, and examines the various methods (astronavigation, complicated by the hazy atmosphere, but possible at selected wavelengths; dead-reckoning, Doppler navigation using an orbiter, expendable beacons, etc.).
KW - Interplanetary flight
KW - Interplanetary spacecraft
KW - Navigation
KW - Space platforms
KW - Aerial platforms

KW - Astronavigation
KW - Aerospace engineering
SN - 00653438 (ISSN)
LA - English
J2 - Adv Astronaut Sci
M3 - Conference paper
DB - Scopus
N1 - Export Date: 23 February 2023; Cited By: 3; Correspondence Address: R.D. Lorenz; Lunar and Planetary Lab, University of Arizona, Tucson, AZ 85721-0092, United States; email: rlorenz@lpl.arizona.edu; CODEN: ADASA
ER -

TY - JOUR
AU - Paepe, R.
AU - Van Overloop, E.
AU - Hoover, R.B.
TI - Comparison of Snowball Earth and Mars conditions (Is Mars climatically in resonance with the Earth?)
PY - 2002
T2 - Proceedings of SPIE - The International Society for Optical Engineering
VL - 4495
SP - 40
EP - 54
DO - 10.1117/12.454775
UR - https://www.scopus.com/inward/record.uri?eid=2-s2.0-0036395101&doi=10.1117%2f12.454775&partnerID=40&md5=94391f2894156b9ddc86d38f250c4b05
AD - GEOBOUND INTERNATIONAL Ltd/Free University, Herzele, B-9550, Brussels, Doomstraat 27, Belgium
AD - Marshall Space Flight Centre, Huntsville, AL 35812, United States
AB - A number of landscape features of the surface of Mars have revealed similarities of morphology which may imply similarities of development and by and large, the presence of water, ice and permafrost. A systematic comparative study between landscapes of the Earth and those of Mars may be useful for the understanding of the evolution of Mars and the possible presence of global climatic changes on Mars. The comparative study along a landscape traverse from the poles till the equator on Earth can for this purpose successfully be compared with the Mars. It is concluded that Snowball Earth Stages similar to the Mars icy global stage today occurred periodically on Earth as well. Furthermore the evidence of melting water phenomena observed on Mars images point at protracted thawing inferring a Mars Global Warming today in resonance with the Earth.
KW - Earth (planet)
KW - Geomorphology
KW - Martian surface analysis
KW - Morphology
KW - Astrobiology
KW - Geo-dynamic changes
KW - Geomorphic traverse

KW - Global changes
KW - Intertropical convergent zone changes
KW - Landscape
KW - Melting water phenomena
KW - Snowball earth
KW - Permafrost
KW - Geo-dynamic changes
KW - Geomorphic traverse
KW - Global changes
KW - ITCZ-changes
KW - Permafrost
SN - 0277786X (ISSN)
LA - English
J2 - Proc SPIE Int Soc Opt Eng
M3 - Article
DB - Scopus
N1 - Export Date: 23 February 2023; Cited By: 2
ER -

TY - CONF
AU - Ellery, A.
AU - Ball, A.
AU - Cockell, C.
AU - Coste, P.
AU - Dickensheets, D.
AU - Edwards, H.
AU - Hu, H.
AU - Kolb, C.
AU - Lammer, H.
AU - Lorenz, R.
AU - McKee, G.
AU - Richter, L.
AU - Winfield, A.
AU - Welch, C.
TI - Robotic astrobiology - The need for sub-surface penetration of Mars
PY - 2002
T2 - European Space Agency, (Special Publication) ESA SP
IS - 518
SP - 313
EP - 317
UR - https://www.scopus.com/inward/record.uri?eid=2-s2.0-0036766064&partnerID=40&md5=18685216352c9cc47a7acbc4a4144338
AD - School of Engineering, Kingston University, London, United Kingdom
AD - Planet./Space Sci. Res. Institute, Open University, Milton Keynes, United Kingdom
AD - British Antarctic Survey, Cambridge, United Kingdom
AD - Mechanical Engineering Department, ESTEC, Holland, MI, United States
AD - Dept. of Elec./Computer Engineering, Montana State University, Nozeman, MT, United States
AD - Dept. of Chem. and Forensic Sciences, University of Bradford, Bradford, United Kingdom

AD - Department of Computer Science, University of Essex, Colchester, United Kingdom
AD - Institut fur Weltraumforschung, Osterreichische Akad. der Wiss., Graz, Austria
AD - Lunar and Planetary Laboratory, University of Arizona, Tucson, AZ, United States
AD - Department of Computer Science, Cybernet. and Electronic Engineering, University of Reading, Reading, United Kingdom
AD - DLR, Cologne, Germany
AD - Intelligent Autonomous Syst. Lab., University of the West of England, Bristol, United Kingdom
AB - Recent interest in the astrobiological investigation of Mars has culminated in the only planned astrobiology-focussed robotic mission to Mars - the Beagle2 mission to be carried to Mars by the Mars Express spacecraft in 2003. Beagle2 will be primarily investigating the surface and near-surface environment of Mars. However, the results from the Viking Mars lander indicated that the Martian surface is saturated in peroxides and super-oxides which would rapidly degrade any organic material. Furthermore, recent models of gardening due to meteoritic impacts on the Martian surface suggest that the depth of this oxidising layer could extend to depths of 2-3m. Given that the discovery of organic fossilised residues will be the primary target for astrobiological investigation, this implies that future robotic astrobiology missions to Mars must penetrate to below these depths. The need to penetrate into the sub-surface of Mars has recently been given greater urgency with the discovery of extensive water ice-fields as little as 1m from the surface. We review the different technologies that make this penetration into the sub-surface a practical possibility on robotic missions. We further briefly present one such implementation of these technologies through the use of ground-penetrating moles - the Vanguard Mars mission proposal.
KW - Ice
KW - Meteorites
KW - Peroxides
KW - Robotics
KW - Spacecraft
KW - Water
KW - Robotic astrobiology
KW - Martian surface analysis
SN - 03796566 (ISSN)
LA - English
J2 - Eur Space Agency Spec Publ ESA SP
M3 - Conference paper
DB - Scopus
N1 - Export Date: 23 February 2023; Cited By: 8; Correspondence Address: A. Ellery; School of Engineering, Kingston University, London, United Kingdom; email: a.ellery@kingston.ac.uk; CODEN: ESPUD
ER -

TY - JOUR
AU - Chyba, C.F.
AU - Phillips, C.B.
TI - Europa as an abode of life

PY - 2002
T2 - Origins of Life and Evolution of the Biosphere
VL - 32
IS - 1
SP - 47
EP - 67
DO - 10.1023/A:1013958519734
UR - https://www.scopus.com/inward/record.uri?eid=2-s2.0-0036008698&doi=10.1023%2fA%3a1013958519734&partnerID=40&md5=0635262c5077e4c2eccaa153f37b6602
AD - Center for the Study of Life in the Universe, SETI Institute, Mountain View, CA, United States
AD - Department of Geological and Environmental Sciences, Stanford University, Stanford, CA, United States
AB - Life as we know it on Earth depends on liquid water, a suite of 'biogenic' elements (most famously carbon) and a useful source of free energy. Here we review Europa's suitability for life from the perspective of these three requirements. It is likely, though not yet certain, that Europa harbors a subsurface ocean of liquid water whose volume is about twice that of Earth's oceans. Little is known about Europa's inventory of carbon, nitrogen, and other biogenic elements, but lower bounds on these can be placed by considering the role of cometary delivery over Europa's history. Sources of free energy are challenging for a world covered with an ice layer kilometers thick, but it is possible that hydrothermal activity and/or organics and oxidants provided by the action of radiation chemistry at Europa's surface and subsequent mixing into Europa's ocean could provide the electron donors and acceptors needed to power a Europan ecosystem. It is not premature to draw lessons from the search for life on Mars with the Viking spacecraft for planning exobiological missions to Europa.
KW - Biogenesis
KW - Carbon
KW - Exobiology
KW - Jupiter
KW - Magnetics
KW - Space Flight
KW - Thermodynamics
KW - Water
KW - carbon
KW - water
KW - Europa
KW - exobiology
KW - Jupiter
KW - origin of life
KW - article
KW - astronomy
KW - biogenesis
KW - biology
KW - chemistry
KW - magnetism
KW - space flight
KW - thermodynamics
KW - Astrobiology

KW - Europa
KW - Exobiology
KW - Jupiter
KW - Oceans
KW - Radiation
PB - Springer Netherlands
SN - 01696149 (ISSN)
C2 - 11889917
LA - English
J2 - Orig. Life Evol. Biosph.
M3 - Article
DB - Scopus
N1 - Export Date: 23 February 2023; Cited By: 105; CODEN: OLEBE
ER -

TY - JOUR
AU - Maurel, M.-C.
AU - Counil, J.-L.
TI - Mars samples analysis: Program and objectives
PY - 2002
T2 - European Space Agency, (Special Publication) ESA SP
IS - 518
SP - 541
EP - 542
UR - https://www.scopus.com/inward/record.uri?eid=2-s2.0-0036766656&partnerID=40&md5=05435636597216aa27095f8641a3ad33
AD - Institut Jacques Monod, Lab. Biochim. Evol. Adaptabilite M., 75251 Paris Cedex 05, Tour 43, 2 place Jussieu, France
AD - C.N.E.S. et C.S.E.E.M, Centre de Toulouse, 31401 Toulouse Cedex 4, 18 ave. Edouard Belin, France
AB - The main goals of French Mars exploration program are the development of a netlander for geophysical studies. This project is conducted in the framework of a consortium of European partners (Finland, Germany and Belgium), and the development, of an orbiter vehicle of the future Mars sample return mission (MSR) is performed in cooperation with NASA. Also, we discuss the preparatory program for volatile and rare gas analysis, sample pollution, site selection and Exo/Astrobiology. Multi-disciplinary groups of biologists emerged in France as a result of two workshops organized by the French Committee (CSEEM) devoted to Mars sample return. These groups are currently developing strategies to detect potential extinct and/or extant life on Mars. The most sensible idea to address numerous questions is to bring safely to Earth laboratories selected samples from the surface and subsurface of Mars. Most of the investigations will be conducted under quarantine.
KW - Geophysics
KW - Inert gases
KW - Orbits
KW - Research laboratories
KW - Space research
KW - Astrobiology
KW - Martian surface analysis
SN - 03796566 (ISSN)
LA - English

J2 - Eur Space Agency Spec Publ ESA SP
M3 - Article
DB - Scopus
N1 - Export Date: 23 February 2023; Cited By: 0; Correspondence Address: M.-C. Maurel; Institut Jacques Monod, Lab. Biochim. Evol. Adaptabilite M., 75251 Paris Cedex 05, Tour 43, 2 place Jussieu, France; email: maurel@ijm.jussieu.fr; CODEN: ESPUD
ER -

TY - CONF
AU - Bartolini, C.
AU - Benelli, M.
AU - Molina, M.
AU - Solmi, L.
TI - Teaching astrobiology by means of a multimedia system
PY - 2002
T2 - European Space Agency, (Special Publication) ESA SP
IS - 518
SP - 501
EP - 502
UR - https://www.scopus.com/inward/record.uri?eid=2-s2.0-0036766414&partnerID=40&md5=5397d543d6cde8befa573b58c6e07ad2
AD - Depart. Astronomy, Univ. of Bologna, 40127 Bologna, via Ranzani 1, Italy
AB - At Bologna University Astrobiology is taught with an interactive method involving the students in the improvement of a hypertext. The updating is done discussing the information taken from new books, magazines and the Internet. The main topics are: basic characteristics of life, life in extreme environments, life without Sun, panspermia, possibility of life on Mars and Europa. The main point is the astronomical fact that, at least 10 billions years ago, all elements necessary for life, as on Earth, had been already forged by the primeval stars.
KW - Earth (planet)
KW - Internet
KW - Multimedia systems
KW - Planets
KW - Students
KW - Sun
KW - Teaching
KW - Astrobiology
KW - Astrophysics
SN - 03796566 (ISSN)
LA - English
J2 - Eur Space Agency Spec Publ ESA SP
M3 - Conference paper
DB - Scopus
N1 - Export Date: 23 February 2023; Cited By: 0; Correspondence Address: C. Bartolini; Depart. Astronomy, Univ. of Bologna, 40127 Bologna, via Ranzani 1, Italy; email: bartolini@bo.astro.it; CODEN: ESPUD
ER -

TY - JOUR

AU - Arnold, L.
AU - Gillet, S.
AU - Lardière, O.
AU - Riaud, P.
AU - Schneider, J.
TI - A test for the search for life on extrasolar planets. Looking for the terrestrial vegetation signature in the Earthshine spectrum
PY - 2002
T2 - Astronomy and Astrophysics
VL - 392
IS - 1
SP - 231
EP - 237
DO - 10.1051/0004-6361:20020933
UR - https://www.scopus.com/inward/record.uri?eid=2-s2.0-0036732363&doi=10.1051%2f0004-6361%3a20020933&partnerID=40&md5=11e5f6620f4fff243f709801d8f264d3
AD - Observatoire de Haute-Provence (OHP), CNRS, 04870 Saint-Michel-l'Observatoire, France
AD - Laboratoire d'Interférométrie Stellaire et Exoplanétaire (LISE), CNRS, 04870 Saint-Michel-l'Observatoire, France
AD - Observatoire de Paris-Meudon, 92195 Meudon Cedex, France
AB - We report spectroscopic observations (400-800 nm, R ≈ 100) of Earthshine in June, July and October 2001 from which normalized Earth albedo spectra have been derived. The resulting spetra clearly show the blue colour of the Earth due to Rayleigh diffusion in its atmosphere. They also show the signatures of oxygen, ozone and water vapour. We tried to extract from these spectra the signature of Earth vegetation. A variable signal (4 to 10 ± 3%) around 700 nm has been measured in the Earth albedo. It is interpreted as being due to the vegetation red edge, expected to be between 2 to 10% of the Earth albedo at 700 nm, depending on models. We discuss the primary goal of the present observations: their application to the detection of vegetation-like biosignatures on extrasolar planets.
KW - Astronomy
KW - Diffusion
KW - Oxygen
KW - Ozone
KW - Astrobiology
KW - Extrasolar planets
KW - Stars
KW - Planets
KW - Astrobiology
KW - Stars: planetary systems
PB - EDP Sciences
SN - 00046361 (ISSN)
LA - English
J2 - Astron. Astrophys.
M3 - Article
DB - Scopus
N1 - Export Date: 23 February 2023; Cited By: 119; Correspondence Address: L. Arnold; OHP CNRS, 04870 Saint-Michel-1'Observatoire, France; email: arnold@obs-hp.fr; CODEN: AAEJA
ER -

TY - JOUR
AU - Cleland, C.E.
AU - Chyba, C.F.
TI - Defining 'life'
PY - 2002
T2 - Origins of Life and Evolution of the Biosphere
VL - 32
IS - 4
SP - 387
EP - 393
DO - 10.1023/A:1020503324273
UR - https://www.scopus.com/inward/record.uri?eid=2-s2.0-0036669242&doi=10.1023%2fA%3a1020503324273&partnerID=40&md5=b285eaa54a9e52b75d6badd7ecda47fa
AD - Department of Philosophy, Center for Astrobiology, University of Colorado, Boulder, CO 80309, United States
AD - Center for the Study of Life in the Universe, SETI Institute, Mountain View, CA 94043, 2035 Landings Drive, United States
AD - Department of Geological and Environmental Sciences, Stanford University, Stanford, CA 94404, United States
AB - There is no broadly accepted definition of 'life.' Suggested definitions face problems, often in the form of robust counter-examples. Here we use insights from philosophical investigations into language to argue that defining 'life' currently poses a dilemma analogous to that faced by those hoping to define 'water' before the existence of molecular theory. In the absence of an analogous theory of the nature of living systems, interminable controversy over the definition of life is inescapable.
KW - Biogenesis
KW - Evolution, Chemical
KW - Exobiology
KW - Extraterrestrial Environment
KW - Life
KW - Philosophy
KW - Water
KW - water
KW - exobiology
KW - origin of life
KW - philosophy
KW - astronomy
KW - biogenesis
KW - biology
KW - life
KW - microclimate
KW - philosophy
KW - review
KW - Astrobiology
KW - Definition of life
KW - Europa
KW - Natural kinds
KW - Viking
SN - 01696149 (ISSN)
C2 - 12458739

LA - English
J2 - Orig. Life Evol. Biosph.
M3 - Review
DB - Scopus
N1 - Export Date: 23 February 2023; Cited By: 228; CODEN: OLEBE
ER -

TY - CONF
AU - Trautner, R.
AU - Simões, F.
TI - Detection and characterization of ice and water deposits on Mars by means of mutual impedance probes on surface and subsurface vehicles
PY - 2002
T2 - European Space Agency, (Special Publication) ESA SP
IS - 518
SP - 319
EP - 322
UR - https://www.scopus.com/inward/record.uri?eid=2-s2.0-0036766091&partnerID=40&md5=9c83863367bdaf000cf8a8593b867d5d
AD - Space Science Department, ESA, ESTEC, 2200 AG Noordwijk, Netherlands
AB - In the field of Astrobiology, water is generally seen as one of the most important ingredients that allow the development of life. This is also valid for Mars, where recent research suggests the presence of considerable amounts of water in the Martian subsurface. The expected electrical characteristics of Martian material / ice mixtures are recapitulated. We introduce the design features of Mutual Impedance (MI) probes. Assumptions and methods used for model calculations are explained, and simulation results are presented. MI probe architectures for surface mapping and subsurface characterization are suggested, and strategies for identifying promising sampling locations are discussed.
KW - Acoustic impedance
KW - Characterization
KW - Ice
KW - Vehicles
KW - Water
KW - Astrobiology
KW - Martian surface analysis
SN - 03796566 (ISSN)
LA - English
J2 - Eur Space Agency Spec Publ ESA SP
M3 - Conference paper
DB - Scopus
N1 - Export Date: 23 February 2023; Cited By: 0; Correspondence Address: R. Trautner; Space Science Department, ESA, ESTEC, 2200 AG Noordwijk, Netherlands; email: Roland.Trautner@esa.int; CODEN: ESPUD
ER -

TY - JOUR
AU - Hecht, M.H.
TI - Aqueous environments on contemporary Mars?
PY - 2002

T2 - Proceedings of SPIE - The International Society for Optical Engineering
VL - 4495
SP - 69
EP - 80
DO - 10.1117/12.454777
UR - https://www.scopus.com/inward/record.uri?eid=2-s2.0-0036396886&doi=10.1117%2f12.454777&partnerID=40&md5=f1cde49a0e0672b47a32ec24ebc3fff1
AD - Jet Propulsion Laboratory, California Institute of Technology, United States
AB - If Mars evolved from a warm, wet, and biologically active planet to the cold desert conditions we observe today, then the last remnants of life would certainly have adapted to extreme tundra-like conditions. Such adaptation might take the form of an ability to thrive in occasional summer meltwater pools, or even the ability to subtly modify its environment to encourage the formation and persistence of such pools. Surprisingly, recent evidence of ubiquitous gullies and seeps at high latitudes suggest that such seasonal melting may have persisted even to the present day. Calculations presented here give additional credence to that suggestion by demonstrating that water is sufficiently metastable to form seasonally recurring meltwater pools under thin crusts of ice on present-day Mars. Moreover, the simplest of biological mechanisms, by affecting evaporative cooling rates and emissivity, could both encourage and prolong the lifetime of such pools.
KW - Biology
KW - Cooling
KW - Evaporation
KW - Melting
KW - Planetary surface analysis
KW - Water
KW - Astrobiology
KW - Mars
KW - Tundra like conditions
KW - Astrophysics
KW - Astrobiology
KW - Mars
KW - Water
SN - 0277786X (ISSN)
LA - English
J2 - Proc SPIE Int Soc Opt Eng
M3 - Article
DB - Scopus
N1 - Export Date: 23 February 2023; Cited By: 4
ER -

TY - CONF
AU - Leuko, S.
AU - Weidler, G.
AU - Radax, C.
AU - Legat, A.
AU - Kömle, N.
AU - Kargl, G.

AU - Stan-Lotter, H.
TI - Examining the physico-chemical resistance of halobacteria with the live-dead kit, following exposure to simulated Martian atmospheric conditions and heat
PY - 2002
T2 - European Space Agency, (Special Publication) ESA SP
IS - 518
SP - 473
EP - 474
UR - https://www.scopus.com/inward/record.uri?eid=2-s2.0-0036766093&partnerID=40&md5=8812bfcb1068bc09625ed298b4251bb7
AD - Inst. of Genet. and General Biology, University of Salzburg, A-5020 Salzburg, Hellbrunnerstr. 34, Austria
AD - Space Research Institute, Austrian Academy of Sciences, A-8042 Graz, Schmiedlstrasse 6, Austria
AB - Extraterrestrial halite has been detected in meteorites from Mars and from the asteroids; in addition, the Jovian moon Europa is thought to contain a salty ocean. Therefore halobacteria would be useful model organisms when considering the search for extraterrestrial life. We are developing experimental protocols to evaluate the effects of physico-chemical stress factors, in particular present-day Martian conditions, on halobacteria. But the effect of higher temperatures is also of interest, since Mars may have been warmer in the past, and the Alpine salt sediments are known to have experienced local temperature peaks.
KW - Bacteria
KW - Heat treatment
KW - Sediments
KW - Stress analysis
KW - Physico-chemical resistances
KW - Space research
SN - 03796566 (ISSN)
LA - English
J2 - Eur Space Agency Spec Publ ESA SP
M3 - Conference paper
DB - Scopus
N1 - Export Date: 23 February 2023; Cited By: 3; Correspondence Address: S. Leuko; Inst. of Genet. and General Biology, University of Salzburg, A-5020 Salzburg, Hellbrunnerstr. 34, Austria; email: Stefan.leuko@sbg.ac.at; CODEN: ESPUD
ER -

TY - JOUR
AU - Kaiser, R.I.
AU - Balucani, N.
TI - Astrobiology - the final frontier in chemical reaction dynamics
PY - 2002
T2 - International Journal of Astrobiology
VL - 1
IS - 1
SP - 15
EP - 23
DO - 10.1017/S1473550402001015

UR - https://www.scopus.com/inward/record.uri?eid=2-s2.0-0011704668&doi=10.1017%2fS1473550402001015&partnerID=40&md5=b873fdbdb06cb6bc744a98b10f25e2a4
AD - Department of Chemistry, University of York, York YO10 5DD, United Kingdom
AD - Dipartimento di Chimica, Università di Perugia, 06123, Perugia, Italy
AB - Crossed-beam experiments on the reactions of cyano CN(X2Σ+) and ethinyl C2H(X2Σ+) radicals with the unsaturated hydrocarbons acetylene, ethylene, methylacetylene allene and benzene have been carried out under single-collision conditions to investigate synthetic routes to form nitriles, polyynes and substituted allenes in hydrocarbon-rich atmospheres of planets and their moons. All reactions were found to proceed without an entrance barrier, to have exit barriers well below the energy of the reactant molecules and to be strongly exothermic. The predominant identification of the radical versus atomic hydrogen exchange channel makes these reactions compelling candidates for the formation of complex organic chemicals – precursors to biologically important amino acids – in Solar system environments and in the interstellar medium. © 2002, Cambridge University Press. All rights reserved.
KW - amino acids
KW - astrobiology
KW - crossed-beam experiments
KW - laboratory studies
KW - nitriles
KW - polyynes
KW - prebiotic ozone
SN - 14735504 (ISSN)
LA - English
J2 - Int. J. Astrobiology
M3 - Article
DB - Scopus
N1 - Export Date: 23 February 2023; Cited By: 16
ER -

TY - JOUR
AU - Stan-Lotter, H.
AU - Radax, C.
AU - Gruber, C.
AU - Legat, A.
AU - Pfaffenhuemer, M.
AU - Wieland, H.
AU - Leuko, S.
AU - Weidler, G.
AU - Kömle, N.
AU - Kargl, G.
TI - Astrobiology with haloarchaea from Permo-Triassic rock salt
PY - 2002
T2 - International Journal of Astrobiology
VL - 1
IS - 4
SP - 271
EP - 284

DO - 10.1017/S1473550403001307
UR - https://www.scopus.com/inward/record.uri?eid=2-s2.0-2342605577&doi=10.1017%2fS1473550403001307&partnerID=40&md5=e429296a424a7ba8020be81944c2e522
AD - Institute of Genetics and General Biology, University of Salzburg, Hellbrunnerstr. 34, A-5020 Salzburg, Austria
AD - Space Research Institute, Austrian Academy of Sciences, Schmiedlstr. 6, A-8042 Graz, Austria
AB - Several viable halophilic archaebacteria were isolated previously from rock salt of Permo-Triassic age in an Austrian salt mine; one of these strains was the first to be recognized as a novel species from subterranean halite and was designated Halococcus salifodinae. The halophilic microorganisms have apparently survived in the salt sediments over extremely long periods of time. Halobacteria could therefore be suitable model organisms for exploring the possibility of long-term survival of microbes on other planets, in particular, since extraterrestrial halite has been detected in meteorites and is assumed to be present in the subsurface ocean on Europa. Our efforts are directed at the identification of the microbial content of ancient rock salt and the development of procedures for the investigation of the halobacterial response to extreme environmental conditions. Using modified culture media, further halophilic strains were isolated from freshly blasted rock salt and bore cores; in addition, growth of several haloarchaea was substantially improved. Molecular methods indicated the presence of at least 12 different 16S rRNA gene species in a sample of Alpine rock salt, but these strains have not been cultured yet. The exploration of Mars is a target of space missions in the 21st century; therefore, testing the survival of haloarchaea under conditions comparable to present-day Mars, using a simulation chamber, was begun. Preliminary results with Halococcus and Halobacterium species suggested at least tenfold higher survival rates when cells were kept in liquid brines than under dry conditions; staining of cells with the LIVE–DEAD kit, which discriminates between damaged and intact membranes, corroborated these data. © 2002, Cambridge University Press. All rights reserved.
KW - extraterrestrial halite
KW - haloarchaea
KW - halobacteria
KW - Halococcus
KW - LIVE–DEAD staining
KW - longevity
KW - Mars simulation chamber
KW - microbial survival
KW - salt mine
SN - 14735504 (ISSN)
LA - English
J2 - Int. J. Astrobiology
M3 - Article
DB - Scopus
N1 - Export Date: 23 February 2023; Cited By: 36
ER -

TY - JOUR

AU - Pikuta, E.V.
AU - Hoover, R.B.
TI - Sulfate- and sulfur-reducing bacteria as terrestrial analogs for microbial life on Jupiter's satellite Io
PY - 2002
T2 - Proceedings of SPIE - The International Society for Optical Engineering
VL - 4495
SP - 232
EP - 254
DO - 10.1117/12.492485
UR - https://www.scopus.com/inward/record.uri?eid=2-s2.0-0036395151&doi=10.1117%2f12.492485&partnerID=40&md5=a9373577d7e5052ad1496a18d1f8ff16
AD - Ctr. Of Space Plasma Aeronautic Res, University of Alabama at Huntsville, Huntsville, AL 35899, United States
AD - Astrobiology Group Lead - Space Science Directorate/SD-50, NASA-Marshall Space Flight Center, Huntsville, AL 35812, United States
AB - Jupiter's moon Io is the most volcanic body in the solar system with more than 300 active volcanoes detected. Thus, it is important to consider the possibility of the existence of not only the geochemical factors forming the surface geology of Io, but also the active influence of microbiological processes. In particular, the main agents on Earth responsible for the sulfur cycle at anaerobic conditions are the StRB and SrRB. These Sulfate- and Sulfur-reducing bacteria of Earth may provide good analogs for microbial life forms that could possibly exist in association with volcanoes and fumaroles of Jupiter's volcanic moon Io.
KW - Bacteria
KW - Ecosystems
KW - Electron energy levels
KW - Microbiology
KW - Satellites
KW - Solar system
KW - Spectrometry
KW - Sulfur compounds
KW - Anaerobic ecosystems
KW - Archaea
KW - Astrobiology
KW - Electron acceptors
KW - Electron donors
KW - Jupiter
KW - Microbial life
KW - Sulfate reducing bacteria
KW - Sulfur reducing bacteria
KW - Terrestrial analogs
KW - Astronomy
KW - Archaea
KW - Astrobiology
KW - Io
KW - Microbial extremophiles
KW - Sulfate- reducing bacteria
KW - Sulfidogenesis

KW - Sulfur-reducing bacteria
KW - Thermococcus sulfurophilus sp. nov
SN - 0277786X (ISSN)
LA - English
J2 - Proc SPIE Int Soc Opt Eng
M3 - Article
DB - Scopus
N1 - Export Date: 23 February 2023; Cited By: 6
ER -

TY - JOUR
AU - Fagents, S.A.
AU - Lanagan, P.
AU - Greeley, R.
TI - Rootless cones on Mars: A consequence of lava-ground ice interaction
PY - 2002
T2 - Geological Society Special Publication
VL - 202
SP - 295
EP - 317
DO - 10.1144/GSL.SP.2002.202.01.15
UR - https://www.scopus.com/inward/record.uri?eid=2-s2.0-12244307866&doi=10.1144%2fGSL.SP.2002.202.01.15&partnerID=40&md5=fefad669a8e2960cc30ad972104b55be
AD - Department of Geological Sciences, Arizona State University, Tempe, AZ 85287-1404, United States
AD - Lunar and Planetary Laboratory, University of Arizona, Tucson, AZ 85721, United States
AD - Hawaii Inst. of Geophys./Planetology, University of Hawaii at Manoa, Honolulu, HI 96822, 2525 Correa Road, United States
AB - Fields of small cratered cones on Mars are interpreted to have formed by rootless eruptions due to explosive interaction of lava with ground ice contained within the regolith beneath the flow. Melting and vaporization of the ice, and subsequent explosive expansion of the vapour, act to excavate the lava and construct a rootless cone around the explosion site. Similar features are found in Iceland, where flowing lavas encountered water-saturated substrates. The martian cones have basal diameters of c. 30-1000 m and are located predominantly in the northern volcanic plains. High-resolution Mars Orbiter Camera images offer significant improvements over Viking data for interpretation of cone origins. A new model of the dynamics of cone formation indicates that very modest amounts of water ice are required to initiate and sustain the explosive interactions that produced the observed features. This is consistent with the likely low availability of water ice in the martian regolith. The scarcity of impact craters on many of the host lava flows indicates very young ages, suggesting that ground ice was present as recently as <10-100 Ma, and may persist today. Rootless cones therefore act as a spatial and temporal probe of the distribution of ground ice on Mars, which is of key significance in understanding the evolution of the martian climate. The location of water in liquid or solid form is of great importance to future robotic and human exploration strategies, and to the search for

extraterrestrial life.
KW - crater
KW - ice-structure interaction
KW - lava
KW - Mars
KW - planetary surface
KW - volcanic eruption
SN - 03058719 (ISSN)
LA - English
J2 - Geol. Soc. Spec. Publ.
M3 - Article
DB - Scopus
N1 - Export Date: 23 February 2023; Cited By: 67; Correspondence Address: S.A. Fagents; Hawaii Inst. of Geophys./Planetology, University of Hawaii at Manoa, Honolulu, HI 96822, 2525 Correa Road, United States; email: fagents@higp.hawaii.edu
ER -

TY - JOUR
AU - Parnell, J.
AU - Mazzini, A.
AU - Honghan, C.
TI - Fluid inclusion studies of chemosynthetic carbonates: Strategy for seeking life on mars
PY - 2002
T2 - Astrobiology
VL - 2
IS - 1
SP - 43
EP - 57
DO - 10.1089/153110702753621330
UR - https://www.scopus.com/inward/record.uri?eid=2-s2.0-0036490458&doi=10.1089%2f153110702753621330&partnerID=40&md5=639bff99069e4ac97c84c72c52da84cd
AD - Department of Geology and Petroleum Geology, University of Aberdeen, King's College, Aberdeen, United Kingdom
AD - Department of Geology and Petroleum Geology, University of Aberdeen, King s College, Aberdeen AB24 3UE, United Kingdom
AB - Fluid inclusions in minerals hold the potential to provide important data on the chemistry of the ambient fluids during mineral precipitation. Especially interesting to astrobiologists are inclusions in low-temperature minerals that may have been precipitated in the presence of microorganisms. We demonstrate that it is possible to obtain data from inclusions in chemosynthetic carbonates that precipitated by the oxidation of organic carbon around methane-bearing seepages. Chemosynthetic carbonates have been identified as a target rock for astrobiological exploration. Other surficial rock types identified as targets for astrobiological exploration include hydrothermal deposits, speleothems, stromatolites, tufas, and evaporites, each of which can contain fluid inclusions. Fracture systems below impact craters would also contain precipitates of minerals with fluid inclusions. As fluid inclusions are sealed microchambers, they preserve fluids in regions where water is now absent, such as regions of the martian surface.

Although most inclusions are <5 μm, the possibility to obtain data from the fluids, including biosignatures and physical remains of life, underscores the advantages of technological advances in the study of fluid inclusions. The crushing of bulk samples could release inclusion waters for analysis, which could be undertaken in situ on Mars. © Mary Ann Liebert, Inc.
KW - Carbonates
KW - Life
KW - Mars
KW - carbonic acid derivative
KW - article
KW - astronomy
KW - chemistry
KW - life
KW - Carbonates
KW - Chemosynthesis
KW - Fluid inclusions
KW - Mars
KW - Planetary exploration strategy
SN - 15311074 (ISSN)
C2 - 12449854
LA - English
J2 - Astrobiology
M3 - Article
DB - Scopus
N1 - Export Date: 23 February 2023; Cited By: 18; Correspondence Address: J. Parnell; Department of Geology and Petroleum Geology, University of Aberdeen, King s College, Aberdeen AB24 3UE, United Kingdom; email: J.Parnell@abdn.ac.uk
ER -

TY - JOUR
AU - Scott, H.P.
AU - Williams, Q.
AU - Ryerson, F.J.
TI - Experimental constraints on the chemical evolution of large icy satellites
PY - 2002
T2 - Earth and Planetary Science Letters
VL - 203
IS - 1
SP - 399
EP - 412
DO - 10.1016/S0012-821X(02)00850-6
UR - https://www.scopus.com/inward/record.uri?eid=2-s2.0-0037107978&doi=10.1016%2fS0012-821X%2802%2900850-6&partnerID=40&md5=2c793e6880b7434ab5f91a5efe477e5d
AD - Department of Earth Science, University of California at Santa Cruz, Santa Cruz, CA 95064, United States
AD - Institute of Geophysics and Planetary Physics, Lawrence Livermore National Laboratory, Livermore, CA, 94550, United States
AB - We derive experimental constraints on the interior structure and mineralogy of large icy satellites by reacting material of chondritic chemistry with water at a pressure of 1.5 GPa,

temperatures between 300°C and 800°C and a range of oxygen fugacities. Our results document the existence of three chemical processes that probably occur in large icy satellites as a result of high pressure hydrothermal processing: (1) the formation of low-density hydrated silicates, (2) the alloying of iron and sulfur to form FeS-dominated cores, and (3) the instability of organic material relative to carbonates. We construct new internal models of the thermal and structural state of Ganymede, and infer that the magnetic field of this body arises from convection within a mostly iron sulfide core. Simple thermochemical calculations are conducted to further explore the likely effects of composition and oxygen fugacity on the high pressure chemistry undergone by organic material within icy satellites. Both experimental and calculated results show that primordial organics are likely to have been largely oxidized to carbonates through hydrothermal processing early in Ganymede’s history, potentially sterilizing Ganymede’s H2O layer.
© 2002 Elsevier Science B.V. All rights reserved.
KW - Callisto
KW - chemical process
KW - hydrothermal activity
KW - ice
KW - satellite
KW - Astrobiology
KW - Callisto
KW - Ganymede satellite
KW - Hydrothermal
KW - Icy satellites
SN - 0012821X (ISSN)
LA - English
J2 - Earth Plan. Sci. Lett.
M3 - Article
DB - Scopus
N1 - Export Date: 23 February 2023; Cited By: 48
ER -

TY - CONF
AU - Henry, O.Y.F.
AU - Piletsky, S.
AU - Grant, W.D.
AU - Sims, M.R.
AU - Cullen, D.C.
TI - Robust molecular imprinted polymer thin-films for an astrobiology biomimetic sensor array
PY - 2002
T2 - European Space Agency, (Special Publication) ESA SP
IS - 518
SP - 513
EP - 514
UR - https://www.scopus.com/inward/record.uri?eid=2-s2.0-0036766604&partnerID=40&md5=40f6e77a79fdade3dccc0a10ea9dcd3c
AD - Cranfield Biotechnology Centre, Cranfield University, Silsoe, Bedfordshire MK45 4DT, United Kingdom
AD - Dept of Microbiology and Immunology, University of Leicester, Leicester LEI 7RH, United Kingdom

AD - Space Research Centre, Dept of Physics and Astronomy, U. of Leicester, Leicester LE1 7RH, United Kingdom
AB - We have previously proposed a concept for a biomimetic sensor array for use in astrobiology planetary-lander missions to search for molecular biomarkers related to extinct or extant life. The concept utilises molecular imprinted polymers (MIPs) as the molecular recognition components. As part of the realisation of the preceding concept, we have investigated the intimate incorporation of MIP thin-films onto transducer relevant surfaces. The use of surface immobilised photo-initiators for the in situ photo-polymerisation of the MIP thin-films resulted in photo-grafted and homogeneous layers with controlled thickness in the range of single to ten's of nanometres suitable for sensing applications. Further physical characterisation of the films after exposure to simulated extreme environments likely in astrobiology-relevant conditions demonstrated the stability of the MIP thin-films. Thus, an underpinning part of the proposed robust biomimetic sensor array is being demonstrated.
KW - Biomarkers
KW - Biomimetic materials
KW - Photopolymerization
KW - Sensors
KW - Thin films
KW - Biomimetic sensor arrays
KW - Astrophysics
SN - 03796566 (ISSN)
LA - English
J2 - Eur Space Agency Spec Publ ESA SP
M3 - Conference paper
DB - Scopus
N1 - Export Date: 23 February 2023; Cited By: 3; Correspondence Address: O.Y.F. Henry; Cranfield Biotechnology Centre, Cranfield University, Silsoe, Bedfordshire MK45 4DT, United Kingdom; email: o.y.f.henry@cranfield.ac.uk; CODEN: ESPUD
ER -

TY - JOUR
AU - Ness, P.K.
AU - Orme, G.M.
TI - Spider-ravine models and plant-like features on mars-possible geophysical and biogeophysical modes of origin
PY - 2002
T2 - JBIS - Journal of the British Interplanetary Society
VL - 55
IS - 3-4
SP - 85
EP - 108
UR - https://www.scopus.com/inward/record.uri?eid=2-s2.0-0142069822&partnerID=40&md5=b672a25fed2cfda3847b182b4402663d
AD - Shinjuku-Ku, Tokyo 160-0012, 18-35 Minami Moto-machi, Japan
AD - Annerley, 4103, QLD, 62 Juliette St., Australia
AB - The Martian South Polar region of Mars is littered with crypto-organic structures, which look like trees and plants. They vary in shape, size and colour seasonally and some may disappear

over winter. A number of models for their origin are proposed and discussed: (1) Dry venting of CO2 gas and dust up joints. (2) Head-ward erosion: Fluid derived from sub-surface layers is expelled up fissures eroding joints to create tributaries capped with mud-like material and/or ice. (3) Modified Clathrate-hydrate model: Structures form as the outside of the flow chills. (4) Hydrothermal-type sources, and (5) Magma pressurizes overlying fluids, expelling mud-like material, hydrothermal fluids or basalt. The morphology of 'spiders' appears to be controlled by bedding and local jointing of the rocks; implying that expelled fluids are derived from within a few hundred metres of the Martian surface. Some spider-ravines modify, some destroy and others create crust in a dynamic near-surface process that mimics subduction zones. Ancient forms of these structures have had a more extensive coverage. Some complex 'spiders' may revert to ravines in the summer. Some valley networks may be formed by similar processes. We consider the possibility that organic material, microbes, or even simple plants might co-exist with these inorganic models.
KW - Astrobiology
KW - Electric connectors
KW - Water
KW - Clathrate hydrate
KW - Hydrothermal
KW - Hydrothermal fluids
KW - Mars
KW - Organic
KW - Organic materials
KW - Organic structures
KW - South polar region
KW - Morphology
KW - Exobiology
KW - Hydrothermal
KW - Jointing
KW - Mars
KW - Organic
KW - Water
PB - British Interplanetary Society
SN - 0007084X (ISSN)
LA - English
J2 - JBIS J. Br. Interplanet. Soc.
M3 - Article
DB - Scopus
N1 - Export Date: 23 February 2023; Cited By: 8; Correspondence Address: P. K. Ness; Shinjuku-Ku, Tokyo 160-0012, 18-35 Minami Moto-machi, Japan; email: phness@sage.ocn.ne.jp
ER -

TY - CONF
AU - Wainwright, M.
AU - Wickramasinghe, N.C.
AU - Narlikar, J.V.
AU - Rajaratnam, P.
TI - Evidence for microorganisms in stratospheric air samples collected at a height of 41 km

PY - 2002
T2 - Proceedings of SPIE - The International Society for Optical Engineering
VL - 4859
SP - 164
EP - 167
DO - 10.1117/12.509505
UR - https://www.scopus.com/inward/record.uri?eid=2-s2.0-0037515770&doi=10.1117%2f12.509505&partnerID=40&md5=1214d0c611a415686cef44d85aa1485a
AD - Dept. of Mol. Biol. and Biotechnol., University of Sheffield, Sheffield, S10 2TN, United Kingdom
AD - Cardiff Centre for Astrobiology, Cardiff, CF10 3DY, 2 North Road, United Kingdom
AD - Inter-Univ. Ctr. Astro. Astrophys., Pune 411 077, Post Bag 4, Ganeshkhind, India
AD - Indian Space Research Organisation, Bangalore 560 094, Antariksh Bhavan, New Bel Road, India
AB - Samples of air removed from the stratosphere, at an altitude of 41km, were previously found to contain viable, but non-cultureable bacteria (cocci and rods). Here, we describe experiments aimed at growing these organisms, together with any others, present in the samples. Two bacteria (Bacillus simplex and Staphylococcus pasteuri) and a single fungus, Engyodontium albus (Limber)de Hoog were isolated from the samples. Contamination can never be ruled out when space-derived samples are studied on earth, however, we are confident that the organisms isolated here originated from the stratosphere.
KW - Aerosols
KW - Air
KW - Balloons
KW - Contamination
KW - Membranes
KW - Microorganisms
KW - Scanning electron microscopy
KW - Surfaces
KW - Upper atmosphere
KW - Bacillus simplex
KW - Exobiology
KW - Non-cultureable bacteria
KW - Panspermia
KW - Staphylococcus pasteuri
KW - Astrophysics
KW - Astrobiology
KW - Exobiology
KW - Extreme environments
KW - Panspermia
SN - 0277786X (ISSN)
LA - English
J2 - Proc SPIE Int Soc Opt Eng
M3 - Conference paper
DB - Scopus
N1 - Export Date: 23 February 2023; Cited By: 1; Correspondence Address: M. Wainwright; Dept. of Mol. Biol. and Biotechnol.,

University of Sheffield, Sheffield, S10 2TN, United Kingdom; email: m.wainwright@sheffield.ac.uk; CODEN: PSISD
ER -

TY - JOUR
AU - Greenberg, R.
AU - Geissler, P.
AU - Hoppa, G.
AU - Tufts, B.R.
TI - Tidal-tectonic processes and their implications for the character of Europa's icy crust
PY - 2002
T2 - Reviews of Geophysics
VL - 40
IS - 2
SP - 1
EP - 1
DO - 10.1029/2000RG000096
UR - https://www.scopus.com/inward/record.uri?eid=2-s2.0-0036628182&doi=10.1029%2f2000RG000096&partnerID=40&md5=915c77105f26ed38c91397a730240566
AD - Lunar and Planetary Laboratory, University of Arizona, Tucson, AZ 85721-0092, 1629 East University Blvd., United States
AB - Jupiter's satellite Europa is covered by an icy crust, likely overlying a deep, global liquid water ocean. The mutually dependent relationship between the orbit and tidal processes controls Europa's rotation, heating, and stress. Observations of the surface and investigation of tidal-tectonic processes indicate that the dominant types of surface terrain have been created by frequent repeated exposure of an underlying ocean to the surface. Surface lineaments, including ubiquitous cycloidal (chains of arcs) features, are correlated with tidal stress patterns, demonstrating that they form by crustal cracking but only if a substantial ocean is present to give adequate tidal amplitude. Stratigraphy of tectonic features, combined with models of how and where they formed, demonstrates a moderate rate of nonsynchronous rotation. Tidal driving of strike-slip displacement (in which surface areas shear passed one another) by daily (85 hours on Europa) tidal stresses suggests that cracks penetrate to a liquid layer. The characteristic ridge sets that cover tectonic terrain may be built by tidal pumping of fluid and slush to the surface on a daily basis. Widespread tectonic dilation creates new surface as material rises from below. In addition to those tectonic terrains, nearly half of the surface is chaotic terrain, with morphology and other characteristics indicative of melt-through from below, consistent with the tectonic indications of thin ice and plausible tidal heating rates. Formation of both chaotic and tectonic terrains has continually resurfaced the satellite, while connecting the ocean to the surface. Surface colorants correlate with locations where ocean water reached the surface, such as along large-scale ridge systems and around chaotic terrain. As a result of tides, liquid water may have bathed crustal cracks and surfaces with heat, transporting and mixing substances vertically. Such daily transported materials would include substances from the oceanic reservoir, oxidants, and fuels created

at the surface, as well as any organisms and their chemical products. Thus a variety of habitable environments likely exist in the crust. Deactivation of individual cracks after thousands of years (due to the tidally driven nonsynchronous rotation) constrains processes like ridge building and strike-slip displacement and would require any living organisms to adapt to such change.
KW - crust
KW - Europa
KW - Jupiter
KW - planetary satellite
KW - planetary surface
KW - tectonics
KW - tide
KW - Astrobiology
KW - Europa
KW - Rotational dynamics
KW - Tectonics
KW - Tidal heating
KW - Tidal stress
PB - Blackwell Publishing Ltd
SN - 87551209 (ISSN)
LA - English
J2 - Rev. Geophys.
M3 - Article
DB - Scopus
N1 - Export Date: 23 February 2023; Cited By: 30; Correspondence Address: R. Greenberg; Lunar and Planetary Laboratory, University of Arizona, Tucson, AZ 85721-0092, 1629 East University Blvd, United States; email: greenberg@lpl.arizona.edu
ER -

TY - CONF
AU - Marsic, D.
AU - Pikuta, E.V.
AU - Hoover, R.B.
AU - Ng, J.D.
TI - Cloning of the 16S ribosomal RNA gene of a psychrophilic bacterium from the Alaskan fox permafrost tunnel
PY - 2002
T2 - Proceedings of SPIE - The International Society for Optical Engineering
VL - 4495
SP - 266
EP - 272
DO - 10.1117/12.454764
UR - https://www.scopus.com/inward/record.uri?eid=2-s2.0-0036397937&doi=10.1117%2f12.454764&partnerID=40&md5=3e1bc0de52efd6c7061a0aca0f3ef7b7
AD - Laboratory for Structural Biology, Department of Biological Sciences, University of Alabama in Huntsville, Huntsville, AL 35899, United States
AB - Extreme cold environments on Earth, such as polar regions or deep ocean harbor a variety of life forms that have developed unique molecular mechanisms that allow them not only to survive, but also

to proliferate under hostile conditions. Such organisms are specially relevant to astrobiology studies because they help determine the environmental limits within which life can exist. They can also have a huge potential for biotechnological applications, because of the unique properties of their macromolecules. In this study we focused on a newly isolated bacterium from the Fox Permafrost Tunnel, FTR1, that grows anaerobically at +2°C. We describe the molecular phylogenetic analysis of this microorganism, through the cloning, sequencing and analysis of its 16S ribosomal RNA gene. Our results suggests that FTR1 is a novel species belonging to the Carnobacterium genus.
KW - Biotechnology
KW - Cloning
KW - Genes
KW - Geographical regions
KW - Macromolecules
KW - Permafrost
KW - RNA
KW - Astrobiology
KW - Carnobacterium
KW - Molecular phylogenetic analysis
KW - Molecular phylogeny
KW - Polar regions
KW - Psychrophilic bacterium
KW - Bacteria
KW - 16S rRNA
KW - Astrobiology
KW - Molecular phylogeny
KW - Permafrost
KW - Psychrophile
SN - 0277786X (ISSN)
LA - English
J2 - Proc SPIE Int Soc Opt Eng
M3 - Conference paper
DB - Scopus
N1 - Export Date: 23 February 2023; Cited By: 3; CODEN: PSISD
ER -

TY - CONF
AU - Mitskevich, I.N.
AU - Poglazova, M.N.
AU - Abyzov, S.S.
TI - The study of the microorganisms preserved in the Central Antarctic glacier in connection with the problems of Astrobiology
PY - 2002
T2 - Proceedings of SPIE - The International Society for Optical Engineering
VL - 4939
SP - 174
EP - 181
DO - 10.1117/12.486689
UR - https://www.scopus.com/inward/record.uri?eid=2-s2.0-0037837540&doi=10.1117%2f12.486689&partnerID=40&md5=d7ff9d71383ea52bc61c6b5741db4037

AD - Institute of Microbiology, Russian Academy of Sciences, 117811, Moscow, Russian Federation
AB - The microbiological investigations of icy formations on the Earth, including Central Antarctic glacier, are closely related to the problem of origin of life on the Earth and other planets, as well as to the problem of transferring of some primitive life forms through the space. The study of the Central Antarctic ice core (50 - 3540 m depth) provided us with the information on abundance and morphological diversity of procaryotic and eucaryotic microorganisms brought on glacier surface by atmospheric currents in different geological epochs. Some microorganisms remained viable owing to the anabiotic state. The incubation of the thawed water of ice core samples showed that the intensity of bacterial propagation depended on several factors including initial cell concentration, organic matter content in the sample and incubation temperature. The quantity of microorganisms increased more noticeably at 20, 25 and 28°c than at 15°C. These results suggest the occurrence of mesophilic microorganisms in the samples from the horizons studied. It is obvious that these microorganisms are resistant to such extremal environments. In addition the results obtained may indirectly evidence for climatic conditions in ancient epochs.
KW - Astrophysics
KW - Bacteria
KW - Earth (planet)
KW - Geology
KW - Glacial geology
KW - Glaciers
KW - Ice
KW - Microorganisms
KW - Anabiosis
KW - Astrobiology
KW - Central antarctic glacier
KW - Eucaryotic microorganism
KW - Exobiology
KW - Procaryotic microorganism
KW - Biology
KW - Anabiosis
KW - Antarctica
KW - Exobiology
KW - Ice
KW - Microorganisms
SN - 0277786X (ISSN)
LA - English
J2 - Proc SPIE Int Soc Opt Eng
M3 - Conference paper
DB - Scopus
N1 - Export Date: 23 February 2023; Cited By: 0; Correspondence Address: I.N. Mitskevich; Institute of Microbiology, Russian Academy of Sciences, 117811, Moscow, Russian Federation; email: mitsk@inmi.msk.ru; CODEN: PSISD
ER -

TY - CONF
TI - Proceedings of SPIE: Instruments, Methods, and Missions for

Astrobiology IV
PY - 2002
T2 - Proceedings of SPIE - The International Society for Optical Engineering
VL - 4495
UR - https://www.scopus.com/inward/record.uri?eid=2-s2.0-0036395163&partnerID=40&md5=3310199eabbd5301f5f554cbb67711c9
AB - This Volume 4495 of the conference proceedings contains 33 papers. Topics discussed include astrobiology, biomarkers and microfossils in ancient rocks and astromaterials, water, ice and permafrost on Mars and Europa, search for organics and life on Mars, origin and transfer of microbial life and microbial extremophiles.
KW - Astronomy
KW - Biology
KW - Biomarkers
KW - Computer simulation
KW - Engineering research
KW - Martian surface analysis
KW - Optical sensors
KW - Permafrost
KW - Remote sensing
KW - Rocks
KW - Space research
KW - Astrobiology
KW - Astromaterials
KW - EiRev
KW - Mars
KW - Microbial life detection
KW - Microcosm
KW - Microfossils
KW - Paleontology
KW - Optical engineering
SN - 0277786X (ISSN)
LA - English
J2 - Proc SPIE Int Soc Opt Eng
M3 - Conference review
DB - Scopus
N1 - Export Date: 23 February 2023; Cited By: 0; CODEN: PSISD
ER -

TY - JOUR
AU - Mautner, M.N.
TI - Planetary bioresources and astroecology. 1. Planetary microcosm bioassays of martian and carbonaceous chondrite materials: Nutrients, electrolyte solutions, and algal and plant responses
PY - 2002
T2 - Icarus
VL - 158
IS - 1
SP - 72
EP - 86
DO - 10.1006/icar.2002.6841
UR - https://www.scopus.com/inward/record.uri?eid=2-s2.0-0036638982&doi=10.1006%2ficar.2002.6841&partnerID=40&md5=0790d8

02f41c61ee76e8499cb316f4e3
AD - Soil, Plant and Ecological Sciences Division, Lincoln University, Canterbury, 8150, New Zealand
AD - Department of Chemistry, University of Canterbury, Christchurch, 8002, New Zealand
AB - The biological fertilities of planetary materials can be assessed using microcosms based on meteorites. This study applies microcosm tests to martian meteorites and analogues and to carbonaceous chondrites. The biological fertilities of these materials are rated based on the soluble electrolyte nutrients, the growth of mesophile and cold-tolerant algae, and plant tissue cultures. The results show that the meteorites, in particular the Murchison CM2 carbonaceous chondrite and DaG 476 martian shergottite, contain high levels of water-extractable Ca, Mg, and SO4-S. The martian meteorites DaG 476 and EETA 79001 also contain higher levels of extractable essential nutrients NO3-N (0.013-0.017 g kg-1) and PO4-P (0.019-0.046 g kg-1) than the terrestrial analogues. The yields of most of the water-extractable electrolytes vary only by factors of 2-3 under a wide range of planetary conditions. However, the long-term extractable phosphate increases significantly under a CO2 atmosphere. The biological yields of algae and plant tissue cultures correlate with extractable NO3-N and PO4-P, identifying these as the limiting nutrients. Mesophilic algae and Asparagus officinalis cultures are identified as useful bioassay agents. A fertility rating system based on microcosm tests is proposed. The results rate the fertilities in the order martian basalts > terrestrial basalt, agricultural soil > carbonaceous chondrites, lava ash > cumulate igneous rock. The results demonstrate the application of planetary microcosms in experimental astroecology to rate planetary materials as targets for astrobiology exploration and as potential space bioresources. For example, the extractable materials in Murchison suggest that concentrated internal solutions in carbonaceous asteroids (3.8 mol L-1 electrolytes and 10 g L-1 organics) can support and disperse microorganisms introduced by natural or directed panspermia in early solar systems. The results also suggest that carbonaceous asteroids and martian basalts can serve as potential future resources for substantial biological populations in the Solar System. © 2002 Elsevier Science (USA).
KW - Algae
KW - Asteroids
KW - Astrobiology
KW - Ecology
KW - Mars
KW - Meteorites
PB - Elsevier
SN - 00191035 (ISSN)
LA - English
J2 - Icarus
M3 - Article
DB - Scopus
N1 - Export Date: 23 February 2023; Cited By: 19; Correspondence Address: M.N. Mautner; Soil Plant/Ecological Sciences Div., Lincoln University, Canterbury 8150, New Zealand; email:

mautnerm@lincoln.ac.nz; CODEN: ICRSA
ER -

TY - JOUR
AU - Crawford, R.L.
AU - Paszczynski, A.
AU - Lang, Q.
AU - Erwin, D.P.
AU - Allenbach, L.
AU - Corti, G.
AU - Anderson, T.J.
AU - Cheng, I.F.
AU - Wai, C.
AU - Barnes, B.
AU - Wells, R.
AU - Assefi, T.
AU - Mojarradi, M.
TI - Measurement of microbial activity in soil by colorimetric observation ofinsitu dye reduction: An approach to detection of extraterrestrial life
PY - 2002
T2 - BMC Microbiology
VL - 2
C7 - 1
SP - 1
EP - 8
DO - 10.1186/1471-2180-2-22
UR - https://www.scopus.com/inward/record.uri?eid=2-s2.0-19044391449&doi=10.1186%2f1471-2180-2-22&partnerID=40&md5=c14c789d61bbf8b25050bd583e450ca1
AD - Environ. Biotechnology Institute, University of Idaho, Moscow, ID 83844, United States
AD - Department of Chemistry, University of Idaho, Moscow, ID 83844, United States
AD - Electrical Engineering, University of Idaho, Moscow, ID 83844, United States
AD - Mechanical Engineering, University of Idaho, Moscow, ID 83844, United States
AD - Microlectron. Res. and Commun. Inst., University of Idaho, Moscow, ID 83844, United States
AD - Jet Propulsion Laboratory, California Institute of Technology, Pasadena, CA, United States
AB - Background: Detecting microbial life in extraterrestrial locations is a goal of space exploration because of ecological and health concerns about possible contamination of other planets with earthly organisms, and vice versa. Previously we suggested a method for life detection based on the fact that living entities require a continual input of energy accessed through coupled oxidations and reductions (an electron transport chain). We demonstrated using earthly soils that the identification of extracted components of electron transport chains is useful for remote detection of a chemical signature of life. The instrument package developed used supercritical carbon dioxide for soil extraction, followed by chromatography or electrophoresis to separate extracted compounds,

with final detection by voltammetry and tandem mass-spectrometry. Results: Here we used Earth-derived soils to develop a related life detection system based on direct observation of a biological redox signature. We measured the ability of soil microbial communities to reduce artificial electron acceptors. Living organisms in pure culture and those naturally found in soil were shown to reduce 2,3-dichlorophenol indophenol (DCIP) and the tetrazolium dye 2,3-bis(2-methoxy-4-nitro-5-sulfophenyl)-2H-tetrazolium-5-carboxanilide inner salt (XTT). Uninoculated or sterilized controls did not reduce the dyes. A soil from Antarctica that was determined by chemical signature and DNA analysis to be sterile also did not reduce the dyes. Conclusion: Observation of dye reduction, supplemented with extraction and identification of only a few specific signature redox-active biochemicals such as porphyrins or quinones, provides a simplified means to detect a signature of life in the soils of other planets or their moons.
KW - Bacteria
KW - Colorimetry
KW - Coloring Agents
KW - Extraterrestrial Environment
KW - Oxidation-Reduction
KW - Porphyrins
KW - Quinones
KW - Soil Microbiology
KW - 2,3 bis(2 methoxy 4 nitro 5 sulfophenyl) 2h tetrazolium 5 carboxanilide
KW - 2,3 dichlorophenol indophenol
KW - carbon dioxide
KW - phenol derivative
KW - tetrazolium
KW - unclassified drug
KW - coloring agent
KW - porphyrin
KW - quinone derivative
KW - article
KW - chemical structure
KW - chromatography
KW - colorimetry
KW - controlled study
KW - DNA determination
KW - electron transport
KW - electrophoresis
KW - microbial activity
KW - microclimate
KW - microorganism detection
KW - nonhuman
KW - observation
KW - oxidation reduction reaction
KW - potentiometry
KW - reduction
KW - respiratory chain
KW - soil
KW - soil microflora
KW - supercritical fluid

KW - tandem mass spectrometry
KW - bacterium
KW - colorimetry
KW - isolation and purification
KW - metabolism
KW - microbiology
PB - BioMed Central Ltd.
SN - 14712180 (ISSN)
C2 - 12150716
LA - English
J2 - BMC Microbiol.
M3 - Article
DB - Scopus
N1 - Export Date: 23 February 2023; Cited By: 8; Correspondence Address: R.L. Crawford; Environ. Biotechnology Institute, University of Idaho, Moscow, ID 83844, United States; email: crawford@uidaho.edu
ER -

TY - CONF
AU - Levin, G.V.
TI - Scientific logic for life on Mars
PY - 2002
T2 - Proceedings of SPIE - The International Society for Optical Engineering
VL - 4495
SP - 81
EP - 88
DO - 10.1117/12.454778
UR - https://www.scopus.com/inward/record.uri?eid=2-s2.0-0036397981&doi=10.1117%2f12.454778&partnerID=40&md5=efe2a57f471333d274c66175e5a94ce3
AD - Spherix Incorporated, Beltsville, MD 20705, 12051 Indian Creek Court, United States
AB - Scientific findings and theoretical analysis over the past several years have increased the probability for extant life on Mars. Discoveries have revealed terrestrial organisms flourishing in environments thought hostile and barren of life. Experiments with extremophile organisms, including some of those newly discovered, have demonstrated their extraordinary and unanticipated hardiness, including under conditions comparable to, or approaching those on present-day Mars. Microorganisms subjected to extreme g forces survived shock as severe as meteoric impact. Calculations and experiments based on Viking data allow for water to be liquid on the surface of Mars for biologically significant periods. Direct observations of Mars by subsequent missions support this likelihood. These new developments provide a workable Panspermia model for the transport, survival and growth of terrestrial life on Mars. No insurmountable obstacles to their survival to the present have been demonstrated. Organisms transported to Mars from Earth and/or from other sources may have been responsible for the positive results returned from Mars by the Viking Labeled Release experiment in 1976. A simple robotic experiment can resolve the issue.
KW - Earth (planet)

KW - Extraterrestrial atmospheres
KW - Microorganisms
KW - Planetary surface analysis
KW - Space research
KW - Extraterrestrial life
KW - Mars
KW - Panspermia model
KW - Terrestrial organisms
KW - Viking mission
KW - Astrophysics
KW - Astrobiology
KW - Extraterrestrial life
KW - Labeled release experiment
KW - Life on Mars
KW - Panspermia
KW - Viking mission
SN - 0277786X (ISSN)
LA - English
J2 - Proc SPIE Int Soc Opt Eng
M3 - Conference paper
DB - Scopus
N1 - Export Date: 23 February 2023; Cited By: 7; Correspondence Address: G.V. Levin; Spherix Incorporated, Beltsville, MD 20705, 12051 Indian Creek Court, United States; email: glevin@spherix.com; CODEN: PSISD
ER -

TY - CONF
AU - Hickman, C.S.
TI - Mollusc-Microbe mutualisms extend the potential for life in hypersaline systems
PY - 2003
T2 - Astrobiology
VL - 3
IS - 3
SP - 631
EP - 644
DO - 10.1089/153110703322610717
UR - https://www.scopus.com/inward/record.uri?eid=2-s2.0-3843053768&doi=10.1089%2f153110703322610717&partnerID=40&md5=2562eb65a8dd072fedea354a0165cb10
AD - Department of Integrative Biology and Museum of Paleontology, University of California, Berkeley, CA 94720-3140, 3060 VLSB, #3140, United States
AB - Metazoans in extreme environments have evolved mutualisms with microbes that extend the physical and chemical capabilities of both partners. Some of the best examples are bivalve molluscs in evaporite and hypersaline settings. Mollusc tissue is developmentally and evolutionarily amenable to housing vast numbers of symbiotic microbes. Documented benefits to the host are nutritional. Multiple postulated benefits to the microbes are related to optimizing metabolic performance at interfaces, where heterogeneity and steep gradients that cannot be negotiated by microbes can be spanned by larger metazoan hosts. A small cockle,

Fragum erugatum, and its photosymbiotic microbes provide a remarkable example of a mutualistic partnership in the hypersaline reaches of Shark Bay, Western Australia. Lucinid bivalves and their endosymbiotic chemolithotrophic bacteria provide examples in which hosts span oxic/anoxic interfaces on behalf of their symbionts at sites of seafloor venting. Multiple lines of evidence underscore the antiquity of mutualisms and suggest that they may have played a significant role in life's first experiments above the prokaryotic grade of complexity. The study of metazoan–microbe mutualisms and their signatures in extreme environments in the geologic record will provide a significant augmentation to microbial models in paleobiology and astrobiology. There are strong potential links between mutualisms and the early history of life on Earth, the persistence of life in extreme environments at times of global crisis and mass extinction, and the possibilities for life elsewhere in the universe. © Mary Ann Liebert, Inc.
KW - Animals
KW - Australia
KW - Biomass
KW - Life
KW - Microbiology
KW - Mollusca
KW - Osmolar Concentration
KW - Photosynthesis
KW - Seawater
KW - Sodium Chloride
KW - Water
KW - Bacteria (microorganisms)
KW - Bivalvia
KW - Chondrichthyes
KW - Fragum erugatum
KW - Lucinidae
KW - Metazoa
KW - Mollusca
KW - Prokaryota
KW - sea water
KW - sodium chloride
KW - water
KW - animal
KW - article
KW - Australia
KW - biomass
KW - life
KW - microbiology
KW - mollusc
KW - osmolarity
KW - photosynthesis
KW - Bivalves
KW - Brine seeps
KW - Chemosymbiosis
KW - Hypersaline
KW - Lagoons
KW - Microbes
KW - Photosymbiosis

SN - 15311074 (ISSN)
C2 - 14678671
LA - English
J2 - Astrobiology
M3 - Conference paper
DB - Scopus
N1 - Export Date: 23 February 2023; Cited By: 5; Correspondence Address: C. S. Hickman; Department of Integrative Biology and Museum of Paleontology, University of California, Berkeley, CA 94720-3140, 3060 VLSB, #3140, United States; email: caroleh@socrates.berkeley.edu
ER -

TY - JOUR
AU - Grady, M.M.
TI - Astrobiology: The search for life beyond the Earth
PY - 2003
T2 - Geology Today
VL - 19
IS - 3
SP - 99
EP - 103
DO - 10.1046/j.1365-2451.2003.00403.x
UR - https://www.scopus.com/inward/record.uri?eid=2-s2.0-0347532680&doi=10.1046%2fj.1365-2451.2003.00403.x&partnerID=40&md5=861000dfdf180294028f56dfd10e49d8
AD - Department of Mineralogy, The Natural History Museum, London SW7 5BD, Cromwell Road, United Kingdom
AB - Over the past decade, planets have been discovered that orbit other stars, a vast ocean has been detected below the icy crust of Jupiter's satellite, Europa, and fossils have been reported in a rock from Mars. On Earth, microorganisms have been identified that inhabit environments previously thought to be too inhospitable to support life. All these observations have helped to define the interdisciplinary subject that has come to be known as astrobiology.
KW - exobiology
KW - extraterrestrial matter
KW - origin of life
SN - 02666979 (ISSN)
LA - English
J2 - Geol. Today
M3 - Article
DB - Scopus
N1 - Export Date: 23 February 2023; Cited By: 2
ER -

TY - JOUR
AU - Chown, M.
TI - The big blue
PY - 2003
T2 - New Scientist
VL - 180
IS - 2421
SP - 38

EP - 41
UR - https://www.scopus.com/inward/record.uri?eid=2-s2.0-1542541373&partnerID=40&md5=2c107c10b32f0f00445dfec79317f906
AB - The depth of any planetary ocean would depend on a complex mixture of factors. The gravitational pull of any planet bigger than this means it would hold onto a shroud of hydrogen and helium, which would turn it into a gas giant. Another factor is the planet's surface temperature. This depends on the internal radioactivity which keeps a planet's interior warm long after the fires of its birth had died out, its proximity to its parent star and the composition of its atmosphere, which may contain heat-trapping greenhouse gases like carbon dioxide and water vapor. With the ocean covering an area almost six times that of Earth's oceans, there could be a volume of water 240 times greater than Earth's.
KW - Extraterrestrial atmospheres
KW - Gravitational effects
KW - Planets
KW - Space research
KW - Extrasolar planets
KW - Extraterrestrial life
KW - Ocean planets
KW - Astronomy
SN - 02624079 (ISSN)
LA - English
J2 - New Sci.
M3 - Article
DB - Scopus
N1 - Export Date: 23 February 2023; Cited By: 0; CODEN: NWSCA
ER -

TY - JOUR
AU - Wainwright, M.
AU - Wickramasinghe, N.C.
AU - Narlikar, J.V.
AU - Rajaratnam, P.
TI - Microorganisms cultured from stratospheric air samples obtained at 41 km
PY - 2003
T2 - FEMS Microbiology Letters
VL - 218
IS - 1
SP - 161
EP - 165
DO - 10.1016/S0378-1097(02)01138-2
UR - https://www.scopus.com/inward/record.uri?eid=2-s2.0-0037458181&doi=10.1016%2fS0378-1097%2802%2901138-2&partnerID=40&md5=d0bdd47ab0e84685500eaa59f8719409
AD - Dept. of Molec. Biol./Biotech., University of Sheffield, Sheffield S10 2TN, United Kingdom
AD - Cardiff Centre for Astrobiology, Cardiff University, Cardiff CT10 3DY, 2 North Road, United Kingdom
AD - Inter-Univ. Ctr. Astron./Astrophys., Ganeshkhind, Pune 411 007, Post Bag 4, India
AD - Indian Space Research Organisation, Bangalore 560 094,

Antariksh Bhavan, New Bel Road, India
AB - Samples of air removed from the stratosphere, at an altitude of 41 km, were previously found to contain viable, but non-cultureable bacteria (cocci and rods). Here, we describe experiments aimed at growing these, together with any other organisms, present in these samples. Two bacteria (Bacillus simplex and Staphylococcus pasteuri) and a single fungus, Engyodontium album (Limber) de Hoog were isolated from the samples. Although the possibility of contamination can never be ruled out when space-derived samples are studied on earth, we are confident that the organisms originated from the stratosphere. Possible mechanisms by which these organisms could have attained such a height are discussed. © 2002 Federation of European Microbiological Societies. Published by Elsevier Science B.V. All rights reserved.
KW - Altitude
KW - Atmosphere
KW - Bacillus
KW - Environment
KW - Extraterrestrial Environment
KW - Fungi
KW - Phylogeny
KW - Staphylococcus
KW - Bacillus simplex
KW - Engyodontium album
KW - Fungi
KW - Staphylococcus pasteuri
KW - air sampling
KW - article
KW - Bacillus
KW - bacillus simplex
KW - bacterium culture
KW - bacterium isolation
KW - controlled study
KW - engyodontium album
KW - fungus
KW - fungus culture
KW - fungus isolation
KW - microbial contamination
KW - microbial growth
KW - microorganism
KW - nonhuman
KW - priority journal
KW - Staphylococcus
KW - staphylococcus pasteuri
KW - stratosphere
KW - Astrobiology
KW - Exobiology
KW - Extreme environment
KW - Panspermia
SN - 03781097 (ISSN)
C2 - 12583913
LA - English
J2 - FEMS Microbiol. Lett.
M3 - Article

DB - Scopus
N1 - Export Date: 23 February 2023; Cited By: 148; Correspondence Address: M. Wainwright; Dept. of Molec. Biol./Biotech., University of Sheffield, Sheffield S10 2TN, United Kingdom; email: m.wainwright@sheffield.ac.uk; CODEN: FMLED
ER -

TY - CHAP
AU - Grady, M.M.
TI - The physical constraints on extraterrestrial life
PY - 2003
T2 - Evolution on Planet Earth: The Impact of the Physical Environment
SP - 397
EP - 414
DO - 10.1016/B978-012598655-7/50048-3
UR - https://www.scopus.com/inward/record.uri?eid=2-s2.0-84904077454&doi=10.1016%2fB978-012598655-7%2f50048-3&partnerID=40&md5=60152ff7347eaf5b1622fd841fe74d4f
AD - Department of Mineralogy, The Natural History Museum, London SW7 5BD, Cromwell Road, United Kingdom
AB - This chapter discusses the origin and evolution of life on the Earth and the possibilities for analogous developments in other planetary environments within the solar system and beyond. Terrestrial fossil record indicates that life on the Earth started up the evolutionary pathway only a matter of a few hundreds of millions of years following earth's differentiation, although it has been suggested that primitive life might have started more than once during this first epoch, only to be eliminated during periods of bombardment. Owing to the range of conditions over which microbes exist and flourish on the Earth, it seems fairly clear that there are many niches throughout the solar system that could provide a suitable habitat in which life might emerge. As a starting point to the search for life elsewhere in the solar system, the environments in which life exists on the Earth can be examined, and where similar environments might exist elsewhere both now and in the past can also be answered. It is far from clear that life exists on other bodies, which is why there is currently so much interest in the study of exobiology and the planning of space missions to investigate and explore other worlds. © 2003 Elsevier Ltd. All rights reserved.
PB - Elsevier Ltd
SN - 978-012598655-7 (ISBN)
LA - English
J2 - Evol. on Planet Earth: The Impact of the Phys. Environ.
M3 - Book chapter
DB - Scopus
N1 - Export Date: 23 February 2023; Cited By: 0
ER -

TY - CONF
AU - Trombka, J.I.
AU - McCoy, T.J.
AU - Starr, R.D.
AU - Schweitzer, J.

AU - Eisen, Y.
AU - Evans, L.G.
AU - Hoover, R.
AU - Floyd, S.
AU - Lim, L.
AU - Groves, J.
AU - James, R.B.
TI - The use of neutron/gamma-ray techniques for astrobiological and geochemical exploration of planetary bodies
PY - 2003
T2 - IEEE Aerospace Conference Proceedings
VL - 2
C7 - 1235468
SP - 581
EP - 595
DO - 10.1109/AERO.2003.1235468
UR - https://www.scopus.com/inward/record.uri?eid=2-s2.0-84879342650&doi=10.1109%2fAERO.2003.1235468&partnerID=40&md5=b4787f8b89763d7b0ea9da67393ddad5
AD - NASA Goddard Space Flight Center, Code 691, Greenbelt, MD 20771, United States
AD - Nat. Museum Natural History, Dept. Mineral Sciences, Smithsonian Institution, Washington, DC 20560, United States
AD - Catholic University of America, Washington, DC 20064, 620 Michigan Avenue, United States
AD - University of Connecticut, Unit 3046, Stems, CT 06269-3046, 2152 Hillside Road, United States
AD - SOREQIsrael Atomic Energy Com., SOREQ NRC, Yavne, 81800, Israel
AD - Computer Sciences Corporation, Lanham-Seabrook, MD 20706, 7700 Hubble Drive, United States
AD - NASA Marshall Space Flight Center, Space Sciences Laboratory, ES-82, Huntsville, AL, 35812, United States
AD - EMR Photoelectric, Princeton Junction, NJ 08550, Wallace Road, United States
AD - Brookhaven National Laboratory, Upton, NY 11973-5000, United States
AB - Orbital and in situ measurements of characteristic gamma-ray and neutron emission spectra are used in geochemical explorations of condensed bodies in our solar system. Orbital studies measure the characteristic gamma-ray emissions produced by the natural radioactive elements K, Th, and U, and the secondary cosmic-ray induced gamma-ray and neutron emissions. The Moon, Mars, Eros, and Phobos have been studied successfully using orbital gamma-ray and neutron spectrometers. A neutrodgamma-ray system to identify samples of geochemical or astrobiological interest for further in situ analysis or potential sample return is of great interest. This instrument could determine the presence of sub-surface water (permafrost) and pinpoint areas of biological interest. © 2003 IEEE.
KW - Geochemistry
KW - Neutron emission
KW - Uranium
KW - Gamma-ray emission
KW - Geochemical exploration

KW - In-situ analysis
KW - In-situ measurement
KW - Neutron emission spectrum
KW - Planetary bodies
KW - Sample return
KW - Sub-surfaces
KW - Gamma rays
SN - 1095323X (ISSN); 078037651X (ISBN); 978-078037651-9 (ISBN)
LA - English
J2 - IEEE Aerosp. Conf. Proc.
M3 - Conference paper
DB - Scopus
N1 - Export Date: 23 February 2023; Cited By: 0
ER -

TY - CONF
AU - Tang, C.M.
AU - Roopnarine, P.D.
TI - Complex morphological variability in complex evaporitic systems: Thermal spring snails from the chihuahuan desert, Mexico
PY - 2003
T2 - Astrobiology
VL - 3
IS - 3
SP - 597
EP - 607
DO - 10.1089/153110703322610681
UR - https://www.scopus.com/inward/record.uri?eid=2-s2.0-3843090771&doi=10.1089%2f153110703322610681&partnerID=40&md5=6fe2c4aeef1d33d1c3388794e604ad26
AD - Invertebrate Zoology and Geology, California Academy of Sciences, San Francisco, CA 94118, Golden Gate Park, United States
AB - Thermal springs in evaporitic environments provide a unique biological laboratory in which to study natural selection and evolutionary diversification. These isolated systems may be an analogue for conditions in early Earth or Mars history. One modern example of such a system can be found in the Chihuahuan Desert of north-central Mexico. The Cuatro Cienegas basin hosts a series of thermal springs that form a complex of aquatic ecosystems under a range of environmental conditions. Using landmark-based morphometric techniques, we have quantified an unusually high level of morphological variability in the endemic gastropod Mexipyrgus from Cuatro Cienegas. The differentiation is seen both within and between hydrological systems. Our results suggest that this type of environmental system is capable of producing and maintaining a high level of morphological diversity on small spatial scales, and thus should be a target for future astrobiological research. © Mary Ann Liebert, Inc.
KW - Animals
KW - Desert Climate
KW - Environment
KW - Geography
KW - Hot Springs
KW - Life

KW - Mexico
KW - Phylogeny
KW - Water
KW - Gastropoda
KW - Hydrobiidae
KW - Mexipyrgus
KW - Mollusca
KW - water
KW - animal
KW - article
KW - chemistry
KW - desert climate
KW - environment
KW - geography
KW - life
KW - Mexico
KW - phylogeny
KW - thermal spring
KW - Biology
KW - Evolutionary
KW - Hydrobiidae
KW - Mollusca
KW - Morphometrics
SN - 15311074 (ISSN)
C2 - 14678668
LA - English
J2 - Astrobiology
M3 - Conference paper
DB - Scopus
N1 - Export Date: 23 February 2023; Cited By: 8; Correspondence Address: C. M. Tang; Invertebrate Zoology and Geology, California Academy of Sciences, San Francisco, CA 94118, Golden Gate Park, United States; email: ctang@calacademy.org
ER -

TY - JOUR
AU - McKinnon, W.B.
AU - Zolensky, M.E.
TI - Sulfate content of Europa's ocean and shell: Evolutionary considerations and some geological and astrobiological implications
PY - 2003
T2 - Astrobiology
VL - 3
IS - 4
SP - 879
EP - 897
DO - 10.1089/153110703322736150
UR - https://www.scopus.com/inward/record.uri?eid=2-s2.0-3242806659&doi=10.1089%2f153110703322736150&partnerID=40&md5=20eb386da551df580bd2865b96446092
AD - Department of Earth and Planetary Sciences, McDonnell Center for the Space Sciences, Washington University, St. Louis, MO 63130, United States
AD - NASA Johnson Spacecraft Center, Houston, TX, United States

AB - Recent models for the origin of Jupiter indicate that the Galilean satellites were mostly derived from largely unprocessed solar nebula solids and planetesimals. In the jovian subnebula the solids that built Europa were first heated and then cooled, but the major effect was most likely partial or total devolatilization, and less likely to have been wholesale thermochemical reprocessing of rock + metal compositions (e.g., oxidation of Fe and hydration of silicates). Ocean formation and substantial alteration of interior rock by accreted water and ice would occur during and after accretion, but none of the formation models predicts or implies accretion of sulfates. Europa's primordial ocean was most likely sulfidic. After accretion and later radiogenic and tidal heating, the primordial ocean would have interacted hydrothermally with subjacent rock. It has been hypothesized that sulfides could be converted to sulfates if sufficient hydrogen was lost to space, but pressure effects and the impermeability of serpentinite imply that extraction of sulfate from thoroughly altered Europa-rock would have been inefficient (if indeed Mg sulfates formed at all). Permissive physical limits on the extent of alteration limit the sulfate concentration of Europa's evolved ocean to 10% by weight MgSO 4 or equivalent. Later oxidation of the deep interior of Europa may have also occurred because of water released by the breakdown of hydrated silicates, ultimately yielding S magma and/or SO 2 gas. Geological and astrobiological implications are considered. © Mary Ann Liebert, Inc.
KW - Carbon
KW - Electrons
KW - Evolution
KW - Exobiology
KW - Extraterrestrial Environment
KW - Ice
KW - Iron
KW - Jupiter
KW - Magnesium Sulfate
KW - Models, Chemical
KW - Oceans and Seas
KW - Oxygen
KW - Pressure
KW - Scattering, Radiation
KW - Sulfates
KW - Sulfur Oxides
KW - Temperature
KW - Water
KW - carbon
KW - ice
KW - iron
KW - magnesium sulfate
KW - oxygen
KW - sulfate
KW - sulfur oxide
KW - water
KW - article
KW - astronomy
KW - biology

KW - chemical model
KW - chemistry
KW - electron
KW - evolution
KW - microclimate
KW - pressure
KW - radiation scattering
KW - sea
KW - temperature
KW - Accretion
KW - Europa
KW - Extraterrestrial Oceans
KW - Hydrothermal Systems
KW - Io
KW - Meteorites
KW - Sulfate
SN - 15311074 (ISSN)
C2 - 14987488
LA - English
J2 - Astrobiology
M3 - Article
DB - Scopus
N1 - Export Date: 23 February 2023; Cited By: 74; Correspondence Address: W. B. McKinnon; Department of Earth and Planetary Sciences, McDonnell Center for the Space Sciences, Washington University, St. Louis, MO 63130, United States; email: mckinnon@wustl.edu
ER -

TY - JOUR
AU - Greeley, R.
AU - Kuzmin, R.O.
AU - Nelson, D.M.
AU - Farmer, J.D.
TI - Eos Chasma, Mars: Regional setting for a potential landing site for astrobiology
PY - 2003
T2 - Journal of Geophysical Research: Planets
VL - 108
IS - 12
SP - ROV24
EP - 1
DO - 10.1029/2002je002014
UR - https://www.scopus.com/inward/record.uri?eid=2-s2.0-1542349160&doi=10.1029%2f2002je002014&partnerID=40&md5=1afad2327c0e8908749dff578699dcdf
AD - Department of Geological Sciences, Arizona State University, Tempe, AZ 85287-1404, United States
AD - Vernadsky Institute, Russian Academy of Sciences, Moscow 117975, Kosygin St 19, Russian Federation
AB - The floor of Eos Chasma, centered at about 13.4°S and 41.5°W at an elevation of -3.5 km, is proposed as a future landing site on Mars. Deposits in this area were derived from a variety of sources, including chaotic terrain (interpreted to reflect the release of subsurface water) and possible paleolake materials, which could shed

light on the astrobiological potential of Mars, climate history, and the evolution of the surface. Analyses of the physical properties of the proposed site suggest a rock abundance of 10-18% (based on Viking Orbiter Infrared Thermal Mapper data), set in a matrix of medium to coarse sands (based on Mars Global Surveyor Thermal Emission Spectrometer data), sparse covering by dust (based on albedo measurements), and a surface roughness of <1 to 1.7 in (derived from the Mars Orbiter Laser Altimeter). Although these attributes are within the engineering constraints for current lander designs, models of the atmosphere suggest that wind shear (generated primarily by the proximity to local topography, i.e., the Chasma canyon wall) might preclude the site with current landing systems. Copyright 2003 by the American Geophysical Union.
KW - exobiology
KW - exploration
KW - Mars
KW - paleohydrology
KW - Mars astrobiology
KW - Mars Eos Chasma
KW - Mars landing site
PB - American Geophysical Union
SN - 01480227 (ISSN)
LA - English
J2 - J. Geophys. Res. E Planets
M3 - Article
DB - Scopus
N1 - Export Date: 23 February 2023; Cited By: 6; Correspondence Address: R. Greeley; Department of Geological Sciences, Arizona State University, Temple, AZ 85287-1404, United States; email: greeley@asu.edu
ER -

TY - JOUR
AU - Hill, H.G.M.
AU - Nuth, J.A.
TI - The catalytic potential of cosmic dust: Implications for prebiotic chemistry in the solar nebula and other protoplanetary systems
PY - 2003
T2 - Astrobiology
VL - 3
IS - 2
SP - 291
EP - 304
DO - 10.1089/153110703769016389
UR - https://www.scopus.com/inward/record.uri?eid=2-s2.0-1842864474&doi=10.1089%2f153110703769016389&partnerID=40&md5=f511638e9cb6b58552ee23861d5e9087
AD - International Space University, Strasbourg Central Campus, 67400 Illkirch-Graffenstaden, Parc d'Innovation, France
AD - Laboratory for Extraterrestrial Physics, NASA Goddard Space Flight Center, Greenbelt, MD, United States
AB - The synthesis of important prebiotic molecules is fundamentally reliant on basic starting ingredients: water, organic

species [e.g., methane (CH 4)], and reduced nitrogen compounds [e.g., ammonia (NH3), methyl cyanide (CH3CN) etc.]. However, modern studies conclude that the primordial Earth's atmosphere was too rich in CO, CO2, and water to permit efficient synthesis of such reduced molecules as envisioned by the classic Miller-Urey experiment. Other proposed sources of terrestrial nitrogen reduction, like those within submarine vent systems, also seem to be inadequate sources of chemically reduced C-H-O-N compounds. Here, we demonstrate that nebular dust analogs have impressive catalytic properties for synthesizing prebiotic molecules. Using a catalyst analogous to nebular iron silicate condensate, at temperatures ranging from 500K to 900K, we catalyzed both the Fischer-Tropsch conversion of CO and H2 to methane and water, and the corresponding Haber-Bosch synthesis of ammonia from N2 and H 2. Remarkably, when CO, N2, and H2 were allowed to react simultaneously, these syntheses also yielded nitrogen-containing organics such as methyl amine (CH3NH2), acetonitrile (CH3CN), and N-methyl methylene imine (H3CNCH 2). A fundamental consequence of this work for astrobiology is the potential for a natural chemical pathway to produce complex chemical building blocks of life throughout our own Solar System and beyond.
© Mary Ann Liebert, Inc.
KW - Biogenesis
KW - Carbon Dioxide
KW - Catalysis
KW - Cosmic Dust
KW - Extraterrestrial Environment
KW - Organic Chemicals
KW - Planets
KW - Solar System
KW - Water
KW - carbon dioxide
KW - organic compound
KW - water
KW - article
KW - astronomy
KW - biogenesis
KW - catalysis
KW - microclimate
KW - Astrochemistry
KW - Catalysis
KW - Fischer-Tropsch synthesis
KW - Haber-Bosch synthesis
KW - Interstellar dust
KW - Origin of life
KW - Prebiotic molecules
SN - 15311074 (ISSN)
C2 - 14577878
LA - English
J2 - Astrobiology
M3 - Article
DB - Scopus
N1 - Export Date: 23 February 2023; Cited By: 56; Correspondence Address: H. G. M. Hill; International Space University, Strasbourg Central Campus, 67400 Illkirch-Graffenstaden, Parc d'Innovation,

France; email: hill@isu.isunet.edu
ER -

TY - JOUR
AU - Lappin, G.
TI - Biology meets Nuclear Physics
PY - 2003
T2 - Biologist
VL - 50
IS - 4
SP - 183
EP - 187
UR - https://www.scopus.com/inward/record.uri?eid=2-s2.0-0043074451&partnerID=40&md5=f6e206a06d7b0c4974b1f69a5505d4cc
AD - Xceleron Ltd., University of York, York, United Kingdom
AB - This is the story of radioactive carbon and the incalculable knowledge that has resulted from its discovery. Radioactive carbon is used routinely to elucidate biochemical pathways, date archaeological artefacts and to follow the currents of the atmosphere and oceans. The story takes us into the murky world of quantum theory and touches even upon the search for extraterrestrial life.
SN - 00063347 (ISSN)
LA - English
J2 - Biologist
M3 - Review
DB - Scopus
N1 - Export Date: 23 February 2023; Cited By: 0; Correspondence Address: G. Lappin; Xceleron Ltd., University of York, York, United Kingdom; email: graham.lappin@alltox.com; CODEN: BLGTB
ER -

TY - JOUR
AU - Horiuchi, T.
AU - Kobayashi, K.
AU - Takano, Y.
AU - Marumo, K.
AU - Nakashima, M.
AU - Yamagishi, A.
AU - Ishibashi, J.
AU - Urabe, T.
TI - Organics in chimneys and water samples from deep-sea hydrothermal systems: implications for sub-vent biosphere.
PY - 2003
T2 - Biological sciences in space = Uchū seibutsu kagaku
VL - 17
IS - 3
SP - 190
EP - 191
UR - https://www.scopus.com/inward/record.uri?eid=2-s2.0-47149096918&partnerID=40&md5=7006376bed6476594b71e3aac4d8ad77
AD - Department of Chemistry and Biotechnology, Yokohama National University., Japan
AB - Searching for life in extreme terrestrial environments can be

a model of that for extraterrestrial life. Submarine hydrothermal system is one of promising sites for the frontier of life on the earth. Here seawater and vent chimnies were collected from deep-sea hydrothermal vents at Suiyo Seamount, Izu-bonin arc, Pacific Ocean as a part of Archaean Park Project. Pure seawater sample of 300 degrees C (purity>97%) could be collected. Dissolved and total hydrolyzable amino acids were determined by ion-exchange HPLC, and their enantiomeric ratio was measured by reversed-phase HPLC for the first time. Glycine and serine were two most abundant amino acids, followed by other proteinous amino acids such as alanine, glutamic acid and aspartic acid. Non-proteinous amino acids were detected as minor constituents. Most of the amino acids detected were of the L-form. Thus amino acids of abiotic origin were quite minor, and most of the amino acids detected were formed biologically. These results, together with analytical results of the vent chimney samples, suggest that there is active microbial activities near the hydrothermal systems.
KW - Amino Acids
KW - Heat
KW - Pacific Ocean
KW - Seawater
KW - Water Microbiology
KW - amino acid
KW - sea water
KW - article
KW - chemistry
KW - heat
KW - microbiology
KW - Pacific Ocean
SN - 09149201 (ISSN)
C2 - 14676368
LA - English
J2 - Biol. Sci. Space
M3 - Article
DB - Scopus
N1 - Export Date: 23 February 2023; Cited By: 0
ER -

TY - JOUR
AU - Ida, S.
TI - Extrasolar terrestrial planets and possibility of extraterrestrial life
PY - 2003
T2 - Biological sciences in space = Uchū seibutsu kagaku
VL - 17
IS - 4
SP - 318
EP - 323
DO - 10.2187/bss.17.318
UR - https://www.scopus.com/inward/record.uri?eid=2-s2.0-21644441710&doi=10.2187%2fbss.17.318&partnerID=40&md5=c9c49d4d6891454617977ffdeb8e31fc
AD - Tokyo Institute of Technology., Japan
AB - Recent development of research on extrasolar planets are

reviewed. About 120 extrasolar Jupiter-mass planets have been discovered through the observation of Doppler shift in the light of their host stars that is caused by acceleration due to planet orbital motions. Although the extrasolar planets so far observed may be limited to gas giant planets and their orbits differ from those of giant planets in our Solar system (Jupiter and Saturn), the theoretically predicted probability of existence of extrasolar terrestrial planets that can have liquid water ocean on their surface is comparable to that of detectable gas giant planets. Based on the number of extrasolar gas giants detected so far, about 100 life-sustainable planets may exist within a range of 200 light years. Indirect observation of extrasolar terrestrial planets would be done with space telescopes within several years and direct one may be done within 20 years. The latter can detect biomarkers on these planets as well.
KW - Astronomy
KW - Exobiology
KW - Extraterrestrial Environment
KW - Life
KW - Planets
KW - Space Flight
KW - Water
KW - water
KW - astronomy
KW - biology
KW - life
KW - microclimate
KW - review
KW - space flight
SN - 09149201 (ISSN)
C2 - 15136756
LA - Japanese
J2 - Biol. Sci. Space
M3 - Review
DB - Scopus
N1 - Export Date: 23 February 2023; Cited By: 0
ER -

TY - JOUR
AU - Gilichinsky, D.A.
AU - Rivkina, E.M.
AU - Laurinavichius, K.S.
AU - Shcherbakova, V.A.
AU - Komarov, I.A.
AU - Volkov, N.G.
TI - Cryopegs and their inhabitants - The model for astrobiology
PY - 2003
T2 - Earth's Cryosphere
VL - 7
IS - 3
SP - 73
EP - 84
UR - https://www.scopus.com/inward/record.uri?eid=2-s2.0-19544374802&partnerID=40&md5=1494dea82c294bfda7026ef7a2f4143e

AD - Institute of Physicochemical and Biological Problems in Soil Sciences, RAS, Pushchino, Moscow Region, Russian Federation
AD - Institute of Biochemistry and Physiology of Microorganisms, RAS, Pushchino, Moscow Region, Russian Federation
AD - Moscow State University, 119992 Moskow, Leninskiye Gory, Russian Federation
AB - The present study for the first time describes the ancient ecosystem represented by overcooled water lenses (cryopegs) and their Pleistocene inhabitants. The total number of microorganisms found in cryopeg is close to that of common aquatic habitats. This is a psychrophilic halotolerance-halophilic microbial community, metabolically active at subzero temperatures and high salinity. The fact of viable bacteria surviving in aquatic environment throughout its geologic history has general biological and astrobiological importance. The mineralized brines are the only free water that might exist within Martian subsurface permafrost. This is why the isolated microbial community might represent the prototype of plausible Martian life forms. © D.A. Gilichinsky, E.M. Rivkina, K.S. Laurinavichius, V.A. Shcherbakova, I.A. Komarov, N.G. Volkov, 2003.
KW - Bacteria (microorganisms)
KW - aquatic ecosystem
KW - aquatic environment
KW - microbial activity
KW - microbial community
KW - microorganism
KW - permafrost
KW - Pleistocene
KW - Astrobiology
KW - Mars
KW - Metabolic activity
KW - Permafrost
KW - Viable bacteria
KW - Water brines
KW - Water ecosystem
PB - Russian Academy of Sciences
SN - 15607496 (ISSN)
LA - Russian
J2 - Earth's Cryosphere
M3 - Article
DB - Scopus
N1 - Export Date: 23 February 2023; Cited By: 4
ER -

TY - JOUR
AU - Irwin, L.N.
AU - Schulze-Makuch, D.
TI - Strategy for modeling putative multilevel ecosystems on Europa
PY - 2003
T2 - Astrobiology
VL - 3
IS - 4
SP - 813
EP - 821
DO - 10.1089/153110703322736114

UR - https://www.scopus.com/inward/record.uri?eid=2-s2.0-4444318607&doi=10.1089%2f153110703322736114&partnerID=40&md5=c2c5c3dc8d5fb51a96b803f841c87115
AD - Department of Biological Sciences, University of Texas at El Paso, El Paso, TX 79968-0159, 500 West University Avenue, United States
AD - Department of Geological Sciences, University of Texas at El Paso, El Paso, TX, United States
AB - A general strategy for modeling ecosystems on other worlds is described. Two alternative biospheres beneath the ice surface of Europa are modeled, based on analogous ecosystems on Earth in potentially comparable habitats, with reallocation of biomass quantities consistent with different sources of energy and chemical constituents. The first ecosystem models a benthic biosphere supported by chemoautotrophic producers. The second models two concentrations of biota at the top and bottom of the subsurface water column supported by energy harvested from transmembrane ionic gradients. Calculations indicate the plausibility of both ecosystems, including small macroorganisms at the highest trophic levels, with ionotrophy supporting a larger biomass than chemoautotrophy. © Mary Ann Liebert, Inc.
KW - Biomass
KW - Carbon
KW - Carbon Dioxide
KW - Earth (Planet)
KW - Ecosystem
KW - Exobiology
KW - Extraterrestrial Environment
KW - Hydrogen
KW - Ice
KW - Ions
KW - Jupiter
KW - Methane
KW - Models, Theoretical
KW - Oceans and Seas
KW - Water
KW - carbon
KW - carbon dioxide
KW - hydrogen
KW - ice
KW - ion
KW - methane
KW - water
KW - article
KW - astronomy
KW - biology
KW - biomass
KW - chemistry
KW - ecosystem
KW - microclimate
KW - sea
KW - theoretical model
KW - Ecosystem
KW - Europa

KW - Extraterrestrial Life
KW - Planetary Ocean
SN - 15311074 (ISSN)
C2 - 14987484
LA - English
J2 - Astrobiology
M3 - Article
DB - Scopus
N1 - Export Date: 23 February 2023; Cited By: 19; Correspondence Address: L. N. Irwin; Department of Biological Sciences, University of Texas at El Paso, El Paso, TX 79968-0159, 500 West University Avenue, United States; email: lirwin@utep.edu
ER -

TY - JOUR
AU - Tokano, T.
TI - Precession-driven migration of water in the surficial layers of Mars
PY - 2003
T2 - International Journal of Astrobiology
VL - 2
IS - 3
SP - 155
EP - 170
DO - 10.1017/S1473550403001629
UR - https://www.scopus.com/inward/record.uri?eid=2-s2.0-2342490778&doi=10.1017%2fS1473550403001629&partnerID=40&md5=2e5c732dd7de5ec12df3813b2c33b2de
AD - DLR, Institut für Raumsimulation, 51170 Köln, Germany
AB - A linear correlation analysis between the Mars Odyssey neutron fluxes and various surface parameters indicates that the annual maximum surface temperature is the most important factor controlling the soil water content in the surficial (upper few tens of centrimetres) layers of the Martian soil. This is likely to be associated with the higher enthalpy of hydration of minerals in comparison with the enthalpy of sublimation of ice, which is presumably almost absent in the surficial layer. While presently the maximum surface temperature occurs near 30° S because of perihelion in late southern spring, the season of perihelion periodically migrates by virtue of precession. Consequently, the maximum surface temperature as well as the driest place on Mars should move from one hemisphere to the other with a period of about 51 000 yr. A significant amount of surficial (adsorbed) water would then be exchanged between the hemispheres and between the soil and other reservoirs, especially the polar caps and the polar layered deposits, and is probably borne out by the stratigraphic structure of these deposits. It is suggested that the water migration driven by the orbital eccentricity and precession may be as important as the obliquity-driven exchange of water, particularly very close to the surface, where ground ice is unstable. The zones of liquid water stability oscillate somewhat in a north–south direction in the course of the precession cycle, but are most prevalent in parts of the low and mid northern latitudes as well as in the Hellas Basin. The thermal stability of liquid water tends to be high where the

near-surface soil water content is low, indicating that the periodic
melting of ground ice by solar heating is not a likely source of
liquid water on the surface, but some episodic processes should
provide water, if any. An enhanced soil water content near the
surface is always accompanied with a reduced peak ultraviolet flux,
both reducing the chemical reactivity of the soil. In the present
epoch the northern hemisphere may represent astrobiologically more
clement environmental conditions. © 2003, Cambridge University
Press. All rights reserved.
KW - Mars
KW - water
SN - 14735504 (ISSN)
LA - English
J2 - Int. J. Astrobiology
M3 - Article
DB - Scopus
N1 - Export Date: 23 February 2023; Cited By: 7
ER -

TY - JOUR
AU - Gormly, S.
AU - Adams, V.D.
AU - Marchand, E.
TI - Physical simulation for low-energy astrobiology environmental
scenarios
PY - 2003
T2 - Astrobiology
VL - 3
IS - 4
SP - 761
EP - 770
DO - 10.1089/153110703322736088
UR - https://www.scopus.com/inward/record.uri?eid=2-
s2.0-4444364264&doi=10.1089%2f153110703322736088&partnerID=40&md5=05
10c02606c6d753fbda1362e1012343
AD - Department of Civil and Environmental Engineering, University
of Nevada, Reno, NV, United States
AD - Tetra Tech EM, Inc., Reno, NV 89502, 1325 Airmotive Way,
United States
AB - Speculations about the extent of life of independent origin
and the potential for sustaining Earth-based life in subsurface
environments on both Europa and Mars are of current and relevant
interest. Theoretical modeling based on chemical energetics has
demonstrated potential options for viable biochemical metabolism
(metabolic pathways) in these types of environments. Also, similar
environments on Earth show microbial activity. However, actual
physical simulation testing of specific environments is required to
confidently determine the interplay of various physical and chemical
parameters on the viability of relevant metabolic pathways. This
testing is required to determine the potential to sustain life in
these environments on a specific scenario by scenario basis. This
study examines the justification, design, and fabrication of, as
well as the culture selection and screening for, a psychrophilic/
halophilic/anaerobic digester. This digester is specifically

designed to conform to physical testing needs of research relating to potential extent physical environments on Europa and other planetary bodies in the Solar System. The study is a long-term effort and is currently in an early phase, with only screening-level data at this time. Full study results will likely take an additional 2 years. However, researchers in electromagnetic biosignature and in situ instrument development should be aware of the study at this time, as they are invited to participate in planning for future applications of the digester facility. © Mary Ann Liebert, Inc.
KW - Acetates
KW - Evolution, Planetary
KW - Exobiology
KW - Extraterrestrial Environment
KW - Jupiter
KW - Life
KW - Life Support Systems
KW - Mars
KW - Meteoroids
KW - Models, Chemical
KW - Oceans and Seas
KW - Planets
KW - Salts
KW - Solar System
KW - Sulfates
KW - acetic acid derivative
KW - brine
KW - inorganic salt
KW - sulfate
KW - article
KW - astronomy
KW - biology
KW - chemical model
KW - chemistry
KW - life
KW - metabolism
KW - methodology
KW - microclimate
KW - sea
KW - Anaerobic Digestion
KW - Biological Sulfate Reduction
KW - Environmental Simulation
KW - Europa
KW - Halophile
KW - Psychrophile
SN - 15311074 (ISSN)
C2 - 14987481
LA - English
J2 - Astrobiology
M3 - Article
DB - Scopus
N1 - Export Date: 23 February 2023; Cited By: 6; Correspondence Address: S. Gormly; Tetra Tech EM, Inc., Reno, NV 89502, 1325 Airmotive Way, United States; email: Sherwin.Gormly@ttemi.com
ER -

TY - CONF
AU - Knacke, R.F.
TI - Possibilities for the detection of microbial life on extrasolar planets
PY - 2003
T2 - Astrobiology
VL - 3
IS - 3
SP - 531
EP - 541
DO - 10.1089/153110703322610627
UR - https://www.scopus.com/inward/record.uri?eid=2-s2.0-3843125560&doi=10.1089%2f153110703322610627&partnerID=40&md5=ae3338dd97cba54dfec0f88731d6c339
AD - School of Science, Penn State Erie, Behrend College, Erie, PA 16563, Station Road, United States
AB - We consider possibilities for the remote detection of microbial life on extrasolar planets. The Darwin/Terrestrial Planet Finder (TPF) telescope concepts for observations of terrestrial planets focus on indirect searches for life through the detection of atmospheric gases related to life processes. Direct detection of extraterrestrial life may also be possible through well-designed searches for microbial life forms. Satellites in Earth orbit routinely monitor colonies of terrestrial algae in oceans and lakes by analysis of reflected ocean light in the visible region of the spectrum. These remote sensing techniques suggest strategies for extrasolar searches for signatures of chlorophylls and related photosynthetic compounds associated with life. However, identification of such life-related compounds on extrasolar planets would require observations through strong, interfering absorptions and scattering radiances from the remote atmospheres and landmasses. Techniques for removal of interfering radiances have been extensively developed for remote sensing from Earth orbit. Comparable techniques would have to be developed for extrasolar planet observations also, but doing so would be challenging for a remote planet. Darwin/TPF coronagraph concepts operating in the visible seem to be best suited for searches for extrasolar microbial life forms with instruments that can be projected for the 2010-2020 decades, although resolution and signal-to-noise ratio constraints severely limit detection possibilities on terrestrial-type planets. The generation of telescopes with large apertures and extremely high spatial resolutions that will follow Darwin/TPF could offer striking possibilities for the direct detection of extrasolar microbial life. © Mary Ann Liebert, Inc.
KW - Algae
KW - Biogenesis
KW - Evolution
KW - Exobiology
KW - Extraterrestrial Environment
KW - Life
KW - Planets
KW - Solar System
KW - Spectrophotometry

KW - Time
KW - Water
KW - algae
KW - water
KW - alga
KW - article
KW - astronomy
KW - biogenesis
KW - biology
KW - evolution
KW - instrumentation
KW - isolation and purification
KW - life
KW - methodology
KW - microclimate
KW - spectrophotometry
KW - time
KW - Biosignatures
KW - Darwin
KW - Extrasolar planets
KW - Remote sensing
KW - Spectroscopy
KW - Terrestrial planet finder
SN - 15311074 (ISSN)
C2 - 14678662
LA - English
J2 - Astrobiology
M3 - Conference paper
DB - Scopus
N1 - Export Date: 23 February 2023; Cited By: 13; Correspondence Address: R. F. Knacke; School of Science, Penn State Erie, Behrend College, Erie, PA 16563, Station Road, United States; email: rfk2@psu.edu
ER -

TY - CONF
AU - Dalton, J.B.
AU - Mogul, R.
AU - Kagawa, H.K.
AU - Chan, S.L.
AU - Jamieson, C.S.
TI - Near-infrared detection of potential evidence for microscopic organisms on Europa
PY - 2003
T2 - Astrobiology
VL - 3
IS - 3
SP - 505
EP - 529
DO - 10.1089/153110703322610618
UR - https://www.scopus.com/inward/record.uri?eid=2-s2.0-3843145256&doi=10.1089%2f153110703322610618&partnerID=40&md5=396e56322f2931dce2b17d6aecc3ed35
AD - SETI Institute, MS 245-3, NASA Ames Research Center, Moffett

Field, CA 94035-1000, United States
AD - Wilkes Honor College, Florida Atlantic University, Jupiter, FL, United States
AD - University of Hawaii, Manoa, HI, United States
AB - The possibility of an ocean within the icy shell of Jupiter's moon Europa has established that world as a primary candidate in the search for extraterrestrial life within our Solar System. This paper evaluates the potential to detect evidence for microbial life by comparing laboratory studies of terrestrial microorganisms with measurements from the Galileo Near Infrared Imaging Spectrometer (NIMS). If the interior of Europa at one time harbored life, some evidence may remain in the surface materials. Examination of laboratory spectra of terrestrial extremophiles measured at cryogenic temperatures reveals distorted, asymmetric nearinfrared absorption features due to water of hydration. The band centers, widths, and shapes of these features closely match those observed in the Europa spectra. These features are strongest in reddish-brown, disrupted terrains such as linea and chaos regions. Narrow spectral features due to amide bonds in the microbe proteins provide a means of constraining the abundances of such materials using the NIMS data. The NIMS data of disrupted terrains exhibit distorted, asymmetric near-infrared absorption features consistent with the presence of water ice, sulfuric acid octahydrate, hydrated salts, and possibly as much as 0.2 mg cm -3 of carbonaceous material that could be of biological origin. However, inherent noise in the observations and limitations of spectral sampling must be taken into account when discussing these findings. © Mary Ann Liebert, Inc.
KW - Biogenesis
KW - Extraterrestrial Environment
KW - Freezing
KW - Jupiter
KW - Life
KW - Spectrophotometry, Infrared
KW - article
KW - astronomy
KW - biogenesis
KW - freezing
KW - infrared spectrophotometry
KW - instrumentation
KW - life
KW - methodology
KW - microclimate
KW - Europa
KW - Extraterrestrial life
KW - Extremophiles
KW - Infrared spectroscopy
SN - 15311074 (ISSN)
C2 - 14678661
LA - English
J2 - Astrobiology
M3 - Conference paper
DB - Scopus
N1 - Export Date: 23 February 2023; Cited By: 42; Correspondence Address: J. B. Dalton; SETI Institute, MS 245-3, NASA Ames Research

Center, Moffett Field, CA 94035-1000, United States; email: dalton@mail.arc.nasa.gov
ER -

TY - JOUR
AU - Boston, P.J.
AU - Frederick, R.D.
AU - Welch, S.M.
AU - Werker, J.
AU - Meyer, T.R.
AU - Sprungman, B.
AU - Hildreth-Werker, V.
AU - Thompson, S.L.
AU - Murphy, D.L.
TI - Human utilization of subsurface extraterrestrial environments.
PY - 2003
T2 - Gravitational and space biology bulletin : publication of the American Society for Gravitational and Space Biology
VL - 16
IS - 2
SP - 121
EP - 131
UR - https://www.scopus.com/inward/record.uri?eid=2-s2.0-0142120358&partnerID=40&md5=40a076dddc83554701d70a2c2fca1228
AD - Complex Systems Research, Inc., Boulder, CO, United States
AB - Caves have been used in the ancient past as shelter or habitat by many organisms (including humans). Since antiquity, humans have explored caves for the minerals they contain and sometimes for ceremonial purposes. Over the past century, caves have become the target of increasing exploration, scientific research, and recreation. The use of caves on extraterrestrial bodies for human habitation has been suggested by several investigators. Lunar lava tube bases received early attention because lava tubes were clearly visible in lunar images from the Apollo Era. More recently, Mars Observer Camera data has shown us clear evidence of large tubes visible in a number of volcanic regions on Mars. The budding field of cave geomicrobiology has direct application to questions about subsurface life on other planets. Caves contain many unusual organisms making their living from unlikely materials like manganese, iron, and sulfur. This makes caves and other subsurface habitats prime targets for astrobiological missions to Mars and possibly other bodies. We present the results of a completed Phase I and on-going Phase II NASA Institute for Advanced Concepts (NIAC) study that intensively examines the possibilities of using extraterrestrial caves as both a resource for human explorers and as a highly promising scientific target for both robotic and future human missions to Mars and beyond.
KW - Exobiology
KW - Extraterrestrial Environment
KW - Facility Design and Construction
KW - Geology
KW - Humans
KW - Life Support Systems
KW - Mars

KW - Minerals
KW - Space Flight
KW - Volcanic Eruption
KW - mineral
KW - article
KW - astronomy
KW - biology
KW - construction work and architectural phenomena
KW - geology
KW - human
KW - microclimate
KW - space flight
KW - volcano
SN - 1089988X (ISSN)
C2 - 12959139
LA - English
J2 - Gravit Space Biol Bull
M3 - Article
DB - Scopus
N1 - Export Date: 23 February 2023; Cited By: 19; Correspondence Address: P.J. Boston; email: pboston@nmt.edu
ER -

TY - JOUR
AU - Figueredo, P.H.
AU - Greeley, R.
AU - Neuer, S.
AU - Irwin, L.
AU - Schulze-Makuch, D.
TI - Locating potential biosignatures on Europa from surface geology observations
PY - 2003
T2 - Astrobiology
VL - 3
IS - 4
SP - 851
EP - 861
DO - 10.1089/153110703322736132
UR - https://www.scopus.com/inward/record.uri?eid=2-s2.0-4444272907&doi=10.1089%2f153110703322736132&partnerID=40&md5=e7298b8c876977e2e740c9a0701a4145
AD - Department of Geological Sciences, Arizona State University, Tempe, AZ 85287-1404, United States
AD - Department of Biology, Arizona State University, Tempe, AZ, United States
AD - Department of Biological Sciences, University of Texas at El Paso, El Paso, TX, United States
AD - Department of Geological Sciences, University of Texas at El Paso, El Paso, TX, United States
AB - We evaluated the astrobiological potential of the major classes of geologic units on Europa with respect to possible biosignatures preservation on the basis of surface geology observations. These observations are independent of any formational model and therefore provide an objective, though preliminary,

evaluation. The assessment criteria include high mobility of material, surface concentration of non-ice components, relative youth, textural roughness, and environmental stability. Our review determined that, as feature classes, low-albedo smooth plains, smooth bands, and chaos hold the highest potential, primarily because of their relative young age, the emplacement of low-viscosity material, and indications of material exchange with the subsurface. Some lineaments and impact craters may be promising sites for closer study despite the comparatively lower astrobiological potential of their classes. This assessment will be expanded by multidisciplinary examination of the potential for habitability of specific features. © Mary Ann Liebert, Inc.
KW - Exobiology
KW - Extraterrestrial Environment
KW - Geology
KW - Ice
KW - Jupiter
KW - Life
KW - Models, Theoretical
KW - Time Factors
KW - Water
KW - ice
KW - water
KW - article
KW - astronomy
KW - biology
KW - geology
KW - life
KW - microclimate
KW - theoretical model
KW - time
KW - Astrobiology
KW - Biosignature
KW - Diapirism
KW - Europa
KW - Habitat
KW - Tidal Stresses
SN - 15311074 (ISSN)
C2 - 14987486
LA - English
J2 - Astrobiology
M3 - Article
DB - Scopus
N1 - Export Date: 23 February 2023; Cited By: 29; Correspondence Address: P. H. Figueredo; Department of Geological Sciences, Arizona State University, Tempe, AZ 85287-1404, United States; email: figueredo@asu.edu
ER -

TY - JOUR
AU - Wierzchos, J.
AU - Ascaso, C.
AU - Sancho, L.G.
AU - Green, A.

TI - Iron-rich diagenetic minerals are biomarkers of microbial activity in Antarctic rocks
PY - 2003
T2 - Geomicrobiology Journal
VL - 20
IS - 1
SP - 15
EP - 24
DO - 10.1080/01490450303890
UR - https://www.scopus.com/inward/record.uri?eid=2-s2.0-0037248930&doi=10.1080%2f01490450303890&partnerID=40&md5=fb622b68bba63708c25403856634879c
AD - Servei de Microscopia Electronica, Universitat de Lleida, Lleida 25198, Spain
AD - Centro de Ciencias Medioambientales, CSIC, Madrid, Spain
AD - Fac. Farmacia, Universidad Complutense, Madrid, Spain
AD - Biological Sciences, Waikato University, Hamilton, New Zealand
AB - The cold, dry ecosystems of Antarctica have been shown to harbor traces left behind by microbial activity within certain types of rocks, but only two indirect biomarkers of cryptoendolithic activity in the Antarctic cold desert zone have been described to date. These are the geophysical and geochemical bioweathering patterns macroscopically observed in sandstone rock. Here we show that in this extreme environment, minerals are biologically transformed, and as a result, Fe-rich diagenetic minerals in the form of iron hydroxide nanocrystals and biogenic clays are deposited around chasmoendolithic hyphae and bacterial cells. Thus, when microbial life decays, these characteristic neocrystalized minerals act as distinct biomarkers of previous endolithic activity. The ability to recognize these traces may have potential astrobiological implications because the Antarctic Ross Desert is considered a terrestrial analogue of a possible ecosystem on early Mars.
KW - Antarctica
KW - Bacteria (microorganisms)
KW - Fungi
KW - biomarker
KW - biomineralization
KW - granite
KW - iron
KW - microbial activity
KW - Antarctic granite
KW - Biomarkers
KW - Cryptoendoliths
KW - Microfossils
SN - 01490451 (ISSN)
LA - English
J2 - Geomicrobiol. J.
M3 - Article
DB - Scopus
N1 - Export Date: 23 February 2023; Cited By: 39; Correspondence Address: J. Wierzchos; Servei de Microscopia Electronica, Universitat de Lleida, Lleida 25198, Spain; email: jacekw@suic-me.udl.es
ER -

TY - JOUR
AU - Des Marais, D.J.
AU - Allamandola, L.J.
AU - Benner, S.A.
AU - Boss, A.P.
AU - Deamer, D.
AU - Falkowski, P.G.
AU - Farmer, J.D.
AU - Hedges, S.B.
AU - Jakosky, B.M.
AU - Knoll, A.H.
AU - Liskowsky, D.R.
AU - Meadows, V.S.
AU - Meyer, M.A.
AU - Pilcher, C.B.
AU - Nealson, K.H.
AU - Spormann, A.M.
AU - Trent, J.D.
AU - Turner, W.W.
AU - Woolf, N.J.
AU - Yorke, H.W.
TI - The NASA astrobiology roadmap
PY - 2003
T2 - Astrobiology
VL - 3
IS - 2
SP - 219
EP - 235
DO - 10.1089/153110703769016299
UR - https://www.scopus.com/inward/record.uri?eid=2-s2.0-34548074003&doi=10.1089%2f153110703769016299&partnerID=40&md5=cd500f855e8d6785a8cecd6a5d9e199e
AD - Space Science Division, NASA Ames Research Center, Moffett Field, CA, United States
AD - Department of Chemistry, University of Florida and Foundation for Applied Molecular Evolution, Gainesville, FL, United States
AD - Department of Terrestrial Magnetism, Carnegie Institution of Washington, Washington, DC, United States
AD - Department of Chemistry and Biochemistry, University of California, Santa Cruz, CA, United States
AD - Institute of Marine and Coastal Sciences, Department of Geology, Rutgers University, New Brunswick, NJ, United States
AD - Department of Geological Science, Arizona State University, Tempe, AZ, United States
AD - Department of Biology, Pennsylvania State University, University Park, PA, United States
AD - Laboratory for Atmospheric and Space Physics, Department of Geological Sciences, University of Colorado, Boulder, CO, United States
AD - Department of Organismic and Evolutionary Biology, Harvard University, Cambridge, MA, United States
AD - NASA Headquarters, Washington, DC, United States
AD - SIRTF Science Center, California Institute of Technology,

Pasadena, CA, United States
AD - Jet Propulsion Laboratory, California Institute of Technology, Pasadena, CA, United States
AD - Department of Earth Sciences, University of Southern California, Los Angeles, CA, United States
AD - Department of Civil and Environmental Engineering, Stanford University, Stanford, CA, United States
AD - Department of Astronomy, University of Arizona, Tucson, AZ, United States
AB - The NASA Astrobiology Roadmap provides guidance for research and technology development across the NASA enterprises that encompass the space, Earth, and biological sciences. The ongoing development of astrobiology roadmaps embodies the contributions of diverse scientists and technologists from government, universities, and private institutions. The Roadmap addresses three basic questions: How does life begin and evolve, does life exist elsewhere in the universe, and what is the future of life on Earth and beyond? Seven Science Goals outline the following key domains of investigation: understanding the nature and distribution of habitable environments in the universe, exploring for habitable environments and life in our own solar system, understanding the emergence of life, determining how early life on Earth interacted and evolved with its changing environment, understanding the evolutionary mechanisms and environmental limits of life, determining the principles that will shape life in the future, and recognizing signatures of life on other worlds and on early Earth. For each of these goals, Science Objectives outline more specific high-priority efforts for the next 3-5 years. These 18 objectives are being integrated with NASA strategic planning. © Mary Ann Liebert, Inc.
KW - Earth (Planet)
KW - Exobiology
KW - Extraterrestrial Environment
KW - Planets
KW - United States
KW - United States National Aeronautics and Space Administration
KW - article
KW - astronomy
KW - biology
KW - government
KW - methodology
KW - microclimate
KW - United States
KW - Astrobiology
KW - Exobiology
KW - NASA
KW - Policy
KW - Roadmap
SN - 15311074 (ISSN)
C2 - 14577870
LA - English
J2 - Astrobiology
M3 - Article
DB - Scopus

N1 - Export Date: 23 February 2023; Cited By: 65; Correspondence Address: D. J. Des Marais; Mail Stop 239-4, Ames Research Center, Moffett Field, CA 94035-1000, United States; email: David.J.DesMarais@nasa.gov
ER -

TY - JOUR
AU - Prieto-Ballesteros, O.
AU - Rodríguez, N.
AU - Kargel, J.S.
AU - Kessler, C.G.
AU - Amils, R.
AU - Remolar, D.F.
TI - Tírez lake as a terrestrial analog of Europa
PY - 2003
T2 - Astrobiology
VL - 3
IS - 4
SP - 863
EP - 877
DO - 10.1089/153110703322736141
UR - https://www.scopus.com/inward/record.uri?eid=2-s2.0-4444253570&doi=10.1089%2f153110703322736141&partnerID=40&md5=26ef2398c09d2771a38cc7a9a20aac70
AD - Centro de Astrobiología (CSIC-INTA), 28850 Torrejón de Ardoz, Ctra. Ajalvir km. 4, Spain
AD - Astrogeology Team, U.S. Geological Survey, Flagstaff, AZ, United States
AD - Centro de Biología Molecular, Universidad Autónoma de Madrid, Cantoblanco, Madrid, Spain
AB - Tírez Lake (La Mancha, central Spain) is proposed as a terrestrial analogue of Europa's ocean. The proposal is based on the comparison of the hydrogeochemistry of Tírez Lake with the geochemical features of the alteration mineralogy of meteoritic precursors and with Galileo's Near Infrared Mapping Spectrometer data on Europa's surface. To validate the astrobiological potential of Tírez Lake as an analog of Europa, different hydrogeochemical, mineral, and microbial analyses were performed. Experimental and theoretical modeling helped to understand the crystallization pathways that may occur in Europa's crust. Calculations about the oxidation state of the hypothetical Europan ocean were estimated to support the sulfate-rich neutral liquid model as the origin of Europa's observed hydrated minerals and to facilitate their comparison with Tírez's hydrogeochemistry. Hydrogeochemical and mineralogical analyses showed that Tírez waters corresponded to Mg-Na-SO 4-Cl brines with epsomite, hexahydrite, and halite as end members. A preliminary microbial ecology characterization identified two different microbial domains: a photosynthetically sustained community represented by planktonic/benthonic forms and microbial mat communities, and a subsurficial anaerobic realm in which chemolithotrophy predominates. Fluorescence in situ hybridization has been used to characterize the prokaryotic diversity of the system. The subsurficial community seemed to be dominated by sulfate-reducing bacteria and methanogens. Frozen Tírez brines were

analyzed by Fourier-transform infrared techniques providing spectra similar to those reported previously using pure components and to the Galileo spectral data. Calorimetric measurements of Tírez brines showed pathways and phase metastability for magnesium sulfate and sodium chloride crystallization that may aid in understanding the processes involved in the formation of Europa's icy crust. The use of fluorescence hybridization techniques for microbial detection and characterization in hypersaline environments makes this methodology strongly advisable for future Europa astrobiological missions. © Mary Ann Liebert, Inc.
KW - Chemistry
KW - Earth (Planet)
KW - Environment
KW - Evolution, Planetary
KW - Exobiology
KW - Extraterrestrial Environment
KW - Geology
KW - Ice
KW - In Situ Hybridization, Fluorescence
KW - Jupiter
KW - Magnesium Sulfate
KW - Models, Chemical
KW - Oxygen
KW - Salts
KW - Sodium Chloride
KW - Spacecraft
KW - Spectrophotometry, Infrared
KW - Spectroscopy, Fourier Transform Infrared
KW - Sulfates
KW - Temperature
KW - Water
KW - Water Microbiology
KW - Bacteria (microorganisms)
KW - Prokaryota
KW - ice
KW - inorganic salt
KW - magnesium sulfate
KW - oxygen
KW - sodium chloride
KW - sulfate
KW - water
KW - article
KW - astronomy
KW - biology
KW - chemical model
KW - chemistry
KW - environment
KW - fluorescence in situ hybridization
KW - geology
KW - infrared spectrophotometry
KW - infrared spectroscopy
KW - microbiology
KW - microclimate
KW - space flight

KW - temperature
KW - Astrobiological environments of Europa
KW - Evaporite minerals
KW - Halophilic microorganism
KW - Saline lake
SN - 15311074 (ISSN)
C2 - 14987487
LA - English
J2 - Astrobiology
M3 - Article
DB - Scopus
N1 - Export Date: 23 February 2023; Cited By: 19; Correspondence Address: O. Prieto-Ballesteros; Centro de Astrobiología (CSIC-INTA), 28850 Torrejón de Ardoz, Ctra. Ajalvir km. 4, Spain; email: prietobo@inta.es
ER -

TY - JOUR
AU - Gánti, T.
AU - Horváth, A.
AU - Bérczi, S.
AU - Gesztesi, A.
AU - Szathmáry, E.
TI - Dark dune spots: Possible biomarkers on mars?
PY - 2003
T2 - Origins of Life and Evolution of the Biosphere
VL - 33
IS - 4-5
SP - 515
EP - 557
DO - 10.1023/A:1025705828948
UR - https://www.scopus.com/inward/record.uri?eid=2-s2.0-17144460090&doi=10.1023%2fA%3a1025705828948&partnerID=40&md5=f03ca884ca781114b9e5898f19b51673
AD - Collegium Budapest, Budapest, Hungary
AD - Budapest Planetarium Soc. D., Budapest, Hungary
AD - Konkoly Observatory, Budapest, Hungary
AD - Eötvös University, Budapest, Hungary
AB - Dark Dune Spots (DDSs) are transitional geomorphologic formations in the frost-covered polar regions of Mars. Our analysis of the transformations and arrangements of subsequent stages of DDSs into time sequence revealed their: (i) hole-like characteristics, (ii) development and formation from the bottom of the frosted layer till the disapperance of the latter, (iii) repeated (seasonal and annual) appearance in a pattern of multiple DDSs on the surface, and (iv) probable origin. We focused our studies on a model in which DDSs were interpreted as objects triggered by biological activity involved in the frosting and melting processes. We discuss two competing interpretations of DDSs: development by defrosting alone, and by defrosting and melting enhanced by the activity of Martian Surface Organisms (MSOs). MSOs are hypothetical Martian photosynthetic surface organisms thought to absorb sunlight. As a result they warm up by late winter and melt the ice around them, whereby their growth and reproduction become possible. The ice cover

above the liquid water lens harbouring the MSOs provides excellent heat and UV insulation, prevents fast evaporation, and sustains basic living conditions until the ice cover exists. When the frost cover disappears MSOs go to a dormant, desiccated state. We propose further studies to be carried out by orbiters and landers travelling to Mars and by analysis of partial analogues on earth.
KW - Biogenesis
KW - Cold Climate
KW - Earth (Planet)
KW - Exobiology
KW - Extraterrestrial Environment
KW - Geologic Sediments
KW - Ice
KW - Mars
KW - Seasons
KW - ice
KW - biomarker
KW - ice
KW - Mars
KW - microbiology
KW - polar region
KW - water
KW - article
KW - astronomy
KW - biogenesis
KW - biology
KW - cold climate
KW - microclimate
KW - season
KW - sediment
KW - Astrobiology
KW - Dark Dune Spots (DDSs)
KW - Frosting–defrosting
KW - Geomorphologic analysis of DDSs
KW - Life on Mars
KW - Mars Global Surveyor
KW - Mars Odyssey
KW - Martian Surface Organisms (MSOs)
KW - Southern Polar Region of Mars
KW - Water ice and liquid water on Mars
SN - 01696149 (ISSN)
C2 - 14604189
LA - English
J2 - Orig. Life Evol. Biosph.
M3 - Article
DB - Scopus
N1 - Export Date: 23 February 2023; Cited By: 22; Correspondence Address: E. Szathmáry; Collegium Budapest, Budapest, Hungary; email: szathmary@colbud.hu; CODEN: OLEBE
ER -

TY - JOUR
AU - Gilichinsky, D.
AU - Rivkina, E.

AU - Shcherbakova, V.
AU - Laurinavichuis, K.
AU - Tiedje, J.
TI - Supercooled water brines within permafrost - An unknown ecological niche for microorganisms: A model for astrobiology
PY - 2003
T2 - Astrobiology
VL - 3
IS - 2
SP - 331
EP - 341
DO - 10.1089/153110703769016424
UR - https://www.scopus.com/inward/record.uri?eid=2-s2.0-1842591469&doi=10.1089%2f153110703769016424&partnerID=40&md5=7705a5baf8b4e3ea89f0ec1b89d42448
AD - Institute of Physicochemical and Biological Problems in Soil Science, Russian Academy of Sciences, 142290, Pushchino, Moscow Region, Russian Federation
AD - Institute of Biochemistry and Physiology of Microorganisms, Russian Academy of Sciences, Pushchino, Moscow Region, Russian Federation
AD - Center for Microbial Ecology, Michigan State University, East Lansing, MI, United States
AB - This study describes brine lenses (cryopegs) found in Siberian permafrost derived from ancient marine sediment layers of the Arctic Ocean. The cryopegs were formed and isolated from sediment ~100,000-120,000 years ago. They remain liquid at the in situ temperature of -10°C as a result of their high salt content (170-300 g/L). [14C] Glucose is taken up by the cryopeg biomass at -15°C, indicating microbial metabolism at low temperatures in this habitat. Furthermore, aerobic, anaerobic heterotrophs, sulfate reducers, acetogens, and methanogens were detected by most probable number analysis. Two psychrophilic microbes were isolated from the cryopegs, a Clostridium and a Psychrobacter. The closest relatives of each were previously isolated from Antarctica. The cryopeg econiche might serve as a model for extraterrestrial life, and hence is of particular interest to astrobiology. © Mary Ann Liebert, Inc.
KW - Antarctic Regions
KW - Arctic Regions
KW - Biogenesis
KW - Clostridium
KW - Exobiology
KW - Ice
KW - Models, Biological
KW - Psychrobacter
KW - Clostridium
KW - Psychrobacter
KW - ice
KW - Antarctica
KW - Arctic
KW - article
KW - biogenesis
KW - biological model
KW - biology

KW - Clostridium
KW - metabolism
KW - methodology
KW - Psychrobacter
KW - Ancient ecosystem
KW - Astrobiology
KW - Extraterrestrial econiche
KW - Lake Vostok
KW - Mars
KW - Permafrost
KW - Viable microorganisms
KW - Water brines
SN - 15311074 (ISSN)
C2 - 14577882
LA - English
J2 - Astrobiology
M3 - Article
DB - Scopus
N1 - Export Date: 23 February 2023; Cited By: 113; Correspondence Address: D. Gilichinsky; Institute of Physicochemical and Biological Problems in Soil Science, Russian Academy of Sciences, 142290, Pushchino, Moscow Region, Russian Federation; email: gilichin@issp.serpukhov.su
ER -

TY - JOUR
AU - Vishnivetskaya, T.A.
AU - Spirina, E.V.
AU - Shatilovich, A.V.
AU - Erokhina, L.G.
AU - Vorobyova, E.A.
AU - Gilichinsky, D.A.
TI - The resistance of viable permafrost algae to simulated environmental stresses: Implications for astrobiology
PY - 2003
T2 - International Journal of Astrobiology
VL - 2
IS - 3
SP - 171
EP - 177
DO - 10.1017/S1473550403001575
UR - https://www.scopus.com/inward/record.uri?eid=2-s2.0-33750338184&doi=10.1017%2fS1473550403001575&partnerID=40&md5=cc0ee2d85e96102840290c328186a5ed
AD - Laboratory of Soil Cryology, Institute for Physicochemical and Biological Problems in Soil Science, Russian Academy of Sciences, 142290, Pushchino, Moscow Region, Russian Federation
AD - Institute of Basic Biological Problems, Russian Academy of Sciences, 142290, Pushchino, Moscow Region, Russian Federation
AD - Department of Soil Science, Moscow State University, 119899, Moscow, Russian Federation
AB - 54 strains of viable green algae and 26 strains of viable cyanobacteria were recovered from 128 and 56 samples collected from Siberian and Antarctic permafrost, respectively, with ages from

modern to a few million years old. Although species of unicellular green algae belonged to Chlorococcales were subdominant inside permafrost, green algae Pedinomonas sp. were observed in Antarctic permafrost. Filamentous cyanobacteria of Oscillatoriales, Nostocales were just found in Siberian permafrost. Algal biomass in the permanently frozen sediments, expressed as concentration of chlorophyll a, was 0.06–0.46 μg g−1. The number of viable algal cells varied between <102 and 9×103 cfu g−1, but the number of viable bacterial cells was usually higher from 102 to 9.2×105 cfu g−1. Frozen but viable permafrost algae have preserved their morphological characteristics and photosynthetic apparatus in the dark permafrost. In the laboratory, they restored their photosynthetic activity, growth and development in favourable conditions at positive temperatures and with the availability of water and light. The discovery of ancient viable algae within permafrost reflects their ability to tolerate long-term freezing. In this study, the tolerance of algae and cyanobacteria to freezing, thawing and freezing–drying stresses was evaluated by short-term (days to months) low-temperature experiments. Results indicate that viable permafrost microorganisms demonstrate resistance to such stresses. Apart from their ecological importance, the bacterial and algal species found in permafrost have become the focus for novel biotechnology, as well as being considered proxies for possible life forms on cryogenic extraterrestrial bodies. © 2003, Cambridge University Press. All rights reserved.
KW - Antarctica
KW - astrobiology
KW - biotic survival
KW - cyanobacteria
KW - freezing
KW - green algae
KW - permafrost
KW - Siberia
KW - thawing
SN - 14735504 (ISSN)
LA - English
J2 - Int. J. Astrobiology
M3 - Article
DB - Scopus
N1 - Export Date: 23 February 2023; Cited By: 20
ER -

TY - JOUR
AU - Irvine, W.M.
AU - Carramiñana, A.
AU - Carrasco, L.
AU - Schloerb, F.P.
TI - The Large Millimeter Telescope/El Gran Telescopio MiliméTrico: A New Instrument for Astrobiology
PY - 2003
T2 - Origins of Life and Evolution of the Biosphere
VL - 33
IS - 6
SP - 597

EP - 607
DO - 10.1023/A:1025732428637
UR - https://www.scopus.com/inward/record.uri?eid=2-s2.0-0142039751&doi=10.1023%2fA%3a1025732428637&partnerID=40&md5=8c7ed5e9488c6728f8d2981dbc57712a
AD - Department of Astronomy, Five Coll. Radio Astron. Observatory, University of Massachusetts, Amherst, MA, United States
AD - Inst. Nac. Astrofis., Optica E., Puebla, Puebla, AP 51y216, Mexico
AB - The Instituto Nacional de Astrofísica, Óptica y Electrónica in Mexico and the University of Massachusetts in the U.S.A. are collaborating to build the world's largest radio telescope that operates at short millimeter wavelengths. This facility, known as the Large Millimeter Telescope (LMT) or el Gran Telescopio Milimétrico (GTM), is being sited at an altitude of 4600 m on Volcan Sierra Negra in the Mexican state of Puebla. The telescope will be a fully steerable dish with a diameter of 50 m and a surface consisting of 180 panels that are actively adjusted under computer control to correct for deformations due to gravity and temperature gradients. Instruments will include focal plane arrays to image both continuum and spectral line emission from celestial sources. The LMT/GTM will be an extremely powerful facility for studies encompassing almost all areas of astronomy, including astrobiology. In particular, the high sensitivity, angular resolution, and mapping speed will enable detailed investigations of the organic chemistry of interstellar molecular clouds, protoplanetary disks, and comets.
KW - Chemistry, Organic
KW - Exobiology
KW - Extraterrestrial Environment
KW - Massachusetts
KW - Mexico
KW - Solar System
KW - United States
KW - astronomy
KW - biology
KW - comet
KW - equipment
KW - article
KW - astronomy
KW - biology
KW - instrumentation
KW - methodology
KW - Mexico
KW - microclimate
KW - organic chemistry
KW - United States
KW - Astrobiology
KW - Comets
KW - Interstellar chemistry
KW - Molecular clouds
KW - Radio astronomy
KW - Radio telescope
SN - 01696149 (ISSN)
C2 - 14601929

LA - English
J2 - Orig. Life Evol. Biosph.
M3 - Article
DB - Scopus
N1 - Export Date: 23 February 2023; Cited By: 2; Correspondence Address: W.M. Irvine; Department of Astronomy, Five Coll. Radio Astron. Observatory, University of Massachusetts, Amherst, MA, United States; email: irvine@fcraol.astro.umass.edu; CODEN: OLEBE
ER -

TY - JOUR
AU - Patel, M.R.
AU - Bérces, A.
AU - Kolb, C.
AU - Lammer, H.
AU - Rettberg, P.
AU - Zarnecki, J.C.
AU - Selsis, F.
TI - Seasonal and diurnal variations in Martian surface ultraviolet irradiation: Biological and chemical implications for the Martian regolith
PY - 2003
T2 - International Journal of Astrobiology
VL - 2
IS - 1
SP - 21
EP - 34
DO - 10.1017/S1473550402001180
UR - https://www.scopus.com/inward/record.uri?eid=2-s2.0-1542561613&doi=10.1017%2fS1473550402001180&partnerID=40&md5=33fd97d32d3b5f4b9e69447e885ca9d1
AD - Planetary and Space Sciences Research Institute, The Open University, Walton Hall, Milton Keynes MK7 6AA, United Kingdom
AD - MTA-SE Research Group for Biophysics, Semmelweis Med. University, VIII Puskin u.9, H-1444 Budapest, Hungary
AD - Space Research Institute, Department for Extraterrestrial Physics, Austrian Academy of Sciences, Schmiedlstr. 6, A-8042, Graz, Austria
AD - German Aerospace Centre (DLR), Institute of Aerospace Medicine, 51170 Cologne, Germany
AD - Centro de Astrobiologia (CSIC/INTA), Torrejón de Ardoz, Madrid, Carretera Ajalvier, km 4, Spain
AB - The issue of the variation of the surface ultraviolet (UV) environment on Mars was investigated with particular emphasis being placed on the interpretation of data in a biological context. A UV model has been developed to yield the surface UV irradiance at any time and place over the Martian year. Seasonal and diurnal variations were calculated and dose rates evaluated. Biological interpretation of UV doses is performed through the calculation of DNA damage effects upon phage T7 and Uracil, used as examples for biological dosimeters. A solar UV ‘hotspot’ was revealed towards perihelion in the southern hemisphere, with a significant damaging effect upon these species. Diurnal profiles of UV irradiance are also seen to vary markedly between aphelion and perihelion. The

effect of UV dose is also discussed in terms of the chemical environment of the Martian regolith, since UV irradiance can reach high enough levels so as to have a significant effect upon the soil chemistry. We show, by assuming that H2O is the main source of hydrogen in the Martian atmosphere, that the stoichiometrically desirable ratio of 2:1 for atmospheric H and O loss rates to space are not maintained and at present the ratio is about 20:1. A large planetary oxygen surface sink is therefore necessary, in contrast with escape to space. This surface oxygen sink has important implications for the oxidation potential and the toxicology of the Martian soil. UV-induced adsorption of {\rm O}{2}{-} super-radicals plays an important role in the oxidative environment of the Martian surface, and the biologically damaging areas found in this study are also shown to be regions of high subsurface oxidation. Furthermore, we briefly cover the astrobiological implications for landing sites that are planned for future Mars missions. © 2003, Cambridge University Press. All rights reserved.
KW - biologically effective dose
KW - DNA damage
KW - landing sites
KW - Mars
KW - radiation climate
KW - radiative transfer
KW - regolith
KW - toxicology
KW - UV
SN - 14735504 (ISSN)
LA - English
J2 - Int. J. Astrobiology
M3 - Article
DB - Scopus
N1 - Export Date: 23 February 2023; Cited By: 50
ER -

TY - JOUR
AU - Staley, J.T.
TI - Astrobiology, the transcendent science: The promise of astrobiology as an integrative approach for science and engineering education and research
PY - 2003
T2 - Current Opinion in Biotechnology
VL - 14
IS - 3
SP - 347
EP - 354
DO - 10.1016/S0958-1669(03)00073-9
UR - https://www.scopus.com/inward/record.uri?eid=2-s2.0-0038482010&doi=10.1016%2fS0958-1669%2803%2900073-9&partnerID=40&md5=0ffe790ec4195b33833b350656ed73d4
AD - Department of Microbiology, University of Washington, Box 357242, Seattle, WA 98195, United States
AB - Astrobiology is rapidly gaining the worldwide attention of scientists, engineers and the public. Astrobiology's captivation is due to its inherently interesting focus on life, its origins and

distribution in the Universe. Because of its remarkable breadth as a scientific field, astrobiology touches on virtually all disciplines in the physical, biological and social sciences as well as engineering. The multidisciplinary nature and the appeal of its subject matter make astrobiology ideal for integrating the teaching of science at all levels in educational curricula. The rationale for implementing novel educational programs in astrobiology is presented along with specific research and educational policy recommendations.
KW - astrobiology
KW - astronomy
KW - atmosphere
KW - biology
KW - cosmos
KW - education program
KW - engineering
KW - evolution
KW - fossil
KW - geochemistry
KW - human
KW - mysticism
KW - natural science
KW - paleontology
KW - policy
KW - priority journal
KW - research
KW - review
KW - science
KW - sociology
PB - Elsevier Ltd
SN - 09581669 (ISSN)
C2 - 12849791
LA - English
J2 - Curr. Opin. Biotechnol.
M3 - Review
DB - Scopus
N1 - Export Date: 23 February 2023; Cited By: 24; Correspondence Address: J.T. Staley; Department of Microbiology, University of Washington, Box 357242, Seattle, WA 98195, United States; email: jtstaley@u.washington.edu; CODEN: CUOBE
ER -

TY - JOUR
AU - Van Doninck, K.
AU - Schön, I.
AU - Martens, K.
TI - A perspective on the importance of reproductive mode in astrobiology
PY - 2003
T2 - Astrobiology
VL - 3
IS - 4
SP - 657
EP - 671
DO - 10.1089/153110703322735999

UR - https://www.scopus.com/inward/record.uri?eid=2-s2.0-4444331150&doi=10.1089%2f153110703322735999&partnerID=40&md5=5b263137dca843f7a6b12c0a84efb41c
AD - Freshwater Biology Section, Royal Belgian Institute of Natural Sciences, Brussels, Belgium
AD - Laboratorium of Cellular Genetics, Free University of Brussels, Brussels, Belgium
AD - Molecular and Cellular Department, Harvard University, Cambridge, MA 02138, 7 Divinity Avenue, United States
AB - Reproduction is a vital characteristic of life, and sex is the most common reproductive mode in the eukaryotic world. Sex and reproduction are not necessarily linked mechanisms: Sexuality without reproduction exists, while several forms of asexual reproduction are known. The occurrence of sexuality itself is paradoxical, as it is very costly in evolutionary terms. Most of the hypotheses (more than 20) attempting to explain the prevalence of sex fall into two categories: Sex either creates good gene combinations for adaptation to environments or eliminates bad gene combinations counteracting the accumulation of mutations. In spite of this apparent wealth of beneficial effects of sex, asexuality is not rare. Most eukaryotic, asexual lineages are short-lived and can only persist through the presence of sexual roots, but at least two animal groups, bdelloid rotifers and darwinulid ostracods, seem to claim the status of ancient asexuals. Research on (a)sexuality is relevant to astrobiology in a number of ways. First, strong relationships between the origin and persistence of life in extreme environments and reproductive mode are known. Second, the "habitability" of nonterrestrial environments to life greatly depends on reproductive mode. Whereas asexuals can do equally well or better in harsh environments, they fail to adapt fast enough to changing abiotic and biotic environments. Third, it has been shown that plants reproduce mainly asexually in space, and sperm production and motility in some vertebrates are hampered. Both findings indicate that extraterrestrial life under conditions different from Earth might be dominated by asexual reproduction. Finally, for exchange of biological material between planets, the choice of reproductive mode will be important. © Mary Ann Liebert, Inc.
KW - Animals
KW - Evolution
KW - Exobiology
KW - Extraterrestrial Environment
KW - Genotype
KW - Models, Theoretical
KW - Mutation
KW - Planets
KW - Reproduction
KW - Reproduction, Asexual
KW - Animalia
KW - Bdelloida
KW - Darwinulidae
KW - Eukaryota
KW - Ostracoda
KW - Rotifera

KW - Vertebrata
KW - animal
KW - asexual reproduction
KW - astronomy
KW - biology
KW - evolution
KW - genotype
KW - methodology
KW - microclimate
KW - mutation
KW - reproduction
KW - review
KW - theoretical model
KW - Ancient asexuals
KW - Asexual reproduction
KW - Extraterrestrial conditions
KW - Fluctuating selection
KW - Sexual reproduction
SN - 15311074 (ISSN)
C2 - 14987472
LA - English
J2 - Astrobiology
M3 - Review
DB - Scopus
N1 - Export Date: 23 February 2023; Cited By: 3; Correspondence Address: K. Van Doninck; Molecular and Cellular Department, Harvard University, Cambridge, MA 02138, 7 Divinity Avenue, United States; email: kvdoninc@mcb.harvard.edu
ER -

TY - JOUR
AU - Kennedy, C.B.
AU - Scott, S.D.
AU - Ferris, F.G.
TI - Characterizationof bacteriogenic iron oxide deposits from Axial Volcano, Juan de Fuca Ridge, northeast Pacific ocean
PY - 2003
T2 - Geomicrobiology Journal
VL - 20
IS - 3
SP - 199
EP - 214
DO - 10.1080/01490450303873
UR - https://www.scopus.com/inward/record.uri?eid=2-s2.0-0042575641&doi=10.1080%2f01490450303873&partnerID=40&md5=ff6fc124719cf08e7f78d22eaec9ae45
AD - Department of Geology, University of Toronto, Toronto, Ont. M5S 3B1, Canada
AB - Iron oxides from the caldera of Axial Volcano, a site of hydrothermal vent activity along the Juan de Fuca Ridge, are characterized by abundant bacterial structures that closely resemble the sheaths of Leptothrix ochracea, the stalks of Gallionella ferruginea, and the filaments of a novel iron oxidizing PV-I strain. These bacteria are commonly associated with iron-oxide to

precipitates and are proposed to play two causal roles in the formation of iron oxides at Axial Volcano. First, by increasing the rate of Fe2+ oxidation, and second, by lowering the concentration of Fe3+ required for precipitation by providing a reactive surface for heterogeneous nucleation. Rapid rates of oxidation and precipitation caused by the bacteria likely contribute to the poorly ordered nature of the iron oxides, determined to be primarily 2-line ferrihydrite, and in one case, poorly ordered goethite. The iron-oxide precipitates consist dominantly of iron, silicon (mostly diatoms), and organic carbon with a suite of sorbed trace metals. The high metal uptake affinity of these poorly ordered iron oxides may be important in the global cycling of trace elements throughout the world's oceans. Additionally, iron oxides precipitating on the surface of bacteria can preserve individual cells as microfossils making bacteriogenic iron oxides ideal proxies for paleoenvironmental and astrobiological studies.
KW - Pacific Ocean
KW - Bacillariophyta
KW - Bacteria (microorganisms)
KW - Gallionella
KW - Gallionella ferruginea
KW - Leptothrix
KW - bacterium
KW - biogenic mineral
KW - geomicrobiology
KW - hydrothermal vent
KW - iron oxide
KW - redox conditions
KW - submarine volcano
KW - Axial Volcano
KW - Bacteria
KW - Ferrihydrite
KW - Hydrothermal vents
KW - Sea floor
SN - 01490451 (ISSN)
LA - English
J2 - Geomicrobiol. J.
M3 - Article
DB - Scopus
N1 - Export Date: 23 February 2023; Cited By: 97; Correspondence Address: F.G. Ferris; Department of Geology, University of Toronto, Toronto, Ont. M5S 3B1, 22 Russell Street, Canada; email: ferris@geology.utoronto.ca
ER -

TY - JOUR
AU - Rohwerder, T.
AU - Sand, W.
AU - Lascu, C.
TI - Preliminary evidence for a sulphur cycle in Movile Cave, Romania
PY - 2003
T2 - Acta Biotechnologica
VL - 23

IS - 1
SP - 101
EP - 107
DO - 10.1002/abio.200390000
UR - https://www.scopus.com/inward/record.uri?eid=2-s2.0-0037278296&doi=10.1002%2fabio.200390000&partnerID=40&md5=7cb51982b2ac33ae7f90778dc622bebc
AD - Universität Hamburg, Institut für Allgemeine Botanik, Abteilung Mikrobiologie, 22609 Hamburg, Ohnhorststraße 18, Germany
AD - Romanian Academy of Sciences, Inst. of Speleology Emil Racovita, 78114 Bucharest, Str. Frumoasa 11, Romania
AB - In order to investigate the food chain and energy balance of the chemolithoautotrophically-based ecosystem of the sulphur spring in Movile Cave as a model system for extraterrestrial life, a first sampling campaign was done. Microbial diversity and activity were analysed by MPN-enumeration methods and microcalorimetry, respectively. In addition, a speciation of the inorganic sulphur compounds by HPLC and IC techniques was performed. Metabolic activities were predominantly connected with thick microbial mats floating on the water surface of the cave. These mats showed an aerobic heat evolution of about 1200 μW/g and contained about 500 μmol/g elemental sulphur. In contrast, other samples collected from cave water, sediment and rock exhibited only activities of maximal 40 to 60 μW/g and contained only up to 2.5 μmol/g elemental sulphur. As the main primary producers aerobic and facultatively anaerobic sulphur oxidisers were identified at high numbers, occasionally exceeding 107 CFU/g. Methylotrophic bacteria were present in all samples at up to 106 CFU/g, indicating the important role of Cl metabolism for the cave ecosystem. Although reduced sulphur species were biologically oxidised to sulphuric acid, the pH values of the samples ranged from 6.5 to 8.2 due to the high buffering capacity of the cave walls, which consisted mainly of limestone. Surprisingly, not only neutrophilic but also extremely acidophilic bacteria were detected. Sulphate reducers were present in both aerobic and anaerobic zones. The data presented suggest a close interdependence of sulphur oxidation and reduction as well as carbon dioxide and other Cl compound metabolism in the most biologically active zone of the cave ecosystem, i.e. the floating microbial mats.
KW - Movile Cave
KW - Romania
KW - Bacteria (microorganisms)
KW - carbon
KW - carbon dioxide
KW - inorganic compound
KW - limestone
KW - sulfur
KW - sulfuric acid
KW - cave
KW - energy balance
KW - food chain
KW - metabolism
KW - microbial community
KW - sulfur cycle
KW - colony forming unit

KW - controlled study
KW - data analysis
KW - ecosystem
KW - energy balance
KW - food chain
KW - heat
KW - high performance liquid chromatography
KW - methylotrophic bacterium
KW - microbial activity
KW - microbial diversity
KW - microbial mat
KW - microcalorimetry
KW - microclimate
KW - oxidation
KW - pH
KW - reduction
KW - rock
KW - Romania
KW - sampling
KW - sediment
KW - short survey
KW - species differentiation
PB - Wiley-VCH Verlag
SN - 01384988 (ISSN)
LA - English
J2 - Acta Biotechnol.
M3 - Short survey
DB - Scopus
N1 - Export Date: 23 February 2023; Cited By: 23; Correspondence Address: W. Sand; Universität Hamburg, Institut für Allgemeine Botanik, Abteilung Mikrobiologie, 22609 Hamburg, Ohnhorststraße 18, Germany; email: sand@mikrobiologie.uni-hamburg.de; CODEN: ACBTD
ER -

TY - JOUR
AU - Bergin, E.
AU - Calvet, N.
AU - D'Alessio, P.
AU - Herczeg, G.J.
TI - THE EFFECTS of UV CONTINUUM and Lyα RADIATIon on the CHEMICAL EQUILIBRIUM of T TAURI DISKS
PY - 2003
T2 - Astrophysical Journal
VL - 591
IS - 2 II
SP - L159
EP - L162
DO - 10.1086/377148
UR - https://www.scopus.com/inward/record.uri?eid=2-s2.0-0141502366&doi=10.1086%2f377148&partnerID=40&md5=651c4eced815249e793ce8e21b8f7710
AD - Harvard-Smithsonian Ctr Astrophys, Cambridge, MA 02138, 60 Garden Street, United States
AD - Instituto de Astronomía, Univ Nac Auton de México, 58089

Morelia, Michoacan, Apartado Postal 72-3 (Xangari), Mexico
AD - JILA, University of Colorado at Boulder, 440 UCB, Boulder, CO 80309-0440, United States
AB - We show in this Letter that the spectral details of the far-ultraviolet (FUV) radiation fields have a large impact on the chemistry of protoplanetary disks surrounding T Tauri stars. We show that the strength of a realistic stellar FUV field is significantly lower than typically assumed in chemical calculations and that the radiation field is dominated by strong line emission, most notably Lyα radiation. The effects of the strong Lyα emission on the chemical equilibrium in protoplanetary disks have previously been unrecognized. We discuss the impact of this radiation on molecular observations in the context of a radiative transfer model that includes both direct attenuation and scattering. In particular, Lyα radiation will directly dissociate water vapor and may contribute to the observed enhancements of CN/ HCN in disks.
KW - Accretion, accretion disks
KW - Astrobiology
KW - Astrochemistry
KW - Circumstellar matter
KW - Stars: Pre-main-sequence
KW - Ultraviolet: Stars
PB - Institute of Physics Publishing
SN - 0004637X (ISSN)
LA - English
J2 - Astrophys. J.
M3 - Article
DB - Scopus
N1 - Export Date: 23 February 2023; Cited By: 120; Correspondence Address: E. Bergin; Harvard-Smithsonian Ctr Astrophys, Cambridge, MA 02138, 60 Garden Street, United States; email: ebergin@cfa.harvard.edu
ER -

TY - CONF
AU - Ellery, A.
AU - Richter, L.
AU - Parnell, J.
AU - Baker, A.
TI - A low-cost approach to the exploration of mars through a robotic technology demonstrator mission
PY - 2003
T2 - European Space Agency, (Special Publication) ESA SP
IS - 542
SP - 127
EP - 134
UR - https://www.scopus.com/inward/record.uri?eid=2-s2.0-0842311272&partnerID=40&md5=a4c542eb1d4efa1400404702505aaac3
AD - School of Engineering, Kingston University, London SW15 3DW, Roehampton Vale, United Kingdom
AD - DLR, Institute of Space Simulation, D-51170 Cologne, Germany
AD - Department of Geology, University of Aberdeen, Kings College, Aberdeen B24 3UE, United Kingdom
AD - Surrey Satellite Technology Ltd, Ctr. for Satellite Eng.

Research, University of Surrey, Guildford, Surrey G2 5XH, United Kingdom
AB - We present a proposed robotic mission to Mars Vanguard - For the Aurora Arrow programme which combines an extensive technology demonstrator with a high scientific return. The novel aspect of this technology demonstrator is the demonstration of "water mining" capabilities for in-situ resource utilisation in conjunction with high-value astrobiological investigation within a low mass lander package of 70 kg. The basic architecture comprises a small lander, a micro-rover and a number of ground-penetrating moles. This basic architecture offers the possibility of testing a wide variety of generic technologies associated with space systems and planetary exploration. The architecture provides for the demonstration of specific technologies associated with planetary surface exploration, and with the Aurora programme specifically. Technology demonstration of in-situ resource utilisation will be a necessary precursor to any future human mission to Mars. Furthermore, its modest mass overhead allows the reuse of the already built Mars Express bus, making it a very low cost option.
KW - Cost effectiveness
KW - Fiber optics
KW - Ground penetrating radar systems
KW - Magnetometers
KW - Martian surface analysis
KW - Mineralogy
KW - Orbital laboratories
KW - Planetary landers
KW - Robotics
KW - Sensors
KW - Soils
KW - Spectrometers
KW - Entry descent and landing systems (EDLS)
KW - Payloads
KW - Space research
SN - 03796566 (ISSN)
LA - English
J2 - Eur Space Agency Spec Publ ESA SP
M3 - Conference paper
DB - Scopus
N1 - Export Date: 23 February 2023; Cited By: 0; Correspondence Address: A. Ellery; School of Engineering, Kingston University, London SW15 3DW, Roehampton Vale, United Kingdom; email: a.ellery@kingston.ac.uk; CODEN: ESPUD
ER -

TY - CONF
AU - Robigou, V.
TI - REVEL - Research and Education: Volcanoes, Exploration and Life
PY - 2003
T2 - Oceans Conference Record (IEEE)
VL - 4
SP - 1981
UR - https://www.scopus.com/inward/record.uri?eid=2-

s2.0-1542400655&partnerID=40&md5=a43e1e8f6dc91e49ec937e47156c7f9e
AD - University of Washington, St. Louis, MO, United States
AB - REVEL is a professional development program sponsored by the National Science Foundation (NSF) for science teachers who are selected to participate in funded-seagoing research cruises, and to contribute to ongoing scientific research. During the first five years of the program, the research focused on scientific issues ranging from the origin of life to new aspects of biotechnology related to the study of mid-ocean ridges. The interdisciplinary research spanned from earth sciences to astrobiology, including oceanography, chemistry, physics, biology, computer sciences, mathematics, and engineering to cite a few of the scientific disciplines represented in the study of mid-ocean processes. REVEL is a collaborative partnership between researchers, teachers, students, schools, research institutions, and the community.
KW - Education
KW - Natural resources exploration
KW - Ocean engineering
KW - Personnel
KW - Professional aspects
KW - Research and development management
KW - Societies and institutions
KW - Students
KW - Volcanoes
KW - Interdisciplinary research
KW - Researchers
KW - Schools
KW - Teachers
KW - Oceanography
SN - 01977385 (ISSN)
LA - English
J2 - Oceans Conf Rec IEEE
M3 - Conference paper
DB - Scopus
N1 - Export Date: 23 February 2023; Cited By: 0; CODEN: OCNSD
ER -

TY - JOUR
AU - Menou, K.
AU - Tabachnik, S.
TI - Dynamical habitability of known extrasolar planetary systems
PY - 2003
T2 - Astrophysical Journal
VL - 583
IS - 1 I
SP - 473
EP - 488
DO - 10.1086/345359
UR - https://www.scopus.com/inward/record.uri?eid=2-s2.0-0042118316&doi=10.1086%2f345359&partnerID=40&md5=d17b59f078da4c385725579883a1c441
AD - Department of Astrophysical Sciences, Princeton University, Peyton Hall, Princeton, NJ 08544, United States
AD - Department of Astronomy, University of Virginia,

Charlottesville, VA 22903, P. O. Box 3818, United States
AB - Habitability is usually defined as the requirement for a terrestrial planet's atmosphere to sustain liquid water. This definition can be complemented by the dynamical requirement that other planets in the system do not gravitationally perturb terrestrial planets outside of their habitable zone, the orbital region allowing the existence of liquid water. We quantify the dynamical habitability of 85 known extrasolar planetary systems via simulations of their orbital dynamics in the presence of potentially habitable terrestrial planets. When requiring that habitable planets remain strictly within their habitable zone at all times, the perturbing influence of giant planets extends beyond the traditional Hill sphere for close encounters: terrestrial planet excursions outside of the habitable zone are also caused by secular eccentricity variations and, in some cases, strong mean-motion resonances. Our results indicate that more than half the known extrasolar planetary systems (mostly those with distant, eccentric giant planets) are unlikely to harbor habitable terrestrial planets. About one-fourth of the systems (mostly those with close-in giant planets), including one-third of the potential targets for the Terrestrial Planet Finder, appear as dynamically habitable as our own solar system. The influence of yet undetected giant planets in these systems could compromise their dynamical habitability. Some habitable terrestrial planets in our simulations have substantial eccentricities (e > 0.1), which may lead to large seasonal climate variations and thus affect their habitability.
KW - Astrobiology
KW - Celestial mechanics
KW - Methods: n-body simulations
KW - Planetary systems
KW - Stellar dynamics
PB - Institute of Physics Publishing
SN - 0004637X (ISSN)
LA - English
J2 - Astrophys. J.
M3 - Article
DB - Scopus
N1 - Export Date: 23 February 2023; Cited By: 105
ER -

TY - JOUR
AU - Dalton, J.B.
TI - Spectral behavior of hydrated sulfate salts: Implications for Europa mission spectrometer design
PY - 2003
T2 - Astrobiology
VL - 3
IS - 4
SP - 771
EP - 784
DO - 10.1089/153110703322736097
UR - https://www.scopus.com/inward/record.uri?eid=2-s2.0-4444270691&doi=10.1089%2f153110703322736097&partnerID=40&md5=6a4db73b9bb55c708483aba5f7e7282f

AD - SETI Institute, MS 245-3, NASA Ames Research Center, Moffett Field, CA 94035-1000, United States
AB - Remote sensing of the surface of Europa with near-infrared instruments has suggested the presence of hydrated materials, including sulfate salts. Attention has been focused on these salts for the information they might yield regarding the evolution of a putative interior ocean, and the evaluation of its astrobiological potential. These materials exhibit distinct infrared absorption features due to bound water. The interactions of this water with the host molecules lead to fine structure that can be used to discriminate among these materials on the basis of their spectral behavior. This fine structure is even more pronounced at the low temperatures prevalent on icy satellites. Examination of hydrated sulfate salt spectra measured under cryogenic temperature conditions provides realistic constraints for future remote-sensing missions to Europa. In particular, it suggests that a spectrometer system capable of 2-5 nm spectral resolution or better, with a spatial resolution approaching 100 m, would be able to differentiate among proposed hydrated surface materials, if present, and constrain their distributions across the surface. Such information would provide valuable insights into the evolutionary history of Europa. © Mary Ann Liebert, Inc.
KW - Astronomy
KW - Cold
KW - Equipment Design
KW - Evolution, Planetary
KW - Extraterrestrial Environment
KW - Jupiter
KW - Magnesium Sulfate
KW - Salts
KW - Spacecraft
KW - Spectrophotometry, Infrared
KW - Temperature
KW - Time Factors
KW - Water
KW - inorganic salt
KW - magnesium sulfate
KW - water
KW - article
KW - astronomy
KW - chemistry
KW - cold
KW - equipment design
KW - infrared spectrophotometry
KW - instrumentation
KW - methodology
KW - microclimate
KW - space flight
KW - temperature
KW - time
KW - Europa
KW - Hydrated Salts
KW - Infrared Spectroscopy
KW - Remote Sensing

SN - 15311074 (ISSN)
C2 - 14987482
LA - English
J2 - Astrobiology
M3 - Article
DB - Scopus
N1 - Export Date: 23 February 2023; Cited By: 49; Correspondence Address: J. B. Dalton; SETI Institute, MS 245-3, NASA Ames Research Center, Moffett Field, CA 94035-1000, United States; email: dalton@mail.arc.nasa.gov
ER -

TY - JOUR
AU - Delio, I.
TI - Revisiting the Franciscan doctrine of Christ
PY - 2003
T2 - Theological Studies
VL - 64
IS - 1
SP - 3
EP - 23
DO - 10.1177/004056390306400101
UR - https://www.scopus.com/inward/record.uri?eid=2-s2.0-60949697786&doi=10.1177%2f004056390306400101&partnerID=40&md5=dfe2626a0c3e2114ae616e8acc0d562d
AD - Department of Ecclesiastical History, Washington Theological Union
AD - Franciscan Center
AB - Franciscan theologians posit an integral relation between Incarnation and Creation whereby the Incarnation is grounded in the Trinity of love. The primacy of Christ as the fundamental reason for the Incarnation underscores a theocentric understanding of Incarnation that widens the meaning of salvation and places it in a cosmic content. The author explores the primacy of Christ both in its historical context and with a contemporary view toward ecology, world religions, and extraterrestrial life, emphasizing the fullness of the mystery of Christ.
PB - SAGE Publications Inc.
SN - 00405639 (ISSN)
LA - English
J2 - Theol. Stud.
M3 - Article
DB - Scopus
N1 - Export Date: 23 February 2023; Cited By: 18
ER -

TY - JOUR
AU - Smith, M.
TI - Intriguing discoveries from mine fissure water organisms
PY - 2003
T2 - Water Wheel
VL - 2
IS - 5
SP - 12

EP - 15
UR - https://www.scopus.com/inward/record.uri?eid=2-s2.0-33646033494&partnerID=40&md5=a9ece2d6f6a9b180fecde756b90ce9b6
AB - The Astrobiology Institute of America's National Space Agency (NASA)is conducting research on underground water caught in deep anaerobic pockets in South Africa's gold mines. To date three new organisms, unknown to man until now, have been found in the fissure water pockets. A further 20 to 30 are also presumably new, and tests are ongoing to verify this. The project, started in 1996, can provide invaluable guidelines for the future design of instruments to detect life in these presumed waters on Mars missions.
KW - Groundwater
SN - 02582244 (ISSN)
LA - English
J2 - Water Wheel
M3 - Article
DB - Scopus
N1 - Export Date: 23 February 2023; Cited By: 0
ER -

TY - CONF
AU - Ehrenfreund, P.
AU - Cleaves, H.J.
TI - Origin of life
PY - 2003
T2 - European Space Agency, (Special Publication) ESA SP
IS - 539
SP - 195
EP - 203
UR - https://www.scopus.com/inward/record.uri?eid=2-s2.0-3142775812&partnerID=40&md5=79138d2ca4c500908bd0a91278725899
AD - Leiden Observatory, 2300 RA Leiden, P O Box 9513, Netherlands
AD - Soft Matter / Astrobiol. Laboratory, Leiden Institute of Chemistry, 2300 RA Leiden, PO Box 9502, Netherlands
AD - Dept. of Chemistry, UC San Diego, San Diego, CA 92093-0506, 9500 Gilman Drive, United States
AB - Deciphering the origin of life requires some knowledge of the early planetary environment. Unfortunately, we lack definitive evidence of the atmospheric composition, surface temperature, oceanic pH, and other environmental conditions that may have been important for the appearance of the first living systems on Earth. The rock remnants of the early Archean are extremely scarce and most of the record has been lost. The first indications of life from carbon inclusions in rocks and the oldest fossil record are currently under debate but there is a consensus that life started during the first billion years after the Earth formed. Life as we know it is a chemical phenomenon. The chemistry that could have produced self-organizing systems is the central problem in the origin of life. There are several competing theories for how this chemistry may have arisen, In spite of their diversity, proposals for a prebiotic "soup", for the role of submarine hydrothermal vents, or for the extraterrestrial origin of organic compounds have as a common background assumption the idea that abiotic organic compounds were necessary for the emergence of life. It is possible

that a combination of these sources - exogenous and endogenous - contributed building blocks for the origin of life on Earth. In this paper we provide a review of the main ideas on the origin of life from the astrobiological perspective and discuss the probability of life on extrasolar planets.
KW - Absorption
KW - Amino acids
KW - Atmospheric composition
KW - Biosensors
KW - Catalysis
KW - Cells
KW - Earth (planet)
KW - Environmental impact
KW - Galaxies
KW - Nanotechnology
KW - Organic compounds
KW - pH effects
KW - Polycyclic aromatic hydrocarbons
KW - Solar system
KW - Ultraviolet radiation
KW - Aromatic rings
KW - Biomolecules
KW - Heavy elements
KW - Nucleosynthesis
KW - Biochemistry
SN - 03796566 (ISSN)
LA - English
J2 - Eur Space Agency Spec Publ ESA SP
M3 - Conference paper
DB - Scopus
N1 - Export Date: 23 February 2023; Cited By: 0; CODEN: ESPUD
ER -

TY - JOUR
AU - Wentworth, S.J.
AU - Morris, P.A.
TI - Astrobiology: Introduction
PY - 2003
T2 - Astrobiology
VL - 3
IS - 3
SP - 581
EP - 582
DO - 10.1089/153110703322610663
UR - https://www.scopus.com/inward/record.uri?eid=2-s2.0-69249151312&doi=10.1089%2f153110703322610663&partnerID=40&md5=5fd7f694c7ac6dbaa377f48c657c043a
AD - Lockheed Martin, Mail Code C23, Houston, TX 77058, 2400 NASA Parkway, United States
AD - Department of Natural Science, University of Houston-Downtown, Houston, TX, United States
KW - Biogenesis
KW - Exobiology
KW - Life

KW - United States
KW - United States National Aeronautics and Space Administration
KW - Water
KW - water
KW - article
KW - biogenesis
KW - biology
KW - government
KW - life
KW - methodology
KW - United States
SN - 15311074 (ISSN)
C2 - 14678666
LA - English
J2 - Astrobiology
M3 - Editorial
DB - Scopus
N1 - Export Date: 23 February 2023; Cited By: 1; Correspondence Address: S. J. Wentworth; Lockheed Martin, Mail Code C23, Houston, TX 77058, 2400 NASA Parkway, United States; email: susan.j.wentworth@jsc.nasa.gov
ER -

TY - CONF
AU - Jones, H.
TI - Searching for alien life having unearthly biochemistry
PY - 2003
T2 - SAE Technical Papers
DO - 10.4271/2003-01-2668
UR - https://www.scopus.com/inward/record.uri?eid=2-s2.0-85072417590&doi=10.4271%2f2003-01-2668&partnerID=40&md5=19b083ba7d636d27c847a9f2fa7f9b80
AD - NASA Ames Research Center, Mail Stop 239-8, Moffett Field, CA 94035-1000, United States
AB - The search for alien life in the solar system should include exploring unearthlike environments for life having an unearthly biochemistry. We expect alien life to conform to the same basic chemical and ecological constraints as terrestrial life, since inorganic chemistry and the laws of ecosystems appear to be universal. Astrobiologists usually assume alien life will use familiar terrestrial biochemistry and therefore hope to find alien life by searching near water or by supplying hydrocarbons. The assumption that alien life is likely to be based on carbon and water is traditional and plausible. It justifies high priority for missions to search for alien life on Mars and Europa, but it unduly restricts the search for alien life. Terrestrial carbon-water biochemistry is not possible on most of the bodies of our solar system, but all alien life is not necessarily based on terrestrial biochemistry. If alien life has a separate origin from Earth life, and if can survive in an environment extremely different from Earth's, then alien life may have unearthly biochemistry. There may be other solvents than water that support alien life and other elements than carbon that form complex life enabling chain molecules. Rather than making the exploration-restricting assumption

that all life requires carbon, water, and terrestrial biochemistry, we should make the exploration-friendly assumption that indigenous, environmentally adapted, alien life forms might flourish using unearthly biochemistry in many places in the solar system. Alien life might be found wherever there is free energy and a physical/ chemical system capable of using that energy to build living structures. Alien life may be discovered by the detection of some general non-equilibrium chemistry rather than of terrestrial biochemistry. We should explore all the potential abodes of life in the solar system, including those where life based on terrestrial biochemistry can not exist.
KW - Ecology
KW - Free energy
KW - Inorganic compounds
KW - Solar system
KW - Basic chemicals
KW - Chain molecules
KW - Complex life
KW - Environmentally adapted
KW - Inorganic chemistry
KW - Life form
KW - Life on Mars
KW - Non equilibrium
KW - Biochemistry
PB - SAE International
SN - 01487191 (ISSN)
LA - English
J2 - SAE Techni. Paper.
M3 - Conference paper
DB - Scopus
N1 - Export Date: 23 February 2023; Cited By: 1; Correspondence Address: H. Jones; NASA Ames Research Center, Mail Stop 239-8, Moffett Field, CA 94035-1000, United States; email: Harry.W.Jones@nasa.gov
ER -

TY - CONF
AU - Rummel, J.D.
AU - Meyer, M.A.
AU - Lavery, D.B.
TI - NASA'S ASTEP program: Astrobiology science and technology for exploring planets
PY - 2003
T2 - 54th International Astronautical Congress of the International Astronautical Federation (IAF), the International Academy of Astronautics and the International Institute of Space Law
VL - 1
SP - 1295
EP - 1302
UR - https://www.scopus.com/inward/record.uri?eid=2-s2.0-22344442701&partnerID=40&md5=9691ad9d872e8a97866404ebfdcc220f
AD - NASA Headquarters, Washington, DC, United States
AB - NASA's ASTEP Program is an aspect of its ongoing Astrobiology Program, an effort to study three questions about life in the

universe: How does life begin and evolve? Does life exist elsewhere in the universe? What is the future for life on Earth and beyond? In the study of the extreme environments of Earth and other planets, new methods and capabilities for long-term observations and detailed examinations of surface and subsurface environments are needed. ASTEP is intended to comprise a series of terrestrial field projects to define techniques and methods and to validate technologies for long-term planetary science operations, sub-surface access, and in-situ searches for evidence of life - concentrating on analogs of possible life-supporting environments throughout the solar system. For example, the exploration of sub-ice oceans on Europa may entail the use of sub-sea exploration platforms to identify and characterize new environments and life using advanced sensors and instruments. Also needed will be autonomous deployment and operations, and advanced power sources, as well as ice penetration techniques and technologies for robustly passing surface/ice and ice/water interfaces, etc. For the further exploration of other planetary bodies, as well as of sensitive environments like Lake Vostok, a new approach to planetary protection and sample handling will be essential. This paper will review the overall program, the initial project selections made by NASA, and future program planning.
KW - Astrophysics
KW - Constraint theory
KW - Earth (planet)
KW - Gravitation
KW - Ice
KW - Spacecraft
KW - Spacecraft landing
KW - Thermal effects
KW - Water
KW - Astrobiology
KW - Planetary exploration
KW - Planetary mission
KW - Subsurface environments
KW - Planetary surface analysis
LA - English
J2 - Int. Astronaut. Congr. Int. Astronaut. Fed. IAF Int. Acad. Astronaut. Int. Inst. Space Law
M3 - Conference paper
DB - Scopus
N1 - Export Date: 23 February 2023; Cited By: 0
ER -

TY - JOUR
AU - Battersby, S.
TI - The petrolheads of Titan
PY - 2004
T2 - New Scientist
VL - 184
IS - 2470
SP - 43
EP - 46
UR - https://www.scopus.com/inward/record.uri?eid=2-

s2.0-7544232165&partnerID=40&md5=79bcaad37a84c5c83c1ba1df1cced0a0
AB - The nature of terrestrial atmosphere on Saturn's largest moon, Titan, is discussed. The atmosphere of Titan contains organic chemicals which are perfect for building the molecules of life. Its atmosphere is the most Earth like, composed mostly of nitrogen and has only slightly higher pressure. Titan even has clouds made of methane and other hydrocarbon rather than water. The surface temperature of the Titan is around 95 Kelvin, or about 180 °Celcius below freezing.
KW - Computer simulation
KW - Earth (planet)
KW - Hydrocarbons
KW - Microorganisms
KW - Moon
KW - Nitrogen
KW - Organic compounds
KW - Photosynthesis
KW - Space applications
KW - Space research
KW - Spacecraft
KW - Surfaces
KW - Temperature distribution
KW - Water
KW - Astrobiologists
KW - Cartographers
KW - Organic reactions
KW - Titan
KW - Extraterrestrial atmospheres
SN - 02624079 (ISSN)
LA - English
J2 - New Sci.
M3 - Article
DB - Scopus
N1 - Export Date: 23 February 2023; Cited By: 0; CODEN: NWSCA
ER -

TY - JOUR
AU - Bulat, S.A.
AU - Alekhina, I.A.
AU - Blot, M.
AU - Petit, J.-R.
AU - de Angelis, M.
AU - Wagenbach, D.
AU - Lipenkov, V.Y.
AU - Vasilyeva, L.P.
AU - Wloch, D.M.
AU - Raynaud, D.
AU - Lukin, V.V.
TI - DNA signature of thermophilic bacteria from the aged accretion ice of Lake Vostok, Antarctica: Implications for searching for life in extreme icy environments
PY - 2004
T2 - International Journal of Astrobiology
VL - 3

IS - 1
SP - 1
EP - 12
DO - 10.1017/S1473550404001879
UR - https://www.scopus.com/inward/record.uri?eid=2-s2.0-25844439256&doi=10.1017%2fS1473550404001879&partnerID=40&md5=9f41e0a919e676ddad948a9cd069e1e4
AD - Division of Molecular and Radiation Biophysics, Petersburg Nuclear Physics Institute, Gatchina 188300, Leningrad region, Russian Federation
AD - Plasticité et Expression des Génomes Microbiens, UJF, CNRS, Grenoble 38041, France
AD - Laboratoire de Glaciologie, Géophysique de l'Environnement, CNRS, BP96, Saint Martin d'Hères 38402, France
AD - Institut für Umweltphysik, INF 229 Heidelberg 69120, Germany
AD - Arctic and Antarctic Research Institute, St. Petersburg 199397, 38 Bering Str., Russian Federation
AB - We have used 16S ribosomal genes to estimate the bacterial contents of Lake Vostok accretion ice samples at 3551 m and 3607 m, both containing sediment inclusions and formed 20000–15000 yr ago. Decontamination proved to be a critical issue, and we used stringent ice chemistry-based procedures and comprehensive biological controls in order to restrain contamination. As a result, up to now we have only recognized one 16S rDNA bacterial phylotype with confident relevance to the lake environment. It was found in one sample at 3607 m depth and represents the extant thermophilic facultative chemolithoautotroph Hydrogenophilus thermoluteolus of beta-Proteobacteria, and until now had only been found in hot springs. No confident findings were detected in the sample at 3551 m, and all other phylotypes revealed (a total of 16 phylotypes, 336 clones including controls) are presumed to be contaminants. It seems that the Lake Vostok accretion ice is actually microbe-free, indicating that the water body should also be hosting a highly sparse life. The message of thermophilic bacteria suggests that a geothermal system exists beneath the cold water body of Lake Vostok, what is supported by the geological setting, the long-term seismotectonic evidence from 4He degassing and the ‘18O shift’ of the Vostok accretion ice. The seismotectonic activity that seems to operate in deep faults beneath the lake could sustain thermophilic chemolithoautotrophic microbial communities. Such a primary production scenario for Lake Vostok may have relevance for icy planets and the approaches used for estimating microbial contents in accretion ice are clearly relevant for searching for extraterrestrial life. © 2004, Cambridge University Press. All rights reserved.
KW - accretion ice
KW - Antarctica
KW - bacteria
KW - contamination
KW - exobiology
KW - geothermal environment
KW - Lake Vostok
KW - subglacial lake environments
KW - thermophiles
SN - 14735504 (ISSN)

LA - English
J2 - Int. J. Astrobiology
M3 - Article
DB - Scopus
N1 - Export Date: 23 February 2023; Cited By: 84
ER -

TY - CONF
AU - Kruzelecky, R.V.
AU - Haddad, E.
AU - Wong, B.
AU - Lafrance, D.
AU - Jamroz, W.
AU - Ghosh, A.K.
AU - Zheng, W.
AU - Phong, L.
TI - Miniature high-performance infrared spectrometer for space applications
PY - 2004
T2 - European Space Agency, (Special Publication) ESA SP
IS - 554
SP - 203
EP - 210
UR - https://www.scopus.com/inward/record.uri?eid=2-s2.0-8744248405&partnerID=40&md5=d4220ce2798b37b6d79014ccc7f54892
AD - MPB Communications Inc., Pointe Claire, Que. H9R-1E9, 151 Hymus, Canada
AD - Canadian Space Agency, Saint Hubert, Que. J3Y-8Y9, 6767 Route de l'Aéroport, Canada
AB - Infrared spectroscopy probes the characteristic vibrational and rotational modes of chemical bonds in molecules to provide information about both the chemical composition and the bonding configuration of a sample. The significant advantage of the Infrared spectral technique is that it can be used with minimal consumables to simultaneously detect a large variety of chemical and biochemical species with high chemical specificity. To date, relatively large Fourier Transform (FT-IR) spectrometers employing variations of the Michelson interferometer have been successfully employed in space for various IR spectroscopy applications. However, FT-IR systems are mechanically complex, bulky (> 15 kg), and require considerable processing. This paper discusses the use of advanced integrated optics and smart optical coding techniques to significantly extend the performance of miniature IR spectrometers by several orders of magnitude in sensitivity. This can provide the next-generation of compact, high-performance IR spectrometers with monolithically integrated optical systems for robust optical alignment. The entire module can weigh under 3 kg to minimize the mass penalty for space applications. Miniaturized IR spectrometers are versatile and very convenient for small and micro satellite based missions. They can be dedicated to the monitoring of the CO2 in an Earth Observation mission, to Mars exobiology exploration, as well as to vital life support in manned space system; such as the cabin air quality and the quality of the recycled water supply.
KW - Fourier transform infrared spectroscopy

KW - Geostationary satellites
KW - Infrared radiation
KW - Interferometers
KW - Signal to noise ratio
KW - Spectroscopic analysis
KW - Astrobiology
KW - Earth observations
KW - Planetary geology
KW - Satellite based missions
KW - Spectrometers
SN - 03796566 (ISSN)
LA - English
J2 - Eur Space Agency Spec Publ ESA SP
M3 - Conference paper
DB - Scopus
N1 - Export Date: 23 February 2023; Cited By: 2; CODEN: ESPUD
ER -

TY - JOUR
AU - Cockell, C.S.
TI - Impact-shocked rocks - insights into archean and extraterrestrial microbial habitats (and sites for prebiotic chemistry?)
PY - 2004
T2 - Advances in Space Research
VL - 33
IS - 8
SP - 1231
EP - 1235
DO - 10.1016/j.asr.2003.06.027
UR - https://www.scopus.com/inward/record.uri?eid=2-s2.0-2342488886&doi=10.1016%2fj.asr.2003.06.027&partnerID=40&md5=f4ba227d619644a7c151c0f26d4c9d5e
AD - SETI Institute, NASA Ames Research Center, Moffett Field, CA 94035-1000, United States
AD - British Antarctic Survey, Cambridge CB3 0ET, Madingley Road, United Kingdom
AB - Impact-shocked gneiss shocked to greater than 10 GPa in the Haughton impact structure in the Canadian High Arctic has an approximately 25-times greater pore surface area than unshocked rocks. These pore spaces provide microhabitats for a diversity of heterotrophic microorganisms and in the near-surface environment of the rocks, where light levels are sufficient, cyanobacteria. Shocked rocks provide a moisture retaining, UV protected microenvironment. During the Archean, when impact fluxes were more than two orders of magnitude higher than today, the shocked-rock habitat was one of the most common terrestrial habitats and might have provided a UV-shielded refugium for primitive life. These potential habitats are in high abundance on Mars where impact crater habitats could have existed over geologic time periods of billions of years, suggesting that impact-shocked rocks are important sites to search for biomolecules in extraterrestrial life detection strategies. In addition to being favourable sites for life, during the prebiotic period of planetary history impact-shocked rocks might have acted as

a site for the concentration of reactants for prebiotic syntheses. © 2004 COSPAR. Published by Elsevier Ltd. All rights reserved.
KW - Extraterrestrial atmospheres
KW - Microorganisms
KW - Moisture
KW - Ocean habitats
KW - Pore size
KW - Ultraviolet radiation
KW - Impact-shocked rocks
KW - Pore spaces
KW - Prebiotic syntheses
KW - Primitive life
KW - Terrestrial habitats
KW - Archean
KW - exobiology
KW - impact
KW - origin of life
KW - shock metamorphism
KW - Rocks
KW - Archean extraterrestrial microbial habitats
KW - Astrobiology
KW - Prebiotic chemistry
PB - Elsevier Ltd
SN - 02731177 (ISSN)
C2 - 15803603
LA - English
J2 - Adv. Space Res.
M3 - Article
DB - Scopus
N1 - Export Date: 23 February 2023; Cited By: 13; Correspondence Address: C.S. Cockell; SETI Institute, NASA Ames Research Center, British Antarctic Survey, Moffett Field, CA 94035-1000, United States; email: csco@bas.ac.uk; CODEN: ASRSD
ER -

TY - CONF
AU - De Pablo, M.A.
AU - Fairén, A.G.
TI - Atlantis basin, mars: Geologic setting and astrobiological implications
PY - 2004
T2 - European Space Agency, (Special Publication) ESA SP
IS - 545
SP - 195
EP - 196
UR - https://www.scopus.com/inward/record.uri?eid=2-s2.0-2342571015&partnerID=40&md5=0e9021c9d63fe9a6daba93c9784bc1bd
AD - Área de Geología, Departamento de Matemática, Universidad Rey Juan Carlos, 28933 Móstoles, Madrid, Spain
AD - Centro de Biología Molecular, CSIC, Univ. Autónoma de Madrid, 28049-Cantoblanco, Madrid, Spain
AB - The accomplishment of detailed geological studies is a prerequisite for the location of regions in which the prevailing conditions in the past, or at present, may allow the development of

possible forms of life. The Atlantis basin, located in the Southern hemisphere of Mars, is one of these astrobiological interesting regions, where the existence of some geological features like ancient volcanic edifices, sedimentary deposits of an ancient lake, and virtually contemporary gullies, seem to indicate the long-term presence of a thermal source and a water reservoir sufficiently stable and potent to sustain biological processes.
KW - Geology
KW - Lakes
KW - Remote sensing
KW - Reservoirs (water)
KW - Sediments
KW - Tectonics
KW - Thermal effects
KW - Volcanoes
KW - Craters
KW - Edifices
KW - Quadrangles
KW - Ridges
KW - Martian surface analysis
SN - 03796566 (ISSN)
LA - English
J2 - Eur Space Agency Spec Publ ESA SP
M3 - Conference paper
DB - Scopus
N1 - Export Date: 23 February 2023; Cited By: 0; Correspondence Address: M.A. De Pablo; Área de Geología, Departamento de Matemática, Universidad Rey Juan Carlos, 28933 Móstoles, Madrid, Spain; email: madepablo@escet.urjc.es; CODEN: ESPUD
ER -

TY - JOUR
AU - Ćirković, M.M.
AU - Cathcart, R.B.
TI - Geo-engineering gone awry: A new partial solution of Fermi's paradox
PY - 2004
T2 - JBIS - Journal British Interplanetary Society
VL - 57
IS - 5-6
SP - 209
EP - 215
UR - https://www.scopus.com/inward/record.uri?eid=2-s2.0-2642551518&partnerID=40&md5=42adcb3970c40699de7275d0c855004f
AD - Astronomical Observatory Belgrade, 11160 Belgrade, Volgina 7, Serbia
AD - Geographos, Glendale, CA 91205-1524, 1608 East Broadway, Serbia
AB - Another partial solution of Fermi's famous paradox is proposed, based on our increased understanding of geophysics, geo-engineering, and climatology. It has been claimed in the recent astrobiological literature (for instance, in the recent controversial "rare Earth" theory of Ward and Brownlee), that geological activity of a terrestrial planet is an important

precondition for the emergence of complex metazoan life forms. Technological civilizations arising on such planets will be, at some point of their histories or another, tempted to embark upon massive geo-engineering projects. If, for some reasons only very recently understood, large-scale geo-engineering is in fact much more dangerous than previously thought, the scenario in which at least some of the extraterrestrial civilizations in the Milky Way self-destruct in this manner gains plausibility. In addition, we speculate on possible reasons, both physical and culturological, which could make such a threat even more pertinent on an average Galactic terrestrial planet than on Earth.
KW - Climatology
KW - Extraterrestrial atmospheres
KW - Interplanetary flight
KW - Moon
KW - Risk assessment
KW - Solar system
KW - Thermonuclear reactions
KW - Astrobiology
KW - Existential risks
KW - Extraterrestrial intelligence
KW - Geophysics
KW - Astrobiology
KW - Existential risks
KW - Extraterrestrial intelligence
KW - Geophysics
PB - British Interplanetary Society
SN - 0007084X (ISSN)
LA - English
J2 - JBIS J. Br. Interplanet. Soc.
M3 - Review
DB - Scopus
N1 - Export Date: 23 February 2023; Cited By: 4; Correspondence Address: M.M. Ćirković; Astronomical Observatory Belgrade, 11160 Belgrade, Volgina 7, Serbia; email: arioch@eunet.yu
ER -

TY - JOUR
AU - Bignami, G.F.
TI - La vita e i pianeti: Il mito e la scienza
PY - 2004
T2 - ATTI Della Accademia Nazionale Dei Lincei Rendiconti Lincei Scienze Fisiche E Naturali
VL - 15
IS - 3
SP - 267
EP - 279
DO - 10.1007/BF02904466
UR - https://www.scopus.com/inward/record.uri?eid=2-s2.0-20744443184&doi=10.1007%2fBF02904466&partnerID=40&md5=7e914252f4678f99bbdde949dd3399ca
AD - Dipartimento di Fisica Nucleate e Teorica, Università degli Studi di Pavia, 27100 Pavia, Via U. Bassi, 6, Italy
AD - Centre d'Etude Spatiale des Rayonnements, 31028 Toulouse

Cedex, 9, avenue du Colonel Roche, France
AB - Life and planets, myth and science. While the myth of extraterrestrial life is as old as mankind, it has taken up special impetus during the last 400 years of astronomy, i.e. since Galileo gave us the telescope in 1610. The greatest astronomical hoax in history, the now forgotten Lunar Hoax of 1835, was perpetrated by the New York Sun, riding on the success of the South African telescope of Sir William Herschel, the VLT of those days. Pulcinella, in Naples, reads a translation of the American daily but does not believe it. He decides to pay a visit to the moon in person, using a special sailing ship on spoked wheels. A few years later, but still seven years before Jules Vernes, Cappocci, director of the Naples Observatory, writes a booklet on the first trip of a woman to the moon. Even the French rationalism of Flammation is inflamed by such fantasies, and includes them in his extremely popular astronomy books of the end of the 1800s. In those very years, Schiaparelli discovered «canals» on Mars, starting the legend of a Martian race, rendered intelligent by the necessity of survival in a hostile environment. Such legends were much enhanced by the work of Percival Lowell and survived intact until the Nature 1966 article of Carl Sagan. In vast layers of our contemporary society such myths remain alive. The last 40 years of space astronomy, from Mariner 4 of 1964 to Mars Express of 2004, are changing our way of accepting the possibility of life on a planet like Mars. We are living through a historic moment in science. We might be getting final answers on extraterrestrial life in the very near future. It is more probable, however, that we will just have more questions. In the last ten years, astronomy has given us over 100 extra-solar planets, albeit possibly none that is of a «habitable» type. More recently another line of research, exploiting the data of the European satellite Hipparcus, has resulted in the discovery of 17,129 «habstars» closer than 500 light years from us, i.e. in our immediate galactic neighbourhood. A «habstar» is a star whose lifetime, luminosity and stability allows any planets it may have to sustain liquid water for more than three billion years, and thus possibly support life. For sure, we can't go there, but they certainly represent our best targets from which to listen for intelligent messages. The Earth too is being heard in space, through its «Marconi bubble» of electromagnetic radiation which has been expanding at the velocity of light for the last fifty years. Moreover, four NASA planetary probes, launched 30 years ago, are now leaving our solar system carrying with them into the interstellar space, messages, images and sounds of planet Earth.
KW - Extraterrestrial life
KW - History of astronomy
KW - Planetary astronomy
PB - Accademia Nazionale dei Lincei
SN - 11206349 (ISSN)
LA - Italian
J2 - ATTI Accad Naz Lincei Rend Lincei Sci Fis Nat
M3 - Article
DB - Scopus
N1 - Export Date: 23 February 2023; Cited By: 1; Correspondence Address: G.F. Bignami; Dipartimento di Fisica Nucleate e Teorica,

Università degli Studi di Pavia, 27100 Pavia, Via U. Bassi, 6, Italy; email: giovanni.bignami@cesr.fr; CODEN: ANLNE
ER -

TY - CONF
AU - Lipps, J.H.
AU - Delory, G.
AU - Pitman, J.
AU - Rieboldt, S.
TI - Astrobiology of Jupiter's icy moons
PY - 2004
T2 - Proceedings of SPIE - The International Society for Optical Engineering
VL - 5555
C7 - 10
SP - 78
EP - 92
DO - 10.1117/12.560356
UR - https://www.scopus.com/inward/record.uri?eid=2-s2.0-14944352733&doi=10.1117%2f12.560356&partnerID=40&md5=c72a51a60de4fa095ea838df73c05b04
AD - Department of Integrative Biology, Museum of Paleontology, University of California, Berkeley, CA 94720, United States
AD - Ctr. for Integrative Planet. Sci., University of California, Berkeley, CA 94720, United States
AD - Space Sciences Laboratory, University of California, Berkeley, CA 94720, United States
AD - Lockheed Martin Adv. Technol. Center, Palo Alto, CA 94304-1191, United States
AB - Jupiter's Icy Moons, Europa, Ganymede and Callisto, may possess energy sources, biogenic molecules, and oceans below their icy crusts, thus indicating a strong possibility that they were abodes for present or past life. Life in Earth's icy areas lives in a wide variety of habitats associated with the ice, in the water column below the ice, and on the floor of the ocean below the ice. Similar habitats may exist on JIM, have been transported to the icy crust, and be exposed in tectonic or impact features. Europa has a young, dynamic surface with many outcrops exposing older ice, fresh ice, possible materials from the subsurface ocean, and a few impact craters. Ganymede has older, darker, tectonized terrains surrounded by light ice. Callisto has a much older, heavily impacted surface devoid of significant tectonic structures. Past and present life habitats may be exposed in these features, making Europa the most favorable target while Ganymede is of interest, and Callisto seems more unlikely to have detectable life. A proper search strategy requires detailed orbital imaging and spectrometry of the likely places, and surface data collection with microscopic, spectrometric, and biochemical instruments.
KW - Earth (planet)
KW - Energy resources
KW - Ice
KW - Marine biology
KW - Microorganisms
KW - Microscopic examination

KW - Moon
KW - Oceanography
KW - Planets
KW - Remote sensing
KW - Spectrometry
KW - Tectonics
KW - Water
KW - Astrobiology
KW - Callisto
KW - Europa
KW - Ganymede
KW - Ice organisms
KW - Jupiter's icy moons
KW - Life
KW - Biochemistry
KW - Astrobiology
KW - Callisto
KW - Europa
KW - Ganymede
KW - Ice organisms
KW - Jupiter's icy moons
KW - Life
KW - Remote sensing
SN - 0277786X (ISSN)
LA - English
J2 - Proc SPIE Int Soc Opt Eng
M3 - Conference paper
DB - Scopus
N1 - Export Date: 23 February 2023; Cited By: 4; CODEN: PSISD
ER -

TY - CONF
AU - Prockter, L.
TI - The Jupiter icy moons orbiter: An opportunity for unprecedented exploration of the Galilean satellites
PY - 2004
T2 - International Astronautical Federation - 55th International Astronautical Congress 2004
VL - 4
SP - 2708
EP - 2713
UR - https://www.scopus.com/inward/record.uri?eid=2-s2.0-34249085296&partnerID=40&md5=ab99631bf14bbf808bf2e48b4a01e866
AD - Johns Hopkins University, Applied Physics Laboratory, MP3-E128, Laurel, MD 20723, 11100 Johns Hopkins Road, United States
AB - The Jupiter Icy Moons Orbiter (JIMO) will be the first spacecraft in NASA's new Project Prometheus Program, with a projected launch date of 2015. A Science Definition Team (SDT) was assembled to determine the scientific goals and objectives of the mission, based on the enhanced capabilities provided by a nuclear reactor and nuclear electric propulsion (NEP), and building on recommendations from the Solar System Exploration Division Roadmap [1] and the National Research Council Solar System Decadal Survey [2]. The SDT report was delivered to NASA in February 2004 [3] and

the main findings of the report are summarized in this paper. The high power levels, data rates, and mission delivery capabilities provided by nuclear electric propulsion will enable the JIMO spacecraft to accommodate new advanced investigations. Potential investigations include super high resolution optical and hyperspectral imagers, high resolution topographic SARs, and high power active laser spectrometers. The JIMO SDT recommended that the payload mass should be ~1500 kg in order for reasonable advances in scientific study to be made, and that up to 25% of spacecraft resources should be made available for a landed science package for Europa, with an emphasis on astrobiological and geophysical investigations that cannot be carried out from orbit.
KW - Image sensors
KW - Lunar missions
KW - NASA
KW - Nuclear reactors
KW - Project management
KW - Spacecraft propulsion
KW - Laser spectrometers
KW - Nuclear electric propulsion (NEP)
KW - Solar System Exploration
KW - Satellites
LA - English
J2 - Int. Astro. Fed. Int. Astro. Congr.
M3 - Conference paper
DB - Scopus
N1 - Export Date: 23 February 2023; Cited By: 1; Correspondence Address: L. Prockter; Johns Hopkins University, Applied Physics Laboratory, MP3-E128, Laurel, MD 20723, 11100 Johns Hopkins Road, United States; email: Louise.Prockter@jhuapl.edu
ER -

TY - CONF
AU - Fairén, A.G.
AU - Dohm, J.M.
AU - Baker, V.R.
AU - Uceda, E.R.
AU - Ruiz, J.
AU - Cortés, D.
AU - Amils, R.
TI - Prime candidate sites for the astrobiological exploration of Mars according to its hydrogeological history
PY - 2004
T2 - European Space Agency, (Special Publication) ESA SP
IS - 545
SP - 201
EP - 202
UR - https://www.scopus.com/inward/record.uri?eid=2-s2.0-2342456493&partnerID=40&md5=e5106310f1ea1da40846b5b2c36edf96
AD - Centro de Biología Molecular, CSIC, Univ. Autónoma de Madrid, 28049-Cantoblanco, Madrid, Spain
AD - Department of Hydrology, University of Arizona, Tucson, AZ 85721, United States
AD - Lunar and Planetary Laboratory, University of Arizona, Tucson,

AZ 85721, United States
AD - S.E. Hospital Ramón y Cajal, 28937, Madrid, Ctra. Colmenar, km. 9, Spain
AD - Departamento de Geodinámica, Universidad Complutense de Madrid, 28040, Madrid, Spain
AD - Depto. de Ing. Industrial, Universidad Antonio de Nebrija, 28040-Madrid, Spain
AD - Centro de Astrobiologia (CSIC-INTA), 28855-Torrejon de Ardoz, Madrid, Spain
AB - Three prime candidate sites for the astrobiological exploration of Mars have been identified on the basis of its hydrogeological history. Each of the sites corresponds to a major inundation phase of the global hydrological model. The Terra Meridiani site is related to the Noachian to Hesperian time, which suggest an aqueous-hydrothermal origin. The Mangala Valles relates to Late Hesperian to Early Amazonian time, where diminishing martian episodic hydrologic events over geologic time are recorded representing a later pulse of tharsis-driven hydrologic activity. The Orcus Patera site relates to Amazonian time, which is a volcanic caldera or impact crater where reduced ponded bodied of water may had existed during almost contemporary times.
KW - Astronomy
KW - Biomarkers
KW - Earth (planet)
KW - Hydrogeology
KW - Hydrology
KW - Aqueous-hydrothermal origin
KW - Astrobiology
KW - Inundation phase
KW - Martian surface analysis
SN - 03796566 (ISSN)
LA - English
J2 - Eur Space Agency Spec Publ ESA SP
M3 - Conference paper
DB - Scopus
N1 - Export Date: 23 February 2023; Cited By: 0; Correspondence Address: A.G. Fairén; Centro de Biología Molecular, CSIC, Univ. Autónoma de Madrid, 28049-Cantoblanco, Madrid, Spain; email: agfairen@cbm.uam.es; CODEN: ESPUD
ER -

TY - CONF
AU - Gormly, S.
AU - Adams, V.D.
AU - Marchand, E.
TI - Engineering methods for Europan relevant biosignature development
PY - 2004
T2 - SAE Technical Papers
DO - 10.4271/2004-01-2410
UR - https://www.scopus.com/inward/record.uri?eid=2-s2.0-85072440399&doi=10.4271%2f2004-01-2410&partnerID=40&md5=f7efdb58a248ad669982156ac7e9e9f4
AD - Department of Civil and Environmental Engineering, United

States
AB - One of the primary driving forces for space exploration in the foreseeable future is astrobiology, and specifically the search for a plausible sign of life beyond Earth. Because of the size of the potential saltwater ocean involved, Europa is potentially the most interesting, and possibly the only, currently viable (for life) environment in the solar system. It also presents the possibility of remote sensing evaluation for presence or absence of biotic and/or pre-biotic organic material. The material of interest is the non-ice (referring to water ice) surface material near features that have the potential of being in recent communication with the postulated ocean below. An analysis of this material using a full range of inorganic, pre-biotic organic, and metabolically relevant biologic materials as spectrum calibrating target materials, examined under Europan surface conditions, is a daunting proposition. A comprehensive attempt is still pending. This study involves the collection and growth of psychrophilic (low temperature), halophilic (high salt), anaerobic cultures in high sulfate environments. These cultures are intended as reflected spectrum target materials, based on relevant biomass, for comparison to Europan non-ice surface materials. The selection, collection, development (growth) and examination of this biotic material (biomass) requires the use of stringent, and in some cases extreme, environmental controls. It also requires the extrapolation of standard environmental engineering sampling and laboratory analysis procedures for use in comparison with, and interpretation of, data from a profoundly extreme and unfamiliar environment. The function of this study is to demonstrate the use of environmental engineering techniques and processes (test methodology) necessary to develope a reasonable biosignature related to the Europan target environment. Copyright © 2004 SAE International.
KW - Earth (planet)
KW - Ecology
KW - Remote sensing
KW - Space research
KW - Sulfur compounds
KW - Temperature
KW - Anaerobic cultures
KW - Engineering methods
KW - Environmental control
KW - Laboratory analysis
KW - Reflected spectrum
KW - Remote sensing evaluation
KW - Space explorations
KW - Surface conditions
KW - Ice
PB - SAE International
SN - 01487191 (ISSN)
LA - English
J2 - SAE Techni. Paper.
M3 - Conference paper
DB - Scopus
N1 - Export Date: 23 February 2023; Cited By: 0
ER -

TY - CONF
AU - Öman, J.
TI - Terraforming celestial bodies
PY - 2004
T2 - International Astronautical Federation - 55th International Astronautical Congress 2004
VL - 5
SP - 3171
EP - 3182
UR - https://www.scopus.com/inward/record.uri?eid=2-s2.0-34249019190&partnerID=40&md5=cfd5be549b256596c4569bfbec7cea70
AD - Lulea University of Technology, Sweden
AB - The general aim of this paper is to illustrate the extent of Terraforming and the driving factors that would actually make us undertake a project of such magnitude. The word Terraforming means changing an environment until it becomes habitable for life forms from Earth. The concept of Terraforming is interdisciplinary and concerns political and social aspects as well as technological. As many of us live in a world where we do not have to consider basic needs, such as breathable air, warmth, food and a territory to exist on, it might be hard to argue in favor for leaving our 'carefree' lives to barely endure as settlers on another planet. Just to survive is no longer humans' most important issue but to live in a world where social, political and economical forces drives our needs rather than the search for food and shelter. The topic of discussion is mainly but not only the technological difficulties starting an Earth colony on another planet, but also how to motivate people to leave Tellus and move to a settlement somewhere else. Perhaps the most important question is the technical feasibility of Terraforming. There are numerous physical quantities that must be held within certain boundaries to meet the requirement of Earth life forms, which more or less need to be altered on a new celestial body. Human life can exist within a very narrow spectrum of these quantities and even though we might alter DNA to our favor, we still need to breathe a composition of molecules, to keep our temperature stable and consume nutrients. Substantial amounts of water must be available, preferably in liquid form, and the territory in which we can exist, must not be small. A hot subject in this field is putting colonies on the moon and on our neighbouring planet Mars, something that has generated several surveys and popular science articles. This paper is more aimed towards a general way of Terraforming and will not include detailed analyses of technological solutions. Discussions are more examples of approach and to spare the reader from more Mars gibberish the sister planet Venus and Jupiter's moon Europa stands as examples of planetary engineering. A small section is devoted to mainly hypothetical assumptions about how Earth society will change during the years to come. Inspired by the famous Danish future scientist Tor Nørre Tranders and using material from various future scenario researchers this section offers interesting speculations about human development during the generations to come and a reason why terraforming technology might in the end be the one thing that guarantees the survival of human life. And as part of the course contents a general description of Astrobiology is included.

KW - DNA
KW - Earth (planet)
KW - Moon
KW - Nutrients
KW - Project management
KW - Social aspects
KW - Celestial body
KW - Terraforming
KW - Space research
LA - English
J2 - Int. Astro. Fed. Int. Astro. Congr.
M3 - Conference paper
DB - Scopus
N1 - Export Date: 23 February 2023; Cited By: 0; Correspondence Address: J. Öman; Lulea University of Technology, Sweden; email: Joaohm-9@student.ltu.se
ER -

TY - CONF
AU - DiGregorio, B.E.
TI - Accretionary lapilli, tektites or concretions: The ubiquitous spherules of Meridiani Planum
PY - 2004
T2 - Proceedings of SPIE - The International Society for Optical Engineering
VL - 5555
C7 - 15
SP - 139
EP - 152
DO - 10.1117/12.563673
UR - https://www.scopus.com/inward/record.uri?eid=2-s2.0-14944377597&doi=10.1117%2f12.563673&partnerID=40&md5=856ae7da0652f857c548ffcb2f675332
AD - Cardiff Centre for Astrobiology, Wales UK c/o, Middleport, NY 14105, 16 North Hartland Street, United States
AB - The ubiquitous spherules discovered by the NASA Mars Exploration Rover Opportunity (MER-B) at the Meridiani Planum landing site are discussed. Spherules are found attached to and embedded within sedimentary sulfate rock outcrops found at the landing area that is determined by the MER science team. The appearance of most of the Meridiani spherules is similar to the morphology and size of terrestrial accretionary lapilli and show similarities to terrestrial tektites. Evidence from Martian orbit and on the surface indicate that the Meridiani Planum landing ellipse is located within an ancient 800 km diameter impact structure with another 140 km crater under the site.
KW - Astronomy
KW - Earth (planet)
KW - Ice
KW - Image analysis
KW - Iron oxides
KW - Volcanic rocks
KW - Water
KW - Accretionary lapilli

KW - Astrobiology
KW - Concretions
KW - Hematite
KW - Hydrothermal alteration
KW - Meridiani Planum
KW - Rampart crater
KW - Spherules
KW - Tektites
KW - Martian surface analysis
KW - Accretionary lapilli
KW - Astrobiology
KW - Concretions
KW - Hematite
KW - Hydrothermal alteration
KW - Meridiani planum
KW - Rampart crater
KW - Spherules
KW - Tektites
SN - 0277786X (ISSN)
LA - English
J2 - Proc SPIE Int Soc Opt Eng
M3 - Conference paper
DB - Scopus
N1 - Export Date: 23 February 2023; Cited By: 0; Correspondence Address: B.E. DiGregorio; Cardiff Centre for Astrobiology, Wales UK c/o, Middleport, NY 14105, 16 North Hartland Street, United States; email: Icamsr@buffnet.net; CODEN: PSISD
ER -

TY - JOUR
AU - Cleaves II, H.J.
AU - Chalmers, J.H.
TI - Extremophiles may be irrelevant to the origin of life
PY - 2004
T2 - Astrobiology
VL - 4
IS - 1
SP - 1
EP - 9
DO - 10.1089/153110704773600195
UR - https://www.scopus.com/inward/record.uri?eid=2-s2.0-7944225672&doi=10.1089%2f153110704773600195&partnerID=40&md5=a161535e372fbce04e30ae024da9927f
AD - Geosciences Research Division, Scripps Institution of Oceanography, La Jolla, CA 92093-0212, 3270 Sverdrup Hall, United States
AD - Department of Chemistry and Biochemistry, The University of California San Diego, La Jolla, CA, United States
AB - In recent years, Bacteria and Archaea have been discovered living in practically every conceivable terrestrial environment, including some previously thought to be too extreme for survival. Exploration of our solar system has revealed a number of extraterrestrial bodies that harbor environments analogous to many of the terrestrial environments in which extremophiles flourish. The

recent discovery of more than 105 extrasolar planets suggests that planetary systems are quite common. These three findings have led some to speculate that life is therefore common in the universe, as life as we know it can seemingly survive almost anywhere there is liquid water. It is suggested here that while environments capable of supporting life may be common, this does not in itself support the notion that life is common in the universe. Given that interplanetary transfer of life may be unlikely, the actual origin of life may require specific environmental and eological conditions that may be much less common than the mere existence of liquid water. © Mary Ann Liebert, Inc.
KW - Archaea
KW - Bacteria
KW - Biogenesis
KW - Exobiology
KW - Archaea
KW - Bacteria (microorganisms)
KW - Archaebacterium
KW - article
KW - bacterium
KW - biogenesis
KW - biology
KW - Exobiology
KW - Extraterrestrial life
KW - Extreme environments
KW - Extremophiles
KW - Liquid water
KW - Origin of life
PB - Mary Ann Liebert Inc.
SN - 15311074 (ISSN)
C2 - 15104899
LA - English
J2 - Astrobiology
M3 - Article
DB - Scopus
N1 - Export Date: 23 February 2023; Cited By: 32; Correspondence Address: H.J. Cleaves II; Geosciences Research Division, Scripps Institution of Oceanography, La Jolla, CA 92093-0212, 3270 Sverdrup Hall, United States; email: hcleaves@ucsd.edu
ER -

TY - JOUR
AU - German, C.R.
TI - Hydrothermal exploration and astrobiology: Oases for life in distant oceans?
PY - 2004
T2 - International Journal of Astrobiology
VL - 3
IS - 2
SP - 81
EP - 95
DO - 10.1017/S1473550404002009
UR - https://www.scopus.com/inward/record.uri?eid=2-s2.0-71749084749&doi=10.1017%2fS1473550404002009&partnerID=40&md5=d7

fe43baebc0a182b4e7baa8d4e2da74
AD - Southampton Oceanography Centre, European Way, Southampton SO14 3ZH, United Kingdom
AB - High-temperature submarine hydrothermal fields on Earth's mid-ocean ridges play host to exotic ecosystems with fauna previously unknown to science. Because these systems draw significant energy from chemosynthesis rather than photosynthesis, it has been postulated that the study of such systems could have relevance to the origins of life and, hence, astrobiology. A major flaw to that argument, however, is that modern basalt-hosted submarine vents are too oxidizing and lack the abundant free hydrogen required to drive abiotic organic synthesis and/or the energy yielding reactions that the most primitive anaerobic thermophiles isolated from submarine vent-sites apparently require. Here, however, the progress over the past decade in which systematic search strategies have been used to identify previously overlooked venting on the slow-spreading Mid-Atlantic Ridge and the ultra-slow spreading Arctic and SW Indian Ridges is described. Preliminary identification of fault-controlled venting in a number of these sites has led to the discovery of at least two high-temperature hydrothermal fields hosted in ultramafic rocks which emit complex organic molecules in their greater than 360 °C vent-fluids. Whether these concentrations represent de novo organic synthesis within the hydrothermal cell remains open to debate but it is probable that many more such sites exist throughout the Atlantic, Arctic and SW Indian Oceans. One particularly intriguing example is the Gakkel Ridge, which crosses the floor of the Arctic Ocean. On-going collaborations between oceanographers and astrobiologists are actively seeking to develop a new class of free-swimming autonomous underwater vehicle, equipped with appropriate chemical sensors, to conduct long-range missions that will seek out, locate and investigate new sites of hydrothermal venting at the bottom of this, and other, ice-covered oceans. © 2004, Cambridge University Press. All rights reserved.
KW - Earth
KW - Jovian satellites
KW - life:distribution
KW - life:origin
KW - Mars
KW - Panspermia
SN - 14735504 (ISSN)
LA - English
J2 - Int. J. Astrobiology
M3 - Article
DB - Scopus
N1 - Export Date: 23 February 2023; Cited By: 7
ER -

TY - CONF
AU - Konesky, G.A.
TI - In-situ detection of microbial life through respiratory electron analysis
PY - 2004
T2 - Proceedings of SPIE - The International Society for Optical Engineering

VL - 5163
SP - 119
EP - 127
DO - 10.1117/12.502802
UR - https://www.scopus.com/inward/record.uri?eid=2-s2.0-1942454914&doi=10.1117%2f12.502802&partnerID=40&md5=6bd6d1dfc604c80b4565ca0bea4c3d1c
AD - ATH Ventures Inc., Hampton Bays, NY 11946, 3 Rolling Hill Rd., United States
AB - Microbial biofuel cells generate electrical power through the collection of respiratory electrons, which are liberated by the metabolism of nutrients by microorganisms. In the context of in-situ detection of extraterrestrial life, the following question is raised: if microorganisms can be used to generate electrical power, under what circumstances can the generation of electrical power be used to indicate the presence of microbial life? Such an approach to the detection of microorganisms is susceptible to the same ambiguities as similar approaches in that local geochemistry may produce a signal that mimics the presence of life. Consideration is given to time -resolved signal observation to the discrimination of biochemistry and geochemistry and how this approach may be combined with alternate approaches to build a case for, or against, the presence of life. Construction and operation details of microbial biofuel cells, based on marine sediments, are discussed, as are considerations for space flight hardware.
KW - Biochemistry
KW - Electric power distribution
KW - Electrodes
KW - Geochemistry
KW - Ions
KW - Metabolism
KW - Microorganisms
KW - Oxygen
KW - pH effects
KW - Seawater
KW - Sediments
KW - Biochemistry versus geochemistry
KW - In-situ detection
KW - Microbial biofuel cells
KW - Respiratory electrons
KW - Electrons
KW - Biochemistry versus geochemistry
KW - In-situ detection
KW - Microbial biofuel cells
KW - Respiratory electrons
SN - 0277786X (ISSN)
LA - English
J2 - Proc SPIE Int Soc Opt Eng
M3 - Conference paper
DB - Scopus
N1 - Export Date: 23 February 2023; Cited By: 1; Correspondence Address: G.A. Konesky; ATH Ventures Inc., Hampton Bays, NY 11946, 3 Rolling Hill Rd., United States; email: g.konesky@att.net; CODEN: PSISD

ER -

TY - JOUR
AU - Abramov, O.
AU - Kring, D.A.
TI - Numerical modeling of an impact-induced hydrothermal system at the Sudbury crater
PY - 2004
T2 - Journal of Geophysical Research: Planets
VL - 109
IS - 10
SP - E100071
EP - 16
DO - 10.1029/2003JE002213
UR - https://www.scopus.com/inward/record.uri?eid=2-s2.0-10944268677&doi=10.1029%2f2003JE002213&partnerID=40&md5=3b99827c0d4979bcb230334f2f3d09c9
AD - Lunar and Planetary Laboratory, University of Arizona, Tucson, AZ, United States
AB - Large impact events, like the one that formed the Sudbury crater in Ontario, Canada, at 1.85 Ga, significantly increase the temperature of target rocks. The heat sources generated by such an impact event can drive the circulation of groundwater, establishing a hydrothermal system. We report on the results of numerical modeling of postimpact cooling with and without the presence of water at the Sudbury crater. A hydrothermal system is initiated in the annular trough between the peak ring and final crater rim, perhaps venting through faults that bound blocks of the crust in the modification zone of the crater. Although circulation through the overlying breccias may occur in the center of the crater, the central melt sheet is initially impermeable to circulating fluids. By ~ 105 years the central melt sheet crystallizes and partially cools, allowing fluids to flow through it. Host rock permeability is the main factor affecting fluid circulation and lifetimes of hydrothermal systems. High permeabilities lead to a rapid system cooling, while lower permeabilities allow a steady transport of hot fluids to the surface, resulting in high surface temperatures for longer periods of time than cooling by conduction alone. The simulations presented in this paper show that a hydrothermal system at a Sudbury-sized impact crater can remain active for several hundred thousand to several million years, depending on assumed permeability. These results suggest that a hydrothermal system induced by an impact event can remain active for sufficiently long periods of time to be biologically significant, supporting the idea that impact events may have played an important biological role, especially early in Earth's history. Copyright 2004 by the American Geophysical Union.
KW - Canada
KW - North America
KW - Ontario
KW - Sudbury Structure
KW - Western Hemisphere
KW - World
KW - cooling

KW - early Earth
KW - hydrothermal system
KW - impact structure
KW - numerical model
KW - Astrobiology
KW - Crater cooling
KW - Hydrothermal systems
KW - Numerical modeling
KW - Sudbury, impact cratering
PB - American Geophysical Union
SN - 01480227 (ISSN)
LA - English
J2 - J. Geophys. Res. E Planets
M3 - Article
DB - Scopus
N1 - Export Date: 23 February 2023; Cited By: 75; Correspondence Address: O. Abramov; Lunar and Planetary Laboratory, University of Arizona, Tucson, AZ 85721-0092, 1629 East University Blvd., United States; email: abramovo@lpl.arizona.edu
ER -

TY - JOUR
AU - Tang, G.-J.
AU - Chen, Y.-J.
TI - Comment of tracing environmental change with organic carbon isotopes
PY - 2004
T2 - Kuangwu Yanshi
VL - 24
IS - 3
SP - 110
EP - 115
UR - https://www.scopus.com/inward/record.uri?eid=2-s2.0-10644276038&partnerID=40&md5=29f5a76c0db71d3c4e727a4c2f7122c4
AD - School of Earth and Space, Peking University, Beijing 100871, China
AB - As global change becomes a more important research topic, scientists tried different ways on various geologic records in their research. This paper introduces and reviews the organic carbon isotope method used to trace environmental change, including its mechanism, research objects, obtained results and problems. It is considered that 12C prefers to enrich in C-H bond rather than in C-O bond, causing much lower negative δ13C for biotic matter or organic carbon than that for inorganic carbon. Difference in photosynthesis distinguishes plants into three groups with distinctive δ 13C, i. e. C3, C4, and CAM. The C3, plants have δ13C=-20‰- -32‰ , average -28‰ and the C4, plants have 13C= -9‰- -l7‰, average -14‰. Climate affects the style and strength of photosynthesis, causing distinctive development of plant group in unique environment with distinctive total organic δ 13C value. The difference in δ13C of total plants can further result in local characteristic δ 13C of animal fossils on the biological chain, such as fossil teeth, and even characteristic inorganic δ13C. Therefore, carbon-isotopic study of biotic chains and their ramifications or products is an effective

way to understand local or temporal environments. Accordingly, the geologic records are divided into two large classes, i. e. organic and inorganic records. Organic records include fossil plants, such as tree rings, fossil animals, such as teeth, continental sedimentary organic matter, such as coal, marine sedimentary organic matter and soils (ramification of biotic matter). These research records have been used successfully to trace environmental changes of various aims. The authors believe that organic carbon isotopic study will play a more important role in revealing significant mass extinction, finding oldest C4 plants, understanding origin of earliest life on earth, verifying the existence of extraterrestrial life and determining the relation between organic matter and mineral resources. More attention will be paid to the detail of fractionation of carbon isotopes during carbon cycling. Obviously, complexity of these problems needs the inter-discipline collaboration.
KW - Carbon
KW - Geology
KW - Mineral resources
KW - Photosynthesis
KW - carbon isotope
KW - global change
KW - isotopic fractionation
KW - organic carbon
KW - paleoenvironment
KW - Carbon isotopes
KW - Organic carbon
KW - Environmental impact
KW - Carbon isotope
KW - Comment
KW - Environment
KW - Fractionation
KW - Organic carbon
SN - 10016872 (ISSN)
LA - Chinese
J2 - Kuangwu Yanshi
M3 - Article
DB - Scopus
N1 - Export Date: 23 February 2023; Cited By: 3
ER -

TY - JOUR
AU - de Vera, J.-P.
AU - Horneck, G.
AU - Rettberg, P.
AU - Ott, S.
TI - The potential of the lichen symbiosis to cope with the extreme conditions of outer space II: Germination capacity of lichen ascospores in response to simulated space conditions
PY - 2004
T2 - Advances in Space Research
VL - 33
IS - 8
SP - 1236

EP - 1243
DO - 10.1016/j.asr.2003.10.035
UR - https://www.scopus.com/inward/record.uri?eid=2-s2.0-2342649468&doi=10.1016%2fj.asr.2003.10.035&partnerID=40&md5=26d76ac14ea6685db316a1752ee4c497
AD - Botanisches Institut, Heinrich-Heine-Universität, Düsseldorf D-40225, Universitätsstr.1, Germany
AD - DLR German Aerospace Center, Institute of Aerospace Medicine, Radiation Biology, Köln D-51170, Germany
AB - Complementary to the already well-studied microorganisms, lichens, symbiotic organisms of the mycobiont (fungi) and the photobiont (algae), were used as "model systems" in which to examine the ecological potential to resist to extreme environments of outer space. Ascospores (sexual propagules of the mycobiont) of the lichens Fulgensia bracteata, Xanthoria elegans and Xanthoria parietina were exposed to selected space-simulating conditions (up to 16 h of space vacuum at 10^{-3} Pa and UV radiation at 160 nm $\leq\lambda\leq$400 nm), while embedded in the lichen fruiting bodies. After exposure, the ascospores were discharged and their viability was tested as germination capacity on different culture media including those containing Mars regolith simulant. It was found that (i) the germination rate on media containing Mars regolith simulant was as high as on other mineral-containing media, (ii) if enclosed in the ascocarps, the ascospores survived the vacuum exposure, the UV-irradiation as well as the combined treatment of vacuum and UV to a high degree. In general, 50 % or more viable spores were recovered, with ascospores of X. elegans showing the highest survival. It is suggested that ascospores inside the ascocarps are well protected by the anatomical structure, the gelatinous layer and the pigments (parietin and carotene) against the space parameters tested. © 2004 COSPAR. Published by Elsevier Ltd. All rights reserved.
KW - Cell culture
KW - Ecology
KW - Fungi
KW - Microorganisms
KW - Pigments
KW - Ultraviolet radiation
KW - Gelatinous layers
KW - Germination capacities
KW - Symbiosis
KW - exobiology
KW - lichen
KW - Space research
KW - Ascospores
KW - Astrobiology
KW - Germination capacity
KW - Lichen symbiosis
KW - Outer space conditions
KW - Viability
PB - Elsevier Ltd
SN - 02731177 (ISSN)
C2 - 15806704
LA - English

J2 - Adv. Space Res.
M3 - Article
DB - Scopus
N1 - Export Date: 23 February 2023; Cited By: 65; Correspondence Address: J.-P. de Vera; Botanisches Institut, Heinrich-Heine-Universität, Düsseldorf D-40225, Universitätsstr.1, Germany; email: devera@uni-duesseldorf.de; CODEN: ASRSD
ER -

TY - CONF
AU - Digregorio, B.E.
TI - A planetary quarantine laboratory on the moon
PY - 2004
T2 - Proceedings of SPIE - The International Society for Optical Engineering
VL - 5555
C7 - 16
SP - 153
EP - 161
DO - 10.1117/12.563700
UR - https://www.scopus.com/inward/record.uri?eid=2-s2.0-14944345779&doi=10.1117%2f12.563700&partnerID=40&md5=7a9838874fb49ef3071a303810b837d9
AD - Cardiff Centre for Astrobiology, Wales UK c/o, Middleport, NY 14105, 16 North Hartland Street, United States
AB - NASA is planning to establish a human tended Planetary Quarantine Laboratory as part of a scientific outpost on the Moon by 2015-2020. Recent Mars Sample Return (MSR) scenarios have focused on a direct return to the surface of the Earth by means of a passive reentry capsule similar to the Stardust sample capsule designed to use atmospheric friction and ablating to slow its decent instead of a parachute. This scenario offers less planetary protection than LEO examination by a specially trained scientific crew aboard the ISS or space shuttle. The laboratory would offer 100% protection of Earth's biosphere against any toxic or pathogenic bioactive materials from Mars or any other solar system samples returned.
KW - Astronomy
KW - Contamination
KW - Martian surface analysis
KW - Microbiology
KW - Moon bases
KW - Planets
KW - Space research
KW - Space shuttles
KW - Astrobiology
KW - Back contamination
KW - Forward contamination
KW - Life on Mars
KW - Mars sample quarantine facility
KW - Mars Sample Return (MSR)
KW - Orbiting quarantine facility
KW - Planetary protection
KW - Research laboratories
KW - Astrobiology

KW - Back contamination
KW - Forward contamination
KW - Life on Mars
KW - Mars sample quarantine facility
KW - Mars Sample Return
KW - MSR
KW - Orbiting quarantine facility
KW - Planetary protection
SN - 0277786X (ISSN)
LA - English
J2 - Proc SPIE Int Soc Opt Eng
M3 - Conference paper
DB - Scopus
N1 - Export Date: 23 February 2023; Cited By: 0; Correspondence Address: B.E. Digregorio; Cardiff Centre for Astrobiology, Wales UK c/o, Middleport, NY 14105, 16 North Hartland Street, United States; email: Icamsr@buffnet.net; CODEN: PSISD
ER -

TY - JOUR
AU - Johnson, T.V.
TI - A Look at the Galilean Satellites After the Galileo Mission
PY - 2004
T2 - Physics Today
VL - 57
IS - 4
SP - 77
EP - 83
DO - 10.1063/1.1752426
UR - https://www.scopus.com/inward/record.uri?eid=2-s2.0-2042534715&doi=10.1063%2f1.1752426&partnerID=40&md5=00d2405f5daf5a3c7e8b885f8d7b6d0d
AD - California Institute of Technology, Jet Propulsion Laboratory, Pasadena, United States
AB - The findings of the Galileo mission are discussed. Galileo repeatedly made precise observations of the Galilean satellites from unpreedented ranges. Galileo's encounter with Ganymede confirmed the spacecraft's ability to fly by at the moons at altitudes of a few hundred kilometers. One of the most exciting results from the Galileo mission is the evidence it gathered for global liquid-water oceans beneath the icy crusts of Europa, Ganymede, and Callisto. The probable evidence of liquid-water oceans, the presence of carbon-rich material, and the availability of energy in the form of radiogenic and tidal heat, sunlight, and particulate radiation have combined to increase the astrobiological interest in the icy Galilean satellites.
KW - Astrophysics
KW - Extraterrestrial atmospheres
KW - Gravitation
KW - Heat conduction
KW - Heat convection
KW - Ice
KW - Magnetic fields
KW - Magnetosphere

KW - Mathematical models
KW - Planets
KW - Radioisotopes
KW - Spacecraft
KW - Thermodynamics
KW - Water
KW - Dipolar magnetic fields
KW - Gravitational fields
KW - Liquid-water oceans
KW - Radiogenic heat sources
KW - Space research
PB - American Institute of Physics Inc.
SN - 00319228 (ISSN)
LA - English
J2 - Phys Today
M3 - Review
DB - Scopus
N1 - Export Date: 23 February 2023; Cited By: 5; CODEN: PHTOA
ER -

TY - CONF
TI - Proceedings of SPIE: Instruments, Methods, and Missions for Astrobiology VIII
PY - 2004
T2 - Proceedings of SPIE - The International Society for Optical Engineering
VL - 5555
UR - https://www.scopus.com/inward/record.uri?eid=2-s2.0-14944375091&partnerID=40&md5=97d8fa722afd238664482a6f1a8567a5
AB - The proceedings contain 28 papers from the Proceedings of SPIE: Instruments, Methods, and Missions for Astrobiology VIII. The topics discussed include: indigenous microfossils in carbonaceous meteorites; sample size/response relationship as a means of discriminating biotic and abiotic signatures; microbial remains in middle proterozoic rocks of northern Australia; complexes of ribose with silicates, borates, and calcium: implications to astrobiology; astrobiology of comets; testing the role of silicic acid and bio-organic materials in the formation of rock coatings; a planetary quarantine laboratory on the moon; and salt-tolerant and high-pH-resistant hydrogenase from the haloalkaliphilic sulfate-reducing bacterium desulfonatronum thiodismutans.
KW - Astronomy
KW - Bacteria
KW - Biochemistry
KW - Earth (planet)
KW - Energy dispersive spectroscopy
KW - Energy transfer
KW - Fossil fuels
KW - Geochemistry
KW - Moon
KW - Neural networks
KW - Oceanography
KW - Planets
KW - Principal component analysis

KW - Probability
KW - Scanning electron microscopy
KW - Sugars
KW - Astrobiology
KW - Carbonaceous meteorites
KW - Complexity analysis
KW - Cyanobacteria
KW - EiRev
KW - Hierarchical analysis
KW - Mars
KW - Microcoleus orgueil
KW - Microfosils
KW - Paleo-biosignatures
KW - Meteorites
SN - 0277786X (ISSN)
LA - English
J2 - Proc SPIE Int Soc Opt Eng
M3 - Conference review
DB - Scopus
N1 - Export Date: 23 February 2023; Cited By: 0; CODEN: PSISD
ER -

TY - CONF
AU - Rodríguez, N.
AU - Amils, R.
AU - Sanz, J.L.
TI - Methanogenesis in the extreme acidic conditions of the Tinto River
PY - 2004
T2 - European Space Agency, (Special Publication) ESA SP
IS - 545
SP - 279
EP - 280
UR - https://www.scopus.com/inward/record.uri?eid=2-s2.0-2342614862&partnerID=40&md5=eeade0831ad4aa35ffef08549ca2745c
AD - Centro de Astrobiología, INTA-CSIC, Torrejon de Ardoz. 28850 Madrid, Carretera de Torrejon-Ajalvir Km 4, Spain
AD - Centro de Biología Molecular, UAM-CSIC, U. Autónoma de Madrid, Cantoblanco, 28049 Madrid, Spain
AB - An important subject of astrobiological interest is the study of microbial activities that can operate in the extreme conditions found in different planetary bodies. Of those, methanogenic activity is considered an interesting metabolism due to the nature of the substrates (hydrogen, formic acid, methanol, etc.) and the properties of methane, the metabolic product. Due to the peculiarities of the enzymes involved in methane production and the ecology of the microorganisms responsible of the process it is considered a relic of a primitive carbon cycle capable to operate in the subsurface in the absence of radiation (Stevens and McKinle, 1995, Pedersen, 2000), Gómez and Amils, 2002, Amils et al, 2004). Here we report the discovery of methanogenic activity associated to the anoxic sediments of The Tinto River Basin (Iberian Pyritic Belt), an extreme acidic environment product of the chemolithotrophic activity of iron- and sulfuroxidizing

microorganism. This finding is of astrobiological interest, specially in the context of life exploration on Mars (Stocker et al., 2003).
KW - Acidity
KW - Carbon
KW - Catchments
KW - Metabolism
KW - Methane
KW - Redox reactions
KW - Rivers
KW - Anaerobic conditions
KW - Anaerobic reactors
KW - Methanogenic activity
KW - Redox potentials
KW - Planets
SN - 03796566 (ISSN)
LA - English
J2 - Eur Space Agency Spec Publ ESA SP
M3 - Conference paper
DB - Scopus
N1 - Export Date: 23 February 2023; Cited By: 2; CODEN: ESPUD
ER -

TY - CONF
AU - Harper, L.D.
AU - Schmidt, G.K.
AU - Schaefer, R.L.
AU - Rudolph-Angelich, I.L.
AU - Mains, R.
AU - Hughes, D.
AU - Leonard, G.
TI - Biology beyond the planet of origin: The case for biosciences research on the moon
PY - 2004
T2 - SAE Technical Papers
DO - 10.4271/2004-01-2282
UR - https://www.scopus.com/inward/record.uri?eid=2-s2.0-85072436528&doi=10.4271%2f2004-01-2282&partnerID=40&md5=e63f3af9b75bc9d97122edd737297fbf
AD - National Aeronautics and Space Administration, NASA-Ames Research Center, Moffett Field, CA 94035-1000, United States
AD - Lockheed Martin Space Operations, Ames Research Center, United States
AD - Mains Associates, United States
AB - Studying the biology of terrestrial life on another world will offer unique opportunities for understanding the fundamental nature of life in the universe. Still accelerating revolutions in biotechnology, information technology, robotics, and super-miniaturization now make it possible to conduct detailed research into opportunities inherent in living on other worlds. Although close to Earth, the Moon includes features found nowhere else in the solar system. Different gravity, radiation stresses, magnetic fields and day/night cycles are among the biologically relevant forces of interest on the Moon. Results from studying different organisms in

the lunar environment over complete life cycles and multiple generations would provide the first comparative, biological reference data of the transition of life from one world to another, and foundational information for evaluating potential health and safety problems on a Mars mission. In addition, astrobiology studies on the Moon can reveal the solar system forces that shaped the evolution of life on Earth. The Moon has also long been acknowledged as a testbed for human explorations of Mars and other worlds of the solar system. Finally, the scientific, technological and architectural demands of a dedicated lunar biosciences research initiative will drive numerous commercial, social, and educational benefits. Copyright © 2004 SAE International.
KW - Biology
KW - Digital storage
KW - Earth (planet)
KW - Martian surface analysis
KW - Educational benefits
KW - Human exploration
KW - Mars missions
KW - Over-complete
KW - Potential health
KW - Radiation stress
KW - Reference data
KW - Research initiatives
KW - Moon
PB - SAE International
SN - 01487191 (ISSN)
LA - English
J2 - SAE Techni. Paper.
M3 - Conference paper
DB - Scopus
N1 - Export Date: 23 February 2023; Cited By: 0; Correspondence Address: L.D. Harper; National Aeronautics and Space Administration, NASA-Ames Research Center, Moffett Field, CA 94035-1000, United States; email: lharper@mail.arc.nasa.gov
ER -

TY - CONF
TI - Proceedings of SPIE: Instruments, Methods and Mission for Astrobiology VII
PY - 2004
T2 - Proceedings of SPIE - The International Society for Optical Engineering
VL - 5163
UR - https://www.scopus.com/inward/record.uri?eid=2-s2.0-1942423217&partnerID=40&md5=a9550c83165ca500f83abcddc95029e1
AB - The proceedings contains 21 papers of SPIE: Instruments, Methods, and Mission for Astrobiology VII. The topics discussed include; microfossils in Cl and CO carbonaceous meteorites; atlas of bacteriomorphs in carbonaceous chondrites; framboidal structures in earth rocks and in astromaterials; intrinsic selectivity in some prebiotic reactions of urazole with sugars; preliminary identification of fullerenes in the lowermost Jurassic data, Queen Charlotte Islands, British Columbia and CONTOUR forward imager on

the comet nucleus tour mission.
KW - Amino acids
KW - Bacteria
KW - Biomarkers
KW - Carbonaceous adsorbents
KW - DNA
KW - Environmental impact
KW - Geochemistry
KW - Lakes
KW - Mass spectrometry
KW - Permafrost
KW - Precipitation (chemical)
KW - Remote sensing
KW - Rocks
KW - Sediments
KW - Stratigraphy
KW - Water
KW - Bacterial remains
KW - Bacteriomorphs
KW - Carbonaceous meteorites
KW - Comet
KW - EiRev
KW - Impact events
KW - Jurassic
KW - Microfossils
KW - Space
KW - Triassic/Jurassic boundary
KW - Meteorites
SN - 0277786X (ISSN)
LA - English
J2 - Proc SPIE Int Soc Opt Eng
M3 - Conference review
DB - Scopus
N1 - Export Date: 23 February 2023; Cited By: 0; CODEN: PSISD
ER -

TY - JOUR
AU - Tarter, J.C.
TI - Astrobiology and SETI
PY - 2004
T2 - New Astronomy Reviews
VL - 48
IS - 11-12
SP - 1543
EP - 1549
DO - 10.1016/j.newar.2004.09.019
UR - https://www.scopus.com/inward/record.uri?eid=2-s2.0-10644265867&doi=10.1016%2fj.newar.2004.09.019&partnerID=40&md5=9a998d5c5506972cf68e58d8bca7410c
AD - The SETI Institute, Mountain View, CA 94043, 515 N. Whisman Road, United States
AB - "Are we alone?" "Where did we come from?" These are the defining questions of the new field of Astrobiology, and they are also the questions that have tantalized humanity throughout recorded

history. The SKA will afford an excellent opportunity to answer these old questions and thereby calibrate our place in the cosmos. With the SKA we may discover another technological species and chart the pathways by which complex organic molecules assembled in interstellar space were incorporated into, or reassembled within, nascent planetary systems and the role these molecules play in creating habitable worlds. © 2004 Elsevier B.V. All rights reserved.
PB - Elsevier
SN - 13876473 (ISSN)
LA - English
J2 - New Astron. Rev.
M3 - Short survey
DB - Scopus
N1 - Export Date: 23 February 2023; Cited By: 17; Correspondence Address: J.C. Tarter; The SETI Institute, Mountain View, CA 94043, 515 N. Whisman Road, United States; email: tarter@vger.seti.org; CODEN: NAREF
ER -

TY - CONF
AU - Richter, L.
AU - Gromov, V.V.
AU - Tokano, T.
AU - Sims, M.R.
AU - Kossacki, K.J.
TI - Planetary underground tool (PLUTO) for future astrobiological missions to Mars
PY - 2004
T2 - European Space Agency, (Special Publication) ESA SP
IS - 545
SP - 273
EP - 274
UR - https://www.scopus.com/inward/record.uri?eid=2-s2.0-2342616848&partnerID=40&md5=9fde807b89b75c76c4ec6c6c18b3f7ff
AD - DLR, Institut für Raumsimulation, 51170 Köln, Germany
AD - VNIITransmash, St. Petersburg 198323, 2 Zarechnaja St., Russian Federation
AD - Space Research Centre, University of Leicester, Leicester LE1 7RH, University Road, United Kingdom
AD - Institute of Geophysics, Warsaw University, 02-093 Warszawa, Pasteura 7, Poland
AB - While Viking did not find any certain evidence of life or organic material, there is good reason to believe that at depths of a few metres organic material exists whose external or biological origin could be investigated by analyses of in situ soil samples. A prerequisite for such experiments is the equipment of the lander/ rover with a device aimed at taking samples from various depths of subsurface. The prototype of such a device is the Planetary underground Tool (PLUTO) which is part of the payload of the ongoing exobiological Beagle 2 lander of ESA's Mars Express mission. PLUTO is a regolith-penetrating, tethered "Mole" intended for acquisition of several subsurface soil samples from depths between about 10 cm and approximately 1.5 m. PLUTO onboard Beagle 2 is equipped with a sampling device and a temperature sensor, and the analysis of carbon

isotope ratio and organic molecules will be performed on the surface of Mars by the Gas Analysis Package (GAP). In future missions some instruments relevant for astrobiology could also be accommodated directly within the PLUTO such as a compact detector head on a fibre-optic cable for underground deployment to search for biota of biogenic materials whilst concurrently characterising the mineral habitat.
KW - Biomarkers
KW - Drilling
KW - Minerals
KW - Moisture
KW - Mossbauer spectroscopy
KW - Oxidation
KW - Sensors
KW - Soils
KW - Space research
KW - Spectrometers
KW - Temperature measurement
KW - Astrobiology
KW - Biogenic material
KW - Gas analysis package (GAP)
KW - Planetary underground tool (PLUTO)
KW - Martian surface analysis
SN - 03796566 (ISSN)
LA - English
J2 - Eur Space Agency Spec Publ ESA SP
M3 - Conference paper
DB - Scopus
N1 - Export Date: 23 February 2023; Cited By: 1; Correspondence Address: L. Richter; DLR, Institut für Raumsimulation, 51170 Köln, Germany; email: lutz.richter@dlr.de; CODEN: ESPUD
ER -

TY - CONF
AU - Fernández-Remolar, D.
AU - Gómez-Elvira, J.
AU - Gómez, F.
AU - Sebastian, E.
AU - Martíin, J.
AU - Manfredi, J.A.
AU - Torres, J.
AU - González Kesler, C.
AU - Amils, R.
TI - The Tinto River, an extreme acidic environment under control of iron, as an analog of the Terra Meridiani hematite site of Mars
PY - 2004
T2 - Planetary and Space Science
VL - 52
IS - 1-3
SP - 239
EP - 248
DO - 10.1016/j.pss.2003.08.027
UR - https://www.scopus.com/inward/record.uri?eid=2-s2.0-0346500672&doi=10.1016%2fj.pss.2003.08.027&partnerID=40&md5=4fd

144fe3ccd7fec82c430f3951e4f1b
AD - Ctro. de Astrobiol. (CSIC-INTA), Torrejon de Ardoz, 28850 Madrid, Crtra. de Torrejon a Ajalvir, km 4.5, Spain
AD - Centro de Biologia Molecular (CBM), Universidad Autonoma de Madrid, Spain
AB - One remarkable discovery in Mars geology is the extensive Meridiani iron formation. Although thermal oxidation mechanisms have been proposed for its origin, geomorphology and IR spectroscopy studies support an aqueous scenario as the most feasible environment to host its secondary iron-enriched mineralogy. Terrestrial hydrothermal systems or the iron-rich Precambrian oceans have been proposed as analogs to understand these interesting Martian environments. However, the available TES spectra show hematite as a mineral phase of Mars' iron rocks, but not the cherty silica that frequently appears in hydrothermal systems or BIFs. The Tinto River Basin, an extreme acidic environment driven by iron biogeochemistry, has water rich in both ferric iron and sulfate. These acidic waters produce ferric iron-enriched sediments dominated by sulfate and oxyhydroxide associations, in which silicates are absent. Moreover, ancient iron formations, pre-Holocene relicts of similar acidic water environments found in the Tinto River Basin, suggest that the primary iron-rich associations changed with time to goethite-hematite associations through dehydration and desulfatation. These features make the Tinto River Basin a reasonable analog for the Mars hematite site if an aqueous origin for these oxides is considered. © 2003 Elsevier Ltd. All rights reserved.
KW - Dehydration
KW - Geochemistry
KW - Geomorphology
KW - Infrared spectroscopy
KW - Iron
KW - Mineralogy
KW - Rocks
KW - Sediments
KW - Silica
KW - Thermooxidation
KW - Astrobiology
KW - Hematite
KW - Hydrochemistry
KW - Iron mineral formations
KW - Mars
KW - Martian surface analysis
KW - Astrobiology
KW - Hematite
KW - Hydrochemistry
KW - Iron mineral formations
KW - Mars
SN - 00320633 (ISSN)
LA - English
J2 - Planet. Space Sci.
M3 - Conference paper
DB - Scopus
N1 - Export Date: 23 February 2023; Cited By: 101; Correspondence Address: D. Fernández-Remolar; Ctro. de Astrobiol. (CSIC-INTA),

Torrejon de Ardoz, 28850 Madrid, Crtra. de Torrejon a Ajalvir, km 4.5, Spain; email: fernandezrd@inta.es; CODEN: PLSSA
ER -

TY - CONF
AU - Tokano, T.
TI - Precession-driven migration of water in the surficial layers of Mars
PY - 2004
T2 - European Space Agency, (Special Publication) ESA SP
IS - 545
SP - 301
EP - 302
UR - https://www.scopus.com/inward/record.uri?eid=2-s2.0-2342458380&partnerID=40&md5=a4462dd458068a654a462b0b9a9e3f57
AD - DLR, Institut für Raumsimulation, D-51170 Köln, Germany
AB - A linear correlation analysis between the Mars Odyssey neutron fluxes and various surface parameters indicates that the annual maximum surface temperature is the most important factor controlling the soil water content in the surficial layer of the Martian soil. This is likely to be associated with the higher enthalpy of mineral hydration in comparison with the sublimation enthalpy of ice, which is presumably almost absent near the surface. While presently the maximum surface temperature occurs near 30°S because of perihelion in late southern spring, the season of perihelion periodically migrates by virtue of precession. Consequently, the maximum surface temperature as well as the driest place on Mars should move from one hemisphere to the other with a period of about 51000 years. A significant amount of adsorbed water would then be exchanged between the hemispheres and between the soil and other reservoirs, especially with the polar caps and the polar layered deposits. Enhanced soil water content near the surface is always accompanied with a reduced peak ultraviolet flux, both reducing the chemical reactivity of the soil. In the present epoch the northern hemisphere may thus represent astrobiologically more clement conditions.
KW - Astrophysics
KW - Correlation methods
KW - Dehydration
KW - Neutrons
KW - Reservoirs (water)
KW - Soil mechanics
KW - Spectrometers
KW - Sublimation
KW - Surfaces
KW - Thermodynamic stability
KW - Martian soil
KW - Mineral hydration
KW - Neutron fluxes
KW - Soil water content
KW - Martian surface analysis
SN - 03796566 (ISSN)
LA - English
J2 - Eur Space Agency Spec Publ ESA SP
M3 - Conference paper

DB - Scopus
N1 - Export Date: 23 February 2023; Cited By: 0; Correspondence Address: T. Tokano; DLR, Institut für Raumsimulation, D-51170 Köln, Germany; email: tetsuya.tokano@dlr.de; CODEN: ESPUD
ER -

TY - JOUR
AU - Crovisier, J.
AU - Bockelée-Morvan, D.
AU - Biver, N.
AU - Colom, P.
AU - Despois, D.
AU - Lis, D.C.
TI - Ethylene glycol in comet C/1995 O1 (Hale-Bopp)
PY - 2004
T2 - Astronomy and Astrophysics
VL - 418
IS - 3
SP - L35
EP - L38
DO - 10.1051/0004-6361:20040116
UR - https://www.scopus.com/inward/record.uri?eid=2-s2.0-2342628588&doi=10.1051%2f0004-6361%3a20040116&partnerID=40&md5=cab83fb66fd66ce528eecac0483a8599
AD - Observatoire de Paris, 92195 Meudon, France
AD - Observatoire de Bordeaux, 33270 Floirac, BP 89, France
AD - California Institute of Technology, MS 320-47, Pasadena, CA 91125, United States
AB - We report the detection of ethylene glycol (HOCH2CH 2OH) in comet C/1995 O1 (Hale-Bopp) from the analysis of archival radio spectra. Its production rate is ≈0.25% that of water, making it one of the most abundant organic molecules in cometary ices. This detection strengthens the similarity between interstellar and cometary material.
KW - Aldehydes
KW - Electron energy levels
KW - Ethylene
KW - Glycols
KW - Hydrogenation
KW - Interferometers
KW - Mathematical models
KW - Regression analysis
KW - Solar system
KW - Spectrum analysis
KW - Sublimation
KW - Astrobiology
KW - Astrochemistry
KW - Comets: indiduals:C/1995 (Hale-Bopp)
KW - Comets:general
KW - Radio lines: solar system
KW - Solar system: formation
KW - Astronomy
KW - Astrobiology
KW - Astrochemistry

KW - Comets: general
KW - Comets: individuals: C/1995 O1 (Hale-Bopp) radio lines: solar system
KW - Solar system: formation
SN - 00046361 (ISSN)
LA - English
J2 - Astron. Astrophys.
M3 - Article
DB - Scopus
N1 - Export Date: 23 February 2023; Cited By: 90; Correspondence Address: J. Crovisier; Observatoire de Paris, 92195 Meudon, France; email: jacques.crovisier@obspm.fr; CODEN: AAEJA
ER -

TY - JOUR
AU - Shapiro, R.S.
TI - Recognition of fossil prokaryotes in Cretaceous methane seep carbonates: Relevance to astrobiology
PY - 2004
T2 - Astrobiology
VL - 4
IS - 4
SP - 438
EP - 449
DO - 10.1089/ast.2004.4.438
UR - https://www.scopus.com/inward/record.uri?eid=2-s2.0-12144281185&doi=10.1089%2fast.2004.4.438&partnerID=40&md5=2e909136d76faffd0e07c8f608e174e4
AD - Department of Geosciences, University of Nevada, Las Vegas, NV, United States
AD - Department of Geology, Gustavus Adolphus College, St. Peter, MN 56082, United States
AB - Recovery of prokaryotic body fossils from methane seep carbonates such as those of the Cretaceous Tepee Buttes of Colorado serves as a model for sampling in future astrobiological missions. The fossils, found primarily at the interface between paragenetic fabrics, suggest a sharp physicochemical gradient. Evidence of these microbial fossils occurs at a variety of scales. In the field, microbialite is found as meter-scale thrombolitic zones and centimeter-scale stromatolitic crusts lining voids inferred to be the sites of ancient methane seepage. Petrographic fabrics suggestive of microbialite include indistinct peloids (0.1–1 mm in diameter) and crusts of authigenic micrite. Primary evidence obtained from scanning electron microscopy coupled with energy-dispersive x-ray spectroscopy analysis comprises pinnate bacteria (0.3 μm in diameter and 1–1.5 μm long), sheaths (2–4 μm in diameter), coccoids (0.5–1 μm in diameter, up to 40 per cluster), and the presence of framboidal pyrite (6–8 μm in diameter). These results are in agreement with studies of other ancient and modern seeps and suggest a morphological conservatism of microbial form that can be incorporated into studies of extraterrestrial environments where it is presumed that reduced gases drive the metabolic activity of prokaryote-like organisms. Target areas that could serve as conduits for reduced gas seeps include tectonic or

impact-driven faulting, zones of cryosphere melting, or other disruptions in crustal coherence. Ancient seeps, preserved as localized anomalous evaporite deposits in the sedimentary cover, could be detected by remote sensing. © Mary Ann Liebert, Inc.
KW - Archaea
KW - Bacteria
KW - Carbonates
KW - Cold-seep
KW - Microbialite
PB - Mary Ann Liebert Inc.
SN - 15311074 (ISSN)
C2 - 15684725
LA - English
J2 - Astrobiology
M3 - Article
DB - Scopus
N1 - Export Date: 23 February 2023; Cited By: 24; Correspondence Address: R.S. Shapiro; Department of Geology, Gustavus Adolphus College, St Peter, MN 56082, United States; email: rshapiro@gustavus.edu
ER -

TY - JOUR
AU - Benner, S.A.
TI - Understanding nucleic acids using synthetic chemistry
PY - 2004
T2 - Accounts of Chemical Research
VL - 37
IS - 10
SP - 784
EP - 797
DO - 10.1021/ar040004z
UR - https://www.scopus.com/inward/record.uri?eid=2-s2.0-6944225455&doi=10.1021%2far040004z&partnerID=40&md5=ca0dab2103eb218341937160f54f938a
AD - Department of Chemistry, University of Florida, Gainesville, FL 32611-7200, United States
AD - Yale and Harvard Universities, United States
AD - ETH Zurich, Switzerland
AD - University of Florida, United States
AB - This Account describes work done in these laboratories that has used synthetic, physical organic, and biological chemistry to understand the roles played by the nucleobases, sugars, and phosphates of DNA in the molecular recognition processes central to genetics. The number of nucleobases has been increased from 4 to 12, generating an artificially expanded genetic information system. This system is used today in the clinic to monitor the levels of HIV and hepatitis C viruses in patients, helping to manage patient care. Work with uncharged phosphate replacements suggests that a repeating charge is a universal feature of genetic molecules operating in water and will be found in extraterrestrial life (if it is ever encountered). The use of ribose may reflect prebiotic processes in the presence of borate-containing minerals, which stabilize ribose formed from simple organic precursors. A new field, synthetic

biology, is emerging on the basis of these experiments, where chemistry mimics biological processes as complicated as Darwinian evolution.
KW - Base Sequence
KW - DNA
KW - Genes, Synthetic
KW - Molecular Structure
KW - Nucleic Acid Conformation
KW - Nucleic Acids
KW - Ribose
KW - boric acid
KW - DNA
KW - mineral
KW - nucleic acid
KW - nucleic acid base
KW - phosphate
KW - prebiotic agent
KW - ribose
KW - sugar
KW - water
KW - article
KW - biology
KW - chemistry
KW - DNA strand
KW - evolution
KW - Hepatitis C virus
KW - human
KW - Human immunodeficiency virus
KW - laboratory
KW - molecular biology
KW - molecular genetics
KW - molecular recognition
KW - molecule
KW - nonhuman
KW - patient care
KW - polymerase chain reaction
KW - substitution therapy
SN - 00014842 (ISSN)
C2 - 15491125
LA - English
J2 - Acc. Chem. Res.
M3 - Article
DB - Scopus
N1 - Export Date: 23 February 2023; Cited By: 309; Correspondence Address: S.A. Benner; Department of Chemistry, University of Florida, Gainesville, FL 32611-7200, United States; email: benner@chem.ufl.edu; CODEN: ACHRE
ER -

TY - CONF
AU - Brinckerhoff, W.B.
TI - Pulsed laser ablation TOF-MS analysis of planets and small bodies
PY - 2004

T2 - Applied Physics A: Materials Science and Processing
VL - 79
IS - 4-6
SP - 953
EP - 956
DO - 10.1007/s00339-004-2581-6
UR - https://www.scopus.com/inward/record.uri?eid=2-s2.0-4344580256&doi=10.1007%2fs00339-004-2581-6&partnerID=40&md5=1b40080093362a7f6e1ddc90a11b18cf
AD - Applied Physics Laboratory, Johns Hopkins University, Laurel, MD 20723, United States
AB - We are developing miniature time-of-flight mass spectrometers, based on nanosecond pulsed Nd:YAG IR and UV laser ablation, for robotic landed missions to Mars, asteroids, comets, and planetary moons. The goal is to obtain grain-scale and bulk major, minor, and trace composition (both atomic and molecular) from solids, with minimal sample manipulation and preparation. We present here a summary of this effort, focusing on the variety of analyses that could be enabled by pulsed laser ablation for sampling and ionization. We show that a number of geochemical and astrobiological objectives on small bodies or Mars could be addressed with highly simplified mass-spectrometer instrumentation, provided a thorough analysis of the yields of elements and organics to the laser-ablation system is conducted.
KW - Geochemistry
KW - Kinetic energy
KW - Mass spectrometers
KW - Meteorites
KW - Moon
KW - Planets
KW - Spacecraft
KW - Telescopes
KW - Vacuum pumps
KW - Laser ablation mass spectrometers (LAMS)
KW - Mars
KW - Pulsed laser ablation
KW - Time of flight (TOF) mass spectrometry
KW - Laser ablation
PB - Springer Verlag
SN - 09478396 (ISSN)
LA - English
J2 - Appl Phys A
M3 - Conference paper
DB - Scopus
N1 - Export Date: 23 February 2023; Cited By: 12; Correspondence Address: W.B. Brinckerhoff; Applied Physics Laboratory, Johns Hopkins University, Laurel, MD 20723, United States; email: william.brinckerhoff@jhuapl.edu; CODEN: APAMF
ER -

TY - JOUR
AU - Mancinelli, R.L.
AU - Fahlen, T.F.
AU - Landheim, R.

AU - Klovstad, M.R.
TI - Brines and evaporites: Analogs for Martian life
PY - 2004
T2 - Advances in Space Research
VL - 33
IS - 8
SP - 1244
EP - 1246
DO - 10.1016/j.asr.2003.08.034
UR - https://www.scopus.com/inward/record.uri?eid=2-s2.0-2342648944&doi=10.1016%2fj.asr.2003.08.034&partnerID=40&md5=7bdcc5af3dbecf3acaec2ce7eda850fb
AD - SETI Inst/NASA - Ames Res Center, Moffett Field, CA 94035, United States
AB - Data from recent Mars missions suggest that Mars almost certainly had abundant liquid water on its surface at some time in the past. As a result, Mars has emerged as a key solar system target that could have harbored some form of life in the past, and which could perhaps still possess remnants of life in brine-containing permafrost. As Mars lost its atmosphere it became cold and dry. Any remaining water on the surface may have formed saline brine pockets within the permafrost. These brine pockets may either be an "oasis" for an extant Martian biota, or the last refuge of an extinct Martian biota. Eventually, these brine pockets would have dried to form evaporites. Evaporites are deposits that result from the evaporation of saline water, which on earth represent primarily halite (NaCl), gypsum, (CaSO
4 2H 2
O), and anhydrite (CaSO
4). Evaporites that contain bacterial and algal assemblages exist on earth today and are well known in the fossil record. The most likely organism type to survive in a brine or evaporite on earth is a halophile. The objective of this study was to determine the potential of microbes to survive in frozen evaporites. Washed mid-log phase and stationary phase cultures of Haloarcula-G (a species isolated by us during a previous study) and Halobacterium salinarum were either suspended in brine (25% NaCl solution), dried, and then exposed to -20 or -80 °C. For comparison, cultures of Deinococcus radiodurans, Escherichia coli , and Pseudomonas fluorescens were treated similarly, except they were resuspended in 0.5% NaCl solution. Also, to mimic a brine pocket samples of washed mid-log phase cells of each organism were placed in an aqueous solution of 25% NaCl, or in their respective nutrient medium containing 25% NaCl. Periodically, samples of the cells were removed and tested for survival. Data from these experiments suggest that halophiles survive better than non-halophiles under low temperature conditions. These observations would suggest that halophiles might survive in evaporites contained in permafrost. © 2004 COSPAR. Published by Elsevier Ltd. All rights reserved.
KW - Cells
KW - Earth (planet)
KW - Escherichia coli
KW - Evaporation
KW - Microorganisms

KW - Permafrost
KW - Saline water
KW - Solar system
KW - Solutions
KW - Thermal effects
KW - Brine pockets
KW - Evaporites
KW - Liquid water
KW - Mid-log phase
KW - brine
KW - evaporite
KW - exobiology
KW - halophyte
KW - Mars
KW - origin of life
KW - Space research
KW - Analogs for Martian life
KW - Astrobiology
KW - Brines and evaporites
KW - Halophiles
KW - Osmophiles
PB - Elsevier Ltd
SN - 02731177 (ISSN)
LA - English
J2 - Adv. Space Res.
M3 - Article
DB - Scopus
N1 - Export Date: 23 February 2023; Cited By: 83; Correspondence Address: R.L. Mancinelli; SETI Inst/NASA - Ames Res Center, Moffett Field, CA 94035, United States; email: rmancinelli@mail.arc.nasa.gov; CODEN: ASRSD
ER -

TY - JOUR
AU - Buijs, G.J.A.
AU - Goldstein, R.H.
AU - Hasiotis, S.T.
AU - Roberts, J.A.
TI - Preservation of microborings as fluid inclusions
PY - 2004
T2 - Canadian Mineralogist
VL - 42
IS - 5
SP - 1563
EP - 1581
DO - 10.2113/gscanmin.42.5.1563
UR - https://www.scopus.com/inward/record.uri?eid=2-s2.0-12344300565&doi=10.2113%2fgscanmin.42.5.1563&partnerID=40&md5=f40058beeb1b0f622cb4c14309b448c6
AD - Department of Geology, University of Kansas, Lawrence, KS 66045, 1475 Jayhawk Boulevard, United States
AB - In this study, we identify endolithic microbial borings preserved as fluid inclusions in Pennsylvanian and Permian brachiopods from the subsurface of western Kansas. Endolithic

microbial boring is a new biologically controlled mode of formation for inclusion vacuoles. The microborings are preserved as three types of linear, curved, and branched arrays of tubular fluid inclusions, each with a distinct diameter. Each of the thousands of microborings observed in 60 brachiopod fragments has changed shape by forming calcite partitions that have divided the original tubules into separate aligned fluid inclusions. On average, only 35% of the original lengths of the microborings are preserved as fluid inclusions; values range from less than 5% to about 85%. Enlarged diameters of inclusions, variable vapor-to-liquid ratio in paired inclusions, and low salinities of inclusions indicate that closed-system necking down is one of the mechanisms responsible for alteration of the microborings. Preservation of parallel walls, fillings with luminescent calcite and high salinity of included fluid indicate that open-system cementation is the other mechanism responsible for alteration of the microborings. As most inclusions have leaked and refilled or stretched during thermal re-equilibration, these fluid inclusions are not particularly reliable as records of diagenetic history. The linear arrays of fluid inclusions that remain after alteration of the original microboring are excellent as trace fossils of microbial activity. The record preserved here indicates how fluid-inclusion techniques can be applied to identify and determine the timing of entrapment of microbial trace fossils in ancient Earth and extraterrestrial materials.
KW - Calcite
KW - Linear systems
KW - Salinity measurement
KW - Vapors
KW - Endolithic microbial boring
KW - Fluid inclusions
KW - Linear arrays
KW - boring
KW - brachiopod
KW - Mars
KW - origin of life
KW - Permian
KW - taphonomy
KW - trace fossil
KW - Mineralogy
KW - Borings
KW - Endoliths
KW - Extraterrestrial life
KW - Fluid inclusions
KW - Ichnology
KW - Life on Mars
KW - Microbes
KW - Origin of life
KW - Trace fossils
PB - Mineralogical Association of Canada
SN - 00084476 (ISSN)
LA - English
J2 - Can. Mineral.
M3 - Article

DB - Scopus
N1 - Export Date: 23 February 2023; Cited By: 8; Correspondence Address: G.J.A. Buijs; Department of Geology, University of Kansas, Lawrence, KS 66045, 1475 Jayhawk Boulevard, United States; email: gbuijs@ku.edu; CODEN: CAMIA
ER -

TY - JOUR
AU - Goryniuk, M.C.
AU - Rivard, B.A.
AU - Jones, B.
TI - The reflectance spectra of opal-A (0.5-25 μm) from the Taupo Volcanic Zone: Spectra that may identify hydrothermal systems on planetary surfaces
PY - 2004
T2 - Geophysical Research Letters
VL - 31
IS - 24
SP - 1
EP - 4
DO - 10.1029/2004GL021481
UR - https://www.scopus.com/inward/record.uri?eid=2-s2.0-14544272353&doi=10.1029%2f2004GL021481&partnerID=40&md5=ebe49ce6ec86563918b8c6ae1e0736e6
AD - Dept. of Earth/Atmospheric Sciences, University of Alberta, Edmonton, Alta. T6G 2E9, 1-26 Earth Sciences Building, Canada
AB - Opal-A, the main component of siliceous. sinters in many terrestrial hydrothermal systems, is a hydrated silicate that commonly incorporates silicified microorganisms. The detection of opal-A on the surface of Mars, therefore, may carry important implications in the search for extraterrestrial life. Sinters from the discharge apron of Ohaaki Pool (North Island, New Zealand) yielded reflectance spectra (wavelengths between 0.5-25 μm) that indicate the presence of absorbed water, trapped water, and silanol in opal-A. Two classes (1 and 2) of reflectance spectra were detected between the wavelengths of 6-13 μm. The Class-1 spectrum is similar to existing opal spectra, and was collected from well-consolidated samples that had low porosity. The Class-2 spectrum is unique when compared to the spectra of other silica polymorphs, and was collected from poorly consolidated samples that had high porosity. Copyright 2004 by the American Geophysical Union.
KW - Australasia
KW - Eastern Hemisphere
KW - New Zealand
KW - North Island
KW - Taupo Volcanic Zone
KW - World
KW - Geophysics
KW - Hydrates
KW - Hydrothermal synthesis
KW - Microorganisms
KW - Porosity
KW - Reflection
KW - Silica

KW - Sintering
KW - exobiology
KW - hydrothermal system
KW - Mars
KW - mineralogy
KW - opal
KW - spectral reflectance
KW - Hydrated silicate
KW - Hydrothermal systems
KW - Reflectance spectra
KW - Volcanoes
KW - 3934 Mineral physics: Optical, infrared, and Raman spectroscopy
KW - 5460 Planetology: Solid surface planets: Physical properties of materials
KW - 5464 Planetology: Solid surface planets: Remote sensing
KW - 6225 Planetology: Solar system objects: Mars
SN - 00948276 (ISSN)
LA - English
J2 - Geophys. Res. Lett.
M3 - Article
DB - Scopus
N1 - Export Date: 23 February 2023; Cited By: 28; CODEN: GPRLA
ER -

TY - CONF
TI - Proceedings of the III European workshop on exo-astrobiology Mars: The search for life
PY - 2004
T2 - European Space Agency, (Special Publication) ESA SP
IS - 545
UR - https://www.scopus.com/inward/record.uri?eid=2-s2.0-2342460379&partnerID=40&md5=1b0f10243512c83abb39352f9f2a00fd
AB - The proceedings contains 107 papers from the conference of the III European Workshop on Exo-Astrobiology Mars: The Search for Life. The topics discussed include: ground-based IR imaging spectroscopy of Mars; a miniature UV-VIS spectrometer for the surface of mars; UV radiation effects over microorganisms and study of protective agents; use of UV radiation for detecting life related phenomena in planetary atmospheres; importance of iron cycle, relevance to mars exploration and the range of life habitats in volcanic terrains of mars.
KW - Biochemistry
KW - Carbon dioxide
KW - Charge coupled devices
KW - Data reduction
KW - Dehydration
KW - Hydrothermal synthesis
KW - Light sources
KW - Mathematical models
KW - Microbiology
KW - Microorganisms
KW - Oxidation
KW - Photosynthesis

KW - Raman spectroscopy
KW - Ultraviolet radiation
KW - Ultraviolet spectroscopy
KW - Water absorption
KW - Astrobiology
KW - Biogeology
KW - EiRev
KW - Hydrothermal fluids
KW - Mars atmosphere
KW - Mars exploration
KW - Mars geology
KW - Radiation exposure
KW - Radiative transfer
KW - Surface deposits
KW - Martian surface analysis
SN - 03796566 (ISSN)
LA - English
J2 - Eur Space Agency Spec Publ ESA SP
M3 - Conference review
DB - Scopus
N1 - Export Date: 23 February 2023; Cited By: 0; CODEN: ESPUD
ER -

TY - CONF
AU - Hoover, R.B.
AU - Pikuta, E.V.
AU - Wickramasinghe, N.C.
AU - Wallis, M.K.
AU - Sheldon, R.B.
TI - Astrobiology of comets
PY - 2004
T2 - Proceedings of SPIE - The International Society for Optical Engineering
VL - 5555
C7 - 11
SP - 93
EP - 106
DO - 10.1117/12.566496
UR - https://www.scopus.com/inward/record.uri?eid=2-s2.0-14944384630&doi=10.1117%2f12.566496&partnerID=40&md5=9b3fb233eddb375d58dbae464af49b89
AD - Astrobiology Laboratory, NASA/MSFC/NSSTC, Huntsville, AL 35805, 320 Sparkman Dr., United States
AD - Cardiff Centre for Astrobiology, Cardiff University, United Kingdom
AB - We review the current state of knowledge concerning microbial extremophiles and comets and the potential significance of comets to Astrobiology. We model the thermal history of a cometary body, regarded as an assemblage of boulders, dust, ices and organics, as it approaches a perihelion distance of ~ 1AU. The transfer of incident energy from sunlight into the interior leads to the melting of near surface ices, some under stable porous crust, providing possible habitats for a wide range of microorganisms. We provide data concerning new evidence for indigenous microfossils in CI

meteorites, which may be the remains of extinct cometary cores. We discuss the dominant microbial communities of polar sea-ice, Antarctic ice sheet, and cryoconite environments as possible analogs for microbial ecosystems that may grow in sub-crustal pools or in ice/water films in comets.
KW - Astronomy
KW - Bacteria
KW - Ecosystems
KW - Energy transfer
KW - Fossil fuels
KW - Ice
KW - Meteorites
KW - Oceanography
KW - Water
KW - Carbonaceous chondrites
KW - Comets
KW - Cryoconite
KW - Cyanobacteria
KW - Extremophiles
KW - Orgueil
KW - Biochemistry
KW - Carbonaceous chondrites
KW - Comets
KW - Cryoconite
KW - Cyanobacteria
KW - Extremophiles
KW - Meteorites
KW - Orgueil
SN - 0277786X (ISSN)
LA - English
J2 - Proc SPIE Int Soc Opt Eng
M3 - Conference paper
DB - Scopus
N1 - Export Date: 23 February 2023; Cited By: 21; Correspondence Address: R.B. Hoover; Astrobiology Laboratory, NASA/MSFC/NSSTC, Huntsville, AL 35805, 320 Sparkman Dr., United States; email: Richard.Hoover@NASA.GOV; CODEN: PSISD
ER -

TY - CONF
AU - Hoover, R.B.
AU - Pikuta, E.V.
TI - Microorganisms on Comets, Europa and the Polar Ice Caps of Mars
PY - 2004
T2 - Proceedings of SPIE - The International Society for Optical Engineering
VL - 5163
SP - 191
EP - 201
DO - 10.1117/12.513432
UR - https://www.scopus.com/inward/record.uri?eid=2-s2.0-1942423229&doi=10.1117%2f12.513432&partnerID=40&md5=d0c15ccef325e69d5d6a4868641d5ca5

AD - Astrobiology Group, NASA, Natl. Space Sci./Technology Center, Huntsville, AL 35805, 320 Sparkman Dr., United States
AB - Microbial extremophiles live on Earth wherever there is liquid water and a source of energy. Observations by ground-based observatories, space missions, and satellites have provided strong evidence that water ice exists today on comets, Europa, Callisto, and Ganymede and in the snow, permafrost, glaciers and polar ice caps of Mars. Studies of the cryoconite pools and ice bubble systems of Antarctica suggest that solar heating of dark rocks entrained in ice can cause localized melting of ice providing ideal conditions for the growth of microbial communities with the creation of micro-environments where trapped metabolic gasses produce entrained isolated atmospheres as in the Antarctic ice-bubble systems. It is suggested that these considerations indicate that several groups of microorganisms should be capable of episodic growth within liquid water envelopes surrounding dark rocks in cometary ices and the permafrost and polar caps of Mars. We discuss some of the types of microorganisms we have encountered within the permafrost and snow of Siberia, the cryoconite pools of Alaska, and frozen deep within the Antarctic ice sheet above Lake Vostok.
KW - Cosmic rays
KW - Earth (planet)
KW - Fungi
KW - Glaciers
KW - Rocks
KW - Snow
KW - Space applications
KW - Astrobiology
KW - Comets
KW - Europa
KW - Microbials extremophiles
KW - Microorganisms
KW - Astrobiology
KW - Comets
KW - Europa
KW - Lake Vostok
KW - Mars
KW - Microbial extremophiles
KW - Psychrophiles
SN - 0277786X (ISSN)
LA - English
J2 - Proc SPIE Int Soc Opt Eng
M3 - Conference paper
DB - Scopus
N1 - Export Date: 23 February 2023; Cited By: 6; Correspondence Address: R.B. Hoover; Astrobiology Group, NASA, Natl. Space Sci./ Technology Center, Huntsville, AL 35805, 320 Sparkman Dr., United States; email: Richard.Hoover@NASA.GOV; CODEN: PSISD
ER -

TY - JOUR
AU - Hodyss, R.
AU - McDonald, G.
AU - Sarker, N.

AU - Smith, M.A.
AU - Beauchamp, P.M.
AU - Beauchamp, J.L.
TI - Fluorescence spectra of Titan tholins: In-situ detection of astrobiologically interesting areas on Titan's surface
PY - 2004
T2 - Icarus
VL - 171
IS - 2
SP - 525
EP - 530
DO - 10.1016/j.icarus.2004.05.011
UR - https://www.scopus.com/inward/record.uri?eid=2-s2.0-4544381642&doi=10.1016%2fj.icarus.2004.05.011&partnerID=40&md5=9cfcce45ef9743f2f524f009a112b6a5
AD - Noyes Laboratory of Chemical Physics, California Institute of Technology, Pasadena, CA 91125, United States
AD - Jet Propulsion Laboratory, Pasadena, CA 91109, United States
AD - Department of Chemistry, University of Arizona, Tucson, AZ 85721, United States
AB - We report the three-dimensional fluorescence spectra of Titan tholins in water and acetonitrile, and separate some of the fluorescent components of tholins using two-dimensional thin layer chromatography. In acetonitrile, tholins exhibit a broad fluorescence with a maximum at 471 nm, and an excitation maximum at 410 nm. The fluorescence spectrum of a water extraction displays a more complicated spectrum with multiple peaks. TLC results indicate the presence of at least three distinct fluorescent species. In addition, we obtained the two-dimensional fluorescence spectrum of tholins in water ice at 77 K, close to the surface temperature of Titan. The fluorescence of tholins in a 77 K ice matrix is red-shifted in comparison to a water solution, and undergoes a further red-shift when the water solution is heated prior to freezing. These results suggest that a simple fluorescence probe could be used on the surface of Titan to identify sites where tholins have been mixed with water, and possibly reveal information about the extent of heating the tholin-water mixture has undergone. This would be useful for a future mission with the goal of examining the organic chemistry of Titan. © 2004 Elsevier Inc. All rights reserved.
KW - Organic chemistry
KW - Prebiotic chemistry
KW - Prebiotic environments
KW - Titan
SN - 00191035 (ISSN)
LA - English
J2 - Icarus
M3 - Article
DB - Scopus
N1 - Export Date: 23 February 2023; Cited By: 24; Correspondence Address: R. Hodyss; Noyes Laboratory of Chemical Physics, California Institute of Technology, Pasadena, CA 91125, United States; email: hodyss@caltech.edu; CODEN: ICRSA
ER -

TY - CONF
AU - Caulfield, H.J.
TI - What kinds of sense organs can nature evolve?
PY - 2004
T2 - Proceedings of SPIE - The International Society for Optical Engineering
VL - 5555
C7 - 09
SP - 241
EP - 247
DO - 10.1117/12.564557
UR - https://www.scopus.com/inward/record.uri?eid=2-s2.0-14944378505&doi=10.1117%2f12.564557&partnerID=40&md5=0a00b6bcd03f4954590f8a514b930198
AD - Alabama A and M University, Research Institute, Normal, AL 35762, PO Box 313, United States
AB - I argue that much can be said about vision systems in general without invoking any particular instances Should astrobiology be lucky enough to encounter moderately advanced alien creatures (think salamanders not people), we will be able to understand their vision systems, however odd, as special cases of a very general procedure that any complex animal or system must use to operate in its world. Indeed, it is possible to illustrate that hypothesis with terrestrial examples of very weird visual systems that humans have created for animals. Also, many 21 st Century machines exhibit primitive visual perception.
KW - Biological organs
KW - Data processing
KW - Image processing
KW - Information analysis
KW - Lenses
KW - Sensory perception
KW - Spectrum analysis
KW - Astrobiology
KW - Sense organs
KW - Spectral sensitivity curves
KW - Vision
SN - 0277786X (ISSN)
LA - English
J2 - Proc SPIE Int Soc Opt Eng
M3 - Conference paper
DB - Scopus
N1 - Export Date: 23 February 2023; Cited By: 0; CODEN: PSISD
ER -

TY - CONF
AU - Leuko, S.
AU - Weidler, G.
AU - Rittmann, S.
AU - Stan-Lotter, H.
TI - LIVE/DEAD® kit: A powerful tool to detect haloarchaeal survival (and life?) In unknown environmental samples
PY - 2004
T2 - European Space Agency, (Special Publication) ESA SP

IS - 545
SP - 231
EP - 232
UR - https://www.scopus.com/inward/record.uri?eid=2-s2.0-2342594593&partnerID=40&md5=41d1be90d4605d807ed51d8d2abb689a
AD - University of Salzburg, Inst. of Genetic and General Biology, A - 5020 Salzburg, Hellbrunnerstr. 34, Austria
AB - Extremely halophilic archaeabacteria (haloarchaea) can be found in brines as well as in Permo-Triassic rock salt (250 million years old), which indicates their potential capacity for longevity (1). A fast evaluation of the possible presence of bacterial (archaeal) life in certain environmental samples will be highly desirable for further inquiries in Astrobiology. This goal might be reached by using the LIVE/DEAD® Kit, which contains highly sensitive fluorescent dyes. As shown in this study, the kit can be successfully used for staining of species of haloarchaea and the thermoacidophilic archaeon Acidianus brierley, including differentiation between viable and non-viable cells. In addition, attempts to use the LIVE/DEAD® Kit for a direct proof of archaeal (bacterial) presence in dissolved rock salt appeared promising.
KW - Bacteria
KW - Bacteriology
KW - Brines
KW - Cell culture
KW - Cells
KW - Centrifugation
KW - Density (optical)
KW - Dyes
KW - Fluorescence
KW - Genetic engineering
KW - pH effects
KW - Rocks
KW - Suspensions (fluids)
KW - Water treatment
KW - Yeast
KW - Astrobiology
KW - Environmental stresses
KW - Incubation time
KW - Optical density (OD)
KW - Staining
KW - Environmental engineering
SN - 03796566 (ISSN)
LA - English
J2 - Eur Space Agency Spec Publ ESA SP
M3 - Conference paper
DB - Scopus
N1 - Export Date: 23 February 2023; Cited By: 1; Correspondence Address: S. Leuko; University of Salzburg, Inst. of Genetic and General Biology, A - 5020 Salzburg, Hellbrunnerstr. 34, Austria; email: stefan.leuko@sbg.ac.at; CODEN: ESPUD
ER -

TY - CONF
AU - Daly, M.G.

AU - Choi, E.
AU - Michelangeli, D.V.
AU - Carswell, A.
AU - Tremblay, I.
TI - The Canadian MET contribution to the 2007 Phoenix Mars mission
PY - 2004
T2 - International Astronautical Federation - 55th International Astronautical Congress 2004
VL - 10
SP - 6233
EP - 6237
UR - https://www.scopus.com/inward/record.uri?eid=2-s2.0-34249092285&partnerID=40&md5=1b6e6cb32ed5a39346c9e9d3bb6360a2
AD - MD Robotics, Canada
AD - York University, Canada
AD - Optech Incorporated, Canada
AD - Canadian Space Agency, Canada
AB - In August 2003, NASA selected Phoenix as the first of its low-cost, science driven missions under the Mars Scout program. Scheduled for launch in 2007, Phoenix will land in the northern Vastitas Borealis region to assess the astrobiological potential for life at high latitudes. In particular, it will verify the recent discovery of possible subsurface water ice detected by the gamma ray spectrometer aboard the orbiting Mars Odyssey spacecraft. Phoenix will also investigate the mineralogy and geochemistry of the soil and measure the properties of the atmosphere and planetary boundary layer. Canada's contribution to the Phoenix mission will be a Meteorological Station (MET). This system will consist of a lidar (light detection and ranging) instrument that will characterize boundary layer turbulence and dust and cloud circulation in the near-surface atmosphere. The MET will also measure atmospheric pressure and temperature and its lapse rate thereby providing the first surface measurements of these parameters in this Northern region. This paper summarizes the current status of the MET development, provides details about the instrument and describes Canada's participation in the project.
KW - Astrobiology
KW - Gamma ray spectrometers
KW - Ice
KW - NASA
KW - Planets
KW - Spacecraft
KW - Surface waters
KW - Mars Scout programs
KW - Phoenix mission
KW - Planetary boundary layers
KW - Subsurface water ice
KW - Space applications
LA - English
J2 - Int. Astro. Fed. Int. Astro. Congr.
M3 - Conference paper
DB - Scopus
N1 - Export Date: 23 February 2023; Cited By: 0; Correspondence Address: M.G. Daly; MD Robotics, Canada; email: mdaly@mdrobotics.ca

ER -

TY - JOUR
AU - Ozerskaya, S.M.
AU - Ivanushkina, N.E.
AU - Kochkina, G.A.
AU - Fattakhova, R.N.
AU - Gilichinsky, D.A.
TI - Mycelial fungi in cryopegs
PY - 2004
T2 - International Journal of Astrobiology
VL - 3
IS - 4
SP - 327
EP - 331
DO - 10.1017/S1473550405002260
UR - https://www.scopus.com/inward/record.uri?eid=2-s2.0-33847627830&doi=10.1017%2fS1473550405002260&partnerID=40&md5=2d67d8881803fade6e233edaf068a594
AD - All-Russian Collection of Microorganisms, G.K. Skryabin Institute of Biochemistry and Physiology of Microorganisms, Russian Academy of Sciences, Pushchino, Moscow Region 142290, Russian Federation
AD - Institute of Physicochemical and Biological Problems on Soil Science, Russian Academy of Sciences, Pushchino, Moscow Region 142290, Russian Federation
AB - Mycelial fungi from cryopegs (tundra, Kolyma lowland, Russia) have been studied. The use of media with different amounts of salt and cultivation at 4 and 25 °C allowed us to isolate filamentous fungi assigned to 11 species. The micromycetes of genus Geomyces were found most often. The total amount of fungi reached 1–4×102 CFU ml-1 of water. The extreme conditions of the cryopegs – the high salinity of the water (150–200 g l-1) and the constant low temperatures (average annual temperature is from −9 to −11 °C) – might serve as a model for the conditions of interplanetary environments. © 2004, Cambridge University Press. All rights reserved.
KW - astrobiology
KW - biodiversity
KW - mycelial fungi
KW - permafrost
KW - water brines
SN - 14735504 (ISSN)
LA - English
J2 - Int. J. Astrobiology
M3 - Article
DB - Scopus
N1 - Export Date: 23 February 2023; Cited By: 20
ER -

TY - JOUR
AU - Mahaney, W.C.
AU - Milner, M.W.
AU - Netoff, D.I.

AU - Malloch, D.
AU - Dohm, J.M.
AU - Baker, V.R.
AU - Miyamoto, H.
AU - Hare, T.M.
AU - Komatsu, G.
TI - Ancient wet aeolian environments on Earth: Clues to presence of fossil/live microorganisms on Mars
PY - 2004
T2 - Icarus
VL - 171
IS - 1
SP - 39
EP - 53
DO - 10.1016/j.icarus.2004.04.014
UR - https://www.scopus.com/inward/record.uri?eid=2-s2.0-4344569976&doi=10.1016%2fj.icarus.2004.04.014&partnerID=40&md5=9efe93f3c75ff882ad5d426a59ead0ef
AD - Geomorphology and Pedology Lab., York University, North York, Ont. M3J 1P3, 4700 Keele St., Canada
AD - Department of Geography and Geology, Sam Houston State University, Huntsville, TX 77341, United States
AD - Department of Botany, University of Toronto, Toronto, Ont. M5S 1A1, Canada
AD - Dept. of Hydrology/Water Resources, University of Arizona, Tucson, AZ 85721, United States
AD - Lunar and Planetary Laboratory, University of Arizona, Tucson, AZ 85721, United States
AD - Department of Geosystem Engineering, University of Tokyo, Tokyo, Japan
AD - United States Geological Survey, Flagstaff, AZ 86001, United States
AD - Intl. Res. School of Planetary Sci., Università d'Annunzio, 65127 Pescara, Viale Pindaro 42, Italy
AB - Ancient wet aeolian (wet-sabkha) environments on Earth, represented in the Entrada and Navajo sandstones of Utah, contain pipe structures considered to be the product of gas/water release under pressure. The sediments originally had considerable porosity allowing the ingress of living plant structures, microorganisms, clay minerals, and fine-grained primary minerals of silt and sand size from the surface downward in the sedimentary column. Host rock material is of a similar size and porosity and presumably the downward migration of fine-grained material would have been possible prior to lithogenesis and final cementation. Recent field emission scanning electron microscopy (FESEM) and EDS (energy-dispersive spectrometry) examination of sands from fluidized pipes in the Early Jurassic Navajo Sandstone reveal the presence of fossil forms resembling fungal filaments, some bearing hyphopodium-like structures similar to those produced by modern tropical leaf parasites. The tropical origin of the fungi is consistent with the paleogeography of the sandstone, which was deposited in a tropical arid environment. These fossil fungi are silicized, with minor amounts of CaCO3 and Fe, and in some cases a Si/Al ratio similar to smectite. They exist as pseudomorphs, totally depleted in nitrogen,

adhering to the surfaces of fine-grained sands, principally quartz and orthoclase. Similar wet aeolian paleoenvironments are suspected for Mars, especially following catastrophic sediment-charged floods of enormous magnitudes that are believed to have contributed to rapid formation of large water bodies in the northern plains, ranging from lakes to oceans. These events are suspected to have contributed to a high frequency of constructional landforms (also known as pseudocraters) related to trapped volatiles and water-enriched sediment underneath a thick blanket of materials that were subsequently released to the martian surface, forming piping structures at the near surface and constructional landforms at the surface. This constructional process on Mars may help unravel the complex history of some of the piping structures observed on Earth; on Earth, evidence for the constructional landforms has been all but erased and the near-surface piping structures exposed through millions of years of differential erosion and topographic inversion now occur as high-standing promontories. If the features on both Earth and Mars formed by similar processes, especially involving water and other volatiles, and since the piping structures of Earth provided suitable environments for life to thrive in, the martian features in the northern plains should be considered as prime targets for physico/mineral/chemical/microbiological analyses once the astrobiological exploration of the red planet begins in earnest.
© 2004 Elsevier Inc. All rights reserved.
KW - Entombed microorganisms on sand clasts
KW - Extraterrestrial life
KW - Pipe-like structures on Mars and Earth
SN - 00191035 (ISSN)
LA - English
J2 - Icarus
M3 - Article
DB - Scopus
N1 - Export Date: 23 February 2023; Cited By: 25; Correspondence Address: W.C. Mahaney; Geomorphology and Pedology Lab., York University, North York, Ont. M3J 1P3, 4700 Keele St., Canada; email: bmahaney@yorku.ca; CODEN: ICRSA
ER -

TY - JOUR
AU - Kirkland, L.E.
AU - Herr, K.C.
AU - Adams, P.M.
TI - A different perspective for the Mars rover "Opportunity" site: Fine-grained, consolidated hematite and hematite coatings
PY - 2004
T2 - Geophysical Research Letters
VL - 31
IS - 5
SP - L057041
EP - 4
DO - 10.1029/2003gl019284
UR - https://www.scopus.com/inward/record.uri?eid=2-s2.0-18244424270&doi=10.1029%2f2003gl019284&partnerID=40&md5=ef3da5c704de6243a0008e21d40d0085

AD - Lunar and Planetary Institute, Houston, TX 77058, 3600 Bay Area Blvd., United States
AD - The Aerospace Corporation, El Segundo, CA 90245-4691, 2350 East El Segundo Blvd., United States
AB - Since 2001, there have been two, parallel interpretations of Mars Global Surveyor Thermal Emission Spectrometer (TES) observations of Sinus Meridiani, which are: (1) coarse-grained ("gray") hematite is the only spectral match; and (2) fine-grained hematite with particles closer than ~wavelength ("fine-intimate hematite", e.g., coating, ferricrete) is a better match, but coarse hematite is also viable. The TES team interpreted the spectra as consistent only with a large deposit (~750 km × 350 km) of coarse hematite (>5-10 μm grain size). Coarse hematite is considered strong evidence for longstanding water, which led to the decision to land the rover Opportunity there. On the other hand, the Aerospace/LPI remote sensing team argued that fine-intimate hematite can better match TES spectra. A thin coating (~5-10 μm thick) and a low exposure (<5%) could cause the observed signatures. The distinction is important because: (1) It is unknown whether fine-grained hematite implies abundant water; (2) Fine-intimate hematite may explain the non-detection of coexisting aqueous alteration minerals and the lack of hematite wind streaks; (3) Current "hematite abundance maps" may instead map the surface texture; (4) Coatings may be of astrobiology interest; (5) Studies are needed to determine whether visible-infrared spectra can definitively distinguish fine-intimate from coarse hematite. Copyright 2004 by the American Geophysical Union.
KW - Grain size and shape
KW - Planetary landers
KW - Spectrometers
KW - Spectrum analysis
KW - Alternation minerals
KW - Astrobiology
KW - Hematite
KW - hematite
KW - Mars
KW - spectral analysis
KW - spectrometer
KW - Martian surface analysis
PB - American Geophysical Union
SN - 00948276 (ISSN)
LA - English
J2 - Geophys. Res. Lett.
M3 - Article
DB - Scopus
N1 - Export Date: 23 February 2023; Cited By: 8; Correspondence Address: L.E. Kirkland; Lunar and Planetary Institute, Houston, TX 77058, 3600 Bay Area Blvd., United States; email: kirkland@lpi.usra.edu; CODEN: GPRLA
ER -

TY - JOUR
AU - Trevors, J.T.
AU - Abel, D.L.

TI - Chance and necessity do not explain the origin of life
PY - 2004
T2 - Cell Biology International
VL - 28
IS - 11
SP - 729
EP - 739
DO - 10.1016/j.cellbi.2004.06.006
UR - https://www.scopus.com/inward/record.uri?eid=2-s2.0-9244224677&doi=10.1016%2fj.cellbi.2004.06.006&partnerID=40&md5=11880852220d84d8f140357180a83948
AD - Laboratory of Microbial Technology, Department of Environmental Biology, Rm. 3220, Bovey Bldg., Univ. G., United States
AD - The Gene Emergence Project, The Origin-of-Life Foundation Inc., 113 Hedgewood Dr., Greenbelt, M., United States
AB - Where and how did the complex genetic instruction set programmed into DNA come into existence? The genetic set may have arisen elsewhere and was transported to the Earth. If not, it arose on the Earth, and became the genetic code in a previous lifeless, physical-chemical world. Even if RNA or DNA were inserted into a lifeless world, they would not contain any genetic instructions unless each nucleotide selection in the sequence was programmed for function. Even then, a predetermined communication system would have had to be in place for any message to be understood at the destination. Transcription and translation would not necessarily have been needed in an RNA world. Ribozymes could have accomplished some of the simpler functions of current protein enzymes. Templating of single RNA strands followed by retemplating back to a sense strand could have occurred. But this process does not explain the derivation of "sense" in any strand. "Sense" means algorithmic function achieved through sequences of certain decision-node switch-settings. These particular primary structures determine secondary and tertiary structures. Each sequence determines minimum-free-energy folding propensities, binding site specificity, and function. Minimal metabolism would be needed for cells to be capable of growth and division. All known metabolism is cybernetic - that is, it is programmatically and algorithmically organized and controlled. © 2004 International Federation for Cell Biology. Published by Elsevier Ltd. All rights reserved.
KW - DNA
KW - ribozyme
KW - RNA
KW - algorithm
KW - bay region binding site
KW - biogenesis
KW - cell division
KW - cell growth
KW - cell metabolism
KW - cybernetics
KW - DNA binding motif
KW - DNA sequence
KW - DNA structure
KW - DNA template

KW - enzyme activity
KW - gene insertion
KW - genetic analysis
KW - genetic code
KW - human
KW - molecular biology
KW - nonhuman
KW - nucleotide sequence
KW - review
KW - RNA sequence
KW - RNA structure
KW - RNA transcription
KW - RNA translation
KW - Astrobiology
KW - Cellular communication
KW - Chance
KW - DNA
KW - Evolution
KW - Genetic control
KW - Information theory
KW - Life origin
KW - Necessity
KW - Panspermia
KW - RNA
PB - Wiley-Blackwell Publishing Ltd
SN - 10656995 (ISSN)
LA - English
J2 - Cell Biol. Int.
M3 - Article
DB - Scopus
N1 - Export Date: 23 February 2023; Cited By: 39; CODEN: CBIIE
ER -

TY - CONF
AU - Levin, G.V.
TI - Interpretation of new results from Mars with respect to life
PY - 2004
T2 - Proceedings of SPIE - The International Society for Optical Engineering
VL - 5555
C7 - 14
SP - 126
EP - 138
DO - 10.1117/12.561213
UR - https://www.scopus.com/inward/record.uri?eid=2-s2.0-14944381099&doi=10.1117%2f12.561213&partnerID=40&md5=d523ecbd57148beb50232fc4c104e723
AD - Spherix Incorporated, Beltsville, MD 20705, 12051 Indian Creek Court, United States
AB - NASA has frequently stated that its highest priority is the search for extraterrestrial life. However, no life detection instruments have been sent to Mars since the Viking Mission in 1976 produced highly disputed evidence for microbial life in the Martian soil. The unfortunately lost Beagle 2 and the successfully landed

Spirit and Opportunity are all devoid of means to investigate the Viking findings or otherwise to determine whether or not life exists or ever existed on the red planet. However, all of these spacecraft contain instruments that are designed to obtain data ancillary to that vital and supreme question. Imaging and spectral data have now arrived from the European Space Agency's orbiter, Mars Express, and from NASA's Mars Exploration Rovers Spirit and Opportunity. These data are discussed from the standpoint of their impact on the prospects for life on Mars, and, specifically, on the 1976 Viking Labeled Release (LR) experiments that the author claims proved the presence of active microbial life in the topsoil of Mars.
KW - Ammonia
KW - Data reduction
KW - Earth (planet)
KW - Extraterrestrial atmospheres
KW - Methane
KW - Microbiology
KW - Oxidation
KW - Planets
KW - Ammonia on Mars
KW - Life on Mars
KW - Mars data 2004
KW - Mars Express
KW - Methane on Mars
KW - Opportunity
KW - Organics on Mars
KW - Oxidants on Mars
KW - Spirit
KW - Water on Mars
KW - Martian surface analysis
KW - Ammonia on Mars
KW - Life on Mars
KW - Mars data 2004
KW - Mars Express
KW - Methane on mars
KW - Opportunity
KW - Organics on Mars
KW - Oxidants on Mars
KW - Spirit
KW - Water on Mars
SN - 0277786X (ISSN)
LA - English
J2 - Proc SPIE Int Soc Opt Eng
M3 - Conference paper
DB - Scopus
N1 - Export Date: 23 February 2023; Cited By: 0; Correspondence Address: G.V. Levin; Spherix Incorporated, Beltsville, MD 20705, 12051 Indian Creek Court, United States; email: glevin@spherix.com; CODEN: PSISD
ER -

TY - CONF
AU - Bar-Cohen, Y.
AU - Sherrit, S.

AU - Chang, Z.
AU - Wessel, L.
AU - Bao, X.
AU - Doran, P.T.
AU - Fritsen, C.H.
AU - Kenig, F.
AU - McKay, C.P.
AU - Murray, A.
AU - Peterson, T.
TI - Subsurface ice and brine sampling using an ultrasonic/sonic gopher for life detection and characterization in the McMurdo dry valleys
PY - 2004
T2 - Proceedings of SPIE - The International Society for Optical Engineering
VL - 5388
SP - 53
EP - 61
DO - 10.1117/12.539280
UR - https://www.scopus.com/inward/record.uri?eid=2-s2.0-10044247566&doi=10.1117%2f12.539280&partnerID=40&md5=a32f931c874c41e43931029289d90216
AD - JPL/Caltech, M-S 82-105, Pasadena, CA 91109, 4800 Oak Grove Dr, United States
AD - University of Illinois at Chicago, Earth and Environmental Sciences, Chicago, IL 60607, 845 West Taylor Street (MC 186), United States
AD - Desert Research Institute, Division of Earth Sciences, Reno, NV 89512, 2215 Raggio Parkway, United States
AD - NASA Ames Research Center, Division of Space Science, Moffett Field, CA 94035, United States
AD - Cybersonics, Inc., Erie, PA 16510, 5368 Kuhl Rd., United States
AB - There is growing evidence for ice and fluids near the surface of Mars with potential discharge of brines, which may preserve a record of past life on the planet. Proven techniques to sample Mars subsurface will be critical for future NASA astrobiology missions that will search for such records. The required technology studies are underway in the McMurdo Dry valleys, Antarctica, which is serving as a Mars analog. The ice layer on Lake Vida in the dry valleys is estimated to be 20-meter thick where below 16-m depth there is a mix of ice and brine, which has never been sampled directly due to logistical constraints. A novel light weight, low power ultrasonic/sonic driller/corer (USDC) mechanism was developed that overcomes the need for high axial loads required by drilling via conventional techniques. The USDC was modified to produce an Ultrasonic/Sonic Gopher that is being developed to core down to the 20-m depth for in situ analysis and sample collection. Coring ice at -20°C as in Lake Vida suggests that it is a greater challenge and current efforts are focused on the problems of ice core cutting, ice chip handling and potential ice melt (and refreezing) during drilling. An analytical model and a prototype are being developed with an effort to optimize the design while addressing the thermal issues, drilling rate, power, mass and the electromechanical

behavior.
KW - Biodiversity
KW - Characterization
KW - Constraint theory
KW - Drilling
KW - Martian surface analysis
KW - Melting
KW - Sampling
KW - Ice drilling
KW - Life detection
KW - Planetary sampling
KW - Ultrasonic/sonic Gopher
KW - USDC
KW - Ice
KW - Ice drilling
KW - Life detection
KW - Planetary sampling
KW - Ultrasonic/sonic Gopher
KW - USDC
SN - 0277786X (ISSN)
LA - English
J2 - Proc SPIE Int Soc Opt Eng
M3 - Conference paper
DB - Scopus
N1 - Export Date: 23 February 2023; Cited By: 8; Correspondence Address: Y. Bar-Cohen; JPL/Caltech, M-S 82-105, Pasadena, CA 91109, 4800 Oak Grove Dr, United States; email: yosi@jpl.nasa.gov; CODEN: PSISD
ER -

TY - CONF
AU - Pikuta, E.V.
AU - Hoover, R.B.
TI - Astrobiological significance of chemolithoautotrophic acidophiles
PY - 2004
T2 - Proceedings of SPIE - The International Society for Optical Engineering
VL - 5163
SP - 179
EP - 190
DO - 10.1117/12.513419
UR - https://www.scopus.com/inward/record.uri?eid=2-s2.0-1942423237&doi=10.1117%2f12.513419&partnerID=40&md5=a3c02c0306f95ee4535c15e4f3665a86
AD - Natl. Space Sci./Technology Center, NASA, Huntsville, AL 35805, SD-50 320 Sparkman Dr., United States
AB - For more than a century (since Winogradsky discovered lithoautotrophic bacteria) there has been a dilemma in microbiology about life that first inhabited the Earth. Which types of life forms first appeared in the primordial oceans during the earliest geological period on Earth as the primary ancestors of modern biological diversity? How did a metabolism of ancestors evolve: from lithoautotrophic to lithoheterotrophic and organoheterotrophic or

from organoheterotrophic to organoautotrophic and lithomixotrophic
types? At the present time, it is known that chemolithoheterotrophic
and chemolithoautotrophic metabolizing bacteria are wide spread in
different ecosystems. On Earth the acidic ecosystems are associated
with geysers, volcanic fumaroles, hot springs, deep sea hydrothermal
vents, caves, acid mine drainage and other technogenic ecosystems.
Bioleaching played a significant role on a global geological scale
during the Earth's formation. This important feature of bacteria has
been successfully applied in industry. The lithoautotrophs include
Bacteria and Archaea belonging to diverse genera containing
thermophilic and mesophilic species. In this paper we discuss the
lithotrophic microbial acidophiles and present some data with a
description of new acidophilic iron- and sulfur-oxidizing bacterium
isolated from the Chena Hot Springs in Alaska. We also consider the
possible relevance of microbial acidophiles to Venus, Io, and acidic
inclusions in glaciers and icy moons.
KW - Amino acids
KW - Bacteria
KW - Biopolymers
KW - Earth (planet)
KW - Ecosystems
KW - Iron
KW - Metabolism
KW - Polycyclic aromatic hydrocarbons
KW - Acidophile
KW - Astrobiology
KW - Bioleaching
KW - Microbiology
KW - Acidophile
KW - Astrobiology
KW - Bioleaching
KW - Iron- and sulfur-oxidizing bacteria
KW - Lithoautotroph
SN - 0277786X (ISSN)
LA - English
J2 - Proc SPIE Int Soc Opt Eng
M3 - Conference paper
DB - Scopus
N1 - Export Date: 23 February 2023; Cited By: 3; CODEN: PSISD
ER -

TY - JOUR
AU - Takai, K.
AU - Gamo, T.
AU - Tsunogai, U.
AU - Nakayama, N.
AU - Hirayama, H.
AU - Nealson, K.H.
AU - Horikoshi, K.
TI - Geochemical and microbiological evidence for a hydrogen-based,
hyperthermophilic subsurface lithoautotrophic microbial ecosystem
(HyperSLiME) beneath an active deep-sea hydrothermal field
PY - 2004
T2 - Extremophiles

VL - 8
IS - 4
SP - 269
EP - 282
DO - 10.1007/s00792-004-0386-3
UR - https://www.scopus.com/inward/record.uri?eid=2-s2.0-9744226636&doi=10.1007%2fs00792-004-0386-3&partnerID=40&md5=bd92a80d5db9401c590b798eace2fa48
AD - Subground Animalcule Retrieval (SUGAR) Project, Frontier Research System for Extremophiles, Japan Marine Science and Technology Center (JAMSTEC), Yokosuka 237-0061, 2-15 Natsushima-cho, Japan
AD - Division of Earth and Planetary Sciences, Graduate School of Science, Hokkaido University, Sapporo 060-0810, N10, W8, Japan
AD - Department of Earth Sciences, University of Southern California, Los Angeles, CA 90089-0740, 3651 Trousdale Pkwy., United States
AB - Subsurface microbial communities supported by geologically and abiologically derived hydrogen and carbon dioxide from the Earth's interior are of great interest, not only with regard to the nature of primitive life on Earth, but as potential analogs for extraterrestrial life. Here, for the first time, we present geochemical and microbiological evidence pointing to the existence of hyperthermophilic subsurface lithoautotrophic microbial ecosystem (HyperSLiME) dominated by hyperthermophilic methanogens beneath an active deep-sea hydrothermal field in the Central Indian Ridge. Geochemical and isotopic analyses of gaseous components in the hydrothermal fluids revealed heterogeneity of both concentration and carbon isotopic compositions of methane between the main hydrothermal vent (0.08 mM and -13.8‰ PDB, respectively) and the adjacent divergent vent site (0.2 mM and -18.5‰ PDB, respectively), representing potential subsurface microbial methanogenesis, at least in the divergent vent emitting more 13C-depleted methane. Extremely high abundance of magmatic energy sources such as hydrogen (2.5 mM) in the fluids also encourages a hydrogen-based, lithoautotrophic microbial activity. Both cultivation and cultivation-independent molecular analyses suggested the predominance of Methanococcales members in the superheated hydrothermal emissions and chimney interiors along with the other major microbial components of Thermococcales members. These results imply that a HyperSLiME, consisting of methanogens and fermenters, occurs in this tectonically active subsurface zone, strongly supporting the existence of hydrogen-driven subsurface microbial communities. © Springer-Verlag 2004.
KW - Archaea
KW - Bacteria
KW - Base Sequence
KW - DNA, Complementary
KW - Ecosystem
KW - Geology
KW - Hydrogen
KW - Methanococcales
KW - Microbiological Techniques
KW - Oligonucleotide Probes

KW - Phylogeny
KW - Seawater
KW - Methanobacteriales
KW - Methanococcales
KW - Thermococcales
KW - complementary DNA
KW - hydrogen
KW - sea water
KW - Archaebacterium
KW - article
KW - bacterium
KW - chemistry
KW - classification
KW - ecosystem
KW - geology
KW - isolation and purification
KW - Methanococcales
KW - methodology
KW - microbiological examination
KW - microbiology
KW - nucleotide sequence
KW - oligonucleotide probe
KW - phylogeny
KW - Central Indian Ridge
KW - Deep-sea hydrothermal vent
KW - Hydrogen-oxidizing
KW - Hyperthermophilic
KW - Methanogen
KW - Subsurface
SN - 14310651 (ISSN)
C2 - 15309563
LA - English
J2 - Extremophiles
M3 - Article
DB - Scopus
N1 - Export Date: 23 February 2023; Cited By: 241; Correspondence Address: K. Takai; Subground Animalcule Retrieval (SUGAR) Project, Frontier Research System for Extremophiles, Japan Marine Science and Technology Center (JAMSTEC), Yokosuka 237-0061, 2-15 Natsushima-cho, Japan; email: kent@jamstec.go.jp; CODEN: EXTRF
ER -

TY - CONF
AU - González-Toril, E.
AU - Amils, R.
TI - Molecular ecology of the Tinto River, an extreme acidic environment of astrobiology interest
PY - 2004
T2 - European Space Agency, (Special Publication) ESA SP
IS - 545
SP - 207
EP - 208
UR - https://www.scopus.com/inward/record.uri?eid=2-s2.0-2342614887&partnerID=40&md5=38df601a64c420caf78632d6ddcaa267

AD - Centro de Astrobiología, Torrejon de Ardoz, Madrid 28850, Spain
AD - Centro de Biología Molecular, Cantoblanco, Madrid 28049, Spain
AD - INTA, Centro de Astrobiología, Torrejon de Ardoz, Madrid 28850, Spain
AB - Complementary molecular ecology techniques have been used to characterize the Tinto River, an extreme environment with a rather constant acidic pH all along its course (mean pH 2.3) and high concentration of heavy metals (Fe, Cu, Zn, As and Cr). Comparative sequence analysis of amplified 16S rRNAs and 16S rRNA genes resolved by denaturing gradient gel electrophoresis (DGGE) allowed members of four bacterial genera: Acidithiobacillus, Leptospirillum, Acidiphilium and "Ferrimicrobium", and two archaeal genera: Ferroplasma and Thermoplasma to be identified at different sampling stations along the river. The quantitative evaluation of the prokaryotic diversity using in situ hybridization with fluorescence labeled rRNA-targeted oligonucleotides (FISH) showed that the bulk prokaryotic biomass of the water column, up to 80%, corresponded to members of three bacterial species: Acidithiobacillus ferrooxidans, Leptospirillum spp. and Acidiphilium spp., all of them related to iron metabolism. Taking into consideration the characteristics of the habitat, the physiological properties and spatial distribution of the identified microorganisms, a model for the Tinto geomicrobiology based on the iron cycle has been advanced. The geomicrobiological characterization of this habitat is under way to gain information of life systems based on chemolithotrophic energy, a model of astrobiologycal interest.
KW - Acidity
KW - Astronomy
KW - Bacteria
KW - Biodiversity
KW - Biomass
KW - Ecology
KW - Electrophoresis
KW - Fluorescence
KW - Geology
KW - pH effects
KW - Rivers
KW - RNA
KW - Astrobiology
KW - Chemolithotropic energy
KW - Denaturing gradient gel electrophoresis (DGGE)
KW - Geomicrobiology
KW - Microbiology
SN - 03796566 (ISSN)
LA - English
J2 - Eur Space Agency Spec Publ ESA SP
M3 - Conference paper
DB - Scopus
N1 - Export Date: 23 February 2023; Cited By: 0; Correspondence Address: E. González-Toril; Centro de Astrobiología, Torrejon de Ardoz, Madrid 28850, Spain; email: etoril@cbm.uam.es; CODEN: ESPUD
ER -

TY - JOUR
AU - Jura, M.
TI - An observational signature of evolved oceans on extrasolar terrestrial planets
PY - 2004
T2 - Astrophysical Journal
VL - 605
IS - 1 II
SP - L65
EP - L68
DO - 10.1086/386549
UR - https://www.scopus.com/inward/record.uri?eid=2-s2.0-2542599329&doi=10.1086%2f386549&partnerID=40&md5=0c5cae62af7ed4fa3e38b7fd92f22959
AD - Department of Physics, Univ. of California at Los Angeles, Knudsen Hall, Los Angeles, CA 90095-1562, United States
AB - The increase with time in the luminosity of a main-sequence star can eventually lead to substantial evaporation of the oceans on an orbiting terrestrial planet. Subsequently, the gas-phase H2O in the planet's upper atmosphere can be photodissociated by stellar ultraviolet, and the resulting atomic hydrogen then may be lost in a wind. This gaseous envelope may pass in front of the host star and produce transient, detectable ultraviolet absorption in the Lyman lines in systems older than 1 Gyr.
KW - Astrobiology
KW - Planetary systems
PB - Institute of Physics Publishing
SN - 0004637X (ISSN)
LA - English
J2 - Astrophys. J.
M3 - Article
DB - Scopus
N1 - Export Date: 23 February 2023; Cited By: 19; Correspondence Address: M. Jura; Department of Physics, Univ. of California at Los Angeles, Knudsen Hall, Los Angeles, CA 90095-1562, United States; email: jura@clotho.astro.ucla.edu
ER -

TY - CONF
AU - Jonsson, J.
AU - Kobrick, R.L.
TI - Mars in Utah
PY - 2004
T2 - International Astronautical Federation - 55th International Astronautical Congress 2004
VL - 9
SP - 5728
EP - 5737
UR - https://www.scopus.com/inward/record.uri?eid=2-s2.0-34249023895&partnerID=40&md5=bdf8962425ac1b721eeae5e0d25d0d65
AD - Luleå Technical University, Sweden
AD - Pennsylvania State University, United States
AB - Each year the Mars Society makes a call for volunteers from all over the world to man their Mars research stations where they

can simulate a first habitat for humans on the planet Mars. This year Jonas Jonsson was selected as a member of Crew 20 (Dec. 20 - Jan. 4) and Ryan L. Kobrick of Crew 25 (Feb. 25 - Mar. 13) for the Mars Desert Research Station (MDRS) situated in the red desert of Utah. MDRS is one of the two stations that the Mars Society is operating. The other one is FMARS (Flashline Mars Arctic Research Station) and is situated on Devon Island in Canada. The purpose of these stations is to study and learn how humans live and work in Martian situations, to learn as much as possible about a future mission to Mars right here on Earth. Throughout our stay we experienced many obstacles that are realistic to what future explorers might face while living on Mars and we will try to share from our own experiences what we have learned and try to inspire the next generation who will hopefully be the ones that will actually make this reality.
KW - Astrobiology
KW - Computer simulation
KW - Project management
KW - Space stations
KW - Mars Desert Research Station (MDRS)
KW - Mars research stations
KW - Mars Society
KW - Martian surface analysis
LA - English
J2 - Int. Astro. Fed. Int. Astro. Congr.
M3 - Conference paper
DB - Scopus
N1 - Export Date: 23 February 2023; Cited By: 0; Correspondence Address: J. Jonsson; Luleå Technical University, Sweden; email: jonjon-1@student.ltu.se
ER -

TY - JOUR
AU - de Pablo, M.A.
AU - Fairén, A.G.
TI - Atlantis basin, Sirenum Terrae, Mars: Geological setting and astrobiological implications
PY - 2004
T2 - International Journal of Astrobiology
VL - 3
IS - 3
SP - 257
EP - 263
DO - 10.1017/S147355040400196X
UR - https://www.scopus.com/inward/record.uri?eid=2-s2.0-85012958078&doi=10.1017%2fS147355040400196X&partnerID=40&md5=20d6f0c21ef331e4d870027e6062e9b4
AD - Área de Geología. Departamento de Matemática y Física Aplicadas y Ciencias de la Naturaleza, Escuela Superior de Ciencias Experimentales y Tecnología, Universidad Rey Juan Carlos, 28933 Móstoles, Madrid, Spain
AD - Centro de Biología Molecular, CSIC-Universidad Autónoma de Madrid, 28049 Cantoblanco, Madrid, Spain
AB - The accomplishment of detailed geomorphological studies is a

prerequisite for the location of regions in which the prevailing conditions in the past, or at present, may allow the development of possible life forms. The Atlantis basin, located in Sirenum Terrae, Southern hemisphere of Mars, is one of these astrobiologically interesting regions, where the existence of geological features such as ancient volcanic edifices, sedimentary deposits of an ancient lake and recent gullies seem to indicate the long-term presence of a thermal source and a water reservoir deep and stable enough to sustain biological processes. Here we describe the most relevant topographic and geomorphologic features in the region, highlighting the possibility for liquid water to have been present in the basin and outskirts in different moments of Mars' history. We also apply this analysis to an initial discussion of the influence of the hydrogeological evolution of the region in the putative development and/or survival of life forms in Atlantis basin. © 2004, Cambridge University Press. All rights reserved.
KW - astrobiology
KW - Atlantis basin
KW - dikes
KW - geology
KW - lakes
KW - Mars
KW - water
SN - 14735504 (ISSN)
LA - English
J2 - Int. J. Astrobiology
M3 - Article
DB - Scopus
N1 - Export Date: 23 February 2023; Cited By: 7
ER -

TY - CONF
AU - Rummel, J.D.
AU - Race, M.S.
TI - Got life? hours of boredom followed by moments of sheer terror (and that's just with the press)
PY - 2004
T2 - International Astronautical Federation - 55th International Astronautical Congress 2004
VL - 4
SP - 2421
EP - 2423
UR - https://www.scopus.com/inward/record.uri?eid=2-s2.0-34249070480&partnerID=40&md5=e90842675a6fbe01a30a8327cd1dd455
AD - Science Mission Directorate, NASA Headquarters, Washington, DC 20546, United States
AD - SETI Institute, Mountain View, CA 94303, United States
AB - It may be hoped that an initial discovery of extraterrestrial life and its disclosure will be done by accident. An event of that kind would have its own dynamic, and while communications about the discovery might be strained at times, there would be less likelihood that lines of inquiry and discourse would have already been taken by the participants and the Press. In an ideal world (or worlds), the discovery would come ready-made with a picture or pictures that

would be useful as an immediate verification of its reality. But such is not the way of the real world (or worlds, apparently). Lessons learned from the publication of the ALH84001 results in Science magazine are indicative of what may be a more likely scenario. Nonetheless, even that publication was held in confidence for much of the time leading up to NASA's press conference, and the science team doing the work was accordingly insulated from press inquiry while the work was underway. Envisioning a Mars sample return mission, or other, similar sort of endeavor that may involve a dedicated team of scientists - working under continual public scrutiny - it is clear that the circumstances that surround any fundamental discovery about life in the sample would be quite different. Planning for a communications strategy to support the operations of a Mars sample receiving facility (or facilities) must take those circumstances into account. An optimization of the time spent communicating the results of the facility's work should acknowledge the time and effort required, and make provisions for the work to proceed without extensive interruptions - and without being influenced by the expectations of the Press or the public. This paper will discuss some of the initial planning associated with the communications strategy surrounding such a facility.
KW - Martian surface analysis
KW - Optimization
KW - Technical presentations
KW - Extraterrestrial life
KW - Publication
KW - Space research
LA - English
J2 - Int. Astro. Fed. Int. Astro. Congr.
M3 - Conference paper
DB - Scopus
N1 - Export Date: 23 February 2023; Cited By: 0; Correspondence Address: J.D. Rummel; Science Mission Directorate, NASA Headquarters, Washington, DC 20546, United States; email: jrummel@hq.nasa.gov
ER -

TY - CONF
AU - Hyden, W.
AU - Macher, W.
AU - Kargl, G.
AU - Kolb, C.
AU - Lammer, H.
AU - Rucker, H.O.
TI - Implications of dielectric properties of the Martian surface soil on the search for subsurface liquid water or ice reservoirs
PY - 2004
T2 - European Space Agency, (Special Publication) ESA SP
IS - 545
SP - 209
EP - 210
UR - https://www.scopus.com/inward/record.uri?eid=2-s2.0-2342509697&partnerID=40&md5=204bfa62e64d452873b3185cfc6de1e7
AD - Space Research Institute, Austrian Academy of Sciences, A-8042

Graz, Schmiedlstraße 6, Austria
AD - Inst. Geophys., Astrophys./Meteorol., University of Graz, A-8010 Graz, Universitätsplatz 5, Austria
AB - The main goal of the MARSIS experiment on board Mars Express is the search for liquid water and ice on the surface and subsurface of Mars, which is thought to be essential in the search for extraterrestrial life. The reflection and attenuation of the MARSIS radar waves at the surface or at subsurface layers depend strongly on the dielectric properties of the Martian soil. Therefore the exact mineralogical composition of the Martian surface material is an important factor. Especially ferric oxides, such as hematite and maghemite, are expected to contain appreciable portions of nanoscaled phases. These extraordinary small grains may have contributions to the dielectric constant completely different from the 50 µm ones. Therefore we propose laboratory measurements with Martian analogues in the space simulation facility.
KW - Computer simulation
KW - Electric conductivity
KW - Ground penetrating radar systems
KW - Ice
KW - Mineralogy
KW - Permittivity
KW - Porosity
KW - Radar reflection
KW - Soils
KW - Sounding apparatus
KW - Thermal conductivity
KW - Biological activities
KW - Extraterrestrial life
KW - Ice reservoirs
KW - Terrestrial rocks
KW - Martian surface analysis
SN - 03796566 (ISSN)
LA - English
J2 - Eur Space Agency Spec Publ ESA SP
M3 - Conference paper
DB - Scopus
N1 - Export Date: 23 February 2023; Cited By: 0; Correspondence Address: W. Hyden; Space Research Institute, Austrian Academy of Sciences, A-8042 Graz, Schmiedlstraße 6, Austria; email: weh@igam.uni-graz.at; CODEN: ESPUD
ER -

TY - JOUR
AU - Halberg, F.
AU - Cornélissen, G.
AU - Regal, P.
AU - Otsuka, K.
AU - Wang, Z.
AU - Katinas, G.S.
AU - Siegelova, J.
AU - Homolka, P.
AU - Prikryl, P.
AU - Chibisov, S.M.

AU - Holley, D.C.
AU - Wendt, H.W.
AU - Bingham, C.
AU - Palm, S.L.
AU - Sonkowsky, R.P.
AU - Sothern, R.B.
AU - Pales, E.
AU - Mikulecky, M.
AU - Tarquini, R.
AU - Perfetto, F.
AU - Salti, R.
AU - Maggioni, C.
AU - Jozsa, R.
AU - Konradov, A.A.
AU - Kharlitskaya, E.V.
AU - Revilla, M.
AU - Wan, C.
AU - Herold, M.
AU - Syutkina, E.V.
AU - Masalov, A.V.
AU - Faraone, P.
AU - Singh, R.B.
AU - Singh, R.K.
AU - Kumar, A.
AU - Singh, R.
AU - Sundaram, S.
AU - Sarabandi, T.
AU - Pantaleoni, G.
AU - Watanabe, Y.
AU - Kumagai, Y.
AU - Gubin, D.
AU - Uezono, K.
AU - Olah, A.
AU - Borer, K.
AU - Kanabrocki, E.A.
AU - Bathina, S.
AU - Haus, E.
AU - Hillman, D.
AU - Schwartzkopff, O.
AU - Bakken, E.E.
AU - Zeman, M.
TI - Chronoastrobiology: Proposal, nine conferences, heliogeomagnetics, transyears, near-weeks, near-decades, phylogenetic and ontogenetic memories
PY - 2004
T2 - Biomedicine and Pharmacotherapy
VL - 58
IS - SUPPL. 1
SP - S150
EP - S187
DO - 10.1016/S0753-3322(04)80025-8
UR - https://www.scopus.com/inward/record.uri?eid=2-s2.0-18144421468&doi=10.1016%2fS0753-3322%2804%2980025-8&partnerID=40&md5=fa7745e0ac33dd8ea889de02478aa2da

AD - Halberg Chronobiology Center, University of Minnesota, Minneapolis, MN, United States
AD - Tokyo Women's Medical University, Daini Hospital, Tokyo, Japan
AD - Sichuan University, Chengdu, China
AD - Masaryk University, Brno, Czech Republic
AD - Department of Physiopathology, Russian People's Friendship University, Moscow, Russian Federation
AD - San Jose State University, San Jose, CA, United States
AD - Sophia Foundation, Bratislava, Slovakia
AD - 1st Medical Clinic, Comenius University, Bratislava, Slovakia
AD - University of Florence, Florence, Italy
AD - University of Milan, Milan, Italy
AD - University of Pecs, Pecs, Hungary
AD - Institute of Biochemical Physics, Moscow, Russian Federation
AD - University of Valladolid, Valladolid, Spain
AD - University of Innsbruck, Innsbruck, Austria
AD - Scientific Center for Children's Health, Academy of Medical Sciences, Moscow, Russian Federation
AD - Lebedev Physical Institute, Moscow, Russian Federation
AD - Halberg Hospital and Research Institute, Moradabad, India
AD - CSM Medical University, Lucknow, India
AD - Government Medical College (G.M.D. Hospital), Amritsar, India
AD - UAMS-AHEC, Fort Smith, AR, United States
AD - University of L'Aquila, Aquila, Italy
AD - Kitasato University East Hospital, Sagamihara, Kanagawa, Japan
AD - Tyumen Government Medical Institute, Tyumen, Russian Federation
AD - Institute of Health Science, Kyushu University, Fukuoka, Japan
AD - University of Michigan, Ann Arbor, MI, United States
AD - Edward Hines Jr. VA Hospital, Hines, IL, United States
AD - North Hawaii Community Hospita., Kamuela, HI, United States
AD - Department of Animal Physiology and Ethology, Comenius University, Bratislava, Slovakia
AB - "Chronoastrobiology: are we at the threshold of a new science? Is there a critical mass for scientific research?" A simple photograph of the planet earth from outer space was one of the greatest contributions of space exploration. It drove home in a glance that human survival depends upon the wobbly dynamics in a thin and fragile skin of water and gas that covers a small globe in a mostly cold and vast universe. This image raised the stakes in understanding our place in that universe, in finding out where we came from and in choosing a path for survival. Since that landmark photograph was taken, new astronomical and biomedical information and growing computer power have been revealing that organic life, including human life, is and has been connected to invisible (non-photic) forces in that vast universe in some surprising ways. Every cell in our body is bathed in an external and internal environment of fluctuating magnetism. It is becoming clear that the fluctuations are primarily caused by an intimate and systematic interplay between forces within the bowels of the earth - which the great physician and father of magnetism William Gilbert called a 'small magnet' - and the thermonuclear turbulence within the sun, an enormously larger magnet than the earth, acting upon organisms, which are minuscule magnets. It follows and is also increasingly apparent that

these external fluctuations in magnetic fields can affect virtually every circuit in the biological machinery to a lesser or greater degree, depending both on the particular biological system and on the particular properties of the magnetic fluctuations. The development of high technology instruments and computer power, already used to visualize the human heart and brain, is furthermore making it obvious that there is a statistically predictable time structure to the fluctuations in the sun's thermonuclear turbulence and thus to its magnetic interactions with the earth's own magnetic field and hence a time structure to the magnetic fields in organisms. Likewise in humans, and in at least those other species that have been studied, computer power has enabled us to discover statistically defined endogenous physiological rhythms and further direct effects that are associated with these invisible geo- and heliomagnetic cycles. Thus, what once might have been dismissed as noise in both magnetic and physiological data does in fact have structure. And we may be at the threshold of understanding the biological and medical meaning and consequences of these patterns and biological-astronomical linkages as well. Structures in time are called chronomes; their mapping in us and around us is called chronomics. The scientific study of chronomes is chronobiology. And the scientific study of all aspects of biology related to the cosmos has been called astrobiology. Hence we may dub the new study of time structures in biology with regard to influences from cosmo-helio- and geomagnetic rhythms chronoastrobiology. It has, of course, been understood for centuries that the movements of the earth in relation to the sun produce seasonal and daily cycles in light energy and that these have had profound effects on the evolution of life. It is now emerging that rhythmic events generated from within the sun itself, as a large turbulent magnet in its own right, can have direct effects upon life on earth. Moreover, comparative studies of diverse species indicate that there have also been ancient evolutionary effects shaping the endogenous chronomic physiological characteristics of life. Thus the rhythms of the sun can affect us not only directly, but also indirectly through the chronomic patterns that solar magnetic rhythms have created within our physiology in the remote past. For example, we can document the direct exogenous effects of given specific solar wind events upon human blood pressure and heart rate. We also have evidence of endogenous internal rhythms in blood pressure and heart rate that are close to but not identical to the period length of rhythms in the solar wind. These were installed genetically by natural selection at some time in the distant geological past. This interpretive model of the data makes the prediction that the internal and external influences on heart rate and blood pressure can reinforce or cancel each other out at different times. A study of extensive clinical and physiological data shows that the interpretive model is robust and that internal and external effects are indeed augmentative at a statistically significant level. Chronoastrobiological studies are contributing to basic science - that is, our understanding is being expanded as we recognize heretofore unelaborated linkages of life to the complex dynamics of the sun, and even to heretofore unelaborated evolutionary phenomena. Once, one might have thought of solar storms as mere transient

'perturbations' to biology, with no lasting importance. Now we are on the brink of understanding that solar turbulences have played a role in shaping endogenous physiological chronomes. There is even documentation for correlations between solar magnetic cycles and psychological swings, eras of belligerence and of certain expressions of sacred or religious feelings. Chronoastrobiology can surely contribute to practical applications as well as to basic science. It can help develop refinements in our ability to live safely in outer space, where for example at the distance of the moon the magnetic influences of the sun will have an effect upon humans unshielded by the earth's native magnetic field. We should be better able to understand these influences as physiological and mechanical challenges, and to improve our estimations of the effects of exposure. Chronoastrobiology moreover holds great promise in broadening our perspectives and powers in medicine and public health right here upon the surface of the earth. Even the potential relevance of chronoastrobiology for practical environmental and agricultural challenges cannot be ruled out at this early stage in our understanding of the apparently ubiquitous effects of magnetism and hence perhaps of solar magnetism on life. The evidence already mentioned that fluctuations in solar magnetism can influence gross clinical phenomena such as rates of strokes and heart attacks, and related cardiovascular variables such as blood pressure and heart rate, should illustrate the point that the door is open to broad studies of clinical implications. The medical value of better understanding magnetic fluctuations as sources of variability in human physiology falls into several categories: 1) The design of improved analytical and experimental controls in medical research. Epidemiological analyses require that the multiple sources causing variability in physiological functions and clinical phenomena be identified and understood as thoroughly as possible, in order to estimate systematic alterations of any one variable. 2) Preventive medicine and the individual patients'care. There are no flat 'baselines', only reference chronomes. Magnetic fluctuations can be shown statistically to exacerbate health problems in some cases. The next step should be to determine whether vulnerable individuals can be identified by individual monitoring. Such vulnerable patients may then discover that they have the option to avoid circumstances associated with anxiety during solar storms, and/or pay special attention to their medication or other treatments. Prehabilitation by self-help can hopefully complement and eventually replace much costly rehabilitation. 3) Basic understanding of human physiological mechanisms. The chronomic organization of physiology implies a much more subtle dynamic integration of functions than is generally appreciated. All three categories of medical value in turn pertain to the challenges for space science of exploring and colonizing the solar system. The earth's native magnetic field acts like an enormous umbrella that offers considerable protection on the surface from harsh solar winds of charged particles and magnetic fluxes. The umbrella becomes weaker with distance from the earth and will offer little protection for humans, other animals, and plants in colonies on the surface of the moon or beyond. Thus it is important before more distant colonization is planned or implemented to better understand those magnetism-related biological-solar interactions

that now can be studied conveniently on earth. Thorough lifelong maps of chronomes should be generated and made available to the scientific world. Individual workers should not have to rediscover cycles and rhythms, which can be a confusing source of variation when ignored. By contrast, once mapped, the endpoints of a spectral element in chronomes can serve everybody, for instance for the detection of an elevation of vascular disease risk. Chronomic cartography from birth to death is a task for governments to implement, thereby serving the interests of transdisciplinary science and the general public alike. Governments have supported the systematic gathering of physical data for nearly two centuries on earth in order to serve exploration, trade, and battle on land and on the seas, and indeed agriculture. These government functions have been augmented enormously with satellite technology in more recent decades. The biological comparison with regard to government support and chronomic needs would be the mapping of the human genome. The complete sequences of DNA might have eventually become available due simply to countless individual laboratories publishing piecemeal results in scattered journals. But there would have been enormous redundancy and confusion in assembling and piecing the information together. The waste of time and money involved in the redundancy and confusion would have been considerable. © 2004 Elsevier SAS. All rights reserved.
KW - astronomy
KW - biomedicine
KW - blood pressure
KW - chronobiology
KW - chronotherapy
KW - circadian rhythm
KW - computer
KW - genome
KW - government
KW - heart infarction
KW - heart rate
KW - human
KW - magnetic field
KW - magnetism
KW - medical information
KW - medical technology
KW - nonhuman
KW - patient care
KW - phylogeny
KW - physiology
KW - prediction
KW - preventive medicine
KW - priority journal
KW - review
KW - Russian Federation
KW - sampling
KW - statistical significance
KW - stroke
KW - survival
KW - Astrobiology
KW - Chronoastrobiology

KW - Chronobiology
KW - Chronome
KW - Chronomics
PB - Elsevier Masson SAS
SN - 07533322 (ISSN)
C2 - 15754855
LA - English
J2 - Biomed. Pharmacother.
M3 - Article
DB - Scopus
N1 - Export Date: 23 February 2023; Cited By: 58; Correspondence Address: F. Halberg; Halberg Chronobiology Center, University of Minnesota, Minneapolis, MN, United States; email: halbe001@umn.edu; CODEN: BIPHE
ER -

TY - JOUR
AU - Gardner, J.N.
TI - The physical constants as biosignature: An anthropic retrodiction of the Selfish Biocosm Hypothesis
PY - 2004
T2 - International Journal of Astrobiology
VL - 3
IS - 3
SP - 229
EP - 236
DO - 10.1017/S1473550404002162
UR - https://www.scopus.com/inward/record.uri?eid=2-s2.0-84904640709&doi=10.1017%2fS1473550404002162&partnerID=40&md5=9b9a7a93a9a2888a5286366ae9e54afb
AD - Suite 810, Portland, Oregon 97201, Gardner & Gardner, 111 S. W. Columbia Street, United States
AB - Goal 7 of the NASA Astrobiology Roadmap states: 'Determine how to recognize signatures of life on other worlds and on early Earth. Identify biosignatures that can reveal and characterize past or present life in ancient samples from Earth, extraterrestrial samples measured in situ, samples returned to Earth, remotely measured planetary atmospheres and surfaces, and other cosmic phenomena.' The cryptic reference to 'other cosmic phenomena' would appear to be broad enough to include the possible identification of biosignatures embedded in the dimensionless constants of physics. The existence of such a set of biosignatures – a life-friendly suite of physical constants – is a retrodiction of the Selfish Biocosm (SB) hypothesis. This hypothesis offers an alternative to the weak anthropic explanation of our indisputably life-friendly cosmos favoured by (1) an emerging alliance of M-theory-inspired cosmologists and advocates of eternal inflation like Linde and Weinberg, and (2) supporters of the quantum theory-inspired sum-over-histories cosmological model offered by Hartle and Hawking. According to the SB hypothesis, the laws and constants of physics function as the cosmic equivalent of DNA, guiding a cosmologically extended evolutionary process and providing a blueprint for the replication of new life-friendly progeny universes. © 2004, Cambridge University Press. All rights reserved.

KW - anthropic principle
KW - physical constants
SN - 14735504 (ISSN)
LA - English
J2 - Int. J. Astrobiology
M3 - Article
DB - Scopus
N1 - Export Date: 23 February 2023; Cited By: 4
ER -

TY - CONF
AU - Debus, A.
TI - Cospar needs for planetary protection recommendations for sample preservation dedicated to exobiology
PY - 2004
T2 - Advances in Space Research
VL - 34
IS - 11
SP - 2320
EP - 2324
DO - 10.1016/j.asr.2004.01.015
UR - https://www.scopus.com/inward/record.uri?eid=2-s2.0-9444229328&doi=10.1016%2fj.asr.2004.01.015&partnerID=40&md5=9057806b29d324d5399317139219cfa6
AD - Centre National d'Etudes Spatiales, CNES BPI 2003, 31401 Toulouse Cedex 4, 18 avenue Edouard Belin, France
AB - The present COSPAR Planetary Protection policy has been established in order to conduct scientific investigations of possible extraterrestrial life forms, precursors and remnants avoiding to jeopardize exobiological experimentations. This policy is implemented by the issuance of Planetary Protection recommendations in order to control, limit or avoid the biological contamination of other worlds. Their goal is also to protect the Earth from the potential hazard posed by extraterrestrial matter carried by a spacecraft returning from another planet. Policy and recommendations are built upon scientific opinions and, if available, using scientific results coming from exobiological investigations. If sample preservation is not ensured, inducing contamination or change in sample material properties, some material characteristics can be affected and may induce false results concerning presence of life or biohazard. It concerns in situ investigations as well as analysis of extra terrestrial samples on Earth. False results could lead to propose unappropriate Planetary Protection requirements. This paper will propose recommendations and requirements for sample preservation in order to ensure the validity of exobiological science and directly to consolidate COSPAR policy, giving the best assurance to requirements for forward and back-contamination. © 2004 COSPAR. Published by Elsevier Ltd. All rights reserved.
KW - Cleaning
KW - Contamination
KW - Earth (planet)
KW - Extraterrestrial atmospheres
KW - Hazards

KW - Heavy ions
KW - Irradiation
KW - Landing
KW - Moon
KW - Planetary surface analysis
KW - Space research
KW - Spacecraft
KW - Celestial bodies
KW - Exobiology
KW - Extraterrestrial life forms
KW - Planetary protection
KW - Biological materials preservation
KW - Exobiology
KW - Extraterrestrial life forms
KW - Planetary protection
PB - Elsevier Ltd
SN - 02731177 (ISSN)
LA - English
J2 - Adv. Space Res.
M3 - Conference paper
DB - Scopus
N1 - Export Date: 23 February 2023; Cited By: 5; Correspondence Address: A. Debus; Centre National d'Etudes Spatiales, CNES BPI 2003, 31401 Toulouse Cedex 4, 18 avenue Edouard Belin, France; email: andre.debus@cnes.fr; CODEN: ASRSD
ER -

TY - JOUR
AU - Kennedy, C.B.
AU - Scott, S.D.
AU - Ferris, F.G.
TI - Hydrothermal phase stabilization of 2-line ferrihydrite by bacteria
PY - 2004
T2 - Chemical Geology
VL - 212
IS - 3-4 SPEC.ISS.
SP - 269
EP - 277
DO - 10.1016/j.chemgeo.2004.08.017
UR - https://www.scopus.com/inward/record.uri?eid=2-s2.0-8844267671&doi=10.1016%2fj.chemgeo.2004.08.017&partnerID=40&md5=e5141d83a4f023ebe1fc5097a64c3166
AD - Department of Geology, University of Toronto, Toronto, Ont. M5S 3B1, 22 Russell Street, Canada
AD - Deep Biosphere Laboratory, CMB-Microbiology, Göteborg University, SE-405 30 Göteborg, Box 462, Sweden
AB - Poorly ordered 2-line ferrihydrite deposits from the caldera of Axial Volcano, a site of hydrothermal vent activity along the Juan de Fuca Ridge, are characterized by an abundance of bacterial structures resembling Gallionella ferruginea, Leptothrix ochracea and PV-1 filaments encrusted with nanoparticulate ferrihydrite. Ferrihydrite formed in the absence of bacteria can be metastable and will subsequently react to form more structurally ordered iron oxide

phases such as hematite or goethite. Inspection of samples from 2-day 80 °C aqueous heating experiments by electron microscopy and X-ray diffraction revealed that a natural sample of bacteriogenic ferrihydrite from Axial Volcano did not undergo a phase transition, whereas synthetic ferrihydrite, absent of bacteria, transformed to hematite after 2 days. A series of bacterial-ferrihydrite composites exhibited decreasing amounts of transformation to hematite with increased amounts of Fe(III) bound to bacteria. Complete inhibition was observed when 50% of the total Fe was bound as ferric iron and 50% was present as ferrihydrite. For the synthetic ferrihydrite samples containing bacteria, the inhibition of ferrihydrite transformation is attributed to microbial surface functional groups (i.e. carboxyl, phosphoryl, amine) constraining the Brownian motion of surface bound ferrihydrite nanoparticles, thereby eliminating the aggregation and rotation of particles required for crystal growth. The stabilization of ferrihydrite will prolong the longevity of a highly reactive mineral phase, which may have implications for marine geochemistry and environmental remediation. Preservation of bacteria encrusted with transformation resistant ferrihydrite may enhance the potential usefulness of bacteriogenic ferrihydrite to harbour bacterial microfossils, imparting these types of samples with potential use for paleontology and astrobiology. © 2004 Elsevier B.V. All rights reserved.
KW - Axial Volcano
KW - Juan de Fuca Ridge
KW - oceanic regions
KW - Pacific Ocean
KW - World
KW - ferrihydrite
KW - geomicrobiology
KW - hematite
KW - hydrothermal vent
KW - phase transition
KW - Axial Volcano
KW - Bacteria
KW - Ferrihydrite
KW - Hydrothermal
KW - Inhibition
KW - Phase transition
SN - 00092541 (ISSN)
LA - English
J2 - Chem. Geol.
M3 - Article
DB - Scopus
N1 - Export Date: 23 February 2023; Cited By: 80; Correspondence Address: F.G. Ferris; Department of Geology, University of Toronto, Toronto, Ont. M5S 3B1, 22 Russell Street, Canada; email: ferris@geology.utoronto.ca
ER -

TY - JOUR
AU - Bishop, J.L.
AU - Murad, E.
AU - Lane, M.D.

AU - Mancinelli, R.L.
TI - Multiple techniques for mineral identification on Mars: A study of hydrothermal rocks as potential analogues for astrobiology sites on Mars
PY - 2004
T2 - Icarus
VL - 169
IS - 2
SP - 311
EP - 323
DO - 10.1016/j.icarus.2003.12.025
UR - https://www.scopus.com/inward/record.uri?eid=2-s2.0-2942567551&doi=10.1016%2fj.icarus.2003.12.025&partnerID=40&md5=3b7ea7fc7152df10ee0e65a9b22395ae
AD - SETI Institute, NASA-Ames Research Center, Moffett Field, CA 94035, Mail Stop 239-4, United States
AD - Bayerisches Geologisches Landesamt, D-95603 Marktredwitz, Leopoldstrasse 30, Germany
AD - Planetary Science Institute, Tucson, AZ 85705, 1700 E. Fort Lowell Rd., United States
AB - Spectroscopic studies of Mars analog materials combining multiple spectral ranges and techniques are necessary in order to obtain ground truth information for interpretation of rocks and soils on Mars. Two hydrothermal rocks from Yellowstone National Park, Wyoming, were characterized here because they contain minerals requiring water for formation and they provide a possible niche for some of the earliest organisms on Earth. If related rocks formed in hydrothermal sites on Mars, identification of these would be important for understanding the geology of the planet and potential habitability for life. XRD, thermal properties, VNIR, mid-IR, and Raman spectroscopy were employed to identify the mineralogy of the samples in this study. The rocks studied here include a travertine from Mammoth Formation that contains primarily calcite with some aragonite and gypsum and a siliceous sinter from Octopus Spring that contains a variety of poorly crystalline to amorphous silicate minerals. Calcite was detected readily in the travertine rock using any one of the techniques studied. The small amount of gypsum was uniquely identified using XRD, VNIR, and mid-IR, while the aragonite was uniquely identified using XRD and Raman. The siliceous sinter sample was more difficult to characterize using each of these techniques and a combination of all techniques was more useful than any single technique. Although XRD is the historical standard for mineral identification, it presents some challenges for remote investigations. Thermal properties are most useful for minerals with discrete thermal transitions. Raman spectroscopy is most effective for detecting polarized species such as CO3, OH, and CH, and exhibits sharp bands for most highly crystalline minerals when abundant. Mid-IR spectroscopy is most useful in characterizing Si-O (and metal-O) bonds and also has the advantage that remote information about sample texture (e.g., particle size) can be determined. Mid-IR spectroscopy is also sensitive to structural OH, CO3, and SO4 bonds when abundant. VNIR spectroscopy is best for characterizing metal excitational bands and water, and is also a good technique for identification of structural OH, CO3, SO4, or CH

bonds. Combining multiple techniques provides the most comprehensive information about mineralogy because of the different selection rules and particle size sensitivities, in addition to maximum coverage of excitational and vibrational bands at all wavelengths. This study of hydrothermal rocks from Yellowstone provides insights on how to combine information from multiple instruments to identify mineralogy and hence evidence of water on Mars. © 2004 Elsevier Inc. All rights reserved.
KW - Astrobiology
KW - Hydrothermal rocks
KW - Infrared
KW - Mars
KW - Raman
KW - Spectroscopy
PB - Academic Press Inc.
SN - 00191035 (ISSN)
LA - English
J2 - Icarus
M3 - Article
DB - Scopus
N1 - Export Date: 23 February 2023; Cited By: 59; Correspondence Address: J.L. Bishop; SETI Institute, NASA-Ames Research Center, Moffett Field, CA 94035, Mail Stop 239-4, United States; email: jbishop@mail.arc.nasa.gov; CODEN: ICRSA
ER -

TY - CONF
AU - Cosmovici, C.B.
AU - Maccone, C.
AU - Montebugnoli, S.
AU - Monari, J.
AU - Righini, S.
TI - The ITASEL project (Italian search for extraterrestrial life)
PY - 2005
T2 - International Astronautical Federation - 56th International Astronautical Congress 2005
VL - 2
SP - 1078
EP - 1088
UR - https://www.scopus.com/inward/record.uri?eid=2-s2.0-34250799007&partnerID=40&md5=2a487308a6f347308c3df10d74718050
AD - Istitito di Fisica dello Spazio Interplanetario, Istituto Nazionale di Astrofisica, 00133 Roma, Via del Fosso del Cavaliere, Italy
AD - Intern. Academy of Astronautics, Torino (TO) 10155, Via Martorelli, 43, Italy
AD - Istituto di Radioastronomia, Istituto Nazionale di Astrofisica, 40060 - Villafontana, Bologna, Via Fiorentina, Aia Cavicchio, Italy
AB - The Italian Space Agency (ASI) has approved and financed for the first time a project concerning two different aspects of Bioastronomy: 1) Participation in the international SETI-project on a national basis (SETI-ITALIA); 2) Search for the possibility of primordial life on exoplanets. After the detection of the 22 GHz

water MASER line during the impact of Comet Shoemaker/Levy9 with Jupiter in July 1994 (Cosmovici et al, ref.[1]), it has been shown that under particular physical conditions, water MASER emission can be observed from exoplanetary atmospheres. Thus, in 1999 a search program with the 32 m dish of the Medicina Radiotelescope Station started, using a fast multi-channel spectrometer. Up to now 30 exoplanetary systems have been observed; suspect variable emissions have been identified only from Epsilon Eridani, 47 Ursae Majoris, Upsilon Andromedae and EQ Pegasi. So far these detections have not been confirmed from other observatories with different methods. In order to improve the detection limits a new multi-channel spectrometer, which will be used also for the SETI program, is under development. It is a modular system: parallelising several boards it will be possible to increase the bandwidth and the number of channels. The system will be able to operate real-time FFTs (over bands narrower than 100 MHz) in parallel to KLTs (not real time) or other transforms (Wavelets, chirplet, etc...). This feature could play a key role in ordinary spectral line observations and in future applications requiring very high processing/computing power, dramatically increasing the on-line analysis performances. This back-end, together with the installation of new low-noise front-ends on the 22 GHz receiver, will allow far more effective observations.
KW - Fast Fourier transforms
KW - Masers
KW - Online systems
KW - Signal receivers
KW - Space research
KW - Spurious signal noise
KW - Computing power
KW - Exoplanetary atmospheres
KW - Extraterrestrial atmospheres
LA - English
J2 - Int. Astronautical Fed. Int. Astroanautical Congr.
M3 - Conference paper
DB - Scopus
N1 - Export Date: 23 February 2023; Cited By: 2; Correspondence Address: C.B. Cosmovici; Istitito di Fisica dello Spazio Interplanetario, Istituto Nazionale di Astrofisica, 00133 Roma, Via del Fosso del Cavaliere, Italy; email: Cristiano.Cosmovici@ifsi.rm.cnr.it
ER -

TY - JOUR
AU - Ambrosi, R.M.
AU - Talboys, D.L.
AU - Sims, M.R.
AU - Bannister, N.P.
AU - Makarewicz, M.
AU - Stevenson, T.
AU - Hutchinson, I.B.
AU - Watterson, J.I.W.
AU - Lanza, R.C.
AU - Richter, L.
AU - Mills, A.

AU - Fraser, G.W.
TI - Neutron activation analysis, gamma ray spectrometry and radiation environment monitoring instrument concept: GEORAD
PY - 2005
T2 - Nuclear Instruments and Methods in Physics Research, Section A: Accelerators, Spectrometers, Detectors and Associated Equipment
VL - 539
IS - 1-2
SP - 198
EP - 216
DO - 10.1016/j.nima.2004.10.031
UR - https://www.scopus.com/inward/record.uri?eid=2-s2.0-13444257746&doi=10.1016%2fj.nima.2004.10.031&partnerID=40&md5=a758a1c92886e14d872206566dcca5b7
AD - Department of Physics and Astronomy, Space Research Centre, University of Leicester, Leicester LE1 7RH, University Rd., United Kingdom
AD - Agency's Laboratories Seibersdorf, International Atomic Energy Agency, A-1400 Vienna, Wagramer Str. 5, Austria
AD - Dept. of Electron. and Comp. Eng., Brunel University, Uxbridge, Middlesex UB8 3PH, United Kingdom
AD - School of Physics, Schonland Res. Ctr. for Nucl. Sci., University of the Witwatersrand, Johannesburg 2050, P.O. WITS, South Africa
AD - Nuclear Engineering Department, Massachusetts Inst. of Technology, Cambridge, MA 02139, 77 Massachusetts Avenue, United States
AD - Ger. Aerosp. Research Establishment, Institute for Space Simulation, D-51147 Koln, Porz-Wahnheide, Linder Hohe, Germany
AD - Department of Geology, University of Leicester, Leicester LE1 7RH, University Rd., United Kingdom
AB - Geological processes on Earth can be related to those that may have occurred in past epochs on Mars, if analytical methods used on Earth can be operated remotely on the surface of the Red Planet. Nuclear analytical techniques commonly used in terrestrial geology are neutron activation analysis (NAA) and gamma-ray spectroscopy (GRS), which determine the elemental composition, elemental concentration and stratigraphical distribution of water in rocks and soils. We describe a detector concept called GEORAD (GEOlogical and RADiation environment package) for the proposed ExoMars rover within the ESA's Aurora Programme for the exploration of the Solar System. GEORAD consists of a compact neutron source for the NAA of rocks and soils and a GRS. The GRS has a dual role since it can be used for natural radioactivity studies and NAA. A fully depleted silicon detector coupled to neutron sensitive converters measures the solar particle and neutron flux interacting with the Martian surface. We describe how the GEORAD detector suite could contribute to the geological and biological characterisation of Mars both for the detection of extinct or extant life and to evaluate potential hazards facing future manned missions. We show how GEORAD measurements complement the astrobiological objectives of the Aurora programme. © 2004 Elsevier B.V. All rights reserved.
KW - Carbon
KW - Charge coupled devices

KW - Cooling systems
KW - Gamma rays
KW - Isotopes
KW - Particle detectors
KW - Radioactivity
KW - Solar system
KW - Spacecraft
KW - Ultraviolet radiation
KW - Astrobiology
KW - Aurora
KW - ExoMars
KW - Mars
KW - Neutron activation analysis
KW - Astrobiology
KW - Aurora
KW - ExoMars
KW - Mars
KW - Neutron activation analysis
SN - 01689002 (ISSN)
LA - English
J2 - Nucl Instrum Methods Phys Res Sect A
M3 - Article
DB - Scopus
N1 - Export Date: 23 February 2023; Cited By: 9; Correspondence Address: R.M. Ambrosi; Department of Physics and Astronomy, Space Research Centre, University of Leicester, Leicester LE1 7RH, University Rd., United Kingdom; email: rma@star.le.ac.uk; CODEN: NIMAE
ER -

TY - CONF
AU - Hara, K.
AU - Kakegawa, T.
AU - Yamashiro, K.
AU - Maruyama, A.
AU - Ishibashi, J.-I.
AU - Marumo, K.
AU - Urabe, T.
AU - Yamagishi, A.
TI - Analysis of the archaeal sub-seafloor community at Suiyo Seamount on the Izu-Bonin Arc
PY - 2005
T2 - Advances in Space Research
VL - 35
IS - 9 SPEC. ISS.
SP - 1634
EP - 1642
DO - 10.1016/j.asr.2005.04.111
UR - https://www.scopus.com/inward/record.uri?eid=2-s2.0-24644507096&doi=10.1016%2fj.asr.2005.04.111&partnerID=40&md5=e65f56f55d13ba0e88c0d3a65207a3da
AD - Department of Molecular Biology, Tokyo University of Pharmacy and Life Science, Hachioji, Tokyo 192-0392, 1432-1 Horinouchi, Japan
AD - Tohoku University, Graduate School of Science, Aramaki Aza,

Aoba, Sendai 980-0845, Japan
AD - Research Institute of Biological Resources, National Institute of Advanced Industrial Science and Technology (AIST), Tsukuba, Ibaraki 305-8566, 1-1-1 Higashi, Japan
AD - Department of Earth Planetary Sciences, Faculty of Science, Kyushu University, Higashi-ku, Fukuoka 812-8581, 6-101 Hakozaki, Japan
AD - National Institute of Advanced Industrial Science and Technology (AIST), Tsukuba, Ibaraki 305-8566, 1-1-3 No. 7, Japan
AD - Earth and Planetary Science, University of Tokyo, Bunkyo-ku, Tokyo 113-0033, 7-3-1 Hongo, Japan
AB - A sub-surface archaeal community at the Suiyo Seamount in the Western Pacific Ocean was investigated by 16S rRNA gene sequence and whole-cell in situ hybridization analyses. In this study, we drilled and cased holes at the hydrothermal area of the seamount to minimize contamination of the hydrothermal fluid in the sub-seafloor by penetrating seawater. PCR clone analysis of the hydrothermal fluid samples collected from a cased hole indicated the presence of chemolithoautotrophic primary biomass producers of Archaeoglobales and the Methanococcales-related archaeal HTE1 group, both of which can utilize hydrogen as an electron donor. We discuss the implication of the microbial community on the early history of life and on the search for extraterrestrial life. © 2005 COSPAR. Published by Elsevier Ltd. All rights reserved.
KW - Biomass
KW - Cells
KW - Contamination
KW - Genes
KW - Hydrogen
KW - Hydrothermal synthesis
KW - RNA
KW - Seawater
KW - 16S rDNA analysis
KW - Cased drilled holes
KW - Chemolithoautotrophic archaea
KW - Hydrothermal areas
KW - Sub-seafloor
KW - Suiyo Seamount
KW - Biodiversity
KW - 16S rDNA analysis
KW - Cased drilled holes
KW - Chemolithoautotrophic archaea
KW - Hydrothermal area
KW - Sub-seafloor
KW - Suiyo Seamount
PB - Elsevier Ltd
SN - 02731177 (ISSN)
C2 - 16175703
LA - English
J2 - Adv. Space Res.
M3 - Conference paper
DB - Scopus
N1 - Export Date: 23 February 2023; Cited By: 12; Correspondence Address: A. Yamagishi; Department of Molecular Biology, Tokyo

University of Pharmacy and Life Science, Hachioji, Tokyo 192-0392, 1432-1 Horinouchi, Japan; email: yamagish@ls.toyaku.ac.jp; CODEN: ASRSD
ER -

TY - CONF
AU - Gao, Y.
AU - Ellery, A.
AU - Jaddou, M.
AU - Vincent, J.
AU - Eckersley, S.
TI - A novel penetration system for in situ astrobiological studies
PY - 2005
T2 - International Journal of Advanced Robotic Systems
VL - 2
IS - 4
SP - 281
EP - 286
UR - https://www.scopus.com/inward/record.uri?eid=2-s2.0-31144463972&partnerID=40&md5=3231561aa8f26d42c6d12a0b981e4529
AD - Surrey Space Centre, University of Surrey, Guildford, United Kingdom
AD - Centre for Biomimetic and Natural Technologies, University of Bath, United Kingdom
AD - Earth Observation and Science Division, EADS Astrium, Stevenage, United Kingdom
AB - Due to ultraviolet flux in the surface layers of most solar bodies, future astrobiological research is increasingly seeking to conduct subsurface penetration and drilling to detect chemical signature for extant or extinct life. To address this issue, we present a micro-penetrator concept (mass < 10 kg) that is suited for extraterrestrial planetary deployment and in situ investigation of chemical and physical properties. The instrumentation in this concept is a bio-inspired drill to access material beneath sterile surface layer for biomarker detection. The proposed drill represents a novel concept of two-valve-reciprocating motion, inspired by the working mechanism of wood wasp ovipositors. It is lightweight (0.5 kg), driven at low power (3 W), and able to drill deep (1-2 m). Tests have shown that the reciprocating drill is feasible and has potential of improving drill efficiency without using any external force. The overall penetration system provides a small, light and energy efficient solution to in situ astrobiological studies, which is crucial for space engineering. Such a micro-penetrator can be used for exploration of terrestrial-type planets or other small bodies of the solar system with the minimum of modifications.
KW - Biology
KW - Biomarkers
KW - Planets
KW - Space research
KW - Surface phenomena
KW - Ultraviolet radiation
KW - Astrobiology
KW - Ovipositors
KW - Surface layers

KW - Wasp
KW - Astronomy
KW - Astrobiological studies
KW - Bio-inspired systems
KW - Drilling
KW - Planetary penetration
SN - 17298806 (ISSN)
LA - English
J2 - Int. J. Adv. Rob. Syst.
M3 - Conference paper
DB - Scopus
N1 - Export Date: 23 February 2023; Cited By: 20
ER -

TY - JOUR
AU - Marshall, S.
AU - Green, N.
TI - Going the distance
PY - 2005
T2 - New Scientist
VL - 185
IS - 2487
SP - 58
EP - 61
UR - https://www.scopus.com/inward/record.uri?eid=2-s2.0-14044279106&partnerID=40&md5=4409bc21fd855f19b94925694dcd4295
AD - University College London, United Kingdom
AB - Stephen Marshall and Nick Green, authors of the book Your PhD Companion, used their experiences to answer questions of upcoming and interested students PhDians. It was pointed that much of the doctral research process involves focusing on a relatively small problem and then understanding it as thoroughly as one can. Stephen and Nick suggested that if one is an aspiring academic, then taking a PhD straight away is the quickest way to get on the career ladder. It was also pointed that a PhD can provide valuable training for a job in the 'real world'.
KW - Education
KW - Employment
KW - Job analysis
KW - Learning systems
KW - Molecular biology
KW - Project management
KW - Research
KW - Societies and institutions
KW - Students
KW - Technical presentations
KW - Astrobiology
KW - Master's degree
KW - Neuroscience
KW - PhD
KW - Professional aspects
SN - 02624079 (ISSN)
LA - English
J2 - New Sci.

M3 - Review
DB - Scopus
N1 - Export Date: 23 February 2023; Cited By: 0; CODEN: NWSCA
ER -

TY - JOUR
AU - Schulze-Makuch, D.
AU - Dohm, J.M.
AU - Fairén, A.G.
AU - Baker, V.R.
AU - Fink, W.
AU - Strom, R.G.
TI - Venus, Mars, and the ices on Mercury and the Moon: Astrobiological implications and proposed mission designs
PY - 2005
T2 - Astrobiology
VL - 5
IS - 6
SP - 778
EP - 795
DO - 10.1089/ast.2005.5.778
UR - https://www.scopus.com/inward/record.uri?eid=2-s2.0-28844468973&doi=10.1089%2fast.2005.5.778&partnerID=40&md5=f3f5621cbbe9f01a984195ffda0044d2
AD - Department of Geology, Washington State University, Pullman, WA 99164, United States
AD - Department of Hydrology and Water Resources, University of Arizona, Tucson, AZ, United States
AD - Centro de Biología Molecular, CSIC, Universidad Autónoma de Madrid, Madrid, Spain
AD - Division of Physics Mathematics and Astronomy, California Institute of Technology, Pasadena, CA, United States
AD - Department of Planetary Sciences, University of Arizona, Tucson, AZ, United States
AB - Venus and Mars likely had liquid water bodies on their surface early in the Solar System history. The surfaces of Venus and Mars are presently not a suitable habitat for life, but reservoirs of liquid water remain in the atmosphere of Venus and the subsurface of Mars, and with it also the possibility of microbial life. Microbial organisms may have adapted to live in these ecological niches by the evolutionary force of directional selection. Missions to our neighboring planets should therefore be planned to explore these potentially life-containing refuges and return samples for analysis. Sample return missions should also include ice samples from Mercury and the Moon, which may contain information about the biogenic material that catalyzed the early evolution of life on Earth (or elsewhere). To obtain such information, science-driven exploration is necessary through varying degrees of mission operation autonomy. A hierarchical mission design is envisioned that includes spaceborne (orbital), atmosphere (airborne), surface (mobile such as rover and stationary such as lander or sensor), and subsurface (e.g., ground-penetrating radar, drilling, etc.) agents working in concert to allow for sufficient mission safety and redundancy, to perform extensive and challenging reconnaissance, and to lead to a thorough

search for evidence of life and habitability. © Mary Ann Liebert, Inc.
KW - Atmosphere
KW - Ice
KW - Mars
KW - Mercury
KW - Moon
KW - Planets
KW - Space Flight
KW - Venus
KW - Water
KW - Emmelichthys struhsakeri
KW - ice
KW - mercury
KW - water
KW - astronomy
KW - atmosphere
KW - methodology
KW - moon
KW - review
KW - space flight
KW - Inner Solar System
KW - Life
KW - Mars
KW - Mercury
KW - Mission design
KW - Moon
KW - Terrestrial planets
KW - Venus
SN - 15311074 (ISSN)
C2 - 16379531
LA - English
J2 - Astrobiology
M3 - Review
DB - Scopus
N1 - Export Date: 23 February 2023; Cited By: 27; Correspondence Address: D. Schulze-Makuch; Department of Geology, Washington State University, Pullman, WA 99164, United States; email: dirksm@mail.wsu.edu
ER -

TY - JOUR
AU - Raymond, S.N.
AU - Quinn, T.
AU - Lunine, J.I.
TI - Terrestrial planet formation in disks with varying surface density profiles
PY - 2005
T2 - Astrophysical Journal
VL - 632
IS - 1 I
SP - 670
EP - 676
DO - 10.1086/433179

UR - https://www.scopus.com/inward/record.uri?eid=2-s2.0-28044436098&doi=10.1086%2f433179&partnerID=40&md5=d1f05ea19a659cc22b217cccefa715a1
AD - Department of Astronomy, University of Washington, Box 351580, Seattle, WA 98195, United States
AD - Lunar and Planetary Laboratory, University of Arizona, Tucson, AZ 85287, United States
AB - The minimum-mass solar nebula (MMSN) model estimates the surface density distribution of the protoplanetary disk by assuming the planets to have formed in situ. However, significant radial migration of the giant planets likely occurred in the solar system, implying a distortion in the values derived by the MMSN method. The true density profiles of protoplanetary disks is therefore uncertain. Here we present results of simulations of late-stage terrestrial accretion, each starting from a disk of planetary embryos. We assume a power-law surface density profile that scales with heliocentric distance r as r -α and vary α between 1/2 and 5/2 (α = 3/2 for the MMSN model). We find that for steeper profiles (higher values of a), the terrestrial planets (1) are more numerous, (2) form more quickly, (3) form closer to the star, (4) are more massive, (5) have higher iron contents, and (6) have lower water contents. However, the possibility of forming potentially habitable planets does not appear to vary strongly with α.
KW - Astrobiology
KW - Methods: n-body simulations
KW - Planets and satellites: formation
PB - Institute of Physics Publishing
SN - 0004637X (ISSN)
LA - English
J2 - Astrophys. J.
M3 - Review
DB - Scopus
N1 - Export Date: 23 February 2023; Cited By: 81; Correspondence Address: S.N. Raymond; Department of Astronomy, University of Washington, Box 351580, Seattle, WA 98195, United States; email: raymond@astro.washington.edu
ER -

TY - CONF
AU - Abelson, R.D.
AU - Shirley, J.H.
TI - Exploring Europa with a surface lander powered by a small Radioisotope Power System (RPS)
PY - 2005
T2 - AIP Conference Proceedings
VL - 746
SP - 303
EP - 317
DO - 10.1063/1.1867147
UR - https://www.scopus.com/inward/record.uri?eid=2-s2.0-33847336110&doi=10.1063%2f1.1867147&partnerID=40&md5=c017c47b56c89c25b39f1db709904de2
AD - Missions and Systems Architecture Section, Jet Propulsion Laboratory, California Institute of Technology, Pasadena, CA

91109-8099, 4800 Oak Grove Drive, United States
AD - Solar System Imaging Spectroscopy Section, Jet Propulsion Laboratory, California Institute of Technology, Pasadena, CA 91109-8099, 4800 Oak Grove Drive, United States
AB - Europa is a high-priority target for future exploration because of the possibility that it may possess a subsurface liquid ocean that could sustain life. Exploring the surface of this Galilean moon, however, represents a formidable technical challenge due to the great distances involved, the high ambient radiation, and the extremely low surface temperatures. A design concept is presented for a Europa Lander Mission (ELM) powered by a small radioisotope power system (RPS) that could fly aboard the proposed Jupiter Icy Moons Orbiter (JIMO). The ELM would perform in-situ science measurements for a minimum of 30 Earth days, equivalent to approximately 8.5 Europa days. The primary science goals for the Europa lander would include astrobiology and geophysics experiments and determination of surface composition. Science measurements would include visual imagery, microseismometry, Raman spectroscopy, Laser Induced Breakdown Spectroscopy (LIBS), and measurements of surface temperature and radiation levels. The ELM spacecraft would be transported to Europa via the JIMO spacecraft as an auxiliary payload with an extended duration cruise phase (up to 13 years). After arriving at Europa, ELM would separate from JIMO and land on the moon's surface to conduct the nominal science mission. In addition to transportation, the JIMO mothership would be used to relay all lander data back to Earth, thus reducing the size and power requirement of the lander communications system. Conventional power sources were evaluated and found to be impractical for this mission due to the extended duration, low level of solar insolation (~3.7% of Earth's), the low surface temperatures (as low as 85K), and the 1.75 days of eclipse every Europa day. In contrast, a small-RPS would enable the ELM mission by powering the lander and keeping all key instrumentation and subsystems warm during the cruise and landed phases of the mission. The conceptual small-RPS is based on the existing General Purpose Heat Source (GPHS) module using thermoelectric conversion. This would generate 225 Wt (thermal) and 10.1 We (electric) at the end of the mission, and would provide an energy margin exceeding 100%. A small rechargeable lithium-ion battery would be used to handle peak load demands during the short-duration communication events and while using the higher-power instrumentation (LIBS and Raman). In summary, small-RPS technology could enable an exciting, scientifically valuable Europa lander mission designed to verify the existence of a subsurface ocean, and to search for signs of past or present life. © 2005 American Institute of Physics.
SN - 15517616 (ISSN); 0735402302 (ISBN); 978-073540230-0 (ISBN)
LA - English
J2 - AIP Conf. Proc.
M3 - Conference paper
DB - Scopus
N1 - Export Date: 23 February 2023; Cited By: 1; Correspondence Address: R. D. Abelson; Missions and Systems Architecture Section, Jet Propulsion Laboratory, California Institute of Technology, Pasadena, CA 91109-8099, 4800 Oak Grove Drive, United States; email:

Robert.D.Abelson@jpl.nasa.gov
ER -

TY - JOUR
AU - Prieto-Ballesteros, O.
AU - Kargel, J.S.
AU - Fernández-Sampedro, M.
AU - Selsis, F.
AU - Martínez, E.S.
AU - Hogenboom, D.L.
TI - Evaluation of the possible presence of clathrate hydrates in Europa's icy shell or seafloor
PY - 2005
T2 - Icarus
VL - 177
IS - 2
SP - 491
EP - 505
DO - 10.1016/j.icarus.2005.02.021
UR - https://www.scopus.com/inward/record.uri?eid=2-s2.0-26444601223&doi=10.1016%2fj.icarus.2005.02.021&partnerID=40&md5=91227359614ad3a4515f63cc16983c13
AD - Centro de Astrobiología, INTA-CSIC, 28850 Madrid, Carretera de Ajalvir km. 4, Spain
AD - Astrogeology Team, USGS, Flagstaff, AZ, United States
AD - Lafayette College, Easton, PA, United States
AD - Centre de Recherche Astronomique de Lyon, Ecole Normale Supérieure, F-69364 Lyon Cedex 7, 46 allée d'Italie, France
AB - Several substances besides water ice have been detected on the surface of Europa by spectroscopic sensors, including CO2, SO2, and H2S. These substances might occur as pure crystalline ices, as vitreous mixtures, or as clathrate hydrate phases, depending on the system conditions and the history of the material. Clathrate hydrates are crystalline compounds in which an expanded water ice lattice forms cages that contain gas molecules. The molecular gases that may constitute Europan clathrate hydrates may have two possible ultimate origins: they might be primordial condensates from the interstellar medium, solar nebula, or jovian subnebula, or they might be secondary products generated as a consequence of the geological evolution and complex chemical processing of the satellite. Primordial ices and volatile-bearing compounds would be difficult to preserve in pristine form in Europa without further processing because of its active geological history. But dissociated volatiles derived from differentiation of a chondritic rock or cometary precursor may have produced secondary clathrates that may be present now. We have evaluated the current stability of several types of clathrate hydrates in the crust and the ocean of Europa. The depth at which the clathrates of SO2, CO2, H2S, and CH4 are stable have been obtained using both the temperatures observed in the surface [Spencer, J.R., Tamppari, L.K., Martin, T.Z., Travis, L.D., 1999. Temperatures on Europa from Galileo photopolarimeter-radiometer: Nighttime thermal anomalies. Science 284, 1514-1516] and thermal models for the crust. In addition, their densities have been calculated in order to determine their buoyancy in the ocean,

obtaining different results depending upon the salinity of the ocean and type of clathrate. For instance, assuming a eutectic composition of the system MgSO4 H2O for the ocean, CO2, H2S, and CH4 clathrates would float but SO2 clathrate would sink to the seafloor; an ocean of much lower salinity would allow all these clathrates to sink, except that CH4 clathrate would still float. Many geological processes may be driven or affected by the formation, presence, and destruction of clathrates in Europa such as explosive cryomagmatic activity [Stevenson, D.J., 1982. Volcanism and igneous processes in small icy satellites. Nature 298, 142–144], partial differentiation of the crust driven by its clathration, or the local retention of heat within or beneath clathrate-rich layers because of the low thermal conductivity of clathrate hydrates [Ross, R.G., Kargel, J.S., 1998. Thermal conductivity of Solar System ices, with special reference to martian polar caps. In: Schmitt, B., De Berg, C., Festou, M. (Eds.), Solar System Ices. Kluwer Academic, Dordrecht, pp. 33–62]. On the surface, destabilization of these minerals and compounds, triggered by fracture decompression or heating could result in formation of chaotic terrain morphologies, a mechanism that also has been proposed for some martian chaotic terrains [Tanaka, K.L., Kargel, J.S., MacKinnon, D.J., Hare, T.M., Hoffman, N., 2002. Catastrophic erosion of Hellas basin rim on Mars induced by magmatic intrusion into volatile-rich rocks. Geophys. Res. Lett. 29 (8); Kargel, J.S., Prieto-Ballesteros, O., Tanaka K.L., 2003. Is clathrate hydrate dissociation responsible for chaotic terrains on Earth, Mars, Europa, and Triton? Geophys. Res. 5. Abstract 14252]. Models of the evolution of the ice shell of Europa might take into account the presence of clathrate hydrates because if gases are vented from the silicate interior to the water ocean, they first would dissolve in the ocean and then, if the gas concentrations are sufficient, may crystallize. If any methane releases occur in Europa by hydrothermal or biological activity, they also might form clathrates. Then, from both geological and astrobiological perspectives, future missions to Europa should carry instrumentation capable of clathrate hydrate detection. © 2005 Elsevier Inc. All rights reserved.
KW - Europa
KW - Geological processes
KW - Ices
KW - Mineralogy
KW - Satellite surfaces
PB - Academic Press Inc.
SN - 00191035 (ISSN)
LA - English
J2 - Icarus
M3 - Article
DB - Scopus
N1 - Export Date: 23 February 2023; Cited By: 52; Correspondence Address: O. Prieto-Ballesteros; Centro de Astrobiología, INTA-CSIC, 28850 Madrid, Carretera de Ajalvir km. 4, Spain; email: prietobo@inta.es; CODEN: ICRSA
ER -

TY - JOUR

AU - Dupraz, C.
AU - Visscher, P.T.
TI - Microbial lithification in marine stromatolites and hypersaline mats
PY - 2005
T2 - Trends in Microbiology
VL - 13
IS - 9
SP - 429
EP - 438
DO - 10.1016/j.tim.2005.07.008
UR - https://www.scopus.com/inward/record.uri?eid=2-s2.0-23944443706&doi=10.1016%2fj.tim.2005.07.008&partnerID=40&md5=c7f11b75162a36d3d1cf0b592d452211
AD - Institut de Géologie, Université de Neuchâtel, CH-2007 Neuchâtel, Rue Emile-Argand 11, Switzerland
AD - Center for Integrative Geosciences, Department of Marine Sciences, University of Connecticut, Groton, CT 06340, 1080 Shennecossett Road, United States
AB - Lithification in microbial ecosystems occurs when precipitation of minerals outweighs dissolution. Although the formation of various minerals can result from microbial metabolism, carbonate precipitation is possibly the most important process that impacts global carbon cycling. Recent investigations have produced models for stromatolite formation in open marine environments and lithification in shallow hypersaline lakes, which could be highly relevant for interpreting the rock record and searching for extraterrestrial life. Two factors that are controlled by microbial processes and physicochemical characteristics determine precipitation: exopolymeric substances and the saturation index, the latter being determined by the pH, {Ca2+} and {CO32-}. Here, we evaluate community metabolism in microbial mats and hypothesize why these organosedimentary biofilms sometimes lithify and sometimes do not. © 2005 Elsevier Ltd. All rights reserved.
KW - Bacteria
KW - Calcium Carbonate
KW - Ecosystem
KW - Environmental Microbiology
KW - Geologic Sediments
KW - Minerals
KW - Precipitation
KW - calcium ion
KW - carbonic acid
KW - biofilm
KW - carbon cycling
KW - chemical reaction
KW - ecosystem
KW - lake
KW - lithification
KW - marine environment
KW - microbial metabolism
KW - microflora
KW - microorganism
KW - nonhuman

KW - pH
KW - precipitation
KW - priority journal
KW - process model
KW - review
KW - rock
SN - 0966842X (ISSN)
C2 - 16087339
LA - English
J2 - Trends Microbiol.
M3 - Review
DB - Scopus
N1 - Export Date: 23 February 2023; Cited By: 569; Correspondence Address: P.T. Visscher; Center for Integrative Geosciences, Department of Marine Sciences, University of Connecticut, Groton, CT 06340, 1080 Shennecossett Road, United States; email: pieter.visscher@uconn.edu; CODEN: TRMIE
ER -

TY - CONF
AU - Chyba, C.F.
AU - Hand, K.P.
TI - Astrobiology: The study of the living universe
PY - 2005
T2 - Annual Review of Astronomy and Astrophysics
VL - 43
SP - 31
EP - 74
DO - 10.1146/annurev.astro.43.051804.102202
UR - https://www.scopus.com/inward/record.uri?eid=2-s2.0-26844501381&doi=10.1146%2fannurev.astro.43.051804.102202&partnerID=40&md5=3dc066977636e52cf76736f07ce615a0
AD - SETI Institute, Mountain View, CA 94043, United States
AD - Department of Geological and Environmental Sciences, Stanford University, Stanford, CA 94305, United States
AB - Astrobiology is the study of the living universe. Astronomy provides the context for the origin and evolution of life on Earth. Conversely, discoveries about the terrestrial biosphere-from extremophilic microbes to the evolution of intelligence - inform our thinking about prospects for life elsewhere. Astrobiology includes the search for extraterrestrial life via in situ exploration, spectroscopy of solar and extrasolar planetary atmospheres, and the search for extraterrestrial intelligence. This review situates astrobiology within philosophical issues of the definition of life and the biological compatibility of the universe. It reviews the habitability of the Galaxy in general and of planets and moons in particular, and summarizes current controversies in origins-of-life research and in evidence for the earliest life on Earth. It critiques certain "rare Earth" and "anthropic" arguments, and considers four approaches to deciding whether intelligent life exists elsewhere in the Galaxy. It concludes that astrobiology must also speak to the future of human civilization. Copyright © 2005 by Annual Reviews. All rights reserved.
KW - Bioastronomy

KW - Cosmobiology
KW - Exobiology
KW - SETI
SN - 00664146 (ISSN)
LA - English
J2 - Annu. Rev. Astron. Astrophys.
M3 - Conference paper
DB - Scopus
N1 - Export Date: 23 February 2023; Cited By: 108; Correspondence Address: C.F. Chyba; SETI Institute, Mountain View, CA 94043, United States; email: chyba@seti.org
ER -

TY - JOUR
AU - Spiegel, D.S.
AU - Zamojski, M.
AU - Gersch, A.
AU - Donovan, J.
AU - Haiman, Z.
TI - Can we probe the atmospheric composition of an extrasolar planet from its reflection spectrum in a high-magnification microlensing event?
PY - 2005
T2 - Astrophysical Journal
VL - 628
IS - 1 I
SP - 478
EP - 486
DO - 10.1086/430696
UR - https://www.scopus.com/inward/record.uri?eid=2-s2.0-24644506713&doi=10.1086%2f430696&partnerID=40&md5=000063a7665f53fc30c8ef2d8ed89d4d
AD - Department of Astronomy, Columbia University, New York, NY 10027, 550 West 120th Street, United States
AD - Depertment of Astronomy, University of Maryland, College Park, MD 20742, United States
AB - We revisit the possibility of detecting an extrasolar planet around a background star as it crosses the fold caustic of a foreground binary lens. During such an event, the planet's flux can be magnified by a factor of ~100 or more. The detectability of the planet depends strongly on the orientation of its orbit relative to the caustic. If the source star is inside the intercaustic region, detecting the caustic-crossing planet is difficult against the magnified flux of its parent star. In the more favorable configuration, when the star is outside the intercaustic region when the planet crosses the caustic, a close-in Jupiter-like planet around a Sun-like star at a distance of 8 kpc is detectable in 8 minute integrations with a 10 m telescope at maximal signal-to-noise ratio (S/N) ~15 for phase angle φ ~ 10°. In this example, we find further that the presence of methane, at its measured abundance in Jupiter, and/or water, sodium, and potassium, at the abundances expected in theoretical atmosphere models of close-in Jupiters, could be inferred from a nondetection of the planet in strong broad absorption bands at 0.6-1.4 μm caused by these compounds,

accompanied by a S/N ~ 10 detection in adjacent bands. We conclude that future generations of large telescopes might be able to probe the composition of the atmospheres of distant extrasolar planets. © 2005. The American Astronomical Society. All rights reserved.
KW - Astrobiology
KW - Astrochemistry
KW - Gravitational lensing
KW - Planetary systems
KW - Stars: atmospheres
KW - Stars: individual (HD 209458)
PB - Institute of Physics Publishing
SN - 0004637X (ISSN)
LA - English
J2 - Astrophys. J.
M3 - Article
DB - Scopus
N1 - Export Date: 23 February 2023; Cited By: 3; Correspondence Address: D.S. Spiegel; Department of Astronomy, Columbia University, New York, NY 10027, 550 West 120th Street, United States; email: dave@astro.columbia.edu
ER -

TY - JOUR
AU - Córdoba-Jabonero, C.
AU - Zorzano, M.-P.
AU - Selsis, F.
AU - Patel, M.R.
AU - Cockell, C.S.
TI - Radiative habitable zones in martian polar environments
PY - 2005
T2 - Icarus
VL - 175
IS - 2
SP - 360
EP - 371
DO - 10.1016/j.icarus.2004.12.009
UR - https://www.scopus.com/inward/record.uri?eid=2-s2.0-18144421536&doi=10.1016%2fj.icarus.2004.12.009&partnerID=40&md5=2bf04ee6a1cf82dadc31ab1b797883d8
AD - Instituto Nacional de Técnica Aeroespacial, Área de Investigación e Instrumentación Atmosférica, 28850 Madrid, Torrejón de Ardoz, Spain
AD - Centro de Astrobiología, CSIC-INTA, 28850 Madrid, Ctra. Ajalvir km. 4, Torrejon Ardoz, Spain
AD - Centre de Recherche Astronomique de Lyon, Ecole normale Supérieure, F-69364 Lyon Cedex 7, 47 Allée d'Italie, France
AD - Planetary and Space Sciences Research Institute, The Open University, Milton Keynes MK7 6AA, Walton Hall, United Kingdom
AD - British Antarctic Survey, Cambridge CB3 0ET, High Cross, Madingley Road, United Kingdom
AB - The biologically damaging solar ultraviolet (UV) radiation (quantified by the DNA-weighted dose) reaches the martian surface in extremely high levels. Searching for potentially habitable UV-protected environments on Mars, we considered the polar ice caps

that consist of a seasonally varying CO2 ice cover and a permanent H2O ice layer. It was found that, though the CO2 ice is insufficient by itself to screen the UV radiation, at ~1 m depth within the perennial H2O ice the DNA-weighted dose is reduced to terrestrial levels. This depth depends strongly on the optical properties of the H2O ice layers (for instance snow-like layers). The Earth-like DNA-weighted dose and Photosynthetically Active Radiation (PAR) requirements were used to define the upper and lower limits of the northern and southern polar Radiative Habitable Zone (RHZ) for which a temporal and spatial mapping was performed. Based on these studies we conclude that photosynthetic life might be possible within the ice layers of the polar regions. The thickness varies along each martian polar spring and summer between ~1.5 and 2.4 m for H2O ice-like layers, and a few centimeters for snow-like covers. These martian Earth-like radiative habitable environments may be primary targets for future martian astrobiological missions. Special attention should be paid to planetary protection, since the polar RHZ may also be subject to terrestrial contamination by probes. © 2004 Elsevier Inc. All rights reserved.
KW - Arctic Regions
KW - Carbon Dioxide
KW - Dose-Response Relationship, Radiation
KW - Environment
KW - Environmental Exposure
KW - Exobiology
KW - Ice
KW - Mars
KW - Photosynthesis
KW - Radiation Dosage
KW - Seasons
KW - Sunlight
KW - Ultraviolet Rays
KW - Water
KW - carbon dioxide
KW - ice
KW - water
KW - Arctic
KW - article
KW - astronomy
KW - biology
KW - chemistry
KW - environment
KW - environmental exposure
KW - photosynthesis
KW - radiation dose
KW - radiation exposure
KW - radiation response
KW - season
KW - sunlight
KW - ultraviolet radiation
KW - Exobiology
KW - Ices
KW - Mars, surface
KW - Radiative transfer

KW - Solar radiation
KW - Terrestrial planets
SN - 00191035 (ISSN)
C2 - 16044598
LA - English
J2 - Icarus
M3 - Article
DB - Scopus
N1 - Export Date: 23 February 2023; Cited By: 20; Correspondence Address: C. Córdoba-Jabonero; Instituto Nacional de Técnica Aeroespacial, Área de Investigación e Instrumentación Atmosférica, 28850 Madrid, Torrejón de Ardoz, Spain; email: cordobajc@inta.es; CODEN: ICRSA
ER -

TY - CONF
TI - Proceedings of SPIE - Astrobiology and Planetary Missions
PY - 2005
T2 - Proceedings of SPIE - The International Society for Optical Engineering
VL - 5906
UR - https://www.scopus.com/inward/record.uri?eid=2-s2.0-29844453526&partnerID=40&md5=6f12c07b70ea7fe889f17fce094c972b
AB - The proceedings contain 44 papers. The topics discussed include: minimal ecosystems: spirochetes populations as an example of functional stability; morphology and elemental composition of recent and fossil cyanobacteria; fossil microorganisms in Achaean deposits of Northern Karelia; microbiological methodology in astrobiology; evidence for liquid water on comets; the search for life in the universe; mineralized remains of morphotypes of filamentous cyanobacteria in carbonaceous meteorites; geobiological events in the Precambrian era; the use of ultraviolet excitation of native fluorescence for identifying biomarkers in halite crystals; how desert varnish forms?; and radiometric performance results of the New Horizons' ALICE UV Imaging spectrograph.
KW - Astronomy
KW - Bacteria
KW - Biology
KW - Biomarkers
KW - Ecosystems
KW - Imaging techniques
KW - Microbiology
KW - Microorganisms
KW - Morphology
KW - Ultraviolet radiation
KW - Comets
KW - Fossil microorganisms
KW - Geobiological events
KW - Imaging spectrographs
KW - Technical presentations
SN - 0277786X (ISSN)
LA - English
J2 - Proc SPIE Int Soc Opt Eng
M3 - Conference review

DB - Scopus
N1 - Export Date: 23 February 2023; Cited By: 0; CODEN: PSISD
ER -

TY - CONF
AU - Storrie-Lombardi, M.C.
TI - Post-bayesian strategies to optimize astrobiology instrument suites: Lessons from Antarctica and the Pilbara
PY - 2005
T2 - Proceedings of SPIE - The International Society for Optical Engineering
VL - 5906
C7 - 59060Y
SP - 1
EP - 14
DO - 10.1117/12.618279
UR - https://www.scopus.com/inward/record.uri?eid=2-s2.0-29844438664&doi=10.1117%2f12.618279&partnerID=40&md5=afbca11b0f19d6899a2bec9dfc820bbc
AD - Kinohi Institute, Inc., Pasadena, CA 91101, 530 S. Lake Avenue, United States
AB - Artificial neural networks patterned on fundamental neurological features of the human perceptual system have been shown to produce Bayesian probabilistic classifications of galaxies1-3, identify biotic and abiotic alteration of subsurface basalts4, distinguish terrestrial fossils from their background rock matrix5, and detect areas of Archean hydrothermal alteration6. Data inputs for these classification tasks have varied from astronomical or high altitude images and spectra, to sub-micron resolution elemental abundances. However, Bayesian theory assumes an absence of statistical and interpretive ambiguity in a target signal, the antithesis of the problems facing remote and human exploration of extreme environments on Earth and extraterrestrial sites such as Mars, comets, and the icy moons of Jupiter and Saturn. Fundamental to our certainty about the classification of geobiological targets on Earth is a long scientific history of familiarization both with the geochemical evolution of our planet and the reliability and discriminating power of particular instruments. Reduction of the uncertainty associated with a putative extraterrestrial biosignature derived from a single probe is most often attempted by deploying a suite of instruments, each one interrogating distinct morphological and chemical phenomena in a target 7. But understanding the relative weighting appropriate for merging disparate signals or distinct data sets is not a trivial issue8. And, as we have most recently seen in the case of ALH84001, strategies relying on the cumulative statistical power of multiple probes often crumble when subsequent review of abiotic physicochemical phenomena reveals even a single abiotic mechanism, no matter how improbable, capable of replicating the putative biotic signal. Finally, for extend extraterrestrial missions or work in remote environments on Earth, the fundamental "fewest moving parts" reliability rule must come into play. This communication highlights the minimum requirements for an astrobiological instrument suite for remote or human exploration of extreme environments both here on Earth and in our local and

neighboring planetary systems. Critical items of concern include obtaining co-registered data characterizing target morphology, metabolism, and mobility; the face validity and familiarity of the instrumentation to the scientific community, and the choice of instrumentation sufficiently inexpensive and easy to use that it might find wide spread usage within the astrobiology community prior to mission deployment. Preliminary indications are that such an instrument can be implemented for a cost accessible to high school, college, and graduate students interested in geobiological and astrobiological research in extreme or hazardous environments.
KW - Biochemistry
KW - Earth (planet)
KW - Exploratory geochemistry
KW - Extraterrestrial communication links
KW - Hazardous materials
KW - Metabolism
KW - Morphology
KW - Neural networks
KW - Neurology
KW - Antarctica
KW - Astrobiology Missions
KW - Bayesian classification
KW - Extreme Environments
KW - INstrument Selection
KW - Pilbara
KW - Space probes
KW - Antarctica
KW - Astrobiology Missions
KW - Bayesian classification
KW - Extreme Environments
KW - INstrument Selection
KW - Pilbara
SN - 0277786X (ISSN)
LA - English
J2 - Proc SPIE Int Soc Opt Eng
M3 - Conference paper
DB - Scopus
N1 - Export Date: 23 February 2023; Cited By: 9; Correspondence Address: M.C. Storrie-Lombardi; Kinohi Institute, Inc., Pasadena, CA 91101, 530 S. Lake Avenue, United States; email: mike@kinohi.org; CODEN: PSISD
ER -

TY - JOUR
AU - Fink, W.
AU - Dohm, J.M.
AU - Tarbell, M.A.
AU - Hare, T.M.
AU - Baker, V.R.
TI - Next-generation robotic planetary reconnaissance missions: A paradigm shift
PY - 2005
T2 - Planetary and Space Science
VL - 53

IS - 14-15
SP - 1419
EP - 1426
DO - 10.1016/j.pss.2005.07.013
UR - https://www.scopus.com/inward/record.uri?eid=2-s2.0-28844504246&doi=10.1016%2fj.pss.2005.07.013&partnerID=40&md5=726b90c72b39580a7d34ddb9f9514281
AD - Visual and Autonomous Exploration Systems Research Lab., Division of Physics, Mathematics and Astronomy, California Institute of Technology, Pasadena, CA, United States
AD - Department of Hydrology and Water Resources, University of Arizona, Tucson, AZ, United States
AD - United States Geologic Survey, Flagstaff, AZ, United States
AB - A fundamentally new scientific mission concept for remote planetary surface and subsurface reconnaissance will soon replace the engineering and safety constrained mission designs of the past, allowing for optimal acquisition of geologic, paleohydrologic, paleoclimatic, and possible astrobiologic information of Mars and other extraterrestrial targets. Traditional missions have performed local ground-level reconnaissance through rovers and immobile landers, or global mapping performed by an orbiter. The former is safety and engineering constrained, affording limited detailed reconnaissance of a single site at the expense of a regional understanding, while the latter returns immense datasets, often overlooking detailed information of local and regional significance. A "tier-scalable" paradigm integrates multi-tier (orbit⇔atmosphere⇔ground) and multi-agent (orbiter⇔blimps⇔rovers/sensorwebs) hierarchical mission architectures, not only introducing mission redundancy and safety, but enabling and optimizing intelligent, unconstrained, and distributed science-driven exploration of prime locations on Mars and elsewhere, allowing for increased science return, and paving the way towards fully autonomous robotic missions. © 2005 Elsevier Ltd. All rights reserved.
KW - Astronomy
KW - Geology
KW - Hydrology
KW - Redundancy
KW - Robotics
KW - Astrobiology
KW - Mission redundancy and safety
KW - Multi-agents
KW - Multi-tier exploration
KW - Robotic planetary exploration
KW - Tier-scalable mission autonomy
KW - Martian surface analysis
KW - Astrobiology
KW - Mission redundancy and safety
KW - Multi-agents
KW - Multi-tier exploration
KW - Robotic planetary exploration
KW - Tier-scalable mission autonomy
SN - 00320633 (ISSN)
LA - English

J2 - Planet. Space Sci.
M3 - Article
DB - Scopus
N1 - Export Date: 23 February 2023; Cited By: 79; Correspondence Address: W. Fink; Visual and Autonomous Exploration Systems Research Lab., Division of Physics, Mathematics and Astronomy, California Institute of Technology, Pasadena, CA, United States; email: wfink@autonomy.caltech.edu; CODEN: PLSSA
ER -

TY - CONF
AU - Ehlmann, B.L.
AU - Chowdhury, J.
AU - Marzullo, T.C.
AU - Eric Collins, R.
AU - Litzenberger, J.
AU - Ibsen, S.
AU - Krauser, W.R.
AU - Dekock, B.
AU - Hannon, M.
AU - Kinnevan, J.
AU - Shepard, R.
AU - Douglas Grant, F.
TI - Humans to Mars: A feasibility and cost-benefit analysis
PY - 2005
T2 - Acta Astronautica
VL - 56
IS - 9-12
SP - 851
EP - 858
DO - 10.1016/j.actaastro.2005.01.010
UR - https://www.scopus.com/inward/record.uri?eid=2-s2.0-20144388570&doi=10.1016%2fj.actaastro.2005.01.010&partnerID=40&md5=1796bef07e923b9b867c1946dc40f168
AD - Dept. of Earth and Planet. Sciences, Washington University, St. Louis, MO 63130, United States
AD - School of Medicine, University of Alberta, Canada
AD - Neuroscience Program, University of Michigan, United States
AD - School of Oceanography, University of Washington, United States
AD - Department of Mechanical Engineering, Biomedical Engineering Division, Stanford University, United States
AD - Department of Zoology, University of Hawaii, Manoa, United States
AD - NASA Ames Research Center, Moffett Field, CA, United States
AD - Department of Mechanical Engineering, University of Oklahoma, United States
AD - Department of Mechanical Engineering, University of Notre Dame, United States
AD - Department of Electrical Engineering, University of New Hampshire, United States
AD - Department of Geology, University of California, Davis, United States
AD - Department of Geological Sciences, Arizona State University,

United States
AD - Campus Box 1169, St. Louis, MO 63130, 1 Brookings Drive, United States
AB - Mars is a compelling astrobiological target, and a human mission would provide an opportunity to collect immense amounts of scientific data. Exploration alone, however, cannot justify the increased risk. Instead, three factors drive a human mission: economics, education, and exploration. A human mission has a unique potential to inspire the next generation of young people to enter critically needed science and engineering disciplines. A mission is economically feasible, and the research and development program put in place for a human mission would propel growth in related high-technology industries. The main hurdles are human physiological responses to 1-2 years of radiation and microgravity exposure. However, enabling technologies are sufficiently mature in these areas that they can be developed within a few decade timescale. Hence, the decision of whether or not to undertake a human mission to Mars is a political decision, and thus, educational and economic benefits are the crucial factors. © 2005 Elsevier Ltd. All rights reserved.
KW - Aerospace Medicine
KW - Containment of Biohazards
KW - Cost-Benefit Analysis
KW - Feasibility Studies
KW - Humans
KW - International Cooperation
KW - Mars
KW - Radiation Protection
KW - Space Flight
KW - Spacecraft
KW - United States
KW - United States National Aeronautics and Space Administration
KW - Water
KW - Weightlessness
KW - Cost benefit analysis
KW - Decision making
KW - Economic and social effects
KW - Manned space flight
KW - Microgravity processing
KW - Physiology
KW - Solar system
KW - Space research
KW - water
KW - aerospace medicine
KW - article
KW - astronomy
KW - cost benefit analysis
KW - economics
KW - education
KW - feasibility study
KW - government
KW - hazardous waste
KW - human
KW - international cooperation

KW - radiation protection
KW - space flight
KW - United States
KW - weightlessness
KW - Feasibility
KW - Human
KW - Mars
KW - Mission
KW - Political
KW - Martian surface analysis
KW - Feasibility
KW - Human
KW - Mars
KW - Mission
KW - Political
KW - Technical
SN - 00945765 (ISSN)
C2 - 15835029
LA - English
J2 - Acta Astronaut
M3 - Conference paper
DB - Scopus
N1 - Export Date: 23 February 2023; Cited By: 19; Correspondence Address: B.L. Ehlmann; Campus Box 1169, St. Louis, MO 63130, 1 Brookings Drive, United States; email: bethany.ehlmann@wustl.edu; CODEN: AASTC
ER -

TY - JOUR
AU - Sever, M.
TI - Making a public impact
PY - 2005
T2 - Geotimes
VL - 50
IS - 7
SP - 42
EP - 43
UR - https://www.scopus.com/inward/record.uri?eid=2-s2.0-23144468095&partnerID=40&md5=2db21caec3bfd0bdafb57e9d597e34de
AB - As the Deep Impact spacecraft nears its intended target, comet Tempel 1, this month, people around the world are climbing atop their roofs equipped with high- and low-tech telescopes to see if they, too, can witness the comet collision. What they observe or, better yet, capture on film both during the event and in the weeks before and after, could prove useful to the team of NASA scientists trying to understand the comet and the effects of the impact. As the world becomes more interconnected by the Internet, members of the public are increasingly helping scientists with their research - through personal observations of everything from birds and weather to the stars, as well as by allowing scientists use of their home computers for projects ranging from searching for extraterrestrial life (SETIHome) and gravity waves (EinsteinHome), to predicting the changing global climate. These new partnerships not only help the scientists, but also keep the public interested and involved in

current scientific research.
SN - 00168556 (ISSN)
LA - English
J2 - Geotimes
M3 - Article
DB - Scopus
N1 - Export Date: 23 February 2023; Cited By: 1
ER -

TY - JOUR
AU - Methé, B.A.
AU - Nelson, K.E.
AU - Deming, J.W.
AU - Momen, B.
AU - Melamud, E.
AU - Zhang, X.
AU - Moult, J.
AU - Madupu, R.
AU - Nelson, W.C.
AU - Dodson, R.J.
AU - Brinkac, L.M.
AU - Daugherty, S.C.
AU - Durkin, A.S.
AU - DeBoy, R.T.
AU - Kolonay, J.F.
AU - Sullivan, S.A.
AU - Zhou, L.
AU - Davidsen, T.M.
AU - Wu, M.
AU - Huston, A.L.
AU - Lewis, M.
AU - Weaver, B.
AU - Weidman, J.F.
AU - Khouri, H.
AU - Utterback, T.R.
AU - Feldblyum, T.V.
AU - Fraser, C.M.
TI - The psychrophilic lifestyle as revealed by the genome sequence of Colwellia psychrerythraea 34H through genomic and proteomic analyses
PY - 2005
T2 - Proceedings of the National Academy of Sciences of the United States of America
VL - 102
IS - 31
SP - 10913
EP - 10918
DO - 10.1073/pnas.0504766102
UR - https://www.scopus.com/inward/record.uri?eid=2-s2.0-23344439437&doi=10.1073%2fpnas.0504766102&partnerID=40&md5=e9b8a58bbc859a53e8cfd96f71ce992b
AD - Institute for Genomic Research, Rockville, MD 20850, 9712 Medical Center Drive, United States
AD - School of Oceanography and Astrobiology Program, University of

Washington, Seattle, WA 98195, United States
AD - Department of Natural Resource Sciences and Landscape Architecture, University of Maryland, 1108 H. J. Patterson Hall, College Park, MD 20742, United States
AD - Center for Advanced Research in Biotechnology, Biotechnology Institute, University of Maryland, Rockville, MD 20850, 9600 Gudelsky Drive, United States
AD - Department of Biochemistry and Molecular Biology, Pennsylvania State University, University Park, PA 16802, United States
AD - School of Oceanography, Box 357940, University of Washington, Seattle, WA 98195, United States
AB - The completion of the 5,373,180-bp genome sequence of the marine psychrophilic bacterium Colwellia psychrerythraea 34H, a model for the study of life in permanently cold environments, reveals capabilities important to carbon and nutrient cycling, bioremediation, production of secondary metabolites, and cold-adapted enzymes. From a genomic perspective, cold adaptation is suggested in several broad categories involving changes to the cell membrane fluidity, uptake and synthesis of compounds conferring cryotolerance, and strategies to overcome temperature-dependent barriers to carbon uptake. Modeling of three-dimensional protein homology from bacteria representing a range of optimal growth temperatures suggests changes to proteome composition that may enhance enzyme effectiveness at low temperatures. Comparative genome analyses suggest that the psychrophilic lifestyle is most likely conferred not by a unique set of genes but by a collection of synergistic changes in overall genome content and amino acid composition. © 2005 by The National Academy of Sciences of the USA.
KW - Amino Acids
KW - Bacterial Proteins
KW - Carbon
KW - Cold Climate
KW - DNA, Bacterial
KW - Energy Metabolism
KW - Gammaproteobacteria
KW - Genome, Bacterial
KW - Genomics
KW - Marine Biology
KW - Membrane Fluidity
KW - Models, Biological
KW - Molecular Sequence Data
KW - Nitrogen
KW - Proteomics
KW - Species Specificity
KW - Colwellia psychrerythraea 34H
KW - amino acid analysis
KW - article
KW - bacterial genome
KW - bioremediation
KW - carbon cycling
KW - cold acclimatization
KW - Colwellia sychrerythraea
KW - comparative gene mapping
KW - cryoprecipitation

KW - environmental factor
KW - gene sequence
KW - low temperature procedures
KW - membrane fluidity
KW - nonhuman
KW - nutrient cycling
KW - priority journal
KW - proteomics
KW - temperature dependence
KW - three dimensional imaging
KW - Astrobiology
KW - Bioremediation
KW - Proteome
KW - Psychrophily
KW - Three-dimensional homology modeling
SN - 00278424 (ISSN)
C2 - 16043709
LA - English
J2 - Proc. Natl. Acad. Sci. U. S. A.
M3 - Article
DB - Scopus
N1 - Export Date: 23 February 2023; Cited By: 408; Correspondence Address: B.A. Methé; Institute for Genomic Research, Rockville, MD 20850, 9712 Medical Center Drive, United States; email: bmethe@tigr.org; CODEN: PNASA
ER -

TY - JOUR
AU - Tang, B.L.
TI - Many possibilities for life's emergence
PY - 2005
T2 - JBIS - Journal British Interplanetary Society
VL - 58
IS - 7-8
SP - 218
EP - 222
UR - https://www.scopus.com/inward/record.uri?eid=2-s2.0-20744434264&partnerID=40&md5=8d48b18a64909f68b26091a5e62af1aa
AD - Department of Biochemistry, National University of Singapore, Singapore 117597, 8 Medical Drive, Singapore
AB - It was recently cautioned that the discoveries of extremophiles, multiple locations within the solar system where these might flourish and the growing number of extrasolar planets might be irrelevant to the origin of life (Cleaves II and Chalmers, 2004, Astrobiology, 4:1-9). Central to this argument is the notion that emergence of life may require specific environmental and geological conditions that are much less common than the mere existence of liquid water. Although logical, the notion ignores the following relevant possibilities: 1) Life on Earth may have emerged very quickly and efficiently, 2) There may be several routes to life's emergence on a number of terrestrial geological niches, 3) Some extraterrestrial environments bear good resemblances to terrestrial geological niches capable of hosting the emergence of life and 4) There are exotic, but not inconceivable,

extraterrestrial geological niches and geochemical processes that could drive the emergence of life with terrestrial-like biochemistry. Thus, the robustness of life manifested by the widespread existence of extremophiles is not merely a testimony of how well life adapts to different environments, but may also reflect the myriad of possibilities whereby it may emerged from abiotic geochemistry.
KW - Biochemistry
KW - Earth (planet)
KW - Extraterrestrial atmospheres
KW - Geochemistry
KW - Geology
KW - Planets
KW - Satellites
KW - Europa
KW - Extremophiles
KW - FeS
KW - Habitable zone
KW - Mars
KW - Titan
KW - Space research
KW - Europa
KW - Extremophiles
KW - FeS
KW - Habitable zone
KW - Mars
KW - Titan
SN - 0007084X (ISSN)
LA - English
J2 - JBIS J. Br. Interplanet. Soc.
M3 - Article
DB - Scopus
N1 - Export Date: 23 February 2023; Cited By: 2; Correspondence Address: B.L. Tang; Department of Biochemistry, National University of Singapore, Singapore 117597, 8 Medical Drive, Singapore; email: bchtbl@nus.edu.sg
ER -

TY - JOUR
AU - Squyres, S.W.
AU - Knoll, A.H.
TI - Sedimentary rocks at Meridiani Planum: Origin, diagenesis, and implications for life on Mars
PY - 2005
T2 - Earth and Planetary Science Letters
VL - 240
IS - 1
SP - 1
EP - 10
DO - 10.1016/j.epsl.2005.09.038
UR - https://www.scopus.com/inward/record.uri?eid=2-s2.0-27944474775&doi=10.1016%2fj.epsl.2005.09.038&partnerID=40&md5=ae2f6ddf2d64c8e919ae3360b836632b
AD - Department of Astronomy, Cornell University, Ithaca, NY 14853,

Space Sciences Building, United States
AD - Botanical Museum, Harvard University, Cambridge, MA 02138, United States
AB - The MER rover Opportunity has carried out the first outcrop-scale investigation of ancient sedimentary rocks on Mars. The rocks, exposed in craters and along fissures in Meridiani Planum, are sandstones formed via the erosion and re-deposition of fine grained siliciclastics and evaporites derived from the chemical weathering of olivine basalts by acidic waters. A stratigraphic section more than seven meters thick measured in Endurance crater is dominated by eolian dune and sand sheet facies; the uppermost half meter, however, exhibits festoon cross lamination at a length scale that indicates subaqueous deposition, likely in a playa-like interdune setting. Silicates and sulfate minerals dominate outcrop geochemistry, but hematite and Fe3D3 (another ferric iron phase) make up as much as 11% of the rocks by weight. Jarosite in the outcrop matrix indicates precipitation at low pH. Cements, hematitic concretions, and crystal molds attest to a complex history of early diagenesis, mediated by ambient ground waters. The depositional and early diagenetic paleoenvironment at Meridiani was arid, acidic, and oxidizing, a characterization that places strong constraints on astrobiologial inference. © 2005 Published by Elsevier B.V.
KW - Emmelichthys struhsakeri
KW - Biology
KW - Deposition
KW - Erosion
KW - Geochemistry
KW - Lithology
KW - Martian surface analysis
KW - Petrology
KW - Sandstone
KW - Sedimentology
KW - Stratigraphy
KW - Astrobiology
KW - Outcrop geochemistry
KW - Redeposition
KW - basalt
KW - Mars
KW - planetary surface
KW - sedimentology
KW - weathering
KW - Sedimentary rocks
KW - Astrobiology
KW - Mars
KW - Meridiani
KW - Sedimentology
SN - 0012821X (ISSN)
LA - English
J2 - Earth Plan. Sci. Lett.
M3 - Article
DB - Scopus
N1 - Export Date: 23 February 2023; Cited By: 252; Correspondence Address: S.W. Squyres; Department of Astronomy, Cornell University, Ithaca, NY 14853, Space Sciences Building, United States; email:

squyres@astro.cornell.edu
ER -

TY - JOUR
AU - Pierazzo, E.
AU - Artemieva, N.A.
AU - Ivanov, B.A.
TI - Starting conditions for hydrothermal systems underneath Martian craters: Hydrocode modeling
PY - 2005
T2 - Special Paper of the Geological Society of America
VL - 384
SP - 443
EP - 457
DO - 10.1130/0-8137-2384-1.443
UR - https://www.scopus.com/inward/record.uri?eid=2-s2.0-74049131057&doi=10.1130%2f0-8137-2384-1.443&partnerID=40&md5=baeec5bc1f0a542d24b638ba4da9616f
AD - Planetary Science Institute, Tucson, AZ 85719, 1700 E. Ft. Lowell Road, United States
AD - Institute for Dynamics of Geospheres, Russian Academy of Sciences, 117334, Moscow, Leninsky pr., 38-6, Russian Federation
AB - Mars is the first place to look for any sign of present or past extraterrestrial life. Its surface shows many features indicative of the presence of surface and subsurface water, while impact cratering and volcanism have provided temporary and local surface heat sources throughout Mars' geologic history. In particular, impact-generated hydrothermal systems could have been some of the most favorable sites for the origin of life on Mars. We present preliminary results of hydrocode simulations of impacts on Mars aimed at constraining the initial conditions for modeling the onset and evolution of a hydrothermal system on the red planet. Simulations of the early stages of impact cratering can identify the role of target composition and inhomogeneities in characterizing shock melting due to asteroidal and cometary impacts on the Martiansurface. We find that cometary impacts produce signifi cantly larger melt volumes than asteroidal impacts, thus affecting the thickness of the resulting melt sheet and its cooling history. The description of mixed targets is the main challenge of this study: unfortunately at this time, the lack of specifi c experimental data does not allow us to identify what is the best modeling approach (mixed cells versus mixed material equation of state) for mixed material targets. Modeling crater collapse is a necessary step to determine the final thermal state of the target underneath. We find that the combination of shock and plastic heating and the structural uplift of initially deeper strata create a water-bearing zone at depths where water is in the liquid stability field. In the central uplift, the high temperatures cause water to evaporate (steam-driven circulation). The simulations show that for a mid-sized crater (rim diameter around 30 km) the hydrothermal circulation is probably restricted to a "column" contained well within the final crater. © 2005 Geological Society of America.
KW - Computer simulation
KW - Equations of state

KW - Geomorphology
KW - Structural geology
KW - Extraterrestrial life
KW - Hydrocode simulation
KW - Hydrothermal circulation
KW - Hydrothermal system
KW - Impact craters
KW - Mars
KW - Melt production
KW - Target composition
KW - asteroid
KW - crater
KW - cratering
KW - evaporation
KW - heat source
KW - hydrothermal system
KW - inhomogeneity
KW - Mars
KW - numerical model
KW - uplift
KW - volcanism
KW - Meteor impacts
KW - Hydrothermal systems
KW - Impact craters
KW - Mars
KW - Melt production
KW - Numerical simulations
PB - Geological Society of America
SN - 00721077 (ISSN)
LA - English
J2 - Spec. Pap. Geol. Soc. Am.
M3 - Article
DB - Scopus
N1 - Export Date: 23 February 2023; Cited By: 87; Correspondence Address: E. Pierazzo; Planetary Science Institute, Tucson, AZ 85719, 1700 E. Ft. Lowell Road, United States; email: Pierazzo-betty@psi.edu
ER -

TY - JOUR
AU - Fernández-Remolar, D.C.
AU - Morris, R.V.
AU - Gruener, J.E.
AU - Amils, R.
AU - Knoll, A.H.
TI - The Río Tinto Basin, Spain: Mineralogy, sedimentary geobiology, and implications for interpretation of outcrop rocks at Meridiani Planum, Mars
PY - 2005
T2 - Earth and Planetary Science Letters
VL - 240
IS - 1
SP - 149
EP - 167

DO - 10.1016/j.epsl.2005.09.043
UR - https://www.scopus.com/inward/record.uri?eid=2-s2.0-27944449281&doi=10.1016%2fj.epsl.2005.09.043&partnerID=40&md5=03f92b138dd66fcb6079b4c9ea806659
AD - Centro de Astrobiología, INTA, CSIC, 28850 Torrejón de Ardoz, Ctra Ajalvir km. 4, Spain
AD - Astromaterials Research Office, NASA Johnson Space Center, Houston, TX 77058, United States
AD - Department of Organismic and Evolutionary Biology, Harvard University, Cambridge, MA 02138, 26 Oxford Street, United States
AD - Centro de Biología Molecular, CSIC, UAM, Cantoblanco 28049 Madrid, Spain
AB - Exploration by the NASA rover Opportunity has revealed sulfate- and hematite-rich sedimentary rocks exposed in craters and other surface features of Meridiani Planum, Mars. Modern, Holocene, and Plio-Pleistocene deposits of the Río Tinto, southwestern Spain, provide at least a partial environmental analog to Meridiani Planum rocks, facilitating our understanding of Meridiani mineral precipitation and diagenesis, while informing considerations of martian astrobiology. Oxidation, thought to be biologically mediated, of pyritic ore bodies by groundwaters in the source area of the Río Tinto generates headwaters enriched in sulfuric acid and ferric iron. Seasonal evaporation of river water drives precipitation of hydronium jarosite and schwertmannite, while (Mg,Al,Fe3+)-copiapite, coquimbite, gypsum, and other sulfate minerals precipitate nearby as efflorescences where locally variable source waters are brought to the surface by capillary action. During the wet season, hydrolysis of sulfate salts results in the precipitation of nanophase goethite. Holocene and Plio-Pleistocene terraces show increasing goethite crystallinity and then replacement of goethite with hematite through time. Hematite in Meridiani spherules also formed during diagenesis, although whether these replaced precursor goethite or precipitated directly from groundwaters is not known. The retention of jarosite and other soluble sulfate salts suggests that water limited the diagenesis of Meridiani rocks. Diverse prokaryotic and eukaryotic microorganisms inhabit acidic and seasonally dry Río Tinto environments. Organic matter does not persist in Río Tinto sediments, but biosignatures imparted to sedimentary rocks as macroscopic textures of coated microbial streamers, surface blisters formed by biogenic gas, and microfossils preserved as casts and molds in iron oxides help to shape strategies for astrobiological investigation of Meridiani outcrops. © 2005 Elsevier B.V. All rights reserved.
KW - Emmelichthys struhsakeri
KW - Eukaryota
KW - Prokaryota
KW - Evaporation
KW - Groundwater
KW - Hydrolysis
KW - Mineralogy
KW - Oxidation
KW - Precipitation (chemical)
KW - Sedimentary rocks
KW - Weathering

KW - Geobiology
KW - Hematite
KW - Martian astrobiology
KW - chemical weathering
KW - hematite
KW - Mars
KW - natural analog
KW - Martian surface analysis
KW - Chemical weathering
KW - Geobiology
KW - Hematite
KW - Mars
KW - Meridiani Planum
KW - Río Tinto basin
SN - 0012821X (ISSN)
LA - English
J2 - Earth Plan. Sci. Lett.
M3 - Article
DB - Scopus
N1 - Export Date: 23 February 2023; Cited By: 243; Correspondence Address: D.C. Fernández-Remolar; Centro de Astrobiología, INTA, CSIC, 28850 Torrejón de Ardoz, Ctra Ajalvir km. 4, Spain; email: fernandezrd@inta.es
ER -

TY - JOUR
AU - Huedo, E.
AU - Lepinette, A.
AU - Montero, R.S.
AU - Llorente, I.M.
AU - Vázquez, L.
TI - Development and execution of an impact cratering application on a computational Grid
PY - 2005
T2 - Scientific Programming
VL - 13
IS - 1
SP - 19
EP - 30
DO - 10.1155/2005/387521
UR - https://www.scopus.com/inward/record.uri?eid=2-s2.0-21144454598&doi=10.1155%2f2005%2f387521&partnerID=40&md5=f441288cda77a280382bf36ec0418570
AD - Laboratorio de Computación Avanzada, Simulación y Aplicaciones Telemáticas, Centro de Astrobiología (CSIC-INTA), 28850 Torrejón de Ardoz, Spain
AD - Departmento de Arquitectura de Computadores, y Autom Ática, Facultad de Informática, Universidad Complutense de Madrid, 28040 Madrid, Spain
AD - Departamento de Matemática Aplicada, Facultad de Informática, Universidad Complutense de Madrid, 28040 Madrid, Spain
AB - Impact cratering is an important geological process of special interest in Astrobiology. Its numerical simulation comprises the execution of a high number of tasks, since the search space of input

parameter values includes the projectile diameter, the water depth and the impactor velocity. Furthermore, the execution time of each task is not uniform because of the different numerical properties of each experimental configuration. Grid technology is a promising platform to execute this kind of applications, since it provides the end user with a performance much higher than that achievable on any single organization. However, the scheduling of each task on a Grid involves challenging issues due to the unpredictable and heterogeneous behavior of both the Grid and the numerical code. This paper evaluates the performance of a Grid infrastructure based on the Globus toolkit and the GridWay framework, which provides the adaptive and fault tolerance functionality required to harness Grid resources, in the simulation of the impact cratering process. The experiments have been performed on a testbed composed of resources shared by five sites interconnected by RedIRIS, the Spanish Research and Education Network. © 2005 - IOS Press and the authors. All rights reserved.
KW - Client server computer systems
KW - Computational complexity
KW - Computer simulation
KW - Impact testing
KW - Middleware
KW - Problem solving
KW - Projectiles
KW - Space research
KW - Computational grid
KW - Cratering
KW - Execution
KW - Impact cratering
KW - Geology
PB - Hindawi Limited
SN - 10589244 (ISSN)
LA - English
J2 - Sci Program
M3 - Article
DB - Scopus
N1 - Export Date: 23 February 2023; Cited By: 0; Correspondence Address: E. Huedo; Laboratorio de Computación Avanzada, Simulación y Aplicaciones Telemáticas, Centro de Astrobiología (CSIC-INTA), 28850 Torrejón de Ardoz, Spain; email: huedoce@inta.es; CODEN: SCIPE
ER -

TY - JOUR
AU - Bruhn, F.C.
AU - Carsey, F.D.
AU - Köhler, J.
AU - Mowlem, M.
AU - German, C.R.
AU - Behar, A.E.
TI - MEMS enablement and analysis of the miniature autonomous submersible explorer
PY - 2005
T2 - IEEE Journal of Oceanic Engineering
VL - 30

IS - 1
SP - 165
EP - 178
DO - 10.1109/JOE.2004.836420
UR - https://www.scopus.com/inward/record.uri?eid=2-s2.0-21344439772&doi=10.1109%2fJOE.2004.836420&partnerID=40&md5=428c466d247aace24c0d1dbe3b5c8228
AD - Department of Engineering Sciences, Ångström Space Technology Centre (ÅSTC), Uppsala University, SE-751 21 Uppsala, Sweden
AD - NASA, Jet Propulsion Laboratory (JPL), California Institute of Technology, Pasadena, CA 91109, United States
AD - Southampton Oceanography Centre, Southampton SO14 3ZH, United Kingdom
AB - The miniature autonomous submersible explorer (MASE) was designed as a vehicle for astrobiology science by Behar et al. [1]. This paper focuses on the MASE concept and extrapolates a future design based on microelectromechanical systems (MEMS), multifunctional microsystems (MMS), and three-dimensional multichip modules (3-D-MCM). Miniaturization of the electronics increases the payload volumes and power capabilities significantly and this is the main rationale for pursuing extreme miniaturization. The original MASE vehicle accommodated 1-2 instruments while the MEMS enhanced miniature autonomous submersible explorer (MEMSEMASE) accommodates up to six instruments. It is shown that the occupied area of the electronics components is reduced eight times, and the volume 25 times. The vehicle is shaped as a tube with 5 cm in diameter and 20 cm in length and can support 8 W continuously over 5 h. The maximum range is 25 km while the typical onboard instrumentation is conductivity, temperature, depth (CTD), and a high resolution camera. An optical fiber is used for bidirectional communication with the vessel. The goal of this enriched concept is to present an extremely miniaturized submersible design. The vehicle volume is defined to fit inside host vehicles with the goal of future deployment on Europa, oceans on Earth, and bore holes. The paper will focus on showing how electronics can be densely packed into micromachined silicon modules and how these can be designed and interconnected theoretically. © 2005 IEEE.
KW - Microelectromechanical devices
KW - Multichip modules
KW - Optical fibers
KW - Remotely operated vehicles
KW - Submersibles
KW - Microrobot
KW - Miniature autonomous submersible explorer
KW - Multifunctional microsystems
KW - Ocean engineering
KW - Autonomous underwater vehicle (AUV)
KW - Microelectromechanical systems (MEMS)
KW - Microrobot
KW - Optical fiber
SN - 03649059 (ISSN)
LA - English
J2 - IEEE J Oceanic Eng
M3 - Article

DB - Scopus
N1 - Export Date: 23 February 2023; Cited By: 5; Correspondence Address: F.C. Bruhn; Department of Engineering Sciences, Ångström Space Technology Centre (ÅSTC), Uppsala University, SE-751 21 Uppsala, Sweden; email: fredrik.bruhn@angstrom.uu.se; CODEN: IJOED
ER -

TY - CONF
AU - Schindhelm, E.
AU - Cash, W.
AU - Seager, S.
TI - Science simulations for the New Worlds Observer
PY - 2005
T2 - Proceedings of SPIE - The International Society for Optical Engineering
VL - 5905
C7 - 59051G
SP - 1
EP - 9
DO - 10.1117/12.617478
UR - https://www.scopus.com/inward/record.uri?eid=2-s2.0-29844450941&doi=10.1117%2f12.617478&partnerID=40&md5=4e63fded69f4b686e0d9339b166edfeb
AD - University of Colorado, United States
AD - Carnegie Institution of Washington, United States
AB - The New Worlds Observer will be a two spacecraft occultor designed to directly detect and study extrasolar terrestrial planets. An apodized occultor or pinhole creates an image of the planetary system in the focal plane far away, where a second telescope craft orbits to detect the light. In this study we simulate the expected signal of NWO to find the optimal configuration and specifications of the two craft. The efficiency of direct detection through photometric imaging depends strongly on occulter and telescope size, while preliminary studies on absorption biomarker detection and photometric variability measurements are summarized.
KW - Computer simulation
KW - Imaging techniques
KW - Optics
KW - Planets
KW - Space research
KW - Spacecraft
KW - Telescopes
KW - Apodized occultor
KW - Astrobiology
KW - Coronograph
KW - Extrasolar planets
KW - Observatories
KW - Astrobiology
KW - Coronograph
KW - Extrasolar planets
SN - 0277786X (ISSN)
LA - English
J2 - Proc SPIE Int Soc Opt Eng

M3 - Conference paper
DB - Scopus
N1 - Export Date: 23 February 2023; Cited By: 2; CODEN: PSISD
ER -

TY - JOUR
AU - Holtom, P.D.
AU - Bennett, C.J.
AU - Osamura, Y.
AU - Mason, N.J.
AU - Kaiser, R.I.
TI - A combined experimental and theoretical study on the formation of the amino acid glycine (NH2CH2COOH) and its isomer (CH 3NHCOOH) in extraterrestrial ICES
PY - 2005
T2 - Astrophysical Journal
VL - 626
IS - 2 I
SP - 940
EP - 952
DO - 10.1086/430106
UR - https://www.scopus.com/inward/record.uri?eid=2-s2.0-23844535776&doi=10.1086%2f430106&partnerID=40&md5=b6d36a1eb5d1388790d0ed2d3c1395a6
AD - Department of Physics and Astronomy, University College London, London WC1E 6BT, Gower Street, United Kingdom
AD - Department of Physics and Astronomy, Open University, Milton Keynes MK7 6AA, United Kingdom
AD - Department of Chemistry, University of Hawaii at Manoa, Honolulu, HI 96822, United States
AD - Department of Chemistry, Rikkyo University, Tokyo 171-8501, 3-34-1 Nishi-ikebukuro, Japan
AB - We have investigated the synthesis of the simplest amino acid, glycine, by Galactic cosmic-ray particles in extra-terrestrial ices. Laboratory experiments combined with electronic structure calculations showed that a methylamine molecule [CH3NH2(X1 A′)] can be dissociated through interaction with energetic electrons in the track of a cosmicray particle to form atomic hydrogen and the radicals CH 2NH2(X2/A′) and CH3NH(X 2A′). Hydrogen atoms with sufficient kinetic energy could overcome the entrance barrier to add to a carbon dioxide molecule [CO 2(X 1∑g+)], yielding a trans-hydroxycarbonyl radical, HOCO(X2A′). Neighboring radicals with the correct geometric orientation then recombine to form glycine, NH 2CH2COOH(X 1A), and also its isomer, CH 3NHCOOH(X 1A). These findings expose for the first time detailed reaction mechanisms of how the simplest amino acid glycine and its isomer can be synthesized via nonequilibrium chemistry in interstellar and cometary ices. Our results offer an important alternative to aqueous and photon-induced formation of amino acids in comets and in molecular clouds. These results also predict the existence of a hitherto undetected isomer of glycine in the interstellar medium, suggest that glycine should be observable on Saturn's moon Titan, and help to account for the synthesis of more complex amino acids in the Murchison and Orgueil meteorites. © 2005. The American

Astronomical Society. All rights reserved.
KW - Astrobiology
KW - Astrochemistry
KW - Cosmic rays
KW - ISM: General
KW - ISM: Molecules
KW - Molecular processes
PB - Institute of Physics Publishing
SN - 0004637X (ISSN)
LA - English
J2 - Astrophys. J.
M3 - Article
DB - Scopus
N1 - Export Date: 23 February 2023; Cited By: 180; Correspondence Address: R.I. Kaiser; Department of Physics and Astronomy, Open University, Milton Keynes MK7 6AA, United Kingdom; email: kaiser@gold.chem.hawaii.edu
ER -

TY - JOUR
AU - Lipps, J.H.
AU - Rieboldt, S.
TI - Habitats and taphonomy of Europa
PY - 2005
T2 - Icarus
VL - 177
IS - 2
SP - 515
EP - 527
DO - 10.1016/j.icarus.2005.04.010
UR - https://www.scopus.com/inward/record.uri?eid=2-s2.0-26444554025&doi=10.1016%2fj.icarus.2005.04.010&partnerID=40&md5=96066cdccb68cf39f9869f5c3100b950
AD - Department of Integrative Biology, University of California, Berkeley, CA 94720, United States
AD - Museum of Paleontology, University of California, Berkeley, CA 94720, United States
AB - Jupiter's moon Europa possesses an icy shell kilometers thick that may overlie a briny ocean. The inferred presence of water, tidal and volcanic energy, and nutrients suggests that Europa is potentially inhabited by some kind of life; indeed Europa is a primary target in the search for life in the Solar System although no evidence yet exists for any kind of life. The thickness of the icy crust would impose limits on life, but at least 15 broad kinds of habitats seem possible for Europa. They include several on the sea floor, at least 3 in the water column, and many in the ice itself. All of these habitats are in, or could be transported to, the icy shell where they could be exposed by geologic activity or impacts so they might be explored from the surface or orbit by future planetary missions. Taphonomic processes that transport, preserve, and expose habitats include buoyant ice removing bottom habitats and sediment to the underside of the ice, water currents depositing components of water column habitats on the ice bottom, cryovolcanoes depositing water on the surface, tidal pumping

bringing water column and ice habitats to the near-surface ice, and subice freezing and diapiric action incorporating water column and bottom ice habitats into the lower parts of the icy shell. The preserved habitats could be exposed at or near the surface of Europa chiefly in newly-formed ice, tilted or rotated ice blocks, ridge debris, surface deposits, fault scarps, the sides of domes and pits, and impact craters and ejecta. Future exploration of Europa for life must consider careful targeting of sites where habitats are most likely preserved or exist close to the surface. © 2005 Elsevier Inc. All rights reserved.
KW - Astrobiology
KW - Europa
KW - Exploration
KW - Habitats
KW - Taphonomy
SN - 00191035 (ISSN)
LA - English
J2 - Icarus
M3 - Article
DB - Scopus
N1 - Export Date: 23 February 2023; Cited By: 21; Correspondence Address: J.H. Lipps; Department of Integrative Biology, University of California, Berkeley, CA 94720, United States; email: jlipps@berkeley.edu; CODEN: ICRSA
ER -

TY - JOUR
AU - Ellery, A.
AU - Ball, A.J.
AU - Cockell, C.
AU - Dickensheets, D.
AU - Edwards, H.
AU - Kolb, C.
AU - Lammer, H.
AU - Patel, M.
AU - Richter, L.
TI - Vanguard - A European robotic astrobiology-focussed Mars sub-surface mission proposal
PY - 2005
T2 - Acta Astronautica
VL - 56
IS - 3
SP - 397
EP - 407
DO - 10.1016/j.actaastro.2004.05.069
UR - https://www.scopus.com/inward/record.uri?eid=2-s2.0-9944235431&doi=10.1016%2fj.actaastro.2004.05.069&partnerID=40&md5=7aef296b69396fd5700fde370a5e4ed3
AD - Astronautics and Space Systems Group, School of Engineering, Kingston University, Roehampton Vale, London SW15 3WD, Friars Avenue, United Kingdom
AD - Planet. and Space Sci. Res. Inst., Open University, Wallon Hall, Milton Keynes MK7 6AA, United Kingdom
AD - British Antarctic Survey, High Cross, Cambridge CB3 OET,

Madingley Road, United Kingdom
AD - Department of Electrical Engineering, Montana State University, 610 Cobleigh Hall, Bozeman, MT 59717-3780, United States
AD - Department of Chemical Sciences, University of Bradford, Bradford BD7 1DP, Richmond Road, United Kingdom
AD - Institut fur Weltraumforschung, Osterreichische Akad. der Wiss., A-8042 Graz, Schmeidlstrasse 6, Austria
AD - Institute of Space Simulation, DLR, Deutsches Zentrum F. Luft/ Raumfahrt, Linder Hohe, D-51147 Koln, Germany
AD - Surrey Space Centre, Sch. of Electron. and Physics Sci., University of Surrey, Guildford, Surrey GU27 XH, United Kingdom
AB - We present a new European Mars mission proposal to build on the UK-led Beagle2 Mars mission and continue its astrobiology-focussed investigation of Mars. The small surface element to be delivered to the Martian surface - Vanguard - is designed to be carried by a Mars Express-type spacecraft bus to Mars and adopts a similar entry, descent and landing system as Beagle2. The surface element comprises a triad of robotic devices - a lander, a micro-rover of the Sojourner class for surface mobility, and three ground-penetrating moles mounted onto the rover for sub-surface penetration to 5 m depth. The major onboard instruments on the rover include a Raman spectrometer/imager, a laser plasma spectrometer, an infrared spectrometer - these laser instruments provide the basis for in situ "remote" sensing of the sub-surface Martian environment within a powerful scientific package. The moles carry the instruments' sensor head array to the sub-surface. The moles are thus required to undergo a one-way trip down the boreholes without the need for recovery of moles or samples, eliminating much of the robotic complexity invoked by such operations. © 2004 Elsevier Ltd. All rights reserved.
KW - Equipment Design
KW - Exobiology
KW - Geology
KW - Mars
KW - Robotics
KW - Space Flight
KW - Spacecraft
KW - Spectrum Analysis, Raman
KW - Infrared spectrometers
KW - Laser produced plasmas
KW - Planets
KW - Robotics
KW - Spacecraft
KW - Students
KW - Ground-penetrating moles
KW - Raman spectrometers
KW - Robotic astrobiology
KW - Surface mobility
KW - article
KW - astronomy
KW - biology
KW - equipment design
KW - geology
KW - instrumentation

KW - Raman spectrometry
KW - robotics
KW - space flight
KW - Space flight
PB - Elsevier Ltd
SN - 00945765 (ISSN)
C2 - 15754476
LA - English
J2 - Acta Astronaut
M3 - Article
DB - Scopus
N1 - Export Date: 23 February 2023; Cited By: 5; Correspondence Address: A. Ellery; Surrey Space Centre, Sch. of Electron. and Physics Sci., University of Surrey, Guildford, Surrey GU27 XH, United Kingdom; email: a.ellery@eim.surrey.ac.uk; CODEN: AASTC
ER -

TY - CONF
AU - Sheldon, R.B.
AU - Hoover, R.B.
TI - Evidence for liquid water on comets
PY - 2005
T2 - Proceedings of SPIE - The International Society for Optical Engineering
VL - 5906
C7 - 59060E
SP - 1
EP - 19
DO - 10.1117/12.624092
UR - https://www.scopus.com/inward/record.uri?eid=2-s2.0-29844455477&doi=10.1117%2f12.624092&partnerID=40&md5=bbaeb74c5acdaf50d750e3b67bb283e3
AD - NASA/MSFC/NSSTC, Huntsville, AL, 320 Sparkman Dr., United States
AB - The electron-microscope analysis of the Orgeuil carbonaceous chondrite, thought to be the extinct core of a comet, shows many archaen microfossils adapted for both cold and hot liquid water environments. Since water is a prerequisite for life, its presence on a comet would have important implications for interplanetary cross-contamination of the planets as well as strongly impact the dynamics and evolution of a comet. Therefore we develop a wet comet model to explore the consequences of liquid water on Mars-crossing comets and hypothesize that all the periodic comets, such as P/Halley, P/Wild-2, and P/Borrelly show signs of significant liquid water processing. The wet comet model is shown to be compatible with observation, as well as provide significantly better explanations for well-known cometary anomalies. Finally, the model predicts that the results of both Rosetta and Deep Impact missions will deviate from expectations.
KW - Geysers
KW - Martian surface analysis
KW - Orbital laboratories
KW - Orbits
KW - Planets

KW - Tensile strength
KW - Water
KW - Astrobiology
KW - Comets
KW - Deep Impact
KW - Liquid water
KW - Rosetta
KW - Splitting
KW - Extraterrestrial atmospheres
KW - Astrobiology
KW - Comet
KW - Deep Impact
KW - Geysers
KW - Liquid water
KW - Orbital dynamics
KW - Rosetta
KW - Splitting
KW - Tensile strength
SN - 0277786X (ISSN)
LA - English
J2 - Proc SPIE Int Soc Opt Eng
M3 - Conference paper
DB - Scopus
N1 - Export Date: 23 February 2023; Cited By: 6; Correspondence Address: R.B. Sheldon; NASA/MSFC/NSSTC, Huntsville, AL, 320 Sparkman Dr., United States; email: Rob.Sheldon@msfc.nasa.gov; CODEN: PSISD
ER -

TY - JOUR
AU - Bergin, E.A.
AU - Melnick, G.J.
AU - Gerakines, P.A.
AU - Neufeld, D.A.
AU - Whittet, D.C.B.
TI - Spitzer observations of CO2 ice toward field stars in the taurus molecular cloud
PY - 2005
T2 - Astrophysical Journal
VL - 627
IS - 1 II
SP - L33
EP - L36
DO - 10.1086/431932
UR - https://www.scopus.com/inward/record.uri?eid=2-s2.0-24144444102&doi=10.1086%2f431932&partnerID=40&md5=468de2e5a08845a3b5cc350c9dbcf979
AD - Department of Astronomy, University of Michigan, 825 Dennison Building, Ann Arbor, MI 48109, 500 Church Street, United States
AD - Harvard-Smithsonian Center for Astrophysics, Cambridge, MA 02138, 60 Garden Street, United States
AD - Astro- and Solar-System Physics Program, Department of Physics, University of Alabama at Birmingham, Birmingham, AL 35294-1170, United States
AD - Department of Physics and Astronomy, Johns Hopkins University,

Baltimore, MD 21218, 3400 North Charles Street, United States
AD - Department of Physics, Applied Physics, and Astronomy, New York Center for Studies on the Origins of Life, Rensselaer Polytechnic Institute, Troy, NY 12180, 110 Eighth Street, United States
AB - We present the first Spitzer Infrared Spectrograph observations of the 15.2 μm bending mode of CO2 ice toward field stars behind a quiescent dark cloud. CO2 ice is detected toward two field stars (Elias 16 and Elias 3) and a single protostar (HL Tau) with an abundance of ~15%-20% relative to water ice. CO2 ice is not detected toward the source with lowest extinction in our sample, Tamura 17 (AV = 3.9 mag). A comparison of the Elias 16 spectrum with laboratory data demonstrates that the majority of CO2 ice is embedded in a polar, H2O-rich ice component, with ~15% of CO2 residing in an apolar, H 2O-poor mantle. This is the first detection of apolar CO2 toward a field star. We find that the CO2 extinction threshold is AV = 4 ±1 mag, comparable to the threshold for water ice, but significantly less than the threshold for CO ice, the likely precursor of CO2. Our results confirm that CO2 ice forms in tandem with H2O ice along quiescent lines of sight. This argues for CO 2 ice formation by means of a mechanism similar to that responsible for H2O ice formation, viz., simple catalytic reactions on grain surfaces. © 2005. The American Astronomical Society. All rights reserved.
KW - Astrobiology
KW - Astrochemistry
KW - ISM: lines and bands ISM: molecules
PB - Institute of Physics Publishing
SN - 0004637X (ISSN)
LA - English
J2 - Astrophys. J.
M3 - Article
DB - Scopus
N1 - Export Date: 23 February 2023; Cited By: 76; Correspondence Address: E.A. Bergin; Department of Astronomy, University of Michigan, 825 Dennison Building, Ann Arbor, MI 48109, 500 Church Street, United States; email: ebergin@umich.edu
ER -

TY - CONF
AU - Thompson, R.
AU - Francis, S.
AU - Olsen, R.
AU - Parsons, M.
AU - Coffman, R.
AU - Braun, R.
TI - Cadmus: A Europa lander concept
PY - 2005
T2 - IEEE Aerospace Conference Proceedings
VL - 2005
C7 - 1559314
SP - 209
EP - 220
DO - 10.1109/AERO.2005.1559314

UR - https://www.scopus.com/inward/record.uri?eid=2-s2.0-33751530317&doi=10.1109%2fAERO.2005.1559314&partnerID=40&md5=60bc1d9b538968f2d0a177b35660bdf3
AD - Georgia Institute of Technology, Atlanta, GA 30332, United States
AB - The Cadmus mission responds to the need for a Europa surface exploration mission in the 2021 time frame that complements and extends the science performed by the Jupiter Icy Moons Orbiter (JIMO) and Galileo spacecraft. Cadmus will help prepare for future subsurface and sample return missions. Europa is one of the most intriguing outer solar system planetary bodies due to the compelling evidence that an ocean of salty water exists approximately 20 km beneath the surface. This liquid water could make Europa a haven for life. The NASA Office of Space Science (OSS) has identified the search for life in the solar system and the resources necessary to support extraterrestrial life as an important area of study. The Cadmus mission will investigate the habitability of Europa from the surface to determine the likelihood that life exists on the moon. By studying the crustal dynamics of the moon, the Cadmus mission will assess the extent to which a flux of water and ice exists between the possible subsurface ocean and the surface. Cadmus will investigate the presence of nutrients and signs of energy resources in the crustal ice and will also assess the environmental suitability of the Europa environment to the evolution and sustainability of life. To reduce mission operations cost and complexity the mission architecture includes two landers that will land on the surface independently and utilize a high degree of autonomy. © 2005 IEEE.
KW - Approximation theory
KW - Costs
KW - Ice
KW - Planetary surface analysis
KW - Planets
KW - Solar system
KW - Crustal dynamics
KW - Environmental suitability
KW - Extraterrestrial life
KW - Mission operations
KW - Planetary landers
PB - IEEE Computer Society
SN - 1095323X (ISSN); 0780388704 (ISBN); 978-078038870-3 (ISBN)
LA - English
J2 - IEEE Aerosp. Conf. Proc.
M3 - Conference paper
DB - Scopus
N1 - Export Date: 23 February 2023; Cited By: 0; Correspondence Address: R. Thompson; Georgia Institute of Technology, Atlanta, GA 30332, United States; email: robert_thompson@ae.gatech.edu
ER -

TY - JOUR
AU - Naylor, L.A.
TI - The contributions of biogeomorphology to the emerging field of geobiology

PY - 2005
T2 - Palaeogeography, Palaeoclimatology, Palaeoecology
VL - 219
IS - 1-2
SP - 35
EP - 51
DO - 10.1016/j.palaeo.2004.10.013
UR - https://www.scopus.com/inward/record.uri?eid=2-s2.0-15744383679&doi=10.1016%2fj.palaeo.2004.10.013&partnerID=40&md5=aa502a6837cafe51af397f8279a9904e
AD - Sch. of Geogr. and the Environment, University of Oxford, Oxford. OX1 3TB, Mansfield Road, United Kingdom
AD - Tyndall Ctr. for Climate Change Res., University of East Anglia, Norwich, NR4 7TJ, United Kingdom
AD - Ecosystems Team, Science Group, Environment Agency, Wallingford, Oxon, OX10 8BD, Howberry Park, United Kingdom
AB - Biogeomorphology has developed into a well-established research field over the past 15 years, with studies examining a range of two-way interrelations between organisms and geomorphology in a variety of terrestrial and marine environments. This paper starts by defining the core biogeomorphological processes-bioerosion, bioprotection and bioconstruction. Particular emphasis is placed on the study of bioconstructional forms; providing a clear definition, examples of bioconstructions, and, crucially, examining important interactions between bioconstruction and bioerosion. Three key areas where biogeomorphological research can directly contribute to the emerging field of geobiology are identified: (1) the use of biogeomorphological approaches, combined with palaeoecological investigations and predictive modelling, in the growing field of carbon sequestration and climate change mitigation; (2) providing geomorphological expertise to support the emerging field of astrobiology and (3) the potential contributions of biogeomorphology to the blossoming field of bio-geoengineering. Some key research directions and challenges for future biogeomorphological research include: exploring how biogeomorphological studies can contribute to and benefit from collaborations with other fields of geobiology, and assisting in the development of a useful, effective interdisciplinary toolbox of methods to improve quantification of geobiological processes. Importantly, suggestions are made for potentially fruitful collaborations with geomicrobiologists, geochemists and palaeoecologists. © 2004 Elsevier B.V. All rights reserved.
KW - biology
KW - geology
KW - interdisciplinary approach
KW - Biocomplexity
KW - Bioconstruction
KW - Biogeomorphology
KW - Ecosystem engineers
KW - Geobiology
KW - Interdisciplinary approaches
PB - Elsevier B.V.
SN - 00310182 (ISSN)
LA - English

J2 - Palaeogeogr. Palaeoclimatol. Palaeoecol.
M3 - Article
DB - Scopus
N1 - Export Date: 23 February 2023; Cited By: 45; Correspondence Address: L.A. Naylor; Sch. of Geogr. and the Environment, University of Oxford, Oxford. OX1 3TB, Mansfield Road, United Kingdom; email: larissa.naylor@environment-agency.gov.uk; CODEN: PPPYA
ER -

TY - CONF
AU - Raulin, F.
TI - Exo-astrobiological aspects of Europa and Titan: From observations to speculations
PY - 2005
T2 - Space Science Reviews
VL - 116
IS - 1-2
SP - 471
EP - 487
DO - 10.1007/s11214-005-1967-x
UR - https://www.scopus.com/inward/record.uri?eid=2-s2.0-21044458136&doi=10.1007%2fs11214-005-1967-x&partnerID=40&md5=443d45627b7289b5b7ecefc790e2cc45
AD - Observatoire de Paris, Université Pierre et Marie Curie, Meudon, F-92195, France
AD - Laboratoire Interuniversitaire des Systèmes Atmosphériques, LISA-UMR CNRS 7583, Universités Paris 7 et Paris 12, F-94000 Créteil, 61 Ave. du General de Gaulle, France
AB - By extrapolating what we know on the origins of life on Earth, and in particular on the chemical processes which gave rise to the first living system, Europa and Titan appear as two major targets for studies of exo/astrobiology in the outer solar system. With the likely presence of water oceans relatively close to its surface, coupled to possible sources of organics, the emergence and sustaining of life on Europa seems possible. On Titan, it cannot be ruled out. But the main exobiological interest of the largest satellite of Saturn is the presence of a complex organic chemistry which shows many similarities with the prebiotic chemistry which allowed the emergence of life on Earth. © Springer 2005.
KW - Astronomy
KW - Earth (planet)
KW - Martian surface analysis
KW - Observatories
KW - Organic chemicals
KW - Space research
KW - Astrobiology
KW - Biological signatures
KW - Europa
KW - Life
KW - Organic chemistry
KW - Prebiotic chemistry
KW - Titan
KW - Satellites
KW - Astrobiology

KW - Biological signatures
KW - Europa
KW - Exobiology
KW - Life
KW - Organic chemistry
KW - Prebiotic chemistry
KW - Titan
SN - 00386308 (ISSN)
LA - English
J2 - Space Sci Rev
M3 - Conference paper
DB - Scopus
N1 - Export Date: 23 February 2023; Cited By: 19; Correspondence Address: F. Raulin; Laboratoire Interuniversitaire des Systèmes Atmosphériques, LISA-UMR CNRS 7583, Universités Paris 7 et Paris 12, F-94000 Créteil, 61 Ave. du General de Gaulle, France; email: raulin@lisa.univ-paris12.fr; CODEN: SPSRA
ER -

TY - CONF
AU - Lorenz, R.D.
AU - Lunine, J.I.
AU - Zimmerman, W.
TI - Post-Cassini exploration of Titan: Science goals, instrumentation and mission concepts
PY - 2005
T2 - Advances in Space Research
VL - 36
IS - 2
SP - 281
EP - 285
DO - 10.1016/j.asr.2005.03.080
UR - https://www.scopus.com/inward/record.uri?eid=2-s2.0-27644445615&doi=10.1016%2fj.asr.2005.03.080&partnerID=40&md5=0114aaf1b31a18eae0034561e4d80e9c
AD - Lunar and Planetary Laboratory, University of Arizona, Tucson, AZ 85721, 1629 E. University Blvd., United States
AD - Jet Propulsion Laboratory, Pasadena, CA 91109, United States
AB - Titan promises to be a priority target for planetary and astrobiological exploration in the next decades. We survey the likely scientific objectives, instrumentation and mission architectures that may characterize the next visit to Titan. A near-surface airborne platform, perhaps an airship with some kind of surface sampling capability, appears most promising. We review recent work in the understanding of impact melt sheets and cryovolcanic flows as astrobiological 'incubators' wherein tholins produced in the atmosphere may interact with liquid water for prolonged periods, and survey technological developments that support a near-term return to Titan. © 2005 COSPAR. Published by Elsevier Ltd. All rights reserved.
KW - Earth atmosphere
KW - Planets
KW - Astrobiology
KW - Exploration

KW - Prebiotic chemistry
KW - Titan
KW - Astrophysics
KW - Astrobiology
KW - Exploration
KW - Prebiotic chemistry
KW - Titan
PB - Elsevier Ltd
SN - 02731177 (ISSN)
LA - English
J2 - Adv. Space Res.
M3 - Conference paper
DB - Scopus
N1 - Export Date: 23 February 2023; Cited By: 18; Correspondence Address: R.D. Lorenz; Lunar and Planetary Laboratory, University of Arizona, Tucson, AZ 85721, 1629 E. University Blvd., United States; email: rlorenz@lpl.arizona.edu; CODEN: ASRSD
ER -

TY - BOOK
AU - Pentecost, A.
TI - Travertine
PY - 2005
T2 - Travertine
SP - 1
EP - 445
DO - 10.1007/1-4020-3606-X
UR - https://www.scopus.com/inward/record.uri?eid=2-s2.0-84891446421&doi=10.1007%2f1-4020-3606-X&partnerID=40&md5=b3a6f49d8fe7c2d7478501fa83980c19
AD - School of Health and Life Sciences, King's College London, London SE1 9NN, Stamford Street 150, United Kingdom
AB - This is the first book entirely devoted to travertine, a material in use for over 4000 years. The single-author work is a valuable reference source for travertine, covering all aspects of travertine origins, formation, composition, flora and fauna, occurrence and utilisation, as well as covering allied continental carbonates such as lake marls, calcretes and beachrocks. Travertine, some forms of which are often known as 'tufa', is of particular significance as a source of environmental information (fossils and isotope geochemistry), often permitting the reconstruction of past environments. Reviews of dating techniques, classification and geomorphology are included and the author attempts to provide an unbiased but critical appraisal of current models of travertine formation. Currently, travertine is in great demand as a building and ornamental stone. It has also been exploited in unconventional ways, such as 'petrifying springs' and by way of unusual and little known artistic techniques. Scaling of pipes and boilers is often the result of processes allied to travertine formation. The phenomenon is described parallel to methods of scale elimination and compared with natural processes where travertine formation is inhibited. Travertine sites are of special scientific interest, with their rich and often unique floras and faunas displaying their unique biodiversity, and their unusual and often fragile biota.

Conservation issues are discussed, together with the description of travertine fossils and occurrences throughout the geological record. The content will be of interest to carbonate sedimentologists, hydrobiologists, palaeoclimatologists, physical geographers, water treatment engineers, astrobiologists, architects, and sculptors. © Springer-Verlag Berlin Heidelberg 2005. All rights are reserved.
PB - Springer Netherlands
SN - 1402035233 (ISBN); 978-140203523-4 (ISBN)
LA - English
J2 - Travertine
M3 - Book
DB - Scopus
N1 - Export Date: 23 February 2023; Cited By: 733
ER -

TY - JOUR
AU - Schröder, C.
AU - Klingelhöfer, G.
AU - Bailey, B.E.
AU - Staudigel, H.
TI - Mössbauer spectroscopy as a tool in astrobiology
PY - 2005
T2 - Hyperfine Interactions
VL - 166
IS - 1-4
SP - 567
EP - 571
DO - 10.1007/s10751-006-9346-x
UR - https://www.scopus.com/inward/record.uri?eid=2-s2.0-33845781980&doi=10.1007%2fs10751-006-9346-x&partnerID=40&md5=86c327279855ada5d771f3bd8234b053
AD - Institut für Anorganische Chemie und Analytische Chemie, Johannes Gutenberg-Universität, D-55128 Mainz, Staudinger Weg 9, Germany
AD - Institute of Geophysics and Planetary Physics, Scripps Institution of Oceanography, University of California San Diego, San Diego, CA 92093-0225, United States
AB - Two miniaturized Mössbauer spectrometers are part of the Athena instrument package of the NASA Mars Exploration Rovers, Spirit and Opportunity. The primary objectives of their science investigation are to explore two sites on the surface of Mars where water may once have been present, and to assess past environmental conditions at those sites and their suitability for life. Aqueous minerals - jarosite at Meridiani Planum, Opportunity's landing site, and goethite in the Columbia Hills in Gusev Crater, Spirit's landing site - were identified by Mössbauer spectroscopy, thus providing in situ proof of water being present at those sites in the past. The formation of jarosite in particular puts strong constraints on environmental conditions during the time of formation and hence on the evaluation of potential habitability. On Earth Mössbauer spectroscopy was used to investigate microbially induced changes in Fe oxidation states and mineralogy at the Loihi deep sea mount, a hydrothermal vent system, which might serve as an analogue for potential habitats in the Martian subsurface and the sub-ice ocean

of Jupiter's icy moon Europa. © 2006 Springer Science+Business Media, Inc.
KW - Astrobiology
KW - Biogeochemistry
KW - Europa
KW - Hydrothermal vent
KW - Mars
KW - Mössbauer spectroscopy
SN - 03043843 (ISSN)
LA - English
J2 - Hyperfine Interact.
M3 - Article
DB - Scopus
N1 - Export Date: 23 February 2023; Cited By: 2; Correspondence Address: C. Schröder; Institut für Anorganische Chemie und Analytische Chemie, Johannes Gutenberg-Universität, D-55128 Mainz, Staudinger Weg 9, Germany; email: schroedc@uni-mainz.de; CODEN: HYIND
ER -

TY - CONF
AU - Akkurt, H.
AU - Groves, J.L.
AU - Trombka, J.
AU - Starr, R.
AU - Evans, L.
AU - Floyd, S.
AU - Hoover, R.
AU - Lim, L.
AU - McClanahan, T.
AU - James, R.
AU - McCoy, T.
AU - Schweitzer, J.
TI - Pulsed neutron generator system for astrobiological and geochemical exploration of planetary bodies
PY - 2005
T2 - Nuclear Instruments and Methods in Physics Research, Section B: Beam Interactions with Materials and Atoms
VL - 241
IS - 1-4
SP - 232
EP - 237
DO - 10.1016/j.nimb.2005.07.029
UR - https://www.scopus.com/inward/record.uri?eid=2-s2.0-28044470277&doi=10.1016%2fj.nimb.2005.07.029&partnerID=40&md5=1fe6872105063e7c8491cd560cf33bff
AD - Schlumberger Princeton Technology Center, Princeton Junction, NJ 07605, 20 Wallace Road, United States
AD - NASA Goddard Space Flight Center, Greenbelt, MD 20771, United States
AD - Catholic University of America, Washington, DC 20064, United States
AD - Computer Sciences Corporation, Lanham-Seabrook, MD 20706, 7700 Hubble Drive, United States

AD - Brookhaven National Laboratory, Upton, NY 11973, United States
AD - National Museum of Natural History, Smithsonian Institution, Washington, DC 20560, United States
AD - University of Connecticut, Storrs, CT 06269, United States
AB - A pulsed neutron/gamma-ray detection system for use on rovers to survey the elemental concentrations of Martian and Lunar surface and subsurface materials is evaluated. A robotic survey system combining a pulsed neutron generator (PNG) and detectors (gamma ray and neutron) can measure the major constituents to a depth of about 30 cm. Scanning mode measurements can give the major elemental concentrations while the rover is moving; analyzing mode measurements can give a detailed elemental analysis of the adjacent material when the rover is stationary. A detailed map of the subsurface elemental concentrations will provide invaluable information relevant to some of the most fundamental astrobiological questions including the presence of water, biogenic activity, life habitability and deposition processes. © 2005 Elsevier B.V. All rights reserved.
KW - Astrophysics
KW - Deposition
KW - Gamma rays
KW - Geochemistry
KW - Lunar missions
KW - Martian surface analysis
KW - Neutrons
KW - Radiation detectors
KW - Surveys
KW - Water
KW - Elemental concentrations
KW - Neutron generators
KW - Planetary surveys
KW - Subsurface elemental composition
KW - Neutron sources
KW - Neutron generator
KW - Planetary surveys
KW - Subsurface elemental composition
SN - 0168583X (ISSN)
LA - English
J2 - Nucl Instrum Methods Phys Res Sect B
M3 - Conference paper
DB - Scopus
N1 - Export Date: 23 February 2023; Cited By: 9; Correspondence Address: J.L. Groves; Schlumberger Princeton Technology Center, Princeton Junction, NJ 07605, 20 Wallace Road, United States; email: groves@princeton.oilfield.slb.com; CODEN: NIMBE
ER -

TY - JOUR
AU - Fairén, A.G.
AU - Dohm, J.M.
AU - Uceda, E.R.
AU - Rodríguez, A.P.
AU - Baker, V.R.
AU - Fernández-Remolar, D.

AU - Schulze-Makuch, D.
AU - Amils, R.
TI - Prime candidate sites for astrobiological exploration through the hydrogeological history of Mars
PY - 2005
T2 - Planetary and Space Science
VL - 53
IS - 13
SP - 1355
EP - 1375
DO - 10.1016/j.pss.2005.06.007
UR - https://www.scopus.com/inward/record.uri?eid=2-s2.0-26444621376&doi=10.1016%2fj.pss.2005.06.007&partnerID=40&md5=95a046203ff6170aa535e9860cd95092
AD - Centro de Biología Molecular, CSIC-Universidad Autónoma de Madrid, 28049 Cantoblanco, Madrid, Spain
AD - Department of Hydrology and Water Resources, University of Arizona, Tucson, AZ 85721, United States
AD - Servicio de Investigación, Hospital Ramón y Cajal, 28937 Madrid, C. Colmenar km. 9, Spain
AD - Department of Earth and Planetary Science, University of Tokyo, Bunkyo-ku Tokyo 113-0033, 7-3-1 Hongo, Japan
AD - Lunar and Planetary Laboratory, University of Arizona, Tucson, AZ 85721, United States
AD - Centro de Astrobiología (CSIC-INTA), 28855 Torrejon de Ardoz, Madrid, Spain
AD - Department of Geology, Washington State University, Pullman, WA 99164, United States
AB - The hydrogeological evolution of Mars has been proposed to be dominated by the development of the Tharsis Magmatic Complex through superplume activity, with related magmatic-pulse-driven flood inundations that directly influence the shaping of the northern plains, the evolution of the atmosphere and climate, and subsurface and surface water processes. On the other hand, several possible biological models and terrestrial analogues have been suggested for Mars during the last decade, including the description of putative microfossils and the proposal of sedimentary units. Here we revisit these scenarios and present a possible bridge that integrates the geological, paleohydrological, and the putative biological histories of the planet. We primarily focus on the Noachian, a time period that arguably has recorded an inner dynamo, plate tectonics, and an ocean that may have covered one-third of the total surface area of Mars, due to its implications on the possible origin and early evolution of life. This stage is followed by a long-lived cold and dry phase, briefly punctuated by transient magmatic-driven hydrological cycles, dominated by a stagnant-lid/superplume regime, which directly influences the processes of natural selection on the putative early biosphere. Based on this hypothesized evolution of the planet, we suggest three martian locations as prime candidate sites for astrobiological exploration, each one corresponding to an inundation period: Meridiani Planum (Noachian/Early Hesperian), Mangala Valles (Late Hesperian/Early Amazonian), and Orcus Patera (Amazonian). © 2005 Elsevier Ltd. All rights reserved.
KW - Atmospherics

KW - Climatology
KW - Landforms
KW - Martian surface analysis
KW - Mathematical models
KW - Sediments
KW - Atmosphere/hydrosphere evolution
KW - Extremophiles
KW - Microfossils
KW - Prime candidate site for astrobiological exploration
KW - Superplume activity
KW - Hydrogeology
KW - Atmosphere/hydrosphere evolution
KW - Extremophiles
KW - Mars
KW - Prime candidate site for astrobiological exploration
SN - 00320633 (ISSN)
LA - English
J2 - Planet. Space Sci.
M3 - Article
DB - Scopus
N1 - Export Date: 23 February 2023; Cited By: 20; Correspondence Address: A.G. Fairén; Centro de Biología Molecular, CSIC-Universidad Autónoma de Madrid, 28049 Cantoblanco, Madrid, Spain; email: agfairen@cbm.uam.es; CODEN: PLSSA
ER -

TY - JOUR
AU - Skelley, A.M.
AU - Scherer, J.R.
AU - Aubrey, A.D.
AU - Grover, W.H.
AU - Ivester, R.H.C.
AU - Ehrenfreund, P.
AU - Grunthaner, F.J.
AU - Bada, J.L.
AU - Mathies, R.A.
TI - Development and evaluation of a microdevice for amino acid biomarker detection and analysis on Mars
PY - 2005
T2 - Proceedings of the National Academy of Sciences of the United States of America
VL - 102
IS - 4
SP - 1041
EP - 1046
DO - 10.1073/pnas.0406798102
UR - https://www.scopus.com/inward/record.uri?eid=2-s2.0-12844282193&doi=10.1073%2fpnas.0406798102&partnerID=40&md5=9f833e4479656d890353de22be0b7e0c
AD - Department of Chemistry, University of California, Berkeley, CA 94720, United States
AD - Scripps Institution of Oceanography, Univ. of California at San Diego, San Diego, CA 92093, United States
AD - Astrobiology Laboratory, Leiden University, 2300 RA Leiden,

Netherlands
AD - Jet Propulsion Laboratory, Pasadena, CA 91109, 4800 Oak Grove Drive, United States
AB - The Mars Organic Analyzer (MOA), a microfabricated capillary electrophoresis (CE) instrument for sensitive amino acid biomarker analysis, has been developed and evaluated. The microdevice consists of a four-wafer sandwich combining glass CE separation channels, microfabricated pneumatic membrane valves and pumps, and a nanoliter fluidic network. The portable MOA instrument integrates high voltage CE power supplies, pneumatic controls, and fluorescence detection optics necessary for field operation. The amino acid concentration sensitivities range from micromolar to 0.1 nM, corresponding to part-per-trillion sensitivity. The MOA was first used in the lab to analyze soil extracts from the Atacama Desert, Chile, detecting amino acids ranging from 10-600 parts per billion. Field tests of the MOA in the Panoche Valley, CA, successfully detected amino acids at 70 parts per trillion to 100 parts per billion in jarosite, a sulfate-rich mineral associated with liquid water that was recently detected on Mars. These results demonstrate the feasibility of using the MOA to perform sensitive in situ amino acid biomarker analysis on soil samples representative of a Mars-like environment.
KW - Amino Acids
KW - Biological Markers
KW - Chromatography, High Pressure Liquid
KW - Electrophoresis, Capillary
KW - Mars
KW - Atacama
KW - amino acid
KW - biological marker
KW - jarosite
KW - mineral
KW - sulfate
KW - unclassified drug
KW - water
KW - amino acid analysis
KW - analytic method
KW - analyzer
KW - article
KW - astronomy
KW - capillary electrophoresis
KW - device
KW - electric potential
KW - evaluation
KW - feasibility study
KW - fluorescence
KW - instrumentation
KW - liquid
KW - optics
KW - power supply
KW - priority journal
KW - pump
KW - soil analysis
KW - valve
KW - Amino acid analysis

KW - Astrobiology
KW - Capillary electrophoresis
KW - Microfabrication
SN - 00278424 (ISSN)
C2 - 15657130
LA - English
J2 - Proc. Natl. Acad. Sci. U. S. A.
M3 - Article
DB - Scopus
N1 - Export Date: 23 February 2023; Cited By: 227; Correspondence Address: R.A. Mathies; Department of Chemistry, University of California, Berkeley, CA 94720, United States; email: rich@zinc.cchem.berkeley.edu; CODEN: PNASA
ER -

TY - JOUR
AU - Montañés-Rodriguez, P.
AU - Pallé, E.
AU - Goode, P.R.
AU - Hickey, J.
AU - Koonin, S.E.
TI - Globally integrated-measurements of the earth's visible spectral albedo
PY - 2005
T2 - Astrophysical Journal
VL - 629
IS - 2 I
SP - 1175
EP - 1182
DO - 10.1086/431420
UR - https://www.scopus.com/inward/record.uri?eid=2-s2.0-23844505875&doi=10.1086%2f431420&partnerID=40&md5=f97d5071d9772acf0c954bce8e7eea3e
AD - Big Bear Solar Observatory, New Jersey Institute of Technology, Newark, NJ 07102, United States
AD - Palomar Observatory, Palomar Mountain, CA 92060-0200, 35899 Canfield Road, United States
AD - W. K. Kellogg Radiation Laboratory, California Institute of Technology, Pasadena, CA 91125, United States
AB - We report spectroscopic observations of the earthshine reflected from the Moon. By applying our well-developed photometry methodology to spectroscopy, we are able to precisely determine the Earth's reflectance and its variation as a function of wavelength through a single night as the Earth rotates. These data imply that planned regular monitoring of earthshine spectra will yield valuable new inputs for climate models, which would be complementary to those from the more standard broadband measurements of satellite platforms. For our single night of reported observations, we find that Earth's albedo decreases sharply with wavelength from 500 to 600 nm, while being almost flat from 600 to 900 nm. The mean spectroscopic albedo over the visible is consistent with simultaneous broadband photometric measurements. Unlike previous reports, we find no evidence for either an appreciable "red" or "vegetation" edge in the Earth's spectral albedo, or for changes in

this spectral region (700-740 nm) over the 40° of Earth's rotation covered by our observations. Whether or not the absence of a vegetation signature in disk-integrated observations of the Earth is a common feature awaits the analysis of more earthshine data and simultaneous satellite cloud maps at several seasons. If our result is confirmed, it would limit efforts to use the red edge as a probe for Earth-like extrasolar planets. Water vapor and molecular oxygen signals in the visible earthshine, and carbon dioxide and methane in the near-infrared, are more likely to be powerful probes.
KW - Astrobiology
KW - Planetary systems
PB - Institute of Physics Publishing
SN - 0004637X (ISSN)
LA - English
J2 - Astrophys. J.
M3 - Article
DB - Scopus
N1 - Export Date: 23 February 2023; Cited By: 49; Correspondence Address: P. Montañés-Rodriguez; Big Bear Solar Observatory, New Jersey Institute of Technology, Newark, NJ 07102, United States; email: pmr@bbso.njit.edu
ER -

TY - CONF
AU - Dieter, W.R.
AU - Lodder, R.A.
AU - Lumpp Jr., J.E.
TI - Scanning for Extinct Astrobiological Residues and Current Habitats (SEARCH)
PY - 2005
T2 - IEEE Aerospace Conference Proceedings
VL - 2005
C7 - 1559317
SP - 234
EP - 245
DO - 10.1109/AERO.2005.1559317
UR - https://www.scopus.com/inward/record.uri?eid=2-s2.0-33751529770&doi=10.1109%2fAERO.2005.1559317&partnerID=40&md5=186f2fd9b7bb98ea33f364b60418e9d0
AD - Department of Electrical and Computer Engineering, University of Kentucky, Lexington, KY 40506, United States
AB - SEARCH is a new method to seek evidence of extinct life and potential habitats. SEARCH combines innovative spectroscopic integrated sensing and processing with a hyperspace data-analysis algorithm. Using UV, visible, and near-IR spectroscopic integrated sensing and processing, SEARCH is designed to explore and quantitatively assess a local region on the surface of a planet or moon as a potential habitat for life, past or present. In the course of collecting geological data, SEARCH spectrometry can investigate planetary processes of relevance to past habitability, including the role of water. In addition to its own investigations, SEARCH can be used at a distance to guide a rover to areas of interest for application of other analysis tools. Several prototype implementations of SEARCH have been developed and initial

experimental results are presented along with a design for a full-scale version of SEARCH for Mars exploration. © 2005 IEEE.
KW - Algorithms
KW - Astrophysics
KW - Data reduction
KW - Geological surveys
KW - Planetary surface analysis
KW - Scanning
KW - Spectroscopic analysis
KW - Habitats
KW - Near-IR spectroscopic integrated sensing
KW - Scanning for Extinct Astrobiological Residues and Current Habitats (SEARCH)
KW - Space research
PB - IEEE Computer Society
SN - 1095323X (ISSN); 0780388704 (ISBN); 978-078038870-3 (ISBN)
LA - English
J2 - IEEE Aerosp. Conf. Proc.
M3 - Conference paper
DB - Scopus
N1 - Export Date: 23 February 2023; Cited By: 6; Correspondence Address: W.R. Dieter; Department of Electrical and Computer Engineering, University of Kentucky, Lexington, KY 40506, United States; email: dieter@uky.edu
ER -

TY - JOUR
AU - Jura, M.
TI - The age dependence of the detectability of comets orbiting solar-type stars
PY - 2005
T2 - Astrophysical Journal
VL - 620
IS - 1 I
SP - 487
EP - 491
DO - 10.1086/426902
UR - https://www.scopus.com/inward/record.uri?eid=2-s2.0-15944397310&doi=10.1086%2f426902&partnerID=40&md5=ae25acadf9c4d3d333bdb03fa679ca2a
AD - Department of Physics, University of California, Los Angeles, CA 90095-1562, United States
AB - The outflows from comets in orbit around G-type main-sequence stars can be detected when they produce transient OH absorption lines in the spectrum near 3100 Å of the host star. There is only about a 3 × 10-8 probability of detecting an analog to comet Hale-Bopp orbiting an analog to the Sun. However, for young solar-type stars with very large numbers of comets, possibly delivering water to terrestrial planets, there is as much as a 1% chance that any sufficiently sensitive, randomly timed observation may detect such transient absorption. © 2005. The American Astronomical Society. All rights reserved.
KW - Astrobiology
KW - Comets: general

KW - Planetary systems
PB - Institute of Physics Publishing
SN - 0004637X (ISSN)
LA - English
J2 - Astrophys. J.
M3 - Article
DB - Scopus
N1 - Export Date: 23 February 2023; Cited By: 3; Correspondence Address: M. Jura; Department of Physics, University of California, Los Angeles, CA 90095-1562, United States; email: jura@clotho.astro.ucla.edu
ER -

TY - CONF
AU - Scott Hubbard, G.
AU - Chu-Thielbar, L.
TI - Astrobiology: The first decade
PY - 2005
T2 - International Astronautical Federation - 56th International Astronautical Congress 2005
VL - 2
SP - 793
EP - 806
UR - https://www.scopus.com/inward/record.uri?eid=2-s2.0-34250795551&partnerID=40&md5=67bf5f3a18994150224d704469f653dd
AD - NASA Ames Research Center, Moffett Field, CA 94035-1000, United States
AB - Astrobiology, the ten year old multidisciplinary science dedicated to the study of life in the universe, has had a profound effect on space exploration world wide. Defined and initially advocated at NASA Ames Research Center a decade ago, the field has gained international status and represents the scientific heart of the new NASA Vision for Space Exploration. This paper traces the original impetus for defining the field, its decade long history, and its current status. The NASA Astrobiology Institute is described along with the Astrobiology Science Conference, the major biennial international meeting that serves the field. Major science results and advances are discussed. The influence of the field on research, missions, roadmaps and policy ranging from early earth studies, NASA's Mars Exploration Program strategy of "Follow the Water", and NASA's vision and mission statements to current exploration strategies world-wide, is demonstrated. The US Vision for Space Exploration is presented in the context of Astrobiology.
KW - International cooperation
KW - NASA
KW - Strategic planning
KW - Technical presentations
KW - Mission statements
KW - Multidisciplinary science
KW - Space exploration strategies
KW - Astrobiology
LA - English
J2 - Int. Astronautical Fed. Int. Astroanautical Congr.
M3 - Conference paper

DB - Scopus
N1 - Export Date: 23 February 2023; Cited By: 0; Correspondence Address: G. Scott Hubbard; NASA Ames Research Center, Moffett Field, CA 94035-1000, United States; email: G.Scott.Hubbard@nasa.gov
ER -

TY - CONF
AU - Levin, G.V.
TI - Marslife-howDarwinianpressuresmighthaveshapeditsformandfunction
PY - 2005
T2 - Proceedings of SPIE - The International Society for Optical Engineering
VL - 5906
C7 - 59060D
SP - 1
EP - 10
DO - 10.1117/12.624634
UR - https://www.scopus.com/inward/record.uri?eid=2-s2.0-29844437958&doi=10.1117%2f12.624634&partnerID=40&md5=0f332e960fcccb17ffff33383888db11
AD - Biospherix Division, Spherix Incorporated, Annapolis, MD 21401, United States
AB - The possible existence of life on Mars is now gaining credence. Evidence consistent with or supporting the presence of extant microbial life, as reported by a life detection experiment on the Viking Mission in 1976, has been rapidly accumulating from spacecraft orbital and lander operations, and from terrestrial observations. Vast oceans of frozen water near the planet's surface are being discovered, with strong indications of recent or present liquid flows, and theory and laboratory experiment have demonstrated that liquid water should exist on the surface of Mars. The biosphere on Earth has been extended into extreme environments until recently thought inimical to life. Places void of life have become rare. No life requirement has been found lacking on Mars. It is possible that, by the time of this 50th Anniversary SPIE Meeting, the paradigm shift accepting life beyond the Earth may have been made. Mankind will then emerge from its ancient fear of loneliness into a new fear of anticipation of what that still unidentified life might portend. The author attempts to apply Darwinian principles of evolution to life on Mars under the selection pressures, opportunities and constraints that have been imposed by past and present Martian conditions. Starting with the type of cell believed to have begun the evolutionary process on Earth, he speculates on what the current life on Mars may be like in form and function, including what threat or promise it might hold for Earth life.
KW - Biodiversity
KW - Earth (planet)
KW - Life cycle
KW - Microorganisms
KW - Spacecraft
KW - Water
KW - Astrobiology
KW - Evolution on Mars

KW - Extant life on Mars
KW - Extraterrestrial life
KW - Life on Mars
KW - Mars life
KW - Martian biology
KW - Martian life forms
KW - Martian surface analysis
KW - Astrobiology
KW - Evolution on Mars
KW - Extant life on Mars
KW - Extraterrestrial life
KW - Life on Mars
KW - Mars life
KW - Martian biology
KW - Martian life forms
SN - 0277786X (ISSN)
LA - English
J2 - Proc SPIE Int Soc Opt Eng
M3 - Conference paper
DB - Scopus
N1 - Export Date: 23 February 2023; Cited By: 3; Correspondence Address: G.V. Levin; Biospherix Division, Spherix Incorporated, Annapolis, MD 21401, United States; email: glevin@spherix.com; CODEN: PSISD
ER -

TY - JOUR
AU - Botta, O.
TI - Organic chemistry in meteorites, comets, and the interstellar medium
PY - 2005
T2 - Proceedings of the International Astronomical Union
VL - 1
IS - S231
SP - 479
EP - 488
DO - 10.1017/S1743921306007496
UR - https://www.scopus.com/inward/record.uri?eid=2-s2.0-63549108498&doi=10.1017%2fS1743921306007496&partnerID=40&md5=7c540eb33cf09a04c147b97f7a760bb7
AD - International Space Science Institute, 3012 Bern, Switzerland
AD - NASA Goddard Space Flight Center, Code 699, Greenbelt, MD 20771, United States
AB - With the notable exception of those originating on the Moon and Mars, all known meteorites are pieces of objects in the asteroid belt. As such, they have recorded a succession of chemical processes, starting from reactions in the interstellar medium (ISM), followed by reactions that accompanied the formation and evolution of the early solar system, and culminated with reactions during aqueous alteration in the meteorite parent bodies. One of the challenges in meteorite research is to decipher this record and to learn about interstellar formation processes as well as to conditions in the early solar system. The rare class of carbonaceous chondrites contains up to 5% by weight of organic carbon, most of

which is locked in an insoluble macromolecular material and only about 20% of it is in the form of distinct organic compound classes. The molecular and isotopic data of these organic compounds clearly show an interstellar heritage, but a fraction of these precursors were later modified. For example, the amino acids were probably formed inside the meteorite parent body during the aqueous alteration period from simple molecules such as HCN, NH3 and carbonyl compounds. However, the CI type carbonaceous chondrites contain a significantly distinct amino acid composition, indicating that there may be other synthetic processes involved. Polycyclic aromatic hydrocarbons (PAHs) are probably the most abundant form of organic carbon in the gas phase in the ISM. PAHs are among the most abundant organic compounds in carbonaceous meteorites, and they have been shown to have a presolar origin. A fraction of these PAHs are present in an extractable form, while the rest is part of the insoluble macromolecular matter. Progress is being made in the understanding of the evolution of this material in relation to aqueous alteration and oxidation. Although the potential of cometary meteorites cannot be ruled out, no such macro-meteorite has been recognized in the meteorite collections. Therefore, the organic composition of comets has been inferred mostly from astronomical observations. Future in-situ investigation of comets with spacecraft such as Rosetta will deliver new data on their organic composition, in particular the non-volatile fraction. However, in order to understand the contributions of different formation processes in primitive solar system objects, the analysis of the organic composition of meteorites remains essential. © 2006 International Astronomical Union.
KW - Asteroids
KW - Astrobiology
KW - Astrochemistry
KW - Comets: general
KW - Methods: laboratory
KW - Minor planets
KW - Molecular data
KW - Molecular processes
KW - Solar system: formation
KW - Stars: AGB and post-AGB
PB - Cambridge University Press
SN - 17439213 (ISSN)
LA - English
J2 - Proc. Int. Astron. Union
M3 - Article
DB - Scopus
N1 - Export Date: 23 February 2023; Cited By: 5; Correspondence Address: O. Botta; NASA Goddard Space Flight Center, Code 699, Greenbelt, MD 20771, United States; email: obotta@pop600.gsfc.nasa.gov
ER -

TY - CONF
AU - Yamauchi, M.
AU - Sandahl, I.
AU - Nilsson, H.

AU - Lundin, R.
AU - Eliasson, L.
TI - Budget and roles of heavy ions in the solar system
PY - 2005
T2 - European Space Agency, (Special Publication) ESA SP
IS - 588
SP - 397
EP - 400
UR - https://www.scopus.com/inward/record.uri?eid=2-s2.0-33645721494&partnerID=40&md5=db11b2d9231ea01d205c9633b82bfa73
AD - Swedish Institute of Space Physics, Kiruna, Sweden
AB - Oxygen and other major heavy species such as carbon, nitrogen, sodium, and sulfur circulate through the entire planetary system between the subsurface, surface, ocean, atmosphere, exosphere, and even the heliosphere (e.g., as meteors and solar wind) in a complicated manner. The amount of O + escaping from Earth/Mars is as large as 1 - 10 kg/s, and may no longer be ignored on the time scale of planetary evolution. Therefore, studying the space-level circulation of the heavy ions has an obvious cross-disciplinary importance on the subjects such as: Evolution of the Earth, planets, satellites; Modelling ancient Earth (astrobiology); Boundary condition for the geochemistry of reservoirs; Environmental issue; Physics of the planetary exosphere; Solar-terrestrial interaction; Basic plasma physics; It is therefore important to measure the thermal and non-thermal heavy ions for different masses (e.g., separation of C, N, O), energy, and flowing direction in both the Earth's and planetary exospheres even in planetology missions.
KW - Boundary conditions
KW - Earth (planet)
KW - Martian surface analysis
KW - Nitrogen
KW - Oxygen
KW - Sulfur
KW - Oxygen: circulation
KW - Planetary exospheres
KW - Planets: evolution
KW - Solar-terrestrial interaction
KW - Solar system
KW - Oxygen: circulation
KW - Planets: evolution
SN - 03796566 (ISSN); 9290928999 (ISBN); 978-929092899-7 (ISBN)
LA - English
J2 - Eur Space Agency Spec Publ ESA SP
M3 - Conference paper
DB - Scopus
N1 - Export Date: 23 February 2023; Cited By: 2; CODEN: ESPUD
ER -

TY - CONF
AU - Franke, E.A.
AU - Magee, M.J.
AU - Rigney, M.P.
AU - Stone, B.C.
TI - Progress toward the development of lifeform detection

algorithms for the Deep Phreatic Thermal eXplorer (DEPTHX)
PY - 2005
T2 - Proceedings of SPIE - The International Society for Optical Engineering
VL - 5906
C7 - 590613
SP - 1
EP - 10
DO - 10.1117/12.626269
UR - https://www.scopus.com/inward/record.uri?eid=2-s2.0-29844437559&doi=10.1117%2f12.626269&partnerID=40&md5=161c29d452a2941f25a7312d846a0903
AD - Southwest Research Institute, San Antonio, TX 78238-5166, 6220 Culebra Road, United States
AD - Stone Aerospace/PSC, Inc., Gaithersburg, MD 20879-1833, 18912 Glendower Road, United States
AB - Based on observations of seemingly hostile aqueous environments on earth, it is possible for lifeforms not only to evolve but to thrive in conditions that, by human standards, are extreme. Such lifeforms, typically termed "extremophiles" can, for example, live in the vicinity of deep water volcanic vents that are spewing superheated water laden with sulfur compounds at intense pressures. Since similar conditions may exist on Jupiter's moon Europa, there is widespread interest in developing an autonomous search-for-life capability that could be deployed in aqueous, extraterrestrial environments. As one step toward this goal, the DEep Phreatic THermal eXplorer (DEPTHX) is a NASA Astrobiology Science and Technology for Exploring Planets (ASTEP) project to design, develop and field-test a robotic vehicle to explore such environments. The principal astrobiological science objective of DEPTHX is to develop an advanced methodology and protocol for the discrimination of microbial life in a sub-aqueous environment. Implementation requires the design, development, and demonstration of a fully autonomous architecture for intelligent biological sample detection and collection, whereby the robotic device will be capable of performing the following functions: 1. Deep hydrothermal springs will be mapped with great accuracy in three dimensions. 2. Data will be acquired from a hierarchical suite of on-board microbial life detection and sensors and processors and will be analyzed to determine whether life is present. 3. Specimens will be aseptically collected and returned for subsequent ex-situ laboratory analysis preserved under ambient conditions. The paper describes current progress toward these objectives, with an emphasis on the analysis of data acquired from the life sensors for the purpose of detecting lifeforms.
KW - Data acquisition
KW - Life cycle
KW - Microorganisms
KW - Robots
KW - Specimen preparation
KW - Thermal effects
KW - Autonomous
KW - Europa
KW - Life sensor

KW - Microbial
KW - Phreatic
KW - Zacatón
KW - Space probes
KW - Autonomous
KW - Europa
KW - Life sensor
KW - Microbial
KW - Phreatic
KW - Zacatón
SN - 0277786X (ISSN)
LA - English
J2 - Proc SPIE Int Soc Opt Eng
M3 - Conference paper
DB - Scopus
N1 - Export Date: 23 February 2023; Cited By: 3; CODEN: PSISD
ER -

TY - CONF
AU - Cash, W.
AU - Kasdin, J.
AU - Seager, S.
AU - Arenberg, J.
TI - Direct studies of exo-planets with the New Worlds Observer
PY - 2005
T2 - Proceedings of SPIE - The International Society for Optical Engineering
VL - 5899
C7 - 58990S
SP - 1
EP - 12
DO - 10.1117/12.617377
UR - https://www.scopus.com/inward/record.uri?eid=2-s2.0-29344451436&doi=10.1117%2f12.617377&partnerID=40&md5=5d104b75103426693fe092df7b44e9ed
AD - University of Colorado, United States
AD - Princeton University, United States
AD - Carnegie Institution, United States
AD - Northrop-Grumman, United States
AB - The New World Observer has the potential to discover and study planets around other stars without expensive and risky technical heroics. We describe the starshade, a large, deployable sheet on a separate spacecraft that is flown into position along the line of sight to a nearby star. We show how a starshade can be designed and built in a practical and affordable manner to fully remove starlight and leave only planet light entering a telescope. The simulations demonstrate That NWI can detect planetary system features as faint as comets, perform spectroscopy to look for water and life signs, and perform photometry to search for oceans, continents, clouds and polar caps.
KW - Astronomy
KW - Computer simulation
KW - Photometry
KW - Space rendezvous

KW - Spacecraft
KW - Astrobiology
KW - Coronography
KW - Exoplanets
KW - Planets
KW - Astrobiology
KW - Coronography
KW - Exoplanets
SN - 0277786X (ISSN)
LA - English
J2 - Proc SPIE Int Soc Opt Eng
M3 - Conference paper
DB - Scopus
N1 - Export Date: 23 February 2023; Cited By: 9; CODEN: PSISD
ER -

TY - JOUR
AU - Stalport, F.
AU - Coll, P.
AU - Cabane, M.
AU - Person, A.
AU - González, R.N.
AU - Raulin, F.
AU - Vaulay, M.J.
AU - Ausset, P.
AU - McKay, C.P.
AU - Szopa, C.
AU - Zarnecki, J.
TI - Search for past life on Mars: Physical and chemical characterization of minerals of biotic and abiotic origin: Part 1 - Calcite
PY - 2005
T2 - Geophysical Research Letters
VL - 32
IS - 23
C7 - L23205
SP - 1
EP - 5
DO - 10.1029/2005GL023403
UR - https://www.scopus.com/inward/record.uri?eid=2-s2.0-31544435735&doi=10.1029%2f2005GL023403&partnerID=40&md5=fdb49d25ee759df1db720a8cae7ee2bb
AD - Laboratoire Interuniversitaire des Systèmes Atmosphériques (LISA), Université Paris XII, F-94010 Créteil, France
AD - Service d'Aéronomie, CNRS, Université Paris VI, F-75005 Paris, 4 Place Jussieu, France
AD - Laboratoire de Géologie Sédimentaire, Université Paris VI, F-75005 Paris, 4 Place Jussieu, France
AD - Laboratorio de Quimica de Plasmas y Estudios Planetarios, Universidad Nacional Autónoma de México, Mexico City, Mexico
AD - Interfaces Traitements, Organisation et Dynamique de Systèmes (ITODYS), Université Paris VII, F-75005 Paris, France
AD - Space Science Division, NASA Ames Research Center, Moffett Field, CA 94035-1000, MS 245-3, United States

AD - Planetary and Space Sciences Research Institute, Open University, Milton Keynes MK15 0BT, United Kingdom
AB - Several lines of evidence suggest that early Mars once had Liquid water on its surface, a denser atmosphere and a mild climate. Similar environmental conditions led to the origin of life on the Earth more than 3.5 billion years ago; consequently, life might also have originated on Mars. The Viking landers searched for evidence of organic molecules on the surface of Mars, and found that the Martian soil is depleted in organics at ppb levels at the landing sites. We contend that inorganic compounds could give us interesting clues as to the existence of possible biological activity in future astrobiological missions to Mars. Consequently, we have investigated the physical and chemical properties of calcite, which could be expected on Mars because liquid water was certainly present on the surface of early Mars and carbon dioxide was abundant in its atmosphere. Calcite is interesting because on Earth this mineral is produced by abiotic processes as well as by biological activity. One may suppose that crystalline defects and trace element in the crystal lattice and the growth speed of biotic calcites must indicate a difference between them and pure abiotic calcites. We investigated twelve different terrestrial calcite samples from various origins: biotic, diagenetic and abiotic. The minerals were studied by X-ray diffraction and electron scanning microscopy to determine their mineralogical and chemical composition, and differential thermal analysis coupled to thermogravimetric analysis (DTA-TG) to determine their thermal behavior. Our results show that the thermal degradation of abiotic calcite starts at a temperature at least 40°C higher than the degradation temperature of any biotic calcite investigated. Consequently, in the case of a Martian in-situ study or in a sample return mission, the analysis of Martian minerals by DTA-TG represents a promising approach to detect evidence of past biological activity on Mars. Copyright 2005 by the American Geophysical Union.
KW - Astronomy
KW - Atmospheric chemistry
KW - Atmospheric composition
KW - Biology
KW - Calcite
KW - Carbon dioxide
KW - Crystal lattices
KW - Defects
KW - Molecules
KW - Scanning electron microscopy
KW - Trace elements
KW - X ray diffraction analysis
KW - calcite
KW - Mars
KW - Abiotic origin
KW - Astrobiology
KW - Biotic origin
KW - Mars
KW - Martian surface analysis
SN - 00948276 (ISSN)
LA - English

J2 - Geophys. Res. Lett.
M3 - Article
DB - Scopus
N1 - Export Date: 23 February 2023; Cited By: 33; Correspondence Address: F. Stalport; Laboratoire Interuniversitaire des Systèmes Atmosphériques (LISA), Université Paris XII, F-94010 Créteil, France; email: stalport@lisa.univ-paris12.fr; CODEN: GPRLA
ER -

TY - CONF
AU - Balint, T.S.
TI - To Europa and beyond (enabled by a small-RPS)
PY - 2005
T2 - IEEE Aerospace Conference Proceedings
VL - 2005
C7 - 1559396
DO - 10.1109/AERO.2005.1559396
UR - https://www.scopus.com/inward/record.uri?eid=2-s2.0-33751535216&doi=10.1109%2fAERO.2005.1559396&partnerID=40&md5=13f8807816ca2c81b2b2fdf0bec25083
AD - Jet Propulsion Laboratory, California Institute of Technology, M/S 301 -170U, Pasadena, CA 91109-8099, 4800 Oak Grove Drive, United States
AB - Solar system exploration (SSE) to the Outer Planets requires internal power sources, since external power generation, relying on solar power, is not very effective or practical. One of the scientifically interesting destinations in this region is Jupiter's moon Europa, where a lander would require internal power sources, such as batteries or Radioisotope Power Systems (RPS) to achieve mission success. This paper discusses a Europa lander concept as a potential adjunct element to NASA's proposed Jupiter Icy Moons Orbiter (JIMO) mission. The lander would operate on the surface for 3 to 14 days, performing astrobiology, geophysics and geological-compositional measurements as defined by the JIMO Science Definition Team (SDT). The power system trade study for this concept compares small-RPSs up to ~50We against primary batteries. Additional SSE destinations are also outlined, where small-RPSs provide a viable option to accomplish science and mission objectives. © 2005 IEEE.
KW - Measurement theory
KW - Primary batteries
KW - Radioisotopes
KW - Solar energy
KW - Space research
KW - Radioisotope Power Systems (RPS)
KW - Solar system exploration (SSE)
KW - Solar system
SN - 1095323X (ISSN); 0780388704 (ISBN); 978-078038870-3 (ISBN)
LA - English
J2 - IEEE Aerosp. Conf. Proc.
M3 - Conference paper
DB - Scopus
N1 - Export Date: 23 February 2023; Cited By: 0; Correspondence Address: T.S. Balint; Jet Propulsion Laboratory, California Institute of Technology, M/S 301 -170U, Pasadena, CA 91109-8099,

4800 Oak Grove Drive, United States; email:
tibor.balint@jpl.nasa.gov
ER -

TY - CONF
AU - Pikuta, E.V.
AU - Itoh, T.
AU - Hoover, R.B.
TI - Anaerobic decomposition of cellulose by alkaliphilic microbial community of Owens Lake, California
PY - 2005
T2 - Proceedings of SPIE - The International Society for Optical Engineering
VL - 5906
C7 - 590604
SP - 1
EP - 12
DO - 10.1117/12.624093
UR - https://www.scopus.com/inward/record.uri?eid=2-s2.0-29844447126&doi=10.1117%2f12.624093&partnerID=40&md5=d6659bb3aca0474e93e918bb9ccade3d
AD - Astrobiology Laboratory, NASA, National Space Science and Technology Center, Huntsville, AL 35805, 320 Sparkman Dr., United States
AD - Japan Collection of Microorganisms, RIKEN (The Institute of Physical and Chemical Research), Wako, Saitama 351-0198, 2-1 Hirosawa, Japan
AB - The study of alkaliphilic microbial communities from anaerobic sediments of Owens and Mono Lakes in California has established the presence of active microbial cellulolytic processes in both studied lakes. The prior study of the microbial diversity of anaerobes in Mono Lake showed that the trophic chain of organic decomposition includes secondary anaerobes that previously were found to be unknown species (Spirochaeta americana, Tindallia californiensis, and Desulfonatronum thiodismutans). As we published earlier, the secondary anaerobes of Owens Lake morphologically were found to be very similar to those of Mono Lake. However, detailed comparison of the physiology and genetics has led to the conclusion that some links of organic decomposition in the trophic chain of the Owens Lake community are represented by a different unknown species. A new isolate of a sugarlytics free-living spirochete from Owens Lake ASpC2, which morphologically was similar to S. americana AspG1T isolated from Mono Lake, was found to have a different metabolic capacity such as the lack of capability to produce hydrogen during the fermentation of sugars. Furthermore, from the same microbial community of Owens Lake, another sugarlytics spore-forming alkaliphilic strain SCA was isolated in pure culture and described. Here we discuss the universal structure of the microbial community, types of microbial communities, review some hypothesis about Earth's Primordial Ocean and relevant new discoveries about water on Mars. This paper also presents some of the characteristics of novel isolates from anaerobic sediments of Owens Lake as a unique relic ecosystem of Astrobiological significance, and describes the participation of these strains in the process of cellulose

degradation.
KW - Anaerobic Process
KW - Anatomy
KW - Cellulose
KW - Decay
KW - Hydrogen
KW - Lakes
KW - Metabolism
KW - Microorganisms
KW - Anaerobic digestion
KW - Cellulose
KW - Decomposition
KW - Hydrogen
KW - Lakes
KW - Metabolism
KW - Morphology
KW - Alkaliphilic
KW - Mars
KW - Microbial community
KW - Spirochaeta sp.
KW - Sugarlytic anaerobe
KW - Microorganisms
KW - Alkaliphilic
KW - Fermentation
KW - Mars
KW - Microbial community
KW - Spirochaeta sp.
KW - Sugarlytic anaerobe
SN - 0277786X (ISSN)
LA - English
J2 - Proc SPIE Int Soc Opt Eng
M3 - Conference paper
DB - Scopus
N1 - Export Date: 23 February 2023; Cited By: 1; Correspondence Address: E.V. Pikuta; Astrobiology Laboratory, NASA, National Space Science and Technology Center, Huntsville, AL 35805, 320 Sparkman Dr., United States; email: Elena.Pikuta@MSFC.NASA.GOV; CODEN: PSISD
ER -

TY - JOUR
AU - Knoll, A.H.
AU - Carr, M.
AU - Clark, B.
AU - Des Marais, D.J.
AU - Farmer, J.D.
AU - Fischer, W.W.
AU - Grotzinger, J.P.
AU - McLennan, S.M.
AU - Malin, M.
AU - Schröder, C.
AU - Squyres, S.
AU - Tosca, N.J.
AU - Wdowiak, T.
TI - An astrobiological perspective on Meridiani Planum

PY - 2005
T2 - Earth and Planetary Science Letters
VL - 240
IS - 1
SP - 179
EP - 189
DO - 10.1016/j.epsl.2005.09.045
UR - https://www.scopus.com/inward/record.uri?eid=2-s2.0-27944440528&doi=10.1016%2fj.epsl.2005.09.045&partnerID=40&md5=53e03875e83b2d3a3f481d2b8125b7ee
AD - Department of Organismic and Evolutionary Biology, Harvard University, Cambridge, MA 02138, United States
AD - United States Geological Survey, Menlo Park, CA 94025, 345 Middlefield Rd., United States
AD - Ma Lockheed Martin Corporation, Littleton, CO 80127, 12257 State Highway 121, United States
AD - NASA Ames Research Center, Moffett Field, CA 94035-1000, United States
AD - Department of Geological Sciences, Arizona State University, Tempe, AZ 85287-1404, United States
AD - Department of Earth, Atmospheric and Planetary Sciences, Massachusetts Institute of Technology, Cambridge, MA 02139, United States
AD - Department of Geosciences, State University of New York, Stony Brook, NY 11794, United States
AD - Malin Space Science Systems, San Diego, CA 92191, PO Box 910148, United States
AD - Institut für Anorganische und Analytische Chemie, Johannes Gutenberg-Universität, D-55128 Mainz, Staudinger Weg 9, Germany
AD - Department of Astronomy, Cornell University, Ithaca, NY 14853, Space Sciences Building, United States
AD - Department of Physics, University of Alabama, Birmingham, AL 35294, United States
AB - Sedimentary rocks exposed in the Meridiani Planum region of Mars record aqueous and eolian deposition in ancient dune and interdune playa-like environments that were arid, acidic, and oxidizing. On Earth, microbial populations have repeatedly adapted to low pH and both episodic and chronic water limitation, suggesting that, to a first approximation, the Meridiani plain may have been habitable during at least part of the interval when deposition and early diagenesis took place. On the other hand, the environmental conditions inferred for Meridiani deposition would have posed a challenge for prebiotic chemical reactions thought to have played a role in the origin of life on Earth. Orbital observations suggest that the combination of sulfate minerals and hematite found in Meridiani rocks may be unusual on the martian surface; however, there is reason to believe that acidity, aridity, and oxidizing conditions were broadly distributed on ancient Mars. When these conditions were established and how much environmental heterogeneity existed on early Mars remain to be determined. Because sulfates and iron oxides can preserve detailed geochemical records of environmental history as well as chemical, textural and microfossil signatures of biological activity, Meridiani Planum is an attractive candidate for Mars sample return. © 2005 Elsevier B.V. All rights

reserved.
KW - Deposition
KW - Geochemistry
KW - Iron oxides
KW - Microbiology
KW - Oxidation
KW - Sedimentary rocks
KW - Sulfur compounds
KW - Astrobiology
KW - Environmental history
KW - Sulfates
KW - Mars
KW - microbiology
KW - sedimentary rock
KW - Martian surface analysis
KW - Astrobiology
KW - Environmental history
KW - Mars
KW - Meridiani Planum
KW - Microbiology
SN - 0012821X (ISSN)
LA - English
J2 - Earth Plan. Sci. Lett.
M3 - Article
DB - Scopus
N1 - Export Date: 23 February 2023; Cited By: 103; Correspondence Address: A.H. Knoll; Department of Organismic and Evolutionary Biology, Harvard University, Cambridge, MA 02138, United States; email: aknoll@oeb.harvard.edu
ER -

TY - CONF
AU - Abyzov, S.S.
AU - Gerasimenko, L.M.
AU - Hoover, R.B.
AU - Mitskevich, I.N.
AU - Mulyukin, A.L.
AU - Poglazova, M.N.
AU - Rozanov, A.Y.
TI - Microbiological methodology in astrobiology
PY - 2005
T2 - Proceedings of SPIE - The International Society for Optical Engineering
VL - 5906
C7 - 59060A
SP - 1
EP - 17
DO - 10.1117/12.618565
UR - https://www.scopus.com/inward/record.uri?eid=2-s2.0-29844448137&doi=10.1117%2f12.618565&partnerID=40&md5=cfd7a0b0055c776111b1a59d4ba9f49f
AD - Winogradsky Institute of Microbiology, Russian Academy of Sciences, Moscow, 117312, Pr. 60-letiya Oktyabrya 7/2, Russian Federation

AD - NASA National Space Science and Technology Center, Huntsville, AL 35812, United States
AD - Paleontological Institute, Russian Academy of Sciences, Moscow, 117647, Profsoyuznaya 123, Russian Federation
AB - Searching for life in astromaterials to be delivered from the future missions to extraterrestrial bodies is undoubtedly related to studies of the properties and signatures of living microbial cells and microfossils on Earth. The Antarctic glacier and Earth permafrost habitats, where living microbial cells preserved viability for millennia years due to entering the anabiotic state, are often regarded as terrestrial analogs of Martian polar subsurface layers. For the future findings of viable microorganisms in samples from extraterrestrial objects, it is important to use a combined methodology that includes classical microbiological methods, plating onto nutrient media, direct epifluorescence and electron microscopy examinations, detection of the elemental composition of cells, PCR and FISH methods. Of great importance is to ensure authenticity of microorganisms (if any in studied samples) and to standardize the protocols used to minimize a risk of external contamination. Although the convincing evidence of extraterrestrial microbial life will may come from the discovery of living cells in astromaterials, biomorphs and microfossils must also be regarded as a target in search of life evidence bearing in mind a scenario that living microorganisms had not been preserved and underwent mineralization. Regarding the vital importance of distinguishing between biogenic and abiogenic signatures and between living and fossil microorganisms in analyzed samples, it is worthwhile to use previously developed approaches based on electron microscopy examinations and analysis of elemental composition of biomorphs in situ.
KW - Astronomy
KW - Cells
KW - Electron microscopy
KW - Extraterrestrial atmospheres
KW - Glaciers
KW - Martian surface analysis
KW - Microorganisms
KW - Astromaterials
KW - Composition of cells
KW - Elemental composition
KW - Microbial life
KW - Microbiology
SN - 0277786X (ISSN)
LA - English
J2 - Proc SPIE Int Soc Opt Eng
M3 - Conference paper
DB - Scopus
N1 - Export Date: 23 February 2023; Cited By: 4; Correspondence Address: S.S. Abyzov; Winogradsky Institute of Microbiology, Russian Academy of Sciences, Moscow, 117312, Pr. 60-letiya Oktyabrya 7/2, Russian Federation; email: abyzov@inmi.host.ru; CODEN: PSISD
ER -

TY - CONF

AU - Greenberg, R.
AU - Stone, W.C.
AU - Kantor, G.
AU - Wettergreen, D.
AU - Durda, D.D.
AU - Franke, E.A.
TI - The depthx project: Pioneering technologies for exploration of extraterrestrial aqueous channels
PY - 2005
T2 - International Astronautical Federation - 56th International Astronautical Congress 2005
VL - 1
SP - 190
EP - 200
UR - https://www.scopus.com/inward/record.uri?eid=2-s2.0-34250889285&partnerID=40&md5=a98aae47353961b6c9a0846f2514c026
AD - University of Arizona, Tucson, AZ, United States
AD - StoneAeroSPACE, Gaithersburg, MD, United States
AD - Carnegie Mellon University, Pittsburgh, PA, United States
AD - Southwest Research Institute, Boulder, CO, United States
AD - Southwest Research Institute, San Antonio, TX, United States
AB - Liquid water is not unique to planet Earth, and the strategy of following the path to water may lead us to extraterrestrial life. On Jupiter's oceanic moon Europa, the most interesting aqueous environments may be within confined channels of liquid through solid strata, such as where Europa's ocean may penetrate hot springs on its floor or tidal cracks in its surface ice. Those settings may provide habitable conditions. Exploration will require development of vehicles with in situ analysis tools for probing such channels. The issues are quite different from those involved in drilling through solids or moving through wide-open submarine spaces. The Deep Phreatic Thermal Explorer (DEPTHX) project is developing such a fully autonomous underwater vehicle, with architecture for intelligent sample collection and in situ discrimination of micro-organisms. Prototypes are being tested in an unusual terrestrial analog to Europa, a site in Mexico with irregular vertical surfaces, an open water column, and potential hydrothermal vents: the 330-meter-deep (or more) Zacatón Cenoté, which contains diverse microbial mats, but remains uncharted. With advanced navigation, detector, sampling, and analysis systems, DEPTHX will be pushing the limits of autonomous robotics. The adaptability and autonomy at the heart of this project will be potentially applicable in a wide range of settings on Earth and in various other sites in the solar system.
KW - Earth (planet)
KW - Microorganisms
KW - Project management
KW - Robotics
KW - Confined channels
KW - Extraterrestrial life
KW - Microbial mats
KW - Submarine spaces
KW - Space research
LA - English
J2 - Int. Astronautical Fed. Int. Astroanautical Congr.

M3 - Conference paper
DB - Scopus
N1 - Export Date: 23 February 2023; Cited By: 4; Correspondence Address: R. Greenberg; University of Arizona, Tucson, AZ, United States; email: greenberg@lpl.arizona.edu
ER -

TY - JOUR
AU - Rodriguez, J.A.P.
AU - Sasaki, S.
AU - Kuzmin, R.O.
AU - Dohm, J.M.
AU - Tanaka, K.L.
AU - Miyamoto, H.
AU - Kurita, K.
AU - Komatsu, G.
AU - Fairén, A.G.
AU - Ferris, J.C.
TI - Outflow channel sources, reactivation, and chaos formation, Xanthe Terra, Mars
PY - 2005
T2 - Icarus
VL - 175
IS - 1
SP - 36
EP - 57
DO - 10.1016/j.icarus.2004.10.025
UR - https://www.scopus.com/inward/record.uri?eid=2-s2.0-17044399674&doi=10.1016%2fj.icarus.2004.10.025&partnerID=40&md5=648ef848ed34c9b90c1abb41c2a324f0
AD - Department of Earth/Planetary Sc., University of Tokyo, Bunkyo-ku, Tokyo 113-0033, 7-3-1 Hongo, Japan
AD - Nakaharaku, Kawasaki 211-0014, 29-1-202 Tajiri-cho, Japan
AD - Vernadsky Institute, Russian Academy of Sciences, Moscow 119991, Kosygin St. 19, Russian Federation
AD - Dept. of Hydrology/Water Resources, University of Arizona, Tucson, AZ 85721, United States
AD - US Geological Survey, Denver, CO 80225, United States
AD - Astrogeology Team, US Geological Survey, Flagstaff, AZ 86001, United States
AD - Earthquake Research Institute, University of Tokyo, Tokyo 113-0032, Japan
AD - Intl. Res. School of Planetary Sci., Dipartimento di Scienze, Universita' d'Annunzio, 65127 Pescara, Viale Pindaro 42, Italy
AD - Centro de Biología Molecular, Universidad Autonoma de Madrid, 28049 Cantoblanco, Madrid, Spain
AB - The undulating, warped, and densely fractured surfaces of highland regions east of Valles Marineris (located north of the eastern Aureum Chaos, east of the Hydraotes Chaos, and south of the Hydaspis Chaos) resulted from extensional surface warping related to ground subsidence, caused when pressurized water confined in subterranean caverns was released to the surface. Water emanations formed crater lakes and resulted in channeling episodes involved in the excavation of Ares, Tiu, and Simud Valles of the eastern part of

the circum-Chryse outflow channel system. Progressive surface subsidence and associated reduction of the subsurface cavernous volume, and/or episodes of magmatic-driven activity, led to increases of the hydrostatic pressure, resulting in reactivation of both catastrophic and non-catastrophic outflow activity. Ancient cratered highland and basin materials that underwent large-scale subsidence grade into densely fractured terrains. Collapse of rock materials in these regions resulted in the formation of chaotic terrains, which occur in and near the headwaters of the eastern circum-Chryse outflow channels. The deepest chaotic terrain in the Hydaspis Chaos region resulted from the collapse of pre-existing outflow channel floors. The release of volatiles and related collapse may have included water emanations not necessarily linked to catastrophic outflow. Basal warming related to dike intrusions, thermokarst activity involving wet sediments and/or dissected ice-enriched country rock, permafrost exposed to the atmosphere by extensional tectonism and channel incision, and/or the injection of water into porous floor material, may have enhanced outflow channel floor instability and subsequent collapse. In addition to the possible genetic linkage to outflow channel development dating back to at least the Late Noachian, clear disruption of impact craters with pristine ejecta blankets and rims, as well as preservation of fine tectonic fabrics, suggest that plateau subsidence and chaos formation may have continued well into the Amazonian Period. The geologic and paleohydrologic histories presented here have important implications, as new mechanisms for outflow channel formation and other fluvial activity are described, and new reactivation mechanisms are proposed for the origin of chaotic terrain as contributors to flooding. Detailed geomorphic analysis indicates that subterranean caverns may have been exposed during chaos formation, and thus chaotic terrains mark prime locations for future geologic, hydrologic, and possible astrobiologic exploration. © 2004 Elsevier Inc. All rights reserved.
KW - Geological processes
KW - Mars
KW - Surface
SN - 00191035 (ISSN)
LA - English
J2 - Icarus
M3 - Article
DB - Scopus
N1 - Export Date: 23 February 2023; Cited By: 82; Correspondence Address: J.A.P. Rodriguez; Department of Earth/Planetary Sci., University of Tokyo, Bunkyo-ku, Tokyo 113-0033, 7-3-1 Hongo, Japan; email: alexis1709@yahoo.com; CODEN: ICRSA
ER -

TY - JOUR
AU - Bergin, E.A.
AU - Melnick, G.J.
TI - What have we learned from SWAS?
PY - 2005
T2 - Proceedings of the International Astronomical Union
VL - 1

IS - S231
SP - 309
EP - 318
DO - 10.1017/S1743921306007307
UR - https://www.scopus.com/inward/record.uri?eid=2-s2.0-63549126903&doi=10.1017%2fS1743921306007307&partnerID=40&md5=0782c19e698bba5854a9b2a59c32555b
AD - University of Michigan, Department of Astronomy, Ann Arbor, MI 48109, 825 Dennison, 500 Church Street, United States
AD - Harvard-Smithsonian Center for Astrophysics, Cambridge, MA 02138, 60 Garden Street, United States
AB - The Submillimeter Wave Astronomy Satellite (SWAS) has recently completed 5.5 years of successful operation. Among the legacies of the mission has been a greater understanding of the abundance and spatial distribution of H 2O and O2 within molecular clouds. We summarize SWAS results and discuss how the measured low abundance of water vapor and non-detections of molecular oxygen are suggestive of a general lack of atomic oxygen in the dense centers of molecular clouds. We also present a new more comprehensive model for the oxygen chemistry in the interstellar medium. © 2006 International Astronomical Union.
KW - Astrobiology
KW - Astrochemistry
KW - ISM: molecules
KW - Molecular processes
KW - Radiative transfer
PB - Cambridge University Press
SN - 17439213 (ISSN)
LA - English
J2 - Proc. Int. Astron. Union
M3 - Article
DB - Scopus
N1 - Export Date: 23 February 2023; Cited By: 1; Correspondence Address: E.A. Bergin; University of Michigan, Department of Astronomy, Ann Arbor, MI 48109, 825 Dennison, 500 Church Street, United States; email: ebergin@umich.edu
ER -

TY - CONF
AU - Bar-Cohen, Y.
AU - Chang, Z.
AU - Sherrit, S.
AU - Badescu, M.
AU - Bao, X.
TI - The Ultrasonic/Sonic Driller/Corer (USDC) as a subsurface drill, sampler and lab-on-a-drill for planetary exploration applications
PY - 2005
T2 - Proceedings of SPIE - The International Society for Optical Engineering
VL - 5762
C7 - 21
SP - 152
EP - 159

DO - 10.1117/12.599703
UR - https://www.scopus.com/inward/record.uri?eid=2-s2.0-25144475566&doi=10.1117%2f12.599703&partnerID=40&md5=9fb3478d0038de19b06c28a265f7f1f0
AD - Jet Propulsion Laboratory (JPL), Caltech, Pasadena, CA, United States
AB - The search for existing or past life in the Universe is one of the most important objectives of NASA's mission. For this purpose, effective instruments that can sample and conduct in-situ astrobiology analysis are being sought. In support of this objective, a novel Ultrasonic/Sonic Driller/Corer (USDC) based mechanism has been developed to probe and sample rocks, ice and soil. The USDC consists of an ultrasonic actuator that impacts a coring or drilling bit at sonic frequencies through the use of an intermediate free-mass. The USDC can produce both a core and powdered cuttings as well as emit elastic waves into the penetrated medium. For planetary exploration, this mechanism has the important advantage of requiring low axial force, virtually no torque, and can be duty cycled to require low average power. This low axial load advantage overcomes a major limitation of planetary sampling in low gravity environments and when operating from lightweight robots and rovers. The low average power operation produces a minimum temperature rise which is important for the acquisition of biologically meaningful samples. The development of the USDC is being pursued on various fronts ranging from analytical modeling to improvements of the mechanism while seeking a wide range of applications. In this paper, the latest status of the USDC development and applications that are underway is reviewed and discussed.
KW - Actuators
KW - Drilling
KW - Gravitation
KW - In situ processing
KW - Soils
KW - Ultrasonic devices
KW - Astrobiology
KW - Subsurface exploration
KW - Ultrasonic/sonic driller/corer (USDC)
KW - URAT
KW - Planetary surface analysis
KW - Drilling
KW - Sampling
KW - Subsurface exploration
KW - Ultrasonic/Sonic Driller/Corer (USDC)
KW - URAT
SN - 0277786X (ISSN)
LA - English
J2 - Proc SPIE Int Soc Opt Eng
M3 - Conference paper
DB - Scopus
N1 - Export Date: 23 February 2023; Cited By: 10; Correspondence Address: Y. Bar-Cohen; Jet Propulsion Laboratory (JPL), Caltech, Pasadena, CA, United States; email: yosi@jpl.nasa.gov; CODEN: PSISD
ER -

TY - JOUR
AU - Schulze-Makuch, D.
AU - Grinspoon, D.H.
TI - Biologically enhanced energy and carbon cycling on Titan?
PY - 2005
T2 - Astrobiology
VL - 5
IS - 4
SP - 560
EP - 567
DO - 10.1089/ast.2005.5.560
UR - https://www.scopus.com/inward/record.uri?eid=2-s2.0-24344496875&doi=10.1089%2fast.2005.5.560&partnerID=40&md5=154b745ed484924717977d870b6222a5
AD - Department of Geological Sciences, Washington State University, Pullman, WA 99164, United States
AD - Department of Space Studies, Southwest Research Institute, Boulder, CO, United States
AB - With the Cassini-Huygens Mission in orbit around Saturn, the large moon Titan, with its reducing atmosphere, rich organic chemistry, and heterogeneous surface, moves into the astrobiological spotlight. Environmental conditions on Titan and Earth were similar in many respects 4 billion years ago, the approximate time when life originated on Earth. Life may have originated on Titan during its warmer early history and then developed adaptation strategies to cope with the increasingly cold conditions. If organisms originated and persisted, metabolic strategies could exist that would provide sufficient energy for life to persist, even today. Metabolic reactions might include the catalytic hydrogenation of photochemically produced acetylene, or involve the recombination of radicals created in the atmosphere by ultraviolet radiation. Metabolic activity may even contribute to the apparent youth, smoothness, and high activity of Titan's surface via biothermal energy. © Mary Ann Liebert, Inc.
KW - Biogenesis
KW - Carbon
KW - Energy Metabolism
KW - Exobiology
KW - Extraterrestrial Environment
KW - Saturn
KW - Space Simulation
KW - carbon
KW - article
KW - astronomy
KW - biogenesis
KW - biology
KW - energy metabolism
KW - metabolism
KW - microclimate
KW - space flight
KW - Biomass
KW - Environment
KW - Life

KW - Metabolism
KW - Titan
SN - 15311074 (ISSN)
C2 - 16078872
LA - English
J2 - Astrobiology
M3 - Article
DB - Scopus
N1 - Export Date: 23 February 2023; Cited By: 77; Correspondence Address: D. Schulze-Makuch; Department of Geological Sciences, Washington State University, Pullman, WA 99164, United States; email: dirksm@wsu.edu
ER -

TY - JOUR
AU - Ellery, A.
AU - Richter, L.
AU - Parnell, J.
AU - Baker, A.
TI - A low-cost approach to the exploration of Mars through a robotic technology demonstrator mission
PY - 2006
T2 - Acta Astronautica
VL - 59
IS - 8-11
SP - 742
EP - 749
DO - 10.1016/j.actaastro.2005.07.052
UR - https://www.scopus.com/inward/record.uri?eid=2-s2.0-33746844060&doi=10.1016%2fj.actaastro.2005.07.052&partnerID=40&md5=937354828706b6a0e86fe035cf84976a
AD - School of Engineering, Kingston University, London, SW15 3DW, Roehampton Vale, United Kingdom
AD - DLR, Institute of Space Simulation, D-51170 Cologne, Germany
AD - Department of Geology and Petroleum Geology, University of Aberdeen, Kings College, Aberdeen, B24 3UE, United Kingdom
AD - Surrey Satellite Technology Ltd., Centre for Satellite Engineering Research, University of Surrey, Guildford, Surrey G2 5XH, United Kingdom
AB - We present a proposed robotic mission to Mars-Vanguard-for the Aurora Arrow programme which combines an extensive technology demonstrator with a high scientific return. The novel aspect of this technology demonstrator is the demonstration of "water mining" capabilities for in situ resource utilisation (ISRU) in conjunction with high-value astrobiological investigation within a low-mass lander package of 70 kg. The basic architecture comprises a small lander, a micro-rover and a number of ground-penetrating moles. This basic architecture offers the possibility of testing a wide variety of generic technologies associated with space systems and planetary exploration. The architecture provides for the demonstration of specific technologies associated with planetary surface exploration, and with the Aurora programme specifically. Technology demonstration of ISRU will be a necessary precursor to any future human mission to Mars. Furthermore, its modest mass overhead allows the re-use of the

already built Mars Express bus, making it a very low-cost option. © 2005.
KW - Astrophysics
KW - Cost effectiveness
KW - Planets
KW - Robotics
KW - Space flight
KW - Spacecraft
KW - Astrobiological investigation
KW - In situ resource utilisation (ISRU)
KW - Robotic mission
KW - Robotic technology
KW - Space research
SN - 00945765 (ISSN)
LA - English
J2 - Acta Astronaut
M3 - Article
DB - Scopus
N1 - Export Date: 23 February 2023; Cited By: 2; Correspondence Address: A. Ellery; School of Engineering, Kingston University, London, SW15 3DW, Roehampton Vale, United Kingdom; email: a.ellery@kingston.ac.uk; CODEN: AASTC
ER -

TY - JOUR
AU - Schröder, C.
AU - Bailey, B.
AU - Klingelhöfer, G.
AU - Staudigel, H.
TI - Fe Mössbauer spectroscopy as a tool in astrobiology
PY - 2006
T2 - Planetary and Space Science
VL - 54
IS - 15
SP - 1622
EP - 1634
DO - 10.1016/j.pss.2006.05.042
UR - https://www.scopus.com/inward/record.uri?eid=2-s2.0-33751085377&doi=10.1016%2fj.pss.2006.05.042&partnerID=40&md5=1ee9a1c916b3ed710dfa558d91c26bb7
AD - Institut für Anorganische und Analytische Chemie, Johannes Gutenberg-Universität, D-55128 Mainz, Staudinger Weg 9, Germany
AD - Institute of Geophysics and Planetary Physics, Scripps Institution of Oceanography, University of California San Diego, La Jolla, CA 92093-0225, United States
AB - The element Fe and Fe-bearing minerals occur ubiquitously throughout the field of astrobiology. Cycling between the various oxidation states of Fe provides a source of energy available for life. Banded iron formations may record the rise of oxygenic photosynthesis. The distribution of Fe between Fe-bearing minerals and its oxidation states can help to characterize and understand ancient environments with respect to the suitability for life by constraining the primary rock type and the redox conditions under which it crystallized, the extent of alteration and weathering, the

type of alteration and weathering products, and the processes and environmental conditions for alteration and weathering. Fe Mössbauer spectroscopy is a powerful tool to investigate Fe-bearing compounds. It can identify Fe-bearing minerals, determine Fe oxidation states with high accuracy, quantify the distribution of Fe between mineralogical phases, and provide clues about crystallinity and particle sizes. Two miniaturized Mössbauer spectrometers are on board of the NASA Mars Exploration Rovers Spirit and Opportunity. The Fe-bearing minerals goethite, an iron oxide-hydroxide, and jarosite, an iron hydroxide sulfate, were identified by Mössbauer spectroscopy in Gusev Crater and at Meridiani Planum, respectively, providing in situ proof of an aqueous history of the two landing sites and constraints on their habitability. Hematite identified by Mössbauer spectroscopy at both landing sites adds further evidence for an aqueous history. On Earth, Mössbauer spectroscopy was used to monitor possibly microbially-induced changes of Fe-oxidation states in basaltic glass samples exposed at the Loihi Seamount, a deep sea hydrothermal vent system, which might be analogous to possible extraterrestrial habitats on ancient Mars or the Jovian moon Europa today. © 2006 Elsevier Ltd. All rights reserved.
KW - Crystallization
KW - Earth (planet)
KW - Iron
KW - Moon
KW - Mossbauer spectroscopy
KW - Photosynthesis
KW - Planets
KW - Redox reactions
KW - Astrobiology
KW - Extraterrestrial habitats
KW - Goethite
KW - Hematite
KW - Oxidation state
KW - Space research
KW - Astrobiology
KW - Biogeochemistry
KW - Hydrothermal vent
KW - Mars
KW - Mössbauer spectroscopy
SN - 00320633 (ISSN)
LA - English
J2 - Planet. Space Sci.
M3 - Article
DB - Scopus
N1 - Export Date: 23 February 2023; Cited By: 14; Correspondence Address: C. Schröder; Institut für Anorganische und Analytische Chemie, Johannes Gutenberg-Universität, D-55128 Mainz, Staudinger Weg 9, Germany; email: schroedc@uni-mainz.de; CODEN: PLSSA
ER -

TY - JOUR
AU - Barbieri, R.
AU - Stivaletta, N.
AU - Marinangeli, L.

AU - Ori, G.G.
TI - Microbial signatures in sabkha evaporite deposits of Chott el Gharsa (Tunisia) and their astrobiological implications
PY - 2006
T2 - Planetary and Space Science
VL - 54
IS - 8
SP - 726
EP - 736
DO - 10.1016/j.pss.2006.04.003
UR - https://www.scopus.com/inward/record.uri?eid=2-s2.0-33745813149&doi=10.1016%2fj.pss.2006.04.003&partnerID=40&md5=f52a002a534ae74ae8f9a5c6cb8778bf
AD - Dipartimento di Scienze della Terra e Geologico-Ambientali, Università di Bologna, 40126 Bologna, Via Zamboni 67, Italy
AD - International Research School of Planetary Sciences, Università D'Annunzio, 65127 Pescara, Viale Pindaro 42, Italy
AB - This study investigates the geomicrobiological potential of Upper Pleistocene evaporite deposits of the Chott el Gharsa, a wide continental sabkha in southern Tunisia. Organic and inorganic-derived biosignatures are mostly contained in microcrystalline, laminated gypsum lithofacies consisting of light/dark alternations of concordant laminae, which have precipitated from high salt concentrated waters. These biosignatures include mineralized microbial-interpreted morphologies, such as mucilage, rods, and microfibers, and dumbbell morphologies in the hollow cores of dolomite crystals that are associated with sulfates. Mineral products that are induced by microbial activity and their organic compounds lead to the formation of lenticular-shaped gypsum crystals, with a high length/width ratio, dolomite precipitation and formation of pyrite framboids. Morphological and structural aspects of these biosignatures, and their composition, in laminated, dolomite-rich sulfate deposits could be detected through microscopic investigations and micro-analyses performed by the instrumentation that is planned for ongoing Mars sample return missions. © 2006 Elsevier Ltd. All rights reserved.
KW - Crystalline materials
KW - Deposition
KW - Geochemistry
KW - Gypsum
KW - Microbiology
KW - Microorganisms
KW - Microscopic examination
KW - Precipitation (chemical)
KW - Astrobiology
KW - Chott el Gharsa
KW - Dolomite
KW - Microbial biosignatures
KW - Sabkha environment
KW - Sulfate
KW - Astrophysics
KW - Astrobiology
KW - Chott el Gharsa
KW - Dolomite

KW - Microbial biosignatures
KW - Sabkha environment
KW - Sulfate
SN - 00320633 (ISSN)
LA - English
J2 - Planet. Space Sci.
M3 - Article
DB - Scopus
N1 - Export Date: 23 February 2023; Cited By: 46; Correspondence Address: R. Barbieri; Dipartimento di Scienze della Terra e Geologico-Ambientali, Università di Bologna, 40126 Bologna, Via Zamboni 67, Italy; email: roberto.barbieri@unibo.it; CODEN: PLSSA
ER -

TY - JOUR
AU - Knoll, A.H.
AU - Grotzinger, J.
TI - Water on Mars and the prospect of martian life
PY - 2006
T2 - Elements
VL - 2
IS - 3
SP - 169
EP - 173
DO - 10.2113/gselements.2.3.169
UR - https://www.scopus.com/inward/record.uri?eid=2-s2.0-34247336040&doi=10.2113%2fgselements.2.3.169&partnerID=40&md5=0d5c14f0ad3e215c46100a6b0d8d2cd0
AD - Department of Organismic and Evolutionary Biology, Harvard University, Cambridge, MA 02138, United States
AD - Division of Geological and Planetary Sciences, California Institute of Technology, Pasadena, CA 91125, United States
AB - Evidence of water on Mars dates back to the first observations of channeled landscapes made by Mariner 9 and Viking. More recent images from Mars Global Surveyor and Mars Express strikingly confirm that fluids have sculpted the Martian surface at least episodically through its history. The Mars Exploration Rovers Opportunity and Spirit have added evidence for extensive rock-water chemical interactions in the regions where these remote geologists landed, while OMEGA and THEMIS have shown that similar processes took place in many parts of the planet. Because of the close relationship between water and biological activity on Earth, such observations have been taken as hopeful signs that Mars, as well, might once have supported life and, indeed, might still do so in subterranean oases. There is, however, much more to consider. Water appears to be necessary for life, at least as it exists on Earth and can be contemplated on Mars, but it does not, by itself, insure habitability. In this paper, we review the broader requirements for biological activity as they relate to water and use these to constrain astrobiological inferences about Mars.
KW - Emmelichthyidae
KW - Astrobiology
KW - Martian surface analysis
KW - Water

KW - Chemical interactions
KW - Life
KW - Mars
KW - Mars Exploration Rover
KW - Mars global surveyor
KW - Martian surface
KW - Meridiani
KW - Water on Mars
KW - bioactivity
KW - Mars Pathfinder
KW - origin of life
KW - water availability
KW - water-rock interaction
KW - Bioactivity
KW - Astrobiology
KW - Life
KW - Mars
KW - Meridiani
KW - Water
PB - Mineralogical Society of America
SN - 18115209 (ISSN)
LA - English
J2 - Elements
M3 - Article
DB - Scopus
N1 - Export Date: 23 February 2023; Cited By: 34; Correspondence Address: A.H. Knoll; Department of Organismic and Evolutionary Biology, Harvard University, Cambridge, MA 02138, United States; email: aknoll@fas.harvard.edu
ER -

TY - CONF
AU - Debus, A.
TI - Prevention of backward contamination for mars sample return missions: Risks and technical challenges
PY - 2006
T2 - AIAA 57th International Astronautical Congress, IAC 2006
VL - 3
SP - 1469
EP - 1478
UR - https://www.scopus.com/inward/record.uri?eid=2-s2.0-41149108572&partnerID=40&md5=aea76c68ce35bde4adf97b9abfc688b9
AD - CNES (Centre National d'Etudes Spatiales) Toulouse
AB - The exploration of the solar system needs today space missions towards other worlds, and the scientific investigation of extraterrestrial bodies requires the collection and the analysis of extra-terrestrial samples. Althought in-situ analysis of samples can be envisaged, this kind of investigation is nevertheless limited by the size and the weight of some useful and essential instruments which can only be used on Earth. For this reason, sample return missions are now proposed. From the planetary protection point of view, if the prevention of forward biological contamination has to be ensured as it has been done for a lot of previous missions, the prevention of back-contamination risks is one of the more important

topic to consider presently. Returning and analysing safely extraterrestrial samples to Earth needs engineering studies, new developments and analysis. Returning samples as safely as possible has implications on the mission design, the space systems design and shall foresee the possibility to implement onboard specific means. Analysing safely samples on Earth needs a quarantine phase in a specific facility to be defined, designed and certified, and in which analysis protocols shall be conducted in order to state with the highest possible degree of confidence, avoiding any biohazard. All critical aspects concerning a sample return mission have consequently to be identified and analysed in order to check the feasibility from the technical and scientific point of view, without taking into account cost elements at this level.
KW - Astrobiology
KW - Extraterrestrial atmospheres
KW - Solar system
KW - Space research
KW - Biological contamination
KW - Extra-terrestrial samples
KW - Extraterrestrial samples
KW - Space missions
KW - Space rendezvous
SN - 978-160560039-0 (ISBN)
LA - English
J2 - AIAA Int. Astronaut. Congr. IAC
M3 - Conference paper
DB - Scopus
N1 - Export Date: 23 February 2023; Cited By: 1; Correspondence Address: A. Debus; CNES (Centre National d'Etudes Spatiales) Toulouse, France; email: andre.debus@cnes.fr
ER -

TY - JOUR
AU - Raymond, S.N.
AU - Barnes, R.
AU - Kaib, N.A.
TI - Predicting planets in known extrasolar planetary systems. III. Forming terrestrial planets
PY - 2006
T2 - Astrophysical Journal
VL - 644
IS - 2 II
SP - 1223
EP - 1231
DO - 10.1086/503594
UR - https://www.scopus.com/inward/record.uri?eid=2-s2.0-33748531945&doi=10.1086%2f503594&partnerID=40&md5=f29b792b048ac3faa3770a56f1162f44
AD - Department of Astronomy, University of Washington, Seattle, WA 98195, United States
AD - Laboratory for Atmospheric and Space Physics, University of Colorado, Boulder, CO 80309-0590, United States
AD - NASA Astrobiology Institute
AD - Lunar and Planetary Laboratory, University of Arizona, Tucson,

AZ 85721, United States
AB - Recent results have shown that many of the known extrasolar planetary systems contain regions that are stable for both Earth-mass and Saturn-mass planets. Here we simulate the formation of terrestrial planets in four planetary systems, 55 Cancri, HD 38529, HD 37124, and HD 74156, under the assumption that these systems of giant planets are complete and that their orbits are well determined. Assuming that the giant planets formed and migrated quickly, terrestrial planets may form from a second generation of planetesimals. In each case, Moon- to Mars-sized planetary embryos are placed in between the giant planets and evolved for 100 Myr. We find that planets form relatively easily in 55 Cnc, with masses up to 0.6 M⊕ and, in some cases, substantial water content and orbits in the habitable zone. HD 38529 is likely to support an asteroid belt, but no terrestrial planets of significant mass. No terrestrial planets form in HD 37124 and HD 74156, although in some cases 1-2 lone embryos survive for 100 Myr. If migration occurred later, depleting the planetesimal disk, then massive terrestrial planets are unlikely to form in any of these systems. ©2006. The American Astronomical Society. All rights reserved.
KW - Astrobiology
KW - Methods: n-body simulations
KW - Planets and satellites: formation
PB - Institute of Physics Publishing
SN - 0004637X (ISSN)
LA - English
J2 - Astrophys. J.
M3 - Article
DB - Scopus
N1 - Export Date: 23 February 2023; Cited By: 46; Correspondence Address: S. N. Raymond; Laboratory for Atmospheric and Space Physics, University of Colorado, Boulder, CO 80309-0590, United States; email: raymond@lasp.colorado.edu
ER -

TY - CONF
AU - Ng, E.W.
AU - Skiles, J.W.
TI - Why do we explore space?
PY - 2006
T2 - Collection of Technical Papers - Space 2006 Conference
VL - 2
SP - 1105
EP - 1114
DO - 10.2514/6.2006-7313
UR - https://www.scopus.com/inward/record.uri?eid=2-s2.0-33846557531&doi=10.2514%2f6.2006-7313&partnerID=40&md5=c670c2d6047835b0ed3ff3087f8897dc
AD - JPL, California Institute of Technology, South Pasadena, CA 91031, P. O. Box 1292, United States
AD - Mail Stop 239-20, NASA Ames Research Center, Moffett Field, CA 94040, United States
AB - The launch of the Soviet Sputnik satellite in 1957 challenged the American space enterprise to a race for political and

technological superiority. During the Cold War era, the space program had a very clear goal - to show the world that we were the premier force and player in this new frontier. The American public could, therefore, relate to such a simplistic goal and largely was very supportive of the US space program. Since the end of the Cold War, the raison d'etre for space exploration has been less clear and not as well articulated. This paper is part of a dialogue hoping to solicit input from the public domain on the topic of space exploration. We first examine a previous study on some of the "why's" anticipated by the American public. Then we propose a triumvirate perspective to seek a balance among the romantic, pragmatic and scientific aspects of space faring ventures. Finally, we suggest a somewhat simplistic message that can be more easily related to the common person on the street. We assert that we go to space to "explore the Heavens, enhance the Earth and enrich humankind", and cite numerous concrete examples to support these three themes. (Disclaimer: these are personal ideas and opinions of the authors and do not represent an official NASA position. All references to NASA information are from NASA web pages or in the public domain. This paper is written from an American vantage point due to the authors' experience with the American space agency.).
KW - Earth (planet)
KW - Public policy
KW - Remote sensing
KW - Satellites
KW - Social aspects
KW - Telescopes
KW - Astrobiology
KW - Hubble telescope
KW - Soviet Sputnik satellite
KW - Space sciences
KW - Space venture spin-offs
KW - Space applications
KW - Astrobiology
KW - Challenger
KW - Columbia
KW - Earth science
KW - Enrich humankind
KW - Explore the heavens
KW - Hubble telescope
KW - Remote sensing of Earth
KW - Space science
KW - Space venture spin-offs
KW - Space-borne observatories
KW - Why explore space
PB - American Institute of Aeronautics and Astronautics Inc.
SN - 1563478242 (ISBN); 978-156347824-6 (ISBN)
LA - English
J2 - Coll. Technic. Papers Space 2006 Conf.
M3 - Conference paper
DB - Scopus
N1 - Export Date: 23 February 2023; Cited By: 0
ER -

TY - BOOK
AU - Stackebrandt, E.
TI - Molecular identification, systematics, and population structure of prokaryotes
PY - 2006
T2 - Molecular Identification, Systematics, and Population Structure of Prokaryotes
SP - 1
EP - 320
DO - 10.1007/978-3-540-31292-5
UR - https://www.scopus.com/inward/record.uri?eid=2-s2.0-84900051972&doi=10.1007%2f978-3-540-31292-5&partnerID=40&md5=930a333ef7af7d16baae77c37f8495ca
AD - DSMZGmbH, 38124 Braunschweig, Mascheroder Weg 1b, Germany
AB - Systematic biology has a far wider application than merely the provision of a reliable classification scheme for new strains. With the framework of the hierarchic system stabilizing, genomes, noncoding regions, and genes and their products can now be evaluated in an evolutionary context. This book summarizes recent developments in the molecular characterization of cultured and as-yet uncultured prokaryotes, emphasizing the strengths and weaknesses of individual approaches. The chapters of the book are compiled to stimulate students to enter the field of bacterial diversity, presenting a broad spectrum of fascinating multifaceted disciplines that illuminate the paths to ecosystem functioning, communication within communities, symbiosis, life in extreme environments, astrobiology, and more. © Springer-Verlag Berlin Heidelberg 2006.
PB - Springer Berlin Heidelberg
SN - 3540231552 (ISBN); 978-354023155-4 (ISBN)
LA - English
J2 - Molec. Identification, Systematics, and Popul. Struct. of Prokaryotes
M3 - Book
DB - Scopus
N1 - Export Date: 23 February 2023; Cited By: 11; Correspondence Address: E. Stackebrandt; DSMZGmbH, 38124 Braunschweig, Mascheroder Weg 1b, Germany; email: erko@dsmz.de
ER -

TY - JOUR
AU - Hamdani, S.
AU - Arnold, L.
AU - Foellmi, C.
AU - Berthier, J.
AU - Billeres, M.
AU - Briot, D.
AU - François, P.
AU - Riaud, P.
AU - Schneider, J.
TI - Biomarkers in disk-averaged near-UV to near-IR Earth spectra using Earthshine observations
PY - 2006
T2 - Astronomy and Astrophysics
VL - 460

IS - 2
SP - 617
EP - 624
DO - 10.1051/0004-6361:20065032
UR - https://www.scopus.com/inward/record.uri?eid=2-s2.0-33845422136&doi=10.1051%2f0004-6361%3a20065032&partnerID=40&md5=5d79b546cb5764a3981c7eeee9d12b53
AD - Observatoire de Haute Provence, CNRS, 04870 Saint Michel l'Observatoire, France
AD - ESO, Santiago 19, Casilla 19001, Chile
AD - Laboratoire d'Astrophysique, Observatoire de Grenoble, 38041 Grenoble Cedex 9, BP 53, France
AD - IMCCE, Observatoire de Paris, 75014 Paris, 77 Avenue Denfert-Rochereau, France
AD - Observatoire de Paris-Meudon, 92195 Meudon, 5 place Jules Janssen, France
AD - Institut d'Astrophysique et de Géophysique de Liège, Université de Liège, 4000 Sart-Tilman, Allée du 6 Août, Belgium
AD - EGO/VIRGO, 56021 S. Stefano a Macerata - C. PI, Via E. Amaldi, Italy
AB - Context. The detection of exolife is one of the goals of very ambitious future space missions or extremely large ground-based telescopes that aim to take direct images of Earth-like planets. While associations of simple molecules present in the planet's atmosphere (O2, O3, CO 2 etc.) have been identified as possible global biomarkers, we analyse here the detectability of vegetation on a global scale on Earth's surface. Aims. Considering its specific reflectance spectrum showing a sharp edge around 700 nm, vegetation can be considered as a potential global biomarker. This work, based on observational data, aims to characterise and to quantify this signature in the disk-averaged Earth's spectrum. Methods. Earthshine spectra have been used to test the detectability of the "Vegetation Red Edge" (VRE) in the Earth spectrum. We obtained reflectance spectra from near UV (320 nm) to near IR (1020 nm) for different Earth phases (continents or oceans seen from the Moon) with EMMI on the NTT at ESO/La Silla, Chile. We accurately correct the sky background and take into account the phase-dependent colour of the Moon. VRE measurements require a correction of the ozone Chappuis absorption band and Rayleigh plus aerosol scattering. Results. The near-UV spectrum shows a dark Earth below 350 nm due to the ozone absorption. The Vegetation Red Edge is observed when forests are present (4.0% for Africa and Europe), and is lower when clouds and oceans are mainly visible (1.3% for the Pacific Ocean). Errors are typically ±0.5, and ±1.5 in the worst case. We discuss the different sources of errors and bias and suggest possible improvements. Conclusions. We showed that measuring the VRE or an analog on an Earth-like planet remains very difficult (photometric relative accuracy of 1 % or better). It remains a small feature compared to atmospheric absorption lines. A direct monitoring from space of the global (disk-averaged) Earth's spectrum would provide the best VRE follow-up. © ESO 2006.
KW - Biomarkers
KW - Earth (planet)
KW - Earth atmosphere

KW - Infrared radiation
KW - Moon
KW - Space applications
KW - Ultraviolet radiation
KW - Vegetation
KW - Astrobiology
KW - Earth phases
KW - Earthshine observations
KW - Astrophysics
KW - Astrobiology
KW - Earth
KW - Moon
SN - 14320746 (ISSN)
LA - English
J2 - Astron. Astrophys.
M3 - Article
DB - Scopus
N1 - Export Date: 23 February 2023; Cited By: 43; Correspondence Address: S. Hamdani; EGO/VIRGO, 56021 S. Stefano a Macerata - C. PI, Via E. Amaldi, Italy; email: hamdani@obs-hp.fr; CODEN: AAEJA
ER -

TY - JOUR
AU - Brake, M.
TI - On the plurality of inhabited worlds: A brief history of extraterrestrialism
PY - 2006
T2 - International Journal of Astrobiology
VL - 5
IS - 2
SP - 99
EP - 107
DO - 10.1017/S1473550406002989
UR - https://www.scopus.com/inward/record.uri?eid=2-s2.0-33750355090&doi=10.1017%2fS1473550406002989&partnerID=40&md5=2a9aa107235ebdad41ad7ee79c890a42
AD - Centre for Astronomy and Science Education, University of Glamorgan, Trefforest, 4 Forest Grove, United Kingdom
AB - This paper delineates the cultural evolution of the ancient idea of a plurality of inhabited worlds, and traces its development through to contemporary extraterrestrialism, with its foundation in the physical determinism of cosmology, and its attendant myths of alien contact drawn from examples of British film and fiction. We shall see that, in the evolving debate of the existence of extraterrestrial life and intelligence, science and science fiction have benefited from an increasingly symbiotic relationship. Modern extraterrestrialism has influenced both the scientific searches for extraterrestrial intelligence (SETI), and become one of the most pervasive cultural myths of the 20th century. Not only has pluralism found a voice in fiction through the alien, but fiction has also inspired science to broach questions in the real world. © 2006 Cambridge University Press.
KW - cosmology
KW - cultural history

KW - education
KW - exobiology
KW - extraterrestrial matter
KW - Communication
KW - Culture
KW - Education
SN - 14753006 (ISSN)
LA - English
J2 - Int. J. Astrobiology
M3 - Article
DB - Scopus
N1 - Export Date: 23 February 2023; Cited By: 9; Correspondence Address: M. Brake; Centre for Astronomy and Science Education, University of Glamorgan, Trefforest, 4 Forest Grove, United Kingdom; email: mbrake@glam.ac.uk
ER -

TY - CONF
AU - Choi, E.
AU - Daly, M.
AU - Tripp, J.
AU - Richards, R.
AU - Ghafoor, N.
AU - Sallaberger, C.
TI - The Canadian meteorology(MET) package for the phoenix mars lander
PY - 2006
T2 - AIAA 57th International Astronautical Congress, IAC 2006
VL - 2
SP - 1088
EP - 1093
DO - 10.2514/6.iac-06-a3.3.03
UR - https://www.scopus.com/inward/record.uri?eid=2-s2.0-40549089511&doi=10.2514%2f6.iac-06-a3.3.03&partnerID=40&md5=daeb421b2ebc4c4578371358181d5b2a
AD - MDA, Brampton, Canada
AD - Optech Incorporated, Toronto, Canada
AB - In 2003, NASA selected Phoenix as the first mission of the Mars Scout program. Scheduled for launch in 2007, Phoenix will land in the northern Vastitas Borealis region to assess the astrobiological potential at high latitudes and verify the evidence of subsurface water ice detected by the gamma ray spectrometer aboard the Mars Odyssey orbiter. Phoenix will also investigate the mineralogy and geochemistry of the soil and study the atmosphere. Canada's contribution to Phoenix will be the Meteorological (MET) package, consisting of a lidar to measure the location and extent of clouds and the distribution of scatterers in the atmosphere as well as sensors for measuring pressure and temperature. MET will provide Canadian scientists with a unique opportunity to study the Martian atmosphere and enhance our understanding of the planet in key areas of Canadian expertise. It will also contribute to investigations that have been determined by MEPAG to be of crucial importance: to determine the present distribution of water, carbon dioxide, and dust on Mars; to determine trends in the Martian climate; to

determine the escape rates of atmospheric species, and; to measure the parameters that affect the atmosphere. This paper describes Canada's participation in the mission, provides details about the instrument, and summarizes the current status of the program.
KW - Astrobiology
KW - Carbon dioxide
KW - Martian surface analysis
KW - NASA
KW - Atmospheric species
KW - Martian atmosphere
KW - Phoenix mars lander
KW - Subsurface water
KW - Planetary landers
PB - American Institute of Aeronautics and Astronautics Inc.
SN - 978-160560039-0 (ISBN)
LA - English
J2 - AIAA Int. Astronaut. Congr. IAC
M3 - Conference paper
DB - Scopus
N1 - Export Date: 23 February 2023; Cited By: 2; Correspondence Address: E. Choi; MDA, Brampton, Canada; email: eric.choi@mdacorporation.com
ER -

TY - JOUR
AU - Zheng, W.
AU - Jewitt, D.
AU - Kaiser, R.I.
TI - Temperature dependence of the formation of hydrogen, oxygen, and hydrogen peroxide in electron-irradiated crystalline water ice
PY - 2006
T2 - Astrophysical Journal
VL - 648
IS - 1 I
SP - 753
EP - 761
DO - 10.1086/505901
UR - https://www.scopus.com/inward/record.uri?eid=2-s2.0-33748793084&doi=10.1086%2f505901&partnerID=40&md5=8e99fc4884ea78e858e093ef73607f53
AD - Institute for Astronomy, University of Hawaii at Manoa, Honolulu, HI 96822, United States
AD - Department of Chemistry, University of Hawaii at Manoa, Honolulu, HI 96822, United States
AB - We conducted a systematic study of the irradiation of crystalline water ice in an ultrahigh vacuum chamber at pressures of about 10-10 torr. Crystalline water ices of 115 ± 30 nm thickness were irradiated with energetic electrons at 12,40,60, and 90 K to simulate energetic particle interaction with solar system and interstellar water ices. The production rates of molecular hydrogen (H2), molecular oxygen (O2), and hydrogen peroxide (H2O2) decrease as the temperature rises from 12 to 90 K. These findings strongly indicate a thermal, possibly diffusion-controlled component of the reaction mechanism, which could facilitate the back-reaction of the

primarily formed irradiation products such as the recombination of atomic hydrogen (H) with the hydroxyl radical (OH) to "recycle" water. This study underlines the necessity to provide temperature-dependent rate constants on the formation of molecules in planetary and interstellar ices and to include nonequilibrium as well as thermal chemistry in reaction models simulating the chemical processing of extraterrestrial ices. We estimate the concentrations of newly formed H2O2 molecules in the irradiated water ice at 90 K to be in the same order of magnitude as the concentration of H2O2 observed on the Galilean satellites.
KW - Astrobiology
KW - Astrochemistry
KW - Comets: general
KW - Cosmic rays
KW - Infrared: ISM
KW - ISM: clouds
KW - ISM: molecules
KW - Methods: laboratory
KW - Molecular processes
PB - Institute of Physics Publishing
SN - 0004637X (ISSN)
LA - English
J2 - Astrophys. J.
M3 - Article
DB - Scopus
N1 - Export Date: 23 February 2023; Cited By: 59
ER -

TY - CONF
AU - Brown, A.J.
AU - Storrie-Lombardi, M.
TI - MR PRISM - A spectral analysis tool for the PRISM
PY - 2006
T2 - Proceedings of SPIE - The International Society for Optical Engineering
VL - 6309
C7 - 63090V
DO - 10.1117/12.677107
UR - https://www.scopus.com/inward/record.uri?eid=2-s2.0-33750711778&doi=10.1117%2f12.677107&partnerID=40&md5=4f616cb9fd4dc9a53d92ed1d5942acca
AD - SETI Institute, Mountain View, CA 94043, 515 N. Whisman Rd, United States
AD - Kinohi Institute, Pasadena, CA 91101, United States
AB - We describe a computer application designed to analyze hyperspectral data collected by the Compact Infrared Spectrometer for Mars (CRISM). The application links the spectral, imaging and mapping perspectives on the eventual CRISM dataset by presenting the user with three different ways to analyze the data. One of the goals when developing this instrument is to build in the latest algorithms for detection of spectrally compelling targets on the surface of the Red Planet, so they may be available to the Planetary Science community without cost and with a minimal learning barrier to cross. This will allow the Astrobiology community to look for targets of

interest such as hydrothermal minerals, sulfate minerals and hydrous minerals and be able to map the extent of these minerals using the most up-to-date and effective algorithms. The application is programmed in Java and will be made available for Windows, Mac and Linux platforms. Users will be able to embed Groovy scripts into the program in order to extend its functionality. The first collection of CRISM data will occur in September of 2006 and this data will be made publicly available six months later via the Planetary Datasystem (PDS). Potential users in the community should therefore look forward to a release date mid-2007. Although exploration of the CRISM data set is the motivating force for developing these software tools, the ease of writing additional Groovy scripts to access other data sets makes the tools useful for mineral exploration, crop management, and characterization of extreme environments here on Earth or other terrestrial planets. The system can be easily implemented for use by high school, college, and graduate level students.
KW - Algorithms
KW - Database systems
KW - Martian surface analysis
KW - Planets
KW - Hyperspectral
KW - Planetary Datasystem (PDS)
KW - Terrestrial planets
KW - Astronomy
KW - CRISM
KW - Groovy
KW - Hyperspectral
KW - Java
KW - Mars
KW - MR PRISM
KW - MRO
SN - 0277786X (ISSN); 0819463884 (ISBN); 978-081946388-3 (ISBN)
LA - English
J2 - Proc SPIE Int Soc Opt Eng
M3 - Conference paper
DB - Scopus
N1 - Export Date: 23 February 2023; Cited By: 8; CODEN: PSISD
ER -

TY - JOUR
AU - Brake, M.
AU - Hook, N.
TI - Aliens and time in the machine age
PY - 2006
T2 - International Journal of Astrobiology
VL - 5
IS - 4
SP - 277
EP - 286
DO - 10.1017/S1473550406003351
UR - https://www.scopus.com/inward/record.uri?eid=2-s2.0-33847291801&doi=10.1017%2fS1473550406003351&partnerID=40&md5=b89dfc550e0b64891eb0a39596a93aca

AD - Centre for Astronomy and Science Education, University of Glamorgan, Trefforest, 4 Forest Grove, United Kingdom
AB - The 19th century saw sweeping changes for the development of astrobiology, both in the constituency of empirical science encroaching upon all aspects of life and in the evolution of ideas, with Lyell's Principles of Geology radically raising expectation of the true age of the Earth and the drama of Darwinism questioning biblically literalist accounts of natural history. This paper considers the popular culture spun on the crackling loom of the emergent aspects of astrobiology of the day: Edward Bulwer-Lytton's The Coming Race (1871), which foretold the race of the future, and satirist Samuel Butler's anticipation of machine intelligence, 'Darwin Among the Machines', in his Erewhon (1872). Finally, we look at the way Darwin, Huxley and natural selection travelled into space with French astronomer Camille Flammarion's immensely popular Récits de l'infini (Stories of Infinity, 1872), and the social Darwinism of H.G. Wells' The Time Machine (1895) and The War of the Worlds (1898). These works of popular culture presented an effective and inspiring communication of science; their crucial discourse was the reducible gap between the new worlds uncovered by science and exploration and the fantastic strange worlds of the imagination. As such they exemplify a way in which the culture and science of popular astrobiology can be fused. © 2006 Cambridge University Press.
KW - communication
KW - culture
KW - education
KW - exobiology
KW - Communication
KW - Culture
KW - Education
SN - 14753006 (ISSN)
LA - English
J2 - Int. J. Astrobiology
M3 - Article
DB - Scopus
N1 - Export Date: 23 February 2023; Cited By: 2; Correspondence Address: M. Brake; Centre for Astronomy and Science Education, University of Glamorgan, Trefforest, 4 Forest Grove, United Kingdom; email: mbrake@glam.ac.uk
ER -

TY - CONF
AU - Rummel, J.D.
TI - Special regions in mars exploration: Problems and potential
PY - 2006
T2 - AIAA 57th International Astronautical Congress, IAC 2006
VL - 2
SP - 1143
EP - 1147
DO - 10.2514/6.iac-06-a3.3.09
UR - https://www.scopus.com/inward/record.uri?eid=2-s2.0-40549097549&doi=10.2514%2f6.iac-06-a3.3.09&partnerID=40&md5=271b1175c7a28d9a1944c39417935bb0

AD - Science Mission Directorate, NASA Headquarters, Washington, DC 20546, United States
AB - "Special Regions" on Mars are areas designated in the COSPAR planetary protection policy as areas that may support Earth microbes inadvertently introduced to Mars, or that may have a high probability of supporting indigenous martian life. Since absolutely nothing is known about martian life, the operational definition of a special region is a place that may allow the formation and maintenance of liquid water, on or under the surface of Mars. This paper will review the special regions concept, the implications of recent recommendations on avoiding them, and the work of the Mars science community in providing an operational definition of those area on Mars that are "non-special.".
KW - Interplanetary flight
KW - Martian surface analysis
KW - Space rendezvous
KW - Space research
KW - Water analysis
KW - Liquid water
KW - Mars exploration
KW - Mars science community
KW - Planetary protection policy
KW - Astrobiology
PB - American Institute of Aeronautics and Astronautics Inc.
SN - 978-160560039-0 (ISBN)
LA - English
J2 - AIAA Int. Astronaut. Congr. IAC
M3 - Conference paper
DB - Scopus
N1 - Export Date: 23 February 2023; Cited By: 0; Correspondence Address: J.D. Rummel; Science Mission Directorate, NASA Headquarters, Washington, DC 20546, United States; email: jrummel@hq.nasa.gov
ER -

TY - CONF
AU - Hubbard, G.S.
AU - Kovacs, G.T.A.
AU - Chu-Thielbar, L.
TI - Detecting the fingerprints of life
PY - 2006
T2 - AIAA 57th International Astronautical Congress, IAC 2006
VL - 13
SP - 8605
EP - 8615
UR - https://www.scopus.com/inward/record.uri?eid=2-s2.0-40749144501&partnerID=40&md5=d40ac5e515b587b21b63e01428a7bf76
AD - Stanford University, Mountain View, CA 94043, 515 N. Whisman Rd., United States
AD - Department of Electrical Engineering, Medicine Stanford University, 330 Serra Mall, Stanford, CA 94305-4075, United States
AD - SETI Institute, Mountain View, CA 94043, 515 N. Whisman Rd., United States
AB - Astrobiology asks the following question: "Are the living

organisms that inhabit planet earth the only life that exists or has ever existed in the universe; in other words, are we alone?" We will examine this question by addressing where and how to look for the fingerprints of life. A recurring theme in mission and instrument design is to determine the location of the scientific "hotspot" which offers the best possibility of past or current living systems on other worlds. This exploration challenge falls into at least two categories: "pale blue dot" detection which requires understanding how a biosphere might be remotely detected through a telescope, and in situ life detection on reachable solar system bodies such as on or below the surface of Mars, in the putative oceans of Europa or even prebiotic compounds on comets, the surface of Titan and others. We will survey current thoughts on mission design and instrument development with particular attention to current in situ approaches derived from sensors used in detection of infectious agents and the world of biotechnology. We will also propose areas and directions of future research and development that appear promising.
KW - Biotechnology
KW - Living systems studies
KW - Pattern recognition
KW - Sensors
KW - Biosphere
KW - Instrument design
KW - Living organisms
KW - Astrobiology
SN - 978-160560039-0 (ISBN)
LA - English
J2 - AIAA Int. Astronaut. Congr. IAC
M3 - Conference paper
DB - Scopus
N1 - Export Date: 23 February 2023; Cited By: 0; Correspondence Address: G. S. Hubbard; Stanford University, Mountain View, CA 94043, 515 N. Whisman Rd., United States; email: SHubbard@seti.org
ER -

TY - CONF
AU - Gao, Y.
AU - Ellery, A.
AU - Jaddou, M.
AU - Vincent, J.
TI - Deployable wood wasp drill for planetary subsurface sampling
PY - 2006
T2 - IEEE Aerospace Conference Proceedings
VL - 2006
C7 - 1655756
UR - https://www.scopus.com/inward/record.uri?eid=2-s2.0-34047094505&partnerID=40&md5=1c7b32a3ba84002b0f2ce0dc122973d2
AD - Surrey Space Centre, University of Surrey, Guildford, United Kingdom
AD - Centre for Biomimetic and Natural Technologies, University of Bath, United Kingdom
AB - Growing interest in planetary subsurface exploration has prompted an examination of advanced drilling technologies. One of the major limitations of sampling in low gravity environments (such

as Mars, asteroids, etc) using conventional rotary drills is the need for high axial force, which suffers from big overhead mass, buckling problem, and power hungriness. Though drills using percussive motion may operate in low mass and power, the drilling rate is generally slow. Drawing inspiration from nature for a lightweight and energy efficient solution, we propose a novel drilling method based on the working mechanism of wood wasp ovipositors. The bio-inspired drill requires no reactive external force by applying two-valve-reciprocating motion. The proposed biomimetic system indicates enhanced utility that is critical for space missions where premium is placed on mass, volume and power. Biological systems are similarly constrained making biomimetic technology uniquely suited and advantageous as a model of miniaturized systems. As a result of the European Space Agency (ESA) project on bionics and space system design [6], this paper presents a conceptual design of the wood wasp drill. Lab-based experiments have shown that the bio-inspired drilling method is feasible and has potential of improving drill efficiency without any additional overhead force or mass. © 2006 IEEE.
KW - Astrobiology
KW - Biomimetic materials
KW - Buckling
KW - Drilling
KW - Gravitation
KW - Mathematical models
KW - Biomimetic systems
KW - Drill efficiency
KW - Drilling method
KW - Space missions
KW - Planetary surface analysis
SN - 1095323X (ISSN); 0780395468 (ISBN); 978-078039546-6 (ISBN)
LA - English
J2 - IEEE Aerosp. Conf. Proc.
M3 - Conference paper
DB - Scopus
N1 - Export Date: 23 February 2023; Cited By: 18; Correspondence Address: Y. Gao; Surrey Space Centre, University of Surrey, Guildford, United Kingdom; email: yang.gao@surrey.ac.uk
ER -

TY - CONF
AU - Tarter, J.C.
TI - The evolution of life in the Universe: Are we alone?
PY - 2006
T2 - Proceedings of the International Astronomical Union
VL - 2
IS - 14
SP - 14
EP - 29
DO - 10.1017/S1743921307009829
UR - https://www.scopus.com/inward/record.uri?eid=2-s2.0-37049027729&doi=10.1017%2fS1743921307009829&partnerID=40&md5=a04322e53062ed929eaa3c326b7e756f
AD - SETI Institute, Mountain View, CA 94043, 515 N Whisman Road,

United States
AB - In his book Plurality of Worlds, Steven J. Dick (1984) has chronicled the millennia of discourse about other inhabited worlds, based upon deeply held religious or philosophical belief systems. The popularity of the idea of extraterrestrial life has waxed and waned and, at its nadir, put proponents at mortal risk. The several generations of scientists now attending this General Assembly of the International Astronomical Union at the beginning of the 21 st century have a marvelous opportunity to shed light on this old question of habitable worlds through observation, experimentation, and interpretation, without recourse to belief systems and without risking their lives (though some may experience rather bumpy career paths). The newly-named and funded, multi-disciplinary field of astrobiology is extremely broad in its scope and is encouraging IAU members to learn and speak the languages of previously disparate disciplines in an attempt to answer the big picture questions: Where did we come from?, Where are we going?, and Are we alone? These are questions that the general public understand and support, and these are questions that are attracting students of all ages to science and engineering programs. These questions also push the limits of modern instrumentation to explore the cosmos remotely across space and time, as well as to examine samples of interplanetary space returned to the laboratory and samples of distant time teased out of our own Earth. Within my personal event horizon, the other planetary systems long-predicted by theorists have been uncovered, along with many whose structures were not predicted. The just-so conditions requisite for the comfort of astronomers have been understood to be only a very narrow subset of the conditions that nurture extremophilic, microbial life. Thus the potentially habitable real estate beyond Earth has been greatly expanded and within the next few decades it may be possible to detect the biosignatures or technosignatures of inhabitants on distant worlds, should there be any. © 2007 International Astronomical Union.
SN - 17439221 (ISSN)
LA - English
J2 - Proc. Int. Astron. Union
M3 - Conference paper
DB - Scopus
N1 - Export Date: 23 February 2023; Cited By: 12; Correspondence Address: J.C. Tarter; SETI Institute, Mountain View, CA 94043, 515 N Whisman Road, United States; email: tarter@seti.org
ER -

TY - CONF
AU - Sterns, P.M.
AU - Tennen, L.I.
TI - The future of planetary protection: Is there reason for optimism?
PY - 2006
T2 - AIAA 57th International Astronautical Congress, IAC 2006
VL - 16
SP - 10991
EP - 11000
DO - 10.2514/6.iac-06-e6.5.04

UR - https://www.scopus.com/inward/record.uri?eid=2-s2.0-40749083621&doi=10.2514%2f6.iac-06-e6.5.04&partnerID=40&md5=a63a5695c62a2c631b1c5940c5344e6f
AD - Sterns and Tennen, Phoenix, AZ, United States
AD - IISL
AD - IAA
AD - ASIL
AD - ABA
AD - IBA
AD - ILA
AD - AIAA
AD - Arizona Space Commission
AD - IEAS
AD - IAF
AB - The search for extraterrestrial life has been a primary focus for interplanetary spacecraft. The integrity of scientific investigations for evidence of indigenous life is dependent upon the presence of a pristine extraterrestrial environment, free from Earth-based organic contaminants carried by the very spacecraft sent to conduct exploratory missions. Policies to protect planetary environments have been adopted by the international scientific and legal communities, however, the policies are not static, and can never be assumed to be adequate to the task. Recent discoveries that water may be more plentiful in the solar system than previously envisioned underscore the need for regular review and re-evaluation of the effectiveness of planetary protection policies. This paper examines recent developments in the evolution of planetary protection, and questions whether there is reason for optimism. Copyright © 2006 Sterns and Tennen.
KW - International law
KW - Interplanetary flight
KW - Planetary surface analysis
KW - Solar system
KW - Space rendezvous
KW - Exploratory missions
KW - Extraterrestrial environment
KW - Planetary protection
KW - Interplanetary spacecraft
PB - American Institute of Aeronautics and Astronautics Inc.
SN - 978-160560039-0 (ISBN)
LA - English
J2 - AIAA Int. Astronaut. Congr. IAC
M3 - Conference paper
DB - Scopus
N1 - Export Date: 23 February 2023; Cited By: 0
ER -

TY - CONF
AU - Hoeg, T.
AU - Southard, L.
AU - Boxerbaum, A.
AU - Reis, L.
AU - Antol, J.
AU - Heldmann, J.

AU - Quinn, R.
TI - Tumbleweed rover science mission to Dao Vallis
PY - 2006
T2 - Collection of Technical Papers - 44th AIAA Aerospace Sciences Meeting
VL - 2
SP - 900
EP - 910
UR - https://www.scopus.com/inward/record.uri?eid=2-s2.0-34250721088&partnerID=40&md5=daa3064deaee7f9faefc0572980bf25f
AD - Case Western Reserve University, Cleveland, OH 44106, United States
AD - NASA Langley Research Center, Hampton, VA 23681, United States
AD - NASA Ames Research Center, Moffett Field, CA 94035, United States
AD - 10900 Euclid Ave, United States
AD - AIAA, United States
AD - Mechanical Engineering, 10900 Euclid Ave, United States
AD - Architectures, Missions and Science Branch, MS 462
AD - Space Science Division, MS 245-3, United States
AB - Dao Vallis, a 1200 km long valley in the eastern Hellas region of Mars, presents a compelling scientific target in terms of both Martian hydrology and astrobiology. Dao Vallis was originally formed by surface and subsurface water flow events. The walls of the valley also contain gullies which are evidence of more recent liquid water activity likely formed from shallow aquifer outbursts and/or melting ground ice and snow. Dao Vallis is a logical place to search for life because of the evidence for both past and relatively recent water. The rugged terrain makes it difficult for robots to land and move safely. Power for mobility and onboard science experiments will be at a premium. These problems can be overcome with the use of a group of "tumbleweed" rovers. They will land on relatively flat terrain and deploy above the valley and use the in-situ resource of the wind to move into the gullies on the valley slopes and then into the valley below. They will cooperate to explore the gullies and large areas of the valley. Tumbleweeds will deliver smaller, more easily controlled rovers to places of interest to collect and examine samples. This mission was animated using Dao Vallis topographical data and models of tumbleweed rovers. The behaviors of the rovers in the animation are based on dynamic modeling and wind tunnel tests. Wind tunnel experiments were performed to determine the drag coefficients for several tumbleweed rover designs, and a three-dimensional dynamic model was used to quantify tumbleweed obstacle navigation and control mechanisms. Using those data, a stochastic simulation was created that uses a swarming algorithm to allow the rovers to disperse from a common location and systematically explore the surface of Mars. The results from these simulations support the use of tumbleweed rovers for a mission to Dao Vallis.
KW - Aerospace engineering
KW - Aquifers
KW - Astrobiology
KW - Data acquisition
KW - Drag coefficient

KW - Hydrology
KW - Navigation
KW - Robots
KW - Stochastic control systems
KW - Liquid water activity
KW - Logical place
KW - Obstacle navigation
KW - Onboard science experiments
KW - Swarming algorithm
KW - Martian surface analysis
SN - 1563478072 (ISBN); 978-156347807-9 (ISBN)
LA - English
J2 - Collect. Tech. Pap. Aerosp. Sci. Meet.
M3 - Conference paper
DB - Scopus
N1 - Export Date: 23 February 2023; Cited By: 5
ER -

TY - CONF
AU - Denning, K.
TI - Ten thousand revolutions: Conjectures about civilizations
PY - 2006
T2 - AIAA 57th International Astronautical Congress, IAC 2006
VL - 3
SP - 1682
EP - 1692
DO - 10.2514/6.iac-06-a4.2.02
UR - https://www.scopus.com/inward/record.uri?eid=2-s2.0-41149130190&doi=10.2514%2f6.iac-06-a4.2.02&partnerID=40&md5=be1479ac845c6372c01a6bd08c58a154
AD - Department of Anthropology, York University, Toronto, M3J 1P3, Canada
AB - Ten thousand years ago, no-one on Earth was living a 'civilized' life. What has happened since is remarkable and impossible to fully comprehend; yet, everyone has ideas about civilization, and how the world came to be as it is. Such understandings of civilizations on Earth inevitably influence speculation about extraterrestrial civilizations, in two ways. First, sometimes a specific Earth civilization or historical experience is explicitly used as a basis for inferences about extraterrestrial civilizations. Second, more general assumptions about the development and functioning of Earth's societies shape conjectures about alien societies. This paper focuses on the latter, general assumptions, with the aim of considering how we can use multidisciplinary approaches, and our knowledge of Earth's civilizations, to our best advantage in SETI.
KW - Extraterrestrial atmospheres
KW - Interplanetary flight
KW - Space applications
KW - Space rendezvous
KW - Space research
KW - Extraterrestrial civilizations
KW - Multidisciplinary approaches
KW - Astrobiology

PB - American Institute of Aeronautics and Astronautics Inc.
SN - 978-160560039-0 (ISBN)
LA - English
J2 - AIAA Int. Astronaut. Congr. IAC
M3 - Conference paper
DB - Scopus
N1 - Export Date: 23 February 2023; Cited By: 1; Correspondence Address: K. Denning; Department of Anthropology, York University, Toronto, M3J 1P3, Canada; email: kdenning@yorku.ca
ER -

TY - CONF
AU - Cash, W.
AU - Schindhelm, E.
AU - Arenberg, J.
AU - Polidan, R.
AU - Kilston, S.
AU - Noecker, C.
TI - The new worlds observer: Using occulters to directly observe planets
PY - 2006
T2 - Proceedings of SPIE - The International Society for Optical Engineering
VL - 6265 I
C7 - 62651V
DO - 10.1117/12.672208
UR - https://www.scopus.com/inward/record.uri?eid=2-s2.0-33749033910&doi=10.1117%2f12.672208&partnerID=40&md5=40c3ddef6ca17b4ac7dde9e471af0c97
AD - University of Colorado, United States
AD - Northrop-Grumman, United States
AD - Ball Aerospace, United States
AB - We discuss the progress that has been made in the understanding of the use of external occulters to observe exoplanetary systems. We show how a starshade can be designed and built in a practical and affordable manner to fully remove starlight and leave only planet light entering a telescope. When coupled to a powerful observatory like the James Webb Space Telescope, an occulter can extinguish the starlight and reveal basic details of the planetary systems around our closest, neighboring stars.
KW - Astrophysics
KW - Optical engineering
KW - Solar system
KW - Space research
KW - Sun
KW - Astrobiology
KW - Coronography
KW - Exoplanets
KW - Starlight
KW - Space telescopes
KW - Astrobiology
KW - Coronography
KW - Exoplanets
SN - 0277786X (ISSN); 0819463302 (ISBN); 978-081946330-2 (ISBN)

LA - English
J2 - Proc SPIE Int Soc Opt Eng
M3 - Conference paper
DB - Scopus
N1 - Export Date: 23 February 2023; Cited By: 12; CODEN: PSISD
ER -

TY - JOUR
AU - Navarro-González, R.
AU - Navarro, K.F.
AU - De La Rosa, J.
AU - Iñiguez, E.
AU - Molina, P.
AU - Miranda, L.D.
AU - Morales, P.
AU - Cienfuegos, E.
AU - Coll, P.
AU - Raulin, F.
AU - Amils, R.
AU - McKay, C.P.
TI - The limitations on organic detection in Mars-like soils by thermal volatilization-gas chromatography-MS and their implications for the Viking results
PY - 2006
T2 - Proceedings of the National Academy of Sciences of the United States of America
VL - 103
IS - 44
SP - 16089
EP - 16094
DO - 10.1073/pnas.0604210103
UR - https://www.scopus.com/inward/record.uri?eid=2-s2.0-33750825504&doi=10.1073%2fpnas.0604210103&partnerID=40&md5=ac78b379f6678a1896ee732b7168fb35
AD - Laboratorio de Química de Plasmas y Estudios Planetarios, Instituto de Ciencias Nucleares, Universidad Nacional Autónoma de México, 04510 México D.F., P.O. Box 70-543, Mexico
AD - Instituto de Química, Universidad Nacional Autónoma de México, Ciudad Universitaria, 04510 México D.F., P.O. Box 70-543, Mexico
AD - Instituto de Geología, Universidad Nacional Autónoma de México, Ciudad Universitaria, 04510 México D.F., P.O. Box 70-543, Mexico
AD - Laboratoire Interuniversitaire des Systemes Atmospheriques, Centre National de la Recherche Scientifique, Université Paris 12-Val de Marne and Université Paris 7-Denis Diderot, Créteil Cedex, 61 Avenue du General de Gaulle 94010, France
AD - Centro de Astrobiología, Consejo Superior de Investigaciones Científicas, Instituto Nacional de Tecnica Aeroespacial, Torrejon de Ardoz, 28850 Madrid, Spain
AD - Space Science Division, Ames Research Center, National Aeronautics and Space Administration, Moffett Field, CA 94035-1000, United States
AB - The failure of Viking Lander thermal volatilization (TV) (without or with thermal degradation)-gas chromatography (GC)-MS

experiments to detect organics suggests chemical rather than biological interpretations for the reactivity of the martian soil. Here, we report that TV-GC-MS may be blind to low levels of organics on Mars. A comparison between TV-GC-MS and total organics has been conducted for a variety of Mars analog soils. In the Antarctic Dry Valleys and the Atacama and Libyan Deserts we find 10-90 µg of refractory or graphitic carbon per gram of soil, which would have been undetectable by the Viking TV-GC-MS. In iron-containing soils (jarosites from Rio Tinto and Panoche Valley) and the Mars simulant (palogonite), oxidation of the organic material to carbon dioxide (CO_2) by iron oxides and/or their salts drastically attenuates the detection of organics. The release of 50-700 ppm of CO_2 by TV-GC-MS in the Viking analysis may indicate that an oxidation of organic material took place. Therefore, the martian surface could have several orders of magnitude more organics than the stated Viking detection limit. Because of the simplicity of sample handling, TV-GC-MS is still considered the standard method for organic detection on future Mars missions. We suggest that the design of future organic instruments for Mars should include other methods to be able to detect extinct and/or extant life. © 2006 by The National Academy of Sciences of the USA.
KW - Carbon
KW - Carbon Dioxide
KW - Catalysis
KW - Chromatography, Gas
KW - Mars
KW - Mass Spectrometry
KW - Organic Chemicals
KW - Oxidation-Reduction
KW - Soil
KW - Temperature
KW - Thermodynamics
KW - Volatilization
KW - carbon dioxide
KW - iron oxide
KW - iron salt
KW - organic compound
KW - analytic method
KW - article
KW - astronomy
KW - desert
KW - gas chromatography
KW - mass spectrometry
KW - oxidation
KW - priority journal
KW - soil analysis
KW - thermal volatilization
KW - thermodynamics
KW - volatilization
KW - Astrobiology
KW - Deserts
KW - Detection of organics
KW - Extreme environments
KW - Search for martian life

SN - 00278424 (ISSN)
C2 - 17060639
LA - English
J2 - Proc. Natl. Acad. Sci. U. S. A.
M3 - Article
DB - Scopus
N1 - Export Date: 23 February 2023; Cited By: 123; Correspondence Address: R. Navarro-González; Laboratorio de Química de Plasmas y Estudios Planetarios, Instituto de Ciencias Nucleares, Universidad Nacional Autónoma de México, 04510 México D.F., P.O. Box 70-543, Mexico; email: navarro@nucleares.unam.mx; CODEN: PNASA
ER -

TY - JOUR
AU - Zheng, W.
AU - Jewitt, D.
AU - Kaiser, R.I.
TI - Formation of hydrogen, oxygen, and hydrogen peroxide in electron-irradiated crystalline water ice
PY - 2006
T2 - Astrophysical Journal
VL - 639
IS - 1 I
SP - 534
EP - 548
DO - 10.1086/499231
UR - https://www.scopus.com/inward/record.uri?eid=2-s2.0-33644998699&doi=10.1086%2f499231&partnerID=40&md5=245a7eb19189563c764479ab09e2a6c5
AD - Institute for Astronomy, University of Hawaii at Manoa, Honolulu, HI 96822, United States
AD - Department of Chemistry, University of Hawaii at Manoa, Honolulu, HI 96822, United States
AB - Water ice is abundant both astrophysically, for example, in molecular clouds, and in planetary systems. The Kuiper belt objects, many satellites of the outer solar system, the nuclei of comets, and some planetary rings are all known to be water-rich. Processing of water ice by energetic particles and ultraviolet photons plays an important role in astrochemistry. To explore the detailed nature of this processing, we have conducted a systematic laboratory study of the irradiation of crystalline water ice in an ultrahigh vacuum setup by energetic electrons holding a linear energy transfer of 4.3 ± 0.1 keV µm-1. The irradiated samples were monitored during the experiment both on line and in situ via mass spectrometry (gas phase) and Fourier transform infrared spectroscopy (solid state). We observed the production of hydrogen and oxygen, both molecular and atomic, and of hydrogen peroxide. The likely reaction mechanisms responsible for these species are discussed. Additional formation routes were derived from the sublimation profiles of molecular hydrogen (90-140 K), molecular oxygen (147-151 K), and hydrogen peroxide (170 K). We also present evidence on the involvement of hydroxyl radicals and possibly oxygen atoms as building blocks to yield hydrogen peroxide at low temperatures (12 K) and via a diffusion-controlled mechanism in the warming up phase of the

irradiated sample. © 2006. The American Astronomical Society. All rights reserved.
KW - Astrobiology
KW - Astrochemistry
KW - Comets: general
KW - Cosmic rays
KW - Infrared: ISM
KW - ISM: molecules
KW - Methods: laboratory
KW - Molecular processes
PB - Institute of Physics Publishing
SN - 0004637X (ISSN)
LA - English
J2 - Astrophys. J.
M3 - Article
DB - Scopus
N1 - Export Date: 23 February 2023; Cited By: 109; Correspondence Address: R.I. Kaiser; Department of Chemistry, University of Hawaii at Manoa, Honolulu, HI 96822, United States; email: kaiser@gold.chem.hawaii.edu
ER -

TY - JOUR
AU - Glikson, A.
AU - Vickers, J.
TI - The 3.26-3.24 Ga Barberton asteroid impact cluster: Tests of tectonic and magmatic consequences, Pilbara Craton, Western Australia
PY - 2006
T2 - Earth and Planetary Science Letters
VL - 241
IS - 1-2
SP - 11
EP - 20
DO - 10.1016/j.epsl.2005.10.022
UR - https://www.scopus.com/inward/record.uri?eid=2-s2.0-29544451061&doi=10.1016%2fj.epsl.2005.10.022&partnerID=40&md5=af8600e8e5e074b219d90cd72df6147f
AD - Department of Earth and Marine Science, Australian National University, Canberra, ACT 0200, Australia
AB - The location in the Barberton Greenstone Belt (Kaapvaal Craton) of ~3.26-3.24 Ga asteroid impact ejecta units at, and immediately above, a sharp break between a > 12 km-thick mafic-ultramafic volcanic crust (Onverwacht Group ~3.55-3.26 Ga, including the ~3.298 > 3.258 Ga Mendon Formation) and a turbidite-felsic volcanic rift-facies association (Fig Tree Group ~3.258-2.225 Ga), potentially represents the first documented example of cause-effect relations between extraterrestrial bombardment and major tectonic and igneous events [D.R. Lowe, G.R. Byerly, F. Asaro, F.T. Kyte, Geological and geochemical record of 3400 Ma old terrestrial meteorite impacts, Science 245 (1989) 959-962; D.R. Lowe, G.R. Byerly, F.T. Kyte, A. Shukolyukov, F. Asaro, A. Krull, Spherule beds 3.47-3.34 Ga-old in the Barberton greenstone belt, South Africa: a record of large meteorite impacts and their influence on early

crustal and biological evolution, Astrobiology 3 (2003) 7–48; A.Y. Glikson, The astronomical connection of terrestrial evolution: crustal effects of post-3.8 Ga mega-impact clusters and evidence for major 3.2 ± 0.1 Ga bombardment of the Earth–Moon system, J. Geodyn. 32 (2001) 205–29]. Here we correlate this boundary with a contemporaneous break and peak magmatic and faulting events in the Pilbara Craton, represented by the truncation of a 3.255–3.235 Ga-old volcanic sequence (Sulphur Springs Group–SSG) by a turbidite-banded iron formation–felsic volcanic association (Pincunah Hill Formation, basal Gorge Creek Group). These events are accompanied by ~3.252–3.235 Ga granitoids (Cleland plutonic suite). The top of the komatiite–tholeiite–rhyolite sequence of the SSG is associated with a marker chert defined at 3.238 ± 3–03.235 ± 3 Ga, abruptly overlain by an olistostrome consisting of mega-clasts of felsic volcanics, chert and siltstone up to 250 × 150 m-large, intercalated with siliciclastic sedimentary rocks and felsic volcanics (Pincunah Hill Formation–basal Gorge Creek Group–GCG [R. M. Hill, Stratigraphy, structure and alteration of hanging wall sedimentary rocks at the Sulphur Springs volcanogenic massive sulphide (VMS) prospect, east Pilbara Craton, Western Australia. B.Sc Hon. Thesis, University of Western Australia (1997) 67 pp.; M.J. Van Kranendonk, A.H. Hickman, R.H. Smithies, D.R. Nelson, Geology and tectonic evolution of the Archaean north Pilbara terrain, Pilbara Craton, Western Australia, Econ. Geol. 97 (2002) 695–732; M.J. Van Kranendonk, Geology of the North Shaw 1 : 100 000 Sheet. Geological Survey Western Australia 1 : 100 000 Geological Series (2000) 86 pp., R. Buick, C.A.W. Brauhart, P. Morant, J.R. Thornett, J.G. Maniew, J.G. Archibald, M.G. Doepel, I.R. Fletcher, A.L. Pickard, J.B. Smith, M.B. Barley, N.J. McNaughton, D.I. Groves, Geochronology and stratigraphic relations of the Sulphur Springs Group and Strelley Granite: a temporally distinct igneous province in the Archaean Pilbara Craton, Australia, Precambrian Res. 114 (2002) 87–120]). The structure and scale of the olistostrome, not seen elsewhere in the Pilbara Craton, is interpreted in terms of intense faulting and rifting, supported by topographic relief represented by deep incision of overlying arenites (Corboy Formation) into underlying units [M.J. Van Kranendonk, Geology of the North Shaw 1 : 100 000 Sheet. Geological Survey Western Australia 1 : 100 000 Geological Series (2000) 86 pp.]. The age overlaps between (1) 3.255 ± 4–3.235 ± 3 Ga peak igneous activity represented by the SSG and the Cleland plutonic suite (Pilbara Craton) and the 3.258 ± 3 Ga S2 Barberton impact unit, and (2) 3.235 ±3 Ga top SSG break and associated faulting and the 3.243 ± 4 S3–S4 Barberton impact units may not be accidental. Should correlations between the Barberton S2–S4 impact units and magmatic and tectonic events in the Pilbara Craton be confirmed, they would imply impact-triggered reactivation of mantle convection, crustal anatexis, faulting and strong vertical movements in Archaean granite–greenstone terrains associated with large asteroid impacts, culminating in transformation from sima-dominated crust to continental rift environments. © 2005 Elsevier B.V. All rights reserved.
KW - Australasia
KW - Australia
KW - Pilbara Block

KW - Western Australia
KW - Ficus (angiosperm)
KW - Hordeum vulgare subsp. vulgare
KW - Correlation methods
KW - Geochemistry
KW - Igneous rocks
KW - Meteorites
KW - Sedimentary rocks
KW - Stratigraphy
KW - Tectonics
KW - Asteoroid impact
KW - Asteroids
KW - Olistostrome
KW - Archean
KW - impact structure
KW - Meteor impacts
KW - Archaean
KW - Asteroid
KW - Impact
KW - Olistostrome
KW - Pilbara
SN - 0012821X (ISSN)
LA - English
J2 - Earth Plan. Sci. Lett.
M3 - Article
DB - Scopus
N1 - Export Date: 23 February 2023; Cited By: 24; Correspondence Address: A. Glikson; Department of Earth and Marine Science, Australian National University, Canberra, ACT 0200, Australia; email: Andrew.glikson@anu.edu.au
ER -

TY - JOUR
AU - Raymond, S.N.
TI - The search for other earths: Limits on the giant planet orbits that allow habitable terrestrial planets to form
PY - 2006
T2 - Astrophysical Journal
VL - 643
IS - 2 II
SP - L131
EP - L134
DO - 10.1086/505596
UR - https://www.scopus.com/inward/record.uri?eid=2-s2.0-33746921973&doi=10.1086%2f505596&partnerID=40&md5=2b3e102082902f12d5730c4f55ba83a3
AD - Laboratory for Atmospheric and Space Physics, University of Colorado, UCB 392, Boulder, CO 80309-0392, United States
AD - NASA Astrobiology Institute, United States
AB - Gas giant planets are far easier than terrestrial planets to detect around other stars, and they are thought to form much more quickly than terrestrial planets. Thus, in systems with giant planets, the late stages of terrestrial planet formation are strongly affected by the giant planets' dynamical presence.

Observations of giant planet orbits may therefore constrain the systems that can harbor potentially habitable, Earth-like planets. We present results of 460 N-body simulations of terrestrial accretion from a disk of Moon- to Mars-sized planetary embryos. We systematically vary the orbital semimajor axis of a Jupiter-mass giant planet between 1.6 and 6 AU, and eccentricity between 0 and 0.4. We find that for Sun-like stars, giant planets inside roughly 2.5 AU inhibit the growth of 0.3 Earth-mass planets in the habitable zone. If planets accrete water from volatile-rich embryos past 2-2.5 AU, then water-rich habitable planets can only form in systems with giant planets beyond 3.5 AU. Giant planets with significant orbital eccentricities inhibit both accretion and water delivery. The majority of the current sample of extrasolar giant planets appears unlikely to form habitable planets. © 2006. The American Astronomical Society. All rights reserved.
KW - Astrobiology
KW - Methods: n-body simulations
KW - Planetary systems: formation
PB - Institute of Physics Publishing
SN - 0004637X (ISSN)
LA - English
J2 - Astrophys. J.
M3 - Article
DB - Scopus
N1 - Export Date: 23 February 2023; Cited By: 43; Correspondence Address: S.N. Raymond; Laboratory for Atmospheric and Space Physics, University of Colorado, UCB 392, Boulder, CO 80309-0392, United States; email: raymond@lasp.colorado.edu
ER -

TY - JOUR
AU - Coleman, M.
AU - Rodgers, D.
AU - Jones, J.
TI - A buoyant life investigating mobile platform (BLIMP)
PY - 2006
T2 - Advances in Space Research
VL - 38
IS - 6
SP - 1198
EP - 1208
DO - 10.1016/j.asr.2005.05.058
UR - https://www.scopus.com/inward/record.uri?eid=2-s2.0-33750497268&doi=10.1016%2fj.asr.2005.05.058&partnerID=40&md5=5f647b8d22ede08124476541097a70a3
AD - Jet Propulsion Laboratory, Pasadena, CA 91109, 4800 Oak Grove Drive, United States
AD - Jet Propulsion Laboratory, Pasadena, CA 91109, 4800 Oak Grove Drive, United States
AD - Jet Propulsion Laboratory, Pasadena, CA 91109, 4800 Oak Grove Drive, United States
AB - The search for life in new environments, e.g., Mars/Titan, will be scientifically challenging and have great engineering difficulties. In this paper the authors discuss an approach to

field-testing methods relevant to three scientific thrusts in the detection of life and pre-biotic organics on other worlds. We describe how this can be accomplished through a series of field trials using a mobile aerial vehicle that is a proxy for the exploration approaches and instrument techniques necessary for the next stage of life detection on other planets. We do this by deploying a mobile organic laboratory on Earth to demonstrate the requisite techniques. We show how terrestrial field trials provide new insights on the colonization by life of fresh volcanic flows, and the competition between biotic and abiotic processes on a newly cooling piece of the Earth's crust. This paper suggests that such work could be very effectively conducted on Hawaii, where the erupted lava is basaltic, an important crustal component for terrestrial planets. The presence of water is generally agreed to be a prerequisite for planetary habitability but the combination of basalt and water is chemically unstable at the temperatures to which basalt cools after eruption. The subsequent chemical reactions occur because the total energy of the products is lower than that of the precursor materials and on Earth biological processes result from organisms harvesting that difference in energy. For life processes to succeed they must out-compete the rate at which abiotic chemistry might accomplish the same tasks. Monitoring the rate at which chemical processes occur is therefore a life-detection approach. Biotic involvement in the rate of weathering of basalts is the test case for this new, generic life detection paradigm. This approach would be applicable to the periglacial zones of Mars, if liquid water were proven to be present there. We show that a 15 m autonomous BLIMP could carry various instrument packages including camera, visible spectrometer, tunable diode laser spectrometer (TDLS) for gas and gas isotope analysis, gas chromatograph/mass spectrometer (GCMS). These could be calibrated followed by ground-truthing using field experiments in the interior of Meteor Crater in Arizona. This well understood system could then study the extreme environment of the still active volcanic caldera of Kilauea and the adjacent older lava flows. For Mars the BLIMP is a proxy for a lighter balloon or even a Martian Rover, which could carry a similar suite of instruments and take a similar set of measurements. For Titan, with its dense and high-molecular weight atmosphere calm winds and low gravity, a BLIMP will be the vehicle of choice. The experiments would be directly relevant. We discuss how a Titan BLIMP could search for organic compounds in the post-Cassini exploration of Titan. © 2005 COSPAR.
KW - Airships
KW - Chemical reactions
KW - Earth (planet)
KW - Gas chromatography
KW - Mass spectrometers
KW - Semiconductor lasers
KW - Astrobiology
KW - Instrumentation
KW - Life detection
KW - Mars
KW - Planetary habitability
KW - Titan

KW - Volcanic caldera
KW - Living systems studies
KW - Airships
KW - Astrobiology
KW - Instrumentation
KW - Life detection
KW - Mars
KW - Titan
KW - Volcanic caldera
PB - Elsevier Ltd
SN - 02731177 (ISSN)
LA - English
J2 - Adv. Space Res.
M3 - Article
DB - Scopus
N1 - Export Date: 23 February 2023; Cited By: 3; Correspondence Address: D. Rodgers; Jet Propulsion Laboratory, Pasadena, CA 91109, 4800 Oak Grove Drive, United States; email: David.H.Rodgers@jpl.nasa.gov; CODEN: ASRSD
ER -

TY - CONF
AU - Raulin, F.
AU - Bénilan, Y.
AU - Coll, P.
AU - Coscia, D.
AU - Gazeau, M.-C.
AU - Hébrard, E.
AU - Jolly, A.
AU - Nguyen, M.-J.
AU - Romanzin, C.
AU - Sternberg, R.
TI - The new Titan: An astrobiological perspective
PY - 2006
T2 - Proceedings of SPIE - The International Society for Optical Engineering
VL - 6309
C7 - 63090I
DO - 10.1117/12.675408
UR - https://www.scopus.com/inward/record.uri?eid=2-s2.0-33750710166&doi=10.1117%2f12.675408&partnerID=40&md5=cf493bdf065ee4f7d64659781fa6c5d4
AD - LISA, CNRS, Universités Paris 7 and Paris 12, F-94000 Créteil, 61 Avenue General de Gaulle, France
AB - Since the first Voyager data, Titan, the largest satellite of Saturn and only satellite in the solar system having a dense atmosphere, became one of the key planetary bodies for astrobiological studies, due to: i) its many analogies with planet Earth, in spite of much lower temperatures, ii) the already well observed presence of an active organic chemistry, involving several of the key compounds of prebiotic chemistry, in the gas phase but also assumed to occur in the solid phase through the haze particles. And the potential development of a prebiotic chemistry in liquid water, with a possible water ocean in its internal structure, and

the possible episodic formation of small liquid water bodies for short but not negligible time duration at the surface (from the melting of surface water ice by impact), iii) the resulting possibility that life may have emerged on or in Titan and may have been able to adapt and to persist. These aspects are examined with some of the associated questions on the basis of the already available Cassini-Huygens data.
KW - Astronomy
KW - Ice
KW - Planets
KW - Prebiotic chemistry
KW - Primitive Earth
KW - Tholins
KW - Titan
KW - Space research
KW - Astrobiology
KW - Cassini-Huygens
KW - Prebiotic chemistry
KW - Primitive Earth
KW - Tholins
KW - Titan
SN - 0277786X (ISSN); 0819463884 (ISBN); 978-081946388-3 (ISBN)
LA - English
J2 - Proc SPIE Int Soc Opt Eng
M3 - Conference paper
DB - Scopus
N1 - Export Date: 23 February 2023; Cited By: 2; CODEN: PSISD
ER -

TY - JOUR
AU - Brake, M.
AU - Griffiths, M.
AU - Hook, N.
AU - Harris, S.
TI - Alien worlds: Astrobiology and public outreach
PY - 2006
T2 - International Journal of Astrobiology
VL - 5
IS - 4
SP - 319
EP - 324
DO - 10.1017/S1473550406003041
UR - https://www.scopus.com/inward/record.uri?eid=2-s2.0-33847245354&doi=10.1017%2fS1473550406003041&partnerID=40&md5=5d627ddbaa5c136a16dd96a09c65feb2
AD - Centre for Astronomy and Science Education, University of Glamorgan, Glamorgan, United Kingdom
AB - Over the last three years an outreach programme in astrobiology has been stimulating public interest in South Wales, UK. To date, 550 people have attended an accredited undergraduate course in astrobiology, Alien Worlds. Funded by a European Social Fund grant, this modular course has introduced students to the multidisciplinary nature of astrobiology, coupling academic content to a practical ability to recognise the constellations and objects

of the night sky. This paper outlines the course's background, content, delivery and outcomes as an example of the outreach potential of the science and culture of astrobiology. © 2006 Cambridge University Press.
KW - Eurasia
KW - Europe
KW - South Wales
KW - United Kingdom
KW - Wales
KW - Western Europe
KW - astronomy
KW - biology
KW - curriculum
KW - education
KW - Communication
KW - Culture
KW - Education
SN - 14753006 (ISSN)
LA - English
J2 - Int. J. Astrobiology
M3 - Article
DB - Scopus
N1 - Export Date: 23 February 2023; Cited By: 6; Correspondence Address: M. Brake; Centre for Astronomy and Science Education, University of Glamorgan, Glamorgan, United Kingdom; email: mbrake@glam.ac.uk
ER -

TY - JOUR
AU - Boston, P.J.
AU - Hose, L.D.
AU - Northup, D.E.
AU - Spilde, M.N.
TI - The microbial communities of sulfur caves: A newly appreciated geologically driven system on Earth and potential model for Mars
PY - 2006
T2 - Special Paper of the Geological Society of America
VL - 404
SP - 331
EP - 344
DO - 10.1130/2006.2404(28)
UR - https://www.scopus.com/inward/record.uri?eid=2-s2.0-73949100786&doi=10.1130%2f2006.2404%2828%29&partnerID=40&md5=32b56e577efa765069b3a62a0b0e05f7
AD - Department of Earth and Environmental Science, New Mexico Institute of Mining and Technology, Socorro, NM 87801, United States
AD - National Cave and Karst Research Institute, Carlsbad, NM 88220, United States
AD - Department of Biology, University of New Mexico, Albuquerque, NM 87131, United States
AD - Institute of Meteoritics, University of New Mexico, Albuquerque, NM 87131, United States
AB - A handful of investigative teams in several parts of the world are studying abundant biological communities in caves formed by

sulfuric-acid speleogenesis. These caves are atypical in terms of origin, chemistry, and ecosystem properties. They prominently display sulfur minerals, characteristic cavity topologies, and notable biological diversity and biological productivity resulting directly from the conditions that produce the caves. Even long-inactive systems still harbor some of these indicators. The microbial and macroscopic ecosystems within sulfuric-acid speleogenetic caves are geologically mediated and maintained. This geological mediation is a theme connecting them with other sulfur-driven ecosystems on Earth, including deep-sea hydrothermal vents, sulfurous near-surface hydrothermal systems, and solfataras. Evidence exists for potentially significant microbial participation in the process of speleogenesis itself. Recent results confirming the high relative abundance of sulfur on Mars, an apparent sedimentary basin with high sulfate concentration, near-surface indicators of ice and water, and trace detection of reduced gases (especially methane) in the Martian atmosphere, possibly deriving from subsurface microbial sources, set the stage for suggesting that sulfuric-acid speleogenetic systems may be useful as astrobiological analogs for hypothetical Mars ecosystems. Unique speleogenetic mechanisms may occur on Mars and could provide subsurface void space suitable for habitationby such hypothetical microbial systems. © 2006 Geological Society of America.
KW - Astrobiology
KW - Caves
KW - Hot springs
KW - Microorganisms
KW - Sulfur
KW - Sulfur compounds
KW - Sulfuric acid
KW - Trace analysis
KW - Biological community
KW - Biological productivity
KW - Deep-sea hydrothermal vents
KW - Hydrothermal system
KW - Mars
KW - Martian atmospheres
KW - Microbial communities
KW - Sulfate concentrations
KW - biological production
KW - extraterrestrial matter
KW - hydrothermal system
KW - hydrothermal vent
KW - Mars
KW - microbial community
KW - planetary landform
KW - species diversity
KW - speleology
KW - sulfur
KW - sulfuric acid
KW - Ecosystems
KW - Acid speleogenesis
KW - Astrobiology
KW - Extraterrestrial caves

KW - Mars
KW - Microorganisms
KW - Sulfur
PB - Geological Society of America
SN - 00721077 (ISSN)
LA - English
J2 - Spec. Pap. Geol. Soc. Am.
M3 - Article
DB - Scopus
N1 - Export Date: 23 February 2023; Cited By: 26
ER -

TY - JOUR
AU - Morring Jr., F.
TI - Being there
PY - 2006
T2 - Aviation Week and Space Technology (New York)
VL - 164
IS - 15
SP - 48
EP - 49
UR - https://www.scopus.com/inward/record.uri?eid=2-s2.0-33646002053&partnerID=40&md5=13094cacbe3bdd927326853ab633b1f7
AB - The US astrobiologists displeased by reduction in NASA's science budget expect to find common cause with well-funded human space explorers in their search for signs of extraterrestrial life. US astronauts have joined their academic counterparts in exploring extreme environments on Earth for evidence of life's adaptability to guide exploration on Mars and elsewhere. The search for life, its fossil remnants and clues to its formation and evaluation will be a big driver for NASA's human and robotic exploration efforts. While NASA is focusing its exploration funds on getting humans back to the Moon, the two liquid-water environments beyond Mars are getting almost as much astrobiology attention as Mars itself.
KW - Budget control
KW - Earth (planet)
KW - Martian surface analysis
KW - Robotics
KW - Space flight
KW - Astrobiology
KW - Robotic exploration
KW - Science budget
KW - Space explorers
KW - Space research
SN - 00052175 (ISSN)
LA - English
J2 - Aviat Week Space Technol (New York)
M3 - Review
DB - Scopus
N1 - Export Date: 23 February 2023; Cited By: 0; CODEN: AWSTA
ER -

TY - CONF
AU - Adami, G.

TI - National workshop on astrobiology: The life science involvement of AAS-I Laben
PY - 2006
T2 - Origins of Life and Evolution of the Biosphere
VL - 36
IS - 5-6
SP - 567
EP - 576
DO - 10.1007/s11084-006-9029-2
UR - https://www.scopus.com/inward/record.uri?eid=2-s2.0-33846454385&doi=10.1007%2fs11084-006-9029-2&partnerID=40&md5=5a18c7a12059ba5d217c575deda79068
AD - Alcatel Alenia Space Italia-LABEN, 20090 Vimodrone, Milan, Strada Padana Superiore 290, Italy
AB - The search for traces of past and present life is a complex and multidisciplinary research activity involving several scientific heritages and a specific industrial ability for planetary exploration. Laben was established in 1958 to design and manufacture electronic instruments for research in nuclear physics. In the mid 2004 the company was merged with Alenia Spazio. It is now part of Alcatel Alenia Space, a French Italian joint venture. Alcatel Alenia Space Italia SpA is a Finmeccanica Company. Currently the plant of Vimodrone provides a wide heritage in life science oriented to space application. The experience in Space Life Science is consolidated in the following research areas: (1) Physiology: • Mouse models related to studies on human physiology • Human neuroscience research and dosimetry (2) Animal Adaptation and Behaviour: mice behaviour related to stabling stress (3) Developmental Biology: aquatic microorganisms cultivation (4) Cell culture & Biotechnology: • Protein crystal growth • General purpose Multiwell Next Biotechnology studies and development: - Bio reactor, mainly oriented to tissue engineering - Microsensor for tissue control (organ replacement) - Multiwell for adherent cell culture or for automated biosensor based on cell culture - Experiment Container for organic systems - Experiment Container for small animals - Instrumentation based on fluorescent Biosensors - Sensors for Life science experiments for Biopan capsule and Space Vehicle - Ray Shielding Materials - Random Positioning Machine specialisation (Support ground equipment) The biological features of this heritage is at disposal for the exobiology multi science. The involvement of industries, from the beginning of the exobiology projects, allows a cost effective technologies closed loop development between Research Centres, Principal Investigators and industry. © 2006 Springer Science+Business Media, B.V.
KW - Animals
KW - Biological Sciences
KW - Biomedical Research
KW - Biotechnology
KW - Exobiology
KW - France
KW - Humans
KW - International Cooperation
KW - Italy
KW - Mice

KW - Models, Animal
KW - Organizational Objectives
KW - Animalia
KW - Mus
KW - astronomy
KW - exobiology
KW - exploration
KW - origin of life
KW - space technology
KW - animal
KW - animal model
KW - biology
KW - biomedicine
KW - biotechnology
KW - conference paper
KW - France
KW - human
KW - international cooperation
KW - Italy
KW - medical research
KW - mouse
KW - organization
KW - organization and management
KW - Bio reactor
KW - Biosensor
KW - Countermeasures
KW - Micro-organisms cultivation
KW - Mouse model
KW - Multiwell
KW - Neuroscience research
KW - Protein crystal growth
KW - Ray shielding materials
SN - 01696149 (ISSN)
C2 - 17171428
LA - English
J2 - Orig. Life Evol. Biosph.
M3 - Conference paper
DB - Scopus
N1 - Export Date: 23 February 2023; Cited By: 0; Correspondence Address: G. Adami; Alcatel Alenia Space Italia-LABEN, 20090 Vimodrone, Milan, Strada Padana Superiore 290, Italy; email: giorgio.adami@aleniaspazio.it; CODEN: OLEBE
ER -

TY - JOUR
AU - Rummel, J.D.
AU - Race, M.S.
TI - Got life? Hours of boredom followed by moments of sheer terror (and that's just with the press)
PY - 2006
T2 - Acta Astronautica
VL - 59
IS - 12
SP - 1160

EP - 1162
DO - 10.1016/j.actaastro.2006.04.017
UR - https://www.scopus.com/inward/record.uri?eid=2-s2.0-33748776555&doi=10.1016%2fj.actaastro.2006.04.017&partnerID=40&md5=9647dcc54ed186794690c62561f37651
AD - Science Mission Directorate, NASA Headquarters, 20546 Washington, DC 20546, 300 E. Street, SW, United States
AD - SETI Institute, Mountain View, CA 94303, United States
AB - It may be hoped that an initial discovery of extraterrestrial life and its disclosure will be done by accident. An event of that kind would have its own dynamic, and while communications about the discovery might be strained at times, there would be less likelihood that lines of inquiry and discourse would have already been taken by the participants and the press. In an ideal world (or worlds), the discovery would come ready-made with a picture or pictures that would be useful as an immediate verification of its reality. But such is not the way of the real world (or worlds, apparently). Lessons learned from the publication of the ALH84001 results in Science magazine are indicative of what may be a more likely scenario. Nonetheless, even that publication was held in confidence for much of the time leading up to NASA's press conference, and the science team doing the work was accordingly insulated from press inquiry while the work was underway. Envisioning a Mars sample return mission, or other, similar sort of endeavor that may involve a dedicated team of scientists-working under continual public scrutiny-it is clear that the circumstances that surround any fundamental discovery about life in the sample would be quite different. Planning for a communications strategy to support the operations of a Mars sample receiving facility (or facilities) must take those circumstances into account. An optimization of the time spent communicating the results of the facility's work should acknowledge the time and effort required, and make provisions for the work to proceed without extensive interruptions-and without being influenced by the expectations of the press or the public. This paper will discuss some of the initial planning associated with the communications strategy surrounding such a facility.
KW - Communication systems
KW - Optimization
KW - Orbits
KW - Space flight
KW - Space research
KW - Communications strategy
KW - Extraterrestrial life
KW - Astronomy
SN - 00945765 (ISSN)
LA - English
J2 - Acta Astronaut
M3 - Article
DB - Scopus
N1 - Export Date: 23 February 2023; Cited By: 1; Correspondence Address: J.D. Rummel; Science Mission Directorate, NASA Headquarters, 20546 Washington, DC 20546, 300 E. Street, SW, United States; email: jrummel@hq.nasa.gov; CODEN: AASTC
ER -

TY - CONF
AU - Lawson, P.R.
TI - Navigator program: Exploring new worlds
PY - 2006
T2 - IEEE Aerospace Conference Proceedings
VL - 2006
C7 - 1655731
UR - https://www.scopus.com/inward/record.uri?eid=2-s2.0-34047166000&partnerID=40&md5=cabc95634c8b4d03a249776d25f5f8bf
AD - Jet Propulsion Laboratory, Pasadena, CA 91109-8099, 04800 Oak Grove Drive, United States
AB - NASA's Navigator Program1,2 comprises a suite of interrelated missions that will discover and characterize planetary systems and Earth-like planets around nearby stars. The missions are designed to build on each other's success, each providing an essential step forward toward the goal of discovering habitable planets and life. The missions in the Navigator Program and their relation to each other are described in this paper. Moreover, the Program is described in the wider context of international efforts in the broadening field of extrasolar planet research. Science priorities and research opportunities related to the Navigator Program are also described. © 2006 IEEE.
KW - Astrobiology
KW - Astrophysics
KW - Earth (planet)
KW - Planetary surface analysis
KW - Stars
KW - Extrasolar planet research
KW - Planetary systems
KW - NASA
SN - 1095323X (ISSN); 0780395468 (ISBN); 978-078039546-6 (ISBN)
LA - English
J2 - IEEE Aerosp. Conf. Proc.
M3 - Conference paper
DB - Scopus
N1 - Export Date: 23 February 2023; Cited By: 0; Correspondence Address: P.R. Lawson; Jet Propulsion Laboratory, Pasadena, CA 91109-8099, 04800 Oak Grove Drive, United States; email: Peter.R.Lawson@jpl.nasa.gov
ER -

TY - CONF
AU - Maise, G.
AU - Powell, J.
AU - Paniagua, J.
AU - Powell, J.
AU - Ludewig, H.
TI - MICE: A system of compact mobile nuclear probes to explore the deep interior of Mars' North Polar Cap
PY - 2006
T2 - AIAA 57th International Astronautical Congress, IAC 2006
VL - 3
SP - 1541

EP - 1549
UR - https://www.scopus.com/inward/record.uri?eid=2-s2.0-41149098518&partnerID=40&md5=ae3d6f07a7afdacd419406606c25abef
AD - Plus Ultra Technologies, Inc., Stony Brook, NY 11790, 180 Harbor Road, United States
AD - Scripps Institution of Oceanography, San Diego, CA 92037, United States
AD - Brookhaven National Laboratory, Upton, NY 11974, United States
AB - The multi-kilometer thick Polar Caps on Mars contain unique and important data about the multi-million year history of its climate, geology, meteorology, volcanology, cosmic ray and solar activity, and meteor impacts. They also may hold evidence of past life on Mars, including microbes, microfossils and biological chemicals. The MICE (Mars Ice Cap Explorer) system ' would explore the Polar Cap interiors using mobile probes powered by compact, lightweight nuclear reactors. The probes would travel 100's of meters per day along melt channels in the ice sheets created by hot water jets from the 500 kW(th) nuclear reactors, ascending and descending, either vertically or at an angle to the vertical, reaching bedrock at kilometers beneath the surface. The powerful reactor will be necessary to provide sufficient hot water at high velocity to penetrate the extensive horizontal dust/sand layers that separate layers of ice in the Mars Ice Caps. MICE reactors can operate at 500 kW(th) for more than 4 years, and much longer in practice, since power level will be much lower when the probes are investigating locations in detail at low or zero speed. Multiple probes, e.g. six, would be deployed in an interactive network, continuously communicating by RF and acoustic signals with each other and with the surface lander spacecraft. In turn, the lander would continuously communicate in real time, subject to speed of light delays, with scientists on Earth to transmit data and receive instructions for the MICE probes. Samples collected by the probes could be brought to the lander, for return to the Earth at the end of the mission.
KW - Astrobiology
KW - Cosmic rays
KW - Jets
KW - Martian surface analysis
KW - Nuclear reactors
KW - Biological chemicals
KW - Lightweight nuclear reactors
KW - Water jets
KW - Space probes
SN - 978-160560039-0 (ISBN)
LA - English
J2 - AIAA Int. Astronaut. Congr. IAC
M3 - Conference paper
DB - Scopus
N1 - Export Date: 23 February 2023; Cited By: 0; Correspondence Address: G. Maise; Plus Ultra Technologies, Inc., Stony Brook, NY 11790, 180 Harbor Road, United States; email: maise@optonline.net
ER -

TY - CONF

AU - Debus, A.
TI - The European standard on planetary protection requirements
PY - 2006
T2 - Research in Microbiology
VL - 157
IS - 1
SP - 13
EP - 18
DO - 10.1016/j.resmic.2005.06.013
UR - https://www.scopus.com/inward/record.uri?eid=2-s2.0-32044472509&doi=10.1016%2fj.resmic.2005.06.013&partnerID=40&md5=32498fc85c749d079a1332d2cc0e85e8
AD - CNES (Centre National d'Etudes Spatiales), BPI 2911, 31401 Toulouse Cedex 9, 18, av. Edouard Belin, France
AB - Since the beginning of solar system exploration, numerous spacecrafts have been sent towards others worlds, and one of the main goals of such missions is the search for extraterrestrial forms of life. It is known that, under certain conditions, some terrestrial entities are able to survive during cruises in space and that they may contaminate other planets (forward contamination). At another level, possible extraterrestrial life forms are unknown and their ability to contaminate the Earth's biosphere (back contamination) in the frame of sample return missions cannot be excluded. Article IX of the Outer Space Treaty (London/Washington, January 27, 1967) requires the preservation of planets and the Earth from contamination. All nations taking part in this Treaty must prevent forward and back contamination during missions exploring our solar system. Consequently, the United Nations (UN-COPUOS) has delegated COSPAR (Committee of Space Research) to take charge of planetary protection and, at present, all space-faring nations must comply with COSPAR policy and consequently with COSPAR planetary protection recommendations. Starting from these recommendations and the "CNES Planetary Protection Standard" document, a working group has been set up in the framework of the "European Cooperation for Space Standardization" (ECSS) to establish the main specifications for preventing cross-contamination between target bodies within the solar system and the Earth-moon system. © 2005 Elsevier SAS. All rights reserved.
KW - Earth (Planet)
KW - Environmental Microbiology
KW - Environmental Pollution
KW - Europe
KW - Exobiology
KW - Extraterrestrial Environment
KW - Space Flight
KW - 2 propanol
KW - hydrogen peroxide
KW - astronomy
KW - conference paper
KW - environmental protection
KW - Europe
KW - gamma radiation
KW - instrument sterilization
KW - microbial contamination

KW - microbiology
KW - microclimate
KW - moon
KW - nonhuman
KW - priority journal
KW - space
KW - space flight
KW - spectrometry
KW - spectroscopy
KW - standard
KW - standardization
KW - United Nations
KW - Cross-contamination
KW - Outer Space Treaty
KW - Planetary protection
KW - Solar system
SN - 17697123 (ISSN)
C2 - 16431088
LA - English
J2 - Res. Microbiol.
M3 - Conference paper
DB - Scopus
N1 - Export Date: 23 February 2023; Cited By: 21; Correspondence Address: A. Debus; CNES (Centre National d'Etudes Spatiales), BPI 2911, 31401 Toulouse Cedex 9, 18, av. Edouard Belin, France; email: andre.debus@cnes.fr; CODEN: RMCRE
ER -

TY - CONF
AU - Gupta, N.
AU - Agogino, A.
AU - Tumer, K.
TI - Efficient agent-based models for non-genomic evolution
PY - 2006
T2 - Proceedings of the International Conference on Autonomous Agents
VL - 2006
SP - 58
EP - 64
DO - 10.1145/1160633.1160640
UR - https://www.scopus.com/inward/record.uri?eid=2-s2.0-34247275885&doi=10.1145%2f1160633.1160640&partnerID=40&md5=ed16fbc32409de1b015748b54f5fa596
AD - Computing Lab., Numerical Analysis Group, Oxford Univ., Oxford, OX1 3QD, Parks Road, United Kingdom
AD - UC Santa Cruz, NASA Ames Research Center, Mailstop 269-3, Moffett Field, CA 94035, United States
AD - NASA Ames Research Center, Mailstop 269-4, Moffett Field, CA 94035, United States
AB - Modeling dynamical systems composed of aggregations of primitive proteins is critical to the field of astrobiological science, which studies early evolutionary structures dealing with the origins of life. Current theories on the emergence of early life focus either on RNA-world models [2, 3] or protein world models [4,

5]. Traditional modeling based on either model are generally either too slow to converge or too simplified to provide good tools for exploring the trade-offs in the early stages of the emergence of life. This paper focuses on protein-world models and discusses how to model protein aggregations through a utility based multi-agent system. We define agents to control specific properties of a given set of proteins. These properties determine the dynamics of the system, such as the ability for proteins to join or split apart, while additional properties determine the aggregation's fitness as a viable primitive cell. We show that over a wide range of starting conditions, there are mechanisms that allow protein aggregations to achieve high values of overall fitness. In addition through the use of agent-specific utilities that remain aligned with the overall global utility, we are able to reach these conclusions with 50 times fewer learning steps. Copyright 2006 ACM.
KW - Computer simulation
KW - Genetic engineering
KW - Learning systems
KW - Proteins
KW - RNA
KW - Non-genomic evolution
KW - Primitive cell
KW - Protein aggregations
KW - Multi agent systems
KW - Multiagent systems
KW - Non-genomic evolution
KW - Reinforcement learning
SN - 1595933034 (ISBN); 978-159593303-4 (ISBN)
LA - English
J2 - Proc Int Conf Autonom Agents
M3 - Conference paper
DB - Scopus
N1 - Export Date: 23 February 2023; Cited By: 2; Correspondence Address: N. Gupta; Computing Lab., Numerical Analysis Group, Oxford Univ., Oxford, OX1 3QD, Parks Road, United Kingdom; email: nachi@comlab.ox.ac.uk
ER -

TY - JOUR
AU - Phillips, S.J.M.
AU - Parnell, J.
TI - The detection of organic matter in terrestrial snow and ice: Implications for astrobiology
PY - 2006
T2 - International Journal of Astrobiology
VL - 5
IS - 4
SP - 353
EP - 359
DO - 10.1017/S1473550406003430
UR - https://www.scopus.com/inward/record.uri?eid=2-s2.0-33847279340&doi=10.1017%2fS1473550406003430&partnerID=40&md5=3feab80e67a2021d380d877ac4f69361
AD - Department of Geology and Petroleum Geology, University of

Aberdeen, Aberdeen AB24 3UE, United Kingdom
AB - The discovery of icy bodies in our Solar System has opened up the possibility that life may exist on and below their surfaces. Snow and ice are good sites for the preservation of biomarkers as they trap and preserve organic matter that is deposited onto their surfaces. Terrestrial samples of snow and ice collected from Ben Macdui in north east Scotland have been analysed for organic compounds contained within them. A range of n-alkanes and n-alkanoic acids were found in both samples. The particulate matter contained proportionally more higher weight n-alkanols than the melted water. This is because higher molecular weight molecules are less soluble in water. Therefore, the volume of snow and ice to be sampled on other icy bodies is an important factor, as many important biomarkers have high molecular weights and may not be detected in small quantities of melted water. © 2006 Cambridge University Press.
KW - astronomy
KW - biology
KW - ice
KW - molecular analysis
KW - organic matter
KW - snow
KW - solar system
KW - solubility
KW - Astrobiology
KW - Detection
KW - Ice
KW - Organic
KW - Snow
KW - Solubility
SN - 14753006 (ISSN)
LA - English
J2 - Int. J. Astrobiology
M3 - Article
DB - Scopus
N1 - Export Date: 23 February 2023; Cited By: 5; Correspondence Address: S.J.M. Phillips; Department of Geology and Petroleum Geology, University of Aberdeen, Aberdeen AB24 3UE, United Kingdom; email: s.j.phillips@abdn.ac.uk
ER -

TY - CONF
AU - Gormly, S.
AU - Adams, V.D.
AU - Marchand, E.
AU - Cannon, B.
AU - Flynn, M.
TI - Proof of concept testing of low temperature brine microbial habitats for subsurface mars and europa habitat viability scenario testing and astrobiology biosignature instrument development
PY - 2006
T2 - SAE Technical Papers
DO - 10.4271/2006-01-2008
UR - https://www.scopus.com/inward/record.uri?eid=2-s2.0-85072433380&doi=10.4271%2f2006-01-2008&partnerID=40&md5=f930da8

623f883163f52e86148fe4d3c
AD - University of Nevada, Reno and National Space Grant Foundation, United States
AD - University of Nevada, Reno, United States
AD - NASA Ames Research Center, United States
AB - Presented is a synopsis of ongoing research into the development of techniques and hardware required to produce useable quantities of astrobiology relevant biomass under controlled and repeatable laboratory conditions. This study has developed microbial habitats (referred to as digesters, due to their biomass production function) capable of sustaining microbial communities under low temperature, high salt, high sulfate, anaerobic conditions. This set of basic conditions is necessary to develop biomass material that is analog to the biomass that would be present in subsurface brine habitats on Mars or Europa, from the perspective of several critical biochemical properties. Stable microbial biomass production and substrate utilization of acetate and sulfate was achieved at temperatures from 7°C to -1°C (psychrophilic), ionic strength and composition of Earth normal seawater or approximately 3.5 % as NaCl (mildly halophilic), high sulfate concentrations of up to 2,000 mg/L SO 42-, and strictly anaerobic conditions. Under these conditions, substantial substrate utilization was monitored, to include robust metabolic activity at -1°C. Copyright © 2006 SAE International.
KW - Biomass
KW - Ecosystems
KW - Instrument testing
KW - Ionic strength
KW - Low temperature production
KW - Sodium chloride
KW - Substrates
KW - Sulfur compounds
KW - Temperature
KW - Anaerobic conditions
KW - Biochemical properties
KW - Biomass productions
KW - Instrument development
KW - Laboratory conditions
KW - Microbial communities
KW - Substrate utilization
KW - Sulfate concentrations
KW - Low temperature testing
PB - SAE International
SN - 01487191 (ISSN)
LA - English
J2 - SAE Techni. Paper.
M3 - Conference paper
DB - Scopus
N1 - Export Date: 23 February 2023; Cited By: 0
ER -

TY - JOUR
AU - Porto de Mello, G.
AU - del Peloso, E.F.
AU - Ghezzi, L.

TI - Astrobiologically interesting stars within 10 parsecs of the sun
PY - 2006
T2 - Astrobiology
VL - 6
IS - 2
SP - 308
EP - 331
DO - 10.1089/ast.2006.6.308
UR - https://www.scopus.com/inward/record.uri?eid=2-s2.0-33744817331&doi=10.1089%2fast.2006.6.308&partnerID=40&md5=7972b1008b5dad6b8b0b7ce523ac5140
AD - Observatório do Valongo, Universidade Federal do Rio de Janeiro, Rio de Janeiro 20080-090, Ladeira Pedro Antônio, 43, Brazil
AD - Observatório Nacional, Rio de Janeiro, Brazil
AB - The existence of life based on carbon chemistry and water oceans relies upon planetary properties, chiefly climate stability, and stellar properties, such as mass, age, metallicity, and galactic orbits. The latter can be well constrained with present knowledge. We present a detailed, up-to-date compilation of the atmospheric parameters, chemical composition, multiplicity, and degree of chromospheric activity for the astrobiologically interesting solar-type stars within 10 parsecs of the Sun. We determined their state of evolution, masses, ages, and space velocities, and produced an optimized list of candidates that merit serious scientific consideration by the future space-based interferometry probes aimed at directly detecting Earth-sized extrasolar planets and seeking spectroscopic infrared biomarkers as evidence of photosynthetic life. The initially selected stars number 33 solar-type within the total population (excluding some incompleteness for late M-dwarfs) of 182 stars closer than 10 parsecs. A comprehensive and detailed data compilation for these objects is still lacking; a considerable amount of recent data has so far gone unexplored in this context. We present 13 objects as the nearest "biostars," after eliminating multiple stars, young, chromospherically active, hard x-ray-emitting stars, and low metallicity objects. Three of these "biostars" - Zeta Tucanae, Beta Canum Venaticorum, and 61 Virginis - closely reproduce most of the solar properties and are considered as premier targets. We show that approximately 7% of the nearby stars are optimally interesting targets for exobiology. © Mary Ann Liebert, Inc.
KW - Astrobiology
KW - Solar neighborhood
KW - Solar type stars
KW - Stars
PB - Mary Ann Liebert Inc.
SN - 15311074 (ISSN)
C2 - 16689649
LA - English
J2 - Astrobiology
M3 - Article
DB - Scopus
N1 - Export Date: 23 February 2023; Cited By: 16; Correspondence Address: G. Porto de Mello; Observatório do Valongo, Universidade Federal do Rio de Janeiro, Rio de Janeiro 20080-090, Ladeira Pedro

Antônio, 43, Brazil; email: gustavo@ov.ufrj.br
ER -

TY - CONF
AU - Alonso, S.
AU - González, E.
TI - Astroseti.org: Online collaborative translations for science public outreach
PY - 2006
T2 - AIAA 57th International Astronautical Congress, IAC 2006
VL - 13
SP - 9099
EP - 9107
UR - https://www.scopus.com/inward/record.uri?eid=2-s2.0-40749109039&partnerID=40&md5=745c907d7de683199040f56a82c59add
AD - Astroseti.org, Spain
AB - Despite the current advances in every field of science, and particularly in astronomy, astrophysics and astronautics, an important proportion of the world's population does not receive clear information nor news about the most recent discoveries and developments in these research topics. There are several reasons for this situation. Among them we can cite the low (and usually not very accurate) coverage that science receives in the media. Additionally, the unfamiliarity of a vast majority of the population with English, which has become the de facto standard for science communication and exchange, creates a broader barrier that many people have to overcome in order to have access to updated science information and news. Astroseti.org is a non-profit web community comprising more than one hundred persons from around the world, covering a very wide range of ages (16-69), which tries to minimize the language problems in science popularisation for spanish-speaking people. To do so, we carry out several activities, usually making use of Internet technologies: Our main task is the translation from English to Spanish of scientific divulgation articles, with special interest in the fields of astronomy, astronautics, astrophysics and astrobiology. These translations are published in our web site (http://astroseti.org). To date we have translated more than 3000 articles from different sources as Science@NASA, Astrobiology Magazine, NASA Astrobiology Institute, JPL Planet Quest, etc. In some cases the original sources recognize us as their "official translators" and they publish our translations in their sites or create links to our pages. Every day we publish science related news taken from different sources. We offer the possibility of making comments about them to our readers, which usually helps to better understand and enrich the provided information. We also have web forums and a chat-room that are used to clarify, comment or expose any news, questions and problems about many science topics. To carry out these activities we have developed some specific web tools that help with the organization and edition of the translations and the communication among the members of Astroseti.org. Our iniciative has been very successful: since the launch of our web site in 2002, the number of visitors to it has increased exponentially, the number of registered translators has grown to nearly one hundred, and we have been cited in several important mass-media, as El Pais, Diario

Clarín, La2, Tele5, Canal9 and Cuatro. Nowadays in every field of science and technology, and particularly in astronomy, astrophysics and astronautics new great advances are being developed and integrated into our daily lives. However an important proportion of the world's population does not receive clear information nor any news about the most recent discoveries and developments of science and technology. This situation can be explained by several reasons: Low coverage that science receives in the media, large scale science illiteracy and unfamiliarity of a vast majority of the population with English, which has become the de facto standard for science communication and exchange. In this paper we present Astroseti.org, a nonprofit web community which tries to minimize the language problems in science popularization for Spanish-speaking people. To do so, we carry out several activities, usually making use of Internet technologies: Translation from English to Spanish of scientific divulgation articles, publication of science related news and creation and maintenance of forums and chat rooms in which Astroseti members and readers can clarify, comment or expose any news, questions or problems about many science topics.
KW - Astrophysics
KW - Education
KW - Online searching
KW - Space research
KW - Science outreach
KW - Webcommunity
KW - Computer supported cooperative work
KW - Science outreach
KW - Translations
KW - Volunteer
KW - Webcommunity
SN - 978-160560039-0 (ISBN)
LA - English
J2 - AIAA Int. Astronaut. Congr. IAC
M3 - Conference paper
DB - Scopus
N1 - Export Date: 23 February 2023; Cited By: 0; Correspondence Address: S. Alonso; Astroseti.org, Spain; email: salonso@decsai.ugr.es
ER -

TY - CONF
AU - Levin, G.V.
TI - Modern myths of Mars
PY - 2006
T2 - Proceedings of SPIE - The International Society for Optical Engineering
VL - 6309
C7 - 63090C
DO - 10.1117/12.676304
UR - https://www.scopus.com/inward/record.uri?eid=2-s2.0-33750739296&doi=10.1117%2f12.676304&partnerID=40&md5=95783e39575a7c292aaa485d9d46c3a9
AD - BioSpherix Division, Spherix Incorporated, Annapolis, MD 21401, United States

AB - July 30, 2006 was the 30th anniversary of the Viking Mission's first Labeled Release (LR) life detection experiment on Mars. The strong response, together with supporting results from eight additional LR tests of Martian soil, established the presence of an active agent that was inhibited by heating. The data satisfied the pre-mission criteria for the detection of living microorganisms. However, the scientific community reacted cautiously, generally concluding that the activity in the soil was caused by chemistry or physics. Over the last three decades, investigation of Mars has greatly increased. Soil, rock and atmospheric analyses have been made. Multi-spectral observations have been made from Mars and Earth orbits and from Earth-based telescopes. Knowledge of extreme habitats and bizarre life forms that populate them on Earth has increased dramatically. However, this vast amount of new astrobiological information has yet to be integrated into an objective scientific evaluation of the LR results and the possibilities for life on Mars. Indeed, in part upon misinterpretations of the new findings, myths have been embedded into the scientific literature of Mars. Based on these myths as key ingredients, a false "standard model" of Martian life potential has been developed. It has been accepted by much of the astrobiological community, and, through its endorsement, the world at large. This paper attempts to bring the supportable facts together in calling for a revision of the current consensus regarding life on Mars. It recommends actions to facilitate the paradigm change.
KW - Astronomy
KW - Microorganisms
KW - Soils
KW - Earth orbits
KW - Labeled Release (LR)
KW - Living microorganisms
KW - Martian soil
KW - Planets
KW - Astrobiology, extreme habitats
KW - Life on Mars
KW - Martian environment
KW - Viking mission labeled release experiment
KW - Water on Mars
SN - 0277786X (ISSN); 0819463884 (ISBN); 978-081946388-3 (ISBN)
LA - English
J2 - Proc SPIE Int Soc Opt Eng
M3 - Conference paper
DB - Scopus
N1 - Export Date: 23 February 2023; Cited By: 6; Correspondence Address: G.V. Levin; BioSpherix Division, Spherix Incorporated, Annapolis, MD 21401, United States; email: glevin@spherix.com; CODEN: PSISD
ER -

TY - CONF
AU - Cantó, J.
AU - Satorre, M.A.
AU - Domingo, M.
AU - Luna, R.

AU - Millán, C.
AU - Gomis, Ó.
AU - Vilaplana, R.I.
AU - Santonja, C.
TI - Astrolaboratory in alcoi for astrobiology studies
PY - 2006
T2 - AIAA 57th International Astronautical Congress, IAC 2006
VL - 1
SP - 310
EP - 315
DO - 10.2514/6.iac-06-a1.6.05
UR - https://www.scopus.com/inward/record.uri?eid=2-s2.0-41149100509&doi=10.2514%2f6.iac-06-a1.6.05&partnerID=40&md5=9c6f75bf00ee64c23fa735c9cf1f6c58
AD - Department of Applied Physics, Escola Politècnica Superior d'Alcoi, Universitat Politècnica de València, 03801 Alcoi (Alicante), Plaça Ferràndiz i Carbonell, Spain
AB - There are many ways to study material present in space: observation, theory, computational simulation and laboratory works. Since 2001 and astro-laboratory for physical and chemical studies of material in space is building up in Alcoi. Laboratory experiments are crucial in order to simulate targets or situations where it is impossible to carry out direct measurements. But in the Solar System it is possible to do experiments in order to simulate situations as for example seasonal changes in temperature. It is possible also to study chemical changes due to reactions with ions and UV photons irradiations in controlled environments. All of these studies can be analyzed with different techniques and electromagnetic wavelengths. In this contribution we want to offer a bird view of the instrumentation at our laboratory for astrophysical characterization in order to show the possibilities of our laboratory in order to open possible collaborations. Different targets can be emulated in laboratory: gases, liquids or solid material as silicates, carbonaceous material or ices. We understand ices as the solid phase or molecules that are gases or liquids in standard conditions; for example it is possible to produce ices of water, methane, carbon dioxide, nitrogen, etc. With this purpose, a particular sample prepared in the system, to emulate a particular scenario, is called "analog". It is composed by relevant molecules for a particular astronomical scenario as planets, satellites, comets, trans-neptunian objects in our Solar System and in the interstellar medium. Astrochemistry adds another type of solids to our list of analogs: organic molecules. And finally, a new branch has appeared in recent years, astrobiology, based on results that support amino acids could be produced in space. So, the aim of this contribution is to offer a quick look at our capacities so as to show our laboratory possibilities to make possible future collaborations with other groups.
KW - Computer simulation
KW - Photons
KW - Solar system
KW - Space research
KW - Ultraviolet radiation
KW - Astrolaboratory

KW - Astronomical scenario
KW - Astrobiology
PB - American Institute of Aeronautics and Astronautics Inc.
SN - 978-160560039-0 (ISBN)
LA - English
J2 - AIAA Int. Astronaut. Congr. IAC
M3 - Conference paper
DB - Scopus
N1 - Export Date: 23 February 2023; Cited By: 0; Correspondence Address: J. Cantó; Department of Applied Physics, Escola Politècnica Superior d'Alcoi, Universitat Politècnica de València, 03801 Alcoi (Alicante), Plaça Ferràndiz i Carbonell, Spain; email: jocando@fis.upv.es
ER -

TY - CONF
AU - Rummel, J.D.
TI - Preventing the forward contamination of mars: Concerns, questions, and required actions
PY - 2006
T2 - IEEE Aerospace Conference Proceedings
VL - 2006
C7 - 1655744
UR - https://www.scopus.com/inward/record.uri?eid=2-s2.0-34047129274&partnerID=40&md5=905a60bbb42e3b98c67631b293a4cea2
AD - Science Mission Directorate, NASA Headquarters, Washington, DC 20546, United States
AB - New data are now forthcoming about the nature of the martian environment and its potential to harbor Earth organisms introduced by space missions. The Mars Exploration Rovers have determined that standing liquid water existed on Mars for long periods of time in the past, and the Mars Global Surveyor mission and Mars Odyssey continue to uncover evidence about past water, ice, and the dynamic nature of the planet. Recently, the National Research Council (NRC) issued a report making recommendations to NASA concerning measures required to prevent future missions from contaminating Mars and to allow continued astrobiological exploration of the planet. Interim requirements recommended by the NRC include surface-sterilization of all spacecraft landing on Mars until "special" regions on Mars can be objectively differentiated from "non-special" regions, and a continuing evaluation of martian environments, microbial survival, and spacecraft contamination. This paper will discuss the report, its application to future NASA missions and potentially by other space agencies, and the actions required to protect Mars from Earth microbes and to support the continuing exploration of Mars.
KW - Astrobiology
KW - Decontamination
KW - Space research
KW - Spacecraft
KW - Sterilization (cleaning)
KW - Astrobiological exploration
KW - Martian environments
KW - Space missions
KW - Martian surface analysis

SN - 1095323X (ISSN); 0780395468 (ISBN); 978-078039546-6 (ISBN)
LA - English
J2 - IEEE Aerosp. Conf. Proc.
M3 - Conference paper
DB - Scopus
N1 - Export Date: 23 February 2023; Cited By: 1; Correspondence Address: J.D. Rummel; Science Mission Directorate, NASA Headquarters, Washington, DC 20546, United States; email: jrummel@hq.nasa.gov
ER -

TY - JOUR
AU - Montmerle, T.
AU - Claeys, P.
AU - Gargaud, M.
AU - López-García, P.
AU - Martin, H.
AU - Pascal, R.
AU - Reisse, J.
AU - Selsis, F.
TI - Life on earth... And elsewhere?
PY - 2006
T2 - Earth, Moon and Planets
VL - 98
IS - 1-4
SP - 299
EP - 312
DO - 10.1007/s11038-006-9093-7
UR - https://www.scopus.com/inward/record.uri?eid=2-s2.0-33750438423&doi=10.1007%2fs11038-006-9093-7&partnerID=40&md5=44c3d69b3c7c82bede00eca14b61fef2
AD - Laboratoire d'Astrophysique de Grenoble, Université Joseph Fourier, Grenoble, France
AD - DGLG-WE, Vrije Universiteit Brussel, Brussels, Belgium
AD - Observatoire Aquitain des Sciences de l'Univers, Université Bordeaux 1, Bordeaux, France
AD - Unité d'Ecologie, Systématique et Evolution, Université Paris-Sud, Orsay, France
AD - Laboratoire Magmas et Volcans, Université Blaise Pascal, Clermont-Ferrand, France
AD - Départment de Chimie, Université Montpellier 2, Montpellier, France
AD - Faculté des Sciences Appliquées (CP 165/64), Université Libre de Bruxelles, Brussels, Belgium
AD - Centre de Recherche Astronomique de Lyon, Ecole Normale Supérieure de Lyon, Lyon, France
AB - This concluding chapter is divided into two main parts. The first part is a summary of the main facts and events which constitute the present body of knowledge of the chronology of life in the solar system, in the form of "highlights" in astronomy, geology, chemistry and biology. The second part raises the interrogation "Is life universal?", and tries to provide answers based on these facts and events. These answers turn out to differ widely among the various disciplines, depending on how far they feel

able to extrapolate their current knowledge. © Springer Science+Business Media, Inc. 2006.
KW - Eukaryota
KW - early Earth
KW - exobiology
KW - microfossil
KW - planetary evolution
KW - solar system
KW - Astrobiology
KW - Chemical evolution
KW - Early Earth
KW - Early Mars
KW - Early Venus
KW - Eukaryotes
KW - Exoplanets
KW - Habitable planets
KW - Late Heavy Bombardment
KW - Microfossils
KW - Moon
KW - Origin of life
KW - Prebiotic molecules
KW - Solar system
KW - Star planet information
KW - Supernovae
KW - Universality of life
PB - Springer Netherlands
SN - 01679295 (ISSN)
LA - English
J2 - Earth Moon Planets
M3 - Article
DB - Scopus
N1 - Export Date: 23 February 2023; Cited By: 1; Correspondence Address: T. Montmerle; Laboratoire d'Astrophysique de Grenoble, Université Joseph Fourier, Grenoble, France; email: montmerle@obs.ujf-grenoble.fr
ER -

TY - CONF
AU - Tinetti, G.
TI - Characterizing extrasolar terrestrial planets with reflected, emitted and transmitted spectra
PY - 2006
T2 - Origins of Life and Evolution of the Biosphere
VL - 36
IS - 5-6
SP - 541
EP - 547
DO - 10.1007/s11084-006-9039-0
UR - https://www.scopus.com/inward/record.uri?eid=2-s2.0-33846426124&doi=10.1007%2fs11084-006-9039-0&partnerID=40&md5=c23aac695308942e30a2492d1765cf0e
AD - European Space Agency, Institut d'Astrophysique de Paris, IAP -Section Planètes Extra Solaires, 75014 Paris, 98 bis boulevard Arago, France

AB - NASA and ESA are planning missions to directly detect and characterize terrestrial planets outside our solar system (nominally NASA-Terrestrial Planet Finder and ESA-DARWIN missions). These missions will provide our first opportunity to spectroscopically study the global characteristics of those planets, and search for signs of habitability and life. We have used spatially and spectrally-resolved models to explore the observational sensitivity to changes in atmospheric and surface properties, and the detectability of surface biosignatures, in the globally averaged spectra and light-curves of the Earth. Atmospheric signatures of Earth-size exoplanets might be detected, in a near future, by stellar occultation as well. Detectability depends on planet's size, atmospheric composition, cloud cover and stellar type. According to our simulations, Earth's land vegetation signature (red-edge) is potentially visible in the disk-averaged spectra, even with cloud cover, and when the signal is averaged over the daily time scale. Marine vegetation is far more difficult to detect. We explored also the detectability of an exo-vegetation responsible for producing a signature that is red-shifted with respect to the Earth vegetation's one. © 2006 Springer Science+Business Media, B.V.
KW - Astronomy
KW - Exobiology
KW - Extraterrestrial Environment
KW - Planets
KW - Spectrum Analysis
KW - Earth
KW - exobiology
KW - planetary atmosphere
KW - planetary surface
KW - satellite mission
KW - solar system
KW - spectroscopy
KW - astronomy
KW - biology
KW - chemistry
KW - conference paper
KW - methodology
KW - microclimate
KW - spectroscopy
KW - Astrobiology
KW - Atmospheric science
KW - Biosignatures
KW - Extrasolar planets
KW - Remote sensing spectroscopy
SN - 01696149 (ISSN)
C2 - 17120124
LA - English
J2 - Orig. Life Evol. Biosph.
M3 - Conference paper
DB - Scopus
N1 - Export Date: 23 February 2023; Cited By: 9; Correspondence Address: G. Tinetti; European Space Agency, Institut d'Astrophysique de Paris, IAP -Section Planètes Extra Solaires, 75014 Paris, 98 bis boulevard Arago, France; email: tinetti@iap.fr; CODEN: OLEBE

ER -

TY - CONF
AU - Behar, A.
AU - Matthews, J.
AU - Venkateswaran, K.
AU - Bruckner, J.
AU - Jonsson, J.
TI - A deep sea hydrothermal vent bio-sampler for large volume in-situ filtration of hydrothermal vent fluids
PY - 2006
T2 - Cahiers de Biologie Marine
VL - 47
IS - 4
SP - 443
EP - 447
UR - https://www.scopus.com/inward/record.uri?eid=2-s2.0-33846425753&partnerID=40&md5=f6f7dea3bf47f8d0bdc4e75b67c8e999
AD - NASA, Jet Propulsion Laboratory, Pasadena, CA 91109, 4800 Oak Grove Drive, United States
AD - Lulea University of Technology, 971 87 Lulea, Sweden
AB - The Hydrothermal Vent BioSampler (HVB) currently being developed at the Jet Propulsion Laboratory is designed to collect large-volume hydrothermal vent samples, operating with fluid temperatures reaching 400°C and at vent depths of up to 6,500 metres. The primary goal of the project is to collect 'pristine' samples untainted by the surrounding waters. Analysis of the collected samples can reveal the existence of thermophillic organisms within the vent fluid, extending the upper limits of life with respect to thermo-tolerance. Any biology found at such environments can contribute to research in astrobiology, while the technology developed for the system can contribute to bio-containment techniques useful for Arctic and planetary exploration. The HVB performs in-situ filtering of hydrothermal vent fluids to concentrate a large sample volume to a smaller volume more suitable for transport. The HVB system is currently in the development phase. This paper provides a physical description of the current system, as well as a summary of the preliminary tests conducted in 2005 and 2006: pressure chamber tests and a two dive operations at a hydrothermal vent off the northern coast of Iceland.
KW - biology
KW - filtration
KW - high pressure
KW - high temperature
KW - hydrothermal fluid
KW - hydrothermal vent
KW - laboratory method
KW - testing method
KW - High pressure
KW - High temperature
KW - Hydrothermal vents
KW - In situ filtration
KW - Pristine samples
KW - Water sampler

SN - 00079723 (ISSN)
LA - English
J2 - Cah. Biol. Mar.
M3 - Conference paper
DB - Scopus
N1 - Export Date: 23 February 2023; Cited By: 8; Correspondence Address: A. Behar; NASA, Jet Propulsion Laboratory, Pasadena, CA 91109, 4800 Oak Grove Drive, United States; email: Alberto.Behar@jpl.nasa.gov
ER -

TY - JOUR
AU - Kerr, R.A.
TI - Astrobiology science conference 2006. Life slow enough to live on radioactivity.
PY - 2006
T2 - Science
VL - 312
IS - 5771
SP - 179
DO - 10.1126/science.312.5771.179a
UR - https://www.scopus.com/inward/record.uri?eid=2-s2.0-33645974865&doi=10.1126%2fscience.312.5771.179a&partnerID=40&md5=f7fcef624c414fb381ece1b57bd5e400
KW - Bacteria
KW - Elements, Radioactive
KW - Energy Metabolism
KW - Exobiology
KW - Geologic Sediments
KW - Hydrogen
KW - Oxidation-Reduction
KW - Pacific Ocean
KW - Water
KW - Biogenesis
KW - Evolution, Chemical
KW - Hot Springs
KW - Organic Chemicals
KW - Pressure
KW - Solar System
KW - Temperature
KW - hydrogen
KW - radioactive element
KW - water
KW - organic compound
KW - article
KW - bacterium
KW - biology
KW - chemistry
KW - energy metabolism
KW - metabolism
KW - microbiology
KW - oxidation reduction reaction
KW - Pacific Ocean
KW - sediment

KW - astronomy
KW - biogenesis
KW - pressure
KW - temperature
KW - thermal spring
SN - 10959203 (ISSN)
C2 - 16614184
LA - English
J2 - Science
M3 - Article
DB - Scopus
N1 - Export Date: 23 February 2023; Cited By: 2
ER -

TY - CONF
AU - Townsend, L.W.
AU - Moussa, H.M.
AU - Charara, Y.
TI - Characterization of the lunar radiation environment using the CRaTER detector
PY - 2006
T2 - IEEE Aerospace Conference Proceedings
VL - 2006
C7 - 1655759
UR - https://www.scopus.com/inward/record.uri?eid=2-s2.0-34047154635&partnerID=40&md5=6d848ed5c761903ec4fb04bdf45abbcb
AD - Department of Nuclear Engineering, University of Tennessee, Knoxville, TN 37996-2300, United States
AB - In late 2008, the Lunar Reconnaissance Orbiter (LRO) mission will be launched into orbit around Earth's moon.1,2 The LRO mission will conduct exploration-enabling observations. One of the objectives of this mission is to characterize the lunar radiation environment and its biological impacts on humans. For this purpose a collaboration involving research personnel from Boston University, Massachusetts Institute of Technology, The University of Tennessee, The Aerospace Corporation, Air Force Research Laboratory, and the NOAA Space Environment Center successfully proposed to develop a sensor system called the Cosmic Ray Telescope for the Effects of Radiation (CRaTER). CRaTER will directly measure linear energy transfer (LET) spectra, thereby providing a direct link between the ambient environment and its biological impact on future human missions to the Moon. In this presentation an overview of the CRaTER team and project organization will be presented and the current version of the proposed CRaTER instrument will be described. Preliminary computational predictions of some components of the LET spectra will be presented. © 2006 IEEE.
KW - Astrobiology
KW - Cosmic rays
KW - Energy transfer
KW - Lunar missions
KW - Space telescopes
KW - Cosmic Ray Telescope for the Effects of Radiation (CRaTER)
KW - CRaTER detector
KW - Linear energy transfer (LET)

KW - Lunar radiation
KW - Lunar Reconnaissance Orbiter (LRO) mission
KW - Radiation detectors
SN - 1095323X (ISSN); 0780395468 (ISBN); 978-078039546-6 (ISBN)
LA - English
J2 - IEEE Aerosp. Conf. Proc.
M3 - Conference paper
DB - Scopus
N1 - Export Date: 23 February 2023; Cited By: 0; Correspondence Address: L.W. Townsend; Department of Nuclear Engineering, University of Tennessee, Knoxville, TN 37996-2300, United States; email: ltownsen@tennessee.edu
ER -

TY - JOUR
AU - Montanes-Rodriguez, P.
AU - Pallé, E.
AU - Goode, P.R.
AU - Martín-Torres, F.J.
TI - Vegetation signature in the observed globally integrated spectrum of earth considering simultaneous cloud data: Applications for extrasolar planets
PY - 2006
T2 - Astrophysical Journal
VL - 651
IS - 1 I
SP - 544
EP - 552
DO - 10.1086/507694
UR - https://www.scopus.com/inward/record.uri?eid=2-s2.0-33845685985&doi=10.1086%2f507694&partnerID=40&md5=4ac1f9a8536cdf3cb51cfa8e62534435
AD - Big Bear Solar Observatory, New Jersey Institute of Technology, Newark, NJ 07102, United States
AD - Analytical Services and Materials, Inc., Virtual Planetary Laboratory, Hampton, VA 23666, 1 Enterprise Parkway, United States
AB - A series of missions will be launched over the next few decades that will be designed to detect and characterize extrasolar planets around nearby stars. These missions will search for habitable environments and signs of life (biosignatures) in planetary spectra. The vegetation's "red edge," an enhancement in the Earth's spectrum near 700 nm when sunlight is reflected from greenery, is often suggested as a tool in the search for life in terrestrial-like extrasolar planets. Here, through ground-based observations of the Earth's spectrum, satellite observations of clouds, and an advanced atmospheric radiative-transfer code, we determine the temporal evolution of the vegetation signature of Earth. We find a strong correlation between the evolution of the spectral intensity of the red edge and changes in the cloud-free vegetated area over the course of observations. This relative increase for our single day corresponds to an apparent reflectance change of about 0.0050 ± 0.0005 with respect to the mean albedo of 0.25 at 680 nm (2.0% ± 0.2%). The excellent agreement between models and observations motivated us to probe more deeply into the red-edge

detectability using real cloud observations at longer timescales. Overall, we find the evolution of the red-edge signal in the globally averaged spectra to be weak, and only attributable to vegetation changes when the real land and cloud distributions for the day are known. However, it becomes prominent under certain Sun-Earth-Moon orbital geometries that are applicable to the search for life in extrasolar planets. Our results indicate that vegetation detection in Earth-like planets will require a considerable level of instrumental precision and will be a difficult task, but not as difficult as the normally weak earthshine signal might seem to suggest.
KW - Astrobiology
KW - Earth
KW - Moon
PB - Institute of Physics Publishing
SN - 0004637X (ISSN)
LA - English
J2 - Astrophys. J.
M3 - Article
DB - Scopus
N1 - Export Date: 23 February 2023; Cited By: 75; Correspondence Address: P. Montanes-Rodriguez; Big Bear Solar Observatory, New Jersey Institute of Technology, Newark, NJ 07102, United States; email: pmr@bbso.njit.edu
ER -

TY - CONF
AU - Abelson, R.D.
AU - Spilker, T.R.
AU - Shirley, J.H.
AU - Green, J.R.
AU - Smythe, W.D.
TI - Exploring Europa with an RPS-powered orbiter spacecraft
PY - 2006
T2 - IEEE Aerospace Conference Proceedings
VL - 2006
C7 - 1656005
UR - https://www.scopus.com/inward/record.uri?eid=2-s2.0-34047099455&partnerID=40&md5=7f06ac0ce7cb1606c0fcd04e751e5366
AD - Jet Propulsion Laboratory, California Institute of Technology, Mail Stop 301-445W, Pasadena, CA 91109-8099, 4800 Oak Grove Drive, United States
AB - The National Research Council's Solar System Exploration Decadal Survey identified Jupiter's moon Europa as its highest-priority destination for near-term exploration. Voyager and Galileo missions to the Jupiter system provided evidence consistent with a subsurface ocean on Europa, which is of great interest as a potential abode for extraterrestrial life. This paper describes a conceptual flagship-class Europa orbiter concept that was assumed to launch as early as 2012, arriving at Europa approximately 8 years later using inner solar system gravity assists to reach Jupiter. Jupiter's intense radiation environment limits the mission duration at Europa to 30 days for this study, though the duration is a result of multiple trades and is by no means fixed. The Europa Subgroup of

the Outer Planets Assessment Group identified six primary science objectives for this concept. An ~150-kg instrument suite selected for the study addresses those objectives. Large heliocentric distances, high power levels required, and especially the harsh Jovian radiation environment drove the selection of radioisotope thermoelectric generators (RTGs) for all onboard electrical power, with the excess heat aiding spacecraft thermal control. Mass and architecture trades were performed using different spacecraft trajectories, launch vehicle types, radioisotope power systems, and mission durations. The study shows that new mission constraints allow a scientifically compelling Europa orbiter mission that might also deliver a Europa lander. © 2006 IEEE.
KW - Electric generators
KW - Orbits
KW - Radioisotopes
KW - Solar system
KW - Space research
KW - Thermoelectricity
KW - Galileo missions
KW - Thermoelectric generators
KW - Voyager missions
KW - Spacecraft
SN - 1095323X (ISSN); 0780395468 (ISBN); 978-078039546-6 (ISBN)
LA - English
J2 - IEEE Aerosp. Conf. Proc.
M3 - Conference paper
DB - Scopus
N1 - Export Date: 23 February 2023; Cited By: 1; Correspondence Address: R.D. Abelson; Jet Propulsion Laboratory, California Institute of Technology, Mail Stop 301-445W, Pasadena, CA 91109-8099, 4800 Oak Grove Drive, United States; email: Robert.D.Abelson@jpl.nasa.gov
ER -

TY - JOUR
AU - Trimble, V.
AU - Aschwanden, M.J.
AU - Hansen, C.J.
TI - Astrophysics in 2005
PY - 2006
T2 - Publications of the Astronomical Society of the Pacific
VL - 118
IS - 845
SP - 947
EP - 1047
DO - 10.1086/506157
UR - https://www.scopus.com/inward/record.uri?eid=2-s2.0-33748434639&doi=10.1086%2f506157&partnerID=40&md5=55bbbf3d2066861806636037d8908b92
AD - Department of Physics and Astronomy, University of California, Irvine, CA 92697-4575, United States
AD - Las Cumbres Observatory, Santa Barbara, CA, United States
AD - Solar and Astrophysics Laboratory, Lockheed Martin Advanced Technology Center, Organization ADBS, Palo Alto, CA 94304, 3251

Hanover Street, United States
AD - JILA, Department of Astrophysical and Planetary Sciences, University of Colorado, Boulder, CO 80309, United States
AB - We bring you, as usual, the Sun and Moon and stars, plus some galaxies and a new section on astrobiology. Some highlights are short (the newly identified class of gam+ma-ray bursts, and the Deep Impact on Comet 9P/Tempel 1), some long (the age of the universe, which will be found to have the Earth at its center), and a few metonymic, for instance the term "down-sizing" to describe the evolution of star formation rates with redshift. © 2006. The Astronomical Society of the Pacific. All rights reserved.
SN - 00046280 (ISSN)
LA - English
J2 - Publ. Astron. Soc. Pac.
M3 - Article
DB - Scopus
N1 - Export Date: 23 February 2023; Cited By: 4; Correspondence Address: V. Trimble; Department of Physics and Astronomy, University of California, Irvine, CA 92697-4575, United States; email: vtrimble@uci.edu
ER -

TY - CONF
AU - Agenjo, A.
AU - Amano, M.
AU - Auclair, S.
AU - Betorz, F.
AU - Chartres, J.
AU - Yibing, D.
AU - Eisele, S.
AU - Gagnon, M.
AU - Galley, N.
AU - Garcia, N.
AU - Gauthier, J.-F.
AU - Gill, T.
AU - Griffith, K.
AU - Zhonglai, G.
AU - Harkins, D.T.
AU - Haslam, D.T.
AU - Kwan, T.H.
AU - King, A.
AU - Kuvaeva, V.
AU - MacPhee, S.
AU - Mbezal, H.W.
AU - Murray, M.
AU - O'Gallachoir, P.
AU - O'Regan, J.J.
AU - Onoue, N.
AU - Ozlati, S.
AU - Pell, S.J.
AU - Poirier, S.
AU - Takahashi, H.
AU - Tateshita, Y.
AU - Tranfield, E.

AU - Vandersteen, J.
AU - Marshall, W.
AU - Schingler, R.
AU - Lupisella, M.
TI - Luna Gaia: Minimal capabilities required for a self-sustainable lunar settlement
PY - 2006
T2 - AIAA 57th International Astronautical Congress, IAC 2006
VL - 3
SP - 1724
EP - 1738
UR - https://www.scopus.com/inward/record.uri?eid=2-s2.0-41149128275&partnerID=40&md5=db2752638e9d5e330eec6b55a4c44a4e
AD - ISU-SSP'06, Spain
AD - ISU-SSP'06, Japan
AD - ISU-SSP'06, Canada
AD - ISU-SSP'06, Australia
AD - ISU-SSP'06, China
AD - ISU-SSP'06, United States
AD - ISU-SSP'06, Ireland
AD - ISU-SSP'06, United Kingdom
AD - ISU-SSP'06, Russian Federation
AD - ISU-SSP'06, France
AD - ISU-SSP'06, Belgium
AD - NASA Ames, United Kingdom
AD - Space Generation Advisory Council (SGAC), United States
AD - NASA Goddard SFC, United States
AB - To achieve self-sustainability on the lunar surface, a human outpost will require the capability to replace all critical life-support materials and technologies after the initial transport of supplies and infrastructure from Earth. This paper analyzes architectures to optimize time and resources (such as mass to Lunar surface) to achieve self-sustainability. A timeline identifies the critical path and maps necessary technologies that are both currently available and need to be developed to enable the local replacement of all critical life-support technologies. These requirements should be a key consideration in the priorities and planning for Lunar exploration efforts, and in this context the paper discusses how these requirements can be incorporated into the exploration architecture. Luna Gaia posits a pathway towards new technologies, philosophies, systems applications and infrastructure aimed at achieving a closed loop habitat model for human settlement on the Moon. This report makes recommendations pertaining to the systems architecture, engineering processes, and the research, development and orchestration of separate phased precursor missions which will be required to achieve this vision by the year 2030. The framework that we propose is designed to support an ideal profile of an optimum 11 (maximum 12) member human crew on the lunar surface for a period of 18 - 36 months. The Luna Gaia design solutions focus on the coupling power for all regenerative processes of a network of closed loop life support. Using proven and innovative solutions that produce relatively independent and highly reliable cycles of oxygen, water, energy, food growth and waste processing, the modular, hybrid bioregenerative network of systems particular to the Luna Gaia

design architecture is ambitious but feasible. This report also details ethical and philosophical considerations of lunar settlement and the wider implications for international law, policy and future interplanetary social governance. The authors intend to evolve the current status of thought and practice on these issues to consider new and responsible configurations of resource assets - on Earth and the Moon - and to inspire the will and confidence necessary to propel humanity, and its technology, towards the next frontier of lunar settlement. The management principles are sound, the Earth-based applications are considered and the legal frameworks have been clearly defined. Certain risks are apparent but there are significant opportunities and benefits which will occur. More importantly, the project vision is consistent with the preservation of life and responsible evolution into the solar system. We appeal to interested agencies and research organizations to support the Luna Gaia Vision and to encourage author participation in advancing these mission studies. Luna Gaia affirms our commitment to global participation in the extension of human presence on the Moon, and beyond.
KW - Astrobiology
KW - Life support systems (spacecraft)
KW - Lunar surface analysis
KW - Optimization
KW - Stereo vision
KW - Bioregenerative network
KW - Life-support materials
KW - Luna Gaia
KW - Self sustainability
KW - Lunar missions
SN - 978-160560039-0 (ISBN)
LA - English
J2 - AIAA Int. Astronaut. Congr. IAC
M3 - Conference paper
DB - Scopus
N1 - Export Date: 23 February 2023; Cited By: 0; Correspondence Address: A. Agenjo; ISU-SSP'06, Spain; email: alfredo.agenjo@sener.es
ER -

TY - JOUR
AU - Neish, C.D.
AU - Lorenz, R.D.
AU - O'Brien, D.P.
TI - The potential for prebiotic chemistry in the possible cryovolcanic dome Ganesa Macula on Titan
PY - 2006
T2 - International Journal of Astrobiology
VL - 5
IS - 1
SP - 57
EP - 65
DO - 10.1017/S1473550406002898
UR - https://www.scopus.com/inward/record.uri?eid=2-s2.0-33746390462&doi=10.1017%2fS1473550406002898&partnerID=40&md5=3c

c429aa3fde8e753d5af4c8a9532103
AD - Lunar and Planetary Laboratory, University of Arizona, Tucson, AZ 85721, United States
AD - Planetary Science Institute, Tucson, AZ 85719, 1700 E. Ft. Lowell, United States
AB - New observations of Titan by the Cassini spacecraft suggest the presence of cryovolcanism on the surface. Cryovolcanism has important astrobiological implications, as it provides a means of exposing Titan's organics to liquid water, transforming hydrocarbons and nitriles into more evolved and oxidized prebiotic species. One possible cryovolcano - the 180 km structure Ganesa Macula - resembles the pancake domes seen on Venus by the Magellan spacecraft. To assess the potential of Ganesa Macula for prebiotic chemistry, we estimate its height using radarclinometry and other methods, and calculate the freezing timescale assuming an initially completely liquid dome. Given height constraints of ~200 m to 4 km, we find that liquid water or water-ammonia environments could be sustained in Ganesa Macula for timescales of the order of 102-105 years. These timescales open a window for prebiotic chemistry far wider than can be explored in terrestrial laboratory experiments. Press.
KW - Ganesa
KW - dome
KW - origin of life
KW - planetary surface
KW - Titan
KW - Cryovolcanism
KW - Origin of life
KW - Prebiotic chemistry
KW - Prebiotic environments
KW - Titan
SN - 14753006 (ISSN)
LA - English
J2 - Int. J. Astrobiology
M3 - Article
DB - Scopus
N1 - Export Date: 23 February 2023; Cited By: 37; Correspondence Address: C.D. Neish; Lunar and Planetary Laboratory, University of Arizona, Tucson, AZ 85721, United States; email: cdneish@lpl.arizona.edu
ER -

TY - JOUR
AU - Abyzov, S.S.
AU - Duxbury, N.S.
AU - Bobin, N.E.
AU - Fukuchi, M.
AU - Hoover, R.B.
AU - Kanda, H.
AU - Mitskevich, I.N.
AU - Mulyukin, A.L.
AU - Naganuma, T.
AU - Poglazova, M.N.
AU - Ivanov, M.V.

TI - Super-long anabiosis of ancient microorganisms in ice and terrestrial models for development of methods to search for life on Mars, Europa and other planetary bodies
PY - 2006
T2 - Advances in Space Research
VL - 38
IS - 6
SP - 1191
EP - 1197
DO - 10.1016/j.asr.2005.05.034
UR - https://www.scopus.com/inward/record.uri?eid=2-s2.0-33751027424&doi=10.1016%2fj.asr.2005.05.034&partnerID=40&md5=60d8ad6ea40cedda066856a8f8d98c75
AD - Winogradsky Institute of Microbiology, Russian Academy of Sciences, Moscow, 117312, Pr. 60-letiya Oktyabrya 7/2, Russian Federation
AD - JPL, California Institute of Technology, Pasadena, CA, 4800 Oak Grove Dr, United States
AD - St. Petersburg Mining Institute, St. Petersburg, 199026, Russian Federation
AD - National Institute of Polar Research, Tokyo, Japan
AD - Space Science Department, NASA, NSSTC, Huntsville, AL 35805, 320 Sparkman Dr., United States
AD - School of Biosphere Science, Hiroshima University, Higashi-hiroshima, Japan
AB - Successful missions to Mars, Europe and other bodies of the Solar system have created a prerequisite to search for extraterrestrial life. The first attempts of microbial life detection on the Martian surface by the Viking landed missions gave no biological results. Microbiological investigations of the Martian subsurface ground ice layers seem to be more promising. It is well substantiated to consider the Antarctic ice sheet and the Antarctic and Arctic permafrost as terrestrial analogues of Martian habitats. The results of our long-standing microbiological studies of the Antarctic ice would provide the basis for detection of viable microbial cells on Mars. Our microbiological investigations of the deepest and thus most ancient strata of the Antarctic ice sheet for the first time gave evidence for the natural phenomenon of long-term anabiosis (preservation of viability and vitality for millennia years). A combination of classical microbiological methods, epifluorescence microscopy, SEM, TEM, molecular diagnostics, radioisotope labeling and other techniques made it possible for us to obtain convincing proof of the presence of pro- and eukaryotes in the Antarctic ice sheet. In this communication, we will review and discuss some critical issues related to the detection of viable microorganisms in cold terrestrial environments with regard to future searches for microbial life and/or its biological signatures on extraterrestrial objects. © 2005 COSPAR.
KW - Extraterrestrial atmospheres
KW - Ice
KW - Martian surface analysis
KW - Mathematical models
KW - Permafrost
KW - Antarctic ice sheet

KW - Comets
KW - Icy satellites
KW - Super-long anabiosis
KW - Terrestrial analogs of Martian habitats
KW - Viable microorganisms
KW - Microorganisms
KW - Antarctic ice sheet
KW - Comets
KW - Europa
KW - Icy satellites
KW - Super-long anabiosis
KW - Terrestrial analogs of Martian habitats
KW - Viable microorganisms
PB - Elsevier Ltd
SN - 02731177 (ISSN)
LA - English
J2 - Adv. Space Res.
M3 - Article
DB - Scopus
N1 - Export Date: 23 February 2023; Cited By: 16; Correspondence Address: A.L. Mulyukin; Winogradsky Institute of Microbiology, Russian Academy of Sciences, Moscow, 117312, Pr. 60-letiya Oktyabrya 7/2, Russian Federation; email: andylm@rambler.ru; CODEN: ASRSD
ER -

TY - JOUR
AU - Turnbull, M.C.
AU - Traub, W.A.
AU - Jucks, K.W.
AU - Woolf, N.J.
AU - Meyer, M.R.
AU - Gorlova, N.
AU - Skrutskie, M.F.
AU - Wilson, J.C.
TI - Spectrum of a habitable world: Earthshine in the near-infrared
PY - 2006
T2 - Astrophysical Journal
VL - 644
IS - 1
SP - 551
EP - 559
DO - 10.1086/503322
UR - https://www.scopus.com/inward/record.uri?eid=2-s2.0-33747069610&doi=10.1086%2f503322&partnerID=40&md5=a0fd5174eeb04226e139fc83295107a3
AD - Carnegie Institution of Washington, Washington, DC 20008, 5241 Broad Branch Road, United States
AD - NASA Astrobiology Institute, United States
AD - Jet Propulsion Laboratory, MS 301-451, Pasadena, CA 91109, 4800 Oak Grove Drive, United States
AD - Harvard-Smithsonian Center for Astrophysics, Cambridge, MA 02138, 60 Garden Street, United States
AD - Steward Observatory, University of Arizona, Tucson, AZ 85721, United States

AD - Department of Astronomy, University of Virginia, Charlottesville, VA 22903, United States
AB - To characterize the spectrum of Earth viewed as an extrasolar planet, we observed the spatially integrated near-infrared (0.7-2.4 µm) reflection spectrum of Earth via the dark side of the Moon (earthshine). After contributions from the Sun, Moon, and local atmosphere were removed, the resulting spectrum was fitted with a simple model of the reflectivity of Earth. The best model fit is dominated by the reflection spectrum of the atmosphere above medium-altitude water clouds, with lesser contributions from high-altitude ice clouds and from the ground. The spectral features seen are H2O (six strong band structures from 0.7 to 2.0 µm), CO 2 (six moderate-strength features from 1.4 to 2.1 µm), O 2 (two moderate-strength features at 0.76 and 1.26 µm), and several weak CH4 features. Interpreted as a spectrum of an extrasolar planet, we would confidently conclude that this is a habitable planet, based on the presence of strong water bands. Furthermore, the simultaneous presence of oxygen and methane is a strong indicator of biological activity. We might also conclude that the planet is geologically active, based on the presence of CO2, water, and a dynamic atmosphere (inferred from cirrus clouds, cumulus clouds, and clear-air fractions in our model fit). This suggests that it would be valuable for the Terrestrial Planet Finder-Coronagraph (TPF-C) mission to include near-infrared spectroscopy capability. On the basis of the present work, we suggest that future long-term monitoring of the earthshine would allow us to discern how the globally integrated spectrum changes with planet rotation, cloud cover, and seasons. © 2006. The American Astronomical Society. All rights reserved.
KW - Astrobiology
KW - Earth
KW - Moon
KW - Planetary systems
PB - Institute of Physics Publishing
SN - 0004637X (ISSN)
LA - English
J2 - Astrophys. J.
M3 - Article
DB - Scopus
N1 - Export Date: 23 February 2023; Cited By: 99; Correspondence
Address: M.C. Turnbull; Carnegie Institution of Washington, Washington, DC 20008, 5241 Broad Branch Road, United States; email: turnbull@dtm.ciw.edu
ER -

TY - CONF
AU - Briot, D.
AU - Agabi, K.
AU - Aristidi, E.
AU - Arnold, L.
AU - François, P.
AU - Riaud, P.
AU - Rocher, P.
AU - Schneider, J.

TI - A test for the detection of vegetation on extrasolar planets: Detection of vegetation in Earthshine spectrum and its diurnal variation
PY - 2006
T2 - Proceedings of the International Astronomical Union
VL - 2
IS - 14
SP - 711
EP - 712
DO - 10.1017/S1743921307012379
UR - https://www.scopus.com/inward/record.uri?eid=2-s2.0-37049014761&doi=10.1017%2fS1743921307012379&partnerID=40&md5=075dd4ef981f7072f4118f60e40a487f
AD - Observatoire de Paris-Meudon, F-75014 Paris, 61 avenue de l'Observatoire, France
AD - Université de Nice - Sophia Antipolis, F-06108 Nice cedex 2, 28 avenue Valrose, France
AD - Observatoire de Haute-Provence-CNRS, F-04870 Saint Michel l'Observatoire, France
AD - European Southern Observatory (ESO), Vitacura, Santiago 19, 3107 Av. de Cordova, Chile
AD - Observatoire de Paris-Meudon, F-92195 Meudon, 5 place Jules Janssen, France
AB - The search for life in extraterrestrial planets is to be tested first with the only planet known to shelter life. If the planet Earth is used as an example to search for a signature of life, the vegetation is one of its possible detectable signature, using the Vegetation Red Edge due to chlorophyll in the near infrared (0.725 m). We focus on the test of the detectability of vegetation in the spectrum of Earth seen as a simple dot, using the reflection of the global Earth on the lunar surface, i.e., Earthshine. On the Antartic, the Earthshine can be seen during several hours in a day (not possible at our latitudes) and so variations due to different parts of Earth, that is to say oceans and continents facing the Moon could be detected. © 2007 International Astronomical Union.
KW - Astrobiology
KW - Biomarker
KW - Earth
KW - Earthshine
KW - Exolife
KW - Moon
KW - Techniques: spectroscopy
KW - Vegetation red edge
SN - 17439221 (ISSN)
LA - English
J2 - Proc. Int. Astron. Union
M3 - Conference paper
DB - Scopus
N1 - Export Date: 23 February 2023; Cited By: 1; Correspondence Address: D. Briot; Observatoire de Paris-Meudon, F-75014 Paris, 61 avenue de l'Observatoire, France; email: Danielle.Briot@obspm.fr
ER -

TY - JOUR
AU - Burchell, M.J.
TI - The special issue devoted to papers from the Astrobiology Society of Britain Conference 2006
PY - 2006
T2 - International Journal of Astrobiology
VL - 5
IS - 3
SP - 181
DO - 10.1017/S1473550406003454
UR - https://www.scopus.com/inward/record.uri?eid=2-s2.0-33845544664&doi=10.1017%2fS1473550406003454&partnerID=40&md5=90483d9f6461da646b0585c587138480
AD - Centre for Astrophysics and Planetary Science, School of Physical Science, University of Kent, Canterbury, Kent CT2 7NH, United Kingdom
AB - The Astrobiology Society of Britain (http://www.astrobiologysociety.org/ held its second conference (Life Here, There and Everywhere) in April 2006. The meeting was hosted by the University of Kent and took place on campus in Canterbury from 18-21 April. A total of 48 abstracts were submitted to the meeting. The final schedule featured 35 talks and 14 posters on a range of topics which included Mars, the Moon, comets, exoplanets, impacts, outreach and analytic techniques - all with an astrobiology flavour. With 62 attendees (including overseas delegates from Sweden, Spain, Portugal, Australia and the United States) the meeting also allowed for strong social interaction (and the building of new collaborations and networking) as well as showcasing ongoing research in the field. © 2006 Cambridge University Press.
SN - 14753006 (ISSN)
LA - English
J2 - Int. J. Astrobiology
M3 - Editorial
DB - Scopus
N1 - Export Date: 23 February 2023; Cited By: 1; Correspondence Address: M.J. Burchell; Centre for Astrophysics and Planetary Science, School of Physical Science, University of Kent, Canterbury, Kent CT2 7NH, United Kingdom; email: m.j.burchell@kent.ac.uk
ER -

TY - JOUR
AU - Reid, I.N.
AU - Sparks, W.B.
AU - Lubow, S.
AU - McGrath, M.
AU - Livio, M.
AU - Valenti, J.
AU - Sowers, K.R.
AU - Shukla, H.D.
AU - MacAuley, S.
AU - Miller, T.
AU - Suvanasuthi, R.
AU - Belas, R.
AU - Colman, A.

AU - Robb, F.T.
AU - DasSarma, P.
AU - Müller, J.A.
AU - Coker, J.A.
AU - Cavicchioli, R.
AU - Chen, F.
AU - DasSarma, S.
TI - Terrestrial models for extraterrestrial life: Methanogens and halophiles at Martian temperatures
PY - 2006
T2 - International Journal of Astrobiology
VL - 5
IS - 2
SP - 89
EP - 97
DO - 10.1017/S1473550406002916
UR - https://www.scopus.com/inward/record.uri?eid=2-s2.0-33750338843&doi=10.1017%2fS1473550406002916&partnerID=40&md5=7e75b8de79051aaf692a9f80081b5c62
AD - Maryland Astrobiology Consortium, United States
AD - Space Telescope Science Institute, Baltimore, MD 21218, 3700 San Martin Drive, United States
AD - Marshall Spaceflight Center, Huntsville, AL 35812, United States
AD - University of Maryland Biotechnology Institute, Center of Marine Biotechnology, Baltimore, MD 21202, United States
AD - Department of Biology, Morgan State University, Baltimore, MD 21215, United States
AD - School of Biotechnology and Biomolecular Sciences, University of New South Wales, Sydney, NSW 2052, Australia
AB - Cold environments are common throughout the Galaxy. We are conducting a series of experiments designed to probe the low-temperature limits for growth in selected methanogenic and halophilic Archaea. This paper presents initial results for two mesophiles, a methanogen, Methanosarcina acetivorans, and a halophile, Halobacterium sp. NRC-1, and for two Antarctic cold-adapted Archaea, a methanogen, Methanococcoides burtonii, and a halophile, Halorubrum lacusprofundi. Neither mesophile is active at temperatures below 5 °C, but both cold-adapted microorganisms show significant growth at sub-zero temperatures (-2 °C and -1 °C, respectively), extending previous low-temperature limits for both species by 4-5 °C. At low temperatures, both H. lacusprofundi and M. burtonii form multicellular aggregates, which appear to be embedded in extracellular polymeric substances. This is the first detection of this phenomenon in Antarctic species of Archaea at cold temperatures. The low-temperature limits for both psychrophilic species fall within the temperature range experienced on present-day Mars and could permit survival and growth, particularly in sub-surface environments. We also discuss the results of our experiments in the context of known exoplanet systems, several of which include planets that intersect the Habitable Zone. In most cases, those planets follow orbits with significant eccentricity, leading to substantial temperature excursions. However, a handful of the known gas giant exoplanets could potentially harbour habitable terrestrial

moons. © 2006 Cambridge University Press.
KW - Archaea
KW - Halobacterium
KW - Halobacterium sp. NRC-1
KW - Halorubrum
KW - Halorubrum lacusprofundi
KW - Methanobacteriales
KW - Methanococcoides burtonii
KW - Methanosarcina
KW - Methanosarcina acetivorans
KW - biofilm
KW - cryobiology
KW - exobiology
KW - extraterrestrial matter
KW - Mars
KW - methanogenic bacterium
KW - Archaea
KW - Astrobiology
KW - Biofilm
KW - Extrasolar planets
KW - Mars
KW - Psychrophiles
SN - 14753006 (ISSN)
LA - English
J2 - Int. J. Astrobiology
M3 - Article
DB - Scopus
N1 - Export Date: 23 February 2023; Cited By: 66; Correspondence Address: I.N. Reid; Maryland Astrobiology Consortium, United States; email: inr@stsci.edu
ER -

TY - JOUR
AU - Battler, M.M.
AU - Clarke, J.D.A.
AU - Coniglio, M.
TI - Possible analog sedimentary and diagenetic features for Meridiani planum sediments near Hanksville, Utah: Implications for martian field studies
PY - 2006
T2 - Science and Technology Series
VL - 111
SP - 55
EP - 69
UR - https://www.scopus.com/inward/record.uri?eid=2-s2.0-33845467790&partnerID=40&md5=3362fc53c036a4ae766455b70a4e2ef2
AD - University of New Brunswick, Planetary and Space Science Centre, Fredericton, NB E3B5A3, 2 Bailey Dr., Canada
AD - Australian Centre for Astrobiology, Macquarie University, NSW 2109, Australia
AD - Department of Earth Sciences, University of Waterloo, Waterloo, Ont. N2L 3G1, Canada
AB - Layered hematitic deposits at Meridiani Planum have been extensively studied by the Opportunity rover and found to be

composed of cross bedded, rippled, and laminated sandstone and siltstone, with abundant sulfate molds. Hematite occurs as mostly spheroidal concretions and as crosscutting veins. Flat-lying Jurassic to Cretaceous sediments in the vicinity of the Mars Society's Mars Desert Research Station (MDRS) contain features remarkably similar to those in the Meridiani Planum sediments. The Jurassic Summerville Formation is a marginal marine sandstone and siltstone containing abundant gypsum as scattered crystals, nodules, and cross-cutting veins. The Cretaceous Dakota sandstone contains iron-rich, spheroidal concretions with calcite cement of probable microbially mediated origin, which weather resistantly out of exposed surfaces. This paper provides a preliminary description of these features. The MDRS field area highlights the importance of detailed geologic studies of terrestrial analogs of Martian sedimentological and diagenetic settings, particularly as they bear on the search for extraterrestrial life.
KW - Crystals
KW - Geological surveys
KW - Gypsum
KW - Sandstone
KW - Sediments
KW - Extraterrestrial life
KW - Hematitic deposits
KW - Marine sandstone
KW - Mars Desert Research Station (MDRS)
KW - Martian surface analysis
SN - 02784017 (ISSN)
LA - English
J2 - Sci. Technol. Ser.
M3 - Article
DB - Scopus
N1 - Export Date: 23 February 2023; Cited By: 9; Correspondence Address: M.M. Battler; University of New Brunswick, Planetary and Space Science Centre, Fredericton, NB E3B5A3, 2 Bailey Dr., Canada; email: melissa.battler@unb.ca; CODEN: AASTB
ER -

TY - CONF
AU - O'Malley, T.F.
AU - Weiland, K.J.
AU - Myhre, C.A.
AU - Gati, F.G.
AU - Hill, M.E.
AU - Snead, J.H.
TI - The fluids and combustion facility and first payloads, the multiuser droplet combustion apparatus and the light microscopy module: Enabling research on the ISS
PY - 2006
T2 - AIAA 57th International Astronautical Congress, IAC 2006
VL - 1
SP - 557
EP - 566
DO - 10.2514/6.iac-06-a2.1.07
UR - https://www.scopus.com/inward/record.uri?eid=2-

s2.0-41149116643&doi=10.2514%2f6.iac-06-a2.1.07&partnerID=40&md5=133781512dd1d98de38caa7cb90be624
AD - NASA Glenn Research Center, Cleveland, OH, United States
AB - The Fluids and Combustion Facility (FCF) is an International Space Station (ISS) research facility designed to support physical and biological research and exploration-related technology experiments in space. The FCF consists of two modular, reconfigurable racks called the Combustion Integrated Rack (CIR) and the Fluids Integrated Rack (FIR). The CIR can accommodate experiments that address critical needs in the areas of spacecraft fire safety (i.e., fire prevention, detection and suppression), flame spread, sooting, in-situ resource utilization, environmental monitoring and materials synthesis. The FIR can accommodate experiments that address critical needs for advanced life support (i.e., air revitalization, water reclamation, etc.), power, propulsion, and spacecraft thermal control systems. Specific investigations could include boiling heat transfer, multiphase flow, and liquid/vapor evaporation/ condensation. The initial payload in the CIR is the Multi-User Droplet Combustion Apparatus (MDCA), which will perform the Flame Extinguishment in Microgravity experiment (FLEX). MDCA consists of two major components: a Chamber Insert Assembly and an Avionics Package. The initial FIR payload is the Light Microscopy Module (LMM), which will perform the Constrained Vapor Bubble (CVB) experiment. The LMM is an automated, fully motorized sub-rack mini-facility based on the Leica RXA microscope.
KW - Aircraft propulsion
KW - Fire protection
KW - Optical microscopy
KW - Space rendezvous
KW - Space stations
KW - Combustion Integrated Rack (CIR)
KW - Droplet combustion
KW - Fluids and Combustion Facility (FCF)
KW - Fluids Integrated Rack (FIR)
KW - Astrobiology
PB - American Institute of Aeronautics and Astronautics Inc.
SN - 978-160560039-0 (ISBN)
LA - English
J2 - AIAA Int. Astronaut. Congr. IAC
M3 - Conference paper
DB - Scopus
N1 - Export Date: 23 February 2023; Cited By: 0; Correspondence Address: T. F. O'Malley; NASA Glenn Research Center, Cleveland, OH, United States; email: tfomalley@nasa.gov
ER -

TY - JOUR
AU - DasSarma, S.
TI - Extreme halophiles are models for astrobiology
PY - 2006
T2 - Microbe
VL - 1
IS - 3
SP - 120

EP - 126
DO - 10.1128/microbe.1.120.1
UR - https://www.scopus.com/inward/record.uri?eid=2-s2.0-33750334684&doi=10.1128%2fmicrobe.1.120.1&partnerID=40&md5=d9e8eb2dae8fb68711d2495ba6c27f2d
AD - University of Maryland Biotechnology Institute, Center of Marine Biotechnology, Baltimore, MD, United States
AB - Studying extremophiles such as halophiles on Earth may provide insights helpful in our search for life elsewhere in the universe. A common characteristic among halophiles is that they contain a preponderance of acidic proteins, better enabling them to dissolve and to function in high-salt environments. A model halophile, Halobacterium sp. NRC-1, is being studied for its ability to withstand many environmental insults, including damaging radiation, extremes of temperature, low oxygen, and exposure to heavy metals. Retinal-based phototrophy, first discovered in halophiles and now known to be prevalent in common marine microbes, may be one of the oldest metabolic capabilities on Earth.
KW - Halobacterium
KW - Halobacterium sp. NRC-1
KW - halophilic archaeon
PB - American Society for Microbiology
SN - 15587452 (ISSN)
LA - English
J2 - Microbe
M3 - Review
DB - Scopus
N1 - Export Date: 23 February 2023; Cited By: 61
ER -

TY - JOUR
AU - Shepherd, V.A.
TI - The Cytomatrix as a Cooperative System of Macromolecular and Water Networks
PY - 2006
T2 - Current Topics in Developmental Biology
VL - 75
SP - 171
EP - 223
DO - 10.1016/S0070-2153(06)75006-2
UR - https://www.scopus.com/inward/record.uri?eid=2-s2.0-33748569494&doi=10.1016%2fS0070-2153%2806%2975006-2&partnerID=40&md5=72cedde13261b7b17c080dbfca50638b
AD - Department of Biophysics, School of Physics, The University of NSW NSW 2052, Sydney, Australia
AB - Water was called by Szent-Gyorgi "life's mater and matrix, mother and medium." This chapter considers both aspects of his statement. Many astrobiologists argue that some, if not all, of Earth's water arrived during cometary bombardments. Amorphous water ices of comets possibly facilitated organization of complex organic molecules, kick-starting prebiotic evolution. In Gaian theory, Earth retains its water as a consequence of biological activity. The cell cytomatrix is a proteinaceous matrix/lattice incorporating the cytoskeleton, a pervasive, holistic superstructural network that

integrates metabolic pathways. Enzymes of metabolic pathways are ordered in supramolecular clusters (metabolons) associated with cytoskeleton and/or membranes. Metabolic intermediates are microchanneled through metabolons without entering a bulk aqueous phase. Rather than being free in solution, even major signaling ions are probably clustered in association with the cytomatrix. Chloroplasts and mitochondria, like bacteria and archaea, also contain a cytoskeletal lattice, metabolons, and channel metabolites. Eukaryotic metabolism is mathematically a scale-free or small-world network. Enzyme clusters of bacterial origin are incorporated at a pathway level that is architecturally archaean. The eucaryotic cell may be a product of serial endosymbiosis, a chimera. Cell cytoplasm is approximately 80% water. Water is indisputably a conserved structural element of proteins, essential to their folding, specificity, ligand binding, and to enzyme catalysis. The vast literature of organized cell water has long argued that the cytomatrix and cell water are an entire system, a continuum, or gestalt. Alternatives are offered to mainstream explanations of cell electric potentials, ion channel, enzyme, and motor protein function, in terms of high-order cooperative systems of ions, water, and macromolecules. This chapter describes some prominent concepts of organized cell water, including vicinal water network theory, the association-induction hypothesis, wave-cluster theory, phase-gel transition theories, and theories of low- and high-density water polymorphs. © 2006 Elsevier Inc. All rights reserved.
KW - Animals
KW - Cytoplasm
KW - Humans
KW - Macromolecular Substances
KW - Water
KW - water
KW - animal
KW - chemistry
KW - cytoplasm
KW - human
KW - macromolecule
KW - metabolism
KW - physiology
KW - review
A2 - Schatten G.P.
SN - 00702153 (ISSN); 0121531759 (ISBN); 978-012153175-1 (ISBN)
C2 - 16984813
LA - English
J2 - Curr. Top. Dev. Biol.
M3 - Review
DB - Scopus
N1 - Export Date: 23 February 2023; Cited By: 37
ER -

TY - JOUR
AU - Martinez-Frias, J.
AU - Amaral, G.
AU - Vázquez, L.
TI - Astrobiological significance of minerals on Mars surface

environment
PY - 2006
T2 - Reviews in Environmental Science and Biotechnology
VL - 5
IS - 2-3
SP - 219
EP - 231
DO - 10.1007/s11157-006-0008-x
UR - https://www.scopus.com/inward/record.uri?eid=2-s2.0-33748696784&doi=10.1007%2fs11157-006-0008-x&partnerID=40&md5=f4b8816011256d7d9480893889af4bcc
AD - Centro de Astrobiología (CSIC-INTA), Madrid, Spain
AD - Departamento de Química Física I, Facultad de Ciencias Químicas, Universidad Complutense, 28040 Madrid, Spain
AD - Departamento de Matemática Aplicada, Facultad de Informática, Universidad Complutense de Madrid, 28040 Madrid, Spain
AB - Despite the large amount of geomorphological, geodynamic and geophysical data obtained from Mars missions, much is still unknown about Martian mineralogy and paragenetic assemblages, which is fundamental to an understanding of its entire geological history. Minerals are not only indicators of the physical-chemical settings of the different environments and their later changes, but also they could (and do) play a crucial astrobiological role related with the possibility of existence of extinct or extant Martian life. This paper aims: (1) to present a synoptic review of the main water-related Martian minerals (mainly jarosite and other sulfates) discovered up to the present time; (2) to emphasize their significance as environmental geomarkers, on the basis of their geological settings and mineral parageneses on earth (in particular in the context of some selected terrestrial analogues), and (3) to show that their differential UV shielding properties, against the hostile environmental conditions of the Martian surface, are of a great importance for the search for extraterrestrial life. © Springer Science+Business Media B.V. 2006.
KW - Astrobiology
KW - Extreme environment
KW - Gypsum
KW - Jarosite
KW - Mars minerals
KW - Sulfates
KW - UV radiation
SN - 15691705 (ISSN)
LA - English
J2 - Rev. Environ. Sci. Biotechnol.
M3 - Review
DB - Scopus
N1 - Export Date: 23 February 2023; Cited By: 27; Correspondence Address: J. Martinez-Frias; Centro de Astrobiología (CSIC-INTA), Madrid, Spain; email: martinezfrias@mncn.csic.es; CODEN: RESBC
ER -

TY - JOUR
AU - Covault, C.
TI - Mars Beckons: Water evidence boosts exploration and prospects

for new astrobiology rover
PY - 2006
T2 - Aviation Week and Space Technology (New York)
VL - 165
IS - 23
SP - 26
EP - 28
UR - https://www.scopus.com/inward/record.uri?eid=2-s2.0-33845518325&partnerID=40&md5=3a7c72cddcd9f2ffce4e9c359afa270f
AB - The discovery of Mars Global Surveyor (MGS) which provides the evidence that water exists on the surface of Mars will focus new robotic and manned exploration strategies for the planet. It could accelerate development of a rover that would be the first US spacecraft in 30 years geared specifically to search for alien life. MGS found that at two sites water has gushed out at a rate of 75,000-150,000 gal. at a time in just few months, leaving evaporating water or mineral deposits imaged by the spacecraft. NASA is expected to accelerate development of a new $1.5 billion Mars Astrobiology Field Laboratory (AFL) rover that would be launched in 2016-18 to directly search for current Martian life. The new water discovery will also explore the NASA astrobiology programs largely sacrificed in the budget to pay for recovery from the space shuttle Columbia accident.
KW - Martian surface analysis
KW - Planets
KW - Space shuttles
KW - Spacecraft
KW - Astrobiology
KW - Astrobiology Field Laboratory (AFL)
KW - Mars Global Surveyor (MGS)
KW - Space research
SN - 00052175 (ISSN)
LA - English
J2 - Aviat Week Space Technol (New York)
M3 - Article
DB - Scopus
N1 - Export Date: 23 February 2023; Cited By: 0; CODEN: AWSTA
ER -

TY - CONF
AU - Sheldon, R.B.
AU - Hoover, R.B.
TI - Implications of cometary water: Deep impact, stardust and hayabusa
PY - 2006
T2 - Proceedings of SPIE - The International Society for Optical Engineering
VL - 6309
C7 - 63090L
DO - 10.1117/12.681232
UR - https://www.scopus.com/inward/record.uri?eid=2-s2.0-33750718866&doi=10.1117%2f12.681232&partnerID=40&md5=bded65a45c0d33ad707ef0cb8a9eeb79
AD - NASA/MSFC/NSSTC, Huntsville, AL, 320 Sparkman Dr, United

States
AB - Three recent in situ spacecraft missions have explored comets or asteroids, producing data in conflict with the standard comet paradigm, the Whipple Dirty Snowball Model (DSM). We have developed an alternative Wet Comet Model (WCM) which proposes that comets undergo an irreversible phase change to a wet comet when they enter within Mars orbit. The WCM may explain some of the observational discrepancies seen by Deep Impact, Stardust and Hayabusa. In particular, it accurately predicted Deep Impact observation of organics, biominerals, and meltwater temperatures. Predictions concerning Stardust's returned cometary dust particles have yet to be falsified, but if comets are largely composed of the silicates seen by Stardust, there may be a cometary explanation for Itokawa's low density rubble-pile observed by Hayabusa.
KW - Astronomy
KW - Mathematical models
KW - Spacecraft
KW - Water
KW - Hayabusa
KW - Meltwater temperatures
KW - Orbital dynamics
KW - Stardust
KW - Wet Comet Model (WCM)
KW - Space research
KW - Astrobiology
KW - Comet
KW - Deep impact
KW - Geysers
KW - Hayabusa
KW - Liquid water
KW - Orbital dynamics
KW - Stardust
SN - 0277786X (ISSN); 0819463884 (ISBN); 978-081946388-3 (ISBN)
LA - English
J2 - Proc SPIE Int Soc Opt Eng
M3 - Conference paper
DB - Scopus
N1 - Export Date: 23 February 2023; Cited By: 10; Correspondence Address: R.B. Sheldon; NASA/MSFC/NSSTC, Huntsville, AL, 320 Sparkman Dr, United States; email: Rob.Sheldon@msfc.nasa.gov; CODEN: PSISD
ER -

TY - JOUR
AU - Steven, B.
AU - Léveillé, R.
AU - Pollard, W.H.
AU - Whyte, L.G.
TI - Microbial ecology and biodiversity in permafrost
PY - 2006
T2 - Extremophiles
VL - 10
IS - 4
SP - 259
EP - 267

DO - 10.1007/s00792-006-0506-3
UR - https://www.scopus.com/inward/record.uri?eid=2-s2.0-33744902917&doi=10.1007%2fs00792-006-0506-3&partnerID=40&md5=56f10c84f2d4e0ae2cf2ac1e66861e93
AD - Department of Natural Resource Sciences, McGill University, Ste-Anne de Bellevue, Que. H9X 3V9, 21, 111 Lakeshore Rd, Canada
AD - Canadian Space Agency, Saint-Hubert, Que. J3Y 8Y9, 6767, Route de l'Aéroport, Canada
AD - Department of Geography, McGill University, W. Montreal, Que. H3A 2K6, 805 Sherbrooke St., Canada
AB - Permafrost represents 26% of terrestrial soil ecosystems; yet its biology, essentially microbiology, remains relatively unexplored. The permafrost environment is considered extreme because indigenous microorganisms must survive prolonged exposure to subzero temperatures and background radiation for geological time scales in a habitat with low water activity and extremely low rates of nutrient and metabolite transfer. Yet considerable numbers and biodiversity of bacteria exist in permafrost, some of which may be among the most ancient viable life on Earth. This review describes the permafrost environment as a microbial habitat and reviews recent studies examining microbial biodiversity found in permafrost as well as microbial growth and activity at ambient in situ subzero temperatures. These investigations suggest that functional microbial ecosystems exist within the permafrost environment and may have important implications on global biogeochemical processes as well as the search for past or extant life in permafrost presumably present on Mars and other bodies in our solar system. © Springer-Verlag 2006.
KW - Acclimatization
KW - Bacteria
KW - Biodiversity
KW - Cold Climate
KW - Exobiology
KW - Soil Microbiology
KW - Bacteria (microorganisms)
KW - acclimatization
KW - article
KW - astrobiology
KW - bacterium
KW - biodiversity
KW - biology
KW - cold climate
KW - isolation and purification
KW - metabolism
KW - microbiology
KW - Astrobiology
KW - Cold-adaptation
KW - Dormancy
KW - Permafrost
SN - 14334909 (ISSN)
C2 - 16550305
LA - English
J2 - Extremophiles
M3 - Article

DB - Scopus
N1 - Export Date: 23 February 2023; Cited By: 163; Correspondence Address: L.G. Whyte; Department of Natural Resource Sciences, McGill University, Ste-Anne de Bellevue, Que. H9X 3V9, 21, 111 Lakeshore Rd, Canada; email: whyte@nrs.mcgill.ca; CODEN: EXTRF
ER -

TY - JOUR
AU - Hode, T.
AU - Zebühr, Y.
AU - Broman, C.
TI - Towards biomarker analysis of hydrocarbons trapped in individual fluid inclusions: First extraction by ErYAG laser
PY - 2006
T2 - Planetary and Space Science
VL - 54
IS - 15
SP - 1575
EP - 1583
DO - 10.1016/j.pss.2006.02.008
UR - https://www.scopus.com/inward/record.uri?eid=2-s2.0-33751011876&doi=10.1016%2fj.pss.2006.02.008&partnerID=40&md5=86c384c988a722132c7d52983837b567
AD - Department of Paleozoology, Swedish Museum of Natural History, 10405 Stockholm, P.O. Box 50007, Sweden
AD - Institute of Applied Environmental Research, Stockholm University, Stockholm, Sweden
AD - Department of Geology and Geochemistry, Stockholm University, Stockholm, Sweden
AB - Fluid inclusions act as sealed vessels containing information about the fluid environment in which the minerals precipitated, and until decrepitated, the chemical composition of the fluid inside the inclusion stays intact. In many cases fluid inclusions contain trapped hydrocarbons, which may provide useful information in paleontological, organic geochemical and astrobiological research since they act as containers of non-contaminated organic matter with a defined minimum age. Here we present a novel concept for extraction of fluid inclusions in preparation for application to extract single fluid inclusions. The method is based on the illumination of a sample with an ErYAG laser (λ = 2940 nm). The wavelength of the laser is absorbed by water and organic material, and with the minerals encapsulating the inclusions transparent to the wavelength, the fluid will expand and the inclusion will decrepitate. The initial results of our study demonstrate that fluid inclusions can be extracted by the use of an ErYAG laser, and that organic biomarkers may survive the process, readily available for GC-MS analysis. © 2006 Elsevier Ltd. All rights reserved.
KW - Biomarkers
KW - Composition
KW - Earth (planet)
KW - Lasers
KW - Martian surface analysis
KW - Precipitation (chemical)
KW - Fluid inclusions

KW - GC-MS
KW - Laser extraction
KW - Hydrocarbons
KW - Biomarkers
KW - Early Earth
KW - Fluid inclusions
KW - GC-MS
KW - Hydrocarbons
KW - Laser extraction
KW - Mars
SN - 00320633 (ISSN)
LA - English
J2 - Planet. Space Sci.
M3 - Article
DB - Scopus
N1 - Export Date: 23 February 2023; Cited By: 16; Correspondence Address: T. Hode; Department of Paleozoology, Swedish Museum of Natural History, 10405 Stockholm, P.O. Box 50007, Sweden; email: hode@pdx.edu; CODEN: PLSSA
ER -

TY - JOUR
AU - Willis, M.J.
AU - Ahrens, T.J.
AU - Bertani, L.E.
AU - Nash, C.Z.
TI - Bugbuster-survivability of living bacteria upon shock compression
PY - 2006
T2 - Earth and Planetary Science Letters
VL - 247
IS - 3-4
SP - 185
EP - 196
DO - 10.1016/j.epsl.2006.03.054
UR - https://www.scopus.com/inward/record.uri?eid=2-s2.0-33745640069&doi=10.1016%2fj.epsl.2006.03.054&partnerID=40&md5=109ffc09a2556a5e69507b6a0aff1bf3
AD - Lindhurst Laboratory of Experimental Geophysics, Pasadena, CA 91125, Caltech 252-21, United States
AD - Division of Biological Sci., Pasadena, CA 91125, Caltech 156-29, United States
AD - Division of Geol. Planet. Sci., Pasadena, CA 91125, Caltech 170-25, United States
AB - Shock recovery experiments were conducted on suspensions of 106/ml E. coli bacteria contained in a water-based medium that is emplaced within stainless steel containers. The water is shocked and recovered. These experiments simulate the environment of bacteria residing either in surface bodies of water or in subsurface water-filled cracks in rocks. Early Earth life is likely to have existed in such environments. However, the E. coli are not believed to be representative of early life and are merely used here for initial experiments. Some 10- 2 to 10- 4 of the bacteria population survived initial (800 ns duration) shock pressures in water of 220 and

260 MPa. TEM images of shock recovered bacteria indicate cell wall rupture and delamination. This appears to be the mortality mechanism. The TEM images indicate cell wall indentations may be occurring as would be consistent with Rayleigh–Taylor or Richtmyer–Meshkov fluid instabilities. In the present case, we consider the experiments as representing three layers of fluids: (1) The water-based medium, a stronger and possibly denser cell wall medium, and the interior of the cell cytoplasm. Variations of only 10–15% are expected in density. (2) A second mechanism that may cause cell wall failure is the multiple shock (nearly isentropic) compression freezing of liquid water medium into ice VI or ice VII high pressure phase that are 20% to 25% denser than the liquid. The decrease in volume associated with the transformation is expected to induce overpressures in the still liquid cell cytoplasm. Cell dynamic tensile wall strength thus appears to be a critical parameter from either of the above failure modes. Because the strain rate dependence of cell wall tensile strength is unstudied, we utilize the Grady and Lipkin [D.E. Grady, L. Lipkin, Criteria for impulsive rock fracture, Geophys. Res. Lett. 7 (1980) 255–258] model of tensile failure versus time scale (strain rate). Our single datum is fit to this law and we assume that at low strain rates, overpressures exceeding the cell Turgor pressure require on the order of ~103 s. This model which has been applied to brittle media and metals for describing failure may permit application of short duration laboratory experiments as in the present ones to infer responses of organisms to much lower shock pressures, but for longer time scales (100 to 103 s) of planetary impacts. Using the present data for E. coli and applying the Grady and Lipkin model, we find that a 1.5 km diameter impactor will cause mortality of bacteria within a radius of 102 km but upon stress related attenuation the subsurface bacteria outside of this radius should survive. © 2006.
KW - Biology
KW - Cell membranes
KW - Delamination
KW - Indentation
KW - Liquids
KW - Pressure effects
KW - Shock testing
KW - Strain rate
KW - Tensile strength
KW - Transmission electron microscopy
KW - Cell wall rupture
KW - Grady and Lipkin model
KW - Mortality mechanism
KW - Rayleigh–Taylor fluid instability
KW - Richtmyer–Meshkov fluid instability
KW - Shock compression
KW - Shock recovered bacteria
KW - coliform bacterium
KW - microbial activity
KW - survival
KW - Escherichia coli
KW - astrobiology
KW - bacteria

KW - origin of life
KW - shock compression
SN - 0012821X (ISSN)
LA - English
J2 - Earth Plan. Sci. Lett.
M3 - Article
DB - Scopus
N1 - Export Date: 23 February 2023; Cited By: 30; Correspondence Address: T.J. Ahrens; Lindhurst Laboratory of Experimental Geophysics, Pasadena, CA 91125, Caltech 252-21, United States; email: tja@caltech.edu
ER -

TY - JOUR
AU - Trainer, M.G.
AU - Pavlov, A.A.
AU - DeWitt, H.L.
AU - Jimenez, J.L.
AU - McKay, C.P.
AU - Toon, O.B.
AU - Tolbert, M.A.
TI - Organic haze on Titan and the early Earth
PY - 2006
T2 - Proceedings of the National Academy of Sciences of the United States of America
VL - 103
IS - 48
SP - 18035
EP - 18042
DO - 10.1073/pnas.0608561103
UR - https://www.scopus.com/inward/record.uri?eid=2-s2.0-33845330579&doi=10.1073%2fpnas.0608561103&partnerID=40&md5=42034d889dd56f264f4f51462798f845
AD - Laboratory for Atmospheric and Space Physics, University of Colorado, UCB 392, Boulder, CO 80309, United States
AD - Department of Planetary Sciences, Lunar and Planetary Laboratory, University of Arizona, Tucson, AZ 85721, 1629 East University Boulevard, United States
AD - Department of Chemistry and Biochemistry, Cooperative Institute for Research in Environmental Sciences, University of Colorado, Boulder, CO 80309, United States
AD - Space Science Division, NASA Ames Research Center, Moffett Field, CA 94035, United States
AD - Laboratory for Atmospheric and Space Physics, Department of Atmospheric and Oceanic Sciences, University of Colorado, Boulder, CO 80309, United States
AB - Recent exploration by the Cassini/Huygens mission has stimulated a great deal of interest in Saturn's moon, Titan. One of Titan's most captivating features is the thick organic haze layer surrounding the moon, believed to be formed from photochemistry high in the CH4/N2 atmosphere. It has been suggested that a similar haze layer may have formed on the early Earth. Here we report laboratory experiments that demonstrate the properties of haze likely to form through photochemistry on Titan and early Earth. We have used a

deuterium lamp to initiate particle production in these simulated atmospheres from UV photolysis. Using a unique analysis technique, the aerosol mass spectrometer, we have studied the chemical composition, size, and shape of the particles produced as a function of initial trace gas composition. Our results show that the aerosols produced in the laboratory can serve as analogs for the observed haze in Titan's atmosphere. Experiments performed under possible conditions for early Earth suggest a significant optical depth of haze may have dominated the early Earth's atmosphere. Aerosol size measurements are presented, and implications for the haze layer properties are discussed. We estimate that aerosol production on the early Earth may have been on the order of 1014 g·year−1 and thus could have served as a primary source of organic material to the surface. © 2006 by The National Academy of Sciences of the USA.
KW - carbon
KW - carbon dioxide
KW - carbon monoxide
KW - hydrogen
KW - methane
KW - nitrogen
KW - aerosol
KW - article
KW - astronomy
KW - atmosphere
KW - haze
KW - mass spectrometer
KW - particle size
KW - photochemistry
KW - photolysis
KW - priority journal
KW - transmission electron microscopy
KW - ultraviolet radiation
KW - Archaen
KW - Astrobiology
KW - Atmospheric aerosol
KW - Planetary atmospheres
KW - Tholins
SN - 00278424 (ISSN)
LA - English
J2 - Proc. Natl. Acad. Sci. U. S. A.
M3 - Article
DB - Scopus
N1 - Export Date: 23 February 2023; Cited By: 165; Correspondence Address: M.A. Tolbert; Department of Chemistry and Biochemistry, Cooperative Institute for Research in Environmental Sciences, University of Colorado, Boulder, CO 80309, United States; email: tolbert@cires.colorado.edu; CODEN: PNASA
ER -

TY - JOUR
AU - Bishop, J.L.
AU - Louris, S.K.
AU - Rogoff, D.A.
AU - Rothschild, L.J.

TI - Nanophase iron oxides as a key ultraviolet sunscreen for ancient photosynthetic microbes
PY - 2006
T2 - International Journal of Astrobiology
VL - 5
IS - 1
SP - 1
EP - 12
DO - 10.1017/S1473550406002886
UR - https://www.scopus.com/inward/record.uri?eid=2-s2.0-33746409587&doi=10.1017%2fS1473550406002886&partnerID=40&md5=162af25094cf78dbe398fa6e293b4c2c
AD - SETI Institute, Mountain View, CA 94043, 515 N. Whisman Road, United States
AD - NASA-Ames Research Center, Moffett Field, CA 94035, Mail Stop 239-4, United States
AD - NASA-Ames Research Center, Moffett Field, CA 94035, Mail Stop 239-20, United States
AD - Stanford University, Program in Human Biology, Stanford, CA 94305, United States
AB - We propose that nanophase iron-oxide-bearing materials provided important niches for ancient photosynthetic microbes on the Earth that ultimately led to the oxygenation of the Earth's atmosphere and the formation of iron-oxide deposits. Atmospheric oxygen and ozone attenuate ultraviolet radiation on the Earth today providing substantial protection for photosynthetic organisms. With ultraviolet radiation fluxes likely to have been even higher on the early Earth than today, accessing solar radiation was particularly risky for early organisms. Yet, we know that photosynthesis arose early and played a critical role in subsequent evolution. Of primary importance was protection below 290 nm, where peak nucleic acid (~260 nm) and protein (~280 nm) absorptions occur. Nanophase ferric oxide/oxyhydroxide minerals absorb, and thus block, the lethal ultraviolet radiation, while transmitting light through much of the visible and near-infrared regions of interest to photosynthesis (400 to 1100 nm). Furthermore, they were available in early environments, and are synthesized by many organisms. Based on experiments using nanophase ferric oxide/oxyhydroxide minerals as a sunscreen for photosynthetic microbes, we suggest that iron, an abundant element widely used in biological mechanisms, may have provided the protection that early organisms needed in order to be able to use photosynthetically active radiation while being protected from ultraviolet-induced damage. The results of this study are broadly applicable to astrobiology because of the abundance of iron in other potentially habitable bodies and the evolutionary pressure to utilize solar radiation when available as an energy source. This model could apply to a potential life form on Mars or other bodies where liquid water and ultraviolet radiation could have been present at significant levels. Based on ferric oxide/oxyhydroxide spectral properties, likely geologic processes, and the results of experiments with the photosynthetic organisms, Euglena sp. and Chlamydomonas reinhardtii, we propose a scenario where photosynthesis, and ultimately the oxygenation of the atmosphere, depended on the protection of early microbes by nanophase ferric

oxides/oxyhydroxides.
KW - Chlamydomonas reinhardtii
KW - Euglena
KW - banded iron formation
KW - early Earth
KW - iron oxide
KW - photosynthesis
KW - spectroscopy
KW - ultraviolet radiation
KW - Banded iron formations
KW - Early Earth
KW - Iron oxides
KW - Optical spectroscopy
KW - Oxygenated atmosphere
KW - Photosynthesis
KW - Ultraviolet radiation
SN - 14753006 (ISSN)
LA - English
J2 - Int. J. Astrobiology
M3 - Article
DB - Scopus
N1 - Export Date: 23 February 2023; Cited By: 14; Correspondence Address: J.L. Bishop; SETI Institute, Mountain View, CA 94043, 515 N. Whisman Road, United States; email: jbishop@arc.nasa.gov
ER -

TY - JOUR
AU - Ikoma, M.
AU - Genda, H.
TI - Constraints on the mass of a habitable planet with water of nebular origin
PY - 2006
T2 - Astrophysical Journal
VL - 648
IS - 1 I
SP - 696
EP - 706
DO - 10.1086/505780
UR - https://www.scopus.com/inward/record.uri?eid=2-s2.0-33748808685&doi=10.1086%2f505780&partnerID=40&md5=04596e4a63f197fd086d3c6daf834944
AD - Research Center for the Evolving Earth and Planets, Tokyo Institute of Technology, Ookayama, Meguro-ku, Tokyo 152-8551, Japan
AD - Department of Earth and Planetary Sciences, Tokyo Institute of Technology, Ookayama, Meguro-ku, Tokyo 152-8551, Japan
AB - From an astrobiological point of view, special attention has been paid to the probability of habitable planets in extrasolar systems. The purpose of this study is to constrain a possible range of the mass of a terrestrial planet that can get water. We focus on the process of water production through oxidation of atmospheric hydrogen - the nebular gas having been attracted gravitationally - by oxides available at the planetary surface. For the water production to work well on a planet, a sufficient amount of hydrogen and a temperature high enough to melt the planetary surface are

needed. We have simulated the structure of the atmosphere that connects with the protoplanetary nebula for wide ranges of the heat flux, the opacity, and the density of the nebular gas. We have found that both requirements are fulfilled for an Earth-mass planet for wide ranges of the parameters. We have also found that the surface temperature of planets of ≤0.3ME (where ME is Earth's mass) is lower than the melting temperature of silicate (~ 1500 K). On the other hand, a planet of more than several ME becomes a gas giant through runaway accretion of the nebular gas. © 2006. The American Astronomical Society. All rights reserved.
KW - Astrobiology
KW - Earth
KW - Planets and satellites: formation
PB - Institute of Physics Publishing
SN - 0004637X (ISSN)
LA - English
J2 - Astrophys. J.
M3 - Article
DB - Scopus
N1 - Export Date: 23 February 2023; Cited By: 123; Correspondence Address: M. Ikoma; Research Center for the Evolving Earth and Planets, Tokyo Institute of Technology, Ookayama, Meguro-ku, Tokyo 152-8551, Japan; email: mikoma@geo.titech.ac.jp
ER -

TY - JOUR
TI - AAS to hand out DPS awards
PY - 2006
T2 - Physics Today
VL - 59
IS - 8
SP - 68
EP - 69
DO - 10.1063/1.2349738
UR - https://www.scopus.com/inward/record.uri?eid=2-s2.0-33750033900&doi=10.1063%2f1.2349738&partnerID=40&md5=cb3408681f22779a10dbda031cd510d7
AB - The American Astronomical Society's division for planetary sciences will honor the individuals contributing in the field of lab techniques and instrument design, systems engineering, public outreach, and the study of gas giants. David Grinspoon, curator of astrobiology in the space sciences department at the Denver Museum of Nature and Science, will be awarded with the Carl Sagan Medal for his energetic and successful efforts to get the general public thinking about planets, solar system and wider universe. The Gerard P. Kuiper Prize will be awarded to Dale P. Cruikshank for his pioneering work in the application of infrared spectroscopy to solar system bodies. Gentry Lee, chief engineer for the planetary flight systems directorate at NASA's Jet Propulsion Laboratory at Caltech, will receive the Harold Masursky Award for his fundamental contributions to systems engineering in the complex world of robotic planetary missions.
KW - Infrared spectroscopy
KW - Research laboratories

KW - Robotics
KW - Societies and institutions
KW - Solar system
KW - Systems engineering
KW - Astrobiology
KW - Planetary sciences
KW - Space sciences
KW - Planets
SN - 00319228 (ISSN)
LA - English
J2 - Phys Today
M3 - Article
DB - Scopus
N1 - Export Date: 23 February 2023; Cited By: 0; CODEN: PHTOA
ER -

TY - JOUR
AU - Fendrihan, S.
AU - Legat, A.
AU - Pfaffenhuemer, M.
AU - Gruber, C.
AU - Weidler, G.
AU - Gerbl, F.
AU - Stan-Lotter, H.
TI - Extremely halophilic archaea and the issue of long-term microbial survival
PY - 2006
T2 - Reviews in Environmental Science and Biotechnology
VL - 5
IS - 2-3
SP - 203
EP - 218
DO - 10.1007/s11157-006-0007-y
UR - https://www.scopus.com/inward/record.uri?eid=2-s2.0-33748702485&doi=10.1007%2fs11157-006-0007-y&partnerID=40&md5=295748ca31134e09ff6c95091d5fa95a
AD - Department of Microbiology, University of Salzburg, A-5020 Salzburg, Billrothstr. 11, Austria
AB - Halophilic archaebacteria (haloarchaea) thrive in environments with salt concentrations approaching saturation, such as natural brines, the Dead Sea, alkaline salt lakes and marine solar salterns; they have also been isolated from rock salt of great geological age (195-250 million years). An overview of their taxonomy, including novel isolates from rock salt, is presented here; in addition, some of their unique characteristics and physiological adaptations to environments of low water activity are reviewed. The issue of extreme long-term microbial survival is considered and its implications for the search for extraterrestrial life. The development of detection methods for subterranean haloarchaea, which might also be applicable to samples from future missions to space, is presented. © Springer Science+Business Media B.V. 2006.
KW - Archaea
KW - Halobacteria
KW - Halobacteriaceae

KW - Halobacterium
KW - halophilic archaeon
KW - Extreme halophiles
KW - Haloarchaea
KW - Life detection
KW - Microbial longevity
KW - Salt mines
KW - Salt sediments
KW - Space missions
KW - Subterranean
KW - Taxonomy of halobacteriaceae
SN - 15691705 (ISSN)
LA - English
J2 - Rev. Environ. Sci. Biotechnol.
M3 - Review
DB - Scopus
N1 - Export Date: 23 February 2023; Cited By: 79; Correspondence Address: H. Stan-Lotter; Department of Microbiology, University of Salzburg, A-5020 Salzburg, Billrothstr. 11, Austria; email: helga.stan-lotter@sbg.ac.at; CODEN: RESBC
ER -

TY - CONF
AU - Grinberg, A.
TI - Dust mitigation methods for long duration lunar missions
PY - 2006
T2 - AIAA 57th International Astronautical Congress, IAC 2006
VL - 14
SP - 9312
EP - 9317
UR - https://www.scopus.com/inward/record.uri?eid=2-s2.0-40749091728&partnerID=40&md5=ad91c5b65602ec2ec40ec053fdf9a1d8
AD - International Space University, Strasbourg, France
AB - Lunar dust is one of the primary environmental factors posing a risk to long duration lunar missions. The Apollo missions documented respiratory problems resulting from the presence of lunar dust carried inside the Lunar Module following extra-vehicular activities, as well as engineering issues due to its adhesion and abrasion properties. [1,2] Lunar dust is expected to pose significant challenges during long duration exploration of the lunar surface in terms of respiratory physiology, spacesuit and infrastructure lifetime, and surface operations inside and outside the habitat. [2] This paper discusses the advantages of forming an international, interdisciplinary team of scientists, engineers, flight surgeons, and planners to address the problems lunar dust presents to long duration lunar exploration. Approaching the problem in such a fashion will ensure a balanced, efficient, and useful solution that addresses all necessary components of mission design. These components include the formation of medical and design requirements as well as risk guidelines to ensure detailed designs are safe and reliable for astronauts in terms of dust. Designs for habitats, vehicles, scientific instruments, and spacesuits must be durable to sustain long term operations despite the environmental hazard. Surface operations are also needed to support infrastructure

design and provide dust monitoring, maintenance, and repair activities. Lastly, the Moon is a useful analogue to Mars. The two have many common challenges such as dust mitigation. [3] When designing lunar missions, consideration must be given to strategies that will also benefit future Mars exploration. The Moon is a near-future destination for astronauts, and care must be taken to ensure their health given the autonomous nature and isolation from earth support that these missions present.
KW - Dust
KW - Health risks
KW - Lunar missions
KW - Lunar surface analysis
KW - Pulmonary diseases
KW - Dust mitigation
KW - Extra-vehicular activity
KW - Long duration
KW - Lunar dust
KW - Mission design
KW - Astrobiology
KW - Dust mitigation
KW - Extra-vehicular activity
KW - Habitat
KW - Long duration
KW - Lunar dust
KW - Mission design
KW - Moon
KW - Operations
SN - 978-160560039-0 (ISBN)
LA - English
J2 - AIAA Int. Astronaut. Congr. IAC
M3 - Conference paper
DB - Scopus
N1 - Export Date: 23 February 2023; Cited By: 0; Correspondence Address: A. Grinberg; International Space University, Strasbourg, France; email: Anna.Grinberg@gmail.com
ER -

TY - JOUR
AU - Fernández-Calvo, P.
AU - Näke, C.
AU - Rivas, L.A.
AU - García-Villadangos, M.
AU - Gómez-Elvira, J.
AU - Parro, V.
TI - A multi-array competitive immunoassay for the detection of broad-range molecular size organic compounds relevant for astrobiology
PY - 2006
T2 - Planetary and Space Science
VL - 54
IS - 15
SP - 1612
EP - 1621
DO - 10.1016/j.pss.2006.02.007

UR - https://www.scopus.com/inward/record.uri?eid=2-s2.0-33750993162&doi=10.1016%2fj.pss.2006.02.007&partnerID=40&md5=0e14dca12897c9f1b005d4adab749292
AD - Centro de Astrobiología (INTA-CSIC), 28850 Torrejon de Ardoz, Madrid, Carretera de Ajalvir km 4, Spain
AB - We have developed antibodies and a multi-array competitive immunoassay (MACIA) for the detection of a wide range of molecular size compounds, from single aromatic ring derivatives or polycyclic aromatic hydrocarbons (PAHs), through small peptides, proteins or whole cells (spores). Multiple microarrays containing target molecules are used simultaneously to run several competitive immunoassays. The sensitivity of the MACIA for small organic compounds like naphthalene, 4-phenilphenol or 4-tertbutilphenol is in the range of 100-500 ppb (ng ml-1), for others like the insecticide terbutryn it is at the ppt (ng l-1) level, while for small peptides, as well as for more complex molecules like the protein thioredoxin, the sensitivity is approximately 1-2 ppb, or 104-105 spores of Bacillus subtilis per milliliter. For organic compounds, a water-methanol solution was used in order to achieve a better dissolution of the organics without compromising the antibody-antigen interaction. The above-mentioned compounds were detected by MACIA in water-(10%) methanol extracts from spiked pyrite and hematite-containing rock powder samples, as well as from a spiked-sand sample subjected to organic extraction with dichloromethane-methanol (1/1). © 2006 Elsevier Ltd. All rights reserved.
KW - Cells
KW - Immunology
KW - Methanol
KW - Polycyclic aromatic hydrocarbons
KW - Polypeptides
KW - Proteins
KW - Astrobiology
KW - Molecular size compounds
KW - Pyrite
KW - Assays
SN - 00320633 (ISSN)
LA - English
J2 - Planet. Space Sci.
M3 - Article
DB - Scopus
N1 - Export Date: 23 February 2023; Cited By: 26; Correspondence Address: V. Parro; Centro de Astrobiología (INTA-CSIC), 28850 Torrejon de Ardoz, Madrid, Carretera de Ajalvir km 4, Spain; email: parrogv@inta.es; CODEN: PLSSA
ER -

TY - CONF
AU - Des Marais, D.J.
TI - Advancing astrobiology beyond Viking, Spirit and Opportunity
PY - 2006
T2 - Collection of Technical Papers - Space 2006 Conference
VL - 3
SP - 2032

EP - 2039
DO - 10.2514/6.2006-7415
UR - https://www.scopus.com/inward/record.uri?eid=2-s2.0-33846483857&doi=10.2514%2f6.2006-7415&partnerID=40&md5=463aedce55481ee6c6e9dc4e653819b2
AD - NASA Ames Research Center, Moffett Field, CA 94035-1000, United States
AB - Habitable environments must provide key chemical ingredients, biologically utilizable energy sources, and clement conditions where life can be active at least intermittently and thus ensure its survival. To find evidence of life we must explore atmospheres and crustal deposits that could have retained biosignatures (objects, substances or patterns that require biological origins). Then we must identify those biosignatures definitively. Viking, Spirit and Opportunity revealed that, although habitable environments probably do not occur at the Martian surface today, they might have existed at least intermittently in the past. Opportunity discovered that liquid water once stood on the surface. Spirit found that volcanic rocks had been altered by water in ways that could have provided chemical energy to sustain life. Recent missions revealed that the Martian crust has diverse compositions and has probably preserved an ancient record of aqueous activity and environmental changes. To enhance ongoing exploration of this crustal record for evidence of habitable environments and life, astrobiologists must participate in landing site selection and flight instrument development. Future landers should seek and examine geologic deposits (e.g., clays, evaporites, silica, carbonates, hydrothermal deposits, etc.) that might have formed in habitable environments and also could have preserved biosignatures. We must develop our concepts of biosignatures further to address potentially confusing nonbiological features that will inevitably arise.
KW - Environmental impact
KW - Geology
KW - Martian surface analysis
KW - Probability
KW - Space flight
KW - Volcanic rocks
KW - Astrobiology
KW - Biosignatures
KW - Geologic deposits
KW - Habitable environments
KW - Space research
PB - American Institute of Aeronautics and Astronautics Inc.
SN - 1563478242 (ISBN); 978-156347824-6 (ISBN)
LA - English
J2 - Coll. Technic. Papers Space 2006 Conf.
M3 - Conference paper
DB - Scopus
N1 - Export Date: 23 February 2023; Cited By: 0
ER -

TY - JOUR
AU - Osinski, G.R.
AU - Léveillé, R.

AU - Berinstain, A.
AU - Lebeuf, M.
AU - Bamsey, M.
TI - Terrestrial analogues to Mars and the moon: Canada's role
PY - 2006
T2 - Geoscience Canada
VL - 33
IS - 4
SP - 175
EP - 188
UR - https://www.scopus.com/inward/record.uri?eid=2-s2.0-39149106807&partnerID=40&md5=99e5e3a5742eaf108590421b9e6c0e88
AD - Canadian Space Agency, Space Science, Saint-Hubert, QC J3Y 8Y9, 6767 Route de l'Aeroport, Canada
AB - Terrestrial analogues are places on Earth that approximate, in some respect, the geological, environmental and putative biological conditions on a particular planetary body, either at the present-day or sometime in the past. Analogue studies are driven by the need to understand processes on Earth in order to interpret and groundtruth data sent back from Mars and other planetary bodies by unmanned orbiters and rovers. This presents an ideal opportunity to further collaboration between the solid earth and planetary science communities in Canada and elsewhere. Analogue environments also provide a critical locale for optimizing exploration requirements and strategies for future manned missions to the Moon and Mars. The ideal geography and climate, a wide diversity of analogues sites, and a history of analogue activities, ensures that Canada can play a leading role in the expanding international use of terrestrial analogues sites.
KW - Emmelichthyidae
KW - Astrobiology
KW - Lunar surface analysis
KW - Structural geology
KW - exploration
KW - Mars
KW - Moon
KW - planetary surface
KW - terrestrial environment
KW - Exploration requirements
KW - Planetary science
KW - Terrestrial analogues
KW - Martian surface analysis
SN - 03150941 (ISSN)
LA - English
J2 - Geosci. Can.
M3 - Article
DB - Scopus
N1 - Export Date: 23 February 2023; Cited By: 40; Correspondence Address: G.R. Osinski; Canadian Space Agency, Space Science, Saint-Hubert, QC J3Y 8Y9, 6767 Route de l'Aeroport, Canada; email: gordon.osinski@space.gc.ca; CODEN: GSCNA
ER -

TY - JOUR

AU - Thaler, T.L.
AU - Gibbs, P.R.
AU - Trebino, R.P.
AU - Bommarius, A.S.
TI - Search for extraterrestrial life using chiral molecules: Mandelate racemase as a test case
PY - 2006
T2 - Astrobiology
VL - 6
IS - 6
SP - 901
EP - 910
DO - 10.1089/ast.2006.6.901
UR - https://www.scopus.com/inward/record.uri?eid=2-s2.0-33846068466&doi=10.1089%2fast.2006.6.901&partnerID=40&md5=e437b524314f690c68a7caee85b80454
AD - School of Chemistry and Biochemistry, Georgia Institute of Technology, Atlanta, GA, United States
AD - Parker H. Petit Institute of Bioengineering and Bioscience, Georgia Institute of Technology, Atlanta, GA 30332-0363, 315 Ferst Drive, United States
AD - Stheno Corporation, Atlanta, GA, United States
AD - School of Physics, Georgia Institute of Technology, Atlanta, GA, United States
AD - School of Chemical and Biomolecular Engineering, Georgia Institute of Technology, Atlanta, GA, United States
AB - We have investigated an enzymatic racemization reaction as a marker for extraterrestrial life, which resulted in a change in optical rotation of a mandelic acid over time, as measured by polarimetry. Mandelate racemase was active in aqueous buffer in a temperature range between 0°C and 70°C and also in concentrated ammonium salt solutions and water-in-oil microemulsions in a temperature range between -30°C and 60-70°C; however, the enzyme was not active in several organic cryosolvents. Thus, we have demonstrated that concentrated ammonium salt solutions and water-in-oil microemulsions, both of which are able to form on extraterrestrial planets and moons in the presence of liquid water, are suitable media for enzyme reactions at subzero temperatures. Kinetic data for the mandelate racemase reaction obtained by polarimetry, while reproducible and internally consistent, differed significantly from several sets of data obtained previously by other methods such as chromatography and hydrogen-deuterium exchange. However, we conclude that reactions yielding a polarimetric signal, such as the racemizations employed in this work, are suitable mechanisms by which to utilize a change in chirality over time as a tool to detect signs of life. © Mary Ann Liebert, Inc.
KW - Buffers
KW - Calorimetry, Differential Scanning
KW - Emulsions
KW - Exobiology
KW - Extraterrestrial Environment
KW - Kinetics
KW - Pseudomonas putida
KW - Racemases and Epimerases

KW - Stereoisomerism
KW - Temperature
KW - buffer
KW - isomerase
KW - mandelate racemase
KW - article
KW - biology
KW - chemistry
KW - differential scanning calorimetry
KW - emulsion
KW - enzymology
KW - genetics
KW - kinetics
KW - metabolism
KW - microclimate
KW - Pseudomonas putida
KW - stereoisomerism
KW - temperature
KW - Chirality
KW - Concentrated ammonium salt solution
KW - Mandelate racemase
KW - Microemulsions
KW - Subzero temperature enzyme activity
SN - 15311074 (ISSN)
C2 - 17155888
LA - English
J2 - Astrobiology
M3 - Article
DB - Scopus
N1 - Export Date: 23 February 2023; Cited By: 7; Correspondence Address: A.S. Bommarius; Parker H. Petit Institute of Bioengineering and Bioscience, Georgia Institute of Technology, Atlanta, GA 30332-0363, 315 Ferst Drive, United States; email: andreas.bommarius@chbe.gatech.edu
ER -

TY - CONF
AU - Storrie-Lombardi, M.C.
AU - Hoover, R.B.
AU - Abbas, M.
AU - Jerman, G.
AU - Coston, J.
AU - Fisk, M.
TI - Probabilistic classification of elemental abundance distributions in Nakhla and Apollo 17 lunar dust samples
PY - 2006
T2 - Proceedings of SPIE - The International Society for Optical Engineering
VL - 6309
C7 - 630906
DO - 10.1117/12.690435
UR - https://www.scopus.com/inward/record.uri?eid=2-s2.0-33750689222&doi=10.1117%2f12.690435&partnerID=40&md5=3521973aa0196ec8e6a3b619147b8599

AD - Kinohi Institute, Pasadena, CA 91101, 530 S. Lake Avenue, #117, United States
AD - NASA National Space Science and Technology Center, Huntsville, AL 35805, 320 Sparkman Drive, United States
AD - College of Oceanic and Atmospheric Sciences, Oregon State University, Corvallis, OR 97331, United States
AB - Analysis of spectral and imaging data from meteoritic samples and sample return missions would benefit significantly from a systematic, quantitative statistical classification methodology and a common set of standards for data collection [McDonald and Storrie-Lombardi, 2006]. Stochastic artificial neural networks can be trained using elemental abundance distributions for the detection of macroscopic fossils [Storrie-Lombardi and Hoover, 2004] and extant microbial life [Storrie-Lombardi and Hoover, 2005]. These non-linear algorithms are particularly attractive since they can produce a Bayesian estimate of the classification accuracy of either human experts or automated, unsupervised classification algorithms. In sub-ocean and surface basalts on earth the networks can distinguish regions of biotic and abiotic alteration of basalt glass from unaltered samples using only elemental abundances as inputs [Storrie-Lombardi and Fisk, 2004b]. Recently, evidence has been presented documenting the presence of morphologic signatures in the Mars meteorite Nakhla [Fisk et al., 2004; Fisk et al., 2006] previously noted in regions of biotic alteration in sub-ocean and surface terrestrial basalts [Fisk et al., 2003; Fumes et al., 2004]. The tunneling alterations are not conclusive evidence of biotic alteration of Nakhla on Mars. However, the meteorite is well known to have experienced aqueous alteration prior to arrival on earth and is rich in carbon [Gibson et al., 2006; McKay et al., 2006]. We here present an initial application of our probabilistic classification strategy to assess elemental abundance distributions from multiple target regions in Nakhla and lunar dust samples collected by Apollo 17 astronauts. We present scanning electron microscope images and elemental abundance point distributions (C, N, O, Na_2O, MgO, Al_2O_3, SiO_2, P_2O_5, S, Cl, K_2O, CaO, and FeO) for a series of target regions. We discuss our observations in the context of data previously presented in these meetings for extant cyanobacteria, fossil trilobites, Orgueil meteorite, and terrestrial basalt targets. These data are being added to a database that will made available to the biogeology and astrobiology communities as part of an ongoing effort to provide a quantitative probabilistic methodology for analysis of putative elemental abundance geobiological signatures.
KW - Bacteria
KW - Meteorites
KW - Neural networks
KW - Probability distributions
KW - Scanning electron microscopy
KW - Space rendezvous
KW - Apollo 17
KW - Bayesian probabilities
KW - Elemental abundances
KW - Elementary particles
KW - Apollo 17

KW - Artificial neural networks
KW - Bayesian probabilities
KW - Elemental abundances
KW - Mars
KW - Meteorites
KW - Moon
KW - Nakhla
SN - 0277786X (ISSN); 0819463884 (ISBN); 978-081946388-3 (ISBN)
LA - English
J2 - Proc SPIE Int Soc Opt Eng
M3 - Conference paper
DB - Scopus
N1 - Export Date: 23 February 2023; Cited By: 2; Correspondence Address: M.C. Storrie-Lombardi; Kinohi Institute, Pasadena, CA 91101, 530 S. Lake Avenue, #117, United States; email: mike@kinohi.org; CODEN: PSISD
ER -

TY - JOUR
AU - Tinetti, G.
AU - Rashby, S.
AU - Yung, Y.L.
TI - Detectability of red-edge-shifted vegetation on terrestrial planets orbiting M stars
PY - 2006
T2 - Astrophysical Journal Letters
VL - 644
IS - 2
SP - L32
EP - L129
DO - 10.1086/505746
UR - https://www.scopus.com/inward/record.uri?eid=2-s2.0-85035139428&doi=10.1086%2f505746&partnerID=40&md5=7ca0276c7a7ef713b304dfcb9a773913
AD - European Space Agency, Institut d'Astrophysique de Paris, Extrasolar Planets Section, Paris, 75014, France
AD - California Institute of Technology, Division of Geological and Planetary Sciences, Pasadena, 91125, CA, United States
AB - We have explored the detectability of exovegetation on the surface of a terrestrial planet orbiting an M star. The exovegetation is responsible for producing a pigment-derived surface signature that is redshifted with respect to the Earth vegetation's red edge. The redshift was estimated using a model of leaf optical property spectra (Jacquemoud & Baret) combined with a 3 photon photosynthetic scheme calculated by Wolstencroft & Raven for a possible exovegetation growing on an M star planet. To study the detectability of this surface biosignature on an M star terrestrial planet, we have used the three-dimensional model developed by Tinetti et al. for the case of the Earth. This model can generate disk-averaged spectra and broadband integrated fluxes, which will be useful for future terrestrial planet exploration missions, such as the NASA Terrestrial Planet Finder Coronagraph. Input to this model were the atmospheric profiles and cloud distributions predicted by Joshi and coworkers for a synchronous planet orbiting an M dwarf and

the distinctive surface reflectance of the exovegetation. While on Earth this pigment-derived surface feature would be almost completely masked by water absorption, even in a cloud-free atmosphere, we found that the strength of the edge feature on our simulated M star terrestrial planet can exceed that on Earth, given the right conditions. Obviously, the detectability of such biosignatures would be highly dependent on the extent of vegetation surface area, cloud cover, and viewing angle. © 2006. The American Astronomical Society. All rights reserved. Printed in U.S.A.
KW - Astrobiology
KW - Atmospheric effects
KW - Radiative transfer
KW - Stars: late-type
PB - Institute of Physics Publishing
SN - 20418205 (ISSN)
LA - English
J2 - Astrophys. J. Lett.
M3 - Article
DB - Scopus
N1 - Export Date: 23 February 2023; Cited By: 27
ER -

TY - CONF
AU - Easton, C.
AU - Calder, A.
AU - Kingsmore, D.B.
AU - Pitsiladis, Y.P.
TI - The effects of a novel "fluid-loading" strategy combining creatine and glycerol on fluid retention and distribution in humans
PY - 2006
T2 - AIAA 57th International Astronautical Congress, IAC 2006
VL - 1
SP - 418
EP - 433
DO - 10.2514/6.iac-06-a1.p.1.05
UR - https://www.scopus.com/inward/record.uri?eid=2-s2.0-41149160762&doi=10.2514%2f6.iac-06-a1.p.1.05&partnerID=40&md5=e5824c9d94ec92c57e5b95fb505423fb
AD - Space Research Laboratory, University of Glasgow, United Kingdom
AD - Department of Anaesthetics, Glasgow Royal Infirmary, United Kingdom
AD - Department of Surgery, Gartnavel General Hospital, United Kingdom
AB - The effects of three "fluid-loading" strategies combining creatine (Cr) and glycerol (GIy) on fluid retention and distribution were examined. Three 7-day Cr and Gly loading protocols (LP) were examined: LP1 - 10 g Cr and 75 g of glucose in 1 litre (L) of water (× 2 daily) and a bolus of 1 g Gly·kg-1 2.5 hours prior to testing (n = 6); LP2 - 10 g Cr, 1 g Gly·kg-1 and 75 g of glucose in 1 L of water (× 2 daily) and final supplement consumed 3 hours prior to testing (n = 6); LP3 - as LP2 but final supplement consumed 5 hours prior to testing (n = 23). Only body mass increased (mean ± s.d.) by 1.59 ± 0.21 kg (P < 0.01) post-LP1 and by 1.20 ± 0.37 kg (P < 0.01)

post-LP2. Post-LP3, body mass increased by 0.97 ± 0.28 kg (P < 0.01) with significant increases in total body water (0.87 ± 0.21 L; P < 0.01), intra-cellular and extra-cellular water and plasma volume. Combining Cr and Gly is potentially a clinically useful "fluid-loading" strategy which merits further evaluation for application in spaceflight.
KW - Body fluids
KW - Glycerol
KW - Moisture determination
KW - Plasma (human)
KW - Body water
KW - Cellular water
KW - Fluid retention
KW - Loading protocols (LP)
KW - Plasma volume
KW - Astrobiology
PB - American Institute of Aeronautics and Astronautics Inc.
SN - 978-160560039-0 (ISBN)
LA - English
J2 - AIAA Int. Astronaut. Congr. IAC
M3 - Conference paper
DB - Scopus
N1 - Export Date: 23 February 2023; Cited By: 1; Correspondence Address: C. Easton; Space Research Laboratory, University of Glasgow, United Kingdom; email: c.easton.1@research.gla.ac.uk
ER -

TY - CONF
AU - Scharf, C.A.
TI - Tidally heated moons: From icy worlds to temperate habitats
PY - 2006
T2 - Proceedings of SPIE - The International Society for Optical Engineering
VL - 6309
C7 - 63090K
DO - 10.1117/12.680945
UR - https://www.scopus.com/inward/record.uri?eid=2-s2.0-33750683081&doi=10.1117%2f12.680945&partnerID=40&md5=956dd4ce8526c96fa536a597a82e257e
AD - Columbia Astrobiology Center, Columbia University, MC5247, New York, NY 10027, 550 West 120th St, United States
AB - Moons of giant planets may represent an alternative to the classical picture of habitable worlds. Within our own solar system Europa has long served as an intriguing candidate for a subsurface liquid water ocean. Sustained by tidal heating, such an ocean can exist well beyond the range at which stellar heating could raise surface temperatures to similar levels. For exoplanets, with their extraordinarily diverse orbital architectures, the same situation may arise, along with a host of other possibilities - including those where a combination of tidal and stellar heating results in water rich moons experiencing temperate surface conditions. The next generation of space-based planet finders and ground based large telescopes should begin to probe the population of moons around exoplanets - thereby opening up a new avenue in the search for life.

We discuss some of these possibilities by investigating the
dynamical constraints on moon systems of giant planets and by
studying the characteristics of a set of 74 known extrasolar giant
planets located beyond 0.6 AU from their parent stars - where moons
should be long-lived with respect to removal by stellar tides. By
estimating the stellar insolation that moons would experience for
these exoplanet systems, and the implications for sublimation loss
of volatiles, we find that between 15 and 27% of all known
exoplanets may be capable of harboring small, icy, moons. In
addition, by applying a simplified energy balance model, we find
that some 22-28% of all known exoplanets could potentially harbor
moons which, if large, could experience temperate surface conditions
due to a combination of tidal and stellar heating. Large moons (>
0.1M⊕), at orbital radii commensurate with those of the Galilean
satellites, could maintain temperate, or habitable, surface
conditions during episodes of tidal heat dissipation ranging from
that seen on Europa to 10-100 times greater. We discuss the
implications of these findings in the context of habitability.
KW - Astronomy
KW - Mathematical models
KW - Moon
KW - Solar system
KW - Telescopes
KW - Astrobiology
KW - Giant planets
KW - Stellar heating
KW - Tidal heat dissipation
KW - Space research
KW - Astrobiology
KW - Planetary systems
KW - Planets and satellites
SN - 0277786X (ISSN); 0819463884 (ISBN); 978-081946388-3 (ISBN)
LA - English
J2 - Proc SPIE Int Soc Opt Eng
M3 - Conference paper
DB - Scopus
N1 - Export Date: 23 February 2023; Cited By: 0; Correspondence
Address: C.A. Scharf; Columbia Astrobiology Center, Columbia
University, MC5247, New York, NY 10027, 550 West 120th St, United
States; email: caleb@astro.columbia.edu; CODEN: PSISD
ER -

TY - CONF
AU - Badescu, M.
AU - Sherrit, S.
AU - Olorunsola, A.
AU - Aldrich, J.
AU - Bao, X.
AU - Bar-Cohen, Y.
AU - Chang, Z.
AU - Doran, P.T.
AU - Fritsen, C.H.
AU - Kenig, F.
AU - McKay, C.P.

AU - Murray, A.
AU - Du, S.
AU - Peterson, T.
AU - Song, T.
TI - Ultrasonic/sonic Gopher for subsurface ice and brine sampling: Analysis and fabrication challenges, and testing results
PY - 2006
T2 - Proceedings of SPIE - The International Society for Optical Engineering
VL - 6171
C7 - 617107
DO - 10.1117/12.657487
UR - https://www.scopus.com/inward/record.uri?eid=2-s2.0-33745891522&doi=10.1117%2f12.657487&partnerID=40&md5=50062dfb4dfd0a4d1b6b26553d8457ce
AD - Jet Propulsion Laboratory, California Institute of Technology, MS 67-119, Pasadena, CA 91109-8099, 4800 Oak Grove Drive, United States
AD - University of Illinois at Chicago, Earth and Environmental Sciences, MC186, Chicago, IL 60607, 845 West Taylor Street, United States
AD - Desert Research Institute, Division of Earth and Ecosystem Sciences, Reno, NV 89512, 2215 Raggio Parkway, United States
AD - NASA Ames Research Center, Division of Space Science, Moffett Field, CA 94035, United States
AD - Cybersonics, Inc., Erie, PA 16510, 5368 Kuhl Rd., United States
AB - Rock, soil, and ice penetration by coring, drilling or abrading is of great importance to a large number of space and earth applications. Proven techniques to sample Mars subsurface will be critical for future NASA astrobiology missions that will search for records of past and present life on the planet. An Ultrasonic/Sonic Drill/Corer (USDC) has been developed as an adaptable tool for many of these applications [Bar-Cohen et al., 2001]. The USDC uses a novel drive mechanism to transform the ultrasonic or sonic vibrations of the tip of a horn into a sonic hammering of a drill bit through an intermediate free-flying mass. The USDC design was modified to fabricate an Ultrasonic/Sonic Ice Gopher that is designed to core down to meters depth for in situ analysis and sample collection. This technology was demonstrated at Lake Vida in the Dry Valleys, Antarctica. Coring ice at -20°C as in Lake Vida has been a challenge and efforts were made to develop the required ice core cutting, ice chip handling and potential ice melting (and refreezing) during drilling. The analysis and fabrication challenges and testing results are presented in this paper.
KW - Horn antennas
KW - Ice
KW - Piezoelectric devices
KW - Rock drilling
KW - Rocks
KW - Soils
KW - Ultrasonic waves
KW - Underwater drilling
KW - Ice drilling

KW - Planetary sampling
KW - Ultrasonic drilling
KW - Ultrasonic horn
KW - Ultrasonic/Sonic Drill/Corer (USDC)
KW - Ultrasonic devices
KW - Ice drilling
KW - Piezoelectric devices
KW - Planetary sampling
KW - Ultrasonic drilling
KW - Ultrasonic horn
SN - 0277786X (ISSN); 0819462241 (ISBN); 978-081946224-4 (ISBN)
LA - English
J2 - Proc SPIE Int Soc Opt Eng
M3 - Conference paper
DB - Scopus
N1 - Export Date: 23 February 2023; Cited By: 22; Correspondence Address: M. Badescu; Jet Propulsion Laboratory, California Institute of Technology, MS 67-119, Pasadena, CA 91109-8099, 4800 Oak Grove Drive, United States; email: Mircea.Badescu@jpl.nasa.gov; CODEN: PSISD
ER -

TY - CONF
AU - Belz, A.P.
AU - Cutts, J.A.
TI - Planning for planetary protection and contamination control: Challenges beyond mars
PY - 2006
T2 - IEEE Aerospace Conference Proceedings
VL - 2006
C7 - 1655739
UR - https://www.scopus.com/inward/record.uri?eid=2-s2.0-34047105326&partnerID=40&md5=88b961c2eb84e2d1f6ea11f898c0f88e
AD - Jet Propulsion Laboratory, California Institute of Technology, Pasadena, CA 91109-8099, 4800 Oak Grove Drive, United States
AB - In situ analysis for targets beyond Mars brings new challenges in planetary protection, where planetary protection preserves the chemical environment of a target body for future life-detection exploration and, in sample return missions, protects the Earth from potential extraterrestrial contamination. The National Research Council's Decadal Survey of 2003 and the NASA Solar System Exploration Strategic Program roadmap of 2005 calls for missions to bodies of interest for life-detection or prebiotic science, including Europa, Titan, and comets. These targets present challenges because NASA planetary protection policies specify new requirements for missions to Europa, and new guidelines for Titan are anticipated; furthermore, the comet missions have additional significance because they are envisioned to be sample return missions. This document summarizes the technical challenges to planetary protection and contamination control for these targets of interest and outlines some of the considerations, particularly at the system level, in designing an appropriate technology investment strategy for targets beyond Mars. © 2006 IEEE.
KW - Astrobiology

KW - Contamination
KW - Earth (planet)
KW - Moon
KW - NASA
KW - Regulatory compliance
KW - Contamination control
KW - Planetary protection
KW - Prebiotic science
KW - Titan
KW - Planets
SN - 1095323X (ISSN); 0780395468 (ISBN); 978-078039546-6 (ISBN)
LA - English
J2 - IEEE Aerosp. Conf. Proc.
M3 - Conference paper
DB - Scopus
N1 - Export Date: 23 February 2023; Cited By: 0; Correspondence Address: A.P. Belz; Jet Propulsion Laboratory, California Institute of Technology, Pasadena, CA 91109-8099, 4800 Oak Grove Drive, United States; email: Andrea.P.Belz@jpl.nasa.gov
ER -

TY - CONF
AU - Crawford, I.A.
TI - The astrobiological case for renewed robotic and human exploration of the Moon
PY - 2006
T2 - International Journal of Astrobiology
VL - 5
IS - 3
SP - 191
EP - 197
DO - 10.1017/S1473550406002990
UR - https://www.scopus.com/inward/record.uri?eid=2-s2.0-33845527874&doi=10.1017%2fS1473550406002990&partnerID=40&md5=d40ab9592dd83fe072c9d6e25515e28f
AD - School of Earth Sciences, Birkbeck College, London WC1E 7HX, Malet Street, United Kingdom
AB - An ambitious programme of lunar exploration will reveal much of astrobiological interest. Examples include: (i) better characterization of the impact cratering rate in the Earth-Moon system, with implications for understanding the possible 'impact frustration' of the origin of life; (ii) preservation of ancient meteorites blasted off Earth, Mars and Venus, which may preserve evidence of the early surface environments of these planets, as well as constraining models of lithopanspermia; (iii) preservation of samples of the Earth's early atmosphere not otherwise available; (iv) preservation of cometary volatiles and organics in permanently shadowed polar craters, which would help elucidate the importance of these sources in 'seeding' the terrestrial planets with pre-biotic materials; and (v) possible preservation of extraterrestrial artefacts on the lunar surface, which may permit limits to be placed on the prevalence of technological civilizations in the Galaxy. Much of this valuable information is likely to be buried below the present surface (e.g. in palaeoregolith deposits) and will require a

considerable amount of geological fieldwork to retrieve. This would be greatly facilitated by a renewed human presence on the Moon, and may be wholly impractical otherwise. In the longer term, such lunar operations would pave the way for the human exploration of Mars, which may also be expected to yield astrobiological discoveries not otherwise obtainable. © 2006 Cambridge University Press.
KW - astronomy
KW - exobiology
KW - exploration
KW - Mars
KW - Moon
KW - robotics
KW - terrestrial environment
KW - Venus
KW - Astrobiology
KW - Human spacelight
KW - Lunar exploration
SN - 14753006 (ISSN)
LA - English
J2 - Int. J. Astrobiology
M3 - Conference paper
DB - Scopus
N1 - Export Date: 23 February 2023; Cited By: 17; Correspondence Address: I.A. Crawford; School of Earth Sciences, Birkbeck College, London WC1E 7HX, Malet Street, United Kingdom; email: i.crawford@ucl.ac.uk
ER -

TY - JOUR
AU - Mandell, A.M.
AU - Raymond, S.N.
AU - Sigurdsson, S.
TI - Formation of earth-like planets during and after giant planet migration
PY - 2007
T2 - Astrophysical Journal
VL - 660
IS - 1 I
SP - 823
EP - 844
DO - 10.1086/512759
UR - https://www.scopus.com/inward/record.uri?eid=2-s2.0-34248995511&doi=10.1086%2f512759&partnerID=40&md5=e82689bfe8d4875efcb88ef1883a8818
AD - Department of Astronomy and Astrophysics, Pennsylvania State University, University Park, PA 16803, United States
AD - NASA Goddard Space Flight Center, Code 693, Greenbelt, MD 20771, United States
AD - NASA Postdoctoral Program Fellow, Center for Astrophysics and Space Astronomy, University of Colorado, Boulder, CO 80309-0389, United States
AB - Close-in giant planets are thought to have formed in the cold outer regions of planetary systems and migrated inward, passing through the orbital parameter space occupied by the terrestrial

planets in our own solar system. We present dynamical simulations of the effects of a migrating giant planet on a disk of protoplanetary material and the subsequent evolution of the planetary system. We numerically investigate the dynamics of postmigration planetary systems over 200 million years using models with a single migrating giant planet, one migrating and one nonmigrating giant planet, and excluding the effects of a gas disk. Material that is shepherded in front of the migrating giant planet by moving mean motion resonances accretes into "hot Earths," but survival of these bodies is strongly dependent on dynamical damping. Furthermore, a significant amount of material scattered outward by the giant planet survives in highly excited orbits; the orbits of these scattered bodies are then damped by gas drag and dynamical friction over the remaining accretion time. In all simulations Earth-mass planets accrete on approximately 100 Myr timescales, often with orbits in the habitable zone. These planets range in mass and water content, with both quantities increasing with the presence of a gas disk and decreasing with the presence of an outer giant planet. We use scaling arguments and previous results to derive a simple recipe that constrains which giant planet systems are able to form and harbor Earth-like planets in the habitable zone, demonstrating that roughly one-third of the known planetary systems are potentially habitable. © 2007. The American Astronomical Society. All rights reserved.
KW - Astrobiology
KW - Methods: n-body simulations
KW - Planetary systems: formation
PB - Institute of Physics Publishing
SN - 0004637X (ISSN)
LA - English
J2 - Astrophys. J.
M3 - Article
DB - Scopus
N1 - Export Date: 23 February 2023; Cited By: 106; Correspondence Address: A.M. Mandell; Department of Astronomy and Astrophysics, Pennsylvania State University, University Park, PA 16803, United States; email: mandell@astro.psu.edu
ER -

TY - JOUR
AU - Rinaldi, A.
TI - Space life holds its breath. Pressured by scepticism, budget cuts and the need to prove itself, astrobiology is coming to a crossroads
PY - 2007
T2 - EMBO Reports
VL - 8
IS - 5
SP - 436
EP - 440
DO - 10.1038/sj.embor.7400966
UR - https://www.scopus.com/inward/record.uri?eid=2-s2.0-34247851018&doi=10.1038%2fsj.embor.7400966&partnerID=40&md5=a2ba127aa31584e18b3672a0950d8045
KW - Exobiology

KW - Extraterrestrial Environment
KW - Life
KW - Planets
KW - Water
KW - article
KW - astronomy
KW - biology
KW - microbiology
KW - molecular evolution
KW - politics
KW - pressure
KW - priority journal
KW - research
KW - simulation
KW - space
KW - temperature
KW - thermodynamics
SN - 14693178 (ISSN)
C2 - 17471257
LA - English
J2 - EMBO Rep.
M3 - Article
DB - Scopus
N1 - Export Date: 23 February 2023; Cited By: 1; CODEN: ERMEA
ER -

TY - JOUR
AU - Lognonne, P.
AU - Des Marais, D.
AU - Raulin, F.
AU - Fishbaugh, K.
TI - Epilogue: The origins of life in the solar system and future exploration
PY - 2007
T2 - Space Science Reviews
VL - 129
IS - 1-3
SP - 301
EP - 304
DO - 10.1007/s11214-007-9239-6
UR - https://www.scopus.com/inward/record.uri?eid=2-s2.0-34548082266&doi=10.1007%2fs11214-007-9239-6&partnerID=40&md5=d1359d34871cc24de8909e0813cee028
AB - The habitability of terrestrial planets and life developed on other planetary bodies in the Solar System are discussed. Europa is likely to have an internal, liquid water ocean that may be relatively close to the surface. Models of the internal structure of Europa suggest that this ocean is in contact with underlying bedrock, offering interesting possibilities for prebiotic chemistry. Titan is also likely to possess an internal water ocean and the liquid water layer is probably located between two layers of water ice and is not in contact with the bedrock. The extrasolar planets also offer a significant field of investigation for searching for extraterrestrial life and for studying a potentially very wide

variety of planetary objects. The proposed Darwin mission would be able to systematically determine the chemical composition of the atmosphere of the extrasolar planets and to look for the possible presence of atmospheric biomarkers.
KW - Biomarkers
KW - Extraterrestrial atmospheres
KW - Ice
KW - Bedrocks
KW - Extrasolar planets
KW - Terrestrial planets
KW - Solar system
SN - 15729672 (ISSN)
LA - English
J2 - Space Sci Rev
M3 - Article
DB - Scopus
N1 - Export Date: 23 February 2023; Cited By: 2; Correspondence Address: F. Raulin; Creteil Cedex 94010, France; email: raulin@lisa.univ-paris12.fr; CODEN: SPSRA
ER -

TY - JOUR
AU - Delio, I.
TI - Christ and extraterrestrial life
PY - 2007
T2 - Theology and Science
VL - 5
IS - 3
SP - 249
EP - 265
DO - 10.1080/14746700701622008
UR - https://www.scopus.com/inward/record.uri?eid=2-s2.0-43249134231&doi=10.1080%2f14746700701622008&partnerID=40&md5=1cf61eee4a7f194a981836ac42b6ca6d
AD - Washington Theological Union, United States
AB - This paper explores the relationship between Incarnation and extraterrestrial life in view of the question: can extraterrestrials be saved? The Franciscan theology of Bonaventure and Scotus is used to explore “exoChristology” by examining the Incarnation as a theological rather than anthropological event. The primacy of Christ, held by Franciscan theologians, provides an integral relationship between Christ and creation. From this relationship, it is shown that Incarnation takes place wherever there is intelligible life. It is suggested that all possible worlds, created through the Word of God, bear a spiritual potency within them and are open to spiritual transformation. The divine Word is incarnate in every created order through the appropriate form of intelligible life within that order and completes that order through love. Because Incarnation may take on other extraterrestrial life forms, it is suggested that there may be multiple incarnations but only one Christ. © 2007, Taylor & Francis Group, LLC. All rights reserved.
KW - Bonaventure
KW - Creation
KW - ExoChristology

KW - Extraterrestrial
KW - Incarnation
KW - Rahner
KW - Scotus
KW - Trinity
SN - 14746700 (ISSN)
LA - English
J2 - Theology Sci.
M3 - Article
DB - Scopus
N1 - Export Date: 23 February 2023; Cited By: 2
ER -

TY - JOUR
AU - Gao, Y.
AU - Ellery, A.
AU - Sweeting, M.N.
AU - Vincent, J.
TI - Bioinspired drill for planetary sampling: Literature survey, conceptual design, and feasibility study
PY - 2007
T2 - Journal of Spacecraft and Rockets
VL - 44
IS - 3
SP - 703
EP - 709
DO - 10.2514/1.23025
UR - https://www.scopus.com/inward/record.uri?eid=2-s2.0-34547287232&doi=10.2514%2f1.23025&partnerID=40&md5=210c922c4a150f92e2fcc43723c2076d
AD - University of Surrey, Guildford, GU2 7XH, United Kingdom
AD - University of Bath, Bath, BA2 7AY, United Kingdom
AD - Surrey Space Centre, United Kingdom
AD - Centre for Biomimetic and Natural Technologies, United Kingdom
AB - Growing interest in planetary subsurface exploration has prompted an examination of advanced drilling technologies. One of the major limitations of sampling in low gravity environments (such as Mars, asteroids, etc.) using conventional rotary drills is the need for high axial force, which suffers from big overhead mass, buckling problems, and power hungriness. Though drills using percussive motion may operate in low mass and power, the drilling rate is generally slow. Drawing inspiration from nature for a lighter weight and energy efficient solution, we propose a novel drilling method based on the working mechanism of wood wasp ovipositors. The bioinspired drill requires no reactive external force by applying two-valve-reciprocating motion. The proposed biomimetic system indicates enhanced utility that is critical for space missions where premium is placed on mass, volume, and power. Biological systems are similarly constrained making biomimetic technology uniquely suited and advantageous as a model of miniaturized systems. As a result of the European Space Agency project on bionics and space system design, this paper presents a literature survey of planetary drilling, working principle of the bioinspired drilling mechanism, a conceptual design, and feasibility

study of the first prototype within a space system envelope.
KW - Astrobiology
KW - Biological systems
KW - Drilling
KW - Energy efficiency
KW - Gravitational effects
KW - Systems analysis
KW - Axial force
KW - Drilling rates
KW - Planetary drilling
KW - Planetary sampling
KW - Planetary surface analysis
PB - American Institute of Aeronautics and Astronautics Inc.
SN - 00224650 (ISSN)
LA - English
J2 - J Spacecr Rockets
M3 - Article
DB - Scopus
N1 - Export Date: 23 February 2023; Cited By: 34; Correspondence Address: Y. Gao; University of Surrey, Guildford, GU2 7XH, United Kingdom; email: yang.gao@surrey.ac.uk; CODEN: JSCRA
ER -

TY - JOUR
AU - Gómez, F.
AU - Aguilera, A.
AU - Amils, R.
TI - Soluble ferric iron as an effective protective agent against UV radiation: Implications for early life
PY - 2007
T2 - Icarus
VL - 191
IS - 1
SP - 352
EP - 359
DO - 10.1016/j.icarus.2007.04.008
UR - https://www.scopus.com/inward/record.uri?eid=2-s2.0-35348882085&doi=10.1016%2fj.icarus.2007.04.008&partnerID=40&md5=f7f606dfb9fa37aa46813ad6895de8ad
AD - Centro de Astrobiología, CSIC-INTA, Torrejon de Ardoz, 28850 Madrid, Carretera de Torrejon-Ajalvir, Km 4, Spain
AD - Centro de Biología Molecular, Universidad Autónoma de Madrid, Cantoblanco, 28049 Madrid, Spain
AB - Some recent MER Rover Opportunity results on ancient sedimentary rocks from Mars describe sandstones originated from the chemical weathering of olivine basalts by acidic waters [Squyres, S.W., Knoll, A.H., 2005. Earth Planet. Sci. Lett. 240, 1-10]. The absence of protective components in early Mars atmosphere forced any possible primordial life forms to deal with high doses of UV radiation. A similar situation occurred on the primitive Earth during the development of early life in the Archean [Berkner, L.V., Marshall, L.C., 1965. J. Atmos. Sci. 22 (3), 225-261; Kasting, J.F., 1993. Science 259, 920-926]. It is known that some cellular and/or external components can shield organisms from damaging UV radiation

or quench its toxic effects [Olson, J.M., Pierson, B.K., 1986. Photosynth. Res. 9, 251-259; García-Pichel, F., 1998. Origins Life Evol. B 28, 321-347; Cockell, C., Rettberg, P., Horneck, G., Scherer, K., Stokes, M.D., 2003. Polar Biol. 26, 62-69]. The effectiveness of iron minerals for UV protection has also been reported [Phoenix, V.R., Konhauser, K.O., Adams, D.G., Bottrell, S.H., 2001. Geology 29 (9), 823-826], but nothing is known about the effect of iron in solution. Here we demonstrate the protective effect of soluble ferric iron against UV radiation on acidophilic photosynthetic microorganisms. These results offer an interesting alternative means of protection for life on the surface of early Mars and Earth, especially in light of the geochemical conditions in which the sedimentary minerals, jarosite and goethite, recently reported by the MER missions, were formed [Squyres, S.W., Arvidson, R.E., Bell III, J.F., Brückner, J., Cabrol, N.A., Calvin, W., Carr, M.H., Christensen, P.R., Clark, B.C., Crumpler, L., Des Marais, D.J., d'Uston, C., Economou, T., Farmer, J., Farrand, W., Folkner, W., Golombek, M., Gorevan, S., Grant, J.A., Greeley, R., Grotzinger, J., Haskin, L., Herkenhoff, K.E., Hviid, S., Johnson, J., Klingelhöfer, G., Knoll, A.H., Landis, G., Lemmon, M., Li, R., Madsen, M.B., Malin, M.C., McLennan, S.M., McSween, H.Y., Ming, D.W., Moersch, J., Morris, R.V., Parker, T., Rice Jr., J.W., Richter, L., Rieder, R., Sims, M., Smith, M., Smith, P., Soderblom, L.A., Sullivan, R., Wänke, H., Wdowiak, T., Wolff, M., Yen, A., 2004. Science 306, 1698-1703; Klingelhöfer, G., Morris, R.V., Bernhardt, B., Schröder, C., Rodionov, D.S., de Souza Jr., P.A., Yen, A., Gellert, R., Evlanov, E.N., Zubkov, B., Foh, J., Bonnes, U., Kankeleit, E., Gütlich, P., Ming, D.W., Renz, F., Wdowiak, T., Squyres, S.W., Arvidson, R.E., 2004. Science 306, 1740-1745]. © 2007 Elsevier Inc. All rights reserved.
KW - Astrobiology
KW - Mars surface
SN - 10902643 (ISSN)
LA - English
J2 - Icarus
M3 - Article
DB - Scopus
N1 - Export Date: 23 February 2023; Cited By: 24; Correspondence Address: F. Gómez; Centro de Astrobiología, CSIC-INTA, Torrejon de Ardoz, 28850 Madrid, Carretera de Torrejon-Ajalvir, Km 4, Spain; email: gomezgf@inta.es; CODEN: ICRSA
ER -

TY - BOOK
AU - Briggs, D.E.G.
AU - Crowther, P.R.
TI - Palaeobiology II
PY - 2007
T2 - Palaeobiology II
SP - 1
EP - 583
DO - 10.1002/9780470999295
UR - https://www.scopus.com/inward/record.uri?eid=2-s2.0-84956975020&doi=10.1002%2f9780470999295&partnerID=40&md5=3cf0b9

bd6b578c3f261ca565908dab8d
AD - Department of Geology and Geophysics, Yale University, New Haven, 06511, CT, United States
AD - National Museums and Galleries of Northern Ireland, Ulster Museum, Botanic Gardens, Belfast, BT9 5AB, United Kingdom
AB - Palaeobiology: A Synthesis was widely acclaimed both for its content and production quality. Ten years on, Derek Briggs and Peter Crowther have once again brought together over 150 leading authorities from around the world to produce Palaeobiology II. Using the same successful formula, the content is arranged as a series of concise articles, taking a thematic approach to the subject, rather than treating the various fossil groups systematically. This entirely new book, with its diversity of new topics and over 100 new contributors, reflects the exciting developments in the field, including accounts of spectacular newly discovered fossils, and embraces data from other disciplines such as astrobiology, geochemistry and genetics. Palaeobiology II will be an invaluable resource, not only for palaeontologists, but also for students and researchers in other branches of the earth and life sciences. Written by an international team of recognised authorities in the field. Content is concise but informative. Demonstrates how palaeobiological studies are at the heart of a range of scientific themes. © 2001, 2003 by Blackwell Science Ltd. All rights reserved.
PB - Wiley Blackwell
SN - 978-047099929-5 (ISBN); 0632051477 (ISBN); 978-063205149-6 (ISBN)
LA - English
J2 - Palaeobiology II
M3 - Book
DB - Scopus
N1 - Export Date: 23 February 2023; Cited By: 7
ER -

TY - CONF
AU - Thangavelu, M.
AU - Bidwell, J.
AU - Cheng, J.
AU - Ghandchi, S.
AU - Morris, A.
AU - Martin, M.
AU - Mustillo, M.
AU - Rojdev, K.
TI - Return to the moon: Jules Verne project
PY - 2007
T2 - A Collection of Technical Papers - AIAA Space 2007 Conference
VL - 2
SP - 1896
EP - 1910
UR - https://www.scopus.com/inward/record.uri?eid=2-s2.0-40649120526&partnerID=40&md5=255590114e7c5c4b77d19b79c337f306
AD - USC Astronautics Faculty
AD - Astronautics and Space Technology Division(ASTD), Viterbi School Ofengineering, University of Southern California
AB - A circumlunar traverse of a manned rover that would intercept

payload/logistics landers at regular intervals along the route to deploy a variety of advanced observatories all over the lunar globe is proposed. Crew will also conduct geological and astrobiological exploration during this dynamic, extended mission requiring several crew replacements. Critical concepts which appeared in the report are presented here. They include alternative launchers including international options to augment the Ares family, a crew rescue system based on the CEV, and a broadband free-space Earth Moon Laser communication system network. Since heavy landings of payloads like the large pressurized rover capable of circumlunar navigation is part of this vision, a staged descent system for retrobraking employing solids/hybrids and a small cryogenic stage for lander touchdown stage is proposed. A variety of observatories which are autonomously landed are commissioned at intercepts along the circumlunar route of the rover using rover crew and robotic assistants and crew activities also include geological and astrobiological exploration. Crew activities and work load partitions are explored. Experience gained by operating such a rover system architecture on the lunar surface is directly applicable for more ambitious, rapid, global planetary exploration to come.
KW - Astrobiology
KW - Cryogenics
KW - Logistics
KW - Navigation
KW - Project management
KW - Robotics
KW - Crew replacements
KW - Logistics landers
KW - Lunar globe
KW - Lunar missions
SN - 1563479079 (ISBN); 978-156347907-6 (ISBN); 1563479079 (ISBN); 978-156347907-6 (ISBN)
LA - English
J2 - Collect. Tech. Pap. AIAA Space Conf. Expos.
M3 - Conference paper
DB - Scopus
N1 - Export Date: 23 February 2023; Cited By: 0
ER -

TY - JOUR
AU - Janhunen, P.
AU - Kaartokallio, H.
AU - Oksanen, I.
AU - Lehto, K.
AU - Lehto, H.
TI - Biological feedbacks as cause and demise of neoproterozoic icehouse: Astrobiological prospects for faster evolution and importance of cold conditions
PY - 2007
T2 - PLoS ONE
VL - 2
IS - 2
C7 - e214
DO - 10.1371/journal.pone.0000214

UR - https://www.scopus.com/inward/record.uri?eid=2-s2.0-46149085334&doi=10.1371%2fjournal.pone.0000214&partnerID=40&md5=02f387d1e4470de67aad247957985512
AD - Department of Physical Sciences, University of Helsinki, Helsinki, Finland
AD - Finnish Meteorological Institute, Space Research, Helsinki, Finland
AD - Finnish Institute of Marine Research, Helsinki, Finland
AD - Department of Applied Chemistry and Microbiology, University of Helsinki, Helsinki, Finland
AD - Department of Botany, University of Stockholm, Stockholm, Sweden
AD - Nordic Institute for Theoretical Physics (NORDITA), Copenhagen, Denmark
AD - Department of Biology, University of Turku, Turku, Finland
AD - Tuorla Observatory, University of Turku, Turku, Finland
AB - Several severe glaciations occurred during the Neoproterozoic eon, and especially near its end in the Cryogenian period (630-850 Ma). While the glacial periods themselves were probably related to the continental positions being appropriate for glaciation, the general coldness of the Neoproterozoic and Cryogenian as a whole lacks specific explanation. The Cryogenian was immediately followed by the Ediacaran biota and Cambrian Metazoan, thus understanding the climate-biosphere interactions around the Cryogenian period is central to understanding the development of complex multicellular life in general. Here we present a feedback mechanism between growth of eukaryotic algal phytoplankton and climate which explains how the Earth system gradually entered the Cryogenian icehouse from the warm Mesoproterozoic greenhouse. The more abrupt termination of the Cryogenian is explained by the increase in gaseous carbon release caused by the more complex planktonic and benthic foodwebs and enhanced by a diversification of metazoan zooplankton and benthic animals. The increased ecosystem complexity caused a decrease in organic carbon burial rate, breaking the algal-climatic feedback loop of the earlier Neoproterozoic eon. Prior to the Neoproterozoic eon, eukaryotic evolution took place in a slow timescale regulated by interior cooling of the Earth and solar brightening. Evolution could have proceeded faster had these geophysical processes been faster. Thus, complex life could theoretically also be found around stars that are more massive than the Sun and have main sequence life shorter than 10 Ga. We also suggest that snow and glaciers are, in a statistical sense, important markers for conditions that may possibly promote the development of complex life on extrasolar planets. © 2007 Janhunen et al.
KW - Algae
KW - Biodiversity
KW - Biogenesis
KW - Carbon Dioxide
KW - Carbon Isotopes
KW - Cold Climate
KW - Earth (Planet)
KW - Evolution, Planetary
KW - Exobiology
KW - Feedback, Physiological

KW - Greenhouse Effect
KW - Ice Cover
KW - Marine Biology
KW - Oxygen
KW - Photosynthesis
KW - Phytoplankton
KW - Seawater
KW - Solar System
KW - Solubility
KW - algae
KW - Animalia
KW - Eukaryota
KW - Metazoa
KW - carbon
KW - carbon dioxide
KW - oxygen
KW - sea water
KW - article
KW - benthos
KW - climate change
KW - cold climate
KW - Cryogenian
KW - ecosystem
KW - eukaryote evolution
KW - evolution
KW - feedback system
KW - glaciation
KW - global climate
KW - greenhouse
KW - Neoproterozoic
KW - nonhuman
KW - phytoplankton
KW - plankton
KW - zooplankton
KW - alga
KW - astronomy
KW - biodiversity
KW - biogenesis
KW - biology
KW - chemistry
KW - cold climate
KW - greenhouse effect
KW - growth, development and aging
KW - ice cover
KW - metabolism
KW - photosynthesis
KW - physiology
KW - phytoplankton
KW - solubility
SN - 19326203 (ISSN)
C2 - 17299594
LA - English
J2 - PLoS ONE
M3 - Article

DB - Scopus
N1 - Export Date: 23 February 2023; Cited By: 2; Correspondence Address: P. Janhunen; Department of Physical Sciences, University of Helsinki, Helsinki, Finland; email: pekka.janhunen@fmi.fi
ER -

TY - CONF
AU - Yim, M.
AU - Shirmohammadi, B.
AU - Benelli, D.
TI - Amphibious modular robotic astrobiology
PY - 2007
T2 - Proceedings of SPIE - The International Society for Optical Engineering
VL - 6561
C7 - 65611S
DO - 10.1117/12.719944
UR - https://www.scopus.com/inward/record.uri?eid=2-s2.0-36048951380&doi=10.1117%2f12.719944&partnerID=40&md5=9705e60d47f24bd85cb037258d765cf7
AD - University of Pennsylvania, Philadelphia, PA 19104, 3330 Walnut St., United States
AB - This paper presents the design of a robot that can traverse land, water, as well as quicksand-like mud. The robot is low cost and modular allowing the replacement of a variety of arms suitable for many of the tasks associated with astrobiological exploration. An astrobiologist on a field study will spend most of the time walking around and exploring the site looking for areas of interest which will be tested in situ or sampled for testing offsite. For a robot replicating these tasks, it must be able to locomote in that terrain, sense the interesting features (or provide sensing for teleoperation), and do a variety of manipulation tasks once an area of interest is reached. The configurations for this robot include 10's of modules that can achieve astrobiological tasks such as amphibious locomotion, digging, core sampling, probing, liquid sampling and exploration. This paper also presents results from the first experiments of this platform at Lake Tyrrell, a salt lake in Australia.
KW - Core samples
KW - Cost reduction
KW - Intelligent robots
KW - Remote control
KW - Amphibious robots
KW - Liquid sampling
KW - Quicksand-like mud
KW - Robotic astrobiology
KW - Astrobiology
KW - Amphibious robots
KW - Astrobiology
KW - Modular
KW - Reconfigurable
SN - 0277786X (ISSN); 0819466832 (ISBN); 978-081946683-9 (ISBN)
LA - English
J2 - Proc SPIE Int Soc Opt Eng

M3 - Conference paper
DB - Scopus
N1 - Export Date: 23 February 2023; Cited By: 6; Correspondence Address: M. Yim; University of Pennsylvania, Philadelphia, PA 19104, 3330 Walnut St., United States; email: yim@seas.upenn.edu; CODEN: PSISD
ER -

TY - JOUR
AU - Islam, M.R.
AU - Schulze-Makuch, D.
TI - Adaptations to environmental extremes by multicellular organisms
PY - 2007
T2 - International Journal of Astrobiology
VL - 6
IS - 3
SP - 199
EP - 215
DO - 10.1017/S1473550407003783
UR - https://www.scopus.com/inward/record.uri?eid=2-s2.0-38749107028&doi=10.1017%2fS1473550407003783&partnerID=40&md5=6007e6b79093f73c5864ba43b3bb1a7f
AD - School of Earth and Environmental Sciences, Washington State University, Pullman, WA 99164, United States
AB - Microbes, particularly Archaea, are well-known for their superb adaptation to extreme environments. However, amazing adaptations to extreme stresses do not only occur in microbes, but also in many multicellular organisms. Examples include tardigrades and their capability to survive freezing to near absolute zero, the Pompeii worm withstanding temperatures of up to 105°C, the Crucian carp remaining active in anoxic conditions for several months, and resurrection plants, which can survive near-complete desiccation. Here, we review some of the cryptobiotic strategies and adaptations exhibited in multicellular organisms, and point out their relevance to astrobiology. © 2007 Cambridge University Press.
KW - Alvinellidae
KW - Archaea
KW - Carassius
KW - Cyprinidae
KW - Selaginella pilifera
KW - Tardigrada
KW - adaptation
KW - anoxic conditions
KW - desiccation
KW - microorganism
KW - survival
KW - Adaptation
KW - Anhydrobiosis
KW - Anoxic
KW - Cryobiosis
KW - Cryptobiosis
KW - Extreme environment
KW - Extremophile

KW - Hibernation
KW - Multicellular
KW - Thermobiosis
SN - 14753006 (ISSN)
LA - English
J2 - Int. J. Astrobiology
M3 - Review
DB - Scopus
N1 - Export Date: 23 February 2023; Cited By: 10; Correspondence Address: D. Schulze-Makuch; School of Earth and Environmental Sciences, Washington State University, Pullman, WA 99164, United States; email: dirksm@wsu.edu
ER -

TY - JOUR
AU - Huntsberger, T.
AU - Stroupe, A.
AU - Aghazarian, H.
AU - Garrett, M.
AU - Younse, P.
AU - Powell, M.
TI - TRESSA: Teamed robots for exploration and science on steep areas
PY - 2007
T2 - Journal of Field Robotics
VL - 24
IS - 11-12
SP - 1015
EP - 1031
DO - 10.1002/rob.20219
UR - https://www.scopus.com/inward/record.uri?eid=2-s2.0-37349009678&doi=10.1002%2frob.20219&partnerID=40&md5=b3fe2cd2ffa20af72f68fef45ea5935b
AD - Mobility and Robotic Systems Section, Jet Propulsion Laboratory, Pasadena, CA 91109, United States
AB - Long-duration robotic missions on lunar and planetary surfaces (for example, the Mars Exploration Rovers have operated continuously on the Martian surface for close to 3 years) provide the opportunity to acquire scientifically interesting information from a diverse set of surface and subsurface sites and to explore multiple sites in greater detail. Exploring a wide range of terrain types, including plains, cliffs, sand dunes, and lava tubes, requires the development of robotic systems with mobility enhanced beyond that which is currently fielded. These systems include single as wen as teams of robots. TRESSA (Teamed Robots for Exploration and Science on Steep Areas) is a closely coupled three-robot team developed at the Jet Propulsion Laboratory (JPL) that previously demonstrated the ability to drive on soil-covered slopes up to 70 deg. In this paper, we present results from field demonstrations of the TRESSA system in even more challenging terrain: rough rocky slopes of up to 85 deg. In addition, the integration of a robotic arm and instrument suite has allowed TRESSA to demonstrate semi-autonomous science investigation of the cliffs and science sample collection. TRESSA successfully traversed cliffs and collected samples at three Mars

analog sites in Svalbard, Norway as part of a recent geological and astrobiological field investigation called AMASE: Arctic Mars Analog Svalbard Expedition under the NASA ASTEP (Astrobiology Science and Technology for Exploring Planets) program. © 2007 Wiley Periodicals, Inc.
KW - Astrobiology
KW - Data acquisition
KW - Lunar missions
KW - NASA
KW - Robotic arms
KW - Jet Propulsion Laboratory
KW - Steep areas
KW - Martian surface analysis
SN - 15564967 (ISSN)
LA - English
J2 - J. Field. Rob.
M3 - Article
DB - Scopus
N1 - Export Date: 23 February 2023; Cited By: 34; Correspondence Address: T. Huntsberger; Mobility and Robotic Systems Section, Jet Propulsion Laboratory, Pasadena, CA 91109, United States; email: Terry.Huntsberger@jpl.nasa.gov
ER -

TY - CONF
AU - Raulin, F.
AU - Nguyen, M.-J.
AU - Coll, P.
TI - Titan: An astrobiological laboratory in the solar system
PY - 2007
T2 - Proceedings of SPIE - The International Society for Optical Engineering
VL - 6694
C7 - 66940L
DO - 10.1117/12.732883
UR - https://www.scopus.com/inward/record.uri?eid=2-s2.0-42149192629&doi=10.1117%2f12.732883&partnerID=40&md5=4f01bdf6926f8ac513fa0b2479c1715a
AD - LISA, CNRS, Universités Paris 7 and Paris 12, F-94000 Créteil, 61 Avenue Général de Gaulle, France
AB - After only three years of close observation from the Cassini-Huygens mission, Titan appears more and more as one of the key planetary bodies in the solar system for astrobiological studies. Titans does not look any more like a frozen primitive Earth, but like an evolving planet, geologically active, with cryovolcanism, eolian erosion, clouds and precipitations, and a methane cycle very similar to the water cycle on Earth. The new data also show a very complex organic chemistry in the highest atmospheric zones of the satellite, with the formation in the ionosphere of high molecular weight organics feeding the lower zones, down to the surface. In spite of the low surface temperature, these organics are probably evolving once in contact with water ice and form organic molecules of biological interest. This may explain the reflectance spectrum of Titan' surface observed by the DIRS instrument on Huygens. Thus,

contrary to what was expected, the organic chemistry on Titan seems mainly concentrated in the ionosphere, in the aerosols and on the surface. These astrobiological aspects of Titan are presented and discussed on the basis of the already available Cassini-Huygens data, as well as the needed post Cassini exploration.
KW - Amino acids
KW - Earth (planet)
KW - Molecular weight
KW - Planets
KW - Satellites
KW - Cassini-Huygens mission
KW - Prebiotic chemistry
KW - Tholins
KW - Titan
KW - Astrobiology
KW - Amino acids
KW - Astrobiology
KW - Cassini-Huygens
KW - Prebiotic chemistry
KW - Primitive Earth
KW - Tholins
KW - Titan
SN - 0277786X (ISSN); 978-081946842-0 (ISBN)
LA - English
J2 - Proc SPIE Int Soc Opt Eng
M3 - Conference paper
DB - Scopus
N1 - Export Date: 23 February 2023; Cited By: 9; Correspondence Address: F. Raulin; LISA, CNRS, Universités Paris 7 and Paris 12, F-94000 Créteil, 61 Avenue Général de Gaulle, France; email: raulin@lisa.univ-paris12.fr; CODEN: PSISD
ER -

TY - CONF
AU - Schilizzi, R.T.
TI - The square kilometre array
PY - 2007
T2 - Proceedings of Science
VL - 52
UR - https://www.scopus.com/inward/record.uri?eid=2-s2.0-85055467466&partnerID=40&md5=2935f55c76122a69e4353d366944ea3f
AD - International SKA Project Office, ASTRON, P.O. Box 2, Dwingeloo, 7990AA, Netherlands
AB - The SKA will have a collecting area of up to one million square metres spread over at least 3000 km, providing a sensitivity 50 times higher than the Expanded VLA, and an instantaneous field of view (FOV) of at least several tens of square degrees and possibly 250 square degrees. The SKA science impact will be widely felt in astro-particle physics and cosmology, fundamental physics, galactic and extragalactic astronomy, solar system science and astrobiology. I will outline the main features of the SKA, paying attention to the design activities around the world, and outline plans for the final design and phased implementation of the telescope. Other papers in these Proceedings go into greater detail on SKA science. Copyright ©

owned by the author(s) under the terms of the Creative Commons Attribution-NonCommercial-ShareAlike Licence.
KW - Astrobiology
KW - Astrophysics
KW - Astroparticle physics
KW - Design activity
KW - Extragalactic astronomy
KW - Field of views
KW - Fundamental physics
KW - Science impact
KW - Planets
PB - Sissa Medialab Srl
SN - 18248039 (ISSN)
LA - English
J2 - Proc. Sci.
M3 - Conference paper
DB - Scopus
N1 - Export Date: 23 February 2023; Cited By: 0; Correspondence Address: R.T. Schilizzi; International SKA Project Office, ASTRON, Dwingeloo, P.O. Box 2, 7990AA, Netherlands; email: richard.schilizzi@skatelescope.org
ER -

TY - JOUR
AU - Edwards, H.G.M.
AU - Vandenabeele, P.
AU - Jorge-Villar, S.E.
AU - Carter, E.A.
AU - Perez, F.R.
AU - Hargreaves, M.D.
TI - The Rio Tinto Mars Analogue site: An extremophilic Raman spectroscopic study
PY - 2007
T2 - Spectrochimica Acta - Part A: Molecular and Biomolecular Spectroscopy
VL - 68
IS - 4
SP - 1133
EP - 1137
DO - 10.1016/j.saa.2006.12.080
UR - https://www.scopus.com/inward/record.uri?eid=2-s2.0-36048982884&doi=10.1016%2fj.saa.2006.12.080&partnerID=40&md5=952401845fd3b36bf608be5035c008b0
AD - Centre for Astrobiology and Extremophile Research, School of Life Sciences, University of Bradford, Bradford, BD7 1DP, United Kingdom
AD - Ghent University, Department of Analytical Chemistry, B-9000 Ghent, Proeftuinstraat 86, Belgium
AD - Area Geodinamica Interna, Facultad de Humanidades y Educacion, Universidad de Burgos, 09001 Burgos, Calle Villadiego s/n, Spain
AD - Vibrational Spectroscopy Facility, School of Chemistry, The University, Sydney, NSW 2006, Australia
AD - Cristografia y Mineralogia/Centro de Astrobiologia, Facultad de Ciencias, Universidad de Valladolid, 47011 Valladolid, Prado de

la Magdalena s/n, Spain
AB - The Rio Tinto site is recognised as a terrestrial Mars analogue because of the presence of jarosite and related sulfates which have recently been identified by the NASA Mars Exploration Rover "Opportunity" in the El Capitan region of Meridiani Planum on Mars. It has long been known that acidophilic microbial action is responsible for the deep blood-red colour of the water in Rio Tinto, where the pH varies from about 1.5 to 3.0 and the water is rich in iron and sulfur. Following recent Raman spectroscopic characterisation of the mineral phases of the Rio Tinto system, we report here a study of the biological components found in several specimens of deposited minerals and near the waterside that were collected during a GeoRaman VI Conference organized field trip in 2006. Key biosignatures were found for carotenoids, scytonemin and mycosporine-like amino acids, which are indicative of the biological colonisation of exposed mineral substrates; information from this study will be useful for targeting Martian sites using a miniaturized Raman instrument where the biosignatures of relict or extant life could remain in the geological record. © 2006 Elsevier B.V. All rights reserved.
KW - Exobiology
KW - Extraterrestrial Environment
KW - Mars
KW - Minerals
KW - Space Simulation
KW - Spain
KW - Spectrum Analysis, Raman
KW - Amino acids
KW - Astrobiology
KW - Biomarkers
KW - pH effects
KW - Spectrum analysis
KW - mineral
KW - article
KW - astronomy
KW - biology
KW - chemistry
KW - microclimate
KW - Raman spectrometry
KW - space flight
KW - Spain
KW - Mars analogue
KW - Rio Tinto Mars Analogue
KW - Raman spectroscopy
KW - Acidotrophic
KW - Astrobiology
KW - Mars analogue
KW - Raman spectroscopy
KW - Rio Tinto
KW - Spectral biomarkers
SN - 13861425 (ISSN)
C2 - 17600759
LA - English
J2 - Spectrochim. Acta Part A Mol. Biomol. Spectrosc.

M3 - Article
DB - Scopus
N1 - Export Date: 23 February 2023; Cited By: 51; Correspondence Address: H.G.M. Edwards; Centre for Astrobiology and Extremophile Research, School of Life Sciences, University of Bradford, Bradford, BD7 1DP, United Kingdom; email: h.g.m.edwards@bradford.ac.uk; CODEN: SAMCA
ER -

TY - BOOK
AU - Agutter, P.S.
AU - Wheatley, D.N.
TI - About life: Concepts in modern biology
PY - 2007
T2 - About Life: Concepts in Modern Biology
SP - 1
EP - 244
DO - 10.1007/978-1-4020-5418-1
UR - https://www.scopus.com/inward/record.uri?eid=2-s2.0-84920164996&doi=10.1007%2f978-1-4020-5418-1&partnerID=40&md5=1733b43183cf6b9df0d5f6b7a059880d
AD - Theoretical and Cell Biology Consultancy, Glossop, Derbyshire, United Kingdom
AD - BioMedES, Inverurie, Aberdeenshire, United Kingdom
AB - This book uses modern biological knowledge to tackle the question: "What distinguishes living organisms from the non-living world?" In the first few chapters, the authors draw on recent advances in cell and molecular biology to develop an account of the "living state" that applies to all organisms, but only to organisms. Subsequent chapters use this account to explore questions about evolution, the origin of life and the possibility of extraterrestrial life. Towards the end of the book the authors consider human evolution, intelligence and the extent to which our species can be regarded as biologically unique. About Life is written as far as possible in non-technical language; all scientific terms are explained straightforwardly when they are introduced. It is aimed at the general, non-specialist reader, but the novel approach that it takes to general issues in biology will also interest students of the life sciences. © 2007 Springer. All Rights Reserved.
PB - Springer Netherlands
SN - 978-140205418-1 (ISBN); 1402054173 (ISBN); 978-140205417-4 (ISBN)
LA - English
J2 - About Life: Concepts in Mod. Biol.
M3 - Book
DB - Scopus
N1 - Export Date: 23 February 2023; Cited By: 10
ER -

TY - CONF
AU - Wickramasinghe, J.T.
AU - Wickramasinghe, N.C.
AU - Napier, W.M.

TI - Liquid water in comets: Implications for astrobiology
PY - 2007
T2 - Proceedings of SPIE - The International Society for Optical Engineering
VL - 6694
C7 - 66940A
DO - 10.1117/12.768445
UR - https://www.scopus.com/inward/record.uri?eid=2-s2.0-42149091158&doi=10.1117%2f12.768445&partnerID=40&md5=63bdecd2678f113b22d4a0cda9074e27
AD - Cardiff Centre for Astrobiology, Cardiff University, Cardiff CF10 3DY, 2 North Road, United Kingdom
AB - We show that radiogenic heating in primordial comets of radii in excess of ~10km could produce liquid water cores persisting for hundreds of thousands to millions of years. Supposing comets were seeded with even the smallest numbers of viable microbes at the time of their formation from pre-solar material, there is ample time for exponential amplification within the liquid interiors before refreezing occurs. Freeze-dried biological material is returned to interplanetary and interstellar space during cometary activity as the outer layers of comets are stripped away via sublimation. Modelling of the post-impact 8-12μm spectra of Tempel 1 gives a strong indication of mixtures of clays and organics in comparable quantity, clays in turn providing evidence of a liquid water history of the comet. The totality of comets in a galaxy or a cluster of galaxies, seems to provide a far more promising setting for an origin of life than any setting thus far proposed in relation to the primitive Earth. Once life has originated in a comet mechanisms of interstellar panspermia that have recently been identified will disperse throughout the Galaxy within a few billion years.
KW - Biological materials
KW - Clay
KW - Earth (planet)
KW - Galaxies
KW - Interstellar space
KW - Origin of life
KW - Radiogenic heating
KW - Refreezing
KW - Astrobiology
KW - Clay
KW - Comets
KW - Liquid water
KW - Organics
KW - Origin of life
KW - Panspermia
SN - 0277786X (ISSN); 978-081946842-0 (ISBN)
LA - English
J2 - Proc SPIE Int Soc Opt Eng
M3 - Conference paper
DB - Scopus
N1 - Export Date: 23 February 2023; Cited By: 1; CODEN: PSISD
ER -

TY - JOUR

AU - Dartnell, L.R.
AU - Desorgher, L.
AU - Ward, J.M.
AU - Coates, A.J.
TI - Modelling the surface and subsurface Martian radiation environment: Implications for astrobiology
PY - 2007
T2 - Geophysical Research Letters
VL - 34
IS - 2
C7 - L02207
DO - 10.1029/2006GL027494
UR - https://www.scopus.com/inward/record.uri?eid=2-s2.0-34547582480&doi=10.1029%2f2006GL027494&partnerID=40&md5=6eda458f0c49aecd09f3674620863158
AD - Centre for Mathematics and Physics in the Life Sciences and Experimental Biology (CoMPLEX), University College London, London NW1 2HE, Wolfson House, 4 Stephenson Way, United Kingdom
AD - Physikalisches Institut, University of Bern, CH-3012 Bern, Sidlerstrasse 5, Switzerland
AD - Department of Biochemistry and Molecular Biology, University College London, London WC1E 6BT, Gower Street, United Kingdom
AD - Mullard Space Science Laboratory, University College London, Dorking RH5 6NT, Holmbury St. Mary, United Kingdom
AB - The damaging effect of ionising radiation on cellular structure is one of the prime limiting factors on the survival of life in potential astrobiological habitats. Here we model the propagation of solar energetic protons and galactic cosmic ray particles through the Martian atmosphere and three different surface scenarios: dry regolith, water ice, and regolith with layered permafrost. Particle energy spectra and absorbed radiation dose are determined for the surface and at regular depths underground, allowing the calculation of microbial survival times. Bacteria or spores held dormant by freezing conditions cannot metabolise and become inactivated by accumulating radiation damage. We find that at 2 m depth, the reach of the ExoMars drill, a population of radioresistant cells would need to have reanimated within the last 450,000 years to still be viable. Recovery of viable cells cryopreserved within the putative Cerberus pack-ice. requires a drill depth of at least, 7.5 m. Copyright 2007 by the American Geophysical Union.
KW - Astrobiology
KW - Cells
KW - Cosmic rays
KW - Dosimetry
KW - Particle spectrometers
KW - Protons
KW - Solar energy
KW - cosmic ray
KW - Martian atmosphere
KW - modeling
KW - permafrost
KW - radiation damage
KW - Cellular structure

KW - Particle energy spectra
KW - Radioresistant cells
KW - Martian surface analysis
SN - 00948276 (ISSN)
LA - English
J2 - Geophys. Res. Lett.
M3 - Article
DB - Scopus
N1 - Export Date: 23 February 2023; Cited By: 196; Correspondence Address: L.R. Dartnell; Centre for Mathematics and Physics in the Life Sciences and Experimental Biology (CoMPLEX), University College London, London NW1 2HE, Wolfson House, 4 Stephenson Way, United Kingdom; email: l.dartnell@ucl.ac.uk; CODEN: GPRLA
ER -

TY - JOUR
AU - Shukitt-Hale, B.
AU - Casadesus, G.
AU - Carey, A.N.
AU - Rabin, B.M.
AU - Joseph, J.A.
TI - Exposure to ^{56}Fe irradiation accelerates normal brain aging and produces deficits in spatial learning and memory
PY - 2007
T2 - Advances in Space Research
VL - 39
IS - 6
SP - 1087
EP - 1092
DO - 10.1016/j.asr.2006.11.005
UR - https://www.scopus.com/inward/record.uri?eid=2-s2.0-34249310995&doi=10.1016%2fj.asr.2006.11.005&partnerID=40&md5=06a6273ca08a6ea947ca31e833b97b7c
AD - USDA-ARS, Human Nutrition Research Center on Aging, Tufts University, Boston, MA 02111, 711 Washington Street, United States
AD - Institute of Pathology, Case Western Reserve University, Cleveland, OH 44106, United States
AD - Department of Psychology, UMBC, Baltimore, MD 21250, 1000 Hilltop Circle, United States
AB - Previous studies have shown that radiation exposure, particularly to particles of high energy and charge (HZE particles) such as ^{56}Fe, produces deficits in spatial learning and memory. These adverse behavioral effects are similar to those seen in aged animals. It is possible that these shared effects may be produced by the same mechanism. For example, an increased release of reactive oxygen species, and the subsequent oxidative stress and inflammatory damage caused to the central nervous system, is likely responsible for the deficits seen in aging and following irradiation. Therefore, dietary antioxidants, such as those found in fruits and vegetables, could be used as countermeasures to prevent the behavioral changes seen in these conditions. Both aged and irradiated rats display cognitive impairment in tests of spatial learning and memory such as the

Morris water maze and the radial arm maze. These rats have decrements in the ability to build spatial representations of the environment, and they utilize non-spatial strategies to solve tasks. Furthermore, they show a lack of spatial preference, due to a decline in the ability to process or retain place (position of a goal with reference to a "map" provided by the configuration of numerous cues in the environment) information. These declines in spatial memory occur in measures dependent on both reference and working memory, and in the flexibility to reset mental images. These results show that irradiation with 56
Fe high-energy particles produces age-like decrements in cognitive behavior that may impair the ability of astronauts, particularly middle-aged ones, to perform critical tasks during long-term space travel beyond the magnetosphere.
KW - Astrobiology
KW - Brain
KW - Irradiation
KW - Neurology
KW - Oxygen
KW - Inflammation
KW - Morris water maze
KW - Reactive oxygen species
KW - Spatial memory
KW - Iron
KW - Antioxidants
KW - Cognitive
KW - Countermeasures
KW - Inflammation
KW - Morris water maze
KW - Oxidative stress
PB - Elsevier Ltd
SN - 02731177 (ISSN)
LA - English
J2 - Adv. Space Res.
M3 - Article
DB - Scopus
N1 - Export Date: 23 February 2023; Cited By: 21; Correspondence Address: B. Shukitt-Hale; USDA-ARS, Human Nutrition Research Center on Aging, Tufts University, Boston, MA 02111, 711 Washington Street, United States; email: barbara.hale@tufts.edu; CODEN: ASRSD
ER -

TY - JOUR
AU - Selsis, F.
AU - Kasting, F.
AU - Levrard, B.
AU - Paillet, J.
AU - Ribas, I.
AU - Delfosse, X.
TI - Habitable planets around the star Gliese 581?
PY - 2007
T2 - Astronomy and Astrophysics
VL - 476
IS - 3

SP - 1373
EP - 1387
DO - 10.1051/0004-6361:20078091
UR - https://www.scopus.com/inward/record.uri?eid=2-s2.0-38049187892&doi=10.1051%2f0004-6361%3a20078091&partnerID=40&md5=2190e2faeb3cbd5bc88a6d38819ef68c
AD - CRAL: Centre de Recherche Astrophysique de Lyon, 69007 Lyon, 46 allée d'Italie, France
AD - LAB: Laboratoire D'Astrophysique de Bordeaux (CNRS; Université Bordeaux I), 33270 Floirac, BP 89, France
AD - Dept. of Geosciences, Pennsylvania State University, University Park, PA 16802, United States
AD - IMCCE: Institut de Mécanique Céleste et de Calcul des Ephémérides, 75014 Paris, 77 avenue Denfert-Rochereau, France
AD - ESA/ESTEC SCI-SA, 2200AG Noordwijk, Keplerlaan 1, Netherlands
AD - Institut de Ciéncies de L'Espai (CSIC-IEEC), Campus UAB, 08193 Bellaterra, Spain
AD - LAOG: Laboratoire d'AstrOphysique de Grenoble (CNRS; Université J. Fourier - Grenoble I), 38041 Grenoble Cedex, BP 53X, France
AB - Context.Thanks to remarkable progress, radial velocity surveys are now able to detect terrestrial planets at habitable distance from low-mass stars. Recently, two planets with minimum masses below 10 have been reported in a triple system around the M-type star Gliese 581. These planets are found at orbital distances comparable to the location of the boundaries of the habitable zone of their star. Aims.In this study, we assess the habitability of planets Gl 581c and Gl 581d (assuming that their actual masses are close to their minimum masses) by estimating the locations of the habitable-zone boundaries of the star and discussing the uncertainties affecting their determination. An additional purpose of this paper is to provide simplified formulae for estimating the edges of the habitable zone. These may be used to evaluate the astrobiological potential of terrestrial exoplanets that will hopefully be discovered in the near future.Methods.Using results from radiative-convective atmospheric models and constraints from the evolution of Venus and Mars, we derive theoretical and empirical habitable distances for stars of F, G, K, and M spectral types. Results.Planets Gl 581c and Gl 581d are near to, but outside, what can be considered as the conservative habitable zone. Planet "c" receives 30% more energy from its star than Venus from the Sun, with an increased radiative forcing caused by the spectral energy distribution of Gl 581. This planet is thus unlikely to host liquid water, although its habitability cannot be positively ruled out by theoretical models due to uncertainties affecting cloud properties and cloud cover. Highly reflective clouds covering at least 75% of the day side of the planet could indeed prevent the water reservoir from being entirely vaporized. Irradiation conditions of planet "d" are comparable to those of early Mars, which is known to have hosted surface liquid water. Thanks to the greenhouse effect of CO-ice clouds, also invoked to explain the early Martian climate, planet "d" might be a better candidate for the first exoplanet known to be potentially habitable. A mixture of several greenhouse gases could also maintain habitable conditions on this planet, although the

geochemical processes that could stabilize such a super-greenhouse atmosphere are still unknown. . © 2007 ESO.
KW - Astrobiology
KW - Greenhouse effect
KW - Greenhouse gases
KW - Mathematical models
KW - Stars
KW - Velocity measurement
KW - Atmospheric effects
KW - Atmospheric models
KW - Planetary systems
KW - Spectral energy distribution
KW - Terrestrial planets
KW - Planets
KW - Astrobiology
KW - Atmospheric effects
KW - Stars: planetary systems
PB - EDP Sciences
SN - 00046361 (ISSN)
LA - English
J2 - Astron. Astrophys.
M3 - Article
DB - Scopus
N1 - Export Date: 23 February 2023; Cited By: 375; Correspondence Address: F. Selsis; CRAL: Centre de Recherche Astrophysique de Lyon, 69007 Lyon, 46 allée d'Italie, France; email: franck.selsis@ens-lyon.fr; CODEN: AAEJA
ER -

TY - JOUR
AU - Sheehan, D.P.
TI - Thermosynthetic life
PY - 2007
T2 - Foundations of Physics
VL - 37
IS - 12
SP - 1774
EP - 1797
DO - 10.1007/s10701-007-9168-y
UR - https://www.scopus.com/inward/record.uri?eid=2-s2.0-36249018889&doi=10.1007%2fs10701-007-9168-y&partnerID=40&md5=3f15c213de6b39337c50ead08deeb70c
AD - Department of Physics, University of San Diego, San Diego, CA 92110, United States
AB - Two categories of life are currently recognized-chemosynthetic and photosynthetic-indicating their principal free energy resource as either chemicals or electromagnetic radiation. Building on recent developments in thermodynamics, we posit a third category of life-thermosynthetic life (TL)-which relies on environmental heat rather than traditional free energy sources. Since thermal energy is more abundant than chemicals or light in many settings, thermosynthesis offers compelling evolutionary possibilities for new life forms. Based on variants of standard cellular machinery, a physical model is proposed for the conversion of thermal energy into biochemical

work. Conditions favorable to thermosynthetic life and prospects for its discovery are assessed. Terrestrially, deep-subsurface unicellular anaerobic superthermophiles are deduced to be likely TL candidates. © 2007 Springer Science+Business Media, LLC.
KW - Astrobiology
KW - Entropy
KW - Exobiology
KW - Extremophiles
KW - Second law of thermodynamics
KW - Thermophiles
PB - Springer Netherlands
SN - 00159018 (ISSN)
LA - English
J2 - Found. Phys.
M3 - Article
DB - Scopus
N1 - Export Date: 23 February 2023; Cited By: 3; Correspondence Address: D.P. Sheehan; Department of Physics, University of San Diego, San Diego, CA 92110, United States; email: dsheehan@sandiego.edu
ER -

TY - JOUR
AU - Ruiz, J.
AU - Montoya, L.
AU - López, V.
AU - Amils, R.
TI - Thermal diapirism and the habitability of the icy shell of Europa
PY - 2007
T2 - Origins of Life and Evolution of the Biosphere
VL - 37
IS - 3
SP - 287
EP - 295
DO - 10.1007/s11084-007-9068-3
UR - https://www.scopus.com/inward/record.uri?eid=2-s2.0-34248386077&doi=10.1007%2fs11084-007-9068-3&partnerID=40&md5=31e9defcf2908a48ec27291935c12587
AD - Instituto de Astrofísica de Andalucía, CSIC, 18008 Granada, Camino Bajo de Huétor 50, Spain
AD - Centro de Biología Molecular, CSIC-Universidad Autónoma de Madrid, 28049 Cantoblanco, Madrid, Spain
AD - Seminario de Ciencias Planetarias, Facultad de Ciencias Geológicas, Universidad Complutense de Madrid, 28040 Madrid, Spain
AD - Centro de Astrobiología, INTA, Madrid, Spain
AB - Europa's chaos and lenticulae features may have originated by thermal diapirs related to convective plumes. Warm ice plumes could be habitable, since their temperature is close to the ice melting temperature. Moreover, thermal plumes intruding into the lower stagnant lid warm several kilometers of country ice above 230 K for periods of 105 years, and hundreds of meters above 240 K for periods of 104 years. Diapir coalescence generating chaos areas should provide a large zone with temperature above ~240 K for thousands of

years. A temperature above ~230 K is potentially interesting for astrobiology, since it corresponds to the lowest temperature at which microbial metabolic activity in Antarctic ice has been reported. So, the warming by thermal plumes could cause an aureole of biological activation/reactivation in the country ice. Adaptation of life to either high salinity or low temperature is similar: it requires the synthesis of compatible solutes, like trehalose or glycerol, which are efficient cryoprotectants. We therefore propose that the future astrobiological exploration of Europa should include the search for compatible solutes in chaos and lenticulae features.
© 2007 Springer Science+Business Media, B.V.
KW - Adaptation, Biological
KW - Biogenesis
KW - Convection
KW - Exobiology
KW - Extraterrestrial Environment
KW - Jupiter
KW - Temperature
KW - bioactivity
KW - diapirism
KW - Europa
KW - exobiology
KW - Jupiter
KW - Moon
KW - plume
KW - warming
KW - adaptation
KW - astronomy
KW - biogenesis
KW - biology
KW - methodology
KW - microclimate
KW - physiology
KW - review
KW - temperature
KW - thermodynamics
KW - Astrobiology
KW - Compatible solutes
KW - Europa
KW - Habitability
KW - Thermal diapirism
SN - 01696149 (ISSN)
C2 - 17361321
LA - English
J2 - Orig. Life Evol. Biosph.
M3 - Review
DB - Scopus
N1 - Export Date: 23 February 2023; Cited By: 14; Correspondence Address: J. Ruiz; Instituto de Astrofísica de Andalucía, CSIC, 18008 Granada, Camino Bajo de Huétor 50, Spain; email: ruiz@iaa.es; CODEN: OLEBE
ER -

TY - CONF

AU - Bar-Cohen, Y.
AU - Sherrit, S.
AU - Bao, X.
AU - Badescu, M.
AU - Aldrich, J.
AU - Chang, Z.
TI - Subsurface sampler and sensors platform using the Ultrasonic/Sonic Driller/Corer (USDC)
PY - 2007
T2 - Proceedings of SPIE - The International Society for Optical Engineering
VL - 6529 PART 1
C7 - 65290E
DO - 10.1117/12.715048
UR - https://www.scopus.com/inward/record.uri?eid=2-s2.0-35548969818&doi=10.1117%2f12.715048&partnerID=40&md5=1f0ac23e744a5c37e20a463e2eb206a8
AD - Jet Propulsion Laboratory (JPL), Caltech., Pasadena, CA, United States
AB - The search for existing or past life in the Universe is one of the most important objectives of NASA's mission. For this purpose, effective instruments that can sample and conduct in-situ astrobiology analysis are being developed. In support of this objective, a series of novel mechanisms that are driven by an Ultrasonic/Sonic actuator have been developed to probe and sample rocks, ice and soil. This mechanism is driven by an ultrasonic piezoelectric actuator that impacts a bit at sonic frequencies through the use of an intermediate free-mass. Ultrasonic/Sonic Driller/Corer (USDC) devices were made that can produce both core and powdered cuttings, operate as a sounder to emit elastic waves and serve as a platform for sensors. For planetary exploration, this mechanism has the important advantage of requiring low axial force, virtually no torque, and can be duty cycled for operation at low average power. The advantage of requiring low axial load allows overcoming a major limitation of planetary sampling in low gravity environments or when operating from lightweight robots and rovers. The ability to operate at duty cycling with low average power produces a minimum sample temperature rise allowing for control of the sample integrity and preventing damage to potential biological markers in the acquired sample. The development of the USDC is being pursued on various fronts ranging from analytical modeling to mechanisms improvements while considering a wide range of potential applications. While developing the analytical capability to predict and optimize its performance, efforts are made to enhance its capability to drill at higher power and high speed. Taking advantage of the fact that the bit does not require rotation, sensors (e.g., thermocouple and fiberoptics) were integrated into the bit to examine the borehole during drilling. The sounding effect of the drill was used to emit elastic waves in order to evaluate the surface characteristics of rocks. Since the USDC is driven by piezoelectric actuation mechanism it can designed to operate at extreme temperature environments from very cold as on Titan and Europa to very hot as on Venus. In this paper, a review of the latest development and applications of the USDC will be given.

KW - Astrobiology
KW - Drilling
KW - Ice
KW - NASA
KW - Piezoelectric actuators
KW - Rocks
KW - Sampling
KW - Soils
KW - Space flight
KW - Piezoelectric actuation
KW - Sonic Drillers
KW - Subsurface exploration
KW - Subsurface samplers
KW - Ultrasonic sensors
KW - Drilling
KW - Gopher
KW - Sampling
KW - Subsurface exploration
KW - Ultrasonic/Sonic Driller/Corer (USDC)
SN - 0277786X (ISSN); 0819466506 (ISBN); 978-081946650-1 (ISBN)
LA - English
J2 - Proc SPIE Int Soc Opt Eng
M3 - Conference paper
DB - Scopus
N1 - Export Date: 23 February 2023; Cited By: 21; Correspondence Address: Y. Bar-Cohen; Jet Propulsion Laboratory (JPL), Caltech., Pasadena, CA, United States; email: yosi@jpl.nasa.gov; CODEN: PSISD
ER -

TY - CHAP
AU - Dohm, J.M.
AU - Baker, V.R.
AU - Maruyama, S.
AU - Anderson, R.C.
TI - Traits and evolution of the tharsis superplume, Mars
PY - 2007
T2 - Superplumes: Beyond Plate Tectonics
SP - 523
EP - 536
DO - 10.1007/978-1-4020-5750-2_17
UR - https://www.scopus.com/inward/record.uri?eid=2-s2.0-67349176486&doi=10.1007%2f978-1-4020-5750-2_17&partnerID=40&md5=ede12398d8c0cf934b3001eaf5a54da7
AD - Department of Hydrology and Water Resources (and the Lunar and Planetary Laboratory), University of Arizona, Tucson, AZ 85721-0011, United States
AD - Department of Earth and Planetary Sciences, Tokyo Institute of Technology, Meguro-ku 152-8551, Japan
AD - Jet Propulsion Laboratory, California Institute of Technology, Pasadena, CA 91109, United States
AB - When compared to the previous explanations that attempt to explain the long-lived internal heat engine of Tharsis, Mars (e.g., >3 Ga), the Tharsis Superplume hypothesis provides a cohesive explanation for the evolution of the magmatic complex and forms a

sound basis for further productive inquiry. Contributors to its formation and long-term, pulsating evolution may include: (1) extremely ancient plate tectonism (Baker et al., this volume), (2) an extremely ancient giant impact in the Arabia Terra region, which is located nearly antipodal to the superplume (Dohm et al., 2004), and (3) later smaller impacts (e.g., Hellas and Isidis) when compared to the putative Arabia impact with a primary basin estimated to be at least 3000 km in diameter. The extensive geological and paleohydrological records of the Tharsis Superplume presented here, which includes extremely long-lived magma and liquid and frozen water interactions in the subsurface and the surface, have tremendous implications concerning future missions to Mars that will unfold the potential astrobiological information that awaits discovery. © 2007 Springer.
PB - Springer Netherlands
SN - 978-140205749-6 (ISBN)
LA - English
J2 - Superplumes: Beyond Plate Tectonics
M3 - Book chapter
DB - Scopus
N1 - Export Date: 23 February 2023; Cited By: 40
ER -

TY - JOUR
AU - Dartnell, L.R.
AU - Desorgher, L.
AU - Ward, J.M.
AU - Coates, A.J.
TI - Martian sub-surface ionising radiation: Biosignatures and geology
PY - 2007
T2 - Biogeosciences
VL - 4
IS - 4
SP - 545
EP - 558
DO - 10.5194/bg-4-545-2007
UR - https://www.scopus.com/inward/record.uri?eid=2-s2.0-34547561753&doi=10.5194%2fbg-4-545-2007&partnerID=40&md5=dd1de2f26aa629ca674d5006f7dad2d0
AD - CoMPLEX (Centre for Mathematics and Physics in the Life Sciences and Experimental Biology), University College London, United Kingdom
AD - Physikalisches Institut, University of Bern, Switzerland
AD - Department of Biochemistry and Molecular Biology, University College London, United Kingdom
AD - Mullard Space Science Laboratory, University College London, United Kingdom
AB - The surface of Mars, unshielded by thick atmosphere or global magnetic field, is exposed to high levels of cosmic radiation. This ionising radiation field is deleterious to the survival of dormant cells or spores and the persistence of molecular biomarkers in the subsurface, and so its characterisation is of prime astrobiological interest. Here, we present modelling results of the absorbed

radiation dose as a function of depth through the Martian subsurface, suitable for calculation of biomarker persistence. A second major implementation of this dose accumulation rate data is in application of the optically stimulated luminescence technique for dating Martian sediments. We present calculations of the dose-depth profile in the Martian subsurface for various scenarios: variations of surface composition (dry regolith, ice, layered permafrost), solar minimum and maximum conditions, locations of different elevation (Olympus Mons, Hellas basin, datum altitude), and increasing atmospheric thickness over geological history. We also model the changing composition of the subsurface radiation field with depth compared between Martian locations with different shielding material, determine the relative dose contributions from primaries of different energies, and discuss particle deflection by the crustal magnetic fields.
KW - Bacteria (microorganisms)
KW - biomarker
KW - cosmic ray
KW - geodetic datum
KW - ionization
KW - luminescence dating
KW - magnetic field
KW - Mars
KW - Martian atmosphere
KW - radiation exposure
KW - sporulation
PB - European Geosciences Union
SN - 17264170 (ISSN)
LA - English
J2 - Biogeosciences
M3 - Article
DB - Scopus
N1 - Export Date: 23 February 2023; Cited By: 78; Correspondence Address: L.R. Dartnell; CoMPLEX (Centre for Mathematics and Physics in the Life Sciences and Experimental Biology), University College London, United Kingdom; email: l.dartnell@ucl.ac.uk
ER -

TY - JOUR
AU - Gilichinsky, D.A.
AU - Wilson, G.S.
AU - Friedmann, E.I.
AU - McKay, C.P.
AU - Sletten, R.S.
AU - Rivkina, E.M.
AU - Vishnivetskaya, T.A.
AU - Erokhina, L.G.
AU - Ivanushkina, N.E.
AU - Kochkina, G.A.
AU - Scherbakova, V.A.
AU - Soina, V.S.
AU - Spirina, E.V.
AU - Vorobyova, E.A.
AU - Frodorov-Davydov, D.G.

AU - Hallet, B.
AU - Ozerskaya, S.M.
AU - Sorokovikov, V.A.
AU - Laurinavichyus, K.S.
AU - Shatilovich, A.V.
AU - Chanton, J.P.
AU - Ostroumov, V.E.
AU - Tiedje, J.M.
TI - Microbial populations in Antarctic permafrost: Biodiversity, stage, age, and implication for astrobiology
PY - 2007
T2 - Astrobiology
VL - 7
IS - 2
SP - 275
EP - 311
DO - 10.1089/ast.2006.0012
UR - https://www.scopus.com/inward/record.uri?eid=2-s2.0-34250759399&doi=10.1089%2fast.2006.0012&partnerID=40&md5=a75cb071b8c9f15c323b69733d72092a
AD - Institute of Physicochemical and Biological Problems in Soil Science, Russian Academy of Sciences, Pushchino, Moscow Region, Russian Federation
AD - Geology Department, University of Otago, Dunedin, New Zealand
AD - Space Science Division, NASA Ames Research Center, Moffett Field, CA, United States
AD - Department of Earth and Space Sciences, Quaternary Research Center, University of Washington, Seattle, WA, United States
AD - Institute of Basic Biological Problems, Russian Academy of Sciences, Pushchino, Moscow Region, Russian Federation
AD - Institute of Biochemistry and Physiology of Microorganisms, Russian Academy of Sciences, Pushchino, Moscow Region, Russian Federation
AD - Department of Soil Science, Moscow State University, Moscow, Russian Federation
AD - Department of Oceanography, Florida State University, Tallahassee, FL, United States
AD - Center for Microbial Ecology, Michigan State University, East Lansing, MI, United States
AB - Antarctic permafrost soils have not received as much geocryological and biological study as has been devoted to the ice sheet, though the permafrost is more stable and older and inhabited by more microbes. This makes these soils potentially more informative and a more significant microbial repository than ice sheets. Due to the stability of the subsurface physicochemical regime, Antarctic permafrost is not an extreme environment but a balanced natural one. Up to 104 viable cells/g, whose age presumably corresponds to the longevity of the permanently frozen state of the sediments, have been isolated from Antarctic permafrost. Along with the microbes, metabolic by-products are preserved. This presumed natural cryopreservation makes it possible to observe what may be the oldest microbial communities on Earth. Here, we describe the Antarctic permafrost habitat and biodiversity and provide a model for martian ecosystems. © Mary Ann Liebert, Inc.

KW - Antarctic Regions
KW - Biodiversity
KW - Exobiology
KW - Ice
KW - Soil Microbiology
KW - Water
KW - ice
KW - water
KW - Antarctica
KW - article
KW - biodiversity
KW - biology
KW - microbiology
KW - Ancient ecosystem
KW - Antarctica
KW - Extraterrestrial model
KW - Permafrost
KW - Viable microorganisms
SN - 15311074 (ISSN)
C2 - 17480161
LA - English
J2 - Astrobiology
M3 - Article
DB - Scopus
N1 - Export Date: 23 February 2023; Cited By: 195; Correspondence Address: D.A. Gilichinsky; Institute of Physicochemical and Biological Problems in Soil Science, Russian Academy of Sciences, Pushchino, Moscow Region, Russian Federation; email: gilichin@online.stack.net
ER -

TY - JOUR
AU - Kiang, N.Y.
AU - Segura, A.
AU - Tinetti, G.
AU - Blankenship, R.E.
AU - Cohen, M.
AU - Siefert, J.
AU - Crisp, D.
AU - Meadows, V.S.
TI - Spectral signatures of photosynthesis. II. Coevolution with other stars and the atmosphere on extrasolar worlds
PY - 2007
T2 - Astrobiology
VL - 7
IS - 1
SP - 252
EP - 274
DO - 10.1089/ast.2006.0108
UR - https://www.scopus.com/inward/record.uri?eid=2-s2.0-34249735839&doi=10.1089%2fast.2006.0108&partnerID=40&md5=8841c0dd06c8df6c32f97db9b58ab1b5
AD - NASA Goddard Institute for Space Studies, New York, NY 10025, United States

AD - NASA Astrobiology Institute, Virtual Planetary Laboratory, United States
AD - Instituto de Ciencias Nucleares, Universidad Nacional Autónoma de México, México City, Mexico
AD - Institut d'Astrophysique de Paris, European Space Agency, Paris, France
AD - Departments of Plant Biology and Biochemistry, University of Illinois at Urbana-Champaign, Urbana, IL, United States
AD - Department of Biology and Chemistry, Washington University, St. Louis, MO, United States
AD - Radio Astronomy Laboratory, University of California, Berkeley, CA, United States
AD - Department of Statistics, Rice University, Houston, TX, United States
AD - NASA Jet Propulsion Laboratory, Pasadena, CA, United States
AD - Spitzer Science Center, California Institute of Technology, Pasadena, CA, United States
AB - As photosynthesis on Earth produces the primary signatures of life that can be detected astronomically at the global scale, a strong focus of the search for extrasolar life will be photosynthesis, particularly photosynthesis that has evolved with a different parent star. We take previously simulated planetary atmospheric compositions for Earth-like planets around observed F2V and K2V, modeled M1V and M5V stars, and around the active M4.5V star AD Leo; our scenarios use Earth's atmospheric composition as well as very low O2 content in case anoxygenic photosynthesis dominates. With a line-by-line radiative transfer model, we calculate the incident spectral photon flux densities at the surface of the planet and under water. We identify bands of available photosynthetically relevant radiation and find that photosynthetic pigments on planets around F2V stars may peak in absorbance in the blue, K2V in the red-orange, and M stars in the near-infrared, in bands at 0.93-1.1μm, 1.1-1.4μm, 1.5-1.8 μm, and 1.8-2.5μm. However, underwater organisms will be restricted to wavelengths shorter than 1.4μm and more likely below 1.1μm. M star planets without oxygenic photosynthesis will have photon fluxes above 1.6μm curtailed by methane. Longer-wavelength, multi-photo-system series would reduce the quantum yield but could allow for oxygenic photosystems at longer wavelengths. A wavelength of 1.1μm is a possible upper cutoff for electronic transitions versus only vibrational energy; however, this cutoff is not strict, since such energetics depend on molecular configuration. M star planets could be a half to a tenth as productive as Earth in the visible, but exceed Earth if useful photons extend to 1.1μm for anoxygenic photosynthesis. Under water, organisms would still be able to survive ultraviolet flares from young M stars and acquire adequate light for growth. © Mary Ann Liebert, Inc.
KW - Astronomy
KW - Atmosphere
KW - Earth (Planet)
KW - Evolution, Planetary
KW - Exobiology
KW - Extraterrestrial Environment
KW - Oxygen
KW - Photons

KW - Photosynthesis
KW - Pigments, Biological
KW - Planets
KW - Solar Activity
KW - Water
KW - oxygen
KW - pigment
KW - water
KW - astronomy
KW - atmosphere
KW - biology
KW - microclimate
KW - photon
KW - photosynthesis
KW - review
KW - solar energy
KW - AD Leo
KW - Anoxygenic photosynthesis
KW - Astrobiology
KW - Atmospheric oxygen
KW - Atmospheric photochemistry
KW - Atmospheric radiative transfer
KW - Bacteriochlorophyll
KW - Chlorophyll
KW - Earth-like planets
KW - Extrasolar planets
KW - F stars
KW - G stars
KW - K stars
KW - Light harvesting
KW - M stars
KW - Modeling
KW - Oxygenic photosynthesis
KW - Photosynthesis
KW - Photosynthetic pigments
KW - Photosynthetically active radiation
KW - Photosystems
KW - Radiation spectrum
KW - Sun
KW - Virtual Planet
SN - 15311074 (ISSN)
C2 - 17407410
LA - English
J2 - Astrobiology
M3 - Article
DB - Scopus
N1 - Export Date: 23 February 2023; Cited By: 171; Correspondence Address: N.Y. Kiang; NASA Goddard Institute for Space Studies, New York, NY 10025, United States; email: nkiang@giss.nasa.gov
ER -

TY - JOUR
AU - Bowden, S.A.
AU - Wilson, R.

AU - Taylor, C.
AU - Cooper, J.M.
AU - Parnell, J.
TI - The extraction of intracrystalline biomarkers and other organic compounds from sulphate minerals using a microfluidic format - A feasibility study for remote fossil-life detection using a microfluidic H-cell
PY - 2007
T2 - International Journal of Astrobiology
VL - 6
IS - 1
SP - 27
EP - 36
DO - 10.1017/S147355040600351X
UR - https://www.scopus.com/inward/record.uri?eid=2-s2.0-34548126139&doi=10.1017%2fS147355040600351X&partnerID=40&md5=e9fb7ec1f47df1860c63495ea6b3cdfd
AD - Department of Geology and Petroleum Geology, University of Aberdeen, Aberdeen AB24 3UE, United Kingdom
AD - Department of Electronics and Electrical Engineering, University of Glasgow, Glasgow, United Kingdom
AB - Water-soluble sulphate minerals such as epsomite and gypsum have been reported on a number of planetary bodies of astrobiological interest and their presence is often used as an indicator for potential habitability as it can indicate the presence of liquid water. The sulphate minerals can incorporate biomarkers and biomolecules as intracrystalline inclusions. These intracrystalline inclusions are protected from their exterior chemical environments and their analysis can yield a biomarker record, even in environments where extreme oxidizing conditions may have degraded any record present in non-intracrystalline organic matter. In this study, organic compounds were incorporated within epsomite as intracrystalline inclusions by co-precipitating a mineral from solutions of a mineral and organic compound. A feasibly study utilizing a microfluidic H-cell to process finely ground samples of the inclusion-bearing epsomite indicates that a similar device may be used to extract biomarkers for analysis. © 2007 Cambridge University Press.
KW - biomarker
KW - gypsum
KW - organic compound
KW - sulfate
KW - Extraction
KW - H-cell
KW - Inclusions
KW - Intracrystalline
KW - Life detection
KW - Microfluidic
KW - Sulphate
SN - 14753006 (ISSN)
LA - English
J2 - Int. J. Astrobiology
M3 - Article
DB - Scopus

N1 - Export Date: 23 February 2023; Cited By: 7; Correspondence Address: S.A. Bowden; Department of Geology and Petroleum Geology, University of Aberdeen, Aberdeen AB24 3UE, United Kingdom; email: s.a.bowden@abdn.ac.uk
ER -

TY - JOUR
AU - Jalbout, A.F.
AU - Abrell, L.
AU - Adamowicz, L.
AU - Polt, R.
AU - Apponi, A.J.
AU - Ziurys, L.M.
TI - Sugar synthesis from a gas-phase formose reaction
PY - 2007
T2 - Astrobiology
VL - 7
IS - 3
SP - 433
EP - 442
DO - 10.1089/ast.2006.0083
UR - https://www.scopus.com/inward/record.uri?eid=2-s2.0-34547750352&doi=10.1089%2fast.2006.0083&partnerID=40&md5=b1476cf34ae0316ac888f878eeec28db
AD - Instituto de Quimica, Universidad Nacional Autonoma de Mexico, Ciudad Universitaria, Mexico D.F., Mexico
AD - Department of Physical Chemistry, Institute of Chemistry, National Autonomous University of Mexico, 04510 Mexico City, Col. Coyoacan, Del. Copilco, Mexico
AD - NASA Astrobiology Institute, The University of Arizona, Tucson, AZ, United States
AD - Department of Chemistry, The University of Arizona, Tucson, AZ, United States
AD - Department of Astronomy, The University of Arizona, Tucson, AZ, United States
AD - Steward Observatory, The University of Arizona, Tucson, AZ, United States
AB - Prebiotic possibilities for the synthesis of interstellar ribose through a protic variant of the formose reaction under gas-phase conditions were studied in the absence of any known catalyst. The ion-molecule reaction products, diose and triose, were sought by mass spectrometry, and relevant masses were observed. Ab initio calculations were used to evaluate protic formose mechanism possibilities. A bilateral theoretical and experimental effort yielded a physical model for glycoaldehyde generation whereby a hydronium cation can mediate formaldehyde dimerization followed by covalent bond formation leading to those and water. These results advance the possibility that ion-molecule reactions between formaldehyde (CH2O) and H3O+ lead to formose reaction products and inform us about potential sugar formation processes in interstellar space. © Mary Ann Liebert, Inc.
KW - Biogenesis
KW - Carbohydrates
KW - Diphosphonates

KW - Exobiology
KW - Formaldehyde
KW - Gases
KW - Models, Chemical
KW - Onium Compounds
KW - Ribose
KW - Thermodynamics
KW - bisphosphonic acid derivative
KW - carbohydrate
KW - cation
KW - formaldehyde
KW - formose sugars
KW - hydronium ion
KW - incadronic acid
KW - ribose
KW - unclassified drug
KW - article
KW - biogenesis
KW - biology
KW - chemical model
KW - chemistry
KW - gas
KW - synthesis
KW - thermodynamics
KW - Astrobiology
KW - Interstellar medium, clouds
KW - Interstellar medium, molecules
KW - Laboratory methods
KW - Molecular processes
SN - 15311074 (ISSN)
C2 - 17630839
LA - English
J2 - Astrobiology
M3 - Article
DB - Scopus
N1 - Export Date: 23 February 2023; Cited By: 37; Correspondence Address: A.F. Jalbout; Department of Physical Chemistry, Institute of Chemistry, National Autonomous University of Mexico, 04510 Mexico City, Mexico; email: ajalbout@u.arizona.edu
ER -

TY - JOUR
AU - Channing, A.
AU - Butler, I.B.
TI - Cryogenic opal-A deposition from Yellowstone hot springs
PY - 2007
T2 - Earth and Planetary Science Letters
VL - 257
IS - 1-2
SP - 121
EP - 131
DO - 10.1016/j.epsl.2007.02.026
UR - https://www.scopus.com/inward/record.uri?eid=2-s2.0-34247366021&doi=10.1016%2fj.epsl.2007.02.026&partnerID=40&md5=e

e1887fa8d07565695d40171e65d602e
AD - School of Earth, Ocean and Planetary Sciences, Cardiff University, Cardiff, Wales CF10 3YE, PO Box 914, United Kingdom
AD - School of GeoSciences, The University of Edinburgh, Grant Institute, Edinburgh, Scotland, EH9 3JW, West Mains Road, United Kingdom
AB - Sub-zero winter temperatures on the Yellowstone Plateau alter the opal-A precipitation pathway of fluid erupting from hot springs and geysers. Frozen fluid, often only meters from boiling pools, contains abundant opal-A particles, comprising sheet and filament-like aggregations of opal-A microspheres which are formed by opal-A precipitation in brine pockets, channels and veins by natural cryogelling. Unconsolidated cryogenic opal-A sediment accumulates in and below water-ice where it is locked until spring thaw conditions. Sediment is then either remobilized, contributing large volumes of opal-A particulate to geothermally influenced wetlands, or becomes adhered, in situ, by dehydration and cementation. This strongly seasonal opal-A precipitation regime has been overlooked in investigations of sinter deposition, accretion rates and microbe/ mineral interactions. Natural opal-A textures recorded from Yellowstone may be replicated simply by freezing and thawing synthetic silica-salt solution in the laboratory. Cryogenic process may have influenced mineral precipitation and sediment accumulation in many other geothermal areas. Particularly, active terrestrial springs located at high altitude/latitude, fossil systems influenced by ancient glaciations, plus potential astrobiological targets e.g. Mars and Europa. © 2007 Elsevier B.V. All rights reserved.
KW - Cryogenics
KW - Gems
KW - Geochemistry
KW - Hot springs
KW - Mineralogy
KW - Cryogelling
KW - Mineral precipitation
KW - Opal
KW - Sediment accumulation
KW - accumulation
KW - cryogenic fluid
KW - deposition
KW - freezing
KW - geochemistry
KW - geyser
KW - opal
KW - silica
KW - thawing
KW - thermal spring
KW - Silica
KW - geochemistry
KW - hot spring
KW - opal
KW - silica
KW - Yellowstone
SN - 0012821X (ISSN)
LA - English

J2 - Earth Plan. Sci. Lett.
M3 - Article
DB - Scopus
N1 - Export Date: 23 February 2023; Cited By: 37; Correspondence Address: A. Channing; School of Earth, Ocean and Planetary Sciences, Cardiff University, Cardiff, Wales CF10 3YE, PO Box 914, United Kingdom; email: channinga@cardiff.ac.uk
ER -

TY - CONF
AU - Kociolek, J.P.
TI - Diatoms: Unique eukaryotic extremophiles providing insights into planetary change
PY - 2007
T2 - Proceedings of SPIE - The International Society for Optical Engineering
VL - 6694
C7 - 66940S
DO - 10.1117/12.731445
UR - https://www.scopus.com/inward/record.uri?eid=2-s2.0-42349098892&doi=10.1117%2f12.731445&partnerID=40&md5=f9427436785def43e44e0dffa7404d47
AD - Diatom Collection, California Academy of Sciences, San Francisco, CA 94103, 875 Howard Street, United States
AD - University of Michigan, Biological Station, Pellston, MI 49769, United States
AB - The diatoms (Division Bacillariophyta) are aquatic, pigmented single-celled photosynthetic eukaryotes. They are a major component of the food chain in marine, estuarine and freshwater ecosystems. These diploid organisms have the ability to take dissolved silica out of the water column, and use it to create their cell walls. The taxonomy and classification system for the group is based to a large part on cell wall symmetry, as well as the number, type, position and organization of the many perforations in the glass. As a group, diatoms are found in almost every water type of water body around the globe, including a wide range of extreme environments. Individual species, however, have limited distributions and ecological requirements. Preservation of the glass cell walls in sedimentary basins has left nearly a 120 million year record. Species-related distributions, and well-preserved record make diatoms an excellent tool for environmental reconstruction and monitoring. New research on diatoms includes applications to conservation biology, astrobiology, nanotechnology, and biofuels.
KW - Biodiversity
KW - Biofuels
KW - Climate change
KW - Bacillariophyta
KW - Climate change diatoms
KW - Fossils
KW - Astrobiology
KW - Bacillariophyta
KW - Biodiversity
KW - Biofuels
KW - Climate change diatoms

KW - Extremophiles
KW - Fossils
KW - Water
SN - 0277786X (ISSN); 978-081946842-0 (ISBN)
LA - English
J2 - Proc SPIE Int Soc Opt Eng
M3 - Conference paper
DB - Scopus
N1 - Export Date: 23 February 2023; Cited By: 7; CODEN: PSISD
ER -

TY - CONF
AU - Cabrol, N.A.
AU - Grin, E.A.
AU - Hock, A.N.
TI - Mitigation of environmental extremes as a possible indicator of extended habitat sustainability for lakes on early Mars
PY - 2007
T2 - Proceedings of SPIE - The International Society for Optical Engineering
VL - 6694
C7 - 669410
DO - 10.1117/12.731506
UR - https://www.scopus.com/inward/record.uri?eid=2-s2.0-42349102157&doi=10.1117%2f12.731506&partnerID=40&md5=e3680943b059e06e19656865ce133743
AD - NASA Ames Research Center, SETI Carl Sagan Center, Space Science Division, Moffett Field, CA 94035-1000, United States
AD - UCLA, Dpt. of Earth and Space Sciences, Los Angeles, CA 90095-1567, 595 Charles Young Drive East, United States
AB - The impact of individual extremes on life, such as UV radiation (UVR), temperatures, and salinity is well documented. However, their combined effect in nature is not well-understood while it is a fundamental issue controlling the evolution of habitat sustainability within individual bodies of water. Environmental variables combine in the Bolivian Altiplano to produce some of the highest, least explored and most poorly understood lakes on Earth. Their physical environment of thin atmosphere, high ultraviolet radiation, high daily temperature amplitude, ice, sulfur-rich volcanism, and hydrothermal springs, combined with the changing climate in the Andes and the rapid loss of aqueous habitat provide parallels to ancient Martian lakes at the Noachian/Hesperian transition 3.7-3.5 Ga ago. Documenting this analogy is one of the focuses of the High-Lakes Project (HLP). The geophysical data we collected on three of them located up to 5,916 m elevation suggests that a combination of extreme factors does not necessarily translate into a harsher environment for life. Large and diverse ecosystems adapt to UVR reaching 200%-216% that of sea level in bodies of water sometimes no deeper than 50 cm, massive seasonal freeze-over, and unpredictable daily evolution of UVR and temperature. The HLP project has undertaken the first complete geophysical and biological characterization of these lakes and documents how habitability is sustained and prolonged in declining lakes despite a highly dynamical environment. The same process may have helped life

transition through climate crises over time on both Earth and Mars.
KW - Astrobiology
KW - Geophysical prospecting
KW - Ultraviolet radiation
KW - Habitability
KW - Habitat sustainability
KW - Mars analog
KW - Martian surface analysis
KW - Environmental extremes
KW - Habitability
KW - Habitat sustainability
KW - High-altitude lakes
KW - High-lakes project
KW - Life potential on Mars
KW - Mars
KW - Mars analog
SN - 0277786X (ISSN); 978-081946842-0 (ISBN)
LA - English
J2 - Proc SPIE Int Soc Opt Eng
M3 - Conference paper
DB - Scopus
N1 - Export Date: 23 February 2023; Cited By: 7; Correspondence Address: N. A. Cabrol; NASA Ames Research Center, SETI Carl Sagan Center, Space Science Division, Moffett Field, CA 94035-1000, United States; email: ncabrol@mail.arc.nasa.gov; CODEN: PSISD
ER -

TY - CONF
AU - Liesch, P.J.
AU - Kolb, V.M.
TI - The importance of the Maillard-metal complexes and their silicates in astrobiology
PY - 2007
T2 - Proceedings of SPIE - The International Society for Optical Engineering
VL - 6694
C7 - 66941G
DO - 10.1117/12.731342
UR - https://www.scopus.com/inward/record.uri?eid=2-s2.0-42149180025&doi=10.1117%2f12.731342&partnerID=40&md5=b3a8f019f6c5ab4623256104723560d0
AD - Department of Chemistry, University of Wisconsin-Parkside, Kenosha, WI 53141, United States
AB - The Maillard reaction occurs when sugars and amino acids are mixed together in the solid state or in the aqueous solution. Since both amino acids and sugar-like compounds are found on meteorites, we hypothesized that they would also undergo the Maillard reaction. Our recent work supports this idea. We have shown previously that the water-insoluble Maillard products have substantial similarities with the insoluble organic materials from the meteorites. The Maillard organic materials are also part of the desert varnish on Earth, which is a dark, shiny, hard rock coating that contains iron and manganese and is glazed in silicate. Rocks that are similar in appearance to the desert varnish have been observed on the Martian

surface. They may also contain the organic materials. We have undertaken study of the interactions between the Maillard products, iron and other metals, and silicates, to elucidate the role of the Maillard products in the chemistry of desert varnish and meteorites. Specifically, we have synthesized a series of the Maillard-metal complexes, and have tested their reactivity towards silicates. We have studied the properties of these Maillard-metal-silicate products by the IR spectroscopy. The astrobiological potential of the Maillard-metal complexes is assessed.
KW - Amino acids
KW - Astrobiology
KW - Manganese
KW - Silicates
KW - Maillard reaction
KW - Maillard-metal complexes
KW - Maillard-metal-silicate complexes
KW - Metal complexes
KW - Astrobiology
KW - Maillard reaction
KW - Maillard-metal complexes
KW - Maillard-metal-silicate complexes
SN - 0277786X (ISSN); 978-081946842-0 (ISBN)
LA - English
J2 - Proc SPIE Int Soc Opt Eng
M3 - Conference paper
DB - Scopus
N1 - Export Date: 23 February 2023; Cited By: 1; CODEN: PSISD
ER -

TY - CONF
AU - Gronstal, A.
AU - Cockell, C.S.
AU - Perino, M.A.
AU - Bittner, T.
AU - Clacey, E.
AU - Clark, O.
AU - Ingold, O.
AU - De Oliveira, C.A.
AU - Wathiong, S.
TI - Lunar astrobiology: A review and suggested laboratory equipment
PY - 2007
T2 - Astrobiology
VL - 7
IS - 5
SP - 767
EP - 782
DO - 10.1089/ast.2006.0082
UR - https://www.scopus.com/inward/record.uri?eid=2-s2.0-35548945439&doi=10.1089%2fast.2006.0082&partnerID=40&md5=5afa7927f358c6b54176cb7e0b3d655e
AD - Planetary and Space Sciences Research Institute, Open University, Milton Keynes, United Kingdom
AD - Thales Alenia Space Italia, Torino, Italy

AD - Zentrum für Neuropathologie und Prionforschung, Ludwig-Maximilians-Universität, München, Germany
AD - Swedish Space Corporation, Stockholm, Sweden
AD - Environmental Biology, University of Guelph, Guelph, Canada
AD - International Space University, Strasbourg, France
AD - European Southern Observatory, München, Germany
AB - In October of 2005, the European Space Agency (ESA) and Alcatel Alenia Spazio released a "call to academia for innovative concepts and technologies for lunar exploration." In recent years, interest in lunar exploration has increased in numerous space programs around the globe, and the purpose of our study, in response to the ESA call, was to draw on the expertise of researchers and university students to examine science questions and technologies that could support human astrobiology activity on the Moon. In this mini review, we discuss astrobiology science questions of importance for a human presence on the surface of the Moon and we provide a summary of key instrumentation requirements to support a lunar astrobiology laboratory. © Mary Ann Liebert, Inc.
KW - Exobiology
KW - Extraterrestrial Environment
KW - Humans
KW - Laboratories
KW - Moon
KW - Research Design
KW - biology
KW - conference paper
KW - human
KW - instrumentation
KW - laboratory
KW - methodology
KW - microclimate
KW - moon
KW - Human lunar exploration
KW - Instrumentation
KW - Lunar astrobiology
KW - Microbiology
KW - Planetary protection
SN - 15311074 (ISSN)
C2 - 17963476
LA - English
J2 - Astrobiology
M3 - Conference paper
DB - Scopus
N1 - Export Date: 23 February 2023; Cited By: 17; Correspondence Address: A. Gronstal; Planetary and Space Sciences Research Institute, Open University, Milton Keynes, United Kingdom; email: A.L.Gronstal@open.ac.uk
ER -

TY - JOUR
AU - Fogg, M.J.
AU - Nelson, R.P.
TI - On the formation of terrestrial planets in hot-Jupiter systems
PY - 2007

T2 - Astronomy and Astrophysics
VL - 461
IS - 3
SP - 1195
EP - 1208
DO - 10.1051/0004-6361:20066171
UR - https://www.scopus.com/inward/record.uri?eid=2-s2.0-33846401733&doi=10.1051%2f0004-6361%3a20066171&partnerID=40&md5=17f58ca8cba7f580e5581b4709f65884
AD - Astronomy Unit, Queen Mary, University of London, London E1 4NS, Mile End Road, United Kingdom
AB - Context. There are numerous extrasolar giant planets which orbit close to their central stars. These "hot-Jupiters" probably formed in the outer, cooler regions of their protoplanetary disks, and migrated inward to ~0.1 AU. Since these giant planets must have migrated through their inner systems at an early time, it is uncertain whether they could have formed or retained terrestrial planets. Aims. We present a series of calculations aimed at examining how an inner system of planetesimals/protoplanets, undergoing terrestrial planet formation, evolves under the influence of a giant planet undergoing inward type II migration through the region bounded between 5-0.1 AU. Methods. We have previously simulated the effect of gas giant planet migration on an inner system protoplanet/planetesimal disk using a N-body code which included gas drag and a prescribed migration rate. We update our calculations here with an improved model that incorporates a viscously evolving gas disk, annular gap and inner-cavity formation due to the gravitational field of the giant planet, and self-consistent evolution of the giant's orbit. Results. We find that ≥60% of the solids disk survives by being scattered by the giant planet into external orbits. Planetesimals are scattered outward almost as efficiently as protoplanets, resulting in the regeneration of a solids disk where dynamical friction is strong and terrestrial planet formation is able to resume. A simulation that was extended for a few Myr after the migration of the giant planet halted at 0.1 AU, resulted in an apparently stable planet of ~2 m⊕ forming in the habitable zone. Migration-induced mixing of volatile-rich material from beyond the "snowline" into the inner disk regions means that terrestrial planets that form there are likely to be water-rich. Conclusions. We predict that hot-Jupiter systems are likely to harbor water-abundant terrestrial planets in their habitable zones. These planets may be detected by future planet search missions. © ESO 2007.
KW - Computer simulation
KW - Satellites
KW - Solar system
KW - Protoplanetary disks
KW - Protoplanets
KW - Terrestrial planet formation
KW - Terrestrial planets
KW - Planets
KW - Astrobiology
KW - Methods: N-body simulations
KW - Planets and satellites: Formation

SN - 14320746 (ISSN)
LA - English
J2 - Astron. Astrophys.
M3 - Article
DB - Scopus
N1 - Export Date: 23 February 2023; Cited By: 50; Correspondence Address: M.J. Fogg; Astronomy Unit, Queen Mary, University of London, London E1 4NS, Mile End Road, United Kingdom; email: M.J.Fogg@qmul.ac.uk; CODEN: AAEJA
ER -

TY - CONF
AU - Vakoch, D.A.
TI - Humankind's planetary ecological crisis as a central theme for interstellar message composition
PY - 2007
T2 - International Astronautical Federation - 58th International Astronautical Congress 2007
VL - 2
SP - 1336
EP - 1341
UR - https://www.scopus.com/inward/record.uri?eid=2-s2.0-54949104616&partnerID=40&md5=b64e092554215df9f2ef1da8714e7d75
AD - Clinical Psychology Doctoral Program, California Institute of Integral Studies, San Francisco, CA 94103, 1454 Mission Street, United States
AD - SETI Institute, Mountain View, CA 94043, 515 N. Whisman Road, United States
AB - SETI search strategies typically assume that extraterrestrial civilizations are much longer lived than terrestrial civilization. That is, factor L in the Drake equation, the average lifetime of extraterrestrial civilizations as measured in the time they are actively seeking to make contact with other civilizations, is assumed to be much longer than the time that humans have had the technology and motivation to communicate at interstellar distances. Without this assumption, it is statistically improbable that extraterrestrial and human civilizations will exist sufficiently close enough to within the much longer lifetime of the galaxy to allow contact. This presumed asymmetry in the lifetimes of extraterrestrial and terrestrial civilizations has raised the question, "What would humans have to say that would be of interest to much older civilizations?" Typically it has been assumed that more long-lived civilizations will also be more technologically and scientifically advanced. If so, then humans are unlikely to be able to teach extraterrestrial civilizations much in these realms, at least assuming that there is a convergence of technological developments and scientific discoveries across civilizations, with more advanced civilizations attaining an understanding that encompasses and surpasses that of less advanced civilizations. Following the above assumptions, humankind is in possession of information that could be of significant scientific interest to astrobiologists on other worlds: information about the longevity of our own civilization, as well as factors that threaten our continued existence as a species. Although more advanced civilizations may be

able to glean some information about the threats to our survival as a species by monitoring atmospheric changes and unintentional leakage radiation from Earth, intentional messages describing the social, political, and ecological factors that contribute to the instability of our planet may provide a rare glimpse into the cultures of a young civilization that has some insight into the threats it faces. Whether or not we continue such transmissions over the millennia would be informative to sociologists and psychologists beyond Earth, potentially providing greater insights into the critical years during which civilizations attempt to make the transition to becoming long-lived civilizations themselves. Whether or not humankind succeeds, such messages from Earth could be useful to extraterrestrials attempting to understand better the factors that contribute to L. Copyright IAF/IAA. All rights reserved.
KW - Animal cell culture
KW - Sugar (sucrose)
KW - Technological forecasting
KW - Atmospheric changes
KW - Average lifetimes
KW - Ecological crises
KW - Ecological factors
KW - GREAT-ER
KW - Human civilizations
KW - Interstellar messages
KW - Leakage radiations
KW - Life-times
KW - Political
KW - Possession of informations
KW - Scientific discoveries
KW - Search strategies
KW - Technological developments
KW - Technology transfer
SN - 978-160560150-2 (ISBN)
LA - English
J2 - Int. Astronaut. Fed. - Int. Astronaut. Cong.
M3 - Conference paper
DB - Scopus
N1 - Export Date: 23 February 2023; Cited By: 0; Correspondence Address: D. A. Vakoch; Clinical Psychology Doctoral Program, California Institute of Integral Studies, San Francisco, CA 94103, 1454 Mission Street, United States; email: dvakoch@ciis.edu
ER -

TY - JOUR
AU - Martínez-Frías, J.
AU - Delgado-Huertas, A.
AU - García-Moreno, F.
AU - Reyes, E.
AU - Lunar, R.
AU - Rull, F.
TI - Isotopic signatures of extinct low-temperature hydrothermal chimneys in the Jaroso Mars analog
PY - 2007
T2 - Planetary and Space Science

VL - 55
IS - 4
SP - 441
EP - 448
DO - 10.1016/j.pss.2006.09.004
UR - https://www.scopus.com/inward/record.uri?eid=2-s2.0-33846955812&doi=10.1016%2fj.pss.2006.09.004&partnerID=40&md5=07da13c842ee5f26704f24d119dd5697
AD - Planetary Geology Laboratory, Centro de Astrobiologia (CSIC-INTA), associated to the NASA Astrobiology Institute, 28850 Torrejon de Ardoz, Madrid, Ctra de Ajalvir, km 4, Spain
AD - Department of Earth Sciences and Environmental Chemistry, Estación Experimental del Zaidín (CSIC), 18008 Granada, Prof. Albareda 1, Spain
AD - Departamento de Cristalografia y Mineralogía, Facultad de C.C. Geológicas, Universidad Complutense de Madrid, 28040 Madrid, Spain
AD - Cristalografía y Mineralogia, Unidad Asociada CSIC-Universidad de Valladolid, Valladolid, 47006, Spain
AB - The present work presents a geochemical study, focused on the oxygen and carbon isotopic signatures of shallow-marine, carbonate extinct chimneys, from Jaroso Hydrothermal System (JHS). In each chimney a meticulous sampling from the central orifice to the outer rim of the structure was performed. The isotopic geochemistry study allowed to establish the origin and evolution of the fluids during the formation of the vent structures. The negative δ13C values indicate a source of meteoric water for the Fe-rich fluids. More positive δ13C values are present in ankerite and in some calcite, both related with marine water. δ18O in ankerite indicates low-temperature hydrothermal conditions, while in calcite is showing either primary signatures or early diagenesis at low temperature. On the contrary, calcite displaying more negative δ13C and δ18O values represents a late mineral phase which was formed under meteoric diagenesis. Each chimney resulted from the precipitation of intergranular carbonate cement around a channellized flux of metal-rich fluid crossing a shallow-marine, unconsolidated, sandy-marl substrate. The paleoenvironmental interpretation carried out from the isotopic data emphasizes the importance of the stable isotopes as fluid geomarkers, also advancing in the understanding of an interesting analog for the geological and astrobiological exploration of Mars. © 2006 Elsevier Ltd. All rights reserved.
KW - Calcite
KW - Carbon
KW - Hydrothermal synthesis
KW - Isotopes
KW - Martian surface analysis
KW - Orifices
KW - Oxygen
KW - Ankerite
KW - Central orifice
KW - Jarosite
KW - Jaroso Hydrothermal System (JHS)
KW - Exploratory geochemistry
KW - Carbon
KW - Hydrothermal

KW - Isotopes
KW - Jarosite
KW - Mars
KW - Oxygen
SN - 00320633 (ISSN)
LA - English
J2 - Planet. Space Sci.
M3 - Article
DB - Scopus
N1 - Export Date: 23 February 2023; Cited By: 13; Correspondence Address: J. Martínez-Frías; Planetary Geology Laboratory, Centro de Astrobiologia (CSIC-INTA), associated to the NASA Astrobiology Institute, 28850 Torrejon de Ardoz, Madrid, Ctra de Ajalvir, km 4, Spain; email: martinezfrias@mncn.csic.es; CODEN: PLSSA
ER -

TY - JOUR
AU - Tarter, J.C.
AU - Backus, P.R.
AU - Mancinelli, R.L.
AU - Aurnou, J.M.
AU - Backman, D.E.
AU - Basri, G.S.
AU - Boss, A.P.
AU - Clarke, A.
AU - Deming, D.
AU - Doyle, L.R.
AU - Feigelson, E.D.
AU - Freund, F.
AU - Grinspoon, D.H.
AU - Haberle, R.M.
AU - Hauck II, S.A.
AU - Heath, M.J.
AU - Henry, T.J.
AU - Hollingsworth, J.L.
AU - Joshi, M.M.
AU - Kilston, S.
AU - Liu, M.C.
AU - Meikle, E.
AU - Reid, I.N.
AU - Rothschild, L.J.
AU - Scalo, J.
AU - Segura, A.
AU - Tang, C.M.
AU - Tiedje, J.M.
AU - Turnbull, M.C.
AU - Walkowicz, L.M.
AU - Weber, A.L.
AU - Young, R.E.
TI - A reappraisal of the habitability of planets around M dwarf stars
PY - 2007
T2 - Astrobiology
VL - 7

IS - 1
SP - 30
EP - 65
DO - 10.1089/ast.2006.0124
UR - https://www.scopus.com/inward/record.uri?eid=2-s2.0-34249708910&doi=10.1089%2fast.2006.0124&partnerID=40&md5=115aa36712151c7c6c45d70053168ee8
AD - SETI Institute, Mountain View, CA 94043, 515 North Whisman Road, United States
AD - University of California Los Angeles, Los Angeles, CA, United States
AD - University of California Berkeley, Berkeley, CA, United States
AD - Carnegie Institution of Washington, Washington, DC, United States
AD - British Antarctic Survey, Cambridge, United Kingdom
AD - NASA Goddard Space Flight Center, Greenbelt, MD, United States
AD - Pennsylvania State University, University Park, PA, United States
AD - Southwest Research Institute, Boulder, CO, United States
AD - NASA Ames Research Center, Moffett Field, CA, United States
AD - Case Western Reserve University, Cleveland, OH, United States
AD - Ecospheres Project, Greenwich, United Kingdom
AD - Georgia State University, Atlanta, GA, United States
AD - U.K. Meteorological Office, Reading, United Kingdom
AD - Ball Aerospace and Technologies Corporation, Boulder, CO, United States
AD - University of Hawaii, Honululu, HI, United States
AD - National Center for Science Education, Oakland, CA, United States
AD - Space Telescope Science Institute, Baltimore, MD, United States
AD - University of Texas at Austin, Austin, TX, United States
AD - California Institute of Technology, Pasadena, CA, United States
AD - California Academy of Sciences, San Francisco, CA, United States
AD - Michigan State University, East Lansing, MI, United States
AD - University of Washington, Seattle, WA, United States
AB - Stable, hydrogen-burning, M dwarf stars make up about 75% of all stars in the Galaxy. They are extremely long-lived, and because they are much smaller in mass than the Sun (between 0.5 and 0.08 MSun), their temperature and stellar luminosity are low and peaked in the red. We have re-examined what is known at present about the potential for a terrestrial planet forming within, or migrating into, the classic liquid-surface-water habitable zone close to an M dwarf star. Observations of protoplanetary disks suggest that planet-building materials are common around M dwarfs, but N-body simulations differ in their estimations of the likelihood of potentially habitable, wet planets that reside within their habitable zones, which are only about one-fifth to 1/50th of the width of that for a G star. Particularly in light of the claimed detection of the planets with masses as small as 5.5 and 7.5/Earth orbiting M stars, there seems no reason to exclude the possibility of terrestrial planets. Tidally locked synchronous rotation within

the narrow habitable zone does not necessarily lead to atmospheric collapse, and active stellar flaring may not be as much of an evolutionarily disadvantageous factor as has previously been supposed. We conclude that M dwarf stars may indeed be viable hosts for planets on which the origin and evolution of life can occur. A number of planetary processes such as cessation of geother mal activity or thermal and nonthermal atmospheric loss processes may limit the duration of planetary habitability to periods far shorter than the extreme lifetime of the M dwarf star. Nevertheless, it makes sense to include M dwarf stars in programs that seek to find habitable worlds and evidence of life. This paper presents the summary conclusions of an interdisciplinary workshop (http:// mstars.seti.org) sponsored by the NASA Astrobiology Institute and convened at the SETI Institute. © Mary Ann Liebert, Inc.
KW - Astronomy
KW - Biogenesis
KW - Ecosystem
KW - Exobiology
KW - Extraterrestrial Environment
KW - Planets
KW - astronomy
KW - biogenesis
KW - biology
KW - ecosystem
KW - microclimate
KW - review
KW - Habitability
KW - M dwarfs
KW - Planets
KW - Stars
SN - 15311074 (ISSN)
C2 - 17407403
LA - English
J2 - Astrobiology
M3 - Article
DB - Scopus
N1 - Export Date: 23 February 2023; Cited By: 250; Correspondence Address: J.C. Tarter; SETI Institute, Mountain View, CA 94043, 515 North Whisman Road, United States; email: tarter@seti.org
ER -

TY - JOUR
AU - Pikuta, E.V.
AU - Hoover, R.B.
AU - Tang, J.
TI - Microbial extremophiles at the limits of life
PY - 2007
T2 - Critical Reviews in Microbiology
VL - 33
IS - 3
SP - 183
EP - 209
DO - 10.1080/10408410701451948
UR - https://www.scopus.com/inward/record.uri?eid=2-

s2.0-34547263402&doi=10.1080%2f10408410701451948&partnerID=40&md5=d1aab42e0c81596cd6ce8f5153ef42de
AD - National Space Sciences and Technology Center/NASA, Astrobiology Laboratory, Huntsville, AL, United States
AD - Noblis, Falls Church, VA, United States
AD - National Space Sciences and Technology Center/NASA, VP-62, Astrobiology Laboratory, Huntsville, AL 35805, 320 Sparkman Dr., United States
AB - Prokaryotic extremophiles were the first representatives of life on Earth and they are responsible for the genesis of geological structures during the evolution and creation of all currently known ecosystems. Flexibility of the genome probably allowed life to adapt to a wide spectrum of extreme environments. As a result, modern prokaryotic diversity formed in a framework of physico-chemical factors, and it is composed of: thermophilic, psychrophilic, acidophilic, alkaliphilic, halophilic, barophilic, and radioresistant species. This artificial systematics cannot reflect the multiple actions of different environmental factors since one organism could unite characteristics of several extreme-groups. In this review we show the current status of studies in all fields of extremophiles and summarize the limits of life for different species of microbial extremophiles. We also discuss the finding of extremophiles from unusual places such as soils, and briefly review recent studies of microfossils in meteorites in the context of the significance of microbial extremophiles to Astrobiology. Copyright © Informa Healthcare.
KW - Archaea
KW - Bacterial Physiology
KW - Environmental Microbiology
KW - Microbial Viability
KW - Prokaryota
KW - copper
KW - DNA
KW - iron
KW - messenger RNA
KW - sodium chloride
KW - water
KW - Acidithiobacillus ferrooxidans
KW - acidophile
KW - alkalophile
KW - anaerobic growth
KW - astrobiology
KW - Bacillus
KW - bacterium
KW - barophile
KW - biology
KW - climate
KW - Clostridium
KW - Deinococcaceae
KW - Enterococcus faecalis
KW - environmental factor
KW - extremophile
KW - geography
KW - Haloferax mediterranei

KW - halophile
KW - microorganism
KW - Nanoarchaeota
KW - Natronobacterium
KW - nonhuman
KW - organism community
KW - organisms by habitat
KW - physical chemistry
KW - Picrophilus
KW - priority journal
KW - prokaryote
KW - psychrophile
KW - Pyrolobus
KW - radiosensitivity
KW - review
KW - soil analysis
KW - soil microflora
KW - Sulfolobus acidocaldarius
KW - thermophile
KW - Trichosporon beigelii
KW - Astrobiology
KW - Ecology of microorganisms
KW - Extremophiles
KW - Limits of life
KW - Microbial diversity
SN - 15497828 (ISSN)
C2 - 17653987
LA - English
J2 - Crit. Rev. Microbiol.
M3 - Review
DB - Scopus
N1 - Export Date: 23 February 2023; Cited By: 263; Correspondence Address: E.V. Pikuta; National Space Sciences and Technology Center/ NASA, VP-62, Astrobiology Laboratory, Huntsville, AL 35805, 320 Sparkman Dr., United States; email: elenapikuta@hotmail.com; CODEN: CRVMA
ER -

TY - CONF
AU - Smith, P.H.
TI - The phoenix mission to Mars
PY - 2007
T2 - Proceedings of SPIE - The International Society for Optical Engineering
VL - 6694
C7 - 66940P
DO - 10.1117/12.735887
UR - https://www.scopus.com/inward/record.uri?eid=2-s2.0-42149143597&doi=10.1117%2f12.735887&partnerID=40&md5=fce96a352d47ccd22e93c53b71d7c8f5
AD - Lunar and Planetary Laboratory, University of Arizona, Tucson, AZ 85705, 1415 N. Sixth Avenue, United States
AB - After a picture-perfect launch to Mars on August 4, 2007 (see fig. 1), the Phoenix mission will land near 70° N on the northern

lowlands on May 25, 2008 and perform an in situ investigation of the ice layer discovered by the Mars Odyssey scientists in 2002. Mars undergoes climate change through obliquity and orbital variations on time periods of 50,000 years. By analyzing the minerals, aqueous chemistry, and grain shapes of the soil associated with the ice, Phoenix will determine whether the ice has ever melted and modified the soil properties. Since water is a necessary substance for life on Earth, a major question for the mission is whether the northern plains represent a habitable zone on Mars. Besides water, the Phoenix team will assess the organic content of the soil and ice as well as the abundances of biologically active elements. Finally, the transport of water through soils and atmosphere is measured using a Canadian meteorological station supplemented by probes to evaluate soil conductivity.
KW - Astrobiology
KW - Climate change
KW - Earth (planet)
KW - Soils
KW - Biologically active elements
KW - Habitable zone
KW - Meteorological station
KW - Phoenix mission
KW - Planets
KW - Astrobiology
KW - Mars
KW - Phoenix mission
SN - 0277786X (ISSN); 978-081946842-0 (ISBN)
LA - English
J2 - Proc SPIE Int Soc Opt Eng
M3 - Conference paper
DB - Scopus
N1 - Export Date: 23 February 2023; Cited By: 0; Correspondence Address: P. H. Smith; Lunar and Planetary Laboratory, University of Arizona, Tucson, AZ 85705, 1415 N. Sixth Avenue, United States; email: psmith@lpl.arizona.edu; CODEN: PSISD
ER -

TY - CONF
AU - Cash, W.
AU - Schindhelm, E.
AU - Arenberg, J.
AU - Lo, A.
AU - Polidan, R.
AU - Kasdin, J.
AU - Vanderbei, R.
AU - Kilston, S.
AU - Noecker, C.
TI - External occulters for direct observation of exoplanets: An overview
PY - 2007
T2 - Proceedings of SPIE - The International Society for Optical Engineering
VL - 6687
C7 - 668712

DO - 10.1117/12.732645
UR - https://www.scopus.com/inward/record.uri?eid=2-s2.0-42149178749&doi=10.1117%2f12.732645&partnerID=40&md5=eb39ba1ba35e54d6457e1177c343865f
AD - University of Colorado
AD - Northrop-Grumman Space Technologies
AD - Princeton University
AD - Ball Aerospace
AB - Perhaps the most compelling piece of science and exploration now under discussion for future space missions is the direct study of planets circling other stars. Indirect means have established planets as common in the universe but have given us a limited view of their actual characteristics. Direct observation holds the potential to map entire planetary systems, view newly forming planets, find Earth-like planets and perform photometry to search for major surface features. Direct observations will also enable spectroscopy of exoplanets and the search for evidence of simple life in the universe. Recent advances in the design of external occulters - starshades that block the light from the star while passing exoplanet light - have lowered their cost and improved their performance to the point where we can now envision a New Worlds Observer that is both buildable and affordable with today's technology. We will summarize recent studies of such missions and show they provide a very attractive alternative near term mission.
KW - Planets
KW - Space research
KW - Coronography
KW - Exoplanets
KW - Astrobiology
KW - Astrobiology
KW - Coronography
KW - Exoplanets
SN - 0277786X (ISSN); 978-081946835-2 (ISBN)
LA - English
J2 - Proc SPIE Int Soc Opt Eng
M3 - Conference paper
DB - Scopus
N1 - Export Date: 23 February 2023; Cited By: 23; CODEN: PSISD
ER -

TY - JOUR
AU - Morring Jr., F.
TI - Top priority
PY - 2007
T2 - Aviation Week and Space Technology (New York)
VL - 167
IS - 2
SP - 27
UR - https://www.scopus.com/inward/record.uri?eid=2-s2.0-34547155219&partnerID=40&md5=8568f4c7b8a16af67806ce0d42f5bff6
AB - NASA is planning to increase its Mars sample-return mission by fitting the landers such as the 2009 Mars Science Laboratory with sample caches that can be retrieved and delivered back to Earth. The Mars astrobiology strategy space studies by the National Research

Council has planned for the launch of a sample-return mission to Mars by 2018. The Mars program represents 46% of the planetary division's budget that includes Mars rock studies to find chemical evidence of life in the planet. NASA's intent for Mars sample-return mission is diverged by the human exploration of the Moon. The European Space Agency (ESA) has planned to launch its ExoMars rover in 2013, which is followed by Mars-launch opportunity in 2016, 2018, and 2020. ESA is expected to cooperate with NASA on the Mars sample-return mission requiring a high bandwidth relay orbiter, as a part of Aurora program.
SN - 00052175 (ISSN)
LA - English
J2 - Aviat. Week Space Technol. (New York)
M3 - Article
DB - Scopus
N1 - Export Date: 23 February 2023; Cited By: 0; CODEN: AWSTA
ER -

TY - CHAP
AU - Muñoz Caro, G.M.
AU - Dartois, E.
TI - Search for solid O- and N-rich organic matter of prebiotic interest in space
PY - 2007
T2 - Advances in Geosciences: Volume 7: Planetary Science (PS)
SP - 155
EP - 167
DO - 10.1142/6494-vol7
UR - https://www.scopus.com/inward/record.uri?eid=2-s2.0-84967642706&doi=10.1142%2f6494-vol7&partnerID=40&md5=12958b6560af335c527d4f3ae02a8b1c
AD - Centro de Astrobiología, INTA-CSIC, Ctra. de Ajalvir, km 4, Torrejón de Ardoz, Madrid, 28850, Spain
AD - Institut d’Astrophysique Spatiale, UMR 8617, Université Paris XI, Baˆt. 121, Orsay, 91405, France
AB - Mainly based on our previous results, this article evaluates the presence of solid organic matter of prebiotic interest in space: from the carbon grains observed toward diffuse interstellar clouds and the organic grain mantles made from ice processing that are likely present in dense interstellar clouds and circumstellar regions, to the carbon component of solar system objects that could have delivered organic species to the early Earth [comets, meteorites, and interplanetary dust particles (IDPs)]. Here the term organic is attributed to hydrocarbon materials rich in O and N that contain prebiotic species or their precursors, and are therefore interesting for astrobiology. Organic residues made in the laboratory from ice ultraviolet-photoprocessing under simulated interstellar conditions are used as representative of organic matter and compared by means of infrared and Raman spectroscopy to carbon-bearing extraterrestrial samples. It is observed that the carbon bulk in grains of the diffuse interstellar medium, carbonaceous chondrites, and the IDPs collected in the stratosphere consists of amorphous carbon, with at most a small percent of organic matter. On the other hand, about 50% of the carbon component in comet Halley is

made of organics that formed in the interstellar/circumstellar medium in the absence of liquid water. The characterization of cometary organic matter is therefore vital to constrain the contribution of extraterrestrial matter to the origin of life on Earth. © 2016 World Scientific Publishing Co Pte Ltd.
PB - World Scientific Publishing Co.
SN - 978-981270892-2 (ISBN); 981270986X (ISBN); 978-981270986-8 (ISBN)
LA - English
J2 - Advances in Geosciences: Volume 7: Planet. Science (PS)
M3 - Book chapter
DB - Scopus
N1 - Export Date: 23 February 2023; Cited By: 4
ER -

TY - CONF
AU - Ockels, W.J.
TI - Is time a creation of life in response to gravity? This hypothesis suggests new ways for looking at extraterrestrial life
PY - 2007
T2 - International Astronautical Federation - 58th International Astronautical Congress 2007
VL - 2
SP - 1285
EP - 1301
UR - https://www.scopus.com/inward/record.uri?eid=2-s2.0-54949105784&partnerID=40&md5=b38e2d92f35060ee77b04c9111c0c39c
AD - Delft University of Technology
AD - European Space Agency
AD - Delft University of Technology, Institute of Energy and Environment, University of Groningen
AD - GB Delft, Postbus 2600, Netherlands
AB - From his personal experience during a space flight (Challenger 1985) onward, the author has been struck repeatedly by the remarkable influence of Earth's environment on life, in particular by its most inevitable elements: time and gravity. Our life might be peculiar to the local Earth conditions, and not cosmic per se. In this article the hypothesis is postulated that our 'speed' of life, in relation to the speed of information (in this case the speed of light), is specific to humankind. Life is the process that 'makes' time. In this approach the constancy of the speed of light is not so much a property of the external world, but rather a consequence of our getting older at a fixed 'time-speed'. We are sitting in a 'time train'and all the information that we observe from the outside world is travelling relative to us at the same speed as our train. The train speed is, in a sense, the speed of light. If that is the case, then the expanding universe is an illusion. The remnants of the 'big bang' are standing 'still' while we move away and see all distances increasing. It is also shown in this article that there is a relationship between this time-speed and gravity, and that it can be the result of a process in the brain. By interpreting gravity as the result of a rotating motion, rather than a linear upward acceleration, time is introduced. In today's science we consider all universal processes in respect to our present (= now). In fact, we

believe that the universe started 13.6 billion years ago. This approach to science is set against the history of centralism: from the geocentrism of Ptolemy to the heliocentrism of Galilei, extrapolated to the 'chronocentrism' of today. An intriguing consequence of this theory is that extraterrestrial life would have a different speed of light. To couch this in a metaphor: we are living in a green world and see only green, while the others live in a red world and see only red. Each of these worlds can be part of one system, but we cannot see each other. Ideas are presented on how one might be able to communicate with these extraterrestrial living systems. Based on the assumption that different time propagations are still made up from the same time quanta, but with different lengths of empty time in between, one can imagine that those time speeds could be transferred by a 'replay' at our speed, like the frames in a film. Following the same assumption, a Lorentz transformation could connect both worlds, implying that the signals from another world would, for us, be split into two signals, each with a different time speed and distance speed not corresponding to our speed of light. Detecting those two signals simultaneously could lead to an intriguing experiment. Copyright IAF/IAA. All rights reserved.
KW - Astrophysics
KW - Cosmology
KW - Electromagnetic waves
KW - Extractive metallurgy
KW - Gravitation
KW - Gravitational effects
KW - High energy physics
KW - Light velocity
KW - Locomotives
KW - Mathematical transformations
KW - Railroad cars
KW - Space flight
KW - Big bangs
KW - Different speeds
KW - External-
KW - Extraterrestrial lives
KW - Living systems
KW - Lorentz transformations
KW - Personal experiences
KW - Ptolemy
KW - Rotating motions
KW - Speed of light
KW - Time propagations
KW - Time quantum
KW - Train speeds
KW - Speed
SN - 978-160560150-2 (ISBN)
LA - English
J2 - Int. Astronaut. Fed. - Int. Astronaut. Cong.
M3 - Conference paper
DB - Scopus
N1 - Export Date: 23 February 2023; Cited By: 0; Correspondence Address: W. J. Ockels; Delft University of Technology, Institute of

Energy and Environment, University of Groningen, Netherlands; email: w.j.ockels@tudeltt.nl
ER -

TY - CONF
AU - Badescu, M.
AU - Kassab, S.
AU - Sherrit, S.
AU - Aldrich, J.
AU - Bao, X.
AU - Bar-Cohen, Y.
AU - Chang, Z.
TI - Ultrasonic/sonic driller/corer as a hammer-rotary drill
PY - 2007
T2 - Proceedings of SPIE - The International Society for Optical Engineering
VL - 6529 PART 1
C7 - 65290S
DO - 10.1117/12.716493
UR - https://www.scopus.com/inward/record.uri?eid=2-s2.0-35548960941&doi=10.1117%2f12.716493&partnerID=40&md5=78c912bc485171fba0a5057afaf45783
AD - Jet Propulsion Laboratory, California Institute of Technology, MS 67-119, Pasadena, CA 91109-8099, 4800 Oak Grove Drive, United States
AD - Purdue University, West Lafayette, IN 47907, 400 Centennial Mall Dr., United States
AB - Rock, soil, and ice penetration by coring, drilling or abrading is of great importance for a large number of space and earth applications. Proven techniques to sample Mars subsurface will be critical for future NASA astrobiology missions that will search for past and present life on the planet. The Ultrasonic/Sonic Drill/Corer (USDC) has been developed as an adaptable tool for many of these applications [Bar-Cohen et al., 2001]. The USDC uses a novel drive mechanism to transform the ultrasonic or sonic vibrations of the tip of a horn into a sonic hammering of a drill bit through an intermediate free-flying mass. For shallow drilling the cuttings travel outside the hole due to acoustic vibrations of the bit. Various methods to enhance the drilling/coring depth of this device have been considered including pneumatic [Badescu et al., 2006] and bit rotation [Chang et al., 2006]. The combination of bit rotation at low speed for cuttings removal and bit hammering at sonic frequencies are described in this paper. The theoretical background and testing results are presented.
KW - Drilling
KW - Hammers
KW - Ice
KW - NASA
KW - Piezoelectric devices
KW - Rocks
KW - Soils
KW - Space applications
KW - Ice penetration
KW - Rotary hammer drills

KW - Sonic drillers
KW - Ultrasonic drilling
KW - Ultrasonic devices
KW - Piezoelectric devices
KW - Rotary-hammer drill
KW - Soil drill
KW - Ultrasonic drilling
SN - 0277786X (ISSN); 0819466506 (ISBN); 978-081946650-1 (ISBN)
LA - English
J2 - Proc SPIE Int Soc Opt Eng
M3 - Conference paper
DB - Scopus
N1 - Export Date: 23 February 2023; Cited By: 12; Correspondence Address: M. Badescu; Jet Propulsion Laboratory, California Institute of Technology, MS 67-119, Pasadena, CA 91109-8099, 4800 Oak Grove Drive, United States; email: Mircea.Badescu@jpl.nasa.gov; CODEN: PSISD
ER -

TY - CONF
AU - Raulin, F.
TI - Titan: A new astrobiological vision from Cassini-Huygens
PY - 2007
T2 - International Astronautical Federation - 58th International Astronautical Congress 2007
VL - 1
SP - 140
UR - https://www.scopus.com/inward/record.uri?eid=2-s2.0-54949099244&partnerID=40&md5=4e0394bfff1704fb86b95a91a0427809
AD - Université de Paris XII et Paris VII, Créteil, France
AB - After less than three years of close observation by remote sensing and in situ instrumentations from the Cassini-Huygens mission, Titan looks more and more as one of the key planetary bodies in the solar system for astrobiological studies. Titans does not look anymore like a frozen primitive Earth, but it looks now like an evolving planet, geologically active, with cryovolcanism, eolian erosion, clouds and precipitations, and a methane cycle very similar to the water cycle on Earth. But the new data also show that a very complex organic chemistry is taking place in the very high atmospheric zones of the satellite, with the formation in the ionosphere of high molecular weight (up to several 1000 Daltons) organics feeding the lower zones with these compounds, down to the surface. In spite of the low surface temperature, these organics are probably evolving once in contact with water ice and form organic molecules of biological interest. This evolution may explain the reflectance spectrum of Titan' surface observed by the DIRS instrument on Huygens, although, the lack of spectroscopic data in the near IR makes difficult the retrieving of these data. Thus, contrary to what was expected, the organic chemistry on Titan seems mainly concentrated in the ionosphere, on the surface and in the aerosols. These aspects will be examined, some of the associated questions will be answered on the basis of the already available Cassini-Huygens data. The needed post Cassini exploration and the associated future instrumentation will be also discussed.

KW - Aerosols
KW - Air pollution
KW - Instruments
KW - Ionosphere
KW - Ionospheric measurement
KW - Methane
KW - Remote sensing
KW - Solar system
KW - Space optics
KW - Cassini
KW - Cryovolcanism
KW - Huygens
KW - Huygens datum
KW - Huygens missions
KW - In situ instrumentations
KW - Low surface temperatures
KW - Near-ir
KW - Organic chemistries
KW - Organic molecules
KW - Organics
KW - Planetary bodies
KW - Reflectance spectrums
KW - Spectroscopic datum
KW - Water cycles
KW - Water ices
KW - Chemistry
SN - 978-160560150-2 (ISBN)
LA - English
J2 - Int. Astronaut. Fed. - Int. Astronaut. Cong.
M3 - Conference paper
DB - Scopus
N1 - Export Date: 23 February 2023; Cited By: 0; Correspondence Address: F. Raulin; Université de Paris XII et Paris VII, Créteil, France; email: raulin@lisa.univ-paris12.fr
ER -

TY - JOUR
AU - Costas, E.
AU - Flores-Moya, A.
AU - Perdigones, N.
AU - Maneiro, E.
AU - Blanco, J.L.
AU - García, M.E.
AU - López-Rodas, V.
TI - How eukaryotic algae can adapt to the Spain's Rio Tinto: A neo-Darwinian proposal for rapid adaptation to an extremely hostile ecosystem
PY - 2007
T2 - New Phytologist
VL - 175
IS - 2
SP - 334
EP - 339
DO - 10.1111/j.1469-8137.2007.02095.x

UR - https://www.scopus.com/inward/record.uri?eid=2-s2.0-34250881505&doi=10.1111%2fj.1469-8137.2007.02095.x&partnerID=40&md5=d1e17af6653a5e1f3603c2b092aedfff
AD - Genética (Producción Animal), Facultad de Veterinaria, Universidad Complutense, E-28040 Madrid, Spain
AD - Biología Vegetal (Botánica), Facultad de Ciencias, Universidad de Málaga, E-29071 Málaga, Spain
AD - Microbiología (Sanidad Animal), Facultad de Veterinaria, Universidad Complutense, E-28040 Madrid, Spain
AB - • Microalgae contributed 60% of the total biomass in the extremely hostile (pH 2 and metal-rich waters) environment of Rio Tinto (which is used as a model for the astrobiology of Mars). These algae are closely related to nonextreme lineages, suggesting that adaptation to Rio Tinto water (RTW) must occur rapidly. • Fitness from both the microalga Dictyosphaerium chlorelloides and the cyanobacterium Microcystis aeruginosa was inhibited when they were cultured in RTW. After further incubation for several weeks, D. chlorelloides survived, as a result of the growth of a variant that was resistant to RTW, but RTW-resistant cells did not appear in M. aeruginosa • A Luria-Delbrück fluctuation test revealed that D. chlorelloides RTW-resistant cells arose randomly by rare spontaneous mutations before the RTW exposure (1.38 × 10-6 mutants per cell division). The mutants with a diminished fitness are maintained in nonextreme waters as the result of a balance between new RTW-resistant cells arising by mutation and RTW-resistant mutants eliminated by natural selection (equilibrium at c. 15 RTW-resistant per 107 wild-type cells). • Rapid adaptation of eukaryotic algae to RTW could be the result of selection of RTW-resistant mutants occurring spontaneously in nonextreme populations that arrived fortuitously at the river in the past, or in the present continuously.
KW - Acids
KW - Adaptation, Physiological
KW - Algae
KW - Ecosystem
KW - Evolution
KW - Rivers
KW - Spain
KW - Time Factors
KW - Water
KW - Andalucia
KW - Eurasia
KW - Europe
KW - Huelva [Andalucia]
KW - Rio Tinto [(RVR) Huelva]
KW - Southern Europe
KW - Spain
KW - algae
KW - Dictyosphaerium
KW - Dictyosphaerium chlorelloides
KW - Eukaryota
KW - Luria
KW - Microcystis
KW - Microcystis aeruginosa

KW - acid
KW - water
KW - adaptation
KW - alga
KW - biomass
KW - cyanobacterium
KW - fitness
KW - natural selection
KW - adaptation
KW - alga
KW - article
KW - chemistry
KW - ecosystem
KW - evolution
KW - genetics
KW - physiology
KW - river
KW - Spain
KW - time
KW - Acidic environments
KW - Adaptation
KW - Dictyosphaerium
KW - Microcystis
KW - Rio Tinto
SN - 14698137 (ISSN)
C2 - 17587381
LA - English
J2 - New Phytol.
M3 - Article
DB - Scopus
N1 - Export Date: 23 February 2023; Cited By: 40; Correspondence Address: A. Flores-Moya; Biología Vegetal (Botánica), Facultad de Ciencias, Universidad de Málaga, E-29071 Málaga, Spain; email: floresa@uma.es; CODEN: NEPHA
ER -

TY - JOUR
AU - Brake, M.
AU - Hook, N.
TI - Darwin to the double helix: Astrobiology in fiction
PY - 2007
T2 - International Journal of Astrobiology
VL - 6
IS - 4
SP - 273
EP - 280
DO - 10.1017/S1473550407003898
UR - https://www.scopus.com/inward/record.uri?eid=2-s2.0-37549035407&doi=10.1017%2fS1473550407003898&partnerID=40&md5=4c73f7a476d7306c89fe5edd12c9c019
AD - Centre for Astronomy and Science Education, University of Glamorgan, Trefforest, 4 Forest Grove, United Kingdom
AB - From Darwin to the double helix, the last 150 years have seen revolutionary changes in our understanding of life in the Universe.

In this time, works of science fiction have provided a valuable, and often overlooked, reflection of the assumptions and attitudes held by society to such changes. This paper will consider key works of film and fiction as a commentary on emergent features of astrobiology, including the work of Fred Hoyle, Olaf Stapledon's Last and First Men (1930) and Star Maker (1937), and Arthur C. Clarke and Stanley Kubrick's cinematic classic, 2001: A Space Odyssey (1968). These popular culture texts represent a useful and inspiring communication of science. Their critical discourse is the reducible gap between the new worlds uncovered by science and the fantastic strange worlds of the imagination. As such, they exemplify a way in which the culture and science of popular astrobiology can be fused. © 2007 Cambridge University Press.
KW - culture
KW - education
KW - exobiology
KW - Communication
KW - Culture
KW - Education
SN - 14753006 (ISSN)
LA - English
J2 - Int. J. Astrobiology
M3 - Article
DB - Scopus
N1 - Export Date: 23 February 2023; Cited By: 1; Correspondence Address: M. Brake; Centre for Astronomy and Science Education, University of Glamorgan, Trefforest, 4 Forest Grove, United Kingdom; email: mbrake@glam.ac.uk
ER -

TY - JOUR
AU - Selsis, F.
AU - Chazelas, B.
AU - Bordé, P.
AU - Ollivier, M.
AU - Brachet, F.
AU - Decaudin, M.
AU - Bouchy, F.
AU - Ehrenreich, D.
AU - Grießmeier, J.-M.
AU - Lammer, H.
AU - Sotin, C.
AU - Grasset, O.
AU - Moutou, C.
AU - Barge, P.
AU - Deleuil, M.
AU - Mawet, D.
AU - Despois, D.
AU - Kasting, J.F.
AU - Léger, A.
TI - Could we identify hot ocean-planets with CoRoT, Kepler and Doppler velocimetry?
PY - 2007
T2 - Icarus

VL - 191
IS - 2
SP - 453
EP - 468
DO - 10.1016/j.icarus.2007.04.010
UR - https://www.scopus.com/inward/record.uri?eid=2-s2.0-35548985464&doi=10.1016%2fj.icarus.2007.04.010&partnerID=40&md5=91ce8c956da1649f8ab7450bac1c602c
AD - Centre de Recherche Astrophysique de Lyon (CNRS UMR 5574), Université de Lyon, Ecole Normale Supérieure de Lyon, F-69007 Lyon, 46 allée d'Italie, France
AD - Institut d'Astrophysique, Spatiale, Université Paris-Sud, CNRS (UMR 8617), F-91405 Orsay, bat 121, France
AD - Harvard-Smithsonian Center for Astrophysics, Cambridge, MA 02138, 60 Garden Street, United States
AD - Institut d'Astrophysique de Paris, CNRS (UMR 7095), Université Pierre and Marie Curie, F-75014 Paris, 98 bis, boulevard Arago, France
AD - LESIA, CNRS-Observatoire de Paris, 92195 Meudon, France
AD - Space Research Institute, Austrian Academy of Sciences, A-8042 Graz, Schmiedlstr. 6, Austria
AD - Géophysique, Université de Nantes, F-44321 Nantes Cedex 3, France
AD - Laboratoire d'Astrophysique de Marseille, LAM/OAMP, CNRS, Traverse du Siphon, 13376 Marseille Cedex 12, BP 8, France
AD - Université de Liège, 4000 Sart-Tilman, 17 allée du 6 Août, Belgium
AD - Observatoire de Bordeaux (INSU/CNRS), F-33270 Floirac, B.P. 89, France
AD - Department of Geosciences, The Pennsylvania State University, University Park, PA 16802, United States
AB - Planets less massive than about 10 MEarth are expected to have no massive H-He atmosphere and a cometary composition (~50% rocks, 50% water, by mass) provided they formed beyond the snowline of protoplanetary disks. Due to inward migration, such planets could be found at any distance between their formation site and the star. If migration stops within the habitable zone, this may produce a new kind of planets, called ocean-planets. Ocean-planets typically consist in a silicate core, surrounded by a thick ice mantle, itself covered by a 100 km-deep ocean. The possible existence of ocean-planets raises important astrobiological questions: Can life originate on such body, in the absence of continent and ocean-silicate interfaces? What would be the nature of the atmosphere and the geochemical cycles? In this work, we address the fate of hot ocean-planets produced when migration ends at a closer distance. In this case the liquid/gas interface can disappear, and the hot H2O envelope is made of a supercritical fluid. Although we do not expect these bodies to harbor life, their detection and identification as water-rich planets would give us insight as to the abundance of hot and, by extrapolation, cool ocean-planets. The water reservoir of these planets seems to be weakly affected by gravitational escape, provided that they are located beyond some minimum distance, e.g. 0.04 AU for a 5-Earth-mass planet around a Sun-like star. The swelling of their water atmospheres by the high stellar flux is

expected not to significantly increase the planets' radii. We have studied the possibility of detecting and characterizing these hot ocean-planets by measuring their mean densities using transit missions in space-CoRoT (CNES) and Kepler (NASA)-in combination with Doppler velocimetry from the ground-HARPS (ESO) and possible future instruments. We have determined the domain in the [stellar magnitude, orbital distance] plane where discrimination between ocean-planets and rocky planets is possible with these instruments. The brightest stars of the mission target lists and the planets closest to their stars are the most favorable cases. Full advantage of high precision photometry by CoRoT, and particularly Kepler, can be obtained only if a new generation of Doppler instruments is built. © 2007 Elsevier Inc. All rights reserved.
KW - Extrasolar planets
KW - Ices
KW - migration
KW - Planets
PB - Academic Press Inc.
SN - 00191035 (ISSN)
LA - English
J2 - Icarus
M3 - Article
DB - Scopus
N1 - Export Date: 23 February 2023; Cited By: 70; Correspondence Address: A. Léger; Institut d'Astrophysique, Spatiale, Université Paris-Sud, CNRS (UMR 8617), F-91405 Orsay, bat 121, France; email: alain.leger@ias.u-psud.fr; CODEN: ICRSA
ER -

TY - JOUR
AU - Raymond, S.N.
AU - Scalo, J.
AU - Meadows, V.S.
TI - A decreased probability of habitable planet formation around low-mass stars
PY - 2007
T2 - Astrophysical Journal
VL - 669
IS - 1
SP - 606
EP - 614
DO - 10.1086/521587
UR - https://www.scopus.com/inward/record.uri?eid=2-s2.0-38849183160&doi=10.1086%2f521587&partnerID=40&md5=83a1a9a06ecb9d3f233879baa9485c40
AD - Center for Astrophysics and Space Astronomy, University of Colorado, 389 UCB, Boulder, CO 80309-0389, United States
AD - NASA Postdoctoral Program
AD - NASA Astrobiology Institute
AD - Department of Astronomy, University of Texas, Austin, TX, United States
AD - Infrared Processing and Analysis Center, California Institute of Technology, Pasadena, CA, United States
AB - Smaller terrestrial planets (≲0.3⊕) are less likely to retain

the substantial atmospheres and ongoing tectonic activity probably required to support life. A key element in determining whether sufficiently massive sustainably habitable planets can form is the availability of solid planet-forming material. We use dynamical simulations of terrestrial planet formation from planetary embryos and simple scaling arguments to explore the implications of correlations between terrestrial planet mass, disk mass, and the mass of the parent star. We assume that the protoplanetary disk mass scales with stellar mass as Mdisk αfM∗h, where f measures the relative disk mass and 1/2 < h < 2, so that disk mass decreases with decreasing stellar mass. We consider systems without Jovian planets, based on current models and observations for M stars. We assume the mass of a planet formed in some annulus of a disk with given parameters is proportional to the disk mass in that annulus and show with a suite of simulations of late-stage accretion that the adopted prescription is surprisingly accurate. Our results suggest that the fraction of systems with sufficient disk mass to form >0.3 M⊕ habitable planets decreases for low-mass stars for every realistic combination of parameters. This habitable fraction is small for stellar masses below a mass in the interval 0.5-0.8 M⊕, depending on disk parameters, an interval that excludes most M stars. Radial mixing and therefore water delivery are inefficient in the lower mass disks commonly found around low-mass stars, such that terrestrial planets in the habitable zones of most low-mass stars are likely to be small and dry. © 2007. The American Astronomical Society. All rights reserved.
KW - Astrobiology
KW - Planetary systems: formation
KW - Planetary systems: protoplanetary disks
KW - Stars: low-mass, brown dwarfs
PB - Institute of Physics Publishing
SN - 0004637X (ISSN)
LA - English
J2 - Astrophys. J.
M3 - Article
DB - Scopus
N1 - Export Date: 23 February 2023; Cited By: 137; Correspondence Address: S.N. Raymond; Center for Astrophysics and Space Astronomy, University of Colorado, 389 UCB, Boulder, CO 80309-0389, United States; email: raymond@lasp.colorado.edu
ER -

TY - JOUR
AU - Hand, K.P.
AU - Chyba, C.F.
TI - Empirical constraints on the salinity of the europan ocean and implications for a thin ice shell
PY - 2007
T2 - Icarus
VL - 189
IS - 2
SP - 424
EP - 438
DO - 10.1016/j.icarus.2007.02.002

UR - https://www.scopus.com/inward/record.uri?eid=2-s2.0-34447278812&doi=10.1016%2fj.icarus.2007.02.002&partnerID=40&md5=5954439c33805e24fce8633972e3697f
AD - Department of Geological and Environmental Sciences, Stanford University, Stanford, CA 94305, United States
AD - Carl Sagan Center for the Study of Life in the Universe, SETI Institute, Mountain View, CA 94043, 515 N. Whisman Road, United States
AD - Department of Astrophysical Sciences, Princeton University, Princeton, NJ 08544, Peyton Hall, United States
AB - Induced electrical currents within Europa inferred from Galileo spacecraft magnetometer instrument data have been interpreted as due to a salty europan ocean. Published compositional models for Europa's ocean, based on aqueous leaching of carbonaceous chondrites, range over five orders of magnitude in predicted magnesium sulfate concentrations. We combine the Galileo spacecraft magnetometer-derived oceanic conductivities and radio Doppler data-derived interior models with laboratory conductivity vs concentration data for both magnesium sulfate solutions and terrestrial seawater to determine empirically the range of salt concentrations permitted for Europa's ocean. Solutions for both a three-layer spherical model, and a five-layer half-space model, that satisfy current preferred best fits to magnetometer data imply high, near-saturation salt concentrations and require a europan ice shell of less than 15 km thick, with a best fit at 4 km ice thickness. Adding a conductive core and mantle has a negligible effect on the amplitude when ocean conductivities are greater than a few Siemens per meter. Similarly, we find that including a realistic ionosphere has a negligible effect. We examine the implications of these results for the subsurface habitability of Europa. © 2007 Elsevier Inc. All rights reserved.
KW - Astrobiology
KW - composition
KW - Europa
KW - Jupiter
KW - magnetosphere
KW - Satellites
SN - 10902643 (ISSN)
LA - English
J2 - Icarus
M3 - Article
DB - Scopus
N1 - Export Date: 23 February 2023; Cited By: 99; Correspondence Address: K.P. Hand; Department of Geological and Environmental Sciences, Stanford University, Stanford, CA 94305, United States; email: khand@stanford.edu; CODEN: ICRSA
ER -

TY - JOUR
AU - Tajika, E.
TI - Long-term stability of climate and global glaciations throughout the evolution of the Earth
PY - 2007
T2 - Earth, Planets and Space

VL - 59
IS - 4
SP - 293
EP - 299
DO - 10.1186/BF03353107
UR - https://www.scopus.com/inward/record.uri?eid=2-s2.0-34347366414&doi=10.1186%2fBF03353107&partnerID=40&md5=62b94e4adecc2eeae3a1fae3918734f2
AD - Department of Earth and Planetary Science, Graduate School of Science, University of Tokyo, Tokyo, Japan
AB - Earth's climate is considered to be stable on the order of > 106 years, owing to a negative feedback mechanism in a carbon cycle system. However, any decrease in net input flux of CO2 to the atmosphere-ocean system (i.e., volcanic-metamorphic CO2 flux minus excess organic carbon burial flux) lowers the surface temperature and would eventually initiate global glaciation. The FD-FBO diagram (FD: the total CO2 degassing flux, FBO: the organic carbon burial flux) is proposed as a measure of the susceptibility of the Earth to global glaciations. By using this diagram with the carbon fluxes estimated from a carbon cycle model during the Phanerozoic, the net input flux of CO2 is found to have been very close to the critical condition for a global glaciation at the Late Paleozoic. During the Proterozoic, a carbon isotope mass balance model with this diagram shows that global glaciations occurred probably due to a decrease in the CO2 degassing in addition to an increase in the organic carbon burial. Because the Sun becomes brighter as it evolves, the critical level of atmospheric CO2 pressure to cause global glaciation will be lower than the critical CO2 pressure for photosynthesis of C4 plants within 500 million years. At this point, the net input flux of CO2 will be too large to cause global glaciations. Continuous volcanic-metamorphic activities (i.e., plate tectonics) may be one of the necessary conditions for the Earth and Earth-like planets in extrasolar planetary systems to keep liquid water and life over the timescales of planetary evolution. Copyright © The Society of Geomagnetism and Earth, Planetary and Space Sciences (SGEPSS); The Seismological Society of Japan; The Volcanological Society of Japan; The Geodetic Society of Japan; The Japanese Society for Planetary Sciences; TERRAPUB.
KW - atmosphere-ocean system
KW - carbon cycle
KW - carbon isotope
KW - Earth
KW - glaciation
KW - mass balance
KW - paleoclimate
KW - Paleozoic
KW - photosynthesis
KW - planetary evolution
KW - Proterozoic
KW - Astrobiology
KW - Carbon cycle
KW - Global glaciation
KW - Snowball Earth
PB - Springer Berlin

SN - 13438832 (ISSN)
LA - English
J2 - Earth Planets Space
M3 - Article
DB - Scopus
N1 - Export Date: 23 February 2023; Cited By: 22; Correspondence Address: E. Tajika; Department of Earth and Planetary Science, Graduate School of Science, University of Tokyo, Tokyo, Japan; email: tajika@eps.s.u-tokyo.ac.jp
ER -

TY - JOUR
AU - Walker, J.J.
AU - Pace, N.R.
TI - Endolithic microbial ecosystems
PY - 2007
T2 - Annual Review of Microbiology
VL - 61
SP - 331
EP - 347
DO - 10.1146/annurev.micro.61.080706.093302
UR - https://www.scopus.com/inward/record.uri?eid=2-s2.0-35848962742&doi=10.1146%2fannurev.micro.61.080706.093302&partnerID=40&md5=438a6d03c08b8bfdb3d76bd60ed297af
AD - University of Colorado at Boulder, Department of Molecular, Cellular and Developmental Biology, Boulder, CO 80309-0347, United States
AB - The endolithic environment, the pore space in rocks, is a ubiquitous microbial habitat and an interface between biology and geology. Photosynthesis-based endolithic communities inhabit the outer centimeters of rocks exposed to the surface, and offer model systems for microbial ecology, geobiology, and astrobiology. Endolithic ecosystems are among the simplest microbial ecosystems known and as such provide tractable models for testing ecological hypotheses. Such hypotheses have been difficult to test because microbial ecosystems are extraordinarily diverse. We review here recent culture-independent, ribosomal RNA-based studies that evaluate hypotheses about endolithic ecosystems, and provide insight for understanding general principles in microbial ecology. Comparison of endolithic communities supports the principle that patterns of microbial diversity are governed by similar principles observed in macroecological systems. Recent results also explore geobiological processes that shape the current biosphere and potentially provide clues to life's history on Earth and where to seek life elsewhere in the Solar System. Copyright © 2007 by Annual Reviews. All rights reserved.
KW - Archaea
KW - Bacteria
KW - Cyanobacteria
KW - Ecology
KW - Ecosystem
KW - Fungi
KW - Geology
KW - Light

KW - Phylogeny
KW - Temperature
KW - ribosome RNA
KW - water
KW - bacterial colonization
KW - bacterial flora
KW - bacterial metabolism
KW - biogeography
KW - ecology
KW - ecosystem
KW - environment
KW - geology
KW - light
KW - microbial diversity
KW - molecular ecology
KW - nonhuman
KW - nutrient
KW - phylogeny
KW - priority journal
KW - review
KW - temperature
KW - Endolith
KW - Geobiology
KW - Microbial diversity
KW - Microbial ecology
KW - rRNA
SN - 00664227 (ISSN)
C2 - 17506683
LA - English
J2 - Annu. Rev. Microbiol.
M3 - Review
DB - Scopus
N1 - Export Date: 23 February 2023; Cited By: 115; Correspondence Address: J.J. Walker; University of Colorado at Boulder, Department of Molecular, Cellular and Developmental Biology, Boulder, CO 80309-0347, United States; email: jeffrey.walker@colorado.edu; CODEN: ARMIA
ER -

TY - CONF
AU - Fink, W.
AU - Dohm, J.M.
AU - Tarbell, M.A.
AU - Hare, T.M.
AU - Baker, V.R.
AU - Schulze-Makuch, D.
AU - Furfaro, R.
AU - Fairén, A.G.
AU - Ferré, T.P.A.
AU - Miyamoto, H.
AU - Komatsu, G.
AU - Mahaney, W.C.
TI - Tier-scalable reconnaissance missions for the autonomous exploration of planetary bodies

PY - 2007
T2 - IEEE Aerospace Conference Proceedings
C7 - 4161289
DO - 10.1109/AERO.2007.352715
UR - https://www.scopus.com/inward/record.uri?eid=2-s2.0-34548739634&doi=10.1109%2fAERO.2007.352715&partnerID=40&md5=4fdfa5080760ef68ff836bfeede01484
AD - Visual and Autonomous Exploration Systems Research Laboratory, Division of Physics, Mathematics and Astronomy, California Institute of Technology, Pasadena, CA 91125, United States
AD - Dept. of Hydrology and Water Resources, University of Arizona, Tucson, AZ, United States
AD - Lunar and Planetary Laboratory, University of Arizona, Tucson, AZ, United States
AD - United States Geologic Survey, Flagstaff, AZ, United States
AD - Dept. of Geology, Washington State University, Pullman, WA, United States
AD - Aerospace and Mechanical Engineering Dept., University of Arizona, Tucson, AZ, United States
AD - Centro de Biología Molecular, Universidad Autónoma de Madrid, Madrid, Spain
AD - Department of Geosystem Engineering, University of Tokyo, Tokyo, Japan
AD - International Research School of Planetary Sciences, Università d'Annunzio, Pescara, Italy
AD - Geomorphology and Pedology Laboratory, York University, Canada
AB - A fundamentally new (scientific) reconnaissance mission concept, termed tier-scalable reconnaissance, for remote planetary (including Earth) atmospheric, surface and subsurface exploration recently has been devised [1-5] that soon will replace the engineering and safety constrained mission designs of the past, allowing for optimal acquisition of geologic, paleohydrologic, paleoclimatic, and possible astrobiologic information of Venus, Mars, Europa, Ganymede, Titan, Enceladus, Triton, and other extraterrestrial targets [6, 7]. This paradigm is equally applicable to potentially hazardous or inaccessible operational areas on Earth such as those related to military or terrorist activities, or areas that have been exposed to biochemical agents, radiation, or natural disasters. Traditional missions have performed local, ground-level reconnaissance through rovers and immobile landers, or global mapping performed by an orbiter. The former is safety and engineering constrained, affording limited detailed reconnaissance of a single site at the expense of a regional understanding, while the latter returns immense datasets, often overlooking detailed information of local and regional significance. © 2007 IEEE.
KW - Astrobiology
KW - Autonomous agents
KW - Planetary landers
KW - Planetary surface analysis
KW - Remote sensing
KW - Safety factor
KW - Datasets
KW - Planetary bodies
KW - Reconnaissance missions

KW - Subsurface exploration
KW - Space research
SN - 1095323X (ISSN); 1424405254 (ISBN); 978-142440525-1 (ISBN)
LA - English
J2 - IEEE Aerosp. Conf. Proc.
M3 - Conference paper
DB - Scopus
N1 - Export Date: 23 February 2023; Cited By: 37; Correspondence Address: W. Fink; Visual and Autonomous Exploration Systems Research Laboratory, Division of Physics, Mathematics and Astronomy, California Institute of Technology, Pasadena, CA 91125, United States; email: wfink@autonomy.caltech.edu
ER -

TY - JOUR
AU - Abe, S.
AU - Ebizuka, N.
AU - Yano, H.
AU - Watanabe, J.-i.
AU - Borovička, J.
TI - Search for OH(A-X) and detection of N
2 + (B-X) in
ultraviolet meteor spectrum
PY - 2007
T2 - Advances in Space Research
VL - 39
IS - 4
SP - 538
EP - 543
DO - 10.1016/j.asr.2007.03.051
UR - https://www.scopus.com/inward/record.uri?eid=2-s2.0-34248229738&doi=10.1016%2fj.asr.2007.03.051&partnerID=40&md5=37af595a35f9987d15c55ae89d81e6bd
AD - Department of Earth and Planetary Sciences, Faculty of Science, Kobe University, Nada, Kobe, 657-8501, Japan
AD - Department of Physics, Konan University, RIKEN, Kobe, 658-8501, Higashi-Nada, Japan
AD - Institute of Space and Astronautical Science, JAXA, Sagamihara, Kanagawa, 229-8510, 3-1-1 Yoshinodai, Japan
AD - National Astronomical Observatory of Japan, National Institutes of Natural Sciences, NINS, Mitaka, Tokyo, 181-8588, 2-21-1 Osawa, Japan
AD - Astronomical Institute, the Academy of Sciences, Ondrejov, 25165, 298 Fricova, Czech Republic
AB - An ultraviolet-visible spectrum between 300 and 450 nm of a cometary meteoroid originated from 55P/Tempel-Tuttle was investigated. The spectroscopy was carried out an intensified high definition TV camera with a slit-less reflection grating during the 2001 Leonid meteor shower over Japan. A best fit calculation mixed with atoms and molecules confirmed the first discovery of N 2
+ B 2
Σ u +
→ X 2 Σ

g + bands in the UV meteor spectrum. N_2^+ temperature was estimated to 10,000 K with a low number density of 1.55 × 10^5 cm^{-3}. We also discuss the possibility that enhanced emissions in a meteor and a train around 310 nm are caused by the band head of OH $A^2Σ^+ → X^2Π$. Since cometary dust may have contributed organics and water to the Earth from its early period until now, OH A–X (0, 0) must be investigated. © 2007.
KW - Astrobiology
KW - Atomic physics
KW - Spectroscopic analysis
KW - Spectrum analysis
KW - Comets: individual(55P/Tempel–Tuttle)
KW - Interplanetary medium
KW - Meteors
KW - Molecular processes
KW - Meteorites
KW - Astrobiology
KW - Comets: individual(55P/Tempel–Tuttle)
KW - Interplanetary medium
KW - Meteoroids
KW - Meteors
KW - Molecular processes
PB - Elsevier Ltd
SN - 02731177 (ISSN)
LA - English
J2 - Adv. Space Res.
M3 - Article
DB - Scopus
N1 - Export Date: 23 February 2023; Cited By: 7; Correspondence Address: S. Abe; Department of Earth and Planetary Sciences, Faculty of Science, Kobe University, Nada, Kobe, 657-8501, Japan; email: avell@kobe-u.ac.jp; CODEN: ASRSD
ER -

TY - CHAP
AU - Sagan, C.
TI - A Light in the Darkness
PY - 2007
T2 - Oracles of Science: Celebrity Scientists versus God and Religion
DO - 10.1093/acprof:oso/9780195310726.003.0004
UR - https://www.scopus.com/inward/record.uri?eid=2-s2.0-84921670722&doi=10.1093%2facprof%3aoso%2f9780195310726.003.0004&partnerID=40&md5=26289330528df706e2119e313ca81683
AB - Carl Sagan is a public intellectual and the best-selling author of Cosmos, The Demon-Haunted World: Science as a Candle in the Dark, The Dragons of Eden: Speculations on the Evolution of Human Intelligence, and many other books. His science fiction novel, Contact, was made into a popular, major motion picture in 1997.

Sagan is well known for his interests in extraterrestrial life and is closely linked to the SETI (Search for Extra Terrestrial Intelligence). As a scientist, Sagan educated the public about "Nuclear Winter", the idea that a nuclear war could precipitate an unprecedented ice age that might render the Earth largely uninhabitable. Sagan became notorious in certain circles for his forays into religion, which he viewed with skepticism. © 2007 by Oxford University Press, Inc. All rights reserved.
KW - Contact
KW - Cosmos
KW - Extra-terrestrial life
KW - Nuclear winter
KW - Public intellectual
KW - SETI
KW - Skepticism
KW - The demon-haunted world
KW - The dragons of eden
PB - Oxford University Press
SN - 978-019978517-9 (ISBN); 978-019531072-6 (ISBN)
LA - English
J2 - Oracles of Sci.: Celebr. Sci. versus God and Relig.
M3 - Book chapter
DB - Scopus
N1 - Export Date: 23 February 2023; Cited By: 0
ER -

TY - CONF
AU - Balaban, E.
AU - Narasimhan, S.
AU - Cannon, H.N.
AU - Brownston, L.S.
TI - Model-based fault detection and diagnosis system for NASA mars subsurface drill prototype
PY - 2007
T2 - IEEE Aerospace Conference Proceedings
C7 - 4161685
DO - 10.1109/AERO.2007.352947
UR - https://www.scopus.com/inward/record.uri?eid=2-s2.0-34548712317&doi=10.1109%2fAERO.2007.352947&partnerID=40&md5=d8e2870e617be4f7aa4476d6686f7eb5
AD - NASA Ames Research Center, QSS, Moffett Field, CA 94035
AD - NASA Ames Research Center, USRA, Moffett Field, CA 94035
AD - NASA Ames Research Center, Moffett Field, CA 94035
AB - The Drilling Automation for Mars Environment (DAME) project, led by NASA Ames Research Center, is aimed at developing a lightweight, low-power drill prototype that can be mounted on a Mars lander and be capable of drilling down several meters below the Mars surface for conducting geology and astrobiology research. The DAME drill system incorporates a large degree of autonomy - from quick diagnosis of system state and fault conditions to taking the appropriate recovery actions - while also striving to achieve as many of the operational objectives as possible. This paper outlines, on a general level, the overall DAME architecture, equipment, and autonomy package. The main focus, however, is on describing the

model-based fault detection and diagnosis system, including the modeling approach, the fault modes handled, and the diagnostic algorithms. The results of the latest field tests, conducted in 2006 in Haughton Crater on Devon Island (a Mars analogue site in Canadian Arctic), are also discussed. ©2007 IEEE.
KW - Algorithms
KW - Astrobiology
KW - Fault detection
KW - Geology
KW - Mathematical models
KW - NASA
KW - Autonomy package
KW - Diagnosis systems
KW - Mars landers
KW - Drills
SN - 1095323X (ISSN); 1424405254 (ISBN); 978-142440525-1 (ISBN)
LA - English
J2 - IEEE Aerosp. Conf. Proc.
M3 - Conference paper
DB - Scopus
N1 - Export Date: 23 February 2023; Cited By: 6; Correspondence Address: E. Balaban; NASA Ames Research Center, QSS, Moffett Field, CA 94035; email: balaban@email.arc.nasa.gov
ER -

TY - JOUR
AU - Glikson, A.
AU - Vickers, J.
TI - Asteroid mega-impacts and Precambrian banded iron formations: 2.63 Ga and 2.56 Ga impact ejecta/fallout at the base of BIF/argillite units, Hamersley Basin, Pilbara Craton, Western Australia
PY - 2007
T2 - Earth and Planetary Science Letters
VL - 254
IS - 1-2
SP - 214
EP - 226
DO - 10.1016/j.epsl.2006.11.037
UR - https://www.scopus.com/inward/record.uri?eid=2-s2.0-33846336128&doi=10.1016%2fj.epsl.2006.11.037&partnerID=40&md5=7f7167dbc783a9d853072cf46a2d068e
AD - Department of Earth and Marine Science, the Planetary Research Institute, Australian National University, Canberra, A.C.T. 0200, Australia
AB - The temporal association between late Archaean to earliest Proterozoic asteroid impact ejecta/fallout units and overlying banded iron formations suggests that, in some instances, these impacts were closely followed by significant transformation in the nature of source terrains of the sediments. The Jeerinah Impact Layer (JIL) [B.M. Simonson, D. Davies, S.W. Hassler, Discovery of a layer of probable impact melt spherules in the late Archean Jeerinah Formation, Fortescue Group, Western Australia. Aust. J. Earth Sci. 47 (2000) 315–325; B.M. Simonson, S.W. Hassler, Revised correlations in the early Precambrian Hamersley Basin based on a horizon of

resedimented impact spherules. Aust. J. Earth Sci. 44 (1997) 37–48; B.M. Simonson, B.P. Glass, Spherule layers – records of ancient impacts. Ann. Rev. Earth Planet. Sci. 32 (2004) 329–361; A.Y. Glikson, Early Precambrian asteroid impact-triggered tsunami: excavated seabed, debris flows, exotic boulders, and turbulence features associated with 3.47–2.47 Ga-old asteroid impact fallout units, Pilbara Craton, Western Australia. Astrobiology 4 (2001) 19–50; S.W. Hassler, B.M. Simonson, D.Y. Sumner, D. Murphy, Neoarchaean impact spherule layers in the Fortescue and Hamersley Groups, Western Australia: stratigraphic and depositional implications of re-correlation. Aust. J. Earth Sci. 52 (2005) 759–772; B. Rasmussen, C. Koeberl, Iridium anomalies and shocked quartz in a late Archean spherule layer from the Pilbara Craton: new evidence for a major asteroid impact at 2.63 Ga. Geology 32 (2004) 1029–1032; B. Rasmussen, T.S. Blake, I.R. Fletcher, U–Pb zircon age constraints on the Hamersley spherule beds: Evidence for a single 2.63 Ga Jeerinah-Carawine impact ejecta layer. Geology, 33 (2005) 725–728.] overlies an argillite-dominated unit (Jeerinah Formation, 2684 ± 6 Ma [A.F. Trendall, W. Compston, D.R. Nelson, J.R. deLaeter, V.C. Bennett, SHRIMP zircon ages constraining the depositional chronology of the Hamersley Group, Western Australia. Aust. J. Earth Sci. 51 (2004) 621–644.]) and lies directly below a thin volcanic tuff (2629 ± 5 Ma, [A.F. Trendall, W. Compston, D.R. Nelson, J.R. deLaeter, V.C. Bennett, SHRIMP zircon ages constraining the depositional chronology of the Hamersley Group, Western Australia. Aust. J. Earth Sci. 51 (2004) 621–644.]) and banded iron formation (BIF) (upper part of Marra Mamba Iron Formation, 2597 ± 5 Ma [A.F. Trendall, W. Compston, D.R. Nelson, J.R. deLaeter, V.C. Bennett, SHRIMP zircon ages constraining the depositional chronology of the Hamersley Group, Western Australia. Aust. J. Earth Sci. 51 (2004) 621–644.]). The Spherule Marker Bed (SMB) [B.M. Simonson, Geological evidence for an early Precambrian microtektite strewn field in the Hamersley Basin of Western Australia. Geol. Soc. Am. Bull. 104 (1992) 829–839; B.M. Simonson, S.W. Hassler, K.A. Schubel, Lithology and proposed revisions in stratigraphic nomenclature of the Wittenoom Formation (Dolomite) and overlying formations, Hamersley Group, Western Australia. Geol. Surv. W. Aust. Rep. 345 (1993) 65–79; S.W. Hassler, B.M. Simonson, D.Y. Sumner, D. Murphy, Neoarchaean impact spherule layers in the Fortescue and Hamersley Groups, Western Australia: stratigraphic and depositional implications of re-correlation. Aust. J. Earth Sci. 52 (2005) 759–772. [5]], which includes two impact cycles [A.Y. Glikson, Early Precambrian asteroid impact-triggered tsunami: excavated seabed, debris flows, exotic boulders, and turbulence features associated with 3.47–2.47 Ga-old asteroid impact fallout units, Pilbara Craton, Western Australia. Astrobiology 4 (2001) 19–50.], is located at the top of a carbonate/calcareous siltstone-dominated sequence (Bee Gorge Member, Wittenoom Formation, 2565 ± 9 Ma [A.F. Trendall, W. Compston, D.R. Nelson, J.R. deLaeter, V.C. Bennett, SHRIMP zircon ages constraining the depositional chronology of the Hamersley Group, Western Australia. Aust. J. Earth Sci. 51 (2004) 621–644.]) and below a carbonate-poor siltstone-chert-BIF sequence (Mount Sylvia Formation, Bruno's Band [BIF], Mount McRae Shale, 2504 ± 5 Ma [B. Rasmussen, T.S. Blake, I.R. Fletcher, U–Pb zircon age

constraints on the Hamersley spherule beds: Evidence for a single 2.63 Ga Jeerinah-Carawine impact ejecta layer. Geology, 33 (2005) 725-728.]). No ferruginous sediments overlie impact layers hosted by stromatolitic carbonates (< 2.63 Ga microkrystite spherule-bearing Carawine mega-breccia, east Hamersley Basin; ~ 2.6-2.65 Ga Monteville impact layer; 2567 Ma Reivilo Formation, west Griqualand Basin, Transvaal) - a lack possibly attributable to enclosed oxygenated high-pH reef environment. Barring a possible presence of undocumented hiatuses between the impact layers and directly overlying units, and within the accuracy limits of U-Pb zircon age data, it follows that the JIL and SMB mega-impacts were succeeded by enhanced supply of ferruginous and clastic materials. The location of 5 out of 8 Archaean to earliest Proterozoic impact fallout/ejecta units below iron-rich sediments [A.Y. Glikson, Asteroid impact ejecta units overlain by iron-rich sediments in 3.5-2.4 Ga terrains, Pilbara and Kaapvaal cratons: Accidental or cause-effect relationships? Earth Planet. Sci. Lett. 246 (2006) 149-160.], including 3.47 Ga, 3.26 Ga, 3.24 Ga, 2.63 Ga and 2.56 Ga units, unless accidental, suggests enrichment of sea water in soluble ferrous iron, possibly derived from impact-triggered mafic volcanic and hydrothermal activity. The scarcity of shocked quartz grains in the ejecta suggests impacts occurred in oceanic regions of the late Archaean Earth [A.Y. Glikson, Early Precambrian asteroid impact-triggered tsunami: excavated seabed, debris flows, exotic boulders, and turbulence features associated with 3.47-2.47 Ga-old asteroid impact fallout units, Pilbara Craton, Western Australia. Astrobiology 4 (2001) 19-50; A.Y. Glikson, Oceanic mega-impacts and crustal evolution, Geology 27 (1999) 341-387; B.M. Simonson, D. Davies, M. Wallace, S. Reeves, S.W. Hassler, Iridium anomaly but no shocked quartz from late Archean microkrystite layer: oceanic impact ejecta? Geology 26 (1998) 195-198.]. Should further examples of sedimentary facies changes associated with large impact events be identified, the impact factor will need to be taken into account in accounting for the crustal transformations during the transition from the end-Archaean to the earliest Proterozoic. © 2006 Elsevier B.V. All rights reserved.
KW - Australasia
KW - Australia
KW - Hamersley Basin
KW - Pilbara Block
KW - Western Australia
KW - Apoidea
KW - Crocodylus niloticus
KW - Spherules
KW - Sylvia
KW - Geochronology
KW - Iron
KW - Petrology
KW - Sedimentary rocks
KW - Stratigraphy
KW - Zircon
KW - Asteroid impact
KW - Australia
KW - Banded iron formation

KW - Jeerinah Impact Layer
KW - asteroid
KW - banded iron formation
KW - impact structure
KW - Precambrian
KW - Planetary surface analysis
KW - asteroid impact
KW - banded iron formation
KW - Hamersley
KW - Pilbara
KW - Western Australia
SN - 0012821X (ISSN)
LA - English
J2 - Earth Plan. Sci. Lett.
M3 - Article
DB - Scopus
N1 - Export Date: 23 February 2023; Cited By: 13; Correspondence Address: A. Glikson; Department of Earth and Marine Science, the Planetary Research Institute, Australian National University, Canberra, A.C.T. 0200, Australia; email: Andrew.glikson@anu.edu.au
ER -

TY - JOUR
AU - Raymond, S.N.
TI - Terrestrial planet formation in extra-solar planetary systems
PY - 2007
T2 - Proceedings of the International Astronomical Union
VL - 3
IS - S249
SP - 233
EP - 250
DO - 10.1017/S1743921308016645
UR - https://www.scopus.com/inward/record.uri?eid=2-s2.0-44249099059&doi=10.1017%2fS1743921308016645&partnerID=40&md5=388e8a8ae5a4758e9f4a95996f1e70c5
AD - NASA
AD - Center for Astrophysics and Space Astronomy, Center for Astrobiology, University of Colorado, Boulder, CO, 80309-0389, United States
AB - Terrestrial planets form in a series of dynamical steps from the solid component of circumstellar disks. First, km-sized planetesimals form likely via a combination of sticky collisions, turbulent concentration of solids, and gravitational collapse from micron-sized dust grains in the thin disk midplane. Second, planetesimals coalesce to form Moon- to Mars-sized protoplanets, also called planetary embryos. Finally, full-sized terrestrial planets accrete from protoplanets and planetesimals. This final stage of accretion lasts about 10-100 Myr and is strongly affected by gravitational perturbations from any gas giant planets, which are constrained to form more quickly, during the 1-10 Myr lifetime of the gaseous component of the disk. It is during this final stage that the bulk compositions and volatile (e.g., water) contents of terrestrial planets are set, depending on their feeding zones and the amount of radial mixing that occurs. The main factors that

influence terrestrial planet formation are the mass and surface density profile of the disk, and the perturbations from giant planets and binary companions if they exist. Simple accretion models predicts that low-mass stars should form small, dry planets in their habitable zones. The migration of a giant planet through a disk of rocky bodies does not completely impede terrestrial planet growth. Rather, hot Jupiter systems are likely to also contain exterior, very water-rich Earth-like planets, and also hot Earths, very close-in rocky planets. Roughly one third of the known systems of extra-solar (giant) planets could allow a terrestrial planet to form in the habitable zone. © 2008 International Astronomical Union.
KW - Astrobiology
KW - Methods: n-body simulations
KW - Planetary systems: formation
KW - Planetary systems: protoplanetary disks
KW - Solar system: formation
KW - Stars: late-type
SN - 17439221 (ISSN)
LA - English
J2 - Proc. Int. Astron. Union
M3 - Article
DB - Scopus
N1 - Export Date: 23 February 2023; Cited By: 3; Correspondence Address: S. N. Raymond; Center for Astrophysics and Space Astronomy, Center for Astrobiology, University of Colorado, Boulder, CO, 80309-0389, United States; email: raymond@lasp.colorado.edu
ER -

TY - CHAP
AU - Martinez-Frias, J.
AU - Amaral, G.
AU - Vázquez, L.
TI - Astrobiological significance of minerals on Mars surface environment
PY - 2007
T2 - Life in Extreme Environments
VL - 9781402062858
SP - 55
EP - 67
DO - 10.1007/978-1-4020-6285-8_4
UR - https://www.scopus.com/inward/record.uri?eid=2-s2.0-84919631810&doi=10.1007%2f978-1-4020-6285-8_4&partnerID=40&md5=4d60e41c42c6278d25a72591c09b2d36
AD - Centro de Astrobiología (CSIC-INTA), Torrejón de Ardoz, Madrid, Spain
AD - Departamento de Química Física I, Facultad de Ciencias Químicas, Universidad Complutense, Madrid, Spain
AD - Departamento de Matemática Aplicada, Facultad de Informática, Universidad Complutense de Madrid, Madrid, Spain
AB - Despite the large amount of geomorphological, geodynamic and geophysical data obtained from Mars missions, much is still unknown about Martian mineralogy and paragenetic assemblages, which is fundamental to an understanding of its entire geological history. Minerals are not only indicators of the physical-chemical settings

of the different environments and their later changes, but also they could (and do) play a crucial astrobiological role related with the possibility of existence of extinct or extant Martian life. This paper aims: (1) to present a synoptic review of the main water-related Martian minerals (mainly jarosite and other sulfates) discovered up to the present time; (2) to emphasize their significance as environmental geomarkers, on the basis of their geological settings and mineral parageneses on earth (in particular in the context of some selected terrestrial analogues), and (3) to show that their differential UV shielding properties, against the hostile environmental conditions of the Martian surface, are of a great importance for the search for extraterrestrial life. © 2007 Springer. All Rights Reserved.
KW - Astrobiology
KW - Extreme environment
KW - Gypsum
KW - Jarosite
KW - Mars minerals
KW - Sulfates
KW - UV radiation
PB - Springer Netherlands
SN - 978-140206285-8 (ISBN); 978-140206284-1 (ISBN)
LA - English
J2 - Life in Extrem. Environ.
M3 - Book chapter
DB - Scopus
N1 - Export Date: 23 February 2023; Cited By: 2
ER -

TY - CONF
AU - Gallardo, V.A.
AU - Espinoza, C.
TI - Large multicellular filamentous bacteria under the oxygen minimum zone of the eastern South Pacific: A forgotten biosphere
PY - 2007
T2 - Proceedings of SPIE - The International Society for Optical Engineering
VL - 6694
C7 - 66941H
DO - 10.1117/12.782209
UR - https://www.scopus.com/inward/record.uri?eid=2-s2.0-42149116384&doi=10.1117%2f12.782209&partnerID=40&md5=87ef3fef1dcd789c7ab5b9ecb06a481e
AD - Laboratorio de Bentos, Departamento de Oceanografía, Universidad de Concepción, Concepción, Chile
AB - In the soft reduced sediments of the continental shelf, below the oxygen minimum, zone (OMZ) of the eastern South Pacific (ESP), peculiar microbial communities have been disclosed which include a variety of large prokaryotes, protists and small metazoans. Dominant among the prokaryotes are large multi-cellular filamentous bacteria which, according to their size range, are roughly divided into megabacteria and macrobacteria. The former group is made up of a few species of Gamma Proteobacteria of the genera Thioploca and Beggiatoa and the second group includes a diversity of phenotypes.

Protists include ciliates, flagellates, and foraminifers and the metazoans are mostly nematodes and small polychaetes. A significant similarity has been found in the exploitation of the area/volume relationship among these large bacteria and their fossil analog forms as described from pre-Cambrian rocks. For the same reason, the latter have mostly been referred to as algae or cyanobacteria in the literature. The presence of these seemingly ancient bacteria in the sediments of the oxygen minimum zones of the ESP, one of the most productive but also ecologically most inefficient marine ecosystems of the world, suggests that such setting must have prevailed throughout the geological history of the planet allowing for their survival and further that it might be considered an analog of Proterozoic ocean conditions. These non-cyanobacterial communities offer an alternative hypothesis to students of the evolution of life on Earth and maybe of special interest to astrobiologists looking for life or traces of life in terrestrial or extraterrestrial, environments since these do not necessarily imply a photosynthesis-based metabolism.
KW - Algae
KW - Biospherics
KW - Extraterrestrial atmospheres
KW - Metabolism
KW - Molecular oxygen
KW - Sediments
KW - Eastern boundary currrent system
KW - Eastern South Pacific (ESP)
KW - Filamentous megabacteria
KW - Gamma Proteobacteria
KW - Protists
KW - Thioploca
KW - Bacteria
KW - Eastern boundary currrent system
KW - ESP south pacific
KW - Filamentous megabacteria
KW - Foraminifers
KW - Macrobacteria
KW - Microbial mats
KW - Oxygen minimum
KW - Protists
KW - Sulfide-rich sediments
KW - Thioploca
KW - Zone
SN - 0277786X (ISSN); 978-081946842-0 (ISBN)
LA - English
J2 - Proc SPIE Int Soc Opt Eng
M3 - Conference paper
DB - Scopus
N1 - Export Date: 23 February 2023; Cited By: 14; CODEN: PSISD
ER -

TY - JOUR
AU - Von Bloh, W.
AU - Bounama, C.
AU - Cuntz, M.

AU - Franck, S.
TI - The habitability of super-Earths in Gliese 581
PY - 2007
T2 - Astronomy and Astrophysics
VL - 476
IS - 3
SP - 1365
EP - 1371
DO - 10.1051/0004-6361:20077939
UR - https://www.scopus.com/inward/record.uri?eid=2-s2.0-38049174475&doi=10.1051%2f0004-6361%3a20077939&partnerID=40&md5=7063dfbc327af48cf0e3e3368982bbb8
AD - Potsdam Institute for Climate Impact Research, 14412 Potsdam, PO Box 60 12 03, Germany
AD - Department of Physics, University of Texas at Arlington, Box 19059, Arlington, TX 76019, United States
AB - Aims.The planetary system around the M star Gliese 581 consists of a hot Neptune (Gl 581b) and two super-Earths (Gl 581c and Gl 581d). The habitability of this system with respect to the super-Earths is investigated following a concept that studies the long-term possibility of photosynthetic biomass production on a dynamically active planet.Methods.A thermal evolution model for a super-Earth is used to calculate the sources and sinks of atmospheric carbon dioxide. The habitable zone is determined by the limits of photosynthetic life on the planetary surface. Models with different ratios of land / ocean coverage are investigated.Results.The super-Earth Gl 581c is clearly outside the habitable zone, since it is too close to the star. In contrast, Gl 581d is a tidally locked habitable super-Earth near the outer edge of the habitable zone. Despite the adverse conditions on this planet, at least some primitive forms of life may be able to exist on its surface. Therefore, Gl 581d is an interesting target for the planned TPF/Darwin missions to search for biomarkers in planetary atmospheres.. © 2007 ESO.
KW - Astrobiology
KW - Biomarkers
KW - Biomass
KW - Carbon dioxide
KW - Stars
KW - Atmospheric carbon dioxide
KW - Hot Neptune
KW - Photosynthetic biomass
KW - Planetary atmospheres
KW - Planetary systems
KW - Earth (planet)
KW - Astrobiology
KW - Stars: individual: Gl 581
KW - Stars: planetary systems
SN - 14320746 (ISSN)
LA - English
J2 - Astron. Astrophys.
M3 - Article
DB - Scopus
N1 - Export Date: 23 February 2023; Cited By: 75; Correspondence

Address: W. Von Bloh; Potsdam Institute for Climate Impact Research, 14412 Potsdam, PO Box 60 12 03, Germany; email: bloh@pik-potsdam.de;
CODEN: AAEJA
ER -

TY - JOUR
AU - Benerjee, N.R.
AU - Simonetti, A.
AU - Furnes, H.
AU - Muehlenbachs, K.
AU - Staudigel, H.
AU - Heaman, L.
AU - Van Kranendonk, M.J.
TI - Direct dating of Archean microbial ichnofossils
PY - 2007
T2 - Geology
VL - 35
IS - 6
SP - 487
EP - 490
DO - 10.1130/G23534A.1
UR - https://www.scopus.com/inward/record.uri?eid=2-s2.0-34249809862&doi=10.1130%2fG23534A.1&partnerID=40&md5=6a4651acc3899e142e6da4ed800b70cd
AD - Department of Earth Sciences, University of Western Ontario, London, ON N6A 5B7, Canada
AD - Department of Earth and Atmospheric Sciences, University of Alberta, Edmonton, AB T6G 2E3, Canada
AD - Center for Geobiology, Department of Earth Science, University of Bergen, 5007 Bergen, Allegaten 41, Norway
AD - Scripps Institution of Oceanography, University of California, La Jolla, CA 92093-0225, United States
AD - Geological Survey of Western Australia, East Perth, WA 6004, 100 Plain Street, Australia
AB - Well-preserved Archean pillow lavas from the ca. 3.35 Ga Euro Basalt of the Pilbara Craton, Western Australia, contain micron-sized tubular structures mineralized by titanite ($CaTiSiO4$) with residual organic carbon preserved along their margins. Direct U-Pb dating of titanite in the tubular structures demonstrates an Archean age. These tubular microstructures are identical to microbial ichnofossils in modern basalts, ophiolites, and greenstone belts, and are interpreted as a biogenic signature in these ancient rocks. Microbial colonization of basaltic glass thus appears to have been part of a deep subsurface biosphere established early in Earth's history. © 2007 The Geological Society of America.
KW - Australasia
KW - Australia
KW - Pilbara Block
KW - Western Australia
KW - Astrobiology
KW - Basalt
KW - Mineralogy
KW - Organic carbon
KW - Titanate minerals

KW - Biosphere
KW - Greenstone belts
KW - Microbial ichnofossils
KW - Ophiolites
KW - Archean
KW - basalt
KW - fossil
KW - geochronology
KW - greenstone belt
KW - ichnology
KW - microbial activity
KW - pillow lava
KW - titanite
KW - uranium-lead dating
KW - Geochronology
KW - Astrobiology
KW - Early life
KW - Geochronology
KW - Greenstone belt
KW - Ichnofossil
KW - Pilbara craton
SN - 00917613 (ISSN)
LA - English
J2 - Geology
M3 - Article
DB - Scopus
N1 - Export Date: 23 February 2023; Cited By: 80; Correspondence Address: N.R. Benerjee; Department of Earth Sciences, University of Western Ontario, London, ON N6A 5B7, Canada; email: neil.banerjee@uwo.ca; CODEN: GLGYB
ER -

TY - JOUR
AU - Van Thienen, P.
AU - Benzerara, K.
AU - Breuer, D.
AU - Gillmann, C.
AU - Labrosse, S.
AU - Lognonné, P.
AU - Spohn, T.
TI - Water, life, and planetary geodynamical evolution
PY - 2007
T2 - Space Science Reviews
VL - 129
IS - 1-3
SP - 167
EP - 203
DO - 10.1007/s11214-007-9149-7
UR - https://www.scopus.com/inward/record.uri?eid=2-s2.0-34548102422&doi=10.1007%2fs11214-007-9149-7&partnerID=40&md5=7037fdfd0136a6811baac9b0904fcd7e
AD - Faculty of Geosciences, Utrecht University, 3508 TA Utrecht, P.O. Box 80.021, Netherlands
AD - Institut de Minéralogie et de Physique des Milieux Condensés,

UMR 7590, Institut de Physique du Globe de Paris, 75015 Paris, 140 Rue de Lourmel, France
AD - German Aerospace Center (DLR), 12489 Berlin, Rutherfordstrasse 2, Germany
AD - Équipe Études Spatiales et Planétologie, Institut de Physique du Globe de Paris, 94107 Saint-Maur-des-Fossés, 4 Avenue de Neptune, France
AD - Laboratoire des Sciences de la Terre, École Normale Supérieure de Lyon, 69364 Lyon Cedex 07, 46 Allée d'Italie, France
AB - In our search for life on other planets over the past decades, we have come to understand that the solid terrestrial planets provide much more than merely a substrate on which life may develop. Large-scale exchange of heat and volatile species between planetary interiors and hydrospheres/atmospheres, as well as the presence of a magnetic field, are important factors contributing to the habitability of a planet. This chapter reviews these processes, their mutual interactions, and the role life plays in regulating or modulating them. © 2007 Springer Science+Business Media, Inc.
KW - Geology
KW - Magnetic field effects
KW - Habitability
KW - Mantle dynamics
KW - Thermal evolution
KW - Astrobiology
KW - Habitability
KW - Magnetic field
KW - Mantle dynamics
KW - Thermal evolution
KW - Volatiles
SN - 15729672 (ISSN)
LA - English
J2 - Space Sci Rev
M3 - Article
DB - Scopus
N1 - Export Date: 23 February 2023; Cited By: 26; Correspondence Address: P. Van Thienen; Faculty of Geosciences, Utrecht University, 3508 TA Utrecht, P.O. Box 80.021, Netherlands; email: thienen@geo.uu.nl; CODEN: SPSRA
ER -

TY - CONF
AU - Scorei, R.I.
AU - Cimpoiaşu, V.M.
AU - Popa, R.
TI - TD- 1HNMR measurements show enantioselective dissociation of ribose and glucose in the presence of H 2 17O
PY - 2007
T2 - Astrobiology
VL - 7
IS - 5
SP - 733
EP - 744
DO - 10.1089/ast.2006.0076
UR - https://www.scopus.com/inward/record.uri?eid=2-

s2.0-35549004140&doi=10.1089%2fast.2006.0076&partnerID=40&md5=c46018bd00b7f2603ee77e1186087a43
AD - University of Craiova, Craiova, Romania
AD - Portland State University, Portland, OR, United States
AD - Portland State University, Portland, OR 97201, 1719 SW 10th Avenue, United States
AB - We used Time Domain 1H Nuclear Magnetic Resonance (NMR) to characterize changes in proton exchange between water and sugar enantiomers at different concentrations of H 2 17O (~15-450 mM) and found that dissociation of the (-)-enantiomers of glucose and ribose occurs at significantly higher rates at higher concentrations of H 2 17O. The mechanism behind this enantioselective effect is unclear. The hypothesis we propose is that the large magnetic field (B o ~0.6T) applied during NMR measurements induces electric moments opposite in sign for the D and L-isomers. Because 17O has a nuclear electric quadrupole moment ≠ 0, asymmetrically hydrated complexes may form between the B o-polarized enantiomers and H 2 17O. Either H 2 17O is more often hydrating the (+) than the (-)-enantiomers - and consequently pK differences between H 2 16O and H 2 17O lead to differences in proton exchange between enantiomers and water - or the orientation of H 2 17O relative to the B o-polarized enantiomers is different, in total or in part, which leads to hydrated complexes with different spatial geometries and different proton exchange properties. This effect is significant for Magneto-Chiral Stereo-Chemistry (MCSC) and astrobiology, and it may help us better understand specific instances of mass independent isotopic fractionation and aid in the development of new technologies for chiral and isotopic separation. © Mary Ann Liebert, Inc.
KW - Biogenesis
KW - Glucose
KW - Magnetic Resonance Spectroscopy
KW - Molecular Conformation
KW - Oxygen Isotopes
KW - Ribose
KW - Water
KW - glucose
KW - oxygen
KW - ribose
KW - water
KW - article
KW - biogenesis
KW - chemistry
KW - conformation
KW - nuclear magnetic resonance spectroscopy
KW - Chirality
KW - Enantioselective
KW - Glucose
KW - H ₂ ¹⁷O
KW - NMR
KW - Proton exchange
KW - Ribose
SN - 15311074 (ISSN)
C2 - 17963473
LA - English

J2 - Astrobiology
M3 - Conference paper
DB - Scopus
N1 - Export Date: 23 February 2023; Cited By: 13; Correspondence Address: R. Popa; Portland State University, Portland, OR 97201, 1719 SW 10th Avenue, United States; email: rpopa@pdx.edu
ER -

TY - JOUR
AU - Scappini, F.
AU - Casadei, F.
AU - Zamboni, R.
AU - Monti, S.
AU - Giorgianni, P.
AU - Capobianco, M.L.
TI - Laboratory simulation of UV irradiation from the Sun on amino acids. I: Irradiation of tyrosine
PY - 2007
T2 - International Journal of Astrobiology
VL - 6
IS - 2
SP - 123
EP - 129
DO - 10.1017/S1473550407003588
UR - https://www.scopus.com/inward/record.uri?eid=2-s2.0-34547127158&doi=10.1017%2fS1473550407003588&partnerID=40&md5=78f7d412522f5446abbe99ce02d217c4
AD - Istituto per lo Studio dei Materiali Nanostrutturati, C.N.R., 40129 Bologna, Via P. Gobetti 101, Italy
AD - Istituto per la Sintesi Organica e la Fotoreattività, C.N.R., 40129 Bologna, Via P. Gobetti 101, Italy
AB - The effects of ultraviolet (UV) irradiation on water solutions of tyrosine (HO - C6H4 - CH2 - CHNH2COOH) have been investigated using a Xe lamp in the region 200-800 nm. This is a step in laboratory simulation towards reproducing the action of the Solar radiation on the building blocks of life, specifically α-amino acids, in the primitive Earth anoxic conditions. Results are presented showing the photostability of tyrosine against different UV doses. Degradation products partly maintain life building capability and partly do not. A tendency towards structure complexification was observed. The analysis of the irradiated tyrosine solutions was conducted using various spectroscopic and analytic techniques. The laboratory results are discussed in the light of a primordial life-emerging scenario. © Cambridge University Press 2007.
KW - amino acid
KW - exobiology
KW - simulation
KW - ultraviolet radiation
KW - Amino acids
KW - Astrobiology
KW - Chemical evolution
KW - Origin of life
KW - UV irradiation of amino acids

SN - 14753006 (ISSN)
LA - English
J2 - Int. J. Astrobiology
M3 - Article
DB - Scopus
N1 - Export Date: 23 February 2023; Cited By: 6; Correspondence Address: F. Scappini; Istituto per lo Studio dei Materiali Nanostrutturati, C.N.R., 40129 Bologna, Via P. Gobetti 101, Italy; email: f.scappini@bo.ismn.cnr.it
ER -

TY - CONF
AU - Picado, A.
AU - Bebianno, M.J.
AU - Costa, M.H.
AU - Ferreira, A.
AU - Vale, C.
TI - Biomarkers: A strategic tool in the assessment of environmental quality of coastal waters
PY - 2007
T2 - Hydrobiologia
VL - 587
IS - 1
SP - 79
EP - 87
DO - 10.1007/s10750-007-0695-5
UR - https://www.scopus.com/inward/record.uri?eid=2-s2.0-34250732212&doi=10.1007%2fs10750-007-0695-5&partnerID=40&md5=49b2cb61be40650eb9a4e966ef4bb527
AD - INETI, 1649-038 Lisbon, Estrada Paço Lumiar, Portugal
AD - CIMA, University of Algarve, Campus de Gambelas, Faro 8000, Portugal
AD - IMAR, DCEA, New University of Lisbon, Caparica 2829-516, Quinta da Torre, Portugal
AD - INIAP-IPIMAR, Lisbon 1449-006, Av. Brasilia, Portugal
AB - Ecosystems are under the pressure of complex mixtures of contaminants whose effects are not always simple to assess. Biomarkers, acting as early warning signals of the presence of potentially toxic xenobiotics, are useful tools for assessing either exposure to, or the effects of these compounds providing information about the toxicant bioavailability. In fact, it has been argued that a full understanding of ecotoxicological processes must consider an integrated multi-level approach, in which molecular impact is related with higher-order biological consequences at the individual, population and community levels. Monitoring programs should make use of this tool to link contaminants and ecological responses fulfilling strategies like those launched by OSPAR (Commissions of Oslo and Paris) Convention on the protection of the marine environment of the North-East Atlantic and the International Council for the Exploration of the Sea (ICES). An overview of the work done in the past few years using biomarkers as in situ tools for pollution assessment in Portuguese coastal waters is presented as a contribution to the set up of a biomonitoring program for the Portuguese coastal zone. Considering the data set available the

biomonitoring proposal should include the analysis of biomarkers and effects at individual levels. The aim of the program will include a spatial and temporal characterization of the biomarkers acetyl-cholinesterase, metallothioneins, DNA damage, adenylate energy charge and scope-for-growth levels. The investigation of the spatial variation of biomarkers is crucial to define sites for long term monitoring, which will be integrated with a chemical monitoring program. This framework will be a major contribution to the implementation of a national database for the use of biomarkers along the Portuguese coast. © 2007 Springer Science+Business Media B.V.
KW - Eurasia
KW - Europe
KW - Portugal
KW - Southern Europe
KW - Air quality
KW - Astrobiology
KW - Coastal zones
KW - Data acquisition
KW - Environmental impact assessments
KW - Impurities
KW - Information analysis
KW - Pollution
KW - Toxic materials
KW - biomarker
KW - biomonitoring
KW - coastal water
KW - ecotoxicology
KW - environmental assessment
KW - environmental quality
KW - marine pollution
KW - pollution monitoring
KW - spatial variation
KW - spatiotemporal analysis
KW - Biomonitoring
KW - Coastal waters
KW - Ecotoxicological processes
KW - Environmental quality
KW - Toxic xenobiotics
KW - Toxicant bioavailability
KW - Biomarkers
KW - Biomarkers of defence
KW - Biomarkers of exposure
KW - Coastal waters
KW - Environment quality
KW - Monitoring
SN - 15735117 (ISSN)
LA - English
J2 - Hydrobiologia
M3 - Conference paper
DB - Scopus
N1 - Export Date: 23 February 2023; Cited By: 35; Correspondence Address: A. Picado; INETI, 1649-038 Lisbon, Estrada Paço Lumiar, Portugal; email: ana.picado@ineti.pt; CODEN: HYDRB

ER -

TY - JOUR
AU - Plankensteiner, K.
AU - Reiner, H.
AU - Rode, B.M.
AU - Mikoviny, T.
AU - Wisthaler, A.
AU - Hansel, A.
AU - Märk, T.D.
AU - Fischer, G.
AU - Lammer, H.
AU - Rucker, H.O.
TI - Discharge experiments simulating chemical evolution on the surface of Titan
PY - 2007
T2 - Icarus
VL - 187
IS - 2
SP - 616
EP - 619
DO - 10.1016/j.icarus.2006.12.018
UR - https://www.scopus.com/inward/record.uri?eid=2-s2.0-33847386276&doi=10.1016%2fj.icarus.2006.12.018&partnerID=40&md5=0ec6b34328737c94dbd50f1b70f67967
AD - Theoretical Chemistry, University of Innsbruck, A-6020 Innsbruck, Innrain 52a, Austria
AD - Institute for Ion Physics and Applied Physics, University of Innsbruck, A-6020 Innsbruck, Technikerstrasse 25, Austria
AD - Space Research Institute, Austrian Academy of Sciences, A-8042 Graz, Schmiedlstraße 6, Austria
AB - Saturn's moon Titan has been considered as one of the few places in our Solar System, where atmospheric and surface conditions could have produced organic compounds essential as precursors for an evolution of life. The Cassini-Huygens mission has provided new data on Titan's atmosphere and surface, which enabled us to simulate the chemical processes occurring under these conditions. Possible lightning events on Titan cannot only produce higher hydrocarbons, but also allow surface water ice to participate in the reaction scenario, resulting in C{single bond}H{single bond}O, C{single bond}H{single bond}N, and C{single bond}H{single bond}O{single bond}N compounds including several molecules relevant for the formation of amino acids and nucleic acids. © 2007 Elsevier Inc. All rights reserved.
KW - Astrobiology
KW - Atmospheres
KW - chemistry
KW - Prebiotic chemistry
KW - Satellites
KW - surfaces
KW - Titan
SN - 10902643 (ISSN)
LA - English
J2 - Icarus

M3 - Article
DB - Scopus
N1 - Export Date: 23 February 2023; Cited By: 24; Correspondence Address: B.M. Rode; Theoretical Chemistry, University of Innsbruck, A-6020 Innsbruck, Innrain 52a, Austria; email: bernd.m.rode@uibk.ac.at; CODEN: ICRSA
ER -

TY - CONF
AU - Edwards, H.G.M.
TI - Question 2: Raman spectroscopic approach to analytical astrobiology: The detection of key biomolecular markers in the search for life
PY - 2007
T2 - Origins of Life and Evolution of Biospheres
VL - 37
IS - 4-5
SP - 335
EP - 339
DO - 10.1007/s11084-007-9079-0
UR - https://www.scopus.com/inward/record.uri?eid=2-s2.0-34548011946&doi=10.1007%2fs11084-007-9079-0&partnerID=40&md5=88485c82f559cda32297bd837acc0cea
AD - Centre for Astrobiology and Extremophiles Research, School of Life Sciences, University of Bradford, Bradford BD7 1DP, United Kingdom
AB - The recognition of extinct or extant life signatures in the terrestrial geological record is fundamentally dependent upon the understanding of both the structural morphology and chemical composition of relict biomaterials; the identification of cyanobacterial colonies that have adapted biogeologically their mineral matrices in early evolutionary processes is a fundamental step in the acquisition of analytical data from remote planetary probes designed for life-detection experiments, particularly on Mars and on the planetary satellite moons, Europa and Titan. A key factor in the assessment of early life signatures is the molecular presence of chemicals designed to protect the emerging organisms from the damaging effect of radiation exposure and of desiccation and temperature changes; in this respect the non-destructive capability of Raman spectroscopy to delineate the interfacial interactions between substrates and endolithic biology is now deemed an essential part of the ExoMars life-detection suite of instrumentation planned by the European Space Agency in the AURORA programme. A description of the scientific basis for the biogeological discrimination offered by Raman spectroscopy between organic and inorganic moieties in specimens from terrestrial Mars analogue sites is followed by selected examples of the type of analytical information provided, which will be complementary to the elementary and microscopic data obtained from other instrumentation on the same mission. © 2007 Springer Science+Business Media B.V.
KW - Biological Markers
KW - Exobiology
KW - Life
KW - Mars

KW - Spectrum Analysis, Raman
KW - Cyanobacteria
KW - biological marker
KW - chemical composition
KW - cyanobacterium
KW - Mars
KW - molecular analysis
KW - radiation exposure
KW - Raman spectroscopy
KW - spectroscopy
KW - analytical method
KW - astronomy
KW - biomarker
KW - desiccation
KW - detection method
KW - Europa
KW - geological record
KW - morphology
KW - Titan
KW - article
KW - astronomy
KW - biology
KW - instrumentation
KW - life
KW - methodology
KW - Raman spectrometry
KW - Biogeological modification
KW - ExoMars "search for traces of life" mission
KW - Protectant biosignatures
KW - Raman spectroscopy
KW - Spectral biomarkers
SN - 01696149 (ISSN)
C2 - 17592755
LA - English
J2 - Orig. Life Evol. Biosph.
M3 - Conference paper
DB - Scopus
N1 - Export Date: 23 February 2023; Cited By: 10; Correspondence Address: H.G.M. Edwards; Centre for Astrobiology and Extremophiles Research, School of Life Sciences, University of Bradford, Bradford BD7 1DP, United Kingdom; email: h.g.m.edwards@bradford.ac.uk; CODEN: OLEBE
ER -

TY - CONF
AU - Hoehn, A.
AU - Lynch, K.L.
AU - Clawson, J.
AU - Freeman, J.B.
AU - Kapit, J.
AU - Young, S.M.M.
AU - Kounaves, S.P.
AU - Brown, I.I.
TI - Microbial detection array (MDA), a novel instrument for

unambiguous detection of microbial metabolic activity in
astrobiology applications
PY - 2007
T2 - SAE Technical Papers
DO - 10.4271/2007-01-3190
UR - https://www.scopus.com/inward/record.uri?eid=2-s2.0-85072441463&doi=10.4271%2f2007-01-3190&partnerID=40&md5=e56c6e1e2f69797a07b6b0b7c2ffd07c
AD - BioServe Space Technologies, Aerospace Engineering Sciences, University of Colorado, Boulder, CO 80309-0429, United States
AD - In-Situ Planetary Chemical Analysis Laboratory, Department of Chemistry, Tufts University, Medford, MA 02155, United States
AD - NASA Johnson Space Center, United States
AB - MDA is designed as a test bed for an astrobiology field instrument to detect microbial metabolic activity in terrestrial or extraterrestrial geological soil samples. MDA employs electrochemical sensors in a unique differential chamber configuration, able to detect minute changes in the chemical composition between the two otherwise identical chambers. Both chambers are filled with identical autoclave-sterilized, sample-water mixtures. Only one of the chambers receives an additional minute, non-sterilized inoculation sample. Under the minimal assumptions that the geological sample contained nutrients (energy), organisms, and required water to initiate growth, the differential electrochemical measurements would now allow detection of metabolic activity, in addition to the electrochemical characterization of the soil samples in both chambers.
KW - Geology
KW - Metabolism
KW - Soil surveys
KW - Chemical compositions
KW - Electrochemical characterizations
KW - Electrochemical measurements
KW - Field instruments
KW - Geological samples
KW - Metabolic activity
KW - Soil sample
KW - Water mixture
KW - Electrochemical sensors
PB - SAE International
SN - 01487191 (ISSN)
LA - English
J2 - SAE Techni. Paper.
M3 - Conference paper
DB - Scopus
N1 - Export Date: 23 February 2023; Cited By: 0; Correspondence Address: A. Hoehn; BioServe Space Technologies, Aerospace Engineering Sciences, University of Colorado, Boulder, CO 80309-0429, United States; email: hoehn@colorado.edu
ER -

TY - JOUR
AU - Bénilan, Y.
AU - Cottin, H.

TI - Comets, titan and mars: Astrobiology and space projects
PY - 2007
T2 - Advances in Astrobiology and Biogeophysics
SP - 347
EP - 428
DO - 10.1007/978-3-540-33693-8_13
UR - https://www.scopus.com/inward/record.uri?eid=2-s2.0-33846097208&doi=10.1007%2f978-3-540-33693-8_13&partnerID=40&md5=9ab9c1c647bf841230b77fd8083ec093
AD - CNRS, Laboratoire Interuniversitaire des Systèmes Atmosphériques, Université Paris 12, Paris, France
AB - To date, there is nothing that could allow us to figure out if we will ever know whether the Earth is the only inhabited planet in the universe. However, astrobiology has gone a long way since the very first experiments by Miller in 1950 and since then, questions related to astrobiology are feeding the exploration of our solar system, and even beyond with ambitious programs for the observation of extrasolar planets (TPF, Darwin). The answer is maybe already at hand, by a rock analyzed with a Martian rover, or further away, under a few kilometers of ice in the Jupiter neighborhood... This chapter deals with the preliminary steps of the astrobiological exploration of the solar system, we are currently witnessing. We focused on the search of organic matter and liquid water, but none of them is a clear indicator of past or present life. We did not discuss the next issue about measurement(s) (chemical, geological, spectroscopic), which could allow us to claim that we have finally detected life on another planet. The scientific debate about it is still at its very first stages, and opens the most fascinating prospects. © 2007 Springer-Verlag Berlin Heidelberg.
A2 - Gargaud M.
A2 - Obs. Aquitain des Sci. de l'Univers, Universite Bordeaux 1, 33270 Floirac, 2 Rue de l'Observatoire
A2 - Herve M.
A2 - Laboratoire Magmas et Volcans, Universite Blaise Pascal, 63038 Clermont-Ferrand, 5 Rue Kessler
A2 - Claeys P.
A2 - Department of Geology, Vrije Universiteit Brussel, 1050 Brussels, Pleinlaan 2
SN - 16131851 (ISSN); 3540336923 (ISBN); 978-354033692-1 (ISBN)
LA - English
J2 - Adv. Astrobiol. Biogeophys.
M3 - Article
DB - Scopus
N1 - Export Date: 23 February 2023; Cited By: 1; Correspondence Address: Y. Bénilan; CNRS, Laboratoire Interuniversitaire des Systèmes Atmosphériques, Université Paris 12, Paris, France; email: benilan@lisa.univ-paris12.fr
ER -

TY - JOUR
AU - Hand, K.P.
AU - Carlson, R.W.
AU - Chyba, C.F.
TI - Energy, chemical disequilibrium, and geological constraints on

Europa
PY - 2007
T2 - Astrobiology
VL - 7
IS - 6
SP - 1006
EP - 1022
DO - 10.1089/ast.2007.0156
UR - https://www.scopus.com/inward/record.uri?eid=2-s2.0-37749024056&doi=10.1089%2fast.2007.0156&partnerID=40&md5=238f208c02f70799887f20eb345688f8
AD - Department of Geological and Environmental Sciences, Stanford University, Stanford, CA, United States
AD - Jet Propulsion Laboratory, California Institute of Technology, Pasadena, CA, United States
AD - Department of Astrophysical Sciences, Princeton University, Princeton, NJ, United States
AD - Department of Astrophysical Sciences, Princeton University, Peyton Hall, Princeton, NJ 08544, United States
AB - Europa is a prime target for astrobiology. The presence of a global subsurface liquid water ocean and a composition likely to contain a suite of biogenic elements make it a compelling world in the search for a second origin of life. Critical to these factors, however, may be the availability of energy for biological processes on Europa. We have examined the production and availability of oxidants and carbon-containing reductants on Europa to better understand the habitability of the subsurface ocean. Data from the Galileo Near-Infrared Mapping Spectrometer were used to constrain the surface abundance of CO2 to 0.036 by number relative to water. Laboratory results indicate that radiolytically processed CO2-rich ices yield CO and H2CO3; the reductants H2CO, CH3OH, and CH4 are at most minor species. We analyzed chemical sources and sinks and concluded that the radiolytically processed surface of Europa could serve to maintain an oxidized ocean even if the surface oxidants (O2, H2O2, CO2, SO2, and SO4 2-) are delivered only once every ~0.5 Gyr. If delivery periods are comparable to the observed surface age (30-70 Myr), then Europa's ocean could reach O2 concentrations comparable to those found in terrestrial surface waters, even if ~109 moles yr-1 of hydrothermally delivered reductants consume most of the oxidant flux. Such an ocean would be energetically hospitable for terrestrial marine macrofauna. The availability of reductants could be the limiting factor for biologically useful chemical energy on Europa.© 2007 Mary Ann Liebert, Inc.
KW - Biogenesis
KW - Ecosystem
KW - Energy-Generating Resources
KW - Geology
KW - Jupiter
KW - Oceans and Seas
KW - Oxidants
KW - Oxidation-Reduction
KW - Oxygen
KW - Pulse Radiolysis
KW - Insectivora

KW - oxidizing agent
KW - oxygen
KW - article
KW - astronomy
KW - biogenesis
KW - chemistry
KW - ecosystem
KW - energy resource
KW - geology
KW - oxidation reduction reaction
KW - pulse radiolysis
KW - sea
KW - Europa
KW - Habitability
KW - Oxygen
KW - Planetary science
KW - Radiolysis
SN - 15311074 (ISSN)
C2 - 18163875
LA - English
J2 - Astrobiology
M3 - Article
DB - Scopus
N1 - Export Date: 23 February 2023; Cited By: 143; Correspondence Address: K.P. Hand; Department of Astrophysical Sciences, Princeton University, Princeton, NJ, United States; email: khand@stanford.edu
ER -

TY - JOUR
AU - Snyder, L.J.
AU - Weber, T.P.
TI - Introduction
PY - 2007
T2 - Studies in History and Philosophy of Science Part A
VL - 38
IS - 3
SP - 567
EP - 569
DO - 10.1016/j.shpsa.2007.06.010
UR - https://www.scopus.com/inward/record.uri?eid=2-s2.0-34548539608&doi=10.1016%2fj.shpsa.2007.06.010&partnerID=40&md5=61e2895855932955ae6a0db0ae155f57
AD - Department of Philosophy, St. John's University, NY, United States
AD - I-21014 Laveno Mombello (VA), Via Marsala 17, Italy
AB - The question of the existence of intelligent life on other worlds has never been a purely scientific one. Philosophical, religious and literary issues have been intertwined with scientific ones throughout the history of the "plurality of worlds" debate. This collection of papers in -Studies in History and Philosophy of Science- explores the interrelation of science, philosophy, religion and literature in debates about extraterrestrial life. © 2007 Elsevier Ltd. All rights reserved.
PB - Elsevier Ltd

SN - 00393681 (ISSN)
LA - English
J2 - Stud. Hist. Philos. Sci. Part A
M3 - Editorial
DB - Scopus
N1 - Export Date: 23 February 2023; Cited By: 0; Correspondence Address: L.J. Snyder; Department of Philosophy, St. John's University, NY, United States; email: snyderl@stjohns.edu; CODEN: SHPSB
ER -

TY - JOUR
AU - Lynne, B.Y.
AU - Campbell, K.A.
AU - James, B.J.
AU - Browne, P.R.J.
AU - Moore, J.
TI - Tracking crystallinity in siliceous hot-spring deposits
PY - 2007
T2 - American Journal of Science
VL - 307
IS - 3
SP - 612
EP - 641
DO - 10.2475/03.2007.03
UR - https://www.scopus.com/inward/record.uri?eid=2-s2.0-34447286263&doi=10.2475%2f03.2007.03&partnerID=40&md5=7cce73386fa61fffc0b5c7bdfc7b7d47
AD - University of Auckland, Department of Geology, Auckland, Private Bag 92019, New Zealand
AD - University of Utah, Energy and Geosciences Institute, Salt Lake City, UT 84108, 423 Wakara Way, United States
AB - Siliceous hot spring deposits (sinters) entrap paleoenvironmentally significant components and are used as extreme-environment analogs in the search for early Earth and extraterrestrial life. However, sinters undergo a series of textural and mineralogical changes during diagenesis that can modify and overprint original environmental signals. For ancient hydrothermal settings including those close to the dawn of life, these transformations have long since occurred, so that study of diagenetic processes and effects is best undertaken in much younger deposits still undergoing change. Three young sinters preserve the entire diagenetic sequence of silica phases, from opal-A to quartz. The 6000 to ~ 11,500 years BP ± 70 years sinter at Steamboat Springs, Nevada, the ~ 1600 - 1900 ± 160 years BP Opal Mound sinter at Roosevelt Hot Springs, Utah, and the ~ 456 ± 35 years BP deposit at Sinter Island, Taupo Volcanic Zone, New Zealand, provide an opportunity to track crystallographic, mineralogic and morphologic transitions of sinter diagenesis using standard and new analytical approaches. Worldwide, sinter forms from cooling, alkali chloride waters as noncrystalline opal-A, transforming first into noncrystalline opal-A/CT, then paracrystalline opal-CT ± moganite, paracrystalline opal-C, and eventually to microcrystalline quartz. In this study, these changes were identified by the novel and

combined application of electron backscatter diffraction, X-ray powder diffraction, and scanning electron and optical microscopy techniques. We show that mineralogical changes precede morphological and accompanied crystallographic transformations. During this modification, silica particles grow and shrink several times from the micron- to nano-meter scales via dissolution, reprecipitation and recrystallization, and diagenesis follows the Ostwald Step rule. All deposits followed nearly identical diagenetic pathways, with time as the only variable in the march toward physicochemically stable quartz crystals. Diagenesis alters original environmental signatures trapped within sinters. After five silica phase changes, filamentous microfossils are modified but still remain recognizable within sinter from the Opal Mound and Steamboat Springs deposits, and during the opal-A to opal-CT silica phase transformations at Sinter Island. Therefore, delineating diagenetic components and how they affect sinters is necessary to accurately identify biosignals from ancient hot-spring deposits.
KW - Australasia
KW - New Zealand
KW - North Island
KW - Taupo Volcanic Zone
KW - crystallinity
KW - crystallography
KW - hydrothermal deposit
KW - mineralogy
KW - quartz
KW - recrystallization
KW - scanning electron microscopy
KW - siliceous deposit
KW - texture
KW - thermal spring
KW - X-ray diffraction
SN - 00029599 (ISSN)
LA - English
J2 - Am. J. Sci.
M3 - Article
DB - Scopus
N1 - Export Date: 23 February 2023; Cited By: 70; Correspondence Address: B.Y. Lynne; University of Auckland, Department of Geology, Auckland, Private Bag 92019, New Zealand; email: b.lynne@auckland.ac.nz
ER -

TY - JOUR
AU - Smith, D.S.
AU - Scalo, J.
TI - Solar X-ray flare hazards on the surface of Mars
PY - 2007
T2 - Planetary and Space Science
VL - 55
IS - 4
SP - 517
EP - 527
DO - 10.1016/j.pss.2006.10.001

UR - https://www.scopus.com/inward/record.uri?eid=2-s2.0-33846900653&doi=10.1016%2fj.pss.2006.10.001&partnerID=40&md5=e9488245c1dcbb63b3cc503366974f83
AD - Department of Astronomy, The University of Texas at Austin, Austin, TX 78712, United States
AB - Putative organisms on the Martian surface would be exposed to potentially high doses of ionizing radiation during strong solar X-ray flares. We extrapolate the observed flare frequency-energy release scaling relation to total X-ray energies much larger than seen so far for the Sun, an assumption supported by observations of flares on other solar- and subsolar-mass main sequence stars. Flare spectra are taken as power laws, with the logarithmic slope a parameter based on the observed statistics of the most energetic hard X-ray flare spectra. We calculate surface spectra using a Monte Carlo code we developed for the transport of X-rays and gamma rays, including photoabsorption and detailed Compton scattering. Biological doses from indirect genome damage are calculated for each parameterized flare spectrum by integration over the X-ray opacity of water. The resulting doses depend sensitively on spectral slope, which varies greatly and unsystematically for solar flares. Using the roughly uniform observed distribution of spectral slopes, we estimate the mean waiting time for solar flares producing a given biological dose of ionizing radiation on Mars and compare with lethal dose data for a wide range of terrestrial organisms. These timescales range from decades for significant human health risk to 0.5 Myr for D. radiodurans lethality. Such doses require total flare energies of 1033-1038 erg, the lower range of which has been observed for other stars. Flares are intermittent bursts, so acute lethality will only occur on the sunward hemisphere during a sufficiently energetic flare, unlike low-dose-rate, extended damage by cosmic rays. We estimate the soil and CO2 ice columns required to provide 1 / e shielding as 4 - 9 g cm- 2, depending on flare mean energy and atmospheric column density. Topographic altitude variations give a factor of two variation in dose for a given flare. Life in ice layers that may exist ~ 100 g cm- 2 below the surface would be well protected. Finally, we point out that designing spacesuits to sufficiently block this radiation on Mars missions may be difficult, given the conflict between solutions for lightweight protection from energetic particles and those from X-rays. © 2006 Elsevier Ltd. All rights reserved.
KW - Gamma rays
KW - Manned space flight
KW - Radiation hazards
KW - Radiation protection
KW - Solar energy
KW - Solar radiation
KW - Spectrum analysis
KW - Sun
KW - X rays
KW - Habitable planets
KW - Solar flares
KW - Subsolar masses
KW - X ray flares
KW - Martian surface analysis

KW - Astrobiology
KW - Gamma-rays
KW - Habitable planets
KW - Ionizing radiation
KW - Manned space missions
KW - Mars
KW - Radiation protection
KW - Solar flares
KW - X-rays
SN - 00320633 (ISSN)
LA - English
J2 - Planet. Space Sci.
M3 - Article
DB - Scopus
N1 - Export Date: 23 February 2023; Cited By: 9; Correspondence Address: D.S. Smith; Department of Astronomy, The University of Texas at Austin, Austin, TX 78712, United States; email: dss@astro.as.utexas.edu; CODEN: PLSSA
ER -

TY - CONF
AU - Pikuta, E.V.
AU - Hoover, R.B.
TI - Microbial extremophiles in evolutionary aspect
PY - 2007
T2 - Proceedings of SPIE - The International Society for Optical Engineering
VL - 6694
C7 - 66941C
DO - 10.1117/12.742289
UR - https://www.scopus.com/inward/record.uri?eid=2-s2.0-42149185691&doi=10.1117%2f12.742289&partnerID=40&md5=b5175174f48c76840c60cb8b3d9e4c27
AD - Astrobiology Laboratory, NASA/MSFC/NSSTC, Huntsville, AL 35805, 320 Sparkman Dr., United States
AB - The microflora of the cryosphere of planet Earth provides the best analogs for life forms that might be found in the permafrost or polar ice caps of Mars, near the surface of the cometary nuclei, or in the liquid water beneath the ice crusts of icy moons of Jupiter and Saturn. For astrobiology the focus on the study alkaliphilic microorganisms was enhanced by the findings of abundant carbonates and carbonate globules rimmed with possibly biogenic magnetites in association with the putative microfossils in the ALH84001 meteorite. Although the ALH84001 "nanofossils" were too small and simple to be unambiguously recognized as biogenic, they stimulated Astrobiology research and studies of microbial extremophiles and biomarkers in ancient rocks and meteorites. Recent studies of CI and CM carbonaceous meteorites have resulted in the detection of the well-preserved mineralized remains of coccoidal and filamentous microorganisms in cyanobacterial mats. Energy Dispersive X-ray Analysis has shown anomalous biogenic element ratios clearly indicating they are not recent biological contaminants. This paper reviews microbial extremophiles in context of their significance to Astrobiology and the evolution of life. Extremophilic microorganisms

on Earth are models for life that might endure high radiation environments in the ice near the surface of comets or on the icy moons of Jupiter and Saturn and in the seafloor deep beneath the icy crusts of Europa and Enceladus.
KW - Astrobiology
KW - Earth (planet)
KW - Ecosystems
KW - Energy dispersive X ray analysis
KW - Meteorites
KW - Biogenic magnetites
KW - Liquid water
KW - Microbial extremophiles
KW - Microflora
KW - Microorganisms
KW - Astrobiology
KW - Ecosystems
KW - Evolution
KW - Extremophilic microorganisms
KW - Meteorites
SN - 0277786X (ISSN); 978-081946842-0 (ISBN)
LA - English
J2 - Proc SPIE Int Soc Opt Eng
M3 - Conference paper
DB - Scopus
N1 - Export Date: 23 February 2023; Cited By: 0; CODEN: PSISD
ER -

TY - CONF
AU - Levin, G.V.
TI - The revival of life on Mars
PY - 2007
T2 - Proceedings of SPIE - The International Society for Optical Engineering
VL - 6694
C7 - 66940M
DO - 10.1117/12.731034
UR - https://www.scopus.com/inward/record.uri?eid=2-s2.0-42149189538&doi=10.1117%2f12.731034&partnerID=40&md5=da76f2cf7f4111f643264e94b5045a23
AD - BioSpherix Division, Spherix Incorporated, Annapolis, MD 21401, United States
AB - Few, if any, major scientific quests have taken such frequent, diametrically opposed changes in prospect and direction as has the search for life on Mars. The erratic courses and their supporting rationales are traced: from Percival Lowell's astonishing pronouncement of intelligent beings on the red planet; to their denouement by Mariner 4; to the strong contraindications of any form of Martian life relayed by Mariners 6 and 7; to the discovery of a different, perhaps once-habitable, Mars revealed by the detailed orbital images, including the first evidence of ancient rivers, taken by Mariner 9; to the still-controversial claim of the detection of microbial life by the 1976 Viking Missions; to NASA's subsequent prohibition against any Mars life detection experiments; to the recently emerged consensus, propelled by findings of

Pathfinder, the Mars Exploratory Rovers and the continuing discoveries of life in extreme environments on Earth, that past or extant life on Mars is likely. Against this background, the future Mars missions' experiments bearing on the life issue are reviewed. The case is made that none of these experiments, as currently planned, still subject to the prohibition against direct life detection experiments, can resolve this paramount and fundamental question that bears so heavily on the origin and distribution of life, and our place in the universe.
KW - Astrobiology
KW - Extraterrestrial atmospheres
KW - Martian surface analysis
KW - NASA
KW - Extraterrestrial life
KW - Origin of life
KW - Planets
KW - Astrobiology
KW - Extraterrestrial life
KW - Life on mars
KW - Mars
KW - Origin of life
KW - Search for life on Mars
SN - 0277786X (ISSN); 978-081946842-0 (ISBN)
LA - English
J2 - Proc SPIE Int Soc Opt Eng
M3 - Conference paper
DB - Scopus
N1 - Export Date: 23 February 2023; Cited By: 1; Correspondence Address: G. V. Levin; BioSpherix Division, Spherix Incorporated, Annapolis, MD 21401, United States; email: glevin@spherix.com; CODEN: PSISD
ER -

TY - CONF
AU - Sheldon, R.B.
AU - Hoover, R.B.
TI - The cometary biosphere
PY - 2007
T2 - Proceedings of SPIE - The International Society for Optical Engineering
VL - 6694
C7 - 66940H
DO - 10.1117/12.729094
UR - https://www.scopus.com/inward/record.uri?eid=2-s2.0-42149187118&doi=10.1117%2f12.729094&partnerID=40&md5=f46541631bdabc2ab5a106bce5761362
AD - USRA, NSSTC, Huntsville, AL, 320 Sparkman Dr., United States
AD - NASA, MSFC, NSSTC, Huntsville, AL, 320 Sparkman Dr., United States
AB - Recent observations of cyanobacterial fossils on carbonaceous chondrites have conclusively established the presence of fossil organisms on extraterrestrial bodies widely presumed to be comets. Likewise, the data from four cometary flyby (and one impact) missions and the exploration of a peculiar S-type asteroid, show

evidence of liquid water in the past or present. In addition, sand grains returned from the tail of comet P/Wild-2 demonstrate that comets accrete inner Solar System material. So it is a short step to propose the separate and independent existence of a cometary biosphere, the ecosystem of organisms that exploit the niche of an extraterrestrial environment. This paper attempts to lay the framework for such a hypothetical ecosystem, and establish criteria for its continued existence and spread.
KW - Asteroids
KW - Astrobiology
KW - Ecosystems
KW - Meteorites
KW - Microorganisms
KW - Solar system
KW - Cyanobacteria
KW - Panspermia
KW - Biospherics
KW - Astrobiology
KW - Biosphere
KW - Comet
KW - Cyanobacteria
KW - Panspermia
SN - 0277786X (ISSN); 978-081946842-0 (ISBN)
LA - English
J2 - Proc SPIE Int Soc Opt Eng
M3 - Conference paper
DB - Scopus
N1 - Export Date: 23 February 2023; Cited By: 9; Correspondence Address: R. B. Sheldon; USRA, NSSTC, Huntsville, AL, 320 Sparkman Dr., United States; email: Rob.Sheldon@msfc.nasa.gov; CODEN: PSISD
ER -

TY - CONF
AU - Kruzelecky, R.V.
AU - Wong, B.
AU - Haddad, E.
AU - Jamroz, W.
AU - Cloutis, E.
AU - Ghafoor, N.
AU - Jessen, S.
TI - Inukshuk landed robotic Canadian mission to mars using a miniature sample analysis lab for planetary mineralogy and microbiology
PY - 2007
T2 - SAE Technical Papers
DO - 10.4271/2007-01-3104
UR - https://www.scopus.com/inward/record.uri?eid=2-s2.0-85072444968&doi=10.4271%2f2007-01-3104&partnerID=40&md5=ece023891e7ed7516eb505773141202c
AD - MPB Communications Inc., Canada
AD - Univ of Winnipeg, Canada
AD - MDA Space Missions, Canada
AB - This paper discusses the Inukshuk landed rover mission to Mars that is currently undergoing the Phase 0 mission study for the

Canadian Space Agency. The Inukshuk landed rover mission addresses key science themes for planetary exploration; focusing on the search for hydrated mineralogy and subsurface water sites that can provide evidence of past or present life. New exploration and science will be accomplished using an innovative tethered combination of a small rover and a self-elevating sky-cam aerostat. The elevating visible (VIS) imager, at about 10 m altitude, will provide an informative high-resolution 2-D view of the rover below and surrounding terrain to greatly assist the semi-autonomous navigation of the rover around obstacles and selection of sites for detailed subsurface exploration. The solar-powered rover will employ MDA expertise in robotics and drilling with MPB's expertise in miniature infrared (IR) spectrometers and fiber-optic sensors to provide subsurface analysis of mineralogy and temperature distributions at depths to about 1 m. Mission cost effectiveness is achieved through a synergistic instrument suite based on advanced but mature miniaturization technologies that enable high IR spectral measurement performance with minimal mass and power. Insitu systematic sample analysis at depths to about 1 m will be performed using a monolithic fiber-optic coupled probe integrated directly with the tethered mole driller. For space-based systems, the important drivers are reliability, power consumption, mass and simplicity of operation. MPB has advanced its patented IOSPEC technology for miniature integrated IR spectrometers to provide high performance comparable to large laboratory spectrometers but in a very compact and ruggedized footprint weighing under 2.0 kg. Inukshuk sample analysis will employ the data synergy provided by a miniature suite of high-performance instruments, including IR reflection between 0.8 and 4.5 microns at about 4 nm resolution, microscopic VIS colour imaging to 1 µm spatial resolution, and complementary IR Raman spectroscopy between 500 and 3500 cm-1 for direct C-C biological detection. The combined data can, for the first time, directly and unambiguously detect H2O and determine its state (ice/liquid/ structural), distinguish key mineral species (including those associated with favorable habitats for microbial activity) and determine their hydration states, as well as detect and differentiate various C-H and C-C molecular structures for astrobiological investigations. Copyright © 2007 SAE International.
KW - Cost effectiveness
KW - Fiber optics
KW - Hydration
KW - Interplanetary spacecraft
KW - Minerals
KW - Patents and inventions
KW - Robotics
KW - Robots
KW - Solar energy
KW - Spectrometers
KW - Autonomous navigation
KW - Canadian Space Agency
KW - IR- Raman spectroscopies
KW - Microbial activities
KW - Miniaturization technologies
KW - Planetary exploration

KW - Spectral measurement
KW - Subsurface exploration
KW - Fiber optic sensors
PB - SAE International
SN - 01487191 (ISSN)
LA - English
J2 - SAE Techni. Paper.
M3 - Conference paper
DB - Scopus
N1 - Export Date: 23 February 2023; Cited By: 0; Correspondence Address: R.V. Kruzelecky; MPB Communications Inc., Canada; email: roman.kruzelecky@mpbc.ca
ER -

TY - CONF
AU - Liesch, P.J.
AU - Kolb, V.M.
TI - Importance of the interaction between sodium silicate and organic materials to astrobiology: Alcohol-based organo-silicates as potential biosignatures
PY - 2007
T2 - Proceedings of SPIE - The International Society for Optical Engineering
VL - 6694
C7 - 669405
DO - 10.1117/12.731345
UR - https://www.scopus.com/inward/record.uri?eid=2-s2.0-42149145352&doi=10.1117%2f12.731345&partnerID=40&md5=d165e335adbeb02ba8aeef156f80935c
AD - Department of Chemistry, University of Wisconsin-Parkside, Kenosha, WI 53141, United States
AB - We have found that alcohols catalyze a rapid polymerization of silicic acid to silica gel upon the addition of alcohols. Like amino acids, alcohols could be preserved in the silica gel by two mechanisms. In the first mechanism, the alcohols become entombed in the silica gel. In this case, only Si-O-Si bonds would be observed in the infrared (IR) spectra. In the second mechanism, the alcohols make chemical bonds with the silicic acid, to create organo-silicates. In this case, Si-O-C bonds would be observed in the IR. We have found that steric hindrance plays a major role in the volume of an alcohol required. Amino alcohols are more effective in creating a viscous gel than the alcohols, and appeared to form covalent Si-O-C bonds in some cases. Characteristic shifts in the Si-O-Si bands of over 50 cm-1 towards lower IR frequencies can be observed in the alcohol gels. The hydrolysis of known organo-silicates has led to the identification of the Si-O-C band in the region of 1225-1150 cm -1, and the Si-O-Si band in the region of 1125-1000 cm-1. Alcohols play an important role in many biological pathways, and could serve as biosignatures in extreme environments including meteorites and other planets.
KW - Alcohols
KW - Astrobiology
KW - Chemical bonds
KW - Gels

KW - Silica
KW - Alcohol silicates
KW - Biosignatures
KW - Organo-silicates
KW - Silicic acid
KW - Silicates
KW - Alcohol silicates
KW - Astrobiology
KW - Biosignature
SN - 0277786X (ISSN); 978-081946842-0 (ISBN)
LA - English
J2 - Proc SPIE Int Soc Opt Eng
M3 - Conference paper
DB - Scopus
N1 - Export Date: 23 February 2023; Cited By: 0; CODEN: PSISD
ER -

TY - JOUR
AU - Raven, J.
TI - Astrobiology: Photosynthesis in watercolours
PY - 2007
T2 - Nature
VL - 448
IS - 7152
SP - 418
DO - 10.1038/448418a
UR - https://www.scopus.com/inward/record.uri?eid=2-s2.0-34547196740&doi=10.1038%2f448418a&partnerID=40&md5=d78e83fbc089d9ea6456a5a480d75b0b
AD - College of Life Sciences, University of Dundee, Dundee DD2 5DA, Invergowrie, United Kingdom
AB - The spectrum of stellar radiation available to an organism is altered by the atmosphere and water on the planet it inhabits. Study of this relationship can outline the limits to photosynthesis. ©2007 Nature Publishing Group.
KW - water
KW - astronomy
KW - atmosphere
KW - photosynthesis
KW - water
KW - astronomy
KW - atmosphere
KW - color
KW - electromagnetic radiation
KW - energy resource
KW - light absorption
KW - photochemistry
KW - photosynthesis
KW - priority journal
KW - reflectometry
KW - seaweed
KW - short survey
KW - solar radiation
KW - spectral sensitivity

PB - Nature Publishing Group
SN - 00280836 (ISSN)
C2 - 17653178
LA - English
J2 - Nature
M3 - Short survey
DB - Scopus
N1 - Export Date: 23 February 2023; Cited By: 24; Correspondence Address: J. Raven; College of Life Sciences, University of Dundee, Dundee DD2 5DA, Invergowrie, United Kingdom; email: j.a.raven@dundee.ac.uk; CODEN: NATUA
ER -

TY - JOUR
AU - Fernández-Remolar, D.C.
AU - Gómez, F.
AU - Prieto-Ballesteros, O.
AU - Schelble, R.T.
AU - Rodríguez, N.
AU - Amils, R.
TI - Some ecological mechanisms to generate habitability in planetary subsurface areas by chemolithotrophic communities: The Ro Tinto subsurface ecosystem as a model system
PY - 2008
T2 - Astrobiology
VL - 8
IS - 1
SP - 157
EP - 173
DO - 10.1089/ast.2006.0022
UR - https://www.scopus.com/inward/record.uri?eid=2-s2.0-39749115524&doi=10.1089%2fast.2006.0022&partnerID=40&md5=99bbd0125308447c086667e7ad67a789
AD - Centro de Astrobiología, CSIC-INTA, Torrejón de Ardoz, Spain
AD - Geophysical Laboratory, Carnegie Institution of Washington, Washington, DC, United States
AD - Centro de Biología Molecular, Universidad Autónoma de Madrid, Cantoblanco, Spain
AD - Centro de Astrobiología, CSIC-INTA, E-28850 Torrejón de Ardoz, Ctra. Ajalvir km. 4, Spain
AB - Chemolithotrophic communities that colonize subsurface habitats have great relevance for the astrobiological exploration of our Solar System. We hypothesize that the chemical and thermal stabilization of an environment through microbial activity could make a given planetary region habitable. The MARTE project ground-truth drilling campaigns that sampled cryptic subsurface microbial communities in the basement of the Ro Tinto headwaters have shown that acidic surficial habitats are the result of the microbial oxidation of pyritic ores. The oxidation process is exothermic and releases heat under both aerobic and anaerobic conditions. These microbial communities can maintain the subsurface habitat temperature through storage heat if the subsurface temperature does not exceed their maximum growth temperature. In the acidic solutions of the Ro Tinto, ferric iron acts as an effective buffer for

controlling water pH. Under anaerobic conditions, ferric iron is the oxidant used by microbes to decompose pyrite through the production of sulfate, ferrous iron, and protons. The integration between the physical and chemical processes mediated by microorganisms with those driven by the local geology and hydrology have led us to hypothesize that thermal and chemical regulation mechanisms exist in this environment and that these homeostatic mechanisms could play an essential role in creating habitable areas for other types of microorganisms. Therefore, searching for the physicochemical expression of extinct and extant homeostatic mechanisms through physical and chemical anomalies in the Mars crust (i.e., local thermal gradient or high concentration of unusual products such as ferric sulfates precipitated out from acidic solutions produced by hypothetical microbial communities) could be a first step in the search for biological traces of a putative extant or extinct Mars biosphere. © Mary Ann Liebert, Inc.
KW - Chemoautotrophic Growth
KW - Ecosystem
KW - Exobiology
KW - Geologic Sediments
KW - Geology
KW - Heat
KW - Homeostasis
KW - Hydrogen-Ion Concentration
KW - Iron
KW - Rivers
KW - Spain
KW - Sulfur
KW - iron
KW - sulfur
KW - article
KW - biology
KW - chemistry
KW - chemoautotrophy
KW - ecosystem
KW - geology
KW - heat
KW - homeostasis
KW - microbiology
KW - pH
KW - physiology
KW - river
KW - sediment
KW - Spain
KW - Habitability
KW - Homeostasis
KW - Microbial communities
SN - 15311074 (ISSN)
C2 - 18237256
LA - English
J2 - Astrobiology
M3 - Article
DB - Scopus
N1 - Export Date: 23 February 2023; Cited By: 17; Correspondence

Address: D.C. Fernández-Remolar; Centro de Astrobiología, CSIC-INTA, E-28850 Torrejón de Ardoz, Ctra. Ajalvir km. 4, Spain; email: fernandezrd@inta.es
ER -

TY - JOUR
AU - Ruiz-Bermejo, M.
AU - Menor-Salván, C.
AU - Mateo-Martí, E.
AU - Osuna-Esteban, S.
AU - Martín-Gago, J.A.
AU - Veintemillas-Verdaguer, S.
TI - CH4/N2/H2 spark hydrophilic tholins: A systematic approach to the characterization of tholins
PY - 2008
T2 - Icarus
VL - 198
IS - 1
SP - 232
EP - 241
DO - 10.1016/j.icarus.2008.07.008
UR - https://www.scopus.com/inward/record.uri?eid=2-s2.0-54049151907&doi=10.1016%2fj.icarus.2008.07.008&partnerID=40&md5=c6c83af3228826228d63e58bef4a405b
AD - Centro de Astrobiología, Consejo Superior Investigaciones Cientificas-Instituto Nacional de Tecnica Aeroespacial (CSIC-INTA), E-28850 Torrejon de Ardoz, Madrid, Carretera Torrejon-Ajalvir, Km. 4.2, Spain
AD - Instituto de Ciencia de Materiales de Madrid (CSIC), Cantoblanco, E-28049 Madrid, C/ Sor Juana Ines de la Cruz, 3, Spain
AB - Tholins are complex organic materials produced by irradiation of several carbon and nitrogen rich atmospheres. They could have played a key role in the origin of life on Earth because their ability to release important bioorganics, which are nowadays present in proteins, nucleic-acid bases and other important biostructures. Usually, the yield of these compounds is higher after acid hydrolysis, however little is known about the structure and chemical composition of the tholins. In this work, we propose the use of different spectroscopic and separation techniques, which are not usually applied in this field, in order to obtain complete information about the tholin structure and behavior. Two different simulation experiments of prebiotic synthesis were carried out in CH4/N2/H2 atmosphere out from spark discharge activation of aqueous aerosols and liquid water, respectively. In both cases, a hydrophilic tholin and a hydrophobic tholin were obtained. Herein, we report the application of this methodology to our hydrophilic tholins and we review, briefly, some astrobiological aspects related to these complex substances. © 2008 Elsevier Inc. All rights reserved.
KW - Organic chemistry
KW - Prebiotic chemistry
KW - Prebiotic environments
KW - Spectroscopy
SN - 10902643 (ISSN)

LA - English
J2 - Icarus
M3 - Article
DB - Scopus
N1 - Export Date: 23 February 2023; Cited By: 26; Correspondence Address: M. Ruiz-Bermejo; Centro de Astrobiología, Consejo Superior Investigaciones Cientificas-Instituto Nacional de Tecnica Aeroespacial (CSIC-INTA), E-28850 Torrejon de Ardoz, Madrid, Carretera Torrejon-Ajalvir, Km. 4.2, Spain; email: ruizbm@inta.es; CODEN: ICRSA
ER -

TY - BOOK
AU - Chela-Flores, J.
TI - A second genesis: Stepping-stones towards the intelligibility of nature
PY - 2008
T2 - A Second Genesis: Stepping-Stones Towards the Intelligibility of Nature
SP - 1
EP - 229
DO - 10.1142/7029
UR - https://www.scopus.com/inward/record.uri?eid=2-s2.0-84967432014&doi=10.1142%2f7029&partnerID=40&md5=92c6c9dc988b3974db3a6732b94ad763
AD - The Abdus Salam ICTP, Instituto de Wstudios Avanzados, United States
AB - A Second Genesis enquires why nature is intelligible. The fast growth of technology and deeper understanding of the humanities have provided significant clues. Answering the question why nature can be understood requires an introduction to the new science of astrobiology and the exploration of the Solar System. A careful discussion of a ”second Genesis” is presented, namely our present awareness that life may have emerged on other worlds. Writing this volume has been motivated by the need to encourage a constructive dialogue between science and faith. Such an objective for a new book is timely, since science is inserted with well-defined frontiers in the context of human culture. Similarly, the frontiers of faith do not require religion to justify itself in scientific terms, avoiding current unnecessary controversies. This book intends to engage readers interested in the position of humans in nature. It makes a serious effort to avoid demanding detailed knowledge of science, philosophy, or theology, but will require some careful reading and meditation. © 2008 by World Scientific Publishing Co. Pte. Ltd. All rights reserved.
PB - World Scientific Publishing Co.
SN - 978-981283504-8 (ISBN); 978-981283503-1 (ISBN)
LA - English
J2 - A Second Genesis: Stepping-Stones Towards the Intelligibility of Nat.
M3 - Book
DB - Scopus
N1 - Export Date: 23 February 2023; Cited By: 2
ER -

TY - JOUR
AU - Brandes, J.A.
AU - Hazen, R.M.
AU - Yoder Jr., H.S.
TI - Inorganic nitrogen reduction and stability under simulated hydrothermal conditions
PY - 2008
T2 - Astrobiology
VL - 8
IS - 6
SP - 1113
EP - 1126
DO - 10.1089/ast.2007.0187
UR - https://www.scopus.com/inward/record.uri?eid=2-s2.0-66149189584&doi=10.1089%2fast.2007.0187&partnerID=40&md5=7f7eca403ebff20d65e46b894194dc0a
AD - Skidaway Institute of Oceanography, Savannah, GA 31411, 10 Ocean Science Circle, United States
AD - Carnegie Institution of Washington, Geophysical Laboratory, Washington, DC, United States
AB - Availability of reduced nitrogen is considered a prerequisite for the genesis of life from prebiotic precursors. Most atmospheric and oceanic models for the Hadean Earth predict a mildly oxidizing environment that is conducive to the formation and stability of only oxidized forms of nitrogen. A possible environment where reduction of oxidized nitrogen to ammonium has been speculated to occur is aqueous hydrothermal systems. We examined a suite of transition metal oxides and sulfides for their ability to reduce nitrate and nitrite, as well as oxidize ammonia, under hot (300°C) high-pressure (50-500 MPa) aqueous conditions. In general, iron sulfides exhibited the most rapid and complete conversion noted, followed by nickel and copper sulfides to a much lower degree. Of the oxides examined, only magnetite exhibited any ability to reduce NO 3 - or NO 2 -. Ammonium was stable or exhibited small losses (<20) in contact with all the mineral phases and conditions tested. The results support the idea that hydrothermal systems could have provided significant amounts of reduced nitrogen to their immediate environments. The enhanced availability of reduced nitrogen in hydrothermal systems also has important implications for prebiotic metabolic pathways where nitrogen availability is critical to the production of amino acids and other nitrogenous compounds. © 2008 Mary Ann Liebert, Inc..
KW - Ammonia
KW - Computer Simulation
KW - Minerals
KW - Nitrates
KW - Nitrites
KW - Nitrogen
KW - Nitrogen Oxides
KW - Oxidation-Reduction
KW - Oxides
KW - Pressure
KW - Quaternary Ammonium Compounds
KW - Sulfides

KW - Temperature
KW - Transition Elements
KW - Water
KW - ammonia
KW - mineral
KW - nitrate
KW - nitrite
KW - nitrogen
KW - nitrogen oxide
KW - oxide
KW - quaternary ammonium derivative
KW - sulfide
KW - transition element
KW - water
KW - article
KW - chemistry
KW - computer simulation
KW - oxidation reduction reaction
KW - pressure
KW - temperature
KW - Ammonia, Astrobiology 8, 1113?1126
KW - Hydrothermal
KW - Nitrogen
KW - Prebiotic
KW - Sulfides
SN - 15311074 (ISSN)
C2 - 19191539
LA - English
J2 - Astrobiology
M3 - Article
DB - Scopus
N1 - Export Date: 23 February 2023; Cited By: 23; Correspondence Address: J. A. Brandes; Skidaway Institute of Oceanography, Savannah, GA 31411, 10 Ocean Science Circle, United States; email: jay.brandes@skio.usg.edu
ER -

TY - JOUR
AU - Horneck, G.
TI - Astrobiological aspects of Mars and human presence: Pros and cons
PY - 2008
T2 - Hippokratia
VL - 12
IS - SUPPL. 1
SP - 49
EP - 52
UR - https://www.scopus.com/inward/record.uri?eid=2-s2.0-52949097626&partnerID=40&md5=7f691b0f418a674221153eecf65b5013
AD - Deutsches Zentrum für Luft- und Raumfahrt, Institut für Luft- und Raumfahrtmedizin, D51170 Köln, Germany
AD - Deutsches Zentrum fór Luft- und Raumfahrt, Institut fór Luft- und Raumfahrtmedizin, 51170 Koln, Germany
AB - After the realization of the International Space Station,

human exploratory missions to Moon or Mars, i.e. beyond low Earth
orbit, are widely considered as the next logical step of peaceful
cooperation in space on a global scale. Besides the human desire to
extend the window of habitability, human exploratory missions are
driven by several aspects of science, technology, culture and
economy. Mars is currently considered as a major target in the
search for life beyond the Earth. Understanding the history of water
on Mars appears to be one of the clues to the puzzle on the
probability of life on Mars. On Earth microorganisms have flourished
for more than 3.5 Ga and have developed strategies to cope with so-
called extreme conditions (e.g., hot vents, permafrost, subsurface
regions, rocks or salt crystals). Therefore, in search for life on
Mars, microorganisms are the most likely candidates for a putative
biota on Mars and the search for morphological or chemical
signatures of life or its relics is one of the primary and most
exciting goals of Mars exploration. The presence of humans on the
surface of Mars will substantially increase this research potential,
e.g., by supporting deep subsurface drilling and by allowing
intellectual collection and sophisticated in situ analysis of
samples of astrobiological interest. On the other hand, such long-
duration missions beyond LEO will add a new dimension to human space
flight, concerning the distance of travel, the radiation
environment, the gravity levels, the duration of the mission, and
the level of confinement and isolation the crew will be exposed to.
This will raise the significance of several health issues, above all
radiation protection, gravity related effects as well as
psychological issues. Furthermore, the import of internal and
external microorganisms inevitably accompanying any human mission to
Mars, or brought purposely to Mars as part of a bioregenerative life
support system needs careful consideration with regard to planetary
protection issues. Therefore, before planning any human exploratory
mission, the critical issues concerning human health and wellbeing
as well as protection of Mars in its pristine condition need to be
investigated.
KW - article
KW - astronomy
KW - biota
KW - economic aspect
KW - gravity
KW - microclimate
KW - permafrost
KW - probability
KW - psychological aspect
KW - radiation protection
KW - space flight
KW - Astrobiology
KW - Human exploratory missions
KW - Mars
KW - Search for extraterrestrial life
SN - 11084189 (ISSN)
LA - English
J2 - Hippokratia
M3 - Article
DB - Scopus

N1 - Export Date: 23 February 2023; Cited By: 7; Correspondence Address: G. Horneck; Deutsches Zentrum fór Luft- und Raumfahrt, Institut fór Luft- und Raumfahrtmedizin, 51170 Koln, Germany; email: gerda.horneck@dlr.de
ER -

TY - CONF
AU - Johnson, L.
AU - Gilbert, P.
TI - NASA'S discovery program: Moving toward the edge (of the solar system)
PY - 2008
T2 - JBIS - Journal of the British Interplanetary Society
VL - 61
IS - 8
SP - 300
EP - 303
UR - https://www.scopus.com/inward/record.uri?eid=2-s2.0-47749145684&partnerID=40&md5=9e2da08f41dfb726f0dab60b3ad37036
AD - NASA George C. Marshall Space Flight Center, Huntsville, AL 35812, United States
AB - The goal of NASA's Discovery program is to launch many smaller missions with fast development times to increase our understanding of the solar system. Discovery missions are solicited from the broad planetary science community approximately every 2 years. Active missions within the program include several with direct scientific or engineering connections to potential future missions to the edge of the solar system and beyond. The Dawn mission, launched in late 2007, uses ion drive as its primary propulsion system. Ion propulsion is one of only two technologies that appear feasible for early interstellar precursor missions with practical flight times. The Kepler mission will explore the structure and diversity of extrasolar planetary systems, with an emphasis on the detection of Earth-size planets around other stars. Kepler will survey nearby solar systems searching for planets that may fall within the 'habitable zone,' a region surrounding a star within which liquid water may exist on a planet's surface - an essential ingredient for life as we know it. In addition, the first New Frontiers mission is the New Horizons mission to Pluto, which will explore this 38-AU distant dwarf planet and potentially some Kuiper Belt objects beyond.
KW - Extrasolar planets
KW - NASA
KW - Solar system
KW - Stars
KW - Dawn missions
KW - Discovery mission
KW - Discovery program
KW - Extraterrestrial life
KW - Kepler mission
KW - Kuiper belt objects
KW - Planetary science
KW - Primary propulsion
KW - Manned space flight

KW - Dawn mission
KW - Discovery program
KW - Extraterrestrial life
KW - Kepler mission
SN - 0007084X (ISSN)
LA - English
J2 - JBIS J. Br. Interplanet. Soc.
M3 - Conference paper
DB - Scopus
N1 - Export Date: 23 February 2023; Cited By: 0; Correspondence Address: L. Johnson; NASA George C. Marshall Space Flight Center, Huntsville, AL 35812, United States; email: c.les.johnson@nasa.gov
ER -

TY - JOUR
AU - Fernández-Remolar, D.C.
AU - Prieto-Ballesteros, O.
AU - Rodríguez, N.
AU - Gómez, F.
AU - Amils, R.
AU - Gómez-Elvira, J.
AU - Stoker, C.R.
TI - Underground habitats in the Río tinto basin: A model for subsurface life habitats on mars
PY - 2008
T2 - Astrobiology
VL - 8
IS - 5
SP - 1023
EP - 1047
DO - 10.1089/ast.2006.0104
UR - https://www.scopus.com/inward/record.uri?eid=2-s2.0-58049126240&doi=10.1089%2fast.2006.0104&partnerID=40&md5=e92a487cd71b2efecf18cb874c4f5c56
AD - Centro de Astrobiología (INTA-CSIC), Torrejón de Ardoz, Madrid, Spain
AD - Centro de Biología Molecular, Universidad Autónoma de Madrid, Cantoblanco, Madrid, Spain
AD - NASA Ames Research Center, Moffet Field, CA, United States
AD - Centro de Astrobiologa, INTA-CSIC, 28850 Torrejn de Ardoz, Ctra Ajalvir km. 4, Spain
AB - A search for evidence of cryptic life in the subsurface region of a fractured Paleozoic volcanosedimentary deposit near the source waters of the Río Tinto River (Iberian pyrite belt, southwest Spain) was carried out by Mars Astrobiology Research and Technology Experiment (MARTE) project investigators in 2003 and 2004. This conventional deep-drilling experiment is referred to as the MARTE ground truth drilling project. Boreholes were drilled at three sites, and samples from extracted cores were analyzed with light microscopy, scanning electron microscopy-energy dispersive spectroscopy (SEM-EDS), X-ray diffraction (XRD), and Fourier transform infrared spectroscopy. Core leachates were analyzed with ion chromatography, and borehole fluids were analyzed with ion and gas chromatography. Key variables of the groundwater system (e.g.,

pO2, pH, and salinity) exhibit huge ranges probably due to surficial oxygenation of overall reducing waters, physical mixing of waters, and biologically mediated water-rock interactions. Mineral distribution is mainly driven by the pH of subsurface solutions, which range from highly acidic to neutral. Borehole fluids contain dissolved gases such as CO2, CH4, and H2. SEM-EDS analyses of core samples revealed evidence of microbes attacking pyrite. The Río Tinto alteration mechanisms may be similar to subsurface weathering of the martian crust and provide insights into the possible (bio)geochemical cycles that may have accompanied underground habitats in extensive early Mars volcanic regions and associated sulfide ores. © 2008 Mary Ann Liebert, Inc.
KW - Ecosystem
KW - Exobiology
KW - Extraterrestrial Environment
KW - Geologic Sediments
KW - Hydrogen-Ion Concentration
KW - Mars
KW - Microscopy, Electron, Scanning
KW - Minerals
KW - Models, Biological
KW - Spain
KW - X-Ray Diffraction
KW - mineral
KW - article
KW - astronomy
KW - biological model
KW - biology
KW - ecosystem
KW - microclimate
KW - pH
KW - scanning electron microscopy
KW - sediment
KW - Spain
KW - X ray diffraction
KW - Mars analogue
KW - Río Tinto
KW - Subsurface habitats
SN - 15311074 (ISSN)
C2 - 19105758
LA - English
J2 - Astrobiology
M3 - Article
DB - Scopus
N1 - Export Date: 23 February 2023; Cited By: 64; Correspondence Address: D. C. Fernández-Remolar; Centro de Astrobiologa, INTA-CSIC, 28850 Torrejn de Ardoz, Ctra Ajalvir km. 4, Spain; email: fernandezrd@inta.es
ER -

TY - JOUR
AU - Lamb, M.P.
AU - Dietrich, W.E.
AU - Aciego, S.M.

AU - DePaolo, D.J.
AU - Manga, M.
TI - Formation of Box Canyon, Idaho, by megaflood: Implications for seepage erosion on earth and mars
PY - 2008
T2 - Science
VL - 320
IS - 5879
SP - 1067
EP - 1070
DO - 10.1126/science.1156630
UR - https://www.scopus.com/inward/record.uri?eid=2-s2.0-44449127707&doi=10.1126%2fscience.1156630&partnerID=40&md5=1f7e918816b4114e2a0f7029673554bc
AD - Department of Earth and Planetary Science, University of California, Berkeley, CA 94720-4768, United States
AB - Amphitheater-headed canyons have been used as diagnostic indicators of erosion by groundwater seepage, which has important implications for landscape evolution on Earth and astrobiology on Mars. Of perhaps any canyon studied, Box Canyon, Idaho, most strongly meets the proposed morphologic criteria for groundwater sapping because it is incised into a basaltic plain with no drainage network upstream, and approximately 10 cubic meters per second of seepage emanates from its vertical headwall. However, sediment transport constraints, 4He and 14C dates, plunge pools, and scoured rock indicate that a megaflood (greater than 220 cubic meters per second) carved the canyon about 45,000 years ago. These results add to a growing recognition of Quaternary catastrophic flooding in the American northwest, and may imply that similar features on Mars also formed by floods rather than seepage erosion.
KW - Geography
KW - Geologic Sediments
KW - Idaho
KW - Mars
KW - Natural Disasters
KW - Time
KW - Water
KW - Box Canyon
KW - Colorado
KW - Idaho
KW - North America
KW - United States
KW - carbon 14
KW - ground water
KW - helium
KW - water
KW - basalt
KW - canyon
KW - Earth
KW - erosion
KW - flooding
KW - landscape evolution
KW - Mars
KW - sediment transport

KW - seepage
KW - article
KW - astronomy
KW - flooding
KW - geomorphology
KW - landscape
KW - priority journal
KW - Quaternary (period)
KW - sediment
KW - soil erosion
KW - United States
KW - disaster
KW - geography
KW - sediment
KW - time
SN - 10959203 (ISSN)
C2 - 18497296
LA - English
J2 - Science
M3 - Article
DB - Scopus
N1 - Export Date: 23 February 2023; Cited By: 103; Correspondence Address: M. P. Lamb; Department of Earth and Planetary Science, University of California, Berkeley, CA 94720-4768, United States; email: mpl@berkeley.edu; CODEN: SCIEA
ER -

TY - JOUR
AU - Parro, V.
AU - Fernández-Calvo, P.
AU - Rodríguez Manfredi, J.A.
AU - Moreno-Paz, M.
AU - Rivas, L.A.
AU - García-Villadangos, M.
AU - Bonaccorsi, R.
AU - González-Pastor, J.E.
AU - Prieto-Ballesteros, O.
AU - Schuerger, A.C.
AU - Davidson, M.
AU - Gómez-Elvira, J.
AU - Stoker, C.R.
TI - SOLID2: An antibody array-based life-detector instrument in a Mars Drilling Simulation Experiment (MARTE)
PY - 2008
T2 - Astrobiology
VL - 8
IS - 5
SP - 987
EP - 999
DO - 10.1089/ast.2007.0126
UR - https://www.scopus.com/inward/record.uri?eid=2-s2.0-58049120497&doi=10.1089%2fast.2007.0126&partnerID=40&md5=d1db2c75213ce830632195ea7a1d41a5
AD - Centro de Astrobiología (INTA-CSIC), Torrejón de Ardoz,

Madrid, Spain, Spain
AD - Department of Plant Pathology, Kennedy Space Center, University of Florid, FL, United States
AD - NASA Ames Research Center, Moffett Field, California, United States
AD - Department of Geosciences, Princeton University, Princeton, NJ, United States
AD - Carretera de Ajalvir Km 4 (INTA), Torrejón de Ardoz 28850 Madrid, Spain
AB - A field prototype of an antibody array-based life-detector instrument, Signs Of LIfe Detector (SOLID2), has been tested in a Mars drilling mission simulation called MARTE (Mars Astrobiology Research and Technology Experiment). As one of the analytical instruments on the MARTE robotic drilling rig, SOLID2 performed automatic sample processing and analysis of ground core samples (0.5 g) with protein microarrays that contained 157 different antibodies. Core samples from different depths (down to 5.5 m) were analyzed, and positive reactions were obtained in antibodies raised against the Gram-negative bacterium Leptospirillum ferrooxidans, a species of the genus Acidithiobacillus (both common microorganisms in the Río Tinto area), and extracts from biofilms and other natural samples from the Río Tinto area. These positive reactions were absent when the samples were previously subjected to a high-temperature treatment, which indicates the biological origin and structural dependency of the antibody-antigen reactions. We conclude that an antibody array-based life-detector instrument like SOLID2 can detect complex biological material, and it should be considered as a potential analytical instrument for future planetary missions that search for life. © 2008 Mary Ann Liebert, Inc. 2008.
KW - Antibodies
KW - Antigens
KW - Bacillus subtilis
KW - DNA
KW - Exobiology
KW - Fluorescence
KW - Immunoassay
KW - Laboratories
KW - Mars
KW - Protein Array Analysis
KW - Reproducibility of Results
KW - Space Simulation
KW - Acidithiobacillus
KW - Leptospirillum ferrooxidans
KW - Negibacteria
KW - antibody
KW - antigen
KW - DNA
KW - article
KW - astronomy
KW - Bacillus subtilis
KW - biology
KW - fluorescence
KW - immunoassay
KW - immunology

KW - instrumentation
KW - laboratory
KW - methodology
KW - protein microarray
KW - reproducibility
KW - space flight
KW - Antibody microarray
KW - MARTE project
KW - Río Tinto subsurface as Mars analogue
KW - SOLID instrument
SN - 15311074 (ISSN)
C2 - 19105755
LA - English
J2 - Astrobiology
M3 - Article
DB - Scopus
N1 - Export Date: 23 February 2023; Cited By: 45; Correspondence Address: V. Parro; Carretera de Ajalvir Km 4 (INTA), Torrejón de Ardoz 28850 Madrid, Spain; email: parrogv@inta.es
ER -

TY - CONF
AU - Tremberger Jr., G.
AU - Holden, T.
AU - Cheung, E.
AU - Subramaniam, R.
AU - Sullivan, R.
AU - Schneider, P.
AU - Flamholz, A.
AU - Marchese, P.
AU - Lieberman, D.
AU - Cheung, T.
TI - Nucleotide fluctuation of the RecA repair gene in Siberian permafrost Psychrobacter cryohalolentis K5
PY - 2008
T2 - Proceedings of SPIE - The International Society for Optical Engineering
VL - 7097
C7 - 70970U
DO - 10.1117/12.794390
UR - https://www.scopus.com/inward/record.uri?eid=2-s2.0-55549130245&doi=10.1117%2f12.794390&partnerID=40&md5=fea6a3922ef039c6dd4958cd597bfd20
AD - CUNY Queensborough Community College, Bayside, NY 11364, 222-05 56th Ave., United States
AB - A nucleotide sequence can be expressed as a numerical sequence when each nucleotide is assigned its proton number. A resulting gene numerical sequence can be investigated for its fractal dimension in terms of evolution and chemical properties for comparative studies. We have investigated such nucleotide fluctuation in the RecA repair gene of Psychrobacter cryohalolentis K5, Psychrobacter arcticus 273-4, and Psychrobacter sp. PRwf-1. The fractal dimension was found to correlate with the gene's operating temperature with the highest fractal dimension associated with P. cryohalolentis K5 living at the

low temperatures found in Siberian permafrost. The CpG dinucleotide content was found to be about 5% for the three species of Psychrobacters, which is substantially lower than that of Deinococcus radiodurans at about 12%. The average nucleotide pair-wise free energy was found to be lowest for Psychrobacter sp. PRwf-1, the species with the lowest fractal dimension of the three, consistent with the recent finding that Psychrobacter sp. PRw-f1 has a temperature growth maximum of 15–20°C higher than P. arcticus 273-4 and P. cryohaloentis K5. The results suggest that microbial vitality in extreme environments is associated with fractal dimension as well as high CpG dinucleotide content, while the average nucleotide pair-wise free energy is related to the operating environment. Evidence that extreme temperature operation would impose constraints measurable by Shannon entropy is also discussed. A quantitative estimate of an entropy-based measure having the characteristics of a mechanical pressure shows that the Psychrobacter RecA sequence experiences lower pressure than that of the human HAR1 sequence.
KW - Astrobiology
KW - Chemical properties
KW - Entropy
KW - Fractals
KW - Free energy
KW - Maintenance
KW - Nucleic acids
KW - Nucleotides
KW - Partial discharges
KW - Permafrost
KW - Comparative studies
KW - Deinococcus radiodurans
KW - Dinucleotide
KW - Extreme environments
KW - Extreme temperatures
KW - Low temperatures
KW - Lower pressures
KW - Mechanical pressures
KW - Nucleotide pairs
KW - Nucleotide sequence
KW - Nucleotide sequences
KW - Numerical sequences
KW - Operating environments
KW - Operating temperatures
KW - Psychrobacter
KW - Quantitative estimates
KW - RecA
KW - Shannon entropies
KW - Temperature growths
KW - Fractal dimension
KW - Entropy
KW - Fractal
KW - Free energy
KW - Nucleotide sequence
KW - Psychrobacter
KW - RecA

SN - 0277786X (ISSN); 978-081947317-2 (ISBN)
LA - English
J2 - Proc SPIE Int Soc Opt Eng
M3 - Conference paper
DB - Scopus
N1 - Export Date: 23 February 2023; Cited By: 3; CODEN: PSISD
ER -

TY - CONF
AU - Akin, D.L.
AU - Carignan, C.R.
AU - Roderick, S.
TI - Autonomous dexterous sampling: From the arctic ocean to mars and beyond
PY - 2008
T2 - Earth and Space Conference 2008: Proceedings of the 11th Aerospace Division International Conference on Engineering, Science, Construction, and Operations in Challenging Environments
VL - 323
UR - https://www.scopus.com/inward/record.uri?eid=2-s2.0-70349379086&partnerID=40&md5=0070241b9233b1536019ed0b8800feeb
AD - Space Systems Laboratory, University of Maryland, United States
AB - Under support from the NASA Astrobiology Science and Technology for Exploring the Planets (ASTEP) program, the University of Maryland designed and developed a deep-submergence dexterous sampling system, designed to operate from an autonomous undersea vehicle (AUV) at depths up to 6000 meters. The system was comprised of the Subsea Arctic Manipulator for Underwater Retrieval and Autonomous Interventions (SAMURAI), a six degree-of-freedom dexterous electrically-driven manipulator, and the Autonomous Vision Application for Target Acquisition and Ranging (AVATAR), an underwater stereo camera pair for target identification and localization. Working together, these systems would be brought to a hydrothermal vent field by a deep-submergence AUV specially designed for this task by Woods Hole under the ASTEP program. Once within the target vent field, the AVATAR system would locate targets for sampling, which might include biological or geological targets, based on size, shape, and color as determined by mission scientists prior to the AUV sortie. The stereo cameras on AVATAR register the local targets, calculate their positions in space relative to the carrier vehicle, and direct SAMURAI to perform the sampling tasks. The manipulator performs the sampling using one of a suite of potential end effectors picked prior to submergence, and stores the sample in one of several containers for the return trip to the surface. This paper details the engineering development and testing of the SAMURAI manipulator and AVATAR autonomous vision system. A number of developmental challenges were met and overcome, including severe limits on in-water weight and power available from the carrier AUV, selecting sensors for actuator positional feedback capable of operating at pressures up to 8000 psi, and keeping the system robust enough to survive the rough handling expected on shipboard. Extensive use was made of technologies developed for space flight in the SSL's Ranger Telerobotic Shuttle Experiment,

leading to the first-ever use of distributed "intelligent" actuator modules and a modular manipulator design for underwater applications. Although external events prevented the planned sea trials in 2006, work is ongoing to integrate SAMURAI/AVATAR to various undersea vehicles for operational use. This paper further discusses potential applications of the SAMURAI/AVATAR architectures and technologies to planetary exploration tasks beyond those of Europa-type oceans. Extensions of the Arctic sampling system would enable a number of high-priority near-term objectives in planetary exploration, most specifically a Mars sample collection and return mission. © 2008 ASCE.
KW - Actuators
KW - Astrobiology
KW - Cameras
KW - Earth (planet)
KW - Hot springs
KW - Interplanetary spacecraft
KW - Manipulators
KW - NASA
KW - Oceanography
KW - Space flight
KW - Stereo vision
KW - Submersibles
KW - Targets
KW - Arctic ocean
KW - Autonomous interventions
KW - Carrier vehicles
KW - Engineering development
KW - Hydrothermal vent
KW - Modular manipulators
KW - Operational use
KW - Planetary exploration
KW - Potential applications
KW - Sample collection
KW - Sampling systems
KW - Science and Technology
KW - Sea trial
KW - Six degree-of-freedom
KW - Stereo cameras
KW - Target acquisition
KW - Target identification
KW - Underwater application
KW - Underwater stereo
KW - University of Maryland
KW - Vision applications
KW - Vision systems
KW - Autonomous underwater vehicles
SN - 978-078440988-6 (ISBN)
LA - English
J2 - Proc. Int. Conf. Eng., Sci., Constr., Oper. Chall. Environ.
M3 - Conference paper
DB - Scopus
N1 - Export Date: 23 February 2023; Cited By: 3
ER -

TY - CONF
AU - Wilson, A.T.
TI - The perennially ice covered lakes of the cold and rainless deserts of the Antarctic, and by extension, Mars (Implications for finding Martian life)
PY - 2008
T2 - Proceedings of SPIE - The International Society for Optical Engineering
VL - 7097
C7 - 70970Y
DO - 10.1117/12.806579
UR - https://www.scopus.com/inward/record.uri?eid=2-s2.0-55549111858&doi=10.1117%2f12.806579&partnerID=40&md5=b96b205daff3b963ff62daaf6b79c4cd
AD - Dept. of Geosciences, University of Arizona, Tucson, AZ 85721, United States
AB - The salt geochemistry of Mars is predicted as an extrapolation of the salt geochemistry of the Dry (ice-free) Valleys in Antarctica. It is hard to escape the implication that there must be calcium/magnesium brine lakes in the enclosed drainage basins associated with the Northern Ice Cap. Because of the extreme cold these lakes will have acquired an ice cover. At the interface between the ice cover and the brine, one may find a thin layer of relatively fresh water. This might be the best and easiest place to look for Martian life.
KW - Arid regions
KW - Brines
KW - Catchments
KW - Earth sciences
KW - Geochemistry
KW - Ice
KW - Lakes
KW - Landforms
KW - Snow
KW - Water
KW - Exobiology
KW - Martian brines
KW - Martian exploration
KW - Martian fluvial processes
KW - Martian lakes
KW - Astrobiology
KW - Exobiology
KW - Martian brines
KW - Martian exploration
KW - Martian fluvial processes
KW - Martian lakes
SN - 0277786X (ISSN); 978-081947317-2 (ISBN)
LA - English
J2 - Proc SPIE Int Soc Opt Eng
M3 - Conference paper
DB - Scopus
N1 - Export Date: 23 February 2023; Cited By: 0; Correspondence Address: A. T. Wilson; Dept. of Geosciences, University of Arizona,

Tucson, AZ 85721, United States; email: atwilson@U.arizona.edu;
CODEN: PSISD
ER -

TY - CHAP
AU - Sotin, C.
AU - Prieur, D.
TI - Jupiter's Moon Europa: Geology and Habitability
PY - 2008
T2 - Complete Course in Astrobiology
SP - 253
EP - 271
DO - 10.1002/9783527618996.ch10
UR - https://www.scopus.com/inward/record.uri?eid=2-s2.0-84948663666&doi=10.1002%2f9783527618996.ch10&partnerID=40&md5=371b89921293c844a8b00fb07c445471
AB - After reviewing the current knowledge of the geological processes and the surface composition of Jupiter's moon Europa, this chapter concentrates on the geophysical data that suggest below the ice crust the presence of a deep ocean that would be in contact with a silicate core. Models of Europa's thermal evolution are then described. Based on the ocean hypothesis of Europa, the chapter then considers the astrobiological aspects of Europa and the perspectives of potential life at the interface between the ocean and the silicate core. Reference is given to the adaptability of terrestrial life to extreme environments. As outlook, some experiments are described that could be carried out onboard future missions to the Jupiter system and its moon Europa in order to resolve some of the important issues related to putative habitability in the outer Solar System. © 2007 WILEY-VCH Verlag GmbH & Co. KGaA. All rights reserved.
KW - Geology
KW - Silicates
KW - Deep ocean
KW - Extreme environment
KW - Future mission
KW - Geological process
KW - Geophysical data
KW - Jupiters
KW - Outer solar system
KW - Thermal evolution
KW - Moon
PB - wiley
SN - 978-352761899-6 (ISBN); 978-352740660-9 (ISBN)
LA - English
J2 - Complete Course in Astrobiol.
M3 - Book chapter
DB - Scopus
N1 - Export Date: 23 February 2023; Cited By: 4
ER -

TY - CONF
AU - Astafieva, M.M.
AU - Rozanov, A.Yu.

AU - Cornell, D.H.
AU - Hoover, R.B.
TI - Development of living organisms on the lava-water interface of Palaeoproterozoic Ongeluk lavas of South Africa
PY - 2008
T2 - Proceedings of SPIE - The International Society for Optical Engineering
VL - 7097
C7 - 709704
DO - 10.1117/12.791901
UR - https://www.scopus.com/inward/record.uri?eid=2-s2.0-55549135331&doi=10.1117%2f12.791901&partnerID=40&md5=ff5e4303e2423fd0aab877871e2a1708
AD - Paleontological Institute, Russian Academy of Sciences, 117647 Moscow, Profsoyusnaya 123, Russian Federation
AD - Department of Earth Sciences, Gothenburg University, SE-405 30, Box 460, Sweden
AD - NASA/National Space Science and Technology Center, Huntsville, AL 35802, 320 Sparkman Dr., United States
AB - An investigation of the Early Proterozoic pillow lavas of South-Africa shows that the lava-water boundary is very interesting from the point of view of bacterial paleontology. In the pillow selvages corresponding to this boundary, forms such as bacteria (including cyanobacteria) developed. Cyanobacterial or bacterial mats formed and probably even such highly organized forms as eukaryotes existed.
KW - Astrobiology
KW - Biology
KW - Bacterial mats
KW - Boundary water-lava
KW - Cyanobacteria
KW - Early Proterozoic, pillow-lavas
KW - Eukaryotes
KW - Living organisms
KW - Proterozoic
KW - South Africa
KW - Water interfaces
KW - Bacteriology
KW - Bacteria
KW - Boundary water-lava
KW - Cyanobacteria
KW - Early Proterozoic, pillow-lavas
KW - Eukaryotes
SN - 0277786X (ISSN); 978-081947317-2 (ISBN)
LA - English
J2 - Proc SPIE Int Soc Opt Eng
M3 - Conference paper
DB - Scopus
N1 - Export Date: 23 February 2023; Cited By: 6; Correspondence Address: M. M. Astafieva; Paleontological Institute, Russian Academy of Sciences, 117647 Moscow, Profsoyusnaya 123, Russian Federation; email: astafieva@paleo.ru; CODEN: PSISD
ER -

TY - JOUR
AU - Taverna, M.A.
AU - Morring Jr., F.
TI - Bringing it home
PY - 2008
T2 - Aviation Week and Space Technology (New York)
VL - 169
IS - 6
SP - 62
EP - 63
UR - https://www.scopus.com/inward/record.uri?eid=2-s2.0-49649121316&partnerID=40&md5=adb9e736203d965ab2a70b90b3f1fac6
AB - Teams of scientists and engineers are looking forward to an international mission to bring back soil and rock samples from Mars to continue the search for life on Mars. A group of experts from the US, Canada, Europe, Japan, and Australia have issued a report that concludes that such a mission would be scientifically justified and technically feasible, based on know-how currently available or in the near-term. The findings of NASA's Mars Phoenix lander, couples with earlier work by NASA's follow-the-water series of orbiters and landers and expected results from the upcoming Mars Science Laboratory (MSL) and ExoMars missions, will ensure a key role for astrobiology in the mission. The International Mars Architecture for the Return of Samples (iMARS) scenarios envisions a mission go-ahead in 2013, after several years spent developing enabling technologies, with a launch around 2020-22 and return to Earth three years later.
SN - 00052175 (ISSN)
LA - English
J2 - Aviat. Week Space Technol. (New York)
M3 - Article
DB - Scopus
N1 - Export Date: 23 February 2023; Cited By: 1; CODEN: AWSTA
ER -

TY - JOUR
AU - Raulin, F.
TI - Astrobiology and habitability of Titan
PY - 2008
T2 - Space Science Reviews
VL - 135
IS - 1-4
SP - 37
EP - 48
DO - 10.1007/s11214-006-9133-7
UR - https://www.scopus.com/inward/record.uri?eid=2-s2.0-46749099729&doi=10.1007%2fs11214-006-9133-7&partnerID=40&md5=8f48db62b24ff86fd9622e0e8f7dae44
AD - Laboratoire Interuniversitaire des Systèmes Atmosphériques, LISA-UMR CNRS 7583, Universités Paris 7 et Paris 12, F-94000 Créteil, 61 Avenue du Général de Gaulle, France
AB - Largest satellite of Saturn and the only in the solar system having a dense atmosphere, Titan is one of the key planetary bodies for astrobiological studies, due to several aspects. (i) Its analogies with planet Earth, in spite of much lower temperatures,

with, in particular, a methane cycle on Titan analogous to the water cycle on Earth. (ii) The presence of an active organic chemistry, involving several of the key compounds of prebiotic chemistry. The recent data obtained from the Huygens instruments show that the complex organic matter in Titan's low atmosphere is mainly concentrated in the aerosol particles. The formation of biologically interesting compounds may also occur in the deep water ocean, from the hydrolysis of complex organic material included in the chrondritic matter accreted during the formation of Titan. (iii) The possible emergence and persistence of Life on Titan. All ingredients which seem necessary for Life to appear and even develop - liquid water, organic matter and energy - are present on Titan. Consequently, it cannot be excluded that life may have emerged on or in Titan. In spite of the extreme conditions in this environment life may have been able to adapt and to persist. Many data are still expected from the Cassini-Huygens mission and future astrobiological exploration mission of Titan are now under consideration. Nevertheless, Titan already looks like another world, with an active organic chemistry, in the absence of permanent liquid water, on the surface: a natural laboratory for prebiotic-like chemistry. © 2007 Springer Science+Business Media, Inc.
KW - Astrobiology
KW - Biogeochemistry
KW - Biological materials
KW - Computer networks
KW - Earth (planet)
KW - Fluids
KW - Food products
KW - Interplanetary flight
KW - Methane
KW - Mixtures
KW - Organic compounds
KW - Satellites
KW - Solar energy
KW - Solar system
KW - Tellurium compounds
KW - (1 1 0) surface
KW - Aerosol particles
KW - Business media
KW - Cassini-Huygens mission
KW - Deep waters
KW - exploration missions
KW - Extreme conditions
KW - Huygens (Spacecraft)
KW - liquid water
KW - Organic Chemistry
KW - Organic materials
KW - Organic matter (OM)
KW - planet earth
KW - Planetary bodies
KW - prebiotic chemistry
KW - Springer (CO)
KW - Water cycling
KW - Chemistry

KW - Astrobiology
KW - Cassini-Huygens
KW - Prebiotic chemistry
KW - Primitive Earth
KW - Tholins
KW - Titan
SN - 15729672 (ISSN)
LA - English
J2 - Space Sci Rev
M3 - Article
DB - Scopus
N1 - Export Date: 23 February 2023; Cited By: 41; Correspondence Address: F. Raulin; Laboratoire Interuniversitaire des Systèmes Atmosphériques, LISA-UMR CNRS 7583, Universités Paris 7 et Paris 12, F-94000 Créteil, 61 Avenue du Général de Gaulle, France; email: raulin@lisa.univ-paris12.fr; CODEN: SPSRA
ER -

TY - JOUR
AU - Pizent, A.
AU - Macan, J.
AU - Jurasović, J.
AU - Varnai, V.M.
AU - Milković-Kraus, S.
AU - Kanceljak-Macan, B.
TI - Association of toxic and essential metals with atopy markers and ventilatory lung function in women and men
PY - 2008
T2 - Science of the Total Environment
VL - 390
IS - 2-3
SP - 369
EP - 376
DO - 10.1016/j.scitotenv.2007.10.049
UR - https://www.scopus.com/inward/record.uri?eid=2-s2.0-37149030156&doi=10.1016%2fj.scitotenv.2007.10.049&partnerID=40&md5=76dfa991f06b59e85055c98e9f469cca
AD - Institute for Medical Research and Occupational Health, Zagreb, Croatia
AB - The association of age, smoking, alcohol, superoxide dismutase (SOD), glutathione peroxidase (GPx), blood lead (BPb) and cadmium (BCd) levels, and serum levels of copper (SCu), zinc (SZn) and selenium (SSe) with atopic status and ventilatory function was examined in the groups of 166 women and 50 men with no occupational exposure to metals or other xenobiotics. Markers of atopy included serum total IgE, skin prick test (SPT) to common inhalatory allergens, non-specific nasal reactivity (NNR) and non-specific bronchial reactivity (NBR). Parameters of ventilatory function included forced vital capacity (FVC) and forced expiratory volume in the first second (FEV1). Significantly higher BPb, SZn, IgE and prevalence of positive SPT, and lower SCu and NNR was found in men than in women. Fifteen women taking female sex hormones (HT) had significantly higher SCu than women without HT. Regression models showed significant inverse associations between IgE and SCu (P =

0.021) and NNR and SCu (P = 0.044) in women. When excluding women with HT, the association of SCu and total IgE became of borderline significance (P = 0.051), association between SCu and NNR disappeared, and significant positive association between total IgE and BPb emerged (P = 0.046). In men, significant inverse association was found between positive SPT and SSe, and between NBR and SSe. A decrease in FVC% and FEV1% was associated with an increase in smoking intensity (P < 0.001) and a decrease in SZn (P = 0.043 and P = 0.053, respectively). These results were observed at the levels of the metals comparable to those in general populations worldwide. The observed differences between men and women may partly be explained by different levels of relevant toxic and essential metals, and their combination. The role of female HT in associations of atopy markers and SCu should be further investigated. © 2007 Elsevier B.V. All rights reserved.
KW - Adult
KW - Aged
KW - Bronchial Provocation Tests
KW - Cadmium
KW - Copper
KW - Female
KW - Forced Expiratory Volume
KW - Glutathione Peroxidase
KW - Humans
KW - Hypersensitivity, Immediate
KW - Immunoglobulin E
KW - Lead
KW - Male
KW - Metals, Heavy
KW - Middle Aged
KW - Respiratory Tract Diseases
KW - Selenium
KW - Skin Tests
KW - Superoxide Dismutase
KW - Urban Population
KW - Vital Capacity
KW - Zinc
KW - Astrobiology
KW - Biomarkers
KW - Enzymes
KW - Hormones
KW - Mathematical models
KW - Medical problems
KW - allergen
KW - cadmium
KW - copper
KW - glutathione peroxidase
KW - immunoglobulin E
KW - lead
KW - metal
KW - selenium
KW - sex hormone
KW - superoxide dismutase
KW - toxic substance

KW - zinc
KW - alcohol
KW - enzyme
KW - heavy metal
KW - sex hormone
KW - smoking
KW - toxic substance
KW - ventilation
KW - adult
KW - aged
KW - article
KW - atopy
KW - bronchus reactivity
KW - controlled study
KW - disease association
KW - female
KW - forced expiratory volume
KW - human
KW - human experiment
KW - lung function
KW - lung ventilation
KW - male
KW - population
KW - prevalence
KW - prick test
KW - priority journal
KW - regression analysis
KW - sex difference
KW - smoking
KW - Airway reactivity
KW - Allergens
KW - Anti-oxidant enzymes
KW - Atopy
KW - Atopy markers
KW - Glutathione peroxidase (GPx)
KW - Superoxide dismutase (SOD)
KW - Xenobiotics
KW - Toxic materials
KW - Airway reactivity
KW - Anti-oxidant enzymes
KW - Atopy
KW - Toxic and essential metals
KW - Ventilatory function
SN - 00489697 (ISSN)
C2 - 18045657
LA - English
J2 - Sci. Total Environ.
M3 - Article
DB - Scopus
N1 - Export Date: 23 February 2023; Cited By: 11; Correspondence Address: A. Pizent; Institute for Medical Research and Occupational Health, Zagreb, Croatia; email: apizent@imi.hr; CODEN: STEVA
ER -

TY - JOUR
AU - Horikawa, D.D.
AU - Kunieda, T.
AU - Abe, W.
AU - Watanabe, M.
AU - Nakahara, Y.
AU - Yukuhiro, F.
AU - Sakashita, T.
AU - Hamada, N.
AU - Wada, S.
AU - Funayama, T.
AU - Katagiri, C.
AU - Kobayashi, Y.
AU - Higashi, S.
AU - Okuda, T.
TI - Establishment of a rearing system of the extremotolerant tardigrade Ramazzottius varieornatus: A new model animal for astrobiology
PY - 2008
T2 - Astrobiology
VL - 8
IS - 3
SP - 549
EP - 556
DO - 10.1089/ast.2007.0139
UR - https://www.scopus.com/inward/record.uri?eid=2-s2.0-48849107154&doi=10.1089%2fast.2007.0139&partnerID=40&md5=406eed78966dcf113a0d18b0ca8685fc
AD - Graduate School of Environmental Earth Science, Hokkaido University, Sapporo, Japan
AD - Graduate School of Science, University of Tokyo, Tokyo, Japan
AD - Anhydrobiosis Research Unit, National Institute of Agrobiological Sciences, Tsukuba, Japan
AD - Insect-Microbe Research Unit, National Institute of Agrobiological Sciences, Tsukuba, Japan
AD - Microbeam Radiation Biology Group, Japan Atomic Energy Agency, Takasaki, Japan
AD - Department of Quantum Biology, Gunma University Graduate School of Medicine, Maebashi, Japan
AD - Institute of Low Temperature Science, Hokkaido University, Sapporo, Japan
AD - NASA Ames Research Center, Moffett Field, CA, United States
AD - NASA Ames Research Center, Mail Stop 239-20, Bldg. N239, Moffett Field, CA 94035, United States
AB - Studies on the ability of multicellular organisms to tolerate specific environmental extremes are relatively rare compared to those of unicellular microorganisms in extreme environments. Tardigrades are extremotolerant animals that can enter an ametabolic dry state called anhydrobiosis and have high tolerance to a variety of extreme environmental conditions, particularly while in anhydrobiosis. Although tardigrades have been expected to be a potential model animal for astrobiological studies due to their excellent anhydrobiotic and extremotolerant abilities, few studies of tolerance with cultured tardigrades have been reported, possibly

due to the absence of a model species that can be easily maintained under rearing conditions. We report the successful rearing of the herbivorous tardigrade, Ramazzottius varieornatus, by supplying the green alga Chlorella vulgaris as food. The life span was 35 ± 16.4 d, deposited eggs required 5.7 ± 1.1 d to hatch, and animals began to deposit eggs 9 d after hatching. The reared individuals of this species had an anhydrobiotic capacity throughout their life cycle in egg, juvenile, and adult stages. Furthermore, the reared adults in an anhydrobiotic state were tolerant of temperatures of 90°C and −196°C, and exposure to 99.8% acetonitrile or irradiation with 4000 Gy 4He ions. Based on their life history traits and tolerance to extreme stresses, R. varieornatus may be a suitable model for astrobiological studies of multicellular organisms. © 2008 Mary Ann Liebert, Inc.
KW - Adaptation, Physiological
KW - Animals
KW - Desiccation
KW - Environment
KW - Exobiology
KW - Life Cycle Stages
KW - Models, Animal
KW - Ovum
KW - Parasites
KW - Survival Analysis
KW - Time Factors
KW - Water
KW - Animalia
KW - Chlorella vulgaris
KW - Chlorophyta
KW - Ramazzottius
KW - Tardigrada
KW - water
KW - adaptation
KW - animal
KW - animal model
KW - article
KW - biology
KW - cytology
KW - desiccation
KW - environment
KW - growth, development and aging
KW - life cycle
KW - metabolism
KW - methodology
KW - oocyte
KW - parasite
KW - survival
KW - time
KW - ultrastructure
KW - Anhydrobiosis
KW - Extreme environments
KW - Extremophiles
KW - Ramazzottius varieornatus
KW - Tardigrades

KW - Tolerance
SN - 15311074 (ISSN)
C2 - 18554084
LA - English
J2 - Astrobiology
M3 - Article
DB - Scopus
N1 - Export Date: 23 February 2023; Cited By: 90; Correspondence Address: D. D. Horikawa; NASA Ames Research Center, Moffett Field, CA, United States; email: horikawa@biol.s.u-tokyo.ac.jp
ER -

TY - CONF
AU - Rozanov, A.Yu.
AU - Astafieva, M.M.
AU - Hoover, R.B.
TI - The Early Earth and its environments
PY - 2008
T2 - Proceedings of SPIE - The International Society for Optical Engineering
VL - 7097
C7 - 709708
DO - 10.1117/12.791902
UR - https://www.scopus.com/inward/record.uri?eid=2-s2.0-55549085198&doi=10.1117%2f12.791902&partnerID=40&md5=a0cf4a155a5632b12bb1edfc3780f14a
AD - Paleontological Institute, Russian Academy of Sciences, 117647 Moscow, Profsoyusnaya 123, Russian Federation
AD - NASA/National Space Science and Technology Center, Huntsville, AL 35802, 320 Sparkman Dr., United States
AB - The set of discovered Archaean organisms does not indicate anaerobic atmosphere as early as 3.5 Ga. The RNA-world must have existed at least at 3.9 GA and, likely, close to the beginning of degasification and appearance of water on the Earth surface. The earliest cellular organisms (membranes) also appeared before 4.0 Ga. Possibilities of evaluation of conditions on the Earth's surface are under the consideration of both geological-geochemical and possibly mainly paleontological data. The important element in the analysis of the Earth conditions is the level of organisms organization presented in the paleontological record, because it is connected with the existence of peculiar abiogenic conditions, such as temperature, amount of oxygen, amount of water etc.
KW - Analytical geochemistry
KW - Astrobiology
KW - Cell membranes
KW - Gallium
KW - Nucleic acids
KW - Oxygen
KW - RNA
KW - Water analysis
KW - Archaean
KW - Cyanobacteria
KW - Eucaryots
KW - Phosphorites

KW - Prasinophyceae
KW - Procaryots
KW - Proterozoic
KW - Residual soils
KW - Earth (planet)
KW - Archaean
KW - Bacteria
KW - Cyanobacteria
KW - Eucaryots
KW - Phosphorites
KW - Prasinophyceae
KW - Procaryots
KW - Proterozoic
KW - Residual soils
SN - 0277786X (ISSN); 978-081947317-2 (ISBN)
LA - English
J2 - Proc SPIE Int Soc Opt Eng
M3 - Conference paper
DB - Scopus
N1 - Export Date: 23 February 2023; Cited By: 6; CODEN: PSISD
ER -

TY - CONF
AU - Holden, T.
AU - Tremberger Jr., G.
AU - Cheung, E.
AU - Subramaniam, R.
AU - Sullivan, R.
AU - Schneider, P.
AU - Flamholz, A.
AU - Marchese, P.
AU - Hiciano, O.
AU - Yao, H.
AU - Lieberman, D.
AU - Cheung, T.
TI - Methanosarcina acetivorans 16S rRNA and transcription factor nucleotide fluctuation with implications in exobiology and pathology
PY - 2008
T2 - Proceedings of SPIE - The International Society for Optical Engineering
VL - 7097
C7 - 70970V
DO - 10.1117/12.794398
UR - https://www.scopus.com/inward/record.uri?eid=2-s2.0-55649098619&doi=10.1117%2f12.794398&partnerID=40&md5=8c77d3bf42d01171c43dc39eed29e322
AD - CUNY Queensborough Community College, Bayside, NY 11364, 222-05 56th Ave., United States
AB - Cultures of the methane-producing archaea Methanosarcina, have recently been isolated from Alaskan sediments. It has been proposed that methanogens are strong candidates for exobiological life in extreme conditions. The spatial environmental gradients, such as those associated with the polygons on Mars' surface, could have been produced by past methanogenesis activity. The 16S rRNA gene has been

used routinely to classify phenotypes. Using the fractal dimension of nucleotide fluctuation, a comparative study of the 16S rRNA nucleotide fluctuation in Methanosarcina acetivorans C2A, Deinococcus radiodurans, and E. coli was conducted. The results suggest that Methanosarcina acetivorans has the lowest fractal dimension, consistent with its ancestral position in evolution. Variation in fluctuation complexity was also detected in the transcription factors. The transcription factor B (TFB) was found to have a higher fractal dimension as compared to transcription factor E (TFE), consistent with the fact that a single TFB in Methanosarcina acetivorans can code three different TATA box proteins. The average nucleotide pair-wise free energy of the DNA repair genes was found to be highest for Methanosarcina acetivorans, suggesting a relatively weak bonding, which is consistent with its low prevalence in pathology. Multitasking capacity comparison of type-I and type-II topoisomerases has been shown to correlate with fractal dimension using the methicillin-resistant strain MRSA 252. The analysis suggests that gene adaptation in a changing chemical environment can be measured in terms of bioinformatics. Given that the radiation resistant Deinococcus radiodurans is a strong candidate for an extraterrestrial origin and that the cold temperature Psychrobacter cryohalolentis K5 can function in Siberian permafrost, the fractal dimension comparison in this study suggests that a chemical resistant methanogen could exist in extremely cold conditions (such as that which existed on early Mars) where demands on gene activity are low. In addition, the comparative study of the Methanococcoides burtonii cold shock domain sequence has provided further support for the correlation between multitasking capacity and fractal dimension.
KW - Astrobiology
KW - Bioinformatics
KW - Earth (planet)
KW - Escherichia coli
KW - Fractals
KW - Genes
KW - Methane
KW - Methanogens
KW - Multitasking
KW - Nucleic acids
KW - Nucleotides
KW - Organic acids
KW - Partial discharges
KW - Pathology
KW - Proteins
KW - Sedimentation
KW - Transcription
KW - Transcription factors
KW - Water pollution
KW - 16S rRNA
KW - 16S rRNA genes
KW - Archaea
KW - Chemical environments
KW - Chemical resistants
KW - Cold conditions

KW - Cold shocks
KW - Cold temperatures
KW - Comparative studies
KW - Deinococcus radiodurans
KW - Dna repairs
KW - Do-mains
KW - E. coli
KW - Environmental gradients
KW - Extraterrestrial origins
KW - Extreme conditions
KW - Gene activities
KW - Methanogenesis
KW - Methanosarcina
KW - Methanosarcina acetivorans
KW - Methicillin
KW - Nucleotide pairs
KW - Nucleotide sequence
KW - Radiation resistants
KW - Resistant strains
KW - TATA boxes
KW - Topoisomerase
KW - Topoisomerases
KW - Weak bondings
KW - Fractal dimension
KW - Archaea
KW - Fractal
KW - Methanosarcina
KW - Nucleotide sequence
KW - Topoisomerase
KW - Transcription factor sequence
SN - 0277786X (ISSN); 978-081947317-2 (ISBN)
LA - English
J2 - Proc SPIE Int Soc Opt Eng
M3 - Conference paper
DB - Scopus
N1 - Export Date: 23 February 2023; Cited By: 1; CODEN: PSISD
ER -

TY - JOUR
AU - Menor-Salván, C.
AU - Ruiz-Bermejo, M.
AU - Osuna-Esteban, S.
AU - Muñoz-Caro, G.
AU - Veintemillas-Verdaguer, S.
TI - Synthesis of polycyclic aromatic hydrocarbons and acetylene polymers in ice: A prebiotic scenario
PY - 2008
T2 - Chemistry and Biodiversity
VL - 5
IS - 12
SP - 2729
EP - 2739
DO - 10.1002/cbdv.200890228
UR - https://www.scopus.com/inward/record.uri?eid=2-

s2.0-59649093859&doi=10.1002%2fcbdv.200890228&partnerID=40&md5=c53a0a6824cb3ebaa1f7180ac16dc133
AD - Centro de Astrobiología, Consejo Superior de Investigaciones Científicas, Instituto Nacional de Técnica Aeroespacial, E-28850 Torrejón de Ardoz Madrid, Carretera Torrejón-Ajalvir, Km. 4,200, Spain
AB - The recent evidences of presence of subsurface oceans of liquid water and ice on Saturn's moons, and the possible presence and astrobiological importance of polycyclic aromatic hydrocarbons (PAHs) in these environments, provide strong motivation for the exploration of the prebiotic chemistry in ice and to test if PAHs could be experimentally synthesized in ice surfaces under atmospheres containing methane as carbon source. In this work, we present a new design for prebiotic-chemistry experiments in ice matrix. Using this design, a mixture of products including PAHs, polar aromatic compounds, and hydrophilic acetylene-based polymers was obtained. We propose that acetylene generation in a methane/ nitrogen atmosphere and subsequent polymerization to PAHs and polyynes could be a favored pathway in the presence of water freeze-melt cycles. These results shed light on the processes involved in PAH synthesis in icy environments and on the physical factors that drive the different competing pathways in methane/ nitrogen atmospheres. © 2008 Verlag Helvetica Chimica Acta AG, Zürich.
KW - Acetylene
KW - Gas Chromatography-Mass Spectrometry
KW - Ice
KW - Polycyclic Hydrocarbons, Aromatic
KW - Polymers
KW - Solid Phase Microextraction
KW - Surface Properties
KW - acetylene
KW - carbon
KW - ice
KW - methane
KW - nitrogen
KW - polycyclic aromatic hydrocarbon
KW - polymer
KW - article
KW - astronomy
KW - atmosphere
KW - chemical structure
KW - gas chromatography
KW - hydrophilicity
KW - mass spectrometry
KW - moon
KW - polymerization
KW - sea
KW - simulation
KW - solid phase microextraction
KW - synthesis
KW - chemistry
KW - mass fragmentography
KW - surface property
SN - 16121880 (ISSN)

C2 - 19089832
LA - English
J2 - Chem. Biodiversity
M3 - Article
DB - Scopus
N1 - Export Date: 23 February 2023; Cited By: 14; Correspondence Address: C. Menor-Salván; Centro de Astrobiología, Consejo Superior de Investigaciones Científicas, Instituto Nacional de Técnica Aeroespacial, E-28850 Torrejón de Ardoz Madrid, Carretera Torrejón-Ajalvir, Km. 4,200, Spain; email: menorsc@inta.es
ER -

TY - JOUR
AU - Horneck, G.
TI - The microbial case for Mars and its implication for human expeditions to Mars
PY - 2008
T2 - Acta Astronautica
VL - 63
IS - 7-10
SP - 1015
EP - 1024
DO - 10.1016/j.actaastro.2007.12.002
UR - https://www.scopus.com/inward/record.uri?eid=2-s2.0-48549091185&doi=10.1016%2fj.actaastro.2007.12.002&partnerID=40&md5=6a41986288d5ceb2569b72ef64c348f0
AD - Deutsches Zentrum für Luft- und Raumfahrt, Institut für Luft- und Raumfahrtmedizin, D51170 Köln, Germany
AB - Mars is considered as key target for the search of life beyond the Earth. As well as carbon based chemistry and an adequate energy source, water in liquid phase has been considered a prerequisite for habitability. By analogy with terrestrial extremophilic microbial communities, e.g., those thriving in arid, cold, salty environments and/or those exposed to intense UV radiation, potential oases on Mars are suggested. They are connected with areas where liquid water still exists under the current conditions, but also sulfur rich subsurface areas for chemoautotrophic communities, rocks for endolithic communities, permafrost regions, hydrothermal vents, soil or evaporite crusts are of interest. The presence of humans on the surface of Mars will substantially increase the research potential; however, prior to any human exploratory mission the critical issues concerning human health and wellbeing as well as planetary protection issues need to be investigated. © 2008 Elsevier Ltd. All rights reserved.
KW - Arsenic compounds
KW - Hot springs
KW - Liquid phase epitaxy
KW - Offshore oil well production
KW - Sulfur
KW - Ultraviolet radiation
KW - Carbon based
KW - Critical issues
KW - Energy sourcing
KW - Evaporite

KW - Exploratory missions
KW - Human health
KW - Hydrothermal vents
KW - Intense (CO)
KW - Liquid water
KW - Liquid-phase
KW - Microbial communities
KW - Permafrost regions
KW - Planetary Protection (PP)
KW - Research potential
KW - Salty environments
KW - Surface of Mars
KW - Wellbeing
KW - Animal cell culture
KW - Extraterrestrial life
KW - Extremophiles
KW - Human exploratory missions
KW - Mars
KW - Microbial communities
SN - 00945765 (ISSN)
LA - English
J2 - Acta Astronaut
M3 - Article
DB - Scopus
N1 - Export Date: 23 February 2023; Cited By: 17; Correspondence Address: G. Horneck; Deutsches Zentrum für Luft- und Raumfahrt, Institut für Luft- und Raumfahrtmedizin, D51170 Köln, Germany; email: gerda.horneck@dlr.de; CODEN: AASTC
ER -

TY - JOUR
AU - Villanueva, G.L.
AU - Mumma, M.J.
AU - Novak, R.E.
AU - Hewagama, T.
TI - Identification of a new band system of isotopic CO2 near 3.3 µm: Implications for remote sensing of biomarker gases on Mars
PY - 2008
T2 - Icarus
VL - 195
IS - 1
SP - 34
EP - 44
DO - 10.1016/j.icarus.2007.11.014
UR - https://www.scopus.com/inward/record.uri?eid=2-s2.0-42049087372&doi=10.1016%2fj.icarus.2007.11.014&partnerID=40&md5=0b9c80316620a334917590006088875d
AD - NASA Goddard Space Flight Center, Greenbelt, MD 20771, Mailstop 693, United States
AD - NASA Goddard Space Flight Center, Greenbelt, MD 20771, Mailstop 690.3, United States
AD - Department of Physics, Iona College, New Rochelle, NY 10801, United States
AD - Department of Astronomy, University of Maryland, College Park,

MD 20742-2421, United States
AB - We present the discovery of a new vibrational band system of isotopic CO2 (carbon dioxide) near 3.3 μm, with multiple strong P, Q and R lines in the prime spectral region used to search for Mars CH4 (methane). The band system was discovered on Mars using high-resolution spectrometers (λ / δ λ > 40, 000, CSHELL and NIRSPEC) at telescopes (NASA-IRTF and Keck-2) atop Mauna Kea, HI. The observed line intensities and frequencies agree very well with values predicted by a vibrational band model that we developed using known parameters for the molecular levels involved. Using this model, we synthesized spectra for different observing conditions (from Space and ground-based telescopes) and for different spectral resolving powers (5000 to 40, 000). Although the total atmospheric burden on Mars is more than 150 times smaller than on Earth, the greater mixing ratio of CO2 ensures that its column abundance on Mars is almost 20 times greater than on Earth. Thus, weak telluric CO2 band systems appear much stronger on Mars. Many molecules of possible biological and geothermal interest have strong signatures at these wavelengths, in particular hydrocarbons owing to their strong ro-vibrational C{single bond}H stretching modes. For example, the new isotopic CO2 band-system encompasses lines of CH4, C2H6 (ethane), CH3OH (methanol) and H2O (water). Implications for previous and future searches of biomarker gases are presented. © 2007 Elsevier Inc. All rights reserved.
KW - Astrobiology
KW - atmosphere
KW - Infrared observations
KW - Mars
KW - Spectroscopy
SN - 10902643 (ISSN)
LA - English
J2 - Icarus
M3 - Article
DB - Scopus
N1 - Export Date: 23 February 2023; Cited By: 56; Correspondence Address: G.L. Villanueva; NASA Goddard Space Flight Center, Greenbelt, MD 20771, Mailstop 693, United States; email: geronimo.villanueva@nasa.gov; CODEN: ICRSA
ER -

TY - JOUR
AU - Pallé, E.
AU - Ford, E.B.
AU - Seager, S.
AU - Montañés-Rodríguez, P.
AU - Vazquez, M.
TI - Identifying the rotation rate and the presence of dynamic weather on extrasolar earth-like planets from photometric observations
PY - 2008
T2 - Astrophysical Journal
VL - 676
IS - 2
SP - 1319

EP - 1329
DO - 10.1086/528677
UR - https://www.scopus.com/inward/record.uri?eid=2-s2.0-42649121633&doi=10.1086%2f528677&partnerID=40&md5=8e64eb77e905f09d9f10ed3bcfe3c613
AD - Instituto de Astrofisica de Canarias, La Laguna E-3 8200, Spain
AD - Department of Astronomy, University of Florida, 211 Bryant Space Science Center, Gainesville, FL 32611-2055, P.O. Box 112055, United States
AD - EAPS, Massachusetts Institute of Technology, Cambridge, MA 021394307, United States
AB - With the recent discoveries of hundreds of extrasolar planets, the search for planets like Earth and life in the universe is quickly gaining momentum. In the future, large space observatories could directly detect the light scattered from rocky planets, but they would not be able to spatially resolve a planet's surface. Using reflectance models and real cloud data from satellite observations, here we show that, despite Earth's dynamic weather patterns, the light scattered by the Earth to a hypothetical distant observer as a function of time contains sufficient information to accurately measure Earth's rotation period. This is because ocean currents and continents result in relatively stable averaged global cloud patterns. The accuracy of these measurements will vary with the viewing geometry and other observational constraints. If the rotation period can be measured with accuracy, data spanning several months could be coherently combined to obtain spectroscopic information about individual regions of the planetary surface. Moreover, deviations from a periodic signal can be used to infer the presence of relatively short-lived structures in its atmosphere (i.e., clouds). This could provide a useful technique for recognizing exoplanets that have active weather systems, changing on a timescale comparable to their rotation. Such variability is likely to be related to the atmospheric temperature and pressure being near a phase transition and could support the possibility of liquid water on the planet's surface. © 2008. The American Astronomical Society. All rights reserved.
KW - Astrobiology
KW - Earth
KW - Scattering
KW - Techniques: photometric
PB - Institute of Physics Publishing
SN - 0004637X (ISSN)
LA - English
J2 - Astrophys. J.
M3 - Article
DB - Scopus
N1 - Export Date: 23 February 2023; Cited By: 79; Correspondence Address: E. Pallé; Instituto de Astrofisica de Canarias, La Laguna E-3 8200, Spain; email: epalle@iac.es
ER -

TY - CHAP
AU - Stan-Lotter, H.

TI - Extremophiles, the Physicochemical Limits of Life(Growth and Survival)
PY - 2008
T2 - Complete Course in Astrobiology
SP - 121
EP - 150
DO - 10.1002/9783527618996.ch5
UR - https://www.scopus.com/inward/record.uri?eid=2-s2.0-84948672561&doi=10.1002%2f9783527618996.ch5&partnerID=40&md5=ba1b1b1f16ccf2bdba04ce2f8067fb90
AB - This chapter covers extreme environments, characterized by physical and chemical parameters that had been considered to lie outside the range suitable for life. In recent years, the notion of what constitutes life-limiting conditions in an environment has undergone dramatic changes: microorganisms and occasionally higher organisms have been discovered that not only tolerate but thrive under ranges of temperatures, pH values, pressures, salinity, ionizing radiation, etc., previously thought to destruct biomolecules and organisms. The presence of liquid water is a prerequisite for growth under all kinds of conditions, but survival of resting stages, such as spores or dormant forms, was shown to be possible under vacuum and in space for several months. The detection of numerous types of viable prokaryotes in subterranean locations, such as granite, ancient halite, and sediments, suggests the possibility of even more extensive longevity, namely, over geological time periods, i.e., over millions of years. The several-fold implications of these discoveries for the search for extraterrestrial life are discussed. © 2007 WILEY-VCH Verlag GmbH & Co. KGaA. All rights reserved.
KW - Ionizing radiation
KW - Sodium chloride
KW - Extraterrestrial life
KW - Extreme environment
KW - Extremophiles
KW - Geological time
KW - Limiting condition
KW - Liquid water
KW - pH value
KW - Physical and chemical parameters
KW - Microorganisms
PB - wiley
SN - 978-352761899-6 (ISBN); 978-352740660-9 (ISBN)
LA - English
J2 - Complete Course in Astrobiol.
M3 - Book chapter
DB - Scopus
N1 - Export Date: 23 February 2023; Cited By: 8
ER -

TY - JOUR
AU - Zheng, W.
AU - Jewitt, D.
AU - Osamura, Y.
AU - Kaiser, R.I.

TI - Formation of nitrogen and hydrogen-bearing molecules in solid ammonia and implications for solar system and interstellar ices
PY - 2008
T2 - Astrophysical Journal
VL - 674
IS - 2
SP - 1242
EP - 1250
DO - 10.1086/523783
UR - https://www.scopus.com/inward/record.uri?eid=2-s2.0-40249087204&doi=10.1086%2f523783&partnerID=40&md5=3101f1a49539b803de23e974815fe8dc
AD - Institute for Astronomy, University of Hawaii-Manoa, Honolulu, HI 96822, United States
AD - Department of Chemistry, University of Hawaii-Manoa, Honolulu, HI 96822, United States
AD - Department of Chemistry, Rikkyo University, Tokyo, 171-8501, Japan
AB - We irradiated solid ammonia (NH3) in the temperature range of 10-60 K with high-energy electrons to simulate the processing of ammonia-bearing ices in the interstellar medium and in the solar system. By monitoring the newly formed molecules online and in situ, the synthesis of hydrazine (N 2H4), diazene (N2H2 isomers), hydrogen azide (HN3), the amino radical (NH2), molecular hydrogen (H2), and molecular nitrogen (N2) has been confirmed. Our results show that the production rates of hydrazine, diazene, hydrogen azide, molecular hydrogen, and molecular nitrogen are higher in amorphous ammonia than those in crystalline ammonia; this behavior is similar to the production of molecular hydrogen, molecular oxygen, and hydrogen peroxide found in electron-irradiated water ices. However, the formation of hydrazine in crystalline ammonia does not show any temperature dependence. Our experimental results give hints to the origin of molecular nitrogen in the Saturnian system and possibly in the atmospheres of proto-Earth and Titan; our research may also guide the search of hitherto unobserved nitrogen-bearing molecules in the interstellar medium and in our solar system. © 2008. The American Astronomical Society. All rights reserved.
KW - Astrobiology
KW - Astrochemistry
KW - Cosmic rays
KW - Infrared: general
KW - Methods: laboratory planets and satellites: general
PB - Institute of Physics Publishing
SN - 0004637X (ISSN)
LA - English
J2 - Astrophys. J.
M3 - Article
DB - Scopus
N1 - Export Date: 23 February 2023; Cited By: 58
ER -

TY - JOUR
AU - Geminale, A.
AU - Formisano, V.

AU - Giuranna, M.
TI - Methane in Martian atmosphere: Average spatial, diurnal, and seasonal behaviour
PY - 2008
T2 - Planetary and Space Science
VL - 56
IS - 9
SP - 1194
EP - 1203
DO - 10.1016/j.pss.2008.03.004
UR - https://www.scopus.com/inward/record.uri?eid=2-s2.0-45849110001&doi=10.1016%2fj.pss.2008.03.004&partnerID=40&md5=80fec1b66f01175bfba744d5a073d662
AD - I.F.S.I., INAF, 00133 Rome, Via Fosso del Cavaliere 100, Italy
AB - A large number of spectra measured by the planetary Fourier spectrometer aboard the European Mars Express mission have been studied to identify the average properties of methane in the Martian atmosphere. Using the line at 3018 cm-1, we have studied the seasonal, diurnal, and spatial variations of methane through the analysis of large averages of spectra (more than 1000 measurements). Methane mixing ratio has been obtained simultaneously with water vapour mixing ratio and water ice content, by best fitting (minimising the χ2) the computed averages with synthetic spectra. These spectra were computed for different values of the three parameters (methane and water vapour mixing ratio, and water ice optical depth). The methane mixing ratio shows a slow decrease from northern spring to southern summer with an average value of 14±5 ppbv (part per billion by volume) and it does not show a particular trend with latitude. The methane mixing ratio seems not to be uniform in longitude in the Martian atmosphere, as already reported by Formisano et al. [2004. Detection of methane in the atmosphere of Mars. Science 306, 1758-1761]. Two maxima are present at -40°E and +70°E longitude. In local time, the methane mixing ratio seems to follow the water vapour diurnal cycle. The most important point for future understanding is, however, that there are special orbits in which methane mixing ratio has a very high value. © 2008 Elsevier Ltd. All rights reserved.
KW - Martian surface analysis
KW - Microfluidics
KW - Spectrometers
KW - (PL) properties
KW - European
KW - Mars Express Mission
KW - Martian atmospheres
KW - Planetary Fourier spectrometer (PFS-MEX)
KW - SPECTRA (CO)
KW - Methane
KW - Astrobiology
KW - Atmosphere
KW - Mars
KW - Methane
SN - 00320633 (ISSN)
LA - English
J2 - Planet. Space Sci.

M3 - Article
DB - Scopus
N1 - Export Date: 23 February 2023; Cited By: 91; Correspondence Address: A. Geminale; I.F.S.I., INAF, 00133 Rome, Via Fosso del Cavaliere 100, Italy; email: anna.geminale@ifsi-roma.inaf.it; CODEN: PLSSA
ER -

TY - JOUR
AU - Lacelle, D.
AU - Fisher, D.
AU - Clark, I.D.
AU - Berinstain, A.
TI - Distinguishing between vapor- and liquid-formed ground ice in the northern martian regolith and potential for biosignatures preserved in ice bodies
PY - 2008
T2 - Icarus
VL - 197
IS - 2
SP - 458
EP - 469
DO - 10.1016/j.icarus.2008.05.017
UR - https://www.scopus.com/inward/record.uri?eid=2-s2.0-51949111070&doi=10.1016%2fj.icarus.2008.05.017&partnerID=40&md5=8c5ccf8f2b00a317d48a5164c6133637
AD - Planetary Exploration and Space Astronomy, Canadian Space Agency, Que. J3Y 8Y9, 6767 route de l'aeroport, St-Hubert, Canada
AD - Glaciology Section, Geological Survey of Canada, NRCan, Ottawa, Ont., K1A 0E8, 601 Booth Street, Canada
AD - Ottawa-Carleton Geoscience Center, Department of Earth Sciences, University of Ottawa, Ottawa, Ont. K1N 6N5, 140 Louis Pasteur, Canada
AB - In this study, various approaches that can potentially distinguish between vapor- and liquid-derived ground ice in the martian regolith (petrography, geochemistry, stable O{single bond}H isotopes, CO2{single bond}O2{single bond}N2{single bond}Ar gas composition) are examined using terrestrial ground ice examples. Although the stable O{single bond}H isotope composition ratios can distinguish between vapor- and liquid-derived terrestrial ground ice, there might be to much mixing between the various water reservoirs on Mars to effectively use it, and, like on Earth, petrographic and geochemical approaches need to be complemented with additional supporting evidences. Of the different approaches currently being employed to determine the origin of terrestrial massive ground ice and icy sediments, it is the concentration of CO2 and the O2/Ar, N2/Ar and N2/O2 ratios of air entrapped in the ice that has proven to be the less ambiguous and most discriminatory. This is because the molar ratios of atmospheric gases change during their dissolution in water due to differences in their relative solubilities, thus providing distinctive ratios for the dissolved gases. The gas composition of air entrapped in the ice not only distinguishes between vapor- and liquid-derived ground ice, but any deviation from the theoretical dissolved values can provide insights

into potential physical and biological processes operating in the subsurface, a key component for astrobiology. Crown Copyright © 2008.
KW - Geological processes
KW - Mars
KW - polar geology
SN - 10902643 (ISSN)
LA - English
J2 - Icarus
M3 - Article
DB - Scopus
N1 - Export Date: 23 February 2023; Cited By: 5; Correspondence Address: D. Lacelle; Planetary Exploration and Space Astronomy, Canadian Space Agency, Que. J3Y 8Y9, 6767 route de l'aeroport, St-Hubert, Canada; email: denis.lacelle@space.gc.ca; CODEN: ICRSA
ER -

TY - CONF
AU - Hoover, R.B.
AU - Pikuta, E.V.
AU - Townsend, A.
AU - Anthony, J.
AU - Guisler, M.
AU - McDaniel, J.
AU - Bej, A.
AU - Storrie-Lombardi, M.
TI - Microbial extremophiles from the 2008 Schirmacher Oasis Expedition: Preliminary results
PY - 2008
T2 - Proceedings of SPIE - The International Society for Optical Engineering
VL - 7097
C7 - 70970L
DO - 10.1117/12.801018
UR - https://www.scopus.com/inward/record.uri?eid=2-s2.0-55549118211&doi=10.1117%2f12.801018&partnerID=40&md5=4d493a133011f30c54b83b492ca68b8c
AD - Astrobiology Laboratory, National Space Science and Technology Center, Huntsville, AL 35805, 320 Sparkman Dr., United States
AD - Biology Dept., University of Alabama in Huntsville, Huntsville, AL 35899, United States
AD - Oakwood University, Huntsville, AL 35896, 7000 Adventist Blvd, United States
AD - Biology Dept., University of Alabama at Birmingham, Birmingham, AL 35294, 1300 University Blvd., United States
AD - Kinohi Institute, Pasadena, CA 91101, 530 S. Lake Avenue, #117, United States
AB - Among the most interesting targets for Astrobiology research are the polar ice caps and the permafrost of Mars and the ice and liquid water bodies that may lie beneath the frozen crusts of comets, the icy moons of Jupiter (Europa, Io and Ganymede) and Saturn (Titan and Enceladus). The permanently ice-covered lakes of Antarctica, such as Lake Vostok and Lake Untersee, provide some of the best terrestrial analogues for these targets. The 2008

International Tawani Schirmacher Oasis/Lake Untersee Expeditions have been organized to conduct studies of novel microbial extremophiles and investigate the biodiversity of the glaciers and ice-covered lakes of Dronning Maud Land, East Antarctica. This paper describes the preliminary analysis of the anaerobic microbial extremophiles isolated from samples collected during the 2008 International Schirmacher Oasis Antarctica Reconnaissance Expedition. These samples showed great diversity of psychrophlic and psychrotolerant bacteria. Six new anaerobic strains have been isolated in pure cultures and partially characterized. Two of them (strains ARHSd-7G and ARHSd-9G) were isolated from a small tidal pool near the colony of African Penguins Spheniscus demersus. Strain ARHSd-7G was isolated on mineral anaerobic medium with 3 % NaCl, pH 7 and D-glucose, it has motile, vibrion shape cells, and is Gram variable. Strain ARHSd-9G grew on anaerobic, alkaline medium with pH 9 and 1 % NaCl at 3°C. The substrate was D-glucose supplemented with yeast extract (0.05 %). Cells of strain ARHSd-9G had morphology of straight or slightly curved elongated rods and demonstrated unusual optical effects under dark-field visible light microscopy. The cells were spore-forming and Gram positive. From the mat sample collected near Lake Zub, the new strain LZ-3 was isolated in pure culture at 3°C. Strain LZ-3 was anaerobic and grew on 0.5 % NaCl mineral medium with Dglucose as a substrate. The gram positive cells were spore-forming. They exhibited a distinctive morphology of large rods with rounded ends and size 1x10 µm. From the sample of ice sculpted by wind and melting by solar heating, containing many entrained black rocks collected near Lake Podprudnoye the new strain ISLP-22 was isolated in pure culture. The cells of this strain had vibrion shape and were spore-forming and had "baseball bat" shapes). This culture preferred 0.1 % NaCl mineral anaerobic medium and grew rapidly at 3°C. Currently, all strains are under physiological study and phylogenetic analysis.
KW - Alkalinity
KW - Astrobiology
KW - Biodiversity
KW - Biology
KW - Cell culture
KW - Cells
KW - Cytology
KW - Glucose
KW - Heating
KW - Ice
KW - Interplanetary flight
KW - Lakes
KW - Mineralogy
KW - Minerals
KW - Optical microscopy
KW - Silica
KW - Silicate minerals
KW - Snow
KW - Sodium chloride
KW - Turbulent flow
KW - Alkali-tolerant
KW - Anaerobes

KW - Antarctica
KW - Extremophiles
KW - Psychrophilic
KW - Psychrotolerant
KW - Strain
KW - Alkali-tolerant
KW - Anaerobes
KW - Antarctica
KW - Extremophiles
KW - Psychrophilic
KW - Psychrotolerant
SN - 0277786X (ISSN); 978-081947317-2 (ISBN)
LA - English
J2 - Proc SPIE Int Soc Opt Eng
M3 - Conference paper
DB - Scopus
N1 - Export Date: 23 February 2023; Cited By: 5; CODEN: PSISD
ER -

TY - JOUR
AU - Jones, B.W.
TI - Mars before the Space Age
PY - 2008
T2 - International Journal of Astrobiology
VL - 7
IS - 2
SP - 143
EP - 155
DO - 10.1017/S1473550408004138
UR - https://www.scopus.com/inward/record.uri?eid=2-s2.0-53149151889&doi=10.1017%2fS1473550408004138&partnerID=40&md5=0c680c7bf0e27f10079f805befaa9cc0
AD - Astronomy Group, The Open University, Milton Keynes MK7 6AA, United Kingdom
AB - Mars has surely been scrutinised since the dawn of humankind. Its appearance every couple of years like a drop of blood in the sky led to warlike attributes in the ancient world. In the 16th century Tycho Brahe made accurate observations of the position of Mars that enabled Johannes Kepler to obtain his first two laws of planetary motion. These in turn were explained by Newton's laws of motion and gravity. In the 17th century the first telescope observations were made, but Mars is small and very little surface detail could be discerned. Throughout the 18th and 19th centuries telescopes improved, revealing many dark areas on the red tinted surface. During the close opposition of 1877 sufficient detail could be seen that enabled Giovanni Schiaparelli to announce that he could see about 40 canali on Mars. This led to the saga of the canals of Mars, finally laid to rest in 1971 when Mariner 9 made observations from Martian orbit showing that the canali/canals do not exist. Belief that there was life on Mars was widespread in the 19th century. However, the majority of astronomers never believed in Martian intelligence. Least controversial was the view that the dark areas were some form of plant life. This view persisted until Mariner 4 flew past Mars in 1965 and discovered a far thinner atmosphere than

previously thought. This was a low point, with impact craters dominating the images. It was Mariner 9 that revealed much more promising landscapes, including volcanic features, and others indicating that water had flowed across the surface, particularly when Mars was young. Thus, the contemporary era of Mars exploration began. Our picture of Mars today is not only much more complete than that before Mariner 4, in several ways it is quite different. The belief, however, that there might be life on Mars persists - subsurface life cannot be ruled out and, failing that, there might be ancient fossils on Mars. © 2008 Cambridge University Press.
KW - astronomy
KW - exobiology
KW - extraterrestrial matter
KW - Mars
KW - Astrobiology
KW - Extraterrestrial life
KW - History of astronomy
KW - Mars
SN - 14753006 (ISSN)
LA - English
J2 - Int. J. Astrobiology
M3 - Article
DB - Scopus
N1 - Export Date: 23 February 2023; Cited By: 5; Correspondence Address: B.W. Jones; Astronomy Group, The Open University, Milton Keynes MK7 6AA, United Kingdom; email: b.w.jones@open.ac.uk
ER -

TY - JOUR
AU - Fernández-Remolar, D.C.
AU - Knoll, A.H.
TI - Fossilization potential of iron-bearing minerals in acidic environments of Rio Tinto, Spain: Implications for Mars exploration
PY - 2008
T2 - Icarus
VL - 194
IS - 1
SP - 72
EP - 85
DO - 10.1016/j.icarus.2007.10.009
UR - https://www.scopus.com/inward/record.uri?eid=2-s2.0-38949207900&doi=10.1016%2fj.icarus.2007.10.009&partnerID=40&md5=f4428b96141ce974f4a390b91f0e7c8d
AD - Centro de Astrobiología, INTA, 28850 Torrejón de Ardoz, Ctra Ajalvir Km. 4, Spain
AD - Department of Organismic and Evolutionary Biology, Harvard University, Cambridge, MA 02138, 26 Oxford Street, United States
AB - Acidic waters of the Rio Tinto, southwestern Spain, evaporate seasonally, precipitating a variety of iron sulfide and oxide minerals. Schwertmannite and nanophase goethite form thin laminae on biological and detrital grain surfaces, replicating, among other things, the morphologies of insect cuticle, plant tissues, fungi, algae, and bacteria. Intergrain cements also incorporate bacterial cells and filaments. Other sulfate minerals precipitated in Rio

Tinto environments are transient and contribute little to short-term preservation. Because the Rio Tinto has been cutting its current valley for several million years, terrace deposits provide a window on longer term fossil preservation. Early and later diagenesis are recorded in terrace deposits formed about one thousand and two million years ago, respectively. The sedimentary structures and mineralogies of these deposits suggest that they formed under physical and chemical conditions comparable to those of modern Rio Tinto sediments. The terrace deposits show quantitative loss of sulfate minerals, increasing crystallinity of goethite and, in the older terrace, replacement of goethite by hematite. Fossils formed originally by schwertmannite and nanophase goethite replication persist through diagenesis, preserving a long term record of local biological diversity. Fossil preservation by iron oxides in the acidic environment of Rio Tinto suggests that if life was present when sedimentary rocks formed at Meridiani Planum, Mars, precipitated minerals could record their presence.
KW - Astrobiology
KW - Mars
SN - 10902643 (ISSN)
LA - English
J2 - Icarus
M3 - Article
DB - Scopus
N1 - Export Date: 23 February 2023; Cited By: 55; Correspondence Address: D.C. Fernández-Remolar; Centro de Astrobiología, INTA, 28850 Torrejón de Ardoz, Ctra Ajalvir Km. 4, Spain; email: fernandezrd@inta.es; CODEN: ICRSA
ER -

TY - CONF
AU - Hipkin, V.J.
AU - Rummel, J.D.
TI - A key role for analogue missions in the search for life
PY - 2008
T2 - International Astronautical Federation - 59th International Astronautical Congress 2008, IAC 2008
VL - 1
SP - 371
EP - 379
UR - https://www.scopus.com/inward/record.uri?eid=2-s2.0-77950475950&partnerID=40&md5=e8a1e0d3ece092fda6006b0b0a544fdc
AD - Canadian Space Agency, Montreal, Canada
AD - East Carolina University, Greenville, NC, United States
AB - The search for life on Mars and on the moons of the outer planets of our solar system is a remarkable challenge. Bathed as we are in Earth life, we face formidable difficulties in developing definitive signatures for extraterrestrial life (biomarkers) that can be detected despite the inevitable contamination of the tools used to select and deliver material to the instruments that will measure those signatures (sample chain contamination), while using only a limited suite of planetary instrumentation due to limitations in landed mass and cost (real life). And all of that without Earth life getting in the way. In order to understand and overcome the

difficulties in detecting extraterrestrial life, practice may make perfect—or at least good—enough. The study of Earth life in the extreme environments of Earth offers an important means of preparing for space missions that can undertake the challenges directly. Analogue missions are in one sense simulations that target terrestrial sites that are scientifically relevant to planetary environments, while to be useful they must have a focus on conducting real science. A well-chosen analogue site can provide a suitable development and testing ground for the sampling and analysis methodologies, but if science is not a key driver in those missions then the test is a charade, and development of improved techniques will suffer accordingly. That being said, scientific relevance is critical to the value of analogue activities. No terrestrial environment can fully represent Mars, Titan, or Europa, but targeting an environment that is relevant to the planetary body and the signature being sought is an essential part of mission preparation. In particular, the confluence of physical circumstances at a site (e.g., pH, temperature, geochemistry, volcanism, ice/water) will drive the biological and organic chemistry studies that may be accomplished, and that may reveal necessary improvements for later "real" missions.
KW - Analytical geochemistry
KW - Interplanetary flight
KW - Planets
KW - Pollution detection
KW - Extraterrestrial life
KW - Extreme environment
KW - Organic Chemistry
KW - Outer planets
KW - Planetary bodies
KW - Planetary environments
KW - Planetary instrumentation
KW - Search for life
KW - Space missions
KW - Terrestrial environments
KW - Testing grounds
KW - Earth (planet)
SN - 978-161567160-1 (ISBN)
LA - English
J2 - Int. Astronaut. Fed. - Int. Astronaut. Congr., IAC
M3 - Conference paper
DB - Scopus
N1 - Export Date: 23 February 2023; Cited By: 0
ER -

TY - CONF
AU - Holden, T.
AU - Marchese, P.
AU - Tremberger. Jr., G.
AU - Cheung, E.
AU - Subramaniam, R.
AU - Sullivan, R.
AU - Schneider, P.
AU - Flamholz, A.

AU - Huerta, M.
AU - Lieberman, D.
AU - Cheung, T.
TI - Phaeodactylum tricornutum photosynthesis and Thalassiosira pseudonana bio-silica formation genes nucleotide fluctuations
PY - 2008
T2 - Proceedings of SPIE - The International Society for Optical Engineering
VL - 7097
C7 - 70970R
DO - 10.1117/12.794414
UR - https://www.scopus.com/inward/record.uri?eid=2-s2.0-55549112551&doi=10.1117%2f12.794414&partnerID=40&md5=5e31b4099c1fde428510b7fb0737e506
AD - CUNY Queensborough Community College, Bayside, NY 11364, 222-05 56th Ave., United States
AB - Diatom bioactivity has been reported to be responsible for about 20% of carbon fixation globally and together with other photosynthetic organisms, the bioactivity can be monitored via satellite ocean imaging. The bioinformatics embedded in the nucleotide fluctuations of photosynthesis and bio-silicate genes in diatoms were studied. The recently reported phosphoenolpyruvate carboxylase PEPC1 and PEPC2 C4-like photosynthesis genes in Phaeodactylum tricornutum were found to have similar fractal dimensions of about 2.01. In comparison, the green alga Chlamydomonas reinhardtii PEPC1 and PEPC2 genes have fractal dimensions of about 2.05. The PEPC CpG dinucleotide content is 8% in P. tricornutum and 10% in C. reinhardtii. Further comparison of the cell wall protein gene showed that the VSP1 gene sequence in C. reinhardtii has a fractal dimension of 2.03 and the bio-silica formation silaffin gene in Thalassiosira pseudonana has a fractal dimension of 2.01. The phosphoenolpyruvate carboxylase PPC1 and PPC2 in T. pseudonana were found to have fractal dimensions and CpG dinucleotide content similar to that of P. tricornutum. The fractal dimension of the dnaB replication helicase gene is about 1.98 for both diatoms as well as for the alga Heterosigma akashiwo. In comparison, the E. coli dnaB gene has a fractal dimension of about 2.03. Given that high fractal dimension and CpG dinucleotide content sequences have been associated with the presence of selective pressures, the relatively low fractal dimension gene sequences of the two unique properties of Earth-bound diatoms (photosynthesis and bio-silica cell wall) suggests the potential for the development of high fractal dimension sequences for adaptation in harsh environments.
KW - Algae
KW - Astrobiology
KW - Atomic physics
KW - Atoms
KW - Bioinformatics
KW - Escherichia coli
KW - Fractals
KW - Gallium alloys
KW - Genes
KW - Initiators (chemical)

KW - Nucleic acids
KW - Nucleotides
KW - Oxides
KW - Partial discharges
KW - Photosynthesis
KW - Phytoplankton
KW - Silica
KW - Silicates
KW - Water pollution
KW - Bio-silicate genes
KW - Carbon fixations
KW - Cell wall proteins
KW - Chlamydomonas reinhardtii
KW - Diatom
KW - Dinucleotide
KW - E. coli
KW - Gene sequences
KW - Green alga
KW - Harsh environments
KW - Helicase genes
KW - Phaeodactylum tricornutum
KW - Phosphoenolpyruvate carboxylases
KW - Photosynthesis genes
KW - Photosynthetic organisms
KW - Selective pressures
KW - Silica cells
KW - Silica formations
KW - Thalassiosira pseudonana
KW - Fractal dimension
KW - Bio-silicate genes
KW - Diatom
KW - Fractal
KW - Photosynthesis genes
SN - 0277786X (ISSN); 978-081947317-2 (ISBN)
LA - English
J2 - Proc SPIE Int Soc Opt Eng
M3 - Conference paper
DB - Scopus
N1 - Export Date: 23 February 2023; Cited By: 0; CODEN: PSISD
ER -

TY - JOUR
AU - Tajika, E.
TI - Snowball planets as a possible type of water-rich terrestrial planet in extrasolar planetary systems
PY - 2008
T2 - Astrophysical Journal
VL - 680
IS - 1 PART 2
SP - L53
EP - L56
DO - 10.1086/589831
UR - https://www.scopus.com/inward/record.uri?eid=2-s2.0-67649619556&doi=10.1086%2f589831&partnerID=40&md5=e01b4afc7c1ae

d6f1c6089053e7ff81d
AD - Department of Earth and Planetary Science, University of Tokyo, Tokyo 113-0033, Japan
AB - Terrestrial planets with abundant water have multiple climate modes, including an ice-free, a partially ice-covered, and a globally ice-covered state. Recent geological studies have revealed that the Earth experienced global glaciations in its history ("snowball Earth" hypothesis). In the snowball glaciations, liquid water is thought to have existed under the ice shell because of geothermal heat flow from the Earth's interior. Here, by analogy with the snowball glaciations, I discuss the conditions for an extrasolar terrestrial planet which is covered with ice but has an internal ocean for the timescale of planetary evolution owing to geothermal heat flow from the planetary interior. I show that liquid water can exist if the planetary mass and the water abundance are comparable to the Earth, although a planet with a mass <0.4 M⊕ (M⊕ is the Earth's mass) would not be able to maintain the internal ocean. Liquid water would be absolutely stable for a planet with a mass ≥4 M⊕ (i.e., super-Earth) either on its surface or beneath the ice, irrespective of planetary orbit and luminosity of the central star. Searches for terrestrial planets in extrasolar planetary systems should consider such a "snowball planet," which is a possible type of water-rich terrestrial planet other than an Earth-like "ocean planet." © 2008, The American Astronomical Society, All rights reserved.
KW - Astrobiology
KW - Planetary systems
KW - Planets and satellites: General
PB - Institute of Physics Publishing
SN - 0004637X (ISSN)
LA - English
J2 - Astrophys. J.
M3 - Article
DB - Scopus
N1 - Export Date: 23 February 2023; Cited By: 20; Correspondence Address: E. Tajika; Department of Earth and Planetary Science, University of Tokyo, Tokyo 113-0033, Japan; email: tajika@eps.s.u-tokyo.ac.jp
ER -

TY - JOUR
AU - Cottin, H.
AU - Coll, P.
AU - Coscia, D.
AU - Fray, N.
AU - Guan, Y.Y.
AU - Macari, F.
AU - Raulin, F.
AU - Rivron, C.
AU - Stalport, F.
AU - Szopa, C.
AU - Chaput, D.
AU - Viso, M.
AU - Bertrand, M.

AU - Chabin, A.
AU - Thirkell, L.
AU - Westall, F.
AU - Brack, A.
TI - Heterogeneous solid/gas chemistry of organic compounds related to comets, meteorites, Titan, and Mars: Laboratory and in lower Earth orbit experiments
PY - 2008
T2 - Advances in Space Research
VL - 42
IS - 12
SP - 2019
EP - 2035
DO - 10.1016/j.asr.2007.09.017
UR - https://www.scopus.com/inward/record.uri?eid=2-s2.0-55949107965&doi=10.1016%2fj.asr.2007.09.017&partnerID=40&md5=ff65f8403956cba7b23d2a43718baf41
AD - LISA, Université Paris 12 et Paris 7, CNRS, 94010 Créteil, 61 Av. du General de Gaulle, France
AD - Service d'aéronomie, UMR 7620 CNRS, 91371 Verrieres le Buisson Cedex, Reduit Verrieres - BP 3, Rte. des Gatines, France
AD - CNES, Centre spatial de Toulouse, 31401 Toulouse Cedex 9, 18 Avenue Edouard Belin, France
AD - CNES, 75039 Paris Cedex 01, 2 Place Maurice Quentin, France
AD - CBM, CNRS, 45071 Orleans Cedex 2, Rue Charles-Sadron, France
AD - LPCE, CNRS, 45071 Orleans Cedex 2, Avenue de la Recherche Scientifique, France
AB - To understand the evolution of organic molecules involved in extraterrestrial environments and with exobiological implications, many experimental programs in the laboratory are devoted to photochemical studies in the gaseous phase as well as in the solid state. The validity of such studies and their applications to extraterrestrial environments can be questioned as long as experiments conducted in space conditions, with the full solar spectrum, especially in the short wavelength domain, have not been implemented. The experiments that are described here will be carried out on a FOTON capsule, using the BIOPAN facility, and on the International Space Station, using the EXPOSE facility. Vented and sealed exposition cells will be used, which will allow us to study the chemical evolution in the gaseous phase as well as heterogeneous processes, such as the degradation of solid compounds and the release of gaseous fragments. Four kinds of experiments will be carried out. The first deal with comets and are related to the Rosetta mission, the second with Titan and are related to the Cassini-Huygens mission, the third with the search for life-related organic compounds on Mars and, finally, the fourth are a continuation of previous studies concerning the behavior of amino acids in space. © 2007 COSPAR.
KW - Meteorites
KW - Organic compounds
KW - Photolysis
KW - Space stations
KW - Astrochemistry
KW - BIOPAN

KW - Comet
KW - EXPOSE
KW - FOTON
KW - International Space stations
KW - Mars
KW - Titan
KW - Orbits
KW - Astrochemistry
KW - BIOPAN
KW - Comet
KW - Exo-astrobiology
KW - EXPOSE
KW - FOTON
KW - International Space Station
KW - Mars
KW - Meteorite
KW - Photolysis
KW - Titan
PB - Elsevier Ltd
SN - 02731177 (ISSN)
LA - English
J2 - Adv. Space Res.
M3 - Article
DB - Scopus
N1 - Export Date: 23 February 2023; Cited By: 36; Correspondence Address: H. Cottin; LISA, Université Paris 12 et Paris 7, CNRS, 94010 Créteil, 61 Av. du General de Gaulle, France; email: cottin@lisa.univ-paris12.fr; CODEN: ASRSD
ER -

TY - CONF
AU - Gallardo, V.A.
AU - Espinoza, C.
TI - The evolution of ocean color
PY - 2008
T2 - Proceedings of SPIE - The International Society for Optical Engineering
VL - 7097
C7 - 70970G
DO - 10.1117/12.794742
UR - https://www.scopus.com/inward/record.uri?eid=2-s2.0-55549111861&doi=10.1117%2f12.794742&partnerID=40&md5=25fb5933f6b6cb6221e93ff84587f28e
AD - Laboratorio de Bentos, Departamento de Oceanografía, Center for Biotechnology, Concepción, Chile
AD - Universidad de Concepción, Concepción, Chile
AB - Analog examples of what primeval oceans might have looked in the Precambrian are probably extant in various regions and at various size scales in present day oceans albeit they have not been sufficiently recognized and/or studied. The Eastern Boundary Current Ecosystems (EBCEs), with their characteristic high productivity-inducing coastal upwelling events, their extensive and intensive anoxic/hypoxic water column and methane and sulfide-rich benthic environment, appear to represent such analogs. Moreover, recent

studies have shown that they possess diverse anaerobic prokaryotic communities of mat-forming large multi-cellular filamentous bacteria similar to fossils found in Archean and Proterozoic rocks. Observations in the Bay of Concepcion, central Chile (~36°S), inserted in the second most productive EBCE of the world, suggests that given similar oceanographic dynamics, past oceans may have presented different predominant colorations after the first probable "red" color of the reduced iron-rich Archean ocean and prior to the present day "blue" color. In this coastal ecosystem a "black" coloration has been observed to form as the result of the floating to the surface layer of sulfide-blackened benthic detritus together with chunks of microbial mats, and a "milky to turquoise" coloration resulting from different concentrations of colloidal, nano-sized particles which may include elemental sulfur and/or microorganisms. If the present is the key to the past we posit that "black" color oceans could have existed during the Proterozoic "Canfield sulfidic ocean" followed by "milky to turquoise" colored oceans during later stages of the Proterozoic. Meso-scale examples of "milky" and "turquoise" portions of oceans, caused by elemental sulfur from oxidized hydrogen sulfide eruptions, have been described from off Namibia and there appear to also exist elsewhere. Examples of "black" oceans have apparently not been reported but the name of the Black Sea, the largest permanent anoxic basin on Earth, suggests that at some point in time it may have been black, at least locally and/or for short periods, prompting the name. We conclude suggesting that analogous to the present "Blue Planet" denomination, in the past our Earth could possibly have deserved the successive names of "Red", "Black" and "Milky-Turquoise" Planet.
KW - Astrobiology
KW - Carbonate minerals
KW - Color
KW - Earth (planet)
KW - Ecology
KW - Ecosystems
KW - Hydrogen
KW - Hydrogen sulfide
KW - Methane
KW - Optical properties
KW - Sulfur
KW - Eastern Boundary Current Ecosystems
KW - Namibia
KW - Ocean color
KW - Proterozoic
KW - Sea discoloration
KW - Ocean currents
KW - Eastern Boundary Current Ecosystems
KW - Namibia
KW - Ocean color
KW - Proterozoic
KW - Sea discoloration
SN - 0277786X (ISSN); 978-081947317-2 (ISBN)
LA - English
J2 - Proc SPIE Int Soc Opt Eng
M3 - Conference paper

DB - Scopus
N1 - Export Date: 23 February 2023; Cited By: 2; CODEN: PSISD
ER -

TY - CONF
AU - Cash, W.
AU - Oakley, P.
AU - Turnbull, M.
AU - Glassman, T.
AU - Lo, A.
AU - Polidan, R.
AU - Kilston, S.
AU - Noecker, C.
TI - The new worlds observer: Scientific and technical advantages of external occulters
PY - 2008
T2 - Proceedings of SPIE - The International Society for Optical Engineering
VL - 7010
C7 - 70101Q
DO - 10.1117/12.789717
UR - https://www.scopus.com/inward/record.uri?eid=2-s2.0-70349496583&doi=10.1117%2f12.789717&partnerID=40&md5=f8e0368537bfe676bbede7a66a6f7b66
AD - University of Colorado, United States
AD - Northrop-Grumman Space Technologies, United States
AD - Ball Aerospace, United States
AB - Perhaps the most compelling piece of science and exploration now under discussion for future space missions is the direct study of planets circling other stars. Indirect means have established planets as common in the universe but have given us a limited view of their actual characteristics. Direct observation holds the potential to map entire planetary systems, view newly forming planets, find Earth-like planets and perform photometry to search for major surface features. Direct observations will also enable spectroscopy of exoplanets and the search for evidence of simple life in the universe. Recent advances in the design of external occulters - starshades that block the light from the star while passing exoplanet light - have lowered their cost and improved their performance to the point where we can now envision a New Worlds Observer that is both buildable and affordable with today's technology. In this paper we explore the comparison of scientific capability of external occulters relative to indirect means and to internal coronagraph missions. We conclude that external occulters logically provide the architecture for the next space mission for exoplanet studies.
KW - Astrobiology
KW - Optical instruments
KW - Optical telescopes
KW - Planets
KW - Space telescopes
KW - Coronography
KW - Direct observation
KW - Earth-like planets

KW - Exoplanet
KW - Exoplanets
KW - Occulters
KW - Planetary system
KW - Space missions
KW - Starshades
KW - Surface feature
KW - Spacecraft observatories
KW - Astrobiology
KW - Coronography
KW - Exoplanets
SN - 0277786X (ISSN); 978-081947220-5 (ISBN)
LA - English
J2 - Proc SPIE Int Soc Opt Eng
M3 - Conference paper
DB - Scopus
N1 - Export Date: 23 February 2023; Cited By: 10; CODEN: PSISD
ER -

TY - JOUR
AU - Holmes, D.S.
TI - Review of International Biohydrometallurgy Symposium, Frankfurt, 2007
PY - 2008
T2 - Hydrometallurgy
VL - 92
IS - 1-2
SP - 69
EP - 72
DO - 10.1016/j.hydromet.2008.01.003
UR - https://www.scopus.com/inward/record.uri?eid=2-s2.0-41749097223&doi=10.1016%2fj.hydromet.2008.01.003&partnerID=40&md5=5345606d1c4ccd10c00f6cd63249fb57
AD - Center for Bioinformatics and Genome Biology, Fundación Ciencia para la Vida, MIFAB, and Andrés Bello University, Santiago, Chile
AB - This article reviews the proceedings of the 17th International Biohydrometallurgy Symposium held in Frankfurt, Germany from September 2-6, 2007. Topics included, but were not limited to: microbiology and engineering of bioleaching, bioremediation, microbe-mineral interfacial processes, biosorption and nano-biotechnology. This review highlights aspects of biotechnological relevance. The various papers amply demonstrate that an ancient biotechnology has come of age with the commissioning of the world's largest microbial bioreactor at Escondida in Chile sending a strong message that bioleaching is a practical technology. A Special Edition of Hydrometallurgy later this year will feature selected papers from this Symposium that have been peer reviewed. © 2008 Elsevier B.V. All rights reserved.
KW - Biofilms
KW - Biomarkers
KW - Bioremediation
KW - Biohydromettalurgy
KW - Microbial diversity

KW - Biodiversity
KW - Biofilms
KW - Biohydromettalurgy
KW - Biomarkers for extraterrestrial life
KW - Biomine Wiki
KW - Bioremediation
KW - European initiatives
KW - Genomics
KW - Microbial diversity
KW - Proteomics
SN - 0304386X (ISSN)
LA - English
J2 - Hydrometallurgy
M3 - Article
DB - Scopus
N1 - Export Date: 23 February 2023; Cited By: 12; Correspondence Address: D.S. Holmes; Center for Bioinformatics and Genome Biology, Fundación Ciencia para la Vida, MIFAB, and Andrés Bello University, Santiago, Chile; email: dsholmes2000@yahoo.com; CODEN: HYDRD
ER -

TY - CHAP
AU - McKay, C.P.
TI - Astrobiology
PY - 2008
T2 - Encyclopedia of Ecology, Five-Volume Set
SP - 272
EP - 275
DO - 10.1016/B978-008045405-4.00602-9
UR - https://www.scopus.com/inward/record.uri?eid=2-s2.0-85069591965&doi=10.1016%2fB978-008045405-4.00602-9&partnerID=40&md5=484645ac1eff5751f955f507baf0f269
AD - NASA Ames Research Center, Moffett Field, CA, United States
AB - Earth is alone in the solar system in having a global ecology. Mars may have had a global ecology in the past and remnants of this life may be found there. Subsurface ecosystems may exist on Europa and Enceladus, two moons of the outer solar system. Restoration of a global ecosystem on Mars may be possible allowing indigenous life there to flourish or allowing for life transplanted from Earth. © 2008 Elsevier B.V. All rights reserved.
KW - Astrobiology
KW - Enceladus
KW - Europa
KW - Life
KW - Mars
KW - Terraforming
PB - Elsevier Inc.
SN - 978-008091456-5 (ISBN); 978-008045405-4 (ISBN)
LA - English
J2 - Encycl. of Ecol., Five-Vol. Set
M3 - Book chapter
DB - Scopus
N1 - Export Date: 23 February 2023; Cited By: 0
ER -

TY - CONF
AU - Edwards, H.G.M.
AU - Hargreaves, M.D.
TI - Raman spectroscopy : the biomolecular detection of life in extreme environments
PY - 2008
T2 - Models, Mysteries and Magic of Molecules
SP - 1
EP - 28
UR - https://www.scopus.com/inward/record.uri?eid=2-s2.0-84900244962&partnerID=40&md5=db810eba38ac31189774a4d0f3227507
AB - The strategic adaptation of extremophiles (organisms which can survive where humans cannot) to survival in hostile terrestrial environments depends critically upon their synthesis of protectant biomolecules in geological niches to combat low wavelength radiation insolation, desiccation, and extremes of temperature and pH. Each year sees the discovery of novel geological scenarios in which organisms have successfully created a tenacious colonisation in "limits of life" habitats. Terrestrial analogues such as hot and cold deserts, volcanoes and geothermal springs provide models for the extraterrestrial study of the evolution of life in our Solar System - astrobiology. In particular, the current robotic exploration of the surface and subsurface of Mars, our neighbouring planet which has held ancient magical significance for our ancestors and is still shrouded in mysteries, is now indicative of the importance of a range of terrestrial scenarios which can be considered as Mars analogues. The next phase of Martian exploration must address the robotic search for extinct or extant life in the geological record, which is essential for the proposed human missions in the next two decades. A key factor in the armoury of remote analytical instrumentation that is now envisaged for inclusion on extended-range Mars landers and rovers is the identification of the chemical and biochemical protectants that might have been produced by extremophiles for survival in the Martian deserts. The molecular signatures evidenced from the Raman spectra of these key protectants will be fundamental for the detection of biological activity on Mars and the adoption of miniaturised Raman spectrometers for Martian exploration is now being seriously considered by NASA and ESA. In this context, the evaluation of prototype Raman spectroscopic instrumentation for the detection of molecules of relevance to a wide range of terrestrial extremophilic activity is now being addressed and forms the subject of this article. Exemplars from various geological scenarios in the Arctic and Antarctic cold deserts and from relevant hot desert habitats, such as volcanic geothermal springs and salt pan evaporites, will reinforce the tenet of this book - that the molecular studies of extremophilic models will be pivotal in the understanding of the magic and mysteries of evolution of life on Earth and the search for life on Mars. © 2008 Springer.
KW - Arid regions
KW - Bioactivity
KW - Chemical analysis
KW - Ecosystems

KW - Molecules
KW - NASA
KW - Raman spectroscopy
KW - Robotics
KW - Skin
KW - Volcanoes
KW - Wavemeters
KW - Analytical Instrumentation
KW - Biomolecular detections
KW - Extreme environment
KW - Molecular signatures
KW - Robotic explorations
KW - Strategic adaptations
KW - Terrestrial environments
KW - Wavelength radiation
KW - Biology
SN - 978-140205940-7 (ISBN)
LA - English
J2 - Model., Mysteries Magic Mol.
M3 - Conference paper
DB - Scopus
N1 - Export Date: 23 February 2023; Cited By: 3
ER -

TY - CONF
AU - Storrie-Lombardi, M.C.
AU - Muller, J.-P.
AU - Fisk, M.R.
AU - Griffiths, A.D.
AU - Coates, A.J.
AU - Hoover, R.B.
TI - Epifluorescence surveys of extreme environments using PanCam imaging systems: Antarctica and the Mars regolith
PY - 2008
T2 - Proceedings of SPIE - The International Society for Optical Engineering
VL - 7097
C7 - 70970P
DO - 10.1117/12.800924
UR - https://www.scopus.com/inward/record.uri?eid=2-s2.0-55549098891&doi=10.1117%2f12.800924&partnerID=40&md5=87b8bbab4b8c69d6a5fff1c06aabd3d6
AD - Kinohi Institute, Pasadena, CA 91101, 530 S. Lake Avenue, #117, United States
AD - Mullard Space Sciences Laboratory, Department of Space and Climate Physics, University College London, Holmbury St. Mary, Surrey, RH5 6NT, United Kingdom
AD - Oregon State University, College of Ocean and Atmospheric Sciences, Corvallis, OR 97331, United States
AD - NASA National Space Science and Technology Center, Huntsville, AL 35805, 320 Sparkman Drive, United States
AB - Rapid discovery of the distribution and relative abundance of organic material without sample destruction or the expenditure of irreplaceable resources is one of the primary requirements for

exploration of novel extreme environments both in remote locations on Earth and on the Mars regolith. A wide variety of organic and biogenic molecular targets including polycyclic aromatic hydrocarbons, aromatic amino acids, nucleic acids, photosynthetic pigments, and critical metabolic components such as flavin adenine dinucleotide and nicotinamide adenine dinucleotide exhibit strong, distinctive fluorescent signatures following excitation by ultraviolet (UV) light sources. These fluorescence signatures are easily imaged with camera systems currently employed on Mars rovers and imaging equipment available during human or robotic exploration of remote extreme environments on Earth. In this paper we discuss recent results with epifluorescent imaging of organic and biological targets using filter bands comparable to those available to ExoMars and review plans for epifluorescence surveys of the Dry Valleys of Eastern Antarctica including the Schirmacher Oasis and the perennially ice-covered, merimictic, oligotrophic Lake Untersee.
KW - Amides
KW - Amines
KW - Amino acids
KW - Aromatic compounds
KW - Astrobiology
KW - Biofiltration
KW - Ecology
KW - Fluorescence
KW - Hydrocarbons
KW - Light
KW - Light emission
KW - Light sources
KW - Lighting
KW - Luminescence
KW - Nucleic acids
KW - Optoelectronic devices
KW - Organic acids
KW - Organic compounds
KW - Polycyclic aromatic hydrocarbons
KW - Surveys
KW - Antarctica
KW - Epifluorescence
KW - Mars
KW - PanCam
KW - Schirmacher oasis
KW - Aromatic hydrocarbons
KW - Antarctica
KW - Epifluorescence
KW - Lake untersee
KW - Mars
KW - PanCam
KW - Schirmacher oasis
SN - 0277786X (ISSN); 978-081947317-2 (ISBN)
LA - English
J2 - Proc SPIE Int Soc Opt Eng
M3 - Conference paper
DB - Scopus
N1 - Export Date: 23 February 2023; Cited By: 5; Correspondence

Address: M. C. Storrie-Lombardi; Kinohi Institute, Pasadena, CA 91101, 530 S. Lake Avenue, #117, United States; email: mike@kinohi.org; CODEN: PSISD
ER -

TY - CONF
AU - Tiffany, M.A.
AU - Wolny, J.
AU - Garrett, M.
AU - Steidinger, K.
AU - Hurlbert, S.H.
TI - Dramatic blooms of Prymnesium sp. and Alexandrium margalefii in the Salton Sea, California
PY - 2008
T2 - Proceedings of SPIE - The International Society for Optical Engineering
VL - 7097
C7 - 70970T
DO - 10.1117/12.800919
UR - https://www.scopus.com/inward/record.uri?eid=2-s2.0-55549098890&doi=10.1117%2f12.800919&partnerID=40&md5=9c8ecd4e1bfcc844c1cb0cdfc3a53700
AD - Department of Biology, Center for Inland Waters, San Diego State University, San Diego, CA 92182, United States
AD - Florida Fish and Wildlife Conservation Commission, Fish and Wildlife Research Institute, St. Petersburg, FL 33701, 100 8th Ave SE, United States
AD - Florida Institute of Oceanography, St. Petersburg, FL 33701, 830 1st St S, United States
AB - In early 2006, unusual algal blooms of two species occurred in the Salton Sea, a large salt lake in southern California. In mid-January local residents reported bioluminescence in the Sea. Starting in February, large rafts of long-lasting foam, also bioluminescent, were observed as well. Microscopy investigations on water and sediment samples collected in March showed the marine dinoflagellate, Alexandrium margalefii, and the prymnesiophyte, Prymnesium sp., both previously unreported in the Salton Sea, to be abundant. Bioluminescence and foam production continued through March. Other dinoflagellate species, recorded during earlier studies, were rare or not detected during these blooms. Despite the fact that many Alexandrium species are known paralytic shellfish poison (PSP) producers, preliminary saxitoxin tests on this population of A. margalefii were negative. Previous reports on A. margalefii do not mention bioluminescence. It appears that the foam was caused by the Prymnesium sp. bloom, probably via protein-rich exudates and lysis of other algal cells, and its glow was due to entrained A. margalefii. This is the first report of A. margalefii in U.S. waters and the first report of it in a lake.
KW - Astrobiology
KW - Blooms (metal)
KW - Lakes
KW - Light emission
KW - Luminescence
KW - Phosphorescence

KW - Sedimentation
KW - Alexandrium
KW - Algal blooms
KW - Algal cells
KW - Allelopathy
KW - California
KW - Cysts
KW - Dinoflagellates
KW - Foam productions
KW - Local residents
KW - Paralytic shellfish poisons
KW - Salt lake
KW - Salt lakes
KW - Salton seas
KW - Sediment samples
KW - Southern california
KW - Bioluminescence
KW - Allelopathy
KW - Bioluminescence
KW - Cysts
KW - Dinoflagellates
KW - Foam
KW - Phytoplankton
KW - Salt lake
SN - 0277786X (ISSN); 978-081947317-2 (ISBN)
LA - English
J2 - Proc SPIE Int Soc Opt Eng
M3 - Conference paper
DB - Scopus
N1 - Export Date: 23 February 2023; Cited By: 0; CODEN: PSISD
ER -

TY - CHAP
AU - Raulin, F.
TI - Astrobiology of Saturn's Moon Titan
PY - 2008
T2 - Complete Course in Astrobiology
SP - 223
EP - 252
DO - 10.1002/9783527618996.ch9
UR - https://www.scopus.com/inward/record.uri?eid=2-s2.0-77949774817&doi=10.1002%2f9783527618996.ch9&partnerID=40&md5=f1c69528ed926fccc3370239934b7f50
AB - The chapter presents the astrobiological case for Titan, the largest satellite of Saturn. Besides Jupiter's moon Europa, Titan appears to be the most interesting body for astrobiology in the outer Solar System because it has an active prebiotic-like chemistry, but liquid water is absent on its surface. This chapter starts with a description of the general properties of Titan and models of its formation and internal structure. Emphasis is placed on the astrobiological aspects, which are based on spacecraft observations, above all the recent Cassini-Huygens mission, on laboratory experiments simulating the atmospheric conditions of Titan, and on photochemical modeling. On the basis of those data,

current astrobiological aspects of Titan are considered with regard to analogies with the early Earth, extraterrestrial organic (even prebiotic-like) chemistry, and possibilities for life. © 2007 WILEY-VCH Verlag GmbH & Co. KGaA. All rights reserved.
KW - Moon
KW - Planets
KW - Satellites
KW - Atmospheric conditions
KW - Cassini-Huygens mission
KW - Internal structure
KW - Laboratory experiments
KW - Outer solar system
KW - Photochemical modeling
KW - Saturn's moons
KW - Spacecraft observations
KW - Interplanetary flight
PB - wiley
SN - 978-352761899-6 (ISBN); 978-352740660-9 (ISBN)
LA - English
J2 - Complete Course in Astrobiol.
M3 - Book chapter
DB - Scopus
N1 - Export Date: 23 February 2023; Cited By: 1
ER -

TY - CONF
AU - Martin, J.P.
AU - Marshall, J.R.
AU - Mason, L.W.
AU - Scheld, D.
TI - In-situ geologic analyzer for Lunar and Martian surfaces
PY - 2008
T2 - Earth and Space Conference 2008: Proceedings of the 11th Aerospace Division International Conference on Engineering, Science, Construction, and Operations in Challenging Environments
VL - 323
DO - 10.1061/40988(323)100
UR - https://www.scopus.com/inward/record.uri?eid=2-s2.0-70349379093&doi=10.1061%2f40988%28323%29100&partnerID=40&md5=5ac8f8a60f4c635970918b3edbc26f17
AD - N-Science Corp., United States
AD - SETI Institute, United States
AD - Lockheed Martin, United States
AB - The miniature instrument with a triple measurement system, works as a robotic field geologist on remote planetary surfaces such as Mars or the Moon. This Mineral Identification and Composition Analyzer (MICA), employs XRF, XRD, and visual imaging for in-situ nondestructive determination of mineralogy and elemental composition of rock or regolith samples. Its key feature is characterizing geological materials without destroying, damaging, or removing the sample from its in-situ location. MICA addresses two key issues: 1) Mars geology/astrobiology relating to physical and chemical evidence of water and its processes and 2) lunar in-situ resource utilization (ISRU). A 1.5 kg MICA prototype was developed and built under NASA

MIDP (Mars Instrument Development Project) and demonstrated with calibration samples and natural rocks on the NASA Ames K-9 test rover robotic arm. MICA serves as a reconnaissance sensor, providing rapid, 10-30 minute analyses for making decisions about further analysis, sample collection, or sample return from a planetary surface. © 2008 ASCE.
KW - Geology
KW - Mica
KW - Moon
KW - NASA
KW - Planetary surface analysis
KW - Robotics
KW - Calibration samples
KW - Chemical evidences
KW - Elemental compositions
KW - Geological materials
KW - In-situ resource utilizations
KW - Measurement system
KW - Mineral identification
KW - Nondestructive determination
KW - Earth (planet)
PB - American Society of Civil Engineers (ASCE)
SN - 978-078440988-6 (ISBN)
LA - English
J2 - Proc. Int. Conf. Eng., Sci., Constr., Oper. Chall. Environ.
M3 - Conference paper
DB - Scopus
N1 - Export Date: 23 February 2023; Cited By: 2
ER -

TY - CONF
AU - Som, S.M.
AU - Collins, R.E.
AU - Schreiber, B.C.
AU - Montgomery, D.R.
TI - Salts on Mars: New perspectives in planetary geomorphology and astrobiological implications
PY - 2008
T2 - International Astronautical Federation - 59th International Astronautical Congress 2008, IAC 2008
VL - 1
SP - 387
EP - 410
UR - https://www.scopus.com/inward/record.uri?eid=2-s2.0-77950463860&partnerID=40&md5=b3e3e39373c2dc1b1308d397ac83193b
AD - Department of Earth and Space Sciences, University of Washington, Seattle, WA 98195, United States
AD - School of Oceanography, University of Washington, Seattle, WA 98195, United States
AB - The positive detection of large hydrated sulfate deposits on the surface on Mars by the OMEGA instrument onboard Mars Express, the CRISM instrument onboard the Mars Reconnaissance Orbiter, as well as by in-situ excavation by the Mars Exploration Rovers motivates examination of the role of salts in the evolution of

Martian topography and its potential habitability. The ability of salts to deform viscously under moderate stress and temperature regime lead to distinct geomorphic signatures in salt-rich terrain, such as collapse, lateral spreading, glacier-like flow, and diapirs. Such processes have been proposed recently to help explain terrain on Mars, such as the Thaumasia highlands, Valles Marineris, wrinkle ridges of the Thaumasia plateau, and the Juventae, Echus and Coprates outflow channels. Furthermore, the propensity of hydrated salts to dewater in the presence of heat could present opportunities for microbial life to be sustained in the resulting brines. Even if this heat was not sustained, evidence for past biological activity is preserved in gypsum and halite crystals on Earth, and thus potentially could be preserved on Mars. In this paper, a fresh comparative planetology discussion is presented, illustrated with terrestrial examples of salt-related landforms found around the world. In addition, we review studies of cold-loving (halophilic and psychrophilic) bacteria and archaea in Arctic brines, and discuss implications for habitability and preservation of microbial life.
KW - Geomorphology
KW - Gypsum
KW - Hydrates
KW - Hydration
KW - Landforms
KW - Ocean habitats
KW - Sodium chloride
KW - Surface topography
KW - Archaea
KW - Biological activities
KW - Comparative planetology
KW - Halite crystals
KW - Hydrated salt
KW - Hydrated sulfates
KW - In-situ
KW - Lateral spreading
KW - Mars Exploration Rover
KW - Mars Reconnaissance Orbiters
KW - MARS-EXPRESS
KW - Microbial life
KW - Outflow channel
KW - Temperature regimes
KW - Valles Marineris
KW - Martian surface analysis
SN - 978-161567160-1 (ISBN)
LA - English
J2 - Int. Astronaut. Fed. - Int. Astronaut. Congr., IAC
M3 - Conference paper
DB - Scopus
N1 - Export Date: 23 February 2023; Cited By: 0; Correspondence Address: S. M. Som; Department of Earth and Space Sciences, University of Washington, Seattle, WA 98195, United States; email: sanjoy@u.washington.edu
ER -

TY - JOUR

AU - Sutter, B.
AU - Brown, A.J.
AU - Stoker, C.R.
TI - Visible-near infrared point spectrometry of drill core samples from Río Tinto, Spain: Results from the 2005 Mars Astrobiology Research and Technology Experiment (MARTE) drilling exercise
PY - 2008
T2 - Astrobiology
VL - 8
IS - 5
SP - 1049
EP - 1060
DO - 10.1089/ast.2007.0146
UR - https://www.scopus.com/inward/record.uri?eid=2-s2.0-58049105005&doi=10.1089%2fast.2007.0146&partnerID=40&md5=7d24ee065f3758e42d46e7f39cf8fa31
AD - NASA Ames Research Center, Moffett Field, CA, United States
AD - SETI Institute, Mountain View, CA, United States
AD - Jacobs ESCG/NASA Johnson Space Center, Houston, TX, United States
AD - Mail Code JE-23 Jacobs Technology, ESCG, Houston, TX 77258-8447, P.O. Box 58447, United States
AB - Sampling of subsurface rock may be required to detect evidence of past biological activity on Mars. The Mars Astrobiology Research and Technology Experiment (MARTE) utilized the Río Tinto region, Spain, as a Mars analog site to test dry drilling technologies specific to Mars that retrieve subsurface rock for biological analysis. This work examines the usefulness of visible-near infrared (VNIR) (450-1000 nm) point spectrometry to characterize ferric iron minerals in core material retrieved during a simulated Mars drilling mission. VNIR spectrometry can indicate the presence of aqueously precipitated ferric iron minerals and, thus, determine whether biological analysis of retrieved rock is warranted. Core spectra obtained during the mission with T1 (893-897 nm) and T2 (644-652 nm) features indicate goethite-dominated samples, while relatively lower wavelength T1 (832-880 nm) features indicate hematite. Hematite/goethite molar ratios varied from 0 to 1.4, and within the 880-898 nm range, T1 features were used to estimate hematite/goethite molar ratios. Post-mission X-ray analysis detected phyllosilicates, which indicates that examining beyond the VNIR (e.g., shortwave infrared, 1000-2500 nm) will enhance the detection of other minerals formed by aqueous processes. Despite the limited spectral range of VNIR point spectrometry utilized in the MARTE Mars drilling simulation project, ferric iron minerals could be identified in retrieved core material, and their distribution served to direct core subsampling for biological analysis. © 2008 Mary Ann Liebert, Inc. 2008.
KW - Absorption
KW - Exobiology
KW - Ferric Compounds
KW - Geography
KW - Geologic Sediments
KW - Iron Compounds
KW - Laboratories
KW - Mars

KW - Minerals
KW - Research
KW - Spain
KW - Spectroscopy, Near-Infrared
KW - Technology
KW - ferric hydroxide
KW - ferric ion
KW - ferric oxide
KW - iron derivative
KW - mineral
KW - absorption
KW - article
KW - astronomy
KW - biology
KW - chemistry
KW - geography
KW - laboratory
KW - methodology
KW - near infrared spectroscopy
KW - research
KW - sediment
KW - Spain
KW - technology
KW - Drilling
KW - Infrared
KW - Iron oxides
KW - Jarosite
KW - Mars
KW - Phyllosilicates
KW - Río Tinto
SN - 15311074 (ISSN)
C2 - 19105759
LA - English
J2 - Astrobiology
M3 - Article
DB - Scopus
N1 - Export Date: 23 February 2023; Cited By: 7; Correspondence Address: B. Sutter; Mail Code JE-23 Jacobs Technology, ESCG, Houston, TX 77258-8447, P.O. Box 58447, United States; email: Brad.Sutter-2@nasa.gov
ER -

TY - JOUR
AU - Ebach, M.C.
AU - Gill, A.C.
AU - Williams, D.M.
TI - The pitfalls of astrobiogeography
PY - 2008
T2 - Astrophysics and Space Science
VL - 317
IS - 3-4
SP - 143
EP - 144
DO - 10.1007/s10509-008-9922-4

UR - https://www.scopus.com/inward/record.uri?eid=2-s2.0-55849137978&doi=10.1007%2fs10509-008-9922-4&partnerID=40&md5=a04b8b35228990774926b09096fc9428
AD - International Institute for Species Exploration, School of Life Sciences, Arizona State University, Tempe, AZ 85287-4501, PO Box 874501, United States
AD - Department of Botany, Natural History Museum, London SW7 5BD, Cromwell Road, United Kingdom
AB - Biogeography, the study of organismal distribution, adheres to the same basic reasoning no matter where organisms are distributed. Wickramasinghe and Wickramasinghe (Astrophys. Space Sci. doi: 10.1007/s10509-008-9851-2), however, are possibly the first to propose a mechanism of distribution for extraterrestrial organisms, thus venturing into the uncharted realm of astrobiogeography. The same logic used in the study of distributions on Earth applies to those of extraterrestrial microbes. Herein we attempt to introduce some pitfalls common in biogeography for those venturing into the new study of astrobiogeography. © 2008 Springer Science+Business Media B.V.
KW - Astrobiology
SN - 1572946X (ISSN)
LA - English
J2 - Astrophys. Space Sci.
M3 - Article
DB - Scopus
N1 - Export Date: 23 February 2023; Cited By: 3
ER -

TY - CONF
AU - Gowen, R.
AU - Smith, A.
AU - Winter, B.
AU - Theobald, C.
AU - Rees, K.
AU - Ball, A.J.
AU - Hagermann, A.
AU - Sheridan, S.
AU - Brown, P.
AU - Oddy, T.
AU - Dougherty, M.
AU - Church, P.
AU - Gao, Y.
AU - Jones, A.
AU - Joy, K.H.
AU - Crawford, I.
AU - Pike, T.
AU - Kumar, S.
AU - Hopf, T.
AU - Wells, N.
AU - Green, K.
AU - Ryden, K.
TI - An update on MoonLITE
PY - 2008
T2 - International Astronautical Federation - 59th International

Astronautical Congress 2008, IAC 2008
VL - 7
SP - 4359
EP - 4369
UR - https://www.scopus.com/inward/record.uri?eid=2-s2.0-77950504116&partnerID=40&md5=a3c0ecef309f8ae0af21d45b8a25998e
AD - MSSL, UCL, Dorking, United Kingdom
AD - Open University, Milton Keynes, United Kingdom
AD - Imperial College London, London, United Kingdom
AD - QinetieQ, Sevenoaks, United Kingdom
AD - Surrey Space Centre, University of Surrey, Guildford, United Kingdom
AD - UCL/Birkbeck Research School of Earth Sciences, UCL, London, WC1E 6BT, Gower Street, United Kingdom
AD - QinetiQ Ltd, Farnborough, United Kingdom
AB - MoonLITE is a proposed, UK led lunar science mission involving 4 scientific penetrators that will make in situ measurements at widely separated locations on the Moon. MoonLITE will create the first global lunar network with nodes near and far-side, and in permanently shaded crater(s). With such a network MoonLITE will be able to determine much about the interior of the Moon, including characterisation of its core. Penetrator(s) at the poles will seek and characterise frozen volatiles, possibly of cometary origin and of great importance both to human exploration and to astrobiology. MoonLITE penetrators will reach the Moon at ~300 m/s and so must be able to stand the forces associated with this impact. As part of a programme aimed to establish reliable penetrator technologies the first full-scale impact trials have been conducted and are described here.
KW - Characterisation
KW - Human exploration
KW - In-situ measurement
KW - Penetrators
KW - Science missions
KW - Lunar missions
SN - 978-161567160-1 (ISBN)
LA - English
J2 - Int. Astronaut. Fed. - Int. Astronaut. Congr., IAC
M3 - Conference paper
DB - Scopus
N1 - Export Date: 23 February 2023; Cited By: 5; Correspondence Address: R. Gowen; MSSL, UCL, Dorking, United Kingdom; email: rag@mssl.ucl.ac.uk
ER -

TY - JOUR
AU - Omelon, C.R.
TI - Endolithic microbial communities in polar desert habitats
PY - 2008
T2 - Geomicrobiology Journal
VL - 25
IS - 7-8
SP - 404
EP - 414

DO - 10.1080/01490450802403057
UR - https://www.scopus.com/inward/record.uri?eid=2-s2.0-54049125729&doi=10.1080%2f01490450802403057&partnerID=40&md5=8f135f0338e261fbc55e90f83566668d
AD - Department of Geological Sciences, The University of Texas at Austin, Austin, TX, United States
AB - Microorganisms inhabiting terrestrial endolithic habitats are widespread in polar environments including the Antarctic Dry Valleys and the Canadian high Arctic. Their ability to survive in these harsh environments is a result of their finding protection from extremes in temperature, aridity, radiation and winds by colonizing nutrient-rich subsurface habitats that provide more amenable conditions for growth, often developing as vertically stratified communities that include fungi, algae, cyanobacteria and heterotrophic bacteria. Despite finding some refuge from climatic extremes, endolithic microorganisms commonly produce extracellular polysaccharides to avoid desiccation and minimize the damaging effects of freeze-thaw cycles. These microorganisms are geochemically reactive with their endolithic surroundings, observed as heterogeneous concentrations and distributions of metals resulting from mineral dissolution and precipitation reactions as well as element and nutrient release and cycling. Novel microscopy techniques such as SEM-BSE reveal much information about the physiological state of these microorganisms in situ, and show how under specific conditions, microbe-mineral interactions produce unique biosignatures of interest to studies in astrobiology. Their ability to change in situ pH conditions shows that can be directly involved in weathering of endolithic habitats, but the ecology of a given endolithic microbial community can have varying effects on rates of rock weathering. These differences in weathering rates may be an important control on microbial species diversity in polar desert endolithic habitats.
KW - Antarctica
KW - Canada
KW - Canadian Arctic
KW - East Antarctica
KW - McMurdo Dry Valleys
KW - North America
KW - algae
KW - Bacteria (microorganisms)
KW - Cyanobacteria
KW - Fungi
KW - alga
KW - cyanobacterium
KW - desert
KW - fungus
KW - geomicrobiology
KW - habitat
KW - microbial community
KW - pH
KW - polysaccharide
KW - scanning electron microscopy
KW - Astrobiology
KW - Biosignatures

KW - Endolithic microorganisms
KW - Geomicrobiology
KW - Polar habits
SN - 15210529 (ISSN)
LA - English
J2 - Geomicrobiol. J.
M3 - Article
DB - Scopus
N1 - Export Date: 23 February 2023; Cited By: 36
ER -

TY - CONF
AU - Schilizzi, R.T.
AU - Dewdney, P.E.F.
AU - Lazio, T.J.W.
TI - The square kilometre array
PY - 2008
T2 - Proceedings of SPIE - The International Society for Optical Engineering
VL - 7012
C7 - 70121I
DO - 10.1117/12.786780
UR - https://www.scopus.com/inward/record.uri?eid=2-s2.0-66749147250&doi=10.1117%2f12.786780&partnerID=40&md5=bbd62474893475d3a874321b60232dd2
AD - SKA Program Development Office, c/- Jodrell Bank Centre for Astrophysics, The University of Manchester, Manchester M13 9PL, United Kingdom
AD - Naval Research Laboratory, WA, DC 20375-5351, 4555 Overlook Ave. SW, United States
AB - The SKA will have a collecting area of up to one million square metres spread over at least 3000 km, providing a sensitivity 50 times higher than the Expanded Very Large Array, and an instantaneous field of view (FoV) of at least several tens of square degrees and possibly 250 square degrees. The SKA science impact will be widely felt in astroparticle physics and cosmology, fundamental physics, galactic and extragalactic astronomy, solar system science and astrobiology. In this paper, we describe the main features of the SKA, paying attention to the design activities around the world, and outline plans for the final design and phased implementation of the telescope.
KW - Airborne telescopes
KW - Astrobiology
KW - Cosmology
KW - Optical instruments
KW - Optical telescopes
KW - Radar astronomy
KW - Radio astronomy
KW - Solar system
KW - Astroparticle physics
KW - Design activity
KW - Extragalactic astronomy
KW - Field of views
KW - Fundamental physics

KW - Science impact
KW - Square kilometre array
KW - System science
KW - Very large arrays
KW - Astronomical satellites
KW - Radio astronomy
KW - Square kilometre array
SN - 0277786X (ISSN); 978-081947222-9 (ISBN)
LA - English
J2 - Proc SPIE Int Soc Opt Eng
M3 - Conference paper
DB - Scopus
N1 - Export Date: 23 February 2023; Cited By: 29; CODEN: PSISD
ER -

TY - JOUR
AU - Stoker, C.R.
AU - Cannon, H.N.
AU - Dunagan, S.E.
AU - Lemke, L.G.
AU - Glass, B.J.
AU - Miller, D.
AU - Gomez-Elvira, J.
AU - Davis, K.
AU - Zavaleta, J.
AU - Winterholler, A.
AU - Roman, M.
AU - Rodriguez-Manfredi, J.A.
AU - Bonaccorsi, R.
AU - Bell, M.S.
AU - Brown, A.
AU - Battler, M.
AU - Chen, B.
AU - Cooper, G.
AU - Davidson, M.
AU - Fernández-Remolar, D.
AU - Gonzales-Pastor, E.
AU - Heldmann, J.L.
AU - Martínez-Frías, J.
AU - Parro, V.
AU - Prieto-Ballesteros, O.
AU - Sutter, B.
AU - Schuerger, A.C.
AU - Schutt, J.
AU - Rull, F.
TI - The 2005 MARTE robotic drilling experiment in Río Tinto, Spain: Objectives, approach, and results of a simulated mission to search for life in the martian subsurface
PY - 2008
T2 - Astrobiology
VL - 8
IS - 5
SP - 921
EP - 945

DO - 10.1089/ast.2007.0217
UR - https://www.scopus.com/inward/record.uri?eid=2-s2.0-58049096286&doi=10.1089%2fast.2007.0217&partnerID=40&md5=d02204923f88b69f662a1b8b7ae92e2b
AD - NASA Ames Research Center, Moffett Field, CA, United States
AD - School of Aerospace and Mechanical Engineering, University of Oklahoma, Norman, OK, United States
AD - Centro de Astrobiología, CSIC/INTA, Madrid, Spain
AD - Honeybee Robotics Spacecraft Mechanisms Corporation, New York, NY, United States
AD - NASA Johnson Space Center, Houston, TX, United States
AD - SETI Institute, r2 Mountain View, CA, United States
AD - University of New Brunswick, Canada
AD - Civil and Environmental Engineering, University of California Berkeley, Berkeley, CA, United States
AD - Department of Geosciences, Princeton University, Princeton, NJ, United States
AD - Department of Plant Pathology, University of Florida, Gainesville, FL, United States
AD - Mars Institute, Moffett Field, CA, United States
AD - University of Western Ontario, Centre for Planetary Science and Exploration, London, ON, Canada
AD - Mail Stop 245-3, NASA Ames Research Center, Moffett Field, CA 94035, United States
AB - The Mars Astrobiology Research and Technology Experiment (MARTE) simulated a robotic drilling mission to search for subsurface life on Mars. The drill site was on Peña de Hierro near the headwaters of the Río Tinto river (southwest Spain), on a deposit that includes massive sulfides and their gossanized remains that resemble some iron and sulfur minerals found on Mars. The mission used a fluidless, 10-axis, autonomous coring drill mounted on a simulated lander. Cores were faced; then instruments collected color wide-angle context images, color microscopic images, visible-near infrared point spectra, and (lower resolution) visible-near infrared hyperspectral images. Cores were then stored for further processing or ejected. A borehole inspection system collected panoramic imaging and Raman spectra of borehole walls. Life detection was performed on full cores with an adenosine triphosphate luciferin-luciferase bioluminescence assay and on crushed core sections with SOLID2, an antibody array-based instrument. Two remotely located science teams analyzed the remote sensing data and chose subsample locations. In 30 days of operation, the drill penetrated to 6 m and collected 21 cores. Biosignatures were detected in 12 of 15 samples analyzed by SOLID2. Science teams correctly interpreted the nature of the deposits drilled as compared to the ground truth. This experiment shows that drilling to search for subsurface life on Mars is technically feasible and scientifically rewarding. © 2008 Mary Ann Liebert, Inc.
KW - Exobiology
KW - Geography
KW - Goals
KW - Mars
KW - Research
KW - Robotics

KW - Space Simulation
KW - Spain
KW - Technology
KW - article
KW - astronomy
KW - biology
KW - geography
KW - methodology
KW - motivation
KW - research
KW - robotics
KW - space flight
KW - Spain
KW - technology
KW - Drilling
KW - Field test
KW - Life detection
KW - Mars
KW - Mission simulation
KW - Subsurface biology
SN - 15311074 (ISSN)
C2 - 19032053
LA - English
J2 - Astrobiology
M3 - Article
DB - Scopus
N1 - Export Date: 23 February 2023; Cited By: 45; Correspondence Address: C. R. Stoker; Mail Stop 245-3, NASA Ames Research Center, Moffett Field, CA 94035, United States; email: cstoker@mail.arc.nasa.gov
ER -

TY - JOUR
AU - Miller, D.P.
AU - Bonaccorsi, R.
AU - Davis, K.
TI - Design and practices for use of automated drilling and sample handling in MARTE while minimizing terrestrial and cross contamination
PY - 2008
T2 - Astrobiology
VL - 8
IS - 5
SP - 947
EP - 965
DO - 10.1089/ast.2007.0147
UR - https://www.scopus.com/inward/record.uri?eid=2-s2.0-58049129496&doi=10.1089%2fast.2007.0147&partnerID=40&md5=42cfc7f01376cc0f5686991f9c3952cb
AD - University of Oklahoma, Norman, OK, United States
AD - Ames Research Center, Moffett Field, CA, United States
AD - Honeybee Robotics Spacecraft Mechanisms Corporation, New York, United States
AD - University of Oklahoma, Norman, OK 73019, 865 Asp Ave, United

States
AB - Mars Astrobiology Research and Technology Experiment (MARTE) investigators used an automated drill and sample processing hardware to detect and categorize life-forms found in subsurface rock at Río Tinto, Spain. For the science to be successful, it was necessary for the biomass from other sources - whether from previously processed samples (cross contamination) or the terrestrial environment (forward contamination) - to be insignificant. The hardware and practices used in MARTE were designed around this problem. Here, we describe some of the design issues that were faced and classify them into problems that are unique to terrestrial tests versus problems that would also exist for a system that was flown to Mars. Assessment of the biomass at various stages in the sample handling process revealed mixed results; the instrument design seemed to minimize cross contamination, but contamination from the surrounding environment sometimes made its way onto the surface of samples. Techniques used during the MARTE Río Tinto project, such as facing the sample, appear to remove this environmental contamination without introducing significant cross contamination from previous samples. © 2008 Mary Ann Liebert, Inc.
KW - Adenosine Triphosphate
KW - Automation
KW - Environmental Pollutants
KW - Exobiology
KW - Geologic Sediments
KW - Luminescent Measurements
KW - Mars
KW - Research
KW - Robotics
KW - Space Simulation
KW - Spacecraft
KW - Spain
KW - Technology
KW - adenosine triphosphate
KW - article
KW - astronomy
KW - automation
KW - biology
KW - instrumentation
KW - luminescence
KW - methodology
KW - pollutant
KW - research
KW - robotics
KW - sediment
KW - space flight
KW - Spain
KW - technology
KW - Contamination
KW - Engineering constraints
KW - Subsurface biosphere
SN - 15311074 (ISSN)
C2 - 19105753
LA - English

J2 - Astrobiology
M3 - Article
DB - Scopus
N1 - Export Date: 23 February 2023; Cited By: 7; Correspondence Address: D. P. Miller; University of Oklahoma, Norman, OK 73019, 865 Asp Ave, United States; email: dpmiller@ou.ed
ER -

TY - JOUR
AU - Spiegel, D.S.
AU - Menou, K.
AU - Scharf, C.A.
TI - Habitable climates
PY - 2008
T2 - Astrophysical Journal
VL - 681
IS - 2
SP - 1609
EP - 1623
DO - 10.1086/588089
UR - https://www.scopus.com/inward/record.uri?eid=2-s2.0-53549099113&doi=10.1086%2f588089&partnerID=40&md5=dcea5749f924a9f20de4a7f350c002a3
AD - Department of Astronomy, Columbia University, New York, NY 10027, 550 West 120th Street, United States
AD - Columbia Astrobiology Center, Columbia University, New York, NY 10027, 550 West 120th Street, United States
AB - According to the standard liquid-water definition, the Earth is only partially habitable. We reconsider planetary habitability in the framework of energy balance models, the simplest seasonal models in physical climatology, to assess the spatial and temporal habitability of Earth-like planets. We quantify the degree of climatic habitability of our models with several metrics of fractional habitability. Previous evaluations of habitable zones may have omitted important climatic conditions by focusing on close solar system analogies. For example, we find that model pseudoEarths with different rotation rates or different land-ocean fractions have fractional habitabilities that differ significantly from that of the Earth itself. Furthermore, the stability of a planet's climate against albedo-feedback snowball events strongly impacts its habitability. Therefore, issues of climate dynamics may be central in assessing the habitability of discovered terrestrial exoplanets, especially if astronomical forcing conditions are different from the moderate solar system cases. © 2008. The American Astronomical Society. All rights reserved.
KW - Astrobiology
KW - Planetary systems
KW - Radiative transfer
PB - Institute of Physics Publishing
SN - 0004637X (ISSN)
LA - English
J2 - Astrophys. J.
M3 - Article
DB - Scopus

N1 - Export Date: 23 February 2023; Cited By: 76; Correspondence Address: D. S. Spiegel; Department of Astronomy, Columbia University, New York, NY 10027, 550 West 120th Street, United States; email: dave@astro.columbia.edu
ER -

TY - JOUR
AU - Ivarsson, M.
AU - Lausmaa, J.
AU - Lindblom, S.
AU - Broman, C.
AU - Holm, N.G.
TI - Fossilized microorganisms from the emperor seamounts: Implications for the search for a subsurface fossil record on earth and mars
PY - 2008
T2 - Astrobiology
VL - 8
IS - 6
SP - 1139
EP - 1157
DO - 10.1089/ast.2007.0226
UR - https://www.scopus.com/inward/record.uri?eid=2-s2.0-66149181918&doi=10.1089%2fast.2007.0226&partnerID=40&md5=c8f0af94b01e95745e8689b35f8d7c7f
AD - Department of Geology and Geochemistry, Stockholm University, S-106 91 Stockholm, Sweden
AD - Swedish National Testing, Research Institute, Bors, Sweden
AD - Swedish Museum of Natural History, Stockholm, Sweden
AB - We have observed filamentous carbon-rich structures in samples drilled at 3 different seamounts that belong to the Emperor Seamounts in the Pacific Ocean: Detroit (81 Ma), Nintoku (56 Ma), and Koko Seamounts (48 Ma). The samples consist of low-temperature altered basalts recovered from all 3 seamounts. The maximum depth from which the samples were retrieved was 954 meters below seafloor (mbsf). The filamentous structures occur in veins and fractures in the basalts, where they are attached to the vein walls and embedded in vein-filling minerals like calcite, aragonite, and gypsum. The filaments were studied with a combination of optical microscopy, environmental scanning electron microscopy (ESEM), Raman spectroscopy, and time-of-flight secondary ion mass spectrometry (ToF-SIMS). Minerals were identified by a combination of optical microscopy, X-ray diffraction, Raman spectrometry, and energy dispersive spectrometry on an environmental scanning electron microscope. Carbon content of the filaments ranges between ~10 wt and ~50 wt and is not associated with carbonates. These results indicate an organic origin of the carbon. The presence of C 2H4, phosphate, and lipid-like molecules in the filaments further supports a biogenic origin. We also found microchannels in volcanic glass enriched in carbon (~10-40 wt) compatible with putative microbial activity. Our findings suggest new niches for life in subseafloor environments and have implications for further exploration of the subseafloor biosphere on Earth and beyond. © 2008 Mary Ann Liebert, Inc..

KW - Bacteria
KW - Biological Markers
KW - Calcium Carbonate
KW - Calcium Sulfate
KW - Earth (Planet)
KW - Extraterrestrial Environment
KW - Fossils
KW - Geography
KW - Glass
KW - Mars
KW - Mass Spectrometry
KW - Minerals
KW - Pacific Ocean
KW - Space Flight
KW - Spectrum Analysis, Raman
KW - Volcanic Eruptions
KW - X-Ray Diffraction
KW - Zeolites
KW - Chamaesyce atrococca
KW - biological marker
KW - calcium carbonate
KW - calcium sulfate
KW - glass
KW - mineral
KW - phillipsite
KW - zeolite
KW - article
KW - astronomy
KW - bacterium
KW - chemistry
KW - fossil
KW - geography
KW - isolation and purification
KW - mass spectrometry
KW - microclimate
KW - Pacific Ocean
KW - Raman spectrometry
KW - space flight
KW - volcano
KW - X ray diffraction
KW - 1139-1157.
KW - Fossilized microorganisms
KW - Martian fossil record Astrobiology 8
KW - Subsurface biosphere
SN - 15311074 (ISSN)
C2 - 19191540
LA - English
J2 - Astrobiology
M3 - Article
DB - Scopus
N1 - Export Date: 23 February 2023; Cited By: 33; Correspondence Address: M. Ivarsson; Department of Geology and Geochemistry, Stockholm University, S-106 91 Stockholm, Sweden; email: magnus.ivarsson@geo.su.se

ER -

TY - JOUR
AU - Barron, L.D.
TI - Chirality and life
PY - 2008
T2 - Space Science Reviews
VL - 135
IS - 1-4
SP - 187
EP - 201
DO - 10.1007/s11214-007-9254-7
UR - https://www.scopus.com/inward/record.uri?eid=2-s2.0-46749093044&doi=10.1007%2fs11214-007-9254-7&partnerID=40&md5=d83ddeed7184a727b7d3852b80e816a1
AD - WestCHEM, Department of Chemistry, University of Glasgow, Glasgow G12 8QQ, United Kingdom
AB - Chirality, meaning handedness, pervades much of modern science, from the physics of elementary particles to the chemistry of life. The amino acids and sugars from which the central molecules of life-proteins and nucleic acids-are constructed exhibit homochirality, which is expected to be a key biosignature in astrobiology. This article provides a brief review of molecular chirality and its significance for the detection of extant or extinct life on other worlds. Fundamental symmetry aspects are emphasized since these bring intrinsic physical properties of the universe to bear on the problem of the origin and role of homochirality in the living world. © 2007 Springer Science+Business Media B.V.
KW - Acids
KW - Amines
KW - Amino acids
KW - Astrobiology
KW - Biomolecules
KW - Enantiomers
KW - Molecules
KW - Nucleic acids
KW - Organic acids
KW - Physical properties
KW - Stereochemistry
KW - Sugar (sucrose)
KW - Sugars
KW - (PL) properties
KW - Business media
KW - Homochirality
KW - Modern science
KW - Molecular chirality
KW - Springer (CO)
KW - Chirality
KW - Absolute enantioselection
KW - Homochirality
KW - Mirror symmetry breaking
KW - Origin of life
SN - 15729672 (ISSN)

LA - English
J2 - Space Sci Rev
M3 - Article
DB - Scopus
N1 - Export Date: 23 February 2023; Cited By: 119; Correspondence Address: L. D. Barron; WestCHEM, Department of Chemistry, University of Glasgow, Glasgow G12 8QQ, United Kingdom; email: laurence@chem.gla.ac.uk; CODEN: SPSRA
ER -

TY - JOUR
AU - Rivas, L.A.
AU - García-Villadangos, M.
AU - Moreno-Paz, M.
AU - Cruz-Gil, P.
AU - Gómez-Elvira, J.
AU - Parro, V.
TI - A 200-antibody microarray biochip for environmental monitoring: Searching for universal microbial biomarkers through immunoprofiling
PY - 2008
T2 - Analytical Chemistry
VL - 80
IS - 21
SP - 7970
EP - 7979
DO - 10.1021/ac8008093
UR - https://www.scopus.com/inward/record.uri?eid=2-s2.0-55549092426&doi=10.1021%2fac8008093&partnerID=40&md5=d153bb2369b0e31912468666a15b8033
AD - Laboratory of Molecular Ecology, Centro de Astrobiología (INTA-CSIC), Torrejón de Ardoz, 28850, Madrid, Spain
AD - Laboratory of Robotics and Planetary Exploration, Centro de Astrobiología (INTA-CSIC), Torrejón de Ardoz, 28850, Madrid, Carretera de Ajalvir km 4, Spain
AB - Environmental biomonitoring approaches require the measurement of either unequivocal biomarkers or specific biological profiles. Antibody microarrays constitute new tools for fast and reliable analysis of up to hundreds of biomarkers simultaneously. Herein we report 150 new polyclonal antibodies against microbial strains and environmental extracts, as well as the construction and validation of an antibody microarray (EMCHIP200, for "Environmental Monitoring Chip") containing 200 different antibodies. Each antibody was tested against its antigen for its specificity and cross-reactivity by a sandwich microarray immunoassay. The limit of detection was 0.2 ng mL-1 for some proteins and 104-105 cells mL-1 for bacterial cells and spores. Partial biochemical characterization allowed identification of polymeric compounds (proteins and polysaccharides) as some of the targets recognized by the antibodies. We have successfully used the EMCHIP200 for the detection of biological polymers in samples from extreme environments around the world (e.g., a deep South African mine, Antarctica's dry valleys, Yellowstone National Park, Iceland, and Rio Tinto surface and subsurface). Clustering analysis permitted us to associate similar

immunoprofiles or patterns to samples from apparently very different environments, indicating that they indeed share similar universal biomarkers. Our EMCHIP200 constitutes a new generation of immunosensors for biomarker detection and profiling, for either environmental, industrial, biotechnological, or astrobiological applications. © 2008 American Chemical Society.
KW - Antibodies
KW - Biological Markers
KW - Environmental Monitoring
KW - Immunoassay
KW - Microarray Analysis
KW - Reproducibility of Results
KW - Soil Microbiology
KW - ABS resins
KW - Antigens
KW - Bioassay
KW - Biomarkers
KW - Biopolymers
KW - Environmental engineering
KW - Immunosensors
KW - Microarrays
KW - Parks
KW - Polysaccharides
KW - Proteins
KW - bacterial polysaccharide
KW - bacterial protein
KW - biological marker
KW - polyclonal antibody
KW - Antarctica
KW - antibody microarray
KW - antibody specificity
KW - antigen antibody reaction
KW - antigen recognition
KW - article
KW - bacterial cell
KW - bacterial spore
KW - biochip
KW - biological monitoring
KW - cluster analysis
KW - cross reaction
KW - environmental monitoring
KW - Iceland
KW - immunoassay
KW - immunosensor
KW - microarray analysis
KW - microbial biomass
KW - microorganism detection
KW - mining
KW - nonhuman
KW - protein analysis
KW - protein determination
KW - South Africa
KW - Antarctica
KW - Antibody microarrays

KW - Applications.
KW - Bacterial cells
KW - Biochemical characterizations
KW - Biological polymers
KW - Clustering analyses
KW - Dry valleys
KW - Environmental biomonitoring
KW - Environmental monitoring
KW - Extreme environments
KW - Iceland
KW - Limit of detections
KW - Microarray immunoassays
KW - Microbial strains
KW - New generations
KW - New tools
KW - Polyclonal antibodies
KW - Polymeric compounds
KW - Reliable analyses
KW - Rio Tinto
KW - Yellowstone National parks
KW - Antibodies
SN - 00032700 (ISSN)
C2 - 18837515
LA - English
J2 - Anal. Chem.
M3 - Article
DB - Scopus
N1 - Export Date: 23 February 2023; Cited By: 59; Correspondence Address: V. Parro; Laboratory of Molecular Ecology, Centro de Astrobiología (INTA-CSIC), Torrejón de Ardoz, 28850, Madrid, Spain; email: parrogv@inta.es; CODEN: ANCHA
ER -

TY - JOUR
AU - David, L.
TI - Mars exploration: Digging deeper
PY - 2008
T2 - Aerospace America
VL - 46
IS - 1
SP - 32
EP - 37
UR - https://www.scopus.com/inward/record.uri?eid=2-s2.0-38849170446&partnerID=40&md5=de15b9f9fa4c0ad5dbac3e261ef85027
AB - The NASA-sponsored National Research Council's Space Studies Board, an arm of the National Academy of Sciences, issued a seminal report 'An Astrobiology Strategy for the Exploration of Mars'. The NRC study reported that the approach should be expanded to include 'follow the carbon' along with water and other key biologically relevant elements. The NRC highlighted the robotic return of the samples as a top priority recommending that the highest-priority science objective for Mars exploration must be the analysis of diverse suits of appropriate samples returned from carefully selected regions on Mars. Report also urges the international

collaboration on Mars activities to make mission more affordable, especially in the areas of sample catching and sample return. One area of relativity straightforward collaboration involves encouraging ESA to include sample-catching capability on the Mars rovers.
SN - 0740722X (ISSN)
LA - English
J2 - Aerosp. Am.
M3 - Article
DB - Scopus
N1 - Export Date: 23 February 2023; Cited By: 0; CODEN: AEAME
ER -

TY - JOUR
AU - Covault, C.
TI - Seeking alien life
PY - 2008
T2 - Aviation Week and Space Technology (New York)
VL - 169
IS - 12
SP - 66
EP - 67
UR - https://www.scopus.com/inward/record.uri?eid=2-s2.0-53349109680&partnerID=40&md5=7ac2b467f3321c948a6ec312ee0f816c
AB - NASA continues to focus on undertaking space research projects aimed at exploring extraterrestrial life. The agency is making efforts at involving international partners in these projects. Its space research programs are facing significant competition from developing space research programs in several European countries, Japan, and China. The agency and its partners are making significant investments in robotic sensor and spacecraft development projects. These space research agencies from all over the world are undertaking these projects, to explore large number of galaxies, suns, and planets that exist in space. NASA continues to fund laboratory experiments, to investigate the origin of life revived planetary science, and sponsored theoretical and observational studies on planetary systems from 1960s.
SN - 00052175 (ISSN)
LA - English
J2 - Aviat. Week Space Technol. (New York)
M3 - Article
DB - Scopus
N1 - Export Date: 23 February 2023; Cited By: 0; CODEN: AWSTA
ER -

TY - CHAP
AU - Horneck, G.
TI - Search for Life in the Universe - What can we Learn from our own Biosphere?
PY - 2008
T2 - Reviews in Modern Astronomy
VL - 19
SP - 215
EP - 235

DO - 10.1002/9783527619030.ch10
UR - https://www.scopus.com/inward/record.uri?eid=2-s2.0-84889826383&doi=10.1002%2f9783527619030.ch10&partnerID=40&md5=c31c0f3822f9d7c7055d2870bf0b52e2
AD - DLR, Institute of Aerospace Medicine, D 51170 Köln, Germany
AB - Space exploration has extended the boundaries of biological investigations beyond the Earth to other planets, moons, comets, meteorites, and space at large. This field is covered by the new multidiscipline of astrobiology which has focused on the different steps of evolutionary pathways through cosmic history that are related to the origin, evolution and distribution of life on Earth, or elsewhere in the universe. The over-riding objective of astrobiological research has been to attain a better understanding of the principles leading to the emergence of life from inanimate matter, its evolution, and its distribution, thereby building the foundations for the construction and testing of meaningful axioms to support a theory of life. In the endeavor to quest for life on other celestial odies of our solar system or beyond, clues can be gained from the history of our own biosphere and its strategies to adapt to changing and even "extreme" environmental conditions. Assuming water in liquid phase as universal prerequisite for habitability, the neighbor planet Mars and Jupiter's moon Europa are now favored targets of astrobiological research.
KW - Astrobiological
KW - Earth
KW - Habitability
KW - Meteorites
KW - Planets
PB - Wiley-VCH Verlag GmbH & Co. KGaA
SN - 352740662X (ISBN); 978-352740662-3 (ISBN)
LA - English
J2 - Rev. in Mod. Astron.
M3 - Book chapter
DB - Scopus
N1 - Export Date: 23 February 2023; Cited By: 3; Correspondence Address: G. Horneck; DLR, Institute of Aerospace Medicine, D 51170 Köln, Germany; email: gerda.horneck@dlr.de
ER -

TY - CONF
AU - Jiao, D.
AU - Perry, R.S.
AU - Engel, M.H.
AU - Sephton, M.A.
TI - Biomarker indicators of bacterial activity and organic fluxes during end Triassic mass extinction event
PY - 2008
T2 - Proceedings of SPIE - The International Society for Optical Engineering
VL - 7097
C7 - 709709
DO - 10.1117/12.796160
UR - https://www.scopus.com/inward/record.uri?eid=2-s2.0-55549110384&doi=10.1117%2f12.796160&partnerID=40&md5=cd05432d6c

7a727a004c29e74f8ead33
AD - Impacts and Astromaterials Research Centre, Department of Earth Science and Engineering, Imperial College, London SW7 2AZ, United Kingdom
AD - Planetary Science Institute, Seattle, WA 98115, United States
AD - School of Geology and Geophysics, University of Oklahoma, Norman, OK 73019, 100 East Boyd St., United States
AB - Lipid biomarker analyses of sedimentary organic matter from a marine Triassic-Jurassic (T-J) section at Queen Charlotte Islands, British Columbia reveal significant bacterial activity and microbial community changes that coincide with faunal extinctions across the T-J boundary. Bacterial activity is indicated by the 25-norhopane biodegradation index (25-norhopanes / 25-norhopanes+regular hopanes). Microbial community changes are revealed by variations in relative abundance of 2-methylhopane, which is mainly generated from cyanobacteria. The 2-methylhopane index (2-methyl hopane/ C30 hopane + C29 25-norhopane) increases above the radiolarian based T-J boundary, and coincides with changes in the 25-norhopane index. The data reveal a complex microbial event involving both autotrophic and heteorotrophic bacteria responding to variations in allochthonous organic matter and nutrient supply.
KW - Astrobiology
KW - Bacteriology
KW - Biochemistry
KW - Biogeochemistry
KW - Biological materials
KW - Degradation
KW - Microbiology
KW - Organic compounds
KW - Allochthonous organic matters
KW - Bacterial activities
KW - Biomarker indicators
KW - British Columbia
KW - Cyanobacteria
KW - Hopanes
KW - Jurassic
KW - Lipid biomarkers
KW - Mass extinction
KW - Mass extinction events
KW - Methylhopane
KW - Microbial communities
KW - Nutrient supplies
KW - Relative abundances
KW - Sedimentary organic matters
KW - Triassic
KW - Triassic-Jurassic
KW - Biodegradation
KW - Biodegradation
KW - Biomarker
KW - Cyanobacteria
KW - Mass extinction
KW - Triassic-Jurassic
SN - 0277786X (ISSN); 978-081947317-2 (ISBN)
LA - English

J2 - Proc SPIE Int Soc Opt Eng
M3 - Conference paper
DB - Scopus
N1 - Export Date: 23 February 2023; Cited By: 5; Correspondence Address: D. Jiao; Impacts and Astromaterials Research Centre, Department of Earth Science and Engineering, Imperial College, London SW7 2AZ, United Kingdom; email: dan.jiao05@imperial.ac.uk; CODEN: PSISD
ER -

TY - CONF
AU - Sheldon, R.B.
AU - Hoover, R.B.
TI - Cosmological evolution: Spatial relativity and the speed of life
PY - 2008
T2 - Proceedings of SPIE - The International Society for Optical Engineering
VL - 7097
C7 - 709716
DO - 10.1117/12.802195
UR - https://www.scopus.com/inward/record.uri?eid=2-s2.0-55549132927&doi=10.1117%2f12.802195&partnerID=40&md5=b9bacb82356855ac4be973242356b6ab
AD - USRA/NSSTC, Huntsville, AL, 320 Sparkman Dr., United States
AD - NASA/MSFC/NSSTC, Huntsville, AL, 320 Sparkman Dr., United States
AB - Three recent discoveries support the existence of an extra-planetary, cometary biosphere that is capable of spanning the galaxy: 1) the discovery of ancient cyanobacterial fossils on carbonaceous chondrites, which are widely believed to be extinct comets, 2) the observations and theory that all short-period comets have irreversibly melted and undergo periodic remeltings, and, 3) the observation that comets can accrete inner solar-system material, including spores from other infected comets. While no direct observation of sub-cellular, fossilized viruses exist, their ubiquity and proximity with cyanobacteria, suggest that the proposed cometary biosphere also carries a full complement of bacteriophages. The recent work transcribing viral DNA of bacteriophages reveals an active horizontal transfer of genes though a vector that doesn't itself benefit from the genes. Thus the cometary biosphere is capable of transporting genes throughout the galaxy that are not themselves expressed in space. This suggests that evolution may occur not just in time, but in space as well, making the Earth and its history less significant for a cosmological theory of evolution. By analogy to relativity, we propose that the ratio of spatial to temporal evolution is more constant than either term alone, which we call the speed of life.
KW - Astrobiology
KW - Biology
KW - Biospherics
KW - Cosmology
KW - Galaxies
KW - Genes

KW - Meteorites
KW - Nucleic acids
KW - Organic acids
KW - Relativity
KW - Solar system
KW - Viruses
KW - Biosphere
KW - Comet
KW - Cometary water
KW - Cyanobacteria
KW - Microfossil
KW - Panspermia
KW - Bacteriophages
KW - Astrobiology
KW - Biosphere
KW - Comet
KW - Cometary water
KW - Cyanobacteria
KW - Microfossil
KW - Panspermia
SN - 0277786X (ISSN); 978-081947317-2 (ISBN)
LA - English
J2 - Proc SPIE Int Soc Opt Eng
M3 - Conference paper
DB - Scopus
N1 - Export Date: 23 February 2023; Cited By: 6; Correspondence Address: R. B. Sheldon; USRA/NSSTC, Huntsville, AL, 320 Sparkman Dr., United States; email: Rob.Sheldon@msfc.nasa.gov; CODEN: PSISD
ER -

TY - JOUR
AU - Benison, K.C.
AU - Jagniecki, E.A.
AU - Edwards, T.B.
AU - Mormile, M.R.
AU - Storrie-Lombardi, M.C.
TI - "hairy blobs:" Microbial suspects preserved in modern and ancient extremely acid lake evaporites
PY - 2008
T2 - Astrobiology
VL - 8
IS - 4
SP - 807
EP - 821
DO - 10.1089/ast.2006.0034
UR - https://www.scopus.com/inward/record.uri?eid=2-s2.0-54049122286&doi=10.1089%2fast.2006.0034&partnerID=40&md5=9ad164d7e24b75766838ed84589e47db
AD - Department of Geology, Central Michigan University, Mt. Pleasant, MI, United States
AD - Department of Biological Sciences, Missouri University of Science and Technology, Rolla, MO, United States
AD - Kinohi Institute, Pasadena, CA, United States
AD - U.S. Oil and Refining Company, Tacoma, WA, United States

AD - Department of Geology, Central Michigan University, Mt. Pleasant, MI 48858, United States
AB - "Hairy blobs" are unusual clumps of organic bodies and sulfate crystals that have been found in evaporite minerals grown in acid saline lakes. Here, we document modern hairy blobs in halite and gypsum from 5 modern acid saline lakes in southern Western Australia, and Permian hairy blobs trapped in halite from the mid-Permian Opeche Shale in the subsurface of North Dakota. These are among the first microbial remains described from acid saline lake environments. They give clues about the role of microorganisms in the acidity, geochemistry, and mineralogy of these extreme environments. This study also may add to the inventory of life in extreme environments and help predict possible martian life-forms and the method of preservation. © 2008 Mary Ann Liebert, Inc.
KW - Australia
KW - Calcium Sulfate
KW - Earth (Planet)
KW - Environmental Microbiology
KW - Extraterrestrial Environment
KW - Geologic Sediments
KW - Hydrogen-Ion Concentration
KW - Lasers
KW - Mars
KW - Microscopy, Ultraviolet
KW - Sodium Chloride
KW - Spectrum Analysis, Raman
KW - United States
KW - Water
KW - Water Microbiology
KW - Acidophiles
KW - calcium sulfate
KW - sodium chloride
KW - water
KW - article
KW - astronomy
KW - Australia
KW - laser
KW - microbiology
KW - microclimate
KW - microscopy
KW - pH
KW - Raman spectrometry
KW - sediment
KW - United States
KW - Acidophiles
KW - Astrobiology
KW - Exobiology
KW - Halophiles
KW - Life in acid saline environments
KW - Microfossils
SN - 15311074 (ISSN)
C2 - 18498219
LA - English
J2 - Astrobiology

M3 - Article
DB - Scopus
N1 - Export Date: 23 February 2023; Cited By: 45; Correspondence Address: K. C. Benison; Department of Geology, Central Michigan University, Mt. Pleasant, MI 48858, United States; email: benis1kc@cmich.edu
ER -

TY - JOUR
AU - Marion, G.M.
AU - Kargel, J.S.
TI - Limits for life
PY - 2008
T2 - Advances in Astrobiology and Biogeophysics
SP - 79
EP - 99
DO - 10.1007/978-3-540-75679-8_4
UR - https://www.scopus.com/inward/record.uri?eid=2-s2.0-37549021438&doi=10.1007%2f978-3-540-75679-8_4&partnerID=40&md5=56f7fc74a21a94a40f409a20f5503e78
AD - Earth and Ecosystem Sciences, Desert Research Institute, Reno, NV 89512, 2215 Raggio Parkway, United States
AD - Department of Hydrology and Water Resources, University of Arizona, Tucson, AZ 85721, United States
AB - The current mantra of astrobiology is "Follow the Water" Where there is water, there may be life. The FREZCHEM model can determine the presence or absence of water down to the eutectic temperature, below which only solid phases are thermodynamically stable. Salinity, the desiccation potential, and acidity are other potentially life-limiting factors that are calculated by FREZCHEM. In Chapter 4, we discuss potential life-limiting factors such as temperature, salinity, acidity, desiccation, radiation, pressure, and time. © 2008 Springer-Verlag Berlin Heidelberg.
A2 - Marion null
A2 - Earth and Ecosystem Sciences, Desert Research Institute, Reno, NV 89512, 2215 Raggio Parkway
A2 - Kargel null
A2 - University of Arizona, Departement of Hydrology and, Water Resources, Tucson, AZ 85721
SN - 16131851 (ISSN); 978-354075678-1 (ISBN)
LA - English
J2 - Adv. Astrobiol. Biogeophys.
M3 - Article
DB - Scopus
N1 - Export Date: 23 February 2023; Cited By: 0
ER -

TY - CHAP
AU - Miteva, V.
TI - Bacteria in snow and glacier ice
PY - 2008
T2 - Psychrophiles: From Biodiversity to Biotechnology
SP - 31
EP - 50

DO - 10.1007/978-3-540-74335-4_3
UR - https://www.scopus.com/inward/record.uri?eid=2-s2.0-57449083753&doi=10.1007%2f978-3-540-74335-4_3&partnerID=40&md5=f33de77c7876a14a3a403252b3cec9db
AD - Department of Biochemistry and Molecular Biology, Pennsylvania State University, PA 16803, University Park, United States
AB - By definition, the cryosphere is the portion of the Earth where water is in solid form as snow or ice. It includes vast areas of sea ice, freshwater ice, glaciers, ice sheets, snow cover and permafrost. Because of the extremely harsh climatic conditions, these frozen environments had been considered for a long time to be devoid of life or serving merely as repositories for wind-transported microorganisms trapped in the ice (Cowan and Tow 2004). However, the increasing number of recent studies on the microbial ecology and diversity of natural ice samples have changed this view. Although still limited, they have shown that permanently frozen environments harbor abundant, live and diverse microorganisms that may be detected and recovered by cultivation. Priscu and Christner (2004) calculated the total number of bacterial cells in the Antarctic and Greenland ice sheets to be 9.61×1025, which corresponds to a significant carbon pool of 2.65×10-3 Pg (1 Pg = 1015 g) and represents a considerable reservoir of microbial diversity. At present, the cryosphere is important not only as an integral part of the global climate system but as one of the major habitable ecosystems of Earth's biosphere and as the best analogue for the search of extraterrestrial life. This chapter presents the current knowledge about the detection, diversity, survival and activity of bacteria in snow and glacier ice. Because this topic has been previously reviewed (Priscu and Christner 2004; Cowan and Tow 2004; Priscu et al. 2007), the focus here will be to outline major earlier findings and to present the most recent advances in glacier ice and snow microbiology. © 2008 Springer-Verlag Berlin Heidelberg.
PB - Springer Berlin Heidelberg
SN - 978-354074334-7 (ISBN)
LA - English
J2 - Psychrophiles: From Biodiv. to Biotech.
M3 - Book chapter
DB - Scopus
N1 - Export Date: 23 February 2023; Cited By: 89; Correspondence Address: V. Miteva; Department of Biochemistry and Molecular Biology, Pennsylvania State University, PA 16803, University Park, United States; email: vim1@psu.edu
ER -

TY - JOUR
AU - Schorghofer, N.
TI - The lifetime of ice on main belt asteroids
PY - 2008
T2 - Astrophysical Journal
VL - 682
IS - 1
SP - 697
EP - 705
DO - 10.1086/588633

UR - https://www.scopus.com/inward/record.uri?eid=2-s2.0-48749111228&doi=10.1086%2f588633&partnerID=40&md5=6694be5e70bfbba9e703b9a9a8f49877
AD - Institute for Astronomy, NASA Astrobiology Institute, University of Hawaii, Honolulu, HI 96822, 2680 Woodlawn Drive, United States
AB - We theoretically estimate the loss rate of buried ice from spherical bodies 2-3.3 AU from the Sun. The loss rate is explored as a function of about a dozen parameters. We introduce the concept of a "buried snow line," where the loss of ice is sufficiently slow over the age of the solar system. For a dusty surface layer, ice can persist within the top few meters of the surface over billions of years, if the mean surface temperature is less than about 145 K. Variations in surface layer properties within a plausible range are unlikely to change this threshold temperature by more than 10 K. Longevity of ice in the shallow subsurface of asteroid 7968 Elst-Pizarro is plausible. Parameter regions for ice to survive over the age of the solar system exist for all of the main asteroid belt, but preferentially for large distances from the Sun and slowly rotating bodies with surfaces consisting of small particles, leading to low thermal conductivity and short molecular free paths. Rocky surfaces, in contrast to dusty surfaces, are rarely able to retain ice in the shallow subsurface. © 2008. The American Astronomical Society. All rights reserved.
KW - Astrobiology
KW - Comets: general
KW - Minor planets, asteroids
PB - Institute of Physics Publishing
SN - 0004637X (ISSN)
LA - English
J2 - Astrophys. J.
M3 - Article
DB - Scopus
N1 - Export Date: 23 February 2023; Cited By: 145
ER -

TY - JOUR
AU - Olson, A.R.
AU - Tobin, V.V.M.
TI - An eastern orthodox perspective on microbial life on mars
PY - 2008
T2 - Theology and Science
VL - 6
IS - 4
SP - 421
EP - 437
DO - 10.1080/14746700802396171
UR - https://www.scopus.com/inward/record.uri?eid=2-s2.0-69849121000&doi=10.1080%2f14746700802396171&partnerID=40&md5=4a7df6ad595898db70ef67b9c3f07ec0
AD - Department of Environmental Sciences, Nova Scotia Agricultural College, NS, Canada
AD - St. Vladimir Orthodox Church in Halifax, Halifax, NS, Canada
AB - Recent efforts to explore the geology and climate of planets

within our own solar system, especially Mars, have prompted a renewed interest in the search for microorganisms as the most plausible forms of extraterrestrial life. As the scientific search for evidence of microbial life on Mars intensifies, there has been a perceived need to examine the theological implications in advance of such a possible discovery. Religious considerations, thus far, have focused mainly on Western Christianity as represented by the Roman Catholic and various Protestant traditions. Although Eastern Orthodoxy represents the second largest group of Christians worldwide, to date, there is very little information available from these ongoing discussions concerning an Eastern Orthodox perspective. Therefore, we first review the case for the possibility of microbial life on Mars and then explore its fundamental Orthodox theological meaning. The apprehension of any form of extraterrestrial life by the Orthodox Church will be rooted in its collective interpretationof the Holy Scriptures, Patristic and contemporary religious writers, and in the ancient liturgical expression of its worship. © 2008, Taylor & Francis Group, LLC. All rights reserved.
KW - Eastern Orthodoxy
KW - Exobiology
KW - Extraterrestrial microbial life
KW - Mars exploration
KW - Religion and space exploration
KW - Religious implications
SN - 14746700 (ISSN)
LA - English
J2 - Theology Sci.
M3 - Article
DB - Scopus
N1 - Export Date: 23 February 2023; Cited By: 3
ER -

TY - JOUR
AU - Prieto-Ballesteros, O.
AU - Martínez-Frías, J.
AU - Schutt, J.
AU - Sutter, B.
AU - Heldmann, J.L.
AU - Bell, M.S.
AU - Battler, M.
AU - Cannon, H.
AU - Gómez-Elvira, J.
AU - Stoker, C.R.
TI - The subsurface geology of Río Tinto: Material examined during a simulated mars drilling mission for the Mars Astrobiology Research and Technology Experiment (MARTE)
PY - 2008
T2 - Astrobiology
VL - 8
IS - 5
SP - 1013
EP - 1021
DO - 10.1089/ast.2006.0107

UR - https://www.scopus.com/inward/record.uri?eid=2-s2.0-58049103935&doi=10.1089%2fast.2006.0107&partnerID=40&md5=fe78645a574802dfdeed366743a3df19
AD - Centro de Astrobiología (INTA-CSIC), Torrejón de Ardoz, Madrid, Spain
AD - Mars Institute, NASA Research Park, Moffett Field, CA, United States
AD - SETI Institute, Mountain View, CA, United States
AD - NASA Ames Research Center, Moffett Field, CA, United States
AD - Johnson Space Center, NASA, Houston, TX, United States
AD - University of New Brunswick, Fredericton, Canada
AD - University of Western Ontario, Centre for Planetary Science and Exploration, London, ON, Canada
AD - Centro de Astrobiología-INTA-CSIC, Torrejón de Ardoz 28850 Madrid, Carretera de Ajalvir km. 4, Spain
AB - The 2005 Mars Astrobiology Research and Technology Experiment (MARTE) project conducted a simulated 1-month Mars drilling mission in the Río Tinto district, Spain. Dry robotic drilling, core sampling, and biological and geological analytical technologies were collectively tested for the first time for potential use on Mars. Drilling and subsurface sampling and analytical technologies are being explored for Mars because the subsurface is the most likely place to find life on Mars. The objectives of this work are to describe drilling, sampling, and analytical procedures; present the geological analysis of core and borehole material; and examine lessons learned from the drilling simulation. Drilling occurred at an undisclosed location, causing the science team to rely only on mission data for geological and biological interpretations. Core and borehole imaging was used for micromorphological analysis of rock, targeting rock for biological analysis, and making decisions regarding the next day's drilling operations. Drilling reached 606 cm depth into poorly consolidated gossan that allowed only 35 of core recovery and contributed to borehole wall failure during drilling. Core material containing any indication of biology was sampled and analyzed in more detail for its confirmation. Despite the poorly consolidated nature of the subsurface gossan, dry drilling was able to retrieve useful core material for geological and biological analysis. Lessons learned from this drilling simulation can guide the development of dry drilling and subsurface geological and biological analytical technologies for future Mars drilling missions. © 2008 Mary Ann Liebert, Inc.
KW - Exobiology
KW - Geologic Sediments
KW - Geological Phenomena
KW - Mars
KW - Research
KW - Space Simulation
KW - Spain
KW - Technology
KW - article
KW - astronomy
KW - biology
KW - geographic and geological phenomena
KW - instrumentation

KW - research
KW - sediment
KW - space flight
KW - Spain
KW - technology
KW - Drilling core
KW - Iron oxides
KW - Mars
KW - Remote mineralogical analysis
KW - Río Tinto
SN - 15311074 (ISSN)
C2 - 19105757
LA - English
J2 - Astrobiology
M3 - Article
DB - Scopus
N1 - Export Date: 23 February 2023; Cited By: 11; Correspondence Address: O. Prieto-Ballesteros; Centro de Astrobiología-INTA-CSIC, Torrejón de Ardoz 28850 Madrid, Carretera de Ajalvir km. 4, Spain; email: prietobo@inta.es
ER -

TY - JOUR
AU - Des Marais, D.J.
AU - Nuth 3rd., J.A.
AU - Allamandola, L.J.
AU - Boss, A.P.
AU - Farmer, J.D.
AU - Hoehler, T.M.
AU - Jakosky, B.M.
AU - Meadows, V.S.
AU - Pohorille, A.
AU - Runnegar, B.
AU - Spormann, A.M.
TI - The NASA Astrobiology Roadmap.
PY - 2008
T2 - Astrobiology
VL - 8
IS - 4
SP - 715
EP - 730
DO - 10.1089/ast.2008.0819
UR - https://www.scopus.com/inward/record.uri?eid=2-s2.0-58149144269&doi=10.1089%2fast.2008.0819&partnerID=40&md5=1f421c7f46890e6f676de9a31a2c7656
AD - Space Science Division, NASA Ames Research Center, Moffett Field, California, United States
AB - The NASA Astrobiology Roadmap provides guidance for research and technology development across the NASA enterprises that encompass the space, Earth, and biological sciences. The ongoing development of astrobiology roadmaps embodies the contributions of diverse scientists and technologists from government, universities, and private institutions. The Roadmap addresses three basic questions: how does life begin and evolve, does life exist elsewhere

in the universe, and what is the future of life on Earth and beyond? Seven Science Goals outline the following key domains of investigation: understanding the nature and distribution of habitable environments in the universe, exploring for habitable environments and life in our own Solar System, understanding the emergence of life, determining how early life on Earth interacted and evolved with its changing environment, understanding the evolutionary mechanisms and environmental limits of life, determining the principles that will shape life in the future, and recognizing signatures of life on other worlds and on early Earth. For each of these goals, Science Objectives outline more specific high priority efforts for the next three to five years. These eighteen objectives are being integrated with NASA strategic planning.
KW - Biogenesis
KW - Earth (Planet)
KW - Exobiology
KW - Extraterrestrial Environment
KW - Mars
KW - Planets
KW - Solar System
KW - United States
KW - United States National Aeronautics and Space Administration
KW - article
KW - astronomy
KW - biogenesis
KW - biology
KW - government
KW - microclimate
KW - United States
SN - 15578070 (ISSN)
C2 - 18793098
LA - English
J2 - Astrobiology
M3 - Article
DB - Scopus
N1 - Export Date: 23 February 2023; Cited By: 248; Correspondence Address: D.J. Des Marais; email: David.J.DesMarais@nasa.gov
ER -

TY - CONF
AU - Baele, J.-M.
AU - Bouvain, F.
AU - De Jong, J.
AU - Matielli, N.
AU - Papier, S.
AU - Préat, A.
TI - Iron microbial mats in modern and phanerozoic environments
PY - 2008
T2 - Proceedings of SPIE - The International Society for Optical Engineering
VL - 7097
C7 - 70970N
DO - 10.1117/12.801597

UR - https://www.scopus.com/inward/record.uri?eid=2-s2.0-55549124186&doi=10.1117%2f12.801597&partnerID=40&md5=de2ff9a7561f2d556de47c1edd496483
AD - Faculté Polytechnique de Mons, 7000 Mons, 9 rue de Houdain, Belgium
AD - Université de Liège, B20 Sart Tilman, 4000 Liège, Belgium
AD - Université Libre de Bruxelles, 1050 Bruxelles, 50, Avenue F.D. Roosevelt, Belgium
AB - The recognition of iron microbial mats in terrestrial environments is of great relevance for the search for extraterrestrial life, especially on mars where significant iron minerals were identified in the subsurface. Most researches focused on very ancient microbial mats (e.g. BIFs) since they formed on Earth at a time where similar conditions are supposed to have prevailed on Mars too. However, environmental proxies are often difficult to use for these deposits on Earth which, in addition, may be heavily transformed due to diagenesis or even metamorphism. Here we present modern and phanerozoic iron microbial mats occurrences illustrating the wide variety of environments in which they form, including many marine settings, ponds, creeks, caves, volcanoes, etc. Contrarily to their Precambrian counterparts, Modern and Phanerozoic deposits are usually less affected by diagenesis and the environmental conditions likely to be better constrained. Therefore, their investigation may help for the search for morphological and geochemical biosignatures (e.g. iron isotopes) in ancient iron microbial occurrences on Earth but also on other Planets. In particular, many of the case studies presented here show that microstromatolithe-like morphologies may be valuable targets for screening potential biosignatures in various rock types.
KW - Astrobiology
KW - Isotopes
KW - Landforms
KW - Minerals
KW - Sedimentology
KW - Biosignatures
KW - Case studies
KW - Diagenesis
KW - Environmental conditions
KW - Environmental proxies
KW - Extraterrestrial lives
KW - Fe isotopes
KW - Iron isotopes
KW - Iron minerals
KW - Microbial mats
KW - Morpho-logies
KW - Phanerozoic
KW - Precambrian
KW - Rock types
KW - Terrestrial environments
KW - Iron deposits
KW - Biosignatures
KW - Diagenesis
KW - Fe isotopes
KW - Iron microbial mat

KW - Morphology
KW - Phanerozoic
SN - 0277786X (ISSN); 978-081947317-2 (ISBN)
LA - English
J2 - Proc SPIE Int Soc Opt Eng
M3 - Conference paper
DB - Scopus
N1 - Export Date: 23 February 2023; Cited By: 22; Correspondence Address: J.-M. Baele; Faculté Polytechnique de Mons, 7000 Mons, 9 rue de Houdain, Belgium; email: jean-marc.baele@fpms.ac.be; CODEN: PSISD
ER -

TY - JOUR
AU - Gammons, C.H.
AU - Nimick, D.A.
AU - Parker, S.R.
AU - Snyder, D.M.
AU - McCleskey, R.B.
AU - Amils, R.
AU - Poulson, S.R.
TI - Photoreduction fuels biogeochemical cycling of iron in Spain's acid rivers
PY - 2008
T2 - Chemical Geology
VL - 252
IS - 3-4
SP - 202
EP - 213
DO - 10.1016/j.chemgeo.2008.03.004
UR - https://www.scopus.com/inward/record.uri?eid=2-s2.0-46149110497&doi=10.1016%2fj.chemgeo.2008.03.004&partnerID=40&md5=f5449f1443b1e31d80c1ead453d7ed61
AD - Montana Tech of The University of Montana, Department of Geological Engineering, Butte, MT, United States
AD - U.S. Geological Survey, Helena, MT, United States
AD - Montana Tech of The University of Montana, Department of Chemistry and Geochemistry, Butte, MT, United States
AD - U.S. Geological Survey, Boulder, CO, United States
AD - Centro de Biología Molecular, U. Autónoma de Madrid, Madrid, Spain
AD - Centro de Astrobiología, Madrid, Spain
AD - University of Nevada-Reno, Department of Geological Sciences, Reno, NV, United States
AB - A number of investigations have shown that photoreduction of Fe(III) causes midday accumulations of dissolved Fe(II) in rivers and lakes, leading to large diel (24-h) fluctuations in the concentration and speciation of total dissolved iron. Less well appreciated is the importance of photoreduction in providing chemical energy for bacteria to thrive in low pH waters. Diel variations in water chemistry from the highly acidic (pH 2.3 to 3.1) Río Tinto, Río Odiel, and Río Agrio of southwestern Spain (Iberian Pyrite Belt) resulted in daytime increases in Fe(II) concentration of 15 to 66 μM at four diel sampling locations. Dissolved Fe(II)

concentrations increased with solar radiation, and one of the stream sites showed an antithetic relationship between dissolved Fe(II) and Fe(III) concentrations; both results are consistent with photoreduction. The diel data were used to estimate rates of microbially catalyzed Fe(II) oxidation (1 to 3 nmol L− 1 s− 1) and maximum rates of Fe(III) photoreduction (1.7 to 4.3 nmol L− 1 s− 1). Bioenergetic calculations indicate that the latter rates are sufficient to build up a population of Fe-oxidizing bacteria to the levels observed in the Río Tinto in about 30 days. We conclude that photoreduction plays an important role in the bioenergetics of the bacterial communities of these acidic rivers, which have previously been shown to be dominated by autotrophic Fe(II)-oxidizers such as Acidithiobacillus ferrooxidans and Leptospirillum ferrooxidans. Given the possibility of the previous existence of acidic, Fe(III)-rich water on Mars, photoreduction may be an important process on other planets, a fact that could have implications to astrobiological research. © 2008 Elsevier B.V. All rights reserved.
KW - Agrio River
KW - Andalucia
KW - Eurasia
KW - Europe
KW - Huelva [Andalucia]
KW - Odiel River
KW - Rio Tinto [(RVR) Huelva]
KW - Sevilla
KW - Southern Europe
KW - Spain
KW - Acidithiobacillus ferrooxidans
KW - Bacteria (microorganisms)
KW - Leptospirillum ferrooxidans
KW - acidity
KW - biogeochemical cycle
KW - concentration (composition)
KW - diel variation
KW - iron
KW - iron-reducing bacterium
KW - light effect
KW - reduction
KW - river water
KW - solar radiation
KW - water quality
KW - Astrobiology
KW - Geochemistry
KW - Iron
KW - Microbiology
KW - Photoreduction
KW - Tinto
SN - 00092541 (ISSN)
LA - English
J2 - Chem. Geol.
M3 - Article
DB - Scopus
N1 - Export Date: 23 February 2023; Cited By: 29; Correspondence Address: C.H. Gammons; Montana Tech of The University of Montana,

Department of Geological Engineering, Butte, MT, United States;
email: cgammons@mtech.edu
ER -

TY - CONF
AU - Van Heerden, E.
AU - Opperman, D.J.
AU - Bester, A.P.
AU - Van Marwijk, J.
AU - Cason, E.D.
AU - Litthauer, D.
AU - Piater, L.A.
AU - Onstott, T.C.
TI - Metabolic promiscuity from the deep subsurface: A story of Survival or Superiority
PY - 2008
T2 - Proceedings of SPIE - The International Society for Optical Engineering
VL - 7097
C7 - 70970S
DO - 10.1117/12.801142
UR - https://www.scopus.com/inward/record.uri?eid=2-s2.0-55549122649&doi=10.1117%2f12.801142&partnerID=40&md5=7ed13e3a7c81b548fbc136442f637320
AD - University of the Free State, Metagenomics Platform, Dept. of Microbial, Biochemical and Food Biotechnology, Bloemfontein, South Africa
AD - Dept. of Geosciences, Princeton University, Princeton, NJ, United States
AB - The Witwatersrand Supergroup is a 2.9-billion-year-old formation of low permeability sandstone and shale with minor volcanic units and conglomerates with an ambient rock temperature of approximately 60°C. Thermus scotoductus SA-01 was isolated from fissure water at a depth of 3.2 kmbls in a South African gold mine and it shows the ability to reduce a variety of heavy metals under anaerobic conditions. It has been postulated that such microorganisms could play an important role in nutrient and metal cycling within the subsurface. Recently, our studies indicate that the cycling of metals could also occur under aerobic conditions and not only by the action of redox active enzymes, but other diverse metabolic proteins as well. In this study the capability of specific proteins to interact with metals is elucidated. Using Thermus SA-01 and its now completed genome sequence, metal reduction is studied through classic proteomic- and genomic methods. Finally we identify thermostable enzymes responsible for the transformation of various metals (Iron, Chrome, Uranium, Gold, etc) and discuss that reduction occurs via the serendipitous action of enzymes with other primary physiological functions, some of which are classical catabolic enzymes and anabolic proteins. This paper discusses the use of a ubiquitous enzyme/protein performing more than one function, possibly detoxifying the environment and using moonlighting as resource to decrease cellular energy requirements rather than elaborate metabolism in the subsurface.
KW - Actinides

KW - Astrobiology
KW - Biochemistry
KW - Catalysts
KW - Enzymes
KW - Gold
KW - Heavy metals
KW - Heavy water
KW - Probability density function
KW - Proteins
KW - Redox reactions
KW - Simulated annealing
KW - Transuranium elements
KW - Uranium
KW - Chrome
KW - Dehydrogenases
KW - Metabolic versatility
KW - Metal reduction
KW - Reductases
KW - Thermus scotoductus SA-01
KW - Metals
KW - Chrome
KW - Dehydrogenases
KW - Gold
KW - Iron
KW - Metabolic versatility
KW - Metal reduction
KW - Reductases
KW - Thermus scotoductus SA-01
KW - Uranium
SN - 0277786X (ISSN); 978-081947317-2 (ISBN)
LA - English
J2 - Proc SPIE Int Soc Opt Eng
M3 - Conference paper
DB - Scopus
N1 - Export Date: 23 February 2023; Cited By: 1; Correspondence Address: E. Van Heerden; University of the Free State, Metagenomics Platform, Dept. of Microbial, Biochemical and Food Biotechnology, Bloemfontein, South Africa; email: vheerde.sci@ufs.ac.za; CODEN: PSISD
ER -

TY - CONF
TI - Instruments, Methods, and Missions for Astrobiology XI
PY - 2008
T2 - Proceedings of SPIE - The International Society for Optical Engineering
VL - 7097
UR - https://www.scopus.com/inward/record.uri?eid=2-s2.0-55549084201&partnerID=40&md5=46e39737ba2cc2a5b5a91fff02673a0d
AB - The proceedings contain 33 papers. The topics discussed include: microfossils of filamentous prokaryotes in CI1 and CM2 meteorites; development of living organisms on the lava-water interface of palaeoproterozoic ongeluk lavas of South Africa; the origins of amino acids in ancient terrestrial and extraterrestrial

materials; astrobiological relevance of phenols and their silicates; the early earth and its environments; biomarker indicators of bacterial activity and organic fluxes during end triassic mass extinction event; abiotic, biotic, and in-between; inferring climate change from stable isotope compositions of ancient speleothems on earth: possible implications for climatic reconstructions elsewhere in the solar system; and astrobiology-related research activity in the National Astronomical Observatory of Japan.
SN - 0277786X (ISSN); 978-081947317-2 (ISBN)
LA - English
J2 - Proc SPIE Int Soc Opt Eng
M3 - Conference review
DB - Scopus
N1 - Export Date: 23 February 2023; Cited By: 0; CODEN: PSISD
ER -

TY - BOOK
AU - Livio, M.
AU - Sahu, K.
AU - Valenti, J.
TI - A Decade of extrasolar planets around normal stars: Proceedings of the space telescope science institute symposium, held in Baltimore, Maryland May 2–5, 2005
PY - 2008
T2 - A Decade of Extrasolar Planets around Normal Stars: Proceedings of the Space Telescope Science Institute Symposium, Held in Baltimore, Maryland May 2–5, 2005
VL - 9780521897846
SP - 1
EP - 183
DO - 10.1017/CBO9780511536304
UR - https://www.scopus.com/inward/record.uri?eid=2-s2.0-84927043928&doi=10.1017%2fCBO9780511536304&partnerID=40&md5=2062b3b72a8f08374994c653d36c89b8
AD - Space Telescope Science Institute, Baltimore, 21218, MD, United States
AB - Humans have long thought that planetary systems similar to our own should exist around stars other than the Sun, yet the search for planets outside our Solar System has had a dismal history of discoveries that could not be confirmed. However, this all changed in 1995, after which astonishing progress can be seen in this field; we now know of more than 200 extrasolar planets. These findings mark crucial milestones in the search for extraterrestrial life – arguably one of the most intriguing endeavors of modern science. These proceedings from the 2005 Space Telescope Science Institute Symposium on Extrasolar Planets explore one of the hottest topics in astronomy. Discussions include the Kepler mission, observational constraints on dust disk lifetimes and the implications for planet formation, and gravitational instabilities in protoplanetary disks. With review papers written by world experts in their fields, this is an important resource on extrasolar planets. © Cambridge University Press 2008.
KW - Planets
KW - Space telescopes

KW - Stars
KW - Sun
KW - Extraterrestrial life
KW - Gravitational instability
KW - Kepler mission
KW - Modern science
KW - Planet formation
KW - Planetary system
KW - Protoplanetary disks
KW - Review papers
KW - Extrasolar planets
PB - Cambridge University Press
SN - 978-051153630-4 (ISBN); 978-052189784-6 (ISBN)
LA - English
J2 - A Decade of Extrasolar Planets around Normal Stars: Proc. of the Space Telescope Science Inst. Symposium, Held in Baltim., Md. May 2-5, 2005
M3 - Book
DB - Scopus
N1 - Export Date: 23 February 2023; Cited By: 1
ER -

TY - JOUR
AU - Van Hoolst, T.
AU - Rambaux, N.
AU - Karatekin, O.
AU - Dehant, V.
AU - Rivoldini, A.
TI - The librations, shape, and icy shell of Europa
PY - 2008
T2 - Icarus
VL - 195
IS - 1
SP - 386
EP - 399
DO - 10.1016/j.icarus.2007.12.011
UR - https://www.scopus.com/inward/record.uri?eid=2-s2.0-42049099695&doi=10.1016%2fj.icarus.2007.12.011&partnerID=40&md5=81058aa7ee84d20291c942e5010b3e83
AD - Royal Observatory of Belgium, B-1180 Brussels, Ringlaan 3, Belgium
AD - Département de Mathématique, Facultés Univ. N.D. de la Paix, B-5000 Namur, 8 Rempart de la Vierge, Belgium
AB - Europa, the smallest of the Galilean satellites, has a young icy surface and most likely contains an internal ocean. The primary objective of possible future missions to Europa is the unambiguous detection and characterization of a subsurface ocean. The thickness of the overlying icy shell provides important information on the thermal evolution of the satellite and on the interaction between the ocean and the surface, the latter being fundamental for astrobiology. However, the thickness is not well known, and estimates range from several hundred of meters to some ten of kilometers. Here, we investigate the use of libration (rotation variation) observations to study the interior structure of Europa

and in particular its icy shell. A dynamical libration model is developed, which includes gravitational coupling between the icy shell and the heavy solid interior. The amplitude of the main libration signal at 3.55 days (the orbital period) is shown to depend on Europa's shape and structure. Models of the interior structure of Europa are constructed and the equatorial flattening of the internal layers, which are key parameters for the libration, are calculated by assuming that Europa is in hydrostatic equilibrium. Europa's flattened shape is assumed to be due to rotation and permanent tides, and we extend the classical Radau equation for rotationally flattened bodies to include also tidal deformation. We show that the presence of an ocean increases the amplitude of libration by about 10%, depending mainly on the thickness of the icy shell. Therefore, libration observations offer possibility of detection of a subsurface ocean in Europa and estimation of the thickness of its overlying icy shell. © 2008 Elsevier Inc. All rights reserved.
KW - Europa
KW - Interiors
KW - Rotational dynamics
KW - solid body
KW - Spin-orbit resonances
KW - Tides
SN - 10902643 (ISSN)
LA - English
J2 - Icarus
M3 - Article
DB - Scopus
N1 - Export Date: 23 February 2023; Cited By: 62; Correspondence Address: T. Van Hoolst; Royal Observatory of Belgium, B-1180 Brussels, Ringlaan 3, Belgium; email: tim.vanhoolst@oma.be; CODEN: ICRSA
ER -

TY - JOUR
AU - Grindrod, P.M.
AU - Fortes, A.D.
AU - Nimmo, F.
AU - Feltham, D.L.
AU - Brodholt, J.P.
AU - Vočadlo, L.
TI - The long-term stability of a possible aqueous ammonium sulfate ocean inside Titan
PY - 2008
T2 - Icarus
VL - 197
IS - 1
SP - 137
EP - 151
DO - 10.1016/j.icarus.2008.04.006
UR - https://www.scopus.com/inward/record.uri?eid=2-s2.0-49549119968&doi=10.1016%2fj.icarus.2008.04.006&partnerID=40&md5=3e860ed44ce52a484e8d090c02807dd3
AD - Centre for Planetary Sciences, Department of Earth Sciences,

University College London, London, WC1E 6BT, Gower Street, United Kingdom
AD - Department of Earth and Planetary Sciences, University of California at Santa Cruz, Santa Cruz, CA 95064-1077, 1156 High Street, United States
AD - Centre for Polar Observation and Modelling, Department of Earth Sciences, University College London, London, WC1E 6BT, Gower Street, United Kingdom
AB - We model the thermal evolution of a subsurface ocean of aqueous ammonium sulfate inside Titan using a parameterized convection scheme. The cooling and crystallization of such an ocean depends on its heat flux balance, and is governed by the pressure-dependent melting temperatures at the top and bottom of the ocean. Using recent observations and previous experimental data, we present a nominal model which predicts the thickness of the ocean throughout the evolution of Titan; after 4.5 Ga we expect an aqueous ammonium sulfate ocean 56 km thick, overlain by a thick (176 km) heterogeneous crust of methane clathrate, ice I and ammonium sulfate. Underplating of the crust by ice I will give rise to compositional diapirs that are capable of rising through the crust and providing a mechanism for cryovolcanism at the surface. We have conducted a parameter space survey to account for possible variations in the nominal model, and find that for a wide range of plausible conditions, an ocean of aqueous ammonium sulfate can survive to the present day, which is consistent with the recent observations of Titan's spin state from Cassini radar data [Lorenz, R.D., Stiles, B.W., Kirk, R.L., Allison, M.D., del Marmo, P.P., Iess, L., Lunine, J.I., Ostro, S.J., Hensley, S., 2008. Science 319, 1649-1651]. © 2008 Elsevier Inc. All rights reserved.
KW - Astrobiology
KW - Ices
KW - Interiors
KW - Thermal histories
KW - Titan
SN - 10902643 (ISSN)
LA - English
J2 - Icarus
M3 - Article
DB - Scopus
N1 - Export Date: 23 February 2023; Cited By: 64; Correspondence Address: P.M. Grindrod; Centre for Planetary Sciences, Department of Earth Sciences, University College London, London, WC1E 6BT, Gower Street, United Kingdom; email: p.grindrod@ucl.ac.uk; CODEN: ICRSA
ER -

TY - CONF
AU - Pfiffner, S.M.
AU - DiFurio, S.
AU - Gan, Y.-D.
AU - Hoover, R.B.
TI - What do membrane lipids tell us about the microorganisms living in extreme environments?
PY - 2008
T2 - Proceedings of SPIE - The International Society for Optical

Engineering
VL - 7097
C7 - 70970M
DO - 10.1117/12.795378
UR - https://www.scopus.com/inward/record.uri?eid=2-s2.0-55549145433&doi=10.1117%2f12.795378&partnerID=40&md5=c849271f705902783efb7663c055bc8e
AD - Indiana Princeton Tennessee Astrobiology Initiative, Center for Environmental Biotechnology, University of Tennessee, Knoxville, TN 37932, 10515 Research Dr., Ste. 300, United States
AD - NASA National Space Science and Technology Center, Huntsville, AL 35805, 320 Sparkman Drive, United States
AB - To search for extraterrestrial life surrogate extreme environments on Earth have been chosen for investigation. An example of a surrogate site is the Canadian subpermafrost. Investigations into microbial communities occurred by access fracture borehole water in the Lupin gold mine, and drill rock cores and drilling waters in the High Lake region of Nunavut, Canada. Membrane lipid analyses uses GC/MS and HPLC/ES/MS/MS to provide estimates of biomass, phospholipid (PLFA) and respiratory quinone composition, and compositional changes related to membrane stress caused by nutritional limitations or exposure to toxic conditions. Lupin fracture borehole waters were collected from 800 to 1200 meters, while the High Lake rock cores were collected from 335 to 535 meters. Biomass estimates based on PLFA ranged from 0.25 to 22 pmol L -1 for the Lupin waters. High Lake drill waters had biomass that ranged from below detection limits (bdl) to 595 pmol/ml, while rock core samples had biomass estimates ranging from bdl to 32 pmol g-1. PLFA profiles revealed the presence of both Gram +/- bacteria and sulfatereducing bacteria. Specific PLFA ratios indicate that the bacterial communities were physiologically stressed. Menaquinones were the most abundant but varied in the dominant isoprene units between the two sites. Ubiquinone to menaquinone ratio indicated that these samples have been anoxic for a long time. Methods to detect life signatures at surrogate sites on Earth will be critical for assessing extraterrestrial life. Currently, the membrane lipid analyses provide additional information not easily provided by other molecular techniques.
KW - Astrobiology
KW - Bacteriology
KW - Biological materials
KW - Biomass
KW - Drills
KW - Lakes
KW - Metric system
KW - Phospholipids
KW - Renewable energy resources
KW - Rock drilling
KW - Bacterial communities
KW - Borehole waters
KW - Compositional changes
KW - Detection limits
KW - Extraterrestrial lives
KW - Extreme environments

KW - Long times
KW - Mars
KW - Membrane lipids
KW - Membrane stresses
KW - Menaquinones
KW - Microbial communities
KW - Microbial community composition
KW - Molecular techniques
KW - Quinones
KW - Respiratory quinones
KW - Rock cores
KW - Sulfatereducing bacteria
KW - Ubiquinone
KW - Lipids
KW - Mars
KW - Microbial community composition
KW - Permafrost
KW - Phospholipids
KW - Quinones
SN - 0277786X (ISSN); 978-081947317-2 (ISBN)
LA - English
J2 - Proc SPIE Int Soc Opt Eng
M3 - Conference paper
DB - Scopus
N1 - Export Date: 23 February 2023; Cited By: 0; Correspondence Address: S. M. Pfiffner; Indiana Princeton Tennessee Astrobiology Initiative, Center for Environmental Biotechnology, University of Tennessee, Knoxville, TN 37932, 10515 Research Dr., Ste. 300, United States; email: pfiffner@utk.edu; CODEN: PSISD
ER -

TY - CONF
AU - Crawford, I.A.
AU - Baldwin, E.C.
AU - Taylor, E.A.
AU - Bailey, J.A.
AU - Tsembelis, K.
TI - On the survivability and detectability of terrestrial meteorites on the moon
PY - 2008
T2 - Astrobiology
VL - 8
IS - 2
SP - 242
EP - 252
DO - 10.1089/ast.2007.0215
UR - https://www.scopus.com/inward/record.uri?eid=2-s2.0-42049098404&doi=10.1089%2fast.2007.0215&partnerID=40&md5=34b5d6d354335b77c119ab88ad1d35b6
AD - Birkbeck/UCL Research School of Earth Sciences, London, United Kingdom
AD - Department of Physics and Astronomy, Centre for Earth, Planetary, Space and Astronomical Research, Open University, Milton Keynes, United Kingdom

AD - Australian Centre for Astrobiology, Macquarie University, Australia
AD - Materials and Mechanics Branch, Atomic Energy of Canada Ltd., Chalk River Labs, Chalk River, ON, Canada
AD - School of Earth Sciences, Birkbeck College London, London WC1E 7HX, Malet Street, United Kingdom
AB - Materials blasted into space from the surface of early Earth may preserve a unique record of our planet's early surface environment. Armstrong et al. (2002) pointed out that such materials, in the form of terrestrial meteorites, may exist on the Moon and be of considerable astrobiological interest if biomarkers from early Earth are preserved within them. Here, we report results obtained via the AUTODYN hydrocode to calculate the peak pressures within terrestrial meteorites on the lunar surface to assess their likelihood of surviving the impact. Our results confirm the order-of-magnitude estimates of Armstrong et al. (2002) that substantial survivability is to be expected, especially in the case of relatively low velocity (ca. 2.5 km/s) or oblique (≤45°) impacts, or both. We outline possible mechanisms for locating such materials on the Moon and conclude that searching for them would be a scientifically valuable activity for future lunar exploration. © 2008 Mary Ann Liebert, Inc.
KW - Exobiology
KW - Extraterrestrial Environment
KW - Meteoroids
KW - Moon
KW - astronomy
KW - biology
KW - conference paper
KW - microclimate
KW - moon
KW - Biomarkers
KW - Impacts
KW - Meteorites
KW - Moon
SN - 15311074 (ISSN)
C2 - 18393690
LA - English
J2 - Astrobiology
M3 - Conference paper
DB - Scopus
N1 - Export Date: 23 February 2023; Cited By: 36; Correspondence Address: I. A. Crawford; School of Earth Sciences, Birkbeck College London, London WC1E 7HX, Malet Street, United Kingdom; email: i.crawford@ucl.ac.uk
ER -

TY - JOUR
AU - Parkinson, C.D.
AU - Liang, M.-C.
AU - Yung, Y.L.
AU - Kirschivnk, J.L.
TI - Habitability of enceladus: Planetary conditions for life
PY - 2008

T2 - Origins of Life and Evolution of Biospheres
VL - 38
IS - 4
SP - 355
EP - 369
DO - 10.1007/s11084-008-9135-4
UR - https://www.scopus.com/inward/record.uri?eid=2-s2.0-46749110690&doi=10.1007%2fs11084-008-9135-4&partnerID=40&md5=24edf695bc6a91f8b26e0b0b284fc628
AD - Department of Atmospheric, Oceanic, and Space Sciences, University of Michigan, Ann Arbor, MI 48109, 2455 Hayward Street, United States
AD - Division of Geological and Planetary Science, California Institute of Technology, Pasadena, CA, United States
AD - Research Center for Environmental Changes, Academia Sinica, Taipei, Taiwan
AD - Graduate Institute of Astronomy, National Central University, Jhongli, Taiwan
AD - Jet Propulsion Laboratory, California Institute of Technology, Pasadena, CA, United States
AB - The prolific activity and presence of a plume on Saturn's tiny moon Enceladus offers us a unique opportunity to sample the interior composition of an icy satellite, and to look for interesting chemistry and possible signs of life. Based on studies of the potential habitability of Jupiter's moon Europa, icy satellite oceans can be habitable if they are chemically mixed with the overlying ice shell on Myr time scales. We hypothesize that Enceladus' plume, tectonic processes, and possible liquid water ocean may create a complete and sustainable geochemical cycle that may allow it to support life. We discuss evidence for surface/ocean material exchange on Enceladus based on the amounts of silicate dust material present in the Enceladus' plume particles. Microphysical cloud modeling of Enceladus' plume shows that the particles originate from a region of Enceladus' near surface where the temperature exceeds 190 K. This could be consistent with a shear-heating origin of Enceladus' tiger stripes, which would indicate extremely high temperatures (~250-273 K) in the subsurface shear fault zone, leading to the generation of subsurface liquid water, chemical equilibration between surface and subsurface ices, and crustal recycling on a time scale of 1 to 5 Myr. Alternatively, if the tiger stripes form in a mid-ocean-ridge-type mechanism, a half-spreading rate of 1 m/year is consistent with the observed regional heat flux of 250 mW m-2 and recycling of south polar terrain crust on a 1 to 5 Myr time scale as well. © 2008 Springer Science+Business Media B.V.
KW - Atmosphere
KW - Chemistry
KW - Cosmic Dust
KW - Exobiology
KW - Extraterrestrial Environment
KW - Geology
KW - Ice
KW - Life
KW - Models, Chemical

KW - Models, Theoretical
KW - Oxidation-Reduction
KW - Saturn
KW - Temperature
KW - Time Factors
KW - Water
KW - ice
KW - water
KW - astronomy
KW - cloud microphysics
KW - geochemical cycle
KW - habitat
KW - heat flux
KW - Jupiter
KW - Moon
KW - Saturn
KW - article
KW - astronomy
KW - atmosphere
KW - biology
KW - chemical model
KW - chemistry
KW - geology
KW - life
KW - methodology
KW - microclimate
KW - oxidation reduction reaction
KW - temperature
KW - theoretical model
KW - time
KW - Astrobiology
KW - Geochemical cycle
KW - Habitability
KW - Icy moons
KW - Microphysical cloud physics
KW - Plume
KW - Tectonic processes
KW - Tiger stripes
SN - 01696149 (ISSN)
C2 - 18566911
LA - English
J2 - Orig. Life Evol. Biosph.
M3 - Article
DB - Scopus
N1 - Export Date: 23 February 2023; Cited By: 53; Correspondence Address: C. D. Parkinson; Department of Atmospheric, Oceanic, and Space Sciences, University of Michigan, Ann Arbor, MI 48109, 2455 Hayward Street, United States; email: cdp@gps.caltech.edu; CODEN: OLEBE
ER -

TY - CHAP
AU - Brack, A.
TI - Astrobiology: From the Origin of Life on Earth to Life in the

Universe
PY - 2008
T2 - Complete Course in Astrobiology
SP - 1
EP - 22
DO - 10.1002/9783527618996.ch1
UR - https://www.scopus.com/inward/record.uri?eid=2-s2.0-84948704322&doi=10.1002%2f9783527618996.ch1&partnerID=40&md5=c6b4175320aa727425642641064e13a8
AB - This chapter covers the different theories about the steps toward the origin and evolution of life on Earth, and the major requirements for these processes and for life at large are discussed. Conclusions are drawn on the likelihood of life originating and persisting on other places of our Solar System, such as the terrestrial planets and the moons of the giant planets, or beyond in the Universe. © 2007 WILEY-VCH Verlag GmbH & Co. KGaA. All rights reserved.
KW - Giant planets
KW - Origin of life
KW - Terrestrial planets
KW - Planets
PB - wiley
SN - 978-352761899-6 (ISBN); 978-352740660-9 (ISBN)
LA - English
J2 - Complete Course in Astrobiol.
M3 - Book chapter
DB - Scopus
N1 - Export Date: 23 February 2023; Cited By: 2
ER -

TY - CHAP
AU - Fasoulas, S.
AU - Schmiel, T.
TI - Astrodynamics and Technological Aspects of Astrobiology Missions in Our Solar System
PY - 2008
T2 - Complete Course in Astrobiology
SP - 179
EP - 201
DO - 10.1002/9783527618996.ch7
UR - https://www.scopus.com/inward/record.uri?eid=2-s2.0-84862022298&doi=10.1002%2f9783527618996.ch7&partnerID=40&md5=0a3b7875bfaa6bc6f88ecf33a9d28eac
AB - The chapter gives an introduction into some basic technological aspects related to space missions in general and to astrobiology missions to other planets, asteroids, comets, and/or moons in particular. We focus on explaining the payload mass limits and the specific constraints given by orbital mechanics. © 2007 WILEY-VCH Verlag GmbH & Co. KGaA. All rights reserved.
KW - Orbital mechanics
KW - Payload mass
KW - Space missions
KW - Technological aspects
KW - Orbits

PB - wiley
SN - 978-352761899-6 (ISBN); 978-352740660-9 (ISBN)
LA - English
J2 - Complete Course in Astrobiol.
M3 - Book chapter
DB - Scopus
N1 - Export Date: 23 February 2023; Cited By: 1
ER -

TY - JOUR
AU - Furfaro, R.
AU - Dohm, J.M.
AU - Fink, W.
AU - Kargel, J.
AU - Schulze-Makuch, D.
AU - Fairén, A.G.
AU - Palmero-Rodriguez, A.
AU - Baker, V.R.
AU - Ferré, P.T.
AU - Hare, T.M.
AU - Tarbell, M.A.
AU - Miyamoto, H.
AU - Komatsu, G.
TI - The search for life beyond Earth through fuzzy expert systems
PY - 2008
T2 - Planetary and Space Science
VL - 56
IS - 3-4
SP - 448
EP - 472
DO - 10.1016/j.pss.2007.09.006
UR - https://www.scopus.com/inward/record.uri?eid=2-s2.0-39449105721&doi=10.1016%2fj.pss.2007.09.006&partnerID=40&md5=474d9c00d3da62d51b726e97bb65bcae
AD - Aerospace and Mechanical Engineering Department, University of Arizona, Tucson, AZ 85721, United States
AD - Department of Hydrology and Water Resources, University of Arizona, Tucson, AZ 85721, United States
AD - Lunar and Planetary Laboratory, University of Arizona, Tucson, AZ 85721, United States
AD - California Institute of Technology, Visual and Autonomous Exploration Systems Research Laboratory, Division of Physics, Mathematics and Astronomy, Pasadena, CA 91125, United States
AD - Department of Geology, Washington State University, Pullman, WA, United States
AD - NASA Ames Research Center, Space Science and Astrobiology Division, Moffet Field, CA 94035, United States
AD - United States Geologic Survey, Flagstaff, AZ, United States
AD - Department of Earth and Planetary Science, University of Tokyo, Tokyo, 113-0033, Japan
AD - Department of Geosystem Engineering, University of Tokyo, Tokyo, Japan
AD - International Research School of Planetary Sciences, Università d'Annunzio, Pescara, Italy

AB - Autonomy will play a key role in future science-driven, tier-scalable robotic planetary reconnaissance to extremely challenging (by existing means), locales on Mars and elsewhere that have the potential to yield significant geological and possibly exobiologic information. The full-scale and optimal deployment of the agents employed by tier-scalable architectures requires the design, implementation, and integration of an intelligent reconnaissance system. Such a system should be designed to enable fully automated and comprehensive characterization of an operational area, as well as to integrate existing information with acquired, "in transit" spatial and temporal sensor data, to identify and home in on prime candidate locales. These may include locales with the greatest potential of containing life. Founded on the premise that water and energy are key to life, we have designed a fuzzy system that can (1) acquire the appropriate past/present water/energy indicators while the tier-scalable mission architecture is deployed (first layer), and (2) evaluate habitability through a specialized fuzzy knowledge-base of the water and energy information (second layer) acquired in (1). The system has been tested through hypothetical deployments at two hypothesized regions on Mars. The fuzzy-based expert's simulation results corroborate the same conclusions provided by the human expert, and thus highlight the system's potential capability to effectively and autonomously reason as an interdisciplinary scientist in the quest for life. While the approach is demonstrated for Mars, the methodology is general enough to be extended to other planetary bodies. It can be readily modified and updated as our interdisciplinary understanding of planetary environments improves. We believe this work represents a foundational step toward implementing higher-level intelligence in robotic, tier-scalable planetary reconnaissance within and beyond the solar system. © 2007 Elsevier Ltd. All rights reserved.
KW - Expert systems
KW - Information retrieval
KW - Robotics
KW - Space research
KW - Autonomous planetary reconnaissance
KW - Robotic planetary exploration
KW - Tier-scalable mission autonomy
KW - Fuzzy logic
KW - Astrobiology
KW - Autonomous planetary reconnaissance
KW - Fuzzy logic
KW - Robotic planetary exploration
KW - Search for life
KW - Tier-scalable mission autonomy
SN - 00320633 (ISSN)
LA - English
J2 - Planet. Space Sci.
M3 - Article
DB - Scopus
N1 - Export Date: 23 February 2023; Cited By: 22; Correspondence Address: R. Furfaro; Aerospace and Mechanical Engineering Department, University of Arizona, Tucson, AZ 85721, United States; email: robertof@email.arizona.edu; CODEN: PLSSA

ER -

TY - CONF
AU - Rice, C.V.
AU - Wickham, J.R.
AU - Eastman, M.A.
AU - Harrison, W.
AU - Pereira, M.P.
AU - Brown, E.D.
TI - Magnetic resonance tells microbiology where to go; bacterial teichoic acid protects liquid water at sub-zero temperatures
PY - 2008
T2 - Proceedings of SPIE - The International Society for Optical Engineering
VL - 7097
C7 - 70970O
DO - 10.1117/12.800899
UR - https://www.scopus.com/inward/record.uri?eid=2-s2.0-55549106102&doi=10.1117%2f12.800899&partnerID=40&md5=8af69c8ebc97db656d25989d483f2494
AD - Department of Chemistry and Biochemistry, University of Oklahoma, Norman, OK 73019, 620 Parrington Oval, United States
AD - Department of Chemistry, Oklahoma State University, Stillwater, OK 74078, United States
AD - Department of Biochemistry and Biomedical Sciences, McMaster University, Hamilton, ON L8N 3Z5, 1200 Main Street West, Canada
AB - Numerous chemical additives lower the freezing point of water, but life at sub-zero temperatures is sustained by a limited number of biological cryoprotectants. Antifreeze proteins in fish, plants, and insects provide protection to a few degrees below freezing. Microbes have been found to survive at even lower temperatures, although, with a few exceptions, antifreeze proteins are missing. Survival has been attributed to external factors, such as high salt concentration (brine veins) and adhesion to particulates or ice crystal defects. Teichoic acid is a phosphodiester polymer ubiquitous in Gram positive bacteria, composing 50% of the mass of the bacterial cell wall and excreted into the extracellular space of biofilm communities. We have found that when bound to the peptidoglycan cell wall (wall teichoic acid) or as a free molecule (lipoteichoic acid), teichoic acid is surrounded by liquid water at temperatures significantly below freezing. Using solid-state NMR, we are unable to collect 31P CPMAS spectra for frozen solutions of lipoteichoic acid at temperatures above -60°C. For wall teichoic acid in D2O, signals are not seen above -30°C. These results can be explained by the presence of liquid water, which permits rapid molecular motion to remove 1H/ 31P dipolar coupling. 2H quadrupole echo NMR spectroscopy reveals that both liquid and solid water are present. We suggest that teichoic acids could provide a shell of liquid water around biofilms and planktonic bacteria, removing the need for brine veins to prevent bacterial freezing.
KW - ABS resins
KW - Astrobiology
KW - Bacteria
KW - Bacteriology

KW - Biofilms
KW - Biofilters
KW - Cell membranes
KW - Concentration (process)
KW - Crystal defects
KW - Deuterium
KW - Freezing
KW - Nuclear magnetic resonance
KW - Nuclear magnetic resonance spectroscopy
KW - Proteins
KW - Resonance
KW - Cryoprotectant
KW - Extremophile
KW - Lipoteichoic acid
KW - NMR spectroscopy
KW - Peptidoglycan
KW - Psychrophile
KW - Teichoic acid
KW - Wall teichoic acid
KW - Acids
KW - Bacteria
KW - Cryoprotectant
KW - Deuterium quadrupole echo
KW - Extremophile
KW - Ice
KW - Lipoteichoic acid
KW - NMR spectroscopy
KW - Peptidoglycan
KW - Psychrophile
KW - Teichoic acid
KW - Wall teichoic acid
SN - 0277786X (ISSN); 978-081947317-2 (ISBN)
LA - English
J2 - Proc SPIE Int Soc Opt Eng
M3 - Conference paper
DB - Scopus
N1 - Export Date: 23 February 2023; Cited By: 2; CODEN: PSISD
ER -

TY - JOUR
AU - Morris-Smith, P.A.
AU - Grasby, S.
TI - Astrobiology special collection: Extreme Environment Analogue Studies Part II. Introduction.
PY - 2008
T2 - Astrobiology
VL - 8
IS - 4
SP - 805
DO - 10.1089/ast.2008.0731
UR - https://www.scopus.com/inward/record.uri?eid=2-s2.0-58149168600&doi=10.1089%2fast.2008.0731&partnerID=40&md5=a749909cc6cd44d34cd7548ccbacdcd8
KW - Astronomy

KW - Australia
KW - Biogenesis
KW - Chemistry, Organic
KW - Earth (Planet)
KW - Environment
KW - Exobiology
KW - Extraterrestrial Environment
KW - Microscopy, Fluorescence
KW - Solar System
KW - Sulfates
KW - sulfate
KW - MLCS
KW - MLOWN
KW - astronomy
KW - Australia
KW - biogenesis
KW - biology
KW - editorial
KW - environment
KW - fluorescence microscopy
KW - methodology
KW - microclimate
KW - organic chemistry
SN - 15578070 (ISSN)
C2 - 18844458
LA - English
J2 - Astrobiology
M3 - Editorial
DB - Scopus
N1 - Export Date: 23 February 2023; Cited By: 0
ER -

TY - JOUR
AU - Jackson, B.
AU - Barnes, R.
AU - Greenberg, R.
TI - Tidal heating of terrestrial extrasolar planets and implications for their habitability
PY - 2008
T2 - Monthly Notices of the Royal Astronomical Society
VL - 391
IS - 1
SP - 237
EP - 245
DO - 10.1111/j.1365-2966.2008.13868.x
UR - https://www.scopus.com/inward/record.uri?eid=2-s2.0-55849101504&doi=10.1111%2fj.1365-2966.2008.13868.x&partnerID=40&md5=1b60e24e5a82caf7e1869e6e7e31224d
AD - University of Arizona, Lunar and Planetary Laboratory, Tucson, AZ 85721-0092, 1629 E. University Blvd., United States
AB - The tidal heating of hypothetical rocky (or terrestrial) extrasolar planets spans a wide range of values depending on stellar masses and initial orbits. Tidal heating may be sufficiently large (in many cases, in excess of radiogenic heating) and long-lived to

drive plate tectonics, similar to the Earth's, which may enhance the planet's habitability. In other cases, excessive tidal heating may result in Io-like planets with violent volcanism, probably rendering them unsuitable for life. On water-rich planets, tidal heating may generate subsurface oceans analogous to Europa's with similar prospects for habitability. Tidal heating may enhance the outgassing of volatiles, contributing to the formation and replenishment of a planet's atmosphere. To address these issues, we model the tidal heating and evolution of hypothetical extrasolar terrestrial planets. The results presented here constrain the orbital and physical properties required for planets to be habitable. © 2008 RAS.
KW - Earth (planet)
KW - Extrasolar planets
KW - Gravitation
KW - Heating
KW - Orbits
KW - Tectonics
KW - Celestial mechanics
KW - Extrasolar
KW - Planetary system
KW - Planets' atmospheres
KW - Radiogenics
KW - Stellar mass
KW - Sub-surface ocean
KW - Terrestrial planets
KW - Tidal evolution
KW - Tidal heating
KW - Astrophysics
KW - Astrobiology
KW - Celestial mechanics
KW - Planetary systems
PB - Blackwell Publishing Ltd
SN - 00358711 (ISSN)
LA - English
J2 - Mon. Not. R. Astron. Soc.
M3 - Article
DB - Scopus
N1 - Export Date: 23 February 2023; Cited By: 74; Correspondence Address: B. Jackson; University of Arizona, Lunar and Planetary Laboratory, Tucson, AZ 85721-0092, 1629 E. University Blvd., United States; email: bjackson@lpl.arizona.edu; CODEN: MNRAA
ER -

TY - JOUR
AU - Thompson, J.M.T.
TI - Introduction. Progress in astronomy: From gravitational waves to space weather
PY - 2008
T2 - Philosophical Transactions of the Royal Society A: Mathematical, Physical and Engineering Sciences
VL - 366
IS - 1884
SP - 4359

EP - 4364
DO - 10.1098/rsta.2008.0202
UR - https://www.scopus.com/inward/record.uri?eid=2-s2.0-55849152411&doi=10.1098%2frsta.2008.0202&partnerID=40&md5=a50b4d0b920260597dad1bccb2a43895
AD - Department of Applied Mathematics and Theoretical Physics, Centre for Mathematical Sciences, University of Cambridge, Cambridge CB3 0WA, Wilberforce Road, United Kingdom
AB - This brief paper introduces and reviews the 'visions of the future' articles prepared by leading young scientists throughout the world for the first of two Christmas 2008 Triennial issues of Phil. Trans. R. Soc. A, devoted, respectively, to astronomy and Earth science. Contributions in astronomy include the very topical gamma-ray bursts, new ideas on stellar collapse and the unusual atmospheres of synchronized planets orbiting nearby stars. © 2008 The Royal Society.
KW - Astronomy
KW - Atmosphere
KW - Earth (Planet)
KW - Exobiology
KW - Extraterrestrial Environment
KW - Humans
KW - Planets
KW - Weather
KW - Astronomy
KW - Astrophysics
KW - Earth sciences
KW - Gamma rays
KW - Gravitational effects
KW - Gravity waves
KW - Planets
KW - Black holes
KW - Extrasolar planets
KW - Gamma-ray bursts
KW - Gravitational waves
KW - Stellar collapse
KW - Supernovae
KW - astrobiology
KW - astronomy
KW - atmosphere
KW - human
KW - microclimate
KW - weather
KW - Stars
KW - Black holes
KW - Extrasolar planets
KW - Gamma-ray bursts
KW - Gravitational waves
KW - Stellar collapse
KW - Supernovae
PB - Royal Society
SN - 1364503X (ISSN)
C2 - 18812296
LA - English

J2 - Philos. Trans. R. Soc. A Math. Phys. Eng. Sci.
M3 - Article
DB - Scopus
N1 - Export Date: 23 February 2023; Cited By: 0; Correspondence Address: J. M. T. Thompson; Department of Applied Mathematics and Theoretical Physics, Centre for Mathematical Sciences, University of Cambridge, Cambridge CB3 0WA, Wilberforce Road, United Kingdom; email: jmtt@ucl.ac.uk
ER -

TY - JOUR
AU - Schulze-Makuch, D.
AU - Irwin, L.N.
TI - Signatures of life
PY - 2008
T2 - Advances in Astrobiology and Biogeophysics
SP - 165
EP - 182
DO - 10.1007/978-3-540-76817-3_11
UR - https://www.scopus.com/inward/record.uri?eid=2-s2.0-52149114260&doi=10.1007%2f978-3-540-76817-3_11&partnerID=40&md5=3129cda6c78a7f2a2704124667ee57b0
AD - School of Earth and Environmental Sciences, Washington State University, Pullman, WA 99164-2812, United States
AD - Dept. Biological Sciences, University of Texas at El Paso, El Paso, TX 79968-0519, 500 West University Avenue, United States
AB - In this chapter we will elaborate on how evidence for life on other worlds can be sought, and if present, possibly detected. The best evidence for extraterrestrial life, of course, would be recovery of actual specimens or their fossils. For the next one or two decades, the possibility of obtaining such direct evidence is almost surely restricted to samples from Mars and Venus. So detection of life beyond our nearest neighbors will be dependent for the near future on remote sensing. As technology of robotic exploration and remote sensing improves, the possibility of detecting extraterrestrial life will grow. While the size of individual organisms makes their detection at a distance virtually impossible, organisms in the aggregate alter their environments, generating signatures of their functional processes. These direct consequences of biological activity are referred to as "biosignatures". Other effects of the presence of living systems may be detected in global or geological features. These alterations of the geological environment due to life processes, we call "geosignatures". Even on worlds too remote, small, or difficult for whatever reason to monitor for the existence of explicit signatures of life, certain planetary characteristics can be detected that are more likely to be consistent with the presence of life than others. These we refer to as "geoindicators." They consist of parameters that are consistent with life as defined in Chap. 2, and the requirements for life as described in subsequent chapters, including a flow or gradient of energy, presence of an appropriate solvent, and availability of complex polymeric chemistry. While geoindicators point to the potential for supporting life, they do not confirm its existence. Most geoindicators can be detected by remote sensing

methods with relative ease, however, and thus can be used in assessing the plausibility of the existence of life. At the end of the chapter, we apply our discussion of signatures and indicators of life to assess the relative plausibility for the existence of life on other bodies in our Solar System, and discuss recent results on extrasolar planetary detection and their implications for astrobiology. © Springer-Verlag Berlin Heidelberg 2008.
A2 - Schulze-Makuch D.S.-M.
A2 - Washington State University, School of Earth and Environmental Sc, Pullman, WA 99164-2812
A2 - Irwin L.N.I.
A2 - University of Texas at El Paso, Dept. Biological Sciences, El Paso, TX 79968-0519, 500 West University Avenue
SN - 16131851 (ISSN); 978-354076816-6 (ISBN)
LA - English
J2 - Adv. Astrobiol. Biogeophys.
M3 - Article
DB - Scopus
N1 - Export Date: 23 February 2023; Cited By: 2; Correspondence Address: D. Schulze-Makuch; School of Earth and Environmental Sciences, Washington State University, Pullman, WA 99164-2812, United States; email: dirksm@wsu.edu
ER -

TY - JOUR
AU - Jones, B.W.
TI - Exoplanets - Search methods, discoveries, and prospects for astrobiology
PY - 2008
T2 - International Journal of Astrobiology
VL - 7
IS - 3-4
SP - 279
EP - 292
DO - 10.1017/S147355040800428X
UR - https://www.scopus.com/inward/record.uri?eid=2-s2.0-57649129161&doi=10.1017%2fS147355040800428X&partnerID=40&md5=7ea68cafd400205f2670f69c05ec4cea
AD - Astronomy Group, Physics and Astronomy, The Open University, Milton Keynes MK7 6AA, United Kingdom
AB - Whereas the Solar System has Mars and Europa as the best candidates for finding fossil/extant life as we know it - based on complex carbon compounds and liquid water - the 263 (non-pulsar) planetary systems around other stars as known at 15 September 2008 could between them possess many more planets where life might exist. Moreover, the number of these exoplanetary systems is growing steadily, and with this growth there is an increase in the number of planets that could bear carbon-liquid water life. In this brief review the main methods by which exoplanets are being discovered are outlined, and then the discoveries that have so far been made are presented. This is followed by an account of likely future discoveries. Habitability is then discussed, and an outline presented of how a planet could be studied from afar to determine whether it is habitable, and whether it is indeed inhabited. This

review is aimed at the astrobiology community, which spans many disciplines, few of which involve exoplanets. It is therefore at a basic level and concentrates on the major topics. © 2008 Cambridge University Press.
KW - astrophysics
KW - Europa
KW - future prospect
KW - Mars
KW - solar system
KW - Exoplanets - discoveries
KW - Exoplanets - discovery methods
KW - Exoplanets - investigations for habitability/inhabitation
KW - Exoplanets - the future
SN - 14753006 (ISSN)
LA - English
J2 - Int. J. Astrobiology
M3 - Review
DB - Scopus
N1 - Export Date: 23 February 2023; Cited By: 9; Correspondence Address: B.W. Jones; Astronomy Group, Physics and Astronomy, The Open University, Milton Keynes MK7 6AA, United Kingdom; email: b.w.jones@open.ac.uk
ER -

TY - JOUR
AU - Kyle, J.K.
AU - Pedersen, K.
AU - Ferris, G.F.
TI - Virus mineralization at low pH in the Rio Tinto, Spain
PY - 2008
T2 - Geomicrobiology Journal
VL - 25
IS - 7-8
SP - 338
EP - 345
DO - 10.1080/01490450802402703
UR - https://www.scopus.com/inward/record.uri?eid=2-s2.0-54049123289&doi=10.1080%2f01490450802402703&partnerID=40&md5=d65ef28171b67a56c40a951a35fc02f3
AD - Department of Geology, University of Toronto, Earth Sciences Centre, Toronto, ON, Canada
AD - Department of Cell and Molecular Biology, Microbiology Section, Goteborg University, Goteborg, Sweden
AB - Water and sediment samples were collected from the Rio Tinto in southwestern Spain to assess (1) the presence and diversity of viruses in an acid mine drainage system and (2) determine if relationships occur between geochemical parameters and viral abundance. Epifluroescence microscopy and transmission electron microscopy revealed that viruses are not only present, but geochemical evidence and multivariate statistical analyses suggest that viruses in the Rio Tinto participate in mineralization processes. Viral capsids and tails occurred with iron-bearing minerals sorbed to their surfaces, at times with mineralization so extensive that differentiating between viral and inorganic particles

using microscopy was difficult. Moreover, a strong inverse relationship between viral abundance and jarosite saturation state (Pearson correlation coefficient r = −0.71) was observed implying that viruses were removed from suspension owing to ongoing mineral precipitation (i.e., decreasing number of viruses with increasing rates of mineral precipitation, as inferred from saturation state). Viral-mineral interactions may additionally impact virus-host relationships as a weak correlation was found between viral and prokaryotic abundance, a relationship that is usually found to be highly correlated. Viral abundance and pH were strongly correlated (Pearson correlation coefficient r = 0.94) indicating viral sensitivity to low pH conditions.
KW - Andalucia
KW - Eurasia
KW - Europe
KW - Huelva [Andalucia]
KW - Rio Tinto [(RVR) Huelva]
KW - Southern Europe
KW - Spain
KW - Miridae
KW - Prokaryota
KW - abundance
KW - acid mine drainage
KW - bacteriophage
KW - fluorescence spectroscopy
KW - geochemistry
KW - jarosite
KW - mineralization
KW - multivariate analysis
KW - pH
KW - transmission electron microscopy
KW - virus
KW - Acid mine drainage
KW - Astrobiology
KW - Bacteriophage
KW - Jarosite
SN - 15210529 (ISSN)
LA - English
J2 - Geomicrobiol. J.
M3 - Article
DB - Scopus
N1 - Export Date: 23 February 2023; Cited By: 23
ER -

TY - CONF
AU - Kobayashi, K.
AU - Kaneko, T.
AU - Takano, Y.
AU - Takahashi, J.-I.
TI - Formation of amino acid precursors with large molecular weight in dense clouds and their relevance to origins of bio-homochirality
PY - 2008
T2 - Proceedings of the International Astronomical Union
VL - 4

IS - S251
SP - 465
EP - 472
DO - 10.1017/S1743921308022199
UR - https://www.scopus.com/inward/record.uri?eid=2-s2.0-53349171133&doi=10.1017%2fS1743921308022199&partnerID=40&md5=55f0af9e6687c943580cfdf65095cfb8
AD - Graduate School of Engineering, Yokohama National University, Hodogaya-ku, Yokohama 240-8501, 79-5 Tokiwadai, Japan
AD - IFREE, Japan Agency for Marine-Earth Science and Technology, Yokosuka 237-0061, 2-15 Natsushimacho, Japan
AD - NTT Microsystem Integration Laboratories, Atsugi 243-0198, 3-1 Morinosato-Wakamiya, Japan
AB - A wide variety of organic compounds have been found in carbonaceous chondrites and comets, which suggests that extraterrestrial organic compounds could have been an important source of the first terrestrial biosphere. In the Greenberg model, these organic compounds in the small bodies were originally formed in interstellar dusts (ISD) in dense clouds by the action of cosmic rays and ultraviolet light. We irradiated a frozen mixture of methanol, ammonia and water with high-energy heavy ions from an accelerator ("HIMAC" in NIRS, Japan) to simulate the action of cosmic rays in dense clouds. Racemic mixtures of amino acids were detected after hydrolysis of the irradiation products. A mixture of carbon monoxide, ammonia and water also gave such complex amino acid precursors with large molecular weights. When such amino acid precursors were irradiated with circular polarized UV light from a synchrotron, enantiomeric excesses were detected. The yield of amino acids was not largely changed between, before, and after CPL-irradiation. The present results suggest that the seed of homochirality of terrestrial amino acids were originally formed in interstellar space. © 2008 International Astronomical Union.
KW - Astrobiology
KW - Cosmic rays
KW - ISM: dust
KW - ISM: molecules
KW - Ultraviolet: stars
SN - 17439221 (ISSN); 978-012345678-6 (ISBN)
LA - English
J2 - Proc. Int. Astron. Union
M3 - Conference paper
DB - Scopus
N1 - Export Date: 23 February 2023; Cited By: 1; Correspondence Address: K. Kobayashi; Graduate School of Engineering, Yokohama National University, Hodogaya-ku, Yokohama 240-8501, 79-5 Tokiwadai, Japan; email: kkensei@ynu.ac.jp
ER -

TY - JOUR
AU - Lal, A.K.
TI - Origin of life
PY - 2008
T2 - Astrophysics and Space Science
VL - 317

IS - 3-4
SP - 267
EP - 278
DO - 10.1007/s10509-008-9876-6
UR - https://www.scopus.com/inward/record.uri?eid=2-s2.0-55849144367&doi=10.1007%2fs10509-008-9876-6&partnerID=40&md5=b875eb4924eddeed9bdcf53d4c19e760
AD - Ministry of Statistics and Programme Implementation, New Delhi 110001, Sardar Patel Bhawan, Parliament Street, India
AB - The evolution of life has been a big enigma despite rapid advancements in the field of astrobiology, microbiology and genetics in recent years. The answer to this puzzle is as mindboggling as the riddle relating to evolution of the universe itself. Despite the fact that panspermia has gained considerable support as a viable explanation for origin of life on the earth and elsewhere in the universe, the issue, however, remains far from a tangible solution. This paper examines the various prevailing hypotheses regarding origin of life-like abiogenesis, RNA world, iron-sulphur world and panspermia, and concludes that delivery of life-bearing organic molecules by the comets in the early epoch of the earth alone possibly was not responsible for kick-starting the process of evolution of life on our planet. © 2008 Springer Science+Business Media B.V.
KW - Abiogenesis
KW - Comets
KW - Cyanobacteria
KW - DNA
KW - Extremophiles
KW - Iron-sulphur world
KW - LUCA
KW - Microbes
KW - Miller-Urey experiment
KW - Panspermia
KW - RNA
KW - RNA world
KW - Thermophiles
SN - 1572946X (ISSN)
LA - English
J2 - Astrophys. Space Sci.
M3 - Review
DB - Scopus
N1 - Export Date: 23 February 2023; Cited By: 17; Correspondence Address: A. K. Lal; Ministry of Statistics and Programme Implementation, New Delhi 110001, Sardar Patel Bhawan, Parliament Street, India; email: akl1951@yahoo.co.in
ER -

TY - JOUR
AU - Filipović, M.
AU - Ognjanović, S.
AU - Ognjanović, M.
TI - Evidence of molecular adaptation to extreme environments and applicability to space environments
PY - 2008

T2 - Serbian Astronomical Journal
IS - 176
SP - 81
EP - 86
DO - 10.2298/SAJ0876081F
UR - https://www.scopus.com/inward/record.uri?eid=2-s2.0-50049122407&doi=10.2298%2fSAJ0876081F&partnerID=40&md5=55c872497e7436b5098394a0ec112ff6
AD - University of Western Sydney, Penrith South DC, NSW 1797, Locked Bag 1797, Australia
AD - University of Minnesota, Minneapolis, MN 55455, 1-185 Moos 420 Delaware Street SE, United States
AB - This is initial investigation of gene signatures responsible for adapting microscopic life to the extreme Earth environments. We present preliminary results on identification of the clusters of orthologous groups (COGs) common to several hyperthermophiles and exclusion of those common to a mesophile (non-hyperthermophile): Escherichia coli (E. coli K12), will yield a group of proteins possibly involved in adaptation to life under extreme temperatures. Comparative genome analyses represent a powerful tool in discovery of novel genes responsible for adaptation to specific extreme environments. Methanogens stand out as the only group of organisms that have species capable of growth at 0°C (Metarhizium frigidum (M. frigidum) and Methanococcoides burtonii (M. burtonii)) and 110°C (Methanopyrus kandleri (M. kandleri)). Although not all the components of heat adaptation can be attributed to novel genes, the chaperones known as heat shock proteins stabilize the enzymes under elevated temperature. However, highly conserved chaperons found in bacteria and eukaryots are not present in hyperthermophilic Archea, rather, they have a unique chaperone TF55. Our aim was to use software which we specifically developed for extremophile genome comparative analyses in order to search for additional novel genes involved in hyperthermophile adaptation. The following hyperthermophile genomes incorporated in this software were used for these studies: Methanocaldococcus jannaschii (M. jannaschii), M. kandleri, Archaeoglobus fulgidus (A. fulgidus) and three species of Pyrococcus. Common genes were annotated and grouped according to their roles in cellular processes where such information was available and proteins not previously implicated in the heat-adaptation of hyperthermophiles were identified. Additional experimental data are needed in order to learn more about these proteins. To address non-gene based components of thermal adaptation, all sequenced extremophiles were analysed for their GC contents and aminoacid hydrophobicity. Finally, we develop a prediction model for optimal growth temperature.
KW - Astrobiology
SN - 1450698X (ISSN)
LA - English
J2 - Serb. Astron. J.
M3 - Article
DB - Scopus
N1 - Export Date: 23 February 2023; Cited By: 0; Correspondence Address: M. Filipović; University of Western Sydney, Penrith South DC, NSW 1797, Locked Bag 1797, Australia; email:

m.filipovic@uws.edu.au
ER -

TY - CONF
AU - Matthews, C.N.
AU - Minard, R.D.
TI - Hydrogen cyanide polymers connect cosmochemistry and biochemistry
PY - 2008
T2 - Proceedings of the International Astronomical Union
VL - 4
IS - S251
SP - 453
EP - 457
DO - 10.1017/S1743921308022175
UR - https://www.scopus.com/inward/record.uri?eid=2-s2.0-53349122089&doi=10.1017%2fS1743921308022175&partnerID=40&md5=83bf265c9c8b9942bb590728a35b8f3a
AD - University of Illinois, Chicago, IL, United States
AD - Penn State Astrobiology Research Center, Department of Chemistry, Penn State University, University Park, PA 16802, United States
AB - To understand the origin of protein/nucleic acid based life as we know it on Earth, we must "follow" the nitrogen. Because of its unique hydrogen bonding characteristics, nitrogen is the key element in catalytic and/or informational proteins and nucleic acids essential to cell function and reproduction. We present evidence that HCN is the original source of prebiotic protein and nucleobase nitrogen. We also present chemically rational models supporting the radical hypothesis that the polymerization of HCN yields ab initio mundi prebiotic protein and polynucleobase macromolecules of sufficient size and complexity to allow the spontaneous generation of pre-RNA World biopolymers capable of catalysis and information transfer. © 2008 International Astronomical Union.
KW - Astrobiology
KW - Astrochemistry
KW - Comets: general
KW - Infrared: ISM
KW - ISM: molecules
KW - Meteoroids
KW - Meteors
KW - Molecular processes
KW - Planets and satellites: individual (Earth, Titan)
SN - 17439221 (ISSN); 978-012345678-6 (ISBN)
LA - English
J2 - Proc. Int. Astron. Union
M3 - Conference paper
DB - Scopus
N1 - Export Date: 23 February 2023; Cited By: 20; Correspondence Address: C. N. Matthews; University of Illinois, Chicago, IL, United States; email: cnmatthews@cs.com
ER -

TY - CHAP

AU - Horneck, G.
TI - Astrobiology
PY - 2008
T2 - Bioengineering in Cell and Tissue Research
SP - 641
EP - 666
DO - 10.1007/978-3-540-75409-1_27
UR - https://www.scopus.com/inward/record.uri?eid=2-s2.0-84885416004&doi=10.1007%2f978-3-540-75409-1_27&partnerID=40&md5=76c64c67a2c37ec83ef0c35e11039138
AD - German Aerospace Center, Institute of Aerospace Medicine, 51170 Koeln, Germany
AB - Astrobiology addresses questions that have intrigued humans for a long time: How did life originate?, Are we alone in the Universe?, and What is the future of life on Earth and in the Universe? It is a multidisciplinary approach to understand the processes that lead to life's appearance, evolution and distribution as a planetary phenomenon, embedded in the evolution of the Universe. The ultimate goal is to reach a more universal definition of life. Knowledge of the history of life on Earth and of strategies of life to adapt to extreme environments is used as base for assessing the occurrence of potential habitats beyond the Earth, e. g. within our Solar System on Mars or Jupiter's moon Europa. Planetary protection guidelines have been developed to avoid unintentional introduction of terrestrial life forms to those planets or moons of interest, which may jeopardize future search for life studies. Measures include bioload monitoring and/or reduction, depending on target body and type of mission. Finally, radioastronomy has just started to detect extrasolar planetary systems. More sensitive space telescopes are being developed with the aim to detect and characterize Earth-like planets including possible biosignatures on them. © 2008 Springer-Verlag.
PB - Springer Berlin Heidelberg
SN - 978-354075408-4 (ISBN)
LA - English
J2 - Bioeng. in Cell and Tissue Res.
M3 - Book chapter
DB - Scopus
N1 - Export Date: 23 February 2023; Cited By: 0; Correspondence Address: G. Horneck; German Aerospace Center, Institute of Aerospace Medicine, 51170 Koeln, Germany; email: gerda.horneck@dlr.de
ER -

TY - CONF
AU - Sandford, S.A.
TI - Organics in the samples returned from comet 81P/Wild 2 by the stardust spacecraft
PY - 2008
T2 - Proceedings of the International Astronomical Union
VL - 4
IS - S251
SP - 299
EP - 307
DO - 10.1017/S1743921308021777

UR - https://www.scopus.com/inward/record.uri?eid=2-s2.0-53349098939&doi=10.1017%2fS1743921308021777&partnerID=40&md5=d7b98d7977bdfae9e28df080bb203435
AD - Astrophysics Branch, NASA-Ames Research Center, Mail Stop 245-6, Moffett Field, CA, United States
AB - The Stardust Mission collected samples from Comet 81P/Wild 2 on 2 Jan 2004 and returned these samples to Earth on 15 Jan 2006. After recovery, a six month preliminary examination was done on a portion of the samples. Studies of the organics in the samples were made by the Organics Preliminary Examination Team (PET) - a worldwide group of over 55 scientists. This paper provides a brief overview of the findings of the Organics PET. Organics in the samples were studied using a multitude of analytical techniques including spatial determination of C and heteroatom elemental abundances (STXM), functional group identification (micro-FTIR/Raman, C,N,O-XANES), and specific molecular identification of certain classes of organics (HPLC-LIF, L2MS, TOF-SIMS). Analyses were also made of spacecraft components and environmental samples collected near the recovered returned capsule to assess contamination issues. The distribution of organics (abundance, functionality, and relative elemental abundances of C,N,O) is heterogeneous both within and between particles. They are an unequilibrated reservoir that experienced little parent body processing after incorporation into the comet. Some organics look like those seen in IDPs (and to a lesser extent, meteorites), while new aromatic-poor and highly labile organics, not seen in meteoritic materials, are also present. The organics are O,N-rich compared to meteoritic organics. Some of the organics have an interstellar heritage, as evidenced by D and 15N enrichments. © 2008 International Astronomical Union.
KW - Astrobiology
KW - Astrochemistry
KW - Comets
KW - Dust
KW - Isotopes
KW - Organics
KW - Stardust mission
SN - 17439221 (ISSN); 978-012345678-6 (ISBN)
LA - English
J2 - Proc. Int. Astron. Union
M3 - Conference paper
DB - Scopus
N1 - Export Date: 23 February 2023; Cited By: 5; Correspondence Address: S. A. Sandford; Astrophysics Branch, NASA-Ames Research Center, Mail Stop 245-6, Moffett Field, CA, United States; email: Scott.A.Sandford@nasa.gov
ER -

TY - CONF
AU - Amashukeli, X.
AU - Grunthaner, F.J.
AU - Patrick, S.B.
AU - Yung, P.T.
TI - Subcritical water extractor for mars analog soil analysis

PY - 2008
T2 - Astrobiology
VL - 8
IS - 3
SP - 597
EP - 604
DO - 10.1089/ast.2007.0154
UR - https://www.scopus.com/inward/record.uri?eid=2-s2.0-48849089454&doi=10.1089%2fast.2007.0154&partnerID=40&md5=89a49a9860e548688328c631ec0d59d7
AD - Jet Propulsion Laboratory, California Institute of Technology, Pasadena, CA, United States
AD - MS 302-231, Pasadena, CA 91001, 4800 Oak Grove Drive, United States
AB - Technologies that enable rapid and efficient extraction of biomarker compounds from various solid matrices are a critical requirement for the successful implementation of in situ chemical analysis of the martian regolith. Here, we describe a portable subcritical water extractor that mimics multiple organic solvent polarities by tuning the dielectric constant of liquid water through adjustment of temperature and pressure. Soil samples, collected from the Yungay region of the Atacama Desert (martian regolith analogue) in the summer of 2005, were used to test the instrument's performance. The total organic carbon was extracted from the samples at concentrations of 0.2-55.4 parts per million. The extraction data were compared to the total organic carbon content in the bulk soil, which was determined via a standard analytical procedure. The instrument's performance was examined over the temperature range of 25-250°C at a fixed pressure of 20.7 MPa. Under these conditions, water remains in a subcritical fluid state with a dielectric constant varying between ~80 (at 25°C) and ~30 (at 250°C). © 2008 Mary Ann Liebert, Inc.
KW - Desert Climate
KW - Extraterrestrial Environment
KW - Mars
KW - Pressure
KW - Soil
KW - Space Flight
KW - Temperature
KW - Water
KW - water
KW - astronomy
KW - chemistry
KW - conference paper
KW - desert climate
KW - instrumentation
KW - microclimate
KW - pressure
KW - soil
KW - space flight
KW - temperature
KW - Astrobiology and extraterrestrial materials
KW - Atacama Desert
KW - In situ instrument

KW - Instruments and techniques
KW - Mars
KW - Planetary science
KW - Subcritical water extraction
SN - 15311074 (ISSN)
C2 - 18680410
LA - English
J2 - Astrobiology
M3 - Conference paper
DB - Scopus
N1 - Export Date: 23 February 2023; Cited By: 22; Correspondence Address: X. Amashukeli; MS 302-231, Pasadena, CA 91001, 4800 Oak Grove Drive, United States; email: Xenia.Amashukeli@jpl.nasa.gov
ER -

TY - CONF
AU - Serozhkin, Y.
TI - Possibilities of lightning-induced processes in gas-dusty atmosphere of water-containing bodies of solar system
PY - 2008
T2 - Proceedings of the International Astronomical Union
VL - 4
IS - S251
SP - 331
EP - 332
DO - 10.1017/S174392130802187X
UR - https://www.scopus.com/inward/record.uri?eid=2-s2.0-53349120025&doi=10.1017%2fS174392130802187X&partnerID=40&md5=de0b204eb756ab2505cc82d732d46123
AD - Institute of Semiconductor Physics, 03028, Kyiv, 41, Prospekt Nauky, Ukraine
AB - Lightning is considered as one of energy sources for synthesis of biochemical compounds. Numerous theoretical and experimental researches of gas-grain chemistry show that chemical reactions on the gas - ice boundary play a considerable role in such synthesis. In this connection the greatest interest represents studying lightning in gas-dusty atmospheres of water-containing bodies (comets, ice satellites of Jupiter and Saturn). © 2008 International Astronomical Union.
KW - Astrobiology
KW - Comets: general
KW - Planets and satellites: individual (ice satellites)
KW - Plasmas
SN - 17439221 (ISSN); 978-012345678-6 (ISBN)
LA - English
J2 - Proc. Int. Astron. Union
M3 - Conference paper
DB - Scopus
N1 - Export Date: 23 February 2023; Cited By: 0; Correspondence Address: Y. Serozhkin; Institute of Semiconductor Physics, 03028, Kyiv, 41, Prospekt Nauky, Ukraine; email: yuriy.serozhkin@zeos.net
ER -

TY - CONF

AU - Lange, C.
AU - Richter, L.
TI - Analysis and concepts development for an astrobiology-focused exploration of the Jovian Moon Europa
PY - 2009
T2 - 60th International Astronautical Congress 2009, IAC 2009
VL - 2
SP - 1427
EP - 1435
UR - https://www.scopus.com/inward/record.uri?eid=2-s2.0-77953590126&partnerID=40&md5=3ebe6900f667e9106caa6f9f9e1cb4d1
AD - German Aerospace Center (DLR), Institute of Space Systems, Germany
AB - The Galilean Moon Europa is known to have a high astrobiological potential, which might even be comparable to the one of Mars, ever since it has been proven that a liquid water ocean exists on this body under an icy layer of several kilometres thickness. Due to the hostile conditions at the surface, including the extreme radiation, it is speculated that potential traces of either extant or extinct life are best to be found within the subsurface of this moon. To access the subsurface, a variety of different concepts for suitable vehicles have been proposed so far in literature, mostly based on concrete terrestrial experiences applied to an Europan scenario. However, a complete investigation of the whole trade space and evaluation of all types of possible concepts is still missing. Within the research presented here, we have firstly performed a deep investigation of design-driving requirements on the journey to and on Europa, i.e. especially within the subsurface ice layer and the ocean itself. In combination with the essential scientific objectives regarding astrobiology, four operation scenarios have been established by defining sets of essential requirements and problematic issues for each scenario in the respective environment. One of these identified scenarios has been the search for signs of life within the cracks at the ice-water interface, where important capabilities are energy efficient station keeping near the interface, as well as high navigational skills, to increase reliability in the unknown environment. In applying these operation scenarios, we have evaluated the tradespace of existing terrestrial solutions and newly proposed technological concepts, wrt. the operation of the systems and subsystems wrt. the different demands of close-to-reality mission scenarios. Using Multi-Attribute Tradespace Exploration, we were able to determine the concepts utility for each mission scenario, as well as its expenses, i.e. the effort that is needed to realize the utility. Consequently, the concepts weak points were identified. The aim of our research was to investigate potential fields of improvement and to derive enabling technologies. Since most of the time, which is spent for the realization of space exploration missions is used to develop enabling technologies, the knowledge that has been derived by the research presented here, could be a hint for an enhanced technology development, and therefore allow a more focused and more rapid realization of a future in-situ mission to Europa.
KW - Moon
KW - Oceanography

KW - Space research
KW - Technological forecasting
KW - Defining sets
KW - Enabling technologies
KW - Energy efficient
KW - Galilean moons
KW - Ice layers
KW - Ice-water interfaces
KW - In-situ
KW - Jovian moons
KW - Liquid water
KW - Multi-attributes
KW - Potential field
KW - Problematic issues
KW - Scientific objectives
KW - Space exploration mission
KW - Station keeping
KW - Still missing
KW - Technological concept
KW - Technology development
KW - Trade space
KW - Unknown environments
KW - Weak points
KW - Phase interfaces
SN - 978-161567908-9 (ISBN)
LA - English
J2 - Int. Astronaut. Cong., IAC
M3 - Conference paper
DB - Scopus
N1 - Export Date: 23 February 2023; Cited By: 0; Correspondence Address: C. Lange; German Aerospace Center (DLR), Institute of Space Systems, Germany; email: Caroline.Lange@dlr.de
ER -

TY - CONF
AU - Townsend, A.
AU - Pikuta, E.V.
AU - Guisler, M.
AU - Stahl, S.
AU - Hoover, R.B.
TI - Anaerobic psychrophiles from lake zub and lake untersee, Antarctica
PY - 2009
T2 - Proceedings of SPIE - The International Society for Optical Engineering
VL - 7441
C7 - 74410K
DO - 10.1117/12.828877
UR - https://www.scopus.com/inward/record.uri?eid=2-s2.0-70449497581&doi=10.1117%2f12.828877&partnerID=40&md5=c6ba0b2027 32b2da644d6eba0c0c86f5
AD - Astrobiology Laboratory, National Space Science and Technology Center, University of Alabama in Huntsville, Huntsville, AL 35805, 320 Sparkman Dr., United States

AB - The study of samples from Antarctica 2008 expeditions organized and successfully conducted by Richard Hoover led to the isolation of diverse anaerobic strains with psychrotolerant and psychrophilic physiology. Due to the fact that Lake Untersee has never been subject to microbiological study, this work with the samples has significant and pioneering impact to the knowledge about the biology of this unique ecosystem. Also, the astrobiological significance for the study of these ecosystems is based on new findings of ice covered water systems on other bodies of our solar system. Anaerobic psychrotolerant strain LZ-22 was isolated from a frozen sample of green moss with soils around the rhizosphere collected near Lake Zub in Antarctica. Morphology of strain LZ-22 was observed to be motile, rod shaped and spore-forming cells with sizes 1x5-10 µm. This new isolate is a mesophile with the maximum temperature of growth at 35 °C. Strain LZ-22 is able to live on media without NaCl and in media with up to 7 % (w/v) NaCl. It is catalase negative and grows only on sugars with the best growth rate being on lactose. The strain is a neutrophile and grows between pH 4.0 and 9.0 with the optimum at 7.8. Another two strains UL7-96mG and LU-96m7P were isolated from deep water samples of Lake Untersee. Proteolytic strain LU-96m7P had a truly psychrophilic nature and refused to grow at room temperature. Sugarlytic strain UL7-96mG was found to be psychrotolerant, but its rate of growth at 3°C was very high compared with other mesophiles. Two homoacetogenic psychrophilic strains A7AC-96m and AC-DS7 were isolated and purified from samples of Lake Untersee; both of them are able to grow chemolithotrophically on H 2+CO2. In the presence of lactate, these strains are able to grow only at 0-18 oC, and growth at 22 oC was observed only with yeast extract stimulation. In this paper, physiological and morphological characteristics of novel psychrophilic and psychrotolerant isolates from Antarctica 2008 expeditions will be discussed. © 2009 SPIE.
KW - Astrobiology
KW - Ecosystems
KW - Fermentation
KW - Frozen soils
KW - Lakes
KW - Sodium chloride
KW - Solar system
KW - Sugar (sucrose)
KW - Sugars
KW - Anaerobic strains
KW - Antarctica
KW - Deep Water
KW - Frozen sample
KW - Ice covered waters
KW - Maximum temperature
KW - Mesophiles
KW - Morphological characteristic
KW - Proteolytic anaerobe
KW - Psychrophiles
KW - Psychrotolerant
KW - Rod-shaped
KW - Room temperature

KW - Untersee lake
KW - Yeast extracts
KW - Strain
KW - Antarctica
KW - Fermentation
KW - Lake zub
KW - Proteolytic anaerobe
KW - Psychrophiles
KW - Untersee lake
SN - 0277786X (ISSN); 978-081947731-6 (ISBN)
LA - English
J2 - Proc SPIE Int Soc Opt Eng
M3 - Conference paper
DB - Scopus
N1 - Export Date: 23 February 2023; Cited By: 5; CODEN: PSISD
ER -

TY - JOUR
AU - Tokano, T.
TI - Limnological structure of Titan's hydrocarbon lakes and its astrobiological implication
PY - 2009
T2 - Astrobiology
VL - 9
IS - 2
SP - 147
EP - 164
DO - 10.1089/ast.2007.0220
UR - https://www.scopus.com/inward/record.uri?eid=2-s2.0-65249093565&doi=10.1089%2fast.2007.0220&partnerID=40&md5=3327612ca164a7907494545670136532
AD - Institut für Geophysik und Meteorologie, Universität zu Köln, Köln, Germany
AD - Institut für Geophysik und Meteorologie, Universität zu Köln, 50923 Kln, Albertus-Magnus-Platz, Germany
AB - Cassini radar recently detected several putative liquid hydrocarbon lakes in the polar region of Saturn's moon Titan. Such lakes may contain organic sediments deposited from the atmosphere that would promote prebiotic-type chemistry driven by cosmic rays, the result of which could be the production of more complex molecules such as nitrogen-bearing organic polymer or azides. The physical properties of the lake and their temporal evolution under Titan's present climatic setting were investigated by means of a one-dimensional lake thermal stratification model. Lakes can undergo various evolutions, depending on the initial composition and depth of the lake and hydrocarbon abundance in the near-surface atmosphere. Pure methane ponds, which may occasionally form when heavy methane hailstones reach the surface, would be transitory in that they would evaporate, freeze up, and eventually dry up. On the other hand, lakes filled with a mixture of methane, ethane, and nitrogen would be more stable; and freezing or drying would not necessarily occur in most cases. Such lakes undergo a seasonal cycle of thermal stratification in spring and early summer and convective overturning in other seasons. The summer thermal stratification near

the lake surface could be destabilized by bottom heating as a result of an enhanced geothermal heat flux, e.g., in the vicinity of cryovolcanoes. Most likely the composition of the lake and atmosphere would come to equilibrium by way of a small amount of evaporation, but the lake-atmosphere system could be repeatedly brought out of equilibrium by irregular precipitation. The viability of prebiotic-like chemistry in the lake may depend on many lake parameters, such as temperature, liquid or frozen state, and convective mixing. Moreover, convective mixing may drive suspension of solid acetylene and other sediments on the lake bottom and redistribution of dissolved gases, which might be relevant for putative life-forms that consume hydrogen and solid acetylene. Astrobiology 9, 147-164. © 2009 Mary Ann Liebert, Inc. 2009.
KW - Atmosphere
KW - Climate
KW - Ethane
KW - Evolution, Chemical
KW - Exobiology
KW - Hydrocarbons
KW - Methane
KW - Models, Theoretical
KW - Nephelometry and Turbidimetry
KW - Nitrogen
KW - Saturn
KW - Seasons
KW - Temperature
KW - Thermodynamics
KW - ethane
KW - hydrocarbon
KW - methane
KW - nitrogen
KW - article
KW - astronomy
KW - atmosphere
KW - biology
KW - chemistry
KW - climate
KW - photometry
KW - season
KW - temperature
KW - theoretical model
KW - thermodynamics
KW - Hydrocarbon lakes
KW - Limnology
KW - Prebiotic chemistry
KW - Titan
SN - 15311074 (ISSN)
C2 - 19371158
LA - English
J2 - Astrobiology
M3 - Article
DB - Scopus
N1 - Export Date: 23 February 2023; Cited By: 50; Correspondence
Address: T. Tokano; Institut für Geophysik und Meteorologie,

Universität zu Köln, 50923 Kln, Albertus-Magnus-Platz, Germany;
email: tokano@geo.uni-koeln.de
ER -

TY - CONF
AU - Mojib, N.
AU - Huang, J.
AU - Hoover, R.B.
AU - Pikuta, E.V.
AU - Storrie-Lombardi, M.
AU - Sattler, B.
AU - Andersenf, D.
AU - Bej, A.K.
TI - Diversity of bacterial communities in the lakes of schirmacher oasis, Antarctica
PY - 2009
T2 - Proceedings of SPIE - The International Society for Optical Engineering
VL - 7441
C7 - 74410J
DO - 10.1117/12.831289
UR - https://www.scopus.com/inward/record.uri?eid=2-s2.0-70449497583&doi=10.1117%2f12.831289&partnerID=40&md5=46e43deafacfcce5c05acc0580680dc0
AD - University of Alabama at Birmingham, Birmingham, AL 35294, United States
AD - National Space Science and Technology Center, NASA, Huntsville, AL 35805, United States
AD - The Univ. of Alabama at Huntsville, Huntsville, AL 35805, United States
AD - Kinohi Institute, Inc., Pasadena, CA 91101, United States
AD - Institute of Ecology, University of Innsbruck, Innsbruck, Austria
AD - Carl Sagan Center for Study of Life in Universe (SETI Institute), Mountain View, CA 94043, United States
AB - Extreme conditions such as low temperature, aridness, low availability of organic matter, high salinity and UV-radiation in terrestrial Antarctica are key factors limiting the habitation of biotic communities and ecosystem dynamics. In recent studies, it has been discovered that the bacterial communities are highly diverse and distributed widely in the extreme ecosystem of Antarctica. Besides available morphometric data, geology, and thermal profile, limited study on the microbial identification, phylogenetic analysis, diversity and distribution of microorganisms in different lakes of Schirmacher Oasis in East Antarctica has been reported. The objective of this study was to assess the microbial biodiversity and distribution using culture-independent and culture-dependent methodologies based upon bacterial 16S rRNA gene analysis in three categories of lakes, i.e., the land-locked (L), epi-shelf (E), and pro-glacial (P) lakes in Schirmacher Oasis. The water and ice samples were collected during the 2008 Tawani International Scientific Expedition. Direct culturing of the samples on R2A agar media exhibited a wide variety of pigmented bacteria. Two of the pigmented bacteria that were cultured belong to the genera,

Hymenobacter, and Flavobacterium. Cultureindependent methodology of one of the land-locked lakes L27C identified a rich microbial diversity consisting of six different phyla of bacteria. The majority of bacteria (56%) belong to the Class γ-proteobacteria within the phylum Proteobacteria. Within the Class γ-proteobacteria, Acinetobacter dominated (48%) the total microbial load. Characterization of the microbial diversity within the three different types of Antarctic lakes is important because it will help give us a better understanding of the survival mechanisms and the functionality of these bacteria in extremely cold and harsh Antarctic ecosystems. © 2009 SPIE.
KW - Astrobiology
KW - Bacteriology
KW - Biodiversity
KW - Ecosystems
KW - Lakes
KW - Organic compounds
KW - Polysaccharides
KW - RNA
KW - Taxonomies
KW - 16s rRNA
KW - Antarctica
KW - Microbial taxonomy
KW - Phylogenetics
KW - Schirmacher oasis
KW - Bacteria
KW - 16s rRNA
KW - Antarctica
KW - Biodiversity
KW - Lakes
KW - Microbial taxonomy
KW - Microorganisms
KW - Phylogenetics
KW - Schirmacher oasis
SN - 0277786X (ISSN); 978-081947731-6 (ISBN)
LA - English
J2 - Proc SPIE Int Soc Opt Eng
M3 - Conference paper
DB - Scopus
N1 - Export Date: 23 February 2023; Cited By: 8; Correspondence Address: A. K. Bej; University of Alabama at Birmingham, Birmingham, AL 35294, United States; email: abej@uab.edu; CODEN: PSISD
ER -

TY - JOUR
AU - Scappini, F.
AU - Capobianco, M.L.
AU - Casadei, F.
AU - Zamboni, R.
TI - Laboratory simulation of ultraviolet irradiation from the Sun on amino acids. III. Irradiation of glycine-tyrosine
PY - 2009
T2 - International Journal of Astrobiology
VL - 8

IS - 2
SP - 63
EP - 68
DO - 10.1017/S1473550408004333
UR - https://www.scopus.com/inward/record.uri?eid=2-s2.0-74049113251&doi=10.1017%2fS1473550408004333&partnerID=40&md5=8f2bceca6d223ebc376902719cdeafb4
AD - Istituto per Lo Studio Dei Materiali Nanostrutturati Del C.N.R., 40129 Bologna, Via P. Gobetti, 101, Italy
AD - Istituto per la Sintesi Organica e la Fotoreattivitá Del C.N.R., 40129 Bologna, Via P. Gobetti, 101, Italy
AB - The effects of near ultraviolet (UV) radiation on water solutions of tyrosine and glycine-tyrosine are investigated using a broadband xenon lamp in the region 200800 nm. These experiments form a contribution in the laboratory simulation of the solar irradiation on the building blocks of life with regard to the origin of life. Results are presented showing the photodecomposition of tyrosine and glycine-tyrosine, at different concentrations, against UV doses. The analysis of the irradiated solutions is carried out by spectroscopic and analytical techniques. The findings of our laboratory simulations are used to constrain the early stages of the life emerging process. © 2009 Cambridge University Press.
KW - amino acid
KW - analytical method
KW - exobiology
KW - experimental study
KW - irradiation
KW - laboratory method
KW - origin of life
KW - simulation
KW - ultraviolet radiation
KW - Amino acids
KW - Astrobiology
KW - Chemical evolution
KW - Dipeptides
KW - Origin of life
KW - UV irradiation
SN - 14753006 (ISSN)
LA - English
J2 - Int. J. Astrobiology
M3 - Article
DB - Scopus
N1 - Export Date: 23 February 2023; Cited By: 2; Correspondence Address: F. Scappini; Istituto per Lo Studio Dei Materiali Nanostrutturati Del C.N.R., 40129 Bologna, Via P. Gobetti, 101, Italy; email: F.Scappini@bo.ismn.cnr.it
ER -

TY - JOUR
AU - Fendrihan, S.
AU - Musso, M.
AU - Stan-Lotter, H.
TI - Raman spectroscopy as a potential method for the detection of extremely halophilic archaea embedded in halite in terrestrial and

possibly extraterrestrial samples
PY - 2009
T2 - Journal of Raman Spectroscopy
VL - 40
IS - 12
SP - 1996
EP - 2003
DO - 10.1002/jrs.2357
UR - https://www.scopus.com/inward/record.uri?eid=2-s2.0-70449612869&doi=10.1002%2fjrs.2357&partnerID=40&md5=6665ecd414ed9d136356da122116febe
AD - Department of Molecular Biology, Division of Microbiology, University of Salzburg, Billrothstrasse 11, A-5020 Salzburg, Austria
AD - Department of Materials Engineering and Physics, Division of Physics and Biophysics, University of Salzburg, Hellbrunnerstrasse 34, A-5020 Salzburg, Austria
AB - Evidence for thewidespread occurrence of extraterrestrialhalite, particularly on Mars, has led to speculations on thepossibility of halophilic microbial forms of life; these ideas have been strengthened by reports of viable haloarchaea from sediments of geological age (millions of years). Raman spectroscopy, being a sensitive detection method for future astrobiological investigations onsite, has been used in the current study for the detection of nine different extremely halophilic archaeal strains which had been embedded in laboratory-made halite crystals in order to simulate evaporitic conditions. The cells accumulated preferentially in tiny fluid inclusions, in simulation of the precipitation of salt in natural brines. FT-Raman spectroscopy using laser excitationat 1064 nm anddispersive micro Raman spectroscopy at 514.5 nm wereapplied. Thespectra showed prominent peaks at 1507, 1152 and 1002 cm-1 which are attributed to haloarchaeal C50 carotenoid compounds (mainly bacterioruberins). Their intensity varied from strain to strain at 1064-nm laser excitation.Other distinguishable features were peaks due to peptide bonds (amide I, amide III) and to nucleic acids. No evidence for fatty acids was detected, consistent with their general absence in all archaea. These results contribute to a growing database on Raman spectra of terrestrial microorganisms from hypersaline environments and highlight the influence of the different macromolecular composition of diverse strains on these spectra. © 2009 John Wiley & Sons, Ltd.
KW - Astrobiology
KW - Bacterioruberins
KW - Carotenoids
KW - Extremely halophilic archaea
KW - Fluid inclusions
KW - Halite
KW - Martian subsurface
KW - Raman spectroscopy
SN - 10974555 (ISSN)
LA - English
J2 - J. Raman Spectrosc.
M3 - Article
DB - Scopus

N1 - Export Date: 23 February 2023; Cited By: 49; Correspondence Address: M. Musso; Department of Materials Engineering and Physics, Division of Physics and Biophysics, University of Salzburg, Hellbrunnerstrasse 34, A-5020 Salzburg, Austria; email: Maurizio.Musso@sbg.ac.at; CODEN: JRSPA
ER -

TY - JOUR
AU - La Duc, M.T.
AU - Osman, S.
AU - Vaishampayan, P.
AU - Piceno, Y.
AU - Andersen, G.
AU - Spry, J.A.
AU - Venkateswaran, K.
TI - Comprehensive census of bacteria in clean rooms by using DNA microarray and cloning methods
PY - 2009
T2 - Applied and Environmental Microbiology
VL - 75
IS - 20
SP - 6559
EP - 6567
DO - 10.1128/AEM.01073-09
UR - https://www.scopus.com/inward/record.uri?eid=2-s2.0-70349923761&doi=10.1128%2fAEM.01073-09&partnerID=40&md5=4fa34bea45ae8bc747eb2f8dd9a7a34a
AD - Biotechnology and Planetary Protection, NASA Jet Propulsion Laboratory, California Institute of Technology, Pasadena, CA 91109, Oak Grove Dr., United States
AD - Lawrence Berkeley National Laboratory, Berkeley, CA 85721, United States
AB - A census of clean room surface-associated bacterial populations was derived from the results of both the cloning and sequencing of 16S rRNA genes and DNA microarray (PhyloChip) analyses. Samples from the Lockheed Martin Aeronautics Multiple Testing Facility (LMA-MTF), the Kennedy Space Center Payload Hazard and Servicing Facility (KSC-PHSF), and the Jet Propulsion Laboratory Spacecraft Assembly Facility (JPL-SAF) clean rooms were collected during the various assembly phases of the Phoenix and Mars Science Laboratory (MSL) spacecraft. Clone library-derived analyses detected a larger bacterial diversity prior to the arrival of spacecraft hardware in these clean room facilities. PhyloChip results were in agreement with this trend but also unveiled the presence of anywhere from 9- to 70-fold more bacterial taxa than cloning approaches. Among the facilities sampled, the JPL-SAF (MSL mission) housed a significantly less diverse bacterial population than either the LMA-MTF or KSC-PHSF (Phoenix mission). Bacterial taxa known to thrive in arid conditions were frequently detected in MSL-associated JPL-SAF samples, whereas proteobacterial lineages dominated Phoenix-associated KSC-PHSF samples. Comprehensive bacterial censuses, such as that reported here, will help space-faring nations preemptively identify contaminant biomatter that may compromise extraterrestrial life detection experiments. The robust nature and high sensitivity

of DNA microarray technologies should prove beneficial to a wide range of scientific, electronic, homeland security, medical, and pharmaceutical applications and to any other ventures with a vested interest in monitoring and controlling contamination in exceptionally clean environments.

KW - Bacteria
KW - Base Sequence
KW - Biodiversity
KW - Cloning, Molecular
KW - DNA Primers
KW - DNA, Bacterial
KW - Environment, Controlled
KW - Environmental Microbiology
KW - Extraterrestrial Environment
KW - Genes, Bacterial
KW - Molecular Sequence Data
KW - Oligonucleotide Array Sequence Analysis
KW - Phylogeny
KW - RNA, Bacterial
KW - RNA, Ribosomal, 16S
KW - Space Flight
KW - United States
KW - United States National Aeronautics and Space Administration
KW - Bacteria (microorganisms)
KW - Proteobacteria
KW - Clean rooms
KW - Cloning
KW - DNA
KW - Genes
KW - Indoor air pollution
KW - Internal combustion engines
KW - Martian surface analysis
KW - Nucleic acids
KW - Optical transfer function
KW - Space platforms
KW - Surveys
KW - RNA 16S
KW - 16S rRNA gene
KW - Arid conditions
KW - Bacterial diversity
KW - Bacterial population
KW - Bacterial taxa
KW - Biomatter
KW - Clean environment
KW - Clone library
KW - Cloning and sequencing
KW - DNA micro-array
KW - DNA microarray technology
KW - Extraterrestrial life
KW - High sensitivity
KW - Home land security
KW - Jet Propulsion Laboratory
KW - Kennedy space centers
KW - Lockheed Martin Aeronautics

KW - Mars science laboratory
KW - Monitoring and controlling
KW - Multiple testing
KW - Pharmaceutical applications
KW - Phoenix mission
KW - biodiversity
KW - clone
KW - DNA
KW - microbial activity
KW - population structure
KW - RNA
KW - sampling
KW - analytic method
KW - article
KW - bacterium contamination
KW - bacterium detection
KW - biodiversity
KW - DNA microarray
KW - molecular cloning
KW - monitoring
KW - nonhuman
KW - nucleotide sequence
KW - phylogenetic tree
KW - Proteobacteria
KW - sensitivity and specificity
KW - space flight
KW - taxon
KW - unindexed sequence
KW - Bacteriology
SN - 10985336 (ISSN)
C2 - 19700540
LA - English
J2 - Appl. Environ. Microbiol.
M3 - Article
DB - Scopus
N1 - Export Date: 23 February 2023; Cited By: 71; Correspondence Address: K. Venkateswaran; Biotechnology and Planetary Protection, NASA Jet Propulsion Laboratory, California Institute of Technology, Pasadena, CA 91109, Oak Grove Dr., United States; email: kjvenkat@jpl.nasa.gov; CODEN: AEMID
ER -

TY - JOUR
AU - Villanueva, G.L.
AU - Mumma, M.J.
AU - Bonev, B.P.
AU - DiSanti, M.A.
AU - Gibb, E.L.
AU - Böhnhardt, H.
AU - Lippi, M.
TI - A sensitive search for deuterated water in comet 8P/Tuttle
PY - 2009
T2 - Astrophysical Journal
VL - 690

IS - 1 PART 2
SP - L5
EP - L9
DO - 10.1088/0004-637X/690/1/L5
UR - https://www.scopus.com/inward/record.uri?eid=2-
s2.0-67649646251&doi=10.1088%2f0004-637X%2f690%2f1%2fL5&partnerID=40
&md5=a431317853d99f7ae350c4ce8ffae754
AD - Solar System Exploration Division, Mailstop 693, NASA Goddard
Space Flight Center, Greenbelt, MD 20771, United States
AD - Department of Physics, Catholic University of America,
Washington, DC 20064, United States
AD - Department of Physics and Astronomy, University of Missouri-
St. Louis, St. Louis, MO 63121, United States
AD - Max-Planck Institute for Solar System Research, D-37191
Katlenburg-Lindau, Germany
AB - We report a sensitive search for deuterated water (HDO) in
comet 8P/Tuttle using high-resolution spectroscopy at infrared (IR)
wavelengths. The deuterium enrichment of cometary water is one of
the most important cosmogonic indicators in comets. The (D/H)H2O
ratio preserves information about the conditions under which comet
material formed, and tests the possible contribution of comets in
delivering water for Earth's oceans. Water (H 2O) and HDO were
sampled in comet 8P/Tuttle from 2008 January 27 to 2008 February 3
using the new IR spectrometer (Cryogenic Infrared Echelle
Spectrograph) at the 8.2 m Antu telescope of the Very Large
Telescope Observatory atop Cerro Paranal, Chile. Twenty-three lines
of HDO were sampled near 3.7 µm, leading to a production rate of
4.73 ± 1.68 × 1025 s-1. Combining this value with the H2O production
rate of 5790 ± 250 × 1025 s-1 provides a formal value of (D/
H)h2o=4.09± 1.45 × 10-4 in comet 8P/Tuttle. This value is larger by
a factor of 2.62 ±0.93 than Vienna Standard Mean Ocean Water, and is
comparable to enrichment factors measured for three other Oort cloud
comets. The technique described here provides unprecedented
sensitivities, ultimately permitting us to routinely measure this
prime cosmogonic indicator, even in comets having relatively modest
gas production rate like 8P/Tuttle. © 2009. The American
Astronomical Society.
KW - Astrobiology
KW - Astrochemistry
KW - Comets: general
KW - Comets: individual (8P/Tuttle)
KW - Infrared: solar system
PB - Institute of Physics Publishing
SN - 0004637X (ISSN)
LA - English
J2 - Astrophys. J.
M3 - Article
DB - Scopus
N1 - Export Date: 23 February 2023; Cited By: 86; Correspondence
Address: G. L. Villanueva; Solar System Exploration Division,
Mailstop 693, NASA Goddard Space Flight Center, Greenbelt, MD 20771,
United States; email: Geronimo.Villanueva@nasa.gov
ER -

TY - CONF
AU - Reh, K.R.
TI - Titan Saturn system mission
PY - 2009
T2 - IEEE Aerospace Conference Proceedings
C7 - 4839316
DO - 10.1109/AERO.2009.4839316
UR - https://www.scopus.com/inward/record.uri?eid=2-s2.0-70349120991&doi=10.1109%2fAERO.2009.4839316&partnerID=40&md5=ec84f7032619f6edd3c9233443b9c2eb
AD - Jet Propulsion Laboratory, California Institute of Technology, Pasadena, CA 91109, 4800 Oak Grove Dr., United States
AB - Titan is a high priority for exploration, as recommended by NASA's 2006 Solar System Exploration (SSE) Roadmap [1], NASA's 2003 National Research Council (NRC) Decadal Survey [2] and ESA's Cosmic Vision Program Themes. 1,2 Recent revolutionary Cassini-Huygens discoveries have dramatically escalated interest in Titan as the next scientific target in the outer Solar System. Results of this study as documented in the TSSM Final Report [3] and Titan Saturn System Mission (TSSM) NASA/ESA Joint Summary Report [4] demonstrates that an exciting Titan Saturn System Mission (TSSM) that explores two worlds of intense astrobiological interest can be initiated now as a single NASA/ESA collaboration. Following 50 years of space exploration, the Cassini-Huygens mission has revealed the Earth-like world of Saturn's moon Titan and showed the potential habitability of another moon, Enceladus. As anticipated by the 2003 Decadal Survey, recent Cassini-Huygens discoveries have revolutionized our understanding of the Titan system and its potential for harboring the "ingredients" necessary for life. These discoveries reveal that Titan is very rich in organics, possibly contains a vast subsurface ocean, and has energy sources to drive chemical evolution. The complex interaction between the atmosphere and surface produces lakes, dunes, and seasonal changes that are features that Titan shares with Earth. Cassini's discovery of active geysers on Enceladus revealed a second icy moon in the Saturn system that is synergistic with Titan in understanding planetary evolution and in adding another potential abode in the Saturn system for life as we know it. These discoveries have dramatically escalated the interest in Titan as the next scientific target for an outer planet mission. Although the scope of science possible at Titan covers the entire range of planetary science disciplines, the TSSM team has developed a mission that focuses NASA and ESA resources on the highest priority science questions. Results of this study confirm that a flagship-class mission to Titan (including the Saturn system and Enceladus) can be done at acceptable risk within the specified budgetary constraints and can proceed now. ©2009 IEEE.
KW - Astronomy
KW - Cosmology
KW - Earth atmosphere
KW - Interplanetary flight
KW - NASA
KW - Oceanography
KW - Solar equipment
KW - Solar system

KW - Surveys
KW - Technological forecasting
KW - Budgetary constraints
KW - Cassini
KW - Cassini-huygens
KW - Cassini-Huygens mission
KW - Chemical evolution
KW - Complex interaction
KW - Enceladus
KW - Energy source
KW - Icy Moons
KW - National Research Council
KW - Organics
KW - Outer solar system
KW - Outer-planet missions
KW - Planetary evolutions
KW - Planetary science
KW - Roadmap
KW - Saturn's moons
KW - Scientific targets
KW - Seasonal changes
KW - Solar system exploration
KW - Space explorations
KW - Sub-surface ocean
KW - Titan Systems
KW - Lunar missions
SN - 1095323X (ISSN); 978-142442622-5 (ISBN)
LA - English
J2 - IEEE Aerosp. Conf. Proc.
M3 - Conference paper
DB - Scopus
N1 - Export Date: 23 February 2023; Cited By: 3; Correspondence Address: K. R. Reh; Jet Propulsion Laboratory, California Institute of Technology, Pasadena, CA 91109, 4800 Oak Grove Dr., United States; email: kim.r.reh@jpl.nasa.gov
ER -

TY - JOUR
AU - Cabrol, N.A.
AU - Grin, E.A.
AU - Chong, G.
AU - Minkley, E.
AU - Hock, A.N.
AU - Yu, Y.
AU - Bebout, L.
AU - Fleming, E.
AU - Häder, D.P.
AU - Demergasso, C.
AU - Gibson, J.
AU - Escudero, L.
AU - Dorador, C.
AU - Lim, D.
AU - Woosley, C.
AU - Morris, R.L.

AU - Tambley, C.
AU - Gaete, V.
AU - Galvez, M.E.
AU - Smith, E.
AU - Uskin-Peate, I.
AU - Salazar, C.
AU - Dawidowicz, G.
AU - Majerowicz, J.
TI - The High-Lakes Project
PY - 2009
T2 - Journal of Geophysical Research: Biogeosciences
VL - 114
IS - 4
C7 - G00D06
DO - 10.1029/2008JG000818
UR - https://www.scopus.com/inward/record.uri?eid=2-s2.0-77954229018&doi=10.1029%2f2008JG000818&partnerID=40&md5=96ff03acd6bb6de5d86e3e088103fe3b
AD - Space Science and Astrobiology Division, NASA Ames Research Center, MS 245-3, Moffett Field, CA 94035-1000, United States
AD - SETI Carl Sagan Center, Mountain View, CA 94043, 515 North Whisman Road, United States
AD - Centro de Investigación Científica Y Tecnológica para Minería, Santiago, Chile
AD - Department of Biological Sciences, Carnegie Mellon University, Pittsburgh, PA 15213-3890, United States
AD - Department of Earth and Space Sciences, University of California, Los Angeles, CA 90095, 595 Charles Young Drive East, United States
AD - Department of Civil and Environmental Engineering, Carnegie Mellon University, Pittsburgh, PA 15213-3890, United States
AD - Department Botanik, University of Erlangen, D-91058 Erlangen, Staudtstrasse 5, Germany
AD - Centro de Biotecnología, Universidad Católica del Norte, Antofagasta, Avenida Angamos 0610, Chile
AD - Marine Research Laboratories, Tasmanian Aquaculture and Fisheries Institute, University of Tasmania, Hobart, TAS 7001, Private Bag 49, Australia
AD - CHEP, Office 2707, Huechuraba, Santiago, Avenida Del Valle 961, Chile
AD - Departamento de Química, Universidad Católica del Norte, Antofagasta, Avenida Angamos 0610, Chile
AD - Ecole Normale Supérieure, F-75005, Paris, 24 rue Lhomond, France
AD - Discoverer Ketty Lund Exploration Vessel, Key West, FL 33040, 330 Julia Street, United States
AD - Geoscience Department, University of Iowa, 121 Trowbridge Hall, Iowa City, IA 52242, United States
AD - Clínica Mutual de Seguridad, Punta Arenas, Avenida España 01890, Chile
AD - ESRI, F-92190 Meudon, 21 rue des Capucins, France
AB - The High Lakes Project is a multidisciplinary astrobiological investigation studying high-altitude lakes between 4200 m and 6000 m elevation in the Central Andes of Bolivia and Chile. Its primary

objective is to understand the impact of increased environmental stress on the modification of lake habitability potential during rapid climate change as an analogy to early Mars. Their unique geophysical environment and mostly uncharted ecosystems have added new objectives to the project, including the assessment of the impact of low-ozone/high solar irradiance in nonpolar aquatic environments, the documentation of poorly known ecosystems, and the quantification of the impact of climate change on lake environment and ecosystem. Data from 2003 to 2007 show that UV flux is 165% that of sea level with maximum averaged UVB reaching 4 W/m2. Short UV wavelengths (260–270 nm) were recorded and peaked at 14.6 mW/m2. High solar irradiance occurs in an atmosphere permanently depleted in ozone falling below ozone hole definition for 33–36 days and between 30 and 35% depletion the rest of the year. The impact of strong UVB and UV erythemally weighted daily dose on life is compounded by broad daily temperature variations with sudden and sharp fluctuations. Lake habitat chemistry is highly dynamical with notable changes in yearly ion concentrations and pH resulting from low and variable yearly precipitation. The year-round combination of environmental variables define these lakes as end-members. In such an environment, they host ecosystems that include a significant fraction of previously undescribed species of zooplankton, cyanobacterial, and bacterial populations. Copyright 2009 by the American Geophysical Union.
KW - Andes
KW - Bolivia
KW - Chile
KW - Bacteria (microorganisms)
KW - Cyanobacteria
KW - altitude
KW - bacterium
KW - climate change
KW - cyanobacterium
KW - data interpretation
KW - environmental stress
KW - geophysics
KW - habitat quality
KW - lake ecosystem
KW - Mars
KW - ozone depletion
KW - pH
KW - precipitation (climatology)
KW - quantitative analysis
KW - solar radiation
KW - ultraviolet radiation
KW - water chemistry
KW - zooplankton
PB - Blackwell Publishing Ltd
SN - 01480227 (ISSN)
LA - English
J2 - J. Geophys. Res. G Biogeosci.
M3 - Article
DB - Scopus
N1 - Export Date: 23 February 2023; Cited By: 39; Correspondence

Address: N. A. Cabrol; Space Science and Astrobiology Division, NASA Ames Research Center, MS 245-3, Moffett Field, CA 94035-1000, United States; email: nathalie.a.cabrol@nasa.gov
ER -

TY - CONF
AU - Thangavelu, M.
AU - Albarico, K.L.
AU - Doyle, M.K.
AU - Enomoto, J.
AU - Harmon, C.
AU - Hsu, C.Y.
AU - Jordan, N.
AU - Mekonnen, E.
AU - Moring, J.
AU - Patel, A.
AU - Pugh, D.
AU - Shrestha, R.
AU - Stuppy, W.P.
TI - Return to the Moon: Looking Glass 204
PY - 2009
T2 - AIAA Space 2009 Conference and Exposition
C7 - 2009-6612
UR - https://www.scopus.com/inward/record.uri?eid=2-s2.0-77958581384&partnerID=40&md5=cdbbe7a2540553f2c6d1a8c75f6e8049
AD - Astronautics Department, Viterbi School of Engineering, University of Southern California, Los Angeles, CA 90089-1191, United States
AB - As we lay down plans and ramp up development of transportation systems for returning people to the Moon, alternative concepts are being proposed for activities to conduct there in order to gain the experience necessary to prepare for more ambitious human interplanetary expeditions to Mars and beyond. Fully employing NASA's Constellation transportation systems such as the Orion Crew Exploration Vehicle and the Altair Lunar Lander as baseline elements, the USC ASTE527 Return to the Moon: Looking Glass 204 Project pondered the following question: What activities precisely can we do on the Moon, with crew and robots, that can immediately (very short timeframe-2015-2040) benefit not only the science and engineering community, but also humanity as a whole, on a permanent basis ? The establishment of a sturdy cislunar communications system followed by critical crew rescue capability in the proximity of a primary lunar habitat are seen as the foundation blocks for this architecture. Once the foundation is reliably established, essential physical infrastructure to support the emplacement of a suite of permanent, evolvable observatories, long-range traverses to conduct geology and astrobiology, and critical crew support were addressed. Manned, pressurized rovers are essential in order for crew to access observatory sites to set up, calibrate and evolve these man tended facilities which are located along the proposed traverse route. Rovers and crew are also needed to deploy, service and evolve science payloads that are autonomously landed far apart in remote regions of the lunar globe. Participants were tasked to create their own system concepts, which they thought were useful. They presented

material on pertinent concepts listed below: 1. The Altair Descent Stage based Cislunar Communication System 2. A lunar surface CEV / Altair hybrid for Cislunar Ambulance 3. Astronaut Health and Critical Care 4. Lunar Landing Pads, Access Roads and Habitat Platforms/Dust Mitigation 5. Observatories: SPIDAR Concept for VLF Astronomy Using Craters 6. LunaRRT: Lunar Real-Time Teleoperations 7. ISRU: Processing and Use of Lunar Rocks for Infrastructure Development 8. Pressurized Lunar Rover - The Science and Service Cars 9. Lunar Power Peaks - Use of Mountain Peaks for Microwave Relays 10. A South Polar Geological and Astrobiological Traverse - with Twin Rovers 11. Inflatable Technology for Pressurized Rovers and Mobile Habitats 12. Concept for Outreach -LunarSS and Kaijuu These activities will directly advance the experience necessary for interplanetary missions. The philosophy of crew and robots working together employing real-time telerobotics(RTT) to accomplish various buildup activities was preferred over the more conventional, economical, linear, robots first approach, in order to maintain high public interest that is crucial for such a high visibility project. The architecture recommends a substantial outreach component as well as international collaboration and US leadership in the new century. Synopses of the topics listed above are presented here. Copyright © 2009 by Madhu Thangavelu .
KW - Astronomy
KW - Buildings
KW - Communication systems
KW - Ecosystems
KW - Glass
KW - International cooperation
KW - Interplanetary flight
KW - Lunar missions
KW - NASA
KW - Observatories
KW - Robots
KW - Access roads
KW - Communications systems
KW - Crew exploration vehicles
KW - Critical care
KW - Evolvable
KW - Foundation blocks
KW - Infrastructure development
KW - International collaborations
KW - Interplanetary mission
KW - Lunar habitats
KW - Lunar Lander
KW - Lunar rovers
KW - Lunar surface
KW - Mountain peaks
KW - New centuries
KW - Public interest
KW - Remote regions
KW - Rescue capability
KW - Robots working
KW - Science and engineering
KW - System concepts

KW - Tele-operations
KW - Tele-robotics
KW - Transportation system
KW - Lunar landing
SN - 978-156347979-3 (ISBN)
LA - English
J2 - AIAA Space Conf. Expos.
M3 - Conference paper
DB - Scopus
N1 - Export Date: 23 February 2023; Cited By: 5
ER -

TY - JOUR
AU - Stockton, A.M.
AU - Chiesl, T.N.
AU - Lowenstein, T.K.
AU - Amashukeli, X.
AU - Grunthaner, F.
AU - Mathies, R.A.
TI - Capillary electrophoresis analysis of organic amines and amino acids in saline and acidic samples using the mars organic analyzer
PY - 2009
T2 - Astrobiology
VL - 9
IS - 9
SP - 823
EP - 831
DO - 10.1089/ast.2009.0357
UR - https://www.scopus.com/inward/record.uri?eid=2-s2.0-72449125650&doi=10.1089%2fast.2009.0357&partnerID=40&md5=622a6bbba2cdb1b4a51ce46d88d31a9e
AD - Department of Chemistry, University of California, Berkeley, CA 94720, United States
AD - Department of Geological Sciences and Environmental Studies, Binghamton University, Binghamton, NY, United States
AD - Jet Propulsion Laboratories, California Institute of Technology, Pasadena, CA, United States
AB - The Mars Organic Analyzer (MOA) has enabled the sensitive detection of amino acid and amine biomarkers in laboratory standards and in a variety of field sample tests. However, the MOA is challenged when samples are extremely acidic and saline or contain polyvalent cations. Here, we have optimized the MOA analysis, sample labeling, and sample dilution buffers to handle such challenging samples more robustly. Higher ionic strength buffer systems with pKa values near pH 9 were developed to provide better buffering capacity and salt tolerance. The addition of ethylaminediaminetetraacetic acid (EDTA) ameliorates the negative effects of multivalent cations. The optimized protocol utilizes a 75 mM borate buffer (pH 9.5) for Pacific Blue labeling of amines and amino acids. After labeling, 50mM (final concentration) EDTA is added to samples containing divalent cations to ameliorate their effects. This optimized protocol was used to successfully analyze amino acids in a saturated brine sample from Saline Valley, California, and a subcritical water extract of a highly acidic sample from the Río Tinto, Spain. This

work expands the analytical capabilities of the MOA and increases its sensitivity and robustness for samples from extraterrestrial environments that may exhibit pH and salt extremes as well as metal ions. Key Words: Amino acid analysis–Astrobiology– Planetary exploration–Lab-on-a-chip–Micro total analysis systems. © 2009, Mary Ann Liebert, Inc.
KW - Acids
KW - Amines
KW - Amino Acids
KW - Buffers
KW - California
KW - Cations
KW - Edetic Acid
KW - Electrophoresis, Capillary
KW - Extraterrestrial Environment
KW - Magnesium Chloride
KW - Mars
KW - Reference Standards
KW - Salinity
KW - Salts
KW - Sodium Chloride
KW - Spain
KW - acid
KW - amine
KW - amino acid
KW - brine
KW - buffer
KW - cation
KW - edetic acid
KW - inorganic salt
KW - magnesium chloride
KW - sodium chloride
KW - article
KW - astronomy
KW - capillary electrophoresis
KW - chemistry
KW - instrumentation
KW - methodology
KW - microclimate
KW - salinity
KW - Spain
KW - standard
KW - United States
SN - 15311074 (ISSN)
C2 - 19968460
LA - English
J2 - Astrobiology
M3 - Article
DB - Scopus
N1 - Export Date: 23 February 2023; Cited By: 29; Correspondence Address: R. A. Mathies; Department of Chemistry, University of California, Berkeley, CA 94720, United States; email: ramathies@berkeley.edu
ER -

TY - CONF
AU - Gaskin, J.A.
AU - Carini, G.A.
AU - Chen, W.
AU - Geronimo, G.D.
AU - Elsner, R.F.
AU - Keister, J.W.
AU - Kramer, G.
AU - Li, Z.
AU - Ramsey, B.D.
AU - Rehak, P.
AU - Siddons, D.P.
TI - Development of a silicon drift detector array: An X-ray fluorescence spectrometer for remote surface mapping
PY - 2009
T2 - Proceedings of SPIE - The International Society for Optical Engineering
VL - 7441
C7 - 744118
DO - 10.1117/12.826389
UR - https://www.scopus.com/inward/record.uri?eid=2-s2.0-70449468453&doi=10.1117%2f12.826389&partnerID=40&md5=0474e2d1e93492982b66ca41731bc2bd
AD - NASA Marshall Space Flight Center, Space Science Office, VP62, Huntsville, AL 35812, United States
AD - Brookhaven National Laboratory, Upton, NY 11973, United States
AD - Bear Fight Center, Winthrop, WA 98862, United States
AB - Over the past three years NASA Marshall Space Flight Center has been collaborating with Brookhaven National Laboratory to develop a modular Silicon Drift Detector (SDD) X-ray Spectrometer (XRS) intended for fine surface mapping of the light elements of the Moon. The value of fluorescence spectrometry for surface element mapping is underlined by the fact that the technique has recently been employed by three lunar orbiter missions; Kaguya, Chandrayaan-1, and Chang'e. The SDD-XRS instrument we have been developing can operate at a low energy threshold (i.e. is capable of detecting Carbon), comparable energy resolution to Kaguya (<150 eV at 5.9 keV) and an order of magnitude lower power requirement, making much higher sensitivities possible. Furthermore, the intrinsic radiation resistance of the SDD makes it useful even in radiation-harsh environments such as that of Jupiter and its surrounding Moons. © 2009 SPIE.
KW - Astrobiology
KW - Chemical elements
KW - Detectors
KW - Fluorescence
KW - Fluorescence spectroscopy
KW - Mapping
KW - NASA
KW - Positron emission tomography
KW - Spectrometry
KW - X ray spectrometers
KW - Brookhaven national laboratory

KW - Energy resolutions
KW - Fluorescence spectrometry
KW - Intrinsic radiation
KW - Jupiters
KW - Light elements
KW - Low energy thresholds
KW - Lunar orbiter
KW - NASA Marshall
KW - Order of magnitude
KW - Power requirement
KW - Radiation-harsh environment
KW - Remote surfaces
KW - Silicon drift detector
KW - Surface elements
KW - Surface mapping
KW - X-ray fluorescence spectrometer
KW - Lunar missions
KW - Silicon drift detector
KW - Surface elemental mapping
KW - X-ray spectrometer
SN - 0277786X (ISSN); 978-081947731-6 (ISBN)
LA - English
J2 - Proc SPIE Int Soc Opt Eng
M3 - Conference paper
DB - Scopus
N1 - Export Date: 23 February 2023; Cited By: 6; Correspondence Address: J. A. Gaskin; NASA Marshall Space Flight Center, Space Science Office, VP62, Huntsville, AL 35812, United States; email: Jessica.Gaskin@nasa.gov; CODEN: PSISD
ER -

TY - JOUR
AU - Peeters, Z.
AU - Quinn, R.
AU - Martins, Z.
AU - Sephton, M.A.
AU - Becker, L.
AU - Van Loosdrecht, M.C.M.
AU - Brucato, J.
AU - Grunthaner, F.
AU - Ehrenfreund, P.
TI - Habitability on planetary surfaces: Interdisciplinary preparation phase for future Mars missions
PY - 2009
T2 - International Journal of Astrobiology
VL - 8
IS - 4
SP - 301
EP - 315
DO - 10.1017/S1473550409990140
UR - https://www.scopus.com/inward/record.uri?eid=2-s2.0-77149165754&doi=10.1017%2fS1473550409990140&partnerID=40&md5=4ccaee9a6575dc63f2c6711d179c7b38
AD - Leiden Institute of Chemistry, 2333 CC Leiden, Einsteinweg 55,

Netherlands
AD - NASA Goddard Space Flight Center, Code 691, Greenbelt, MD 20771, United States
AD - SETI Institute, NASA Ames Research Center, Moffett Field, CA 94035, United States
AD - Department of Earth Science and Engineering, Imperial College London, London SW7 2AZ, United Kingdom
AD - John Hopkins University, Baltimore, MD 21218, 3400 North Charles St, United States
AD - Delft University of Technology, Faculty of Applied Sciences, Department of Biotechnology, 2628 BC Delft, Julianalaan 67, Netherlands
AD - INAF Osservatorio Astrofisico di Arcetri, 50125 Firenze, L.go E. Fermi 5, Italy
AD - In Situ Exploration Technology Group, NASA Jet Propulsion Laboratory, Pasadena, CA, United States
AD - Space Policy Institute, Elliott School of International Affairs, Washington DC, United States
AB - Life on Earth is one of the outcomes of the formation and evolution of our solar system and has adapted to every explored environment on planet Earth. Recent discoveries have shown that life can exist in extreme environments, such as hydrothermal vents, in deserts and in ice lakes in Antarctica. These findings challenge the definition of the planetary habitable zone. The objective of future international planetary exploration programmes is to implement a long-term plan for the robotic and human exploration of solar system bodies. Mars has been a central object of interest in the context of extraterrestrial life. The search for extinct or extant life on Mars is one of the main goals of space missions to the Red Planet during the next decade. In this paper we describe the investigation of the physical and chemical properties of Mars soil analogues collected in arid deserts. We measure the pH, redox potential and ion concentrations, as well as carbon and amino acid abundances of soils collected from the Atacama desert (Chile and Peru) and the Salten Skov sediment from Denmark. The samples show large differences in their measured properties, even when taken only several meters apart. A desert sample and the Salten Skov sediment were exposed to a simulated Mars environment to test the stability of amino acids in the soils. The presented laboratory and field studies provide limits to exobiological models, evidence on the effects of subsurface mineral matrices, support current and planned space missions and address planetary protection issues. © Cambridge University Press 2009.
KW - exobiology
KW - Mars
KW - planetary surface
KW - satellite mission
KW - Habitability
KW - Mars soil analogues
KW - Planetary organics
SN - 14753006 (ISSN)
LA - English
J2 - Int. J. Astrobiology
M3 - Article

DB - Scopus
N1 - Export Date: 23 February 2023; Cited By: 21
ER -

TY - CONF
AU - Konesky, G.
TI - Can Venus shed microorganisms?
PY - 2009
T2 - Proceedings of SPIE - The International Society for Optical Engineering
VL - 7441
C7 - 74410H
DO - 10.1117/12.828643
UR - https://www.scopus.com/inward/record.uri?eid=2-s2.0-70449472485&doi=10.1117%2f12.828643&partnerID=40&md5=8446c5c59eebecc87611df70cfec82b4
AD - K-Plasma, Ltd., Hampton Bays, NY 11946, 3 Rolling Hill Rd., United States
AB - The pale featureless cloud tops of Venus reveal a rich complexity when viewed in ultraviolet. These features result from an unknown absorber brought up from lower atmospheric levels by convection, particularly at lower latitudes. While the surface of Venus is extremely hostile to life as we know it, there exists a habitable region in the atmosphere, centered at approximately 50 km, where the temperature ranges from 30 to 80°C and the pressure is one bar. Numerous examples of cloud-borne life exist on Earth. However, the environment in the Venus atmospheric habitable zone has only a few ppm of water which is present as misty droplets, strong sulfuric acid, and intense UV illumination. The proposal that putative cloud-borne life forms in Venus' atmospheric habitable zone can be transported to Earth by a solar conveyance face several challenges. Vigorous convective mixing, especially at the lower latitudes is considered as a means of transport to the upper reaches of Venus' atmosphere. Potential propulsive forces imparted by both solar wind and sunlight pressure are considered as a means of achieving escape velocity from Venus. Additional hurdles include direct exposure by such transported life forms to the rigors of the space environment. These are contrasted to those experienced by microorganisms that may be carried within meteorites and comets. A middle ground is perhaps demonstrated by plankton that has been observed at high altitudes on Earth, likely lofted there by a hurricane, which is encased in protective ice crystals. © 2009 SPIE.
KW - Astrobiology
KW - Erosion
KW - Microorganisms
KW - Sulfuric acid
KW - Upper atmosphere
KW - Atmospheric levels
KW - Conjunctions of Venus
KW - Convective mixing
KW - Escape velocities
KW - High altitude
KW - Ice crystals
KW - Life form

KW - Panspermia
KW - Propulsive forces
KW - Solar sailing
KW - Space environment
KW - Sunlight pressure
KW - Temperature range
KW - UV illuminations
KW - Venus habitability
KW - Solar wind
KW - Cloud life
KW - Conjunctions of Venus
KW - Panspermia
KW - Solar sailing
KW - Solar wind erosion
KW - Venus habitability
SN - 0277786X (ISSN); 978-081947731-6 (ISBN)
LA - English
J2 - Proc SPIE Int Soc Opt Eng
M3 - Conference paper
DB - Scopus
N1 - Export Date: 23 February 2023; Cited By: 5; Correspondence Address: G. Konesky; K-Plasma, Ltd., Hampton Bays, NY 11946, 3 Rolling Hill Rd., United States; email: g.konesky@att.net; CODEN: PSISD
ER -

TY - CONF
AU - Hellweg, C.E.
AU - Gerzer, R.
AU - Koch, B.
AU - Boese, A.
AU - Reitz, G.
TI - The Helmholtz Space Life Sciences Research School (SpaceLife): A new perspective in promotion of young researchers
PY - 2009
T2 - 60th International Astronautical Congress 2009, IAC 2009
VL - 1
SP - 419
EP - 426
UR - https://www.scopus.com/inward/record.uri?eid=2-s2.0-77953644266&partnerID=40&md5=38bb4fc80518b807c7e58d5041c71600
AD - German Aerospace Center (DLR), Institute of Aerospace Medicine, Radiobiology, Cologne, Germany
AD - German Aerospace Center (DLR), Institute of Aerospace Medicine, Cologne, Germany
AD - German Aerospace Center (DLR), Cologne, Germany
AB - In the field of space life sciences, the demand of an interdisciplinary and specific training of young researchers is high due to the complex interaction of medical, biological, physical, technical and other questions. The Helmholtz Space Life Sciences Research School (SpaceLife) offers an excellent interdisciplinary training for doctoral students from different fields (biology, biochemistry, biotechnology, physics, psychology, nutrition or sports sciences and related fields) and any country. SpaceLife is

coordinated by the Institute of Aerospace Medicine at the German Aerospace Center (DLR) in Cologne. The German universities in Kiel, Bonn, Aachen, Regensburg, Magdeburg and Berlin, and the German Sports University (DSHS) in Cologne are members of SpaceLife. The universities of Erlangen-Nürnberg, Frankfurt, Hohenheim, and the Beihang University in Beijing are associated partners. In each generation, up to 25 students can participate in the three-year program. Students will learn to develop integrated concepts to solve health issues in human spaceflight and in related disease patterns on Earth, and to further explore the requirements for life in extreme environments, enabling a better understanding of the ecosystem Earth and the search for life on other planets in unmanned and manned missions. The doctoral candidates are coached by two specialist supervisors from DLR and the partner university, and a mentor. All students attend lectures in different subfields of space life sciences to attain an overview of the field: radiation and gravitational biology, astrobiology and space physiology, including psychological aspects of short and long term space missions. Seminars, advanced lectures, laboratory courses and stays at labs at the partner institutions or abroad are offered as elective course and will provide in-depth knowledge of the chosen subfield or will allow to appropriate innovative methods. In Journal Clubs of the participating working groups, doctoral students will learn critical reading of scientific literature, first steps in peer review, scientific writing during preparation of their own publication, and writing of the thesis. The training of soft skills will be offered as block course in cooperation with other Helmholtz Research Schools. The whole program encompasses 303 hours and is organized in semester terms. The first doctoral candidates started the program in spring 2009.
KW - Astrobiology
KW - Aviation medicine
KW - Biochemistry
KW - Coordination reactions
KW - Helmholtz equation
KW - Mobile telecommunication systems
KW - Planets
KW - Research
KW - Societies and institutions
KW - Space flight
KW - Students
KW - Technical writing
KW - Aerospace medicine
KW - Complex interaction
KW - Disease patterns
KW - Elective course
KW - Extreme environment
KW - German aerospace centers
KW - Health issues
KW - Helmholtz
KW - Human spaceflights
KW - In-depth knowledge
KW - Innovative method
KW - Interdisciplinary training

KW - Laboratory course
KW - Long term
KW - Manned missions
KW - Partner institutions
KW - Peer review
KW - Scientific literature
KW - Scientific writing
KW - Search for life
KW - Soft skills
KW - Space life science
KW - Space missions
KW - Space physiology
KW - Subfields
KW - Working groups
KW - Personnel training
SN - 978-161567908-9 (ISBN)
LA - English
J2 - Int. Astronaut. Cong., IAC
M3 - Conference paper
DB - Scopus
N1 - Export Date: 23 February 2023; Cited By: 0; Correspondence Address: C. E. Hellweg; German Aerospace Center (DLR), Institute of Aerospace Medicine, Radiobiology, Cologne, Germany; email: christine.hellweg@dlr.de
ER -

TY - JOUR
AU - Groemer, G.E.
TI - AustroMars and PolAres: Measuring forward contamination during Mars-analogue missions
PY - 2009
T2 - Planetary and Space Science
VL - 57
IS - 5-6
SP - 660
EP - 663
DO - 10.1016/j.pss.2008.07.021
UR - https://www.scopus.com/inward/record.uri?eid=2-s2.0-67349169024&doi=10.1016%2fj.pss.2008.07.021&partnerID=40&md5=1a4a2786c9c44b3cde683bb91c970c09
AD - Austrian Space Forum, Institute of Astro- and Particle Physics, University of Innsbruck, 6020 Innsbruck, Technikerstr. 25/8, Austria
AB - The search for life on Mars is one of the most ambitious scientific challenges in astrobiology. Although the surface of the planet is generally believed to be hostile to life, habitats embedded in the subsurface water repositories might harbor diminutive traces of extant or even extant life requiring advanced analytical capabilities. At the same time, hardware (including a human crew) delivered from Earth introduces the inherent risk of contaminating the very samples to be investigated for tracers of life and as such might compromise the pristinity of the sample leading to a false-positive detection. Deep subsurface drilling activities are considered to be one of the most promising sampling

techniques, but require extensive infrastructure and technologies yet to be developed and increase the probability of such a forward contamination. AustroMars and PolAres are two Mars-analogue research programmes conducted by the Austrian Space Forum to investigate the contamination vectors of human Mars surface activities. Using fluorescent microspherules and non-pathogenic bacteria, an analysis strategy to quantify the bioload transfer rates and its field application in a Mars-analogue setting is described. © 2008 Elsevier Ltd. All rights reserved.
KW - Astrobiology
KW - Bacteria
KW - Martian surface analysis
KW - Research laboratories
KW - Drilling activities
KW - Field applications
KW - Forward contamination
KW - Human crews
KW - Human mission
KW - Inherent risks
KW - Mars exploration
KW - Mars-analogue research
KW - Pathogenic bacteria
KW - Research programmes
KW - Sampling techniques
KW - Search for lives
KW - Subsurface waters
KW - Surface activities
KW - Transfer rates
KW - Contamination
KW - Forward contamination
KW - Human mission
KW - Mars exploration
KW - Mars-analogue research
SN - 00320633 (ISSN)
LA - English
J2 - Planet. Space Sci.
M3 - Article
DB - Scopus
N1 - Export Date: 23 February 2023; Cited By: 4; Correspondence Address: G.E. Groemer; Austrian Space Forum, Institute of Astro- and Particle Physics, University of Innsbruck, 6020 Innsbruck, Technikerstr. 25/8, Austria; email: gernot.groemer@oewf.org; CODEN: PLSSA
ER -

TY - JOUR
AU - Noffke, N.
TI - Journal club. An astrobiologist considers life's oldest oxygen.
PY - 2009
T2 - Nature
VL - 457
IS - 7232
SP - 939

DO - 10.1038/457939e
UR - https://www.scopus.com/inward/record.uri?eid=2-s2.0-62749159405&doi=10.1038%2f457939e&partnerID=40&md5=27f8a33b1aa1133373e9d6e7a77fed49
AD - Old Dominion University, Virginia, Norfolk, United States
KW - Atmosphere
KW - Biogenesis
KW - Biological Markers
KW - Cyanobacteria
KW - Eukaryotic Cells
KW - Evolution, Chemical
KW - Exobiology
KW - Fossils
KW - Oceans and Seas
KW - Oxygen
KW - South Africa
KW - biological marker
KW - oxygen
KW - MLCS
KW - MLOWN
KW - article
KW - astronomy
KW - atmosphere
KW - biogenesis
KW - biology
KW - chemistry
KW - Cyanobacterium
KW - eukaryotic cell
KW - fossil
KW - metabolism
KW - sea
KW - South Africa
SN - 14764687 (ISSN)
C2 - 19225480
LA - English
J2 - Nature
M3 - Article
DB - Scopus
N1 - Export Date: 23 February 2023; Cited By: 0
ER -

TY - CHAP
AU - Velasco, E.
AU - Caravantes, G.
AU - Gómez-Izquierdo, B.
AU - Rodriguez, A.
AU - Ruiz, J.
TI - Europa: New horizons for astrobiology
PY - 2009
T2 - Space Exploration Research
SP - 519
EP - 529
UR - https://www.scopus.com/inward/record.uri?eid=2-s2.0-84892125211&partnerID=40&md5=654330f099abff8bf4cc339514038679

AD - Centro de Biología Molecular, CSIC-Universidad Autónoma de Madrid, Cantoblanco, Madrid, 28049, Spain
AD - Volcano Dynamics Group, The Open University, MK7 6AA, United Kingdom
AD - Seminario de Ciencias Planetarias, Facultad de Ciencias Geológicas, Universidad Complutense de Madrid, Madrid, 28040, Spain
AB - Europa is the smallest of the Galilean satellites of Jupiter, although it is however a remarkable body and the primary focus of astrobiological interest in the Jovian system. Geological and magnetic evidences, as well as theoretical considerations, strongly suggest that Europa has a thin ice shell, maybe a few kilometers or a few tens of kilometers thick, which floats on an internal ocean of liquid water. Some anomalies have been observed in the huge magnetic field of Jupiter whenever Europa goes through it, which seems to indicate that this ocean is made of conductive water, due to a probable high content of salts. Moreover,the low density of impact craters suggest an surface age of about 60 Myr in average, which is very young in geological terms, indicating that internal activity, driven by tidal heating, is resurfacing the moon at the present time. So, Europa shows an overprint of crisscrossing ridges and linear markings, some extending for thousand of kilometers, which are locally disrupted for chaotic terrains, building a colorful and exotic textured surface. All of these features make of Europa a theoretically good niche for life. Biological organisms might exist in the salty internal ocean, in the icy shell (active or latent) at places such as fractures, veins or liquid reservoirs, on the internal sea floor in hydrothermal vents or submarine volcanoes, or even within the rocky interior. © 2009 by Nova Science Publishers, Inc. All rights reserved.
KW - Geology
KW - Hot springs
KW - Reservoirs (water)
KW - Textures
KW - Biological organisms
KW - Chaotic terrain
KW - Conductive water
KW - Galilean satellites
KW - Hydrothermal vent
KW - Internal activity
KW - Liquid reservoirs
KW - Textured surface
KW - Satellites
PB - Nova Science Publishers, Inc.
SN - 978-161668212-5 (ISBN); 978-160692264-4 (ISBN)
LA - English
J2 - Space Explor. Res.
M3 - Book chapter
DB - Scopus
N1 - Export Date: 23 February 2023; Cited By: 0
ER -

TY - JOUR
AU - Blank, C.E.
TI - Phylogenomic dating - The relative antiquity of archaeal

metabolic and physiological traits
PY - 2009
T2 - Astrobiology
VL - 9
IS - 2
SP - 193
EP - 219
DO - 10.1089/ast.2008.0248
UR - https://www.scopus.com/inward/record.uri?eid=2-s2.0-65249127932&doi=10.1089%2fast.2008.0248&partnerID=40&md5=2021232d2ee7284e9c7099e994809685
AD - Department of Geosciences, University of Montana, Missoula, MT, United States
AD - Department of Geosciences, University of Montana, Missoula, MT 59808-1296, 32 Campus Drive #1296, United States
AB - Ancestral trait reconstruction was used to identify the relative ancestry of metabolic and physiological traits in the archaeal domain of life. First, well-resolved phylogenetic trees were inferred with multiple gene sequences obtained from whole genome sequences. Next, metabolic and physiological traits were coded into characters, and ancestral state reconstruction was used to identify ancient and derived traits. Traits inferred to be ancient included sulfur reduction, methanogenesis, and hydrogen oxidation. By using the articulation of the "oxygen age constraint," several other traits were inferred to have arisen at or after 2.32 Ga: aerobic respiration, nitrate reduction, sulfate reduction, thiosulfate reduction, sulfur oxidation, and sulfide oxidation. Complex organic metabolism appeared to be nearly as ancient as autotrophy. Hyperthermophily was ancestral, while hyperacidophily and extreme halophily likely arose after 2.32 Ga. The ancestral euryarchaeote was inferred to have been a hyperthermophilic marine methanogen that lived in a deep-sea hydrothermal vent. In contrast, the ancestral crenarchaeote was most likely a hyperthermophilic sulfur reducer that lived in a slightly acidic terrestrial environment, perhaps a fumarole. Cross-colonization of these habitats may not have occurred until after 2.32 Ga, which suggests that both archaeal lineages exhibited niche specialization on early Earth for a protracted period of time. Astrobiology 9, 193-219. © 2009 Mary Ann Liebert, Inc. 2009.
KW - Aerobiosis
KW - Anaerobiosis
KW - Archaea
KW - Autotrophic Processes
KW - Carbon
KW - Crenarchaeota
KW - Ecosystem
KW - Euryarchaeota
KW - Evolution
KW - Heterotrophic Processes
KW - Hydrogen
KW - Hydrogen Sulfide
KW - Hydrogen-Ion Concentration
KW - Iron
KW - Methane

KW - Nanoarchaeota
KW - Nitrates
KW - Oxidation−Reduction
KW - Phylogeny
KW - Sulfates
KW - Sulfides
KW - Sulfites
KW - Sulfur
KW - Temperature
KW - Thiosulfates
KW - Archaea
KW - Crenarchaeota
KW - Euryarchaeota
KW - carbon
KW - hydrogen
KW - hydrogen sulfide
KW - iron
KW - methane
KW - nitrate
KW - sulfate
KW - sulfide
KW - sulfite
KW - sulfur
KW - thiosulfate
KW - aerobic metabolism
KW - anaerobic growth
KW - Archaebacterium
KW - article
KW - autotrophy
KW - chemistry
KW - classification
KW - Crenarchaeota
KW - ecosystem
KW - Euryarchaeota
KW - evolution
KW - genetics
KW - heterotrophy
KW - metabolism
KW - Nanoarchaeota
KW - oxidation reduction reaction
KW - pH
KW - phylogeny
KW - temperature
KW - Archaea
KW - Metabolism
KW - Microbial evolution
KW - Phylogenetics
KW - Phylogenomic dating
KW - Precambrian
KW - Proterozoic
KW - Rise in oxygen
SN - 15311074 (ISSN)
C2 - 19371161
LA - English

J2 - Astrobiology
M3 - Article
DB - Scopus
N1 - Export Date: 23 February 2023; Cited By: 22; Correspondence Address: C. E. Blank; Department of Geosciences, University of Montana, Missoula, MT 59808-1296, 32 Campus Drive #1296, United States; email: carrine.blank@umontana.edu
ER -

TY - CHAP
AU - Davenport, A.A.
TI - Baroque fire (A note on early-modern angelology)
PY - 2009
T2 - Evidence and Interpretation in Studies on Early Science and Medicine
SP - 369
EP - 397
DO - 10.1163/ej.9789004178786.i-458
UR - https://www.scopus.com/inward/record.uri?eid=2-s2.0-84882728245&doi=10.1163%2fej.9789004178786.i-458&partnerID=40&md5=3f0d0fa65b346eb2f752d2e16be6d262
AD - Boston College, Concord, MA 01742, 96 Bedford Street, United States
AB - Between Galileo's discovery of the moons of Jupiter (1610) and the publication of Newton's Principia (1687), uncertainty regarding the structure of the heavens combined with a lyrical fascination for extraterrestrial life inspired a distinctly Baroque outpouring of speculation in which angels played a key part. English Catholic "recusants," haunted by a feeling of lost unity, vividly illustrate the imaginative character of Baroque speculation. © 2009 by Koninklijke Brill NV, Leiden, The Netherlands. All rights reserved.
KW - angels
KW - Antoine Legrand
KW - Franciscus à Sancta Clara
KW - Kenelm Digby
KW - meteorology
KW - planetary atmosphere
KW - Robert Boyle
KW - Thomas White
PB - Brill
SN - 978-900417878-6 (ISBN)
LA - English
J2 - Evidence and Interpret. in Stud. on Early Sci. and Medicine
M3 - Book chapter
DB - Scopus
N1 - Export Date: 23 February 2023; Cited By: 0; Correspondence Address: A.A. Davenport; Boston College, Concord, MA 01742, 96 Bedford Street, United States; email: adavenport@cfa.harvard.edu
ER -

TY - JOUR
AU - Izenberg, N.R.
AU - Murrray, G.M.
AU - Pilato, R.S.

AU - Baird, L.M.
AU - Levin, S.M.
AU - Van Houten, K.A.
TI - Astrobiological molecularly imprinted polymer sensors
PY - 2009
T2 - Planetary and Space Science
VL - 57
IS - 7
SP - 846
EP - 853
DO - 10.1016/j.pss.2009.02.015
UR - https://www.scopus.com/inward/record.uri?eid=2-s2.0-67349205140&doi=10.1016%2fj.pss.2009.02.015&partnerID=40&md5=4c745168dcd344e824d74c4c929ef1b4
AD - The Johns Hopkins University Applied Physics Laboratory, Laurel, MD 20723-6099, 11100 Johns Hopkins Road, United States
AB - The purpose of the Astrobiological MIP Sensor (AMS) Project is to develop reliable, low-cost, low-mass, low-power consumption detection technologies for in situ analysis of biochemical markers, and other indicators of astrobiological importance. To this end, we are investigating the potential role that molecularly imprinted polymers (MIP) could serve in the recognition of pre-biotic and biotic compounds in planetary, astrobiological and geochemical mission profiles. While MIPs are effective molecular recognition tools, a signal transduction method must be developed so that the recognition of analytes can be realized. In the course of this study, surface plasmon resonance (SPR) will be the detection method of the MIP recognition event. In addition, MIP-coated SPR substrates were subjected to vibration, temperature and radiation testing to demonstrate that they could withstand the rigors of space travel. The methods developed in this study require capture of the biomarkers onto the SPR sensor chip, followed by addition of a MIP. It is the binding of the MIP to the SPR bound analyte that amplifies the SPR signal associated with binding of the low molecular weight analyte. The MIPs, developed in this study are water-soluble processable star polymers while the SPR device used was SensíQ™ by Nomatics. Proof-of-principal experiments were first demonstrated using amino biotin.
KW - Amines
KW - Analytical geochemistry
KW - Molecular biology
KW - Molecular recognition
KW - Plasma waves
KW - Plasmons
KW - Polymers
KW - Sensors
KW - Signal transduction
KW - Analyte
KW - Analytes
KW - Biochemical markers
KW - Detection methods
KW - Detection technologies
KW - In-situ analysis
KW - Low mass

KW - Low molecular weights
KW - Low-power consumption
KW - Mip sensors
KW - Mission profiles
KW - Molecularly imprinted polymer
KW - Molecularly imprinted polymer sensors
KW - Origin of life
KW - Processable
KW - Radiation testing
KW - Space travels
KW - Spr sensor chips
KW - Spr signals
KW - Star polymers
KW - Surface plasmon resonance
KW - Molecularly imprinted polymer
KW - Origin of life
KW - Surface plasmon resonance
SN - 00320633 (ISSN)
LA - English
J2 - Planet. Space Sci.
M3 - Article
DB - Scopus
N1 - Export Date: 23 February 2023; Cited By: 14; Correspondence Address: K.A. Van Houten; The Johns Hopkins University Applied Physics Laboratory, Laurel, MD 20723-6099, 11100 Johns Hopkins Road, United States; email: kelly.van.houten@jhuapl.edu; CODEN: PLSSA
ER -

TY - JOUR
AU - Niederberger, T.D.
AU - Perreault, N.N.
AU - Lawrence, J.R.
AU - Nadeau, J.L.
AU - Mielke, R.E.
AU - Greer, C.W.
AU - Andersen, D.T.
AU - Whyte, L.G.
TI - Novel sulfur-oxidizing streamers thriving in perennial cold saline springs of the Canadian high Arctic
PY - 2009
T2 - Environmental Microbiology
VL - 11
IS - 3
SP - 616
EP - 629
DO - 10.1111/j.1462-2920.2008.01833.x
UR - https://www.scopus.com/inward/record.uri?eid=2-s2.0-61449156448&doi=10.1111%2fj.1462-2920.2008.01833.x&partnerID=40&md5=2f227c985b4877e170ea4ebb73f97061
AD - Department of Natural Resource Sciences, McGill University, Ste-Anne-de-Bellevue, QC H9X 3V9, 21, 111 Lakeshore Rd., Canada
AD - National Research Council Canada, Biotechnology Research Institute, Montreal, Canada
AD - Environment Canada, 11 Innovation Blvd., Saskatoon, SK, Canada

AD - Department of Biomedical Engineering, McGill University, Montreal, QC H3A 2B4, Canada
AD - Jet Propulsion Laboratory, California Institute of Technology, Pasadena, CA 91109, 4800 Oak Grove Dr., United States
AD - Carl Sagan Center, Mountain View, CA, United States
AB - Summary: The perennial springs at Gypsum Hill (GH) and Colour Peak (CP), situated at nearly 80°N on Axel Heiberg Island in the Canadian high Arctic, are one of the few known examples of cold springs in thick permafrost on Earth. The springs emanate from deep saline aquifers and discharge cold anoxic brines rich in both sulfide and sulfate. Grey-coloured microbial streamers form during the winter months in snow-covered regions of the GH spring run-off channels (−1.3°C to 6.9°C, ~7.5% NaCl, 0–20 p.p.m. dissolved sulfide, 1 p.p.m. dissolved oxygen) but disappear during the Arctic summer. Culture- and molecular-based analyses of the 16S rRNA gene (FISH, DGGE and clone libraries) indicated that the streamers were uniquely dominated by chemolithoautotrophic sulfur-oxidizing Thiomicrospira species. The streamers oxidized both sulfide and thiosulfate and fixed CO 2 under in situ conditions and a Thiomicrospira strain isolated from the streamers also actively oxidized sulfide and thiosulfate and fixed CO 2 under cold, saline conditions. Overall, the snow-covered spring channels appear to represent a unique polar saline microhabitat that protects and allows Thiomicrospira streamers to form and flourish via chemolithoautrophic, phototrophic-independent metabolism in a high Arctic winter environment characterized by air temperatures commonly below −40°C and with an annual average air temperature of −15°C. These results broaden our knowledge of the physical and chemical boundaries that define life on Earth and have astrobiological implications for the possibility of life existing under similar Martian conditions. © 2008 Society for Applied Microbiology and Blackwell Publishing Ltd.
KW - Arctic Regions
KW - Canada
KW - Carbon Dioxide
KW - Cluster Analysis
KW - DNA Fingerprinting
KW - DNA, Bacterial
KW - DNA, Ribosomal
KW - Genes, rRNA
KW - In Situ Hybridization, Fluorescence
KW - Molecular Sequence Data
KW - Oxidation-Reduction
KW - Phylogeny
KW - Piscirickettsiaceae
KW - RNA, Bacterial
KW - RNA, Ribosomal, 16S
KW - Sequence Analysis, DNA
KW - Sequence Homology, Nucleic Acid
KW - Sulfur
KW - Water Microbiology
KW - Thiomicrospira
KW - bacterial DNA
KW - bacterial RNA

KW - carbon dioxide
KW - ribosome DNA
KW - RNA 16S
KW - sulfur
KW - Arctic
KW - article
KW - Canada
KW - chemistry
KW - classification
KW - cluster analysis
KW - DNA fingerprinting
KW - DNA sequence
KW - fluorescence in situ hybridization
KW - genetics
KW - isolation and purification
KW - metabolism
KW - microbiology
KW - molecular genetics
KW - nucleotide sequence
KW - oxidation reduction reaction
KW - phylogeny
KW - Piscirickettsiaceae
KW - RNA gene
KW - sequence homology
SN - 14622920 (ISSN)
C2 - 19278448
LA - English
J2 - Environ. Microbiol.
M3 - Article
DB - Scopus
N1 - Export Date: 23 February 2023; Cited By: 29; Correspondence Address: L. G. Whyte; Department of Natural Resource Sciences, McGill University, Ste-Anne-de-Bellevue, QC H9X 3V9, 21, 111 Lakeshore Rd., Canada; email: lyle.whyte@mcgill.ca; CODEN: ENMIF
ER -

TY - JOUR
AU - Davenport, A.A.
TI - Baroque fire (A note on early-modern angelology)
PY - 2009
T2 - Early Science and Medicine
VL - 14
IS - 1
SP - 369
EP - 397
DO - 10.1163/157338209X425623
UR - https://www.scopus.com/inward/record.uri?eid=2-s2.0-67649616004&doi=10.1163%2f157338209X425623&partnerID=40&md5=f75fdbaa7b4399c666a62e8e8a90d7fe
AD - Boston College, United States
AD - Concord, MA 01742, 96 Bedford Street, United States
AB - Between Galileo's discovery of the moons of Jupiter (1610) and the publication of Newton's Principia (1687), uncertainty regarding the structure of the heavens combined with a lyrical fascination for

extraterrestrial life inspired a distinctly Baroque outpouring of speculation in which angels played a key part. English Catholic "recusants," haunted by a feeling of lost unity, vividly illustrate the imaginative character of Baroque speculation. © 2009 Brill Academic Publishers.
KW - Angels
KW - Antoine Legrand
KW - Franciscus à: Sancta Clara
KW - Kenelm Digby
KW - Meteorology
KW - Planetary atmosphere
KW - Robert Boyle
KW - Thomas white
PB - Brill Academic Publishers
SN - 13837427 (ISSN)
LA - English
J2 - Early Sci. Med.
M3 - Article
DB - Scopus
N1 - Export Date: 23 February 2023; Cited By: 0; Correspondence Address: A. A. Davenport; Concord, MA 01742, 96 Bedford Street, United States; email: adavenport@cfa.harvard.edu
ER -

TY - JOUR
AU - Lepper, K.
TI - The effect of evaporated salt solutions on the optical dating properties of jsc mars-1: "seasoning" for a mars soil simulant
PY - 2009
T2 - Astrobiology
VL - 9
IS - 6
SP - 531
EP - 534
DO - 10.1089/ast.2008.0307
UR - https://www.scopus.com/inward/record.uri?eid=2-s2.0-69149103479&doi=10.1089%2fast.2008.0307&partnerID=40&md5=4c71a9c09c14b82e151090b636b2d50e
AD - Optical Dating and Dosimetry Lab, Department of Geosciences, North Dakota State University, Fargo, ND 58105, United States
AB - Optically stimulated luminescence dating, or optical dating, is an established terrestrial geochronometric technique that is being adapted to date sedimentary deposits and landforms on the surface of Mars. Recent discoveries have highlighted the astrobiological significance and occurrence of halite on the surface of Mars. The objective of the experiments in this study was to create a simplistic analogue of the sedimentary material that would result from evaporation of ion-containing pore water out of martian regolith and evaluate the influence the evaporated salts would have on in situ optical dating of silicate sediments. The radiation dose response, as measured by infrared stimulated luminescence (IRSL), from evaporated mixtures of JSC Mars-1 and solutions of sodium chloride and calcium sulfate was documented. The results suggest that the presence of CaSO4 and NaCl within the aggregated particles

does not have adverse effects on IRSL dose response and that aggregates of this type exhibit dose response characteristics that are appropriate for optical dating. © Mary Ann Liebert, Inc.
KW - Calcium Sulfate
KW - Computer Simulation
KW - Extraterrestrial Environment
KW - Infrared Rays
KW - Mars
KW - Optical Phenomena
KW - Sodium Chloride
KW - Solutions
KW - Volatilization
KW - calcium sulfate
KW - sodium chloride
KW - article
KW - astronomy
KW - chemistry
KW - computer simulation
KW - infrared radiation
KW - light related phenomena
KW - microclimate
KW - solution and solubility
KW - volatilization
KW - Evaporites
KW - Halite
KW - Mars geochronology
KW - Optical dating
KW - OSL dating
SN - 15311074 (ISSN)
C2 - 19586394
LA - English
J2 - Astrobiology
M3 - Article
DB - Scopus
N1 - Export Date: 23 February 2023; Cited By: 0; Correspondence Address: K. Lepper; Optical Dating and Dosimetry Lab, Department of Geosciences, North Dakota State University, Fargo, ND 58105, United States; email: ken.lepper@ndsu.edu
ER -

TY - JOUR
AU - Navarro-González, R.
AU - Iñiguez, E.
AU - De La Rosa, J.
AU - McKay, C.P.
TI - Characterization of organics, microorganisms, desert soils, and mars-like soils by thermal volatilization coupled to mass spectrometry and their implications for the search for organics on mars by phoenix and future space missions
PY - 2009
T2 - Astrobiology
VL - 9
IS - 8
SP - 703

EP - 715
DO - 10.1089/ast.2008.0284
UR - https://www.scopus.com/inward/record.uri?eid=2-s2.0-70350334943&doi=10.1089%2fast.2008.0284&partnerID=40&md5=46038d326433e77964a2cd5c98d8bf73
AD - Laboratorio de Química de Plasmas y Estudios Planetarios, Universidad Nacional Autónoma de México, Ciudad Universitaria, Mexico
AD - Space Science Division, NASA Ames Research Center, Moffett Field, CA, United States
AB - A key goal for astrobiology is the search for evidence of life on Mars. Because liquid water is a fundamental environmental requirement for life, the recent set of missions to Mars have focused on a strategy known as "follow the water." Since life is made of organic molecules, a logical next step is "follow the organics." However, organics are expected to be present at very low levels on Mars, which would make their detection challenging. Viking was unable to detect organics at parts per billion (ppb), but the effective upper limit could be higher due to the low efficiency of the thermal volatilization (TV) step in releasing organics. Due to its ease of use, TV is still the method selected for current and future NASA and ESA missions. Here, we show that when organics are present in the soil at levels above 1500 parts per million (ppm), there are several characteristic organic fragments detected by TV-mass spectrometry; however, when the levels are below <150 ppm, TV oxidizes them, and no organic fragments are released. Instead, nitric oxide (NO) is produced and can be used to determine quantitatively the organic content if the C/N ratio is determined. Any atmospheric NO sorbed or mineral nitrogen (e.g., nitrates) present in the soil would release NO by TV at distinctive temperature regimes that would not overlap with the organic nitrogen source. Therefore, we suggest that monitoring NO provides the best chance for Phoenix and other future Mars missions to detect nitrogen-containing organics in the soil or ice. © Copyright 2009 Mary Ann Liebert, Inc.
KW - Exobiology
KW - Mars
KW - Mass Spectrometry
KW - Organic Chemicals
KW - Oxidation-Reduction
KW - Soil
KW - Space Flight
KW - Temperature
KW - Volatilization
KW - organic compound
KW - article
KW - astronomy
KW - biology
KW - instrumentation
KW - mass spectrometry
KW - methodology
KW - oxidation reduction reaction
KW - soil
KW - space flight

KW - temperature
KW - volatilization
KW - Astrobiology
KW - Mars
KW - Mass spectrometry
KW - Organic matter
KW - Planetary instrumentation
SN - 15311074 (ISSN)
C2 - 19845443
LA - English
J2 - Astrobiology
M3 - Article
DB - Scopus
N1 - Export Date: 23 February 2023; Cited By: 34; Correspondence Address: R. Navarro-González; Laboratorio de Química de Plasmas y Estudios Planetarios, Universidad Nacional Autónoma de México, Ciudad Universitaria, México D.F. 04510, Apartado Postal 70-543, Mexico; email: navarro@nucleares.unam.mx
ER -

TY - JOUR
AU - Moskovitz, N.A.
AU - Gaidos, E.
AU - Williams, D.M.
TI - The effect of lunarlike satellites on the orbital infrared light curves of Earth-analog planets
PY - 2009
T2 - Astrobiology
VL - 9
IS - 3
SP - 269
EP - 277
DO - 10.1089/ast.2007.0209
UR - https://www.scopus.com/inward/record.uri?eid=2-s2.0-68249152650&doi=10.1089%2fast.2007.0209&partnerID=40&md5=9e0c6f6d4e3e77288ba053a4a473a7cd
AD - Institute for Astronomy, University of Hawaii at Manoa, Honolulu, HI, United States
AD - Department of Geology and Geophysics, University of Hawaii at Manoa, Honolulu, HI, United States
AD - School of Science, Penn State Erie, Behrend College, Erie, PA, United States
AD - Institute for Astronomy, University of Hawaii at Manoa, Honolulu, HI 96822, 2680 Woodlawn Drive, United States
AB - We have investigated the influence of lunarlike satellites on the infrared orbital light curves of Earth-analog extrasolar planets. Such light curves will be obtained by NASA's Terrestrial Planet Finder (TPF) and ESA's Darwin missions as a consequence of repeat observations to confirm the companion status of a putative planet and determine its orbit. We used an energy balance model to calculate disk-averaged infrared (bolometric) fluxes from planet-satellite systems over a full orbital period (one year). The satellites are assumed to lack an atmosphere, have a low thermal inertia like that of the Moon, and span a range of plausible radii.

The planets are assumed to have thermal and orbital properties that mimic those of Earth, while their obliquities and orbital longitudes of inferior conjunction remain free parameters. Even if the gross thermal properties of the planet can be independently constrained (e.g., via spectroscopy or visible-wavelength detection of specular glint from a surface ocean), only the largest (~Mars-sized) lunarlike satellites can be detected by light curve data from a TPF-like instrument (i.e., one that achieves a photometric signal-to-noise ratio of 10 to 20 at infrared wavelengths). Nondetection of a lunarlike satellite can obfuscate the interpretation of a given system's infrared light curve so that it may resemble a single planet with high obliquity, different orbital longitude of vernal equinox relative to inferior conjunction, and in some cases drastically different thermal characteristics. If the thermal properties of the planet are not independently established, then the presence of a lunarlike satellite cannot be inferred from infrared data, which would thus demonstrate that photometric light curves alone can only be used for preliminary study, and the addition of spectroscopic data will be necessary. © Mary Ann Liebert, Inc.
KW - Atmosphere
KW - Earth (Planet)
KW - Exobiology
KW - Extraterrestrial Environment
KW - Light
KW - Models, Theoretical
KW - Moon
KW - Planets
KW - Seasons
KW - Spectroscopy, Near-Infrared
KW - Time Factors
KW - article
KW - astronomy
KW - atmosphere
KW - biology
KW - light
KW - methodology
KW - microclimate
KW - moon
KW - near infrared spectroscopy
KW - season
KW - theoretical model
KW - time
KW - Astrobiology
KW - Methods: data analysis
KW - Planetary systems
KW - Planets and satellites: general
SN - 15311074 (ISSN)
C2 - 19400731
LA - English
J2 - Astrobiology
M3 - Article
DB - Scopus
N1 - Export Date: 23 February 2023; Cited By: 25; Correspondence
Address: N. A. Moskovitz; Institute for Astronomy, University of

Hawaii at Manoa, Honolulu, HI 96822, 2680 Woodlawn Drive, United States; email: nmosko@ifa.hawaii.edu
ER -

TY - CONF
AU - Levin, G.V.
AU - Straat, P.A.
TI - Methane and life on Mars
PY - 2009
T2 - Proceedings of SPIE - The International Society for Optical Engineering
VL - 7441
C7 - 74410D
DO - 10.1117/12.829183
UR - https://www.scopus.com/inward/record.uri?eid=2-s2.0-70449466825&doi=10.1117%2f12.829183&partnerID=40&md5=c7af1a3246d1c0f58d1a4f692d8bbbcb
AD - National Institutes of Health, Bethesda, MD, 20892, United States
AD - Sykesville, MD, 21784, 380 Windy Knoll, United States
AD - Beyond Center, College of Liberal Arts and Sciences, Arizona State University, Tempe, AZ, 85287, United States
AB - Mumma et al. 1 have confirmed earlier detections of methane in the Martian atmosphere, finding it localized and correlated with atmospheric water vapor. They determined that, because of the short half-life of methane, a continual replenishment is required to account for its presence. They also conclude that the dynamics of methane on Mars require a methane sink in the soil. It is suggested here that both phenomenon could be accounted for by an ecology of methane-producing and methane-consuming microorganisms. Such ecologies exist on Earth, where, generally, anaerobic methanogens live at depth and aerobic methanotrophs live at or near the surface. On Mars, with its essentially anaerobic atmosphere, both types of microorganisms could co-exist at or near the surface. It is possible that the Viking Labeled Release (LR) experiment detected methanogens in addition to other microorganisms evolving carbon dioxide since the LR instrumentation would detect methane, carbon dioxide, or any other carbon gas derived from one of the LR substrates. A simple modification of the LR experiment that could resolve the life on Mars issue is discussed. © 2009 SPIE.
KW - Astrobiology
KW - Carbon dioxide
KW - Electric reactors
KW - Leakage (fluid)
KW - Methanogens
KW - Microbiology
KW - Water vapor
KW - Anaerobic atmosphere
KW - Atmospheric water vapor
KW - Life on Mars
KW - Martian atmospheres
KW - Methane on Mars
KW - Methanotrophs
KW - Simple modifications

KW - Sink-in
KW - Methane
SN - 0277786X (ISSN); 978-081947731-6 (ISBN)
LA - English
J2 - Proc SPIE Int Soc Opt Eng
M3 - Conference paper
DB - Scopus
N1 - Export Date: 23 February 2023; Cited By: 7; Correspondence Address: G. V. Levin; Beyond Center, College of Liberal Arts and Sciences, Arizona State University, Tempe, AZ, 85287, United States; email: gilbert.levin@asu.edu; CODEN: PSISD
ER -

TY - JOUR
AU - Dartnell, L.R.
AU - Burchell, M.J.
TI - Survey on astrobiology research and teaching activities within the United Kingdom
PY - 2009
T2 - Astrobiology
VL - 9
IS - 8
SP - 717
EP - 730
DO - 10.1089/ast.2009.0348
UR - https://www.scopus.com/inward/record.uri?eid=2-s2.0-70350334944&doi=10.1089%2fast.2009.0348&partnerID=40&md5=bd4728b0b09cbf834329d1f6b7731d53
AD - CoMPLEX (Centre for Mathematics and Physics in the Life Sciences and Experimental Biology), University College London, London, United Kingdom
AD - Centre for Planetary Sciences at UCL/Birkbeck, University College London, London, United Kingdom
AD - School of Physical Sciences, University of Kent, Canterbury, Kent, United Kingdom
AB - While astrobiology is apparently growing steadily around the world, in terms of the number of researchers drawn into this interdisciplinary area and teaching courses provided for new students, there have been very few studies conducted to chart this expansion quantitatively. To address this deficiency, the Astrobiology Society of Britain (ASB) conducted a questionnaire survey of universities and research institutions nationwide to ascertain the current extent of astrobiology research and teaching in the UK. The aim was to provide compiled statistics and an information resource for those who seek research groups or courses of study, and to facilitate new interdisciplinary collaborations. The report here summarizes details gathered on 33 UK research groups, which involved 286 researchers (from undergraduate project students to faculty members). The survey indicates that around 880 students are taking university-level courses, with significant elements of astrobiology included, every year in the UK. Data are also presented on the composition of astrobiology students by their original academic field, which show a significant dominance of physics and astronomy students. This survey represents the first

published systematic national assessment of astrobiological academic activity and indicates that this emerging field has already achieved a strong degree of penetration into the UK academic community. © Copyright 2009 Mary Ann Liebert, Inc.
KW - Data Collection
KW - Exobiology
KW - Great Britain
KW - Humans
KW - Research
KW - Societies, Scientific
KW - Teaching
KW - article
KW - biology
KW - health care organization
KW - human
KW - information processing
KW - research
KW - teaching
KW - United Kingdom
KW - Astrobiology Society of Britain (ASB)
KW - Research
KW - Teaching
KW - United Kingdom
SN - 15311074 (ISSN)
C2 - 19845444
LA - English
J2 - Astrobiology
M3 - Article
DB - Scopus
N1 - Export Date: 23 February 2023; Cited By: 7; Correspondence Address: L. R. Dartnell; Centre for Planetary Sciences at UCL/ Birkbeck, University College London, London WC1E 6BT, Gower Street, United Kingdom; email: l.dartnell@ucl.ac.uk
ER -

TY - CONF
AU - Kolb, V.M.
TI - Astrobiology and green chemistry: A new pedagogical connection
PY - 2009
T2 - Proceedings of SPIE - The International Society for Optical Engineering
VL - 7441
C7 - 74410V
DO - 10.1117/12.824164
UR - https://www.scopus.com/inward/record.uri?eid=2-s2.0-70449462266&doi=10.1117%2f12.824164&partnerID=40&md5=5dc1e904a5aa06b89e295b2a338a4c85
AD - Department of Chemistry, University of Wisconsin-Parkside, Kenosha, WI, 53141-2000, United States
AB - Various pedagogical approaches are needed to introduce astrobiology into the chemistry curriculum. We are developing a new approach in which we connect green chemistry with astrobiology. Green chemistry is chemistry which is environmentally friendly. One obvious way for the organic chemistry to be environmentally friendly

is to use water as solvent, instead of more toxic organic solvents. Another approach is to run so-called solventless reactions. For example, as the solid materials are mixed together, the melting point of the mixture is lower than the melting points of its individual components (the principle of the mixed-melting point). In some cases the entire mixture may melt upon mixing. The reactions would then occur in a viscous semi-solid state. An additional approach is to run the reactions by utilizing enzymes or man-made protein mimics as catalysts instead of toxic catalysts, such as those based on the transition metals. These and some other known examples of green chemistry have a great potential for astrobiology. The astrobiological reactions typically occur in water (e.g. prebiotic soup), in the solid mixtures (e.g. on the meteors), and may be catalyzed by various short peptides. The connection between the green chemistry principles and astrobiology represents a new pedagogical approach for infusion of astrobiology into the organic chemistry. 2009 SPIE.
KW - Amines
KW - Astrobiology
KW - Catalysis
KW - Catalysts
KW - Curricula
KW - Ionic liquids
KW - Ionization of liquids
KW - Melting point
KW - Meteorites
KW - Mixtures
KW - Organic solvents
KW - Proteins
KW - Smelting
KW - Solid state reactions
KW - Steelmaking
KW - Toxic materials
KW - Transition metals
KW - Green chemistry
KW - Meteoritic chemistry
KW - Organic reactions in water
KW - Phase-transfer catalysis
KW - Solventness reactions
KW - Chemistry
KW - Astrobiology
KW - Green chemistry
KW - Ionic liquids
KW - Meteoritic chemistry
KW - Organic reactions in water
KW - Phase-transfer catalysis
KW - Solventness reactions
SN - 0277786X (ISSN); 978-081947731-6 (ISBN)
LA - English
J2 - Proc SPIE Int Soc Opt Eng
M3 - Conference paper
DB - Scopus
N1 - Export Date: 23 February 2023; Cited By: 1; Correspondence
Address: V. M. Kolb; Department of Chemistry, University of

Wisconsin-Parkside, Kenosha, WI, 53141-2000, United States; email: kob@uwp.edu; CODEN: PSISD
ER -

TY - JOUR
AU - Vítek, P.
AU - Jehlička, J.
AU - Edwards, H.G.M.
AU - Osterrothová, K.
TI - Identification of β-carotene in an evaporitic matrix-evaluation of Raman spectroscopic analysis for astrobiological research on Mars
PY - 2009
T2 - Analytical and Bioanalytical Chemistry
VL - 393
IS - 8
SP - 1967
EP - 1975
DO - 10.1007/s00216-009-2677-0
UR - https://www.scopus.com/inward/record.uri?eid=2-s2.0-63449087686&doi=10.1007%2fs00216-009-2677-0&partnerID=40&md5=32702f8b0bb4348258abb76a0fbf6e02
AD - Institute of Geochemistry, Mineralogy and Mineral Resources, Charles University in Prague, 128 43 Prague 2, Albertov 6, Czech Republic
AD - School of Life Sciences, Division of Chemical and Forensic Sciences, University of Bradford, Bradford BD7 1DP, United Kingdom
AB - Since evaporitic rocks on the Martian surface could (or still can) serve as potential habitats for microbial life on Mars, there is a reasonable possibility that these rocks may sustain molecular remnants as evidence for the presence of extinct or extant living organisms on Mars and that β-carotene could be a suitable biomarker. In this paper, Raman microspectrometry was tested as a nondestructive method of determining the lowest detectable β-carotene content in experimentally prepared evaporitic matrices-namely, gypsum, halite and epsomite. Two excitation wavelengths were compared-514.5 nm, because of the resonance Raman enhancement in the carotenoid analysis, and 785 nm, as a more universal wavelength now much used in the detection of biomolecules terrestrially. Mixtures were measured directly as well as with a laser beam penetrating the crystals of gypsum and epsomite. We have obtained β-carotene signals at the 0.1 to 10 mg kg-1 level-the number of registered β-carotene Raman bands differed depending on the particular mineral matrix and the excitation wavelength. Concentrations of β-carotene of about one order of magnitude higher were identified when analysed through single crystals of gypsum and epsomite, respectively. © 2009 Springer-Verlag.
KW - beta Carotene
KW - Exobiology
KW - Extraterrestrial Environment
KW - Mars
KW - Reproducibility of Results
KW - Research
KW - Spectrum Analysis, Raman

KW - Astrobiology
KW - Biomarkers
KW - Gypsum
KW - Ocean habitats
KW - Raman scattering
KW - Raman spectroscopy
KW - Single crystals
KW - Sodium chloride
KW - Spectrum analysis
KW - Sulfate minerals
KW - beta carotene
KW - Detection of biomolecules
KW - Epsomite
KW - Evaporites
KW - Excitation wavelengths
KW - Living organisms
KW - Mars
KW - Martian surfaces
KW - matrixes
KW - Microbial lives
KW - Non-destructive methods
KW - Order of magnitudes
KW - Potential habitats
KW - Raman bands
KW - Raman microspectrometry
KW - Resonance raman
KW - article
KW - astronomy
KW - biology
KW - chemistry
KW - methodology
KW - microclimate
KW - Raman spectrometry
KW - reproducibility
KW - research
KW - Spectroscopic analysis
KW - β-Carotene
KW - Astrobiology
KW - Biomarkers
KW - Evaporites
KW - Mars
KW - Raman spectroscopy
SN - 16182650 (ISSN)
C2 - 19296093
LA - English
J2 - Anal. Bioanal. Chem.
M3 - Article
DB - Scopus
N1 - Export Date: 23 February 2023; Cited By: 57; Correspondence Address: J. Jehlička; Institute of Geochemistry, Mineralogy and Mineral Resources, Charles University in Prague, 128 43 Prague 2, Albertov 6, Czech Republic; email: jehlicka@natur.cuni.cz; CODEN: ABCNB
ER -

TY - CONF
AU - Storrie-Lombardi, M.C.
AU - Sattler, B.
TI - Laser-induced fluorescence emission (L.I.F.E.): In situ and remote detection of life in Antarctic and Alaskan ice
PY - 2009
T2 - Proceedings of SPIE - The International Society for Optical Engineering
VL - 7441
C7 - 744109
DO - 10.1117/12.831288
UR - https://www.scopus.com/inward/record.uri?eid=2-s2.0-70449462261&doi=10.1117%2f12.831288&partnerID=40&md5=7ace31ec4b35e1b37e3b15f8f46458f4
AD - Institute of Ecology, University of Innsbruck, 6020 Innsbruck, Technikerstrasse 25, Austria
AD - Kinohi Institute, Pasadena, CA 91101, 530 S. Lake Avenue, #117, United States
AB - Once thought to be a barren desert devoid of life, it now appears that Earth's cryosphere is an ice ecosystem harbouring a rich community of metabolically active microorganisms inhabiting ice, snow, water, and lithic environments. The ability to rapidly survey this ecosystem during in situ and orbital missions is of considerable interest for monitoring Earth's carbon budget and for efficiently searching for life on Mars or any exoplanet with an analogous cryosphere. Laser induced fluorescence emission (L.I.F.E.) imaging and spectroscopy using excitation in ultraviolet (UV) wavelengths have been proposed as non-destructive astrobiological survey tools to search for amino acids, nucleic acids, microbial life, and polycyclic aromatic hydrocarbons (PAHs) deep in the Mars regolith. However, the technique is easily adapted to search for larger, more complex biomolecular targets using longer wavelength sources. Of particular interest is the ability for excitation at blue, green, and red wavelengths to produce visible and near infrared fluorescence of photosynthetic pigments in cyanobacteria-dominated microbial communities populating the ice of alpine, Arctic, and Antarctic lakes, glaciers, ice sheets, and even the supercooled water-ice droplets of clouds. During the Tawani 2008 International Antarctic Expedition we tested the in situ use of the technique as part of a field campaign in the Dry Valleys of Schirmacher Oasis and Lake Untersee, Queen Maud Land, Antarctica. In the spring of 2009, we performed airborne remote sensing tests of the technology in Alaska. In this paper we review our in situ laser detection experiments and present for the first time preliminary results on our efforts to detect cryosphere L.I.F.E. from an airborne platform. © 2009 SPIE.
KW - Amines
KW - Amino acids
KW - Aromatic hydrocarbons
KW - Astrobiology
KW - Emission spectroscopy
KW - Fluorescence
KW - Glaciers

KW - Lakes
KW - Landforms
KW - Laser excitation
KW - Lasers
KW - Nucleic acids
KW - Optical radar
KW - Organic acids
KW - Polycyclic aromatic hydrocarbons
KW - Remote sensing
KW - Surveys
KW - Alaska
KW - Antarctica
KW - Arctic
KW - Epifluorescence
KW - LIDAR
KW - Mars
KW - Schirmacher oasis
KW - Ice
KW - Alaska
KW - Antarctica
KW - Arctic
KW - Epifluorescence
KW - Lake untersee
KW - LIDAR
KW - Mars
KW - Schirmacher oasis
SN - 0277786X (ISSN); 978-081947731-6 (ISBN)
LA - English
J2 - Proc SPIE Int Soc Opt Eng
M3 - Conference paper
DB - Scopus
N1 - Export Date: 23 February 2023; Cited By: 2; CODEN: PSISD
ER -

TY - JOUR
AU - Sittler Jr., E.C.
AU - Ali, A.
AU - Cooper, J.F.
AU - Hartle, R.E.
AU - Johnson, R.E.
AU - Coates, A.J.
AU - Young, D.T.
TI - Heavy ion formation in Titan's ionosphere: Magnetospheric introduction of free oxygen and a source of Titan's aerosols?
PY - 2009
T2 - Planetary and Space Science
VL - 57
IS - 13
SP - 1547
EP - 1557
DO - 10.1016/j.pss.2009.07.017
UR - https://www.scopus.com/inward/record.uri?eid=2-s2.0-70449523662&doi=10.1016%2fj.pss.2009.07.017&partnerID=40&md5=917352e296bf1a525c1b21e654ed9cfb

AD - NASA Goddard Space Flight Center, MD, United States
AD - University of Maryland, College Park, MD, United States
AD - University of Virginia, Charlottesville, VA, United States
AD - Mullard Space Science Laboratory, United Kingdom
AD - Southwest Research Institute, San Antonio, TX, United States
AB - Discovery by Cassini's plasma instrument of heavy positive and negative ions within Titan's upper atmosphere and ionosphere has advanced our understanding of ion neutral chemistry within Titan's upper atmosphere, primarily composed of molecular nitrogen, with ~2.5% methane. The external energy flux transforms Titan's upper atmosphere and ionosphere into a medium rich in complex hydrocarbons, nitriles and haze particles extending from the surface to 1200 km altitudes. The energy sources are solar UV, solar X-rays, Saturn's magnetospheric ions and electrons, solar wind and shocked magnetosheath ions and electrons, galactic cosmic rays (GCR) and the ablation of incident meteoritic dust from Enceladus' E-ring and interplanetary medium. Here it is proposed that the heavy atmospheric ions detected in situ by Cassini for heights >950 km, are the likely seed particles for aerosols detected by the Huygens probe for altitudes <100 km. These seed particles may be in the form of polycyclic aromatic hydrocarbons (PAH) containing both carbon and hydrogen atoms CnHx. There could also be hollow shells of carbon atoms, such as C60, called fullerenes which contain no hydrogen. The fullerenes may compose a significant fraction of the seed particles with PAHs contributing the rest. As shown by Cassini, the upper atmosphere is bombarded by magnetospheric plasma composed of protons, H2+ and water group ions. The latter provide keV oxygen, hydroxyl and water ions to Titan's upper atmosphere and can become trapped within the fullerene molecules and ions. Pickup keV N2+, N+ and CH4+ can also be implanted inside of fullerenes. Attachment of oxygen ions to PAH molecules is uncertain, but following thermalization O+ can interact with abundant CH4 contributing to the CO and CO2 observed in Titan's atmosphere. If an exogenic keV O+ ion is implanted into the haze particles, it could become free oxygen within those aerosols that eventually fall onto Titan's surface. The process of freeing oxygen within aerosols could be driven by cosmic ray interactions with aerosols at all heights. This process could drive pre-biotic chemistry within the descending aerosols. Cosmic ray interactions with grains at the surface, including water frost depositing on grains from cryovolcanism, would further add to abundance of trapped free oxygen. Pre-biotic chemistry could arise within surface microcosms of the composite organic-ice grains, in part driven by free oxygen in the presence of organics and any heat sources, thereby raising the astrobiological potential for microscopic equivalents of Darwin's "warm ponds" on Titan.
KW - Aromatic hydrocarbons
KW - Astrobiology
KW - Atmospherics
KW - Cosmic ray detectors
KW - Cosmic rays
KW - Cosmology
KW - Cyanides
KW - Fullerenes
KW - Interplanetary flight

KW - Ionosphere
KW - Ionospheric measurement
KW - Magnetosphere
KW - Methane
KW - Negative ions
KW - Organic compounds
KW - Oxygen
KW - Polycyclic aromatic hydrocarbons
KW - Seed
KW - Solar energy
KW - Solar system
KW - Solar wind
KW - Space probes
KW - Wind power
KW - Atmospheric ions
KW - Carbon and hydrogens
KW - Carbon atoms
KW - Cassini
KW - Cosmic ray interactions
KW - Cryovolcanism
KW - Enceladus
KW - Energy source
KW - External energy
KW - Free oxygen
KW - Fullerene molecules
KW - Galactic cosmic rays
KW - Haze particles
KW - Heat sources
KW - Heavy negative ions
KW - Hollow shells
KW - Huygens probe
KW - Ice grains
KW - In-situ
KW - Interplanetary medium
KW - Magnetosheaths
KW - Magnetospheric ions
KW - Magnetospheric plasmas
KW - Molecular nitrogen
KW - Neutral chemistry
KW - Organics
KW - Oxygen ions
KW - Saturn's magnetosphere
KW - Seed particle
KW - Solar X-rays
KW - Thermalization
KW - Titan
KW - Titan's atmosphere
KW - Atmospheric aerosols
KW - Aerosols
KW - Astrobiology
KW - Heavy negative ions
KW - Oxygen
KW - Saturn's magnetosphere
KW - Titan

SN - 00320633 (ISSN)
LA - English
J2 - Planet. Space Sci.
M3 - Article
DB - Scopus
N1 - Export Date: 23 February 2023; Cited By: 52; Correspondence Address: E.C. Sittler Jr.; NASA Goddard Space Flight Center, MD, United States; email: Edward.C.Sittler@nasa.gov; CODEN: PLSSA
ER -

TY - JOUR
AU - Bttcher, T.
AU - Huber, L.
AU - Le Corre, L.
AU - Leitner, J.
AU - McCarthy, D.
AU - Nilsson, R.
AU - Teixeira, C.
AU - Vaquer Araujo, S.
AU - Wilson, R.C.
AU - Adjali, F.
AU - Altenburg, M.
AU - Briani, G.
AU - Buchas, P.
AU - Le Postollec, A.
AU - Meier, T.
TI - The HADES mission concept astrobiological survey of Jupiter's icy moon Europa
PY - 2009
T2 - International Journal of Astrobiology
VL - 8
IS - 4
SP - 321
EP - 329
DO - 10.1017/S147355040999022X
UR - https://www.scopus.com/inward/record.uri?eid=2-s2.0-77149176924&doi=10.1017%2fS147355040999022X&partnerID=40&md5=da151e38197123bd33141dd501f5e9ab
AD - Department of Chemistry and Biochemistry, LMU, 81377 Munich, Butenandtstrasse 513, Germany
AD - Physikalisches Institut, Space Research and Planetary Sciences, 3012 Bern, Siedlerstr. 5, Switzerland
AD - CNRS, Université de Nantes, Laboratoire de Planétologie et Géodynamique, Nantes F-44000, rue de la Houssinéire, France
AD - Institute for Astronomy, University of Vienna, A-1180 Vienna, Tuerkenschanzstr. 17, Austria
AD - Department of Microbiology, School of Natural Sciences, National University of Ireland, Galway, Ireland
AD - Department of Astronomy, Stockholm University, AlbaNova University Center, SE-106 91 Stockholm, Roslagstullsbacken 21, Sweden
AD - Centre for Environmental Biology, Animal Biology Department, Faculdade de Cincias of the Universidade de Lisboa, 1749-016 Lisboa, Edifcio C2, 50 piso, Campo Grande, Portugal

AD - Instituto Superior Técnico, Center for Innovation, Technology and Policy Research, Scientific Area, Mechanical Engineering Department, 1049-001 Lisboa, Av. Rovisco Pais, 1, Portugal
AD - Faculty of Medicine, Autonomous University of Barcelona, Edifici M, Campus UAB, 08193 Bellaterra, Spain
AD - Planetary and Space Sciences Research Institute, Open University, Walton Hall, Milton Keynes, United Kingdom
AD - Department of Chemistry, University of Leicester, Leicester, University Road, United Kingdom
AD - Institut de Physique de Rennes, Université de Rennes, Campus de Beaulieu, 35000 Rennes, France
AD - TU Dresden, 01069 Dresden, Helmholtzstraße 10, Germany
AD - Dipartimento di Astronomia e Scienza Dello Spazio, Universitá di Firenze, 50125 Firenze, Largo Enrico Fermi 2, Italy
AD - Xiamen University, 361005 Fujian Province, China
AD - Université de Bordeaux, Laboratoire d'Astrophysique de Bordeaux (LAB), CNRS/INSU, UMR 5804, 33271 Floirac, France
AD - Institute of Communication Networks and Satellite Communications, Graz University of Technology, 8010 Graz, Inffeldgasse 12, Austria
AB - The HADES Europa mission concept aims to provide a framework for an astrobiological in-depth investigation of the Jupiter moon Europa, relying on existing technologies and feasibility. This mission study proposes a system consisting of an orbiter, lander and cryobot as a platform for detailed exploration of Europa. While the orbiter will investigate the presence of a liquid ocean and characterize Europa's internal structure, the lander will survey local dynamics of the ice layer and the surface environment. The lander releases a cryobot, that melts into the ice, will sample the pristine subsurface and is expected to provide data on organic and gaseous content and putative bio-signatures. In summary, we present the scientific objectives for an astrobiological investigation of Europa, resulting in a mission concept with a detailed evaluation of scientific instrumentation, mission sequences, basic design of the spacecraft, technology needs and cost estimations. © Cambridge University Press 2009.
KW - Europa
KW - exobiology
KW - Jupiter
KW - satellite mission
KW - space technology
KW - Astrobiology
KW - Europa
KW - Mission concept
PB - Cambridge University Press
SN - 14735504 (ISSN)
LA - English
J2 - Int. J. Astrobiology
M3 - Article
DB - Scopus
N1 - Export Date: 23 February 2023; Cited By: 5
ER -

TY - CHAP

AU - Deming, J.W.
TI - Extremophiles: Cold Environments
PY - 2009
T2 - Encyclopedia of Microbiology
SP - 147
EP - 158
DO - 10.1016/B978-012373944-5.00280-7
UR - https://www.scopus.com/inward/record.uri?eid=2-s2.0-79952901729&doi=10.1016%2fB978-012373944-5.00280-7&partnerID=40&md5=7726ed4bc1cef2817053cf2bec49df0e
AD - University of Washington, Seattle, WA, United States
AB - Earth has not always been cold. Clear signs of the first glaciation period date to the middle of its history, at about 2.2 billion years ago. Since then, the planet has undergone epochs of warming and cooling, implying that cold-adapted microbes periodically faced extinction as a thermal class or else found refugia, most likely in a cool (≤15°C) deep sea. Current global warming is again reducing the extent of cold, and especially frozen, habitats that arguably provided conditions for the evolution of today's cold-adapted microbes. More than at any time in the history of modern science, research on the extremophiles of cold environments is warranted. Motivations for such research have always included the desire to understand fundamental cellular and molecular aspects of cold adaptation, often centering on how or when a microbe produces lipid membranes flexible enough to transport and exchange solutes and ions with its environment or enzymes capable of catalyzing essential biochemical reactions in the rigidifying cold. There is also now a heightened sense of urgency to understand how microbes currently driving biogeochemical cycles in the cold may respond to environmental warming and thus impact existing food webs and carbon sequestration from the atmosphere. The further expectation that cold-adapted microbes and their products can be harnessed for societal needs, from bioremediation of cold environments to development of alternative energy sources through cold hydrolysis, drives applied research on the extremophiles of the cold. How microbes function or simply survive in frozen environments and unusual ice formations that approach the eutectic has become a central issue in the emerging field of Astrobiology. Most of the current targets for potential (or past) life in our solar system present us only with surfaces at extremely low temperatures, from a planetary-scale average on Mars of -55 (the eutectic for Earth's seawater) to -160°C on Europa, the ice-covered moon to Jupiter believed to harbor an ocean larger than ours. © 2009 Elsevier Inc. All rights reserved..
PB - Elsevier Inc.
SN - 978-012373944-5 (ISBN)
LA - English
J2 - Encycl. of Microbiol.
M3 - Book chapter
DB - Scopus
N1 - Export Date: 23 February 2023; Cited By: 16
ER -

TY - JOUR

AU - Barnes, R.
AU - Jackson, B.
AU - Greenberg, R.
AU - Raymond, S.N.
TI - Tidal limits to planetary habitability
PY - 2009
T2 - Astrophysical Journal
VL - 700
IS - 1 PART 2
SP - L30
EP - L33
DO - 10.1088/0004-637X/700/1/L30
UR - https://www.scopus.com/inward/record.uri?eid=2-s2.0-67650079661&doi=10.1088%2f0004-637X%2f700%2f1%2fL30&partnerID=40&md5=9a7d7c9b49d088931862161b1fa5c064
AD - Department of Astronomy, University of Washington, Seattle, WA 98195-1580, United States
AD - Virtual Planetary Laboratory, United States
AD - Lunar and Planetary Laboratory, University of Arizona, Tucson, AZ 85721, United States
AD - Center for Astrophysics and Space Astronomy, University of Colorado, UCB 389, Boulder, CO 80309-0389, United States
AB - The habitable zones (HZs) of main-sequence stars have traditionally been defined as the range of orbits that intercept the appropriate amount of stellar flux to permit surface water on a planet. Terrestrial exoplanets discovered to orbit M stars in these zones, which are close-in due to decreased stellar luminosity, may also undergo significant tidal heating. Tidal heating may span a wide range for terrestrial exoplanets and may significantly affect conditions near the surface. For example, if heating rates on an exoplanet are near or greater than that on Io (where tides drive volcanism that resurfaces the planet at least every 1 Myr) and produce similar surface conditions, then the development of life seems unlikely. On the other hand, if the tidal heating rate is less than the minimum to initiate plate tectonics, then CO2 may not be recycled through subduction, leading to a runaway greenhouse that sterilizes the planet. These two cases represent potential boundaries to habitability and are presented along with the range of the traditional HZ for main-sequence, low-mass stars. We propose a revised HZ that incorporates both stellar insolation and tidal heating. We apply these criteria to GJ 581 d and find that it is in the traditional HZ, but its tidal heating alone may be insufficient for plate tectonics. © 2009. The American Astronomical Society.
KW - Astrobiology
KW - Planetary systems
KW - Stars: individual (GJ 581)
KW - Stars: low-mass, brown dwarfs
PB - Institute of Physics Publishing
SN - 0004637X (ISSN)
LA - English
J2 - Astrophys. J.
M3 - Article
DB - Scopus
N1 - Export Date: 23 February 2023; Cited By: 77

ER -

TY - JOUR
AU - Stivaletta, N.
AU - Barbieri, R.
TI - Endolithic microorganisms from spring mound evaporite deposits (southern Tunisia)
PY - 2009
T2 - Journal of Arid Environments
VL - 73
IS - 1
SP - 33
EP - 39
DO - 10.1016/j.jaridenv.2008.09.024
UR - https://www.scopus.com/inward/record.uri?eid=2-s2.0-56949086435&doi=10.1016%2fj.jaridenv.2008.09.024&partnerID=40&md5=9e98813ecdc454f2baf0a36fe0a60738
AD - Dipartimento di Scienze della Terra e Geologico-Ambientali, Università di Bologna, 40126 Bologna, Via Zamboni 67, Italy
AB - This paper investigates endolithic microorganisms in modern gypsum crusts located in arid and saline areas of southern Tunisia. In arid regions, the endolithic mode of life facilitates the survival of the microorganisms and protects them from extreme environmental conditions. The gypsum crusts are the product of evaporite precipitation atop spring mounds developed at the margins of the Chott el Jerid, a saline lake close to the Algerian border. The early mineral precipitation has prevented the complete degradation of these microorganisms and their metabolic products because of the rapid sealing provided by the gypsum crusts. The spring mound structures of the Chott el Jerid offer an opportunity to study the endolithic communities in evaporite and arid environments. An understanding of where and how life survives in such extreme conditions, and how life signal prints in the rocks may help in search for life in the geological record and for astrobiological purposes. © 2008 Elsevier Ltd. All rights reserved.
KW - Africa
KW - Chott el Djerid
KW - North Africa
KW - Tunisia
KW - arid region
KW - endolithon
KW - environmental conditions
KW - evaporite
KW - gypsum
KW - microorganism
KW - saline lake
KW - spring (season)
KW - Chott el Jerid
KW - Endoliths
KW - Extreme environments
KW - Gypsum
SN - 1095922X (ISSN)
LA - English
J2 - J. Arid Environ.

M3 - Article
DB - Scopus
N1 - Export Date: 23 February 2023; Cited By: 25; Correspondence Address: N. Stivaletta; Dipartimento di Scienze della Terra e Geologico-Ambientali, Università di Bologna, 40126 Bologna, Via Zamboni 67, Italy; email: nunzia.stivaletta@unibo.it; CODEN: JAEND
ER -

TY - JOUR
AU - Leblanc, F.
AU - Langlais, B.
AU - Fouchet, T.
AU - Barabash, S.
AU - Breuer, D.
AU - Chassefière, E.
AU - Coates, A.
AU - Dehant, V.
AU - Forget, F.
AU - Lammer, H.
AU - Lewis, S.
AU - Lopez-Valverde, M.
AU - Mandea, M.
AU - Menvielle, M.
AU - Pais, A.
AU - Paetzold, M.
AU - Read, P.
AU - Sotin, C.
AU - Tarits, P.
AU - Vennerstrom, S.
TI - Mars environment and magnetic orbiter scientific and measurement objectives
PY - 2009
T2 - Astrobiology
VL - 9
IS - 1
SP - 71
EP - 89
DO - 10.1089/ast.2007.0222
UR - https://www.scopus.com/inward/record.uri?eid=2-s2.0-63049121669&doi=10.1089%2fast.2007.0222&partnerID=40&md5=ad5a8800cd6511791afec81760b23b9b
AD - Service d'Aéronomie du CNRS/IPSL, Université Pierre et Marie Curie, France
AD - Laboratoire de Planétologie et de Géodynamique, Université de Nantes, France
AD - CNRS UMR 6112, Université de Nantes, France
AD - Observatoire de Paris, France
AD - Swedish Institute of Space Physics, Sweden
AD - DLR, Institute of Planetary Research, Germany
AD - Department of Space and Climate Physics, University College London, United Kingdom
AD - Royal Observatory of Belgium, Belgium
AD - Laboratoire de Météorologie Dynamique, IPSL, France
AD - Space Research Institute, Austrian Academy of Sciences,

Austria
AD - Department of Physics and Astronomy, Open University, United Kingdom
AD - AIRE, Instituto di Astrofisica di Andalucia, Spain
AD - GeoForschungsZentrum Potsdam, Germany
AD - CETP, IPSL, France
AD - Department of Physics, University of Coimbra, Portugal
AD - University of Köln, Germany
AD - Atmospheric, Oceanic and Planetary Physics, University of Oxford, United Kingdom
AD - IUEM, Université de Brest, France
AD - Danish National Space Center, Technical University of Denmark, Denmark
AD - Réduit de Verriéres, 91371 Verriéres Le Buisson, BP3, France
AB - In this paper, we summarize our present understanding of Mars' atmosphere, magnetic field, and surface and address past evolution of these features. Key scientific questions concerning Mars' surface, atmosphere, and magnetic field, along with the planet's interaction with solar wind, are discussed. We also define what key parameters and measurements should be performed and the main characteristics of a martian mission that would help to provide answers to these questions. Such a mission - Mars Environment and Magnetic Orbiter (MEMO) - was proposed as an answer to the Cosmic Vision Call of Opportunity as an M-class mission (corresponding to a total European Space Agency cost of less than 300 M€). MEMO was designed to study the strong interconnection between the planetary interior, atmosphere, and solar conditions, which is essential to our understanding of planetary evolution, the appearance of life, and its sustainability. The MEMO main platform combined remote sensing and in situ measurements of the atmosphere and the magnetic field during regular incursions into the martian upper atmosphere. The micro-satellite was designed to perform simultaneous in situ solar wind measurements. MEMO was defined to conduct: • Four-dimensional mapping of the martian atmosphere from the surface up to 120 km by measuring wind, temperature, water, and composition, all of which would provide a complete view of the martian climate and photochemical system; • Mapping of the low-altitude magnetic field with unprecedented geographical, altitude, local time, and seasonal resolutions; • A characterization of the simultaneous responses of the atmosphere, magnetic field, and near-Mars space to solar variability by means of in situ atmospheric and solar wind measurements. © 2009 Mary Ann Liebert, Inc. 2009.
KW - Altitude
KW - Computer Simulation
KW - Evolution, Planetary
KW - Exobiology
KW - Extraterrestrial Environment
KW - Magnetics
KW - Mars
KW - Satellite Communications
KW - Space Flight
KW - Time Factors
KW - altitude
KW - astrobiology

KW - astronomy
KW - computer simulation
KW - magnetism
KW - microclimate
KW - procedures
KW - space flight
KW - telecommunication
KW - time
KW - Atmosphere
KW - Future space mission
KW - Magnetic field
KW - Mars
KW - Solar wind
KW - Surface
PB - Mary Ann Liebert Inc.
SN - 15311074 (ISSN)
C2 - 19317625
LA - English
J2 - Astrobiology
M3 - Article
DB - Scopus
N1 - Export Date: 23 February 2023; Cited By: 8; Correspondence Address: F. Leblanc; Réduit de Verriéres, 91371 Verriéres Le Buisson, BP3, France; email: francois.leblanc@aerov.jussieu.fr
ER -

TY - BOOK
AU - Chris, I.
AU - Jonathan, L.
AU - José, F.
TI - Frontiers of astrobiology
PY - 2009
T2 - Frontiers of Astrobiology
SP - 1
EP - 335
DO - 10.1017/CBO9780511902574
UR - https://www.scopus.com/inward/record.uri?eid=2-s2.0-84924198752&doi=10.1017%2fCBO9780511902574&partnerID=40&md5=d7e30432eba4363cbba5359c3761dfea
AD - University of Arizona, Tucson, 85721, AZ, United States
AD - Cornell University, 402 Space Sciences Building, Ithaca, 14853, NY, United States
AD - Vatican Observatory, Vatican, V-00120, Italy
AB - Astrobiology is an exciting interdisciplinary field that seeks to answer one of the most important and profound questions: are we alone? In this volume, leading international experts explore the frontiers of astrobiology, investigating the latest research questions that will fascinate a wide interdisciplinary audience at all levels. What is the earliest evidence for life on Earth? Where are the most likely sites for life in the Solar System? Could life have evolved elsewhere in the Galaxy? What are the best strategies for detecting intelligent extraterrestrial life? How many habitable or Earth-like exoplanets are there? Progress in astrobiology over the past decade has been rapid and, with evidence accumulating that

Mars once hosted standing bodies of liquid water, the discovery of over 500 exoplanets and new insights into how life began on Earth, the scientific search for our origins and place in the cosmos continues. © Cambridge University Press 2012.
PB - Cambridge University Press
SN - 978-051190257-4 (ISBN); 978-110700641-6 (ISBN)
LA - English
J2 - Frontiers of Astrobiology
M3 - Book
DB - Scopus
N1 - Export Date: 23 February 2023; Cited By: 1
ER -

TY - CONF
AU - Shostak, S.
TI - What ET will look like and why should we care
PY - 2009
T2 - 60th International Astronautical Congress 2009, IAC 2009
VL - 2
SP - 1607
EP - 1613
UR - https://www.scopus.com/inward/record.uri?eid=2-s2.0-77953595967&partnerID=40&md5=2834c65c01de50917b001559e5321025
AD - SETI Institute, Mountain View, CA 94043, 515 N. Whisman Rd., United States
AB - Our experiments to find extraterrestrial life are predicated on the assumption that it is most likely to be found on so-called "habitable worlds." These are planets and moons where surface liquid water exists, and atmospheres of light gases are found. Our searches presume that life on other worlds has a biochemistry at least somewhat similar to our own. While these postulates might be our best guide for finding biology, they could be misleading us in the search for extraterrestrial intelligence (SETI). Timescale arguments suggest that shortly after a sentient species invents the technology for communication, it develops synthetic intelligence. Consequently, SETI's targeted searches of star systems that might have habitable planets in the conventional sense may be chasing a very short-lived prey. In this paper, we discuss what the implications of post-biological intelligence might have in directing our SETI experiments.
KW - Biochemistry
KW - Space research
KW - Conventional sense
KW - Extraterrestrial life
KW - Liquid water
KW - Search for extraterrestrial intelligences
KW - Star systems
KW - Synthetic intelligence
KW - Time-scales
KW - Planets
SN - 978-161567908-9 (ISBN)
LA - English
J2 - Int. Astronaut. Cong., IAC
M3 - Conference paper

DB - Scopus
N1 - Export Date: 23 February 2023; Cited By: 0; Correspondence Address: S. Shostak; SETI Institute, Mountain View, CA 94043, 515 N. Whisman Rd., United States; email: sshostak@seti.org
ER -

TY - JOUR
AU - Stivaletta, N.
AU - Barbieri, R.
AU - Picard, C.
AU - Bosco, M.
TI - Astrobiological significance of the sabkha life and environments of southern Tunisia
PY - 2009
T2 - Planetary and Space Science
VL - 57
IS - 5-6
SP - 597
EP - 605
DO - 10.1016/j.pss.2008.10.002
UR - https://www.scopus.com/inward/record.uri?eid=2-s2.0-67349179179&doi=10.1016%2fj.pss.2008.10.002&partnerID=40&md5=ccc585286d79a69a2bd977156a879551
AD - Dipartimento di Scienze della Terra e Geologico-Ambientali, Università di Bologna, 40126 Bologna, Via Zamboni 67, Italy
AD - Dipartimento di Scienze e Tecnologie Agroambientali, Università di Bologna, 40127 Bologna, Viale Fanin 42, Italy
AB - In high-salinity and water-scarce environments, such as in hot and dry deserts, species develop adaptive strategies that are necessary for living in such harsh conditions. Continental ephemeral salt lakes (sabkhas) with periodic flooding from subsurface groundwater followed by high salt concentrations, such as the ones of the northern Africa Chotts, rank among the geological settings wherein the combined effect of salt concentration and fluctuation of water availability make the environment unstable and can thereafter lead to extreme changes. The present study investigates the continental sabkha environments of southern Tunisia, in which ecological niches (i.e. water and salt precipitates, including halite, gypsum, and dolomite) host microbial life. Halophilic microorganisms can be trapped in the extensive saline crusts of halite and gypsum, which can be regarded as the first step of their delivery to the fossil record. The study of halophiles can provide clues for the understanding of life strategies in extreme terrestrial environments, such as sabkhas, which are potential good terrestrial analogs for evaporite-bearing Martian deposits. © 2008 Elsevier Ltd. All rights reserved.
KW - Arid regions
KW - Astrobiology
KW - Groundwater
KW - Gypsum
KW - Hydrogeology
KW - Microorganisms
KW - Sodium chloride
KW - Adaptive strategies

KW - Combined effects
KW - Dry deserts
KW - Ecological niches
KW - Evaporite
KW - Fossil records
KW - Geological settings
KW - High salt concentrations
KW - Martian environment
KW - Microbial lives
KW - Sabkha
KW - Salt concentrations
KW - Salt lakes
KW - Southern tunisia
KW - Terrestrial environments
KW - Water availabilities
KW - Lead
KW - Astrobiology
KW - Evaporite
KW - Martian environment
KW - Sabkha
SN - 00320633 (ISSN)
LA - English
J2 - Planet. Space Sci.
M3 - Article
DB - Scopus
N1 - Export Date: 23 February 2023; Cited By: 21; Correspondence Address: N. Stivaletta; Dipartimento di Scienze della Terra e Geologico-Ambientali, Università di Bologna, 40126 Bologna, Via Zamboni 67, Italy; email: nunzia.stivaletta@unibo.it; CODEN: PLSSA
ER -

TY - JOUR
AU - Wickramasinghe, C.
TI - Life from space
PY - 2009
T2 - Biochemist
VL - 31
IS - 1
SP - 40
EP - 44
DO - 10.1042/bio03101040
UR - https://www.scopus.com/inward/record.uri?eid=2-s2.0-85100632369&doi=10.1042%2fbio03101040&partnerID=40&md5=5770da97f13478b7932a6473aa3f8fd8
AD - Cardiff University, United Kingdom
KW - abiogenesis
KW - astrobiology
KW - Oparin–Haldane chemistry
KW - panspermia
KW - RNA world
KW - Urey–Miller experiment
PB - Portland Press Ltd
SN - 0954982X (ISSN)
LA - English

J2 - Biochemist
M3 - Article
DB - Scopus
N1 - Export Date: 23 February 2023; Cited By: 0
ER -

TY - CHAP
AU - Armando, A.
AU - Rafael, V.
AU - Raymond, P.
TI - Early mars – cradle or cauldron?
PY - 2009
T2 - Frontiers of Astrobiology
SP - 157
EP - 174
DO - 10.1017/CBO9780511902574.013
UR - https://www.scopus.com/inward/record.uri?eid=2-s2.0-84924216657&doi=10.1017%2fCBO9780511902574.013&partnerID=40&md5=c81f77f8b1e42059e670021ca4c41c74
AD - Departamento de Genética Moleculary Microbiología, Facultad de Ciencias Biológicas, Pontificia Universidad Católica de Chile, Alameda 340, Santiago, Chile
AD - Department of Geophysical Sciences, University of Chicago, 5734 S. Ellis Ave, Chicago, 60637, IL, United States
AB - Mars constitutes one of the most interesting settings for astrobiological studies, not only due to its proximity to Earth, but also because it is conceivable that life may have originated in this seemingly barren planet. Mars has captured man's imagination since the time of Giovanni Schiaparelli, who in 1877 published a detailed map that became a standard reference in planetary cartography. Schiaparelli's original map showed a network of linear markings which went across the entire Martian surface joining different dark areas to one another. He referred to these lines as canali and named them after famous rivers. The Italian word canale (plural canali) was soon incorrectly translated to English as “canals,” which denotes artificially made ducts. Being aware of this mistranslation, Schiaparelli stated that [T]hese names may be regarded as a mere artifice…After all, we speak in a similar way of the seas of the Moon, knowing very well that they do not consist of liquid masses. Thus, the idea of artificially made water courses remained, implying that Mars was a planet harboring life. Later on, Percival Lowell fueled further speculations about possible Martian life forms in his book Mars as the Adobe of Life (1908), popularizing the view that these markings were manifestations of an intelligent civilization. One can analyze the possibility of life on Mars taking into consideration the different geological ages of this planet (now under revision). The Noachian period (named after Noachis Terra), which took place between 4.5 and about 3.7 billion years ago (Gya), is considered to be the warm and wet age of the planet. Extensive erosion by liquid water produced river valley networks whose marks have survived to the present time. There may even have been extensive lakes and oceans at this time, though evidence for such features is at present ambiguous. Thereafter, the Hesperian (named after Hesperia Planum) or volcanic period, from 3.7 to approximately

3.0 Gya, showed catastrophic releases of water that carved extensive outflow channels, with ephemeral lakes or seas. Finally, there is the Amazonian period (named after Amazonis Planitia), which extends from 3.0 Gya until today. It is considered the cold and dry period of Mars, with glacial/periglacial activity and minor releases of liquid water. © Cambridge University Press 2012.
PB - Cambridge University Press
SN - 978-051190257-4 (ISBN); 978-110700641-6 (ISBN)
LA - English
J2 - Frontiers of Astrobiology
M3 - Book chapter
DB - Scopus
N1 - Export Date: 23 February 2023; Cited By: 0
ER -

TY - CONF
AU - Rennó, N.O.
AU - Mehta, M.
AU - Block, B.P.
AU - Braswell, S.
TI - The discovery of liquid water on Mars and its implications for astrobiology
PY - 2009
T2 - Proceedings of SPIE - The International Society for Optical Engineering
VL - 7441
C7 - 74410C
DO - 10.1117/12.832195
UR - https://www.scopus.com/inward/record.uri?eid=2-s2.0-70449502837&doi=10.1117%2f12.832195&partnerID=40&md5=c0c843cd9642e9866b59bbbcfc639415
AD - Department of Atmospheric, Oceanic and Space Sciences, College of Engineering, University of Michigan, Ann Arbor, MI, 48109, 2455 Hayward St., United States
AB - Since liquid water is a key ingredient for life as we know it, NASA has adopted the theme "follow the water" as an strategy for exploring Mars. Recently, Renno et al.1, 2showed evidence that liquid saline-water exists in areas disturbed by the Phoenix Mars Lander. Moreover, they argued that the thermodynamics of freeze-thaw cycles leads to the formation of concentrated saline solutions (brines) with freezing temperatures much higher than current summer ground temperatures where ground ice exists near the surface and therefore liquid saline-water should be common on Mars. Here we summarize these ideas, present some new results, and discuss their implications for astrobiology. We propose a strategy for searching for liquid saline water on Mars and argue that NASA's theme for the exploration of Mars should be updated to "follow the liquid water." © 2009 SPIE.
KW - Astrobiology
KW - Liquids
KW - Martian surface analysis
KW - NASA
KW - Salts
KW - Deliquescence

KW - Habitability
KW - Life
KW - Liquid water
KW - Mars
KW - Phoenix mission
KW - Saline water
KW - Astrobiology
KW - Brines
KW - Deliquescence
KW - Habitability
KW - Life
KW - Liquid water
KW - Mars
KW - Phoenix mission
KW - Salts
SN - 0277786X (ISSN); 978-081947731-6 (ISBN)
LA - English
J2 - Proc SPIE Int Soc Opt Eng
M3 - Conference paper
DB - Scopus
N1 - Export Date: 23 February 2023; Cited By: 4; Correspondence Address: N. O. Rennó; Department of Atmospheric, Oceanic and Space Sciences, College of Engineering, University of Michigan, Ann Arbor, MI, 48109, 2455 Hayward St., United States; email: renno@alum.mit.edu; CODEN: PSISD
ER -

TY - CONF
AU - Davis, M.W.
AU - Slater, D.C.
AU - Gladstone, G.R.
AU - Stern, S.A.
AU - Greathouse, T.K.
AU - Retherford, K.D.
AU - Versteeg, M.H.
AU - Black, R.K.
TI - Radiometric performance results of the lunar reconnaissance orbiter's Lyman alpha mapping project (LRO/LAMP) UV imaging spectrograph
PY - 2009
T2 - Proceedings of SPIE - The International Society for Optical Engineering
VL - 7441
C7 - 744110
DO - 10.1117/12.826068
UR - https://www.scopus.com/inward/record.uri?eid=2-s2.0-70449463986&doi=10.1117%2f12.826068&partnerID=40&md5=cfcff6a4f08e73056f06c8f5de88f746
AD - Southwest Research Institute, San Antonio, TX 78238, 6220 Culebra Rd., United States
AD - Southwest Research Institute, Boulder, CO 80302, 1050 Walnut St., United States
AB - We describe the pre-flight radiometric performance and calibration results of the Lunar Reconnaissance Orbiter's Lyman

Alpha Mapping Project (LRO/LAMP) flight model. LAMP is a lightweight (6.1 kg), low-power (4.5 W), ultraviolet spectrograph based on the ALICE instruments now in flight aboard the European Space Agency's Rosetta spacecraft and NASA's New Horizons spacecraft. Its primary job will be to identify and localize exposed water frost in permanently shadowed regions (PSRs), and to characterize landforms and albedos in PSRs. Detailed radiometric performance results of the LAMP flight model are presented and discussed. © 2009 SPIE.
KW - Astrobiology
KW - Calibration
KW - Interplanetary spacecraft
KW - Moon
KW - NASA
KW - Radiometry
KW - Societies and institutions
KW - Space flight
KW - Spectrographs
KW - Ultraviolet spectroscopy
KW - European Space Agency
KW - Flight models
KW - Low Power
KW - Lunar reconnaissance orbiter
KW - Lunar reconnaissance orbiters
KW - Mapping projects
KW - Pre-flight
KW - Radiometric
KW - Radiometric performance
KW - Rosetta
KW - Shadowed regions
KW - UV imaging
KW - UV spectroscopy
KW - Spacecraft instruments
KW - Calibration
KW - Lunar reconnaissance orbiter
KW - Moon
KW - Radiometric
KW - UV spectroscopy
SN - 0277786X (ISSN); 978-081947731-6 (ISBN)
LA - English
J2 - Proc SPIE Int Soc Opt Eng
M3 - Conference paper
DB - Scopus
N1 - Export Date: 23 February 2023; Cited By: 5; CODEN: PSISD
ER -

TY - CHAP
AU - Julie, C.-R.
AU - Jonathan, L.
TI - Small habitable worlds
PY - 2009
T2 - Frontiers of Astrobiology
SP - 201
EP - 228
DO - 10.1017/CBO9780511902574.015

UR - https://www.scopus.com/inward/record.uri?eid=2-s2.0-84924092952&doi=10.1017%2fCBO9780511902574.015&partnerID=40&md5=7c4d788b8396c9929a76eed65f0e9b3d
AD - Jet Propulsion Laboratory, 4800 Oak Grove Dr, Pasadena, 9110, CA, United States
AD - Cornell University, 402 Space Sciences Building, Ithaca, 14853, NY, United States
AB - Introduction The astrobiological relevance of small bodies has been acknowledged for several decades with regard to their role in delivering volatiles to Earth and the inner Solar system (see Lunine 2006 for a review). However, until recently these objects were considered too small to sustain a deep liquid layer and hydrothermal activity over the long term. The last decade has been marked by a dramatic evolution of our understanding of small bodies, from observational constraints and theoretical arguments. The discoveries of geological activity on Saturn's satellite Enceladus and Pluto's satellite Charon have prompted theoreticians to develop new approaches for modeling the interiors of these objects, some of which are larger and/or warmer than Jupiter's satellite Europa, considered an archetype of a potentially habitable icy world. The purpose of this chapter is to evaluate the habitability potential of certain small bodies, i.e. their potential for sheltering life, whether life could develop in these environments, or was brought in from a different source. This chapter focuses on large wet asteroids, small icy satellites, and trans-Neptunian objects (e.g. see the representatives of each class in Figure 10.1). We evaluate the occurrence in each class of objects of certain parameters that determine their capacity to sustain habitable conditions: the energy necessary to support chemical activity and chemical conditions amenable to the thriving of living organisms. The latter aspect is difficult to fully fathom as life has been found in the most surprising environments and based on unexpected nutrient systems. The question of the origin of life in favorable environments is considered in Chapter 2. © Cambridge University Press 2012.
PB - Cambridge University Press
SN - 978-051190257-4 (ISBN); 978-110700641-6 (ISBN)
LA - English
J2 - Frontiers of Astrobiology
M3 - Book chapter
DB - Scopus
N1 - Export Date: 23 February 2023; Cited By: 4
ER -

TY - CHAP
AU - Davenport, A.A.
TI - Baroque fire (a note on early-modern angelology)
PY - 2009
T2 - Evidence and Interpretation in Studies on Early Science and Medicine: Essays in Honor of John E. Murdoch
SP - 369
EP - 397
DO - 10.1163/ej.9789004178786.i-458.98
UR - https://www.scopus.com/inward/record.uri?eid=2-s2.0-84943790646&doi=10.1163%2fej.9789004178786.i-458.98&partnerID=4

0&md5=e07d804f5e246a03dd0baa47bc7392e1
AD - Boston College, 96 Bedford Street, Concord, 01742, MA, United States
AB - Between Galileo's discovery of the moons of Jupiter (1610) and the publication of Newton's Principia (1687), uncertainty regarding the structure of the heavens combined with a lyrical fascination for extraterrestrial life inspired a distinctly Baroque outpouring of speculation in which angels played a key part. English Catholic "recusants," haunted by a feeling of lost unity, vividly illustrate the imaginative character of Baroque speculation. © 2009 by Koninklijke Brill NV, Leiden, The Netherlands. All rights reserved.
KW - Angels
KW - Antoine Legrand
KW - Franciscus à Sancta Clara
KW - Kenelm Digby
KW - Meteorology
KW - Planetary atmosphere
KW - Robert Boyle
KW - Thomas White
PB - Brill
SN - 978-904744113-7 (ISBN); 978-900417878-6 (ISBN)
LA - English
J2 - Evid. and Interpret. in Stud. on Early Sci. and Med.: Essays in Honor of John E. Murdoch
M3 - Book chapter
DB - Scopus
N1 - Export Date: 23 February 2023; Cited By: 0
ER -

TY - JOUR
AU - Durham, W.B.
AU - Pathare, A.V.
AU - Stern, L.A.
AU - Lenferink, H.J.
TI - Mobility of icy sand packs, with application to Martian permafrost
PY - 2009
T2 - Geophysical Research Letters
VL - 36
IS - 23
C7 - L23203
DO - 10.1029/2009GL040392
UR - https://www.scopus.com/inward/record.uri?eid=2-s2.0-72049100093&doi=10.1029%2f2009GL040392&partnerID=40&md5=4a4b8a7b08fe40ea7a7187452cc49957
AD - Department of Earth, Atmospheric, and Planetary Sciences, Massachusetts Institute of Technology, Cambridge, MA 02139-4307, United States
AD - Planetary Science Institute, Pasadena, CA 91101-3005, 225 S. Lake Ave., United States
AD - U.S. Geological Survey, MS 977, Menlo Park, CA 94025, United States
AB - [1] The physical state of water on Mars has fundamental ramifications for both climatology and astrobiology. The widespread

presence of "softened" Martian landforms (such as impact craters) can be attributed to viscous creep of subsurface ground ice. We present laboratory experiments designed to determine the minimum amount of ice necessary to mobilize topography within Martian permafrost. Our results show that the jammed-to-mobile transition of icy sand packs neither occurs at fixed ice content nor is dependent on temperature or stress, but instead correlates strongly with the maximum dry packing density of the sand component. Viscosity also changes rapidly near the mobility transition. The results suggest a potentially lower minimum volatile inventory for the impact-pulverized megaregolith of Mars. Furthermore, the long-term preservation of partially relaxed craters implies that the ice content of Martian permafrost has remained close to that at the mobility transition throughout Martian history. Copyright 2009 by the American Geophysical Union.
KW - Climatology
KW - Ice
KW - Dry packing
KW - Ground ice
KW - Ice content
KW - Impact craters
KW - Laboratory experiments
KW - Long-term preservation
KW - Physical state
KW - Sand pack
KW - Volatile inventory
KW - Water on Mars
KW - crater
KW - creep
KW - ice
KW - Mars
KW - permafrost
KW - planetary surface
KW - sand
KW - temperature effect
KW - topography
KW - viscosity
KW - Permafrost
SN - 00948276 (ISSN)
LA - English
J2 - Geophys. Res. Lett.
M3 - Article
DB - Scopus
N1 - Export Date: 23 February 2023; Cited By: 23; Correspondence Address: W. B. Durham; Department of Earth, Atmospheric, and Planetary Sciences, Massachusetts Institute of Technology, Cambridge, MA 02139-4307, United States; email: wbdurham@mit.edu; CODEN: GPRLA
ER -

TY - JOUR
AU - Kieffer, S.W.
AU - Lu, X.
AU - McFarquhar, G.

AU - Wohletz, K.H.
TI - A redetermination of the ice/vapor ratio of Enceladus' plumes: Implications for sublimation and the lack of a liquid water reservoir
PY - 2009
T2 - Icarus
VL - 203
IS - 1
SP - 238
EP - 241
DO - 10.1016/j.icarus.2009.05.011
UR - https://www.scopus.com/inward/record.uri?eid=2-s2.0-68749117389&doi=10.1016%2fj.icarus.2009.05.011&partnerID=40&md5=4d2a5d5419d405c82380338204f9d462
AD - Department of Geology, University of Illinois, Urbana, IL 61801, 1301 W Green St, United States
AD - Department of Atmospheric Sciences, University of Illinois, Urbana, IL 61801, 105 S. Gregory St, United States
AD - Los Alamos National Laboratory, Los Alamos, NM 87545, EES11 MS 665, United States
AB - The discovery of plumes of H2O vapor and ice particles erupting from the south pole of Enceladus, the tiny frigid satellite of Saturn, sparked controversy over whether these plumes are produced by boiling, or by sublimation with subsequent recondensation of the sublimated vapor [Porco, C.C., Helfenstein, P., Thomas, P.C., Ingersoll, A.P., Wisdom, J., West, R., Neukum, G., Denk, T., Wagner, R., Roatsch, T., Kieffer, S., Turtle, E., McEwen, A., Johnson, T.V., Rathbun, J., Veverka, J., Wilson, D., Perry, J., Spitale, J., Brahic, A., Burns, J.A., DelGenio, A.D., Dones, L., Murray, C.D., Squyres, S., 2006. Science 311, 1393-1401]. Porco et al.'s analysis that the masses of ice (I) and vapor (V) in the plume were comparable was taken to argue against the occurrence of sublimation and recondensation, leading to the hypothesis that the reservoir was boiling water, possibly as close as 7 m to the surface. Thus, it has been advocated that Enceladus should be a target for astrobiology exploration. Here we show, with recalculations using the original data and methodologies, as well as with new sensitivity studies, that the mass of ice in the column is significantly less than the mass of water vapor, and that by considering three additional effects, I/V is likely to be <0.2-0.1. This means that the plume is dominated by vapor that the thermodynamics permits to be easily produced by sublimation with recondensation. The low I/V ratio provides no compelling criterion for consideration of a liquid water reservoir. The uncertainties on the I/V ratio have not previously been discussed in the literature. Although the I/V ratio is sensitive to particle sizes and size distributions, the masses of ice (I) and vapor (V) are not comparable in any scenario constrained by available observations. We thus discuss the implications of sublimation from a thermodynamic point of view in a context that has not been presented previously. Constraints on I/V ratio from future spacecraft measurements of the plume, in conjunction with consideration of the total plume composition and multicomponent analysis, can help constrain source conditions for the plume. Crown Copyright © 2009.

KW - Enceladus
KW - Ices
SN - 10902643 (ISSN)
LA - English
J2 - Icarus
M3 - Article
DB - Scopus
N1 - Export Date: 23 February 2023; Cited By: 25; Correspondence Address: S.W. Kieffer; Department of Geology, University of Illinois, Urbana, IL 61801, 1301 W Green St, United States; email: slkieffer@gmail.com; CODEN: ICRSA
ER -

TY - JOUR
AU - Grasby, S.E.
AU - Bezys, R.
AU - Beauchamp, B.
TI - Silica chimneys formed by low-temperature brine spring discharge.
PY - 2009
T2 - Astrobiology
VL - 9
IS - 10
SP - 931
EP - 941
DO - 10.1089/ast.2009.0350
UR - https://www.scopus.com/inward/record.uri?eid=2-s2.0-77953045467&doi=10.1089%2fast.2009.0350&partnerID=40&md5=fa8ce16a8abcd9ecd2abdc71acc73a07
AD - Geological Survey of Canada-Calgary, Calgary, T2L 2A7, Alberta, Canada
AB - Vertical pipes comprised of loose silica and lined by chert nodules have been observed in the abandoned Mafeking Quarry in central Manitoba, Canada. Discovery of microfossils within these features of the same age as the carbonate host rock indicates that they are a dissolution/replacement structure rather than infill of karst features by younger sediments. These features occur on the low thermal maturity edge of the intercratonic Williston Basin, are not associated with any known tectonic or hydrothermal activity, and show no sign of localized discharge of high-temperature fluids. Modern low-temperature brine springs with silica-filled discharge channels occur nearby, which suggests the silica chimneys are relic spring channels. Geochemical models have shown that dissolution/replacement reactions would be expected due to mixing of brine spring water with shallow groundwater in the region. Results indicate that silica pipe features in the rock record cannot be assumed to be indicative of hydrothermal activity. At the same time, results increase the astrobiological significance of low-temperature siliceous deposits.
KW - Canada
KW - Cold Temperature
KW - Fresh Water
KW - Geography
KW - Salinity

KW - Salts
KW - Silicon Dioxide
KW - Water
KW - brine
KW - fresh water
KW - inorganic salt
KW - silicon dioxide
KW - water
KW - article
KW - Canada
KW - chemistry
KW - cold
KW - geography
KW - salinity
SN - 15578070 (ISSN)
C2 - 20041746
LA - English
J2 - Astrobiology
M3 - Article
DB - Scopus
N1 - Export Date: 23 February 2023; Cited By: 10; Correspondence Address: S.E. Grasby; email: sgrasby@nrcan.gc.ca
ER -

TY - JOUR
AU - Abe, S.
TI - Meteoroids and meteors - Observations and connection to parent bodies
PY - 2009
T2 - Lecture Notes in Physics
VL - 758
SP - 129
EP - 166
DO - 10.1007/978-3-540-76935-4_5
UR - https://www.scopus.com/inward/record.uri?eid=2-s2.0-62149104387&doi=10.1007%2f978-3-540-76935-4_5&partnerID=40&md5=014adfb1867133b2d9f7a264ed1174b3
AD - Institute of Astronomy, National Central University, Jhongli, Taoyuan 32001, 300 Jhongda Road, Taiwan
AB - Meteoroid are a small rocky bodies traveling through interplanetary space. Meteors are phenomena caused by the interaction of meteoroids with the Earth's upper atmosphere. In this chapter the author, will briefly discuss observational methods and then concentrate on optical observations of meteors. First, the basic properties of meteor phenomenon in the atmosphere and classification of meteoroids are introduced and then coincidental phenomena, e.g., wake, jets, and train, are mentioned. Scientific observations (imaging and spectroscopy) carried out using various observational techniques allow measuring characteristics of meteoroids, e.g., orbits, density, strength, compositions. All information are potentially useful for investigating parent bodies of meteoroids, such as comets and asteroids. Searching for organics-related CHON and water in meteoroids is of particular interest for astrobiology. © 2008 Springer-Verlag Berlin Heidelberg.

A2 - Mann I.
A2 - Mukai T.
A2 - Nakamura A.M.
A2 - Kobe University, Dept. Earth and Planetary Sciences, Nada-ku 657-8501, Rokkodai-cho Kobe
SN - 00758450 (ISSN); 978-354076934-7 (ISBN)
LA - English
J2 - Lect. Notes Phys.
M3 - Article
DB - Scopus
N1 - Export Date: 23 February 2023; Cited By: 2; Correspondence Address: S. Abe; Institute of Astronomy, National Central University, Jhongli, Taoyuan 32001, 300 Jhongda Road, Taiwan; email: shinsuke.avell@gmail.com
ER -

TY - JOUR
AU - Lemke, K.H.
AU - Rosenbauer, R.J.
AU - Bird, D.K.
TI - Peptide synthesis in early earth hydrothermal systems
PY - 2009
T2 - Astrobiology
VL - 9
IS - 2
SP - 141
EP - 146
DO - 10.1089/ast.2008.0166
UR - https://www.scopus.com/inward/record.uri?eid=2-s2.0-65249145113&doi=10.1089%2fast.2008.0166&partnerID=40&md5=9e57d909eb93353c93ff0e706686f20e
AD - Department of Geological and Environmental Sciences, Stanford University, Stanford, CA, United States
AD - United States Geological Survey, Hydrothermal Laboratory, Menlo Park, CA, United Kingdom
AD - Department of Earth Sciences, University of Hong Kong, Hong Kong, Hong Kong
AD - Department of Earth Sciences, University of Hong Kong, Hong Kong, SAR, Pokfulam Road, Hong Kong
AB - We report here results from experiments and thermodynamic calculations that demonstrate a rapid, temperature-enhanced synthesis of oligopeptides from the condensation of aqueous glycine. Experiments were conducted in custom-made hydrothermal reactors, and organic compounds were characterized with ultraviolet-visible procedures. A comparison of peptide yields at 260°C with those obtained at more moderate temperatures (160°C) gives evidence of a significant (13 kJ · mol -1) exergonic shift. In contrast to previous hydrothermal studies, we demonstrate that peptide synthesis is favored in hydrothermal fluids and that rates of peptide hydrolysis are controlled by the stability of the parent amino acid, with a critical dependence on reactor surface composition. From our study, we predict that rapid recycling of product peptides from cool into near-supercritical fluids in mid-ocean ridge hydrothermal systems will enhance peptide chain elongation. It is anticipated

that the abundant hydrothermal systems on early Earth could have provided a substantial source of biomolecules required for the origin of life. Astrobiology 9, 141–146. © 2009 Mary Ann Liebert, Inc. 2009.
KW - Atmospheric Pressure
KW - Biogenesis
KW - Buffers
KW - Chromatography, High Pressure Liquid
KW - Evolution, Chemical
KW - Glycine
KW - Hot Temperature
KW - Hydrogen-Ion Concentration
KW - Peptide Biosynthesis
KW - Phosphates
KW - Seawater
KW - Spectrophotometry, Ultraviolet
KW - Thermodynamics
KW - Time Factors
KW - Water
KW - buffer
KW - glycine
KW - phosphate
KW - sea water
KW - water
KW - article
KW - astronomy
KW - atmospheric pressure
KW - biogenesis
KW - chemistry
KW - heat
KW - high performance liquid chromatography
KW - peptide synthesis
KW - pH
KW - thermodynamics
KW - time
KW - ultraviolet spectrophotometry
KW - Condensation
KW - Gibbs energy
KW - Hydrolysis
KW - Hydrothermal
KW - Origin of life
KW - Peptide
SN - 15311074 (ISSN)
C2 - 19371157
LA - English
J2 - Astrobiology
M3 - Article
DB - Scopus
N1 - Export Date: 23 February 2023; Cited By: 46; Correspondence Address: K. H. Lemke; Department of Earth Sciences, University of Hong Kong, Hong Kong, SAR, Pokfulam Road, Hong Kong; email: kono@hku.hk
ER -

TY - CONF
AU - Guisler, M.
AU - Pikuta, E.V.
AU - Townsend, A.
AU - Hoover, R.B.
TI - Psychrotolerant anaerobes from Lake Podprudnoye, Antarctica and penguin Spheniscus demersus colony, South Africa
PY - 2009
T2 - Proceedings of SPIE - The International Society for Optical Engineering
VL - 7441
C7 - 74410L
DO - 10.1117/12.828880
UR - https://www.scopus.com/inward/record.uri?eid=2-s2.0-70449502834&doi=10.1117%2f12.828880&partnerID=40&md5=d44a69cf3208dd497d2f42801fcd0318
AD - Astrobiology Laboratory, National Space Science and Technology Center, University of Alabama in Huntsville, Huntsville, AL 35805, 320 Sparkman Dr., United States
AB - The study of a sample collected from a wind-made ice sculpture near Lake Podprudnoye, Antarctica led to the isolation of the psychrotolerant strain ISLP-3. Cells of the new isolate are vibrio-shaped that measure 0.5 × 1.0-3.0 μm in size. Growth occurs within the temperature range 5-35°C with the optimum at 22 °C. Salinity range for growth is 0-2 % NaCl with the optimum at 0.25 %. The new isolate grows within a pH range from 6.0 to 9.5 with the optimum at 7.5. Strain ISLP-3 is saccharolytic, growing on the following substrates: D-glucose, D-ribose, D-fructose, D-arabinose, maltose, sucrose, D-trehalose, D-mannose, D-cellobiose, lactose, starch, chitin, triethylamine, N-acetylglucosamine, and urea. The best growth occurred on D-cellobiose. An environmental sample of pond water near a colony of the endemic species of African penguins, Spheniscus demersus, was collected in February 2008 and delivered directly to the Astrobiology laboratory at NSSTC. The microbiological study of this sample led to the isolation of two psychrotolerant strains ARHSd-7G and ARHSd-9G. Both strains are strictly anaerobic bacteria and are able to grow at high pH and low temperatures. The cells of strain ARHSd-7G are motile, vibrio-shaped, spore-forming cells. Optimal growth of this strain occurs at 30 °C, 3 % NaCl, and pH 8.9. The isolate ARHSd-7G combines sugarlytic and proteolytic metabolisms, growing on some proteolysis products including peptone and yeast extract and a number of sugars. The second isolate, ARHSd-9G, exhibits thin, elongated rods that measure 0.4 × 3-5 μm. The cells are motile and spore-forming. Optimal growth of strain ARHSd-9G occurs at 30 °C, 1.75 % NaCl, and pH 8.5. The strain ARHSd-9G is sugarlytic, growing well on substrates such as D-glucose, sucrose, D-cellobiose, maltose, fructose, D-mannose, and trehalose (the only exception is positive growth on yeast extract). In this report, the physiological and morphological characteristics of the novel psychrotolerant, alkaliphilic, and neutrophilic isolates from the Antarctica 2008 expeditions will be discussed. © 2009 SPIE.
KW - Astrobiology
KW - Bacteria

KW - Fructose
KW - Glucose
KW - Lakes
KW - Maltose
KW - Metabolism
KW - Sodium chloride
KW - Sugar (sucrose)
KW - Urea
KW - Yeast
KW - Alkaliphile
KW - Antarctica
KW - Psychrotolerant anaerobe
KW - Spheniscus demersus
KW - Sugarlytic
KW - Strain
KW - Alkaliphile
KW - Antarctica
KW - Lake podprudnoe
KW - Psychrotolerant anaerobe
KW - Spheniscus demersus
KW - Sugarlytic
SN - 0277786X (ISSN); 978-081947731-6 (ISBN)
LA - English
J2 - Proc SPIE Int Soc Opt Eng
M3 - Conference paper
DB - Scopus
N1 - Export Date: 23 February 2023; Cited By: 6; CODEN: PSISD
ER -

TY - JOUR
AU - Vogel, M.B.
AU - Des Marais, D.J.
AU - Turk, K.A.
AU - Parenteau, M.N.
AU - Jahnke, L.L.
AU - Kubo, M.D.Y.
TI - The role of biofilms in the sedimentology of actively forming gypsum deposits at guerrero Negro, Mexico
PY - 2009
T2 - Astrobiology
VL - 9
IS - 9
SP - 875
EP - 893
DO - 10.1089/ast.2008.0325
UR - https://www.scopus.com/inward/record.uri?eid=2-s2.0-72449196233&doi=10.1089%2fast.2008.0325&partnerID=40&md5=787b26d7179a6fd8de7c2349d40465e7
AD - SETI Institute, Mountain View, CA 94043, 415 N. Whisman Rd., United States
AD - Exobiology Branch, NASA Ames Research Center, Moffett Field, CA, United States
AD - Oak Ridge Associated Universities NPP, NASA/ARC Astrobiology Branch, Moffett Field, CA, United States

AB - Actively forming gypsum deposits at the Guerrero Negro sabkha and saltern system provided habitats for stratified, pigmented microbial communities that exhibited significant morphological and phylogenetic diversity. These deposits ranged from meter-thick gypsum crusts forming in saltern seawater concentration ponds to columnar microbial mats with internally crystallized gypsum granules developing in natural anchialine pools. Gypsum-depositing environments were categorized as forming precipitation surfaces, biofilm-supported surfaces, and clastic surfaces. Each surface type was described in terms of depositional environment, microbial diversity, mineralogy, and sedimentary fabrics. Precipitation surfaces developed in high-salinity subaqueous environments where rates of precipitation outpaced the accumulation of clastic, organic, and/or biofilm layers. These surfaces hosted endolithic biofilms comprised predominantly of oxygenic and anoxygenic phototrophs, sulfate-reducing bacteria, and bacteria from the phylum Bacteroidetes. Biofilm-supported deposits developed in lower-salinity subaqueous environments where light and low water-column turbulence supported dense benthic microbial communities comprised mainly of oxygenic phototrophs. In these settings, gypsum granules precipitated in the extracellular polymeric substance (EPS) matrix as individual granules exhibiting distinctive morphologies. Clastic surfaces developed in sabkha mudflats that included gypsum, carbonate, and siliclastic particles with thin gypsum/biofilm components. Clastic surfaces were influenced by subsurface brine sheets and capillary evaporation and precipitated subsedimentary gypsum discs in deeper regions. Biofilms appeared to influence both chemical and physical sedimentary processes in the various subaqueous and subaerially exposed environments studied. Biofilm interaction with chemical sedimentary processes included dissolution and granularization of precipitation surfaces, formation of gypsum crystals with equant and distorted habits, and precipitation of trace carbonate and oxide phases. Fine-scale wrinkle structures visible in clastic surfaces of sabkha environments offered evidence of the biofilm's role in physical sedimentary processes. These findings are highly relevant to astrobiology because they expand and refine the known characteristics of gypsum deposits, including their biological components. Key Words: Analogue- Astrobiology-Biosignatures-Endoliths-Sedimentary environments. © 2009, Mary Ann Liebert, Inc.
KW - Bacteria
KW - Bacterial Physiological Phenomena
KW - Biofilms
KW - Calcium Sulfate
KW - Chemical Precipitation
KW - Ecosystem
KW - Geologic Sediments
KW - Mexico
KW - Microscopy, Electron, Scanning
KW - Minerals
KW - Surface Properties
KW - Bacteria (microorganisms)
KW - Bacteroidetes
KW - calcium sulfate

KW - mineral
KW - article
KW - bacterial phenomena and functions
KW - bacterium
KW - biofilm
KW - chemistry
KW - ecosystem
KW - genetics
KW - Mexico
KW - microbiology
KW - precipitation
KW - scanning electron microscopy
KW - sediment
KW - surface property
SN - 15311074 (ISSN)
C2 - 19968464
LA - English
J2 - Astrobiology
M3 - Article
DB - Scopus
N1 - Export Date: 23 February 2023; Cited By: 24; Correspondence Address: M. B. Vogel; SETI Institute, Mountain View, CA 94043, 415 N. Whisman Rd., United States; email: marilyn.b.vogel@nasa.gov
ER -

TY - CONF
AU - Chung, S.
AU - Ehrenfreund, P.
AU - Rummel, J.
AU - Peter, N.
TI - Bridging the earth science and space exploration communities
PY - 2009
T2 - 60th International Astronautical Congress 2009, IAC 2009
VL - 5
SP - 3971
EP - 3972
UR - https://www.scopus.com/inward/record.uri?eid=2-s2.0-77953637833&partnerID=40&md5=a3faef07cac94cdf2e667751f08a4c98
AD - Space Policy Institute, Washington, DC, United States
AD - East Carolina University, Greenville, NC, United States
AD - European Space Policy Institute, Vienna, Austria
AB - Synergies between space exploration and Earth science do exist that have not been fully recognized and exploited, to date. Protection and understanding of life on Earth, search for extraterrestrial life, and expansion of human presence in outer space all require applications of fundamental knowledge and information about life science. Instrumentation and data systems of Earth observing satellites are based on similar technologies that are used for planetary missions. While planetary surface operations are conducted under different conditions, the investigation of surface and subsurface of all planetary objects, including Earth, uses similar tools, such as radar, seismometers, and drilling devices etc. An interdisciplinary approach is also needed in both fields of Earth science and space exploration to exploit data on

Earth observations, space weather and biodiversity. The Earth observation community has developed some exemplary tools and has featured a successful international cooperation in handling and sharing of data that could equally be applied to robotic space exploration. Both communities suffer from the lack of long-term strategy, adequate funding, and public awareness and support, in addition to being faced with similar challenges in private sector participation, international cooperation, and other policy issues. Sharing of information, knowledge, and experience as well as coordination and collaboration among different communities can help provide answers to some of the challenging questions. To maximize potential synergies different groups and communities (e.g.national/ international, private/public, provider/user, space/non-space, data/ service provider) should be brought together to address the common issues faced by space exploration and Earth science. The establishment of a network among Earth- and space-centric communities is thus proposed, which would enable an exchange of scientific insights and the development of new policies and management strategies. Such a network could provide a vital forum that assists the management of our planet and builds a bridge between the Earth science and space exploration communities.
KW - Biodiversity
KW - Data handling
KW - Earth sciences
KW - International cooperation
KW - Observatories
KW - Space research
KW - Strategic planning
KW - Data systems
KW - Earth observations
KW - Earth observing satellite
KW - Extraterrestrial life
KW - Life-sciences
KW - Long-term strategy
KW - Management strategies
KW - Outer space
KW - Planetary mission
KW - Planetary surfaces
KW - Policy issues
KW - Potential synergies
KW - Private sector participation
KW - Public awareness
KW - Robotic space exploration
KW - Space explorations
KW - Space weather
KW - Earth (planet)
SN - 978-161567908-9 (ISBN)
LA - English
J2 - Int. Astronaut. Cong., IAC
M3 - Conference paper
DB - Scopus
N1 - Export Date: 23 February 2023; Cited By: 0
ER -

TY - CONF
AU - Laan, E.C.
AU - Ahlers, B.
AU - Westrenen, W.V.
AU - Heiligers, J.
AU - Wielders, A.
TI - Moon4You: A combined Raman/LIBS instrument for lunar exploration
PY - 2009
T2 - Proceedings of SPIE - The International Society for Optical Engineering
VL - 7441
C7 - 744114
DO - 10.1117/12.825883
UR - https://www.scopus.com/inward/record.uri?eid=2-s2.0-70449508778&doi=10.1117%2f12.825883&partnerID=40&md5=97128ed39b12fffe6386d186f3b3d689
AD - TNO Science and Industry, Space and Science, 2600 AD Delft, Stieltjesweg 1, Netherlands
AD - VU University Amsterdam, 1081 HV Amsterdam, De Boelelaan 1085, Netherlands
AD - Space Horizon, 2015 LB Haarlem, Duindoornlaan 24, Netherlands
AB - Moon4You is a project led by the Dutch Organisation for Applied Scientific Research TNO, with partners from industry and universities in the Netherlands that aims to provide a combined Raman/LIBS instrument as scientific payload for lunar exploration missions. It is the first time that Raman spectroscopy and LIBS (Laser Induced Breakdown Spectroscopy) are combined into one miniaturised instrument with minimum mass, volume and use of resources and can deliver data-products almost instantly. These characteristics make it the next-generation instrument for mineralogical and elemental (atomic) characterisation of lunar soil and rock samples, as well as for a host of other planetary exploration and terrestrial applications. © 2009 SPIE.
KW - Astrobiology
KW - Instruments
KW - Interplanetary spacecraft
KW - Laser spectroscopy
KW - Lasers
KW - Raman spectroscopy
KW - Spectrometers
KW - Spectrometry
KW - Spectroscopic analysis
KW - Trajectories
KW - Characterisation
KW - Laser induced breakdown spectroscopy
KW - LIBS
KW - Lunar explorations
KW - Lunar soil
KW - Minimum mass
KW - Netherlands
KW - Planetary exploration
KW - Raman
KW - Rock sample

KW - Scientific payloads
KW - Scientific researches
KW - Terrestrial application
KW - Lunar missions
KW - Laser
KW - LIBS
KW - Moon
KW - Raman
KW - Spectrometer
SN - 0277786X (ISSN); 978-081947731-6 (ISBN)
LA - English
J2 - Proc SPIE Int Soc Opt Eng
M3 - Conference paper
DB - Scopus
N1 - Export Date: 23 February 2023; Cited By: 6; Correspondence Address: E. C. Laan; TNO Science and Industry, Space and Science, 2600 AD Delft, Stieltjesweg 1, Netherlands; email: erik.laan@tno.nl; CODEN: PSISD
ER -

TY - JOUR
AU - Mahmoud, D.A.R.
AU - Helmy, W.A.
TI - Potential application of immobilization technology in enzyme and biomass production (Review article)
PY - 2009
T2 - Journal of Applied Sciences Research
VL - 5
IS - 12
SP - 2466
EP - 2476
UR - https://www.scopus.com/inward/record.uri?eid=2-s2.0-77955223020&partnerID=40&md5=4dc86dabaf9b404f2601f8c7eaa99cb3
AD - Chemistry of Natural and Microbial Products Department, National Research Centre, Cairo, Egypt
AB - Immobilization technology creates exciting new opportunities for commercial development and profits in a wide range of industrial sectors including, healthcare and medicine, agriculture and forestry, fine and bulk chemicals production, food technology, fuel and energy production, pollution control and resource recovery. Immobilization technology offers a great deal of hope for solving many of the problems our world faces. This review presents the state of the art of various immobilized biomass applications as well as enzyme immobilization. This review covers the definition and history of immobilization technology, moreover fundamentals of immobilization and focuses on the application of both enzyme and biomass immobilization with special references to those of microbial origin because microbes play numerous key roles in global change, often as silent partners in human activities such as agriculture, mining and water treatment and also microbes are responsible for transforming many of the Earth's most abundant compounds, and thus are central to global changes causing concern. "For much of its history, Earth was a planet of microbes. Even today, microbes dominate the living world in terms of biomass and number". In the

last part of the review, the future developing trend in immobilization technology and the potential applications in astrobiology were also proposed tentatively. © 2009, INSInet Publication.
KW - Enzyme and biomass production
KW - Immobilization technology
SN - 1819544X (ISSN)
LA - English
J2 - J. Appl. Sci. Res.
M3 - Article
DB - Scopus
N1 - Export Date: 23 February 2023; Cited By: 39; Correspondence Address: D.A.R. Mahmoud; Chemistry of Natural and Microbial Products Department, National Research Centre, Cairo, Egypt; email: doaanrc1993@yahoo.com
ER -

TY - CHAP
AU - Priscu, J.C.
AU - Tulaczyk, S.
AU - Studinger, M.
AU - Kennicutt, M.C.
AU - Christner, B.C.
AU - Foreman, C.M.
TI - Antarctic subglacial water: Origin, evolution, and ecology
PY - 2009
T2 - Polar Lakes and Rivers: Limnology of Arctic and Antarctic Aquatic Ecosystems
DO - 10.1093/acprof:oso/9780199213887.003.0007
UR - https://www.scopus.com/inward/record.uri?eid=2-s2.0-84920753665&doi=10.1093%2facprof%3aoso%2f9780199213887.003.0007&partnerID=40&md5=3fcdaa2e59ed0eedc70c0e4bfced2255
AD - Department of Land Resources and Environmental Sciences, Montana State University, Bozeman, 59717, MT, United States
AD - Department of Earth and Planetary Sciences, University of California, Santa Cruz, 95064, CA, United States
AD - Lamont-Doherty Earth Observatory, Columbia University, 61 Route 9W, Palisades, 10964--8000, NY, United States
AD - Texas A and M University, College Station, 77843-1112, TX, United States
AD - Department of Biological Sciences, Louisiana State University, Baton Rouge, 70803, LA, United States
AD - Department of Land Resources and Environmental Sciences, Montana State University, Bozeman, 59717, MT, United States
AB - This chapter provides up-to-date coverage on the geophysical, chemical, and biological properties of the lakes that lie beneath the Antarctic ice cap (subglacial lakes). There are at least 150 lakes beneath this ice cap and many may be connected by networks of subglacial streams and rivers. The most well known of these lakes is Lake Vostok. Recent evidence indicates that subglacial lakes may initiate and maintain rapid ice flow, and should be considered in ice sheet mass balance assessments. The discovery of viable organisms in subglacial environments demonstrates that life has radiated into all aquatic habitats on the planet. Sub-glacial liquid

environments offer an exciting frontier. Their study will provide an improved understanding of the coupling of geological, glaciological, and biological processes in the polar zones. © Oxford University Press 2008. All rights reserved.
KW - Astrobiology
KW - Extreme environments
KW - Ice cap
KW - Ice-sheet mass balance
KW - Lake vostok
KW - Microbial life
KW - Subglacial lakes
KW - Subglacial rivers
PB - Oxford University Press
SN - 978-019170750-6 (ISBN); 978-019921388-7 (ISBN)
LA - English
J2 - Polar Lakes and Rivers: Limnol. of Arct. and Antarct. Aquat. Ecosyst.
M3 - Book chapter
DB - Scopus
N1 - Export Date: 23 February 2023; Cited By: 6
ER -

TY - CONF
AU - Badescu, M.
AU - Sherrit, S.
AU - Jones, J.
AU - Hall, J.
TI - Self-contained harpoon and sample handling device for a remote platform
PY - 2009
T2 - Proceedings of SPIE - The International Society for Optical Engineering
VL - 7292
IS - PART 1
C7 - 72921I
DO - 10.1117/12.815394
UR - https://www.scopus.com/inward/record.uri?eid=2-s2.0-77955677633&doi=10.1117%2f12.815394&partnerID=40&md5=6fa9d6fb5c753b77840f1ca0d50c16b5
AD - Jet Propulsion Laboratory, California Institute of Technology, Pasadena, CA, 4800 Oak Grove Drive, United States
AB - A key objective of the NASA exploration missions is to explore the Solar System and beyond in an implementation that is safe, sustainable and affordable. One of the major enabling technologies for meeting this objective is the development of effective autonomous sampling systems for robotic in-situ analysis and scientific experiments for life and water detection as well as the potential to conduct materials characterization and mineralogy. Rapid sampling techniques with minimum deterioration of the sample and the potential to capture volatiles have long been an objective of the planetary science community. Tethered penetrator sampling whether the penetrator is driven by gravity [Jones et al. 2006], chemical means [Jones et al. 2006], mechanical springs [Backes et al. 2008] or air guns [Lorenz and Shandera 2000] has the potential

to meet this objective. In this paper we present the development of a tethered harpoon sampling and sample handling system operated from an aerial platform for in-situ astrobiological investigations. The harpoon system can be driven into the sample using gravity, pyro, spring or compressed gas mechanisms and is retrieved using a spooling mechanism. The system description and preliminary test results are presented. © 2009 SPIE.
KW - Astronomy
KW - Concrete bridges
KW - Crystallography
KW - Mineralogy
KW - Minerals
KW - NASA
KW - Sensors
KW - Solar system
KW - Aerial platform
KW - Air guns
KW - Airborne sampling
KW - Compressed gas
KW - Enabling technologies
KW - In-situ
KW - In-situ analysis
KW - Key objective
KW - Materials characterization
KW - Mechanical springs
KW - NASA exploration missions
KW - Penetrator
KW - Penetrators
KW - Planetary science
KW - Rapid sampling
KW - Sample handling
KW - Sample handling systems
KW - Sampling systems
KW - System description
KW - Test results
KW - Water detection
KW - Sampling
KW - Airborne sampling
KW - Penetrator
KW - Rapid sampling
SN - 0277786X (ISSN); 978-081947552-7 (ISBN)
LA - English
J2 - Proc SPIE Int Soc Opt Eng
M3 - Conference paper
DB - Scopus
N1 - Export Date: 23 February 2023; Cited By: 1; Correspondence Address: M. Badescu; Jet Propulsion Laboratory, California Institute of Technology, Pasadena, CA, 4800 Oak Grove Drive, United States; email: Mircea.Badescu@jpl.nasa.gov; CODEN: PSISD
ER -

TY - CONF
AU - Kamp, I.
AU - Milosavljević, M.

TI - Stability of organic molecules against shocks in the young Solar nebula
PY - 2009
T2 - AIP Conference Proceedings
VL - 1094
SP - 965
EP - 968
DO - 10.1063/1.3099281
UR - https://www.scopus.com/inward/record.uri?eid=2-s2.0-64849087428&doi=10.1063%2f1.3099281&partnerID=40&md5=babbe58084806a4e95bc6b93eba7fb9e
AD - Kapteyn Astronomical Institute, Groningen, Netherlands
AD - Faculty of Mathematics, University of Belgrade, Serbia
AB - One of the fundamental astrobiology questions is how life has formed in our Solar System. In this context the formation and stability of abiotic organic molecules such as CH4, formic acid and amino acids, is important for understanding how organic material has formed and survived shocks and energetic particle impact from winds in the early Solar System. Shock waves have been suggested as a plausible scenario to create chondrules, small meteoritic components that have been completely molten by energetic events such as shocks and high velocity particle impacts. We study here the formation and destruction of certain gas-phase molecules such as methane and water during such shock events and compare the chemical timescales with the timescales for shocks arising from gravitational instabilities in a protosolar nebula. © 2009 American Institute of Physics.
KW - Chondrules
KW - Protoplanetary disk chemistry
KW - Shocks
SN - 15517616 (ISSN)
LA - English
J2 - AIP Conf. Proc.
M3 - Conference paper
DB - Scopus
N1 - Export Date: 23 February 2023; Cited By: 0
ER -

TY - JOUR
AU - Lammer, H.
AU - Bredehöft, J.H.
AU - Coustenis, A.
AU - Khodachenko, M.L.
AU - Kaltenegger, L.
AU - Grasset, O.
AU - Prieur, D.
AU - Raulin, F.
AU - Ehrenfreund, P.
AU - Yamauchi, M.
AU - Wahlund, J.-E.
AU - Grießmeier, J.-M.
AU - Stangl, G.
AU - Cockell, C.S.
AU - Kulikov, Y.N.
AU - Grenfell, J.L.

AU - Rauer, H.
TI - What makes a planet habitable?
PY - 2009
T2 - Astronomy and Astrophysics Review
VL - 17
IS - 2
SP - 181
EP - 249
DO - 10.1007/s00159-009-0019-z
UR - https://www.scopus.com/inward/record.uri?eid=2-s2.0-67349158305&doi=10.1007%2fs00159-009-0019-z&partnerID=40&md5=0d67e2d9e3078c9869d588959d8a7ed0
AD - Space Research Institute, Austrian Academy of Sciences, Graz, Austria
AD - Institut für Angewandte und Physikalische Chemie, Universität Bremen, Bremen, Germany
AD - Laboratoire d'Etudes Spatiales et d'Instrumentation en Astrophysique (LESIA), Observatoire de Meudon, Meudon, France
AD - Harvard-Smithonian Center for Astrophysics, Cambridge, MA, United States
AD - UMR, Laboratoire de Planétologie et Géodynamique, Nantes, France
AD - Laboratoire de Microbiologie des Environnements Extrêmes, Université de Bretagne Occidentale, Brest, France
AD - Laboratoire Interuniversitaire des Systèmes Atmopshériques, CNRS, Universités Paris 12 et Paris 7, Créteil, France
AD - Astrobiology Group, Leiden Institute of Chemistry, University of Leiden, Leiden, Netherlands
AD - Swedish Institute for Space Physics, Kiruna, Sweden
AD - Swedish Institute for Space Physics, Uppsala, Sweden
AD - ASTRON, Dwingeloo, Netherlands
AD - CEPSAR, Open University, Milton Keynes, United Kingdom
AD - Polar Geophysical Institute, Russian Academy of Sciences, Murmansk, Russian Federation
AD - Zentrum für Astronomie und Astrophysik, Technische Universität Berlin, Berlin, Germany
AD - Deutsches Zentrum für Luft- und Raumfahrt, Institut für Planetenforschung, Berlin, Germany
AB - This work reviews factors which are important for the evolution of habitable Earth-like planets such as the effects of the host star dependent radiation and particle fluxes on the evolution of atmospheres and initial water inventories. We discuss the geodynamical and geophysical environments which are necessary for planets where plate tectonics remain active over geological time scales and for planets which evolve to one-plate planets. The discoveries of methane-ethane surface lakes on Saturn's large moon Titan, subsurface water oceans or reservoirs inside the moons of Solar System gas giants such as Europa, Ganymede, Titan and Enceladus and more than 335 exoplanets, indicate that the classical definition of the habitable zone concept neglects more exotic habitats and may fail to be adequate for stars which are different from our Sun. A classification of four habitat types is proposed. Class I habitats represent bodies on which stellar and geophysical conditions allow Earth-analog planets to evolve so that complex

multi-cellular life forms may originate. Class II habitats includes bodies on which life may evolve but due to stellar and geophysical conditions that are different from the class I habitats, the planets rather evolve toward Venus- or Mars-type worlds where complex life-forms may not develop. Class III habitats are planetary bodies where subsurface water oceans exist which interact directly with a silicate-rich core, while class IV habitats have liquid water layers between two ice layers, or liquids above ice. Furthermore, we discuss from the present viewpoint how life may have originated on early Earth, the possibilities that life may evolve on such Earth-like bodies and how future space missions may discover manifestations of extraterrestrial life. © 2009 Springer-Verlag.
KW - Astrobiology
KW - Earth atmosphere
KW - Extrasolar planets
KW - Geophysics
KW - Interplanetary flight
KW - Moon
KW - Radiation effects
KW - Silicates
KW - Stars
KW - Tectonics
KW - Atmosphere evolution
KW - Earth-like exoplanets
KW - Habitability
KW - Origin of life
KW - Space weather
KW - Sub-surface ocean
KW - Terrestrial planets
KW - Ecosystems
KW - Astrobiology
KW - Atmosphere evolution
KW - Earth-like exoplanets
KW - Habitability
KW - Origin of life
KW - Space weather
KW - Subsurface oceans
KW - Terrestrial planets
PB - Springer Verlag
SN - 09354956 (ISSN)
LA - English
J2 - Astronom Astrophys Rev
M3 - Review
DB - Scopus
N1 - Export Date: 23 February 2023; Cited By: 238; Correspondence Address: H. Lammer; Space Research Institute, Austrian Academy of Sciences, Graz, Austria; email: helmut.lammer@oeaw.ac.at; CODEN: AASRE
ER -

TY - CONF
AU - Oliveira, C.F.
AU - Barufaldi, J.P.
TI - Aliens are us. An innovative course in astrobiology

PY - 2009
T2 - International Journal of Astrobiology
VL - 8
IS - 1
SP - 51
EP - 61
DO - 10.1017/S1473550408004370
UR - https://www.scopus.com/inward/record.uri?eid=2-s2.0-67749095848&doi=10.1017%2fS1473550408004370&partnerID=40&md5=99ce34b39af5f7fb7fe9ba8949d902c1
AD - Center for Science and Mathematics Education, University of Texas at Austin, Austin, TX, United States
AB - We live in a scientific world; paradoxically, the scientific literacy of the population is minimal at best. Science is an ongoing process, a human endeavour; paradoxically, students tend to believe that science is a finished enterprise. Many non-science major students are not motivated in science classes; paradoxically, there is a public fascination with the possibility of life in the Universe, which is nowadays a scientific endeavour. An astrobiology course was developed at the Center for Science and Mathematics Education at The University of Texas at Austin to address these paradoxes and includes the following objectives: (a) to improve scientific literacy; (b) to demonstrate that science is a work in progress; (c) to enhance the inherent interdisciplinary aspect of science; (d) to demonstrate that science is embedded in society and relates with several social sciences; (e) to improve the content knowledge about the nature of science; (f) to illustrate how engaging learning science can be; and (g) to draw from the intrinsic motivation already incorporated in the general population. The course has been offered, taught and revised for the past three years. The informal course student feedback has been very positive and encouraging. The purpose of this paper is to provide a general overview of the course. In addition, the course's background, content, themes and mode of delivery are outlined, discussed and analysed in this paper. This paper subscribes to an educational philosophy that focuses on the multidisciplinary nature of science and includes critical thinking-based teaching strategies using the dynamic discipline of astrobiology. © 2009 Cambridge University Press.
KW - Austin
KW - North America
KW - Texas
KW - United States
KW - astronomy
KW - conference proceeding
KW - curriculum
KW - education
KW - higher education
KW - human geography
KW - interdisciplinary approach
KW - philosophy
KW - student
KW - university sector
KW - Astrobiology

KW - College
KW - Course
KW - Critical thinking
KW - Non-science majors
SN - 14753006 (ISSN)
LA - English
J2 - Int. J. Astrobiology
M3 - Conference paper
DB - Scopus
N1 - Export Date: 23 February 2023; Cited By: 5; Correspondence Address: C.F. Oliveira; Center for Science and Mathematics Education, University of Texas at Austin, Austin, TX, United States; email: carlos.oliveira@mail.utexas.edu
ER -

TY - JOUR
AU - Pollard, W.
AU - Haltigin, T.
AU - Whyte, L.
AU - Niederberger, T.
AU - Andersen, D.
AU - Omelon, C.
AU - Nadeau, J.
AU - Ecclestone, M.
AU - Lebeuf, M.
TI - Overview of analogue science activities at the McGill Arctic Research Station, Axel Heiberg Island, Canadian High Arctic
PY - 2009
T2 - Planetary and Space Science
VL - 57
IS - 5-6
SP - 646
EP - 659
DO - 10.1016/j.pss.2009.01.008
UR - https://www.scopus.com/inward/record.uri?eid=2-s2.0-67349234169&doi=10.1016%2fj.pss.2009.01.008&partnerID=40&md5=f6e95c7bd85e824fa63fd3a95e07fe05
AD - Department of Geography, McGill University, Montreal, Que. H3A 2K6, 805 Sherbrooke St. W, Canada
AD - Department of Natural Resource Sciences, McGill University, Ste. Anne de Bellevue, Que. H9X 3V9, 21111 Lakeshore Blvd, Canada
AD - Carl Sagan Center for the Study of Life in the Universe, SETI Institute, Mountain View, CA 94043, United States
AD - Jackson School of Geosciences, Department of Geological Sciences, The University of Texas at Austin, Austin, TX 78712-0254, 1 University Station, Mail Stop C-1100, United States
AD - Department of Biomedical Engineering, McGill University, Montreal, Que. H3A 2B4, Lyman Duff Medical Building, 3775 University St, Canada
AD - Department of Geography, Trent University, Environmental Sciences Building, Symons Campus, Peterborough, Ont. K9J 7B8, 1600 East Bank Drive, Canada
AD - Department of Space Science, Canadian Space Agency, Saint Hubert, Que. J3Y 8Y9, 6767 Route de l'Aeroport, Canada

AB - The Canadian High Arctic contains several of the highest fidelity Mars analogue sites in the world. Situated at nearly 80° north, Expedition Fjord on Axel Heiberg Island is located within a polar desert climate, with the surrounding landscape and conditions providing an invaluable opportunity to examine terrestrial processes in a cold, dry environment. Through the Canadian Space Agency's Analogue Research Network program, scientific activities based out of the McGill Arctic Research Station (M.A.R.S.) are extremely broad in scope, representing physical, biological, and technological investigations. Some of the most unique hydrogeologic features under investigation near M.A.R.S. are a series of cold saline springs that maintain liquid-state flow year round regardless of air temperature. Previous studies have examined their geomorphic relation to discharge-related formations, water chemistry, temperature monitoring, discharge rates, and combined flow/thermal modeling. Recent investigations have identified microbial communities and characterized biological activity within the springs and within permafrost sections, having direct relevance to astrobiological analogue research goals. Another main thrust of research activities based at M.A.R.S. pertains to the detection, mapping, and quantification of subsurface ice deposits. A long-term study is presently underway examining polygonal terrain, comparing surficial patterns found in the region with those identified on Mars, and using surface morphology to estimate ice wedge volumes through a combination of aerial photography interpretation and ground-based geophysical techniques. Other technological developments include the use of in situ microscopy for the detection of biomarkers and improved permafrost drilling techniques. This paper presents an overview of previous studies undertaken at M.A.R.S. over the past decades and will describe in detail both present and upcoming work.
© 2009 Elsevier Ltd.
KW - Aerial photography
KW - Astrobiology
KW - Drilling
KW - Drilling platforms
KW - Geomorphology
KW - Hydrogeology
KW - Permafrost
KW - Program interpreters
KW - Remote sensing
KW - Technological forecasting
KW - Technology transfer
KW - Axel Heiberg Island
KW - Canada
KW - Mars
KW - Planetary analogues
KW - Polar geomorphology
KW - Landforms
KW - Astrobiology
KW - Axel Heiberg Island
KW - Canada
KW - Mars
KW - Planetary analogues
KW - Polar geomorphology

SN - 00320633 (ISSN)
LA - English
J2 - Planet. Space Sci.
M3 - Article
DB - Scopus
N1 - Export Date: 23 February 2023; Cited By: 32; Correspondence Address: W. Pollard; Department of Geography, McGill University, Montreal, Que. H3A 2K6, 805 Sherbrooke St. W, Canada; email: pollard@geog.mcgill.ca; CODEN: PLSSA
ER -

TY - CONF
AU - Cash, W.
AU - Kendrick, S.
AU - Noecker, C.
AU - Bally, J.
AU - DeMarines, J.
AU - Green, J.
AU - Oakley, P.
AU - Shipley, A.
AU - Benson, S.
AU - Oleson, S.
AU - Content, D.
AU - Folta, D.
AU - Garrison, S.
AU - Gendreau, K.
AU - Hartman, K.
AU - Howard, J.
AU - Hyde, T.
AU - Lakins, D.
AU - Leitner, J.
AU - Leviton, D.
AU - Luquette, R.
AU - Oegerley, B.
AU - Richon, K.
AU - Roberge, A.
AU - Tompkins, S.
AU - Tveekrem, J.
AU - Woodgate, B.
AU - Turnbull, M.
AU - Dailey, D.
AU - Decker, K.
AU - Dehmohseni, R.
AU - Gaugh, B.
AU - Glassman, T.
AU - Haney, M.
AU - Hejal, R.
AU - Lillie, C.
AU - Lo, A.
AU - O'Conner, D.
AU - Oleas, G.
AU - Polidan, R.
AU - Samuele, R.
AU - Shields, S.

AU - Shirvanian, J.
AU - SooHoo, D.
AU - Tinetti, G.
AU - Dorland, B.
AU - Dudik, R.
AU - Gaume, R.
AU - Mason, B.
TI - The new worlds observer: The astrophysics strategic mission concept study
PY - 2009
T2 - Proceedings of SPIE - The International Society for Optical Engineering
VL - 7436
C7 - 743606
DO - 10.1117/12.827486
UR - https://www.scopus.com/inward/record.uri?eid=2-s2.0-77958114379&doi=10.1117%2f12.827486&partnerID=40&md5=981c39cb3e61a78362950379420fe1af
AD - University of Colorado, United States
AD - Ball, United States
AD - CU, United States
AD - GRC
AD - GSFC
AD - GSI
AD - NGC
AD - UCL, United Kingdom
AD - USNO, United States
AB - We present the results of the Astrophysics Strategic Mission Concept Study for the New Worlds Observer (NWO). We show that the use of starshades is the most effective and affordable path to mapping and understanding our neighboring planetary systems, to opening the search for life outside our solar system, while serving the needs of the greater astronomy community. A starshade-based mission can be implemented immediately with a near term program of technology demonstration. © 2009 Copyright SPIE - The International Society for Optical Engineering.
KW - Astrophysics
KW - Optical telescopes
KW - Solar system
KW - Space telescopes
KW - Coronagraphy
KW - Exo-planets
KW - Mission concepts
KW - Planetary system
KW - Search for life
KW - Starshades
KW - Innovation
KW - Astrobiology
KW - Coronagraphy
KW - Exoplanets
SN - 0277786X (ISSN); 978-081947726-2 (ISBN)
LA - English
J2 - Proc SPIE Int Soc Opt Eng
M3 - Conference paper

DB - Scopus
N1 - Export Date: 23 February 2023; Cited By: 36; CODEN: PSISD
ER -

TY - CONF
AU - Kolb, V.M.
AU - Hoover, R.B.
AU - Macko, S.A.
TI - On the search for the amino acids on the lunar surface as it relates to other extraterrestrial bodies
PY - 2009
T2 - Proceedings of SPIE - The International Society for Optical Engineering
VL - 7441
C7 - 744106
DO - 10.1117/12.831232
UR - https://www.scopus.com/inward/record.uri?eid=2-s2.0-70449489669&doi=10.1117%2f12.831232&partnerID=40&md5=1a2e61d8dd40420c35180493d9b3a5fa
AD - Astrobiology Laboratory, NASA/Marshall Space Flight Center, National Space Science and Technology Center, Huntsville, AL, 35805, 320 Sparkman Dr., United States
AD - Department of Environmental Sciences, University of Virginia, Charlottesville, VA 22903, United States
AD - Department of Chemistry, University of Wisconsin-Parkside, Kenosha, WI 53141-2000, United States
AB - The early search for the amino acids in the lunar surface fines indicated such a low amount of the amino acids that it was deemed insignificant, and possibly contamination. Although later studies departed in some ways from the earlier results, they were not pursued. In this paper we critically evaluate the results from the Apollo missions from the new perspective with considerations of the sensitivity of the instrumentation available at the time. We suggest that the relative abundances of amino acids seen in lunar soils are appreciably distinct from terrestrial signatures and are not the result of contamination. We discuss the possible relevance of the lunar results to the findings of the amino acids on the surfaces of other extraterrestrial bodies, such as Mars, as well as a need for further analytical approaches. © 2009 SPIE.
KW - Amination
KW - Amines
KW - Amino acids
KW - Astrobiology
KW - Chromatographic analysis
KW - Ion exchange
KW - Ion exchangers
KW - Mass spectrometers
KW - Mass spectrometry
KW - Meteorites
KW - Moon
KW - Organic acids
KW - Proteins
KW - Carbonaceous meteorites
KW - Cyanobacterial proteins

KW - Gas chromatography-mass spectrometry
KW - Ion-exchange chromatography
KW - Lunar fines
KW - Gas chromatography
KW - Amino acids
KW - Carbonaceous meteorites
KW - Cyanobacterial proteins
KW - Gas chromatography-mass spectrometry
KW - Ion-exchange chromatography
KW - Lunar fines
SN - 0277786X (ISSN); 978-081947731-6 (ISBN)
LA - English
J2 - Proc SPIE Int Soc Opt Eng
M3 - Conference paper
DB - Scopus
N1 - Export Date: 23 February 2023; Cited By: 0; Correspondence Address: V. M. Kolb; Department of Chemistry, University of Wisconsin-Parkside, Kenosha, WI 53141-2000, United States; email: kolb@uwp.edu; CODEN: PSISD
ER -

TY - JOUR
AU - Houtkooper, J.M.
AU - Schulze-Makuch, D.
TI - Possibilities for the detection of hydrogen peroxide-water-based life on Mars by the Phoenix Lander
PY - 2009
T2 - Planetary and Space Science
VL - 57
IS - 4
SP - 449
EP - 453
DO - 10.1016/j.pss.2008.08.018
UR - https://www.scopus.com/inward/record.uri?eid=2-s2.0-63649125675&doi=10.1016%2fj.pss.2008.08.018&partnerID=40&md5=cc853f26f2d76b80eca5134e90b4b3df
AD - Center for Psychobiology and Behavioral Medicine, Justus-Liebig-University of Giessen, 35394 Giessen, Germany
AD - School of Earth and Environmental Sciences, Washington State University, United States
AB - The Phoenix Lander landed on Mars on 25 May 2008. It has instruments on board to explore the geology and climate of subpolar Mars and to explore if life ever arose on Mars. Although the Phoenix mission is not a life detection mission per se, it will look for the presence of organic compounds and other evidence to support or discredit the notion of past or present life. The possibility of extant life on Mars has been raised by a reinterpretation of the Viking biology experiments [Houtkooper, J. M., Schulze-Makuch, D., 2007. A possible biogenic origin for hydrogen peroxide on Mars: the Viking results reinterpreted. International Journal of Astrobiology 6, 147-152]. The results of these experiments are in accordance with life based on a mixture of water and hydrogen peroxide instead of water. The near-surface conditions on Mars would give an evolutionary advantage to organisms employing a mixture of H2O2 and

H2O in their intracellular fluid: the mixture has a low freezing point, is hygroscopic and provides a source of oxygen. The H2O2-H2O hypothesis also explains the Viking results in a logically consistent way. With regard to its compatibility with cellular contents, H2O2 is used for a variety of purposes in terran biochemistry. The ability of the anticipated organisms to withstand low temperatures and the relatively high water vapor content of the atmosphere in the Martian arctic, means that Phoenix will land in an area not inimical to H2O2-H2O-based life. Phoenix has a suite of instruments which may be able to detect the signatures of such putative organisms. © 2008 Elsevier Ltd. All rights reserved.
KW - Astrobiology
KW - Biochemistry
KW - Calorimetry
KW - Hydrogen
KW - Hydrogen peroxide
KW - Mixtures
KW - Organic compounds
KW - Oxidation
KW - Oxygen
KW - Scanning
KW - Water content
KW - Biogenic origins
KW - Cellular contents
KW - Freezing points
KW - High waters
KW - Life detections
KW - Life on mars
KW - Low temperatures
KW - Mars
KW - Near surfaces
KW - Phoenix
KW - Phoenix missions
KW - Scanning calorimetry
KW - Water-based
KW - Water vapor
KW - Hydrogen peroxide-water hypothesis
KW - Mars
KW - Phoenix
KW - Scanning calorimetry
SN - 00320633 (ISSN)
LA - English
J2 - Planet. Space Sci.
M3 - Article
DB - Scopus
N1 - Export Date: 23 February 2023; Cited By: 5; Correspondence Address: J.M. Houtkooper; Center for Psychobiology and Behavioral Medicine, Justus-Liebig-University of Giessen, 35394 Giessen, Germany; email: joophoutkooper@gmail.com; CODEN: PLSSA
ER -

TY - CONF
AU - Sabbatini, M.
TI - Space education and outreach symposium (E1.) - Education

outreach (3.) - Distance learning for ESA human spaceflight
PY - 2009
T2 - 60th International Astronautical Congress 2009, IAC 2009
VL - 10
SP - 8458
EP - 8461
UR - https://www.scopus.com/inward/record.uri?eid=2-s2.0-77953494009&partnerID=40&md5=325538fcb83d11b30d6e511501e30d35
AD - ESA/ESTEC, Noordwijk, Netherlands
AB - In the framework of the Virtual Campus, inaugurated in year 2000 by the Erasmus Centre of the ESA's Directorate of Human Spaceflight (HSF), a number of initiatives and tools have been developed and customised in order to expand the outreach capabilities of the educational and promotional activities of the Directorate. Distance Learning techniques made possible the creation of educational content that is now available to the International Space Station community at large. A number of other tools have been used to expose the large wealth of educational valuable information originated in HSF: lectures from eminent experts, workshops and symposia are available in a novel and multimedia format that can suit almost any educational entity informatics infrastructure. In particular the world first internet based series of lectures in Astrobiology is a good example of how a multi-disciplinary science domain can benefit by this very convenient way to share educational resources across different Institutes, i.e. in this case mainly Astronomy, Biology and Aeronautical Engineering. The lectures were quite innovative in their scope and purpose, addressing the study of the origin, evolution and distribution of life in the universe. Purpose of this paper is to share the experience gained in the implementation of these techniques with other interested parties.
KW - Aerospace engineering
KW - Astrobiology
KW - Learning algorithms
KW - Manned space flight
KW - Multimedia systems
KW - Space stations
KW - Virtual reality
KW - Distance learning
KW - Education outreach
KW - Educational contents
KW - Educational resource
KW - Human spaceflights
KW - Informatics infrastructure
KW - International Space Stations
KW - Internet based
KW - Multi-disciplinary
KW - Multimedia format
KW - Promotional activities
KW - Space education
KW - Virtual campus
KW - Distance education
SN - 978-161567908-9 (ISBN)
LA - English
J2 - Int. Astronaut. Cong., IAC

M3 - Conference paper
DB - Scopus
N1 - Export Date: 23 February 2023; Cited By: 0; Correspondence Address: M. Sabbatini; ESA/ESTEC, Noordwijk, Netherlands; email: massimo.sabbatini@esa.int
ER -

TY - CONF
AU - Retherford, K.D.
AU - Stern, S.A.
AU - Slater, D.C.
AU - Gladstone, G.R.
AU - Davis, M.W.
AU - Parker, J.Wm.
AU - Versteeg, M.H.
AU - Steffl, A.J.
AU - Greathouse, T.K.
AU - Cunningham, N.J.
TI - SwRI's "ALICE" line of ultraviolet spectrographs
PY - 2009
T2 - Proceedings of SPIE - The International Society for Optical Engineering
VL - 7441
C7 - 744111
DO - 10.1117/12.826192
UR - https://www.scopus.com/inward/record.uri?eid=2-s2.0-70449489666&doi=10.1117%2f12.826192&partnerID=40&md5=5ac7092316266c58c3f5575b70b4bc31
AD - Southwest Research Institute, San Antonio, TX 78238, 6220 Culebra Rd., United States
AD - Southwest Research Institute, Boulder, CO 80302, 1050 Walnut St., United States
AB - Southwest Research Institute's (SwRI's) "ALICE" line of ultraviolet spectrographs (UVS) is founded on a lightweight, low power, and highly versatile instrument design. Generally small changes in detector photocathode, pixel size, slit shape, optical coatings, pinhole aperture implementations, and other minor tweaks have enabled a wide variety of applications for the ALICE design, including investigations of comets (Rosetta-ALICE), Pluto (New Horizons-ALICE), the Moon (Lunar Reconnaissance Orbiter (LRO)-Lyman Alpha Mapping Project (LAMP)), and Jupiter (Juno-UVS). ALICE's high capability and our experience with high radiation environment and outer solar system requirements make this UVS a good choice for future planetary mission concepts. © 2009 SPIE.
KW - Astrobiology
KW - Calibration
KW - Moon
KW - Optical coatings
KW - Radiometry
KW - Ultraviolet spectrographs
KW - Ultraviolet spectroscopy
KW - Juno
KW - Lunar reconnaissance orbiter
KW - New horizons

KW - Radiometric
KW - Rosetta
KW - UV spectroscopy
KW - Interplanetary flight
KW - Calibration
KW - Juno
KW - Lunar reconnaissance orbiter
KW - New horizons
KW - Radiometric
KW - Rosetta
KW - UV spectroscopy
SN - 0277786X (ISSN); 978-081947731-6 (ISBN)
LA - English
J2 - Proc SPIE Int Soc Opt Eng
M3 - Conference paper
DB - Scopus
N1 - Export Date: 23 February 2023; Cited By: 5; CODEN: PSISD
ER -

TY - JOUR
AU - Krug, A.Z.
AU - Jablonski, D.
AU - Valentine, J.W.
AU - Roy, K.
TI - Generation of earth's first-order biodiversity pattern
PY - 2009
T2 - Astrobiology
VL - 9
IS - 1
SP - 113
EP - 124
DO - 10.1089/ast.2008.0253
UR - https://www.scopus.com/inward/record.uri?eid=2-s2.0-63049092810&doi=10.1089%2fast.2008.0253&partnerID=40&md5=e4fb1b49f7fab76e2313f9eef1986ce9
AD - Department of Geophysical Sciences, University of Chicago, Chicago, IL, United States
AD - Department of Integrative Biology, Museum of Paleontology, University of California, Berkeley, Berkeley, CA, United States
AD - Section of Ecology, Behavior and Evolution, Division of Biological Sciences, University of California, San Diego, United States
AD - Department of Geophysical Sciences, University of Chicago, Chicago, IL 60637, 5734 South Ellis Ave. HGS 285, United States
AB - The first-order biodiversity pattern on Earth today and at least as far back as the Paleozoic is the latitudinal diversity gradient (LDG), a decrease in richness of species and higher taxa from the equator to the poles. LDGs are produced by geographic trends in origination, extinction, and dispersal over evolutionary timescales, so that analyses of static patterns will be insufficient to reveal underlying processes. The fossil record of marine bivalve genera, a model system for the analysis of biodiversity dynamics over large temporal and spatial scales, shows that an origination and range-expansion gradient plays a major role in generating the

LDG. Peak origination rates and peak diversities fall within the tropics, with range expansion out of the tropics the predominant spatial dynamic thereafter. The origination-diversity link occurs even in a "contrarian" group whose diversity peaks at midlatitudes, an exception proving the rule that spatial variations in origination are key to latitudinal diversity patterns. Extinction rates are lower in polar latitudes (≥60°) than in temperate zones and thus cannot create the observed gradient alone. They may, however, help to explain why origination and immigration are evidently damped in higher latitudes. We suggest that species require more resources in higher latitudes, for the seasonality of primary productivity increases by more than an order of magnitude from equatorial to polar regions. Higher-latitude species are generalists that, unlike potential immigrants, are adapted to garner the large share of resources required for incumbency in those regions. When resources are opened up by extinctions, lineages spread chiefly poleward and chiefly through speciation. Astrobiology 9, 113-124. © 2009 Mary Ann Liebert, Inc. 2009.
KW - Animals
KW - Biodiversity
KW - Biogenesis
KW - Birds
KW - Bivalvia
KW - Earth (Planet)
KW - Extinction, Biological
KW - Geography
KW - Models, Biological
KW - Seawater
KW - Selection Bias
KW - Species Specificity
KW - Bivalvia
KW - sea water
KW - animal
KW - astronomy
KW - biodiversity
KW - biogenesis
KW - biological model
KW - bird
KW - bivalve
KW - classification
KW - epidemiology
KW - geography
KW - review
KW - species difference
KW - species extinction
KW - selection bias
KW - Distribution of complex life
KW - Evolution
KW - Extinction
KW - Latitudinal diversity gradients
KW - Origination
SN - 15311074 (ISSN)
C2 - 19215204
LA - English

J2 - Astrobiology
M3 - Review
DB - Scopus
N1 - Export Date: 23 February 2023; Cited By: 72; Correspondence Address: A. Z. Krug; Department of Geophysical Sciences, University of Chicago, Chicago, IL 60637, 5734 South Ellis Ave. HGS 285, United States; email: akrug@uchicago.edu
ER -

TY - JOUR
AU - Oakley, P.H.H.
AU - Cash, W.
TI - Construction of an earth model: Analysis of exoplanet light curves and mapping the next earth with the new worlds observer
PY - 2009
T2 - Astrophysical Journal
VL - 700
IS - 2
SP - 1428
EP - 1439
DO - 10.1088/0004-637X/700/2/1428
UR - https://www.scopus.com/inward/record.uri?eid=2-s2.0-70449920908&doi=10.1088%2f0004-637X%2f700%2f2%2f1428&partnerID=40&md5=b1194a39a3671a9c6e2a82a9a5fce3af
AD - Center for Astrophysics and Space Astronomy, University of Colorado, Boulder, CO 80309, United States
AB - The orbital light curve of a terrestrial exoplanet will likely contain valuable information about the surface and atmospheric features of the planet, both in its overall shape and hourly variations. We have constructed an empirically based code capable of simulating observations of the Earth from any orientation, at any time of year with continuously updated cloud and snow coverage with a New Worlds Observatory. By simulating these observations over a full orbital revolution at a distance of 10pc we determine that the detection of an obliquity or seasonal terrain change is possible at low inclinations. In agreement with other studies, a 4m New Worlds Observer can accurately determine the rotation rate of the planet at a success rate from ~ 30% to 80% with only 5days of observations depending on the signal to noise of the observations. We also attempt simple inversions of these diurnal light curves to sketch a map of the reflecting planet's surface features. This mapping technique is only successful with highly favorable systems and in particular requires that the cloud coverage must be lower than the Earth's average. Our test case of a 2 M ⊕ planet at 7pc distance with low exo-zodiacal light and 25% cloud coverage produced crude, but successful results. Additionally, with these highly favorable systems NWO may be able to discern the presence of liquid surface water (or other smooth surfaces) though it requires a complex detection available only at crescent phases in high inclination systems. © 2009. The American Astronomical Society. All rights reserved.
KW - Astrobiology
KW - Earth
KW - Instrumentation: miscellaneous

KW - Scattering
KW - Space vehicles
KW - Techniques: photometric
PB - Institute of Physics Publishing
SN - 0004637X (ISSN)
LA - English
J2 - Astrophys. J.
M3 - Article
DB - Scopus
N1 - Export Date: 23 February 2023; Cited By: 52; Correspondence Address: P. H. H. Oakley; Center for Astrophysics and Space Astronomy, University of Colorado, Boulder, CO 80309, United States; email: Phil.Oakley@Colorado.edu
ER -

TY - JOUR
AU - Neish, C.D.
AU - Somogyi, A.
AU - Lunine, J.I.
AU - Smith, M.A.
TI - Low temperature hydrolysis of laboratory tholins in ammonia-water solutions: Implications for prebiotic chemistry on Titan
PY - 2009
T2 - Icarus
VL - 201
IS - 1
SP - 412
EP - 421
DO - 10.1016/j.icarus.2009.01.003
UR - https://www.scopus.com/inward/record.uri?eid=2-s2.0-63749112311&doi=10.1016%2fj.icarus.2009.01.003&partnerID=40&md5=0d03340c1b71631a83d6f68bd7b5ef79
AD - Lunar and Planetary Laboratory, University of Arizona, Tucson, AZ 85721-0092, 1629 E. University Blvd, United States
AD - Department of Chemistry, University of Arizona, Tucson, AZ 85721, United States
AD - Instituto di Fisica dello Spazio Interplanetario INAF-IFSI, 00133 Rome, Italy
AB - Laboratory tholins react rapidly in 13 wt% ammonia-water at low temperature, producing complex organic molecules containing both oxygen and altered nitrogen functional groups. These reactions display first-order kinetics with half-lives between 0.3 and 14 days at 253 K. The reaction timescales are much shorter than the freezing timescales of impact melts and volcanic sites on Titan, providing ample time for the formation of oxygenated, possibly prebiotic, molecules on its surface. Comparing the rates of the hydrolysis reactions in ammonia-water to those measured in pure water [Neish, C.D, Somogyi, A., Imanaka, H., Lunine, J.I., Smith, M.A., 2008a. Astrobiology 8, 273-287], we find that incorporation of oxygen into the tholins is faster in the presence of ammonia. The rate increases could be due to the increased pH of the solution, or to the availability of new reaction pathways made possible by the presence of ammonia. Using labeled 15NH3 water, we find that ammonia does incorporate into some products, and that the reactions with ammonia

are largely independent of those with water. A related study in H2 18O confirms water as the source of the oxygen incorporated into the oxygen containing products. © 2009 Elsevier Inc. All rights reserved.
KW - Prebiotic chemistry
KW - Prebiotic environments
KW - Titan
SN - 10902643 (ISSN)
LA - English
J2 - Icarus
M3 - Article
DB - Scopus
N1 - Export Date: 23 February 2023; Cited By: 49; Correspondence Address: C.D. Neish; Lunar and Planetary Laboratory, University of Arizona, Tucson, AZ 85721-0092, 1629 E. University Blvd, United States; email: cdneish@lpl.arizona.edu; CODEN: ICRSA
ER -

TY - JOUR
AU - Arora, A.K.
TI - Role of metal oxides in chemical evolution: Interaction of ribose nucleotides with alumina
PY - 2009
T2 - Astrobiology
VL - 9
IS - 2
SP - 165
EP - 171
DO - 10.1089/ast.2007.0143
UR - https://www.scopus.com/inward/record.uri?eid=2-s2.0-65249084921&doi=10.1089%2fast.2007.0143&partnerID=40&md5=de5dd2538c6d1df4e217ca860bf3ad15
AD - Department of Chemistry, Indian Institute of Technology Roorkee, Roorkee, India
AD - Department of Chemistry, Indian Institute of Technology, Roorkee 247667 (U.A), India
AB - Interaction of ribonucleotides - namely, 5′-AMP, 5′-GMP, 5′-CMP, and 5′-UMP - with acidic, neutral, and basic alumina has been studied. Purine nucleotides showed higher adsorption on alumina in comparison with pyrimidine nucleotides under acidic conditions. Adsorption data obtained followed Langmuir adsorption isotherm, and Xm and K L values were calculated. On the basis of infrared spectral studies of ribonucleotides, alumina, and ribonucleotide-alumina adducts, we propose that the nitrogen base and phosphate moiety of the ribonucleotides interact with the positive charge surface of alumina. Results of the present study may indicate the importance of alumina in concentrating organic molecules from dilute aqueous solutions in primeval seas in the course of chemical evolution on Earth. Astrobiology 9, 165-171. © 2009 Mary Ann Liebert, Inc. 2009.
KW - Adsorption
KW - Aluminum Oxide
KW - Carbon
KW - Evolution, Chemical
KW - Hydrogen

KW - Hydrogen-Ion Concentration
KW - Nitrogen
KW - Ribonucleotides
KW - Spectrophotometry, Infrared
KW - Spectrophotometry, Ultraviolet
KW - Suspensions
KW - Water
KW - aluminum oxide
KW - carbon
KW - hydrogen
KW - nitrogen
KW - ribonucleotide
KW - water
KW - adsorption
KW - article
KW - astronomy
KW - chemistry
KW - infrared spectrophotometry
KW - pH
KW - suspension
KW - ultraviolet spectrophotometry
KW - Alumina
KW - Chemical evolution
KW - Metal oxides
KW - Origin of life
KW - Ribonucleotides
SN - 15311074 (ISSN)
C2 - 19371159
LA - English
J2 - Astrobiology
M3 - Article
DB - Scopus
N1 - Export Date: 23 February 2023; Cited By: 19; Correspondence Address: Kamaluddin; Department of Chemistry, Indian Institute of Technology, Roorkee 247667 (U.A), India; email: kamalfcy@iitr.ernet.in
ER -

TY - JOUR
AU - Lee, C.-W.
AU - Kim, J.-K.
AU - Moon, E.-S.
AU - Minh, Y.C.
AU - Kang, H.
TI - Formation of glycine on ultraviolet-irradiated interstellar ice-analog films and implications for interstellar amino acids
PY - 2009
T2 - Astrophysical Journal
VL - 697
IS - 1
SP - 428
EP - 435
DO - 10.1088/0004-637X/697/1/428
UR - https://www.scopus.com/inward/record.uri?eid=2-

s2.0-66649089535&doi=10.1088%2f0004-637X%2f697%2f1%2f428&partnerID=40&md5=887a21a9bc8e6a15551d654c2ab3fcb9
AD - Department of Chemistry, Seoul National University, Gwanak-gu, Seoul 151-747, South Korea
AD - Korea Astronomy and Space Science Institute, Yuseong-gu, Daejeon 305-348, 838 Daeduk-daero, South Korea
AB - We report the synthesis of glycine on interstellar ice-analog films composed of water, methylamine (MA), and carbon dioxide under irradiation of ultraviolet (UV) photons. Analysis of the UV-irradiated ice films by in situ mass spectrometric methods revealed glycine and other isomers as photochemical products. Deuterium-labeling experiments were conducted to determine the structures of the photoproducts and to examine their formation pathways. The reactions occur via photocleavages of C-H and N-H bonds in MA, followed by subsequent reactions of the nascent H atom with CO2, leading to the formation of HOCO and then to glycine and carbamic acid. The photochemical synthesis of glycine occurs efficiently at the ice surfaces, and the competing photosynthesis and photodestruction processes can reach a steady-state kinetic balance at an extended UV exposure, maintaining a substantial population level of glycine. The observation suggests that interstellar amino acids can be created on ice grains, and that they can also be stored in the ices by maintaining a kinetic balance under interstellar UV irradiation. As such, the transport of amino acids in interstellar space may be possible without depleting the net abundance of amino acids in the ices but rather increasing the structural diversity of the molecules. © 2009. The American Astronomical Society.
KW - Astrobiology
KW - Astrochemistry
KW - ISM: molecules
KW - Methods: laboratory
KW - Molecular processes
KW - Ultraviolet: ISM
PB - Institute of Physics Publishing
SN - 0004637X (ISSN)
LA - English
J2 - Astrophys. J.
M3 - Article
DB - Scopus
N1 - Export Date: 23 February 2023; Cited By: 58
ER -

TY - JOUR
AU - Skinner Jr., J.A.
AU - Mazzini, A.
TI - Martian mud volcanism: Terrestrial analogs and implications for formational scenarios
PY - 2009
T2 - Marine and Petroleum Geology
VL - 26
IS - 9
SP - 1866
EP - 1878
DO - 10.1016/j.marpetgeo.2009.02.006

UR - https://www.scopus.com/inward/record.uri?eid=2-s2.0-70350026474&doi=10.1016%2fj.marpetgeo.2009.02.006&partnerID=40&md5=13d4d97855ceabe34f73e2fc83ca8245
AD - U.S. Geological Survey, Flagstaff, AZ 86001, Astrogeology Team, 2255 North Gemini Dr., United States
AD - Physics of Geological Processes, University of Oslo, 0316 Oslo, Box 1048, Norway
AB - The geology of Mars and the stratigraphic characteristics of its uppermost crust (mega-regolith) suggest that some of the pervasively-occurring pitted cones, mounds, and flows may have formed through processes akin to terrestrial mud volcanism. A comparison of terrestrial mud volcanism suggests that equivalent Martian processes likely required discrete sedimentary depocenters, volatile-enriched strata, buried rheological instabilities, and a mechanism of destabilization to initiate subsurface flow. We outline five formational scenarios whereby Martian mud volcanism might have occurred: (A) rapid deposition of sediments, (B) volcano-induced destabilization, (C) tectonic shortening, (D) long-term, load-induced subsidence, and (E) seismic shaking. We describe locations within and around the Martian northern plains that broadly fit the geological context of these scenarios and which contain mud volcano-like landforms. We compare terrestrial and Martian satellite images and examine the geological settings of mud volcano provinces on Earth in order to describe potential target areas for piercement structures on Mars. Our comparisons help to evaluate not only the role of water as a functional component of geological processes on Mars but also how Martian mud volcanoes could provide samples of otherwise inaccessible strata, some of which could contain astrobiological evidence.
KW - Astrobiology
KW - Satellites
KW - Sedimentology
KW - Stratigraphy
KW - Analogs
KW - Depocenters
KW - Functional components
KW - Geological process
KW - Geological setting
KW - Mars
KW - Mud volcanism
KW - Mud volcano
KW - Mud volcanoes
KW - Role of water
KW - Satellite images
KW - Seismic shaking
KW - Subsurface Flow
KW - Surface processes
KW - Tectonic shortening
KW - crustal structure
KW - Mars
KW - mud volcano
KW - planetary landform
KW - stratigraphy
KW - volcanism

KW - Volcanoes
KW - Analogs
KW - Astrobiology
KW - Mars
KW - Mud volcano
KW - Surface processes
SN - 02648172 (ISSN)
LA - English
J2 - Mar. Pet. Geol.
M3 - Article
DB - Scopus
N1 - Export Date: 23 February 2023; Cited By: 76; Correspondence Address: J.A. Skinner Jr.; U.S. Geological Survey, Flagstaff, AZ 86001, Astrogeology Team, 2255 North Gemini Dr., United States; email: jskinner@usgs.gov
ER -

TY - JOUR
AU - Sunde, E.P.
AU - Setlow, P.
AU - Hederstedt, L.
AU - Halle, B.
TI - The physical state of water in bacterial spores
PY - 2009
T2 - Proceedings of the National Academy of Sciences of the United States of America
VL - 106
IS - 46
SP - 19334
EP - 19339
DO - 10.1073/pnas.0908712106
UR - https://www.scopus.com/inward/record.uri?eid=2-s2.0-73349109182&doi=10.1073%2fpnas.0908712106&partnerID=40&md5=0e436569be0ddac1dd5025b476b814cd
AD - Department of Biophysical Chemistry, Center for Molecular Protein Science, Lund University, SE-22100 Lund, Sweden
AD - Department of Molecular, Microbial, and Structural Biology, University of Connecticut Health Center, Farmington, CT 06030-3305, United States
AD - Department of Cell and Organism Biology, Lund University, SE-22362 Lund, Sweden
AB - The bacterial spore, the hardiest known life form, can survive in a metabolically dormant state for many years and can withstand high temperatures, radiation, and toxic chemicals. The molecular basis of spore dormancy and resistance is not understood, but the physical state of water in the different spore compartments is thought to play a key role. To characterize this water in situ, we recorded the water 2H and 17O spin relaxation rates in D2O-exchanged Bacillus subtilis spores over a wide frequency range. The data indicate high water mobility throughout the spore, comparable with binary protein-water systems at similar hydration levels. Even in the dense core, the average water rotational correlation time is only 50 ps. Spore dormancy therefore cannot be explained by glass-like quenching of molecular diffusion but may be linked to

dehydration-induced conformational changes in key enzymes. The data demonstrate that most spore proteins are rotationally immobilized, which may contribute to heat resistance by preventing heat-denatured proteins from aggregating irreversibly. We also find that the water permeability of the inner membrane is at least 2 orders of magnitude lower than for model membranes, consistent with the reported high degree of lipid immobilization in this membrane and with its proposed role in spore resistance to chemicals that damage DNA. The quantitative results reported here on water mobility and transport provide important clues about the mechanism of spore dormancy and resistance, with relevance to food preservation, disease prevention, and astrobiology.
KW - Bacillus subtilis
KW - Bacterial Proteins
KW - Cell Membrane Permeability
KW - Hydrogen
KW - Manganese
KW - Oxygen
KW - Spores, Bacterial
KW - Water
KW - Bacillus subtilis
KW - Bacteria (microorganisms)
KW - deuterium oxide
KW - hydrogen
KW - membrane lipid
KW - oxygen 17
KW - water
KW - bacterial protein
KW - manganese
KW - oxygen
KW - spore specific proteins, Bacillus
KW - spore-specific proteins, Bacillus
KW - water
KW - article
KW - Bacillus subtilis
KW - bacterial spore
KW - conformational transition
KW - hydration
KW - nonhuman
KW - physical phase
KW - priority journal
KW - rotation
KW - water flow
KW - water permeability
KW - cell membrane permeability
KW - chemistry
KW - metabolism
KW - Bacillus subtilis
KW - Hydration
KW - Magnetic relaxation dispersion
KW - Spore dormancy
KW - Spore resistance
SN - 10916490 (ISSN)
C2 - 19892742

LA - English
J2 - Proc. Natl. Acad. Sci. U. S. A.
M3 - Article
DB - Scopus
N1 - Export Date: 23 February 2023; Cited By: 118; Correspondence Address: B. Halle; Department of Cell and Organism Biology, Lund University, SE-22362 Lund, Sweden; email: bertil.halle@bpc.lu.se; CODEN: PNASA
ER -

TY - CONF
AU - Winfield, A.F.T.
TI - Towards an engineering science of robot foraging
PY - 2009
T2 - Distributed Autonomous Robotic Systems 8
SP - 185
EP - 192
DO - 10.1007/978-3-642-00644-9_16
UR - https://www.scopus.com/inward/record.uri?eid=2-s2.0-77951675950&doi=10.1007%2f978-3-642-00644-9_16&partnerID=40&md5=90ed9c207a7245b6178432be47d8a90a
AD - Bristol Robotics Laboratory, University of the West of England, Bristol, United Kingdom
AB - Foraging is a benchmark problem in robotics - especially for distributed autonomous robotic systems. The systematic study of robot foraging is important for several reasons: firstly, because foraging is a metaphor for the broad class of problems integrating robotic exploration, navigation and object identification, manipulation and transport; secondly, in multi-robot systems foraging is a canonical problem for the study of robot-robot cooperation; and thirdly, many and diverse actual or potential real-world applications for robotics are instances of foraging robots, for example, for cleaning, harvesting, search and rescue, landmine clearance or planetary astrobiology. This paper sets out a theoretical framework, structured upon an abstract model and taxonomy of robot foraging. A framework which, it is hoped, might provide the basis of a principled approach to the engineering of future real-world robot foraging systems.
KW - Agricultural robots
KW - Landmine detection
KW - Multipurpose robots
KW - Bench-mark problems
KW - Canonical problems
KW - Distributed autonomous robotic system
KW - Engineering science
KW - Multi-robot systems
KW - Object identification
KW - Robotic explorations
KW - Theoretical framework
KW - Robotics
PB - Springer Publishing Company
SN - 978-364200643-2 (ISBN)
LA - English
J2 - Distrib. Auton. Rob. Syst.

M3 - Conference paper
DB - Scopus
N1 - Export Date: 23 February 2023; Cited By: 19; Correspondence Address: A.F.T. Winfield; Bristol Robotics Laboratory, University of the West of England, Bristol, United Kingdom; email: Alan.Winfield@uwe.ac.uk
ER -

TY - JOUR
AU - Fortes, A.D.
AU - Wood, I.G.
AU - Dobson, D.P.
AU - Fewster, P.F.
TI - An icy mineralogy package (IMP) for in-situ studies of Titan's surface
PY - 2009
T2 - Advances in Space Research
VL - 44
IS - 1
SP - 124
EP - 137
DO - 10.1016/j.asr.2008.11.024
UR - https://www.scopus.com/inward/record.uri?eid=2-s2.0-67349183241&doi=10.1016%2fj.asr.2008.11.024&partnerID=40&md5=84b9825483d7696197f6426b90ac27c1
AD - The Centre for Planetary Sciences at UCL/Birkbeck, London, WC1E 6BT, Gower Street, United Kingdom
AD - Department of Earth Sciences, University College London, London, WC1E 6BT, Gower Street, United Kingdom
AD - PANalytical Research Centre, The Sussex Innovation Centre, Brighton, BN1 9SB, United Kingdom
AB - We describe the scientific case for and preliminary design of an instrument whose primary goal is to determine the chemistry (element abundance) and mineralogy (compound identity and abundance) of Titan's surface using a combination of energy dispersive X-ray fluorescence spectroscopy (EDXRF) and X-ray diffraction (XRD). XRD is capable of identifying any crystalline substance present on Titan's surface at relative abundances greater than ~1 wt%, allowing unambiguous identification of, for example, structure I and II clathrates (even in the presence of ice), and various organic solids, which may include C
2 H
2 , C 2
H 4 , C
4 H
2 , HCN, CH
3 CN, HC 3
N, and C 4 N
2). The XRF component of the instrument will obtain elemental abundances for 16 < Z < 60 with minimum detection limits better than 10 ppm (including detection of atmospheric noble gas isotopes), and may achieve detection limits of 0.01-1% for lighter elements down to Z = 6 (carbon). The instrument is well suited to integration with other analytical tools as part of

a light-weight surface chemistry and mineralogy package. Although considerably less sensitive to elemental abundance than GC-MS (10^{-2} vs. 10^{-8}) it is likely to be significantly lighter (<0.5 kg vs. 10 kg). The instrument will be able to address the origin and evolution of Titan by measuring the mineralogy and chemistry of bulk crust (water ice/clathrates), excavated impact ejecta (possibly including XRD-identifiable high-pressure polymorphs of ice), and cryolava or ash (distinguishing between ammonia-methanol and sulfate-carbonate magmas). The instrument will also address atmospheric photochemistry and climate history by measuring the mineralogy and chemistry of organic sediments found in various environments, such as dunes, fluvial and peri-lacustrine areas (including possible evaporites), and possibly exhumed from deep reservoirs by mud volcanism. Lastly, the instrument will address Titan's astrobiological evolution by enabling detection of more complex organic materials (crystalline carboxylic/amino acids or sugars) in areas where sediments have interacted with aqueous solutions. © 2009 COSPAR.
KW - Chemical analysis
KW - Crystalline materials
KW - Energy dispersive spectroscopy
KW - Ethylene
KW - Fluorescence
KW - Fluorescence spectroscopy
KW - Ice
KW - Inert gases
KW - Mineralogy
KW - Minerals
KW - Sediments
KW - Solutions
KW - Sulfur compounds
KW - Surface chemistry
KW - X ray diffraction
KW - Atmospheric photochemistry
KW - Element abundances
KW - Elemental abundance
KW - Energy dispersive X-ray fluorescence
KW - High-pressure polymorph
KW - Minimum detection limits
KW - Titan
KW - X ray fluorescence
KW - Atmospheric chemistry
KW - Mineralogy
KW - Titan
KW - X-ray diffraction
KW - X-ray fluorescence
PB - Elsevier Ltd
SN - 02731177 (ISSN)
LA - English
J2 - Adv. Space Res.
M3 - Article
DB - Scopus
N1 - Export Date: 23 February 2023; Cited By: 7; Correspondence

Address: A.D. Fortes; The Centre for Planetary Sciences at UCL/ Birkbeck, London, WC1E 6BT, Gower Street, United Kingdom; email: andrew.fortes@ucl.ac.uk; CODEN: ASRSD
ER -

TY - CHAP
AU - Lins, U.
AU - Bazylinski, D.A.
TI - Magnetotaxis
PY - 2009
T2 - Encyclopedia of Microbiology
SP - 229
EP - 241
DO - 10.1016/B978-012373944-5.00103-6
UR - https://www.scopus.com/inward/record.uri?eid=2-s2.0-85021265094&doi=10.1016%2fB978-012373944-5.00103-6&partnerID=40&md5=57f7df14e7962832af432321b3e21e17
AD - Universidade Federal do Rio de Janeiro, Rio de Janeiro, Brazil
AD - University of Nevada at Las Vegas, Las Vegas, NV, United States
AB - The most conspicuous tactic behavior of bacteria other than chemo- and phototaxis is magnetotaxis. Magnetotactic bacteria are Gram-negative, motile prokaryotes that synthesize intracellular crystals of magnetite and/or iron sulfide minerals (greigite and precursors). Each crystal is enveloped by a lipid-bilayer membrane and comprises the organelle referred to as the magnetosome. Magnetosomes are usually organized in chains within the cell and cause the bacterium to orient and migrate along geomagnetic field lines, propelled by flagella. Magnetotactic bacteria are indigenous in sediments or water columns where they occur predominantly at the oxic-anoxic interface and the anoxic zone. They represent a diverse group of microorganisms with respect to morphology, physiology, and phylogeny. Many potential biotechnological applications of magnetosomes and their magnetic crystals have been recognized. © 2009 Elsevier Inc. All rights reserved.
KW - Astrobiology
KW - Magneto-aerotaxis
KW - Magnetosome
KW - Magnetospirillum
KW - Magnetotactic bacteria
KW - Magnetotaxis
KW - Redoxtaxis
PB - Elsevier Inc.
SN - 978-012373944-5 (ISBN)
LA - English
J2 - Encycl. of Microbiol.
M3 - Book chapter
DB - Scopus
N1 - Export Date: 23 February 2023; Cited By: 3
ER -

TY - CHAP
AU - James, K.
AU - Joseph, K.

TI - Evolution of a habitable planet
PY - 2009
T2 - Frontiers of Astrobiology
SP - 115
EP - 131
DO - 10.1017/CBO9780511902574.010
UR - https://www.scopus.com/inward/record.uri?eid=2-s2.0-84924197444&doi=10.1017%2fCBO9780511902574.010&partnerID=40&md5=7898e0694807b48a4f8232108616f1c7
AD - Department of Geoscience, Penn State University Park, 443 Deike Building, 16802, PA, United States
AD - Division of Geological and Planetary Sciences, California Institute of Technology, Pasadena, 91125, CA, United States
AB - We saw in the previous chapter that Earth developed a climate that supported liquid water at its surface very early in its history, probably within the first few hundred million years. That was good for life, of course, because all life that we know about on Earth requires liquid water at least episodically. There are good chemical reasons for thinking that this requirement might be universal, some of which were discussed earlier in this volume. Here we are concerned with a somewhat later stage in Earth's history, starting from when the rock record begins, around 3.8–4 billion years ago, or 3.8–4 Gyr ago, and continuing on until the rise of atmospheric oxygen, around 2.3 Gyr ago. This time interval overlaps almost precisely with the geologic time period called the Archean Eon. Although not formally defined as such, the beginning of the Archean corresponds with the beginning of the rock record, as marked by the oldest dated fragments of continental crust at Earth's surface. We know that Earth had older rocks, based on crystals of the mineral, zircon, which survive today as sand grains in mid-Archean quartzites from Western Australia, and can be dated as far back as 4.4 Gyr ago (Valley et al. 2002). Defining the end of Archean time to be precisely 2.5 Gyr ago was a somewhat arbitrary decision of the geological community: at about this time, the dominant type of sedimentary basin switches from the granite-greenstone belt configuration (dominated by ultramafic, magnesium-rich volcanics and chemically immature sedimentary rocks) to basins controlled by thermal subsidence along passive margins. This switch in rock types may or may not have influenced the rise of O2, as discussed later in this chapter. We will henceforth use the term “Archean” to refer to the time period preceding the rise of O2, recognizing that astrobiological and geological terminologies have slightly different meanings. © Cambridge University Press 2012.
PB - Cambridge University Press
SN - 978-051190257-4 (ISBN); 978-110700641-6 (ISBN)
LA - English
J2 - Frontiers of Astrobiology
M3 - Book chapter
DB - Scopus
N1 - Export Date: 23 February 2023; Cited By: 0
ER -

TY - CONF
AU - Koudelka, O.

AU - Kropiunig, C.
AU - Greschitz, E.
AU - Plucinski, W.
AU - Blanzano, B.
AU - Finsterbusch, R.
AU - Preud'Homme, F.
AU - Rosiers, P.
AU - Kufner, E.
AU - Melville, N.
TI - Implementation of the miller-urey experiment in space
PY - 2009
T2 - 60th International Astronautical Congress 2009, IAC 2009
VL - 1
SP - 255
EP - 262
UR - https://www.scopus.com/inward/record.uri?eid=2-s2.0-77953640371&partnerID=40&md5=104f3667a4ec2e8a6227e5a39718c169
AD - Institute of Applied Systems Technology, Joanneum Research, Graz, Austria
AD - Institute of Communication Networks and Satellite Communications, Graz University of Technology, Austria
AD - Verhaert Space, Belgium
AD - European Space Agency, United States
AB - Prof. Stanley Miller and Prof. Harold C. Urey demonstrated already in the 1950s that amino acids, chemical building blocks of life, can be created from gases that were at the time assumed to be the major components of the early Earth atmosphere. The existence of such complex molecules outside the Earth environment has been verified by astronomical observations and analysis of meteorites and most recently in cometary samples returned to Earth by the Stardust mission. A potential environment in which such molecules could be synthesized in space is the so-called solar nebula. Under a contract by European Space Agency Joanneum Research (Graz, Austria), Graz University of Technolgy (Austria) and Verhaert Space (Belgium) are developing a Miller-Urey-type experiment which will be carried out in the microgravity environment of the International Space Station ISS in 2010. It is aimed at the investigation of prebiotic chemical pathways for the synthesis of organic compounds in the solar nebula environment. Solid silica particles serve as surfaces on which thin mantles of water ices should form. These ice-covered particles move through a spark discharge region stimulating chemical reactions similar to the original Miller-Urey-Experiment. The chemical reactions take place in pairs of cooled vials which are filled with different gas mixtures, also similar to the mixtures used by Miller in his original experiments. The vials will be transported back to Earth after completion of the experiment on ISS for analysis in the laboratories of the Science Team. The paper gives an overview of the project and describes the hardware of the Miller-Urey experiment in space. Measurement results obtained during parabolic flight campaigns, drop tower and laboratory tests as well as the operations of the experiment are described.
KW - Amino acids
KW - Chemical compounds
KW - Earth atmosphere

KW - Manned space flight
KW - Organic acids
KW - Silica
KW - Space stations
KW - Synthesis (chemical)
KW - Astronomical observation
KW - Austria
KW - Belgium
KW - Chemical building blocks
KW - Chemical pathways
KW - Complex molecules
KW - Drop towers
KW - Early Earth atmosphere
KW - Earth environment
KW - European Space Agency
KW - International Space Station ISS
KW - International Space Stations
KW - Laboratory test
KW - Measurement results
KW - Microgravity environments
KW - Parabolic flight
KW - Prebiotics
KW - Solar nebula
KW - Solid silica
KW - Space experiment
KW - Space experiments
KW - Spark discharge
KW - Water ice
KW - Experiments
KW - Astrobiology
KW - International Space Station ISS
KW - Miller-Urey experiment
KW - Space experiment
SN - 978-161567908-9 (ISBN)
LA - English
J2 - Int. Astronaut. Cong., IAC
M3 - Conference paper
DB - Scopus
N1 - Export Date: 23 February 2023; Cited By: 0
ER -

TY - CONF
AU - Konesky, G.
TI - The drake equation revisited
PY - 2009
T2 - Proceedings of SPIE - The International Society for Optical Engineering
VL - 7441
C7 - 74410W
DO - 10.1117/12.827406
UR - https://www.scopus.com/inward/record.uri?eid=2-s2.0-70449589631&doi=10.1117%2f12.827406&partnerID=40&md5=f8a3a2e472fe4ff43050711f749eb698
AD - K-Plasma, Ltd., Hampton Bays, NY 11946, 3 Rolling Hill Rd.,

United States
AB - In the almost half century since the Drake Equation was first conceived, a number of profound discoveries have been made that require each of the seven variables of this equation to be reconsidered. The discovery of hydrothermal vents on the ocean floor, for example, as well as the ever-increasing extreme conditions in which life is found on Earth, suggest a much wider range of possible extraterrestrial habitats. The growing consensus that life originated very early in Earth's history also supports this suggestion. The discovery of exoplanets with a wide range of host star types, and attendant habitable zones, suggests that life may be possible in planetary systems with stars quite unlike our Sun. Stellar evolution also plays an important part in that habitable zones are mobile. The increasing brightness of our Sun over the next few billion years, will place the Earth well outside the present habitable zone, but will then encompass Mars, giving rise to the notion that some Drake Equation variables, such as the fraction of planets on which life emerges, may have multiple values.
© 2009 SPIE.
KW - Astrobiology
KW - Hot springs
KW - Oceanography
KW - Radio astronomy
KW - Telecommunication
KW - Drake equation
KW - Emergence of intelligence
KW - Extrasolar planets
KW - Extremophiles
KW - Habitable zones
KW - Interstellar communication
KW - Time-integrated view
KW - Planets
KW - Drake equation
KW - Emergence of intelligence
KW - Extrasolar planets
KW - Extremophiles
KW - Habitable zones
KW - Interstellar communication
KW - Time-integrated view
SN - 0277786X (ISSN); 978-081947731-6 (ISBN)
LA - English
J2 - Proc SPIE Int Soc Opt Eng
M3 - Conference paper
DB - Scopus
N1 - Export Date: 23 February 2023; Cited By: 0; Correspondence Address: G. Konesky; K-Plasma, Ltd., Hampton Bays, NY 11946, 3 Rolling Hill Rd., United States; email: g.konesky@att.net; CODEN: PSISD
ER -

TY - CONF
AU - Rice, C.V.
AU - Harrison, W.
AU - Kirkpatrick, K.

AU - Brown, E.D.
TI - Cryoprotection from bacterial teichoic acid
PY - 2009
T2 - Proceedings of SPIE - The International Society for Optical Engineering
VL - 7441
C7 - 74410M
DO - 10.1117/12.829981
UR - https://www.scopus.com/inward/record.uri?eid=2-s2.0-70449474734&doi=10.1117%2f12.829981&partnerID=40&md5=15557ad8aad5411783df59db85395a01
AD - Department of Chemistry and Biochemistry, University of Oklahoma, Norman, OK 73019, 620 Parrington Oval, United States
AD - Department of Biochemistry and Biomedical Sciences, McMaster University, Hamilton, ON L8N 3Z5, 1200 Main Street West, Canada
AB - Recent studies from our lab demonstrated that teichoic acid is surrounded by liquid water at -40 °C. The size and shape of the liquid water pockets has been visualized with fluorescence microscopy images of aqueous Rhodamine- B solutions. The long, thin channels surround ice crystals with a size of 5-20 microns. Subsequent studies show that B. subtilis Gram-positive bacteria are sequestered into large pockets without added teichoic acid. Here, the ice crystals are orders of manitude larger. When bacteria are mixed with teichoic acid solutions, the distribution of bacteria changes dramatically. The smaller ice crystals allow the bacteria to align in the thin channels of liquid water seen with teichoic acid only. The role of teichoic acid in the freeze tolerance was examined with live/dead fluorescence assays of bacteria mixed with teichoic acid. These quantitative assays were used to determine if teichoic acid acts in a synergetic fashion to enhance the survivability of E. coli, a gram-negative species which lacks teichoic acid. Additionally, we have obtained B. subtilis mutants lacking wall-associated teichoic acids to evaluate cryoprotection compared to the wild-type strain. © 2009 SPIE.
KW - Astrobiology
KW - Bacteria
KW - Bacteriology
KW - Crystals
KW - Escherichia coli
KW - Fluorescence
KW - Fluorescence microscopy
KW - Fluorescence spectroscopy
KW - Ice
KW - Liquids
KW - Cryoprotectant
KW - Extremophile
KW - Lipoteichoic acid
KW - Peptidoglycan
KW - Psychrophile
KW - Teichoic acid
KW - Wall teichoic acid
KW - Acids
KW - Bacteria
KW - Cryoprotectant

KW - Extremophile
KW - Fluorescence spectroscopy
KW - Ice
KW - Lipoteichoic acid
KW - Peptidoglycan
KW - Psychrophile
KW - Teichoic acid
KW - Wall teichoic acid
SN - 0277786X (ISSN); 978-081947731-6 (ISBN)
LA - English
J2 - Proc SPIE Int Soc Opt Eng
M3 - Conference paper
DB - Scopus
N1 - Export Date: 23 February 2023; Cited By: 2; CODEN: PSISD
ER -

TY - JOUR
AU - Cooper, J.F.
AU - Cooper, P.D.
AU - Sittler, E.C.
AU - Sturner, S.J.
AU - Rymer, A.M.
TI - Old Faithful model for radiolytic gas-driven cryovolcanism at Enceladus
PY - 2009
T2 - Planetary and Space Science
VL - 57
IS - 13
SP - 1607
EP - 1620
DO - 10.1016/j.pss.2009.08.002
UR - https://www.scopus.com/inward/record.uri?eid=2-s2.0-70449102704&doi=10.1016%2fj.pss.2009.08.002&partnerID=40&md5=08ad07fc2c84d0dc428595d7dd1bad88
AD - Heliospheric Physics Laboratory, NASA Goddard Space Flight Center, Greenbelt, MD 20771, 8800 Greenbelt Road, United States
AD - Department of Chemistry and Biochemistry, George Mason University, Fairfax, VA 22030, 4400 University Drive, United States
AD - Geospace Physics Laboratory, NASA Goddard Space Flight Center, Greenbelt, MD 20771, Code 673, United States
AD - CRESST, Astroparticle Physics Laboratory, NASA Goddard Space Flight Center, Greenbelt, MD 20771, Code 661, United States
AD - Applied Physics Laboratory, Johns Hopkins Laboratory, Laurel, MD 20723-6005, 11100 Johns Hopkins Road, United States
AB - A new model is presented on how chemically driven cryovolcanism might contribute to episodic outgassing at the icy moon Enceladus and potentially elsewhere including Europa and Kuiper Belt Objects. Exposed water ices can become oxidized from radiolytic chemical alteration of near-surface water ice by space environment irradiation. In contact with primordially abundant reductants such as NH3, CH4, and other hydrocarbons, the product oxidants can react exothermically to produce volatile gases driving cryovolcanism via gas-piston forces on any subsurface liquid reservoirs. Radiolytic oxidants such as H2O2 and O2 can continuously accumulate deep in icy

regoliths and be conveyed by rheological flows to subsurface chemical reaction zones over million-year time scales indicated by cratering ages for active regions of Enceladus and Europa. Surface blanketing with cryovolcanic plume ejecta would further accelerate regolith burial of radiolytic oxidants. Episodic heating from transient gravitational tides, radioisotope decay, impacts, or other geologic events might occasionally accelerate chemical reaction rates and ignite the exothermic release of cumulative radiolytic oxidant energy. The time history for the suggested "Old Faithful" model of radiolytic gas-driven cryovolcanism at Enceladus and elsewhere therefore consists of long periods of chemical energy accumulation punctuated by much briefer episodes of cryovolcanic activity. The most probable sequence for detection of activity in the current epoch is a long evolutionary phase of slow but continuous oxidant accumulation over billions of years followed by continuous but variable high activity over the past 107-108 years. Detectable cryovolcanic activity could then later decline due to near-total oxidation of the rheologically accessible ice crust and depletion the accessible reductant abundances, as may have already occurred for Europa in the more intense radiation environment of Jupiter's magnetosphere. Astrobiological potential of Enceladus could correspondingly be higher than at Europa due to a less extreme state of oxidation and greater residual abundance of organics.
KW - Hydrocarbons
KW - Magnetosphere
KW - Newtonian flow
KW - Oxidants
KW - Oxidation
KW - Petroleum tar
KW - Radiation chemistry
KW - Radioisotopes
KW - Reaction rates
KW - Solar system
KW - Active regions
KW - Chemical alteration
KW - Chemical energy
KW - Cryovolcanism
KW - Enceladus
KW - Energetic particles
KW - Evolutionary phase
KW - High activity
KW - Icy Moons
KW - Jupiter's magnetosphere
KW - Kuiper belt objects
KW - Near-surface
KW - New model
KW - Organics
KW - Radiation environments
KW - Reductants
KW - Rheological flow
KW - Space environment
KW - Subsurface liquid
KW - Time history
KW - Time-scales

KW - Total oxidation
KW - Volatile gas
KW - Water ice
KW - Radiolysis
KW - Cryovolcanism
KW - Enceladus
KW - Energetic particles
KW - Oxidants
KW - Radiolysis
KW - Water
SN - 00320633 (ISSN)
LA - English
J2 - Planet. Space Sci.
M3 - Article
DB - Scopus
N1 - Export Date: 23 February 2023; Cited By: 31; Correspondence Address: J.F. Cooper; Heliospheric Physics Laboratory, NASA Goddard Space Flight Center, Greenbelt, MD 20771, 8800 Greenbelt Road, United States; email: John.F.Cooper@nasa.gov; CODEN: PLSSA
ER -

TY - JOUR
AU - Yang, Y.
AU - Yokobori, S.-i.
AU - Yamagishi, A.
TI - Bacterial survival in response to desiccation and high humidity at above zero and subzero temperatures
PY - 2009
T2 - Advances in Space Research
VL - 43
IS - 8
SP - 1285
EP - 1290
DO - 10.1016/j.asr.2009.01.018
UR - https://www.scopus.com/inward/record.uri?eid=2-s2.0-62649135989&doi=10.1016%2fj.asr.2009.01.018&partnerID=40&md5=8c806f0a6dd6104129a8023d7a3e49fb
AD - Department of Molecular Biology, Tokyo University of Pharmacy and Life Science, Hachioji, Tokyo, 192-0392, 1432-1, Horinouchi, Japan
AB - Earthly microorganisms might have contaminated Mars for millions of years by intellectual activities or natural transfer. Knowledge on the preservation of microorganisms may help our searching for life on outer planets, particularly Mars-contaminated earthly microorganisms at ancient time. Extreme dryness is one of the current Mars characteristics. However, a humid or watery Mars at earlier time was suggested by evidence accumulated in recent decades. It raises the question that whether water helps preservation of the microorganisms or not, particularly those with high possibility of interplanetary transfer like spores and Deinococci. In this study, we examined the effects of desiccation and high humidity on survival and DNA double strand breaks (DSB) of Escherichia coli, Deinococcus radiodurans and spores of Bacillus pumilus at 25, 4 and -70 °C. They exhibited different survival rates

and DSB patterns under desiccation and high humidity. Higher survival and less DSB occurred at lower temperature. We suggest that some Mars-contaminated bacteria might have been viably preserved on cold Mars regions for long periods, regardless of water availability. It is more likely to find ancient spores than ancient Deinococci on Mars. In our search for preserved extraterrestrial life, priority should be given to the Mars Polar Regions. © 2009 COSPAR.
KW - Bacteriology
KW - Escherichia coli
KW - Interplanetary spacecraft
KW - Planets
KW - Temperature
KW - Wood preservation
KW - Bacterial survival
KW - Deinococcus radiodurans
KW - Desiccation
KW - DNA double strand breaks
KW - High humidity
KW - Intellectual activities
KW - Interplanetary transfers
KW - Mars
KW - Driers (materials)
KW - Bacterial survival
KW - Desiccation
KW - High humidity
KW - Mars
KW - Preservation
KW - Temperature
PB - Elsevier Ltd
SN - 02731177 (ISSN)
LA - English
J2 - Adv. Space Res.
M3 - Article
DB - Scopus
N1 - Export Date: 23 February 2023; Cited By: 11; Correspondence Address: A. Yamagishi; Department of Molecular Biology, Tokyo University of Pharmacy and Life Science, Hachioji, Tokyo, 192-0392, 1432-1, Horinouchi, Japan; email: yamagish@ls.toyaku.ac.jp; CODEN: ASRSD
ER -

TY - JOUR
AU - Naganuma, T.
TI - An astrobiological view on sustainable life
PY - 2009
T2 - Sustainability
VL - 1
IS - 4
SP - 827
EP - 837
DO - 10.3390/su1040827
UR - https://www.scopus.com/inward/record.uri?eid=2-s2.0-84866420569&doi=10.3390%2fsu1040827&partnerID=40&md5=37b9634c9b

befd8ebec44a00c7326abd
AD - Graduate School of Biosphere Science, Hiroshima University, Higashi-Hiroshima, 739-8528, Japan
AB - Life on a global biosphere basis is substantiated in the form of organics and organisms, and defined as the intermediate forms (briefly expressed as CH2O) hovering between the reduced (CH4, methane) and (CO2, carbon dioxide) ends, different from the classical definition of life as a complex organization maintaining ordered structure and information. Both definitions consider sustenance of life meant as protection of life against chaos through an input of external energy. The CH2O-life connection is maintained as long as the supply of H and O lasts, which is in turn are provided by the splitting of the water molecule H2O. Water is split by electricity, as well-known from school-level experiments, and by solar radiation and geothermal heat on a global scale. In other words, the Sun's radiation and the Earth's heat as well as radioactivity split water to supply H and O for continued existence of life on the Earth. These photochemical, radiochemical and geothermal processes have influences on the evolution and current composition of the Earth's atmosphere, compared with those of Venus and Mars, and influences on the planetary climatology. This view of life may be applicable to the "search-for-life in space" and to sustainability assessment of astrobiological habitats. © 2009 by the authors.
KW - Entropy
KW - Geothermal
KW - Photochemical
KW - Radiochemical
KW - Reduction-oxidation
KW - Split of water
PB - MDPI
SN - 20711050 (ISSN)
LA - English
J2 - Sustainability
M3 - Review
DB - Scopus
N1 - Export Date: 23 February 2023; Cited By: 1; Correspondence Address: T. Naganuma; Graduate School of Biosphere Science, Hiroshima University, Higashi-Hiroshima, 739-8528, Japan; email: takn@hiroshima-u.ac.jp
ER -

TY - JOUR
AU - Dressing, C.D.
AU - Spiegel, D.S.
AU - Scharf, C.A.
AU - Menou, K.
AU - Raymond, S.N.
TI - Habitable climates: The influence of eccentricity
PY - 2010
T2 - Astrophysical Journal
VL - 721
IS - 2
SP - 1295

EP - 1307
DO - 10.1088/0004-637X/721/2/1295
UR - https://www.scopus.com/inward/record.uri?eid=2-s2.0-78149278456&doi=10.1088%2f0004-637X%2f721%2f2%2f1295&partnerID=40&md5=e91010d61298efe1b79ef4c5ec86276f
AD - Department of Astrophysical Sciences, Princeton University, Peyton Hall, Princeton, NJ 08544, United States
AD - Kavli Institute for Theoretical Physics, UCSB, Santa Barbara, CA 93106-4030, United States
AD - Columbia Astrobiology Center, Columbia Astrophysics Laboratory, Columbia University, New York, NY 10027, 550 West 120th Street, United States
AD - Department of Astronomy, Columbia University, New York, NY 10027, 550 West 120th Street, United States
AD - Université Bordeaux, Observatoire Aquitain des Sciences de L'Univers, F-33271 Floirac Cedex, 2 rue de l'Observatoire, France
AD - CNRS, UMR 5804, Laboratoire D'Astrophysique de Bordeaux, F-33271 Floirac Cedex, 2 rue de l'Observatoire, France
AB - In the outer regions of the habitable zone, the risk of transitioning into a globally frozen "snowball" state poses a threat to the habitability of planets with the capacity to host water-based life. Here, we use a one-dimensional energy balance climate model (EBM) to examine how obliquity, spin rate, orbital eccentricity, and the fraction of the surface covered by ocean might influence the onset of such a snowball state. For an exoplanet, these parameters may be strikingly different from the values observed for Earth. Since, for a constant semimajor axis, the annual mean stellar irradiation scales with (1 - e2)-1/2, one might expect the greatest habitable semimajor axis (for fixed atmospheric composition) to scale as (1 - e 2)-1/4. We find that this standard simple ansatz provides a reasonable lower bound on the outer boundary of the habitable zone, but the influence of both obliquity and ocean fraction can be profound in the context of planets on eccentric orbits. For planets with eccentricity 0.5, for instance, our EBM suggests that the greatest habitable semimajor axis can vary by more than 0.8 AU (78%!) depending on obliquity, with higher obliquity worlds generally more stable against snowball transitions. One might also expect that the long winter at an eccentric planet's apoastron would render it more susceptible to global freezing. Our models suggest that this is not a significant risk for Earth-like planets around Sun-like stars, as considered here, since such planets are buffered by the thermal inertia provided by oceans covering at least 10% of their surface. Since planets on eccentric orbits spend much of their year particularly far from the star, such worlds might turnout to be especially good targets for direct observations with missions such as TPF-Darwin. Nevertheless, the extreme temperature variations achieved on highly eccentric exo-Earths raise questions about the adaptability of life to marginally or transiently habitable conditions. © 2010. The American Astronomical Society. All rights reserved.
KW - Astrobiology
KW - Planetary systems
KW - Radiative transfer
PB - Institute of Physics Publishing

SN - 0004637X (ISSN)
LA - English
J2 - Astrophys. J.
M3 - Article
DB - Scopus
N1 - Export Date: 23 February 2023; Cited By: 88; Correspondence Address: C. D. Dressing; Department of Astrophysical Sciences, Princeton University, Peyton Hall, Princeton, NJ 08544, United States; email: courtney@astro.princeton.edu
ER -

TY - JOUR
AU - Glikson, A.
TI - Archaean asteroid impacts, banded iron formations and MIF-S anomalies: A discussion
PY - 2010
T2 - Icarus
VL - 207
IS - 1
SP - 39
EP - 44
DO - 10.1016/j.icarus.2009.11.024
UR - https://www.scopus.com/inward/record.uri?eid=2-s2.0-77950033148&doi=10.1016%2fj.icarus.2009.11.024&partnerID=40&md5=8e708f4f41d93658975f52f375d98310
AD - Research School of Earth Science, Australian National University, Canberra, ACT 0200, Australia
AB - The origin of mass-independent fractionation (MIF-S) of sulphur isotopes (δ33S) recorded in sediments older than 2.45 Ga is widely interpreted in terms of UV-triggered reactions under oxygen-poor ozone-depleted atmosphere conditions (Farquhar, J., Bao, H., Thiemens, M. [2000] Science, 289, 756; Farquhar, J., Peters, M., Johnston, D.T., Strauss, H., Masterson, A., Wiechert, U., Kaufman, A.J. [2007] Nature, 449, 706-709; Farquhar, J., Wing, B.A. [2003] Earth Planet. Sci. Lett., 213, 1-13; Kaufman, A.J., Johnston, D.T., Farquhar, J., Masterson, A.L., Lyons, T.W., Bates, S., Anbar, A.D., Arnold, G.L., Garvin, J., Buick, R. [2007a] Science, 317, 1900-1903; Kaufman, A.J., Farquhar, J., Johnston, D.T., Lyons, T.W., Arnold, G.L., Anbar, A. [2007b] Deep Time Drilling Project of the NASA Astrobiology Drilling Program). Observed mid-Archaean variability of MIF-S signatures raises questions regarding the extent of atmospheric anoxia (Ohmoto, H., Watanabe, Y., Ikemi, H., Poulson, H.R., Taylor, B. [2006] Nature, 406, 908-991; Farquhar et al., 2007). Late Archaean (~2.7-2.5 Ga) and mid-Archaean (~3.2 Ga) sequences in the Pilbara Craton (Western Australia) and Kaapvaal Craton (South Africa), in which MIF-S data were measured, contain asteroid impact ejecta units dated as 2.48, 2.56, 2.63, 3.24, 3.26 and 3.47 Ga old (Lowe, D.R., Byerly, G.R., Kyte, T., Shukolyukov, A., Asaro, F., Krull, A. [2003] Astrobiology, 3, 7-48; Simonson, B.M., Hassler, S.W. [1997] Aust. J. Earth Sci., 44, 37-48; Simonson, B.M., Glass, B.P. [2004] Ann. Rev. Earth Planet. Sci., 32, 329-361; Glikson, A.Y. [2004] Astrobiology, 4, 19-50; Glikson, A.Y. [2006] Earth Planet. Sci. Lett., 246, 149-160; Glikson, A.Y. [2008] Earth Planet. Sci. Lett., 267, 558-570). Mass balance calculations based

on iridium and 53Cr/52Cr isotopic anomalies (Byerly, G.R., Lowe, D.R. [1994] Geochim. Cosmochim. Acta, 58, 3469–3486; Kyte, F.T., Shukloyukov, A., Lugmair, G.W., Lowe, D.R., Byerly, G.R. [2003] Geology, 31, 283–286) and on impact spherule size distribution (Melosh, H.J., Vickery, A.M. [1991] Nature, 350, 494–497) suggest projectiles several tens of kilometers in diameter (Byerly and Lowe, 1994; Shukloyukov, A., Kyte, F.T., Lugmair, G.W., Lowe, D.R., Byerly, G.R. [2000]. In: Koeberl, C., Gilmour, I. (Eds.), Impacts and the Early Earth, Springer-Verlag, Berlin, pp. 99–116; Kyte, F.T., Shukloyukov, A., Lugmair, G.W., Lowe, D.R., Byerly, G.R. [2003] Geology, 31, 283–286). Due to incomplete preservation these impacts represent a minimum rate of the Archaean impact flux. High UV radiation associated with low ozone levels in the Archaean atmosphere may have been further enhanced by large impacts, accentuating MIF-S anomalies. The appearance of iron-rich sediments above late and mid-Archaean impact ejecta units (Glikson, A.Y. [2006] Earth Planet. Sci. Lett., 246, 149–160; Glikson, A.Y., Vickers, J. [2007] Earth Planet. Sci. Lett., 254, 214–226) may be related either to microbial oxidation of ferrous iron or, alternatively, photochemical oxidation of ferrous to ferric iron. Given post-2.45 Ga diluting of possible MIF-S anomalies by the oxygenating ocean sulfate reservoir (Pavlov, A.A., Kasting, J.F. [2002] Astrobiology, 2, 27–41), similar MIF-S anomalies may have been associated with Proterozoic and Phanerozoic impacts, although to date little evidence exists in this regard (Marouka, T., Koeberl, C., Newton, J., Gilmour, I., Bohor, B.F. [2002] Geological Society of America Special Paper 356, pp. 337–344; Koeberl, C., Thiemens, M. [2008] Multi-sulfur isotopes in cretaceous-tertiary boundary samples from the Western interior-search for photochemical effects 2008. Joint Meeting of the Geological Society of America, Soil Science Society of America, American Society of Agronomy, Crop Science Society of America, Gulf Coast Association of Geological Societies with the Gulf Coast Section of SEPM. <http://gsa.confex.com/gsa/2008AM/finalprogram/abstract_148134.htm> (abstract)). Detailed sampling and isotopic analyses across the impact ejecta fallout units are required in order to test possible relationships between Archaean impacts and MIF-S anomalies. © 2009 Elsevier Inc. All rights reserved.
KW - Archaean
KW - Asteroid
KW - Atmosphere
KW - Impact
KW - Isotopes
KW - Oxygen
KW - Sulphur
SN - 10902643 (ISSN)
LA - English
J2 - Icarus
M3 - Article
DB - Scopus
N1 - Export Date: 23 February 2023; Cited By: 8; Correspondence Address: A. Glikson; Research School of Earth Science, Australian National University, Canberra, ACT 0200, Australia; email: Andrew.glikson@anu.edu.au; CODEN: ICRSA

ER -

TY - JOUR
AU - Kitzmann, D.
AU - Patzer, A.B.C.
AU - Von Paris, P.
AU - Godolt, M.
AU - Stracke, B.
AU - Gebauer, S.
AU - Grenfell, J.L.
AU - Rauer, H.
TI - Clouds in the atmospheres of extrasolar planets: I. Climatic effects of multi-layered clouds for Earth-like planets and implications for habitable zones
PY - 2010
T2 - Astronomy and Astrophysics
VL - 511
IS - 1
C7 - A66
DO - 10.1051/0004-6361/200913491
UR - https://www.scopus.com/inward/record.uri?eid=2-s2.0-78650938046&doi=10.1051%2f0004-6361%2f200913491&partnerID=40&md5=2662532eeb227163b8d7044cfd74f1d2
AD - Zentrum für Astronomie und Astrophysik, Technische Universität Berlin, 10623 Berlin, Hardenbergstr. 36, Germany
AD - Institut für Planetenforschung, Deutsches Zentrum für Luft- und Raumfahrt (DLR), 12489 Berlin, Rutherfordstr. 2, Germany
AB - Aims: The effects of multi-layered clouds in the atmospheres of Earth-like planets orbiting different types of stars are studied. The radiative effects of cloud particles are directly correlated with their wavelength-dependent optical properties. Therefore the incident stellar spectra may play an important role for the climatic effect of clouds. We discuss the influence of clouds with mean properties measured in the Earth's atmosphere on the surface temperatures and Bond albedos of Earth-like planets orbiting different types of main sequence dwarf stars. The influence of clouds on the position of the habitable zone around these central star types is discussed. Methods: A parametric cloud model has been developed based on observations in the Earth's atmosphere. The corresponding optical properties of the cloud particles are calculated with the Mie theory accounting for shape effects of ice particles by the equivalent sphere method. The parametric cloud model is linked with a one-dimensional radiative-convective climate model to study the effect of clouds on the surface temperature and the Bond albedo of Earth-like planets in dependence of the type of central star. Results: The albedo effect of the low-level clouds depends only weakly on the incident stellar spectra because the optical properties remain almost constant in the wavelength range of the maximum of the incident stellar radiation. The greenhouse effect of the high-level clouds on the other hand depends on the temperature of the lower atmosphere, which is itself an indirect consequence of the different types of central stars. In general the planetary Bond albedo increases with the cloud cover of either cloud type. An anomaly was found for the K and M-type stars however,

resulting in a decreasing Bond albedo with increasing cloud cover for certain atmospheric conditions. Depending on the cloud properties, the position of the habitable zone can be located either farther from or closer to the central star. As a rule, low-level water clouds lead to a decrease of distance because of their albedo effect, while the high-level ice clouds lead to an increase in distance. The maximum variations are about 15% decrease and 35% increase in distance compared to the clear sky case for the same mean Earth surface conditions in each case. © 2010 ESO.
KW - Carbon dioxide
KW - Climate models
KW - Clouds
KW - Earth (planet)
KW - Global warming
KW - Greenhouse effect
KW - Optical properties
KW - Planets
KW - Solar radiation
KW - Spheres
KW - Stars
KW - Surface properties
KW - Albedo effects
KW - Atmospheric conditions
KW - Atmospheric effects
KW - Clear sky
KW - Climatic effects
KW - Cloud cover
KW - Cloud models
KW - Cloud particles
KW - Cloud properties
KW - Cloud types
KW - Dwarf stars
KW - Earth surface
KW - Earth-like planets
KW - Extra solar planets
KW - Ice particles
KW - Level ice
KW - Low-level clouds
KW - Lower atmosphere
KW - Mie theory
KW - Multi-layered
KW - Planetary system
KW - Radiative effects
KW - Shape effect
KW - Stellar radiation
KW - Stellar spectra
KW - Surface temperatures
KW - Water clouds
KW - Wavelength ranges
KW - Wavelength-dependent optical properties
KW - Atmospheric temperature
KW - Astrobiology
KW - Atmospheric effects
KW - Planetary systems

SN - 14320746 (ISSN)
LA - English
J2 - Astron. Astrophys.
M3 - Article
DB - Scopus
N1 - Export Date: 23 February 2023; Cited By: 50; Correspondence Address: D. Kitzmann; Zentrum für Astronomie und Astrophysik, Technische Universität Berlin, 10623 Berlin, Hardenbergstr. 36, Germany; email: kitzmann@astro.physik.tu-berlin.de; CODEN: AAEJA
ER -

TY - CONF
AU - Miller, J.D.
AU - Case, M.J.
AU - Straat, P.A.
AU - Levin, G.V.
TI - Likelihood of methane-producing microbes on Mars
PY - 2010
T2 - Proceedings of SPIE - The International Society for Optical Engineering
VL - 7819
C7 - 78190I
DO - 10.1117/12.862230
UR - https://www.scopus.com/inward/record.uri?eid=2-s2.0-78649403807&doi=10.1117%2f12.862230&partnerID=40&md5=9586760f3ab4e6af80b23500e36802ab
AD - Department of Cell and Neurobiology, Keck School of Medicine, USC, Los Angeles, CA 90033, United States
AD - Department of Anatomy and Neurobiology, University of California, Irvine, School of Medicine, Irvine, CA 92697, United States
AD - NIH (Ret.), Sykesville, MD 21784, 830 Windy Knoll, United States
AD - Beyond Center, College of Liberal Arts and Sciences, Arizona State University, Tempe, AZ 85287, United States
AB - We present a likelihood estimate that methane was a significant component of the gas detected by the Labeled Release (LR) experiment in the Viking Mission to Mars of 1976. In comparison with terrestrial methanogen production of methane we estimate the size of the putative microbe population necessary to produce the LR gas, had it been primarily methane. We extrapolate that figure to estimate the number of methanogens necessary to produce the methane content of the Martian atmosphere. Next, we estimate the amount of Martian soil and the amount of water needed for that global population of microbes. Finally, assuming a globally distributed population of such microbes, we estimate the likely sub-surface depth at which such methanogens could be detected. © 2010 Copyright SPIE - The International Society for Optical Engineering.
KW - Experiments
KW - Methanation
KW - Methane
KW - Extraterrestrial life
KW - Global population
KW - Likelihood estimate

KW - Mars
KW - Martian atmospheres
KW - Martian soils
KW - Methane content
KW - Release experiments
KW - Sub-surfaces
KW - Viking
KW - Methanogens
KW - Astrobiology
KW - Extraterrestrial Life
KW - Labeled Release experiment
KW - Mars
KW - methane
KW - Methanogens
KW - Viking
SN - 0277786X (ISSN); 978-081948315-7 (ISBN)
LA - English
J2 - Proc SPIE Int Soc Opt Eng
M3 - Conference paper
DB - Scopus
N1 - Export Date: 23 February 2023; Cited By: 4; Correspondence Address: J. D. Miller; Department of Cell and Neurobiology, Keck School of Medicine, USC, Los Angeles, CA 90033, United States; email: jdm@usc.edu; CODEN: PSISD
ER -

TY - CHAP
AU - Alcazar, A.
AU - Garcia-Descalzo, L.
AU - Cid, C.
TI - Microbial evolution and adaptation in icy worlds
PY - 2010
T2 - Astrobiology: Physical Origin, Biological Evolution and Spatial Distribution
SP - 81
EP - 95
UR - https://www.scopus.com/inward/record.uri?eid=2-s2.0-85030234159&partnerID=40&md5=74c35c43d15d33b2bd80165e3ed84b42
AD - Department of Investigation, Hospital Ramon y Cajal, Madrid, 28034, Spain
AD - Microbial Evolution Laboratory, Center for Astrobiology (CSIC-INTA), Torrejón de Ardoz, Madrid, 28850, Spain
AB - There is considerable interest in astrobiology in investigating icy worlds and the microbial forms that thrive in extreme environments, especially under those cold conditions that can provide a model for some extraterrestrial environments. After the successful missions Galileo and Cassini-Huygens to Jupiter and Saturn systems respectively this priority has been focused on the research of environments potentially habitable in some icy worlds such as Europa, Encelado or Titan. On Earth, the recent discovery of cold-tolerant microorganisms in glaciated and permanently frozen environments has broadened the known range of environmental conditions which support microbial life. These microorganisms, known as psychrophiles are defined as organisms having an optimal

temperature for growth at about 15°C or lower, a maximal temperature for growth at about 20°C, and a minimal temperature for growth at 0°C or below. The lowest temperature limit for life seems to be around −20°C, which is the value reported for bacteria living in permafrost soil and in sea ice. Terrestrial models of extraterrestrial icy worlds are being intensively studied. Among them, bacterial communities from Arctic and Antarctic permafrost, subglacial lakes and high mountains are considered representative of these environments in which psychrophile bacteria are the unique inhabitants. When compared to other known prokaryotes, psychrophilic bacteria posses many unique qualities and molecular mechanisms that allow their adaptation to cold environments. In this regard, some psychrophiles have been found to contain polyunsaturated fatty acids in their plasma membranes, which generally do not occur in other prokaryotes. Further, some of them use enzymes that continue to function at near freezing ambient temperatures. And finally, psychrophilic bacteria are able to produce proteins that are stable at cold temperatures such as heat shock proteins (Hsps) and cold shock proteins (Csps). This chapter will review the general trends concerning the biodiversity and ecology of psychrophilic microorganisms and their molecular mechanisms of adaptation and evolution, as well as summarize our latest results on this topic in samples from icy environments. © 2010 Nova Science Publishers, Inc.
KW - Biodiversity
KW - Cold adaptation
KW - Cold shock proteins
KW - Extremophiles
KW - Heat shock proteins
KW - Microbial ecology
KW - Microbial evolution
KW - Molecular mechanisms
KW - Permafrost
KW - Polyunsaturated fatty acids
KW - Protein interactions
KW - Psychrophiles
PB - Nova Science Publishers, Inc.
SN - 978-161761955-7 (ISBN); 978-160741290-8 (ISBN)
LA - English
J2 - Astrobiology: Physical Origin, Biological Evolution and Spatial Distribution
M3 - Book chapter
DB - Scopus
N1 - Export Date: 23 February 2023; Cited By: 8; Correspondence Address: C. Cid; Microbial Evolution Laboratory, Center for Astrobiology (CSIC-INTA), Torrejón de Ardoz, Madrid, 28850, Spain; email: cidsc@inta.es
ER -

TY - CONF
AU - Sanroma, E.
AU - Pallé, E.
TI - Earth like planets albedo variations versus continental landmass distribution
PY - 2010

T2 - Proceedings of the International Astronomical Union
VL - 6
IS - S276
SP - 547
EP - 548
DO - 10.1017/S1743921311021168
UR - https://www.scopus.com/inward/record.uri?eid=2-s2.0-84882959697&doi=10.1017%2fS1743921311021168&partnerID=40&md5=1c751b77436ddad95df2272501b4c6c4
AD - Instituto de Astrofísica de Canarias (IAC), Vía Lactea s/n 38200, La Laguna, Spain
AB - By making use of real information about the continental and oceanic surface distribution of the Earth, and cloudiness data from the International Satellite Cloud Climatology Project (ISCCP), we have studied the large-scale cloudiness behavior according to latitude and surface types (ice, water, vegetation and desert). These empirical relationships are used here to reconstruct the possible cloud distribution of historical epochs of the Earth history such as Late Cretaceous (90 My ago) and Late Triasic (230 My ago) when the landmass distribution was different. This information can be used to simulate the photometric variability of these planets according to their different geographical distribution. © 2011 International Astronomical Union.
KW - Astrobiology
KW - Atmospheric effects
KW - Earth
KW - Planets and satellites: general
A2 - Sozzetti A.
A2 - Lattanzi M.G.
A2 - INAF - Osservatorio, Astronomico di Torino
A2 - Boss A.P.
A2 - Carnegie Institution of Washington, Washington DC
SN - 17439221 (ISSN); 978-052119652-9 (ISBN)
LA - English
J2 - Proc. Int. Astron. Union
M3 - Conference paper
DB - Scopus
N1 - Export Date: 23 February 2023; Cited By: 0
ER -

TY - JOUR
AU - Greenberg, R.
TI - Transport rates of radiolytic substances into europa's ocean: implications for the potential origin and maintenance of life
PY - 2010
T2 - Astrobiology
VL - 10
IS - 3
SP - 275
EP - 283
DO - 10.1089/ast.2009.0386
UR - https://www.scopus.com/inward/record.uri?eid=2-s2.0-77952517949&doi=10.1089%2fast.2009.0386&partnerID=40&md5=cddd0267f119db66f21cb5609e1792d1

AD - Lunar and Planetary Laboratory, University of Arizona, Tucson, AZ 85721-0092, 1629 East University Blvd., United States
AB - Bombardment of the surface of Europa produces oxidants and other biologically useful substances, but they can only contribute to the habitability of the ocean if they are delivered down through the icy crust. Previous estimates of the thickness of the oxygenated layer of ice assumed that impact gardening is the dominant factor and concluded that the ocean may be habitable if the oxidant delivery time, via undefined mechanisms, is sufficiently short. Consideration of the types of processes that continually resurface Europa suggests that the oxygenated layer is thicker than˜300m, far greater than the few meters indicated by impact gardening alone, and possibly includes the entire ice crust. The estimated delivery rate to the ocean is such that the oxygen levels could now be high enough to support macrofauna; and, at˜31011mol/yr of oxygen, it could maintain 3 million tons of macrofauna, assuming respiration rates similar to terrestrial marine organisms. These values are independent of any additional contributions due to possible photosynthesis. Initial formation of life would be difficult with so much oxygen, but the start of oxidant delivery into the ocean would have been delayed by 1-2 billion years while the crust became loaded with oxidants. In the ocean, this delay would have allowed time for prebiotic assemblages and anaerobic biological development prior to the increasing oxidant concentration to otherwise toxic levels.
Copyright 2010, Mary Ann Liebert, Inc.
KW - Biogenesis
KW - Exobiology
KW - Extraterrestrial Environment
KW - Jupiter
KW - Motion
KW - Oceans and Seas
KW - Radiation
KW - Surface Properties
KW - article
KW - astronomy
KW - biogenesis
KW - biology
KW - microclimate
KW - motion
KW - radiation
KW - sea
KW - surface property
KW - astrobiology
KW - Europa
KW - Geophysics
KW - Oxidants
KW - Surface reprocessing
SN - 15311074 (ISSN)
C2 - 20446868
LA - English
J2 - Astrobiology
M3 - Article
DB - Scopus
N1 - Export Date: 23 February 2023; Cited By: 38; Correspondence

Address: R. Greenberg; Lunar and Planetary Laboratory, University of Arizona, Tucson, AZ 85721-0092, 1629 East University Blvd., United States; email: greenberg@lpl.arizona.edu
ER -

TY - JOUR
AU - German, C.R.
AU - Bowen, A.
AU - Coleman, M.L.
AU - Honig, D.L.
AU - Huber, J.A.
AU - Jakuba, M.V.
AU - Kinsey, J.C.
AU - Kurz, M.D.
AU - Leroy, S.
AU - McDermott, J.M.
AU - De Mercier Lépinay, B.
AU - Nakamura, K.
AU - Seewald, J.S.
AU - Smith, J.L.
AU - Sylva, S.P.
AU - Van Dover, C.L.
AU - Whitcomb, L.L.
AU - Yoerger, D.R.
TI - Diverse styles of submarine venting on the ultraslow spreading Mid-Cayman Rise
PY - 2010
T2 - Proceedings of the National Academy of Sciences of the United States of America
VL - 107
IS - 32
SP - 14020
EP - 14025
DO - 10.1073/pnas.1009205107
UR - https://www.scopus.com/inward/record.uri?eid=2-s2.0-77956293333&doi=10.1073%2fpnas.1009205107&partnerID=40&md5=cf5a53c2cd0601508304081bc19d60c2
AD - Woods Hole Oceanographic Institution, Woods Hole, MA 02543, United States
AD - National Aeronautics and Space Administration Astrobiology Institute, Jet Propulsion Laboratory, California Institute of Technology, Pasadena, CA 91109, United States
AD - Duke University Marine Laboratory, Beaufort, NC 28516, United States
AD - Marine Biological Laboratory, Woods Hole, MA 02543, United States
AD - Australian Centre for Field Robotics, University of Sydney, Sydney, NSW 2006, Australia
AD - Institut des Sciences de la Terre de Paris, Centre National de la Recherche Scientifique, Unité Mixte de Recherche 7193, Paris, France
AD - Centre National de la Recherche Scientifique - GéoAzur, Université de Nice-Sophia Antipolis, Valbonne, France
AD - National Institute of Advanced Science and Technology,

Tsukuba, Ibaraki 305-8567, Japan
AD - Laboratory for Computational Sensing and Robotics, Johns Hopkins University, Baltimore, MD 21218-2681, United States
AB - Thirty years after the first discovery of high-temperature submarine venting, the vast majority of the global mid-ocean ridge remains unexplored for hydrothermal activity. Of particular interest are the world's ultraslow spreading ridges that were the last to be demonstrated to host high-temperature venting but may host systems particularly relevant to prebiotic chemistry and the origins of life. Here we report evidence for previously unknown, diverse, and very deep hydrothermal vents along the ~110 km long, ultra-slow spreading Mid-Cayman Rise (MCR). Our data indicate that the MCR hosts at least three discrete hydrothermal sites, each representing a different type of water-rock interaction, including both mafic and ultramafic systems and, at ~5,000 m, the deepest known hydrothermal vent. Although submarine hydrothermal circulation, in which seawater percolates through and reacts with host lithologies, occurs on all mid-ocean ridges, the diversity of vent types identified here and their relative geographic isolation make the MCR unique in the oceans. These new sites offer prospects for an expanded range of vent-fluid compositions, varieties of abiotic organic chemical synthesis and extremophile microorganisms, and unparalleled faunal biodiversity - all in close proximity.
KW - Biodiversity
KW - Geography
KW - Hot Temperature
KW - Oceans and Seas
KW - Seawater
KW - sea water
KW - water
KW - sea water
KW - article
KW - geochemistry
KW - geography
KW - hydrothermal vent
KW - priority journal
KW - synthesis
KW - temperature
KW - biodiversity
KW - heat
KW - sea
KW - Astrobiology
KW - Hydrothermal activity
KW - Microbiology
KW - Mid-ocean ridge
KW - Ocean chemistry
SN - 10916490 (ISSN)
C2 - 20660317
LA - English
J2 - Proc. Natl. Acad. Sci. U. S. A.
M3 - Article
DB - Scopus
N1 - Export Date: 23 February 2023; Cited By: 120; Correspondence Address: C. R. German; Woods Hole Oceanographic Institution, Woods

Hole, MA 02543, United States; email: cgerman@whoi.edu; CODEN: PNASA
ER -

TY - JOUR
AU - Izawa, M.R.M.
AU - Banerjee, N.R.
AU - Flemming, R.L.
AU - Bridge, N.J.
AU - Schultz, C.
TI - Basaltic glass as a habitat for microbial life: Implications for astrobiology and planetary exploration
PY - 2010
T2 - Planetary and Space Science
VL - 58
IS - 4
SP - 583
EP - 591
DO - 10.1016/j.pss.2009.09.014
UR - https://www.scopus.com/inward/record.uri?eid=2-s2.0-76349100067&doi=10.1016%2fj.pss.2009.09.014&partnerID=40&md5=8e3c81b02eb12ad4c5887a8103b9e7c6
AD - Department of Earth Sciences, University of Western Ontario, London, Ont. N6A 5B7, Canada
AD - Department of Geology, San Jose State University, San Jose, CA 95192-0102, United States
AB - Recent studies have demonstrated that terrestrial subaqueous basalts and hyaloclastites are suitable microbial habitats. During subaqueous basaltic volcanism, glass is produced by the quenching of basaltic magma upon contact with water. On Earth, microbes rapidly begin colonizing the glassy surfaces along fractures and cracks that have been exposed to water. Microbial colonization of basaltic glass leads to the alteration and modification of the rocks and produces characteristic granular and/or tubular bioalteration textures. Infilling of the alteration textures by minerals such as phyllosilicates, zeolites and titanite may enable their preservation through geologic time. Basaltic rocks are a major component of the Martian crust and are widespread on other solar system bodies. A variety of lines of evidence strongly suggests the long-term existence of abundant liquid water on ancient Mars. Recent orbiter, lander and rover missions have found evidence for the presence of transient liquid water on Mars, perhaps persisting to the present day. Many other solar system bodies, notably Europa, Enceladus and other icy satellites, may contain (or have once hosted) subaqueous basaltic glasses. The record of terrestrial glass bioalteration has been interpreted to extend as far back as ~3.5 billion years ago and is widespread in oceanic crust and its metamorphic equivalents. The terrestrial record of glass bioalteration strongly suggests that glassy or formerly glassy basaltic rocks on extraterrestrial bodies that have interacted with liquid water are high-value targets for astrobiological exploration. © 2009 Elsevier Ltd. All rights reserved.
KW - Astronomy
KW - Glass
KW - Interplanetary spacecraft

KW - Liquids
KW - Martian surface analysis
KW - Silicate minerals
KW - Solar equipment
KW - Solar system
KW - Textures
KW - Ancient life
KW - Ancient Mars
KW - Archean
KW - Basaltic glass
KW - Basaltic magma
KW - Basaltic rocks
KW - Basaltic volcanism
KW - Enceladus
KW - Extraterrestrial bodies
KW - Hyaloclastites
KW - Icy satellites
KW - Infilling
KW - Liquid water
KW - Mars exploration
KW - Microbial colonization
KW - Microbial life
KW - Oceanic crust
KW - Planetary exploration
KW - Rover missions
KW - Basalt
KW - Ancient life
KW - Archean
KW - Astrobiology
KW - Basaltic glass
KW - Ichnofossil
KW - Mars exploration
SN - 00320633 (ISSN)
LA - English
J2 - Planet. Space Sci.
M3 - Article
DB - Scopus
N1 - Export Date: 23 February 2023; Cited By: 28; Correspondence Address: M.R.M. Izawa; Department of Earth Sciences, University of Western Ontario, London, Ont. N6A 5B7, Canada; email: matthew.izawa@gmail.com; CODEN: PLSSA
ER -

TY - CONF
AU - Bosco, G.L.
TI - James L. Waters Symposium 2009 on near-infrared spectroscopy
PY - 2010
T2 - TrAC - Trends in Analytical Chemistry
VL - 29
IS - 3
SP - 197
EP - 208
DO - 10.1016/j.trac.2010.01.003
UR - https://www.scopus.com/inward/record.uri?eid=2-

s2.0-77649181113&doi=10.1016%2fj.trac.2010.01.003&partnerID=40&md5=776599f5160ffefcf37b92f72961ef57
AD - Duquesne University, Department of Chemistry and Biochemistry, Pittsburgh, PA, 600 Forbes Avenue, United States
AB - This is a report on the Pittcon 2009 Waters Symposium, which featured five speakers on the history and fast-growing applications of near-infrared spectroscopy (NIRS). Often called the "Father of NIRS", Karl Norris described the development and the initial refinement of the first NIR instruments for use in whole-sample grain measurements. Peter Flinn provided an explanation of the science behind this technology and explained the need for and use of chemometrics in interpreting NIRS data. Franklin "Woody" Barton explained how the first official, standardized methods were developed and described the development of compact, dedicated NIR spectrometers for rapid, non-destructive, at-line and in-line analysis of material. Phil Williams detailed the introduction of NIRS to the world of commerce and the spread of NIR worldwide. Finally, Robert Lodder explained some out-of-the-box modern applications of NIRS in the fields of aging, artificial intelligence and astrobiology.
KW - Artificial intelligence
KW - Grain refinement
KW - Infrared devices
KW - Infrared transmission
KW - Quality control
KW - Spectrum analysis
KW - Textiles
KW - Chemometrics
KW - HICI
KW - Hyperspectral integrated computational imaging
KW - In-line
KW - Modern applications
KW - NIR spectrometer
KW - Non destructive
KW - Quality analysis
KW - Standardized methods
KW - Williams
KW - aging
KW - artificial intelligence
KW - atheroma
KW - atherosclerotic plaque
KW - calibration
KW - chemometrics
KW - conference paper
KW - diagnostic imaging
KW - digital imaging
KW - electromagnetic field
KW - food analysis
KW - food quality
KW - grain
KW - human
KW - hyperspectral integrated computational imaging
KW - molecular dynamics
KW - near infrared spectroscopy

KW - online analysis
KW - priority journal
KW - spectrometer
KW - standardization
KW - symposium
KW - Infrared spectroscopy
KW - Chemometrics
KW - Grain-quality analysis
KW - HICI
KW - Hyperspectral integrated computational imaging
KW - James L. Waters Symposium 2009
KW - Near-infrared spectroscopy
KW - NIRS
KW - Pittcon 2009
SN - 01659936 (ISSN)
LA - English
J2 - TrAC Trends Anal. Chem.
M3 - Conference paper
DB - Scopus
N1 - Export Date: 23 February 2023; Cited By: 20; Correspondence Address: G.L. Bosco; Duquesne University, Department of Chemistry and Biochemistry, Pittsburgh, PA, 600 Forbes Avenue, United States; email: boscog@duq.edu; CODEN: TTAED
ER -

TY - CONF
AU - Solé Carretero, A.
TI - Is being a Planetary Scientist the best job in the world?
PY - 2010
T2 - 61st International Astronautical Congress 2010, IAC 2010
VL - 8
SP - 6503
EP - 6508
UR - https://www.scopus.com/inward/record.uri?eid=2-s2.0-79959470120&partnerID=40&md5=4993ad7dda3b578e1896e1b3d31b575d
AD - Open University (Phys. Sci. and Eng.), United States
AB - Planetary science, understood in a broad sense including astrobiology, is a discipline that in addition to generating great fascination and awe through the questions it tries to answer and the discoveries it makes, helps us understand what our place in the universe is, where we come from and ultimately who we are. At the same time, what we have learned through planetary science brings to our attention a planet that is truly unique: the Earth. Our planet is unique in many respects, the most remarkable of which is that it harbours life, from simple unicellular life to complex organisms and what we call intelligent life. But planetary science may in the not too-distant future end up revealing that our planet is in fact not so unique, that life exists and can thrive in other planetary bodies of our solar system or of other solar systems. This discipline thus has not only a very strong and appealing scientific content, but its discoveries can also have profound philosophical, social, cultural and even religious implications. Even if we consider planetary science only from a non-biological perspective, its findings have the capacity of leaving us astonished, as over the decades and over

the centuries it has shown and described our solar system with an ever-increasing level of detail. We have, for instance, gone a long way since the discovery of the Galilean Moons in the 17th century up until the spectacular images of these moons relayed back to Earth by the Voyager spacecrafts in the late 70s and early 80s, and the Galileo spacecraft in the 90s. Finding answers to the kinds of questions that planetary scientists ask, and designing the means that allow to find those answers, are therefore probably some of the most exciting things that one can do in life. However, how does one become a planetary scientist? What and where should you study? And, what is involved in actually working as one? What are the pros and cons? The author presents the results of a related survey conducted among first-line planetary scientists and engineers, as well as his experience learning planetary science at the Open University (UK) and an attempt to identify some optimal approaches and resources to become professionally involved in this field.
KW - Astronomy
KW - Earth (planet)
KW - Philosophical aspects
KW - Galilean moons
KW - Galileo spacecraft
KW - Level of detail
KW - Open universities
KW - Planetary bodies
KW - Planetary science
KW - Planetary scientist
KW - Scientific content
KW - Voyager spacecraft
KW - Interplanetary spacecraft
SN - 978-161782368-8 (ISBN)
LA - English
J2 - Int. Astronaut. Congr., IAC
M3 - Conference paper
DB - Scopus
N1 - Export Date: 23 February 2023; Cited By: 0; Correspondence Address: A. Solé Carretero; Open University (Phys. Sci. and Eng.), United States; email: ascarretero@planetaryprobe.eu
ER -

TY - JOUR
AU - Chastain, B.K.
AU - Kral, T.A.
TI - Approaching mars-like geochemical conditions in the laboratory: Omission of artificial buffers and reductants in a study of biogenic methane production on a smectite clay
PY - 2010
T2 - Astrobiology
VL - 10
IS - 9
SP - 889
EP - 897
DO - 10.1089/ast.2010.0480
UR - https://www.scopus.com/inward/record.uri?eid=2-s2.0-78650037804&doi=10.1089%2fast.2010.0480&partnerID=40&md5=ed23b7

3213458d17f831bb616b2018d2
AD - Department of Biological and Life Sciences, West Kentucky Community and Technical College, Paducah, KY, United States
AD - Department of Biological Sciences, University of Arkansas, Fayetteville, AR, United States
AB - Methanogens have not been shown to metabolize in conditions exactly analogous to those present in Mars' subsurface. In typical studies of methanogenic metabolism, nutrient-rich buffered media and reducing agents are added to the cultures in an attempt to optimize the environment for methanogen survival and growth. To study methanogens in more Mars-relevant laboratory conditions, efforts should be made to eliminate artificial media, buffers, and reducing agents from investigations of methanogenic metabolism. After preliminary work to compare methanogen viability on montmorillonite clay and JSC Mars-1 regolith simulant, a study was conducted to determine whether biological methanogenesis could occur in non-reduced, non-buffered environments containing only H2, CO2, montmorillonite, and the liquid fraction extracted from a montmorillonite/deionized water suspension. Biogenic methane was observed in the microenvironments despite the omission of traditional media, buffers, and reducing agents. Mean headspace methane concentration after 96 days of observation was 10.23%±0.64% (% vol±=, n=4). However, methane production was severely decreased with respect to reduced, buffered microenvironments (Day 28: 31.98% ±0.19%, n=3). Analysis of results and comparison to previous work indicate that montmorillonite clay has a strong ability to supply micronutrients necessary for methanogenic metabolism, and the liquid fraction from a montmorillonite/deionized water slurry can successfully be used as an alternative to reduced and buffered nutritive media in Mars-relevant studies of methanogenic metabolism. Key Words: Mars-Methane-Methanogens-Mars geochemistry. Astrobiology 10, 889-897. © Mary Ann Liebert, Inc.
KW - Aluminum Silicates
KW - Buffers
KW - Hydrogen-Ion Concentration
KW - Methane
KW - Methanobacteriaceae
KW - Planets
KW - Reducing Agents
KW - Silicates
KW - Water
KW - aluminum silicate
KW - buffer
KW - methane
KW - reducing agent
KW - silicate
KW - silicon dioxide
KW - water
KW - article
KW - astronomy
KW - biosynthesis
KW - chemistry
KW - clay
KW - metabolism

KW - Methanobacteriaceae
KW - pH
SN - 15311074 (ISSN)
C2 - 21118022
LA - English
J2 - Astrobiology
M3 - Article
DB - Scopus
N1 - Export Date: 23 February 2023; Cited By: 16; Correspondence
Address: B. K. Chastain; Department of Biological and Life Sciences, West Kentucky Community and Technical College, Waller Hall Main Office, Paducah, KY 42002-7380, 4810 Alben Barkley Dr, United States; email: brendon.chastain@kctcs.edu
ER -

TY - JOUR
AU - Zeller, B.E.
TI - Extraterrestrial biblical hermeneutics and the making of heaven's gate
PY - 2010
T2 - Nova Religio
VL - 14
IS - 2
SP - 34
EP - 60
DO - 10.1525/nr.2010.14.2.34
UR - https://www.scopus.com/inward/record.uri?eid=2-s2.0-77958553873&doi=10.1525%2fnr.2010.14.2.34&partnerID=40&md5=4caf46995a0c5931e31063d6f9916b10
AB - The new religious movement popularly called Heaven's Gate emerged in the mid-1970s. This article argues that its two co-founders, Marshall Herff Applewhite (1932-1997) and Bonnie Lu Nettles (1928-1985), employed what I call extraterrestrial biblical hermeneutics in constructing the theological worldview of Heaven's Gate. This hermeneutics developed out of the New Age movement and its broader interest in ufology, extraterrestrial life, and alien visitation, and postulates a series of close encounters and alien visitations. Borrowing from its New Age and ufological origins, the hermeneutics assumes an extraterrestrial interest in assisting human beings to self-develop, as well as a technological materialism antithetical to supernaturalist readings of the Bible. As I argue here, this extraterrestrial biblical hermeneutics led Applewhite and Nettles to read the Bible as supporting a message of alien visitation, self-transformation, and ultimately extraterrestrial technological rapture. © 2010 by The Regents of the University of California. All rights reserved.
SN - 15418480 (ISSN)
LA - English
J2 - Nova Relig.
M3 - Review
DB - Scopus
N1 - Export Date: 23 February 2023; Cited By: 8
ER -

TY - CONF
AU - Dumas, S.
AU - Dutil, Y.
AU - Joncas, G.
TI - Detection of biomarkers using infra-red spectroscopy
PY - 2010
T2 - Acta Astronautica
VL - 67
IS - 11-12
SP - 1356
EP - 1360
DO - 10.1016/j.actaastro.2010.01.018
UR - https://www.scopus.com/inward/record.uri?eid=2-s2.0-77956871908&doi=10.1016%2fj.actaastro.2010.01.018&partnerID=40&md5=016e3bf0c11854bbd49d1bb4da53683a
AD - Dpt. de Physique, de Génie Physique et d'Optique, Centre de Recherche en Astrophysique du Québec, G1V 0A6, Canada
AB - In the near future, more missions of exploration will be sent to our neighbour planets and moons to detect signs of life. The automatisation of such search is not an easy task and selecting the right samples for the analysis is even less easy. This paper proposes a technique using infra-red spectroscopy coupled with statistical and multivariate algorithms to preselect targets for detailed search for biomarkers. © 2010 Elsevier Ltd. All rights reserved.
KW - Infrared
KW - Multivariant analysis
KW - Astrobiology
KW - Biomarkers
KW - Infrared
KW - Spectroscopy
SN - 00945765 (ISSN)
LA - English
J2 - Acta Astronaut
M3 - Conference paper
DB - Scopus
N1 - Export Date: 23 February 2023; Cited By: 1; Correspondence Address: S. Dumas; Dpt. de Physique, de Génie Physique et d'Optique, Centre de Recherche en Astrophysique du Québec, G1V 0A6, Canada; email: stephane_dumas@sympatico.ca; CODEN: AASTC
ER -

TY - CONF
AU - Kereszturi, A.
AU - Bérczi, Sz.
AU - Horváth, A.
AU - Pócs, T.
AU - Sik, A.
AU - Szathmáry, E.
TI - Astrobiological characteristics and possibility of life in the polar dark dune spots of Mars
PY - 2010
T2 - Proceedings of SPIE - The International Society for Optical Engineering

VL - 7819
C7 - 78190K
DO - 10.1117/12.862839
UR - https://www.scopus.com/inward/record.uri?eid=2-s2.0-78649426764&doi=10.1117%2f12.862839&partnerID=40&md5=58e8313c27af823e98b93631745fcd23
AD - Collegium Budapest Institute for Advanced Study, H-1014 Budapest, Szentharomag u. 2, Hungary
AD - Eotvos Lorand University of Sciences, Planetary Research Group, Hungary
AD - Eszterházy Károly College, Hungary
AB - The circumpolar region of Mars was surveyed for possible habitats in the area of seasonal ice cap. Dark Dune Spots were found to be good candidates as they are covered by water ice there without carbon-dioxide ice, and show flow-like features in springtime. Based on theoretical calculations interfacial water or bulk brine may be present there that decomposes the aggressive oxidants. Temperature values in springtime around noon could be favorable for metabolism of known extremophyles on Earth, and water uptake may be possible at nighttime. During the warm daytime period the water loss could be lowered by densely packed grain structure of the soil, hygroscopic salts, and polysaccharide-like materials, as it was observed in the samples of cryptobiotic crust from hot and cold deserts on Earth. This work outlines the basic elements of these possible circumpolar microhabitats. © 2010 Copyright SPIE - The International Society for Optical Engineering.
KW - Arid regions
KW - Ecosystems
KW - Basic elements
KW - Circumpolar regions
KW - Cold deserts
KW - Dark Dune Spots
KW - Hygroscopic salts
KW - Ice-caps
KW - Interfacial water
KW - life
KW - Mars
KW - Microhabitats
KW - Packed grains
KW - Polar Regions
KW - Temperature values
KW - Theoretical calculations
KW - Water ice
KW - Water loss
KW - Water uptake
KW - Ice
KW - Dark Dune Spots
KW - life
KW - Mars
KW - polar regions
KW - water
SN - 0277786X (ISSN); 978-081948315-7 (ISBN)
LA - English
J2 - Proc SPIE Int Soc Opt Eng

M3 - Conference paper
DB - Scopus
N1 - Export Date: 23 February 2023; Cited By: 0; CODEN: PSISD
ER -

TY - JOUR
AU - Managadze, G.
TI - Plasma and collision processes of hypervelocity meteorite impact in the prehistory of life
PY - 2010
T2 - International Journal of Astrobiology
VL - 9
IS - 3
SP - 157
EP - 174
DO - 10.1017/S147355041000008X
UR - https://www.scopus.com/inward/record.uri?eid=2-s2.0-77954563465&doi=10.1017%2fS147355041000008X&partnerID=40&md5=bf7feb918382da175646a496a6234aeb
AD - Space Research Institute (IKI), Russian Academy of Sciences, Moscow 117997, Profsouznaya 84/32, GSP7, Russian Federation
AB - A new concept is proposed, according to which the plasma and collision processes accompanying hypervelocity impacts of meteorites can contribute to the arising of the conditions on early Earth, which are necessary for the appearance of primary forms of living matter. It was shown that the processes necessary for the emergence of living matter could have started in a plasma torch of meteorite impact and have continued in an impact crater in the case of the arising of the simplest life form. It is generally accepted that planets are the optimal place for the origin and evolution of life. In the process of forming the planetary systems the meteorites, space bodies feeding planet growth, appear around stars. In the process of Earth's formation, meteorite sizes ranged from hundreds and thousands of kilometres. These space bodies consisted mostly of the planetesimals and comet nucleus. During acceleration in Earth's gravitational field they reached hypervelocity and, hitting the surface of planet, generated powerful blowouts of hot plasma in the form of a torch. They also created giant-size craters and dense dust clouds. These bodies were composed of all elements needed for the synthesis of organic compounds, with the content of carbon being up to 5%–15%. A new idea of possible synthesis of the complex organic compounds in the hypervelocity impact-generated plasma torch was proposed and experimentally confirmed. A previously unknown and experimentally corroborated feature of the impact-generated plasma torch allowed a new concept of the prehistory of life to be developed. According to this concept the intensive synthesis of complex organic compounds arose during meteoritic bombardment in the first 0.5 billion years at the stage of the planet's formation. This most powerful and destructive action in Earth's history could have played a key role and prepared conditions for the origin of life. In the interstellar gas-dust clouds, the synthesis of simple organic matter could have been explained by an identical process occurring in the plasma torch of hypervelocity collisions between submicron size dust particles. It is assumed that the processes occurred in

the highly unbalanced hot plasma simultaneously with the synthesis of simple and complicated organic compounds, thereby ensuring their ordering and assembly. Bona fide experimental evidence presented below indicates that the physical fields generated in the plasma environment in the process of the formation and expansion of the torch meet the main requirements toward true local chiral fields. These fields were very likely to be capable to trigger the initial, weak breaking of enantiomer symmetry and determine the sign of the asymmetry of the bioorganic world. These fields could have worked as trapping fields influencing spontaneous processes occurring in highly overheated and nonequilibrium plasma in the state that is far from the thermodynamical branch of equilibrium and may have contributed to the formation of an environment needed for the synthesis of homochiral molecular structures, which, in turn, were needed for the emergence of the primary forms of living matter. It has been shown experimentally that the plasma-chemical processes in the torch have high catalytic properties and assure the rise of the chemical reaction rates by 10-100 million times. In the process of the plasma flyaway this in turn can assure the fast formation of simple and complicated organic compounds, including hyper-branched polymers. It is possible to assume that predominantly inorganic substances from meteorites were used for the synthesis of complicated organic compounds on early Earth. A laboratory experiment with hypervelocity impact plasma torch modelling by a laser with a Q-switch mode has shown the possibility of high-molecular organic compound synthesis, with mass of approximately 5000 a.m.u. by meteorite impact with an effective diameter of 100 mkm. The target contained only H, C, N and O elements in inorganic forms. The approximation of the curve received in these experiments has shown that molecular structures comparable in mass with the protoviroid (a hypothetical primogenitor of the biosphere) and could have been synthesized as a result of the impact of a meteorite of a millimetre-size range. Observable characteristics of the synthesis processes suggest high catalytic activity of the plasma medium and high speed of plasma-chemical reactions, combined with ordering and assemblage processes. This suggests that the plasma torch with a huge local density of energy and matter may be the optimal medium for the synthesis of complex organic compounds needed for prebiotic evolution and the development of the primary form of living matter. A new view of the impact crater provides the most interesting and unexpected consequence of the concept proposed. When considering the problem, it became evident that at a prebiotic stage of evolution there should be an environment in which a photogenic creature could have survived. The crater of the meteoric impact, which is capable of producing a primogenitor of the biosphere environment sated with organic matter, moderate temperature and water for considerable time and becoming a life cradle, appears to be such an environment. Having enormous energy, the meteorite impact is capable of injecting the newly created complicated organic compounds deep into the space body surfaces, including subsurface water reservoirs, such as Europe, Enchilada and Titan. In this case the meteorite impact has no natural alternative in the creation of initial conditions for the origin of extraterrestrial life. This possibility was confirmed by a laboratory impact model experiment, in which the plasma torch was

created under the water surface. The concept proposed is based on physical processes occurring in nature and on experimental results of impact experiments and subsequent modelling of their analogues in laboratory conditions. Thus, the realizability and survivability of this concept should be taken as well grounded due to the simplicity and clarity of the physical processes. Copyright © Cambridge University Press 2010.
KW - crater
KW - early Earth
KW - experimental study
KW - impact
KW - meteorite
KW - modeling
KW - organic compound
KW - origin of life
KW - planetesimal
KW - plasma
KW - a biogenic synthesis
KW - assembling
KW - break of mirror symmetry
KW - charity's
KW - enantiomer's
KW - impact crater
KW - meteorite impact
KW - ordering
KW - plasma torch
KW - protoviroid
SN - 14753006 (ISSN)
LA - English
J2 - Int. J. Astrobiology
M3 - Article
DB - Scopus
N1 - Export Date: 23 February 2023; Cited By: 7; Correspondence Address: G. Managadze; Space Research Institute (IKI), Russian Academy of Sciences, Moscow 117997, Profsouznaya 84/32, GSP7, Russian Federation; email: managa@iki.rssi.ru
ER -

TY - JOUR
AU - Wang, G.
AU - Hao, Z.
AU - Huang, Z.
AU - Chen, L.
AU - Li, X.
AU - Hu, C.
AU - Liu, Y.
TI - Raman spectroscopic analysis of a desert cyanobacterium Nostoc sp. in response to UVB radiation
PY - 2010
T2 - Astrobiology
VL - 10
IS - 8
SP - 783
EP - 788

DO - 10.1089/ast.2009.0407
UR - https://www.scopus.com/inward/record.uri?eid=2-s2.0-78649507294&doi=10.1089%2fast.2009.0407&partnerID=40&md5=a0ddb8fbf2beebf3a8fbab4185a6985e
AD - State Key Laboratory of Freshwater Ecology and Biotechnology, Institute of Hydrobiology, Chinese Academy of Sciences, Wuhan 430072, China
AD - Graduate School of Chinese Academy of Sciences, Beijing, China
AD - College of Pharmacy, Wuhan University, Wuhan, China
AD - College of Resource and Environment Sciences, Wuhan University, Wuhan, China
AB - Cyanobacteria are capable of tolerating environmental extremes. To survive in extreme environments, cyanobacteria have developed the capability to adapt to a variety of stresses. For example, cyanobacteria have adopted a number of strategies with which to survive UV stress, including expression of UV-screening pigments and antioxidant systems. We have previously shown that several antioxidants are significantly expressed in Nostoc sp. by UVB irradiation. We report here that the content of UV-responsive biomarkers such as β-carotene and scytonemin can be easily detected by Fourier transform Raman spectroscopy with use of a small sample size and that the content of β-carotene is dependant on the UVB intensity and exposure time. Our results indicate that Raman spectroscopy may be a helpful tool to analyze UV-protective molecules of cyanobacterium in astrobiological studies without access to large sample sizes and complicated extractions, which are needed by other analytical techniques such as high-performance liquid chromatography and mass spectrometry. © Copyright 2010, Mary Ann Liebert, Inc. 2010.
KW - Antioxidants
KW - beta Carotene
KW - Exobiology
KW - Indoles
KW - Nostoc
KW - Phenols
KW - Photosynthesis
KW - Pigments, Biological
KW - Spectrum Analysis, Raman
KW - Ultraviolet Rays
KW - Cyanobacteria
KW - Nostoc sp.
KW - antioxidant
KW - beta carotene
KW - indole derivative
KW - phenol derivative
KW - pigment
KW - scytonemin
KW - article
KW - astrobiology
KW - metabolism
KW - methodology
KW - Nostoc
KW - photosynthesis
KW - radiation exposure

KW - Raman spectrometry
KW - ultraviolet radiation
KW - 783-788.
KW - Nostoc sp.-UVB-Raman spectra-b-carotene. Astrobiology 10
SN - 15311074 (ISSN)
C2 - 21087158
LA - English
J2 - Astrobiology
M3 - Article
DB - Scopus
N1 - Export Date: 23 February 2023; Cited By: 29; Correspondence Address: Y. Liu; State Key Laboratory of Freshwater Ecology and Biotechnology, Institute of Hydrobiology, Chinese Academy of Sciences, Wuhan 430072, China; email: space@ihb.ac.cn
ER -

TY - JOUR
AU - Warner, N.H.
AU - Farmer, J.D.
TI - Subglacial hydrothermal alteration minerals in Jökulhlaup deposits of Southern Iceland, with implications for detecting past or present habitable environments on mars
PY - 2010
T2 - Astrobiology
VL - 10
IS - 5
SP - 523
EP - 547
DO - 10.1089/ast.2009.0425
UR - https://www.scopus.com/inward/record.uri?eid=2-s2.0-78650069656&doi=10.1089%2fast.2009.0425&partnerID=40&md5=ba03dde9888648b738d69b75d9608c21
AD - Department of Earth Science and Engineering, Imperial College London, South Kensington Campus, London, SW7 2AZ, United Kingdom
AD - School of Earth and Space Exploration, Arizona State University, Tempe, AZ, United States
AB - Jökulhlaups are terrestrial catastrophic outfloods, often triggered by subglacial volcanic eruptions. Similar volcano-ice interactions were likely important on Mars where magma/lava may have interacted with the planet's cryosphere to produce catastrophic floods. As a potential analogue to sediments deposited during martian floods, the Holocene sandurs of Iceland are dominated by basaltic clasts derived from the subglacial environment and deposited during jökulhlaups. Palagonite tuffs and breccias, present within the deposits, represent the primary alteration lithology. The surface abundance of palagonite on the sandurs is 1-20%. X-ray diffraction (XRD) analysis of palagonite breccias confirms a mineral assemblage of zeolites, smectites, low-quartz, and kaolinite. Oriented powder X-ray diffractograms (<2μm fraction) for palagonite breccia clasts and coatings reveal randomly ordered smectite, mixed layer smectite/illite, zeolites, and quartz. Visible light-near infrared (VNIR) and shortwave infrared (SWIR) lab spectroscopic data of the same palagonite samples show H2O/OH- absorptions associated with clays and zeolites. SWIR spectra derived from Advanced

Spaceborne Thermal Emission and Reflection Radiometer (ASTER) images of the sandurs reveal Al–OH– and Si–OH– absorption features. The identified alteration mineral assemblage is consistent with low temperature (100–140°C) hydrothermal alteration of basaltic material within the subglacial environment. These results suggest that potential martian analog sites that contain a similar suite of hydrated minerals may be indicative of past hydrothermal activity and locations where past habitable environments for microbial life may be found. © Mary Ann Liebert, Inc.
KW - Exobiology
KW - Extraterrestrial Environment
KW - Geography
KW - Geologic Sediments
KW - Ice Cover
KW - Iceland
KW - Mars
KW - Microscopy, Electron, Scanning
KW - Minerals
KW - Satellite Communications
KW - Spectrum Analysis
KW - Surface Properties
KW - Temperature
KW - Water
KW - X-Ray Diffraction
KW - mineral
KW - water
KW - article
KW - astrobiology
KW - astronomy
KW - chemistry
KW - geography
KW - ice cover
KW - Iceland
KW - methodology
KW - microclimate
KW - scanning electron microscopy
KW - sediment
KW - spectroscopy
KW - surface property
KW - telecommunication
KW - temperature
KW - X ray diffraction
KW - Mars-Reflectance spectroscopy-Astrobiology-Hydrothermal systems-Habitability
SN - 15311074 (ISSN)
C2 - 20624060
LA - English
J2 - Astrobiology
M3 - Article
DB - Scopus
N1 - Export Date: 23 February 2023; Cited By: 30; Correspondence Address: N. H. Warner; Department of Earth Science and Engineering, Imperial College London, South Kensington Campus, London, SW7 2AZ, United Kingdom; email: n.warner@imperial.ac.uk

ER -

TY - CONF
AU - Benner, S.A.
TI - Chemistry, life, and the search for aliens
PY - 2010
T2 - Proceedings of SPIE - The International Society for Optical Engineering
VL - 7819
C7 - 781910
DO - 10.1117/12.866981
UR - https://www.scopus.com/inward/record.uri?eid=2-s2.0-78649417023&doi=10.1117%2f12.866981&partnerID=40&md5=8782515e5d8b0052131b160a4e6211d0
AD - Foundation for Applied Molecular Evolution, Gainesville, FL 32604, P.O. Box 13174, United States
AB - While "life" may universally be a self-sustaining chemical system capable of Darwinian evolution, alien life may be quite different in its chemistry from the terran life that we know here on Earth. In this case, it will be difficult to recognize, especially if it has not advanced beyond the single cell life forms that have dominated much of the terran biosphere. This review summarizes what we might infer from general physical and chemical law about how such "weird" life might be structured, what solvents other than water it might inhabit, what genetic molecules it might contain, and what metabolism it might exploit. © 2010 Copyright SPIE - The International Society for Optical Engineering.
KW - Alien life
KW - Chemical systems
KW - Darwinian evolution
KW - Extraterrestrial life
KW - planetary exploration
KW - Single cells
KW - weird life
KW - Interplanetary spacecraft
KW - Alien life
KW - extraterrestrial life
KW - planetary exploration
KW - water
KW - weird life
SN - 0277786X (ISSN); 978-081948315-7 (ISBN)
LA - English
J2 - Proc SPIE Int Soc Opt Eng
M3 - Conference paper
DB - Scopus
N1 - Export Date: 23 February 2023; Cited By: 1; CODEN: PSISD
ER -

TY - JOUR
AU - Amato, P.
AU - Doyle, S.M.
AU - Battista, J.R.
AU - Christner, B.C.
TI - Implications of subzero metabolic activity on long-term

microbial survival in terrestrial and extraterrestrial permafrost
PY - 2010
T2 - Astrobiology
VL - 10
IS - 8
SP - 789
EP - 798
DO - 10.1089/ast.2010.0477
UR - https://www.scopus.com/inward/record.uri?eid=2-s2.0-78649508781&doi=10.1089%2fast.2010.0477&partnerID=40&md5=b051f707d89354c874c6e8dc35cc8429
AD - Louisiana State University, Department of Biological Sciences, Life Sciences Building, Baton Rouge, LA 70803, United States
AD - Laboratoire de Synthèse et Etudes de Systèmes À Intérêt Biologique, CNRS-Université Blaise Pascal, Aubière, France
AB - The survival of microorganisms over extended time frames in frozen subsurface environments may be limited by chemical (i.e., via hydrolysis and oxidation) and ionizing radiation-induced damage to chromosomal DNA. In an effort to improve estimates for the survival of bacteria in icy terrestrial and extraterrestrial environments, we determined rates of macromolecular synthesis at temperatures down to -15°C in bacteria isolated from Siberian permafrost (Psychrobacter cryohalolentis K5 and P. arcticus 273-4) and the sensitivity of P. cryohalolentis to ionizing radiation. Based on experiments conducted over ≈400 days at -15°C, the rates of protein and DNA synthesis in P. cryohalolentis were <1 to 16 proteins cell-1 d-1 and 83 to 150 base pairs (bp) cell-1 d-1, respectively; P. arcticus synthesized DNA at rates of 20 to 1625bp cell-1 d -1 at -15°C under the conditions tested. The dose of ionizing radiation at which 37% of the cells survive (D37) of frozen suspensions of P. cryohalolentis was 136Gy, which was ~2-fold higher (71Gy) than identical samples exposed as liquid suspensions. Laboratory measurements of [3H]thymidine incorporation demonstrate the physiological potential for DNA metabolism at -15°C and suggest a sufficient activity is possible to offset chromosomal damage incurred in near-subsurface terrestrial and martian permafrost. Thus, our data imply that the longevity of microorganisms actively metabolizing within permafrost environments is not constrained by chromosomal DNA damage resulting from ionizing radiation or entropic degradation over geological time. © Copyright 2010, Mary Ann Liebert, Inc. 2010.
KW - Exobiology
KW - Extraterrestrial Environment
KW - Freezing
KW - Psychrobacter
KW - Siberia
KW - Bacteria (microorganisms)
KW - Psychrobacter cryohalolentis
KW - article
KW - astrobiology
KW - freezing
KW - metabolism
KW - microclimate
KW - Psychrobacter
KW - Russian Federation

KW - 789-798.
KW - Extraterrestrial life-Radiation resistance-Cryosphere-DNA-Dormancy. Astrobiology 10
SN - 15311074 (ISSN)
C2 - 21087159
LA - English
J2 - Astrobiology
M3 - Article
DB - Scopus
N1 - Export Date: 23 February 2023; Cited By: 32; Correspondence Address: B. C. Christner; Louisiana State University, Department of Biological Sciences, Life Sciences Building, Baton Rouge, LA 70803, United States; email: xner@lsu.edu
ER -

TY - JOUR
AU - Morris, J.
TI - Astrobiology factors heavily in planetary science decadal survey
PY - 2010
T2 - Aviation Week and Space Technology (New York)
VL - 172
IS - 17
UR - https://www.scopus.com/inward/record.uri?eid=2-s2.0-77953071707&partnerID=40&md5=bd2be5fa9c438092584bc79c6be5404b
AB - The National Academies' National Research Council has organized the Planetary Science Decadal Survey at the request of NASA and the National Science Foundation. Many of those missions focus on astrobiology and include a Mars Trace Gas Orbiter that would study the recently discovered methane in the Martian atmosphere. A sample return mission that would send a rover to Mars to collect a suite of samples, a lander to recover and launch them into orbit, and an orbiter to dock with the samples and return them to Earth. A mission to Jupiter's moon Europa that would study the subsurface ocean thought to exist under its crust and determine its suitability for life. Researchers at NASA, the University of California at Los Angeles and Arizona State University have for the first time found diverse microscopic fossils in the soft mineral gypsum, which precipitates out of saltwater when it dries up.
SN - 00052175 (ISSN)
LA - English
J2 - Aviat. Week Space Technol. (New York)
M3 - Review
DB - Scopus
N1 - Export Date: 23 February 2023; Cited By: 0; CODEN: AWSTA
ER -

TY - JOUR
AU - Oehler, D.Z.
AU - Allen, C.C.
TI - Evidence for pervasive mud volcanism in Acidalia Planitia, Mars
PY - 2010
T2 - Icarus

VL - 208
IS - 2
SP - 636
EP - 657
DO - 10.1016/j.icarus.2010.03.031
UR - https://www.scopus.com/inward/record.uri?eid=2-s2.0-77954661468&doi=10.1016%2fj.icarus.2010.03.031&partnerID=40&md5=3a28fae631649b2cca215b73df0c4db4
AD - Astromaterials Research and Exploration Science Directorate, NASA-Johnson Space Center, Houston, TX 77058, 2101 NASA Parkway, United States
AB - We have mapped 18,000+ circular mounds in a portion of southern Acidalia Planitia using their sizes, shapes, and responses in Nighttime IR. We estimate that 40,000+ of these features could occur in the area, with a distribution generally corresponding to the southern half of the proposed Acidalia impact basin. The mounds have average diameters of about 1. km and relief up to 180. m and most overlie units mapped as Early Amazonian.High resolution images of mound surfaces show relatively smooth veneers, apron-like extensions onto the plains, moats, and concentric circular crestal structures. Some images show lobate and flow-like features associated with the mounds. Albedo of the mounds is generally higher than that of the surrounding plains. Visible and near-infrared spectra suggest that the mounds and plains have subtle mineralogical differences, with the mounds having enhanced coatings or possibly greater quantities of crystalline ferric oxides.Multiple analogs for these structures were assessed in light of new orbital data and regional mapping. Mud volcanism is the closest terrestrial analogy, though the process in Acidalia would have had distinctly martian attributes. This interpretation is supported by the geologic setting of the Acidalia which sits at the distal end of the Chryse-Acidalia embayment into which large quantities of sediments were deposited through the Hesperian outflow channels. In its distal position, Acidalia would have been a depocenter for accumulation of mud and fluids from outflow sedimentation.Thus, the profusion of mounds in Acidalia is likely to be a consequence of this basin's unique geologic setting. Basinwide mud eruption may be attributable to overpressure (developed in response to rapid outflow deposition) perhaps aided by regional triggers for fluid expulsion related to events such as tectonic or hydrothermal pulses, destabilization of clathrates, or sublimation of a frozen body of water. Significant release of gas may have been involved, and the extensive mud volcanism could have created long-lived conduits for upwelling groundwaters, providing potential habitats for an in situ microbiota.Mud volcanism transports minimally-altered materials from depth to the surface, and mud volcanoes in Acidalia, therefore, could provide access to samples from deep zones that would otherwise be inaccessible. Since the distal setting of Acidalia also would favor concentration and preservation of potentially-present organic materials, samples brought to the surface by mud volcanism could include biosignatures of possible past or even present life. Accordingly, the mounds of Acidalia may offer a new class of exploration target. © 2010 Elsevier Inc.
KW - Astrobiology

KW - Mars, Surface
SN - 10902643 (ISSN)
LA - English
J2 - Icarus
M3 - Article
DB - Scopus
N1 - Export Date: 23 February 2023; Cited By: 98; Correspondence Address: D.Z. Oehler; Astromaterials Research and Exploration Science Directorate, NASA-Johnson Space Center, Houston, TX 77058, 2101 NASA Parkway, United States; email: dorothy.z.oehler@nasa.gov; CODEN: ICRSA
ER -

TY - JOUR
AU - Levy, J.S.
AU - Marchant, D.R.
AU - Head, J.W.
TI - Thermal contraction crack polygons on Mars: A synthesis from HiRISE, Phoenix, and terrestrial analog studies
PY - 2010
T2 - Icarus
VL - 206
IS - 1
SP - 229
EP - 252
DO - 10.1016/j.icarus.2009.09.005
UR - https://www.scopus.com/inward/record.uri?eid=2-s2.0-75949104636&doi=10.1016%2fj.icarus.2009.09.005&partnerID=40&md5=3a1abf140c0ebc96653c697b2e920b0f
AD - Department of Geological Sciences, Brown University, Providence, RI 02912, United States
AD - Department of Earth Science, Boston University, Boston, MA 02215, 675 Commonwealth Ave, United States
AB - Thermal contraction crack polygons are complex landforms that have begun to be deciphered on Earth and Mars by the combined investigative efforts of geomorphology, environmental monitoring, physical models, paleoclimate reconstruction, and geochemistry. Thermal contraction crack polygons are excellent indicators of the current or past presence of ground ice, ranging in ice content from weakly cemented soils to debris-covered massive ice. Relative to larger topographic features, polygons may form rapidly, and reflect climate conditions at the time of formation-preserving climate information as relict landforms in the geological record. Polygon morphology and internal textural characteristics can be used to distinguish surfaces modified by the seasonal presence of a wet active layer or dry active layer, and to delimit subsurface ice conditions. Analysis of martian polygon morphology and distribution indicates that geologically-recent thermal contraction crack polygons on Mars form predominantly in an ice-rich latitude-dependent mantle, more likely composed of massive ice deposited by precipitation than by cyclical vapor diffusion into regolith. Regional and local heterogeneities in polygon morphology can be used to distinguish variations in ice content, deposition and modification history, and to assess microclimate variation on

timescales of ka to Ma. Analyses of martian polygon morphology, guided by investigations of terrestrial analog thermal contraction crack polygons, strongly suggest the importance of excess ice in the formation and development of many martian thermal contraction crack polygons-implying the presence of an ice-rich substrate that was fractured during and subsequent to obliquity-driven depositional periods and continually modified by ongoing vapor equilibration processes. © 2009 Elsevier Inc. All rights reserved.
KW - Astrobiology
KW - Ices
KW - Mars
KW - Mars, Polar Geology
KW - Mars, Surface
SN - 10902643 (ISSN)
LA - English
J2 - Icarus
M3 - Article
DB - Scopus
N1 - Export Date: 23 February 2023; Cited By: 105; Correspondence Address: J.S. Levy; Department of Geological Sciences, Brown University, Providence, RI 02912, United States; email: joseph_levy@brown.edu; CODEN: ICRSA
ER -

TY - JOUR
AU - Gómez Felipe, F.
AU - Mateo-Martí, E.
AU - Prieto-Ballesteros, O.
AU - Martín-Gago, J.
AU - Amils, R.
TI - Protection of chemolithoautotrophic bacteria exposed to simulated Mars environmental conditions
PY - 2010
T2 - Icarus
VL - 209
IS - 2
SP - 482
EP - 487
DO - 10.1016/j.icarus.2010.05.027
UR - https://www.scopus.com/inward/record.uri?eid=2-s2.0-77956269580&doi=10.1016%2fj.icarus.2010.05.027&partnerID=40&md5=8c663f6035375751ba09747201d045bb
AD - Centro de Astrobiología (CSIC-INTA), Carretera de Torrejón-Ajalvir, 28850 Madrid, Km 4, Torrejón de Ardoz, Spain
AD - Centro de Biología Molecular Severo Ochoa (UAM-CSIC), Universidad Autónoma de Madrid, Cantoblanco, 28049 Madrid, Spain
AB - Current surface conditions (strong oxidative atmosphere, UV radiation, low temperatures and xeric conditions) on Mars are considered extremely challenging for life. The question is whether there are any features on Mars that could exert a protective effect against the sterilizing conditions detected on its surface. Potential habitability in the subsurface would increase if the overlaying material played a protective role. With the aim of evaluating this possibility we studied the viability of two

microorganisms under different conditions in a Mars simulation chamber. An acidophilic chemolithotroph isolated from Río Tinto belonging to the Acidithiobacillus genus and Deinococcus radiodurans, a radiation resistant microorganism, were exposed to simulated Mars conditions under the protection of a layer of ferric oxides and hydroxides, a Mars regolith analogue. Samples of these microorganisms were exposed to UV radiation in Mars atmospheric conditions at different time intervals under the protection of 2 and 5. mm layers of oxidized iron minerals. Viability was evaluated by inoculation on fresh media and characterization of their growth cultures. Here we report the survival capability of both bacteria to simulated Mars environmental conditions. © 2010 Elsevier Inc.
KW - Astrobiology
KW - Mars
KW - Mars, Surface
KW - Regoliths
SN - 10902643 (ISSN)
LA - English
J2 - Icarus
M3 - Article
DB - Scopus
N1 - Export Date: 23 February 2023; Cited By: 35; Correspondence Address: F. Gómez; Centro de Astrobiología (CSIC-INTA), Carretera de Torrejón-Ajalvir, 28850 Madrid, Km 4, Torrejón de Ardoz, Spain; email: gomezgf@inta.es; CODEN: ICRSA
ER -

TY - JOUR
TI - Two rovers to the same site on mars, 2018: Possibilities for cooperative science
PY - 2010
T2 - Astrobiology
VL - 10
IS - 7
SP - 663
EP - 685
DO - 10.1089/ast.2010.0526
UR - https://www.scopus.com/inward/record.uri?eid=2-s2.0-77958166354&doi=10.1089%2fast.2010.0526&partnerID=40&md5=a5adb039835cdd7451e0b821caff8ca8
AB - Within the framework of the proposed joint NASA/ESA 2018 mission to Mars, the 2-Rover International Science Analysis Group (2R-iSAG) committee was convened by theMars Exploration ProgramAnalysis Group (MEPAG) to evaluate the potential for incremental science return through the simultaneous operation at the same landing site of two rovers, specifically, ESA's ExoMars and aNASA-sourced rover concept designated here as MAX-C (Mars Astrobiology Explorer-Cacher). The group was asked to consider collaborative science opportunities fromtwo perspectives: (1) no change to either rover and (2) some change allowed. As presently planned and envisioned, the ExoMars and MAX-C rovers would have complementary scientific objectives and payloads. Initiated in 2002 and currently approved for launch in 2018, ESA's ExoMars has the following scientific objectives: (1) to search for signs of past and

present life and (2) to characterize the subsurface in terms of its physical structure, the presence of water/ice, and its geochemistry. The payload selected to achieve these goals is centered on the ability to obtain samples from the subsurface with a 2m drill. The payload comprises panoramic and high-resolution cameras and a close-up imager (microscope) as well as a ground-penetrating radar to characterize the surface and subsurface environment and to choose relevant sites for drilling. Infrared spectroscopy would provide downhole mineralogy, while the mineralogy of the drilled materials would be obtained by IR/Raman spectroscopy and X-ray diffraction. Laser desorption-gas chromatography-mass spectrometry and pyrolysis gas chromatography-mass spectrometry would determine the composition of organic molecules, including any chiral preference in molecular structure. A life marker chip is designed to detect and identify markers of fossil or extant life. The currently proposed objectives of MAX-C are to cache suitable samples fromwell-characterized sites that might contain evidence of past life and prebiotic chemistry in preparation for a possible future Mars Sample Return (MSR) mission. The emphasis is on detailed site evaluation to determine the potential for past habitability and preservation of physical and chemical biosignatures. The strawman payload (which has not been selected) is therefore likely to include instrumentation for surface characterization, for example: an abrading tool; a 5 cm drill; a panoramic camera and near-IR spectrometer; a set of armmounted instruments capable of interrogating the abraded surfaces by creating co-registered 2-Dmaps of visual texture,major element geochemistry, mineralogy, and organic geochemistry; and a rock core acquisition, encapsulation, and caching system. The value of collaborative activity can only be judged with respect to a stated scientific objective. To this end, the previously stated objectives of ExoMars and MAX-C as independent entities have been analyzed for significant common aspects. We conclude that these two rovers have two crucial shared objectives that could, in fact, form the basis of highly significant collaborative exploration activity. We therefore propose the following set of shared scientific objectives for a 2018 dual rover mission that consists of both a shared component and an independent component. (1) At a site interpreted to contain evidence of past environments with high habitability potential and high preservation potential for physical and chemical biosignatures, (a) evaluate the paleoenvironmental conditions, (b) assess the potential for preservation of biotic/prebiotic signatures, (c) search for possible evidence of past life and prebiotic chemistry. (2) Collect, document, and package in a suitable manner a set of samples sufficient to achieve the scientific objectives of a possible future sample return mission. Achieving these shared objectives would result in greater science return than would be likelywith two independent rovers. Because the rovers would not be identical, they would have separate capabilities that could be exercised independently in addition to their contributions to the above shared objectives. Separate objectives for ExoMars would include (3) characterize the stratigraphy of ancient rocks and the aqueous/ geochemical environment as a function of depth in the shallow subsurface (up to 2m depth) and (4) search for possible signs of present life; and for MAX-C (5) characterize exposed sequences of

geological units across a lateral extent of several kilometers and document geological and geochemical variation at scales from 103 down to 10-5 m. The proposed payloads for the ExoMars and MAX-C rovers have complementary capability. Most obviously, Exo-Mars plans vertical exploration capabilities, via a drill, that would not be present on MAX-C; and MAX-C would have better horizontal mobility and rapid reconnaissance capabilities. A primary finding of this analysis is that, given this complementarity and the scientific objectives listed above, there are a number of ways in which cooperative exploration activity by these two rovers would add significant value without the need to make hardware changes to either. For instance, MAX-C could enhance the scintific value of Exo-Mars drilling operations by exploring and gathering data to help choose drill sites and better characterize the geological context of the drill samples. If some hardware change is allowed, even more important scientific value could be added through cooperative action. For example, if one or more of the ExoMars samples from depth could be added to the MAX-C sample cache, it could represent a major upgrade to the sample collection that could be returned by a later mission. If a hardware modification somewhere in the system is possible, we have concluded that the following four changes would have the most beneficial impact on the total science return of a possible two-rover mission: (1) Landing hazard avoidance to allow landing in a mixed-terrain site; (2) Improvements to the ExoMars and MAX-C sample transfer systems to allow subsurface ExoMars samples to be cached for possible return to Earth; (3) An ability to command and receive adequate data from each rover twice per sol to significantly enhance efficient surface science operations; (4) Extension of the ExoMars roving capabilities to ~10 km and its nominal lifetime from 180 to 360 sols. To be complete, carrying out cooperative two-rover science activities would imply making certain compromises for each rover. Some important consequences of carrying out cooperative activity include (1) less time available for pursuing each rover's independent objectives, (2) the need to share a landing site that might not be optimized for either rover (e.g., safe site for sky crane and pallet, ExoMars restrictions for a "go-to" site, need for hazard avoidance), and (3) the need for some hardware modifications. The cooperative added value of these activities, however, warrants their consideration. © Copyright 2010, Mary Ann Liebert, Inc.
KW - Cooperative Behavior
KW - Exobiology
KW - Extraterrestrial Environment
KW - Mars
KW - Research
KW - Space Flight
KW - Spacecraft
KW - United States
KW - United States National Aeronautics and Space Administration
KW - Water
KW - Anasa
KW - Emmelichthyidae
KW - water
KW - article

KW - astrobiology
KW - astronomy
KW - chemistry
KW - cooperation
KW - government
KW - instrumentation
KW - methodology
KW - microclimate
KW - organization and management
KW - research
KW - space flight
KW - United States
SN - 15311074 (ISSN)
C2 - 20932131
LA - English
J2 - Astrobiology
M3 - Article
DB - Scopus
N1 - Export Date: 23 February 2023; Cited By: 6
ER -

TY - JOUR
AU - Shostak, S.
TI - What et will look like and why should we care
PY - 2010
T2 - Acta Astronautica
VL - 67
IS - 9-10
SP - 1025
EP - 1029
DO - 10.1016/j.actaastro.2010.06.028
UR - https://www.scopus.com/inward/record.uri?eid=2-s2.0-77956063362&doi=10.1016%2fj.actaastro.2010.06.028&partnerID=40&md5=f611d911625e690947c61a51d0dfcfb4
AD - SETI Institute, Mountain View, CA 94043, 515 North Whisman Road, United States
AB - Our experiments to find extraterrestrial life are predicated on the assumption that it is most likely to be found on so-called "habitable worlds." These are planets and moons where surface liquid water exists, and atmospheres of light gases are found. Our searches presume that life on other worlds has a biochemistry at least somewhat similar to our own. While these postulates might be our best guide for finding biology, they could be misleading us in the search for extraterrestrial intelligence (SETI). Timescale arguments suggest that shortly after a sentient species invents the technology for communication, it develops synthetic intelligence. Consequently, SETI's targeted searches of star systems that might have habitable planets in the conventional sense may be chasing a very short-lived prey. In this paper, we discuss what the implications of post-biological intelligence might have in directing our SETI experiments. © 2010 Elsevier Ltd. All rights reserved.
KW - Artificial intelligence
KW - Biochemistry
KW - Space research

KW - Conventional sense
KW - Extraterrestrial life
KW - Liquid water
KW - Search for extraterrestrial intelligences
KW - SETI
KW - Star systems
KW - Strategies
KW - Synthetic intelligence
KW - Time-scales
KW - Planets
KW - Artificial intelligence
KW - Extraterrestrial life
KW - SETI
KW - Strategies
SN - 00945765 (ISSN)
LA - English
J2 - Acta Astronaut
M3 - Article
DB - Scopus
N1 - Export Date: 23 February 2023; Cited By: 14; Correspondence Address: S. Shostak; SETI Institute, Mountain View, CA 94043, 515 North Whisman Road, United States; email: sshostak@seti.org; CODEN: AASTC
ER -

TY - JOUR
AU - Von Paris, P.
AU - Gebauer, S.
AU - Godolt, M.
AU - Grenfell, J.L.
AU - Hedelt, P.
AU - Kitzmann, D.
AU - Patzer, A.B.C.
AU - Rauer, H.
AU - Stracke, B.
TI - The extrasolar planet Gliese 581d: A potentially habitable planet?
PY - 2010
T2 - Astronomy and Astrophysics
VL - 522
IS - 2
C7 - A23
DO - 10.1051/0004-6361/201015329
UR - https://www.scopus.com/inward/record.uri?eid=2-s2.0-78049344641&doi=10.1051%2f0004-6361%2f201015329&partnerID=40&md5=691fd51b7331841755559b0e0837672c
AD - Institut für Planetenforschung, Deutsches Zentrum für Luft- und Raumfahrt, 12489 Berlin, Rutherfordstr. 2, Germany
AD - Zentrum für Astronomie und Astrophysik, Technische Universität Berlin, 10623 Berlin, Hardenbergstr. 36, Germany
AD - CNRS, UMR 5804, Laboratoire d'Astrophysique de Bordeaux, 33271 Floirac Cedex, 2 rue de l'Observatoire, BP 89, France
AD - Université de Bordeaux, Observatoire Aquitain des Sciences de l'Univers, 33271 Floirac Cedex, 2 rue de l'Observatoire, BP 89,

France
AB - Aims. The planetary system around the M star Gliese 581 contains at least three close-in potentially low-mass planets, Gl 581c, d, and e. In order to address the question of the habitability of Gl 581d, we performed detailed atmospheric modeling studies for several planetary scenarios. Methods. A 1D radiative-convective model was used to calculate temperature and pressure profiles of model atmospheres, which we assumed to be composed of molecular nitrogen, water, and carbon dioxide. The model allows for changing surface pressures caused by evaporation/condensation of water and carbon dioxide. Furthermore, the treatment of the energy transport has been improved in the model to account in particular for high CO2, high-pressure Super-Earth conditions. Results. For four high-pressure scenarios of our study, the resulting surface temperatures were above 273 K, indicating a potential habitability of the planet. These scenarios include three CO2- dominated atmospheres (95% CO2 concentration with 5, 10, and 20 bar surface pressure) and a high-pressure CO2-enriched atmosphere (5% CO2 concentration with 20 bar surface pressure). For all other considered scenarios, the calculated Gl 581d surface temperatures were below the freezing point of water, suggesting that Gl 581d would not be habitable then. The results for our CO2-dominated scenarios confirm very recent model results by Wordsworth et al. (2010). However, our model calculations imply that also atmospheres that are not CO2-dominated (i.e., 5% vmr instead of 95% vmr) could result in habitable conditions for Gl 581d. © 2010 ESO.
KW - Atmospheric temperature
KW - Carbon dioxide
KW - Planets
KW - Stars
KW - Surface properties
KW - Atmospheric modeling
KW - Close-in
KW - Energy transport
KW - Extra solar planets
KW - Freezing point
KW - Low-mass
KW - M stars
KW - Model atmosphere
KW - Model calculations
KW - Model results
KW - Molecular nitrogen
KW - Planetary system
KW - Planetary systems
KW - Planets and satellites: atmospheres
KW - Planets and satellites: individual: Gl 581d
KW - Pressure profiles
KW - Stars: individual: Gl 581
KW - Surface pressures
KW - Surface temperatures
KW - Satellites
KW - Astrobiology
KW - Planetary systems
KW - Planets and satellites: atmospheres

KW - Planets and satellites: individual: Gl 581d
KW - Stars: individual: Gl 581
SN - 14320746 (ISSN)
LA - English
J2 - Astron. Astrophys.
M3 - Article
DB - Scopus
N1 - Export Date: 23 February 2023; Cited By: 54; Correspondence Address: P. Von Paris; Institut für Planetenforschung, Deutsches Zentrum für Luft- und Raumfahrt, 12489 Berlin, Rutherfordstr. 2, Germany; email: philip.vonparis@dlr.de; CODEN: AAEJA
ER -

TY - CHAP
AU - Raulin, F.
AU - McKay, C.
AU - Lunine, J.
AU - Owen, T.
TI - Titan's Astrobiology
PY - 2010
T2 - Titan from Cassini-Huygens
SP - 215
EP - 233
DO - 10.1007/978-1-4020-9215-2_9
UR - https://www.scopus.com/inward/record.uri?eid=2-s2.0-84891450129&doi=10.1007%2f978-1-4020-9215-2_9&partnerID=40&md5=1f34931cd174cbd030894f166ab89193
AD - LISA, University of Paris, Paris 12 and 7, France
AD - NASA-Ames, United States
AD - LPL, University of Arizona, Tucson, AZ, United States
AD - University of Hawaii, United States
AB - This chapter describes the aspects of Saturn's moon Titan of astrobiological interest. Titan's prebiotic-like chemistry is reviewed, from the high atomosphere to the surface and subsurface, using the Cassini-Huygens data, with the help of theoretical modeling and experimental simulations. Similarities with and differences from the environment of the pre-biotic Earth are presented, and the lessons to be learned for Earth's organic chemical evolution on the prebiotic Earth discussed. The question of habitability and life on and in Titan is then considered, including the possibility of an exotic type of life that might exist in the liquid methane/ ethane lakes. Finally, the relation between Titan and the destiny of life on Earth is discussed. © 2010 Springer Science+Business Media B.V.
PB - Springer Netherlands
SN - 978-140209214-5 (ISBN)
LA - English
J2 - Titan from Cassini-Huygens
M3 - Book chapter
DB - Scopus
N1 - Export Date: 23 February 2023; Cited By: 14; Correspondence Address: F. Raulin; LISA, University of Paris, Paris 12 and 7, France; email: raulin@lisa.univ-paris12.fr
ER -

TY - JOUR
AU - Guo, J.
AU - Zhang, F.
AU - Han, Z.
TI - Habitable zones of host stars during the post-MS phase
PY - 2010
T2 - Astrophysics and Space Science
VL - 327
IS - 2
SP - 233
EP - 238
DO - 10.1007/s10509-010-0321-2
UR - https://www.scopus.com/inward/record.uri?eid=2-s2.0-77951974979&doi=10.1007%2fs10509-010-0321-2&partnerID=40&md5=363fce0afc014d0d65dc8a4c2edd8d75
AD - National Astronomical Observatories/Yunnan Observatory, Chinese Academy of Sciences, Kunming 650011, China
AD - Key Laboratory for the Structure and Evolution of Celestial Objects, Chinese Academy of Sciences, Kunming 650011, China
AD - Graduate School of the Chinese Academy of Sciences, Beijing 100049, China
AB - A star will become brighter and brighter with stellar evolution, and the distance of its habitable zone will become larger and larger. Some planets outside the habitable zone of a host star during the main sequence phase may enter the habitable zone of the host star during other evolutionary phases. A terrestrial planet within the habitable zone of its host star is generally thought to be suitable for the existence of life. Furthermore, a rocky moon around a giant planet may be also suitable for life to survive, provided that the planet-moon system is within the habitable zone of its host star. Using Eggleton's code and the boundary flux of the habitable zone, we calculate the habitable zone of our Solar system after the main sequence phase. It is found that Mars' orbit and Jupiter's orbit will enter the habitable zone of the Solar system during the subgiant branch phase and the red giant branch phase, respectively. And the orbit of Saturn will enter the habitable zone of Solar during the He-burning phase for about 137 million years. Life is unlikely at any time on Saturn, as it is a giant gaseous planet. However, Titan, the rocky moon of Saturn, may be suitable for biological evolution and become another Earth during that time. For low-mass stars, there are similar habitable zones during the He-burning phase as our Solar, because there are similar core masses and luminosities for these stars during that phase. © Springer Science+Business Media B.V. 2010.
KW - Astrobiology
KW - Planets: moons
KW - Stars: horizontal branch
SN - 1572946X (ISSN)
LA - English
J2 - Astrophys. Space Sci.
M3 - Article
DB - Scopus
N1 - Export Date: 23 February 2023; Cited By: 2; Correspondence

Address: J. Guo; National Astronomical Observatories/Yunnan Observatory, Chinese Academy of Sciences, Kunming 650011, China; email: guojianpo1982@hotmail.com
ER -

TY - CONF
AU - Lali, M.
TI - Analysis and design of a human spaceflight to Mars, Europa, and Titan
PY - 2010
T2 - AIP Conference Proceedings
VL - 1208
SP - 557
EP - 565
DO - 10.1063/1.3326284
UR - https://www.scopus.com/inward/record.uri?eid=2-s2.0-76749111141&doi=10.1063%2f1.3326284&partnerID=40&md5=2dca1bc0963cdedbcd8b0a081d2430e6
AD - Department of Physics and Astrophysics, University of North Dakota, Grand Forks, ND 58203, United States
AB - Like Mars, Europa and Titan are worlds for which we have a special fascination. In the past two centuries, our curiosity has been stimulated by the possibility of the existence of life on Mars, and that it would be the first planet to be visited by humans. Nevertheless, there are several driving motivations that impel humans to survey Europa for astrobiological purposes and colonize Titan in order to utilize its resources. The biggest constraint for this manned spaceflight will however be the very long distances between Earth, Jupiter and Saturn. In consequence, this will lead to a very long mission duration which will be the main concern from the standpoint of human factors, especially based on our current technological capability in such fields. It is also important to note that all our eggs are currently in one basket like the dinosaurs before the extinction. If we do not make a push into space and all things go environmentally wrong, we will be just as extinct. This environmental exploration for such missions will help us save the planet and humans. In order to cut down on the mission duration, a fast transit method known as "one-tangent-burn" trajectory is recommended to be used instead of Hohmann transfer. But this will cost an increase in the required total ΔV. Most importantly, a rare launch-window opportunity is conceived to occur in 2078, in which these sites i.e. Mars, Europa and Titan will be aligned in such a way that they can be visited in one mission taking advantage of the gravity assists from Mars and Jupiter. This specific launch window will not recur for decades. The trajectories, orbits and planetary configurations both at the time of this launch window and arrival to these sites as well as the return missions are calculated and depicted by the computer programs designed in MATLAB for this purpose. Finally, it is shown that the gravity assist that the spacecraft receives at each swingby maneuver will drastically decrease the mission duration. © 2010 American Institute of Physics.
KW - Europa
KW - Human spaceflight
KW - Mars

KW - Mission design
KW - Titan
SN - 15517616 (ISSN); 978-073540749-7 (ISBN)
LA - English
J2 - AIP Conf. Proc.
M3 - Conference paper
DB - Scopus
N1 - Export Date: 23 February 2023; Cited By: 0; Correspondence Address: M. Lali; Department of Physics and Astrophysics, University of North Dakota, Grand Forks, ND 58203, United States; email: mehdi.lali@und.edu
ER -

TY - CONF
AU - Ryu, D.
AU - Kim, S.-W.
AU - Kim, D.W.
AU - Lee, J.-M.
AU - Lee, H.
AU - Park, W.H.
AU - Seong, S.
AU - Ham, S.-J.
TI - Integrated ray tracing simulation of annual variation of spectral bio-signatures from cloud free 3D optical earth model
PY - 2010
T2 - Proceedings of SPIE - The International Society for Optical Engineering
VL - 7819
C7 - 78190E
DO - 10.1117/12.860706
UR - https://www.scopus.com/inward/record.uri?eid=2-s2.0-78649397305&doi=10.1117%2f12.860706&partnerID=40&md5=8cbbc78ca5203286e328c36b4604c126
AD - Space Optics Laboratory, Yonsei University, South Korea
AD - Institute of Space Science and Technology, Yonsei University, South Korea
AD - College of Optical Sciences, University of Arizona, United States
AD - Atmospheric, Oceanic and Planetary Physics' and 'Astrophysics', University of Oxford, United Kingdom
AD - McDonald Observatory, University of Texas, Austin, TX, United States
AB - Understanding the Earth spectral bio-signatures provides an important reference datum for accurate de-convolution of collapsed spectral signals from potential earth-like planets of other star systems. This study presents a new ray tracing computation method including an improved 3D optical earth model constructed with the coastal line and vegetation distribution data from the Global Ecological Zone (GEZ) map. Using non-Lambertian bidirectional scattering distribution function (BSDF) models, the input earth surface model is characterized with three different scattering properties and their annual variations depending on monthly changes in vegetation distribution, sea ice coverage and illumination angle. The input atmosphere model consists of one layer with Rayleigh

scattering model from the sea level to 100 km in altitude and its radiative transfer characteristics is computed for four seasons using the SMART codes. The ocean scattering model is a combination of sun-glint scattering and Lambertian scattering models. The land surface scattering is defined with the semi empirical parametric kernel method used for MODIS and POLDER missions. These three component models were integrated into the final Earth model that was then incorporated into the in-house built integrated ray tracing (IRT) model capable of computing both spectral imaging and radiative transfer performance of a hypothetical space instrument as it observes the Earth from its designated orbit. The IRT model simulation inputs include variation in earth orientation, illuminated phases, and seasonal sea ice and vegetation distribution. The trial simulation runs result in the annual variations in phase dependent disk averaged spectra (DAS) and its associated bio-signatures such as NDVI. The full computational details are presented together with the resulting annual variation in DAS and its associated bio-signatures. © 2010 Copyright SPIE - The International Society for Optical Engineering.
KW - Disks (structural components)
KW - Distribution functions
KW - Earth (planet)
KW - Ecology
KW - Instruments
KW - Radiative transfer
KW - Ray tracing
KW - Scattering
KW - Sea ice
KW - Sea level
KW - Vegetation
KW - Disk Averaged Spectrum(Spectra)
KW - Earth models
KW - Earth observing
KW - Extra solar planets
KW - Integrated Ray Tracing
KW - Spectral Bio-signature of the Earth
KW - Terrestrial planets
KW - Three dimensional
KW - Astrobiology
KW - Disk Averaged Spectrum(Spectra)
KW - Earth Observing Simulation
KW - Extra-solar Planet
KW - Full 3D Earth Model
KW - Integrated Ray Tracing
KW - Spectral Bio-signature of the Earth
KW - Terrestrial Planet Finding
SN - 0277786X (ISSN); 978-081948315-7 (ISBN)
LA - English
J2 - Proc SPIE Int Soc Opt Eng
M3 - Conference paper
DB - Scopus
N1 - Export Date: 23 February 2023; Cited By: 7; Correspondence Address: D. Ryu; Space Optics Laboratory, Yonsei University, South Korea; email: antinneo@gmail.com; CODEN: PSISD

ER -

TY - JOUR
AU - Wu, J.
AU - Zhu, G.-W.
AU - Zhao, H.
AU - Wang, C.
AU - Li, L.
AU - Sun, Y.-Q.
AU - Guo, W.
AU - Huang, C.-L.
TI - Scientific Objectives of China-Russia Joint Mars Exploration Program YH-1
PY - 2010
T2 - Chinese Astronomy and Astrophysics
VL - 34
IS - 2
SP - 163
EP - 173
DO - 10.1016/j.chinastron.2010.04.009
UR - https://www.scopus.com/inward/record.uri?eid=2-s2.0-77952275964&doi=10.1016%2fj.chinastron.2010.04.009&partnerID=40&md5=8cdf1e46630c572805990ec5677660c0
AD - Center for Space Science and Applied Research, Chinese Academy of Sciences, Beijing 100190, China
AD - Shanghai Astronomical Observatory, Chinese Academy of Sciences, Shanghai 200030, China
AB - Compared with other planets, Mars is a planet most similar with the earth and most possible to find the extraterrestrial life on it, and therefore especially concerned about by human beings. In recent years, some countries have launched Mars probes and announced their manned Mars exploration programs. China has become the fifth country in the world to launch independently artificial satellites, and the third country able to carry out an independent manned space program. However, China is just at the beginning of deep space explorations. In 2007, China and Russia signed an agreement on a joint Mars exploration program by sending a Chinese micro-satellite Yinghuo-1 (YH-1) to the Mars orbit. Once YH-1 enters its orbit, it will carry out its own exploration, as well as the joint exploration with the Russian Phobos-Grunt probe. This paper summarizes the scientific background and objectives of YH-1 and describes briefly its payloads for realizing these scientific objectives. In addition, the main exploration tasks of YH-1 and a preliminary prospect on its exploration results are also given. © 2010.
KW - Planets and satellites: individual (Mars)
KW - Solar wind
KW - Space vehicles: instruments
KW - Sun: magnetosphere
SN - 02751062 (ISSN)
LA - English
J2 - Chin. Astron. Astrophys.
M3 - Article
DB - Scopus
N1 - Export Date: 23 February 2023; Cited By: 7; Correspondence

Address: J. Wu; Center for Space Science and Applied Research, Chinese Academy of Sciences, Beijing 100190, China; email: wuji@cssar.ac.cn; CODEN: CASGE
ER -

TY - JOUR
AU - Chavadar, M.S.
AU - Bajekal, S.S.
TI - Microaerophilic magnetotactic bacteria from Lonar Lake, India
PY - 2010
T2 - Journal of Pure and Applied Microbiology
VL - 4
IS - 2
SP - 681
EP - 685
UR - https://www.scopus.com/inward/record.uri?eid=2-s2.0-83455221177&partnerID=40&md5=8bd94383f0c6246805d209aedad2187c
AD - Department of Microbiology, Yashwantrao Chavan College of Science, Karad Vidyanagar, Karad- 415 124, India
AB - Magnetotactic bacteria (MTB), the major constituents of natural microbial communities in sediments and chemically stratified water columns. The hallmark feature of MTB is the presence of unique, nanometer sized, intracellular inclusions the 'magnetosomes' that confers 'magnetotaxis 'to these cells. The 'magnetosomes', the 'magnetotaxis' and the possible applications of these bacteria grown curiosity about the MTB. Presently they received the celebrity status, as MTB might be tool for searching extraterrestrial life. Lonar Lake is the only meteorite impact crater in the world formed basaltic rock. The closed basin lake has a unique environment characterized by high alkalinity and salinity. Evidences of magnetic activity in the surrounding rocks and soil are also found, and reported. The role of MTB in biomineralization encouraged us to find such bacteria in this unique environment. The sediments samples collected from the lake were used to isolate the microaerophilic magnetotactic bacteria (mMTB). The mMTB were enriched by 'magnetic collection' method, isolated by the 'capillary racetrack' (CRT) method and cultivated under microaerophilic conditions using a chemically defined medium. The three isolated mMTB showed were tested for response to the magnetic field using magnetic field in a 'hanging drop' under a microscope and also the on semisolid agar medium. They showed migration towards the South Pole of the magnet and precise alignment at the edge of the hanging drop. On semisolid agar also they showed migration toward magnetic South Pole. The intracellular iron content of the isolated mMTB cultures was measured using atomic absorption spectroscopy (AAS), which indirectly confirms the presence of magnetosomes in these the isolated MTB; similar result was obtained by R.P. Blakemore during discovery of MTB.
KW - alkalinity
KW - article
KW - astronomy
KW - atomic absorption spectrometry
KW - bacterium
KW - basalt

KW - biomineralization
KW - India
KW - lake
KW - magnetic field
KW - magnetosome
KW - magnetotactic bacterium
KW - microbial community
KW - nonhuman
KW - salinity
KW - Capillary race track method
KW - Magnetic collection
KW - Magnetosome
KW - Magnetotaxis
SN - 09737510 (ISSN)
LA - English
J2 - J. Pure Appl. Microbiol.
M3 - Article
DB - Scopus
N1 - Export Date: 23 February 2023; Cited By: 1; Correspondence Address: M. S. Chavadar; Department of Microbiology, Yashwantrao Chavan College of Science, Karad Vidyanagar, Karad- 415 124, India; email: maheshchavadar@yahoo.co.in
ER -

TY - JOUR
AU - Keresztturi, A.
AU - Möhlmann, D.
AU - Berczi, Sz.
AU - Ganti, T.
AU - Horvath, A.
AU - Kuti, A.
AU - Sik, A.
AU - Szathmary, E.
TI - Indications of brine related local seepage phenomena on the northern hemisphere of Mars
PY - 2010
T2 - Icarus
VL - 207
IS - 1
SP - 149
EP - 164
DO - 10.1016/j.icarus.2009.10.012
UR - https://www.scopus.com/inward/record.uri?eid=2-s2.0-77950297517&doi=10.1016%2fj.icarus.2009.10.012&partnerID=40&md5=637947a20ce99ba9469a351619abd719
AD - Collegium Budapest, Institute for Advanced Study, H-1041 Budapest, Szentharomsag 2, Hungary
AD - DLR Institut für Planetenforschung, D-12489 Berlin, Rutherfordstr. 2, Germany
AD - Eotvos Lorand University of Sciences, Institute of Physics, H-1117 Budapest, Pázmány 1/A, Hungary
AD - Eotvos Lorand University of Sciences, Institute of Geography and Earth Sciences, H-1117 Budapest, Pázmány 1/c, Hungary
AD - Eotvos Lorand University of Sciences, Institute of Physics,

Department of Astronomy, H-1117 Budapest, Pázmány 1/c, Hungary
AD - Konkoly Observatory, H-1525 Budapest, Pf. 67, Hungary
AB - Springtime low albedo features, called Dark Dune Spots, on the seasonal frost covered dunes on Mars between 77°N and 84°N latitude have been analyzed. Two groups of these spots have been identified: "small" and "large" ones, where large spots have diameters above 4 m, and complex internal structure. From these "large" spots branching seepage-like features emanate and grow on the steep slopes. They show a characteristic sequence of changes: first only wind-blown features emanate from them, while later a bright circular and elevated ring forms, and dark seepage-features start from the spots. These streaks grow with a speed between 0.3 m/day and 7 m/day respectively, first only from the spots, later from all along the dune crest. During this "seepage period" the temperature is between 150 K and 180 K at a 3-9 km spatial resolution scale, indicating that CO2 ice-free parts must be present at the observed dark spots. Around the receding northern seasonal CO2 cap, an annulus of water ice lags behind, which is probably present in the spots too where the CO2 frost has sublimated. Our model estimates show in the present work and in Kereszturi et al. (Kereszturi, A., Möhlmann, D., Berczi, Sz., Ganti, T., Kuti, A., Sik, A., Horvath, A. [2009b]. Icarus 201, 492-503) that the warming driven by solar insolation may produce not only interfacial water, but also bulk brines around the dune grains. The brine can support the movement of liquids and dune grains, enhances the sublimation of CO2 frost, and produce the dark features, as well as liquid modifies the optical properties of the surface. Signs of movement of dune material after the total defrosting of the terrain is also visible but it is uncertain because of the limit of resolution. In our previous work (Kereszturi et al., 2009b) we showed that resembling seepage-like streaks at the southern hemisphere might have been formed by ephemeral interfacial water, as well as these northern features. Such wet environments may have astrobiological importance too. © 2009 Elsevier Inc. All rights reserved.
KW - Ices
KW - Mars, Surface
SN - 10902643 (ISSN)
LA - English
J2 - Icarus
M3 - Article
DB - Scopus
N1 - Export Date: 23 February 2023; Cited By: 36; Correspondence Address: A. Kereszturi; Collegium Budapest, Institute for Advanced Study, H-1041 Budapest, Szentharomsag 2, Hungary; email: akos@colbud.hu; CODEN: ICRSA
ER -

TY - JOUR
AU - Spiegel, D.S.
AU - Raymond, S.N.
AU - Dressing, C.D.
AU - Scharf, C.A.
AU - Mitchell, J.L.
TI - Generalized milankovitch cycles and long-term climatic

habitability
PY - 2010
T2 - Astrophysical Journal
VL - 721
IS - 2
SP - 1308
EP - 1318
DO - 10.1088/0004-637X/721/2/1308
UR - https://www.scopus.com/inward/record.uri?eid=2-s2.0-78149257429&doi=10.1088%2f0004-637X%2f721%2f2%2f1308&partnerID=40&md5=f0fddfeeb9a2de2ca75e05397be61276
AD - Department of Astrophysical Sciences, Princeton University, Peyton Hall, Princeton, NJ 08544, United States
AD - Kavli Institute for Theoretical Physics, UCSB, Santa Barbara, CA 93106-4030, United States
AD - Université de Bordeaux, Observatoire Aquitain des Sciences de L'Univers, F-33271 Floirac Cedex, 2 rue de l'Observatoire, France
AD - CNRS, UMR 5804, Laboratoire D'Astrophysique de Bordeaux, F-33271 Floirac Cedex, 2 rue de l'Observatoire, France
AD - Columbia Astrobiology Center, Columbia Astrophysics Lab., Columbia University, New York, NY 10027, 550 West 120th Street, United States
AD - Department of Astronomy, Columbia University, New York, NY 10027, 550 West 120th Street, United States
AD - Institute for Advanced Study, School of Natural Sciences, Princeton, NJ 08540, 1 Einstein Drive, United States
AD - Earth and Space Sciences, Atmospheric and Oceanic Sciences, IGPP, UCLA, Los Angeles, CA 90095, 595 Charles Young Drive East, United States
AB - Although Earth's orbit is never far from circular, terrestrial planets around other stars might experience substantial changes in eccentricity. Eccentricity variations could lead to climate changes, including possible "phase transitions" such as the snowball transition (or its opposite). There is evidence that Earth has gone through at least one globally frozen, "snowball" state in the last billion years, which it is thought to have exited after several million years because global ice-cover shut off the carbonate-silicate cycle, thereby allowing greenhouse gases to build up to sufficient concentration to melt the ice. Due to the positive feedback caused by the high albedo of snow and ice, susceptibility to falling into snowball states might be a generic feature of water-rich planets with the capacity to host life. This paper has two main thrusts. First, we revisit one-dimensional energy balance climate models as tools for probing possible climates of exoplanets, investigate the dimensional scaling of such models, and introduce a simple algorithm to treat the melting of the ice layer on a globally frozen planet. We show that if a terrestrial planet undergoes Milankovitch-like oscillations of eccentricity that are of great enough magnitude, it could melt out of a snowball state. Second, we examine the kinds of variations of eccentricity that a terrestrial planet might experience due to the gravitational influence of a giant companion. We show that a giant planet on a sufficiently eccentric orbit can excite extreme eccentricity oscillations in the orbit of a habitable terrestrial planet. More generally, these two

results demonstrate that the long-term habitability (and astronomical observables) of a terrestrial planet can depend on the detailed architecture of the planetary system in which it resides. © 2010. The American Astronomical Society. All rights reserved.
KW - Astrobiology
KW - Planetary systems
KW - Radiative transfer
PB - Institute of Physics Publishing
SN - 0004637X (ISSN)
LA - English
J2 - Astrophys. J.
M3 - Article
DB - Scopus
N1 - Export Date: 23 February 2023; Cited By: 73; Correspondence Address: D. S. Spiegel; Department of Astrophysical Sciences, Princeton University, Peyton Hall, Princeton, NJ 08544, United States; email: dsp@astro.princeton.edu
ER -

TY - JOUR
AU - Chela-Flores, J.
TI - Instrumentation for the search for habitable ecosystems in the future exploration of Europa and Ganymede
PY - 2010
T2 - International Journal of Astrobiology
VL - 9
IS - 2
SP - 101
EP - 108
DO - 10.1017/S1473550410000029
UR - https://www.scopus.com/inward/record.uri?eid=2-s2.0-77954516419&doi=10.1017%2fS1473550410000029&partnerID=40&md5=34b81df37e954b2f7403a23c29fed514
AD - Abdus Salam ICTP, 34014 Trieste, Strada Costiera 11, Italy
AD - Instituto de Estudios Avanzados, IDEA, Caracas 1015A, Venezuela
AB - The extensive evidence of an ocean over a silicate nucleus makes Europa a candidate for the emergence of a second evolutionary pathway of autochthonous life. We argue that the most urgent question in astrobiology is the origin of habitable ecosystems (a question in geochemistry), rather than the alternative search for the origin of life itself (a question in chemical evolution). Since certain Solar System bodies may share a similar geophysical past with Earth, our more modest approach forces upon us the question: Can available instrumentation be the pioneer in the discovery of habitable ecosystems in geophysical environments similar to the early Earth? It will be shown that a central piece in this dilemma is the chemical element sulphur (S). The Europan non-ice surficial elements that distort the water-ice absorption bands were found to be widespread, patchy and, most likely, endogenous. The Galileo Mission discovered these patches, which were subsequently confirmed by the 2007 flyby of New Horizons. We argue that penetrators should be inserted into orbital probes in the future exploration of Jupiter's System. Penetrators provide what could be a key instrument

in the exploration of Europa, given the adverse space weather in its environment due to the Jovian magnetosphere and radiation. Indeed, there are alternative views on the radiation-induced S-cycles produced on the surficial molecules that are present on the icy surface; however, S is common to both interpretations. Hence, mass spectrometry should be an essential part of any future payload. The largest S-fractionations are due to microbial reduction and not to thermochemical processes, allowing a test of the hypothesis for the origin of habitable ecosystems. The microbial fractionation of stable S-isotopes argue in favour of penetrators for the survey of the surfaces of both Europa and Ganymede. Copyright © Cambridge University Press 2010.
KW - biogeochemistry
KW - biomarker
KW - exobiology
KW - Jupiter
KW - magnetosphere
KW - mass spectrometry
KW - origin of life
KW - biogeochemistry
KW - biomarkers
KW - dilational bands
KW - dissimilatory sulphate reduction
KW - Europa
KW - Europa Jupiter System Mission
KW - Ganymede
KW - landing sites
KW - LAPLACE Mission
KW - mass spectrometry
KW - sulphur patches on the Europan surface
SN - 14753006 (ISSN)
LA - English
J2 - Int. J. Astrobiology
M3 - Article
DB - Scopus
N1 - Export Date: 23 February 2023; Cited By: 14; Correspondence Address: J. Chela-Flores; Abdus Salam ICTP, 34014 Trieste, Strada Costiera 11, Italy; email: chelaf@ictp.it
ER -

TY - CONF
AU - Crawford, I.A.
AU - Cockell, C.S.
TI - Astrobiology on the Moon
PY - 2010
T2 - Astronomy and Geophysics
VL - 51
IS - 4
SP - 4.11
EP - 4.14
DO - 10.1111/j.1468-4004.2010.51411.x
UR - https://www.scopus.com/inward/record.uri?eid=2-s2.0-77955239103&doi=10.1111%2fj.1468-4004.2010.51411.x&partnerID=40&md5=0904896c6594ed336b6cb2d276b5e120

AD - Birkbeck College, London, United Kingdom
AD - Open University, United Kingdom
AB - Meeting reportIan Crawford and Charles Cockell report on an RAS Specialist Discussion Meeting held on Friday 14 May 2010. © 2010 Royal Astronomical Society.
KW - Geophysics
KW - Geology
PB - Blackwell Publishing Ltd
SN - 13668781 (ISSN)
LA - English
J2 - Astron. Geophys.
M3 - Conference paper
DB - Scopus
N1 - Export Date: 23 February 2023; Cited By: 3; Correspondence Address: I.A. Crawford; Birkbeck College, London, United Kingdom; email: i.crawford@ucl.ac.uk
ER -

TY - CONF
AU - Edwards, H.G.M.
AU - Hutchinson, I.B.
AU - Ingley, R.
TI - Raman spectroscopy of extremophiles from hot and cold deserts: An astrobiological journey from terrestrial extreme environments to planetary exploration
PY - 2010
T2 - AIP Conference Proceedings
VL - 1267
SP - 216
EP - 217
DO - 10.1063/1.3482475
UR - https://www.scopus.com/inward/record.uri?eid=2-s2.0-78649963976&doi=10.1063%2f1.3482475&partnerID=40&md5=3736cd03e2f1f689464b84ed1c073cd5
AD - Centre for Astrobiology and Extremophiles Research, School of Life Sciences, University of Bradford, Bradford, West Yorkshire, BD7 1DP, United Kingdom
AD - Department of Physics and Astronomy, University of Leicester, Leicester LE1 7RH, United Kingdom
SN - 15517616 (ISSN); 978-073540818-0 (ISBN)
LA - English
J2 - AIP Conf. Proc.
M3 - Conference paper
DB - Scopus
N1 - Export Date: 23 February 2023; Cited By: 0
ER -

TY - JOUR
AU - Raut, A.A.
AU - Bajekal, S.S.
TI - Growth of non-symbiotic nitrogen fixing microaerophiles from Lonar Crater on inorganic medium of martian soil simulant components
PY - 2010
T2 - Journal of Pure and Applied Microbiology

VL - 4
IS - 2
SP - 819
EP - 822
UR - https://www.scopus.com/inward/record.uri?eid=2-s2.0-83455239014&partnerID=40&md5=c0ee9e8d82e6d3ba0b91210edff791c0
AD - Department of Microbiology, Yashwantrao Chavan College of Science, Karad, Vidyanagar, Karad - 415 124, India
AB - Lonar Crater situated in the village Lonar (Lat. 19058', Long. 76034') in Buldhana District of Maharashtra State, India is the only one in the world formed by meteorite impact in basaltic rock. Formed some 52,000 years ago, it is today a closed basin lake that is saline and alkaline, rich in carbonates and bicarbonates. As it constitutes an extreme environment (alkalinity) and as there are no previous reports of nitrogen fixing bacteria from there, a study was undertaken to isolate and characterize nitrogen fixing bacteria adapted to this environment. Nitrogen fixing bacteria could be considered to constitute an important group of primary producers in an ecosystem due to their ability to make available a very important nutrient to other species. Though vast majorities of the species known today are chemoorganotrophs, there are many autotrophic nitrogen fixers also known. Several such bacteria have also been discovered in environments that resemble the Martian environment as well. Considering the fact that the Lonar Lake is of meteorite origin, an extraterrestrial object, it was decided to test the ability of the nitrogen fixers isolated from here to grow on Martian simulated soil. This paper presents results ofthat study. Of the 32 bacterial isolates obtained, only four were capable of growing on the simulated Martian soil component extract medium that was lacking in both nitrogen as well as carbon. This meant they were both fixers of atmospheric nitrogen as well as carbon. This holds encouraging signs to the search for extraterrestrial life using such organisms as possible indicators.
KW - Bacteria (microorganisms)
KW - bicarbonate
KW - carbon
KW - carbonic acid
KW - nitrogen
KW - alkalinity
KW - article
KW - astronomy
KW - bacterial growth
KW - bacterium isolation
KW - basalt
KW - ecosystem
KW - India
KW - lake
KW - nitrogen fixation
KW - nonhuman
KW - Chemoorganotrophs
KW - Extraterrestrial
KW - Lonar
KW - Martian soil
KW - Meteorite

KW - Nitrogen fixers
KW - Simulated
SN - 09737510 (ISSN)
LA - English
J2 - J. Pure Appl. Microbiol.
M3 - Article
DB - Scopus
N1 - Export Date: 23 February 2023; Cited By: 0; Correspondence Address: A. A. Raut; Department of Microbiology, Yashwantrao Chavan College of Science, Karad, Vidyanagar, Karad - 415 124, India; email: avinashraut@aol.in
ER -

TY - CONF
AU - Alajtal, A.I.
AU - Edwards, H.G.M.
AU - Scowen, I.J.
TI - Raman spectroscopic analysis of minerals and organic molecules of relevance to astrobiology
PY - 2010
T2 - Analytical and Bioanalytical Chemistry
VL - 397
IS - 1
SP - 215
EP - 221
DO - 10.1007/s00216-009-3362-z
UR - https://www.scopus.com/inward/record.uri?eid=2-s2.0-77951257092&doi=10.1007%2fs00216-009-3362-z&partnerID=40&md5=7d6cd34b5e1bbc630d5e1e423273624f
AD - Centre for Astrobiology and Extremophiles Research, School of Life Sciences, University of Bradford, Bradford, West Yorkshire BD7 1DP, United Kingdom
AD - University Analytical Centre, University of Bradford, Bradford, West Yorkshire BD7 1DP, United Kingdom
AB - Characteristic geological features and hydrated minerals recently found on the surface of Mars by the NASA planetary rovers Spirit and Opportunity suggest that a possible biosphere could have once existed there. Analytical instrumentation protocols for the unequivocal detection of biomarkers in suitable geological matrices are critical for future unmanned explorations, including the forthcoming ESA-ExoMars mission scheduled for 2018. Raman spectroscopy is currently a part of the Pasteur instrumentation suite of the ExoMars mission scheduled for 2018 for the remote detection of extant or extinct life signatures in the Martian surface and subsurface. Terrestrial analogues of Martian sites have been identified, and the biogeological modifications incurred as a result of extremophilic survival activity have been studied. Polyaromatic hydrocarbons (PAHs) are recognised as a class of degradation product that occur from biological processes terrestrially. In this work, various concentrations of polyaromatic hydrocarbons in matrices of gypsum, calcite and quartz have been investigated by Raman microspectrometry to determine the lowest detectable organic levels. The studies are conceived in simulation of their potential PAHs identification in geobiological conditions

in Martian scenarios. Two laser source wavelengths, namely, 785 and 633 nm, were adopted to excite Raman spectra from the PAHs, which represent degraded carbons and therefore potentially provide a key bimolecular marker of ancient life. © 2009 Springer-Verlag.
KW - Biological Markers
KW - Calcium Carbonate
KW - Calcium Sulfate
KW - Exobiology
KW - Minerals
KW - Polycyclic Hydrocarbons, Aromatic
KW - Quartz
KW - Space Flight
KW - Space Simulation
KW - Spectrum Analysis, Raman
KW - Emmelichthyidae
KW - Carbonate minerals
KW - Gypsum
KW - Hydrates
KW - Hydrocarbons
KW - Instruments
KW - Minerals
KW - NASA
KW - Oxide minerals
KW - Photodegradation
KW - Planetary landers
KW - Quartz
KW - Raman scattering
KW - Raman spectroscopy
KW - Spectroscopic analysis
KW - biological marker
KW - calcium carbonate
KW - calcium sulfate
KW - mineral
KW - polycyclic aromatic hydrocarbon
KW - silicon dioxide
KW - Analytical Instrumentation
KW - Biological process
KW - Degradation products
KW - ExoMars mission
KW - Geological features
KW - Instrumentation suite
KW - Laser sources
KW - Mars analogue
KW - Martian surface
KW - Organic molecules
KW - Planetary rovers
KW - Polyaromatic hydrocarbons
KW - Raman microspectrometry
KW - Raman spectra
KW - Raman spectroscopic
KW - Remote detection
KW - Surface of Mars
KW - Unmanned explorations
KW - biology

KW - chemistry
KW - conference paper
KW - Raman spectrometry
KW - space flight
KW - Organic minerals
KW - Mars analogue
KW - Minerals
KW - PAHs
KW - Raman spectroscopy
SN - 16182650 (ISSN)
C2 - 20020110
LA - English
J2 - Anal. Bioanal. Chem.
M3 - Conference paper
DB - Scopus
N1 - Export Date: 23 February 2023; Cited By: 12; Correspondence Address: H. G. M. Edwards; Centre for Astrobiology and Extremophiles Research, School of Life Sciences, University of Bradford, Bradford, West Yorkshire BD7 1DP, United Kingdom; email: h.g.m.edwards@bradford.ac.uk; CODEN: ABCNB
ER -

TY - JOUR
AU - Loftus, D.J.
AU - Rask, J.C.
AU - McCrossin, C.G.
AU - Tranfield, E.M.
TI - The Chemical Reactivity of Lunar Dust: From Toxicity to Astrobiology
PY - 2010
T2 - Earth, Moon and Planets
VL - 107
IS - 1
SP - 95
EP - 105
DO - 10.1007/s11038-010-9376-x
UR - https://www.scopus.com/inward/record.uri?eid=2-s2.0-79952446272&doi=10.1007%2fs11038-010-9376-x&partnerID=40&md5=1018409da629b38f2e5d7c6293e0378b
AD - NASA Ames Research Center, Moffett Field, CA 94035, United States
AB - The chemical reactivity of lunar dust is an important topic of inquiry, of fundamental scientific value and of practical relevance to human exploration of the Moon. Lunar specimens brought back to Earth by the Apollo astronauts provide a key resource for ground-based studies which help to define the initial avenues of inquiry. Even among the limited samples obtained from equatorial exploration sites, however, chemical reactivity analyses indicates that lunar dust is heterogeneous, a finding that parallels heterogeneity revealed by remote sensing studies. The region-to-region variability of lunar dust argues that a full understanding of its chemical reactivity will require in situ analysis, on a region-to-region basis. The data from such investigations will help to shape our understanding of the potential for lunar dust toxicity, and will

provide insight into the types of reactions that may occur with when lunar dust interacts with organic molecules on the surface of the Moon. © 2011 Springer Science+Business Media B.V. (outside the USA).
KW - dust
KW - free radical
KW - light effect
KW - lunar soil
KW - Moon
KW - reaction kinetics
KW - toxicity
KW - ultraviolet radiation
KW - Chemical reactivity
KW - Free radical
KW - Hydroxyl radical
KW - Lunar dust
KW - Moon
KW - Radiation
KW - Ultraviolet light
SN - 01679295 (ISSN)
LA - English
J2 - Earth Moon Planets
M3 - Article
DB - Scopus
N1 - Export Date: 23 February 2023; Cited By: 18; Correspondence Address: D. J. Loftus; NASA Ames Research Center, Moffett Field, CA 94035, United States; email: David.j.loftus@nasa.gov
ER -

TY - JOUR
AU - Doughty, C.E.
AU - Wolf, A.
TI - Detecting tree-like multicellular life on extrasolar planets
PY - 2010
T2 - Astrobiology
VL - 10
IS - 9
SP - 869
EP - 879
DO - 10.1089/ast.2010.0495
UR - https://www.scopus.com/inward/record.uri?eid=2-s2.0-78650054276&doi=10.1089%2fast.2010.0495&partnerID=40&md5=e45c91f7360924e1af1306c04fb7b41b
AD - Department of Global Ecology, Carnegie Institution, Stanford, CA, United States
AB - Over the next two decades, NASA and ESA are planning a series of space-based observatories to find Earth-like planets and determine whether life exists on these planets. Previous studies have assessed the likelihood of detecting life through signs of biogenic gases in the atmosphere or a red edge. Biogenic gases and the red edge could be signs of either single-celled or multicellular life. In this study, we propose a technique with which to determine whether tree-like multicellular life exists on extrasolar planets. For multicellular photosynthetic organisms on Earth, competition for light and the need to transport water and nutrients has led to a

tree-like body plan characterized by hierarchical branching networks. This design results in a distinct bidirectional reflectance distribution function (BRDF) that causes differing reflectance at different sun/view geometries. BRDF arises from the changing visibility of the shadows cast by objects, and the presence of tree-like structures is clearly distinguishable from flat ground with the same reflectance spectrum. We examined whether the BRDF could detect the existence of tree-like structures on an extrasolar planet by using changes in planetary albedo as a planet orbits its star. We used a semi-empirical BRDF model to simulate vegetation reflectance at different planetary phase angles and both simulated and real cloud cover to calculate disk and rotation-averaged planetary albedo for a vegetated and non-vegetated planet with abundant liquid water. We found that even if the entire planetary albedo were rendered to a single pixel, the rate of increase of albedo as a planet approaches full illumination would be comparatively greater on a vegetated planet than on a non-vegetated planet. Depending on how accurately planetary cloud cover can be resolved and the capabilities of the coronagraph to resolve exoplanets, this technique could theoretically detect tree-like multicellular life on exoplanets in 50 stellar systems. Key Words: TPF-Darwin-Extrasolar planets-Astrobiology-BRDF-Remote sensing-multicellular life. Astrobiology 10, 869-879. © Mary Ann Liebert, Inc.
KW - Astronomy
KW - Evolution, Planetary
KW - Exobiology
KW - Extraterrestrial Environment
KW - Models, Theoretical
KW - Planets
KW - Spacecraft
KW - Water
KW - water
KW - article
KW - astrobiology
KW - astronomy
KW - microclimate
KW - space flight
KW - theoretical model
SN - 15311074 (ISSN)
C2 - 21118020
LA - English
J2 - Astrobiology
M3 - Article
DB - Scopus
N1 - Export Date: 23 February 2023; Cited By: 10; Correspondence Address: C. E. Doughty; Carnegie Institution, Department of Global Ecology, Stanford, CA 92697-3100, United States; email: Chris.doughty@ouce.ox.ac.uk
ER -

TY - JOUR
AU - Raulin Cerceau, F.
TI - What possible life forms could exist on other planets: A

historical overview
PY - 2010
T2 - Origins of Life and Evolution of Biospheres
VL - 40
IS - 2
SP - 195
EP - 202
DO - 10.1007/s11084-010-9200-7
UR - https://www.scopus.com/inward/record.uri?eid=2-s2.0-77951769412&doi=10.1007%2fs11084-010-9200-7&partnerID=40&md5=f696cb98becd704d2d509b69d9b63ce6
AD - Centre Alexandre Koyré (UMR 8560/CNRS-EHESS-MNHN-CSI), Muséum national d'Histoire naturelle, 75005 Paris, Case postale 25 - 57 rue Cuvier, France
AB - Speculations on living beings existing on other planets are found in many written works since the Frenchman Bernard de Fontenelle spoke to the Marquise about the inhabitants of the solar system in his Entretiens sur la pluralité des mondes (1686). It was an entertainment used to teach astronomy more than real considerations about the habitability of our solar system, but it opened the way to some reflections about the possible life forms on other planets. The nineteenth century took up this idea again in a context of planetary studies showing the similarities as well as the differences of the celestial bodies orbiting our Sun. Astronomers attempted to look deeper into the problem of habitability such as Richard Proctor or Camille Flammarion, also well-known for their fine talent in popular writings. While the Martian canals controversy was reaching its height, they imagined how the living forms dwelling in other planets could be. Nowadays, no complex exo-life is expected to have evolved in our solar system. However, the famous exobiologist Carl Sagan and later other scientists, formulated audacious ideas about other forms of life in the light of recent discoveries in planetology. Through these few examples, this paper underlines the originality of each author's suggestions and the evolution and contrast of ideas about the possible life forms in the universe. © Springer Science+Business Media B.V. 2010.
KW - Exobiology
KW - Extraterrestrial Environment
KW - Life
KW - exobiology
KW - invasive species
KW - planet
KW - solar system
KW - article
KW - biology
KW - life
KW - microclimate
KW - Exotic life forms
KW - Planets habitability
KW - Plurality of worlds
KW - Xenobiology
SN - 01696149 (ISSN)
C2 - 20186488
LA - English

J2 - Orig. Life Evol. Biosph.
M3 - Article
DB - Scopus
N1 - Export Date: 23 February 2023; Cited By: 0; Correspondence Address: F. Raulin Cerceau; Centre Alexandre Koyré (UMR 8560/CNRS-EHESS-MNHN-CSI), Muséum national d'Histoire naturelle, 75005 Paris, Case postale 25 - 57 rue Cuvier, France; email: raulin@mnhn.fr; CODEN: OLEBE
ER -

TY - JOUR
AU - Segura, A.
TI - La tierra vista como exoplaneta
PY - 2010
T2 - Revista Mexicana de Ciencias Geologicas
VL - 27
IS - 2
SP - 374
EP - 385
UR - https://www.scopus.com/inward/record.uri?eid=2-s2.0-77956009247&partnerID=40&md5=7a5386461f8f9c0c479d753131aba2d6
AD - Instituto de Ciencias Nucleares, Universidad Nacional Autónoma de México, 04510 México D.F., Circuito Exterior C.U. A.Postal 70-543, Mexico
AB - Earth, as the only example of a habitable world, offers the first elements to characterize the spectra of terrestrial planets around other stars. Those planets may be detected in the next decade by missions like CoRoT and Kepler, and characterized by Terrestrial Planet finder and Darwin. In this paper, I reviewed the research that uses Earth to determine the possible characteristics of habitable worlds around other stars. Comparing Earth's characteristics with those of the terrestrial planets in the Solar System, the main properties of a habitable world have been determined. A habitable planet must have atmosphere, liquid water and the right size to keep that atmosphere and to maintain tectonic activity for long geologic periods. A habitable world could be recognized as such by the detection of biosignatures on its spectrum. Simulations of past and present Earth-like atmospheres and the knowledge of the geological evolution of our planet indicate that oxygen (O2) is an excellent signature of life, in particular if it comes along with compounds like methane and nitrous oxide. Also, the pigments used by photosynthetic organisms could generate a signature in a planet's spectrum. This signature may be similar to the chlorophyll absorption on Earth. Earthshine observations help to analyze the disk average spectrum of our planet and to determine the changes of the biosignatures given certain conditions of illumination and geometry. From such observations and models that generate disk averaged spectra of Earth it has been found that clouds are the biggest challenge to identify biosignatures and characteristics of the planetary surface in general. The atmospheric abundance of the compounds produced by life depends on the amount of ultraviolet radiation received by the planet as it drives most of the atmospheric chemistry. This radiation depends on the stellar type of the planet's parent star. The characterization of

terrestrial planets requires the knowledge of the target star properties (age, effective temperature, radiation emitted from the ultraviolet to the infrared), as well as to build spectra libraries that allow recognizing habitable worlds from those that are not.
KW - Astrobiology
KW - Biosignatures
KW - Exoplanets
KW - Habitable planets
SN - 10268774 (ISSN)
LA - Spanish
J2 - Rev. Mex. Cienc. Geol.
M3 - Article
DB - Scopus
N1 - Export Date: 23 February 2023; Cited By: 1; Correspondence Address: A. Segura; Instituto de Ciencias Nucleares, Universidad Nacional Autónoma de México, 04510 México D.F., Circuito Exterior C.U. A.Postal 70-543, Mexico; email: antigona@nucleares.unam.mx
ER -

TY - JOUR
AU - Wilson, R.
AU - Bowden, S.A.
AU - Parnell, J.
AU - Cooper, J.M.
TI - Signal enhancement of surface enhanced raman scattering and surface enhanced resonance raman scattering using in situ colloidal synthesis in microfluidics
PY - 2010
T2 - Analytical Chemistry
VL - 82
IS - 5
SP - 2119
EP - 2123
DO - 10.1021/ac100060g
UR - https://www.scopus.com/inward/record.uri?eid=2-s2.0-77749252883&doi=10.1021%2fac100060g&partnerID=40&md5=6e721aedc4a9423f47f400bbef03c18c
AD - Department of Electronics and Electrical Engineering, University of Glasgow, Rankine Building, Glasgow G12 8LT, Oakfield Avenue, United Kingdom
AD - Department of Geology and Petroleum Geology, University of Aberdeen, Aberdeen AB24 3UE, United Kingdom
AB - We demonstrate the enhanced analytical sensitivity of both surface enhanced Raman scattering (SERS) and surface enhanced resonance Raman scattering (SERRS) responses, resulting from the in situ synthesis of silver colloid in a microfluidic flow structure, where both mixing and optical interrogation were integrated on-chip. The chip-based sensor was characterized with a model Raman active label, rhodamine-6G (R6G), and had a limit of detection (LOD) of ca. 50 fM (equivalent to single molecule detection). The device was also used for the determination of the natural pigment, scytonemin, from cyanobacteria (as an analogue for extraterrestrial life existing in extreme environments). The observed LOD of ~10 pM (ca. <400 molecules) demonstrated the analytical advantages of working with

freshly synthesized colloid in such a flow system. In both cases, sensitivities were between 1 and 2 orders of magnitude greater in the microfluidic system than those measured using the same experimental parameters, with colloid synthesized off-chip, under quiescent conditions. © 2010 American Chemical Society.
KW - Colloids
KW - Limit of Detection
KW - Microfluidics
KW - Spectrum Analysis, Raman
KW - Colloid chemistry
KW - Microfluidics
KW - Resonance
KW - Scattering
KW - Silver
KW - Synthesis (chemical)
KW - Active labels
KW - Colloidal synthesis
KW - Experimental parameters
KW - Extraterrestrial life
KW - Extreme environment
KW - Flow systems
KW - In-situ
KW - In-situ synthesis
KW - Limit of detection
KW - Micro fluidic system
KW - Microfluidic flow
KW - Natural pigment
KW - Off-chip
KW - On chips
KW - Optical interrogation
KW - Orders of magnitude
KW - Quiescent conditions
KW - Rhodamine 6G
KW - Signal enhancement
KW - Silver colloid
KW - Single-molecule detection
KW - Surface-enhanced Raman scattering
KW - Surface-enhanced resonance Raman scattering
KW - article
KW - colloid
KW - methodology
KW - microfluidics
KW - Raman spectrometry
KW - statistical parameters
KW - synthesis
KW - Raman scattering
SN - 00032700 (ISSN)
C2 - 20121214
LA - English
J2 - Anal. Chem.
M3 - Article
DB - Scopus
N1 - Export Date: 23 February 2023; Cited By: 64; Correspondence Address: J. Parnell; Department of Geology and Petroleum Geology,

University of Aberdeen, Aberdeen AB24 3UE, United Kingdom; email: j.parnell@abdn.ac.uk; CODEN: ANCHA
ER -

TY - JOUR
AU - Stalport, F.
AU - Yong Guan, Y.
AU - Audrey, N.
AU - Coll, P.
AU - Szopa, C.
AU - MacAri, F.
AU - Person, A.
AU - Chaput, D.
AU - Raulin, F.
AU - Cottin, H.
TI - UVolution, a photochemistry experiment in low earth orbit: Investigation of the photostability of carbonates exposed to martian-like UV radiation conditions
PY - 2010
T2 - Planetary and Space Science
VL - 58
IS - 12
SP - 1617
EP - 1624
DO - 10.1016/j.pss.2010.08.005
UR - https://www.scopus.com/inward/record.uri?eid=2-s2.0-77956610410&doi=10.1016%2fj.pss.2010.08.005&partnerID=40&md5=adbcdf785ff198b22ac7debed674efd9
AD - Laboratoire Interuniversitaire des Systmes Atmosphriques (LISA), UMR 7583 CNRS, Universities of Paris 7, 94010 Crteil Cedex, 61 Avenue du Gnral de Gaulle, France
AD - NASA, Goddard Space Flight Center (GSFC), Code 699, Greenbelt, MD 20771, 8800 Greenbelt Rd., United States
AD - UPMC Univ. Paris 06, Universit Versailles St-Quentin; CNRS/INSU, LATMOS-IPSL, 75005 Paris Cedex, 4 place Jussieu, France
AD - Laboratoire de Biominralisations et Paloenvironnements, University Pierre et Marie Curie-Paris VI, Paris, France
AD - Centre National d'Etudes Spatiales, Belin 31401 Toulouse Cedex 9, 18 avenue Edouard, France
AB - The detection and identification of carbonates on Mars are of prime importance to establish the evolution of its atmosphere, correlated to the history of the liquid water, or even to determine the existence of a possible ancient biological activity. Till date, no large deposits of carbonates have been found. In fact, their detection is specific to local areas and in very low amounts. The absence of such deposits is commonly attributed to the harsh environmental conditions at the surface of Mars. Additionally, the presence of UV radiation has been proposed to explain their photodecomposition and hence their absence. However, contradictory results from laboratory experiments mimicking Mars' surface UV radiation did not resolve the behaviour of carbonates in such an environment, which is why we exposed, in low Earth orbit and in laboratory experiments, both abiotic and biotic calcium carbonates to UV radiation of wavelength above 200 nm, the same spectral

distribution as the one reaching the surface of Mars. For low Earth orbit (LEO) exposure, this was done for the UVolution experiment on board the BIOPAN ESA module, which was set outside a Russian Foton automated capsule, and exposed to space conditions for 12 days in September 2007. The targeted carbonates are biominerals and abiotic samples. Our laboratory results mainly show that the exposed carbonates appear to be stable to UV radiation if directly exposed to it. The LEO experiment results tend to the same conclusion, but the integrated exposition time to Solar UV during the experiment is not sufficient to be conclusive. However, the stability of the biominerals derived from the laboratory experiment could strengthen the interest to explore deeper their potential as life records at Mars. Hence, they should be considered as primary targets for in situ analyses during future missions. © 2010 Elsevier Ltd. All rights reserved.
KW - Calcium
KW - Calcium carbonate
KW - Communication satellites
KW - Deposits
KW - Earth (planet)
KW - Laboratories
KW - Orbits
KW - Radiation
KW - Ultraviolet radiation
KW - Biological activities
KW - Biominerals
KW - Environmental conditions
KW - Future mission
KW - In-situ analysis
KW - Laboratory experiments
KW - Liquid water
KW - Low earth orbits
KW - Low-Earth orbit
KW - Mars
KW - Photo-decomposition
KW - Photo-stability
KW - Space conditions
KW - Spectral distribution
KW - Surface of Mars
KW - UV radiation
KW - Experiments
KW - Astrobiology
KW - Biominerals
KW - Carbonates
KW - Low Earth orbit
KW - Mars
KW - UV radiation
SN - 00320633 (ISSN)
LA - English
J2 - Planet. Space Sci.
M3 - Article
DB - Scopus
N1 - Export Date: 23 February 2023; Cited By: 9; Correspondence Address: F. Stalport; NASA, Goddard Space Flight Center (GSFC), Code

699, Greenbelt, MD 20771, 8800 Greenbelt Rd., United States; email: Fabien.Stalport@lisa.univ-paris12.fr; CODEN: PLSSA
ER -

TY - JOUR
AU - Sugitani, K.
AU - Lepot, K.
AU - Nagaoka, T.
AU - Mimura, K.
AU - Van Kranendonk, M.
AU - Oehler, D.Z.
AU - Walter, M.R.
TI - Biogenicity of morphologically diverse carbonaceous microstructures from the ca. 3400 Ma strelley pool formation, in the pilbara craton, Western Australia
PY - 2010
T2 - Astrobiology
VL - 10
IS - 9
SP - 899
EP - 920
DO - 10.1089/ast.2010.0513
UR - https://www.scopus.com/inward/record.uri?eid=2-s2.0-78650042646&doi=10.1089%2fast.2010.0513&partnerID=40&md5=d7601697010a7fd7cd47bf90d3a22c00
AD - Department of Environmental Engineering and Architecture, Graduate School of Environmental Studies, Nagoya University, Nagoya, Japan
AD - Department of Geology, University of Liège, Liège, Belgium
AD - School of Informatics and Sciences, Nagoya University, Nagoya, Japan
AD - Department of Earth and Environmental Sciences, Graduate School of Environmental Studies, Nagoya University, Nagoya, Japan
AD - Geological Survey of Western Australia, Department of Mines and Petroleum, East Perth, Australia
AD - Australian Centre for Astrobiology, University of New South Wales, Sydney, Australia
AD - Astromaterials Research and Exploration Science, NASA Johnson Space Center, Houston, TX, United States
AB - Morphologically diverse structures that may constitute organic microfossils are reported from three remote and widely separated localities assigned to the ca. 3400 Ma Strelley Pool Formation in the Pilbara Craton, Western Australia. These localities include the Panorama, Warralong, and Goldsworthy greenstone belts. From the Panorama greenstone belt, large (>40μm) lenticular to spindle-like structures, spheroidal structures, and mat-forming thread-like structures are found. Similar assemblages of carbonaceous structures have been identified from the Warralong and Goldsworthy greenstone belts, though these assemblages lack the thread-like structures but contain film-like structures. All structures are syngenetic with their host sedimentary black chert, which is associated with stromatolites and evaporites. The host chert is considered to have been deposited in a shallow water environment. Rigorous assessment of biogenicity (considering composition, size range, abundance,

taphonomic features, and spatial distributions) suggests that cluster-forming small (<15 μm) spheroids, lenticular to spindle-like structures, and film-like structures with small spheroids are probable microfossils. Thread-like structures are more likely fossilized fibrils of biofilm, rather than microfossils. The biogenicity of solitary large (>15 μm) spheroids and simple film-like structures is less certain. Although further investigations are required to confirm the biogenicity of carbonaceous structures from the Strelley Pool Formation, this study presents evidence for the existence of morphologically complex and large microfossils at 3400 Ma in the Pilbara Craton, which can be correlated to the contemporaneous, possible microfossils reported from South Africa. Although there is still much to be learned, they should provide us with new insights into the early evolution of life and shallow water ecosystems. Key Words: Archean-Biogenicity-Microfossils-Pilbara. Astrobiology 10, 899-920. © Mary Ann Liebert, Inc.
KW - Biogenesis
KW - Biological Evolution
KW - Ecosystem
KW - Fossils
KW - Geologic Sediments
KW - Meteoroids
KW - South Africa
KW - Western Australia
KW - article
KW - astronomy
KW - Australia
KW - biogenesis
KW - chemistry
KW - ecosystem
KW - evolution
KW - fossil
KW - sediment
KW - South Africa
SN - 15311074 (ISSN)
C2 - 21118023
LA - English
J2 - Astrobiology
M3 - Article
DB - Scopus
N1 - Export Date: 23 February 2023; Cited By: 75; Correspondence Address: K. Sugitani; Department of Environmental Engineering and Architecture, Graduate School of Environmental Studies, Nagoya University, Nagoya 464-8601, Japan; email: sugi@info.human.nagoya-u.ac.jp
ER -

TY - JOUR
AU - Kaltenegger, L.
AU - Henning, W.G.
AU - Sasselov, D.D.
TI - Detecting volcanism on extrasolar planets
PY - 2010
T2 - Astronomical Journal

VL - 140
IS - 5
SP - 1370
EP - 1380
DO - 10.1088/0004-6256/140/5/1370
UR - https://www.scopus.com/inward/record.uri?eid=2-s2.0-78049364077&doi=10.1088%2f0004-6256%2f140%2f5%2f1370&partnerID=40&md5=17b9acd09c9cded45f3d7b1980433227
AD - Harvard University, Cambridge, MA 02138, 60 Garden Street, United States
AD - Harvard University, Cambridge, MA 02138, EPS, 20 Oxford Street, United States
AB - The search for extrasolar rocky planets has already found the first transiting rocky super-Earth, Corot 7b, with a surface temperature that allows for magma oceans. Here, we investigate whether we could distinguish rocky planets with recent major volcanism by remote observation. We develop a model for volcanic eruptions on an Earth-like exoplanet based on the present-day Earth and derive the observable features in emergent and transmission spectra for multiple scenarios of gas distribution and cloud cover. We calculate the observation time needed to detect explosive volcanism on exoplanets in primary as well as secondary eclipse and discuss the likelihood of observing volcanism on transiting Earth-sized to super-Earth-sized exoplanets. We find that sulfur dioxide from large explosive eruptions does present a spectral signal that is remotely detectable especially for secondary eclipse measurements around the closest stars and ground-based telescopes, and report the frequency and magnitude of the expected signatures. The transit probability of a planet in the habitable zone decreases with distance from the host star, making small, nearby host stars the best targets. © 2010. The American Astronomical Society. All rights reserved. Printed in the U.S.A.
KW - Astrobiology
KW - Earth
KW - Methods: Observational
KW - Planets and satellites: Atmospheres
KW - Planets and satellites: Detection
KW - Planets and satellites: tectonics
KW - Techniques: spectroscopic
SN - 00046256 (ISSN)
LA - English
J2 - Astron. J.
M3 - Article
DB - Scopus
N1 - Export Date: 23 February 2023; Cited By: 46; Correspondence Address: L. Kaltenegger; Harvard University, Cambridge, MA 02138, 60 Garden Street, United States; email: lkaltene@cfa.harvard.edu
ER -

TY - JOUR
AU - Fairén, A.G.
TI - A cold and wet Mars
PY - 2010
T2 - Icarus

VL - 208
IS - 1
SP - 165
EP - 175
DO - 10.1016/j.icarus.2010.01.006
UR - https://www.scopus.com/inward/record.uri?eid=2-s2.0-77953537211&doi=10.1016%2fj.icarus.2010.01.006&partnerID=40&md5=e023c1e6a1f499c9f90520b3dab25be7
AD - NASA Ames Research Center, Space Science and Astrobiology Division, Moffett Field, 94035 CA, United States
AB - Water on Mars has been explained by invoking controversial and mutually exclusive solutions based on warming the atmosphere with greenhouse gases (the " warm and wet" Mars) or on local thermal energy sources acting in a global freezing climate (the " cold and dry" Mars). Both have critical limitations and none has been definitively accepted as a compelling explanation for the presence of liquid water on Mars. Here is considered the hypothesis that cold, saline and acidic liquid solutions have been stable on the sub-zero surface of Mars for relatively extended periods of time, completing a hydrogeological cycle in a water-enriched but cold planet. Computer simulations have been developed to analyze the evaporation processes of a hypothetical martian fluid with a composition resulting from the acid weathering of basalt. This model is based on orbiter- and lander-observed surface mineralogy of Mars, and is consistent with the sequence and time of deposition of the different mineralogical units. The hydrological cycle would have been active only in periods of dense atmosphere, as having a minimum atmospheric pressure is essential for water to flow, and relatively high temperatures (over ~245. K) are required to trigger evaporation and snowfall; minor episodes of limited liquid water on the surface could have occurred at lower temperatures (over ~225. K). During times with a thin atmosphere and even lesser temperatures (under ~225. K), only transient liquid water can potentially exist on most of the martian surface. Assuming that surface temperatures have always been maintained below 273. K, Mars can be considered a " cold and wet" planet for a substantial part of its geological history. © 2010 Elsevier Inc.
KW - Astrobiology
KW - Geological processes
KW - Mars, Climate
KW - Mars, Surface
SN - 10902643 (ISSN)
LA - English
J2 - Icarus
M3 - Article
DB - Scopus
N1 - Export Date: 23 February 2023; Cited By: 115; Correspondence Address: A.G. Fairén; NASA Ames Research Center, Space Science and Astrobiology Division, Moffett Field, 94035 CA, United States; email: alberto.g.fairen@nasa.gov; CODEN: ICRSA
ER -

TY - JOUR
AU - Foster, I.S.

AU - King, P.L.
AU - Hyde, B.C.
AU - Southam, G.
TI - Characterization of halophiles in natural MgSO4 salts and laboratory enrichment samples: Astrobiological implications for Mars
PY - 2010
T2 - Planetary and Space Science
VL - 58
IS - 4
SP - 599
EP - 615
DO - 10.1016/j.pss.2009.08.009
UR - https://www.scopus.com/inward/record.uri?eid=2-s2.0-76349097575&doi=10.1016%2fj.pss.2009.08.009&partnerID=40&md5=2a9c3620888799992520a0c7f43190cb
AD - Department of Earth Sciences, The University of Western Ontario, London, Ont. N6A 5B7, Canada
AD - Institute of Meteoritics, University of New Mexico, Albuquerque, NM 87131, United States
AB - The presence of sulfate salts and limited subsurface water (ice) on Mars suggests that any liquid water on Mars today will occur as (magnesium) sulfate-rich brines in regions containing sources of magnesium and sulfur. The Basque Lakes of British Columbia, Canada, represent a hypersaline terrestrial analogue site, which possesses chemical and physical properties similar to those observed on Mars. The Basque Lakes also contain diverse halophilic organisms representing all three Kingdoms of life, growing in surface and near-subsurface environments. Of interest from an astrobiological perspective, crushed magnesium sulfate samples that were analyzed using a modified Lowry protein assay contained biomass in every crystal inspected, with biomass values from 0.078 to 4.21 mgbiomass/gsalt; average=0.74±0.7 mgbiomass/gsalt. Bacteria and Archaea cells were easily observed even in low-biomass samples using light microscopy, and bacteria trapped within magnesium sulfate crystals were observed using confocal microscopy. Regions within the salt also contained bacterial pigments, e.g., carotenoids, which were separate from the cells, indicating that cell lysis might have occurred during entrapment within the salt matrix. These biosignatures, cells, and any 'soluble' organic constituents were primarily found trapped within fluid inclusions or fluid-filled void spaces between intergrown crystals. Diffuse reflectance infrared Fourier transform spectroscopy (reflectance IR) analysis of enrichment cultures, containing cyanobacteria, Archaea, or dissimilatory sulfate-reducing bacteria, highlighted molecular biosignature features between 550-1650 and 2400-3000 cm-1. Spectra from natural salts demonstrated that we can detect biomass within salt crystals using the most sensitive biosignatures, which are the 1530-1570 cm-1, C-N, N-H, -COOH absorptions and the 1030-1050 cm-1 C-OH, C-N, PO43- bond features. The lowest detection limit for a biosignature absorption feature using reflectance IR was with a natural sample that possessed 0.78 mgbiomass/gsalt. In a model cell, i.e., a 0.5 by 1 μm bacillus, this biomass value corresponds to approximately 7.8×108 cells/gsalt. Based on its ability to detect biomass entrapped within natural sulfate salts, reflectance IR may

make an effective remote-sensing tool for finding enrichments of
organic carbon within outcrops and surficial sedimentary deposits on
Mars. © 2009 Elsevier Ltd. All rights reserved.
KW - Bacteriology
KW - Cell culture
KW - Confocal microscopy
KW - Crystals
KW - Fourier transform infrared spectroscopy
KW - Lakes
KW - Magnesium
KW - Mineralogy
KW - Organic carbon
KW - Reflection
KW - Remote sensing
KW - Salts
KW - Spectroscopy
KW - Sulfur
KW - Archaea
KW - Biomass samples
KW - Biosignatures
KW - British Columbia , Canada
KW - Cell lysis
KW - Detection limits
KW - Diffuse reflectance infrared Fourier transform spectroscopies
KW - Enrichment culture
KW - Fluid inclusion
KW - Fluid-filled
KW - Halophiles
KW - Hypersaline
KW - Light microscopy
KW - Liquid water
KW - Magnesium sulfate
KW - Organic constituents
KW - Protein assays
KW - Salt crystals
KW - Salt matrices
KW - Sedimentary deposits
KW - Sensing tools
KW - Subsurface environment
KW - Subsurface waters
KW - Sulfate reducing bacteria
KW - Sulfate salts
KW - Void space
KW - Biomass
KW - Confocal microscopy
KW - Halophiles
KW - Infrared spectroscopy
KW - Mars
KW - Sulfates
SN - 00320633 (ISSN)
LA - English
J2 - Planet. Space Sci.
M3 - Article
DB - Scopus

N1 - Export Date: 23 February 2023; Cited By: 30; Correspondence Address: I.S. Foster; Department of Earth Sciences, The University of Western Ontario, London, Ont. N6A 5B7, Canada; email: Ian.S.Foster@live.com; CODEN: PLSSA
ER -

TY - JOUR
AU - Wacey, D.
TI - Stromatolites in the ~3400 Ma strelley pool formation, Western Australia: Examining biogenicity from the macro- to the nano-scale
PY - 2010
T2 - Astrobiology
VL - 10
IS - 4
SP - 381
EP - 395
DO - 10.1089/ast.2009.0423
UR - https://www.scopus.com/inward/record.uri?eid=2-s2.0-77953606316&doi=10.1089%2fast.2009.0423&partnerID=40&md5=1f75c0ad99dc58e01d92c8900f1e088d
AD - Centre for Microscopy, Characterisation and Analysis, School of Earth and Environment, University of Western Australia, Crawley, Australia
AB - The 3426-3350 Ma Strelley Pool Formation (SPF) is a silicified, dominantly sedimentary unit within the Pilbara Supergroup, Western Australia. It is found widely across the East Pilbara Terrane, and it forms a prominent marker horizon and separates the largely volcanic 3520-3427 Ma Warrawoona and 3350-3315 Ma Kelly groups. It has become one of the key formations for study by astrobiologists, following reports of some of the world's oldest stromatolites. Abundant contextural and morphological evidence has been presented over the last decade in support of a biological role in SPF stromatolite formation. This evidence is reviewed here, and additional data are presented from recent fieldwork carried out across the ~25 km of SPF outcrops in the East Strelley greenstone belt of the East Pilbara Terrane. In addition to contextural and morphological evidence, a compelling claim for early life requires geochemical evidence for biological cycling. A potential avenue of approach to obtain such evidence for the SPF stromatolites (and other ancient examples) is discussed in the context of a pilot study in which nano-scale secondary ion mass spectrometry (NanoSIMS) was used. © Mary Ann Liebert, Inc.
KW - Biogenesis
KW - Geologic Sediments
KW - Nanostructures
KW - Spectrometry, Mass, Secondary Ion
KW - Time Factors
KW - Western Australia
KW - nanomaterial
KW - article
KW - Australia
KW - biogenesis
KW - chemistry
KW - mass spectrometry

KW - sediment
KW - time
KW - Archean
KW - Biogenicity
KW - NanoSIMS
KW - Strelley Pool Formation
KW - Stromatolites
SN - 15311074 (ISSN)
C2 - 20528193
LA - English
J2 - Astrobiology
M3 - Article
DB - Scopus
N1 - Export Date: 23 February 2023; Cited By: 53; Correspondence Address: D. Wacey; Centre for Microscopy, Characterisation and Analysis (M010), University of Western Australia, Crawley, WA 6009, Australia; email: David.Wacey@uwa.edu.au
ER -

TY - JOUR
AU - Court, R.W.
AU - Baki, A.O.
AU - Sims, M.R.
AU - Cullen, D.
AU - Sephton, M.A.
TI - Novel solvent systems for in situ extraterrestrial sample analysis
PY - 2010
T2 - Planetary and Space Science
VL - 58
IS - 11
SP - 1470
EP - 1474
DO - 10.1016/j.pss.2010.07.004
UR - https://www.scopus.com/inward/record.uri?eid=2-s2.0-77955558452&doi=10.1016%2fj.pss.2010.07.004&partnerID=40&md5=3b041540c8be195574bab91eb188cf80
AD - Department of Earth Science and Engineering, South Kensington Campus, Imperial College London, London SW7 2AZ, United Kingdom
AD - Department of Physics and Astronomy, Michael Atiyah Building, University of Leicester, Leicester LE1 7RH, United Kingdom
AD - Cranfield Health, Cranfield University, Silsoe, Bedfordshire MK45 4DT, United Kingdom
AB - The life marker chip (LMC) is being designed to test for the chemical signature of life in the soil and rocks of Mars. It will use an antibody array as part of its detection and characterisation system and aims to detect both polar and non-polar molecules at the sub-ppm to tens of ppb level. It is necessary to use a solvent to transfer organic compounds from the Martian samples to the LMC itself, but organic solvents such as dichloromethane or hexane, commonly used to dissolve non-polar molecules, are incompatible with the LMC antibodies. Hence, an aqueous-based solvent capable of dissolving the biomarkers that might exist in the soil or rocks of Mars is required. Solvent extractions of a Martian soil analogue,

JSC Mars-1, spiked with a range of standards show that a 20:80 (vol:vol) mixture of methanol and water is incapable of extracting compounds insoluble in water. However, addition of 1.5 mg ml -1 of the surfactant polysorbate 80 produces extraction efficiencies of the aliphatic standards, hexadecane and phytane, equal to 2530% of those produced by the common organic solvent mixture 93:7 (vol:vol) dichloromethane:methanol. Extraction of squalene and stigmasterol using the polysorbate solution is less efficient but still successful, at 510% of the efficiency of 93:7 dichloromethane:methanol. Such aliphatic compounds with occasional functional groups represent the compound classes to which most fossil organic biomarkers belong. The polysorbate solution did not extract the aromatic compounds pyrene and anthracene with great efficiency. A solvent of 20:80 methanol:water with 1.5 mg ml-1 polysorbate 80 is therefore capable of selectively extracting aliphatic biomarkers from Martian samples and transferring them to the antibody sites on the life marker chip. © 2010 Elsevier B.V.. All rights reserved.
KW - Antibodies
KW - Chemical detection
KW - Dichloromethane
KW - Dissolution
KW - Functional groups
KW - Hexane
KW - Methanol
KW - Organic solvents
KW - Paraffins
KW - Soils
KW - Surface active agents
KW - Aliphatic compound
KW - Antibody arrays
KW - Characterisation
KW - Chemical signatures
KW - ExoMars
KW - Extracting compounds
KW - Extraction efficiencies
KW - Extraterrestrial life
KW - Hexadecane
KW - In-situ
KW - Martian soils
KW - Nonpolar molecules
KW - Novel solvents
KW - Polysorbate 80
KW - Polysorbates
KW - Ppb levels
KW - Sample analysis
KW - Solvent extraction
KW - Astrobiology
KW - ExoMars
KW - Extraterrestrial life
KW - Mars
SN - 00320633 (ISSN)
LA - English
J2 - Planet. Space Sci.

M3 - Article
DB - Scopus
N1 - Export Date: 23 February 2023; Cited By: 16; Correspondence Address: R. W. Court; Department of Earth Science and Engineering, South Kensington Campus, Imperial College London, London SW7 2AZ, United Kingdom; email: r.court@imperial.ac.uk; CODEN: PLSSA
ER -

TY - JOUR
AU - Crawford, I.A.
TI - Introduction to the Special Issue on Astrobiology on the Moon
PY - 2010
T2 - Earth, Moon and Planets
VL - 107
IS - 1
SP - 1
DO - 10.1007/s11038-010-9373-0
UR - https://www.scopus.com/inward/record.uri?eid=2-s2.0-78649326209&doi=10.1007%2fs11038-010-9373-0&partnerID=40&md5=59641eb52a55aa0ad5defe5548f608bb
AD - Department of Earth and Planetary Sciences, Birkbeck College London, London WC1E 7HX, United Kingdom
KW - exobiology
KW - exploration
KW - lunar soil
KW - Moon
KW - satellite mission
SN - 01679295 (ISSN)
LA - English
J2 - Earth Moon Planets
M3 - Article
DB - Scopus
N1 - Export Date: 23 February 2023; Cited By: 2; Correspondence Address: I. A. Crawford; Department of Earth and Planetary Sciences, Birkbeck College London, London WC1E 7HX, United Kingdom; email: i.crawford@ucl.ac.uk
ER -

TY - BOOK
AU - Hegedű, S.
AU - Csonka, J.
TI - Astrobiology: Physical origin, biological evolution and spatial distribution
PY - 2010
T2 - Astrobiology: Physical Origin, Biological Evolution and Spatial Distribution
SP - 1
EP - 219
UR - https://www.scopus.com/inward/record.uri?eid=2-s2.0-85030212212&partnerID=40&md5=e1e08a631de95653f135f72f963d3207
AB - Astrobiology is the study of the origin, evolution, distribution, and future of life in the universe. This book offers an analysis undertaken from the perspective of science education, to discuss the nature of the particular challenges faced by those

seeking to facilitate public education in the field of astrobiology. The authors also address the question of the formation of a protocell and its function at the system level. It will further deal with the environmental pressures likely present on the early Earth, as they would have influenced the lifetime of unstable or reactive components needed for the start of life. In addition, the method of the transition from prebiotic organic microsystems to the primordial communities of prokaryotes is explored. The authors also explain some aspects related to aqueous interfacial processes in the context of prebiotic chemistry under the point of view of our current experimental results. Moreover, there is considerable interest in astrobiology in investigating icy worlds and the microbial forms that thrive in extreme environments, especially under those cold conditions that can provide a model for some extraterrestrial environments. This book reviews the general trends concerning the biodiversity and ecology of psychrophilic microorganisms and their molecular mechanisms of adaptation and evolution, as well as summarize the latest results on this topic in samples from icy environments. Other chapters summarize the morphological signatures of flowing water, the astrobiological potential of the dunes, and the theoretical modelling background of the presence of liquid water on Mars today. © 2010 by Nova Science Publishers, Inc. All rights reserved.
PB - Nova Science Publishers, Inc.
SN - 978-161761955-7 (ISBN); 978-160741290-8 (ISBN)
LA - English
J2 - Astrobiology: Physical Origin, Biological Evolution and Spatial Distribution
M3 - Book
DB - Scopus
N1 - Export Date: 23 February 2023; Cited By: 0
ER -

TY - JOUR
AU - Mecham, M.
TI - Institute seeks world-class lunar research
PY - 2010
T2 - Aviation Week and Space Technology (New York)
VL - 172
IS - 32
UR - https://www.scopus.com/inward/record.uri?eid=2-s2.0-84903426913&partnerID=40&md5=2d8822967078f6d98ca38497022e4b3f
AB - NASA's Lunar Science Institute (NLSI) at the Ames Research Center has adopted the same interdisciplinary approach that the center's Astrobiology Institute has been pursuing for years. Seven teams averaging 15 members each are at work under four-year grants studying everything that includes Moon's formative years and its dust. The amount, $2 million, has been spent at Ames from the NLSI's $13-million annual budget. NLSI broadly defines lunar science in three ways that involves the investigations of moon, which includes geology and history that might not be conducted specifically on the lunar surface, but include studying the natural satellite for insights into the evolution of the solar system. Another definition, which involves investigations on the moon, specifically looks at the

effects of the lunar environment on terrestrial life and the third area involves investigations from the moon that see the lunar surface as a platform for scientific investigations, which include observations of the Earth and other celestial phenomena.
PB - McGraw-Hill Companies
SN - 00052175 (ISSN)
LA - English
J2 - Aviat. Week Space Technol. (New York)
M3 - Short survey
DB - Scopus
N1 - Export Date: 23 February 2023; Cited By: 0; CODEN: AWSTA
ER -

TY - JOUR
AU - Kolb, V.M.
TI - On the applicability of the green chemistry principles to sustainability of organic matter on asteroids
PY - 2010
T2 - Sustainability
VL - 2
IS - 6
SP - 1624
EP - 1631
DO - 10.3390/su2061624
UR - https://www.scopus.com/inward/record.uri?eid=2-s2.0-78649397086&doi=10.3390%2fsu2061624&partnerID=40&md5=f4659889cb36430fe68ff2860927e97f
AD - Department of Chemistry, University of Wisconsin-Parkside, Kenosha, WI 53141-2000, United States
AB - The connection between astrobiology and green chemistry represents a new approach to sustainability of organic matter on asteroids or similar bodies. Green chemistry is chemistry which is environmentally friendly. One obvious way for chemistry to be green is to use water as a solvent, instead of more toxic organic solvents. Many astrobiological reactions occur in the aqueous medium, for example in the prebiotic soup or during the aqueous alteration period on asteroids. Thus any advances in the green organic reactions in water are directly applicable to astrobiology. Another green chemistry approach is to abolish use of toxic solvents. This can be accomplished by carrying out the reactions without a solvent in the solventless or solid-state reactions. The advances in these green reactions are directly applicable to the chemistry on asteroids during the periods when water was not available. Many reactions on asteroids may have been done in the solid mixtures. These reactions may be responsible for a myriad of organic compounds that have been isolated from the meteorites. © 2010 by the authors.
KW - Alnus
KW - Asteroids
KW - Astrobiology
KW - Diels-Alder reactions in water
KW - Green chemistry
KW - Meteorites
KW - Solid-state reactions

KW - Solventless reactions
PB - MDPI
SN - 20711050 (ISSN)
LA - English
J2 - Sustainability
M3 - Article
DB - Scopus
N1 - Export Date: 23 February 2023; Cited By: 10; Correspondence Address: V.M. Kolb; Department of Chemistry, University of Wisconsin-Parkside, Kenosha, WI 53141-2000, United States; email: kolb@uwp.edu
ER -

TY - CHAP
AU - Wettlaufer, J.S.
TI - Sea Ice and Astrobiology
PY - 2010
T2 - Sea Ice: Second Edition
SP - 579
EP - 594
DO - 10.1002/9781444317145.ch15
UR - https://www.scopus.com/inward/record.uri?eid=2-s2.0-84870929896&doi=10.1002%2f9781444317145.ch15&partnerID=40&md5=76d2db65625af0d6cc2c1729afe0ec5d
AD - Yale University, New Haven, CT 06520-8109, United States
KW - Abiotic and biotic factors controlling EPS and AFP production in situ
KW - Controlled studies of biopolymer-ice interactions and grain boundary melting
KW - Equilibrium phase behaviour of ice
KW - Exopolymeric substances
KW - Known terrestrial habitats in terms of robust physical features
KW - Liquid water for persistence of all known forms of life
KW - Sea ice and astrobiology
KW - Search for past and present exobiological communities - frozen surface of Mars and icy moons of Jupiter
KW - Search for unintelligent1 life - that of a dowser in an enormous freezer
KW - Shear flow at ice-ocean interface influencing patterns
PB - Wiley-Blackwell
SN - 978-140518580-6 (ISBN)
LA - English
J2 - Sea Ice: Second Ed.
M3 - Book chapter
DB - Scopus
N1 - Export Date: 23 February 2023; Cited By: 5
ER -

TY - JOUR
AU - Ferl, R.J.
AU - Paul, A.-L.
TI - Lunar plant biology-a review of the apollo era
PY - 2010

T2 - Astrobiology
VL - 10
IS - 3
SP - 261
EP - 274
DO - 10.1089/ast.2009.0417
UR - https://www.scopus.com/inward/record.uri?eid=2-s2.0-77952512735&doi=10.1089%2fast.2009.0417&partnerID=40&md5=6cdde0885198273c1af3d11d4450131f
AD - University of Florida, Interdisciplinary Center for Biotechnology Research and the Horticultural Sciences Department, Gainesville, FL, United States
AB - Recent plans for human return to the Moon have significantly elevated scientific interest in the lunar environment with emphasis on the science to be done in preparation for the return and while on the lunar surface. Since the return to the Moon is envisioned as a dedicated and potentially longer-term commitment to lunar exploration, questions of the lunar environment and particularly its impact on biology and biological systems have become a significant part of the lunar science discussion. Plants are integral to the discussion of biology on the Moon. Plants are envisioned as important components of advanced habitats and fundamental components of advanced life-support systems. Moreover, plants are sophisticated multicellular eukaryotic life-forms with highly orchestrated developmental processes, well-characterized signal transduction pathways, and exceedingly fine-tuned responses to their environments. Therefore, plants represent key test organisms for understanding the biological impact of the lunar environment on terrestrial life-forms. Indeed, plants were among the initial and primary organisms that were exposed to returned lunar regolith from the Apollo lunar missions. This review discusses the original experiments involving plants in association with the Apollo samples, with the intent of understanding those studies within the context of the first lunar exploration program and drawing from those experiments the data to inform the studies critical within the next lunar exploration science agenda. Copyright 2010, Mary Ann Liebert, Inc.
KW - Exobiology
KW - Extraterrestrial Environment
KW - Moon
KW - Plants
KW - Space Flight
KW - Spacecraft
KW - Eukaryota
KW - biology
KW - growth, development and aging
KW - metabolism
KW - microclimate
KW - moon
KW - plant
KW - review
KW - space flight
KW - astrobiology
KW - metabolism

KW - plant
KW - plant development
KW - trends
KW - Apollo
KW - Lunar Receiving Laboratory
KW - Lunar regolith
KW - Plant biology
SN - 15311074 (ISSN)
C2 - 20446867
LA - English
J2 - Astrobiology
M3 - Review
DB - Scopus
N1 - Export Date: 23 February 2023; Cited By: 18; Correspondence Address: R. J. Ferl; University of Florida, Interdisciplinary Center for Biotechnology Research and the Horticultural Sciences Department, Gainesville, FL 3201-0690, PO Box 110690, United States; email: robferl@ufl.edu
ER -

TY - JOUR
AU - Williams, K.E.
AU - McKay, C.P.
AU - Toon, O.B.
AU - Head, J.W.
TI - Do ice caves exist on Mars?
PY - 2010
T2 - Icarus
VL - 209
IS - 2
SP - 358
EP - 368
DO - 10.1016/j.icarus.2010.03.039
UR - https://www.scopus.com/inward/record.uri?eid=2-s2.0-77956286390&doi=10.1016%2fj.icarus.2010.03.039&partnerID=40&md5=1ac3460b93cb598915873f36354aefb6
AD - NASA Ames Research Center, Division of Space Sciences and Astrobiology, Moffett Field, CA 94035, Mail Stop 245-3, United States
AD - Dept. of Atmospheric and Oceanic Sciences, Laboratory for Atmospheric and Space Physics, LASP UCB 392, University of Colorado, Boulder, CO 80309-0392, United States
AD - Dept. of Geological Sciences, Brown University, Providence, RI 02906, 324 Brook Street, Box 1846, United States
AB - We have developed a numerical model for assessing the lifetime of ice deposits in martian caves that are open to the atmosphere. Our model results and sensitivity tests indicate that cave ice would be stable over significant portions of the surface of Mars. Ice caves on Earth commonly occur in lava tubes, and Mars has been significantly resurfaced by volcanic activity during its history, including the two main volcanic provinces, the Tharsis and Elysium rises. These areas, known or suspected of having subsurface caves and related voids are among the most favorable regions for the occurrence of ice stability. The martian ice cave model predicts

regions which, if caves occur, would potentially be areas of astrobiological importance as well as possible water sources for future human missions to Mars. © 2010 Elsevier Inc.
KW - Ices
KW - Mars, Atmosphere
KW - Mars, Climate
KW - Mars, Surface
SN - 10902643 (ISSN)
LA - English
J2 - Icarus
M3 - Article
DB - Scopus
N1 - Export Date: 23 February 2023; Cited By: 40; Correspondence Address: K.E. Williams; NASA Ames Research Center, Division of Space Sciences and Astrobiology, Moffett Field, CA 94035, Mail Stop 245-3, United States; email: kaj.williams@gmail.com; CODEN: ICRSA
ER -

TY - JOUR
AU - Ulrich, R.
AU - Kral, T.
AU - Chevrier, V.
AU - Pilgrim, R.
AU - Roe, L.
TI - Dynamic temperature fields under mars landing sites and implications for supporting microbial life
PY - 2010
T2 - Astrobiology
VL - 10
IS - 6
SP - 643
EP - 650
DO - 10.1089/ast.2010.0472
UR - https://www.scopus.com/inward/record.uri?eid=2-s2.0-77956134667&doi=10.1089%2fast.2010.0472&partnerID=40&md5=b2cfcd5820415daaeb77e0c1401ff625
AD - Department of Chemical Engineering, Bell 3202, University of Arkansas, Fayetteville, AR 72701, United States
AD - Department of Biological Sciences, University of Arkansas, Fayetteville, AR, United States
AD - Arkansas Center for Space and Planetary Sciences, University of Arkansas, Fayetteville, AR, United States
AD - Department of Mechanical Engineering, University of Arkansas, Fayetteville, AR, United States
AB - While average temperatures on Mars may be too low to support terrestrial life-forms or aqueous liquids, diurnal peak temperatures over most of the planet can be high enough to provide for both, down to a few centimeters beneath the surface for some fraction of the time. A thermal model was applied to the Viking 1, Viking 2, Pathfinder, Spirit, and Opportunity landing sites to demonstrate the dynamic temperature fields under the surface at these well-characterized locations. A benchmark temperature of 253 K was used as a lower limit for possible metabolic activity, which corresponds to the minimum found for specific terrestrial microorganisms.

Aqueous solutions of salts known to exist on Mars can provide liquid solutions well below this temperature. Thermal modeling has shown that 253 K is reached beneath the surface at diurnal peak heating for at least some parts of the year at each of these landing sites. Within 40° of the equator, 253 K beneath the surface should occur for at least some fraction of the year; and, within 20°, it will be seen for most of the year. However, any life-form that requires this temperature to thrive must also endure daily excursions to far colder temperatures as well as periods of the year where 253 K is never reached at all. © Copyright 2010, Mary Ann Liebert, Inc. 2010.
KW - Exobiology
KW - Extraterrestrial Environment
KW - Mars
KW - Microbial Viability
KW - Temperature
KW - Water
KW - water
KW - article
KW - astrobiology
KW - astronomy
KW - chemistry
KW - microbial viability
KW - microclimate
KW - temperature
KW - Brine
KW - Lander
KW - Liquid
KW - Mars
KW - Temperature
SN - 15311074 (ISSN)
C2 - 20735254
LA - English
J2 - Astrobiology
M3 - Article
DB - Scopus
N1 - Export Date: 23 February 2023; Cited By: 14; Correspondence Address: R. Ulrich; Department of Chemical Engineering, Bell 3202, University of Arkansas, Fayetteville, AR 72701, United States; email: rulrich@uark.edu
ER -

TY - CONF
AU - Gibson, C.H.
AU - Wickramasinghe, N.C.
AU - Schild, R.E.
TI - Primordial planets, comets, and moons foster life in the cosmos
PY - 2010
T2 - Proceedings of SPIE - The International Society for Optical Engineering
VL - 7819
C7 - 781914
DO - 10.1117/12.864734
UR - https://www.scopus.com/inward/record.uri?eid=2-

s2.0-78649404778&doi=10.1117%2f12.864734&partnerID=40&md5=9419c2a013ff7a10030bd8ca06aba7bf
AD - UCSD, San Diego, CA 92093-0411, United States
AD - Cardiff Univ., Cardiff, United Kingdom
AB - A key result of hydrogravitational dynamics cosmology relevant to astrobiology is the early formation of vast numbers of hot primordial-gas planets in million-solar-mass clumps as the dark matter of galaxies and the hosts of first life. Photon viscous forces in the expanding universe of the turbulent big bang prevent fragmentations of the plasma for mass scales smaller than protogalaxies. At the plasma to gas transition 300,000 years after the big bang, the 107 decrease in kinematic viscosity ν explains why ~3×107 planets are observed to exist per star in typical galaxies like the Milky Way, not eight or nine. Stars form by a binary accretional cascade from Earth-mass primordial planets to progressively larger masses that collect and recycle the stardust chemicals of life produced when stars overeat and explode. The astonishing complexity of molecular biology observed on Earth is possible to explain only if enormous numbers of primordial planets and their fragments have hosted the formation and wide scattering of the seeds of life virtually from the beginning of time. Geochemical and biological evidence suggests that life on Earth appears at the earliest moment it can survive, in highly evolved forms with complexity requiring a time scale in excess of the age of the galaxy. This is quite impossible within standard cold-dark-matter cosmology where planets are relatively recent, rare and cold, completely lacking mechanisms for intergalactic transport of life forms. © 2010 Copyright SPIE - The International Society for Optical Engineering.
KW - Cosmology
KW - Molecular biology
KW - Stars
KW - Big Bang
KW - Dark matter
KW - Kinematic viscosity
KW - Life form
KW - Mass scale
KW - Milky ways
KW - Planet formation
KW - Protogalaxies
KW - star formation
KW - Time-scales
KW - Viscous forces
KW - Planets
KW - astrobiology
KW - Cosmology
KW - planet formation
KW - star formation
SN - 0277786X (ISSN); 978-081948315-7 (ISBN)
LA - English
J2 - Proc SPIE Int Soc Opt Eng
M3 - Conference paper
DB - Scopus
N1 - Export Date: 23 February 2023; Cited By: 2; Correspondence

Address: C. H. Gibson; Depts. of MAE, SIO, CASS, United States; email: cgibson@ucsd.edu; CODEN: PSISD
ER -

TY - JOUR
AU - Jorge-Villar, S.E.
AU - Edwards, H.G.M.
TI - Raman spectroscopy of volcanic lavas and inclusions of relevance to astrobiological exploration
PY - 2010
T2 - Philosophical Transactions of the Royal Society A: Mathematical, Physical and Engineering Sciences
VL - 368
IS - 1922
SP - 3127
EP - 3135
DO - 10.1098/rsta.2010.0102
UR - https://www.scopus.com/inward/record.uri?eid=2-s2.0-77955189563&doi=10.1098%2frsta.2010.0102&partnerID=40&md5=7e3e71cfb457357a559d1d32a98c51e2
AD - Area Geodinamica Interna, Facultad de Humanidades y Educacion, Universidad de Burgos, 09001 Burgos, Calle Villadiego s/n, Spain
AD - Centre for Astrobiology and Extremophiles Research, School of Life Sciences, University of Bradford, Bradford BD7 1DP, United Kingdom
AB - Volcanic eruptions and lava flows comprise one of the most highly stressed terrestrial environments for the survival of biological organisms; the destruction of botanical and biological colonies by molten lava, pyroclastic flows, lahars, poisonous gas emissions and the deposition of highly toxic materials from fumaroles is the normal expectation from such events. However, the role of lichens and cyanobacteria in the earlier colonization of volcanic lava outcrops has now been recognized. In this paper, we build upon earlier Raman spectroscopic studies on extremophilic colonies in old lava flows to assess the potential of finding evidence of biological colonization in more recent lava deposits that would inform, first, the new colonization of these rocks and also provide evidence for the relict presence of biological colonies that existed before the volcanism occurred and were engulfed by the lava. In this research, samples were collected from a recent expedition to the active volcano at Kilauea, Hawaii, which comprises very recent lava flows, active fumaroles and volcanic rocks that had broken through to the ocean and had engulfed a coral reef. The Raman spectra indicated that biological and geobiological signatures could be identified in the presence of geological matrices, which is encouraging for the planned exploration of Mars, where it is believed that there is evidence of an active volcanism that perhaps could have preserved traces of biological activity that once existed on the planet's surface, especially in sites near the old Martian oceans. © 2010 The Royal Society.
KW - Exobiology
KW - Minerals
KW - Spectrum Analysis, Raman
KW - Volcanic Eruptions

KW - Biological materials
KW - Gas emissions
KW - Molten materials
KW - Oceanography
KW - Raman scattering
KW - Raman spectroscopy
KW - Spectroscopic analysis
KW - Toxic materials
KW - Volcanoes
KW - mineral
KW - Active volcanoes
KW - Biological activities
KW - Biological organisms
KW - Coral reef
KW - Kilauea
KW - Lava flows
KW - Martian ocean
KW - Organic signature
KW - Poisonous gas
KW - Pyroclastic flows
KW - Raman spectra
KW - Raman spectroscopic study
KW - Terrestrial environments
KW - Volcanic eruptions
KW - article
KW - biology
KW - Raman spectrometry
KW - volcano
KW - Volcanic rocks
KW - Fumaroles
KW - Lava flows
KW - Organic signatures
KW - Raman spectroscopy
KW - Volcanic outcrops
PB - Royal Society
SN - 1364503X (ISSN)
C2 - 20529949
LA - English
J2 - Philos. Trans. R. Soc. A Math. Phys. Eng. Sci.
M3 - Article
DB - Scopus
N1 - Export Date: 23 February 2023; Cited By: 9; Correspondence Address: S. E. Jorge-Villar; Area Geodinamica Interna, Facultad de Humanidades y Educacion, Universidad de Burgos, 09001 Burgos, Calle Villadiego s/n, Spain; email: susanajorgevillar@hotmail.com
ER -

TY - JOUR
AU - Kerr, R.A.
TI - Growing prospects for life on Mars divide astrobiologists
PY - 2010
T2 - Science
VL - 330
IS - 6000

SP - 26
EP - 27
DO - 10.1126/science.330.6000.26
UR - https://www.scopus.com/inward/record.uri?eid=2-s2.0-77957362563&doi=10.1126%2fscience.330.6000.26&partnerID=40&md5=1380745c3d198233345cb5abfdc81877
KW - Exobiology
KW - Extraterrestrial Environment
KW - Life
KW - Mars
KW - Methane
KW - Organic Chemicals
KW - Perchlorates
KW - Spacecraft
KW - Temperature
KW - Water
KW - carbon dioxide
KW - carbonic acid
KW - ground water
KW - mineral
KW - organic matter
KW - perchlorate
KW - methane
KW - organic compound
KW - perchlorate
KW - water
KW - carbon
KW - chemical compound
KW - Mars
KW - planetary atmosphere
KW - planetary landform
KW - astronomy
KW - climate change
KW - environmental temperature
KW - geological time
KW - glacier
KW - priority journal
KW - scientist
KW - sea
KW - short survey
KW - soil analysis
KW - astrobiology
KW - life
KW - microclimate
KW - space flight
KW - temperature
PB - American Association for the Advancement of Science
SN - 00368075 (ISSN)
C2 - 20929785
LA - English
J2 - Science
M3 - Short survey
DB - Scopus
N1 - Export Date: 23 February 2023; Cited By: 5; CODEN: SCIEA

ER -

TY - JOUR
AU - Bhattacharya, A.B.
AU - Sarkar, S.
AU - Bhattacharya, R.
TI - An alternative search strategy for interstellar communication
PY - 2010
T2 - Indian Journal of Physics
VL - 84
IS - 5
SP - 511
EP - 515
DO - 10.1007/s12648-010-0042-y
UR - https://www.scopus.com/inward/record.uri?eid=2-s2.0-79551635328&doi=10.1007%2fs12648-010-0042-y&partnerID=40&md5=c8c6b8c6f412019ba4394bd3eb232a44
AD - Department of Physics, University of Kalyani, Kalyani-741 235, West Bengal, India
AD - Department of Environmental Science, University of Kalyani, Kalyani-741 235, West Bengal, India
AB - Are there any better wavelengths (or frequencies) for gathering information of extraterrestrial intelligence? In recent years scientists have realized that intelligent life may well be found throughout the universe and we are, perhaps, not the only civilization in our galaxy. The paper suggests an alternative search strategy of extraterrestrial life through sky noise windows emphasizing both the 'water-holes' and the associated wavelengths. It also suggests that instead of pointing the antenna at known stars, an all sky search would be more appropriate for interstellar communication. © 2010 IACS.
KW - Extraterrestrial intelligence
KW - Interstellar communication
KW - Sky noise windows
PB - Scientific Publishers
SN - 09731458 (ISSN)
LA - English
J2 - Indian J. Phys.
M3 - Article
DB - Scopus
N1 - Export Date: 23 February 2023; Cited By: 2; Correspondence Address: A. B. Bhattacharya; Department of Physics, University of Kalyani, Kalyani-741 235, West Bengal, India; email: asit1951@yahoo.com
ER -

TY - JOUR
AU - Ramírez, S.I.
AU - Coll, P.
AU - Buch, A.
AU - Brassé, C.
AU - Poch, O.
AU - Raulin, F.
TI - The fate of aerosols on the surface of Titan

PY - 2010
T2 - Faraday Discussions
VL - 147
SP - 419
EP - 427
DO - 10.1039/c003925j
UR - https://www.scopus.com/inward/record.uri?eid=2-s2.0-78649513374&doi=10.1039%2fc003925j&partnerID=40&md5=f88ef30dbd901cf71bd780c6fea5aa79
AD - Centro de Investigaciones Químicas, Universidad Autónoma Del Estado de Morelos, Mexico
AD - Laboratoire Interuniversitaire des Systémes Atmosphériques, CNRS-IPSL, Universités Paris, Est-Créteil et Denis-Diderot, France
AD - École Centrale de Paris, Chatenay-Malabry, France
AB - A laboratory study based on the chemical transformation that Titan's aerosol analogues suffer when placed under putative surface conditions of the satellite was performed. In order to understand the role that aqueous ammonia may play on the chemical transformation of atmospheric aerosols once they reach the surface, we synthesized laboratory analogues of Titan's aerosols from an N2:CH4 (98:2) mixture irradiated at low temperatures under a continuous flow regime by a cold plasma discharge of 180 W. The analogues were recovered, partitioned in several 10.0 mg samples and placed inside different ammonia concentrations during 10 weeks at temperatures as low as those reported for Titan's surface. After a derivatization process performed to the aerosols' refractory phase with MTBSTFA in DMF, the products were identified and quantified using a GC-MS system. We found derived residues related to amino acids as well as urea. The simplest amino acids aminoethanoic acid (glycine) and 2-aminopropanoic acid (alanine) as well as diaminomethanal (urea), are found regardless of the ammonia concentration and temperature value to which the aerosol analogues were exposed. Our results have important astrobiological implications to Titan's environment particularly if the existence of the suggested subsurface water-ammonia mixture and its deposition on the satellite's surface is validated. © 2010 The Royal Society of Chemistry.
SN - 13645498 (ISSN)
LA - English
J2 - Faraday Discuss.
M3 - Article
DB - Scopus
N1 - Export Date: 23 February 2023; Cited By: 16; Correspondence Address: S. I. Ramírez; Centro de Investigaciones Químicas, Universidad Autónoma Del Estado de Morelos, Mexico; email: ramirez_sandra@uaem.mx
ER -

TY - JOUR
AU - Crawford, I.A.
TI - Astrobiological benefits of human space exploration
PY - 2010
T2 - Astrobiology
VL - 10

IS - 6
SP - 577
EP - 587
DO - 10.1089/ast.2010.0476
UR - https://www.scopus.com/inward/record.uri?eid=2-s2.0-77956145609&doi=10.1089%2fast.2010.0476&partnerID=40&md5=c57e7d61430e4acdcdd1a78656ddf759
AD - Department of Earth and Planetary Sciences, Birkbeck College, University of London, London, United Kingdom
AB - An ambitious program of human space exploration, such as that envisaged in the Global Exploration Strategy and considered in the Augustine Commission report, will help advance the core aims of astrobiology in multiple ways. In particular, a human exploration program will confer significant benefits in the following areas: (i) the exploitation of the lunar geological record to elucidate conditions on early Earth; (ii) the detailed study of near-Earth objects for clues relating to the formation of the Solar System; (iii) the search for evidence of past or present life on Mars; (iv) the provision of a heavy-lift launch capacity that will facilitate exploration of the outer Solar System; and (v) the construction and maintenance of sophisticated space-based astronomical tools for the study of extrasolar planetary systems. In all these areas a human presence in space, and especially on planetary surfaces, will yield a net scientific benefit over what can plausibly be achieved by autonomous robotic systems. A number of policy implications follow from these conclusions, which are also briefly considered. © Copyright 2010, Mary Ann Liebert, Inc. 2010.
KW - Adaptation, Physiological
KW - Exobiology
KW - Extraterrestrial Environment
KW - Geological Phenomena
KW - Humans
KW - Mars
KW - Moon
KW - Risk Assessment
KW - Solar System
KW - Space Flight
KW - adaptation
KW - article
KW - astrobiology
KW - astronomy
KW - geographic and geological phenomena
KW - human
KW - instrumentation
KW - methodology
KW - microclimate
KW - moon
KW - risk assessment
KW - space flight
KW - Astrobiology
KW - Human spaceflight
KW - Space exploration
SN - 15311074 (ISSN)
C2 - 20735249

LA - English
J2 - Astrobiology
M3 - Article
DB - Scopus
N1 - Export Date: 23 February 2023; Cited By: 9; Correspondence Address: I. A. Crawford; Department of Earth and Planetary Sciences, Birkbeck College London, London WC1E 7HX, Malet Street, United Kingdom; email: i.crawford@ucl.ac.uk
ER -

TY - CONF
AU - Barry, R.K.
AU - Deming, L.D.
TI - Alien earth: Glint observations of a remote planet
PY - 2010
T2 - Proceedings of the International Astronomical Union
VL - 6
IS - S276
SP - 471
EP - 472
DO - 10.1017/S1743921311020849
UR - https://www.scopus.com/inward/record.uri?eid=2-s2.0-84882943693&doi=10.1017%2fS1743921311020849&partnerID=40&md5=d8d4ca833528158652ad2e66eba22d02
AD - National Aeronautics and Space Administration, Goddard Space Flight Center, Greenbelt, MD, United States
AB - We give a preliminary report on a multi-wavelength study of specular reflections from the oceans and clouds of Earth. We use space-borne observations from a distance sufficient to ensure that light rays reflected from all parts of Earth are closely parallel, as they will be when studying exoplanets. We find that the glint properties of Earth in this far-field vantage point are surprising-in the sense that some of the brightest reflections are not from conventional ocean-glints, but appear to arise from cirrus cloud crystals. The Earth observations discussed here were acquired with the High Resolution Instrument (HRI)-a 0.3 m f/35 telescope on the Deep Impact (DI) spacecraft during the Extrasolar Planet Observation and Characterization (EPOCh) investigation. © 2011 International Astronomical Union.
KW - Astrobiology
KW - Astrochemistry
KW - Earth
KW - Infrared: planetary systems
KW - Planetary systems
A2 - Sozzetti A.
A2 - Lattanzi M.G.
A2 - INAF - Osservatorio, Astronomico di Torino
A2 - Boss A.P.
A2 - Carnegie Institution of Washington, Washington DC
SN - 17439221 (ISSN); 978-052119652-9 (ISBN)
LA - English
J2 - Proc. Int. Astron. Union
M3 - Conference paper
DB - Scopus

N1 - Export Date: 23 February 2023; Cited By: 0
ER -

TY - JOUR
AU - Zegers, T.E.
AU - Oosthoek, J.H.P.
AU - Rossi, A.P.
AU - Blom, J.K.
AU - Schumacher, S.
TI - Melt and collapse of buried water ice: An alternative hypothesis for the formation of chaotic terrains on Mars
PY - 2010
T2 - Earth and Planetary Science Letters
VL - 297
IS - 3-4
SP - 496
EP - 504
DO - 10.1016/j.epsl.2010.06.049
UR - https://www.scopus.com/inward/record.uri?eid=2-s2.0-77956012862&doi=10.1016%2fj.epsl.2010.06.049&partnerID=40&md5=67fe0e7fb939a4265ac21e188005085f
AD - Faculty of Geoscience, Department of Earth Sciences, Utrecht University, 3508 TA Utrecht, Budapestlaan 4, Netherlands
AD - TNO/Deltares, Geological Survey of the Netherlands, 3508 TA Utrecht, P.O. Box 80015, Netherlands
AD - International Space Science Institute, CH-3012 Bern, Hallerstrasse 6, Switzerland
AD - Dept. of Applied Earth Sciences, Delft University of Technology, 2628 CN Delft, Stevinweg 1, Netherlands
AD - Solar System Missions Division, Research and Scientific Support Department of ESA, ESTEC, 2200 AG Noordwijk, Netherlands
AB - Chaotic terrains and the associated massive outflow channels are some of the most enigmatic features on Mars. Over hundreds of kilometres of rock units are fractured, tilted, and have subsided, forming chaotic terrain basins (Sharp, 1973). Large quantities of water emanated from these chaotic terrains in short periods of time in the Hesperian epoch (~3.7-3.3Ga), carving huge outflow channels, thousands of kilometres long, and more than 1km deep (Baker, 2001). However, the subsurface mechanism by which chaotic terrains form, and thereby suddenly produce very large quantities of water (>105km3) is poorly understood. Here we explore if these features can form by melting and collapse of buried water ice in a confined basin. 2D thermal modelling, using boundary conditions derived from the geology of Aram Chaos, demonstrates that a buried ice unit will start melting when 1-2km of overburden has accumulated. The thickness of the liquid subsurface layer depends primarily on the crustal heat flux, the thermal conductivity of the overburden sediments, and the surface temperature. A subsurface liquid water layer of 1 to 2km can be achieved under present day surface temperature conditions and a crustal heat flux of 15-30mWm-2. To a first order, the geological features of chaotic terrains and their outflow channels are consistent with a scenario in which a subsurface lake forms by melting of buried water ice, followed by collapse and rapid outflow of water. If correct, this hypothesis

suggests that subsurface lakes on Mars may have existed for extensive (>100Ma) periods of time. Such subsurface lakes would be of major interest for astrobiology. © 2010 Elsevier B.V.
KW - Hesperia
KW - Atmospheric temperature
KW - Gallium
KW - Heat flux
KW - Lakes
KW - Landforms
KW - Liquids
KW - Martian surface analysis
KW - Melting
KW - Surface properties
KW - Chaotic terrain
KW - Confined basins
KW - First order
KW - Geological features
KW - Mars
KW - Outflow channel
KW - Rock units
KW - Short periods
KW - Subsurface layer
KW - Subsurface liquid
KW - Surface temperatures
KW - Thermal modelling
KW - Water ice
KW - complex terrain
KW - heat flux
KW - ice
KW - Mars
KW - melting
KW - outflow
KW - Ice
KW - Chaotic terrain
KW - Heat flux
KW - Mars
KW - Outflow channel
SN - 0012821X (ISSN)
LA - English
J2 - Earth Plan. Sci. Lett.
M3 - Article
DB - Scopus
N1 - Export Date: 23 February 2023; Cited By: 42; Correspondence Address: T.E. Zegers; Faculty of Geoscience, Department of Earth Sciences, Utrecht University, 3508 TA Utrecht, Budapestlaan 4, Netherlands; email: tanja@geo.uu.nl
ER -

TY - CHAP
AU - Kereszturi, A.
TI - Visualization in the education of astrobiology
PY - 2010
T2 - Astrobiology: Physical Origin, Biological Evolution and Spatial Distribution

SP - 131
EP - 141
UR - https://www.scopus.com/inward/record.uri?eid=2-s2.0-84863888609&partnerID=40&md5=5e457fb2badfbf028b4adb95e7b3ebc7
AD - Collegium Budapest Institute for Advanced Study, Hungarian Astronomical Association, Karoly Nagy Astronomical Foundation, Hungary
AB - This chapter summarizes the experiences regarding the general knowledge of undergraduate students in astrobiology, and presents methods we used in the education of astrobiology at university level in Hungary. Based on these, we found the following important issues. They have good general knowledge in the fields, which are well known for decades. Liquid water is thought to be widespread by the students, but they lack important information (like oceans inside icy moons) in the recent results. The most important is to develop and improve methods for the students to use the knowledge learned at different subjects more frequent in astrobiology. Useful examples, like the astrobiology- and Drake-matrixes are presented to compare different environments based on numerical values, graphics and tables. © 2010 Nova Science Publishers, Inc.
PB - Nova Science Publishers, Inc.
SN - 978-161761955-7 (ISBN); 978-160741290-8 (ISBN)
LA - English
J2 - Astrobiology: Physical Origin, Biological Evolution and Spatial Distribution
M3 - Book chapter
DB - Scopus
N1 - Export Date: 23 February 2023; Cited By: 3; Correspondence Address: A. Kereszturi; Collegium Budapest Institute for Advanced Study, Hungarian Astronomical Association, Karoly Nagy Astronomical Foundation, Hungary; email: akos@colbud.hu
ER -

TY - CONF
AU - Brack, A.
AU - Coradini, M.
TI - Terrestrial atmosphere, water and astrobiology
PY - 2010
T2 - EPJ Web of Conferences
VL - 9
SP - 241
EP - 265
DO - 10.1051/epjconf/201009020
UR - https://www.scopus.com/inward/record.uri?eid=2-s2.0-84921487224&doi=10.1051%2fepjconf%2f201009020&partnerID=40&md5=0bd4e110d837a2ab2844947f0d63679a
AD - Centre de Biophysique Moléculaire, CNRS, Rue Charles Sadron, Orléans Cedex 2, 45071, France
AD - ESA, 8-10 rue Mario Nikis, Paris, 75015, France
AB - Primitive life, defined as a chemical system capable to transfer its molecular information via self-replication and also capable to evolve, originated about 4 billion years ago from the processing of organic molecules by liquid water. Terrestrial atmosphere played a key role in the process by allowing the

permanent presence of liquid water and by participating in the production of carbon-based molecules. Water molecules exhibit specific properties mainly due to a dense network of hydrogen bonds. The carbon-based molecules were either home made in the atmosphere and/or in submarine hydrothermal systems or delivered by meteorites and micrometeorites. The search for possible places beyond the earth where the trilogy atmosphere/water/life could exist is the main objective of astrobiology. Within the Solar System, exploration missions are dedicated to Mars, Europa, Titan and the icy bodies. The discovery of several hundreds of extrasolar planets opens the quest to the whole Milky Way. © Owned by the authors, published by EDP Sciences, 2010.
A2 - Boutron C.
PB - EDP Sciences
SN - 21016275 (ISSN); 978-275980599-0 (ISBN)
LA - English
J2 - EPJ Web Conf.
M3 - Conference paper
DB - Scopus
N1 - Export Date: 23 February 2023; Cited By: 0
ER -

TY - JOUR
AU - Benford, G.
AU - Benford, J.
AU - Benford, D.
TI - Searching for cost-optimized interstellar beacons
PY - 2010
T2 - Astrobiology
VL - 10
IS - 5
SP - 491
EP - 498
DO - 10.1089/ast.2009.0394
UR - https://www.scopus.com/inward/record.uri?eid=2-s2.0-78650041300&doi=10.1089%2fast.2009.0394&partnerID=40&md5=c8b7271422f55aa9ac122300cfc0f207
AD - Physics and Astronomy Department, University of California Irvine, Irvine, CA, United States
AD - Microwave Sciences, Inc., Lafayette, CA 94549, United States
AD - Observational Cosmology Lab, NASA Goddard Space Flight Center, Greenbelt, MD, United States
AB - What would SETI beacon transmitters be like if built by civilizations that had a variety of motives but cared about cost? In a companion paper, we presented how, for fixed power density in the far field, a cost-optimum interstellar beacon system could be built. Here, we consider how we should search for a beacon if it were produced by a civilization similar to ours. High-power transmitters could be built for a wide variety of motives other than the need for two-way communication; this would include beacons built to be seen over thousands of light-years. Extraterrestrial beacon builders would likely have to contend with economic pressures just as their terrestrial counterparts do. Cost, spectral lines near 1GHz, and interstellar scintillation favor radiating frequencies substantially

above the classic "water hole." Therefore, the transmission strategy for a distant, cost-conscious beacon would be a rapid scan of the galactic plane with the intent to cover the angular space. Such pulses would be infrequent events for the receiver. Such beacons built by distant, advanced, wealthy societies would have very different characteristics from what SETI researchers seek. Future searches should pay special attention to areas along the galactic disk where SETI searches have seen coherent signals that have not recurred on the limited listening time intervals we have used. We will need to wait for recurring events that may arrive in intermittent bursts. Several new SETI search strategies have emerged from these ideas. We propose a new test for beacons that is based on the Life Plane hypotheses. © Mary Ann Liebert, Inc.
KW - Communication
KW - Exobiology
KW - Extraterrestrial Environment
KW - Galaxies
KW - Microwaves
KW - article
KW - astrobiology
KW - astronomy
KW - economics
KW - instrumentation
KW - interpersonal communication
KW - microclimate
KW - microwave radiation
KW - SETI-METI-Microwave-Power beaming-Beacons-Radio astronomy-Array antennas-High-power microwaves.
SN - 15311074 (ISSN)
C2 - 20624057
LA - English
J2 - Astrobiology
M3 - Article
DB - Scopus
N1 - Export Date: 23 February 2023; Cited By: 32; Correspondence Address: J. Benford; Microwave Sciences, Inc., Lafayette, CA 94549, United States; email: jbenford@earthlink.net
ER -

TY - CONF
AU - Wang, C.
TI - Launch cavity ringdown spectroscopy from Earth to space
PY - 2010
T2 - Proceedings of the 12th International Conference on Engineering, Science, Construction, and Operations in Challenging Environments - Earth and Space 2010
SP - 1644
EP - 1654
DO - 10.1061/41096(366)149
UR - https://www.scopus.com/inward/record.uri?eid=2-s2.0-78649301952&doi=10.1061%2f41096%28366%29149&partnerID=40&md5=bf7a4add0d475255c6a0289110a3ad05
AD - Department of Physics and Astronomy, Mississippi State University, Mississippi State, MS 39762, P. O. Box 5167, United

States
AD - Institute for Clean Energy Technology, Mississippi State University, Starkville, MS 39759, 205 Research Boulevard, United States
AB - Cavity ringdown spectroscopy and its associated techniques have been widely implemented for measurement, monitoring, and sensing in many applications in diverse fields, including energy, medicine, materials, environment, infrastructure, etc. Cavity ringdown technique has not been actually extended beyond its applications on Earth to new applications in space although ringdown instruments have been loaded to an aircraft to conduct real-time survey of atmospheric species. In addition to gas sensing, recent developments have advanced ringdown techniques for physical sensor developments by using a section of optical fiber as a conceptual high-reflectivity cavity without sacrificing the intrinsic natures of ringdown: near-real time response, high sensitivity, and measuring time constants to sense a quantity. Cavity ringdown has great potential to help address three significant issues in sensing applications associated with space science and engineering: time, logistics, and intelligence. The purpose of this paper is to introduce cavity ringdown to a new community: intelligent sensors for space science and engineering and to explore new applications, e.g., light-weight, multi-function, intelligent sensors for space vehicle structure health morning and control, real-time chemical sensors for methane, carbon dioxide, water, and carbon isotopes for extraterrestrial life exploration, and medical sensors for astronauts' metabolic status monitoring. © 2010 ASCE.
KW - Aircraft instruments
KW - Aircraft materials
KW - Astronomy
KW - Carbon dioxide
KW - Intelligent control
KW - Intelligent materials
KW - Isotopes
KW - Manned space flight
KW - Methane
KW - Monitoring
KW - Optical fibers
KW - Smart sensors
KW - Space research
KW - Structural health monitoring
KW - Atmospheric species
KW - Carbon isotopes
KW - Cavity ring-down
KW - Cavity ring-down spectroscopy
KW - Diverse fields
KW - Extraterrestrial life
KW - Gas sensing
KW - High reflectivity
KW - High sensitivity
KW - Intelligent sensors
KW - Intrinsic nature
KW - Light weight
KW - Medical sensors

KW - Multi-functions
KW - Near-real time
KW - New applications
KW - Physical sensors
KW - Sensing applications
KW - Smart materials
KW - Space explorations
KW - Space science
KW - Space vehicles
KW - Time constants
KW - Earth (planet)
KW - Fibers
KW - Smart materials
KW - Space exploration
SN - 978-078441096-7 (ISBN)
LA - English
J2 - Proc. Int. Conf. Eng., Sci., Constr., Oper. Challenging Environ. - Earth and Space
M3 - Conference paper
DB - Scopus
N1 - Export Date: 23 February 2023; Cited By: 0; Correspondence Address: C. Wang; Department of Physics and Astronomy, Mississippi State University, Mississippi State, MS 39762, P. O. Box 5167, United States; email: cw175@msstate.edu
ER -

TY - JOUR
AU - Vidal-Madjar, A.
AU - Arnold, L.
AU - Ehrenreich, D.
AU - Ferlet, R.
AU - Lecavelier Des Etangs, A.
AU - Bouchy, F.
AU - Segransan, D.
AU - Boisse, I.
AU - Hébrard, G.
AU - Moutou, C.
AU - Désert, J.-M.
AU - Sing, D.K.
AU - Cabanac, R.
AU - Nitschelm, C.
AU - Bonfils, X.
AU - Delfosse, X.
AU - Desort, M.
AU - Diaz, R.F.
AU - Eggenberger, A.
AU - Forveille, T.
AU - Lagrange, A.-M.
AU - Lovis, C.
AU - Pepe, F.
AU - Perrier, C.
AU - Pont, F.
AU - Santos, N.C.
AU - Udry, S.

TI - The Earth as an extrasolar transiting planet: Earth's atmospheric composition and thickness revealed by Lunar eclipse observations
PY - 2010
T2 - Astronomy and Astrophysics
VL - 523
IS - 3
C7 - A55
DO - 10.1051/0004-6361/201014762
UR - https://www.scopus.com/inward/record.uri?eid=2-s2.0-78449241488&doi=10.1051%2f0004-6361%2f201014762&partnerID=40&md5=adec233a317d6591cd97805a50a312b4
AD - Institut d'Astrophysique de Paris, UMR7095 CNRS, Université Pierre and Marie Curie, 75014 Paris, 98bis boulevard Arago, France
AD - Observatoire de Haute-Provence, CNRS/OAMP, 04870 Saint-Michel-l'Observatoire, France
AD - Laboratoire d'Astrophysique de Grenoble, Université Joseph Fourier, CNRS (UMR 5571), 38041 Grenoble Cedex 9, BP 53, France
AD - Observatoire de Genève, Université de Genève, 1290 Sauverny, 51 Chemin des Maillettes, Switzerland
AD - Laboratoire d'Astrophysique de Marseille, Université de Provence, CNRS (UMR6110), 13376 Marseille Cedex 12, Technopôle Marseille Itoile, France
AD - Harvard-Smithsonian Center for Astrophysics, Cambridge, MA 02138, 60 Garden Street, United States
AD - School of Physics, University of Exeter, Exeter EX4 4QL, United Kingdom
AD - Observatoire Midi-Pyrénées, TBL, 65000 Tarbes, 57 Ave d'Azereix, France
AD - Instituto de Astronomía, Universidad Católica Del Norte, Antofagasta, Avenida Angamos 0610, Chile
AD - Centro de Astrofisica, Universidade Do Porto, 4150-762 Porto, Rua das Estrelas, Portugal
AB - Context. An important goal within the quest for detecting an Earth-like extrasolar planet, will be to identify atmospheric gaseous bio-signatures. Aims. Observations of the light transmitted through the Earth's atmosphere, as for an extrasolar planet, will be the first important step for future comparisons. We have completed observations of the Earth during a lunar eclipse, a unique situation similar to that of a transiting planet. We aim at showing what species could be detected in its atmosphere at optical wavelengths, where a lot of photons are available in the masked stellar light. Methods. We present observations of the 2008 August 16 Moon eclipse performed with the SOPHIE spectrograph at the Observatoire de Haute-Provence (France). Locating the spectrograph's fibers in the penumbra of the eclipse, the Moon irradiance is then a mix of direct, unabsorbed Sun light and solar light that has passed through the Earth's atmosphere. This mixture essentially reproduces what is recorded during the transit of an extrasolar planet. Results. We report here the clear detection of several Earth atmospheric compounds in the transmission spectra, such as ozone, molecular oxygen, and neutral sodium as well as molecular nitrogen and oxygen through the Rayleigh signature. Moreover, we present a method that allows us to derive the thickness of the atmosphere versus the

wavelength for penumbra eclipse observations. We quantitatively evaluate the altitude at which the atmosphere becomes transparent for important species like molecular oxygen and ozone, two species thought to be tightly linked to the presence of life. Conclusions. The molecular detections presented here are an encouraging first attempt, necessary to better prepare for the future of extremely-large telescopes and transiting Earth-like planets. Instruments like SOPHIE will be mandatory when characterizing the atmospheres of transiting Earth-like planets from the ground and searching for bio-marker signatures. © 2010 ESO.
KW - Astrobiology
KW - Atmospheric composition
KW - Earth (planet)
KW - Light
KW - Molecular oxygen
KW - Moon
KW - Ozone
KW - Satellites
KW - Spectrographs
KW - eclipses
KW - Extremely Large Telescopes
KW - Methods:observational
KW - Molecular detection
KW - Molecular nitrogen
KW - Planets and satellites: atmospheres
KW - Techniques: spectroscopic
KW - Transmission spectrums
KW - Extrasolar planets
KW - astrobiology
KW - Earth
KW - eclipses
KW - methods: observational
KW - planets and satellites: atmospheres
KW - techniques: spectroscopic
PB - EDP Sciences
SN - 00046361 (ISSN)
LA - English
J2 - Astron. Astrophys.
M3 - Article
DB - Scopus
N1 - Export Date: 23 February 2023; Cited By: 12; Correspondence Address: A. Vidal-Madjar; Institut d'Astrophysique de Paris, UMR7095 CNRS, Université Pierre and Marie Curie, 75014 Paris, 98bis boulevard Arago, France; email: alfred@iap.fr; CODEN: AAEJA
ER -

TY - JOUR
AU - Cockell, C.S.
TI - Astrobiology—What Can We Do on the Moon?
PY - 2010
T2 - Earth, Moon and Planets
VL - 107
IS - 1
SP - 3

EP - 10
DO - 10.1007/s11038-010-9363-2
UR - https://www.scopus.com/inward/record.uri?eid=2-s2.0-79952402823&doi=10.1007%2fs11038-010-9363-2&partnerID=40&md5=75edc9deb5c857038dfc9d12099a5a1b
AD - CEPSAR, Open University, Milton Keynes MK7 6AA, United Kingdom
AB - The Moon does not seem to be a place for a biologist. However, it offers the possibility of unravelling a better understanding of the conditions for habitability on the Earth and the conditions for life on the early Earth. It will be a place where much of the life sciences technologies required to establish a permanent human presence in space can be tested to complete reliability. Specifically, a long-term life sciences laboratory on the Moon can be used to investigate three areas of science that are currently poorly understood: (1) the linearity or non-linearity of the effects of different magnitudes of space environmental stresses on organisms, particularly gravity; (2) the effects of cumulative environmental effects both in individual organisms and across generations, (3) the synergistic effects of different space environmental parameters on organisms. The close proximity and scientific importance of the Moon makes it a useful permanent location and staging post for the human expansion into space. © 2010 Springer Science+Business Media B.V.
KW - Earth science
KW - Mars
KW - Moon
KW - planetary evolution
KW - Astrobiology
KW - Life sciences
KW - Life support
KW - Mars
KW - Moon
SN - 01679295 (ISSN)
LA - English
J2 - Earth Moon Planets
M3 - Article
DB - Scopus
N1 - Export Date: 23 February 2023; Cited By: 16; Correspondence Address: C. S. Cockell; CEPSAR, Open University, Milton Keynes MK7 6AA, United Kingdom; email: c.s.cockell@open.ac.uk
ER -

TY - JOUR
AU - Chung, S.Y.
AU - Ehrenfreund, P.
AU - Rummel, J.D.
AU - Peter, N.
TI - Synergies of Earth science and space exploration
PY - 2010
T2 - Advances in Space Research
VL - 45
IS - 1
SP - 155
EP - 168

DO - 10.1016/j.asr.2009.10.025
UR - https://www.scopus.com/inward/record.uri?eid=2-s2.0-71649105289&doi=10.1016%2fj.asr.2009.10.025&partnerID=40&md5=3256f7f6add51a70952a647ba04e4238
AD - Space Policy Institute, Elliott School of International Affairs, The George Washington University, Washington, DC 20052, 1957 E Street NW, United States
AD - Institute for Coastal Science and Policy, East Carolina University, Greenville, NC 27858-4353, 2200 South Charles Boulevard 1500, United States
AD - European Space Policy Institute, A-1030 Vienna, Schwarzenbergplatz 6, Austria
AB - A more flexible policy basis from which to manage our planet in the 21st century is desirable. As one contribution, we note that synergies between space exploration and the preservation of our habitat exist, and that protecting life on Earth requires similar concepts and information as investigations of life beyond the Earth, including the expansion of human presence in space. Instrumentation and data handling to observe both planetary objects and planet Earth are based on similar techniques. Moreover, while planetary surface operations are conducted under different conditions, the technology to probe the surface and subsurface of both the Earth and other planets requires similar tools, such as radar, seismometers, and drilling devices. The Earth observation community has developed some exemplary tools and has featured successful international cooperation in data handling and sharing that could be equally well applied to robotic planetary exploration. Here we propose a network involving both communities that will enable the interchange of scientific insights and the development of new policies and management strategies. Those tools can provide a vital forum through which the management of this planet can be assisted, and in which a new bridge between the Earth-centric and space-centric communities can be built. © 2009 COSPAR.
KW - Astrobiology
KW - Biodiversity
KW - Data handling
KW - International cooperation
KW - Interplanetary spacecraft
KW - Observatories
KW - Space research
KW - Earth observations
KW - Management strategies
KW - Planet earth
KW - Planetary surfaces
KW - Robotic planetary exploration
KW - Space cooperation
KW - Space explorations
KW - Space weather
KW - Earth (planet)
KW - Astrobiology
KW - Biodiversity
KW - Earth observation
KW - International space cooperation
KW - Space exploration

KW - Space weather
PB - Elsevier Ltd
SN - 02731177 (ISSN)
LA - English
J2 - Adv. Space Res.
M3 - Article
DB - Scopus
N1 - Export Date: 23 February 2023; Cited By: 8; Correspondence Address: P. Ehrenfreund; Space Policy Institute, Elliott School of International Affairs, The George Washington University, Washington, DC 20052, 1957 E Street NW, United States; email: pehren@gwu.edu; CODEN: ASRSD
ER -

TY - CONF
AU - Schilizzi, R.T.
AU - Dewdney, P.E.F.
AU - Lazioa, T.J.W.
TI - The square kilometre array
PY - 2010
T2 - Proceedings of SPIE - The International Society for Optical Engineering
VL - 7733
IS - PART 1
C7 - 773318
DO - 10.1117/12.856344
UR - https://www.scopus.com/inward/record.uri?eid=2-s2.0-77958131754&doi=10.1117%2f12.856344&partnerID=40&md5=65484d33726f00b635a5488d010afcb8
AD - SKA Program Development Office, Jodrell Bank Centre for Astrophysics, University of Manchester, Manchester M13 9PL, United Kingdom
AD - Naval Research Laboratory, Washington, DC 20375-5351, 4555 Overlook Ave. SW, United States
AB - The Square Kilometre Array is intended to be the next-generation radio wavelength observatory. With a Key Science program addressing fundamental physics, astronomy, and astrobiology, the SKA will have a collecting area of up to one million square metres spread over at least 3000 km, providing a sensitivity 50 times higher than the Expanded Very Large Array, and an instantaneous field of view (FoV) of at least several tens of square degrees and possibly 250 square degrees. In this paper, we describe the main features of the SKA, paying attention to the design activities around the world, and outline plans for the final design and phased implementation of the telescope. © 2010 SPIE.
KW - Airborne telescopes
KW - Optical telescopes
KW - Radar astronomy
KW - Radio astronomy
KW - Design activity
KW - Field of views
KW - Fundamental physics
KW - Radio wavelengths
KW - Science programs

KW - Square kilometre array
KW - Very large arrays
KW - Astrobiology
KW - Radio astronomy
KW - Square kilometre array
SN - 0277786X (ISSN); 978-081948223-5 (ISBN)
LA - English
J2 - Proc SPIE Int Soc Opt Eng
M3 - Conference paper
DB - Scopus
N1 - Export Date: 23 February 2023; Cited By: 16; CODEN: PSISD
ER -

TY - JOUR
AU - Weidner, C.
AU - Horne, K.
TI - Limits on the orbits and masses of moons around currently-known transiting exoplanets
PY - 2010
T2 - Astronomy and Astrophysics
VL - 521
IS - 8
C7 - A76
DO - 10.1051/0004-6361/201014955
UR - https://www.scopus.com/inward/record.uri?eid=2-s2.0-77958548084&doi=10.1051%2f0004-6361%2f201014955&partnerID=40&md5=8d3441f8503658a79cf917b01e0cf6de
AD - Scottish Universities Physics Alliance (SUPA), School of Physics and Astronomy, University of St. Andrews, North Haugh, St. Andrews, Fife KY16 9SS, United Kingdom
AB - Aims. Current and upcoming space missions may be able to detect moons of transiting extra-solar planets. In this context it is important to understand if exomoons are expected to exist and what their possible properties are. Methods. Using estimates for the stability of exomoon orbits from numerical studies, a list of 87 known transiting exoplanets is tested for the potential to host large exomoons. Results. For 92% of the sample, moons larger than Luna can be excluded on prograde orbits, unless the parent exoplanet's internal structure is very different from the gas-giants of the solar system. Only WASP-24b, OGLE2-TR-L9, CoRoT-3b and CoRoT-9b could have moons above 0.4 m⊕, which is within the likely detection capabilities of current observational facilities. Additionally, the range of possible orbital radii of exomoons of the known transiting exoplanets, with two exceptions, is below 8 Jupiter-radii and therefore rather small. © 2010 ESO.
KW - Interplanetary flight
KW - Planets
KW - Solar system
KW - Detection capability
KW - Exo-planets
KW - Exoplanet
KW - Extra solar planets
KW - Internal structure
KW - Jupiters

KW - Numerical studies
KW - Planets and satellites
KW - planets and satellites: formation
KW - Space missions
KW - Satellites
KW - astrobiology
KW - planets and satellites: detection
KW - planets and satellites: dynamical evolution and stability
KW - planets and satellites: formation
SN - 14320746 (ISSN)
LA - English
J2 - Astron. Astrophys.
M3 - Article
DB - Scopus
N1 - Export Date: 23 February 2023; Cited By: 30; Correspondence Address: C. Weidner; Scottish Universities Physics Alliance (SUPA), School of Physics and Astronomy, University of St. Andrews, North Haugh, St. Andrews, Fife KY16 9SS, United Kingdom; email: cw60@st-andrews.ac.uk; CODEN: AAEJA
ER -

TY - JOUR
AU - Davila, A.F.
AU - Duport, L.G.
AU - Melchiorri, R.
AU - Jänchen, J.
AU - Valea, S.
AU - De Los Rios, A.
AU - Fairén, A.G.
AU - Möhlmann, D.
AU - McKay, C.P.
AU - Ascaso, C.
AU - Wierzchos, J.
TI - Hygroscopic salts and the potential for life on mars
PY - 2010
T2 - Astrobiology
VL - 10
IS - 6
SP - 617
EP - 628
DO - 10.1089/ast.2009.0421
UR - https://www.scopus.com/inward/record.uri?eid=2-s2.0-77956160834&doi=10.1089%2fast.2009.0421&partnerID=40&md5=45e981dd76dfab2b7f8926f8baf0c227
AD - SETI Institute, Mountain View, CA, 514 Wishman Road, United States
AD - Universidad de Vigo, Campus Lagoas Marcosende, Vigo, Spain
AD - SRI International, Molecular Physics Laboratory, Physical Science Division, Menlo Park, CA, United States
AD - Technical University of Applied Sciences Wildau, Wildau, Germany
AD - Instituto de Recursos Naturales, CCMA, CSIC, Madrid, Spain
AD - DLR Institut für Planetenforschung, Berlin, Germany
AD - NASA Ames Research Center, Moffett Field, CA, United States

AB - Hygroscopic salts have been detected in soils in the northern latitudes of Mars, and widespread chloride-bearing evaporitic deposits have been detected in the southern highlands. The deliquescence of hygroscopic minerals such as chloride salts could provide a local and transient source of liquid water that would be available for microorganisms on the surface. This is known to occur in the Atacama Desert, where massive halite evaporites have become a habitat for photosynthetic and heterotrophic microorganisms that take advantage of the deliquescence of the salt at certain relative humidity (RH) levels. We modeled the climate conditions (RH and temperature) in a region on Mars with chloride-bearing evaporites, and modeled the evolution of the water activity (aw) of the deliquescence solutions of three possible chloride salts (sodium chloride, calcium chloride, and magnesium chloride) as a function of temperature. We also studied the water absorption properties of the same salts as a function of RH. Our climate model results show that the RH in the region with chloride-bearing deposits on Mars often reaches the deliquescence points of all three salts, and the temperature reaches levels above their eutectic points seasonally, in the course of a martian year. The aw of the deliquescence solutions increases with decreasing temperature due mainly to the precipitation of unstable phases, which removes ions from the solution. The deliquescence of sodium chloride results in transient solutions with a w compatible with growth of terrestrial microorganisms down to 252 K, whereas for calcium chloride and magnesium chloride it results in solutions with aw below the known limits for growth at all temperatures. However, taking the limits of aw used to define special regions on Mars, the deliquescence of calcium chloride deposits would allow for the propagation of terrestrial microorganisms at temperatures between 265 and 253 K, and for metabolic activity (no growth) at temperatures between 253 and 233 K. © Copyright 2010, Mary Ann Liebert, Inc. 2010.
KW - Absorption
KW - Climate
KW - Exobiology
KW - Extraterrestrial Environment
KW - Humidity
KW - Hygroscopic Agents
KW - Mars
KW - Partial Pressure
KW - Salts
KW - Steam
KW - Water
KW - hygroscopic agent
KW - inorganic salt
KW - water
KW - absorption
KW - article
KW - astrobiology
KW - astronomy
KW - chemistry
KW - climate
KW - humidity
KW - microclimate

KW - pressure
KW - water vapor
KW - Atacama Desert
KW - Deliquescence
KW - Endoliths
KW - Evaporites
KW - Hygroscopic salts
KW - Life
KW - Mars
KW - Temperature
KW - Water activity
SN - 15311074 (ISSN)
C2 - 20735252
LA - English
J2 - Astrobiology
M3 - Article
DB - Scopus
N1 - Export Date: 23 February 2023; Cited By: 126; Correspondence Address: A. F. Davila; SETI Institute, Mountain View, CA, 514 Wishman Road, United States; email: adavila@seti.org
ER -

TY - JOUR
AU - Rommevaux-Jestin, C.
AU - Ménez, B.
TI - Potential of cathodoluminescence microscopy and spectroscopy for the detection of prokaryotic cells on minerals
PY - 2010
T2 - Astrobiology
VL - 10
IS - 9
SP - 921
EP - 932
DO - 10.1089/ast.2010.0490
UR - https://www.scopus.com/inward/record.uri?eid=2-s2.0-78650071254&doi=10.1089%2fast.2010.0490&partnerID=40&md5=fe776e63bda8949d5df3aa0ee82a0185
AD - IPGP/IMPMC, CNRS, Centre de Recherches sur le Stockage Géologique du CO2 (IPGP/TOTAL/SCHLUMBERGER/ADEME), Paris, France
AB - Detecting mineral-hosted ecosystems to assess the extent and functioning of the biosphere from the surface to deep Earth requires appropriate techniques that provide, beyond the morphological criteria, indubitable clues of the presence of prokaryotic cells. Here, we evaluate the capability of cathodoluminescence microscopy and spectroscopy, implemented on a scanning electron microscope, to identify prokaryotes on mineral surfaces. For this purpose, we used, as a first step, a simple model of either unstained or stained cultivable cells (Escherichia coli, Deinococcus radiodurans) deposited on minerals that are common in the oceanic crust (basaltic glass, amphibole, pyroxene, and magnetite). Our results demonstrate that the detection of cells is possible at the micrometric level on the investigated minerals through the intrinsic fluorescence of their constituting macromolecules (aromatic amino and nucleic acids, coenzymes). This allows us to distinguish biomorph inorganic phases

from cells. This easily implemented technique permits an exploration of colonized rock samples. In addition, the range of spectrometric techniques available on a scanning electron microscope can provide additional information on the nature and chemistry of the associated mineral phases, which would lead to a simultaneous characterization of cells, their microhabitats, and a better understanding of their potential relationships. Key Words: Prokaryotic cells–Cathodoluminescence–Intrinsic fluorophores/autofluorescence–Scanning electron microscope–Mineral-hosted ecosystems. Astrobiology 10, 921–932. © Mary Ann Liebert, Inc.
KW - Deinococcus
KW - Escherichia coli
KW - Ferrosoferric Oxide
KW - Fluorescence
KW - Microscopy, Electron, Scanning
KW - Minerals
KW - Silicates
KW - Deinococcus radiodurans
KW - Escherichia coli
KW - Prokaryota
KW - magnetite
KW - mineral
KW - pyroxene
KW - silicate
KW - article
KW - basalt
KW - chemistry
KW - Deinococcus
KW - Escherichia coli
KW - fluorescence
KW - metabolism
KW - scanning electron microscopy
KW - ultrastructure
SN - 15311074 (ISSN)
C2 - 21118024
LA - English
J2 - Astrobiology
M3 - Article
DB - Scopus
N1 - Export Date: 23 February 2023; Cited By: 2; Correspondence Address: C. Rommevaux-Jestin; IPGP/IMPMC, CNRS, Bureau 140, 75238 PARIS cedex 05, 1 rue Jussieu, France; email: rommevau@ipgp.fr
ER -

TY - JOUR
AU - Crawford, I.A.
AU - Fagents, S.A.
AU - Joy, K.H.
AU - Rumpf, M.E.
TI - Lunar Palaeoregolith Deposits as Recorders of the Galactic Environment of the Solar System and Implications for Astrobiology
PY - 2010
T2 - Earth, Moon and Planets
VL - 107

IS - 1
SP - 75
EP - 85
DO - 10.1007/s11038-010-9358-z
UR - https://www.scopus.com/inward/record.uri?eid=2-s2.0-79952444470&doi=10.1007%2fs11038-010-9358-z&partnerID=40&md5=bbc57d4ea1b65d222ae2a71bf7f7a147
AD - Department of Earth and Planetary Sciences, Birkbeck College, University of London, London WC1E 7HX, Malet Street, United Kingdom
AD - Centre for Planetary Sciences at UCL/Birkbeck, University College London, London WC1E 6BT, Gower Street, United Kingdom
AD - Hawaii Institute of Geophysics and Planetology, University of Hawaii, Honolulu, HI 96822, United States
AD - The Center for Lunar Science and Exploration, Lunar and Planetary Institute, Houston, TX 77058, United States
AD - The NASA Lunar Science Institute, Houston, TX, United States
AB - One of the principal scientific reasons for wanting to resume in situ exploration of the lunar surface is to gain access to the record it contains of early Solar System history. Part of this record will pertain to the galactic environment of the Solar System, including variations in the cosmic ray flux, energetic galactic events (e.g., supernovae and/or gamma-ray bursts), and passages of the Solar System through dense interstellar clouds. Much of this record is of astrobiological interest as these processes may have affected the evolution of life on Earth, and perhaps other Solar System bodies. We argue that this galactic record, as for that of more local Solar System processes also of astrobiological interest, will be best preserved in ancient, buried regolith ('palaeoregolith') deposits in the lunar near sub-surface. Locating and sampling such deposits will be an important objective of future lunar exploration activities. © 2010 Springer Science+Business Media B.V.
KW - exobiology
KW - exploration
KW - lunar soil
KW - Moon
KW - regolith
KW - solar system
KW - Astrobiology
KW - Galactic history
KW - Galactic structure
KW - Lunar exploration
KW - Lunar regolith
KW - Moon
SN - 01679295 (ISSN)
LA - English
J2 - Earth Moon Planets
M3 - Article
DB - Scopus
N1 - Export Date: 23 February 2023; Cited By: 31; Correspondence Address: I. A. Crawford; Department of Earth and Planetary Sciences, Birkbeck College, University of London, London WC1E 7HX, Malet Street, United Kingdom; email: i.crawford@ucl.ac.uk
ER -

TY - JOUR
AU - Mielke, R.E.
AU - Russell, M.J.
AU - Wilson, P.R.
AU - McGlynn, S.E.
AU - Coleman, M.
AU - Kidd, R.
AU - Kanik, I.
TI - Design, fabrication, and test of a hydrothermal reactor for origin-of-life experiments
PY - 2010
T2 - Astrobiology
VL - 10
IS - 8
SP - 799
EP - 810
DO - 10.1089/ast.2009.0456
UR - https://www.scopus.com/inward/record.uri?eid=2-s2.0-78649505629&doi=10.1089%2fast.2009.0456&partnerID=40&md5=a3119b5ddc6472a58b5fa6a11a140e1d
AD - Planetary Science, Jet Propulsion Laboratory, California Institute of Technology, Pasadena, CA 91109-8099, 4800 Oak Grove Drive, United States
AD - NASA Astrobiology Institute, United States
AD - Department of Chemistry and Biochemistry, Astrobiology Biogeocatalysis Research Center, Montana State University, Bozeman, MT, United States
AB - We describe a continuous high-pressure flow reactor designed to simulate the unforced convective interaction of hydrothermal solutions and ocean waters with submarine crust on early Earth-conditions appropriate to those that may have led to the onset of life. The experimental operating conditions are appropriate for investigating kinetic hydrothermal processes in the early history of any sizable wet, rocky planet. Beyond the description of the fabrication, we report an initial experiment that tested the design and investigated the feasibility of sulfide and silica dissolution in alkaline solution from iron sulfide and basaltic rock, and their possible subsequent transport as HS- and H2SiO2-4 in hot alkaline solutions. Delivery of hydrogen sulfide and dihydrogen silicate ions would have led to the precipitation of ferrous hydroxide, hydroxysilicates, and iron sulfides as integral mineral components of an off-ridge compartmentalized hydrothermal mound in the Hadean. Such a mound could, we contend, have acted as a natural chemical and electrochemical reactor and, ultimately, as the source of all biochemistry on our planet. In the event, we show that an average of ~1mM/kg of both sulfide and silica were released throughout, though over 10mM/kg of HS- was recorded for ~100 minutes in the early stages of the experiment. This alkaline effluent from the reactor was injected into a reservoir of a simulacrum of ferrous iron-bearing "Hadean Ocean" water in an experiment that demonstrated the capacity of such fluids to generate hydrothermal chimneys and a variety of contiguous inorganic microgeode precipitates bearing disseminations of discrete metal sulfides. Comparable natural

composite structures may have acted as hatcheries for emergent life in the Hadean. © Copyright 2010, Mary Ann Liebert, Inc. 2010.
KW - Biogenesis
KW - Hot Temperature
KW - Hydrogen Sulfide
KW - Silicates
KW - Water
KW - hydrogen sulfide
KW - silicate
KW - water
KW - article
KW - basalt
KW - biogenesis
KW - chemistry
KW - heat
KW - 799–810.
KW - Hydrothermal reactor–Hydrothermal mound–Hydrothermal chimneys–Geodes– Transition–metal sulfides–Hadean Ocean. Astrobiology 10
SN - 15311074 (ISSN)
C2 - 21087160
LA - English
J2 - Astrobiology
M3 - Article
DB - Scopus
N1 - Export Date: 23 February 2023; Cited By: 55; Correspondence Address: M. J. Russell; Planetary Science, Jet Propulsion Laboratory, California Institute of Technology, Pasadena, CA 91109-8099, 4800 Oak Grove Drive, United States; email: mrussell@jpl.nasa.gov
ER -

TY - JOUR
AU - Raulin, F.
AU - Hand, K.P.
AU - McKay, C.P.
AU - Viso, M.
TI - Exobiology and planetary protection of icy moons
PY - 2010
T2 - Space Science Reviews
VL - 153
IS - 1-4
SP - 511
EP - 535
DO - 10.1007/s11214-009-9610-x
UR - https://www.scopus.com/inward/record.uri?eid=2-s2.0-78049369304&doi=10.1007%2fs11214-009-9610-x&partnerID=40&md5=064766f60196402aa8a56cb61487ca84
AD - Jet Propulsion Laboratory, California Institute of Technology, Pasadena, CA, United States
AD - NASA Ames Research Center, M.S. 245-3, Moffett Field, CA, United States
AD - CNES/DSP/EU, Paris, France
AB - The outer solar system is an important area of investigation for exobiology, the study of life in the universe. Several moons of

the outer planets involve processes and structures comparable to those thought to have played an important role in the emergence of life on Earth, such as the formation and exchange of organic materials between different reservoirs. The study of these prebiotic processes on, and in, outer solar system moons is a key goal for exobiology, together with the question of habitability and the search for evidence of past or even present life. This chapter reviews the aspects of prebiotic chemistry and potential presence of life on Europa, Enceladus and Titan, based on the most recent data obtained from space missions as well as theoretical and experimental laboratory models. The habitability of these extraterrestrial environments, which are likely to include large reservoirs of liquid water in their internal structure, is discussed as well as the particular case of Titan's hydrocarbon lakes. The question of planetary protection, especially in the case of Europa, is also presented. © 2010 Springer Science+Business Media B.V.
KW - Astronomy
KW - Hydrocarbons
KW - Liquids
KW - Planets
KW - Reservoirs (water)
KW - Europa's ocean
KW - Exobiology
KW - Extraterrestrial life
KW - Habitability
KW - Liquid water
KW - Planetary protections
KW - Prebiotic chemistry
KW - Solar system
KW - Enceladus liquid water reservoir
KW - Europa's ocean
KW - Exobiology
KW - Extraterrestrial life
KW - Habitability
KW - Planetary protection
KW - Prebiotic chemistry
SN - 15729672 (ISSN)
LA - English
J2 - Space Sci Rev
M3 - Article
DB - Scopus
N1 - Export Date: 23 February 2023; Cited By: 10; Correspondence Address: F. Raulin; LISA CNRS, University Paris 7 and Paris 12, Créteil, France; email: Francois.Raulin@lisa.univ-paris12.fr; CODEN: SPSRA
ER -

TY - JOUR
AU - Varnali, T.
AU - Edwards, H.G.M.
TI - Iron-scytonemin complexes: DFT calculations on new UV protectants for terrestrial cyanobacteria and astrobiological implications
PY - 2010

T2 - Astrobiology
VL - 10
IS - 7
SP - 711
EP - 716
DO - 10.1089/ast.2009.0457
UR - https://www.scopus.com/inward/record.uri?eid=2-s2.0-77958183285&doi=10.1089%2fast.2009.0457&partnerID=40&md5=ca243df9d76144ec8affa4714e84c9db
AD - Bogazici University, Department of Chemistry, Istanbul, Turkey
AD - Chemical and Forensic Sciences, School of Life Sciences, University of Bradford, Bradford, United Kingdom
AB - Cyanobacterial colonies produce the radiation-protectant biomolecule scytonemin as part of their response strategy for survival in environmentally stressed conditions in hot and cold deserts. These colonies frequently use sandstone rocks as host matrices for subsurface colonization, which is accompanied by a zone of depletion of iron and transportation of iron compounds to the mineral surface. It is suggested that an iron-scytonemin complex could feature in this survival strategy and facilitate the movement of iron through the rock. Calculations were carried out on several hypothetical iron-scytonemin complexes to evaluate the most stable structure energetically and examine the effect of the complexation of the biomolecule upon the electronic absorption characteristics of the radiation-protectant species. The implications for extraterrestrial planetary detection and analytical monitoring of an iron-scytonemin complex are assessed. © Copyright 2010, Mary Ann Liebert, Inc.
KW - Cyanobacteria
KW - Indoles
KW - Iron
KW - Iron Chelating Agents
KW - Models, Theoretical
KW - Molecular Structure
KW - Phenols
KW - Radiation-Protective Agents
KW - Ultraviolet Rays
KW - Cyanobacteria
KW - indole derivative
KW - iron
KW - iron chelating agent
KW - phenol derivative
KW - radioprotective agent
KW - scytonemin
KW - article
KW - chemical structure
KW - chemistry
KW - cyanobacterium
KW - radiation exposure
KW - theoretical model
KW - ultraviolet radiation
KW - Cyanobacteria
KW - DFT calculations
KW - Extremophiles

KW - Iron complexes of scytonemin
KW - Scytonemin
KW - UV radiation protectant
SN - 15311074 (ISSN)
C2 - 20879865
LA - English
J2 - Astrobiology
M3 - Article
DB - Scopus
N1 - Export Date: 23 February 2023; Cited By: 17; Correspondence Address: T. Varnali; Bogazici University, Department of Chemistry, Istanbul, Turkey; email: varnali@boun.edu.tr
ER -

TY - JOUR
AU - Haghighipour, N.
AU - Vogt, S.S.
AU - Paul Butler, R.
AU - Rivera, E.J.
AU - Laughlin, G.
AU - Meschiari, S.
AU - Henry, G.W.
TI - The lick-carnegie exoplanet survey: A saturn-mass planet in the habitable zone of the nearby M4V star hip 57050
PY - 2010
T2 - Astrophysical Journal
VL - 715
IS - 1
SP - 271
EP - 276
DO - 10.1088/0004-637X/715/1/271
UR - https://www.scopus.com/inward/record.uri?eid=2-s2.0-77951844960&doi=10.1088%2f0004-637X%2f715%2f1%2f271&partnerID=40&md5=35c39916346de975ca63f40d65694c48
AD - Institute for Astronomy, NASA Astrobiology Institute, University of Hawaii-Manoa, Honolulu, HI 96822, United States
AD - Department of Astronomy and Astrophysics, UCO/Lick Observatory, University of California at Santa Cruz, Santa Cruz, CA 95064, United States
AD - Department of Terrestrial Magnetism, Carnegie Institute of Washington, Washington, DC 20015, United States
AD - Center of Excellence in Information Systems, Tennessee State University, Nashville, TN 37209, United States
AB - Precision radial velocities (RV) from Keck/HIRES reveal a Saturn-mass planet orbiting the nearby M4V star HIP 57050. The planet has a minimum mass of Msin i 0.3 M J, an orbital period of 41.4days, and an orbital eccentricity of 0.31. V-band photometry reveals a clear stellar rotation signature of the host star with a period of 98days, well separated from the period of the RV variations and reinforcing a Keplerian origin for the observed velocity variations. The orbital period of this planet corresponds to an orbit in the habitable zone of HIP 57050, with an expected planetary temperature of 230 K. The star has a metallicity of [Fe/H] = 0.32 0.06 dex, of order twice solar and among the highest

metallicity stars in the immediate solar neighborhood. This newly discovered planet provides further support that the well-known planet-metallicity correlation for F, G, and K stars also extends down into the M-dwarf regime. The a priori geometric probability for transits of this planet is only about 1%. However, the expected eclipse depth is 7%, considerably larger than that yet observed for any transiting planet. Though long on the odds, such a transit is worth pursuing as it would allow for high quality studies of the atmosphere via transmission spectroscopy with Hubble Space Telescope. At the expected planetary effective temperature, the atmosphere may contain water clouds. © 2010. The American Astronomical Society. All rights reserved.
KW - Astrobiology
KW - Planetary systems
KW - Stars: individual (HIP 57050)
PB - Institute of Physics Publishing
SN - 0004637X (ISSN)
LA - English
J2 - Astrophys. J.
M3 - Article
DB - Scopus
N1 - Export Date: 23 February 2023; Cited By: 40
ER -

TY - JOUR
AU - Robinson, T.D.
AU - Meadows, V.S.
AU - Crisp, D.
TI - Detecting oceans on extrasolar planets using the glint effect
PY - 2010
T2 - Astrophysical Journal Letters
VL - 721
IS - 1 PART 2
SP - L67
EP - L71
DO - 10.1088/2041-8205/721/1/L67
UR - https://www.scopus.com/inward/record.uri?eid=2-s2.0-78649241652&doi=10.1088%2f2041-8205%2f721%2f1%2fL67&partnerID=40&md5=8218888ee44879367df0a6953836e557
AD - Astronomy Department, University of Washington, Seattle, WA 98195, United States
AD - Jet Propulsion Laboratory, Pasadena, CA 91109, United States
AD - NASA Astrobiology Institute, United States
AB - Glint, the specular reflection of sunlight off Earth's oceans,may reveal the presence of oceans on an extrasolar planet. As an Earth-like planet nears crescent phases, the size of the ocean glint spot increases relative to the fraction of the illuminated disk, while the reflectivity of this spot increases. Both effects change the planet's visible reflectivity as a function of phase. However, strong forward scattering of radiation by clouds can also produce increases in a planet's reflectivity as it approaches crescent phases, and surface glint can be obscured by Rayleigh scattering and atmospheric absorption. Here, we explore the detectability of glint in the presence of an atmosphere and

realistic phase-dependent scattering from oceans and clouds. We use the NASA Astrobiology Institute's Virtual Planetary Laboratory three-dimensional line-by-line, multiple-scattering spectral Earth model to simulate Earth's broadband visible brightness and reflectivity over an orbit. Our validated simulations successfully reproduce phase-dependent Earthshine observations. We find that the glinting Earth can be as much as 100% brighter at crescent phases than simulations that do not include glint, and that the effect is dependent on both orbital inclination and wavelength, where the latter dependence is caused by Rayleigh scattering limiting sensitivity to the surface. We show that this phenomenon may be observable using the James Webb Space Telescope paired with an external occulter. © 2010. The American Astronomical Society. All rights reserved.
KW - Astrobiology
KW - Earth
KW - Planets and satellites: composition
KW - Radiative transfer
KW - Scattering
KW - Techniques: photometric
PB - Institute of Physics Publishing
SN - 20418205 (ISSN)
LA - English
J2 - Astrophys. J. Lett.
M3 - Article
DB - Scopus
N1 - Export Date: 23 February 2023; Cited By: 89; Correspondence Address: T. D. Robinson; Astronomy Department, University of Washington, Seattle, WA 98195, United States; email: robinson@astro.washington.edu
ER -

TY - JOUR
AU - Haas, J.R.
TI - The potential feasibility of chlorinic photosynthesis on exoplanets
PY - 2010
T2 - Astrobiology
VL - 10
IS - 9
SP - 953
EP - 963
DO - 10.1089/ast.2009.0364
UR - https://www.scopus.com/inward/record.uri?eid=2-s2.0-78650043630&doi=10.1089%2fast.2009.0364&partnerID=40&md5=d984f2fbb0389756ea8686570b0c7abb
AD - Department of Geosciences, Western Michigan University, Kalamazoo, MI, United States
AB - The modern search for life-bearing exoplanets emphasizes the potential detection of O2 and O3 absorption spectra in exoplanetary atmospheres as ideal signatures of biology. However, oxygenic photosynthesis may not arise ubiquitously in exoplanetary biospheres. Alternative evolutionary paths may yield planetary atmospheres tinted with the waste products of other dominant

metabolisms, including potentially exotic biochemistries. This paper defines chlorinic photosynthesis (CPS) as biologically mediated photolytic oxidation of aqueous Cl- to form halocarbon or dihalogen products, coupled with CO2 assimilation. This hypothetical metabolism appears to be feasible energetically, physically, and geochemically, and could potentially develop under conditions that approximate the terrestrial Archean. It is hypothesized that an exoplanetary biosphere in which chlorinic photosynthesis dominates primary production would tend to evolve a strongly oxidizing, halogen-enriched atmosphere over geologic time. It is recommended that astronomical observations of exoplanetary outgoing thermal emission spectra consider signs of halogenated chemical species as likely indicators of the presence of a chlorinic biosphere. Planets that favor the evolution of CPS would probably receive equivalent or greater surface UV flux than is produced by the Sun, which would promote stronger abiotic UV photolysis of aqueous halides than occurred during Earth's Archean era and impose stronger evolutionary selection pressures on endemic life to accommodate and utilize halogenated compounds. Ocean-bearing planets of stars with metallicities equivalent to, or greater than, the Sun should especially favor the evolution of chlorinic biospheres because of the higher relative seawater abundances of Cl, Br, and I such planets would tend to host. Directed searches for chlorinic biospheres should probably focus on G0-G2, F, and A spectral class stars that have bulk metallicities of +0.0 Dex or greater.Key Words: Atmospheric compositions-Biogeochemistry- Exoplanets-Life detection-Metabolism. Astrobiology 10, 953-963. © Mary Ann Liebert, Inc.
KW - Astronomy
KW - Chlorine
KW - Earth (Planet)
KW - Evolution, Planetary
KW - Exobiology
KW - Oxygen
KW - Photons
KW - Photosynthesis
KW - Planets
KW - Solar Activity
KW - chlorine
KW - oxygen
KW - article
KW - astrobiology
KW - astronomy
KW - chemistry
KW - photon
KW - photosynthesis
KW - solar energy
SN - 15311074 (ISSN)
C2 - 21118026
LA - English
J2 - Astrobiology
M3 - Article
DB - Scopus
N1 - Export Date: 23 February 2023; Cited By: 8; Correspondence Address: J. R. Haas; Department of Geosciences, Western Michigan

University, Kalamazoo, MI, United States; email: johnson.haas@wmich.edu
ER -

TY - JOUR
AU - Lefticariu, L.
AU - Pratt, L.A.
AU - LaVerne, J.A.
AU - Schimmelmann, A.
TI - Anoxic pyrite oxidation by water radiolysis products - A potential source of biosustaining energy
PY - 2010
T2 - Earth and Planetary Science Letters
VL - 292
IS - 1-2
SP - 57
EP - 67
DO - 10.1016/j.epsl.2010.01.020
UR - https://www.scopus.com/inward/record.uri?eid=2-s2.0-77249135782&doi=10.1016%2fj.epsl.2010.01.020&partnerID=40&md5=de745a36722d9447a476186a3b1bac7b
AD - Department of Geology, Southern Illinois University, Carbondale, IL, United States
AD - Department of Geological Sciences, Indiana University, IN 47405, United States
AD - Radiation Laboratory, Department of Physics, University of Notre Dame, Notre Dame, IN 46556, United States
AD - Indiana Princeton Tennessee Astrobiology Initiative (IPTAI), NASA Astrobiology Institute, Indiana University, Bloomington, IN, United States
AB - Radiolysis on rocky planetary bodies provides chemical species across redox gradients that can supply energy for microbial life in subsurface environments. We investigated the oxidation of pyrite to aqueous sulfate (SO42-) by water γ-radiolysis products with concomitant production of molecular hydrogen (H2). The production of H2, the only gaseous product recovered at the end of pyrite-water irradiation experiments, was found to be dependent on pyrite/water ratios. The yield of radiolytically-produced SO42- correlated with the total irradiation dose. The effectiveness of γ-radiation in oxidative dissolution of pyrite is determined by (1) redox reactions between radiolytically-produced oxidants and pyrite, and (2) the interaction between γ-radiation and pyrite's crystalline structure. Radiolytic oxidation of reduced sulfur occurs with the oxidants HO{filled circle} (hydroxyl radical) and Fe3+ (ferric iron) involving two different pathways. The radiolytic production of these two chemical oxidants is self-sustaining in the presence of water and Fe2+ in the system. Radiolytic oxidation can produce significant sulfur isotope effects by preferentially bringing 34S into solution as sulfate and leaving a 32S-enriched elemental sulfur layer on the pyrite surface. Experimental abiotic fractionations of sulfur isotopes between original pyrite and its sulfur oxidation products are significant and indicate that isotopically distinct sulfate is being produced during oxidation. Based on measured radiolysis constants for pyrite and radiation dose estimates for continental

crust, we show that radiolysis of water coupled to oxidation of metallic sulfides could be a significant source of sulfate in many geological environments. Implications of this work are broad, impacting our assessment of the potential for life to exist in subsurface environments on Earth as well as in extraterrestrial environments. © 2009 Elsevier B.V.
KW - Dissolution
KW - Hydrogen
KW - Irradiation
KW - Isotopes
KW - Metal recovery
KW - Oxidants
KW - Oxidation
KW - Petroleum tar
KW - Radiation chemistry
KW - Redox reactions
KW - Sulfur
KW - Chemical oxidants
KW - Chemical species
KW - Concomitant production
KW - Continental crusts
KW - Crystalline structure
KW - Elemental sulfur
KW - Extraterrestrial environments
KW - Ferric iron
KW - Gaseous products
KW - Geological environment
KW - Hydroxyl radicals
KW - Irradiation dose
KW - Irradiation experiments
KW - Microbial life
KW - Molecular hydrogen
KW - Oxidative dissolution
KW - Planetary bodies
KW - Potential sources
KW - Presence of water
KW - Pyrite oxidation
KW - Pyrite surfaces
KW - Radiation dose
KW - Radiolysis products
KW - Radiolytic oxidation
KW - Radiolytic production
KW - Redox gradients
KW - Subsurface environment
KW - Sulfur isotope
KW - Sulfur oxidation
KW - Water coupled
KW - Water radiolysis
KW - aqueous solution
KW - exobiology
KW - gamma ray radiation
KW - hydrogen
KW - oxidation
KW - pyrite

KW - redox conditions
KW - sulfate
KW - Radiolysis
KW - astrobiology
KW - hydrogen
KW - pyrite oxidation
KW - radiolysis
KW - sulfate
SN - 0012821X (ISSN)
LA - English
J2 - Earth Plan. Sci. Lett.
M3 - Article
DB - Scopus
N1 - Export Date: 23 February 2023; Cited By: 31; Correspondence Address: L. Lefticariu; Department of Geology, Southern Illinois University, Carbondale, IL, United States; email: Lefticariu@geo.siu.edu
ER -

TY - JOUR
AU - Hart, J.
TI - Cosmic Commons: Contact and community
PY - 2010
T2 - Theology and Science
VL - 8
IS - 4
SP - 371
EP - 392
DO - 10.1080/14746700.2010.517637
UR - https://www.scopus.com/inward/record.uri?eid=2-s2.0-79957872209&doi=10.1080%2f14746700.2010.517637&partnerID=40&md5=a1d7a361c73adf3e20c87a8da761ae20
AB - As we Homo sapiens on planet Earth prepare for possible contact with extraterrestrial life (ET)especially extraterrestrial intelligent (ETI) lifewe need to consider ethics. What will be the ethical posture we adopt as Earth meets space? This article advocates an ethic of praxis based upon the concept of a Cosmic Commons. Intelligent extraterrestrial beings, from their experiences in biotic and abiotic environments, should share with us a universal regard for intelligence (intellilife), respect for biotic evolution, and a sense of responsibility for shared common space in our places in the universe. As we venture from Earth into space, what will be required? Not anxiety over human dis-placement to new worlds; nor should we invest our efforts in irresponsibly exploiting the natural goods of other planets. Rather, we need an ethical transformation of terrestrial human thought and conduct prior to, during, and as a consequence of extraterrestrial explorations and engagements. We need an anticipatory Cosmic Charter. © 2010 Center for Theology and the Natural Sciences.
KW - Avatar
KW - Cosmic Charter
KW - Cosmic Commons
KW - ETI
KW - Praxis ethics

KW - Stephen Hawking
SN - 14746719 (ISSN)
LA - English
J2 - Theology Sci.
M3 - Article
DB - Scopus
N1 - Export Date: 23 February 2023; Cited By: 7
ER -

TY - CHAP
AU - Balme, M.R.
AU - Gallagher, C.J.
AU - Page, D.P.
AU - Murray, J.B.
AU - Muller, J.-P.
AU - Kim, J.-R.
TI - The Western Elysium Planitia Paleolake
PY - 2010
T2 - Lakes on Mars
SP - 275
EP - 305
DO - 10.1016/B978-0-444-52854-4.00010-6
UR - https://www.scopus.com/inward/record.uri?eid=2-s2.0-80054026447&doi=10.1016%2fB978-0-444-52854-4.00010-6&partnerID=40&md5=f95b4cfe393c319da7207021fc4e96e8
AD - Department of Earth and Environmental Sciences, The Open University, Walton Hall, MK, United Kingdom
AD - The Planetary Science Institute, Tucson, AZ, United States
AD - University College Dublin School of Geography, University College Dublin, Belfield, Dublin, Ireland
AD - Planetary and Space Science Research Institute, The Open University, Walton Hall, MK, United Kingdom
AD - Department of Space and Climate Physics, University College London, Mullard Space Science Laboratory, Dorking, RH, United Kingdom
AB - This chapter concentrates on the Western Elysium Basin and describes the regional topography, the morphology of the landforms, and the geological evidence in support of the interpretation that this is a paleolake basin. Neither continuous, internal flow margins nor evidence for vent structures in the main Western Elysium Basin was found. The topographic and mapping data support a fluvial, rather than volcanic, emplacement mechanism for platy terrain in the Western Elysium Basin, based on the current knowledge of terrestrial flood lavas. The distinctive landforms of the Western Elysium Basin are consistent with formation in an ice-rich, particulate substrate. The sorted patterned ground and the back-wasting of polygonized terrain are indicative of an ice-rich regolith modified by freeze-thaw or melt processes. These morphological assertions are consistent with stratigraphic observations in the wider Cerberus Plains region that show landforms superposing impact craters. Taken together, these observations provide evidence for a recent, warmer climate and a recent, low-latitude, periglacial landscape across the Elysium Planitia. The low-latitude, geologically young, lacustrine deposits in Western Elysium Basin provide an important target for

future in situ astrobiological investigations. © 2010 Elsevier B.V. All rights reserved.
PB - Elsevier
SN - 978-044452854-4 (ISBN)
LA - English
J2 - Lakes on Mars
M3 - Book chapter
DB - Scopus
N1 - Export Date: 23 February 2023; Cited By: 14
ER -

TY - CHAP
AU - Kereszturi, A.
AU - Möhlmann, D.
AU - Berczi, S.
AU - Ganti, T.
AU - Horvath, A.
AU - Kuti, A.
AU - Sik, A.
TI - Seepages and the astrobiological potential of polar dunes on mars
PY - 2010
T2 - Astrobiology: Physical Origin, Biological Evolution and Spatial Distribution
SP - 97
EP - 113
UR - https://www.scopus.com/inward/record.uri?eid=2-s2.0-85030260866&partnerID=40&md5=b8bf5db8d96f90bba309ffbb4bfab794
AD - Collegium Budapest Institute for Advanced Study, Szentharomsag 2, Budapest, H-1041, Hungary
AD - DLR Institute für Planetenforschung, Rutherfordstr. 2., Berlin, D-12489, Germany
AD - Eotvos Lorand University of Sciences, Institute of Geography and Earth Sciences, Planetary Science Research Group, Pázmány 1/c, Budapest, H-1117, Hungary
AD - Eotvos Lorand University of Sciences, Department of Astronomy, Pázmány 1/c., Budapest, H-1117, Hungary
AD - Konkoly Observatory, Pf. 67., Budapest, H-1525, Hungary
AB - At the polar dunes of Mars branching dark slope streaks emanating from Dark Dune Spots were observed in local springtime on MOC and HiRISE images. They seep downward with an average speed of 1.4 meter/sol when the temperature of the terrain on 3 km spatial resolution is between 180 and 250 K. Theoretical considerations and calculations suggest interfacial liquid-like water has to be present on Mars, and possibly is causing the observed seepage-like features. The thin water lubricated grains may slide over each other, and/or the stress caused by the refreezing of this interfacial water could also produce such markings, as well as the moving front of solid ice / liquid water phase changes. Such an ephemerally wet environment between the dune grains may have astrobiological significance. © 2010 Nova Science Publishers, Inc.
PB - Nova Science Publishers, Inc.
SN - 978-161761955-7 (ISBN); 978-160741290-8 (ISBN)
LA - English

J2 - Astrobiology: Physical Origin, Biological Evolution and Spatial Distribution
M3 - Book chapter
DB - Scopus
N1 - Export Date: 23 February 2023; Cited By: 1; Correspondence Address: A. Kereszturi; Collegium Budapest Institute for Advanced Study, Budapest, Szentharomsag 2, H-1041, Hungary; email: akos@colbud.hu
ER -

TY - CONF
TI - EPJ Web of Conferences
PY - 2010
T2 - EPJ Web of Conferences
VL - 9
SP - 285
UR - https://www.scopus.com/inward/record.uri?eid=2-s2.0-84921810398&partnerID=40&md5=2b245d1803747b352740c9134d3039e1
AB - The proceedings contain 21 papers. The topics discussed include: overview of major processes and mechanisms affecting the mercury cycle on different spatial and temporal scales; atmospheric emissions and long-range transport of persistent organic chemicals; air-sea interactions of semi-volatile organic compounds in the tropical environment of southeast Asia; radiative effects of the cloudy atmosphere from ground and satellite based observations; fire and climate: biomass burning recorded in ice and lake cores; uncertainties in climate change projections, from the global to the regional scale; climate change in China and China's policies and actions for addressing climate change; a brief review of the use of environmental chambers for gas phase studies of kinetics, chemical mechanisms and characterisation of field instruments; terrestrial atmosphere, water and astrobiology; and atmospheres and surfaces of small bodies and dwarf planets in the Kuiper Belt.
A2 - Boutron C.
PB - EDP Sciences
SN - 21016275 (ISSN); 978-275980599-0 (ISBN)
LA - English
J2 - EPJ Web Conf.
M3 - Conference review
DB - Scopus
N1 - Export Date: 23 February 2023; Cited By: 0
ER -

TY - JOUR
AU - Hemley, R.J.
TI - Percy W. Bridgman's second century
PY - 2010
T2 - High Pressure Research
VL - 30
IS - 4
SP - 581
EP - 619
DO - 10.1080/08957959.2010.538974
UR - https://www.scopus.com/inward/record.uri?eid=2-

s2.0-78650440505&doi=10.1080%2f08957959.2010.538974&partnerID=40&md5=4912a28076a672d982ba50ef9e61aca1
AD - Geophysical Laboratory, Carnegie Institution of Washington, Washington, DC 20015, United States
AB - Percy Williams Bridgman's impact on science began in 1909 with his first three experimental papers. These publications on high pressure calibration, techniques, and compressibility, together with the many articles that followed, established his influence on the course of modern high pressure research. Grounded in classical thermodynamics and practical mechanics, his developments showed how the variable of pressure leads to myriad transformations in materials. Studies carried out under a broader range of conditions now provide unprecedented insights into chemical and physical properties at multimegabar pressures and temperatures from millikelvins to thousands of degrees, where novel electronic, magnetic, and superconducting phases are now being discovered. With careful attention to experimental techniques and material performance, Bridgman extended the available pressure range that could be achieved in the laboratory with the development of new devices. We are now witness to continued refinement of static and dynamic compression methods and in situ measurement techniques, including the marriage of high pressure methods with large facilities such as synchrotron, neutron, and laser sources. Bridgman showed the broad range of implications of this work; the modern field of high pressure research now spans physics and chemistry, geosciences and planetary science, materials science and technology, and biology. Selected examples illustrate Bridgman's impact and legacy in this, his second century. For dense hydrogen, new insights have been obtained from high P-T measurements as well as studies of alloys and compounds of hydrogen, leading to the creation of new metallic and superconducting phases. Studies of other hydrogen-rich systems provide both tests of fundamental theory and potentially useful materials for hydrogen storage. High pressure studies of oxides have led to new ferroelectric and multiferroic materials and phases with remarkable properties that guide the design and fabrication of new devices. With the discovery of super-Earths outside our solar system, the high pressure properties of silicates, oxides, volatiles, and the full complement of planetary materials are now problems of cosmic importance well beyond the conditions found on and within the Earth. Developments in high pressure biology are addressing the question of the depth of the biosphere, the processes and reservoirs of carbon in our planet, and new insights into the origins of life as we know it, as well as the possibility of extraterrestrial life. Improved materials that can withstand high P-T conditions and other extreme environments include new forms of diamond, which are advancing experimental methods and finding numerous applications in advanced technology. These developments dovetail with synergetic advances in a broad spectrum of radiation techniques including coherent X-ray, intense neutron, and ultrabright laser sources. © 2010 Taylor & Francis.
KW - Astronomy
KW - Biology
KW - Diamonds
KW - Earth (planet)

KW - Ecology
KW - Ferroelectric materials
KW - High pressure effects
KW - Hydrogen
KW - Hydrogen storage
KW - Materials science
KW - Melting
KW - Polymorphism
KW - Silicates
KW - Superconducting materials
KW - Superconductivity
KW - Teaching
KW - Thermodynamics
KW - Van der Waals forces
KW - Electrical phenomena
KW - Geosciences
KW - High-pressure technique
KW - Planetary science
KW - Van der Waals compound
KW - High pressure engineering
KW - biology
KW - carbon
KW - diamond
KW - electrical phenomena
KW - geosciences
KW - high pressure techniques
KW - hydrogen
KW - melting
KW - planetary science
KW - polymorphism
KW - van der Waals compounds
SN - 14772299 (ISSN)
LA - English
J2 - High. Pressure. Res.
M3 - Article
DB - Scopus
N1 - Export Date: 23 February 2023; Cited By: 41; Correspondence Address: R. J. Hemley; Geophysical Laboratory, Carnegie Institution of Washington, Washington, DC 20015, United States; email: rhemley@ciw.edu; CODEN: HPRSE
ER -

TY - JOUR
AU - Hynek, B.M.
TI - Extraterrestrial digital elevation models: Constraints on planetary evolution, with focus on Mars
PY - 2010
T2 - International Journal of Remote Sensing
VL - 31
IS - 23
SP - 6259
EP - 6274
DO - 10.1080/01431160903403078
UR - https://www.scopus.com/inward/record.uri?eid=2-

s2.0-78650111004&doi=10.1080%2f01431160903403078&partnerID=40&md5=f6a2256413369231266087 23bd56df8a
AD - Department of Geological Sciences, Laboratory for Atmospheric and Space Physics, 392 UCB, University of Colorado, Boulder, CO 80309, United States
[truncated: 5,526,998 more chars]
